# Regulatory Circuit of Human MicroRNA Biogenesis

**DOI:** 10.1371/journal.pcbi.0030067

**Published:** 2007-04-20

**Authors:** Ji Lee, Zhihua Li, Rachel Brower-Sinning, Bino John

**Affiliations:** 1 Department of Computational Biology, University of Pittsburgh School of Medicine, Pittsburgh, Pennsylvania, United States of America; 2 Department of Bioengineering, Pennsylvania State University, University Park, Pennsylvania, United States of America; 3 University of Pittsburgh Cancer Institute, Pittsburgh, Pennsylvania, United States of America; University of California San Diego, United States of America

## Abstract

miRNAs (microRNAs) are a class of endogenous small RNAs that are thought to negatively regulate protein production. Aberrant expression of many miRNAs is linked to cancer and other diseases. Little is known about the factors that regulate the expression of miRNAs. We have identified numerous regulatory elements upstream of miRNA genes that are likely to be essential to the transcriptional and posttranscriptional regulation of miRNAs. Newly identified regulatory motifs occur frequently and in multiple copies upstream of miRNAs. The motifs are highly enriched in G and C nucleotides, in comparison with the nucleotide composition of miRNA upstream sequences. Although the motifs were predicted using sequences that are upstream of miRNAs, we find that 99% of the top-predicted motifs preferentially occur within the first 500 nucleotides upstream of the transcription start sites of protein-coding genes; the observed preference in location underscores the validity and importance of the motifs identified in this study. Our study also raises the possibility that a considerable number of well-characterized, disease-associated transcription factors (TFs) of protein-coding genes contribute to the abnormal miRNA expression in diseases such as cancer. Further analysis of predicted miRNA–protein interactions lead us to hypothesize that TFs that include c-Myb, NF-Y, Sp-1, MTF-1, and AP-2α are master-regulators of miRNA expression. Our predictions are a solid starting point for the systematic elucidation of the causative basis for aberrant expression patterns of disease-related (e.g., cancer) miRNAs. Thus, we point out that focused studies of the TFs that regulate miRNAs will be paramount in developing cures for miRNA-related diseases. The identification of the miRNA regulatory motifs was facilitated by a new computational method, K*-*Factor. K-Factor predicts regulatory motifs in a set of functionally related sequences, without relying on evolutionary conservation.

## Author Summary

microRNAs (miRNAs) are unusually small RNAs that are thought to control the production of proteins in the cell. Recent studies have linked miRNAs to several types of cancers. Several studies strongly suggest that miRNAs could be useful as diagnostic and prognostic markers of various cancers. Thus, although miRNAs appear to have opened up a new chapter in cancer biology, the fundamental question regarding why miRNAs are strongly associated with diseases such as cancer remain unclear. Here, we endeavored to systematically identify the factors that regulate miRNA biogenesis. We first identified a large number of DNA sequence elements that are characteristic of miRNA genes, using a new computational method named K-Factor. The sequence elements were then used to match known protein binding sites to identify specific proteins (transcription factors (TF)) that regulate miRNA biogenesis. Based on our observations, we put forward the hypothesis that a number of known TFs are primarily responsible for the aberrant regulation of miRNAs in cancer and other diseases.

## Introduction

microRNAs (miRNAs) are endogenous nonprotein-coding RNAs that are thought to negatively regulate gene expression [1–4]. Although hundreds of human miRNAs have been discovered, the functions of most miRNAs are unknown [5–7]. miRNAs are present in organisms as diverse as viruses, flies, worms, humans, and plants, where they regulate fundamental cellular processes such as cell differentiation, cell proliferation, and apoptosis [8–16]. Computational predictions, supported by experimental evidence, indicate that miRNAs regulate a large fraction of metazoan genes [17–27]. Aberrant expression of miRNAs is linked to diseases such as cancer [6,11,14,16,28–38]. A recent study of global expression levels of miRNAs in various cancers indicates that miRNA expression patterns are generally more useful than messenger RNA (mRNA) profiles to classify tumors [39]. A clear understanding of the miRNA pathway is thus necessary to unveil the role of miRNAs in gene regulation and their causal effects on complex genetic diseases.

Little is known about the transcription and posttranscriptional processing of miRNA genes. Approximately 25% of miRNAs are located in introns of protein-coding genes and are likely to be transcribed along with their host genes. Multiple transcription start sites [40] within such genomic regions may lead to autonomous transcription of miRNAs and their host protein-coding genes. Studies indicate that miRNA genes are generally transcribed from their own promoters by RNA Polymerase II [41,42]. However, the possibility that a number of miRNA genes may be transcribed by other RNA polymerases (e.g., pol III) cannot be excluded [43,44]. In the nucleus, miRNA genes are transcribed into primary transcripts (pri-miRNA) that are generally thought to be several thousand bases in length [45,46]. The pri-miRNAs are cleaved into shorter, approximately 60 nucleotide (nt) stretches of stem-loop–forming transcripts (pre-miRNA) by an assembly of Drosha, an RNase III enzyme, and its cofactor, DGCR8 [47]. Following nuclear processing by Drosha, pre-miRNAs are exported to the cytoplasm where they are cleaved and processed by another RNase III enzyme, Dicer, to generate mature miRNAs [2].

Several lines of evidence imply that the information for transcription and sequential processing of miRNAs is embedded in the upstream regions of miRNAs. First, a recent study using chromatin immunoprecipitation, coupled with DNA microarrays, suggested that at least one of the three transcription factors (TFs), OCT4, SOX2, and NANOG regulates the transcription of 14 miRNA genes. Specific sequence elements upstream of *miR-1*, *miR-223,* and *miR-17* are also known to interact with specific TFs [13,48,49]. A computational scan of upstream regions of miRNA genes in nematodes provided evidence for a sequence motif that is present upstream of almost all independently transcribed nematode miRNA genes [50]. It is reasonable to assume that a number of miRNA TFs are sequence specific and bind to the upstream regions of miRNAs at select sites. Hence, the TF binding sites (TFBS) and other cis-regulatory motifs (CRMs) that are upstream of miRNAs are crucial to the regulation of the expression of miRNAs.

Several computational methods have been developed to identify CRMs of protein-coding genes [51–54]. The computational methods that identify CRMs in a set (input) of upstream gene sequences can be classified into two distinct strategies. One class of methods relies on previously characterized CRMs to identify similar motifs that occur in the input set. The second class of methods is based on de novo identification of regulatory elements. Both methods frequently make use of evolutionarily conserved candidate CRMs to increase the accuracy of their predictions. De novo identification of CRMs has the advantage of predicting novel CRMs that may be missed by other methods [55–60]. In particular, de novo methods, such as FastCompare [59] and HexDiff [58], are useful to identify sequences of defined length, termed *k*-mers (e.g., hexamers, *k* = 6), that frequently occur upstream of transcribed sequences.

We endeavored to develop a de novo method to discover and study the characteristics of CRMs that are upstream sequences of human miRNAs. The de novo methods, such as FastCompare [59], identify functionally relevant *k*-mers by comparing the frequency distribution of *k*-mers in the set of input sequences of interest to an appropriate background model (e.g., randomly generated set of sequences [61]). The choice of the background model is context dependent [56], and the interpretation of the resulting observations must be based on a clear understanding of the background model. For example, the hexamer AAAAAT is 543 times more abundant than CGCGTA in the human X chromosome sequence. However, the overrepresentation of these motifs based on a background model of randomly generated sequences may also be interpreted as a mere outcome of genome expansion [62–64]. We chose a background model that closely reflects the evolution of genome by making use of sequences arbitrarily extracted from the human genome.

To date, no computational method has been applied to systematically study the regulatory motifs that control the biogenesis of human miRNA genes. In this report, we build upon the concept of using *k*-mers to reliably identify CRMs that control the transcription and post-transcriptional processing of miRNAs. We investigated whether CRMs of human miRNA genes may be discovered using a new method, K-Factor. To help accurately identify *k*-mer–based CRMs that are likely to be biologically relevant, we incorporated the intrinsic distribution of *k*-mers in genomes of interest into K-Factor. For a given genome, K-Factor detects overrepresented *k-*mers in a set of sequences (e.g., upstream miRNA sequences), based on a background model of many sets of sequences that are randomly extracted from the genome of interest. We applied K-Factor to identify TFBSs in human miRNA upstream sequences. In summary, our work detects CRMs upstream of human miRNA sequences, identifies specific miRNAs that are likely regulated by CRMs, and predicts TFs that regulate specific miRNAs by matching predicted CRMs to known TFBS. We also find that the majority of the CRMs predicted for miRNA genes are also preferentially located towards the known transcription start sites of protein-coding genes.

## Results

### Upstream Sequences of miRNAs Contain Highly Overrepresented Motifs

**Figure 1:**
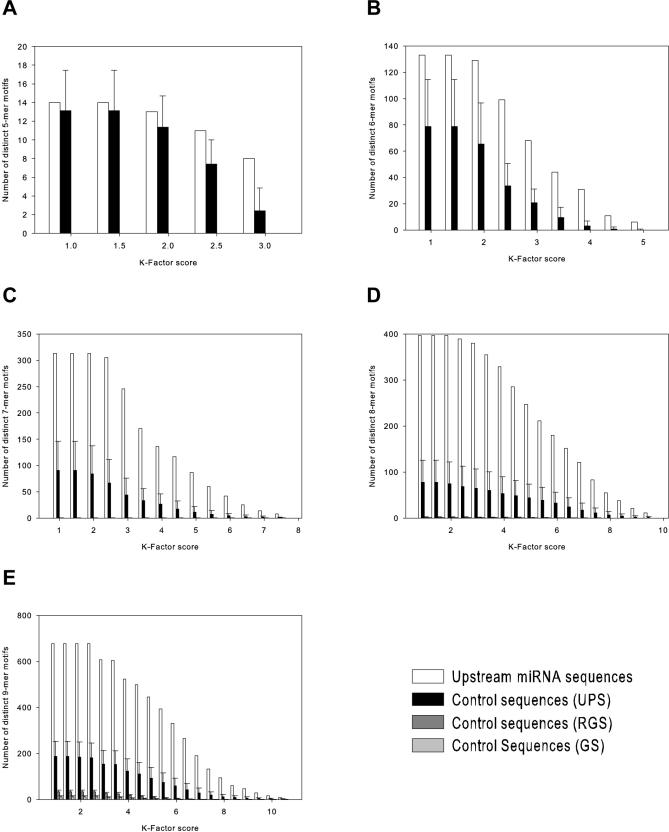
Accuracy of K-Factor Predictions in Identifying CRMs That Regulate miRNA Biogenesis The number of distinct CRMs predicted for biologically relevant miRNA upstream sequences and for 100 sets of miRNA upstream-like sequences (control sequences) are represented at various thresholds of K-Factor score (Z^f^, Z^N^ ≥ 7.0) and five different values of *k* : *k* = 5 (A), 6 (B), 7 (C), 8 (D), and 9 (E). The error bars for each threshold of K-Factor score correspond to the standard deviation of the number of predictions for 100 control datasets generated using one of the three methods (UPS, RGS, GS). Note that the control sequence sets corresponding to RGS and GS yield no predictions (noise) for *k-*mer sizes 5, 6, and 7.

To identify *k*-mers that are overrepresented in upstream regions of miRNA genes, we first analyzed the 10-kilobase (kb) regions that are immediately upstream of 214 representative human pre-miRNA sequences using K-Factor (*k* = 5, 6, 7, 8, and 9). We compared the number of predictions for the upstream miRNA sequences (signal) with that of the control sequences (noise) that were identical in length and consisted of approximately the same mononucleotide composition (A:T:C:G = 0.26:0.28:0.23:0.23) as the 10-kb miRNA upstream regions (Table S1). K-Factor generally predicted many more motifs for *k-*mers of size six to nine in upstream sequences of miRNA sequences than in control sequences (Figure 1). It is important to realize that upstream protein-coding sequences (UPS) are regulated by common proteins, such as the protein components of the RNA Polymerase II complex, and hence will share many common regulatory elements. Such commonly occurring regulatory motifs will lead to the overestimation of noise and hence an underestimation in the accuracy of the predictions. However, we chose to use UPS as a control set to provide added confidence to our predictions and to compare the distribution of *k-*mers in upstream miRNA sequences versus protein-coding genes. In summary, our data indicate that miRNA upstream regions generally contain more overrepresented regulatory motifs in comparison to protein-coding genes.

Seemingly due to their small size, pentamers did not yield statistically significant signal (Methods) based on the UPS dataset (Figure 1A). Although the signal to noise ratio (SNR) values that are calculated based on UPS dataset are likely to underestimate the accuracy of K-Factor, very few (14) pentamers could be classified as overrepresented in the miRNA upstream regions. On the contrary, hundreds of hexamers and longer *k*-mers were overrepresented in regions that immediately precede miRNA genes (Figure 1B–1D). For *k*-mers larger than five, K-Factor scores above 2.5 were generally sufficient to obtain statistically significant signals.

### Motifs That Are Overrepresented Upstream of miRNAs Are Also Preferentially Located within 1 Kb of Protein-Coding Genes

**Figure 2:**
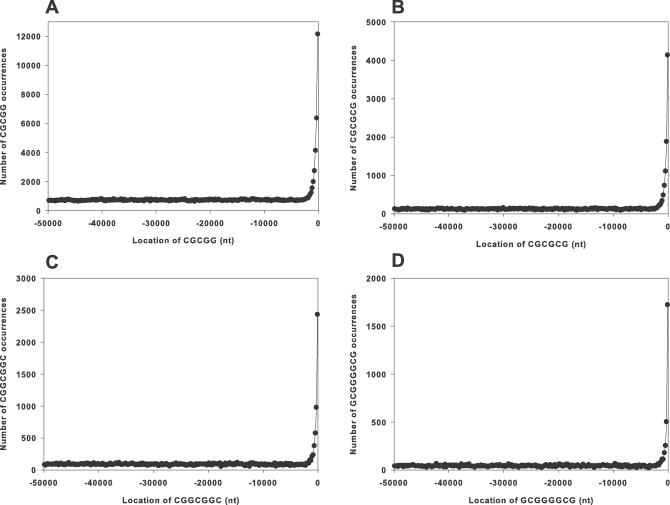
Overrepresented k-mers in Upstream miRNA Sequences Are Preferentially Located towards the Genomic Loci of Protein-Coding Genes Representative motifs show strong bias to occur near the genomic loci of protein-coding genes. The number of occurrences of CGCGG (A), CGCGCG (B), CGGCGGC (C), and GCGGGGCG (D) motifs are plotted at 200-nt intervals in the upstream regions (50 kb) of protein-coding genes. The number of occurrences of the motif in each 200-nt bin significantly increases towards the first 1,000 nt that are directly upstream of the protein-coding genes (−1,000 to 0 nt region).

**Figure 3:**
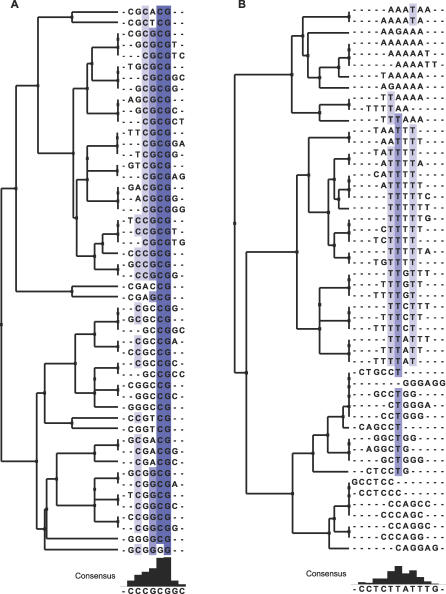
G and C Nucleotides Strongly Dominate the Predicted Motifs Comparison of the top 50 predicted hexamers (A) and the 50 most abundant hexamers in miRNA upstream regions (B) illustrate that the observed abundance of G and C nucleotides in the predicted motifs is not a consequence of nucleotide bias in upstream miRNA sequences.

We hypothesized that there may be common characteristics between *k-*mers that are overrepresented in miRNA upstream sequences and those upstream of protein-coding genes, because both miRNAs and protein-coding genes are generally transcribed by RNA Polymerase II [41,42]. Since motifs such as the TATA box occur immediately upstream of protein-coding genes, we reasoned that if the predictions are accurate, at least some of the motifs must occur immediately upstream of protein-coding genes. To our surprise, irrespective of the *k-*mer size, the majority (approximately 99%) of the about 400 top predicted motifs were preferentially located within the first 500 nt upstream of the genomic locations of protein-coding genes (Figure 2, Figures S1 to S5). Even more surprising was that a significant number of motifs occurred most profusely within the first 200 nt upstream of protein-coding genes. As a control, we also analyzed the distance distribution of an equal number of randomly selected motifs that occurred in miRNA upstream regions (Figures S6 to S10). Comparison of the distribution of the predicted motifs and control motifs clearly demonstrate that the predicted motifs play a major role in the transcription of protein-coding genes. We note that all predicted motifs are significantly enriched in G and C nucleotides (Figure 3A), and the observed enrichment of GC pairs is not a consequence of the nucleotide composition of miRNA upstream sequences (Figure 3B, Table S1).

### Predicted Motifs Are Evolutionarily Conserved

We investigated whether the top predicted motifs were also overrepresented in the upstream regions of miRNAs of mouse and opossum. Based on the top ranking 50 hexamers in each species, we found that the motifs significantly overlapped in human–mouse (34/50) and human–opossum (28/50) comparisons. The likelihood of obtaining the observed or greater number of hexamers common in human–mouse and human–opossum comparisons is estimated at 1.2 × 10^−59^ and 1.7 × 10^−44^, respectively (Methods). Moreover, among the top 50 motifs of miRNA upstream sequences of the human, mouse, and opossum genomes, 22 hexamers overlapped across all three species.

Since the estimation of statistical significance does not consider the evolutionary relationship between the genomes, we also performed another validation experiment using control motifs. We generated 100 sets of 50 control motifs that were equally as abundant (Methods) as the predicted motifs in miRNA upstream regions of mouse and analyzed their evolution with respect to the predicted motifs in human. We did not detect any overlap between the predicted human motifs and any of the 100 sets of control motifs in mouse. Additionally, we generated a single set of 50 human motifs that were equally abundant as the predicted human motifs and studied the evolution with respect to the aforementioned 100 sets of 50 control motifs in mouse. The average number of overlaps in the human–mouse comparison was 1.8 with a standard deviation of 2.2. Taken together, the predicted motifs are strongly conserved in evolution and the observed rate of evolutionary conservation is much higher than can be explained by the sequence conservation of human and mouse miRNA upstream regions.

Although the overlap between the predictions across human–mouse and human–opossum were statistically significant, the number of commonly occurring motifs decreased significantly with increasing lengths of *k-*mer. For instance, the comparison of the top 50 9-mer motifs of human and opossum yielded just four conserved motifs (100% sequence identity). Thus, subsequences of size six appeared to be an optimal size for the prediction of regulatory motifs because hexamers manifest strong overrepresentation in upstream miRNA regions, are well-conserved across species, are preferentially located towards the genomic locations of protein-coding genes, and thus are the shortest *k-*mers that appear to be sufficient to predict CRMs. Therefore, to identify TFs that interact with the predicted motifs, and to identify specific miRNAs that were regulated by TFs, we focused our analysis on predicted hexamers.

### Known TFs Interact with Predicted Hexamers

**Figure 4:**
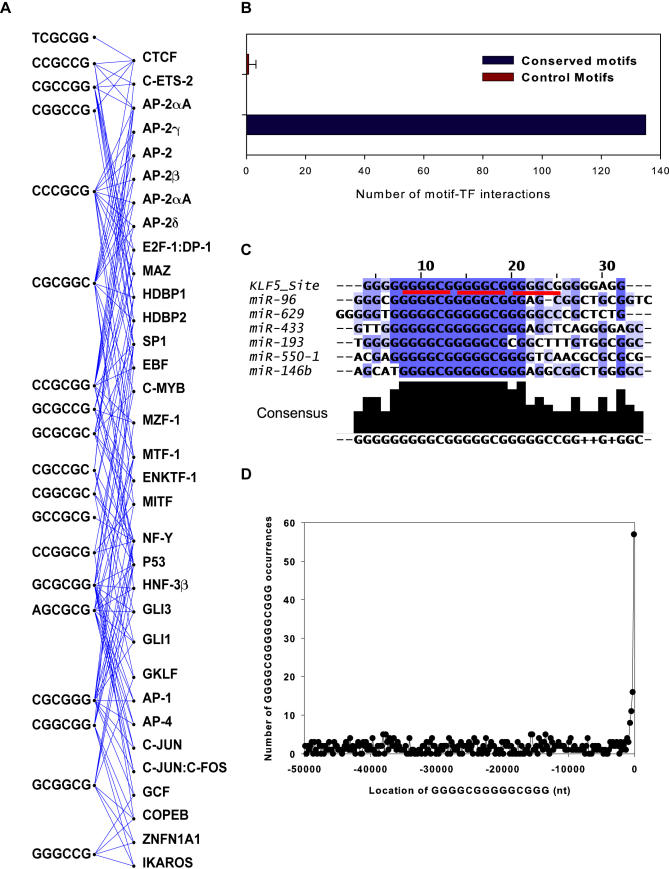
CRMs That Mediate the Transcription of miRNAs (A) Network of CRMs that are overrepresented in the upstream regions of human, mouse, and opossum miRNAs and their predicted TF interactors. (B) Numbers of predicted CRM–TF interactions for 22 evolutionarily conserved mammalian CRMs and 22 control motifs (Methods) in 100 control datasets. The error bar corresponds to the standard deviation of the number of predictions for the 100 control datasets. (C) The known KLF5 binding site (KLF5_Site) has three tandem copies of the GGGGCG motif (red underscore). A 15-nt long subsequence of the known KLF5 binding site is near perfectly conserved in six miRNA promoters (blue block). (D) The GGGGCGGGGGCGGG motif is also preferentially located towards the putative transcription start sites of protein-coding genes (also see Figure 2 legend).

The evolutionary preservation of a significant fraction (22/50) of the predicted human miRNA motifs in distantly related species (mouse and opossum) suggested that the conserved motifs are intolerant to mutations. We probed whether such motifs correspond to binding sites of known sequence-specific TFs. The 22-hexamer motifs were scanned against the TRANSFAC [65] database of human TFBS (Methods) to identify TF regulatory elements that match the predicted motifs (Figure 4A). As a control experiment, we generated 100 sets of 22 control motifs that were equally as abundant as the predicted motifs in miRNA upstream regions (Methods) and scanned them against TRANSFAC (Figure 4B). We identified 135 interactions between predicted 6-mers and known TFs. The control experiment yielded an average of 0.83 TF–hexamer interactions (σ = 2.8), which corresponded to a SNR of 162:1.

Little is known about the functions of the predicted hexamer CRMs in the context of the longer host TFBS. The top predicted hexamer motif is the inverse palindromic sequence CGCGCG which is also an evolutionarily conserved CRM. The heterodimeric complex of the two TFs, E2F4 and DP2, is known to bind each half of the palindromic CRM (CGC and GCG) [66]. The TF families that include E2F and DP proteins regulate the expression of multiple cell cycle genes and are well-conserved across mammals and many other eukaryotes [67]. E2Fs can function as transcriptional activators (e.g., E2F1) or repressors (e.g., E2F4) [68]. The consistent overrepresentation of the motif across three distantly related genomes raises the possibility that the E2F and DP families of proteins modulate miRNAs that regulate cell cycle. Members of a set of clustered miRNAs, *miR-17–5p* and *miR-20a,* are known to downregulate the expression E2F1, a transcriptional target of c-Myc that promotes cell cycle progression [13]. miRNAs are also required for stem cells to bypass the normal G1/S checkpoint in cell cycle [69]. We predict that E2F1, MITF, c-Myb, and p53 are TFs that regulate miRNAs via the CGCGCG motif. Interestingly, all four TFs are involved in the regulation of the cell cycle [68,70–72]. We also noticed that CGCGCG is predominantly located within the first 1,000 nt of the putative transcription start sites of protein-coding genes (Figure 2B). Although transcription start sites of miRNA genes are not known, due to uncertainty of the length of the miRNA primary transcript, they are likely not very distant from the genomic locations of miRNA precursors. Therefore, to identify specific miRNAs that are regulated by the motif, we used a conservative criterion that at least three CGCGCG motifs occur within a contiguous stretch of 2,000 nt in the upstream regions (<10,000 nt) of miRNA precursors. We identified 25 distinct miRNA loci (Table S2) corresponding to 42 pre-miRNAs that satisfied the criterion. The majority of the miRNAs are either expressed in neurons or are associated with specific stages of cell development. We speculate that several of the 233 pre-miRNAs in seven loci *(miR-9–1, −9–2, −17, −18a, −18b, −19a, −19b-1, −19b-2, −20a, −20b, −25, −92–1, −92–2, −93, −96, −106a, −106b, −124a, −182, −183, −345, −363,* and *-486),* the mature miRNA transcripts expressed during various developmental stages, are involved in modulating the cell cycle. We have also identified five instances of CGCGCG within a short region of 1,700 nt upstream of *miR-20a* that is known to regulate E2F1 [13]. Remarkably, 11 miRNAs *(miR-9, −92b, −96, −101, −124a, −129, −132, −135b, −191, −212,* and *-425)* that correspond to eight distinct miRNA loci are highly expressed in brain. The coincidence of the presence of the CGCGCG motif in the upstream region of miRNAs and the restricted expression patterns of these miRNAs indicate that this motif may be a fundamental factor in the transcription of many miRNAs.

Another predicted CRM, GGGGCG occurs three consecutive times within a known 29-nt long binding site of Kruppel-like factor 5 (KLF5) [73] (Figure 4C).The KLF5 is known to bind to sequences that contain GGGGCG [73]. KLF5 is a TF that is involved in cellular proliferation and cancer [74–76]. The KLF family of proteins can be transcriptional activators or repressors, and they are thought to bind to similar DNA sequences that are rich in GC content. A 15-nt long subregion of the repetitive segment within the known KLF5 binding site is near perfectly conserved in the upstream region (<10,000 nt) of six different evolutionarily unrelated miRNAs (Figure 4C). In addition, a 16 nt long motif, GGGGCGGGGGCGGGAG, is perfectly conserved between three miRNAs *(miR-433, miR-146b,* and *miR-96);* this motif is located within the first 5 kb upstream of the three miRNAs. Across the human genome, the 16-mer occurs at a frequency of 6.3 instances in every one billion bases. Based on the observed frequency of the motif in the genome, only 0.007 (6.3 × 10^−9^ × 214 × 5,000) of the miRNAs are expected to contain the motif by chance within our dataset. Therefore, the co-occurrence of the motif in three different miRNA sequences is likely relevant to the regulation of their expression patterns. Moreover, an analysis of the location of the GGGGCGGGGGCG motif 50 kb upstream of all protein-coding genes revealed that the motif is preferentially located within the first 200-nt region of the genomic locations of 55 genes (Figure 4D). Additionally, our previous analysis of the location of the subsequence GGGGCG in upstream regions of protein-coding genes also underscores the functional relevance of this motif (Figure S4). Taken together, our study suggests that GGGGCG is involved in the transcriptional control of several miRNAs and numerous protein-coding genes, which are potentially regulated by Sp1 and the KLF family of TFs.

### TF–Motif Interactions Exert Combinatorial Control on miRNA Expression

The predicted *k-*mers are associated with the transcriptional and posttranscriptional regulation of miRNAs. However, the presence of a single *k-*mer in the upstream region of a miRNA is likely insufficient to control miRNA expression. Therefore, we used the cumulative K-Factor score (Methods) to identify specific miRNAs that are regulated by the top 50 hexamer motifs. As a control experiment, an equivalent analysis was performed using 100 sets of 50 control motifs (Methods). Using the representative set of miRNA upstream sequences, we identified 18 miRNAs that are likely regulated by specific CRMs at the cumulative score threshold of 20. In contrast, the control experiment yielded an average of 0.5 sequences (σ = 0.8) at the threshold of 20 (SNR of 36:1). Encouraged by a reasonably high SNR, we extended the analysis to upstream sequences (2 kb) of all known 460 human miRNA genes. The extended analysis predicted 48 miRNAs that are regulated by the predicted CRMs (Table S3).

The predicted TF–CRM and CRM–miRNA interactions enabled us to link known TFs to 48 miRNAs via their common predicted CRMs (Table S3). Among the predicted miRNA–protein interactions, the TFs c-Myb, NF-Y, and Sp-1 are predicted to be involved in the regulation of all 48 miRNAs (Table S4). The aforementioned observation led us to characterize several of the predicted TFs as putative master regulators of miRNA expression (Table S4). We found that combinatorial interactions of several TFs are generally involved in regulating the expression of miRNAs. For example, *miR-132,* previously shown to be differentially upregulated in six solid cancer types (breast, colon, lung, pancreas, prostate, and stomach carcinomas) [77], is predicted to be combinatorially regulated by 24 CRMs. In addition to the upregulated expression of *miR-132* in solid tumors, it is present in embryonic stem cells, and normal brain [78,79]. In cortical neurons, *miR-132* was identified through a genome-wide screen as a transcriptional target of the redox sensitive TF, cAMP-response element binding protein (CREB) [80]. We have predicted the redox sensitive TFs AP-1, AP-2, c-Myb, EGR-1, EGR-2, MTF-1, and Sp-1 as potential TFs of *miR-132* (Table S3). In rat duodenal mucosa, EGR-1 is known to form a molecular complex containing CREB and all six other redox sensitive TFs [81]. Taken together, our results suggest that in addition to CREB, *miR-132* is regulated by coordinated interactions of other redox sensitive TFs.

## Discussion

We developed and applied a new method, K-Factor, to upstream miRNA sequences to identify CRMs that regulate the biogenesis of miRNAs. We extended our analysis to identify candidate TFBSs and TFs that mediate the regulation of specific miRNAs. Our results indicate that miRNA expression is regulated by numerous regulatory elements that frequently occur in multiple copies in the upstream sequences of miRNAs. The preference of the predicted motifs to occur towards the genomic loci of protein-coding genes suggests that transcription of miRNAs and protein-coding genes are controlled by similar factors. The TFs that regulate miRNAs also appear to be numerous. It is conceivable that the dynamic range of expression of miRNAs is a direct outcome of the combinatorial regulation of many transcriptional activators and suppressors rather than the control exerted by one or a few TFs.

We focused our analysis on a set of high-confidence predictions, which likely represent core miRNA regulatory elements, to identify putative TFs that regulate miRNA expression. Our study suggests that a considerable number of disease-associated TFs of protein-coding genes may significantly contribute to the abnormal expression of miRNAs. Although mechanisms such as altered genomic copy numbers of miRNAs [82] and irregularities in the miRNA processing pathways are also known to cause aberrant expression of miRNAs [83], most TFs that we predicted to regulate miRNAs are strongly associated with cancer. Thus, the deregulation of miRNAs by TFs may be a root cause of aberrant miRNA expression in cancer. Additionally, it appears that the transcription of many miRNAs is controlled by coordinated interactions of multiple TFs with miRNA CRMs. Thus, it is conceivable that the broken interactions within the complex network of TF–miRNA regulation will lead to the downregulation or upregulation of miRNAs. Our predictions will thus be a valuable starting point to systematically elucidate the causative basis of aberrant expression patterns of miRNAs in cancer. A focused study of expression of specific sets of miRNAs that are associated with cancer and their predicted TFs will provide valuable insights into cancer progression and miRNA biology. For instance, the most interesting predicted TFs (c-Myb, NF-Y, Sp-1, MTF-1, and AP-2α) can be knocked down via RNAi, and the expression profiles of miRNAs can be measured using a microarray analysis.

We found that a significant fraction of the top predicted sequence motifs for human miRNA genes are also enriched among the upstream miRNA regions in mouse and opossum. We demonstrated that the overlap is highly statistically significant, based on a commonly used hypergeometric model [59]. The significant overlap between the predicted CRMs of the three genomes strongly suggests that the sequence level evolution of the motifs is constrained, an indication that the motifs that we identified are crucial to miRNA biogenesis. We observed that the content of guanine and cytosine nucleotides among the predicted motifs is very high. The percentage of G and C nucleotides within the top 50 hexamers are 48.0% and 42.6%, respectively. In stark contrast to the approximately uniform nucleotide composition of the miRNA upstream sequences (Table S1), the combined proportion of GC base pairs (90%) is significantly higher than the AT base pairs (9.3%) for the predicted hexamer motifs. Similar proportions of GC base pairs were also observed for longer *k-*mer motifs. Moreover, the GC content for the 22 evolutionarily conserved hexamers was 96.2%, higher than the aforementioned ratios. What might be the evolutionary and biological significance of the high proportion of G and C nucleotides among the miRNA regulatory elements? Elevated GC content is a hallmark of transcriptionally active regions of the mammalian protein-coding genes [84]. In addition, evolution of miRNAs in distantly related mammals (human, mouse, and opossum) has preferentially preserved the GC-rich CRMs. Therefore, it appears that the GC-rich miRNA transcriptional regulatory elements are more resistant to mutations. The resistance of these regulatory elements is likely a direct outcome of the sequence specificity of their protein interactors, such as evolutionarily well-conserved TFs that operate in key biological pathways that are conserved across mammals.

We note that the predicted CRMs do not represent all possible motifs that regulate miRNA expression; instead they represent motifs that are identified based on the degree of overrepresentation in miRNA upstream sequences. It is also possible that the some of the upstream sequences used in this study for multiple miRNAs are not representative of their promoter regions. The length of the upstream sequences of miRNAs used in this study to closely estimate the transcriptionally active upstream region of miRNAs is larger than the typical core promoter regions of protein-coding genes. However, due to the lack of a clear understanding of the range of lengths of the miRNA primary transcripts, we chose to err on the length of their putative promoter sequence because we noticed that K-Factor was able to identify biologically relevant motifs in 10-kb–long sequences. The use of 10-kb sequences also provides an opportunity to detect enhancer or silencer elements that may be involved in miRNA expression but are located outside the core promoter region. In addition, the predictions were similar for much shorter miRNA upstream sequences. For instance, among the top 50 hexamers predicted using 10-kb regions, 38 motifs were identical to that of motifs predicted using 1-kb upstream sequences of non-intronic miRNAs. However, errors can occur due to intronic or intergenic miRNAs that may be transcribed along with their host or neighboring genes. There is currently no foolproof method to avoid such errors because it is not clear whether certain miRNAs are transcribed as autonomous units in certain circumstances (e.g., leaky polyadenylation signals of neighboring genes [40]) and as part of larger polycistronic transcripts [85] in other circumstances. In addition, CRMs can be located several thousand nucleotides away from miRNA sequences and do not need to be present within the same strand of DNA nor in a contiguous stretch of DNA. Despite such shortcomings that have no clear immediate resolution, we have been able to predict novel motifs that are biologically relevant to the overall expression of miRNAs and protein-coding genes.

The TFs that may direct the transcription of miRNAs via the predicted CRMs were identified by matching CRMs to known TFBSs. At a reasonably high SNR (36:1), we identified candidate TFs that bind specific regulatory elements. In particular, we identified an unusual 12-nt long tandem repeat of GGGGCG, that likely is bound by the family of KLF TFs. KLFs and Sp1-like proteins are a family of highly related zinc finger proteins that are fundamental to the eukaryotic cellular transcriptional machinery [86]. Individual members of the family can function as transcriptional activators or repressors, depending on the promoters they bind and the coregulators with which they interact. Such switches between activator and repressor states impose additional complexity on understanding the transcriptional regulation of miRNAs. The example of KLF5/Sp-1 TFs highlights the combinatorial interplay between different CRMs that will likely determine the tissue specific expression of miRNAs. Thus, although a simple correlation between global miRNA expression patterns and their regulatory motifs is highly desired, it may not be easily attained. However, if accurate miRNA and TF expression profiles are known in different cell types, it may be possible to use machine learning methods to understand the functional synergy between the expression patterns of miRNAs, their CRMs, and their TFs.

The complex interlinks between expression patterns of miRNAs, their CRMs, and their TFs are apparent in the transcriptional control of *miR-132*. We have identified 24 CRMs that may regulate the expression of *miR-132*. Why does such a large number of CRMs regulate *miR-132*? Sequence similarity searches indicate that *miR-132* is well-conserved across several vertebrate genomes [87]. In mammals, *miR-132* is expressed in embryonic stem cells, cortical neurons, and is aberrantly regulated in several types of cancer. In zebrafish, *miR-132* is expressed in adult female, caudal fin, and liver epithelium [88]. In addition to its known function of regulation of neuronal morphogenesis [80], *miR-132* is predicted to downregulate more than 200 genes [19,26]. Taken together, it appears that *miR-132* is a functionally important gene whose expression must be activated or repressed in several cell types. Our results suggest that the expression of *miR-132* is fine-tuned by the combinatorial interactions of several coexpressed TFs. The expression of *miR-132* in mammalian brain appears to be regulated by eight coexpressed, redox sensitive TFs. Thus, it seems that the observed dynamic range of miRNA expression is an outcome of the combinatorial interaction of multiple TFs to coordinately and selectively activate or repress miRNAs.

In this study we analyzed the sequence elements and their protein interactors involved in the transcriptional and posttranscriptional regulation of miRNAs using a novel method, K-Factor. The central difference between K-Factor and many other computational methods used for the discovery of regulatory motifs is that K-Factor uses a species specific model to predict the most biologically relevant motifs. Namely, our method relies on the random extraction of sequences from the genome of interest. The accuracy of the predictions was estimated using three different control sets. The observation that the majority of the predicted motifs are preferentially located towards the transcription start sites of protein-coding genes further reinforces our findings. The K-Factor method can be extended to predict regulatory motifs in any set of biological sequences of interest. The K-Factor score is a ratio that provides a simple intuitive awareness of the degree of enrichment of a given *k*-mer in a set of biologically related sequences, with respect to a random sample of sequences from the corresponding genome. Thus, a score of 1.0 for a given *k*-mer is generally an indication that the occurrence of the *k*-mer in a given set of input sequences is as frequent as in the random sample. In contrast, a K-factor score of 3.0 suggests that, in comparison with the background evolution of the *k*-mer in the genome, the motif is approximately three times more overrepresented in the input set of sequences. A preliminary java-based, platform-independent version of K-Factor that can identify regulatory motifs in any functionally related sequences is available for download (http://www.johnlab.org/k_factor.html).

Identification of miRNA targets in the 3′ UTR of protein-coding genes could be a useful extended application of K-Factor. If miRNAs extensively mediate regulation of protein-coding genes via the 3′ UTR of the corresponding messenger RNAs (mRNAs), as it is widely believed to be, it is possible that the overrepresented motifs in 3′ UTRs are enriched in miRNA binding sites. Additionally, the analysis of the 3′ UTRs of genes involved in processes such as development, where miRNA expression is strongly observed, may also lead to clearly overrepresented motifs that are preferentially complementary to miRNAs. We hope to conduct similar analyses in the near future.

## Materials and Methods

### miRNA upstream sequences.

Human pre-miRNA sequences were downloaded from miRBase (March 2006) [87]. To eliminate sequence compositional bias introduced by evolutionarily related miRNAs, a subset of representative pre-miRNA sequences that shared no more than 80% sequence identity to other sequences in the set was curated. The representative dataset was mapped onto the human genome (NCBI build 35) to extract 10,000 nucleotides upstream of the miRNA genes. In cases where miRNA upstream regions overlapped due to proximally located miRNAs, we randomly retained one representative miRNA upstream region. The final dataset contained 214 human miRNA upstream sequences.

All known human pre-miRNA sequences were scanned using BLAST [89] (E-value < 10^−10^) against the genomes of mouse (Mus musculus) and opossum (Monodelphis domestica) to identify potential homologous pre-miRNAs. Potential homologs were selected based on the most significant BLAST hit. Representative mouse and opossum pre-miRNAs and their nonoverlapping upstream sequences were subsequently extracted based on the procedure used for human pre-miRNAs. The number of upstream sequences identified in mouse and opossum genomes was 175 and 100, respectively.

### Identification of upstream miRNA-specific sequence motifs.

The K-Factor computational method is designed to identify regulatory sequences of length *k* (*k*-mers) that are embedded in a given set of user-defined DNA sequences, **S**, of a specific genome, *G*. For a given number of sequences (*n_S_*) in the input set **S**, K-Factor executes the following steps. (1) *m* (*m* = 100) sets of sequences (reference sets **R*_i_***; *i* = 1 .. *m*) are extracted from random locations in *G*. Each reference set in **R** consists of *n_S_* sequences that have length distribution that is identical to sequences in **S**. (2) The normalized frequency of all possible *k*-mers in **S** and each of the *m* sets in **R** are calculated. For a given *k*-mer (*k_i_*), the normalized frequency, *f*(*k_i_*,*G*), is computed as the ratio of the number of occurrences of *k_i_* in a given set to the total number of nucleotides in the given set. The number of occurrences of *k_i_* is calculated by counting the number of nonoverlapping instances of *k_i_* in each sequence. The number of sequences, *N*(*k_i_*,*G*), that contain *k_i_* is also determined. (3) Enrichment scores that measure the bias of each *k*-mer to preferentially occur in **S** with respect to each reference set in **R** are determined. The enrichment score for *k_i_* in **S** with respect to **R*_i_*** is defined as the ratio of *f*(*k_i_,G,* **S**) to *f*(*k_i_,G,* **R*_i_***). (4) K-Factor score *K(k_i_,G,* **S**) of each *k_i_* for **S** is computed as the average enrichment score of *k_i_* over all *m* sequence sets in **R**. (5) Two different Z-scores, *Z^f^*(*k_i_,G,S*) and *Z^N^*(*k_i_,G,S*) for each *k_i_* in **S** are calculated based on the average and standard deviation of *f*(*k_i_,G*) and *N*(*k_i_*,*G*) in **R**, respectively. (6) *k*-mer sequences above a predefined threshold of K-Factor score and Z-scores (*Z^f^, Z^N^* ≥ 7.0) are predicted as regulatory elements in **S**. The Z-score cutoffs were chosen so that the observed difference between the occurrence of *k_i_* in **S** and its average distribution in **R** is statistically significant (one-tailed *p*-value ≤ 10^−10^). For each *k-*mer in **S**, *K*(*k_i_,G,* **S**) can be calculated as:

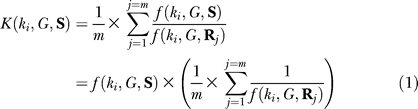



The product in parenthesis corresponds to the average of the reciprocal frequencies of *k_i_*, for a given genome (Equation 1). To increase the speed of the K-Factor algorithm, the average can be precomputed for each genome.

### Conservation of motifs in orthologous species.

Predicted regulatory sequence motifs in the upstream regions of human, mouse, and opossum miRNAs were analyzed to identify common *k*-mers that may be important for miRNA biogenesis. The significance of overlap of the most enriched *k*-mers between the two species was analyzed using a hypergeometric distribution. The probability of observing at least *n_c_* common *k*-mers between two independent sets of DNA sequence motifs that contain *n_1_* and *n_2_ k*-mers in each was calculated as:

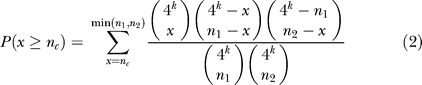



### Prediction of transcription factor–motif interaction.

The predicted hexamer motifs were matched using their full sequences against known human TFBS in the TRANSFAC database [65]. To reduce the number of false matches that may occur by chance, we required that at least three predicted motifs matched a given TFBS. The proteins or protein complexes that bind to the matched binding sites were considered as candidate TFs to regulate miRNAs via the predicted CRM sequences.

### Cumulative K-Factor score.

We devised a strategy to identify specific miRNA sequences that were likely to be regulated by the predicted sequence motifs within the given input set **S**. First, we selected input sequences and CRMs so that the motifs occurred at least twice per 2 kb of each selected sequence, **S_i_**. For each selected CRM (*k_j_*), K-Factor scores *K*(*k_j_,G,* **S_i_**) were determined. Next, a cumulative score that incorporated the combinatorial interaction between selected CRMs that occur in **S_i_** was calculated. The cooperative score for **S_i_** was formulated as the sum of the natural logarithm of *K*(*k_j_,G,* **S_i_**) over all selected CRMs. Finally, sequences for which the cumulative score was above a predefined threshold were selected.

### Control sequences.

We used three different methods to generate control sequences to ensure reliability in the assessment of our predictions. Each control set was designed to precisely match the observed length distribution of the 214 human pre-miRNA upstream sequences. Each method generated 100 distinct sequence sets that contained a total of 21,400 sequences (100 × 214). In the first method, UPS, 100 sets of control sequences were generated using immediate UPSs of genes that were randomly selected from a list of 21,118 protein-coding genes [90]. In the second method, RGS, the repeat masked genome sequence devoid of known repeat elements was used to randomly extract control sequences (http://hgdownload.cse.ucsc.edu/goldenPath/hg18/database/). Similarly, in the third method, GS, sequences were generated by the extraction of sequences from random locations of the complete unmasked genome sequence. K-Factor was applied to each of the 300 control sets, using the same reference sets **(R)** that were used to identify candidate CRMs in upstream regions of human miRNAs. The results for UPS, RGS, and GS were analyzed separately.

### Signal to noise ratio.

The SNR was calculated as the ratio of the number of predictions (*N_p_*) obtained for miRNA upstream sequences, to the average number (μ) of predictions for 100 control sequence datasets. The standard deviation (σ) for the number of predictions for the control sequence datasets was also determined. The observed signal was considered to be statistically significant (one-tailed *p*-value of ≤0.001), if *N_p_* was at least 3.2σ units larger than μ. The control experiments involved the identification of: (1) K-Factor predicted motifs in each of the 100 control datasets, based on several threshold scores; (2) TF–motif interactions, based on the top 22 K-Factor predicted motifs of each control set; and (3) sequences that yielded cumulative K-factor scores greater than a predefined threshold.

### Control motifs.

Control motifs were generated to closely mimic the predicted motifs by extracting *k-*mers that matched the frequency of predicted motifs in the upstream regions of miRNAs. The frequencies were matched within a marginal difference of 1%.

## Supporting Information

Figure S1 Locations of the Top 100 Predicted 9-mers in the Upstream Regions of Protein-Coding Genes (1.3 MB PDF)

Figure S2 Locations of the Top 100 Predicted 8-mers in the Upstream Regions of Protein-Coding Genes (1.3 MB PDF)

Figure S3 Locations of the Top 100 Predicted 7-mers in the Upstream Regions of Protein-Coding Genes (1.4 MB PDF)

Figure S4 Locations of the Top 100 Predicted 6-mers in the Upstream Regions of Protein-Coding Genes (1.5 MB PDF)

Figure S5 Locations of All (14) Predicted 5-mers in the Upstream Regions of Protein-Coding Genes (219 KB PDF)

Figure S6 Locations of 100 Randomly Selected 9-mers in the Upstream Regions of Protein-Coding Genes (1.5 MB PDF)

Figure S7 Locations of 100 Randomly Selected 8-mers in the Upstream Regions of Protein-Coding Genes (1.5 MB PDF)

Figure S8 Locations of 100 Randomly Selected 7-mers in the Upstream Regions of Protein-Coding Genes (1.5 MB PDF)

Figure S9 Locations of 100 Randomly Selected 6-mers in the Upstream Regions of Protein-Coding Genes (1.5 MB PDF)

Figure S10 Locations of 14 Randomly Selected 5-mers in the Upstream Regions of Protein-Coding Genes (233 KB PDF)

Table S1 Single Nucleotide Frequencies in the Various Datasets (9 KB PDF)

Table S2 miRNA Genes That Contain at Least Three Occurrences of the Motif, CGCGCG within a Contiguous Stretch of 2 kb in Their Upstream Regions (<10 kb) (34 KB PDF)

Table S3 Hexamer Motifs and TFs That Are Predicted to Regulate Specific miRNAs (16 KB PDF)

Table S4 A Prioritized List of TFs That Regulate miRNAs (10 KB PDF)

## Abbreviations

CRMs: cis-regulatory motifs
GS: unmasked genome sequences
kb: kilobases
KLF5: Kruppel-like factor 5
miRNA: microRNA
nt: nucleotide
pre-miRNA: precursor miRNA
pri-miRNA: primary miRNA
RGS: repeat masked genome sequences
SNR: signal to noise ratio
TF: transcription factor
TFBS: transcription factor binding sites
UPS: upstream protein-coding sequences
