# Supplementary material for: Regulatory Circuit of Human MicroRNA Biogenesis
Source: PLoS Comput Biol. 2007 Apr 20;3(4):e67. doi: 10.1371/journal.pcbi.0030067 (PMC1853126; doi:10.1371/journal.pcbi.0030067)
Supplement: Supplementary file 1 [file pcbi.0030067.sg001.pdf]

The 100 top-predicted 9-mers are preferentially located upstream of protein-coding genes (pages 2-101). Each graph is a histogram that represents the number of occurrences of each motif (every 200 nt stretch) in the first 50 kb upstream region of protein-coding genes. The motifs preferentially occur within the first 500 nt region (-500 to 0 nt region) that is directly upstream of the protein-coding genes.

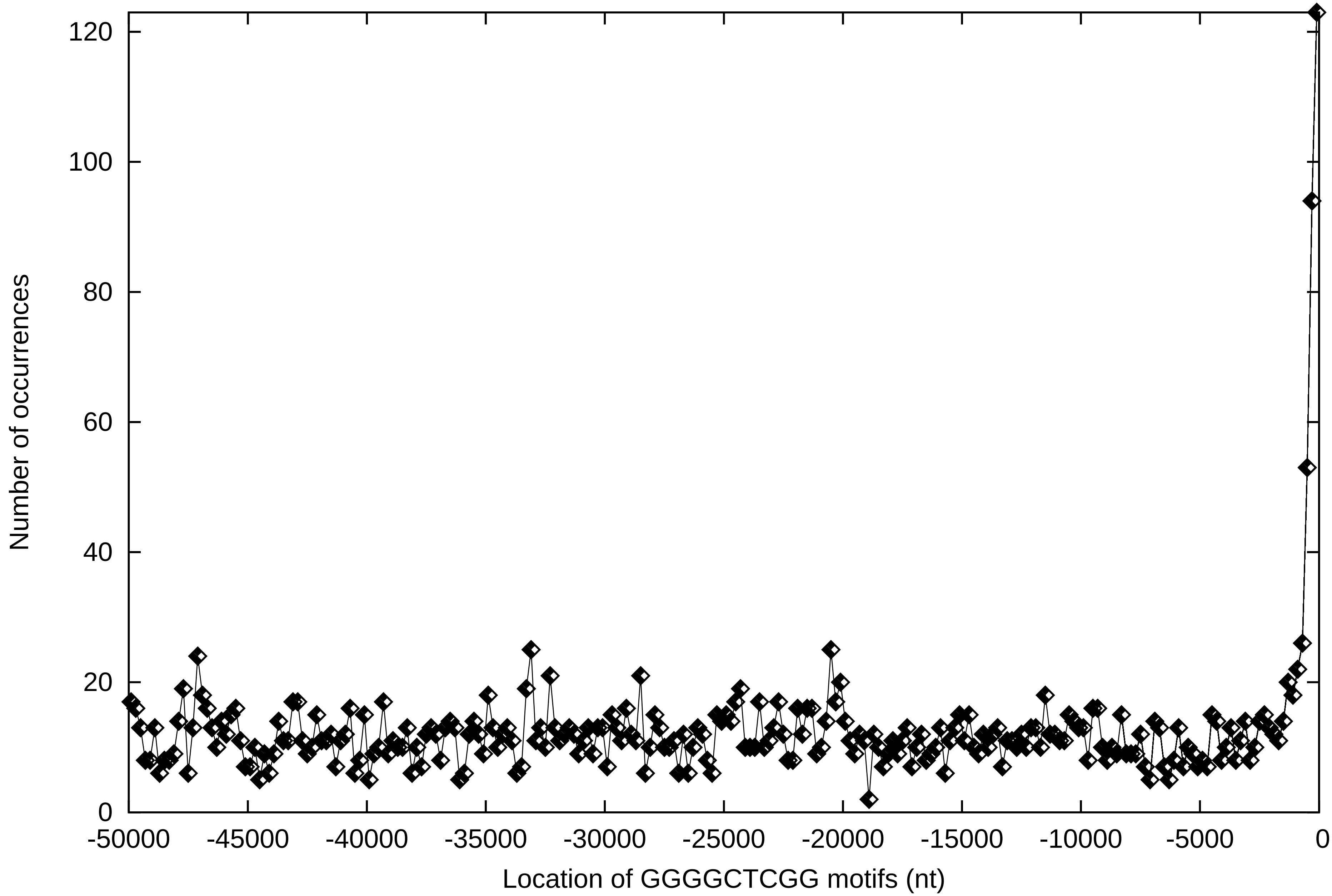

Number of occurrences
0
20
40
60
80
100
120
-50000
-45000
-40000
-35000
-30000
-25000
-20000
-15000
-10000
-5000
0
Location of GGGGCTCGG motifs (nt)

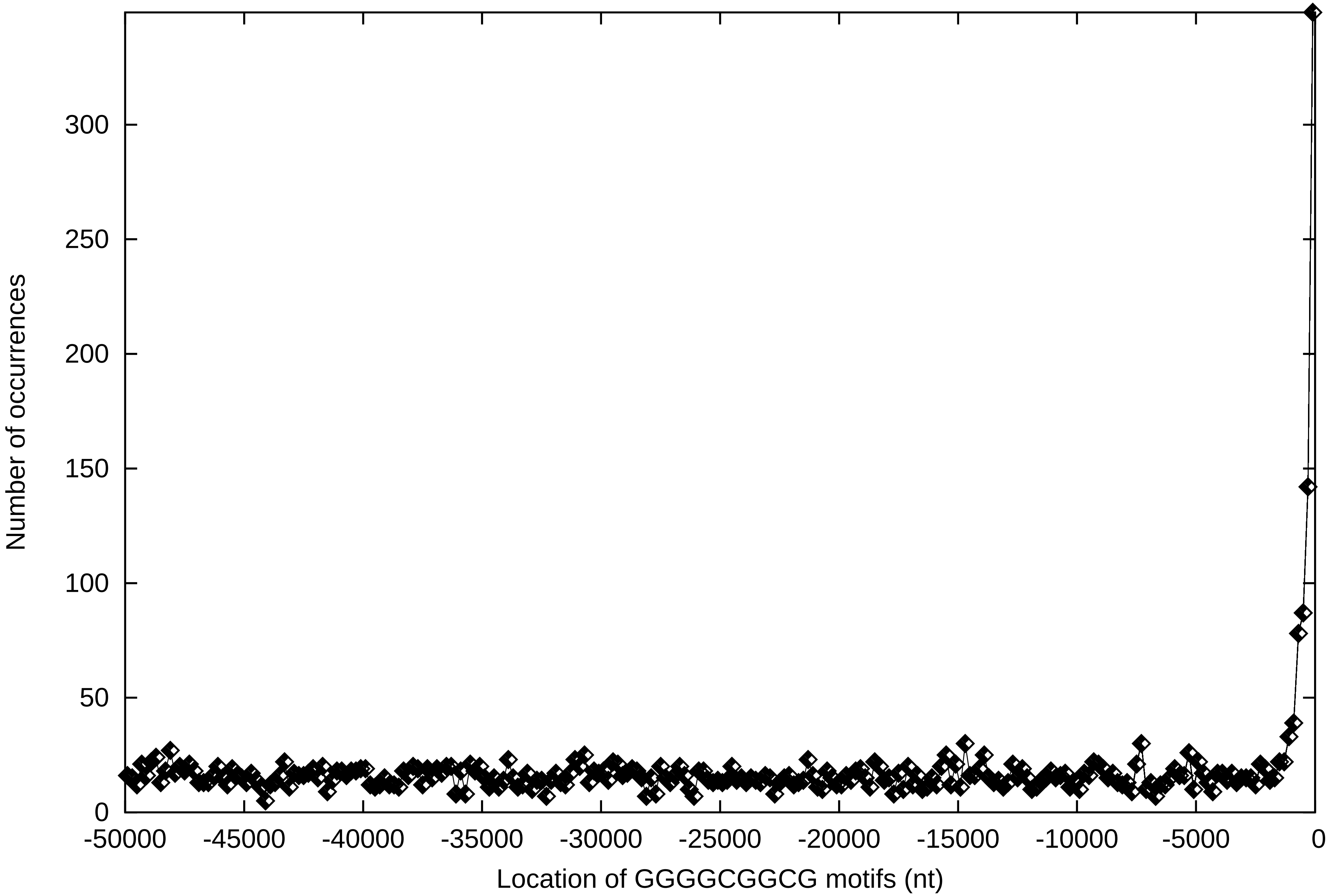

Number of occurrences
0
50
100
150
200
250
300
-50000
-45000
-40000
-35000
-30000
-25000
-20000
-15000
-10000
-5000
0
Location of GGGGCGGCG motifs (nt)

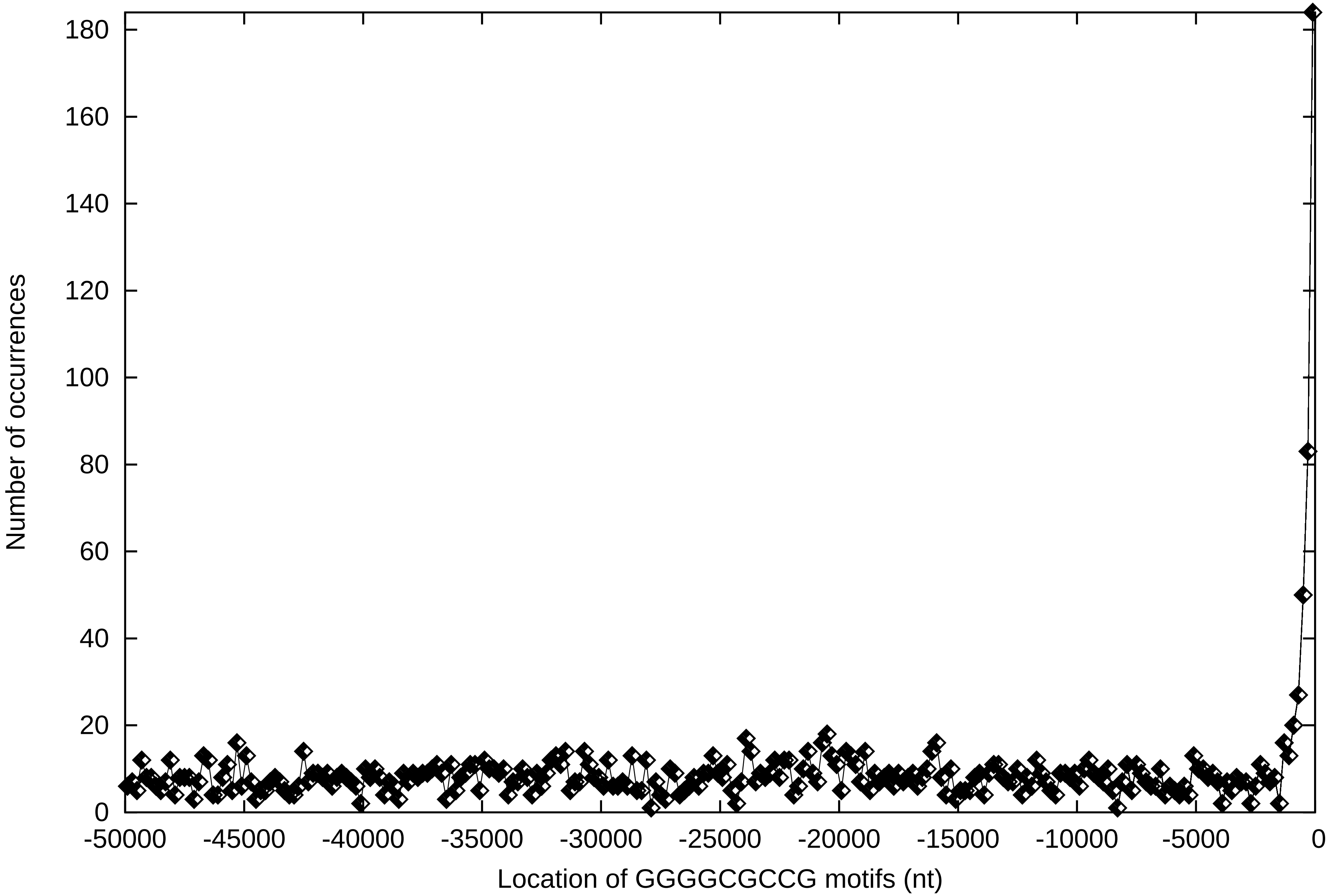

Number of occurrences
0
20
40
60
80
100
120
140
160
180
-50000
-45000
-40000
-35000
-30000
-25000
-20000
-15000
-10000
-5000
0
Location of GGGGCGCCG motifs (nt)

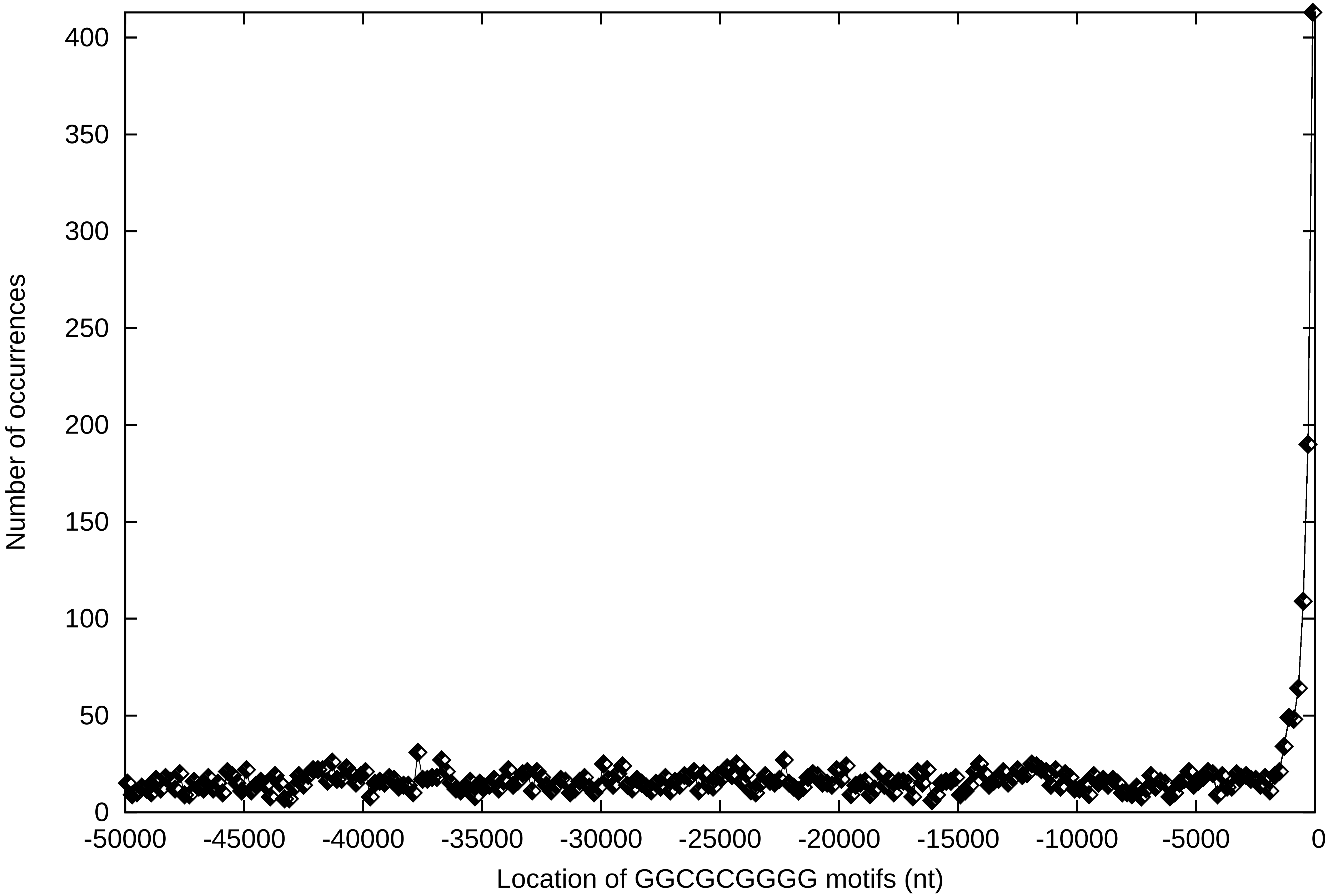

Number of occurrences
0
50
100
150
200
250
300
350
400
-50000
-45000
-40000
-35000
-30000
-25000
-20000
-15000
-10000
-5000
0
Location of GGCGCGGGG motifs (nt)

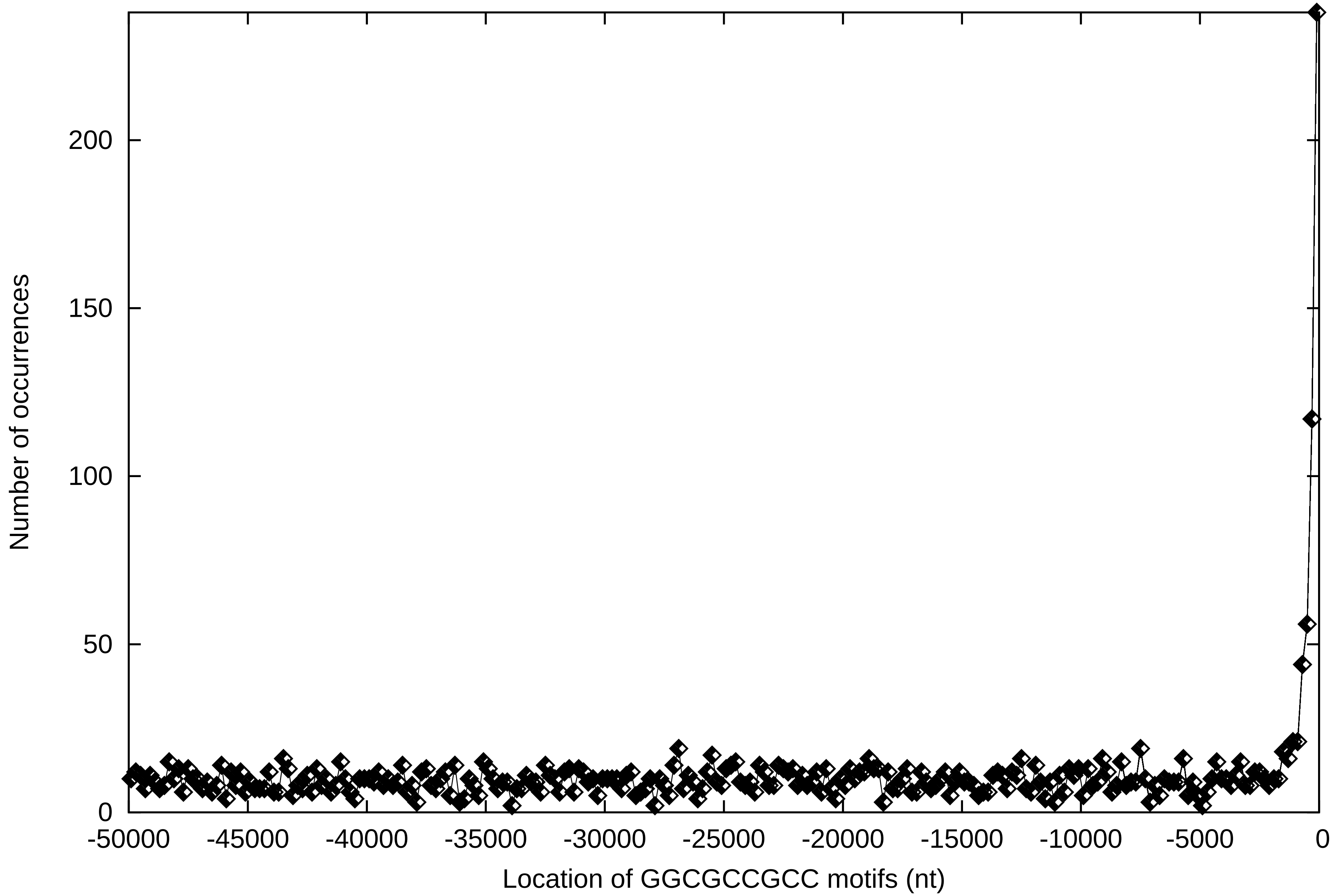
Number of occurrences
0
50
100
150
200
-50000
-45000
-40000
-35000
-30000
-25000
-20000
-15000
-10000
-5000
0
Location of GGCGCCGCC motifs (nt)

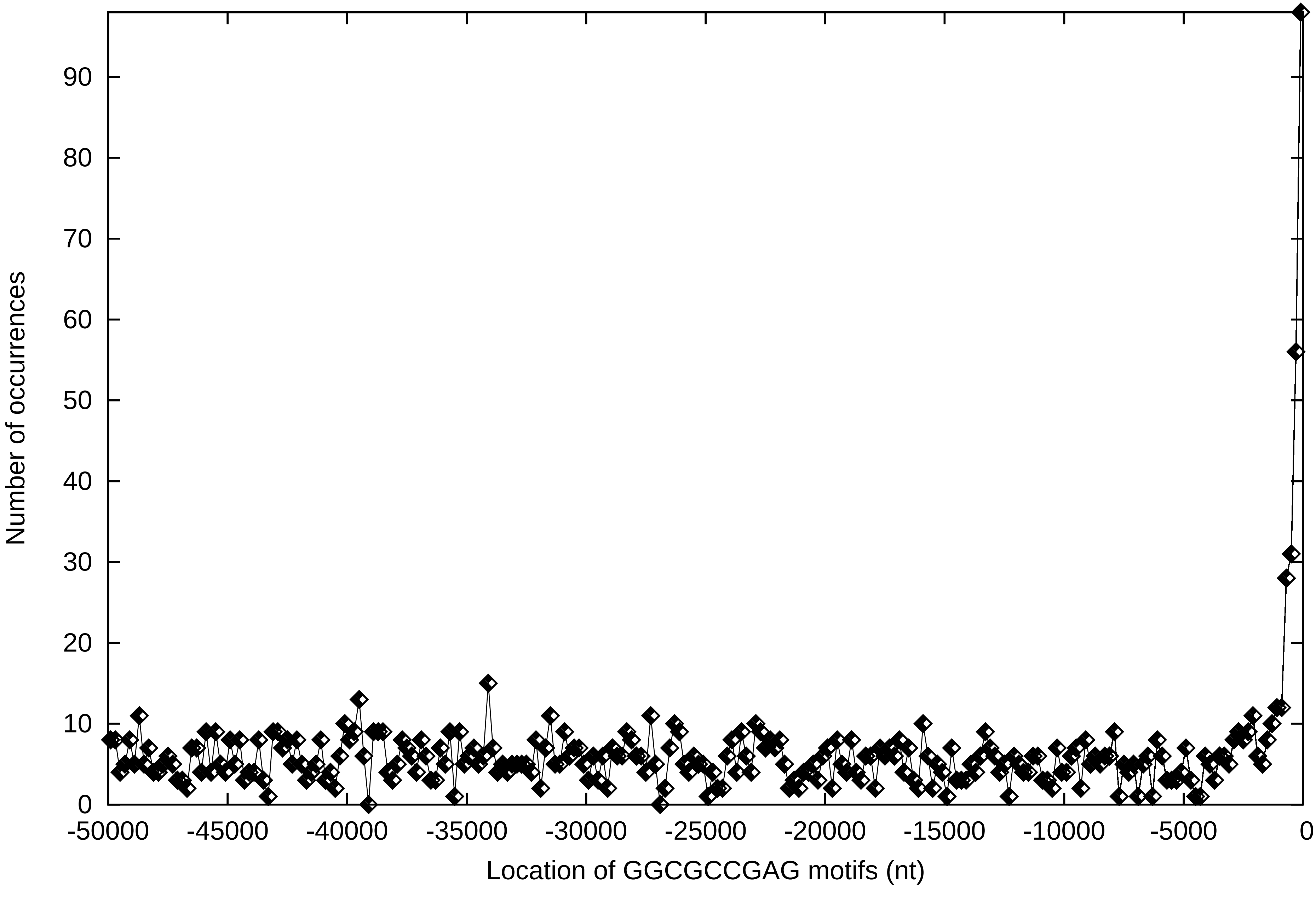

Number of occurrences
Location of GGCGCCGAG motifs (nt)
0
10
20
30
40
50
60
70
80
90
-50000
-45000
-40000
-35000
-30000
-25000
-20000
-15000
-10000
-5000
0

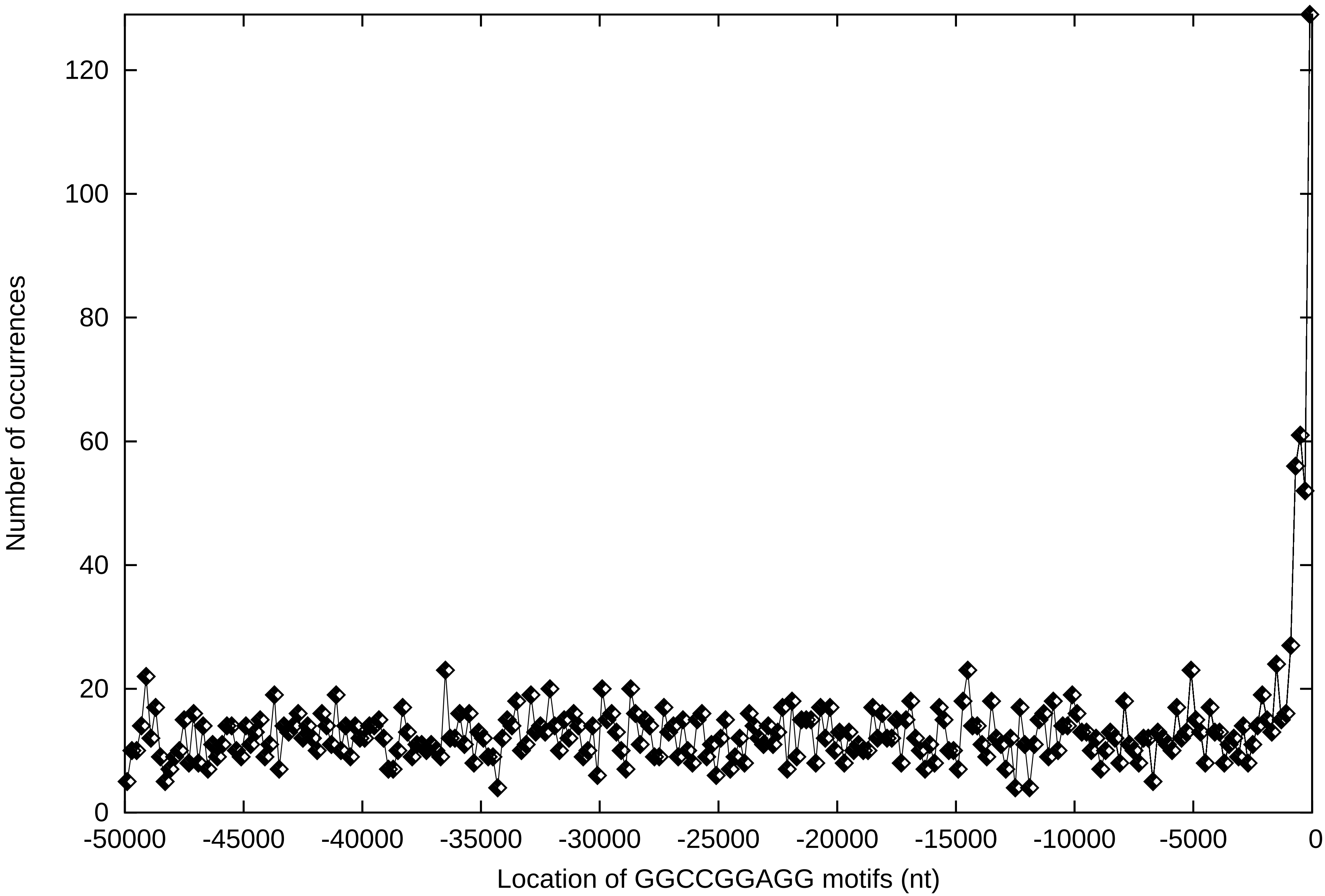

Number of occurrences
Location of GGCCGGAGG motifs (nt)
0
20
40
60
80
100
120
-50000
-45000
-40000
-35000
-30000
-25000
-20000
-15000
-10000
-5000
0

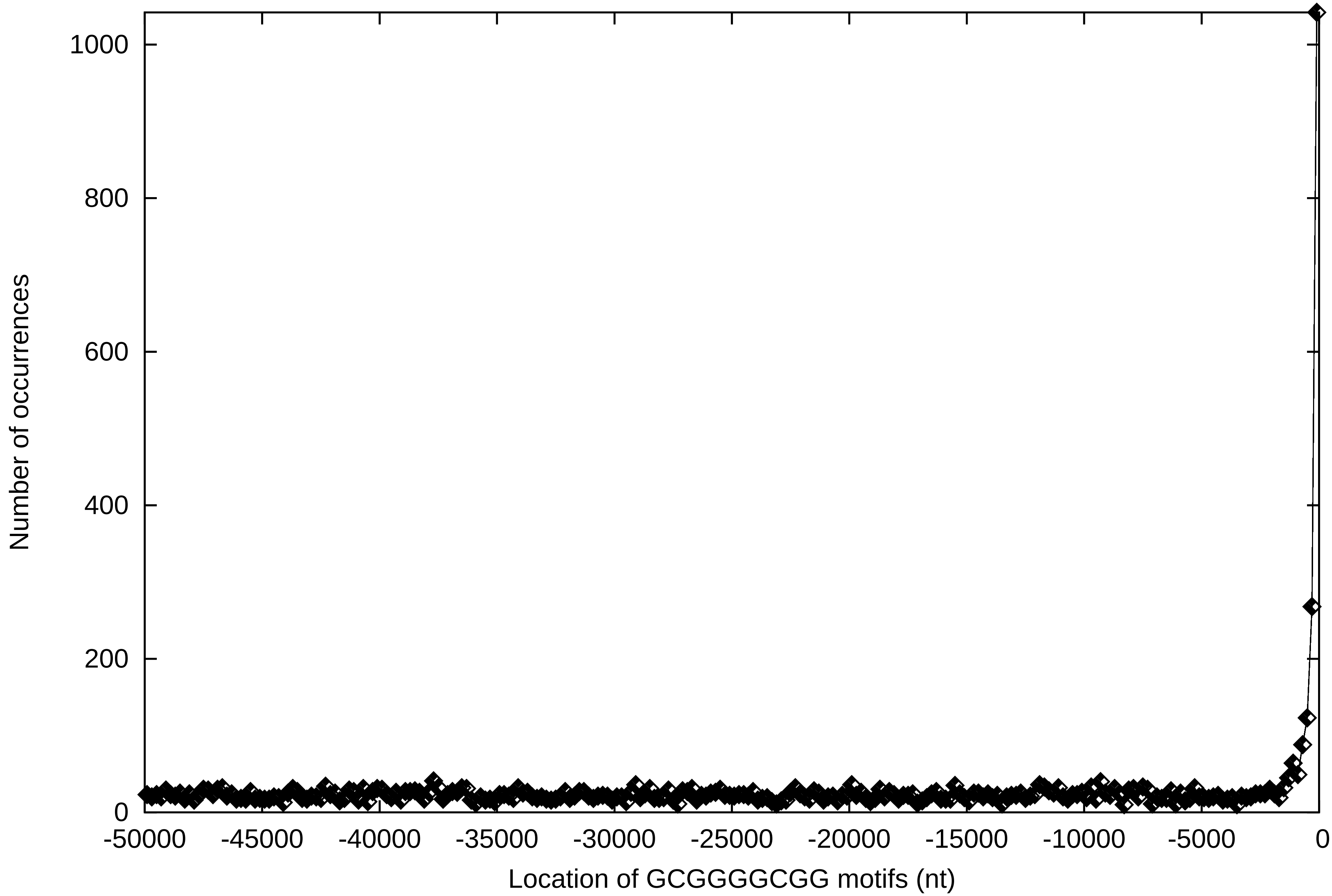

Number of occurrences
0
200
400
600
800
1000
-50000
-45000
-40000
-35000
-30000
-25000
-20000
-15000
-10000
-5000
0
Location of GCGGGGCGG motifs (nt)

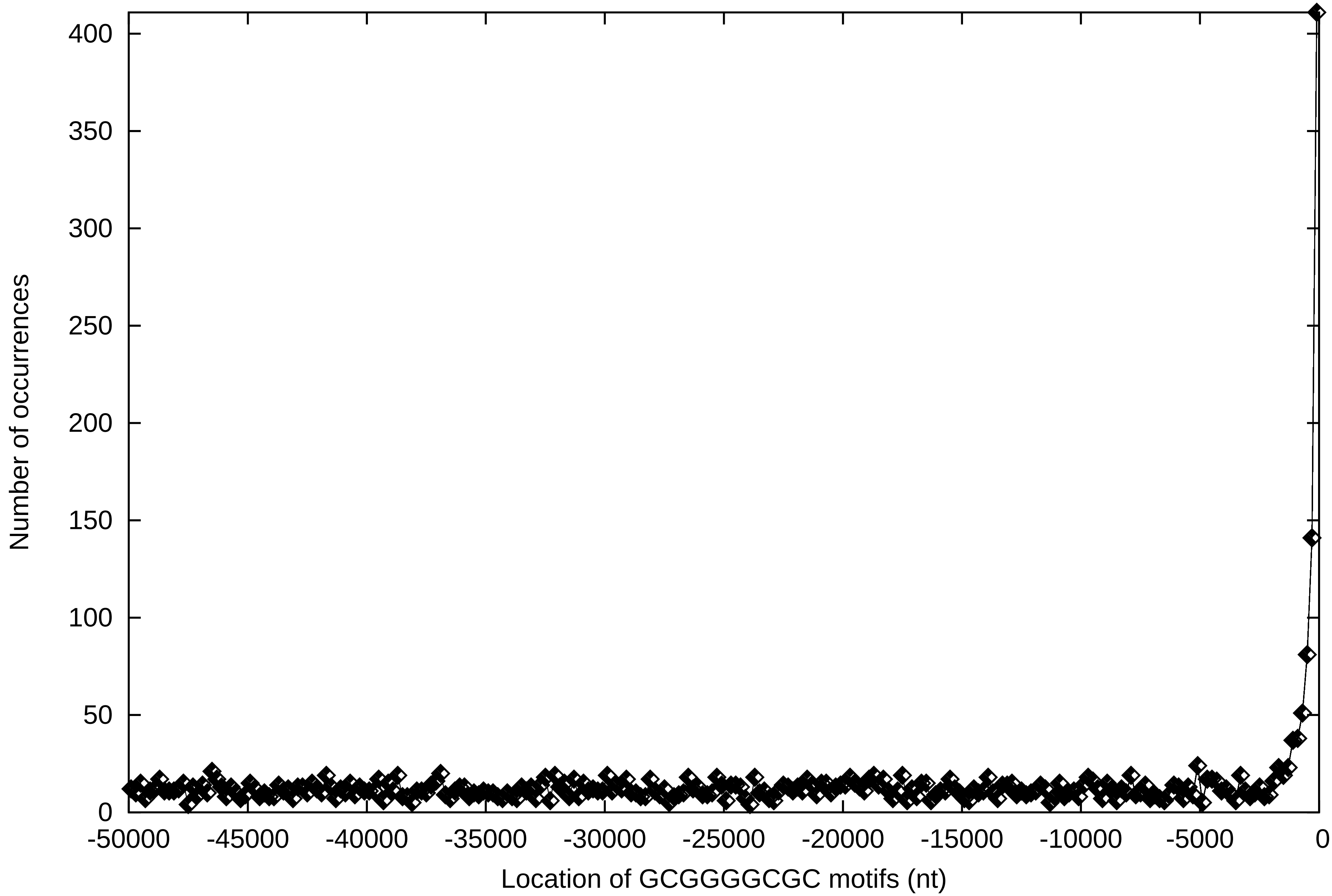

Number of occurrences
0
50
100
150
200
250
300
350
400
-50000
-45000
-40000
-35000
-30000
-25000
-20000
-15000
-10000
-5000
0
Location of GCGGGGCGC motifs (nt)

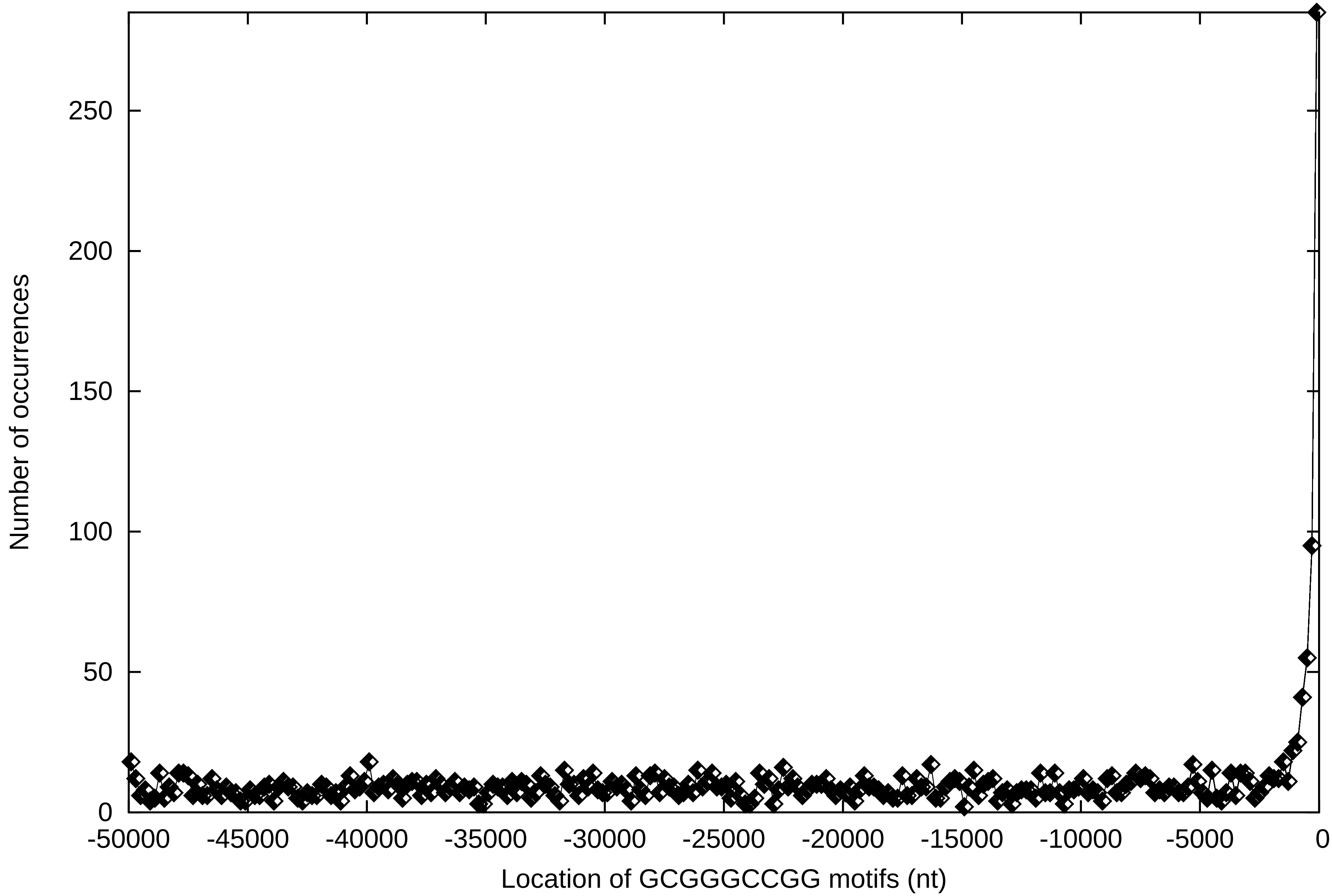

250
200
150
100
50
0
-50000
-45000
-40000
-35000
-30000
-25000
-20000
-15000
-10000
-5000
0
Number of occurrences
Location of GCGGGCCGG motifs (nt)

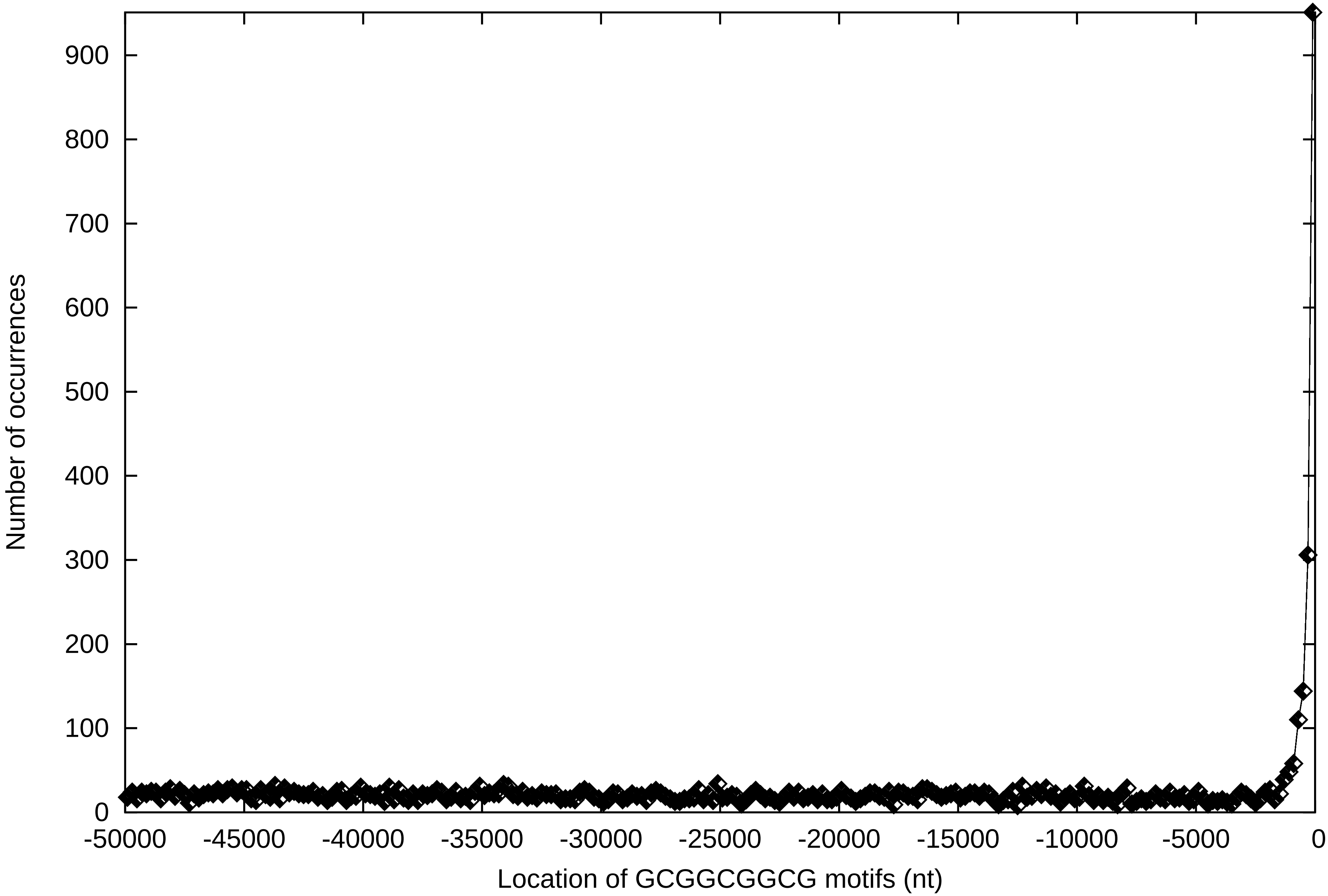

Number of occurrences
0
100
200
300
400
500
600
700
800
900
-50000
-45000
-40000
-35000
-30000
-25000
-20000
-15000
-10000
-5000
0
Location of GCGGCGGCG motifs (nt)

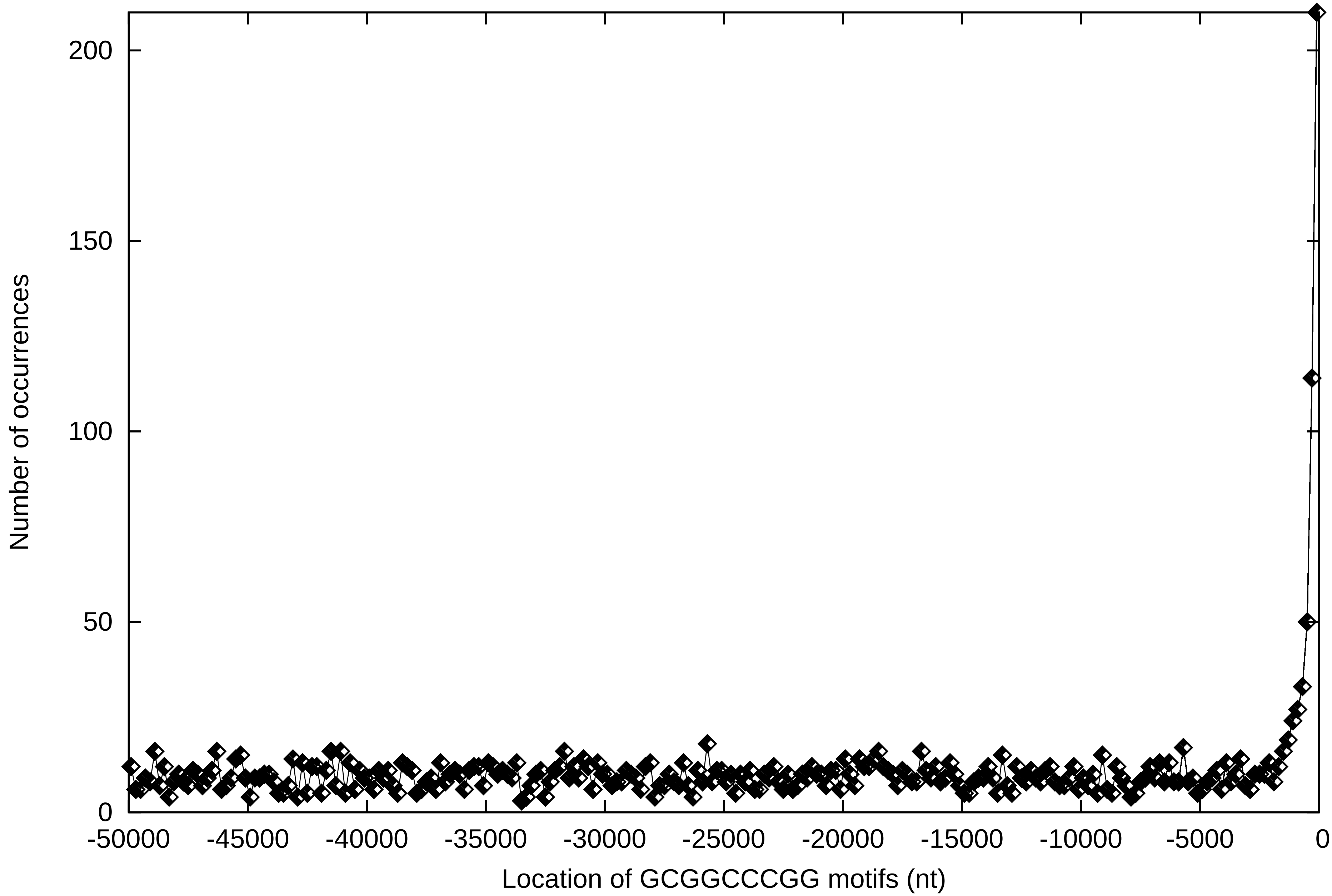

0
50
100
150
200
Number of occurrences
-50000
-45000
-40000
-35000
-30000
-25000
-20000
-15000
-10000
-5000
0
Location of GCGGCCCGG motifs (nt)

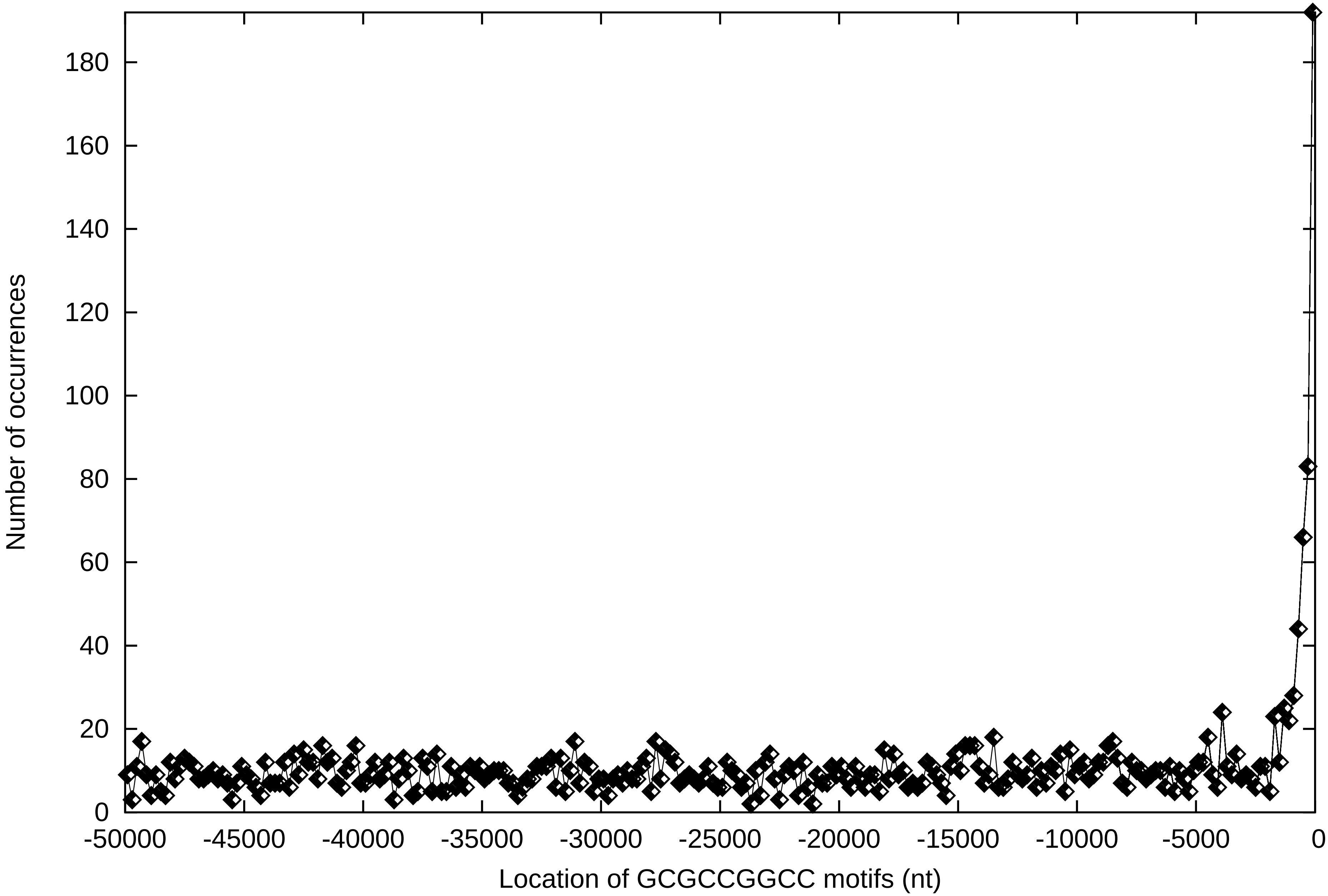

Number of occurrences
0
20
40
60
80
100
120
140
160
180
-50000
-45000
-40000
-35000
-30000
-25000
-20000
-15000
-10000
-5000
0
Location of GCGCCGGCC motifs (nt)

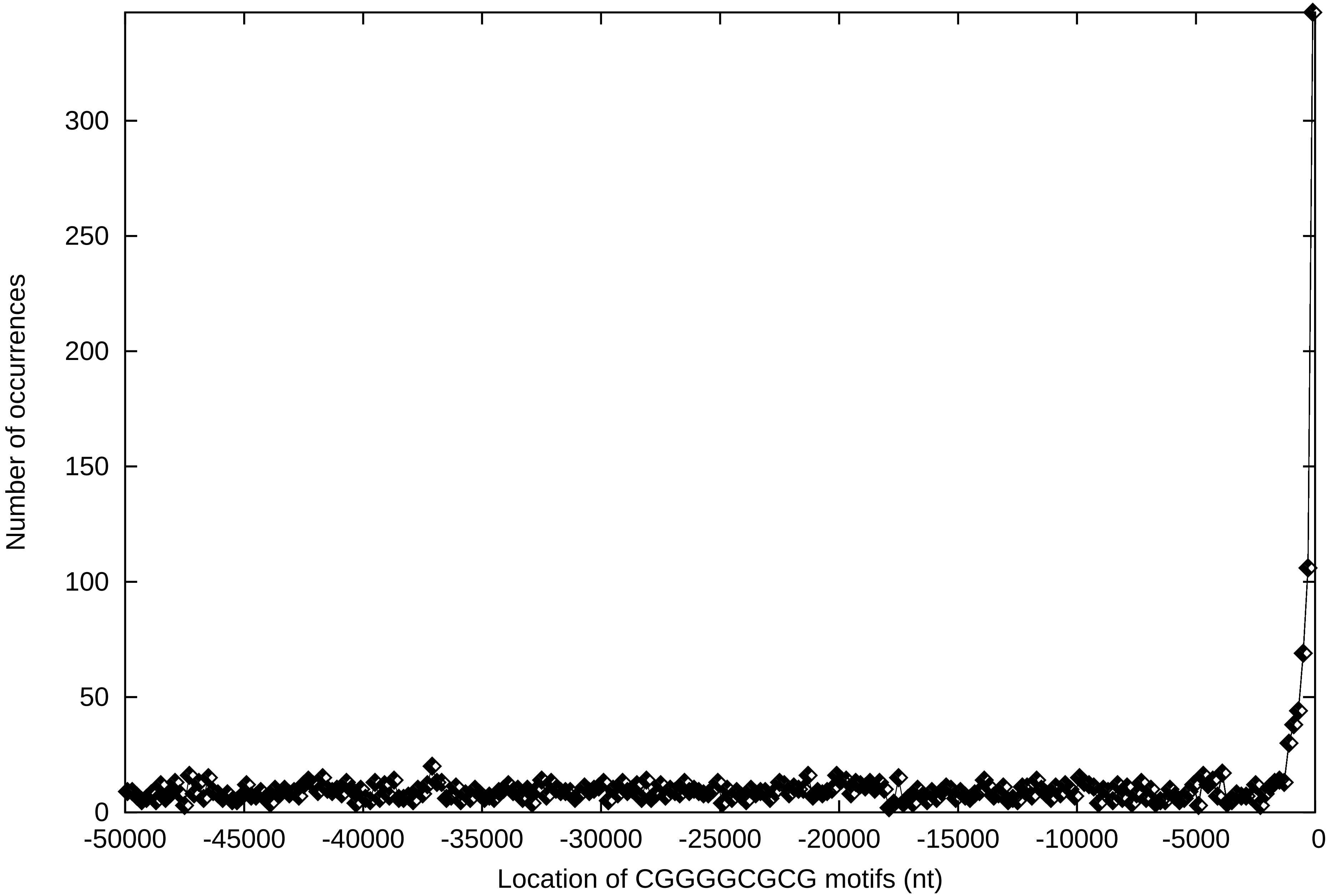

Number of occurrences
300
250
200
150
100
50
0
-50000
-45000
-40000
-35000
-30000
-25000
-20000
-15000
-10000
-5000
0
Location of CGGGGCGCG motifs (nt)

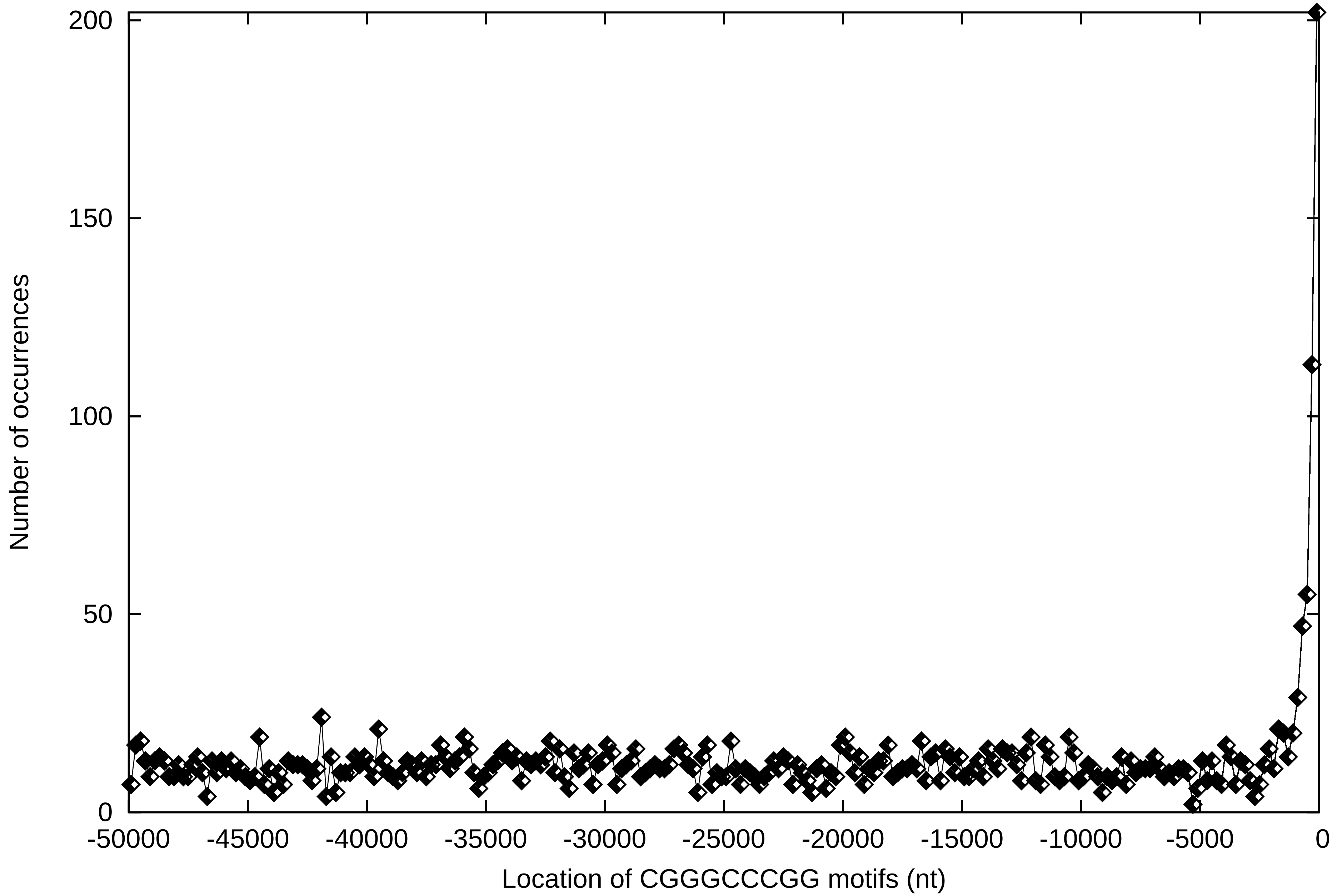
Number of occurrences
200
150
100
50
0
-50000
-45000
-40000
-35000
-30000
-25000
-20000
-15000
-10000
-5000
0
Location of CGGGCCCGG motifs (nt)

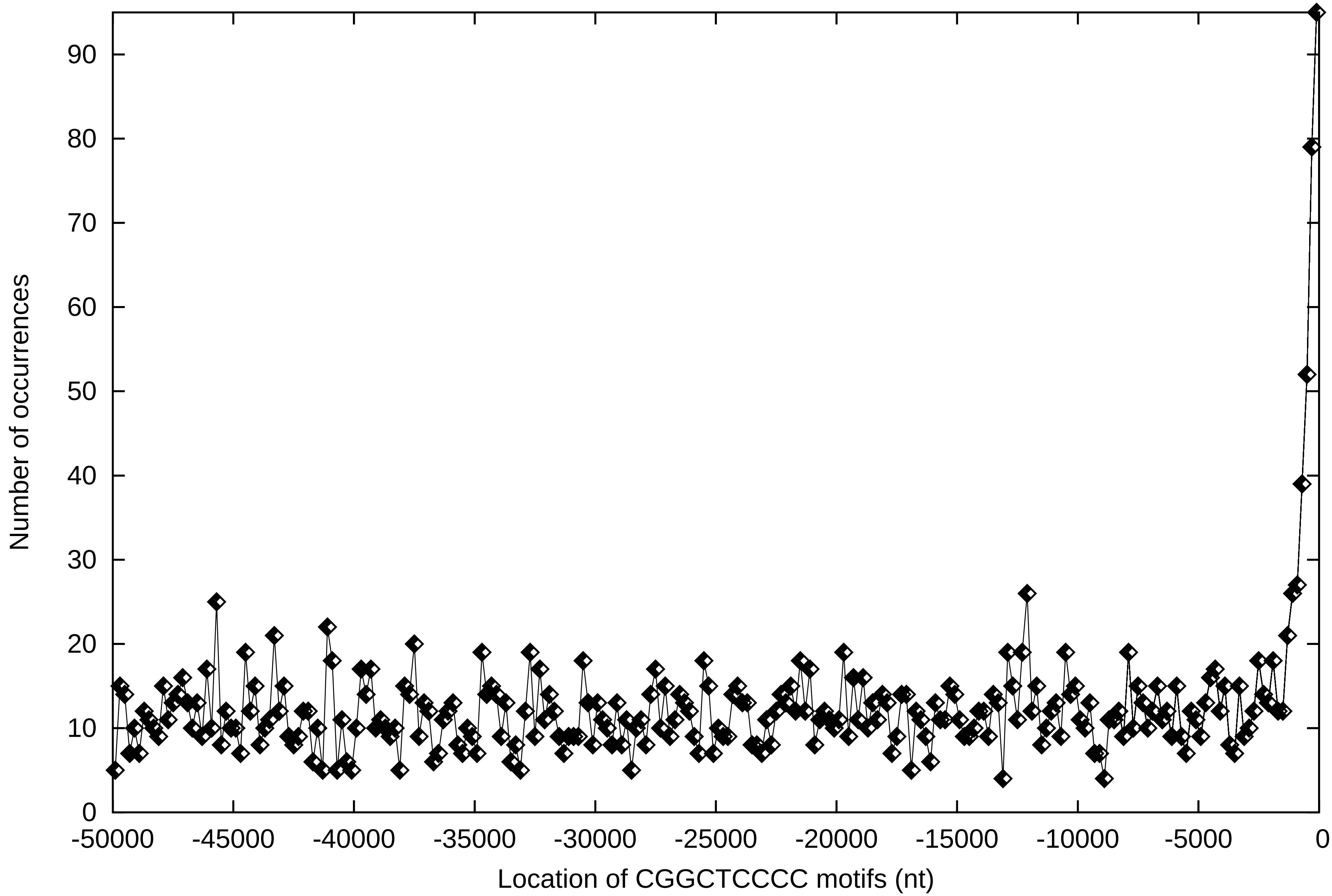

Number of occurrences
0
10
20
30
40
50
60
70
80
90
-50000
-45000
-40000
-35000
-30000
-25000
-20000
-15000
-10000
-5000
0
Location of CGGCTCCCC motifs (nt)

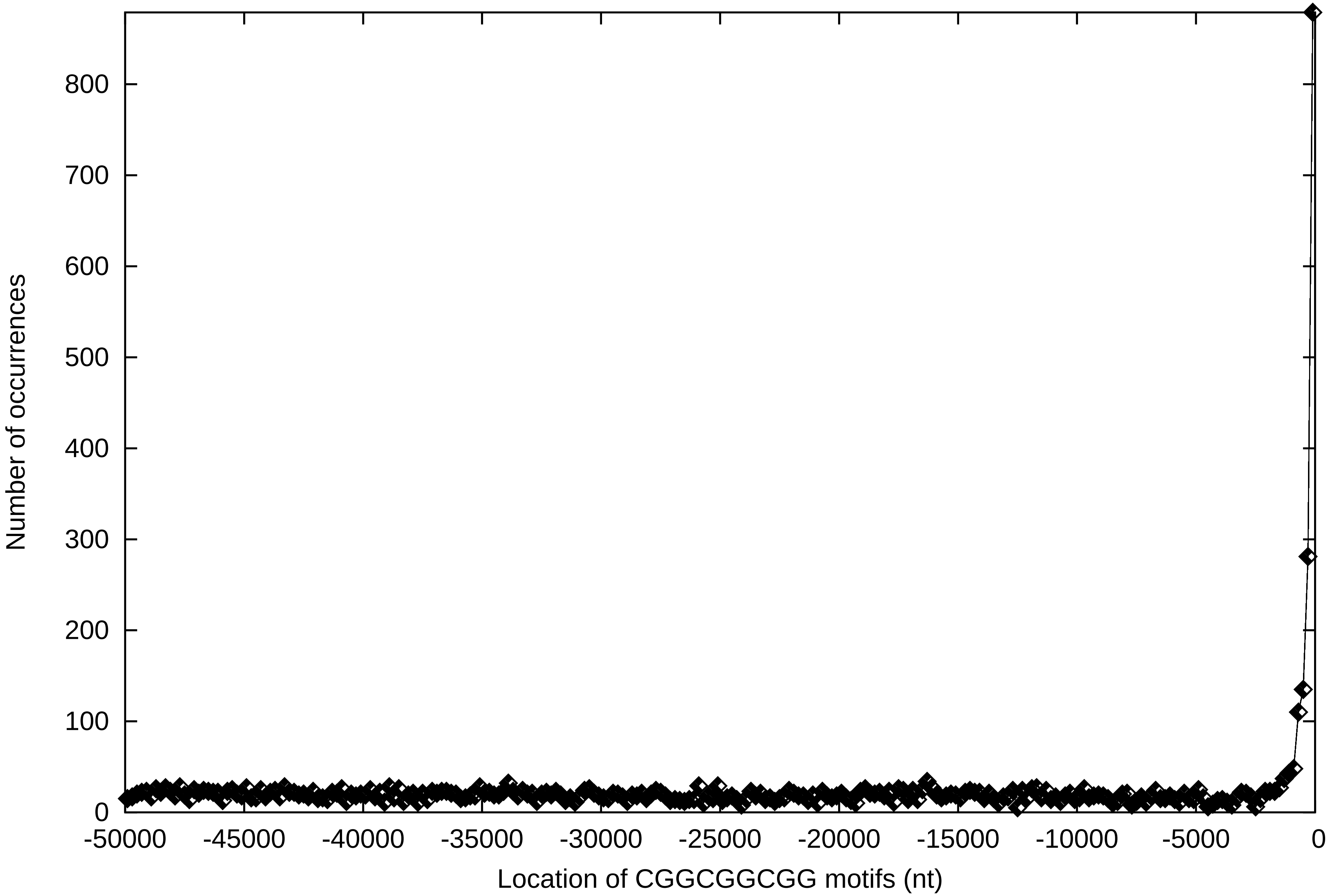

Number of occurrences
0
100
200
300
400
500
600
700
800
-50000
-45000
-40000
-35000
-30000
-25000
-20000
-15000
-10000
-5000
0
Location of CGGCGGCGG motifs (nt)

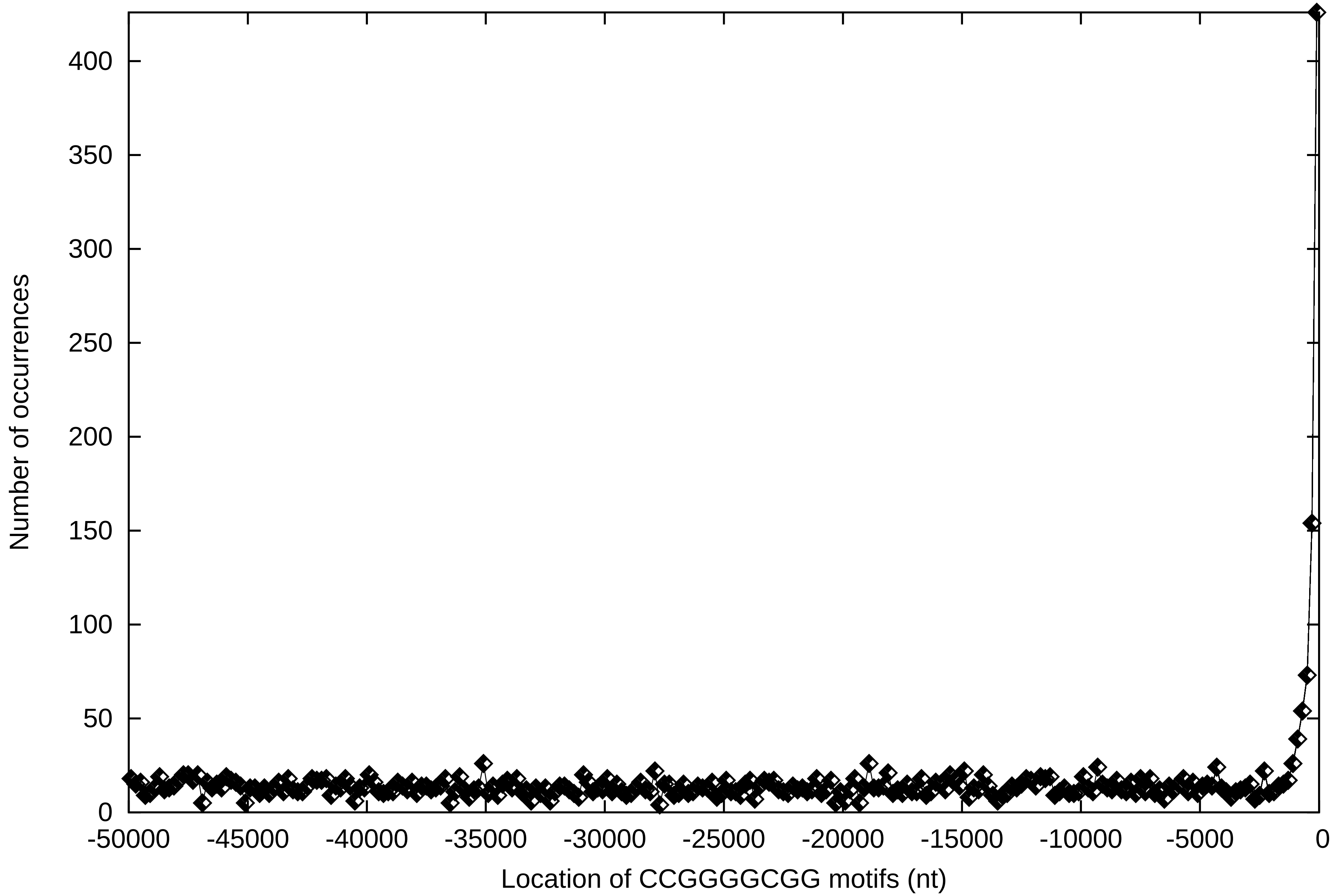
Number of occurrences
0
50
100
150
200
250
300
350
400
-50000
-45000
-40000
-35000
-30000
-25000
-20000
-15000
-10000
-5000
0
Location of CCGGGGCGG motifs (nt)

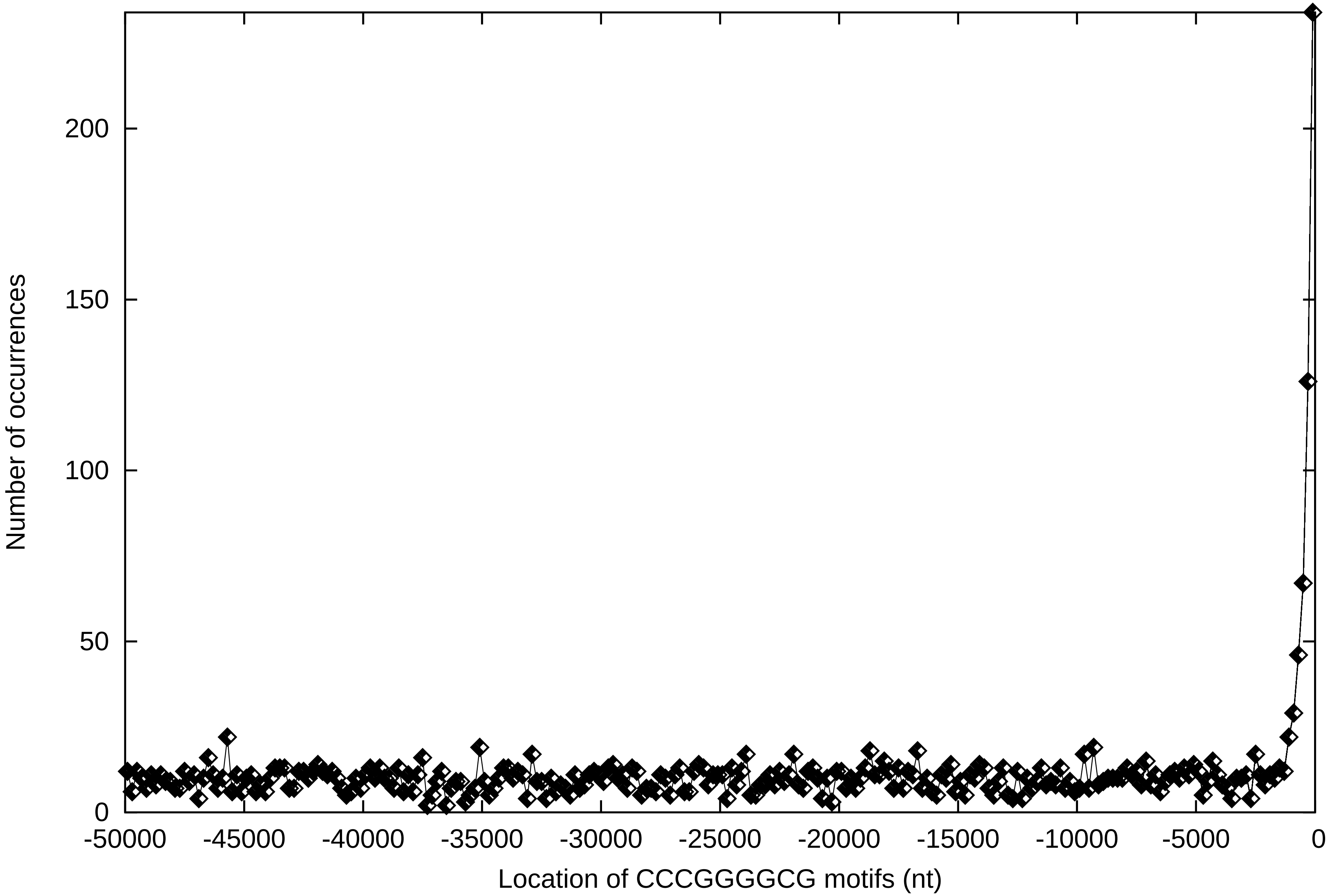
Number of occurrences
200
150
100
50
0
-50000
-45000
-40000
-35000
-30000
-25000
-20000
-15000
-10000
-5000
0
Location of CCCGGGGCG motifs (nt)

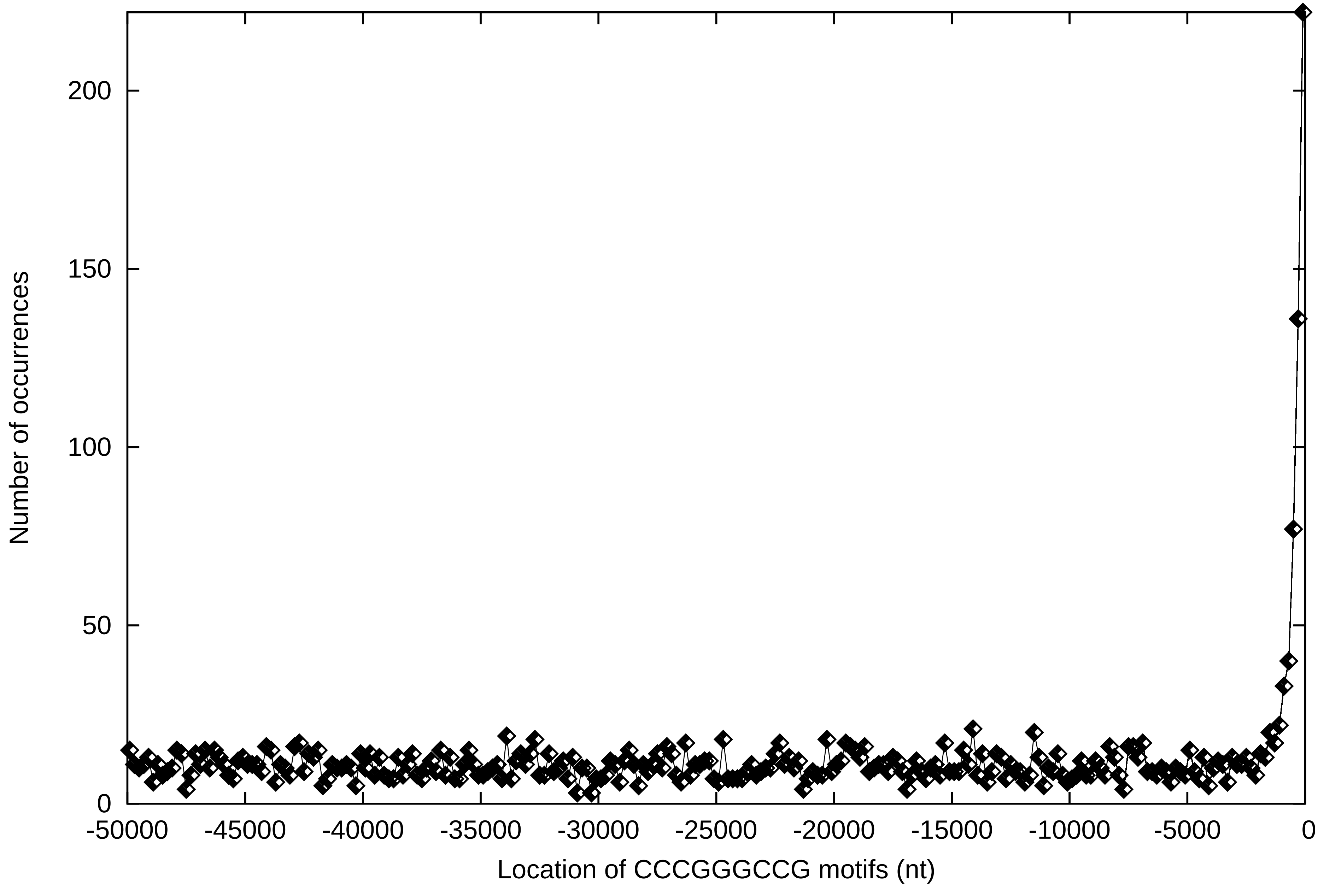

Number of occurrences
200
150
100
50
0
-50000
-45000
-40000
-35000
-30000
-25000
-20000
-15000
-10000
-5000
0
Location of CCCGGGCCG motifs (nt)

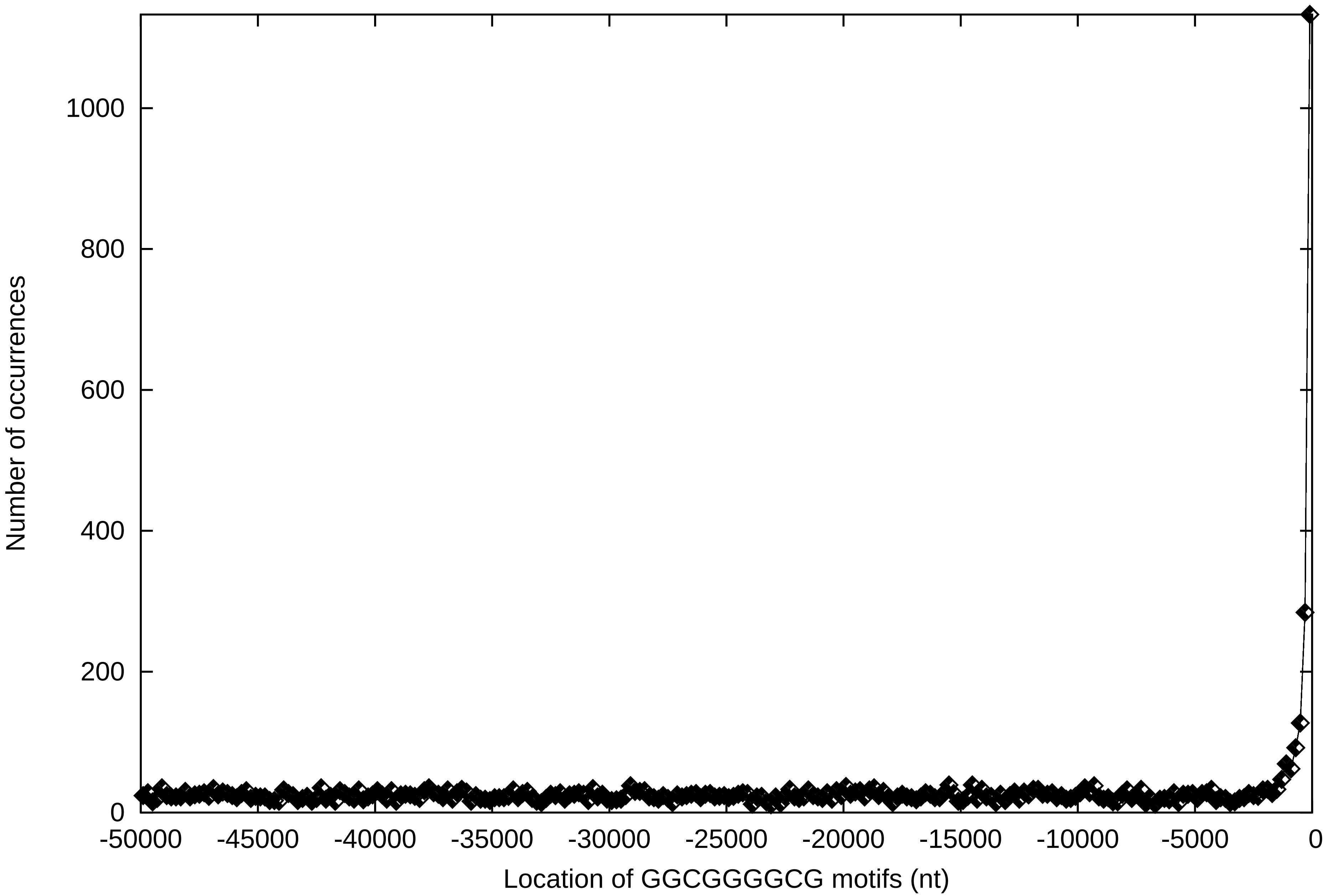

Number of occurrences
1000
800
600
400
200
0
-50000
-45000
-40000
-35000
-30000
-25000
-20000
-15000
-10000
-5000
0
Location of GGCGGGGCG motifs (nt)

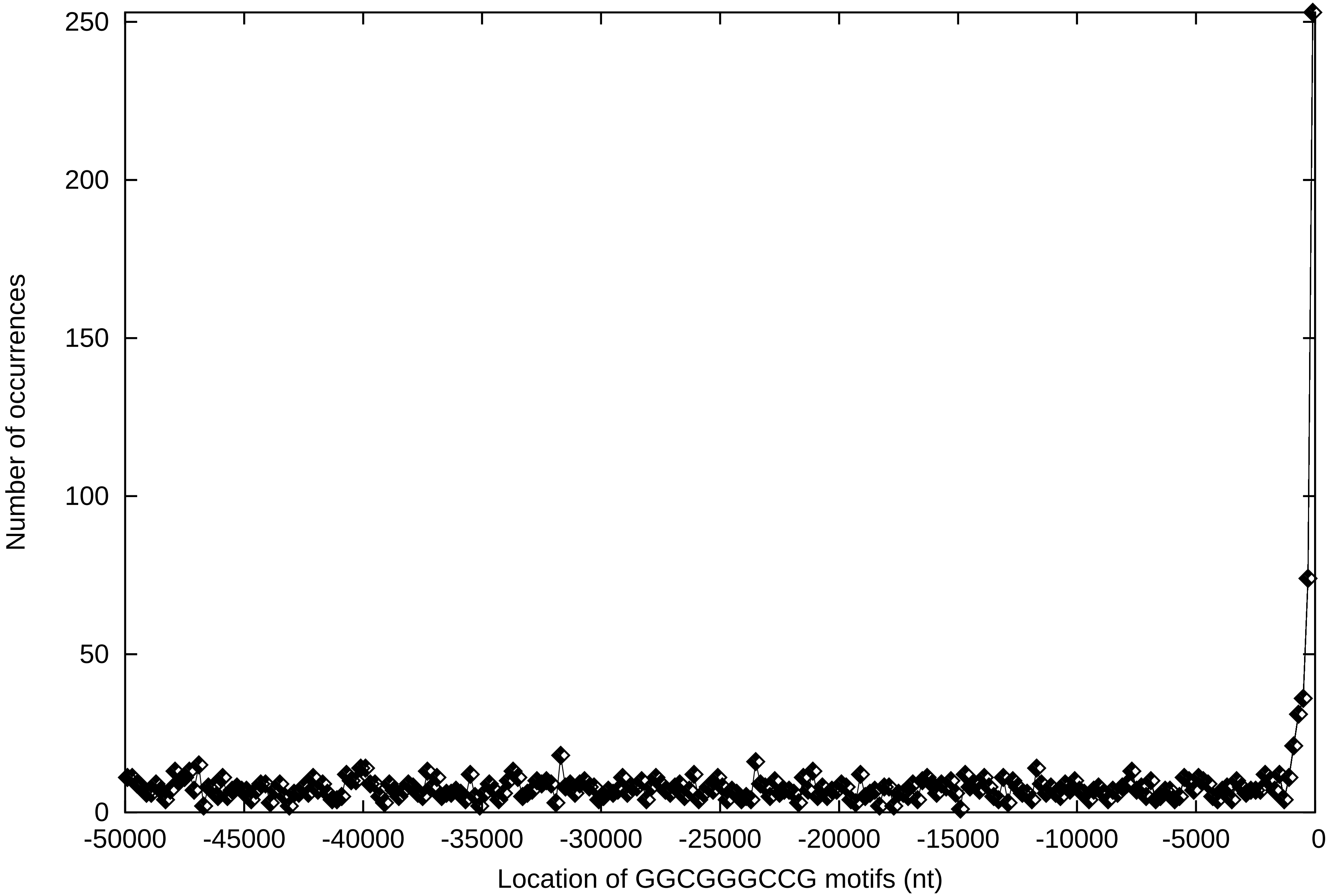

250
200
150
100
50
0
-50000
-45000
-40000
-35000
-30000
-25000
-20000
-15000
-10000
-5000
0
Number of occurrences
Location of GGCGGGCCG motifs (nt)

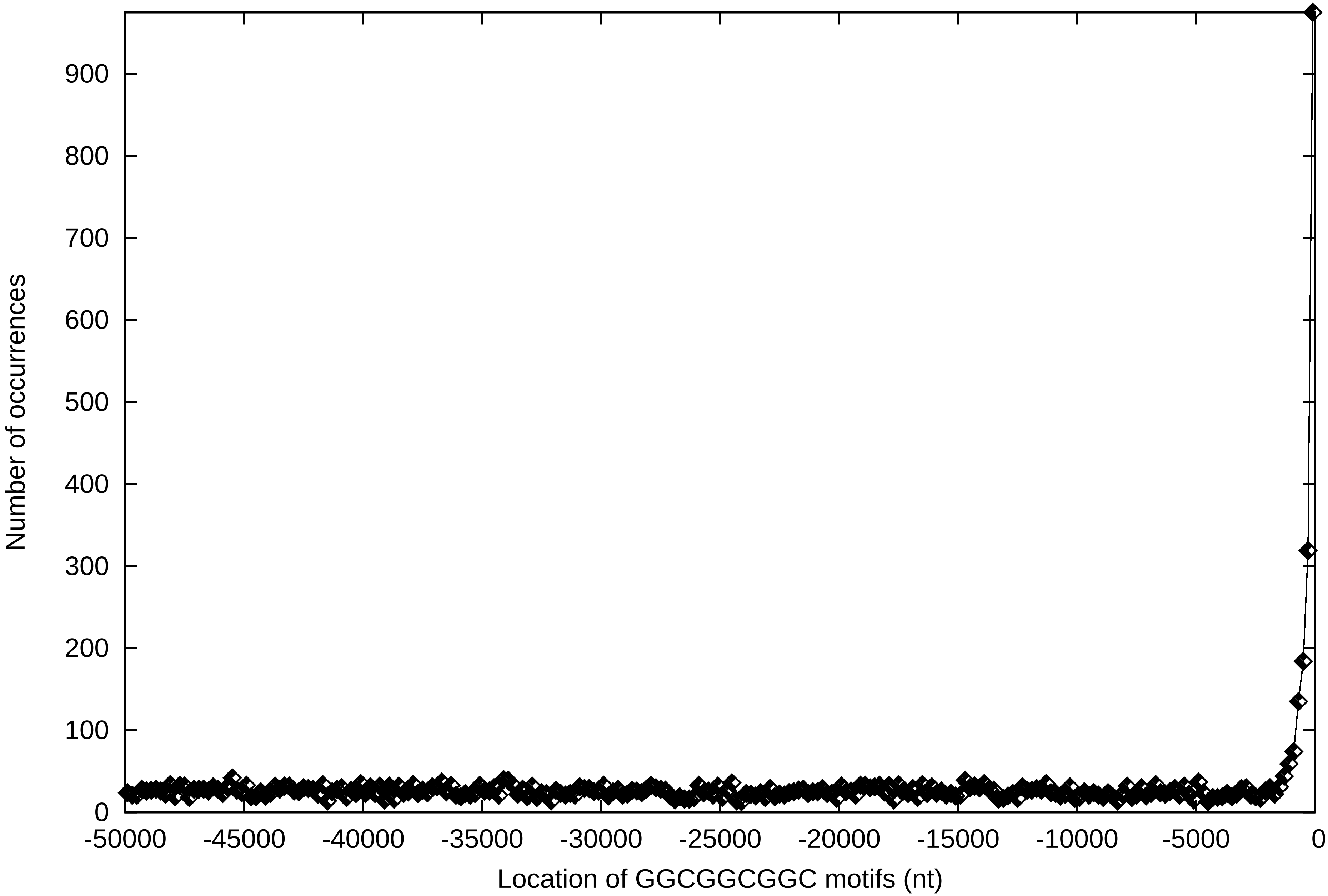

Number of occurrences
900
800
700
600
500
400
300
200
100
0
-50000
-45000
-40000
-35000
-30000
-25000
-20000
-15000
-10000
-5000
0
Location of GGCGGCGGC motifs (nt)

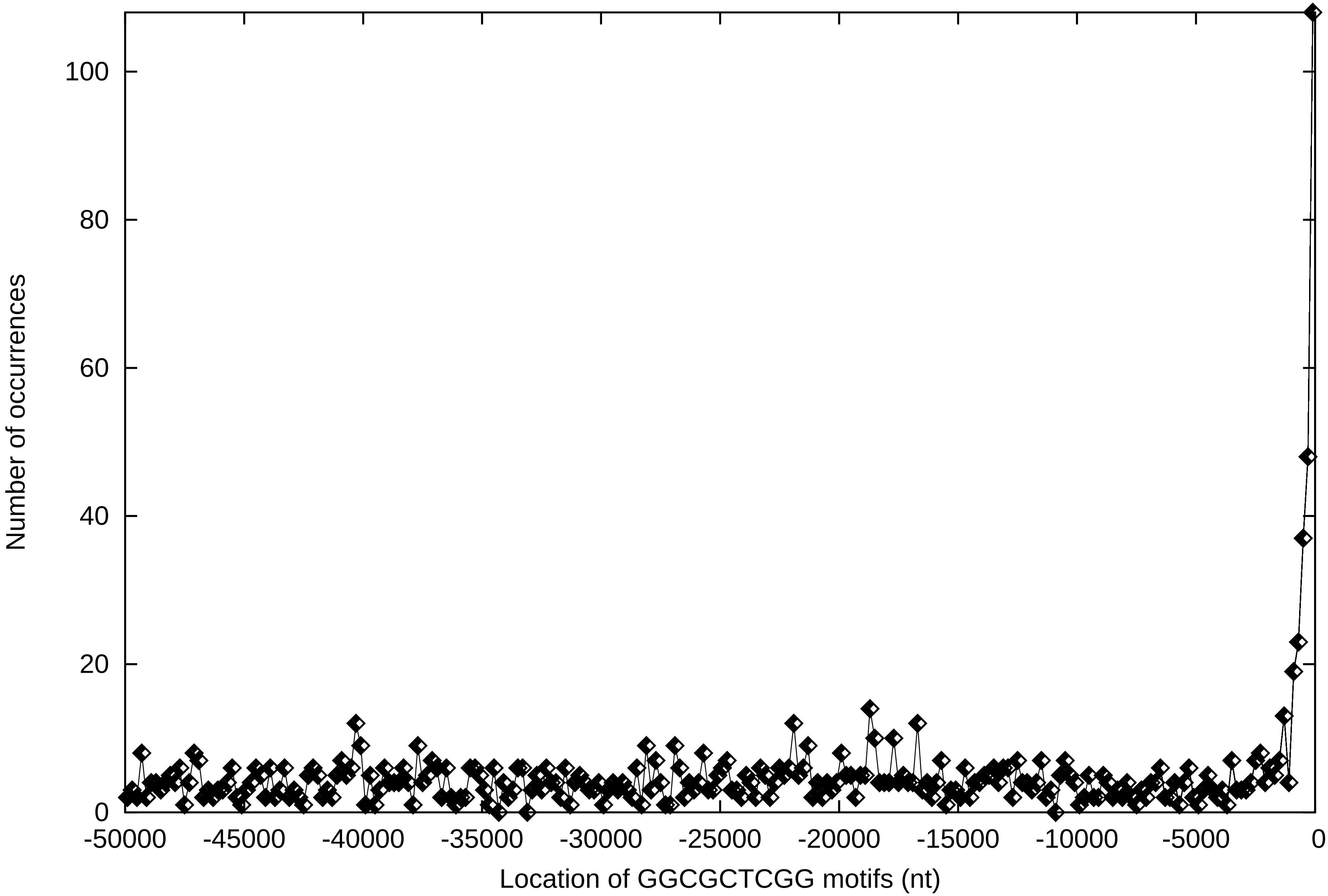

Number of occurrences
0
20
40
60
80
100
-50000
-45000
-40000
-35000
-30000
-25000
-20000
-15000
-10000
-5000
0
Location of GGCGCTCGG motifs (nt)

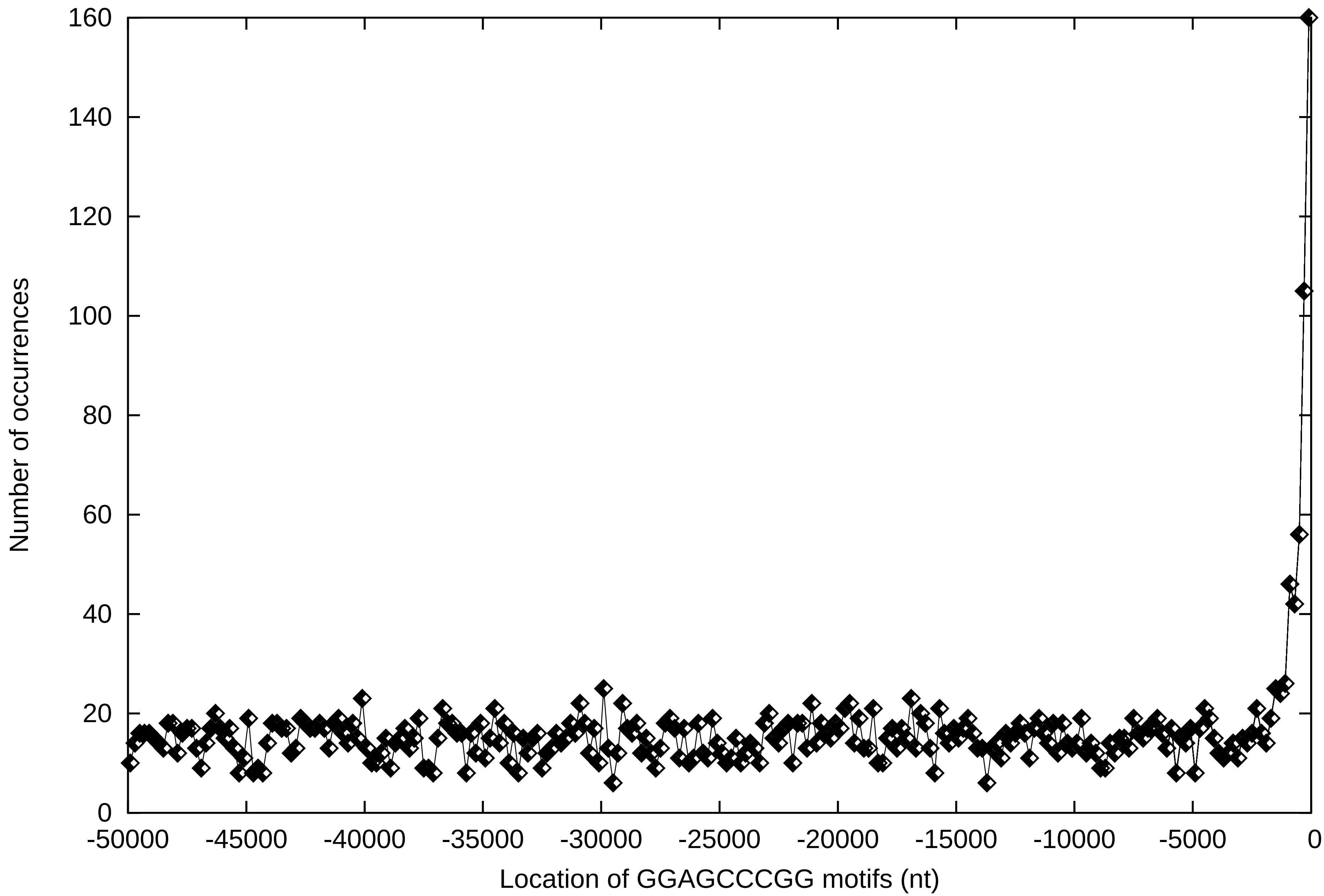

160
140
120
100
80
60
40
20
0
-50000
-45000
-40000
-35000
-30000
-25000
-20000
-15000
-10000
-5000
0
Number of occurrences
Location of GGAGCCCGG motifs (nt)

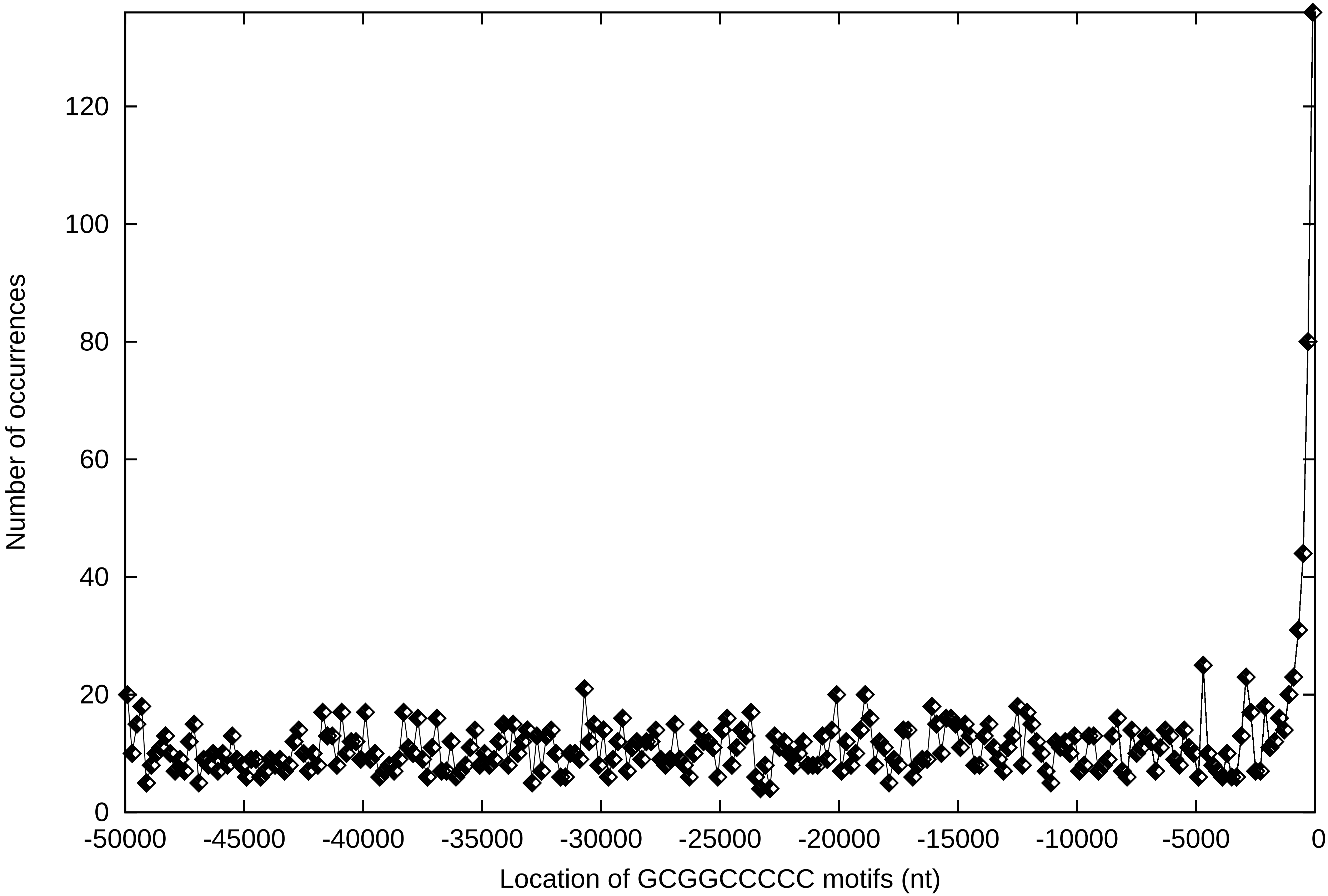

Number of occurrences
0
20
40
60
80
100
120
-50000
-45000
-40000
-35000
-30000
-25000
-20000
-15000
-10000
-5000
0
Location of GCGGCCCCC motifs (nt)

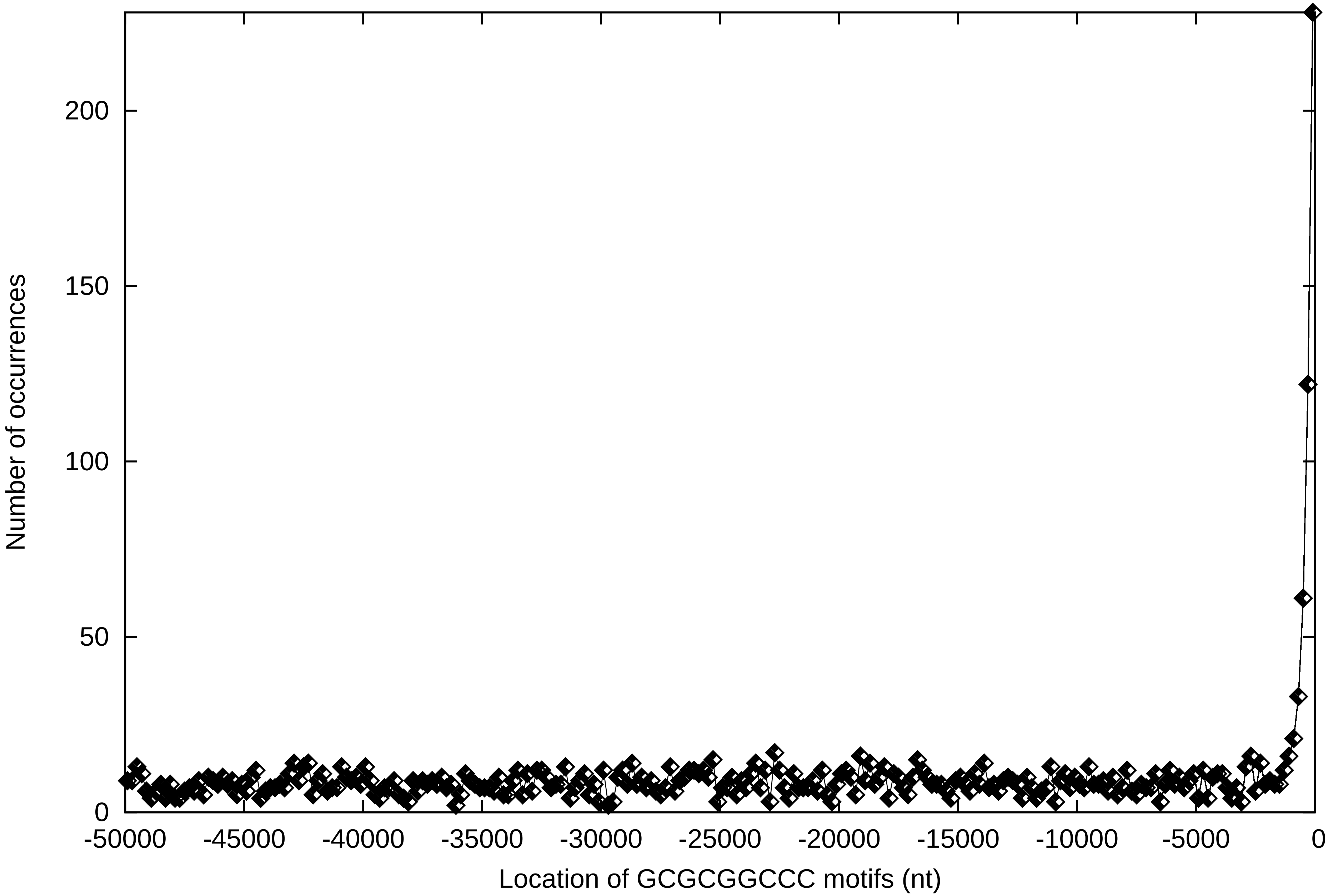

Number of occurrences
200
150
100
50
0
-50000
-45000
-40000
-35000
-30000
-25000
-20000
-15000
-10000
-5000
0
Location of GCGCGGCCC motifs (nt)

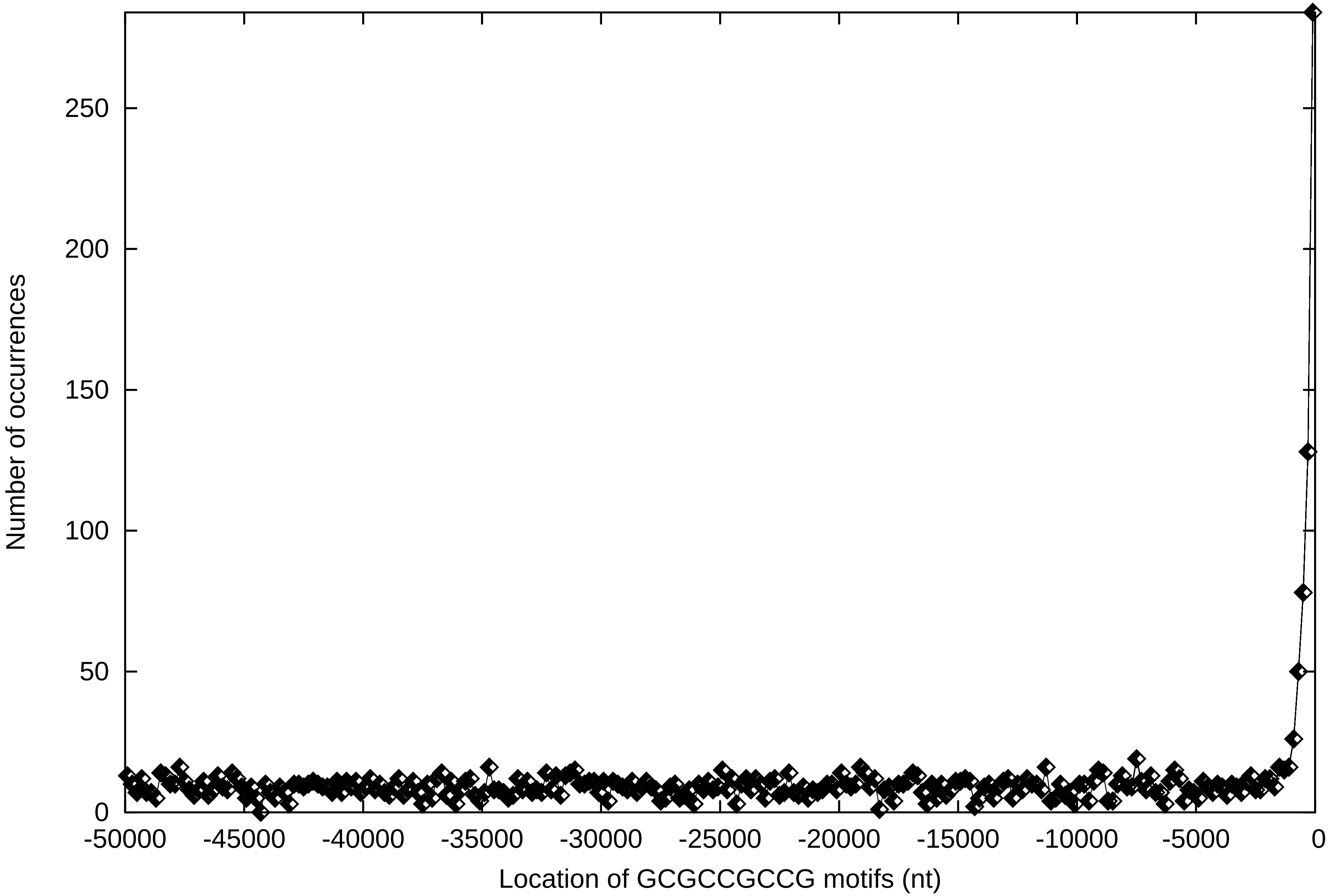

Number of occurrences
250
200
150
100
50
0
-50000
-45000
-40000
-35000
-30000
-25000
-20000
-15000
-10000
-5000
0
Location of GCGCCGCCG motifs (nt)

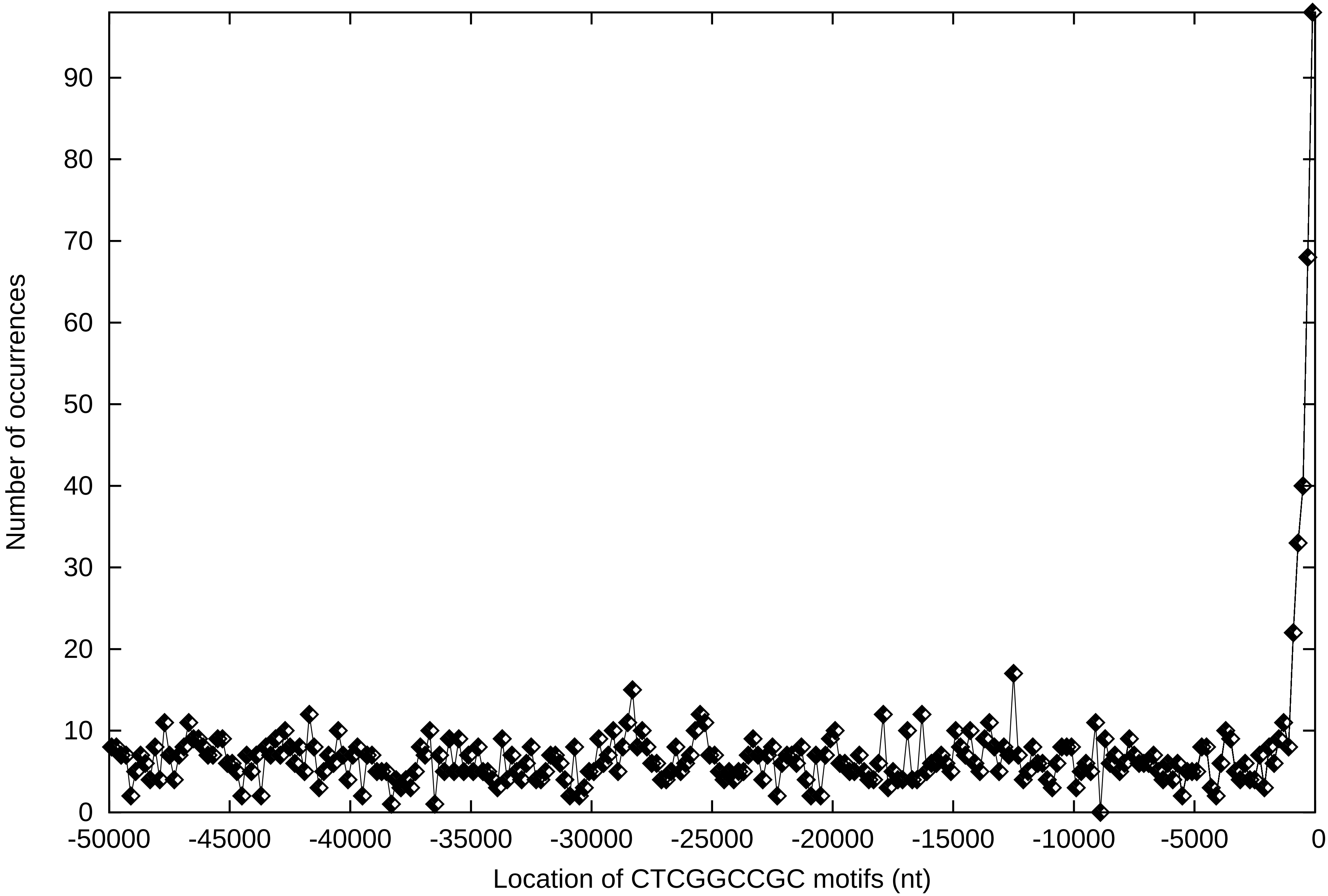

Number of occurrences
0
10
20
30
40
50
60
70
80
90
-50000
-45000
-40000
-35000
-30000
-25000
-20000
-15000
-10000
-5000
0
Location of CTCGGCCGC motifs (nt)

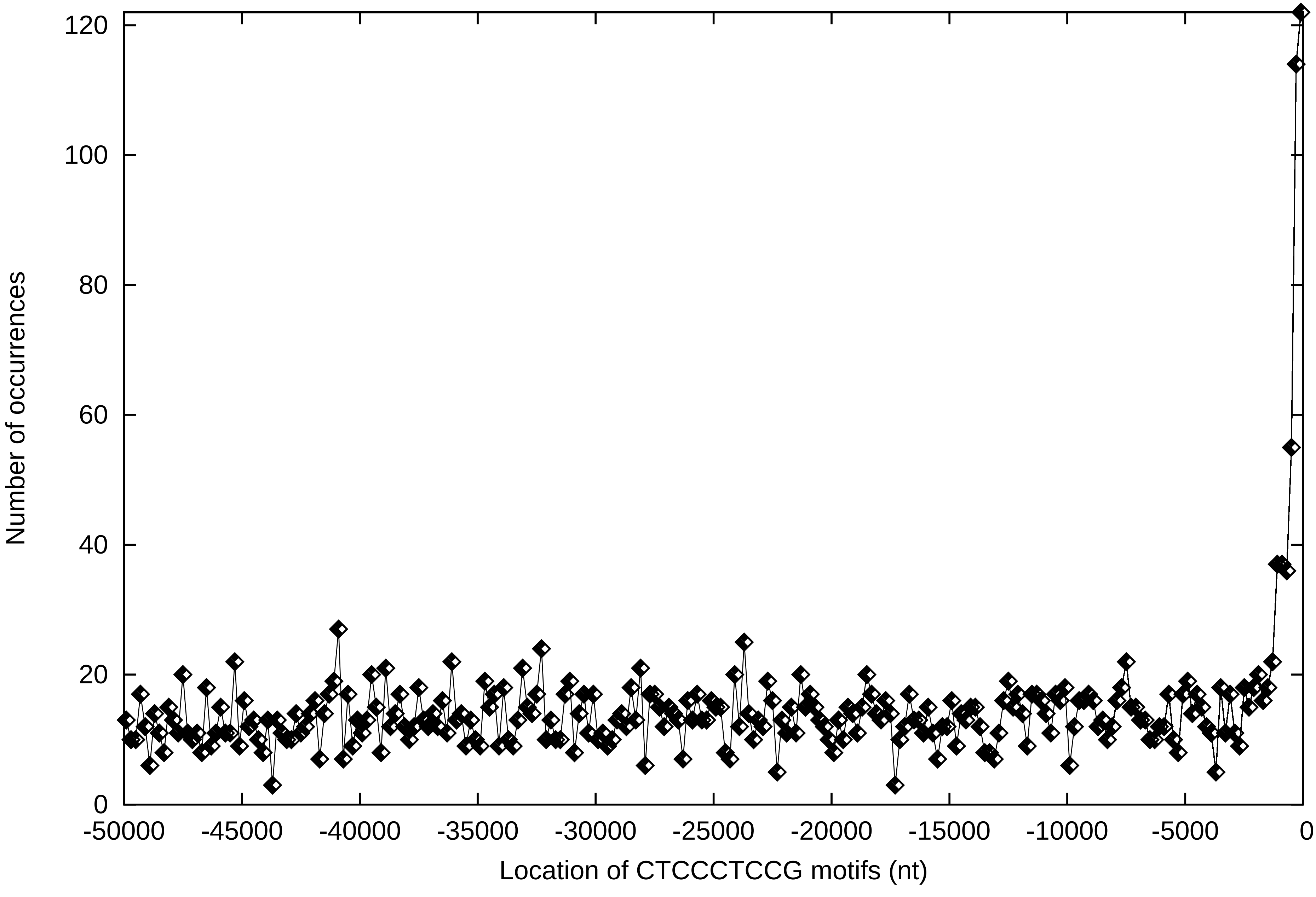

120
100
80
60
40
20
0
-50000
-45000
-40000
-35000
-30000
-25000
-20000
-15000
-10000
-5000
0
Number of occurrences
Location of CTCCCTCCG motifs (nt)

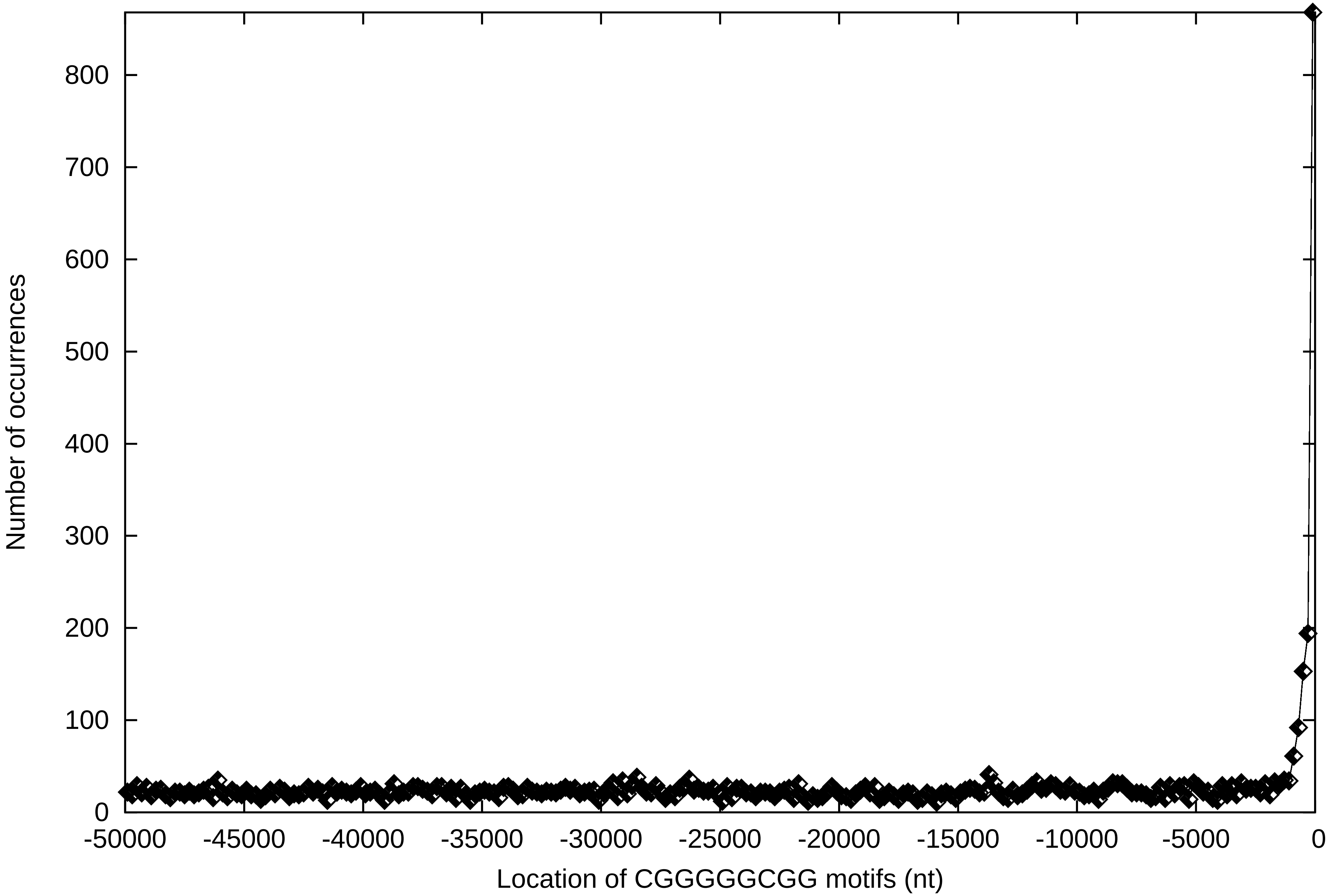

Number of occurrences
800
700
600
500
400
300
200
100
0
-50000
-45000
-40000
-35000
-30000
-25000
-20000
-15000
-10000
-5000
0
Location of CGGGGGCGG motifs (nt)

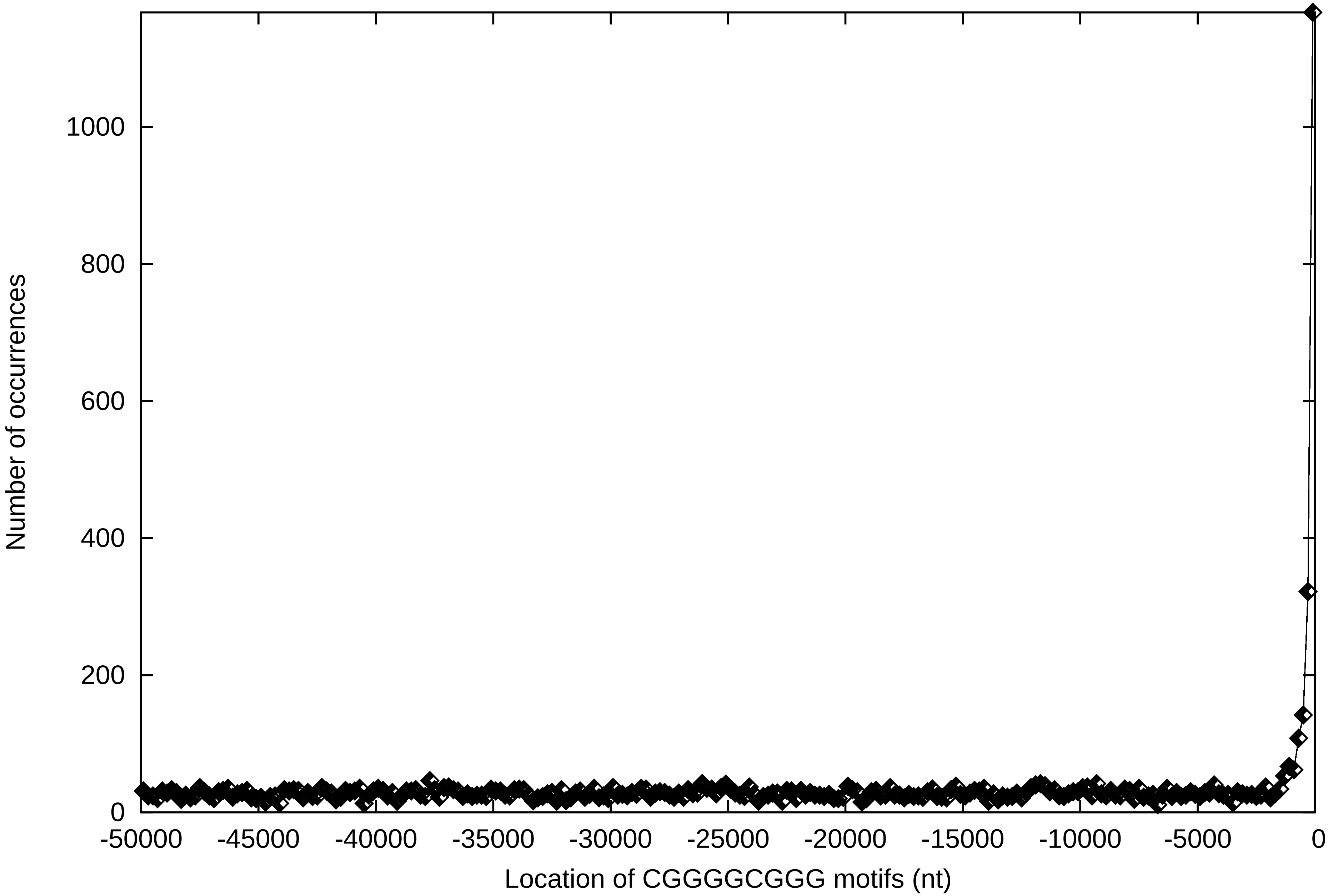

1000
800
600
400
200
0
Number of occurrences
-50000
-45000
-40000
-35000
-30000
-25000
-20000
-15000
-10000
-5000
0
Location of CGGGGCGGG motifs (nt)

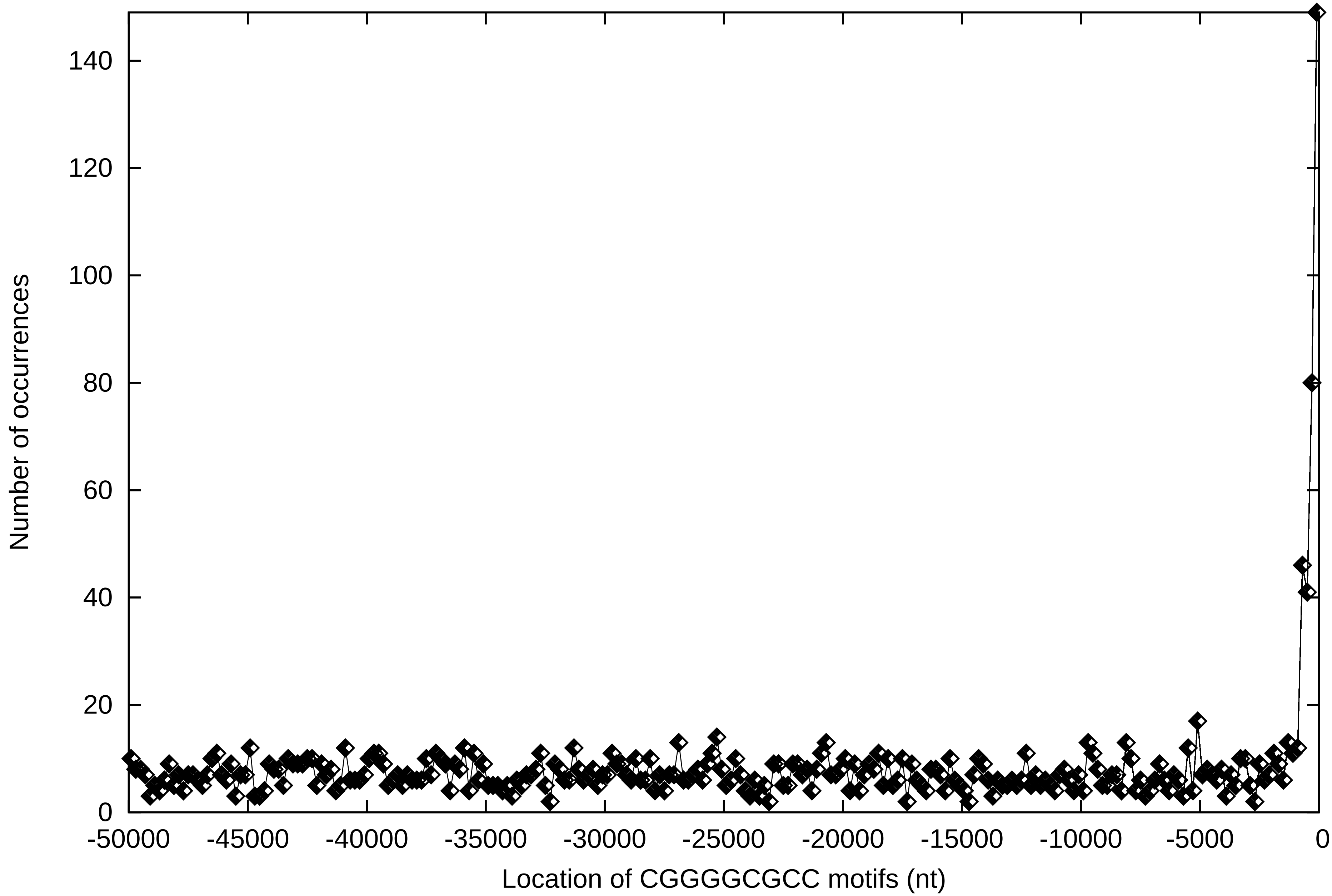

Number of occurrences
0
20
40
60
80
100
120
140
-50000
-45000
-40000
-35000
-30000
-25000
-20000
-15000
-10000
-5000
0
Location of CGGGGCGCC motifs (nt)

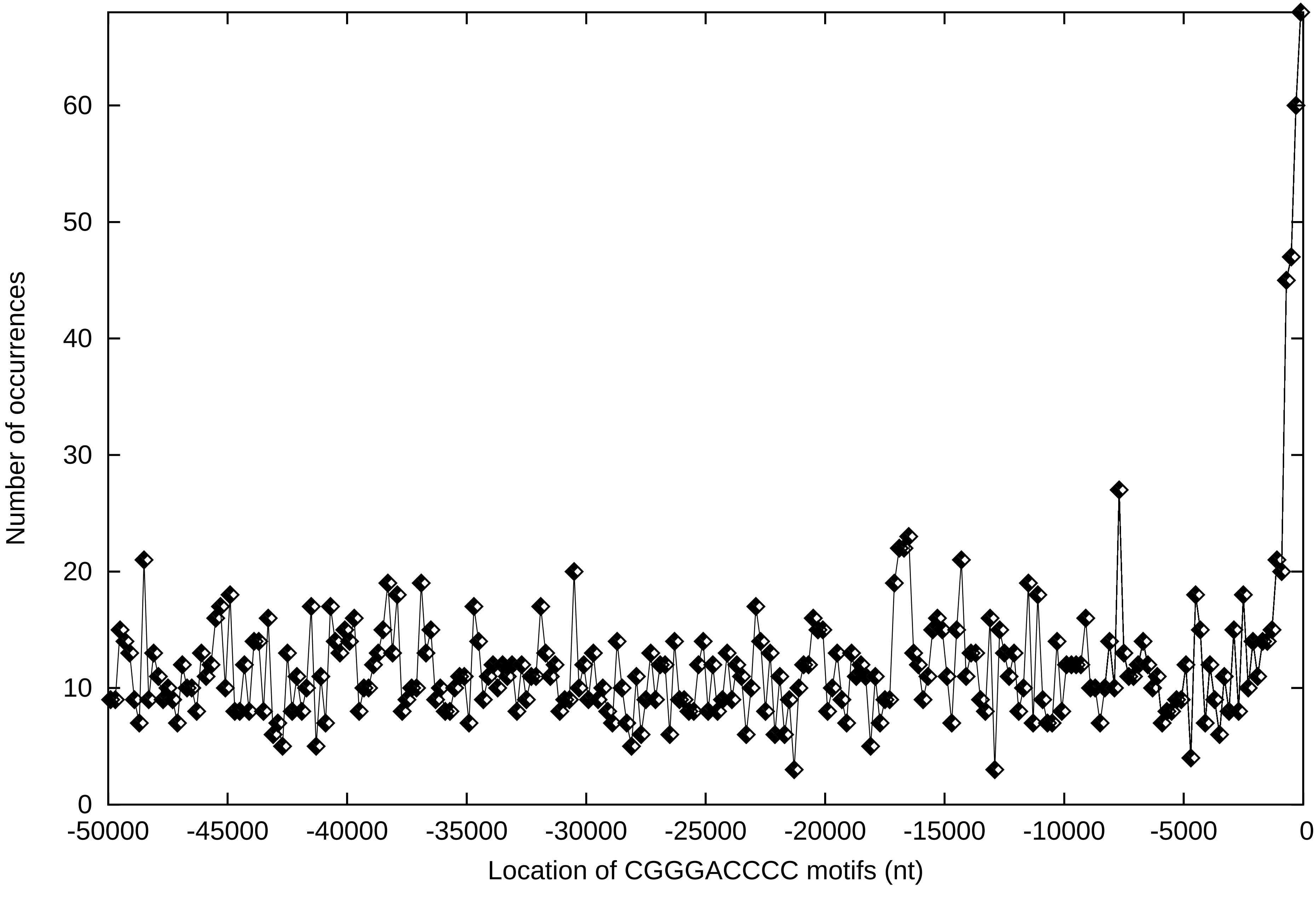
Number of occurrences
Location of CGGGACCCC motifs (nt)
0
10
20
30
40
50
60
-50000
-45000
-40000
-35000
-30000
-25000
-20000
-15000
-10000
-5000
0

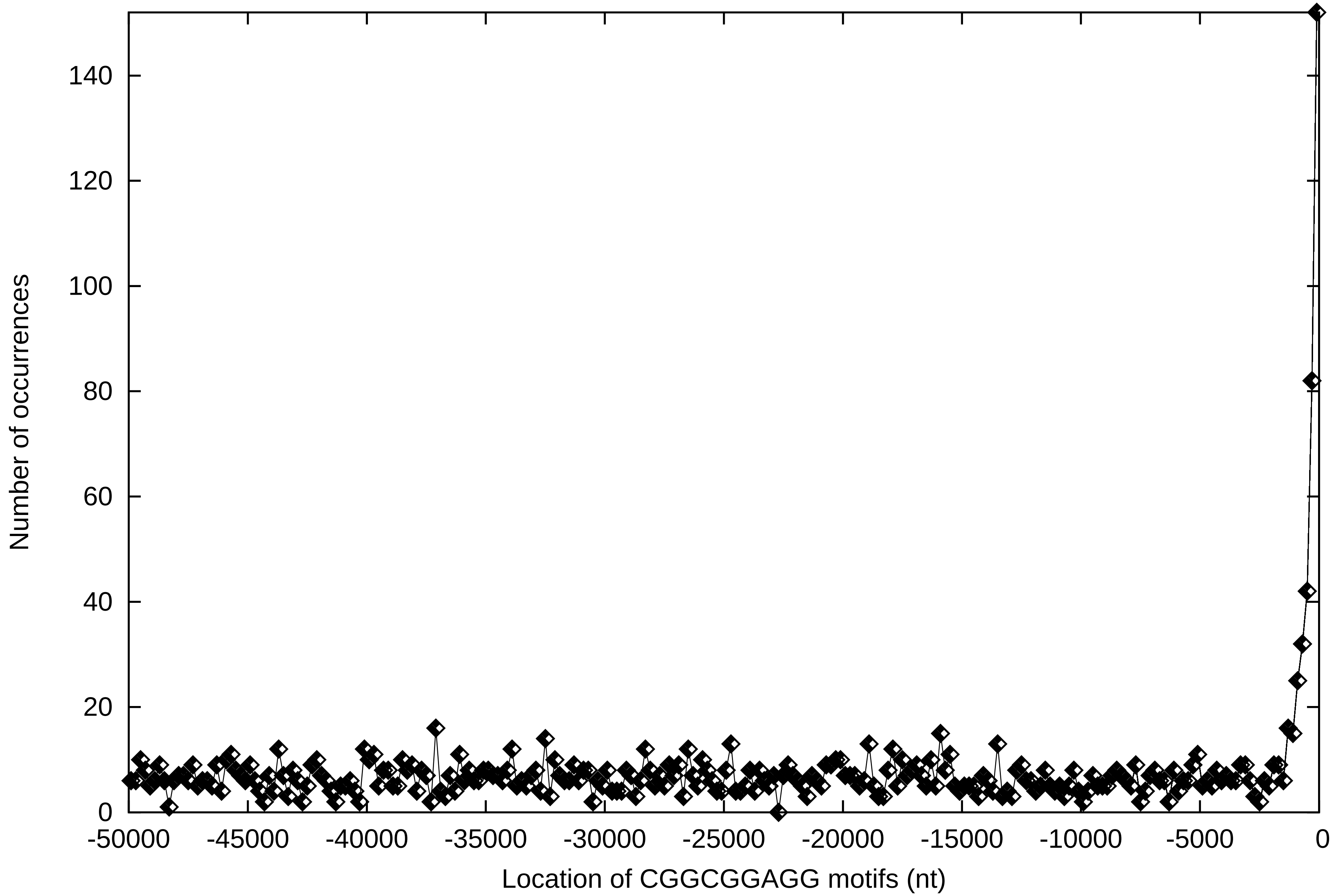
Number of occurrences
0
20
40
60
80
100
120
140
-50000
-45000
-40000
-35000
-30000
-25000
-20000
-15000
-10000
-5000
0
Location of CGGCGGAGG motifs (nt)

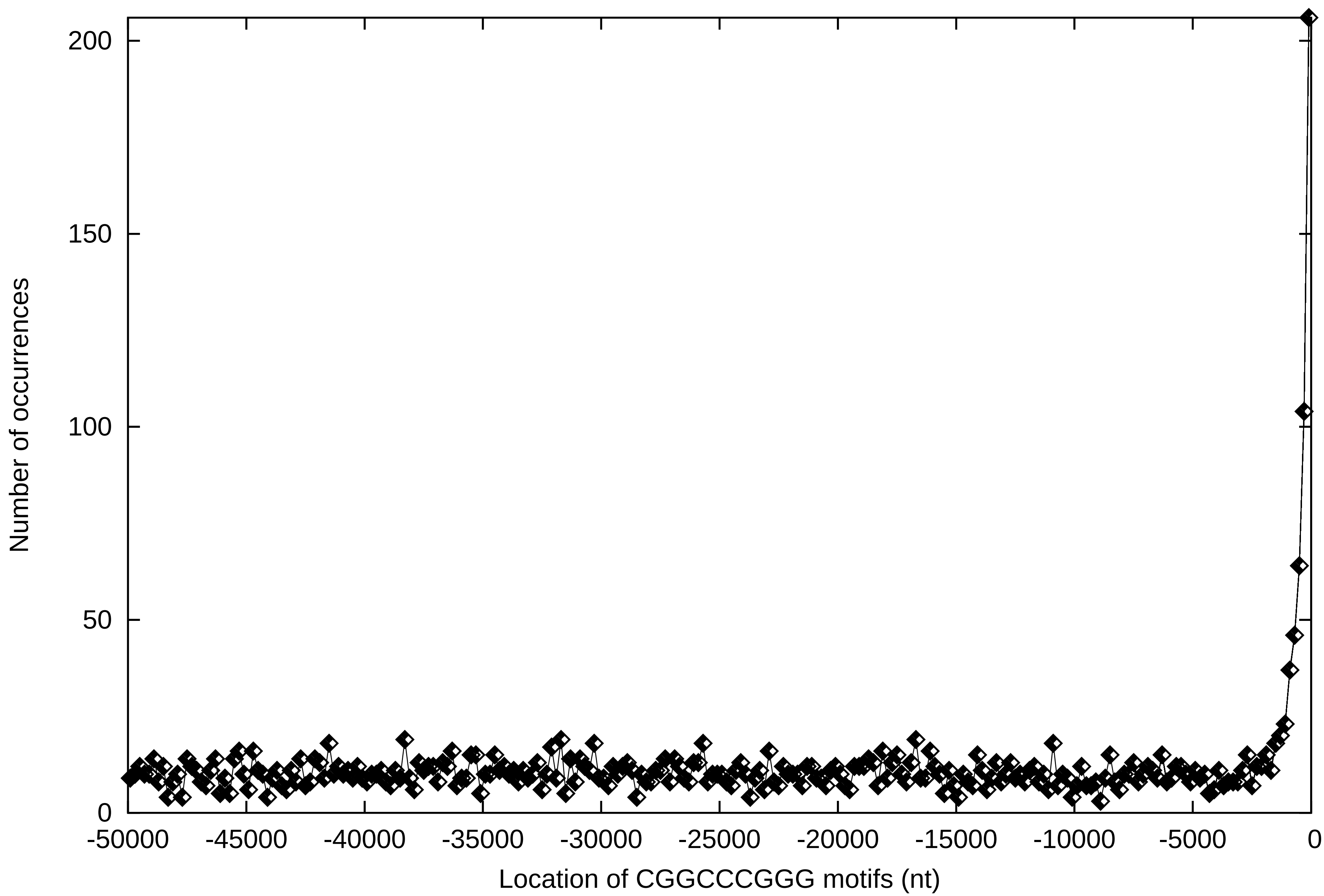
Number of occurrences
Location of CGGCCCGGG motifs (nt)
200
150
100
50
0
-50000
-45000
-40000
-35000
-30000
-25000
-20000
-15000
-10000
-5000
0

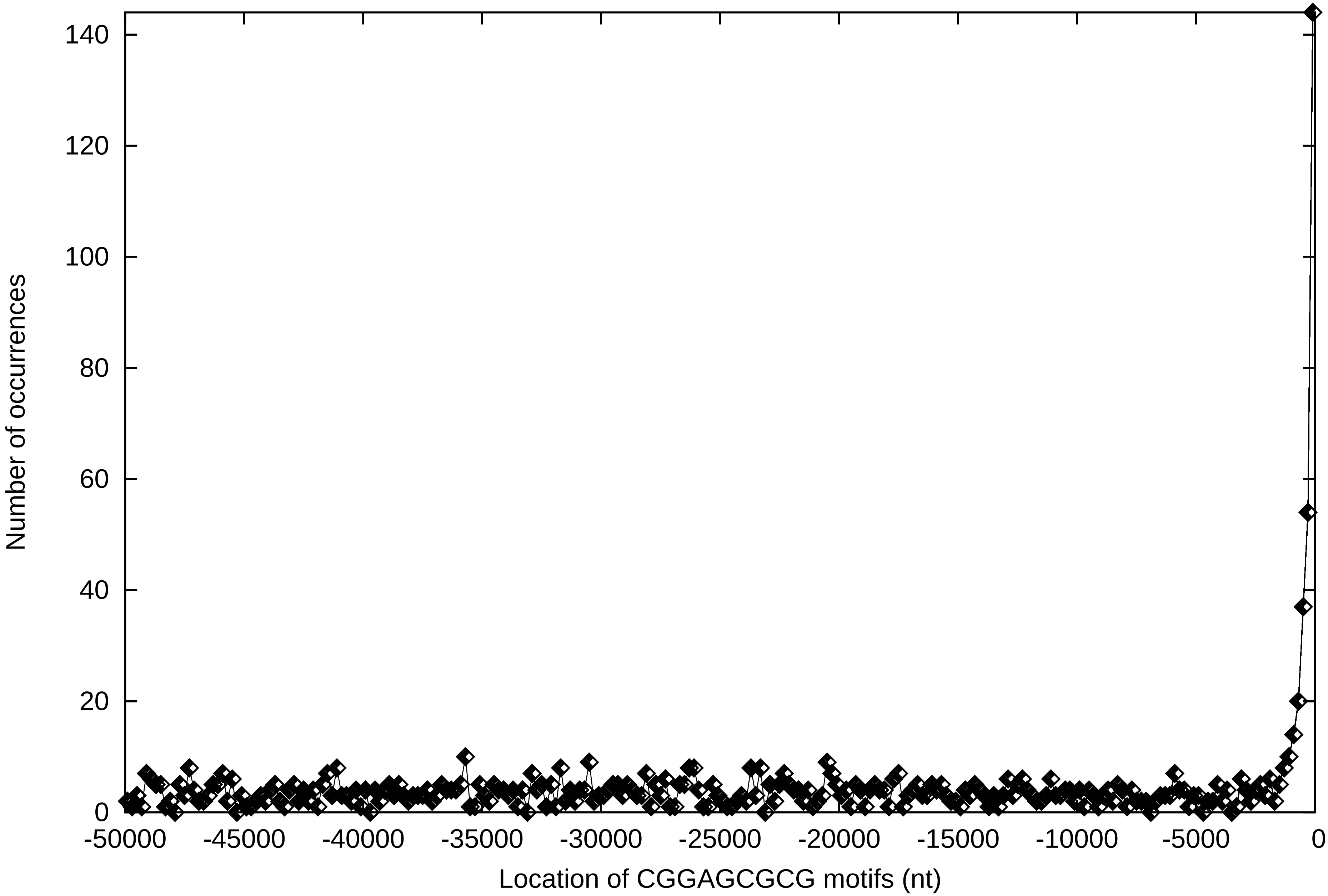

Number of occurrences
0
20
40
60
80
100
120
140
-50000
-45000
-40000
-35000
-30000
-25000
-20000
-15000
-10000
-5000
0
Location of CGGAGCGCG motifs (nt)

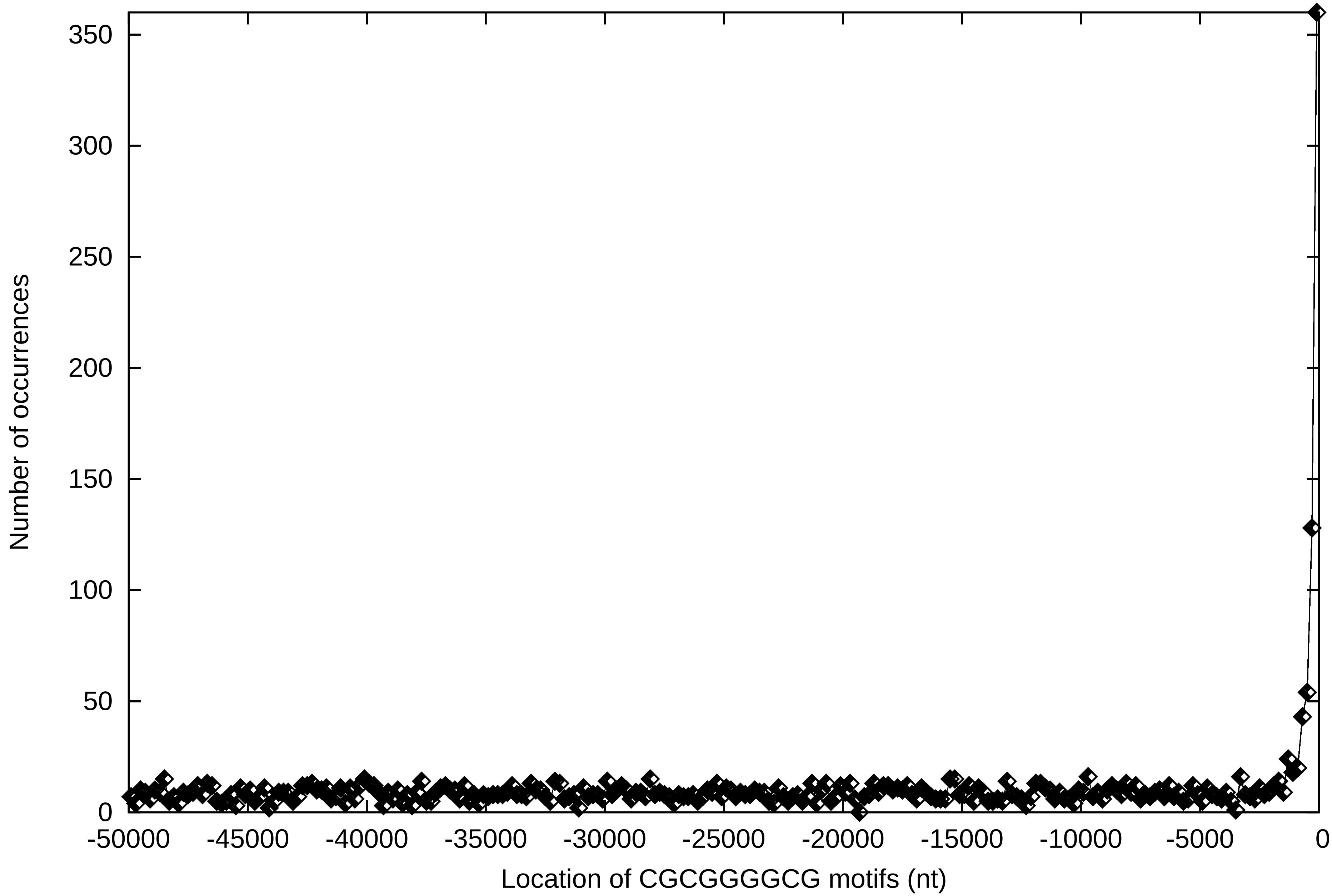
Number of occurrences
0
50
100
150
200
250
300
350
-50000
-45000
-40000
-35000
-30000
-25000
-20000
-15000
-10000
-5000
0
Location of CGCGGGGCG motifs (nt)

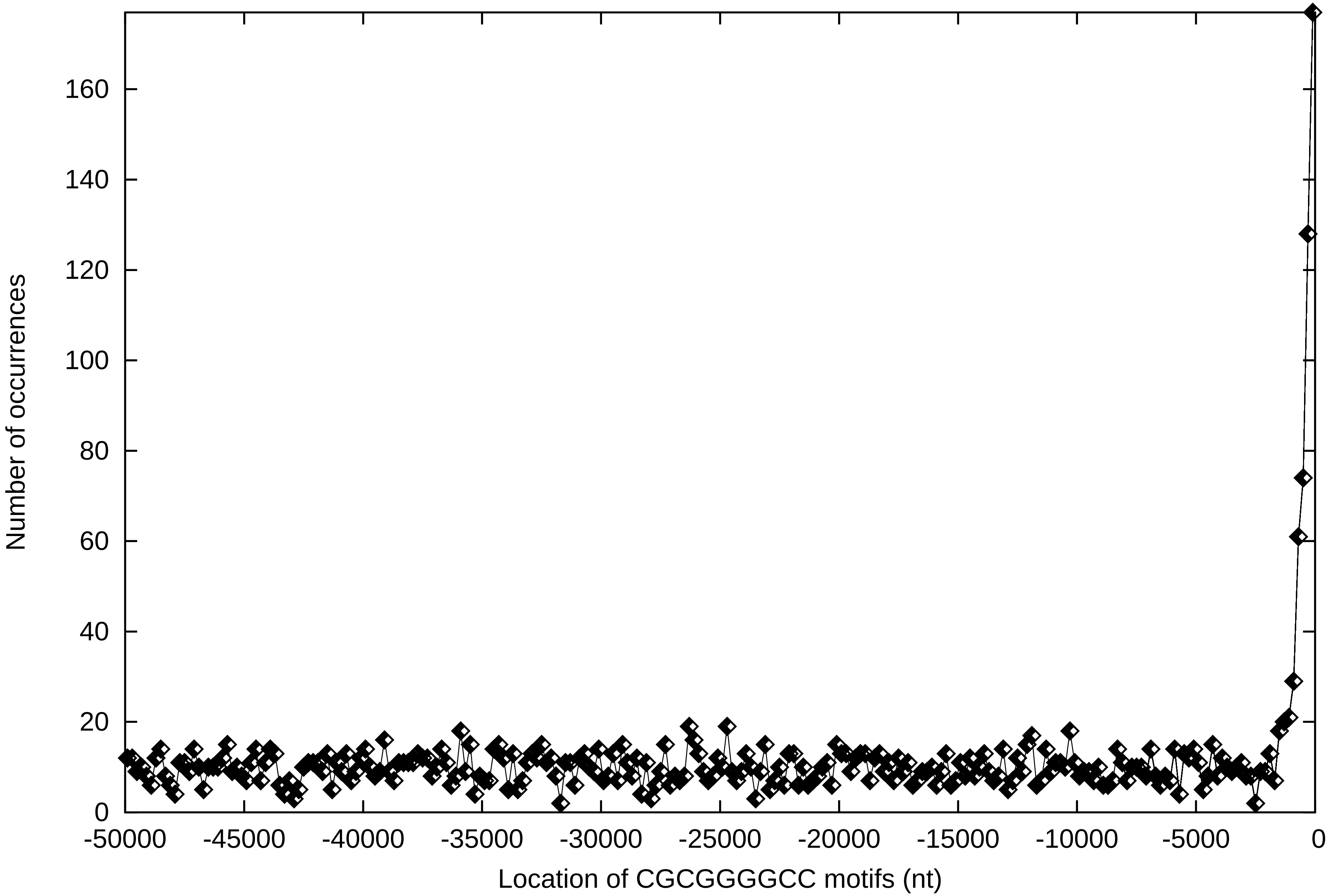

Number of occurrences
0
20
40
60
80
100
120
140
160
-50000
-45000
-40000
-35000
-30000
-25000
-20000
-15000
-10000
-5000
0
Location of CGCGGGGCC motifs (nt)

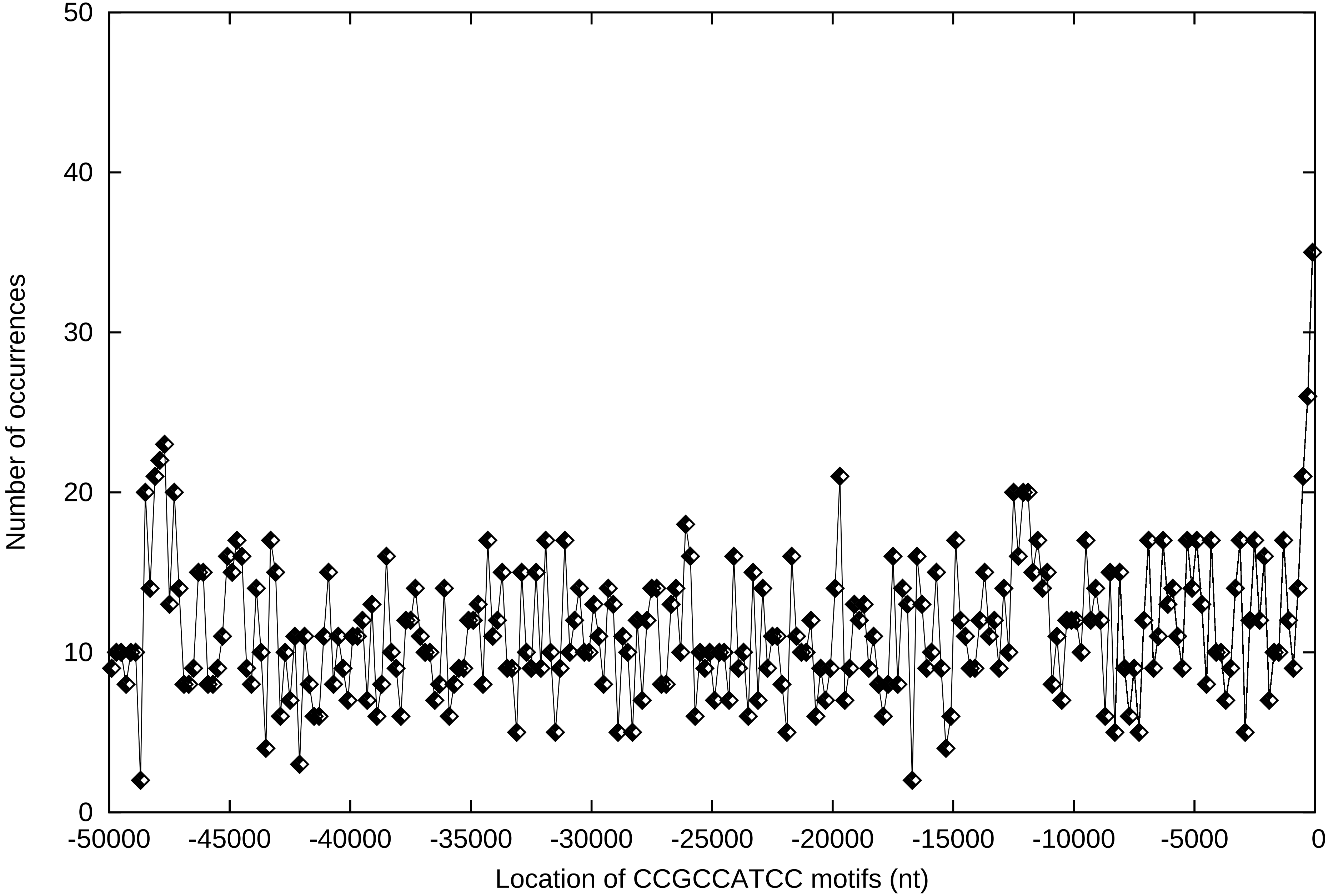

Number of occurrences
Location of CCGCCATCC motifs (nt)
50
40
30
20
10
0
-50000
-45000
-40000
-35000
-30000
-25000
-20000
-15000
-10000
-5000
0

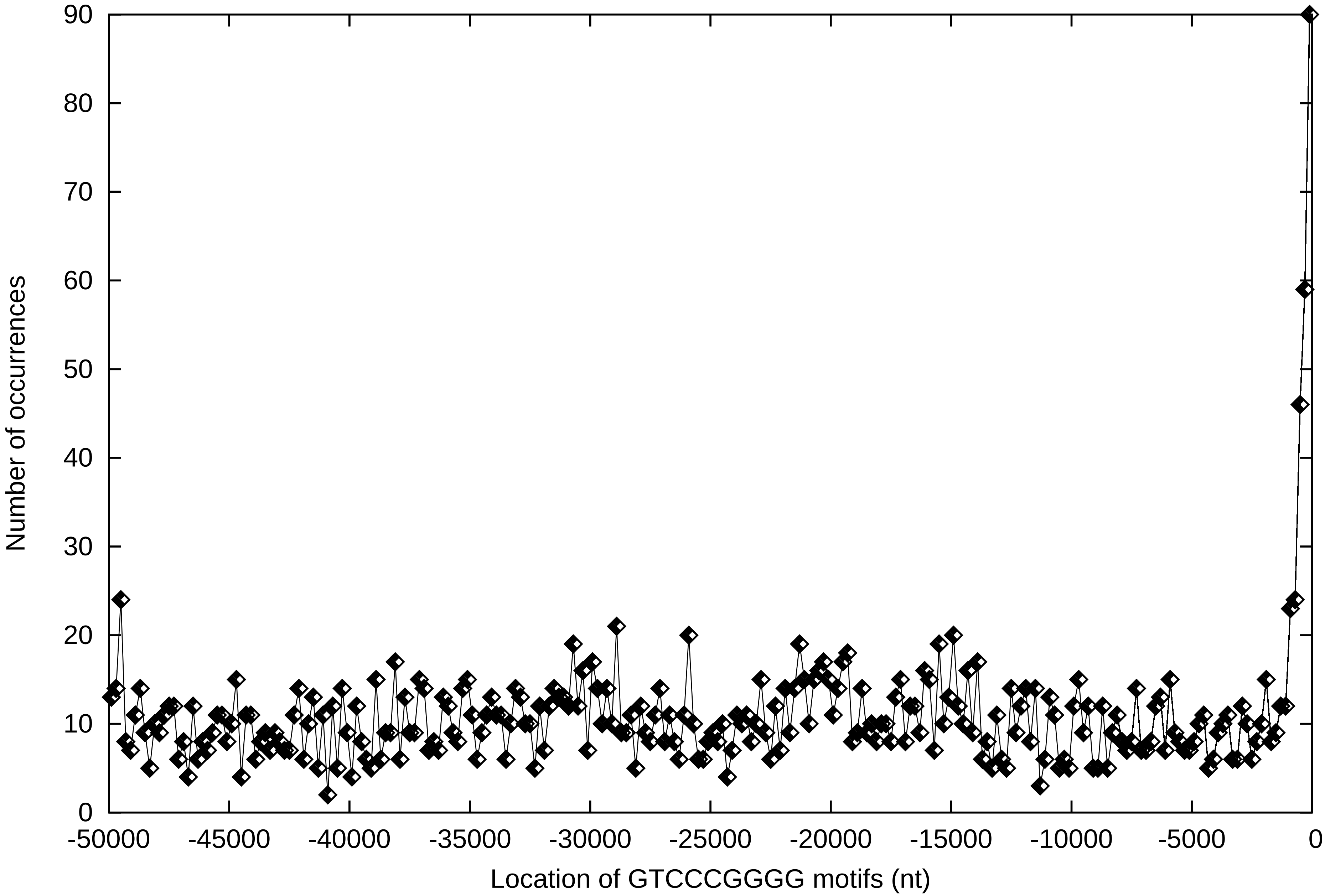

Number of occurrences
90
80
70
60
50
40
30
20
10
0
-50000
-45000
-40000
-35000
-30000
-25000
-20000
-15000
-10000
-5000
0
Location of GTCCCGGGG motifs (nt)

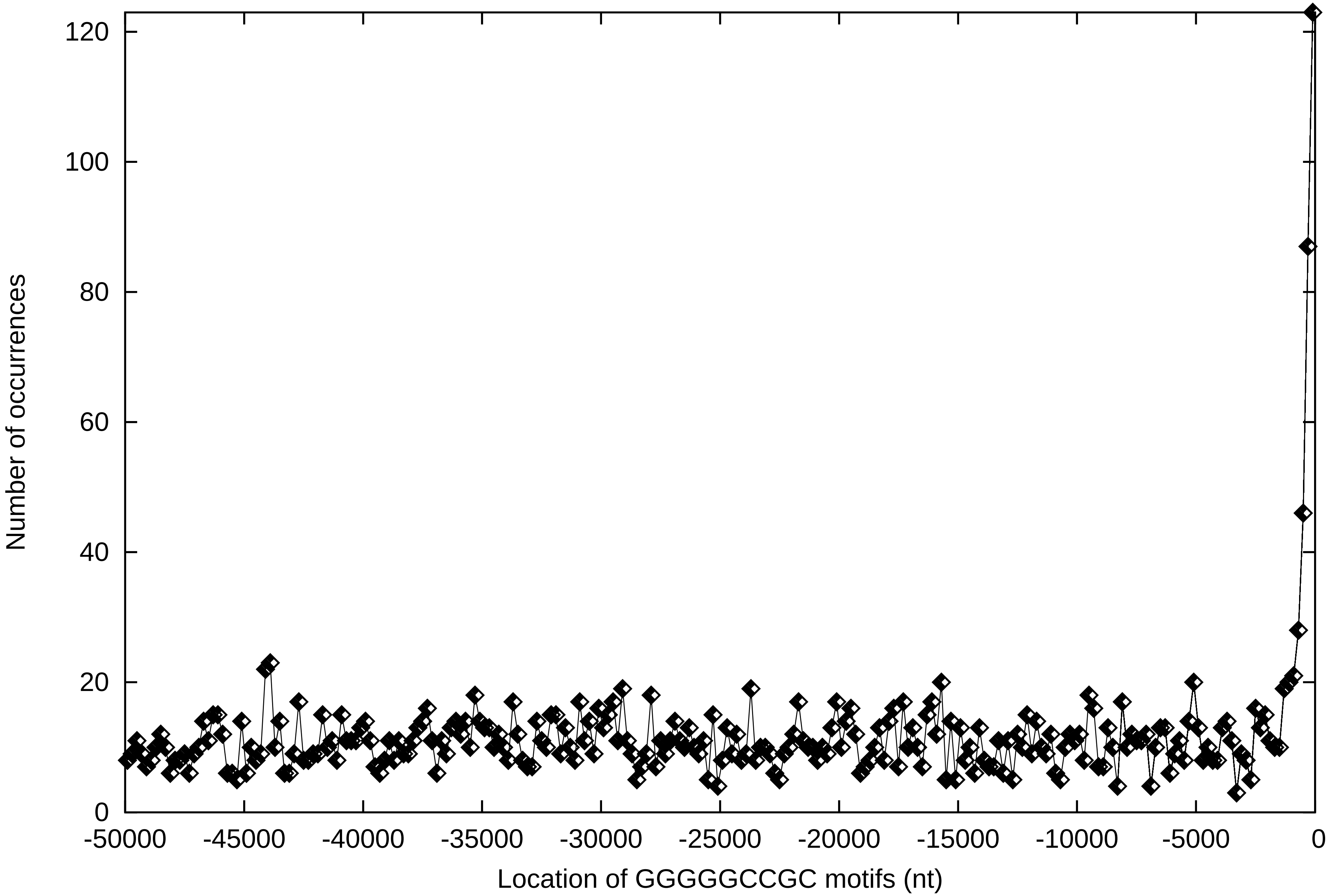
Number of occurrences
Location of GGGGGCCGC motifs (nt)
0
20
40
60
80
100
120
-50000
-45000
-40000
-35000
-30000
-25000
-20000
-15000
-10000
-5000
0

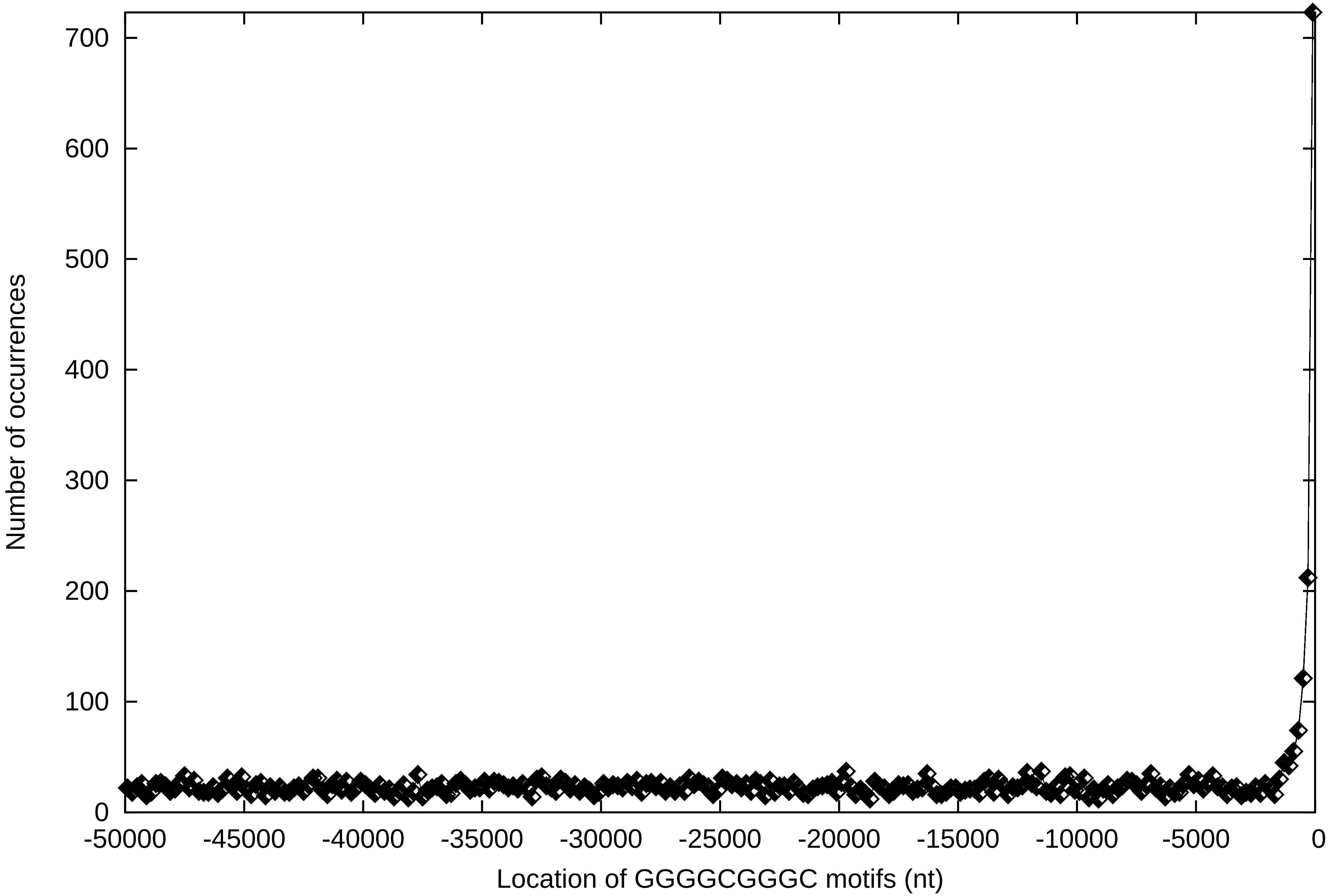
Number of occurrences
0
100
200
300
400
500
600
700
-50000
-45000
-40000
-35000
-30000
-25000
-20000
-15000
-10000
-5000
0
Location of GGGGCGGGC motifs (nt)

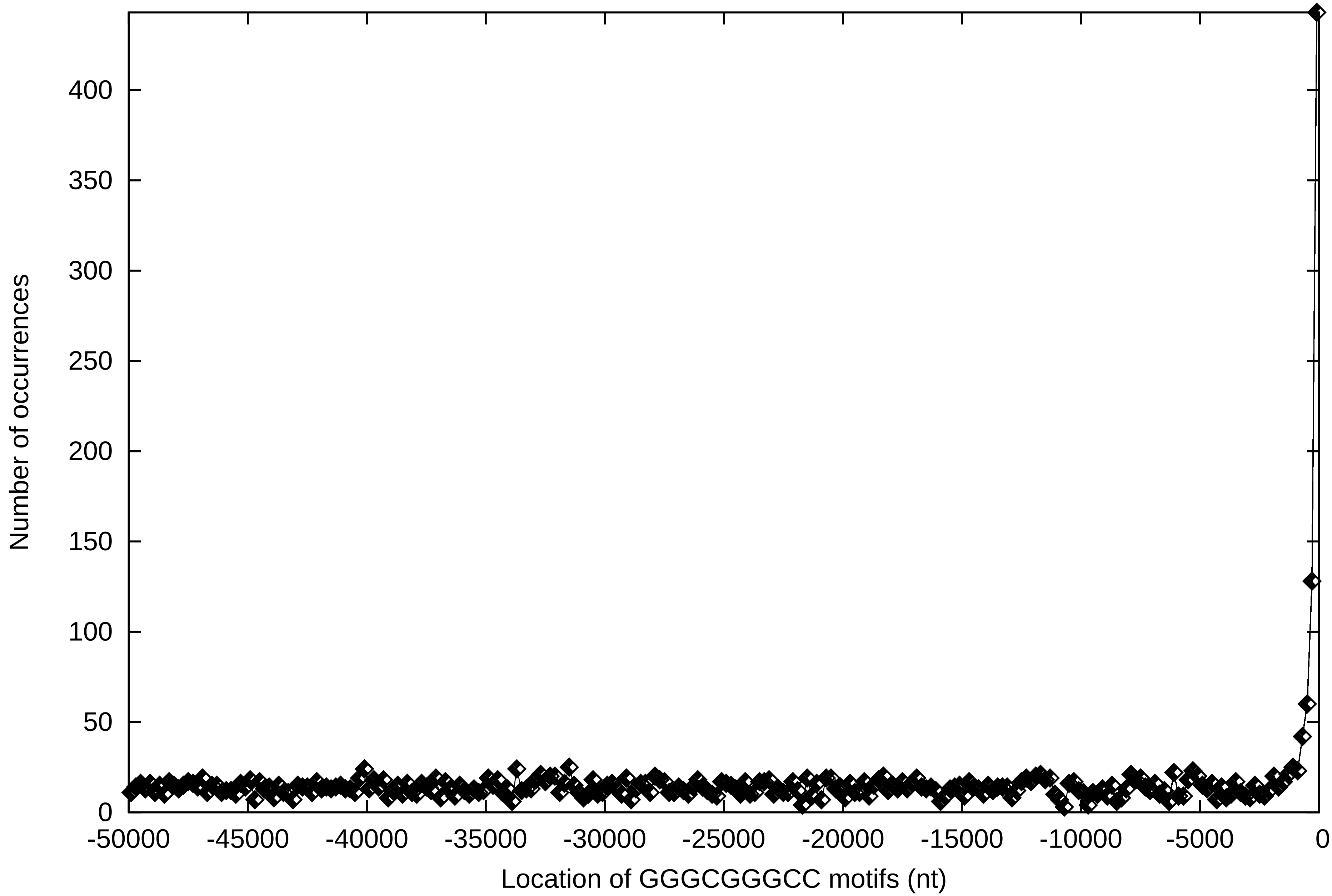

Number of occurrences
400
350
300
250
200
150
100
50
0
-50000
-45000
-40000
-35000
-30000
-25000
-20000
-15000
-10000
-5000
0
Location of GGGCGGGCC motifs (nt)

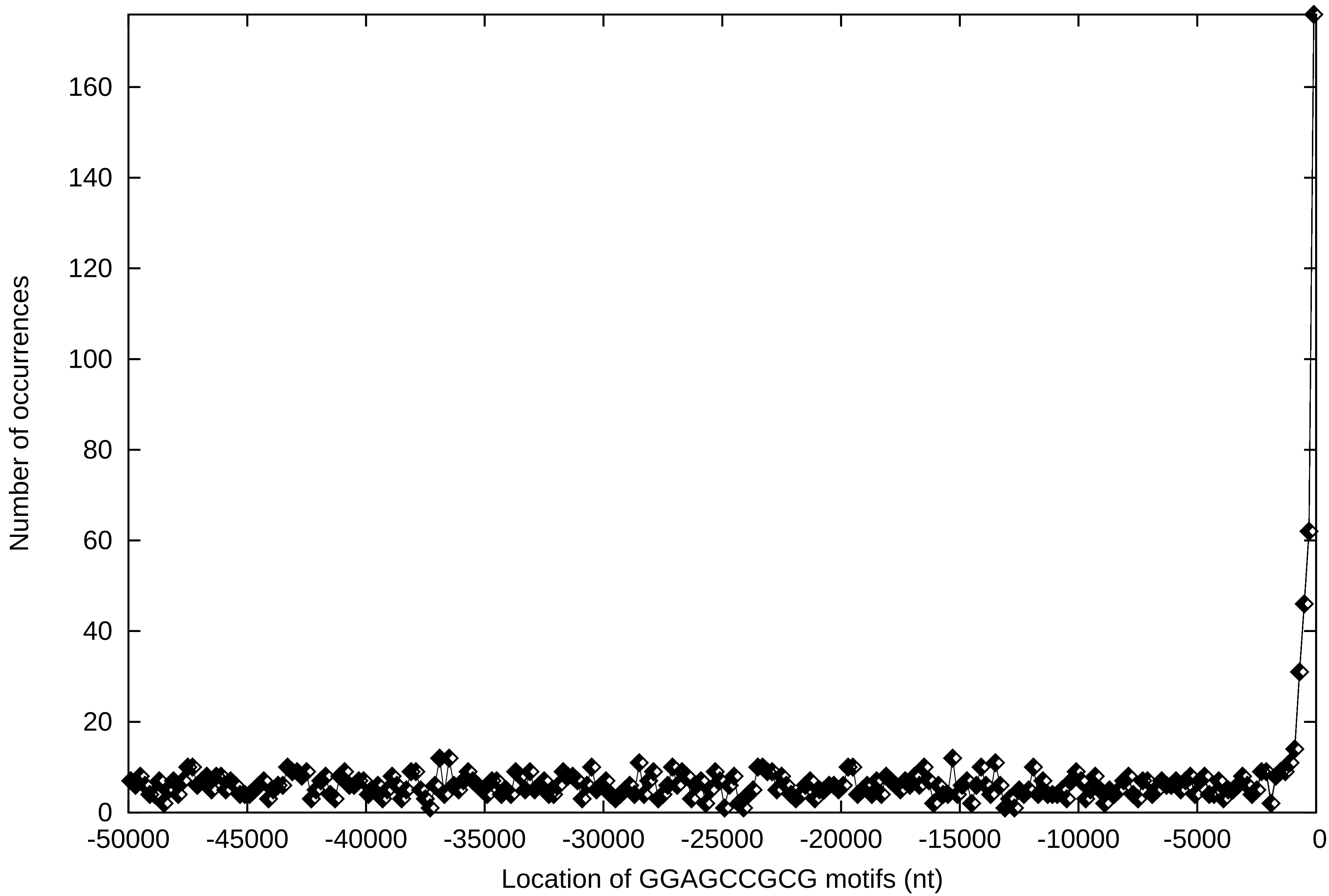

Number of occurrences
0
20
40
60
80
100
120
140
160
-50000
-45000
-40000
-35000
-30000
-25000
-20000
-15000
-10000
-5000
0
Location of GGAGCCGCG motifs (nt)

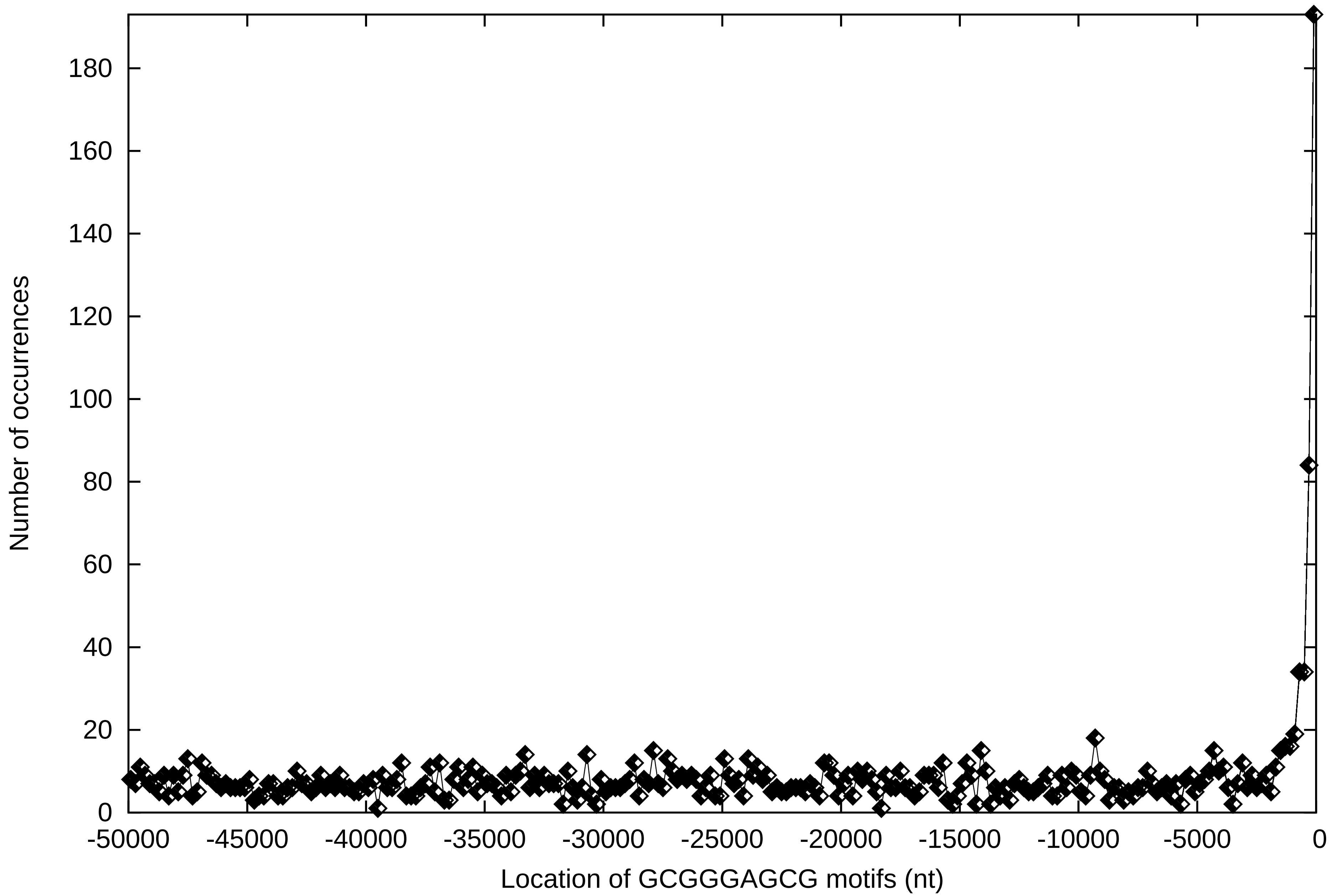

Number of occurrences
0
20
40
60
80
100
120
140
160
180
-50000
-45000
-40000
-35000
-30000
-25000
-20000
-15000
-10000
-5000
0
Location of GCGGGAGCG motifs (nt)

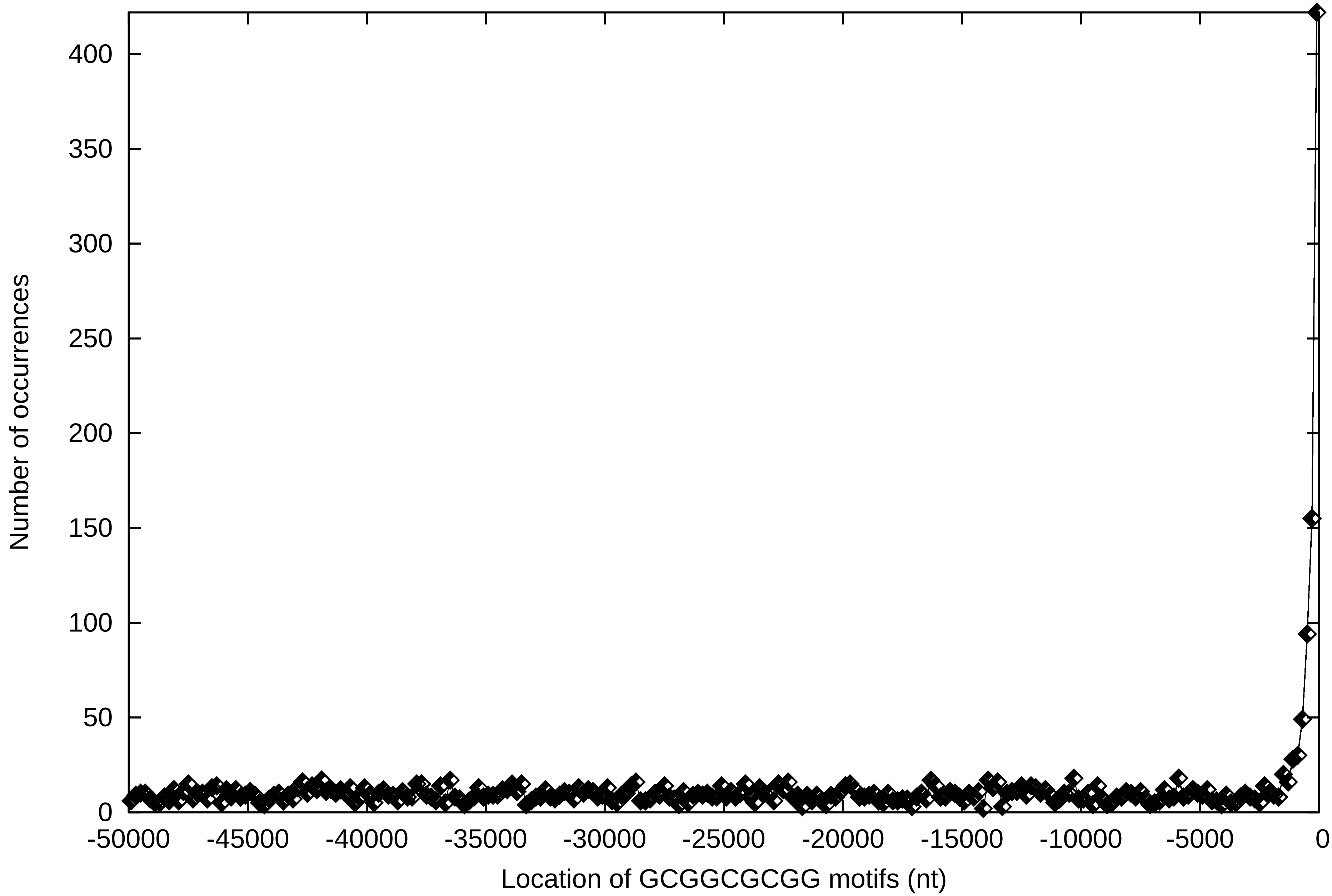

Number of occurrences
400
350
300
250
200
150
100
50
0
-50000
-45000
-40000
-35000
-30000
-25000
-20000
-15000
-10000
-5000
0
Location of GCGGCGCGG motifs (nt)

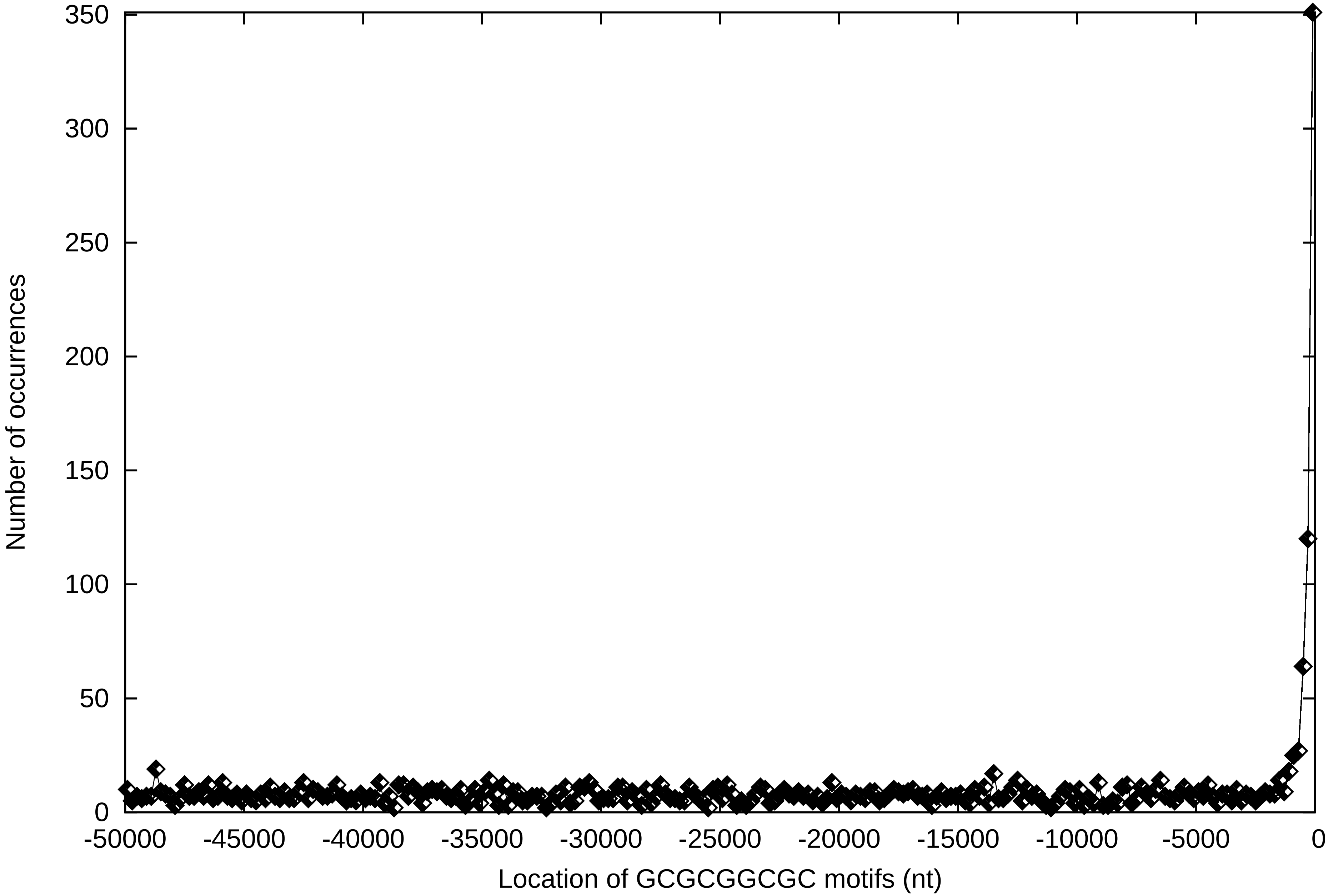

350
300
250
200
150
100
50
0
-50000
-45000
-40000
-35000
-30000
-25000
-20000
-15000
-10000
-5000
0
Number of occurrences
Location of GCGCGGCGC motifs (nt)

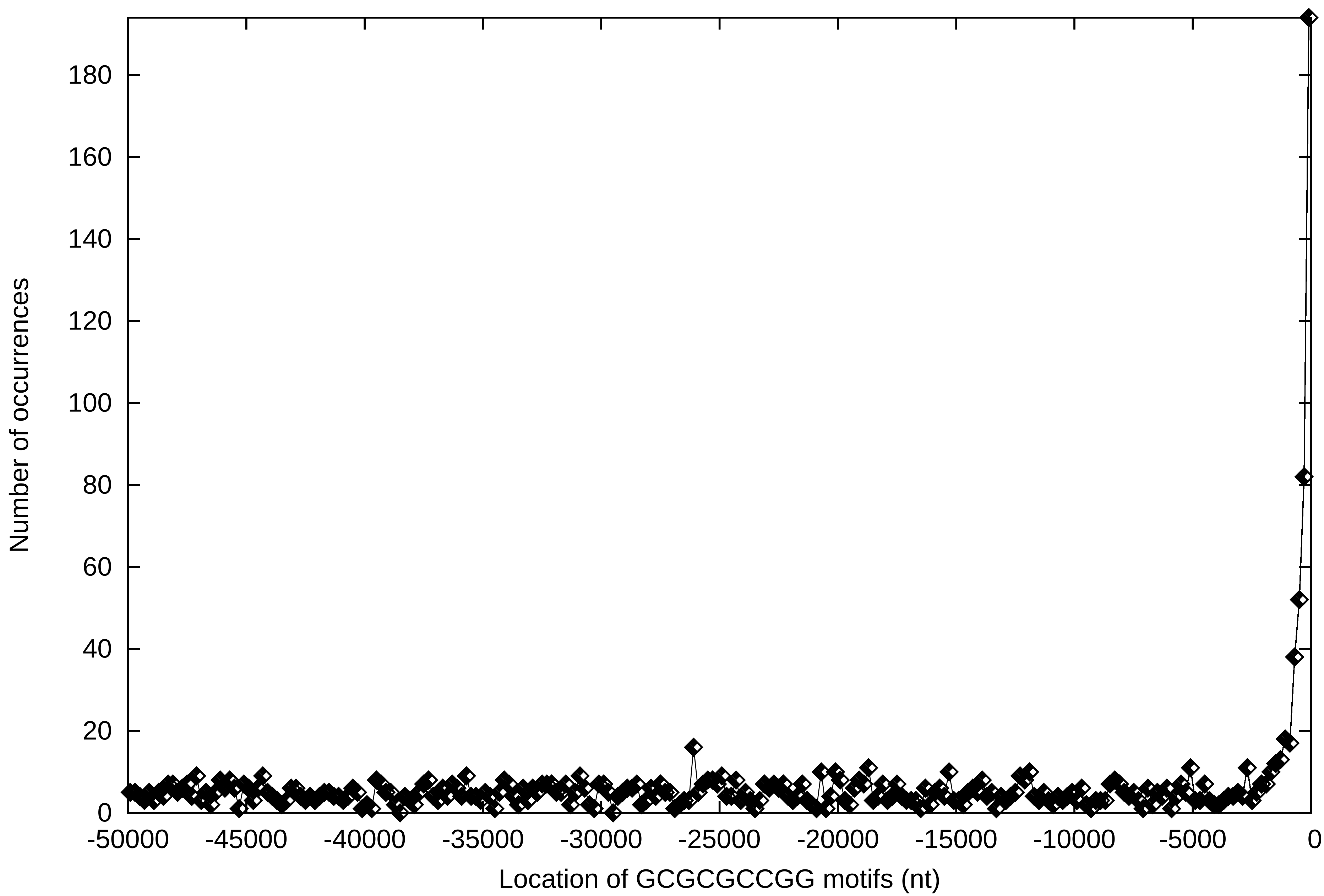

Number of occurrences
0
20
40
60
80
100
120
140
160
180
-50000
-45000
-40000
-35000
-30000
-25000
-20000
-15000
-10000
-5000
0
Location of GCGCGCCGG motifs (nt)

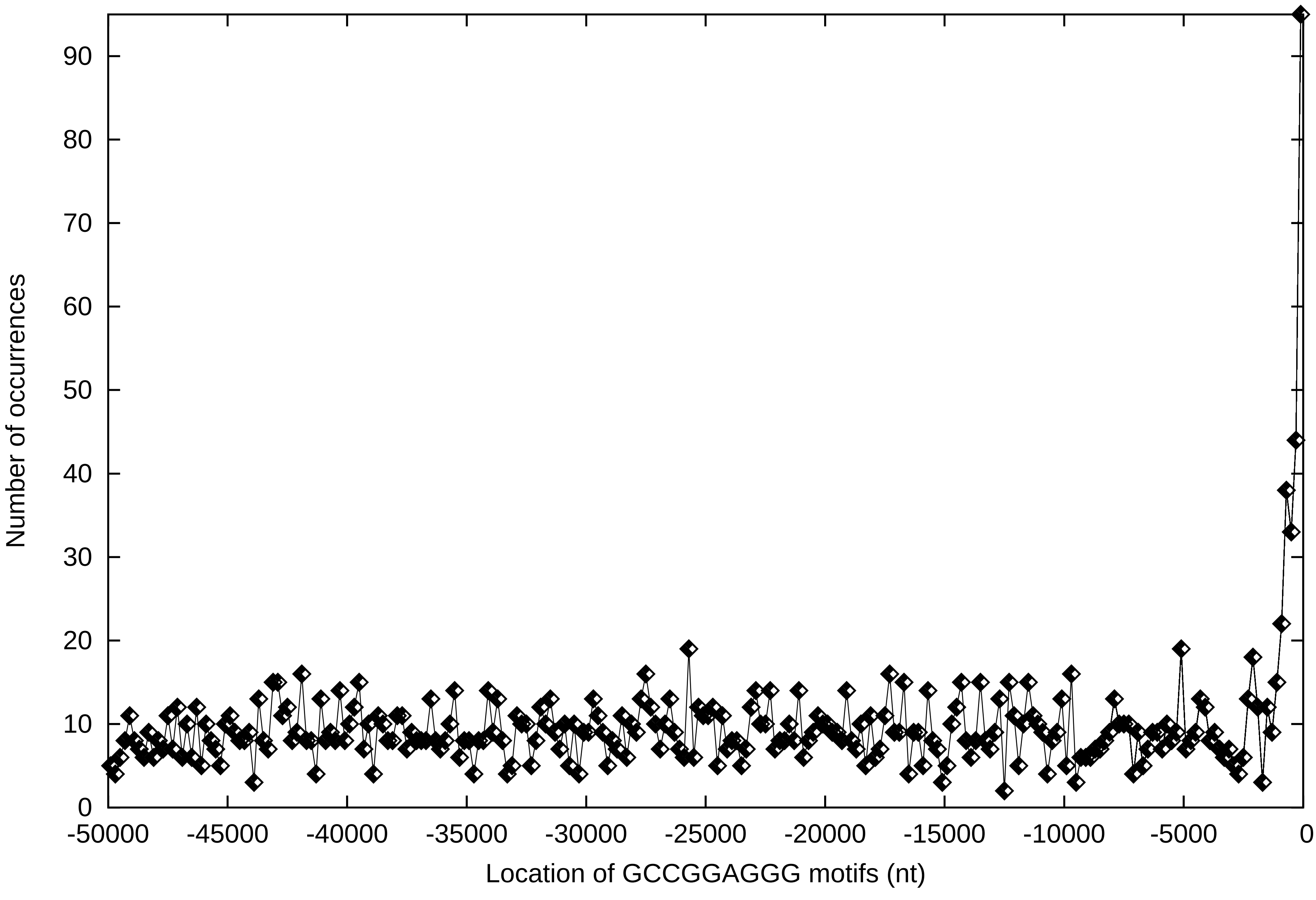

Number of occurrences
0
10
20
30
40
50
60
70
80
90
-50000
-45000
-40000
-35000
-30000
-25000
-20000
-15000
-10000
-5000
0
Location of GCCGGAGGG motifs (nt)

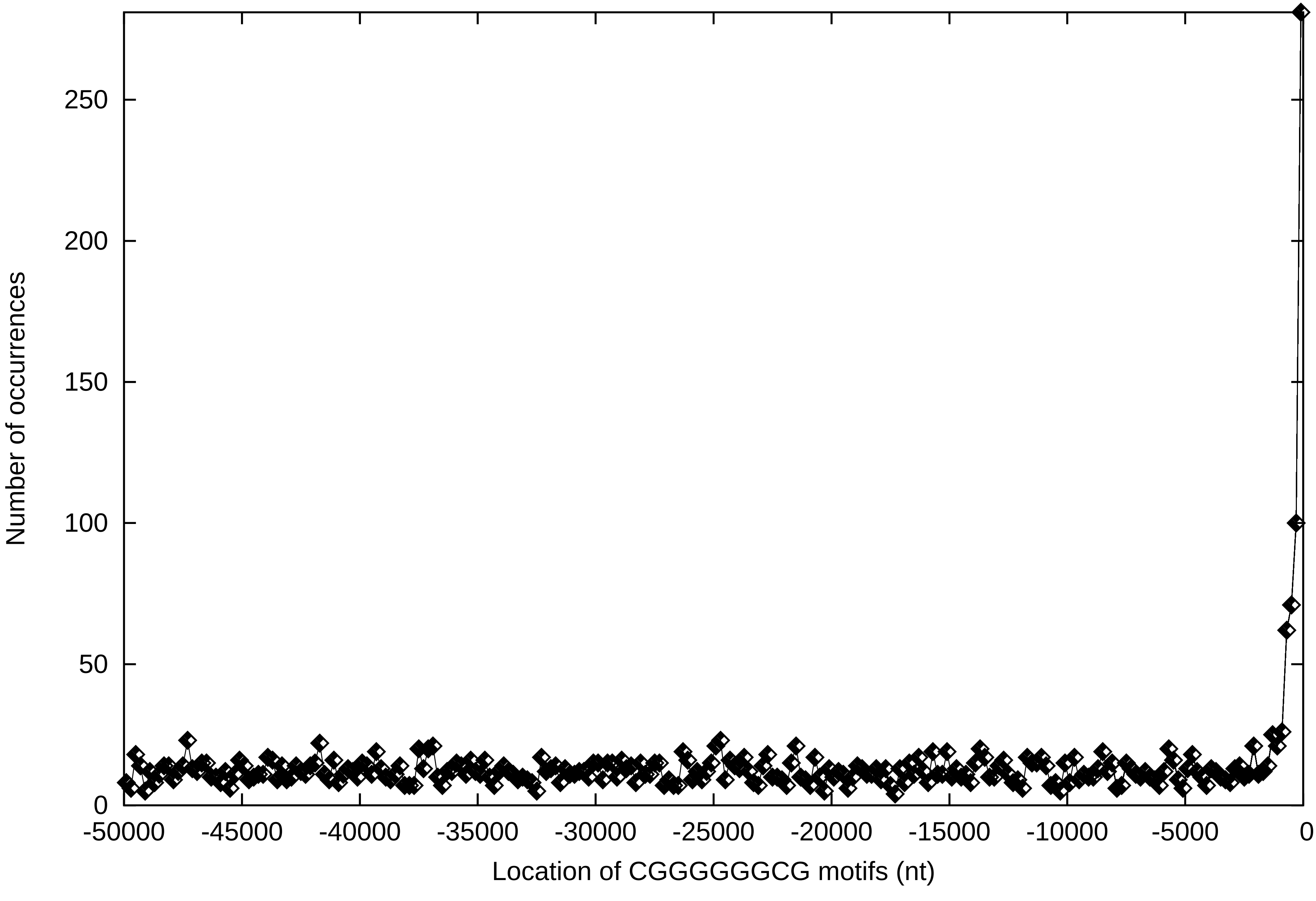

Number of occurrences
0
50
100
150
200
250
-50000
-45000
-40000
-35000
-30000
-25000
-20000
-15000
-10000
-5000
0
Location of CGGGGGGCG motifs (nt)

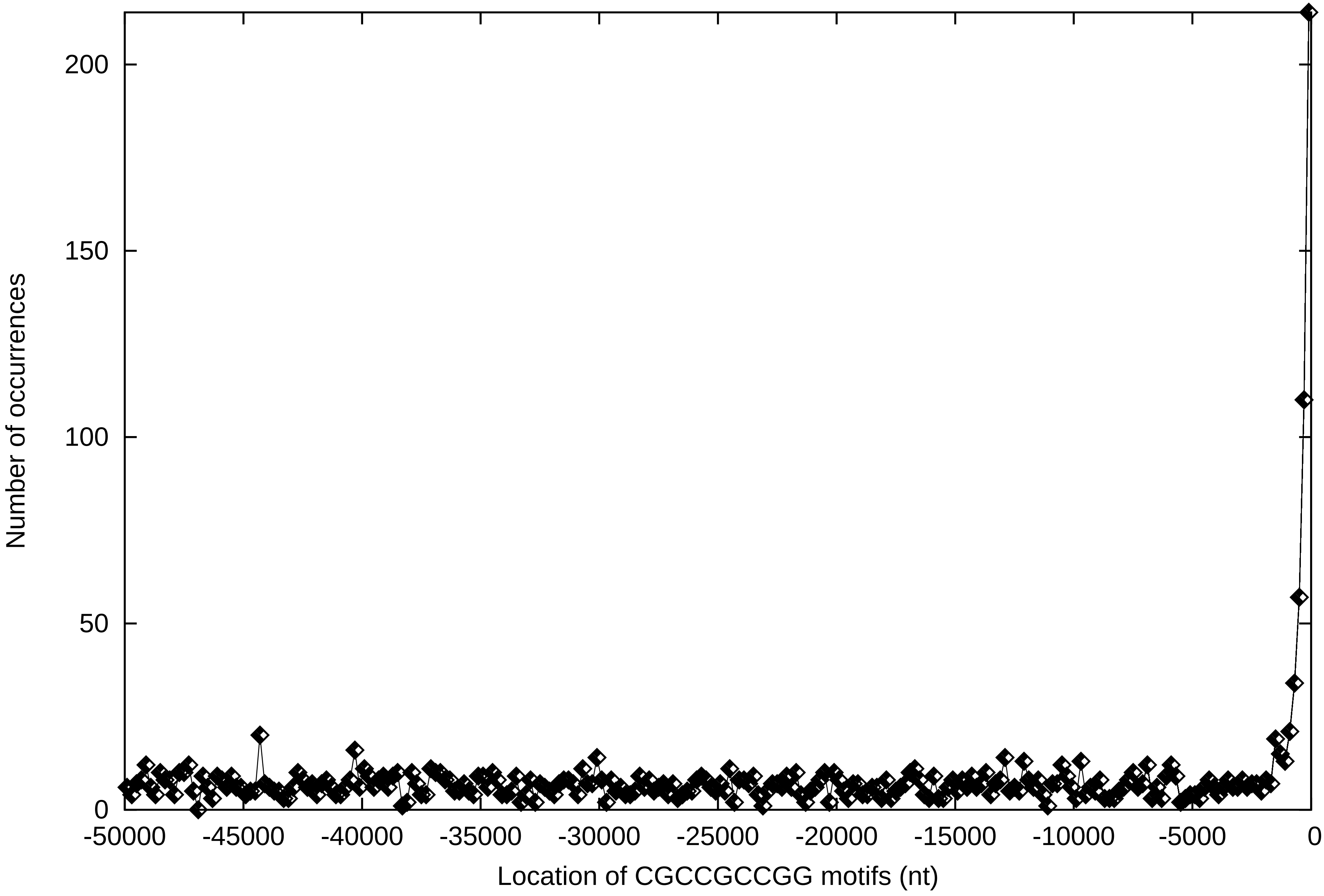
Number of occurrences
0
50
100
150
200
-50000
-45000
-40000
-35000
-30000
-25000
-20000
-15000
-10000
-5000
0
Location of CGCCGCCGG motifs (nt)

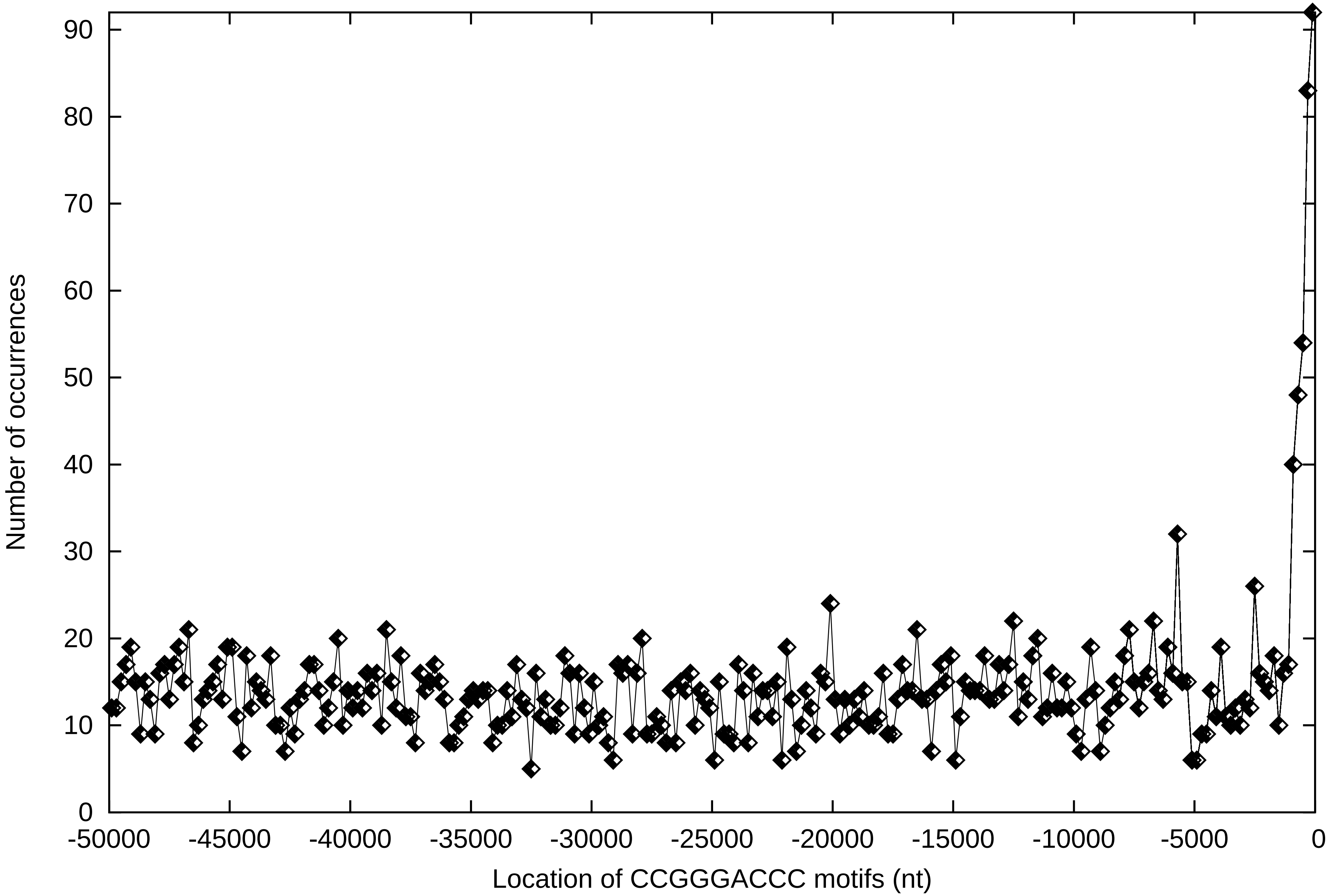
Number of occurrences
Location of CCGGGACCC motifs (nt)
0
10
20
30
40
50
60
70
80
90
-50000
-45000
-40000
-35000
-30000
-25000
-20000
-15000
-10000
-5000
0

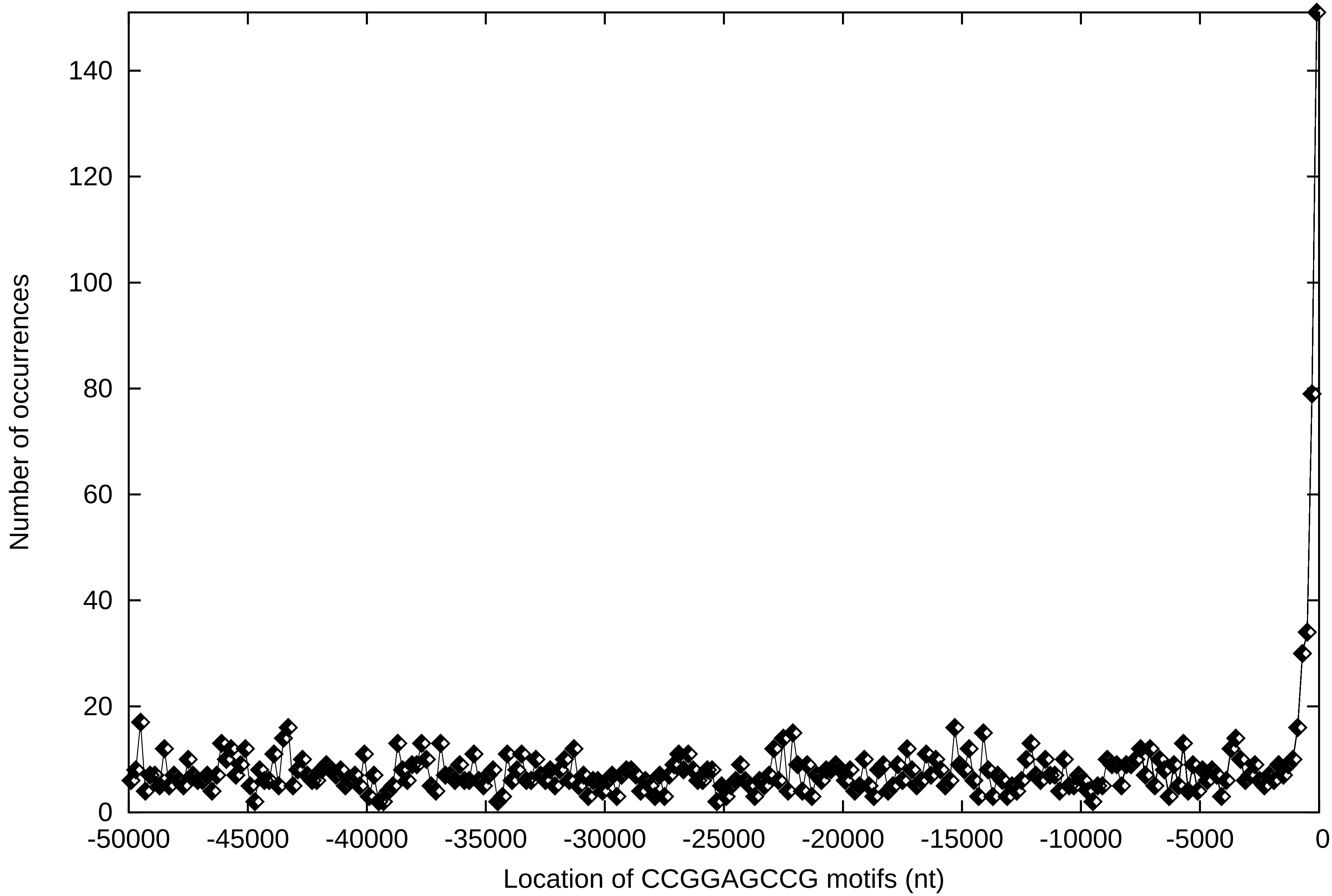

140
120
100
80
60
40
20
0
Number of occurrences
-50000
-45000
-40000
-35000
-30000
-25000
-20000
-15000
-10000
-5000
0
Location of CCGGAGCCG motifs (nt)

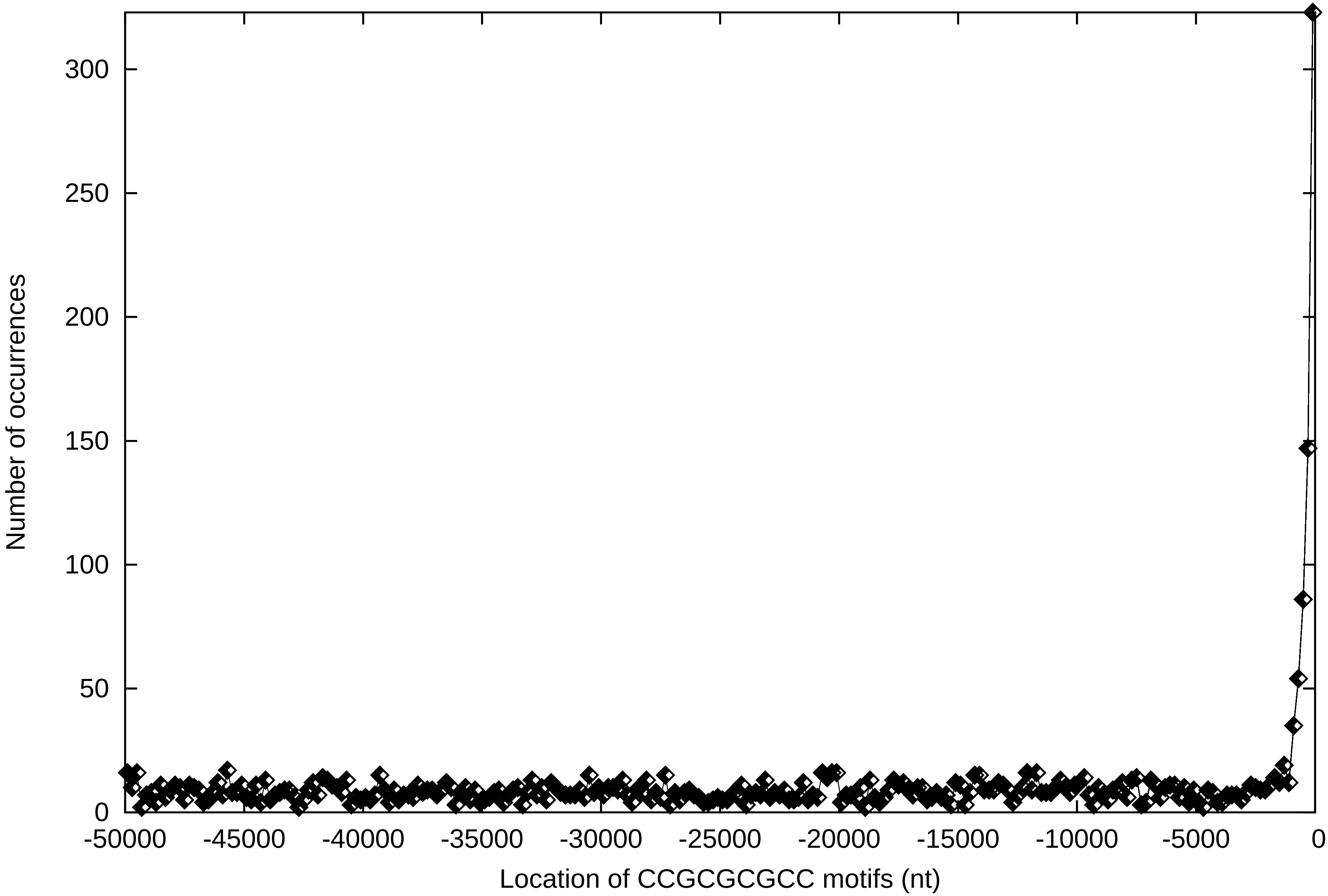

Number of occurrences
300
250
200
150
100
50
0
-50000
-45000
-40000
-35000
-30000
-25000
-20000
-15000
-10000
-5000
0
Location of CCGCGCGCC motifs (nt)

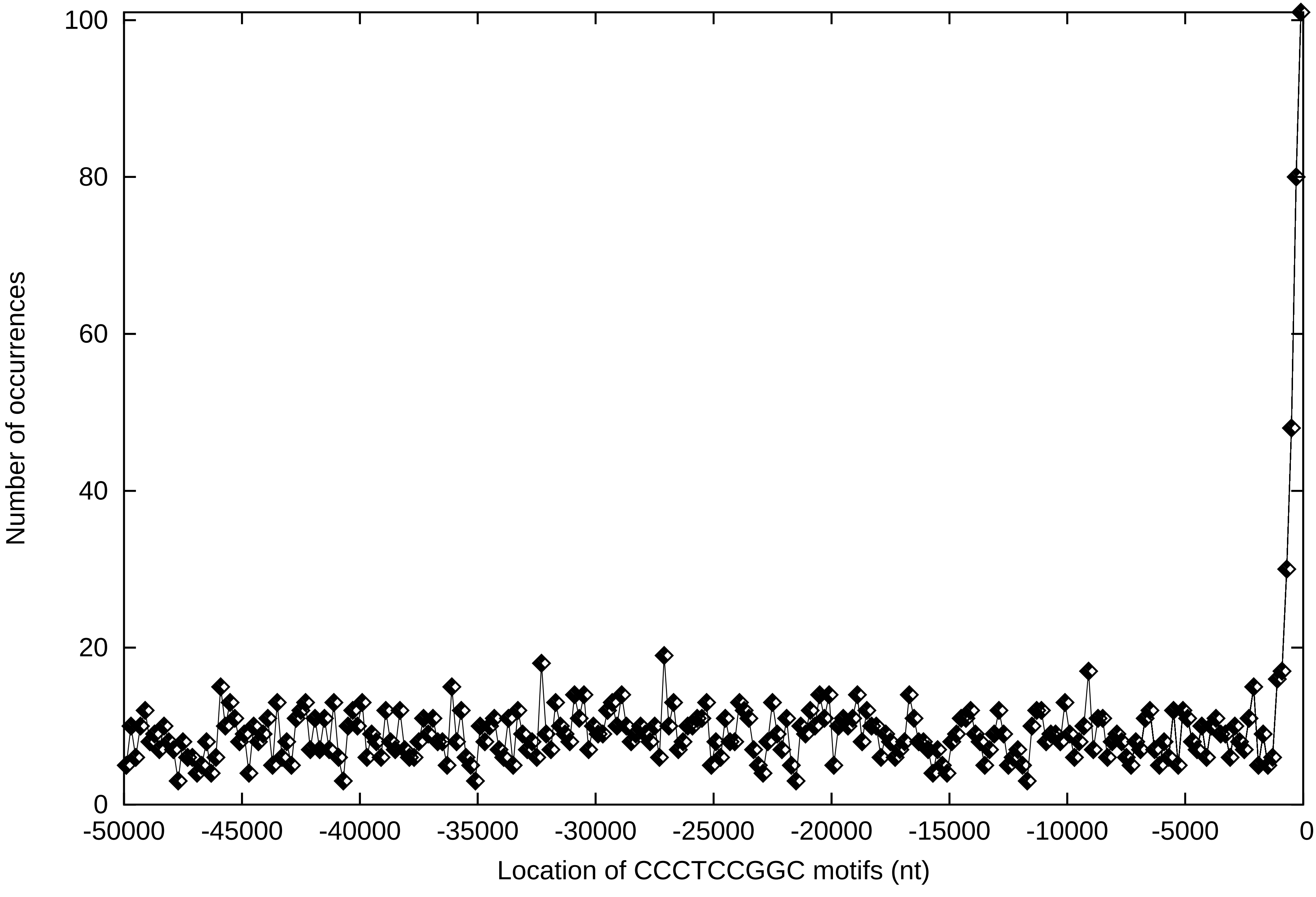

Number of occurrences
100
80
60
40
20
0
-50000
-45000
-40000
-35000
-30000
-25000
-20000
-15000
-10000
-5000
0
Location of CCCTCCGGC motifs (nt)

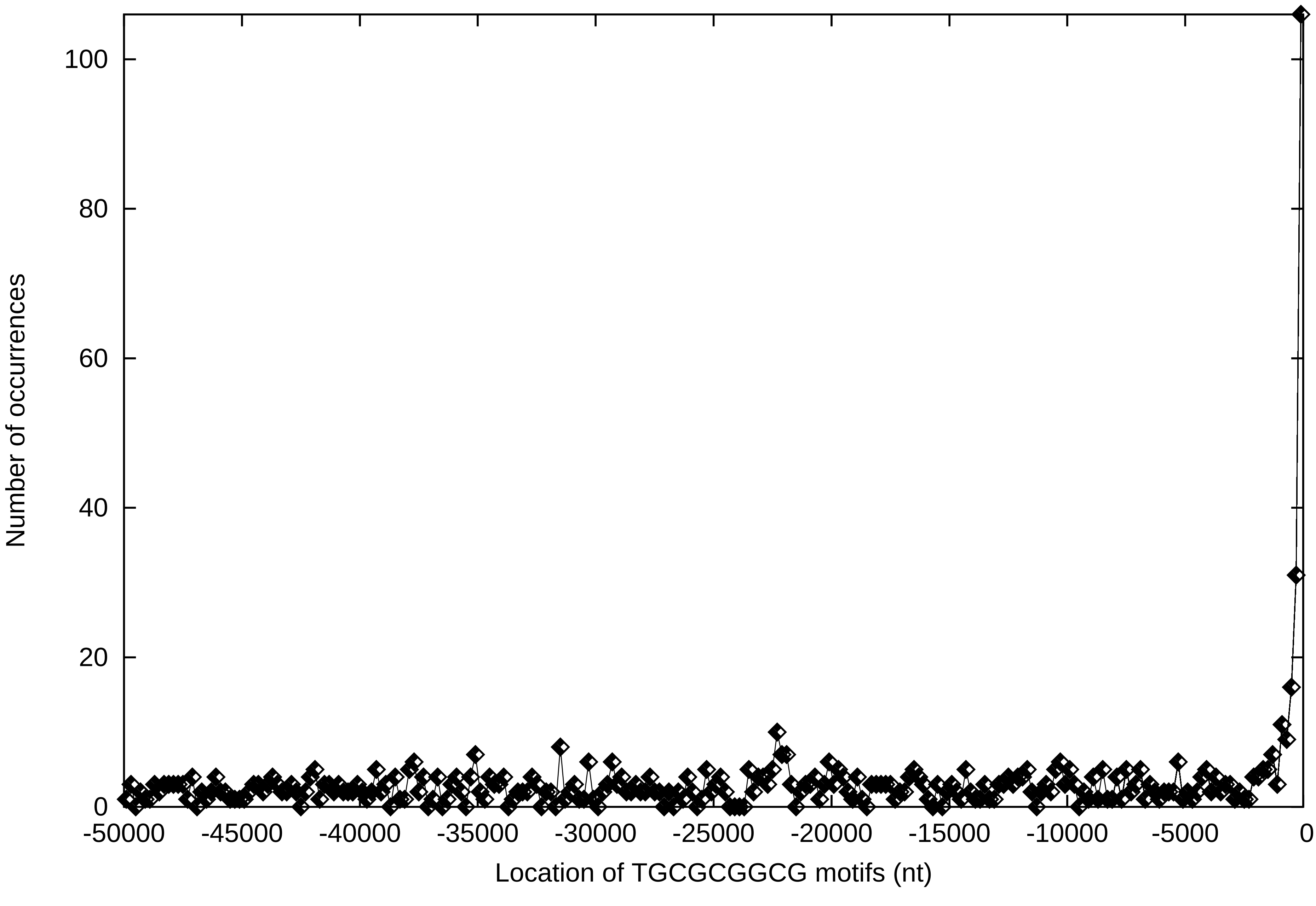

Number of occurrences
0
20
40
60
80
100
-50000
-45000
-40000
-35000
-30000
-25000
-20000
-15000
-10000
-5000
0
Location of TGCGCGGCG motifs (nt)

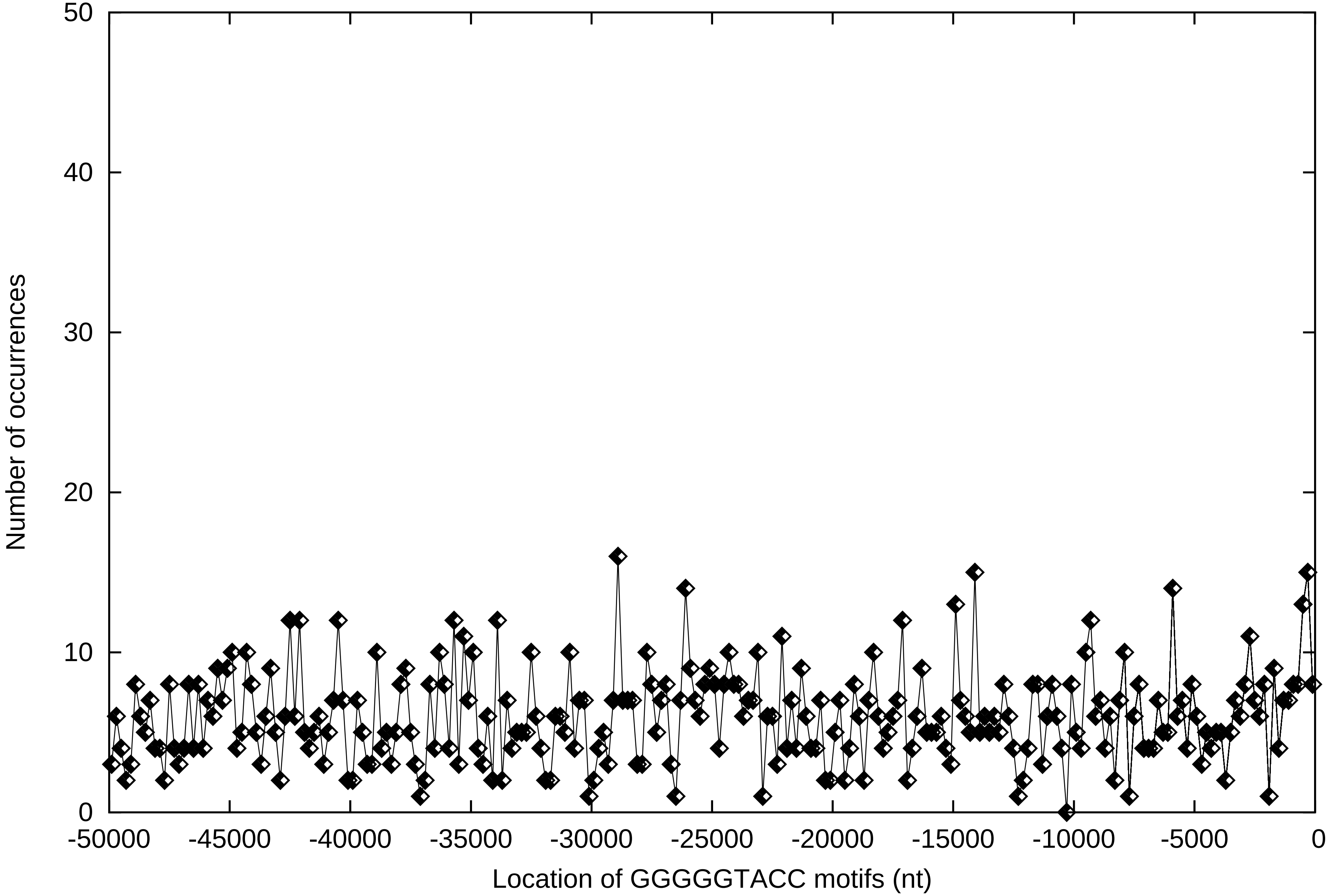

50
40
30
20
10
0
Number of occurrences
-50000
-45000
-40000
-35000
-30000
-25000
-20000
-15000
-10000
-5000
0
Location of GGGGGTACC motifs (nt)

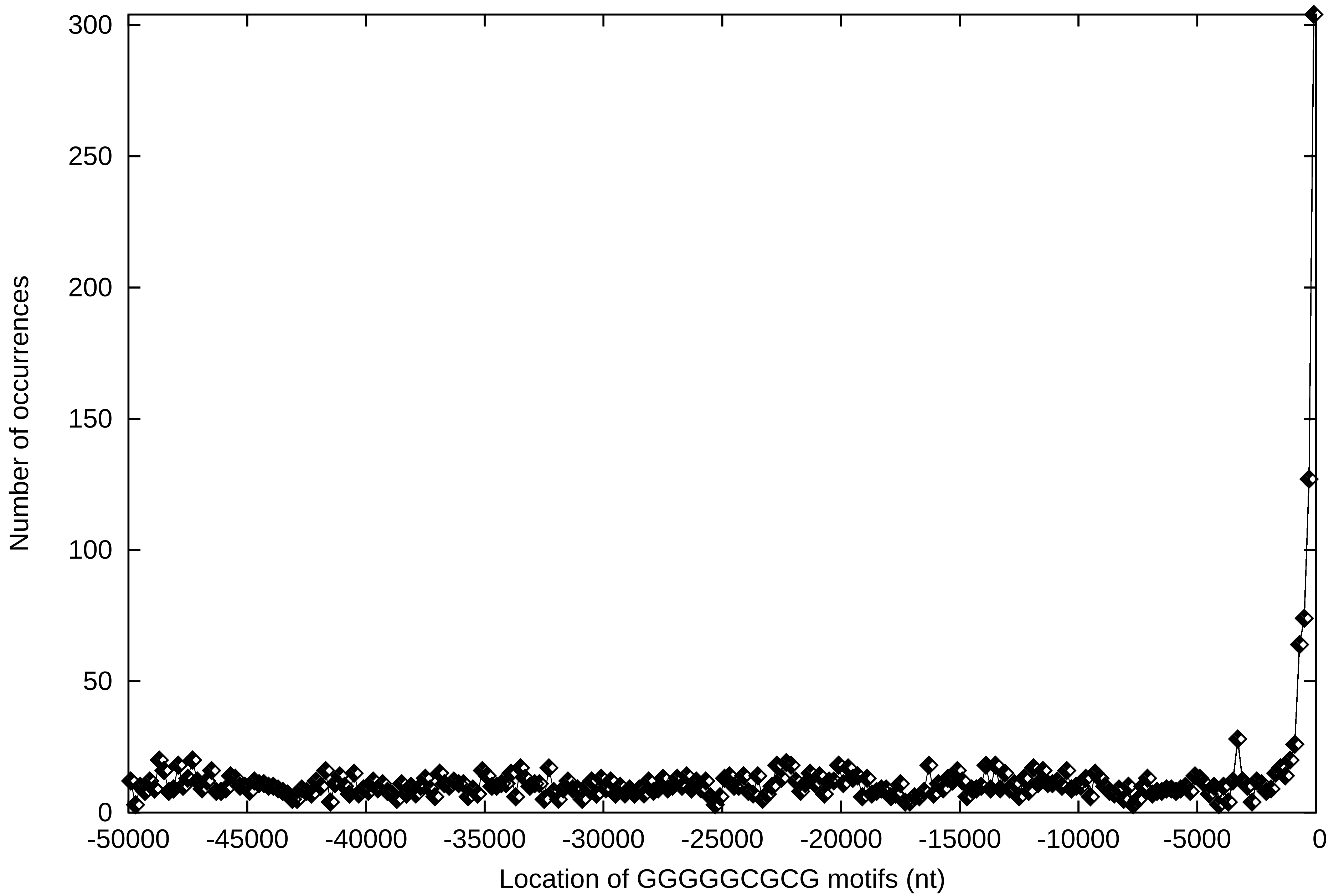

300
250
200
150
100
50
0
Number of occurrences
-50000
-45000
-40000
-35000
-30000
-25000
-20000
-15000
-10000
-5000
0
Location of GGGGGCGCG motifs (nt)

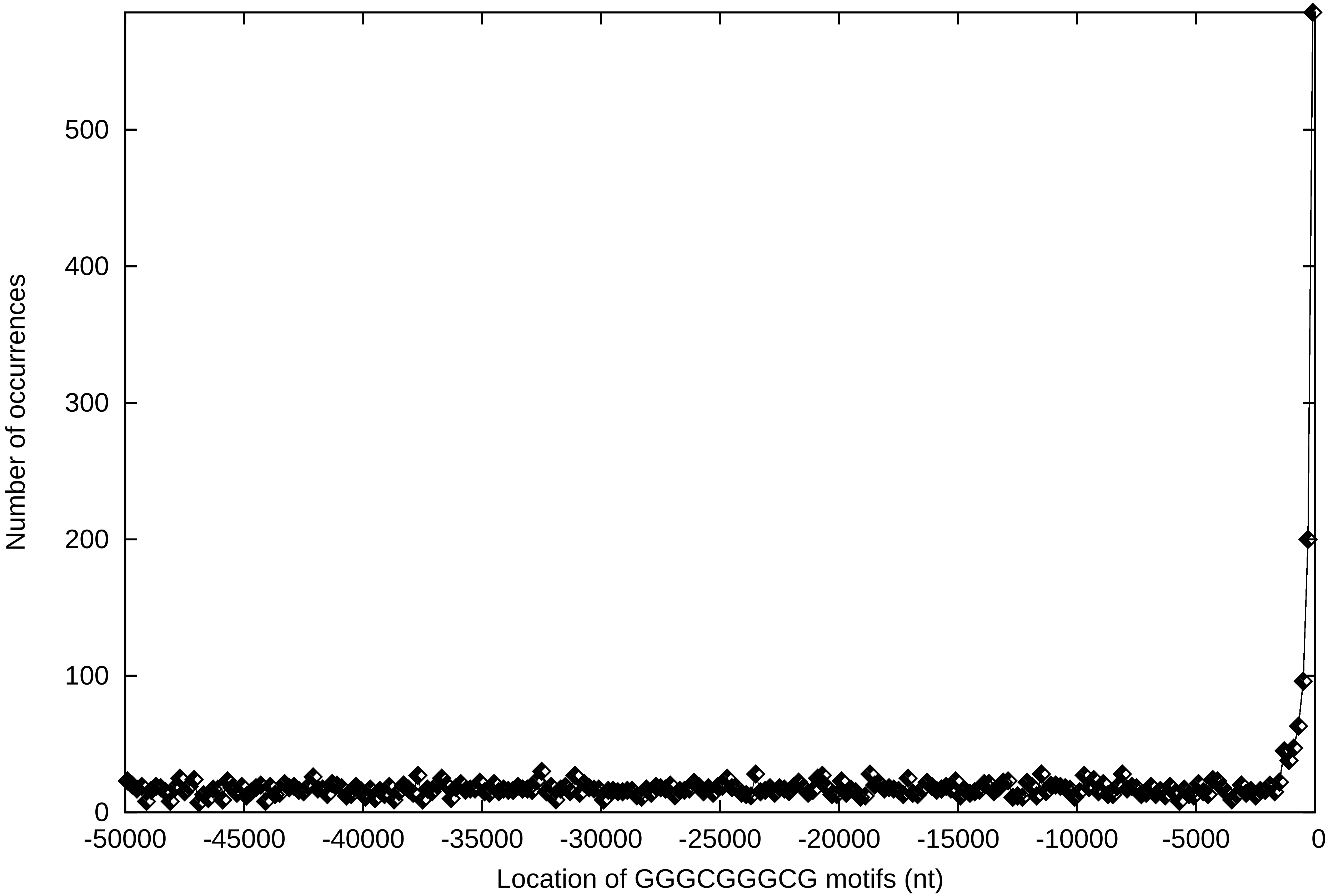

Number of occurrences
0
100
200
300
400
500
-50000
-45000
-40000
-35000
-30000
-25000
-20000
-15000
-10000
-5000
0
Location of GGGCGGGCG motifs (nt)

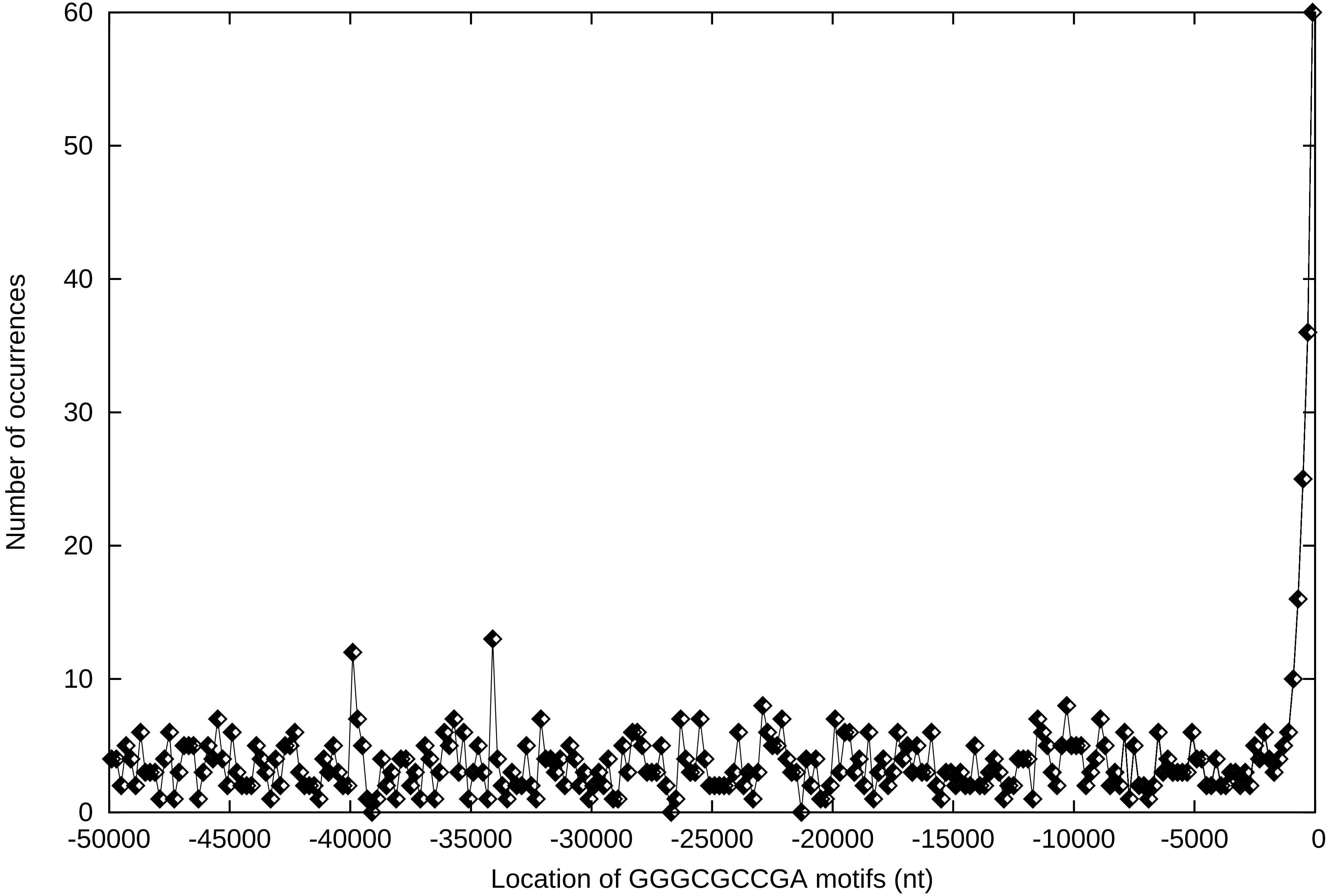

0
10
20
30
40
50
60
Number of occurrences
-50000
-45000
-40000
-35000
-30000
-25000
-20000
-15000
-10000
-5000
0
Location of GGGCGCCGA motifs (nt)

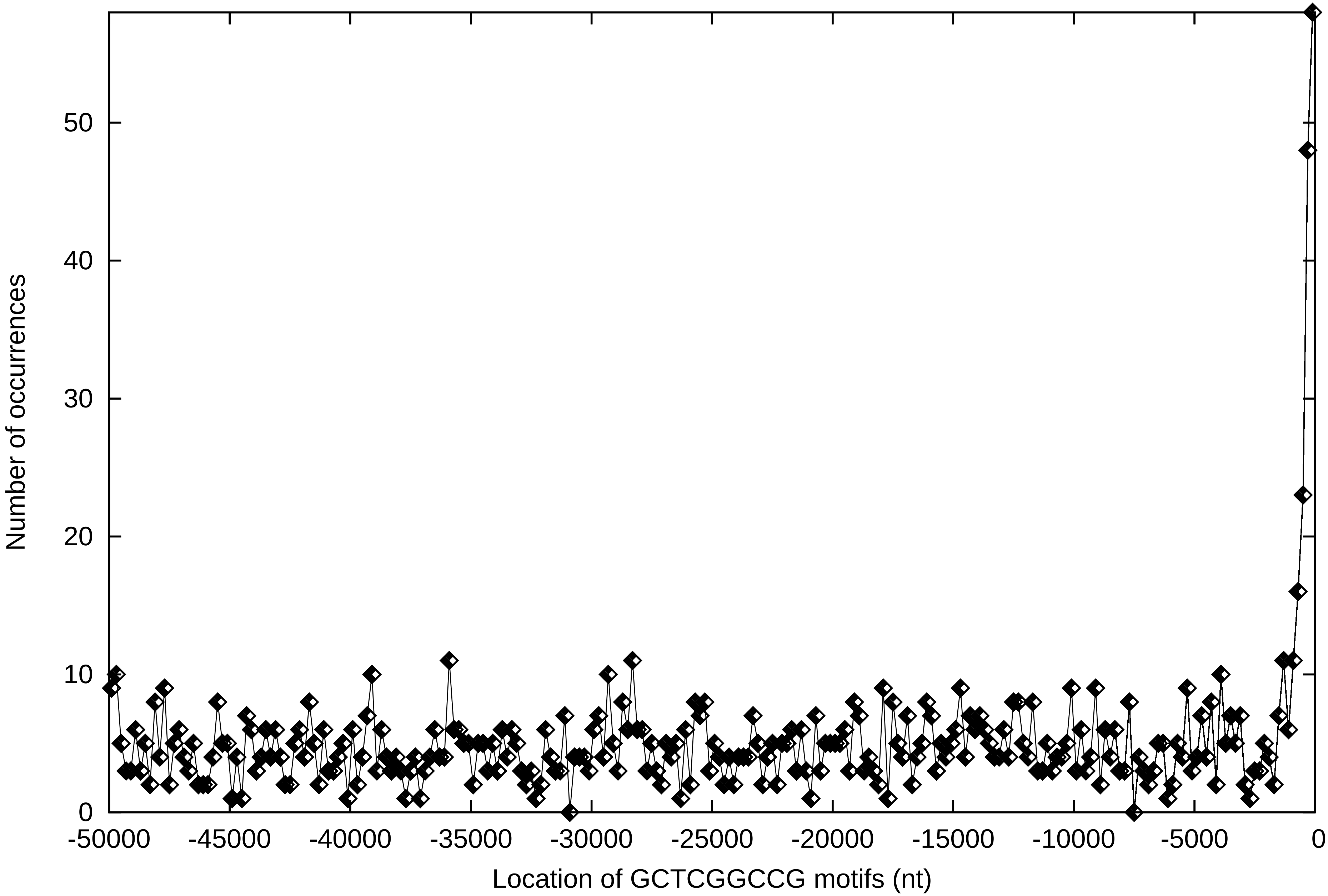

Number of occurrences
0
10
20
30
40
50
-50000
-45000
-40000
-35000
-30000
-25000
-20000
-15000
-10000
-5000
0
Location of GCTCGGCCG motifs (nt)

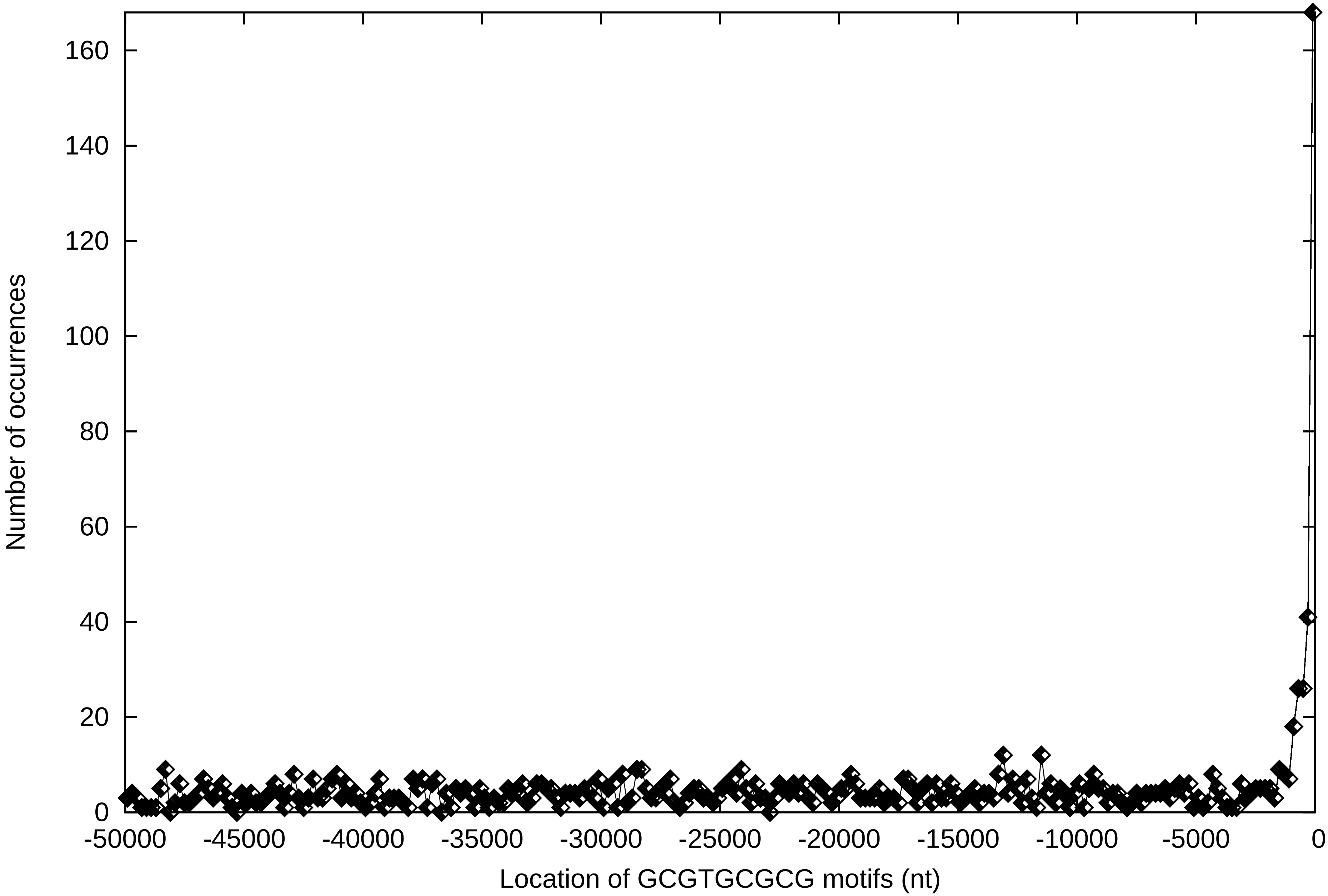
Number of occurrences
160
140
120
100
80
60
40
20
0
-50000
-45000
-40000
-35000
-30000
-25000
-20000
-15000
-10000
-5000
0
Location of GCGTGCGCG motifs (nt)

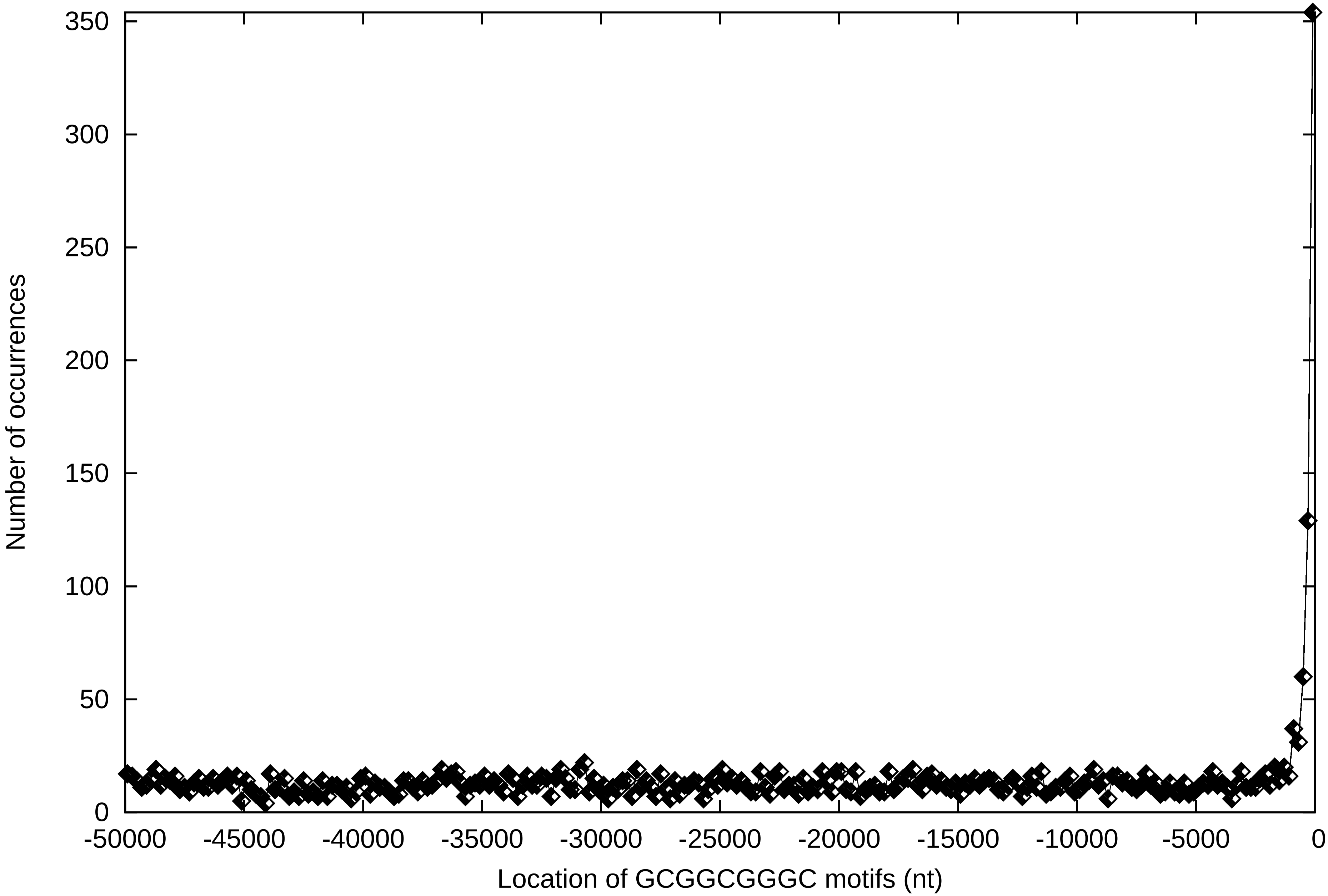

350
300
250
200
150
100
50
0
-50000
-45000
-40000
-35000
-30000
-25000
-20000
-15000
-10000
-5000
0
Number of occurrences
Location of GCGGCGGGC motifs (nt)

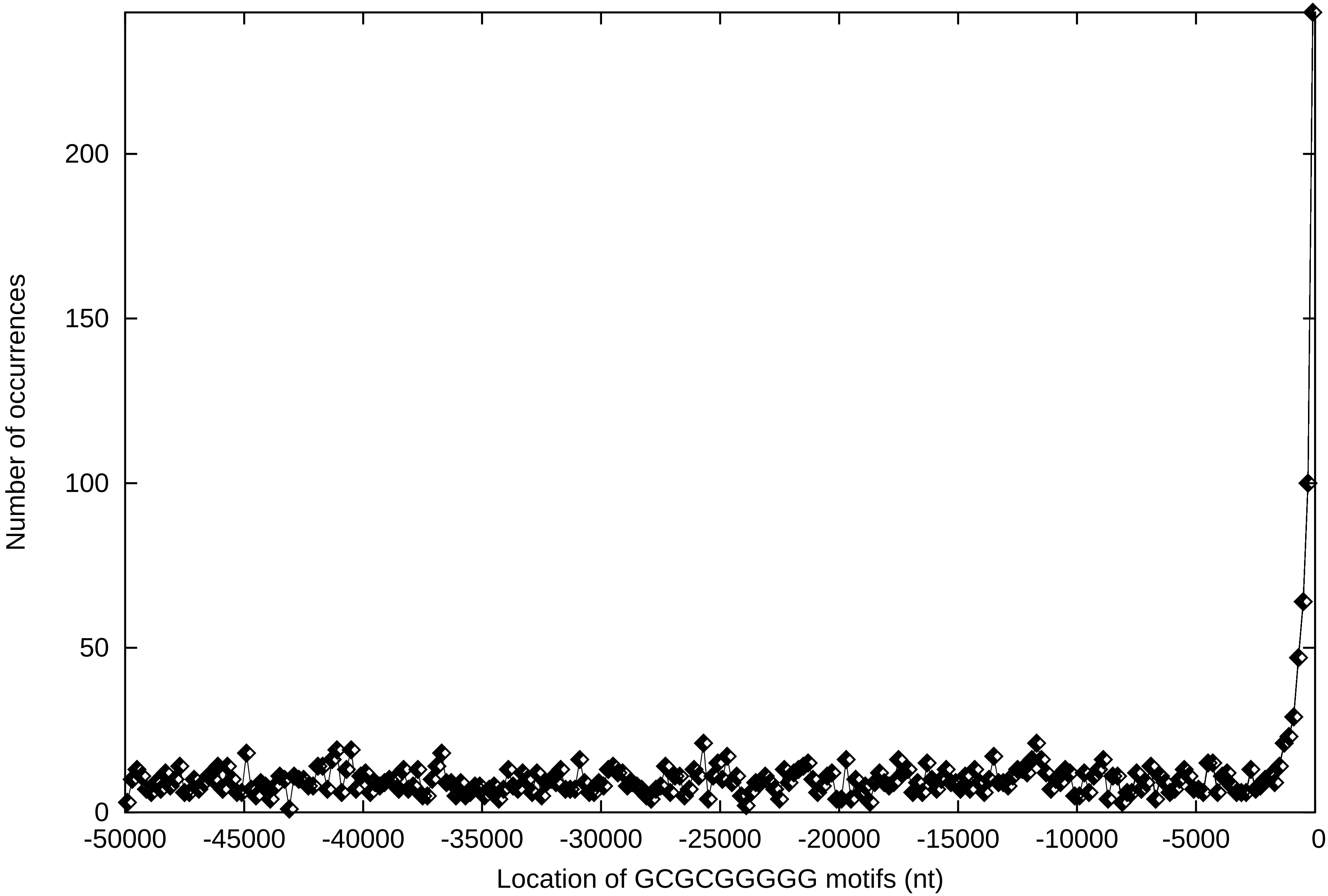

Number of occurrences
0
50
100
150
200
-50000
-45000
-40000
-35000
-30000
-25000
-20000
-15000
-10000
-5000
0
Location of GCGCGGGGG motifs (nt)

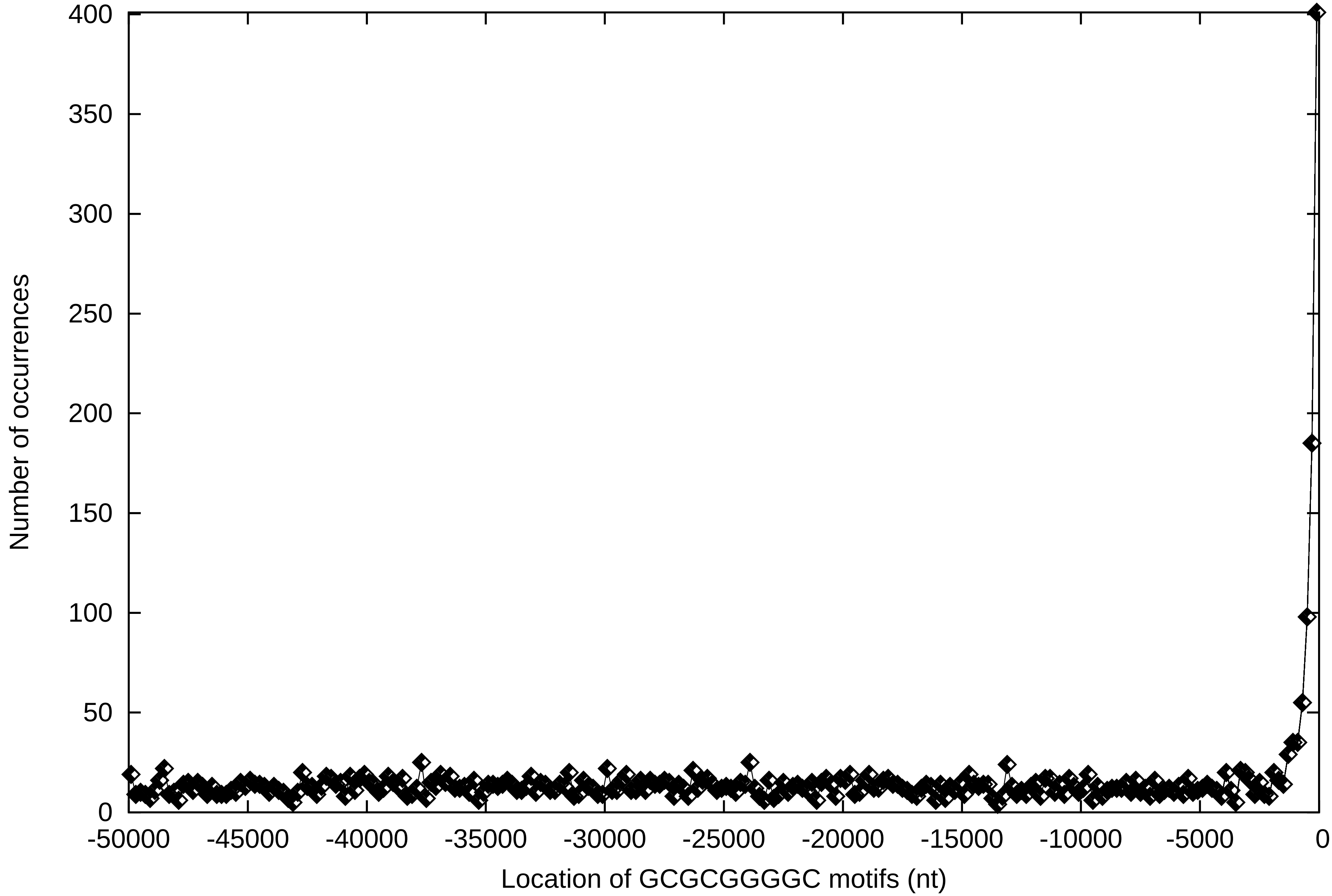

400
350
300
250
200
150
100
50
0
Number of occurrences
-50000
-45000
-40000
-35000
-30000
-25000
-20000
-15000
-10000
-5000
0
Location of GCGCGGGGC motifs (nt)

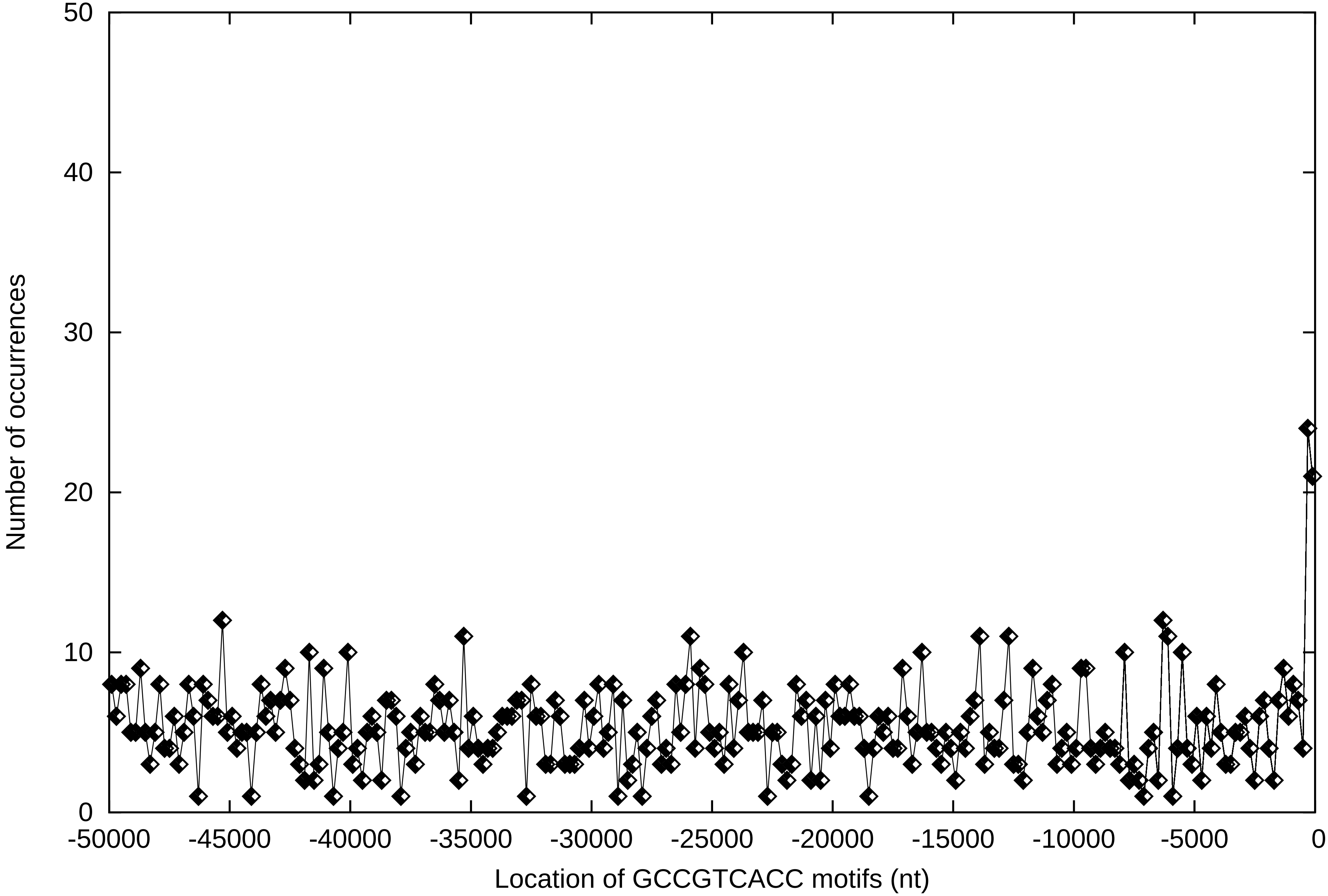

50
40
30
20
10
0
-50000
-45000
-40000
-35000
-30000
-25000
-20000
-15000
-10000
-5000
0
Number of occurrences
Location of GCCGTCACC motifs (nt)

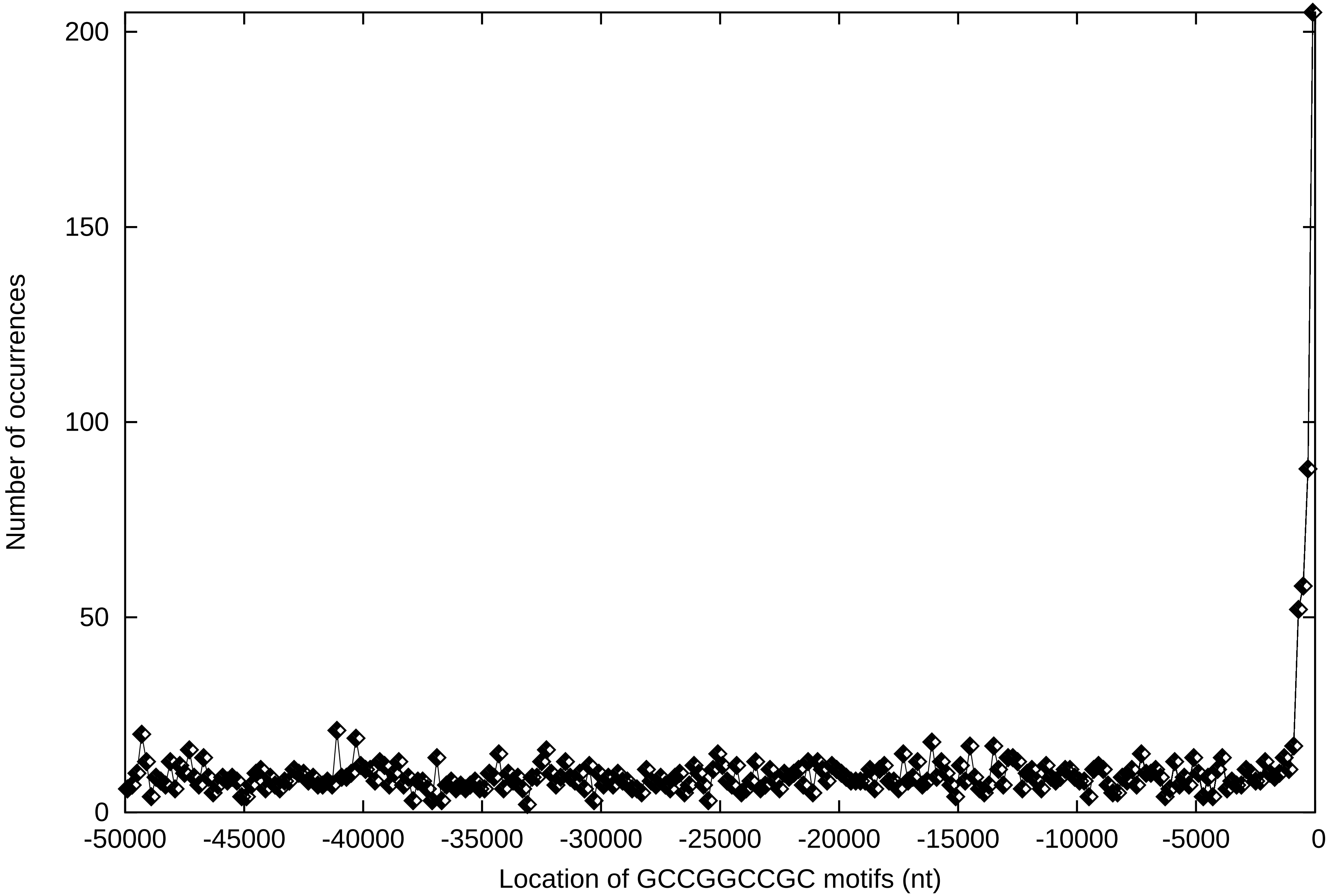

Number of occurrences
Location of GCCGGCCGC motifs (nt)
200
150
100
50
0
-50000
-45000
-40000
-35000
-30000
-25000
-20000
-15000
-10000
-5000
0

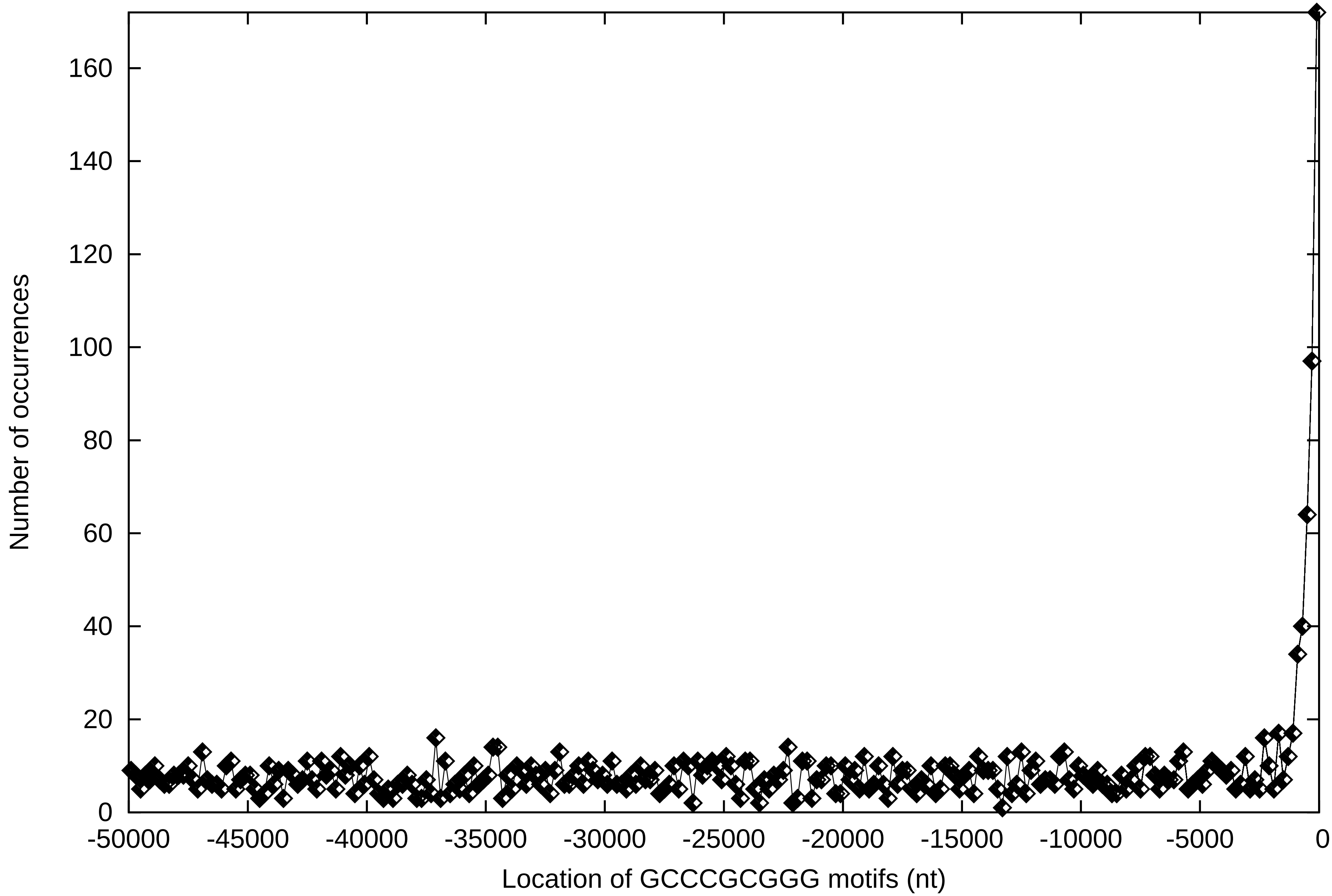
Number of occurrences
0
20
40
60
80
100
120
140
160
-50000
-45000
-40000
-35000
-30000
-25000
-20000
-15000
-10000
-5000
0
Location of GCCCGCGGG motifs (nt)

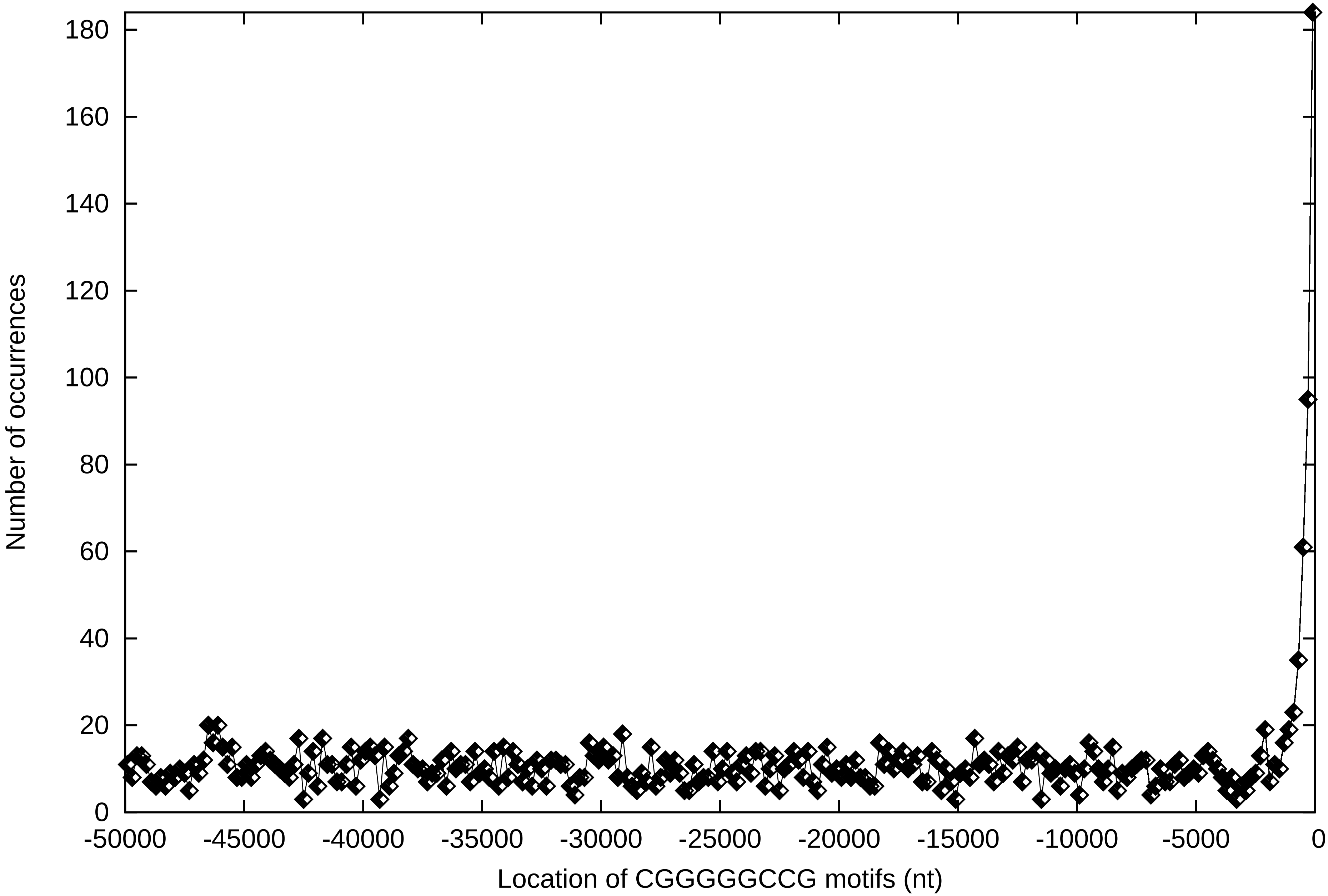

Number of occurrences
0
20
40
60
80
100
120
140
160
180
-50000
-45000
-40000
-35000
-30000
-25000
-20000
-15000
-10000
-5000
0
Location of CGGGGGCCG motifs (nt)

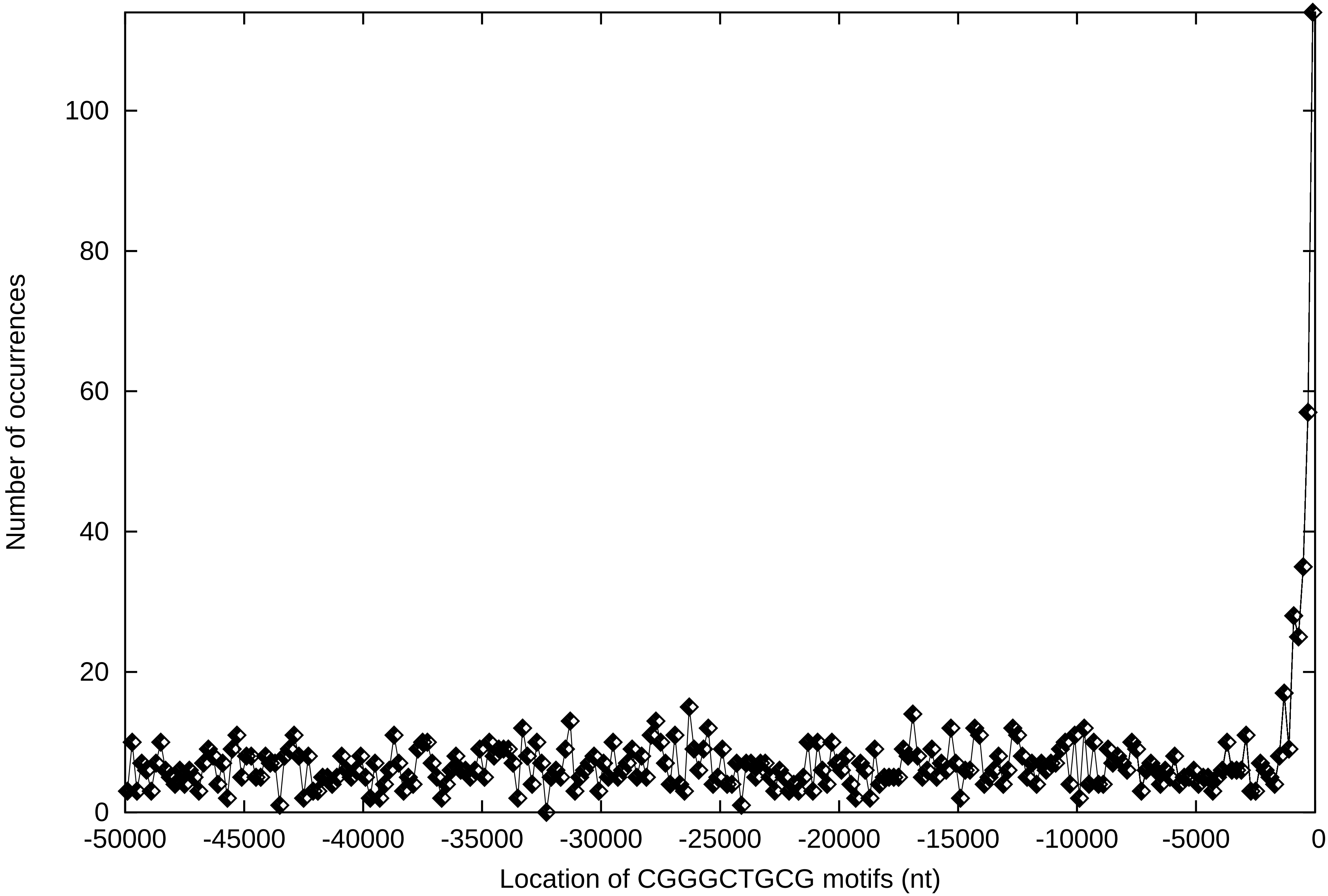

Number of occurrences
0
20
40
60
80
100
-50000
-45000
-40000
-35000
-30000
-25000
-20000
-15000
-10000
-5000
0
Location of CGGGCTGCG motifs (nt)

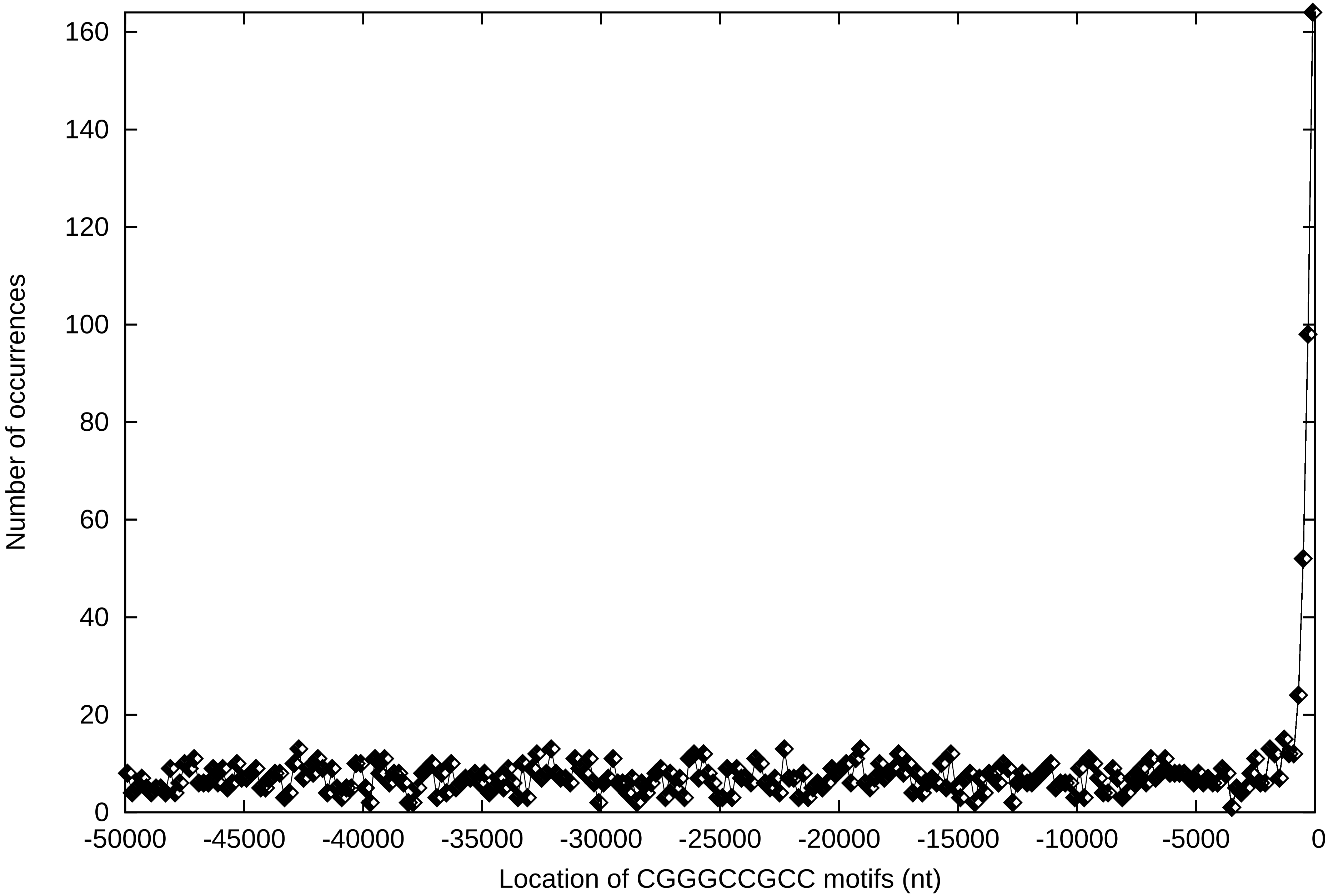

Number of occurrences
0
20
40
60
80
100
120
140
160
-50000
-45000
-40000
-35000
-30000
-25000
-20000
-15000
-10000
-5000
0
Location of CGGGCCGCC motifs (nt)

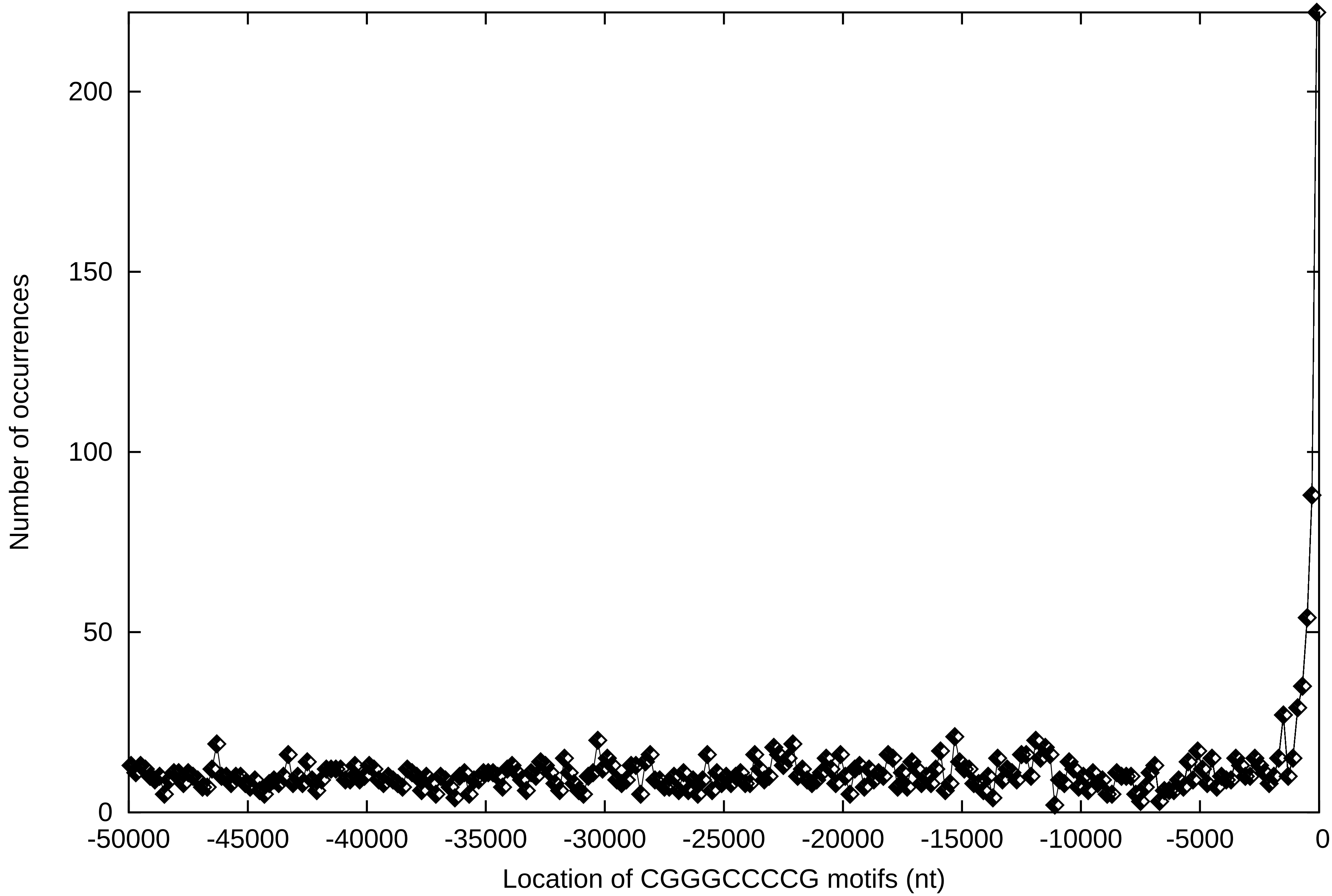

0
50
100
150
200
-50000
-45000
-40000
-35000
-30000
-25000
-20000
-15000
-10000
-5000
0
Number of occurrences
Location of CGGGCCCCG motifs (nt)

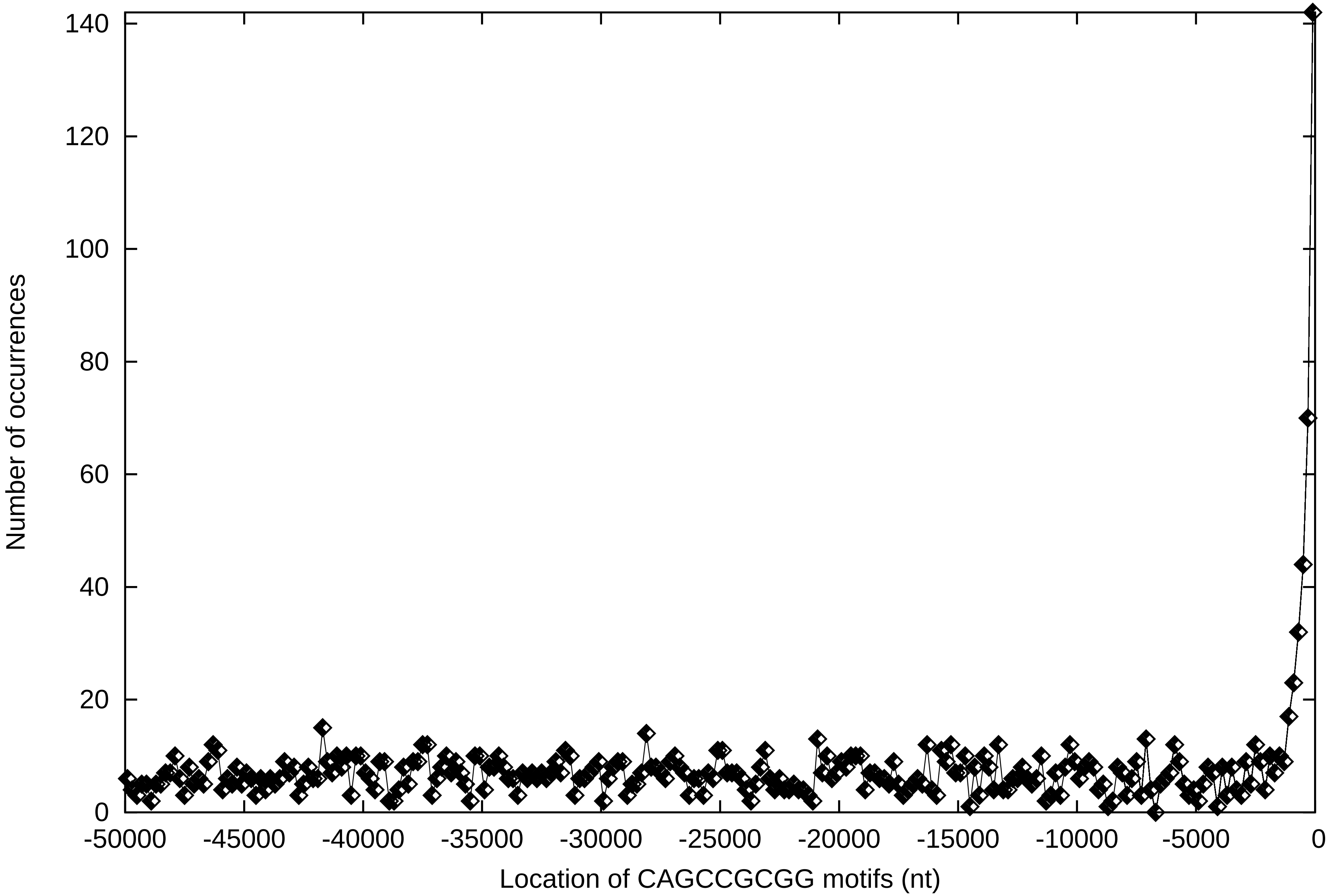

140
120
100
80
60
40
20
0
-50000
-45000
-40000
-35000
-30000
-25000
-20000
-15000
-10000
-5000
0
Number of occurrences
Location of CAGCCGCGG motifs (nt)

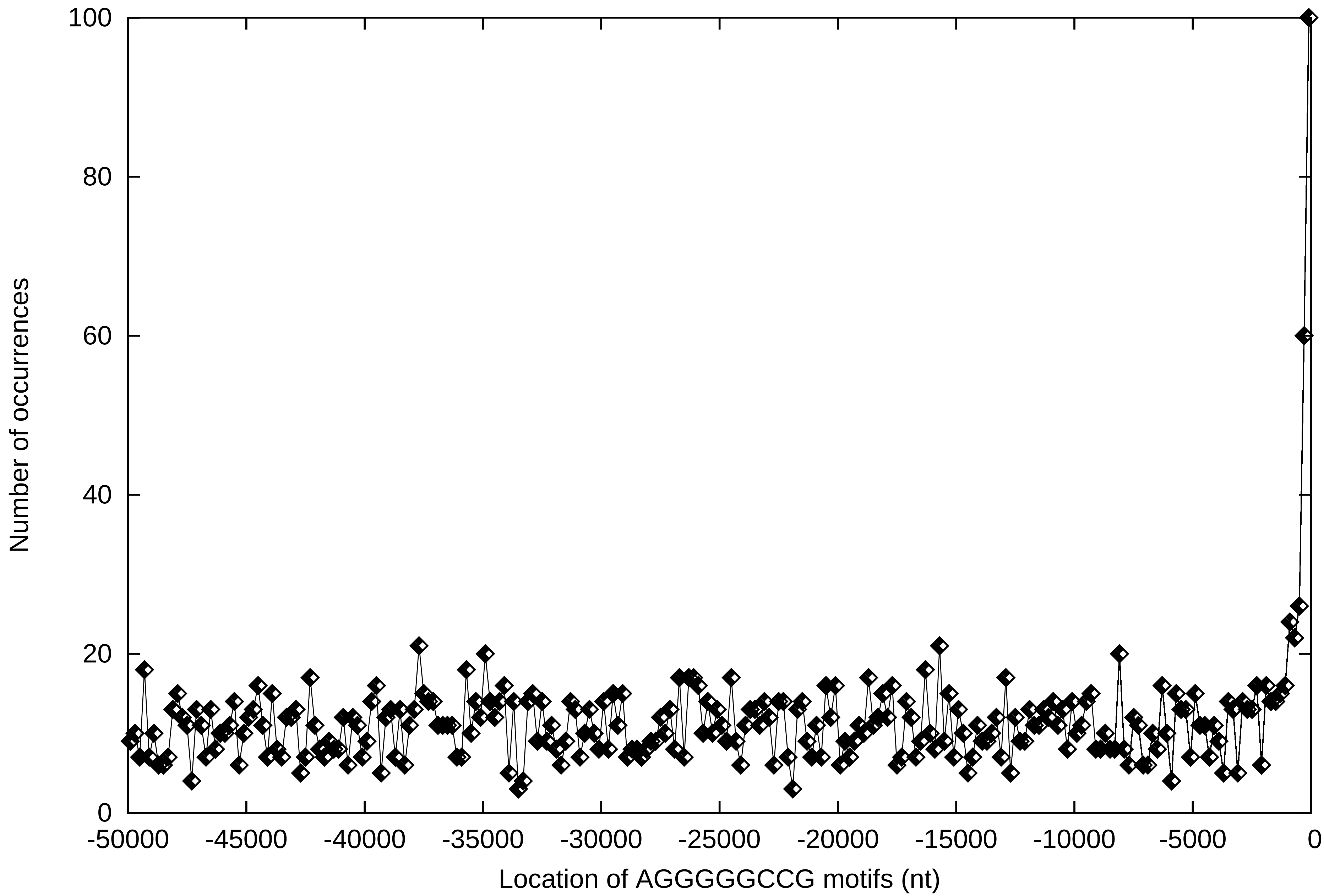
100
80
60
40
20
0
-50000
-45000
-40000
-35000
-30000
-25000
-20000
-15000
-10000
-5000
0
Number of occurrences
Location of AGGGGGCCG motifs (nt)

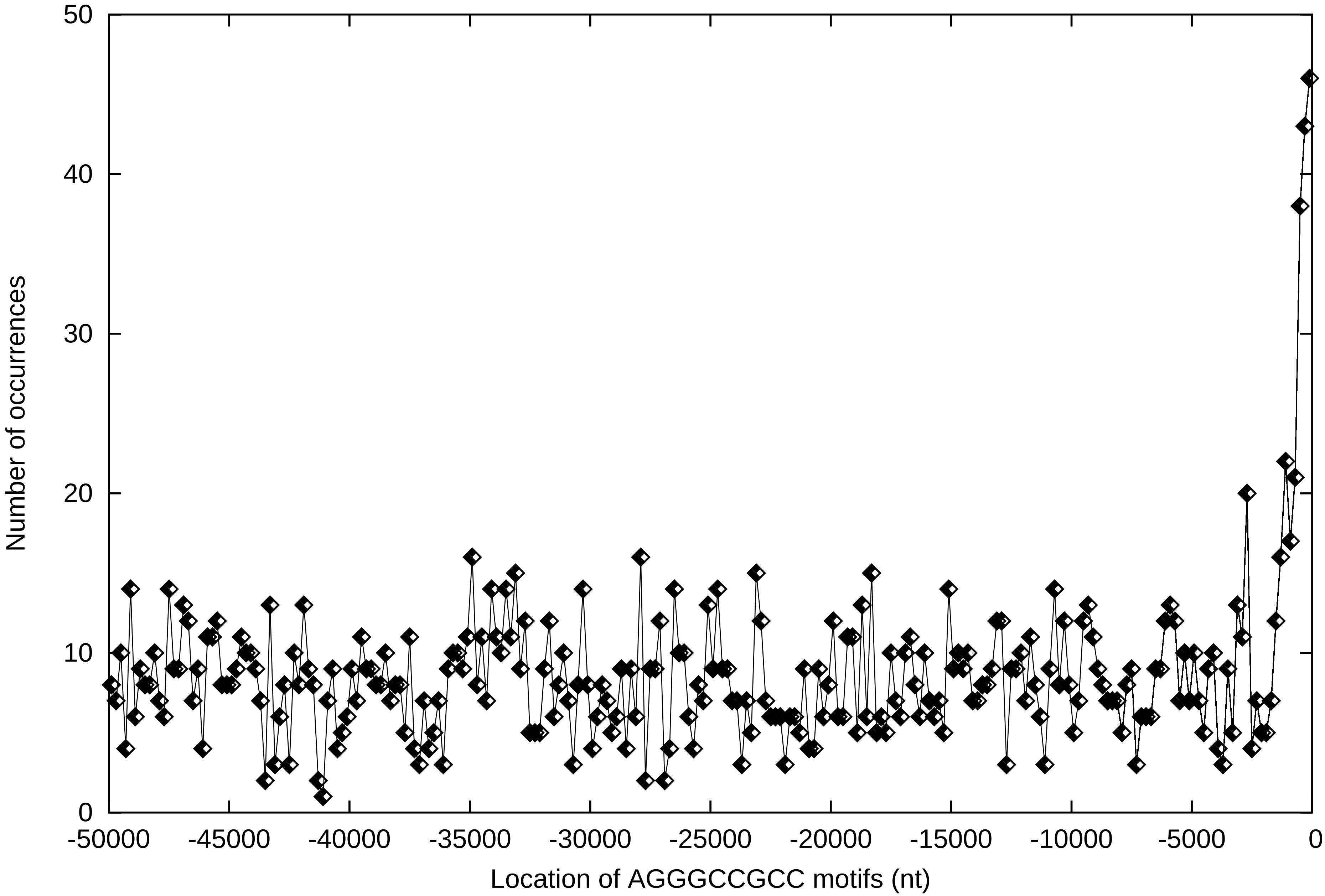

50
40
30
20
10
0
Number of occurrences
-50000
-45000
-40000
-35000
-30000
-25000
-20000
-15000
-10000
-5000
0
Location of AGGGCCGCC motifs (nt)

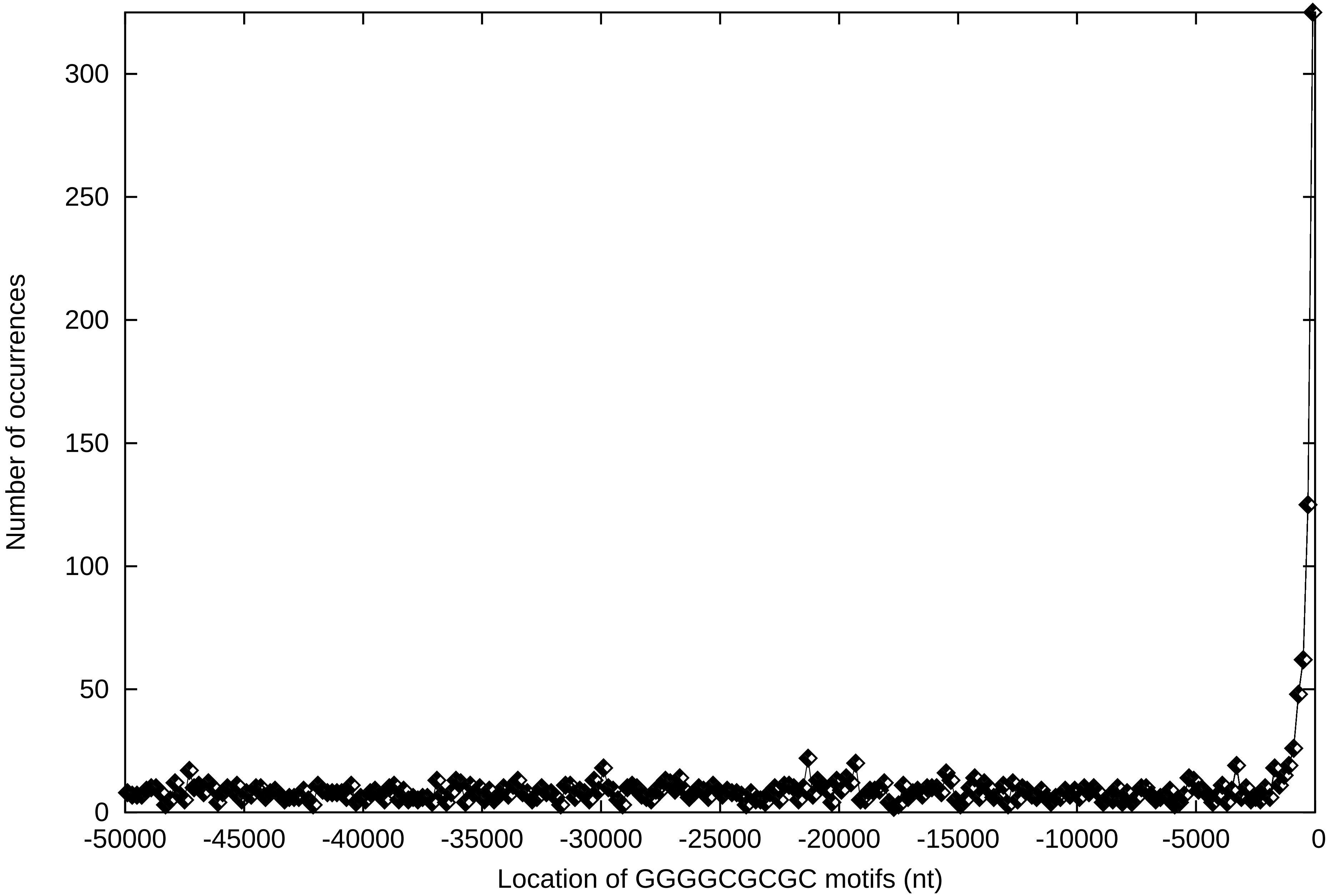

Number of occurrences
300
250
200
150
100
50
0
-50000
-45000
-40000
-35000
-30000
-25000
-20000
-15000
-10000
-5000
0
Location of GGGGCGCGC motifs (nt)

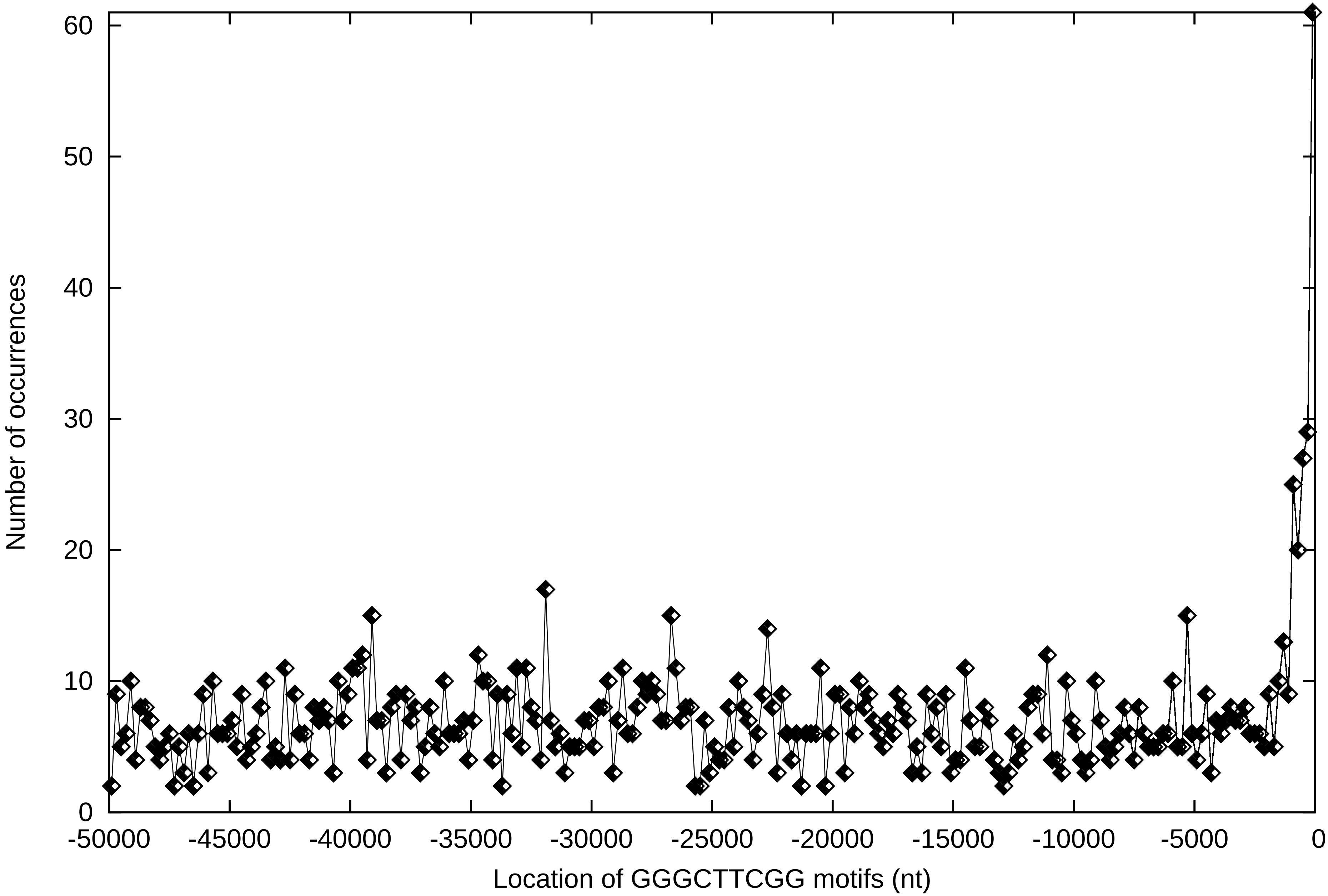

0
10
20
30
40
50
60
-50000
-45000
-40000
-35000
-30000
-25000
-20000
-15000
-10000
-5000
0
Number of occurrences
Location of GGGCTTCGG motifs (nt)

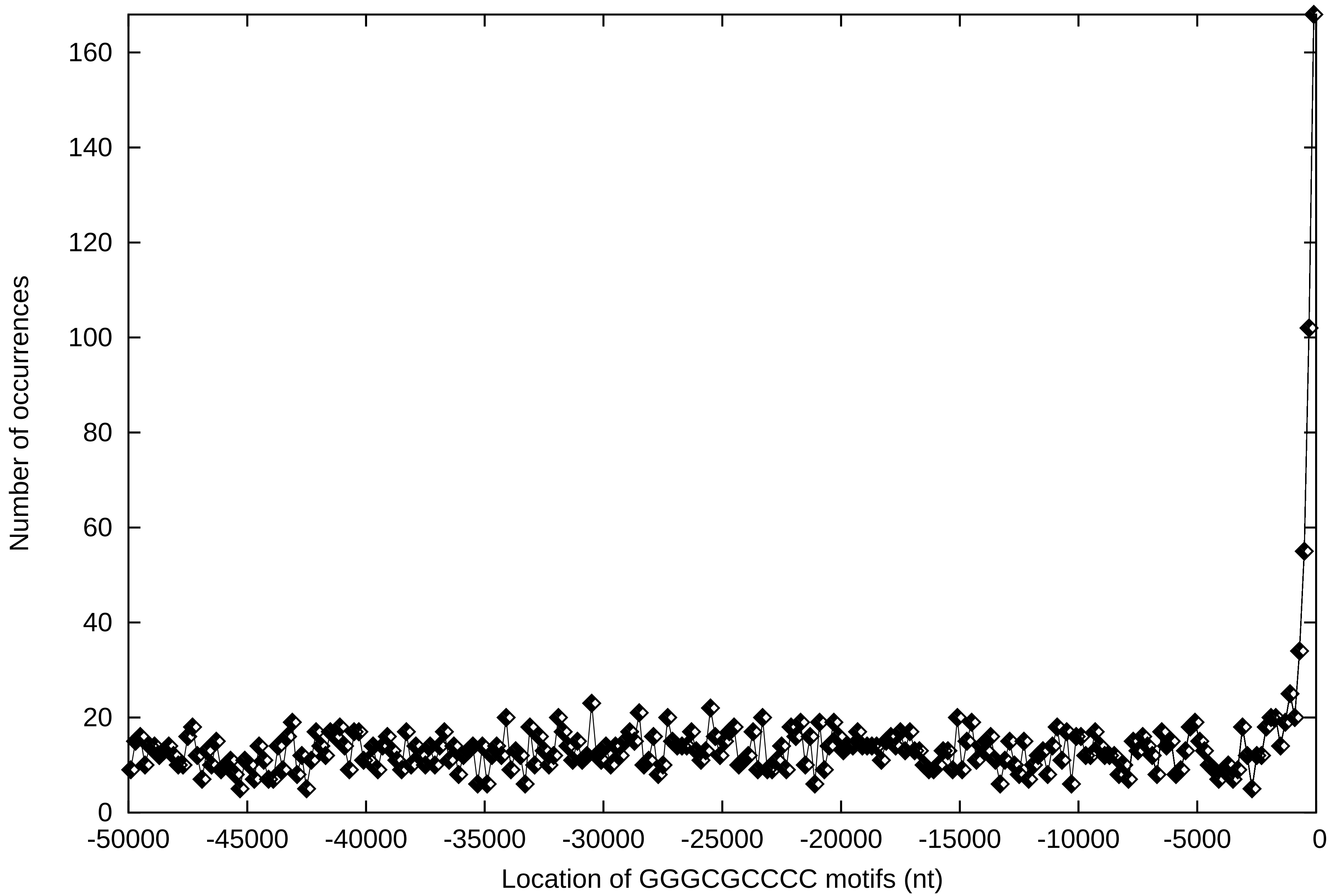

Number of occurrences
0
20
40
60
80
100
120
140
160
-50000
-45000
-40000
-35000
-30000
-25000
-20000
-15000
-10000
-5000
0
Location of GGGCGCCCC motifs (nt)

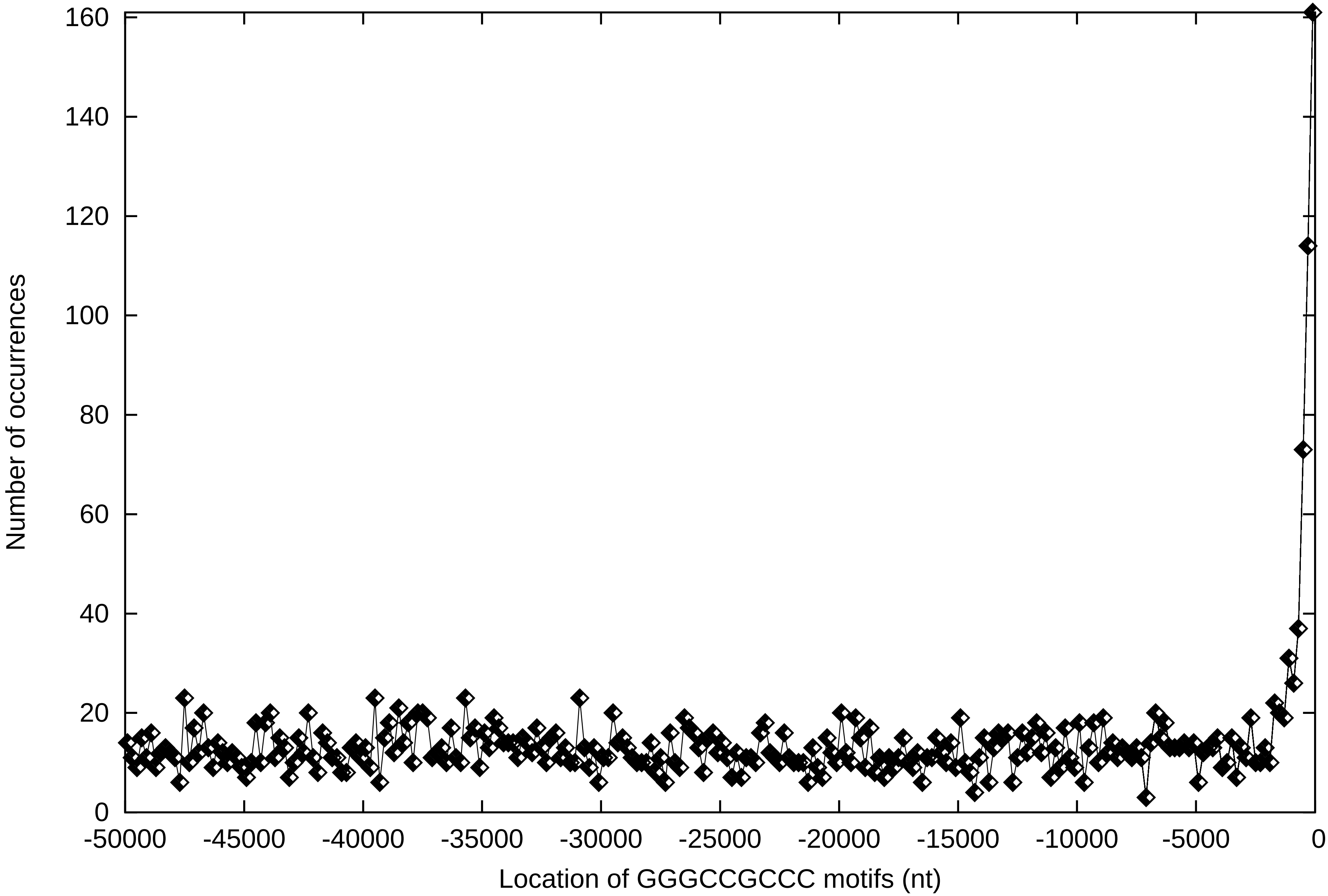

160
140
120
100
80
60
40
20
0
Number of occurrences
-50000
-45000
-40000
-35000
-30000
-25000
-20000
-15000
-10000
-5000
0
Location of GGGCCGCCC motifs (nt)

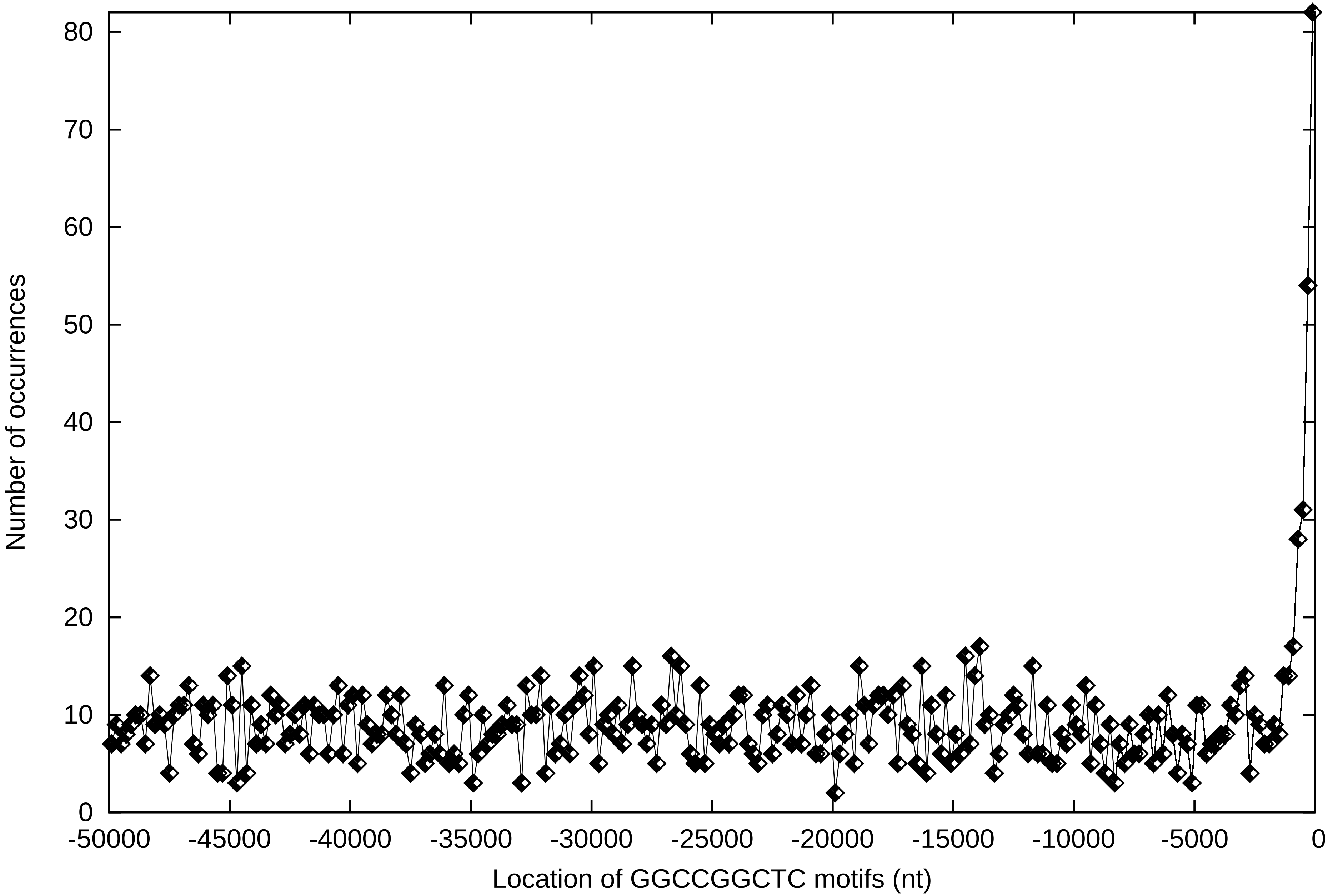

Number of occurrences
Location of GGCCGGCTC motifs (nt)
0
10
20
30
40
50
60
70
80
-50000
-45000
-40000
-35000
-30000
-25000
-20000
-15000
-10000
-5000
0

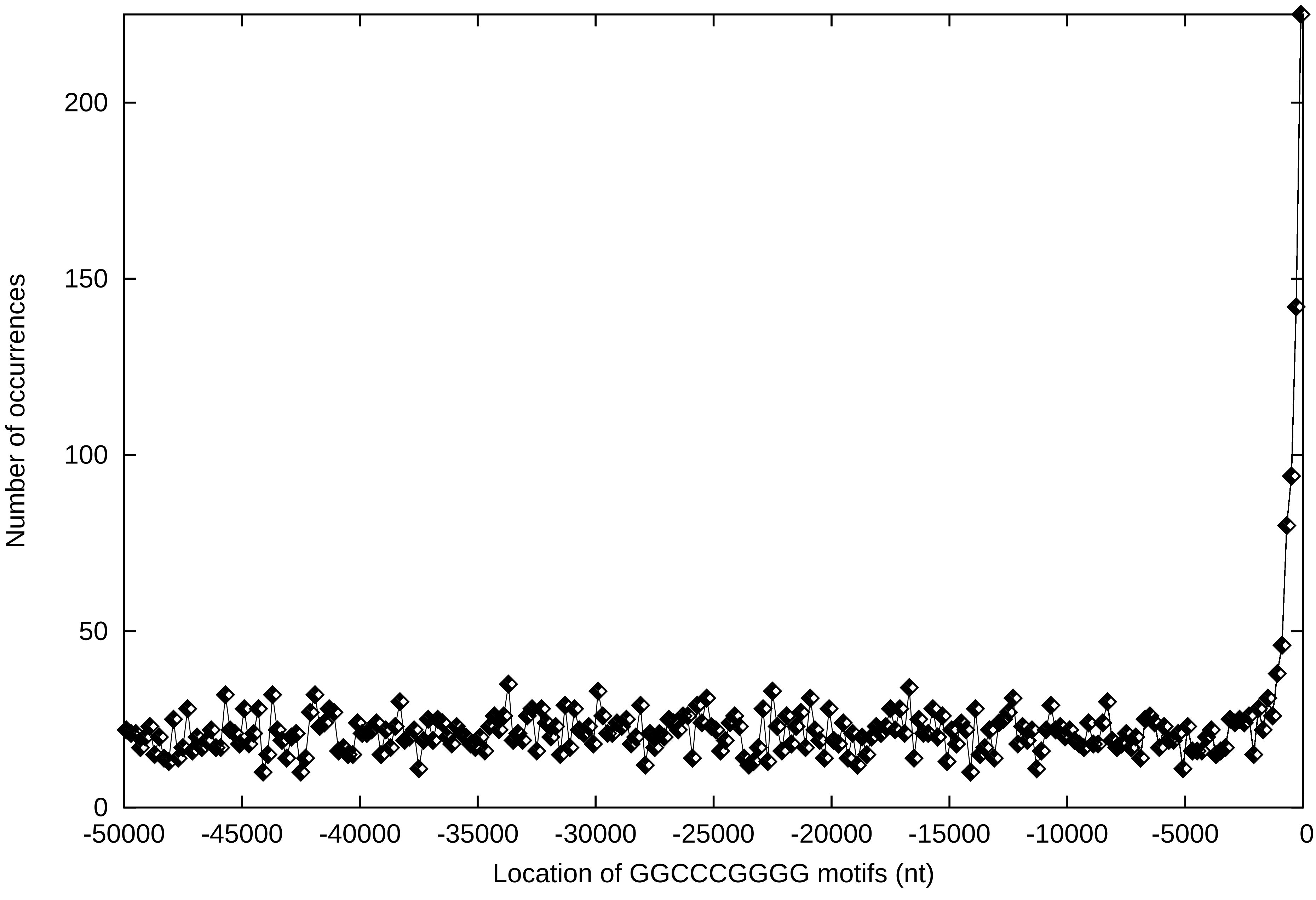

Number of occurrences
0
50
100
150
200
-50000
-45000
-40000
-35000
-30000
-25000
-20000
-15000
-10000
-5000
0
Location of GGCCCGGGG motifs (nt)

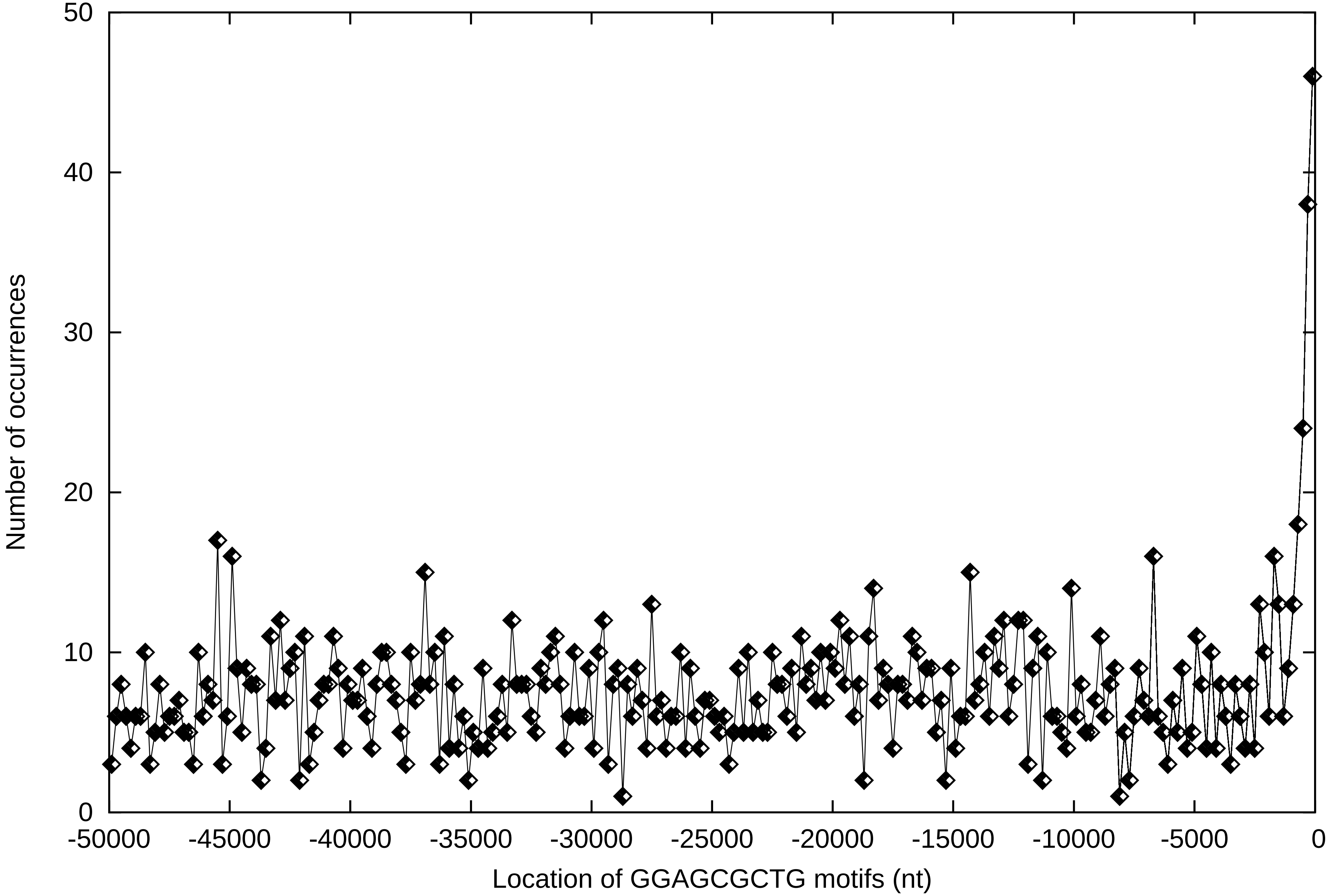

50
40
30
20
10
0
-50000
-45000
-40000
-35000
-30000
-25000
-20000
-15000
-10000
-5000
0
Number of occurrences
Location of GGAGCGCTG motifs (nt)

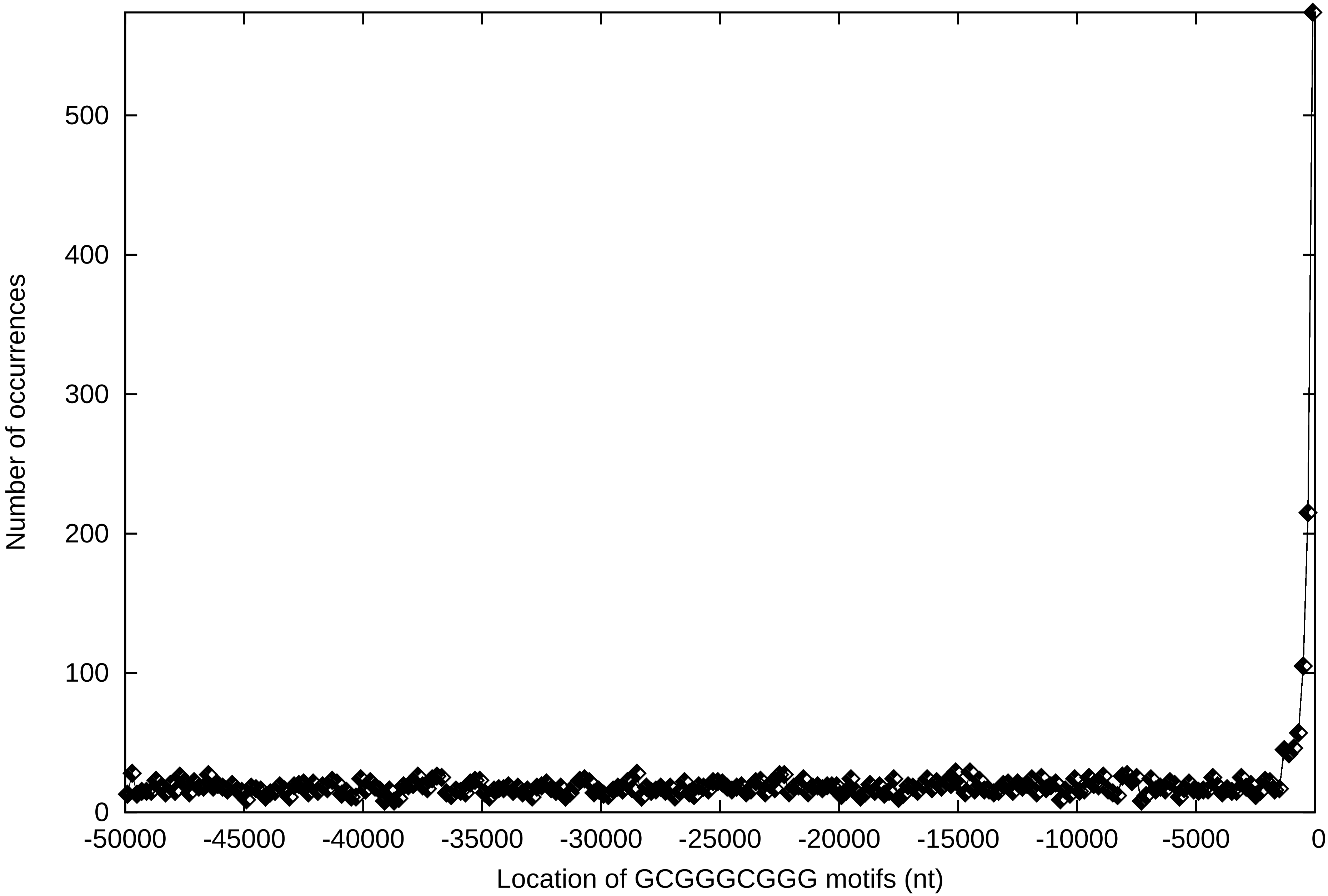

Number of occurrences
0
100
200
300
400
500
-50000
-45000
-40000
-35000
-30000
-25000
-20000
-15000
-10000
-5000
0
Location of GCGGGCGGG motifs (nt)

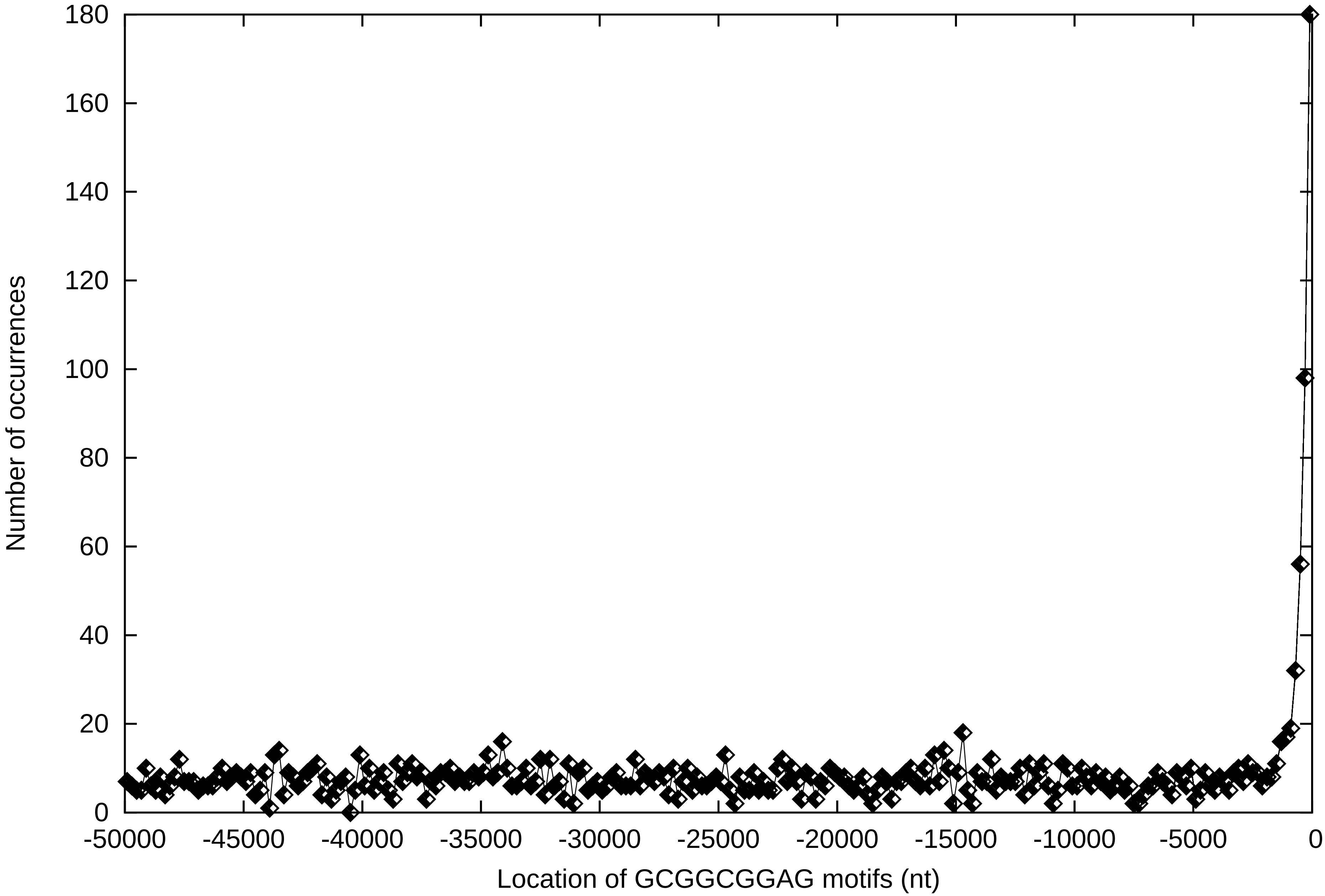

180
160
140
120
100
80
60
40
20
0
-50000
-45000
-40000
-35000
-30000
-25000
-20000
-15000
-10000
-5000
0
Number of occurrences
Location of GCGGCGGAG motifs (nt)

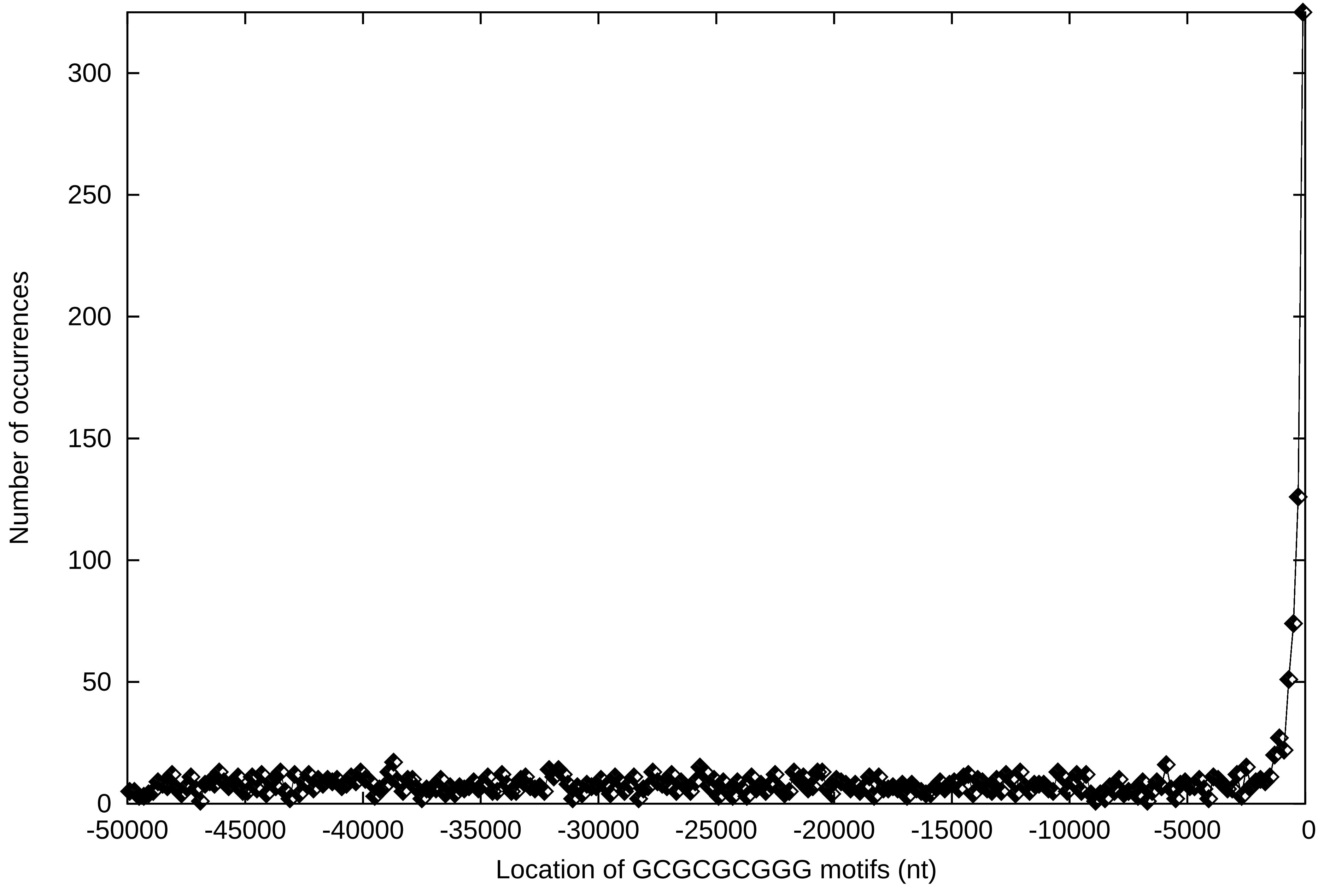

Number of occurrences
0
50
100
150
200
250
300
-50000
-45000
-40000
-35000
-30000
-25000
-20000
-15000
-10000
-5000
0
Location of GCGCGCGGG motifs (nt)

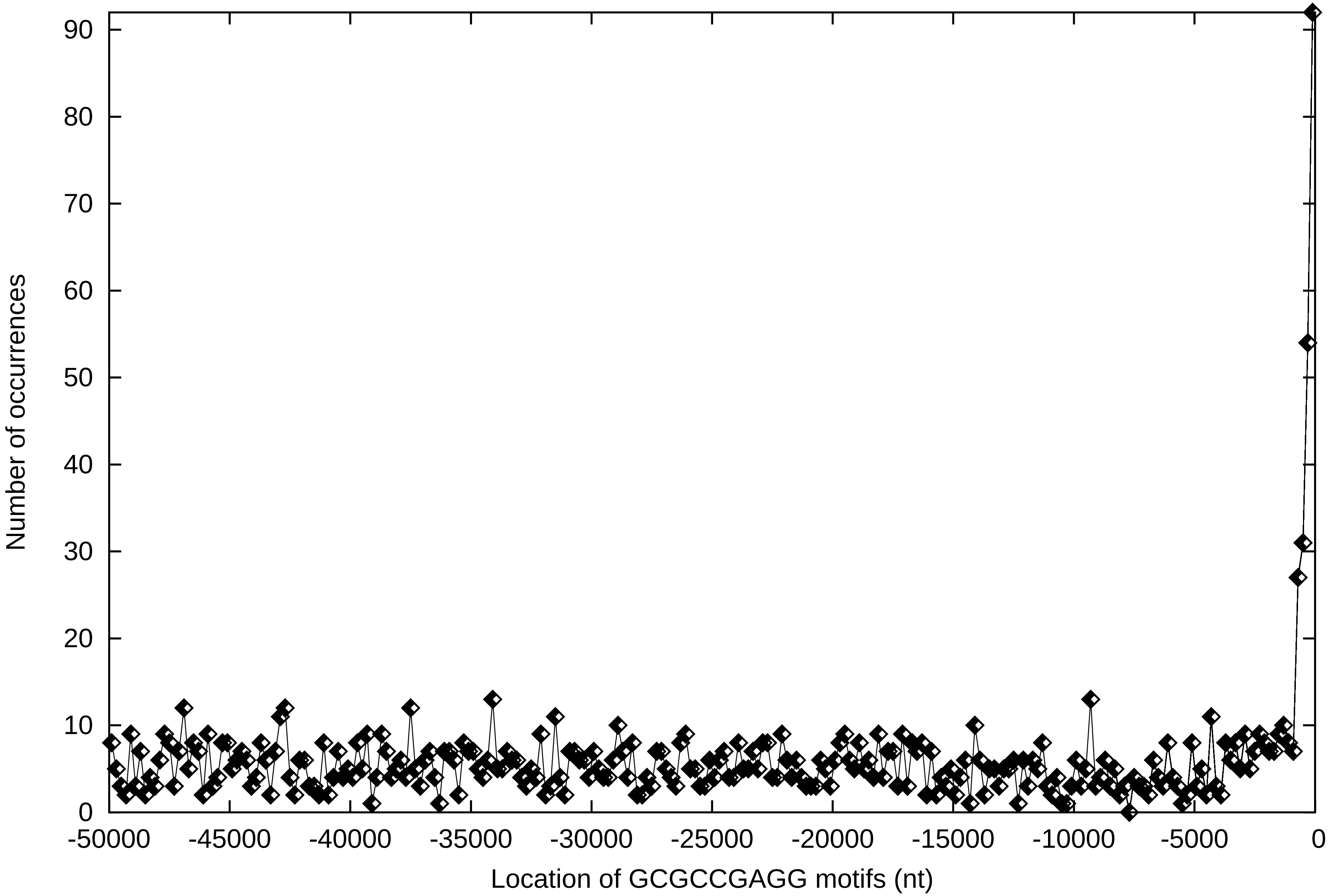

Number of occurrences
Location of GCGCCGAGG motifs (nt)
0
10
20
30
40
50
60
70
80
90
-50000
-45000
-40000
-35000
-30000
-25000
-20000
-15000
-10000
-5000
0

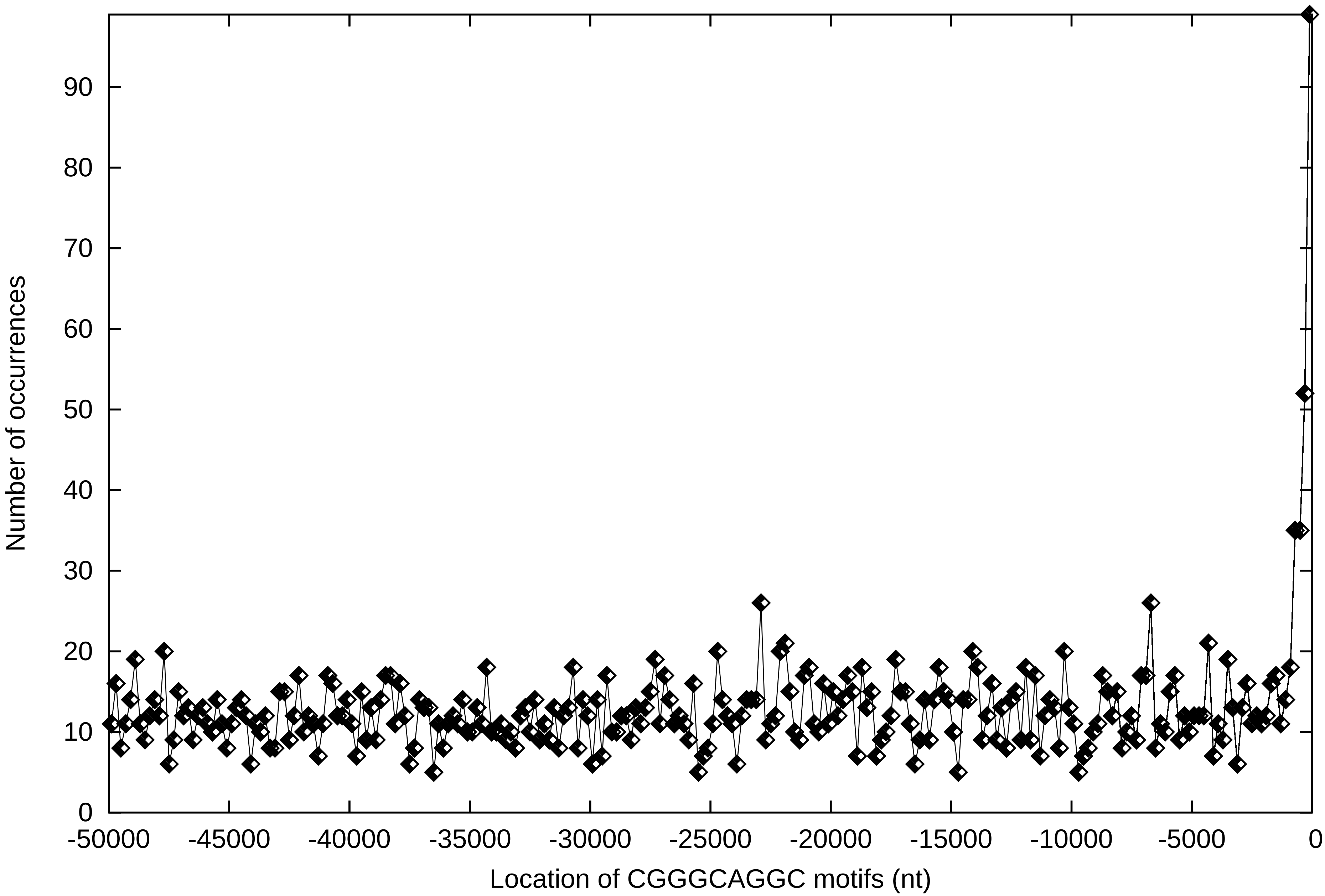

Number of occurrences
0
10
20
30
40
50
60
70
80
90
-50000
-45000
-40000
-35000
-30000
-25000
-20000
-15000
-10000
-5000
0
Location of CGGGCAGGC motifs (nt)

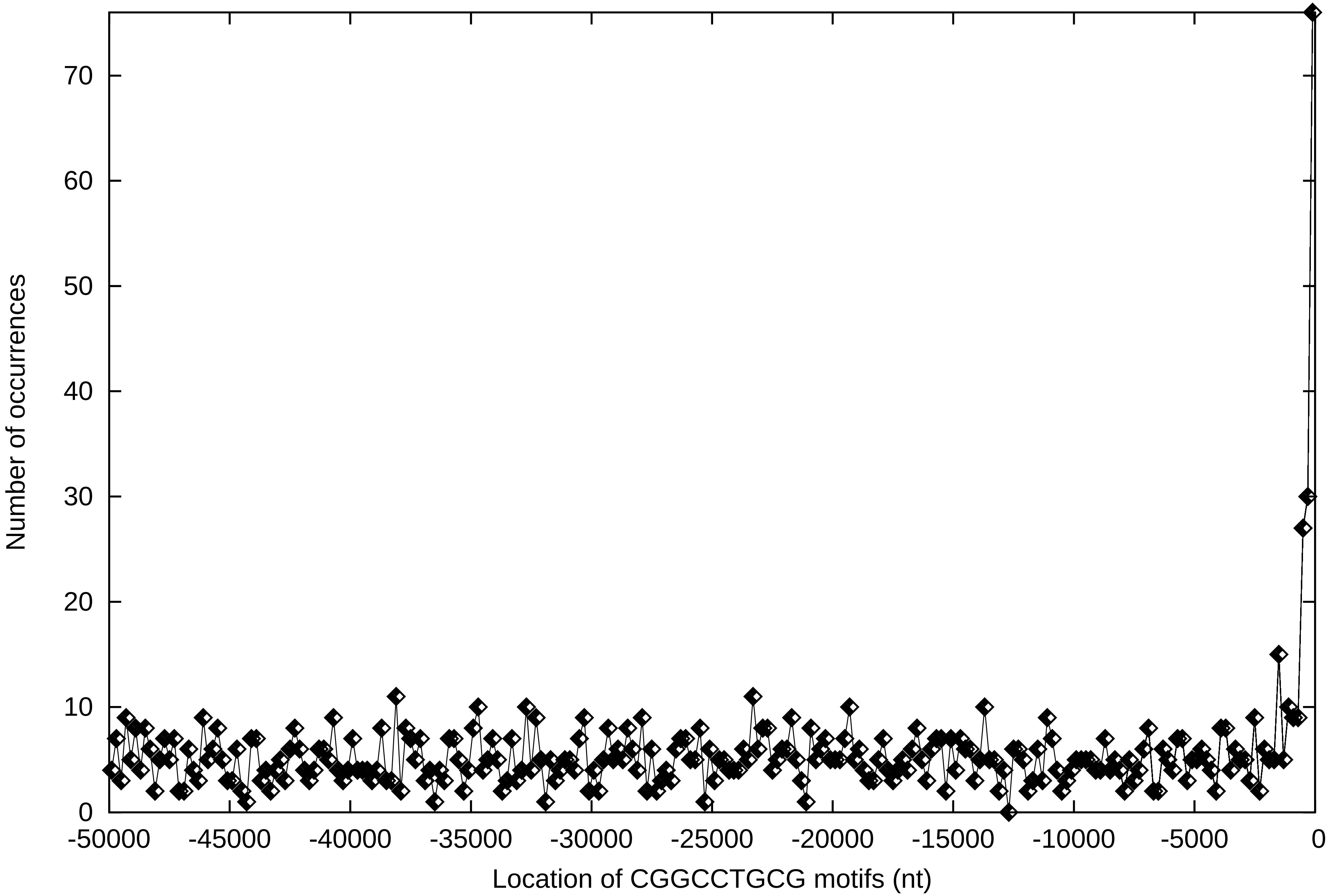

Number of occurrences
0
10
20
30
40
50
60
70
-50000
-45000
-40000
-35000
-30000
-25000
-20000
-15000
-10000
-5000
0
Location of CGGCCTGCG motifs (nt)

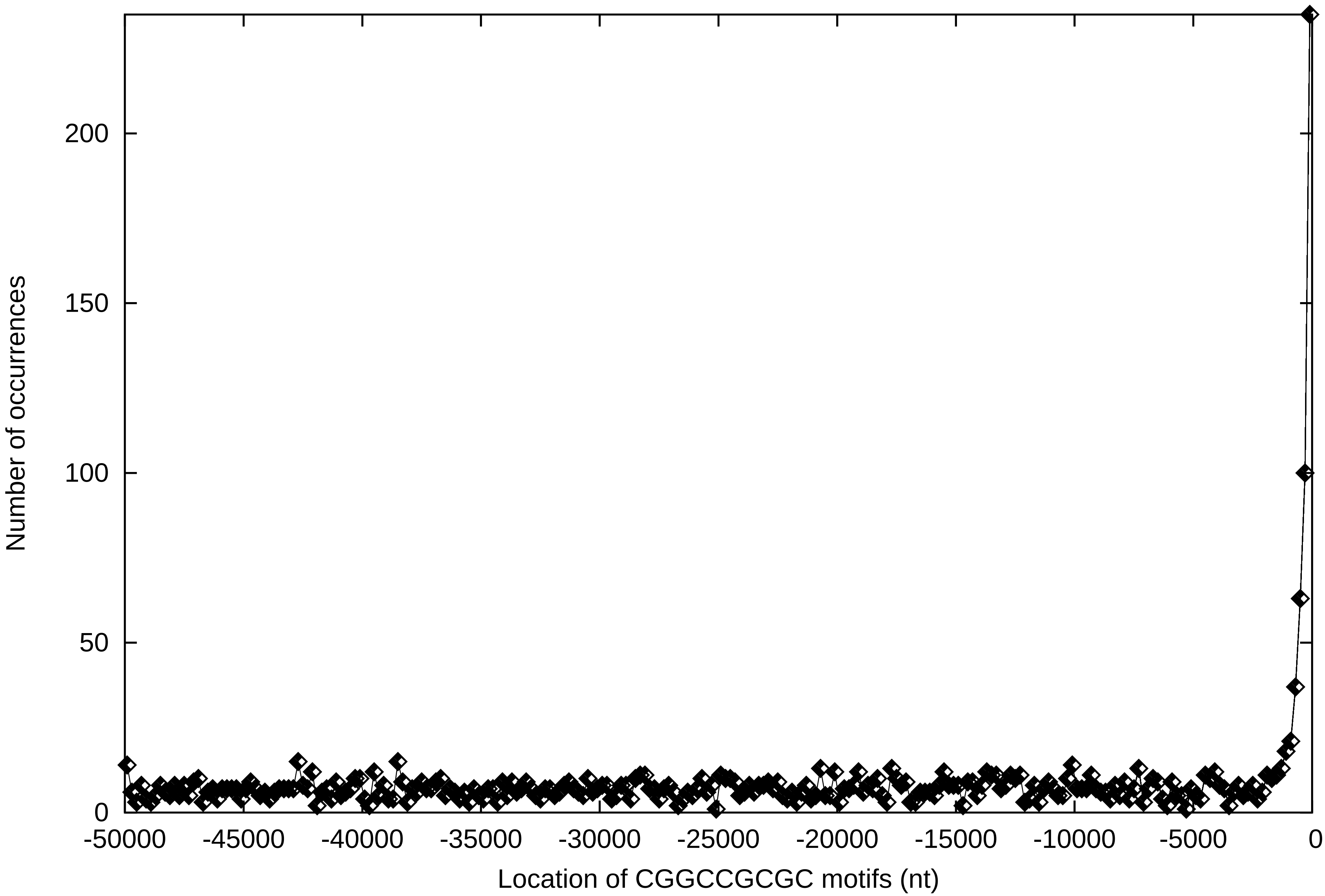

Number of occurrences
Location of CGGCCGCGC motifs (nt)
0
50
100
150
200
-50000
-45000
-40000
-35000
-30000
-25000
-20000
-15000
-10000
-5000
0

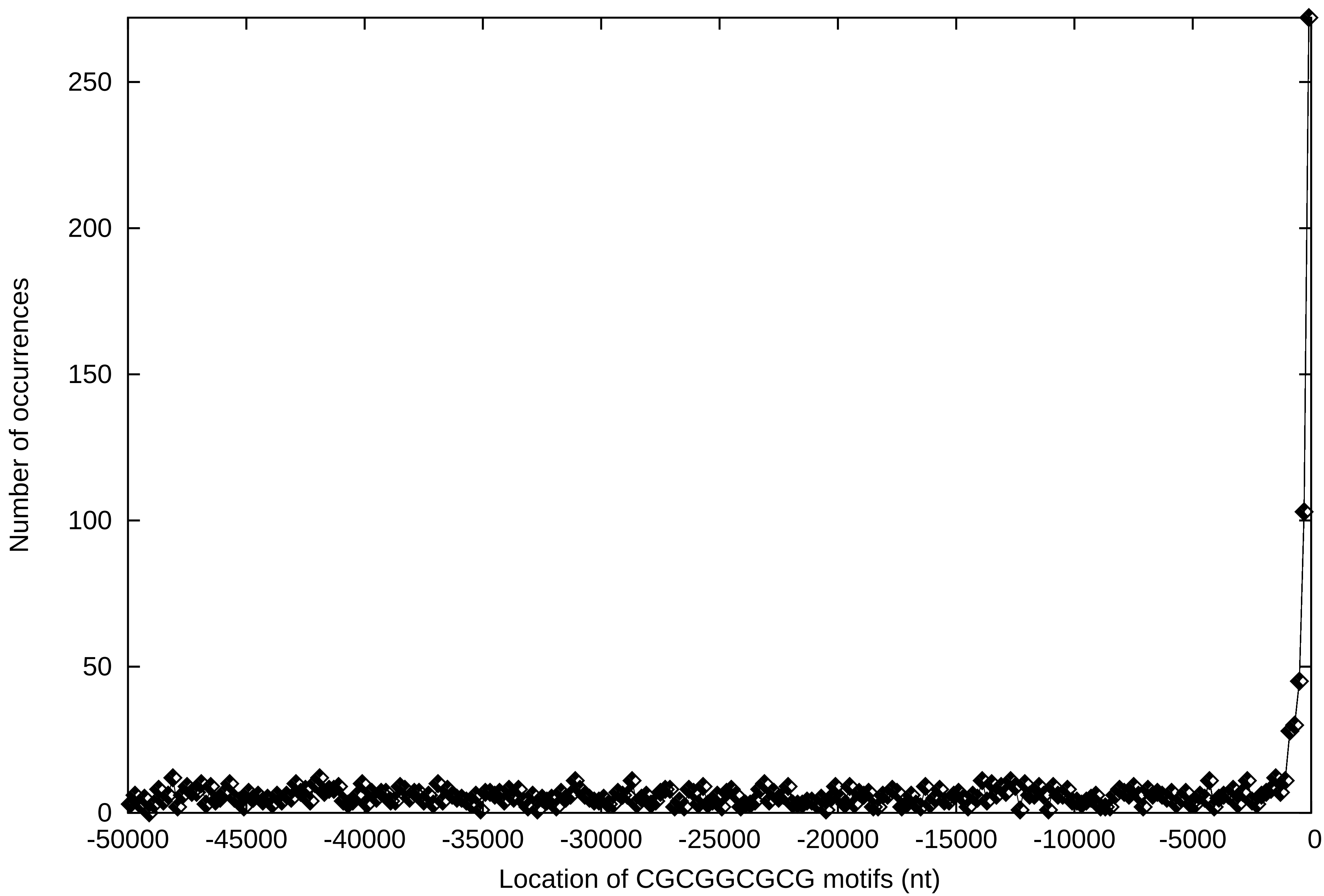

Number of occurrences
250
200
150
100
50
0
-50000
-45000
-40000
-35000
-30000
-25000
-20000
-15000
-10000
-5000
0
Location of CGCGGCGCG motifs (nt)

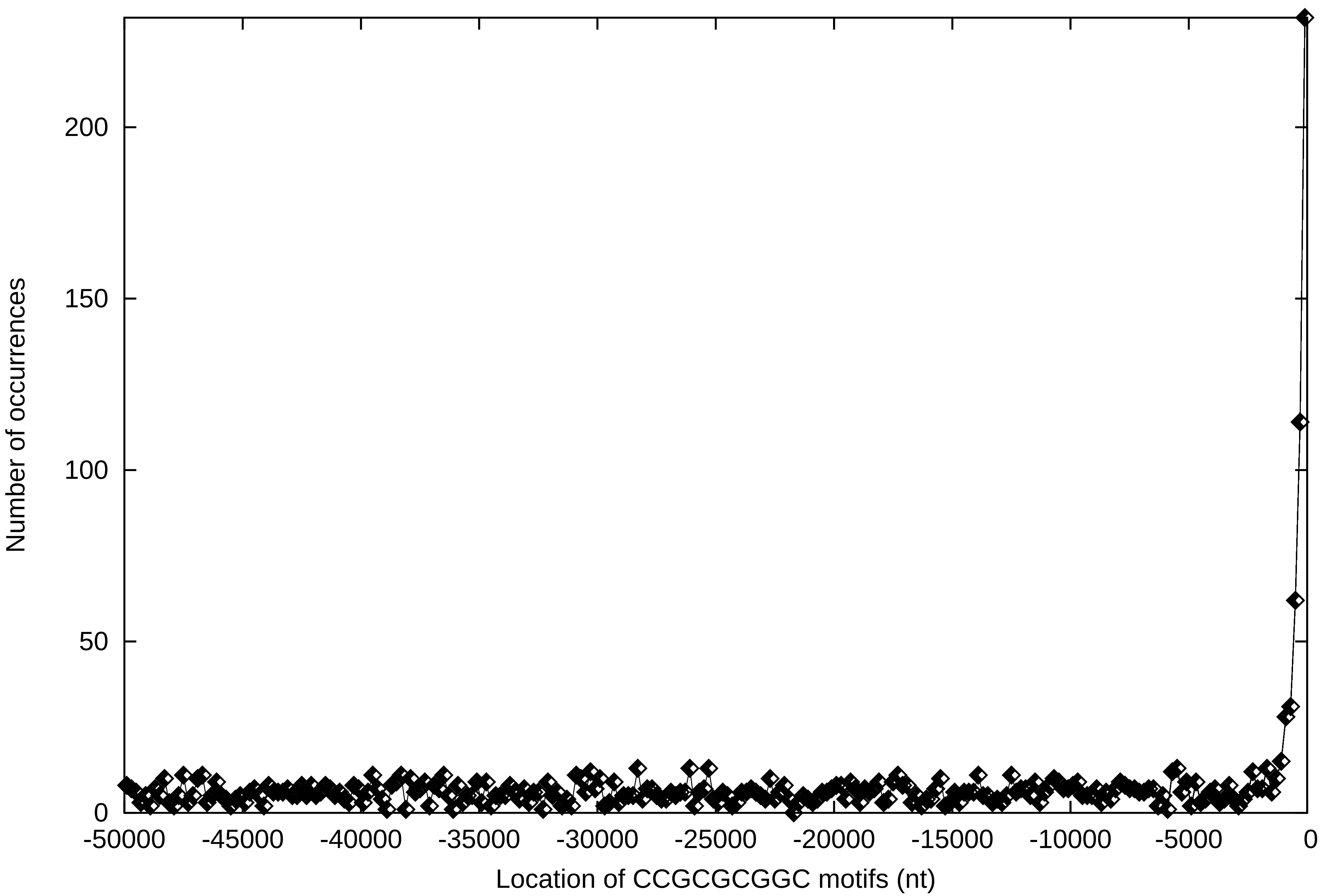

Number of occurrences
Location of CCGCGCGGC motifs (nt)
0
50
100
150
200
-50000
-45000
-40000
-35000
-30000
-25000
-20000
-15000
-10000
-5000
0

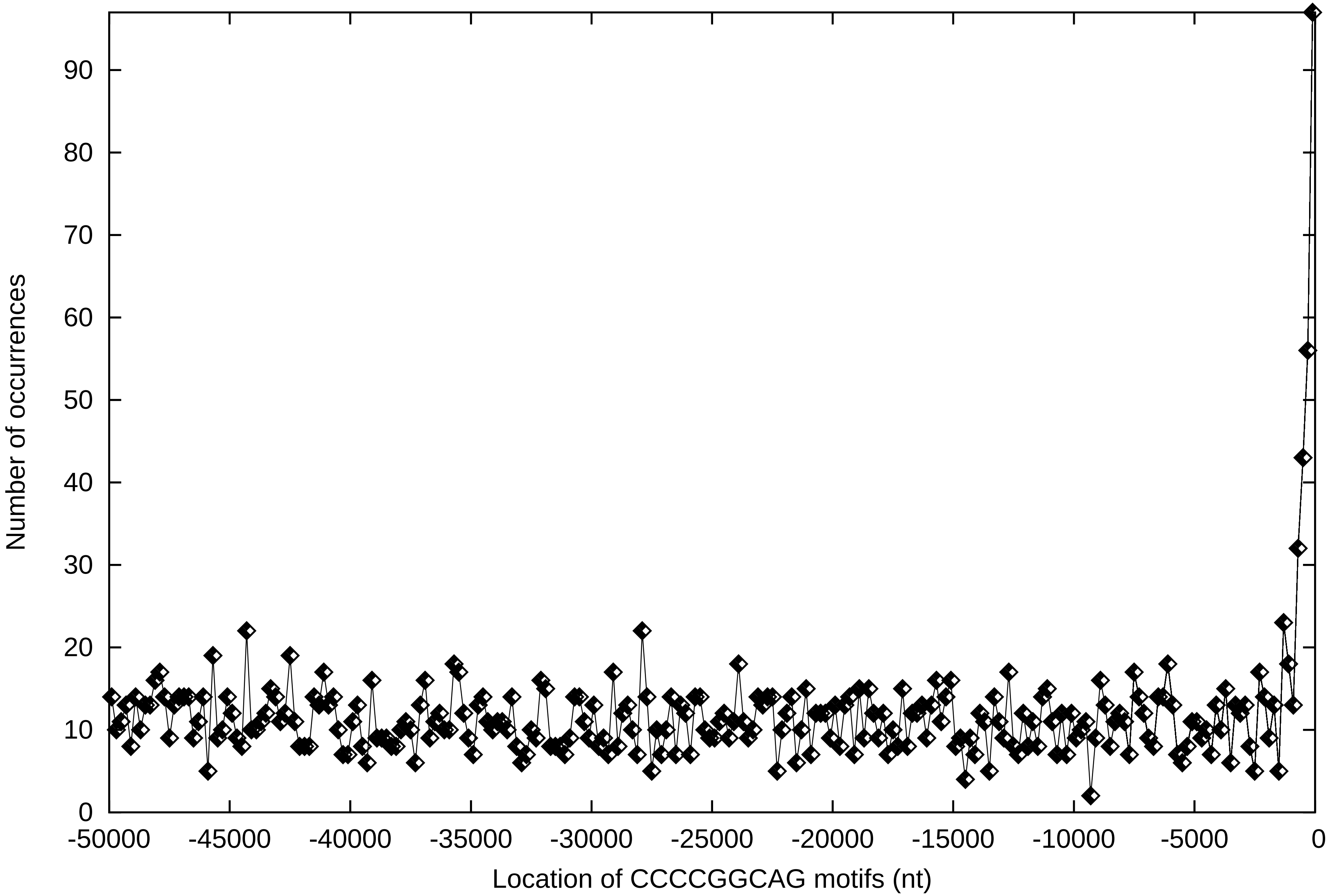

Number of occurrences
0
10
20
30
40
50
60
70
80
90
-50000
-45000
-40000
-35000
-30000
-25000
-20000
-15000
-10000
-5000
0
Location of CCCCGGCAG motifs (nt)

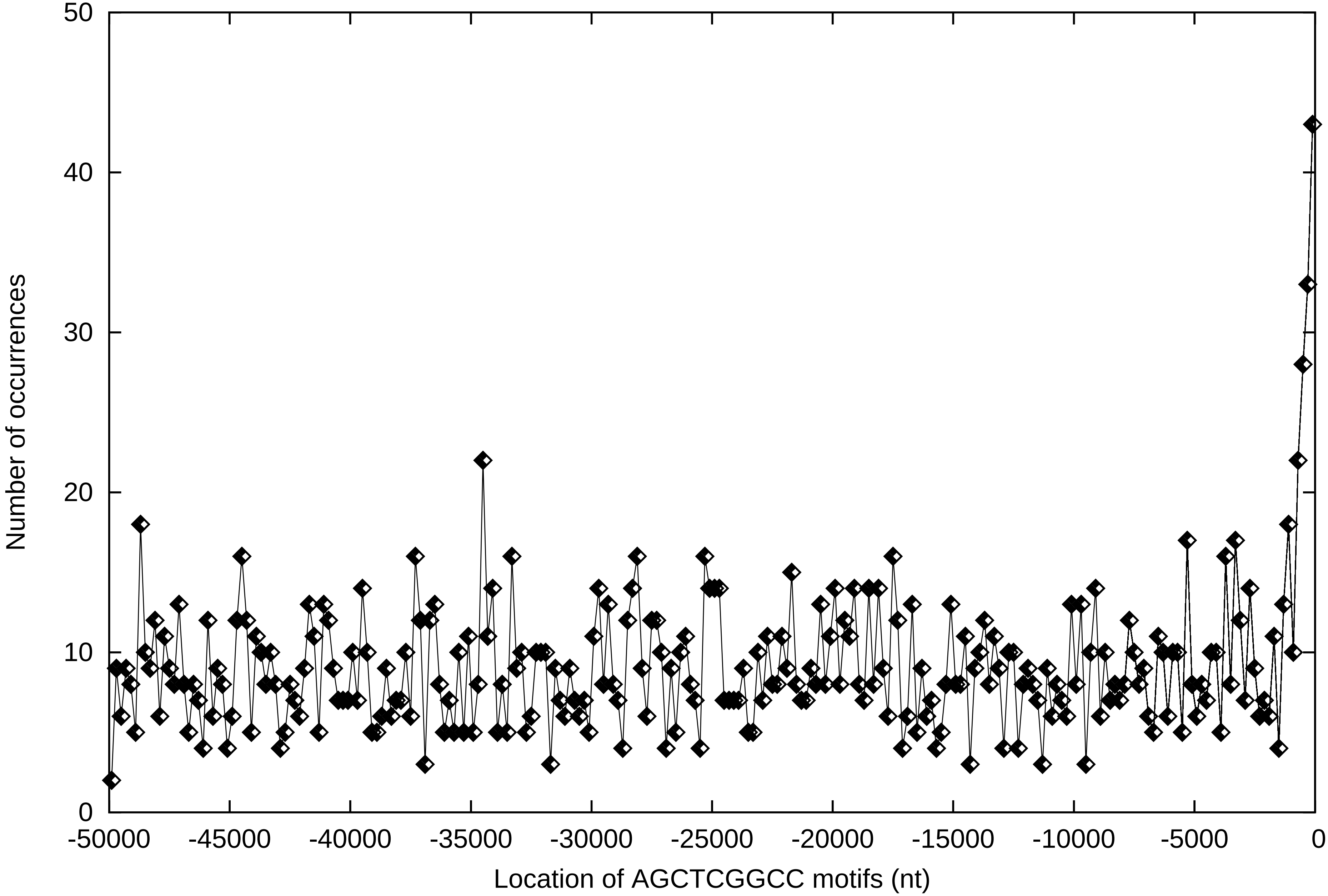

Number of occurrences
0
10
20
30
40
50
-50000
-45000
-40000
-35000
-30000
-25000
-20000
-15000
-10000
-5000
0
Location of AGCTCGGCC motifs (nt)

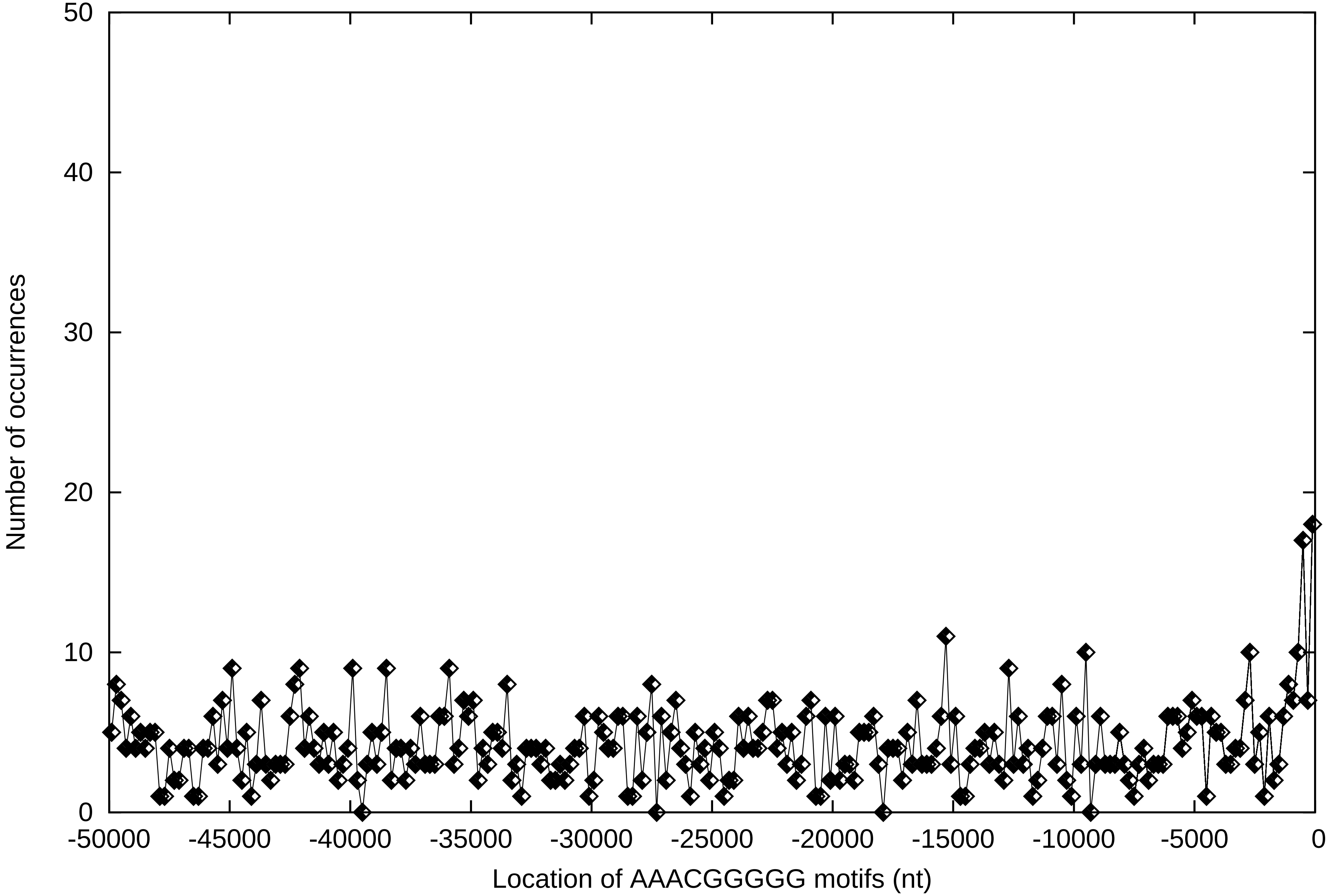

Number of occurrences
Location of AAACGGGGG motifs (nt)
0
10
20
30
40
50
-50000
-45000
-40000
-35000
-30000
-25000
-20000
-15000
-10000
-5000
0

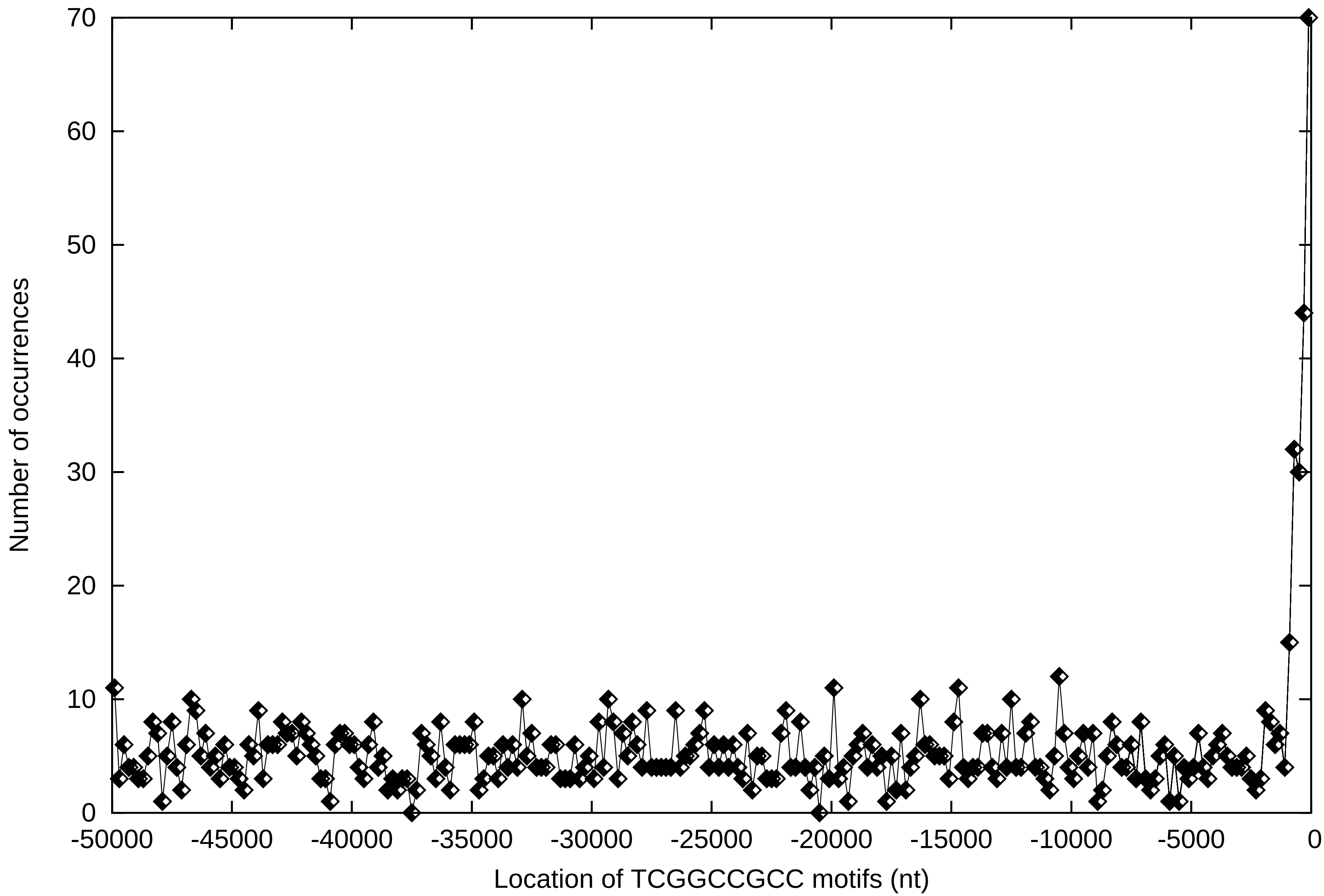

Number of occurrences
70
60
50
40
30
20
10
0
-50000
-45000
-40000
-35000
-30000
-25000
-20000
-15000
-10000
-5000
0
Location of TCGGCCGCC motifs (nt)

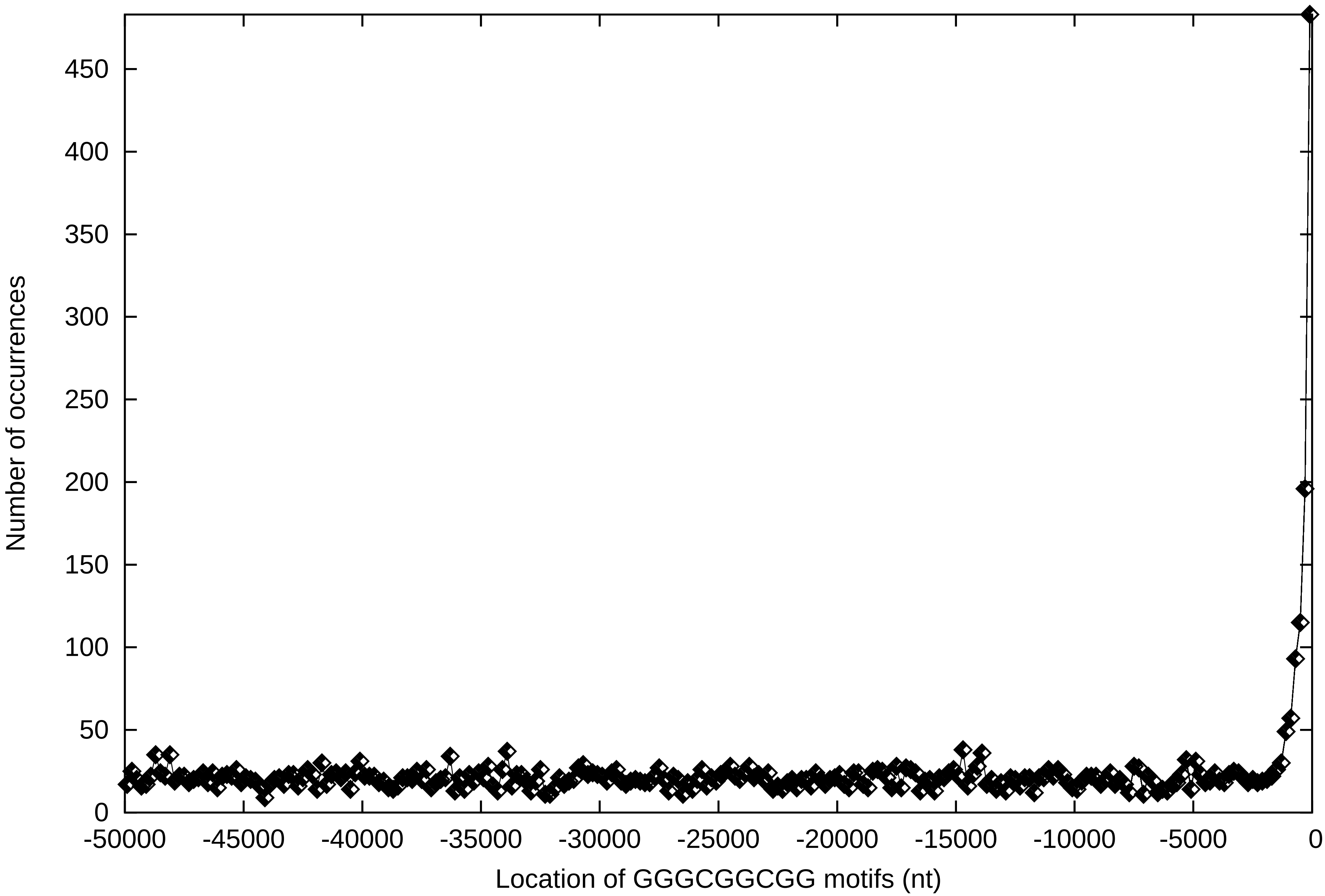

Number of occurrences
0
50
100
150
200
250
300
350
400
450
-50000
-45000
-40000
-35000
-30000
-25000
-20000
-15000
-10000
-5000
0
Location of GGGCGGCGG motifs (nt)

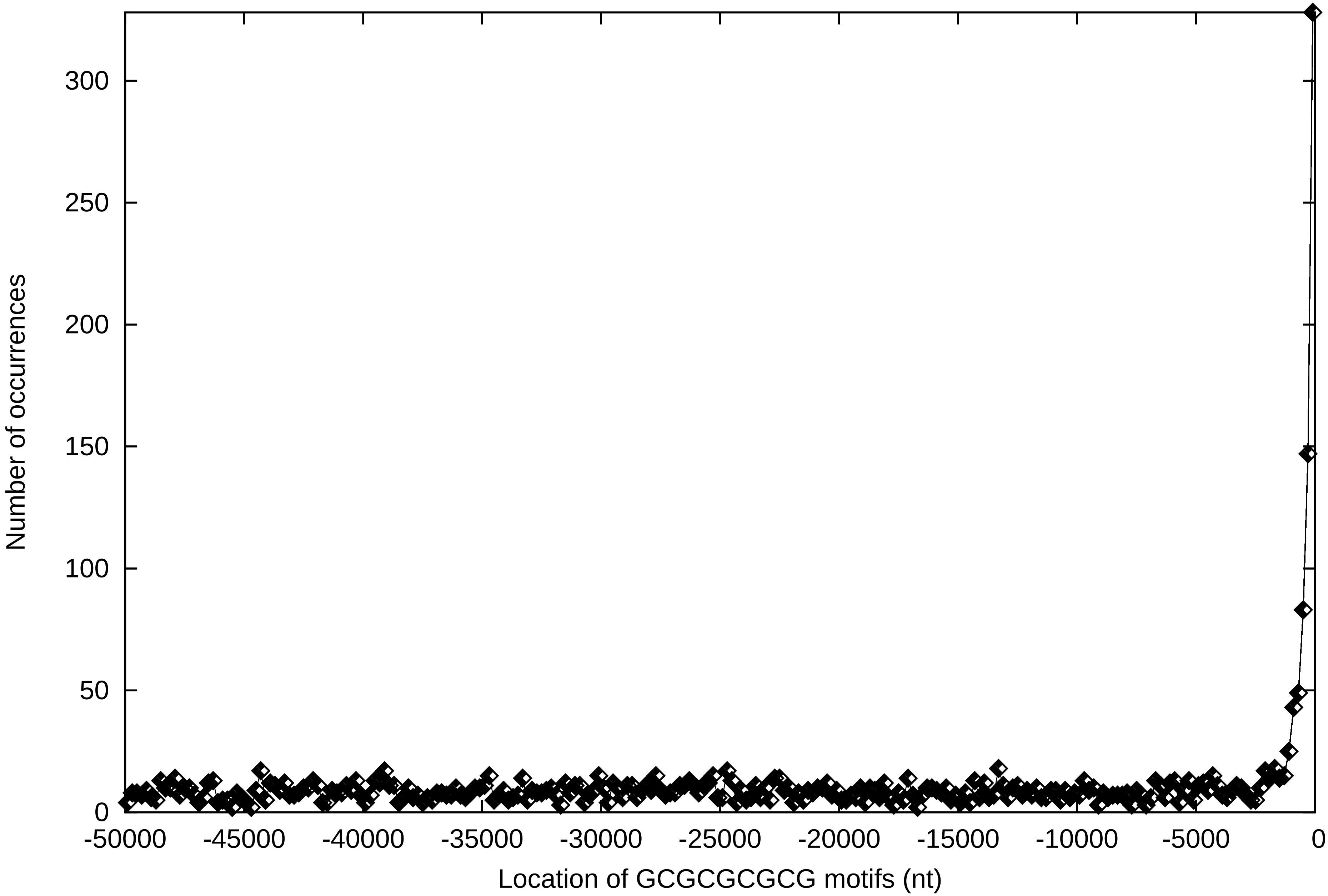

Number of occurrences
0
50
100
150
200
250
300
-50000
-45000
-40000
-35000
-30000
-25000
-20000
-15000
-10000
-5000
0
Location of GCGCGCGCG motifs (nt)

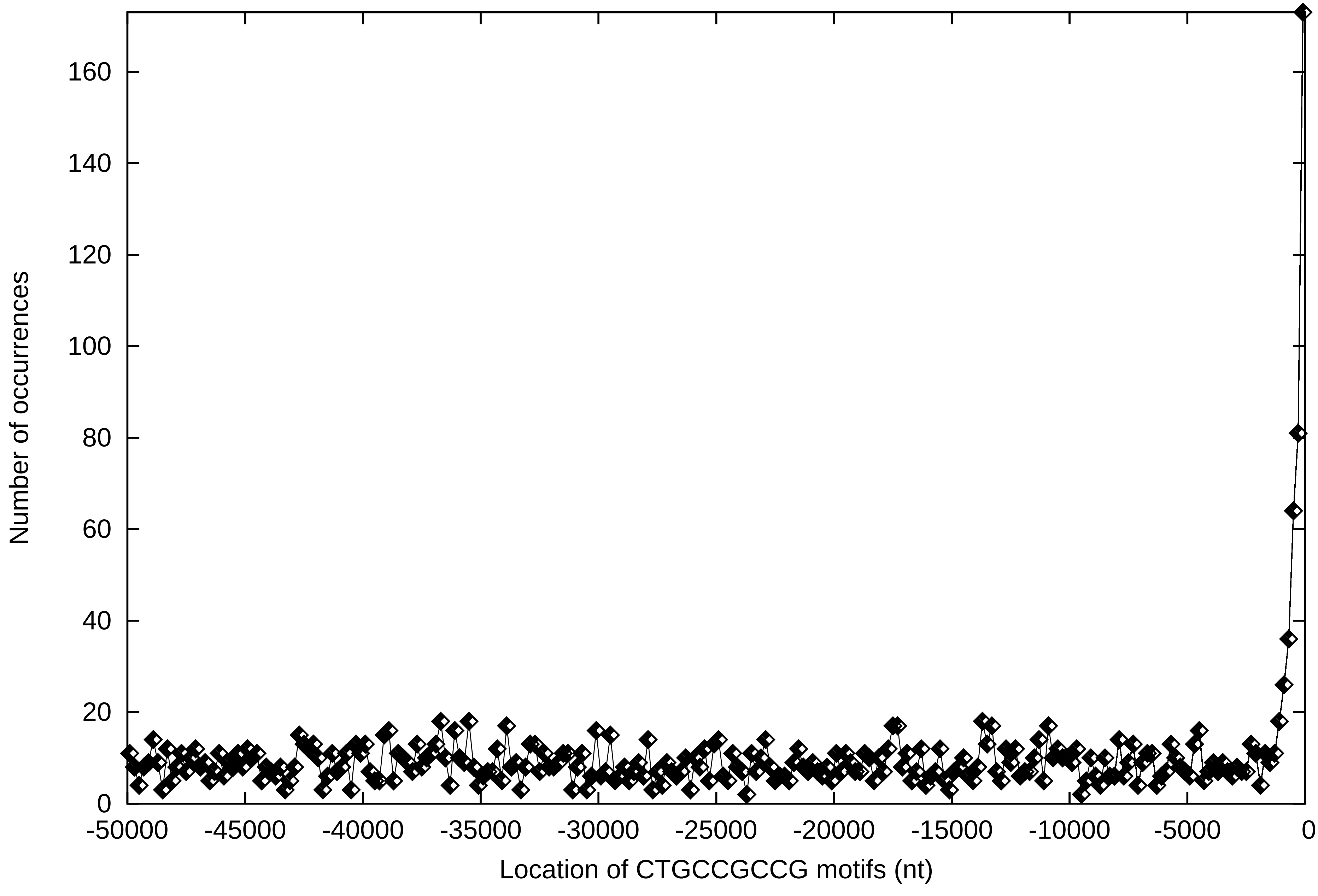
Number of occurrences
0
20
40
60
80
100
120
140
160
-50000
-45000
-40000
-35000
-30000
-25000
-20000
-15000
-10000
-5000
0
Location of CTGCCGCCG motifs (nt)

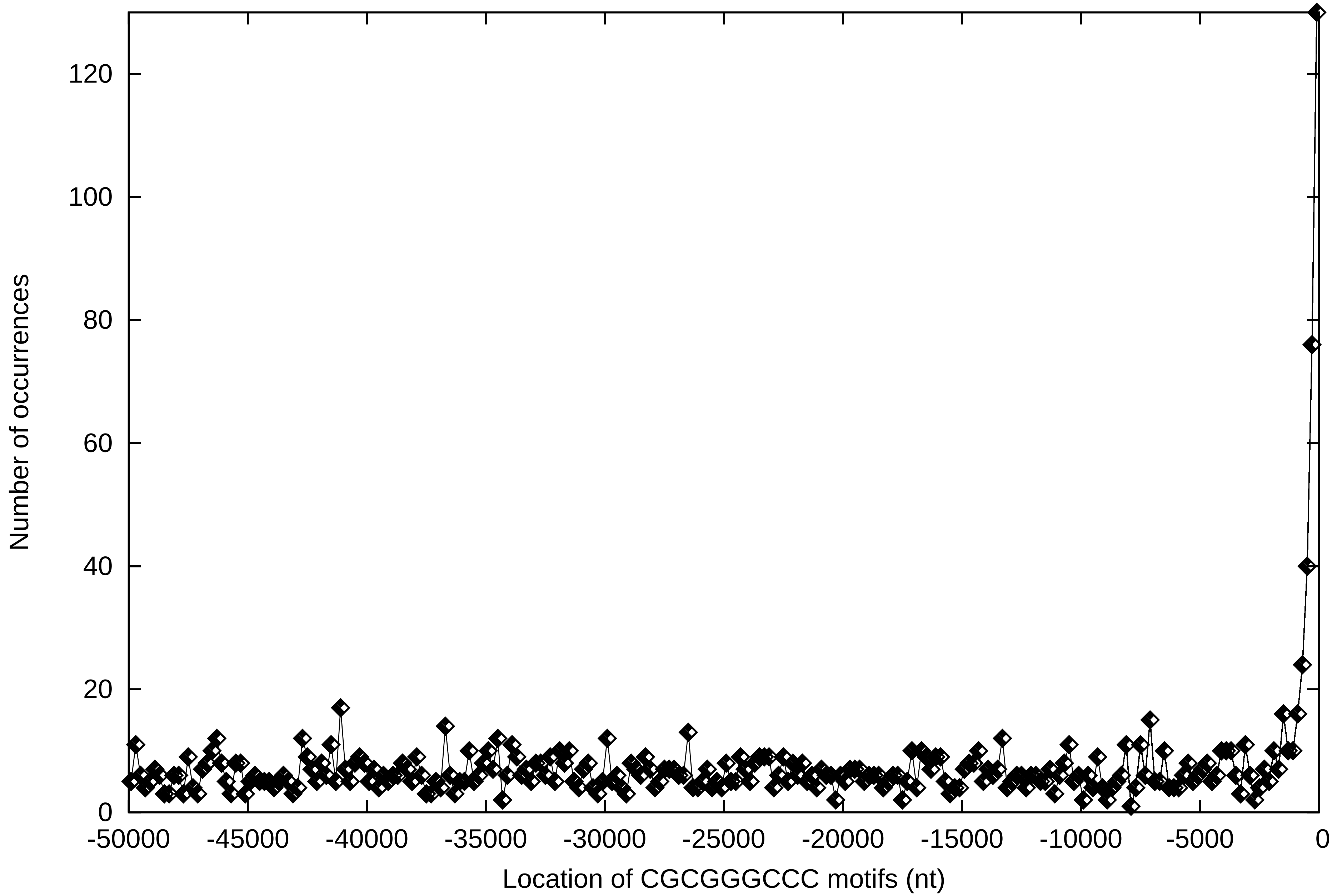

Number of occurrences
Location of CGCGGGCCC motifs (nt)
0
20
40
60
80
100
120
-50000
-45000
-40000
-35000
-30000
-25000
-20000
-15000
-10000
-5000
0
