# Supplementary material for: Regulatory Circuit of Human MicroRNA Biogenesis
Source: PLoS Comput Biol. 2007 Apr 20;3(4):e67. doi: 10.1371/journal.pcbi.0030067 (PMC1853126; doi:10.1371/journal.pcbi.0030067)
Supplement: Supplementary file 2 [file pcbi.0030067.sg002.pdf]

The 100 top-predicted 8-mers are preferentially located upstream of protein-coding genes (pages 2-101). Each graph is a histogram that represents the number of occurrences of each motif (every 200 nt stretch) in the first 50 kb upstream region of protein-coding genes. The motifs preferentially occur within the first 500 nt region (-500 to 0 nt region) that is directly upstream of the protein-coding genes.

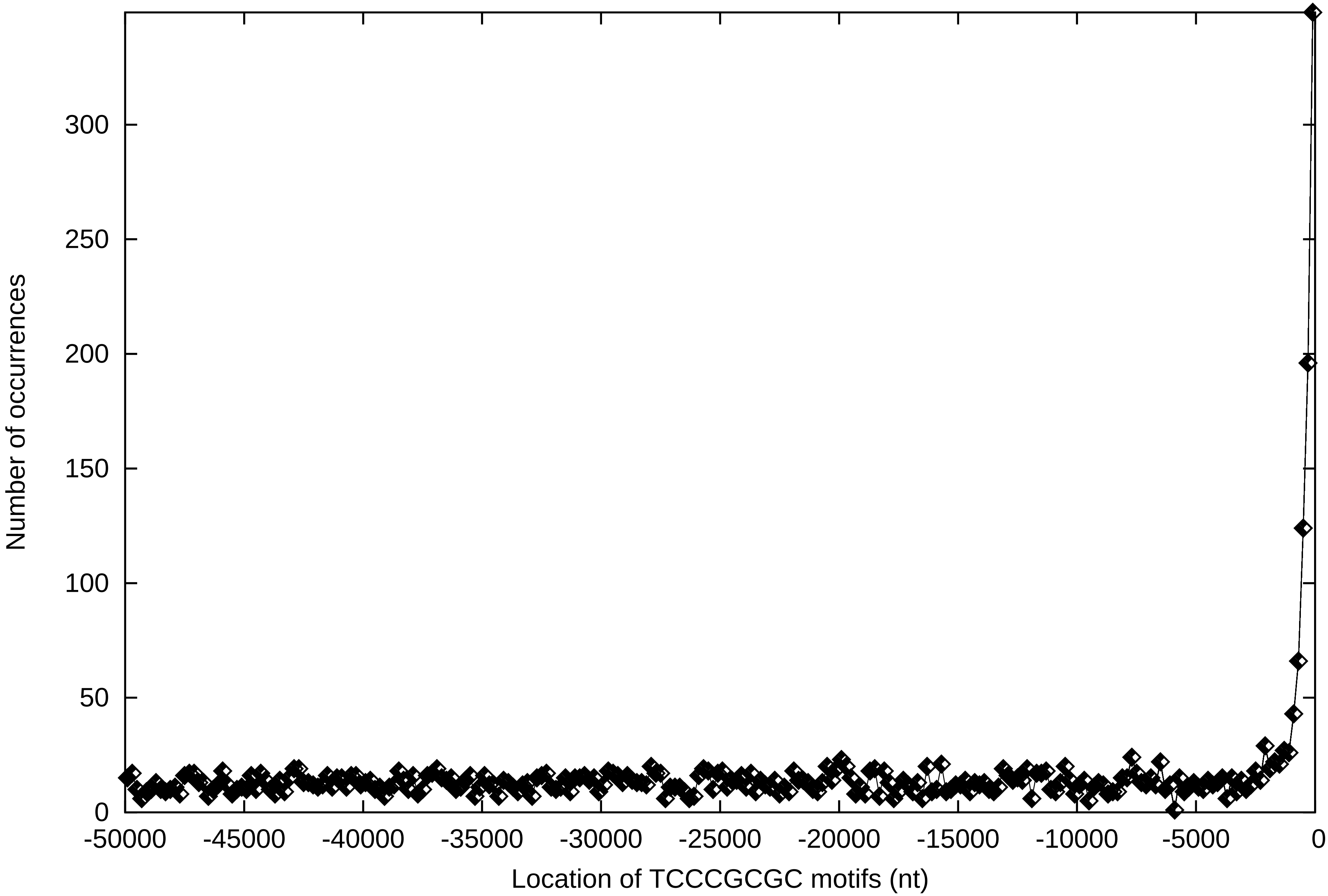

Number of occurrences
300
250
200
150
100
50
0
-50000
-45000
-40000
-35000
-30000
-25000
-20000
-15000
-10000
-5000
0
Location of TCCCGCGC motifs (nt)

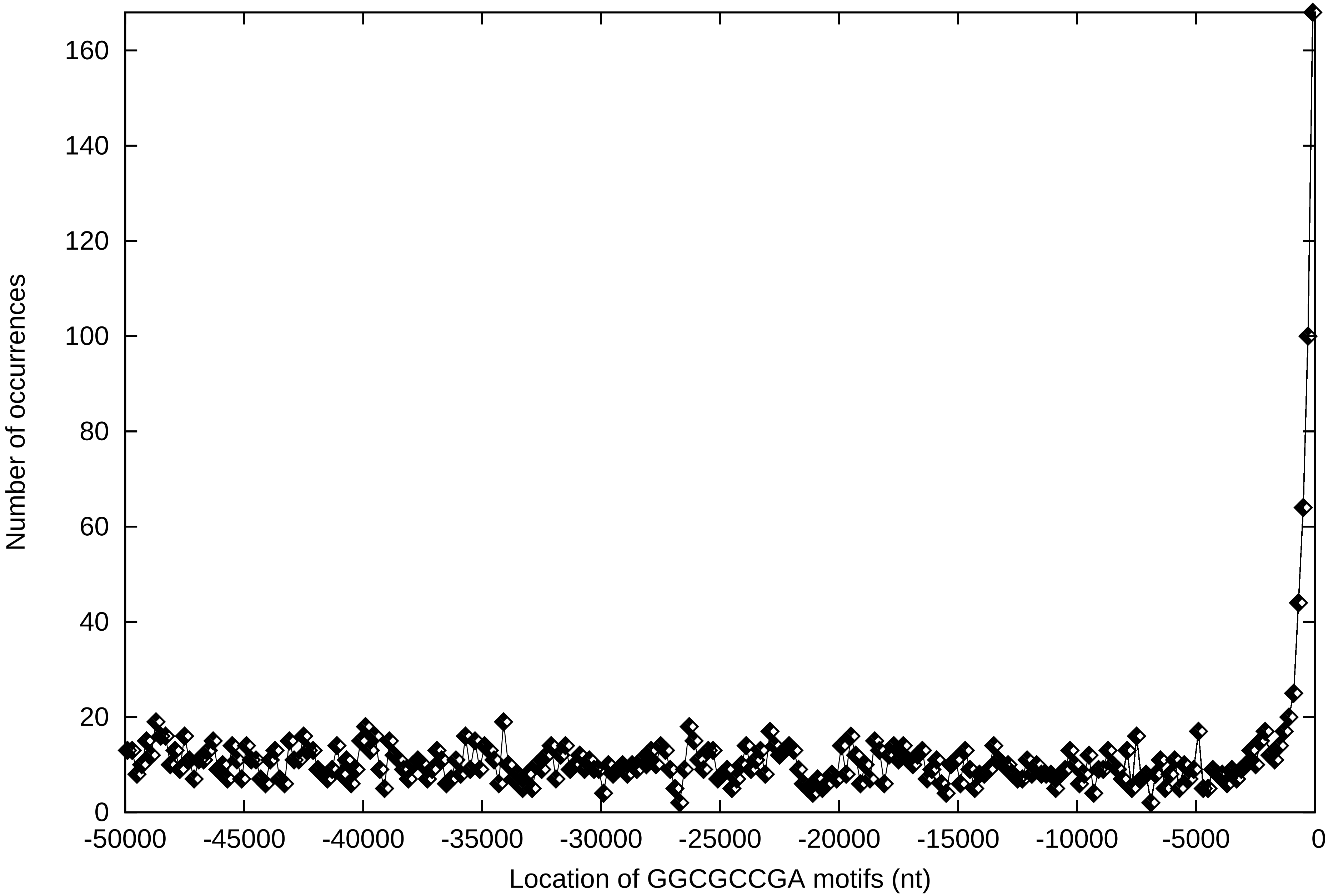

Number of occurrences
0
20
40
60
80
100
120
140
160
-50000
-45000
-40000
-35000
-30000
-25000
-20000
-15000
-10000
-5000
0
Location of GGCGCCGA motifs (nt)

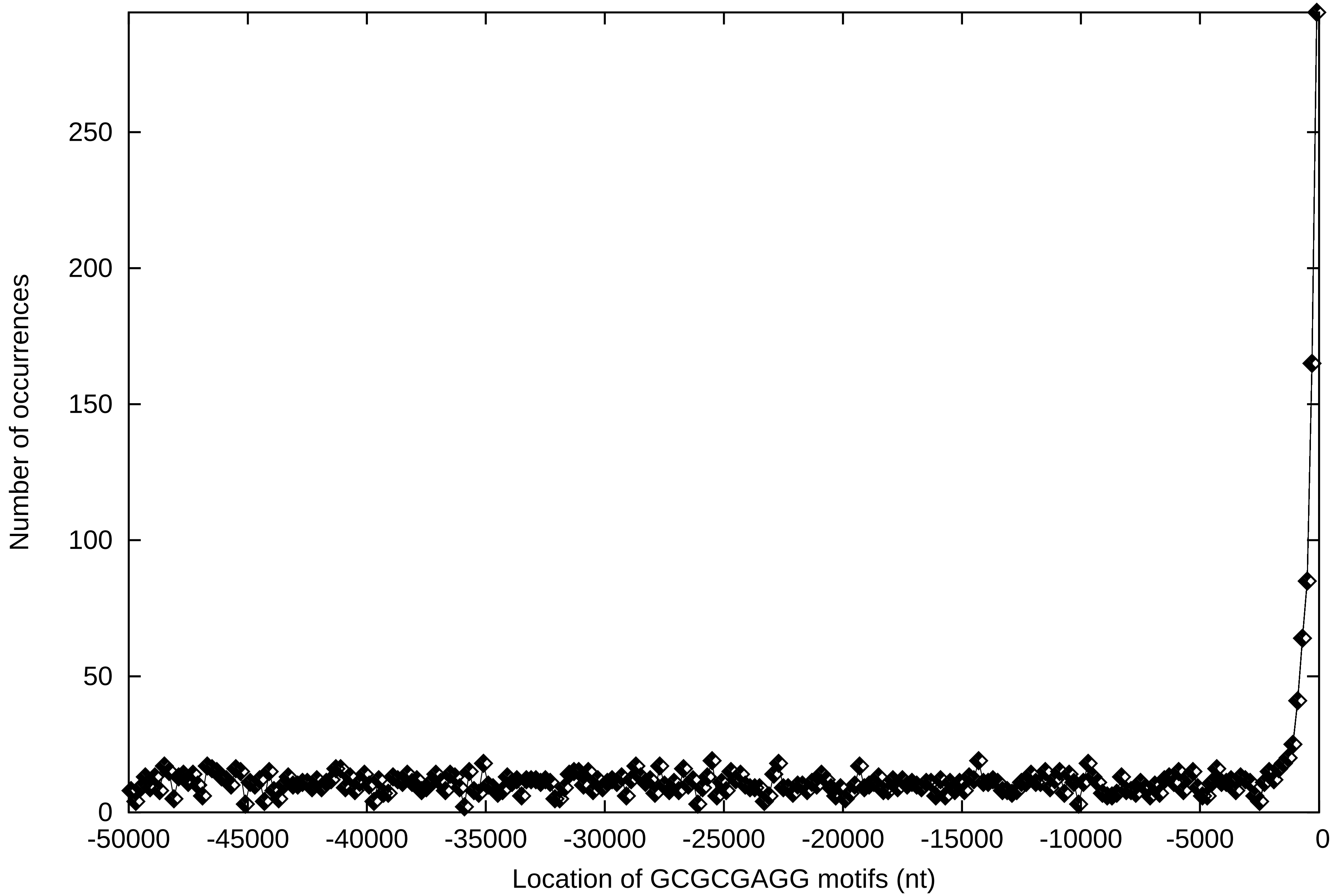
Number of occurrences
0
50
100
150
200
250
-50000
-45000
-40000
-35000
-30000
-25000
-20000
-15000
-10000
-5000
0
Location of GCGCGAGG motifs (nt)

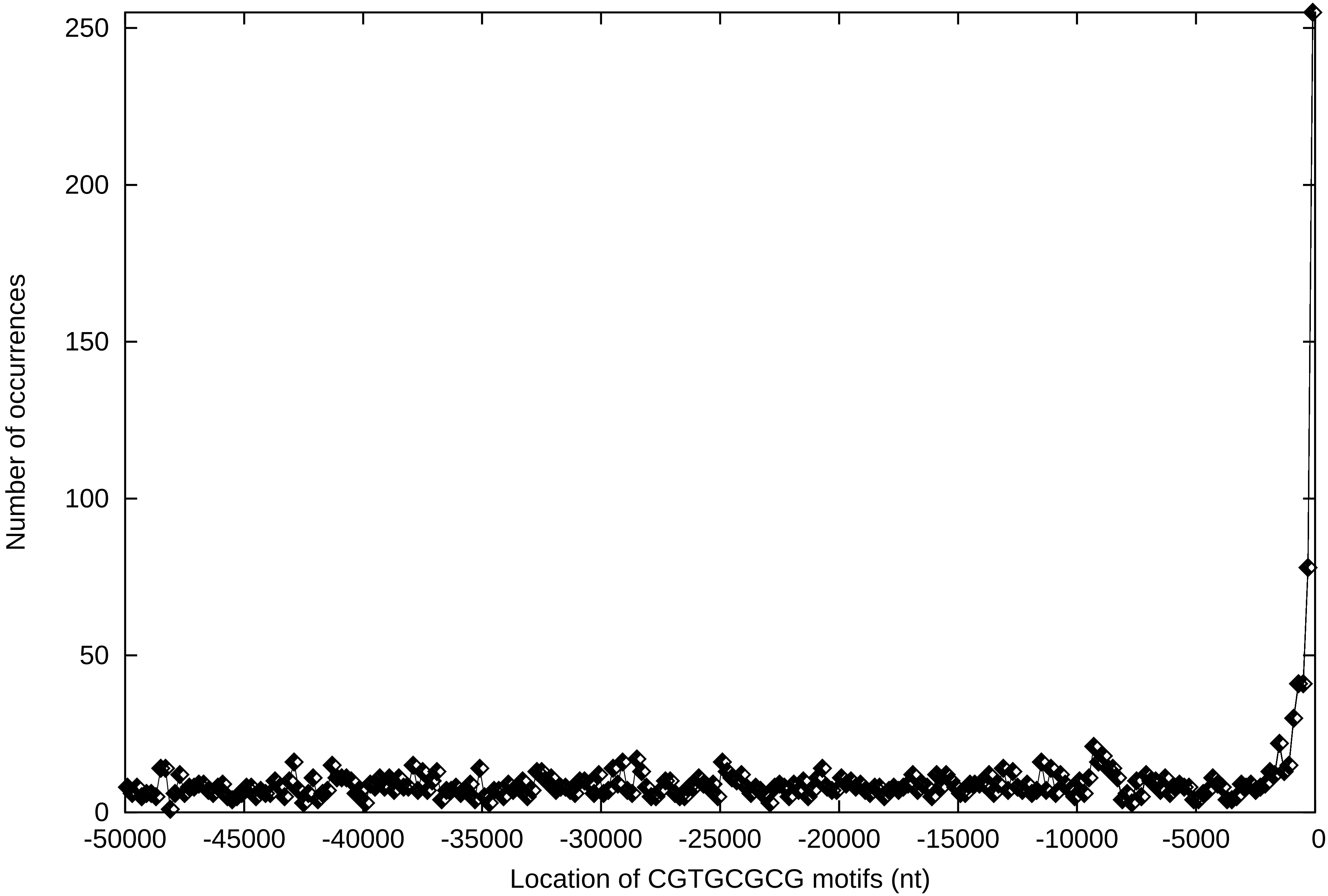
Number of occurrences
0
50
100
150
200
250
-50000
-45000
-40000
-35000
-30000
-25000
-20000
-15000
-10000
-5000
0
Location of CGTGCGCG motifs (nt)

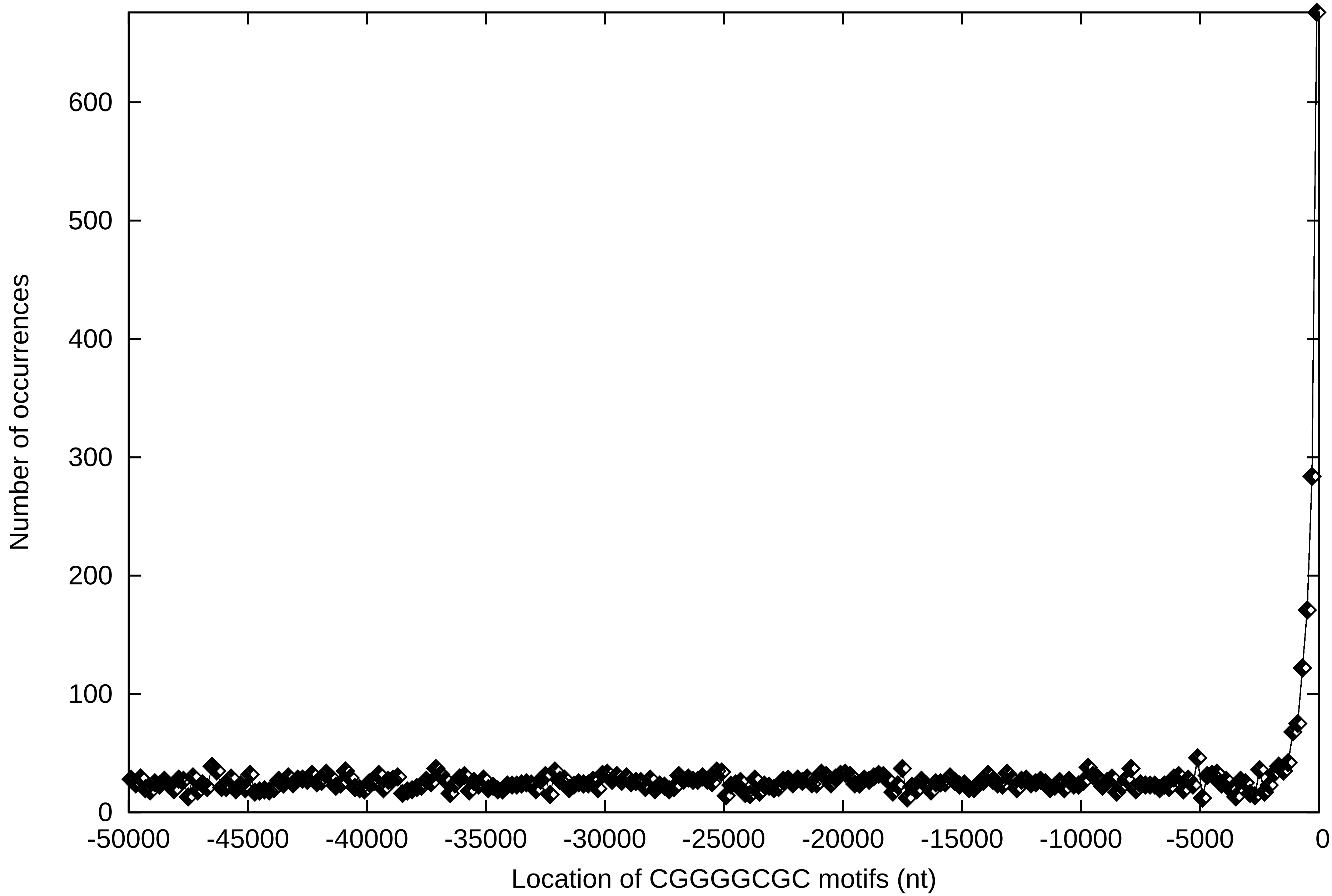

Number of occurrences
0
100
200
300
400
500
600
-50000
-45000
-40000
-35000
-30000
-25000
-20000
-15000
-10000
-5000
0
Location of CGGGGCGC motifs (nt)

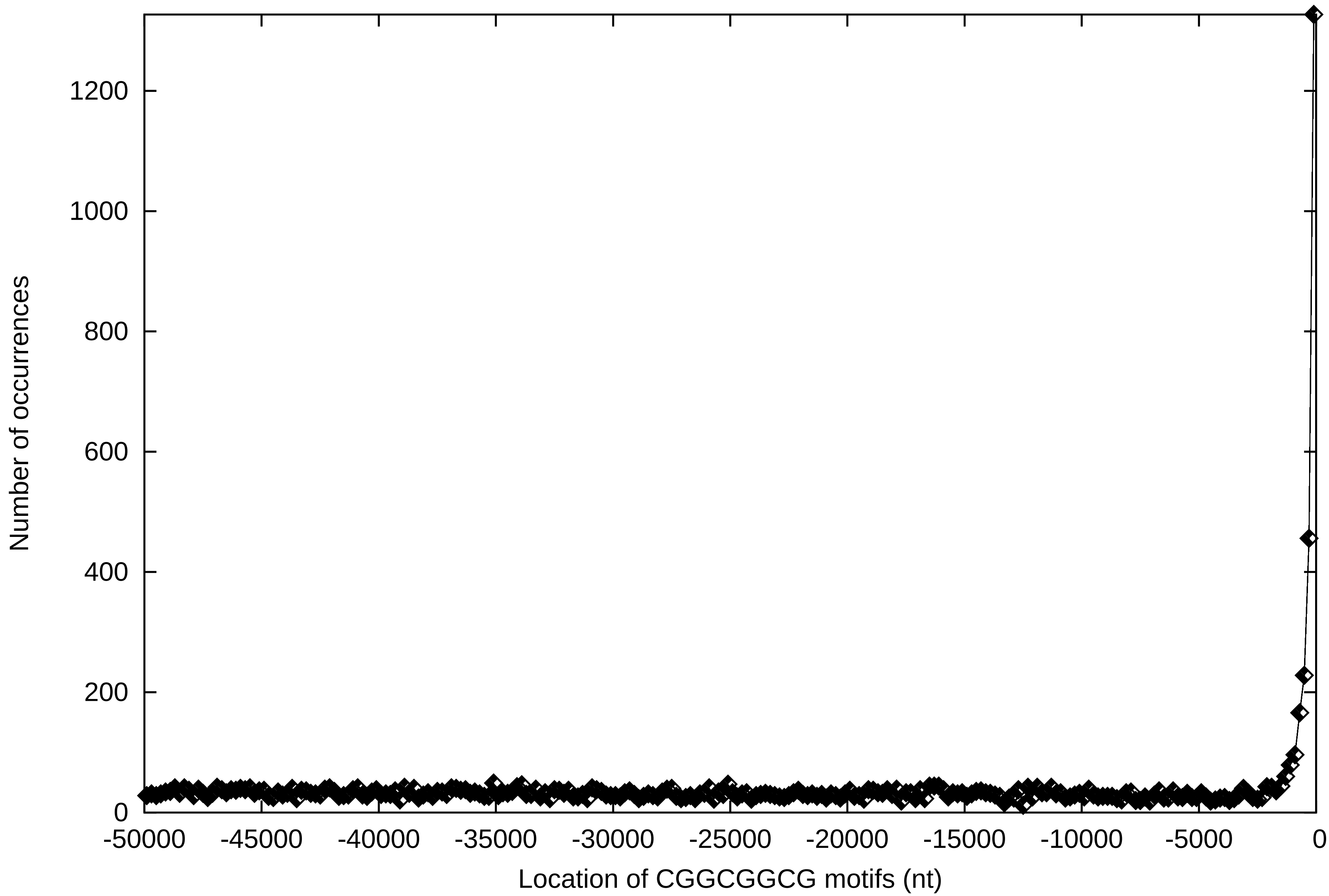

Number of occurrences
1200
1000
800
600
400
200
0
-50000
-45000
-40000
-35000
-30000
-25000
-20000
-15000
-10000
-5000
0
Location of CGGCGGCG motifs (nt)

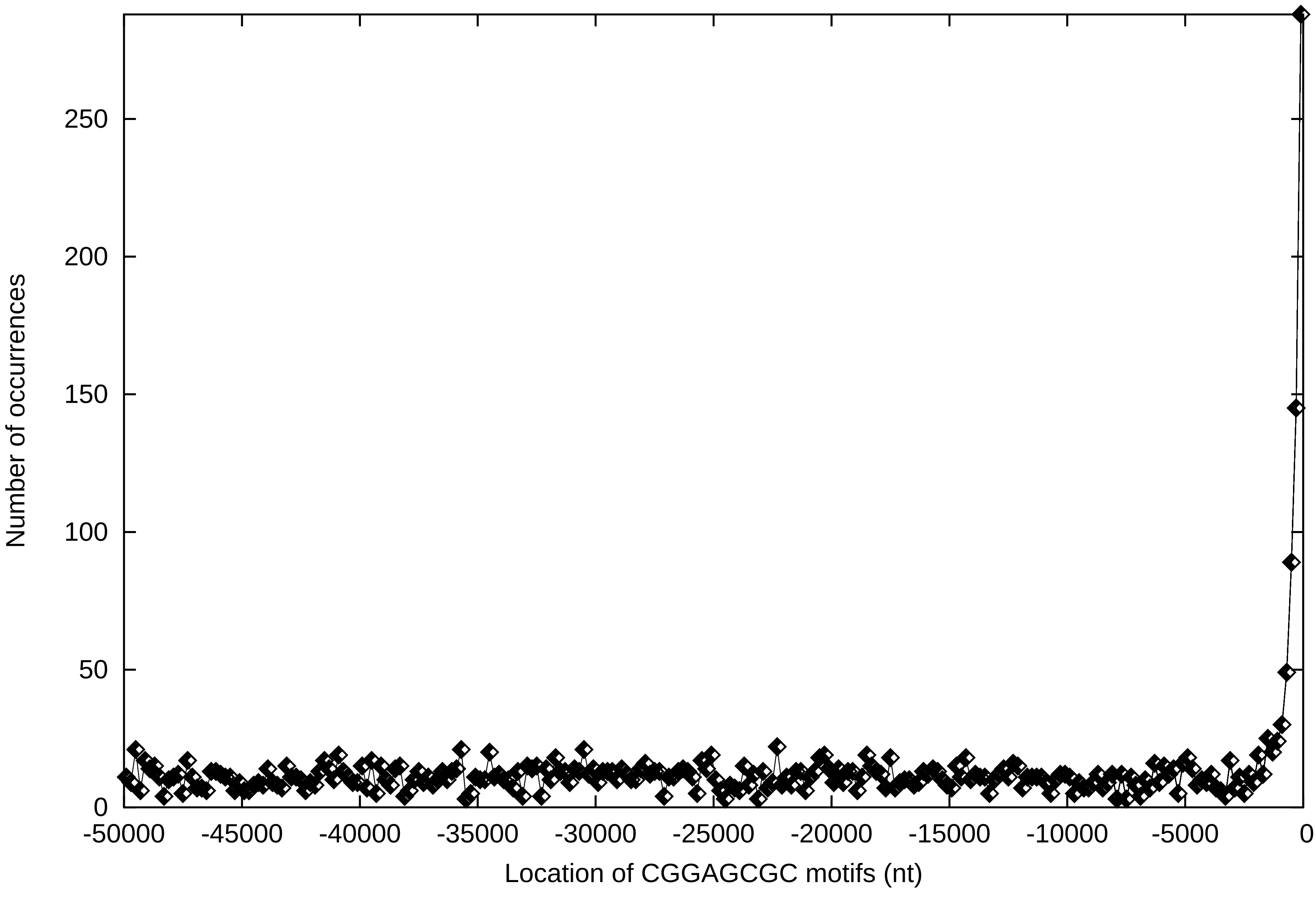

Number of occurrences
0
50
100
150
200
250
-50000
-45000
-40000
-35000
-30000
-25000
-20000
-15000
-10000
-5000
0
Location of CGGAGCGC motifs (nt)

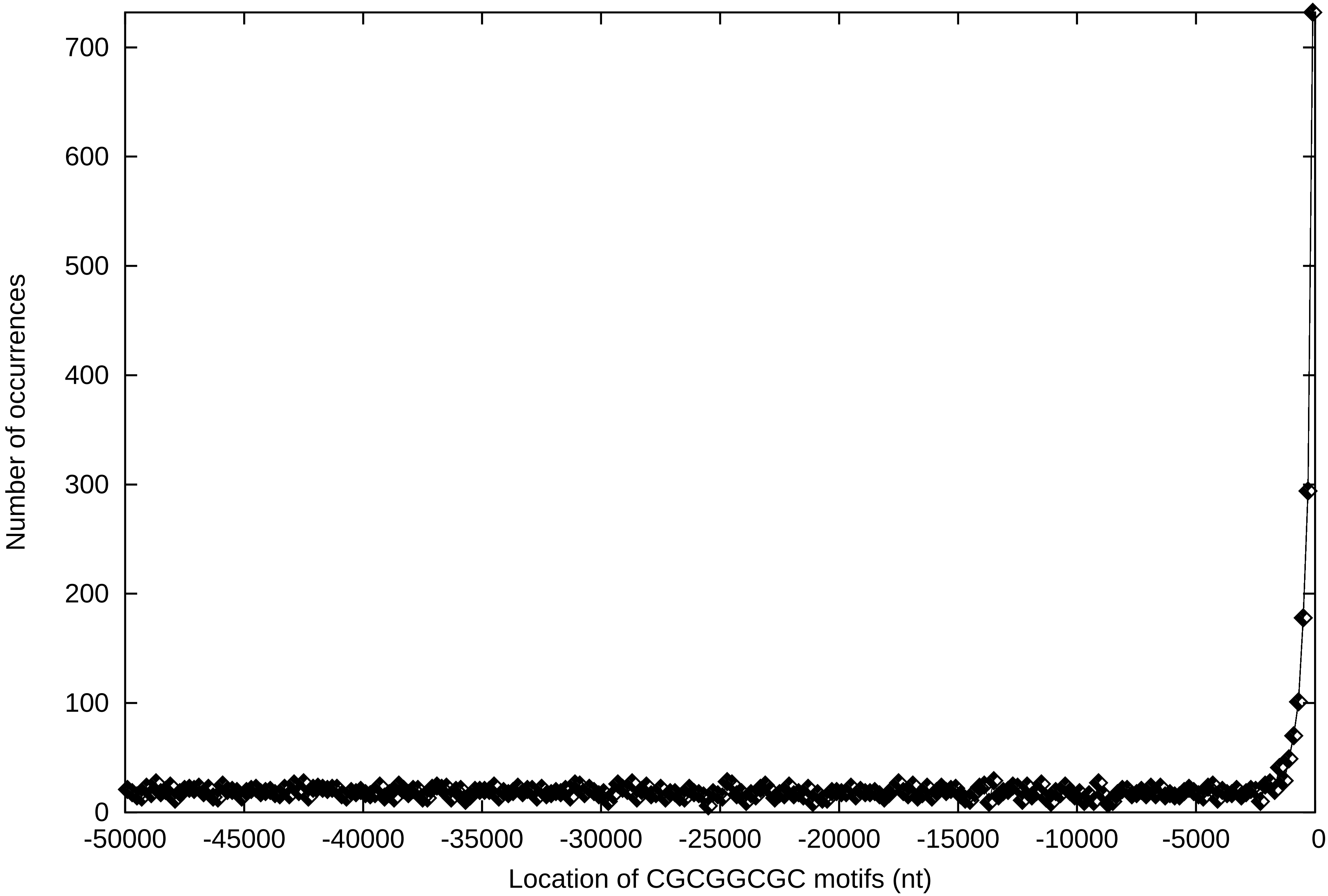
Number of occurrences
0
100
200
300
400
500
600
700
-50000
-45000
-40000
-35000
-30000
-25000
-20000
-15000
-10000
-5000
0
Location of CGCGGCGC motifs (nt)

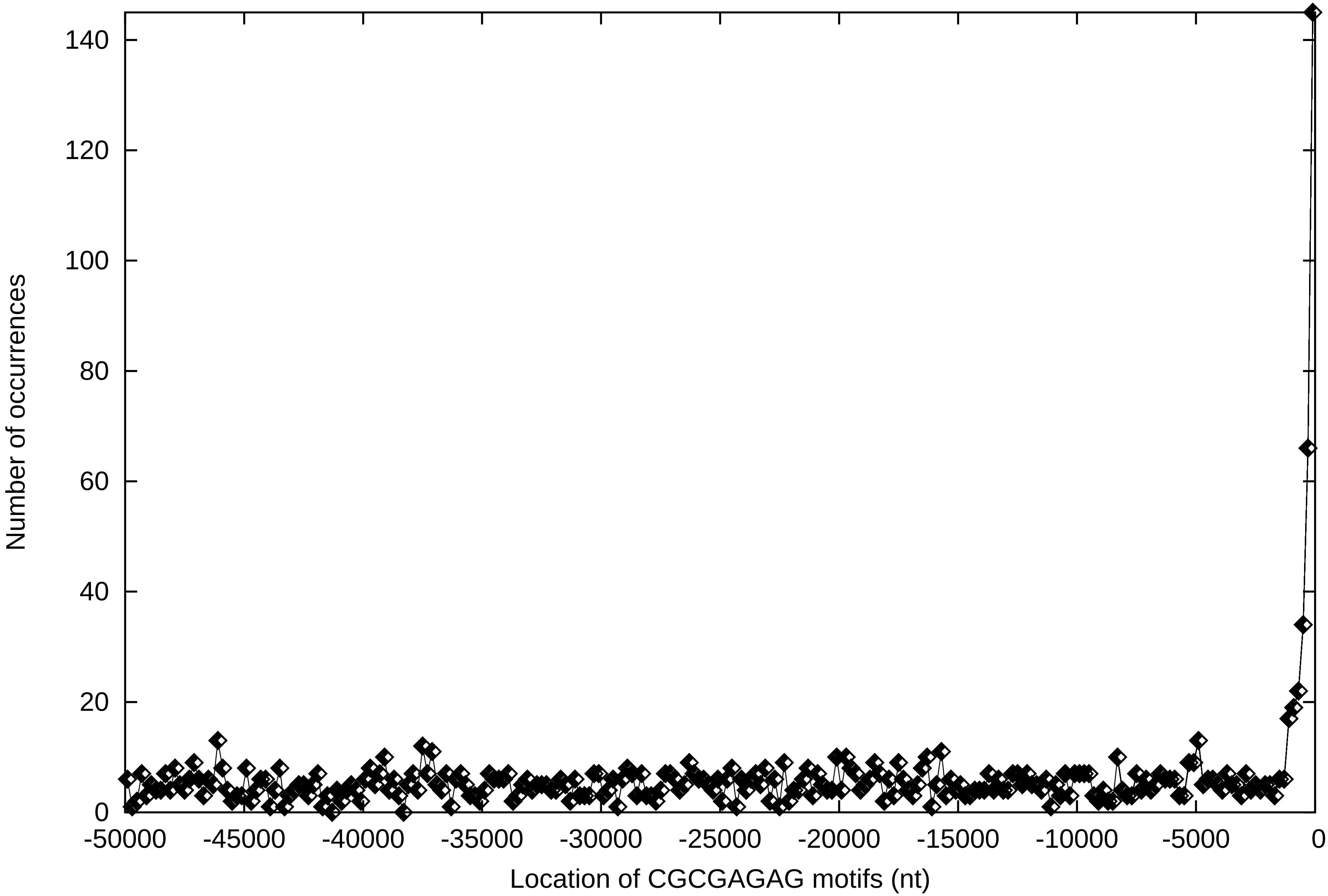

Number of occurrences
140
120
100
80
60
40
20
0
-50000
-45000
-40000
-35000
-30000
-25000
-20000
-15000
-10000
-5000
0
Location of CGCGAGAG motifs (nt)

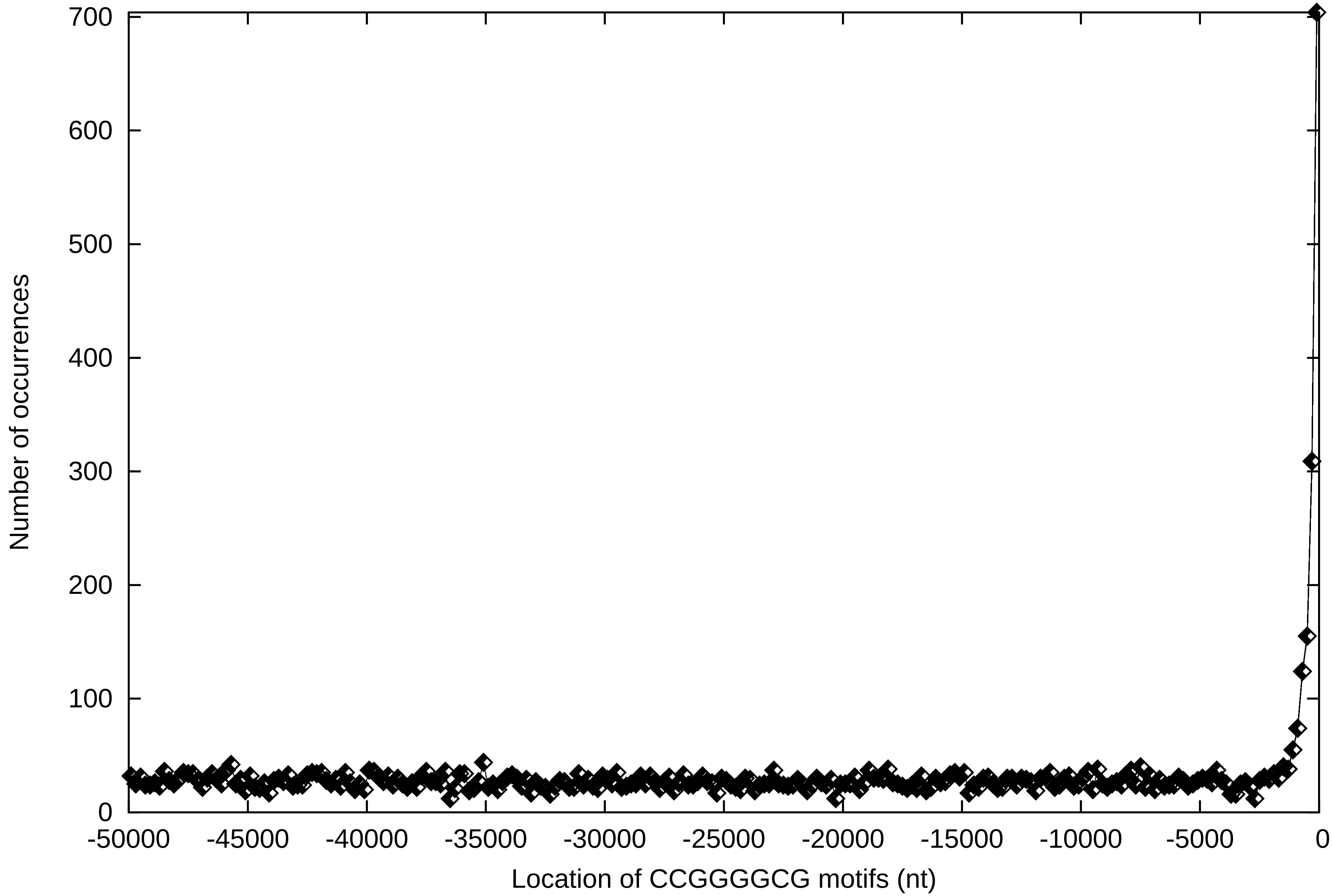

Number of occurrences
700
600
500
400
300
200
100
0
-50000
-45000
-40000
-35000
-30000
-25000
-20000
-15000
-10000
-5000
0
Location of CCGGGGCG motifs (nt)

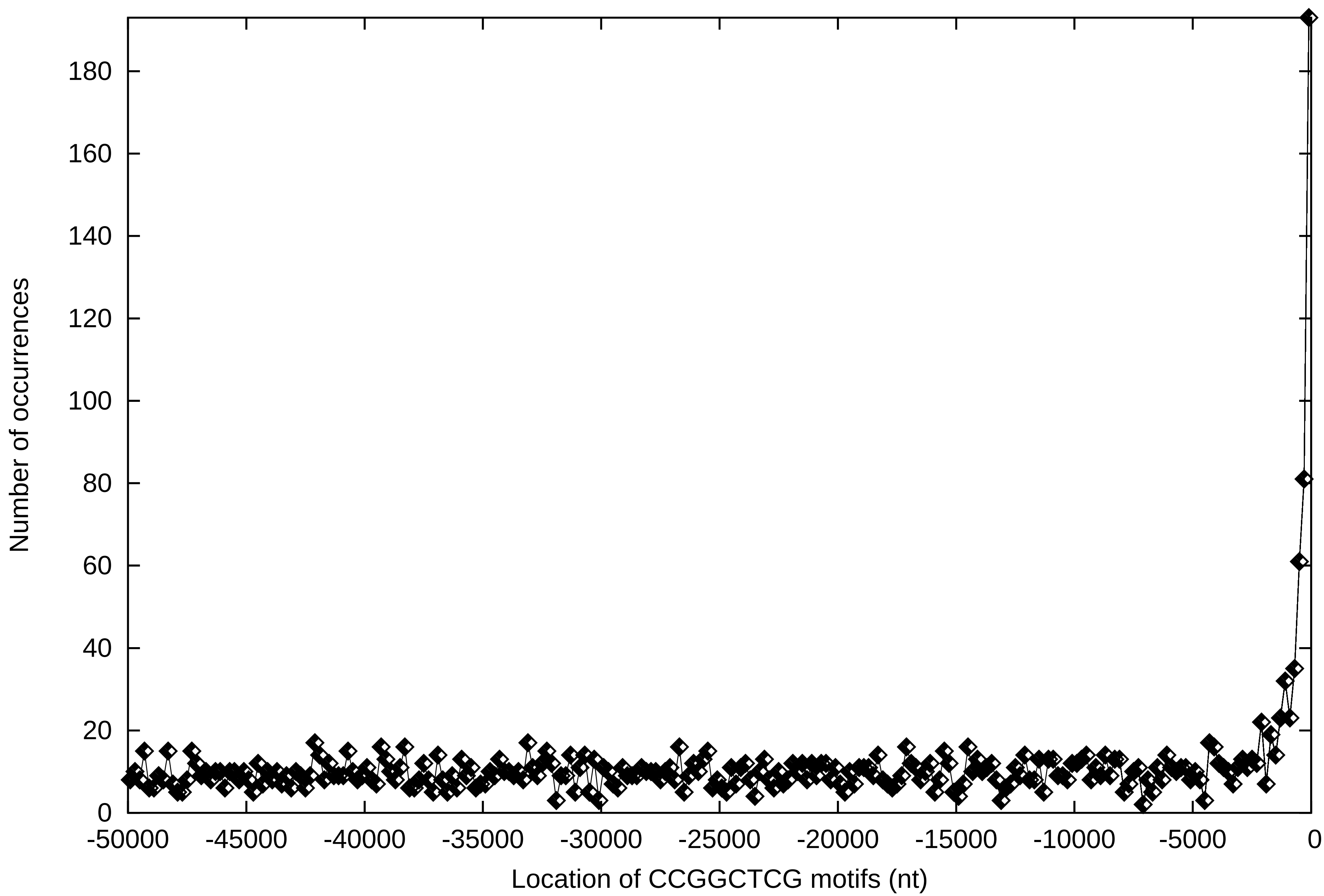

Number of occurrences
0
20
40
60
80
100
120
140
160
180
-50000
-45000
-40000
-35000
-30000
-25000
-20000
-15000
-10000
-5000
0
Location of CCGGCTCG motifs (nt)

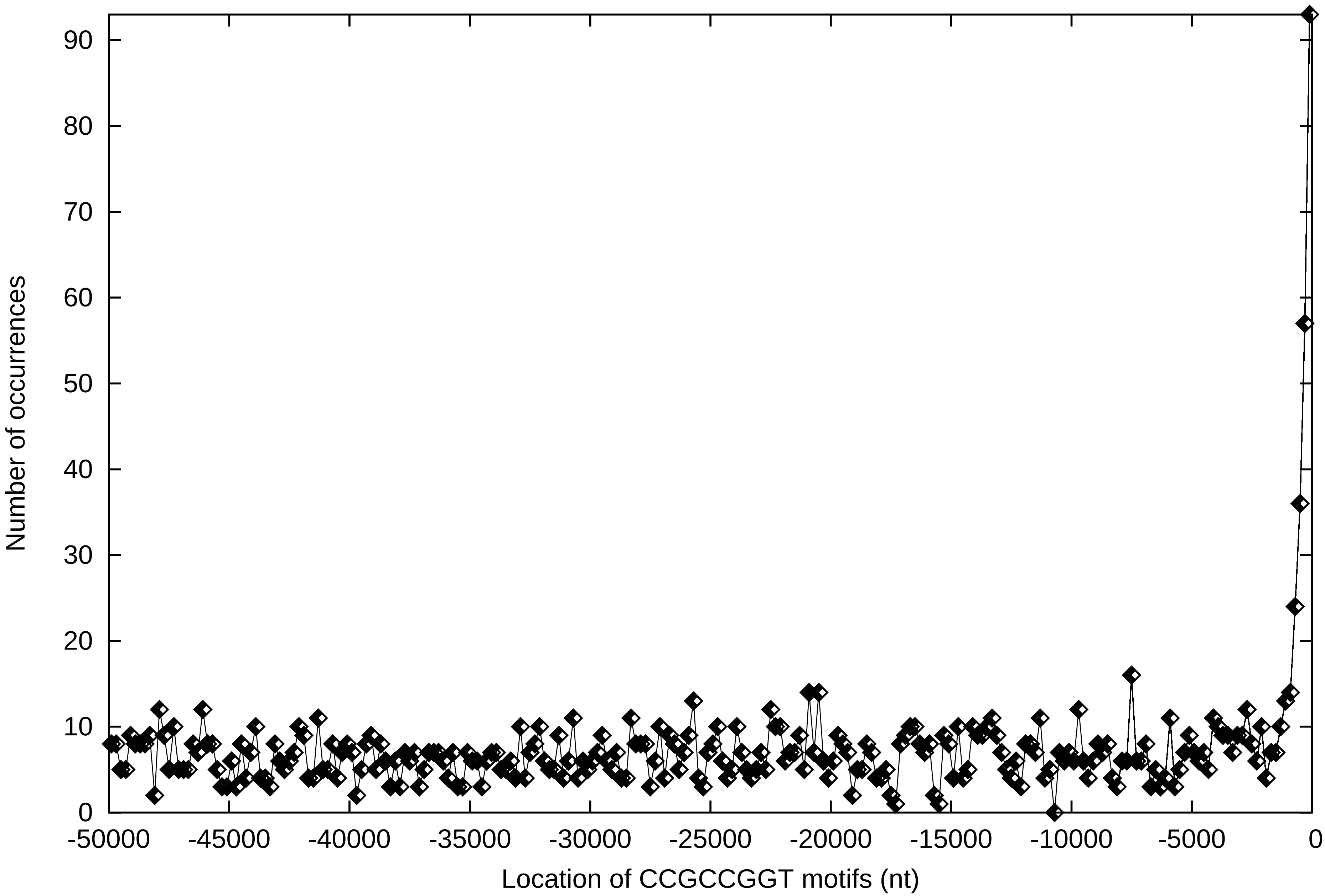

Number of occurrences
0
10
20
30
40
50
60
70
80
90
-50000
-45000
-40000
-35000
-30000
-25000
-20000
-15000
-10000
-5000
0
Location of CCGCCGGT motifs (nt)

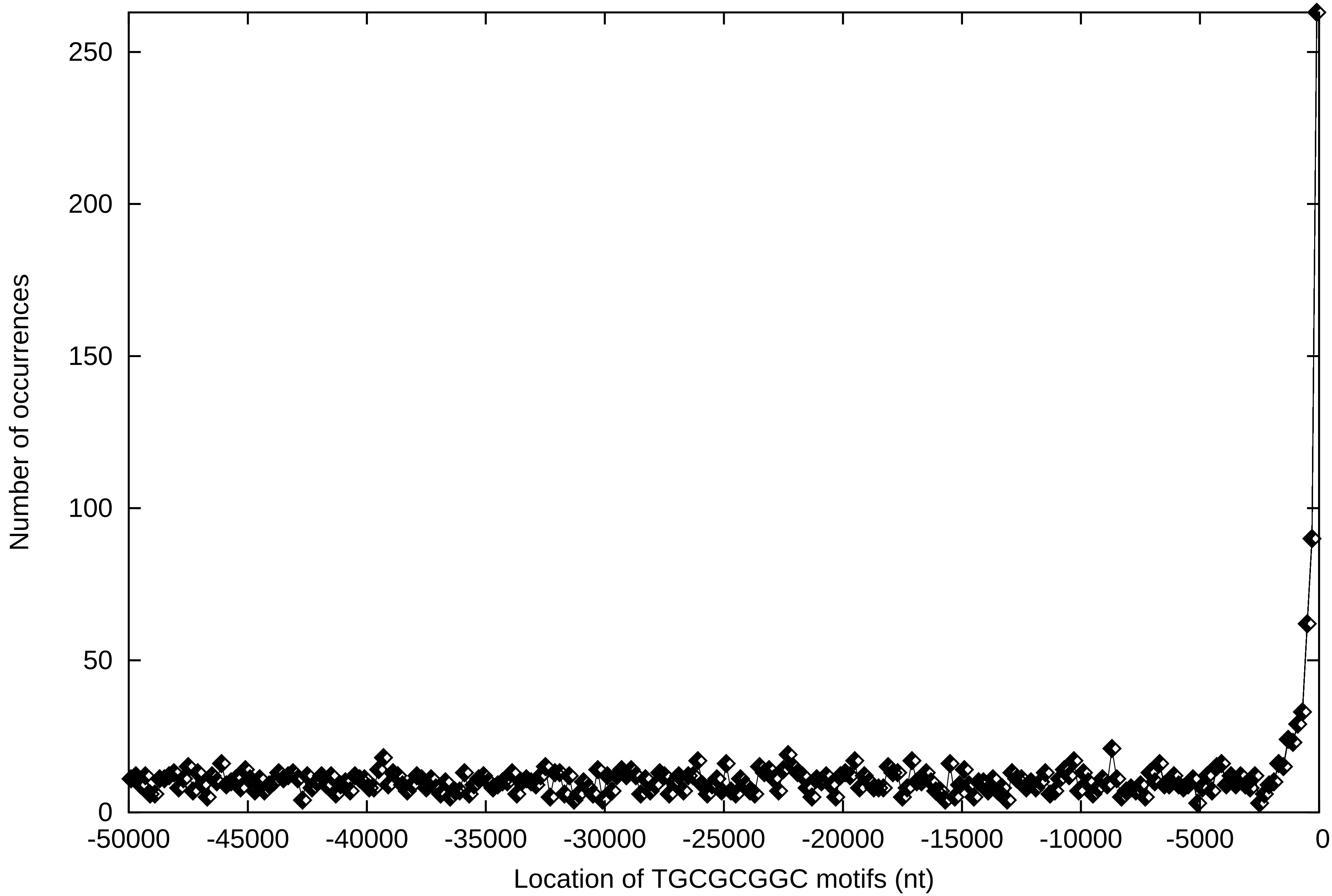
Number of occurrences
250
200
150
100
50
0
-50000
-45000
-40000
-35000
-30000
-25000
-20000
-15000
-10000
-5000
0
Location of TGCGCGGC motifs (nt)

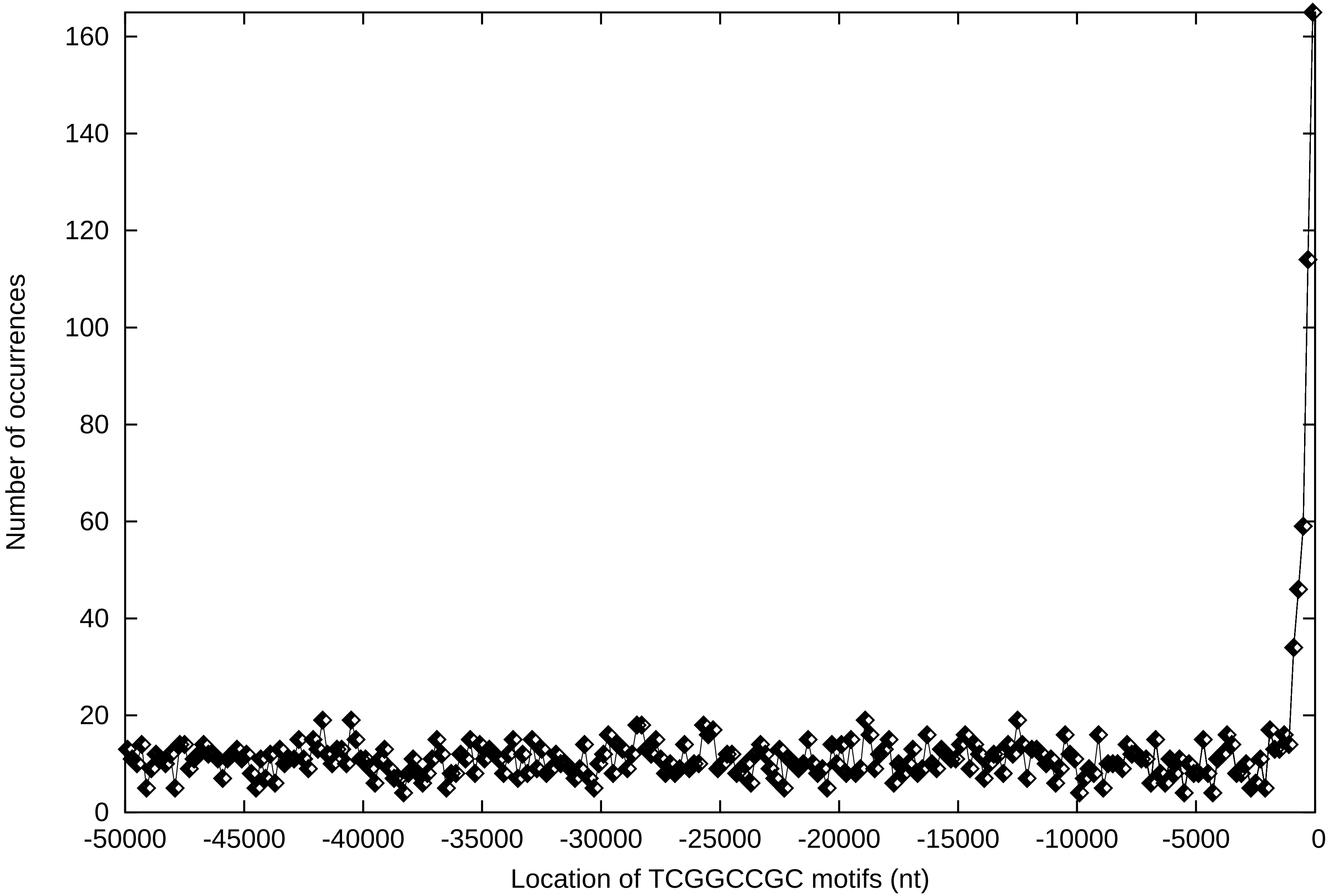
Number of occurrences
0
20
40
60
80
100
120
140
160
-50000
-45000
-40000
-35000
-30000
-25000
-20000
-15000
-10000
-5000
0
Location of TCGGCCGC motifs (nt)

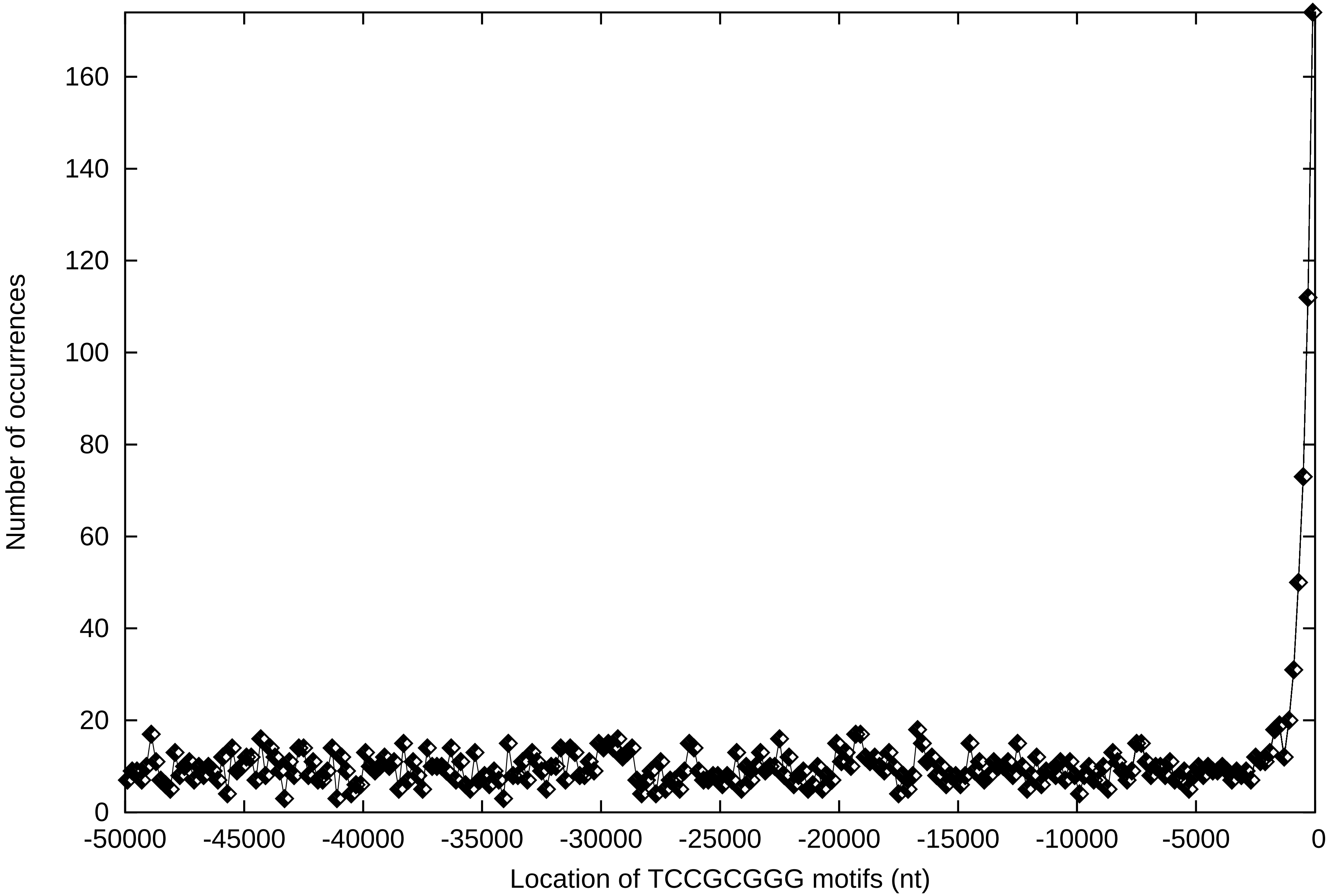

Number of occurrences
0
20
40
60
80
100
120
140
160
-50000
-45000
-40000
-35000
-30000
-25000
-20000
-15000
-10000
-5000
0
Location of TCCGCGGG motifs (nt)

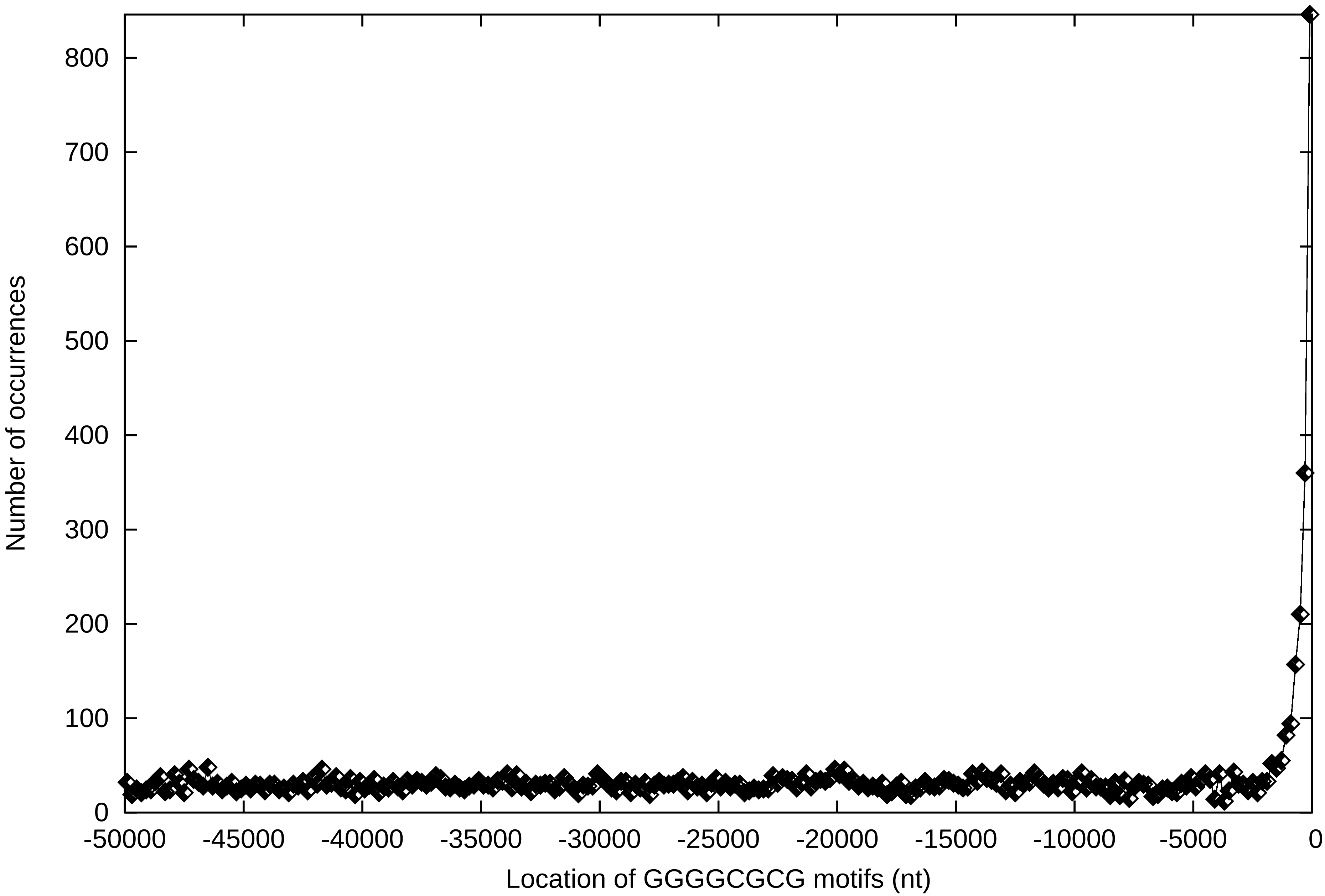

Number of occurrences
0
100
200
300
400
500
600
700
800
-50000
-45000
-40000
-35000
-30000
-25000
-20000
-15000
-10000
-5000
0
Location of GGGGCGCG motifs (nt)

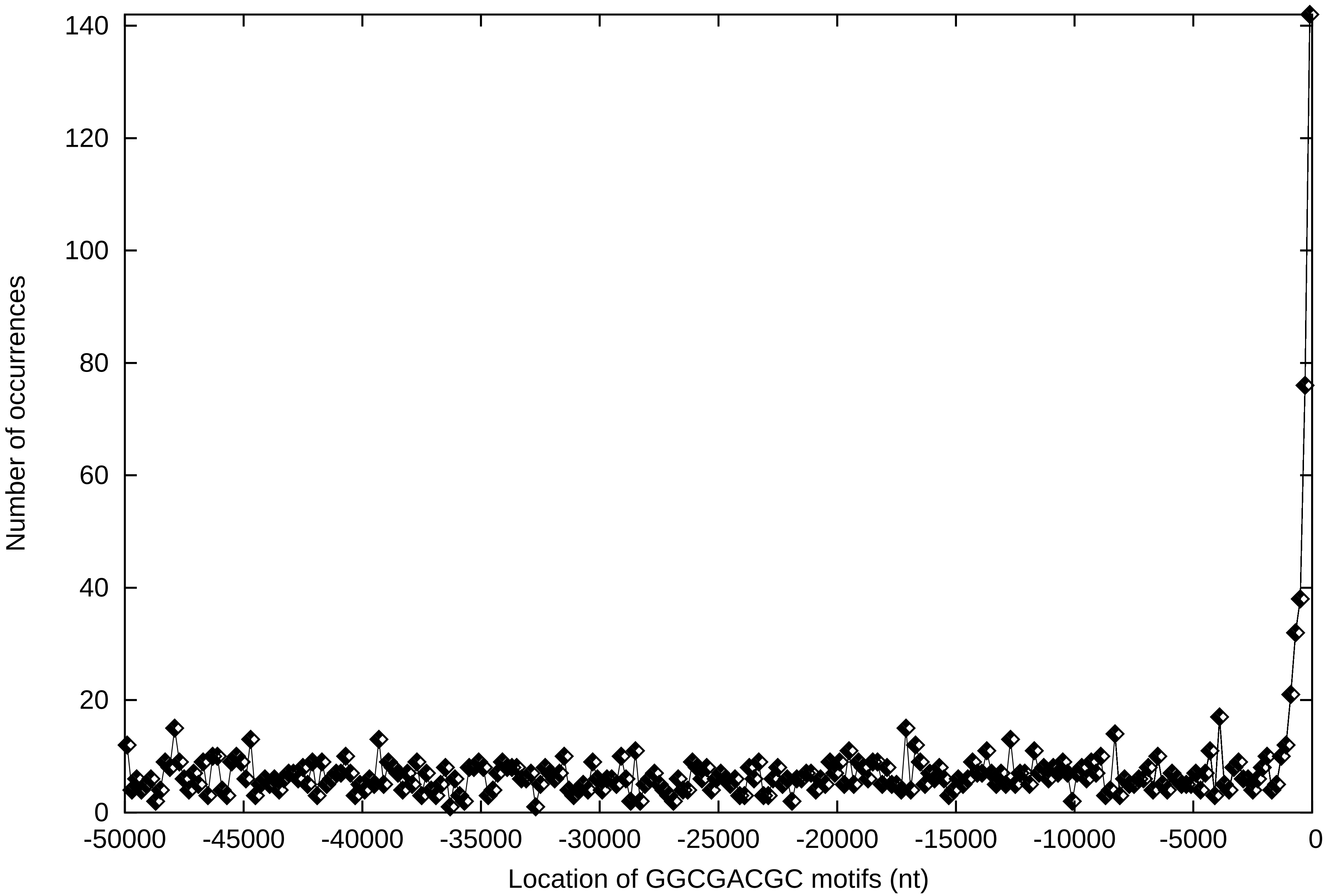

140
120
100
80
60
40
20
0
Number of occurrences
-50000
-45000
-40000
-35000
-30000
-25000
-20000
-15000
-10000
-5000
0
Location of GGCGACGC motifs (nt)

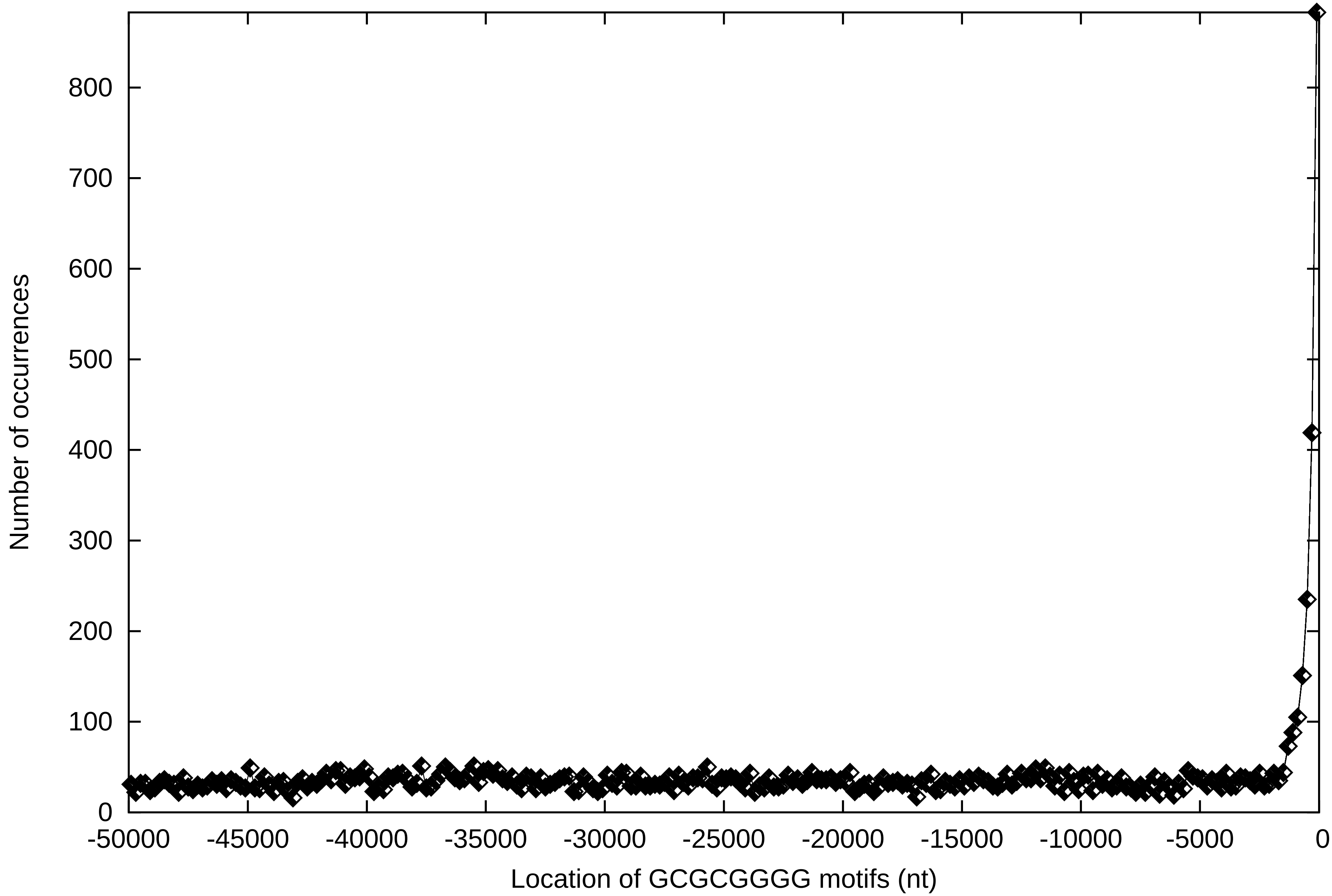

Number of occurrences
0
100
200
300
400
500
600
700
800
-50000
-45000
-40000
-35000
-30000
-25000
-20000
-15000
-10000
-5000
0
Location of GCGCGGGG motifs (nt)

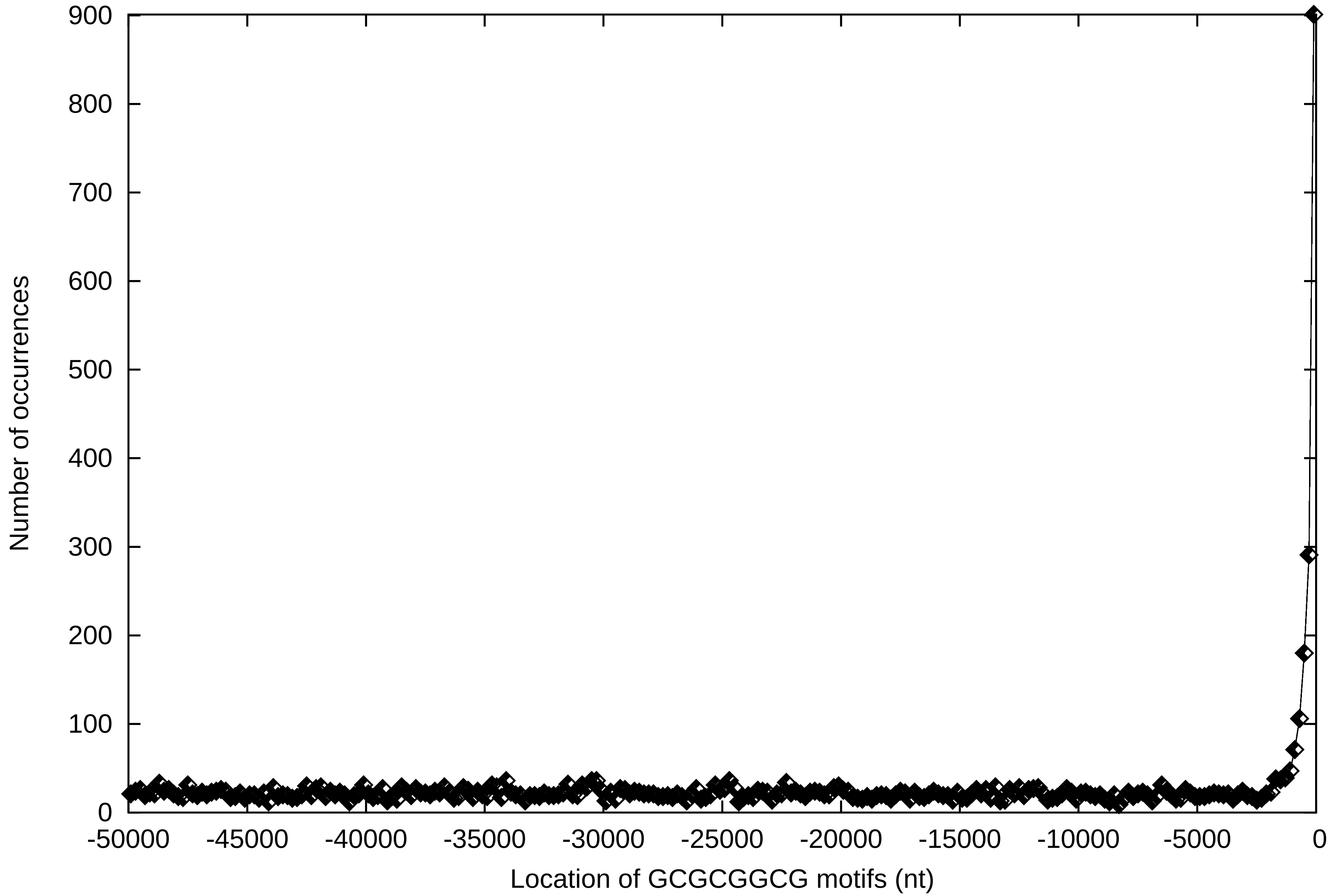

Number of occurrences
900
800
700
600
500
400
300
200
100
0
-50000
-45000
-40000
-35000
-30000
-25000
-20000
-15000
-10000
-5000
0
Location of GCGCGGCG motifs (nt)

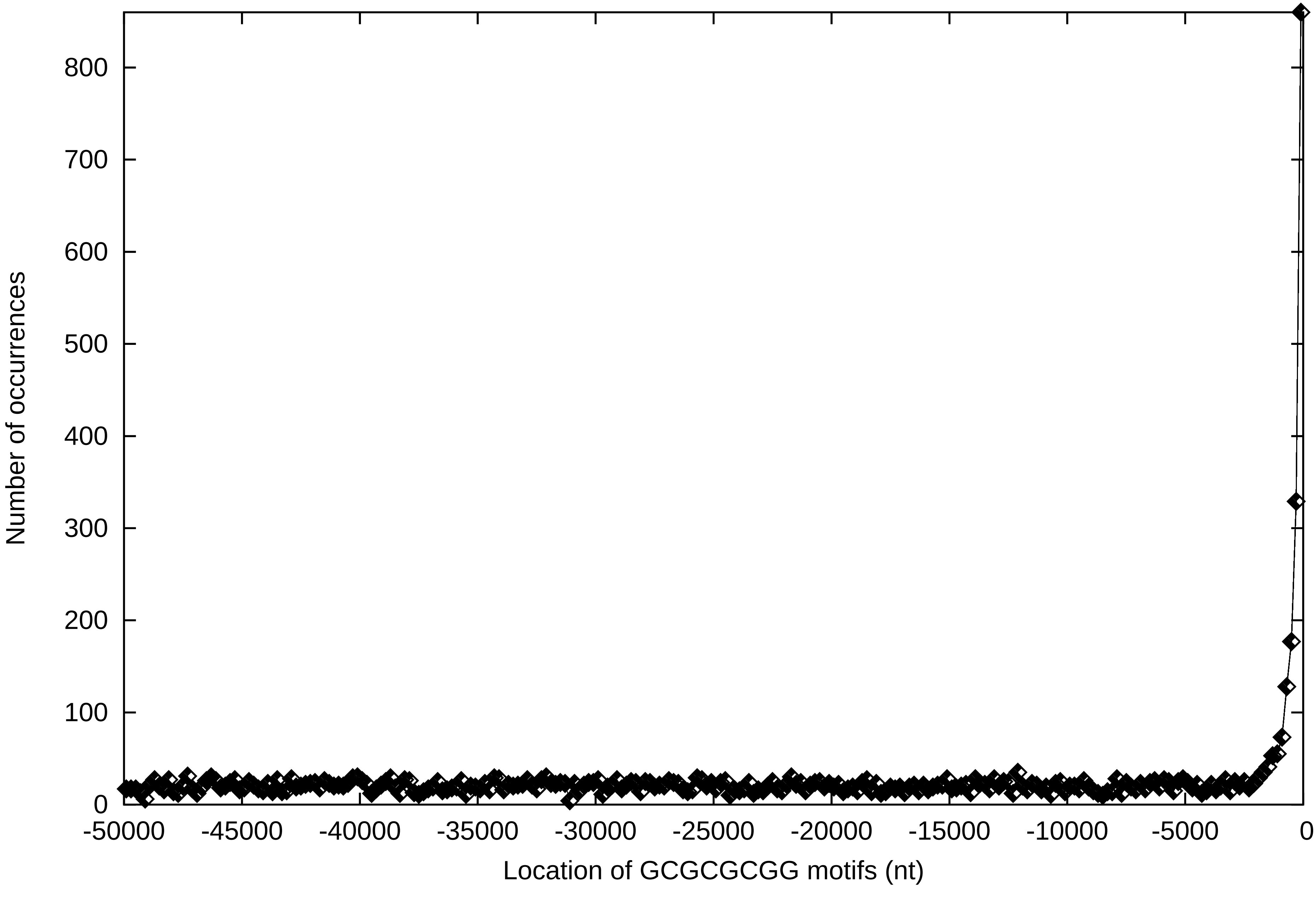

800
700
600
500
400
300
200
100
0
-50000
-45000
-40000
-35000
-30000
-25000
-20000
-15000
-10000
-5000
0
Number of occurrences
Location of GCGCGCGG motifs (nt)

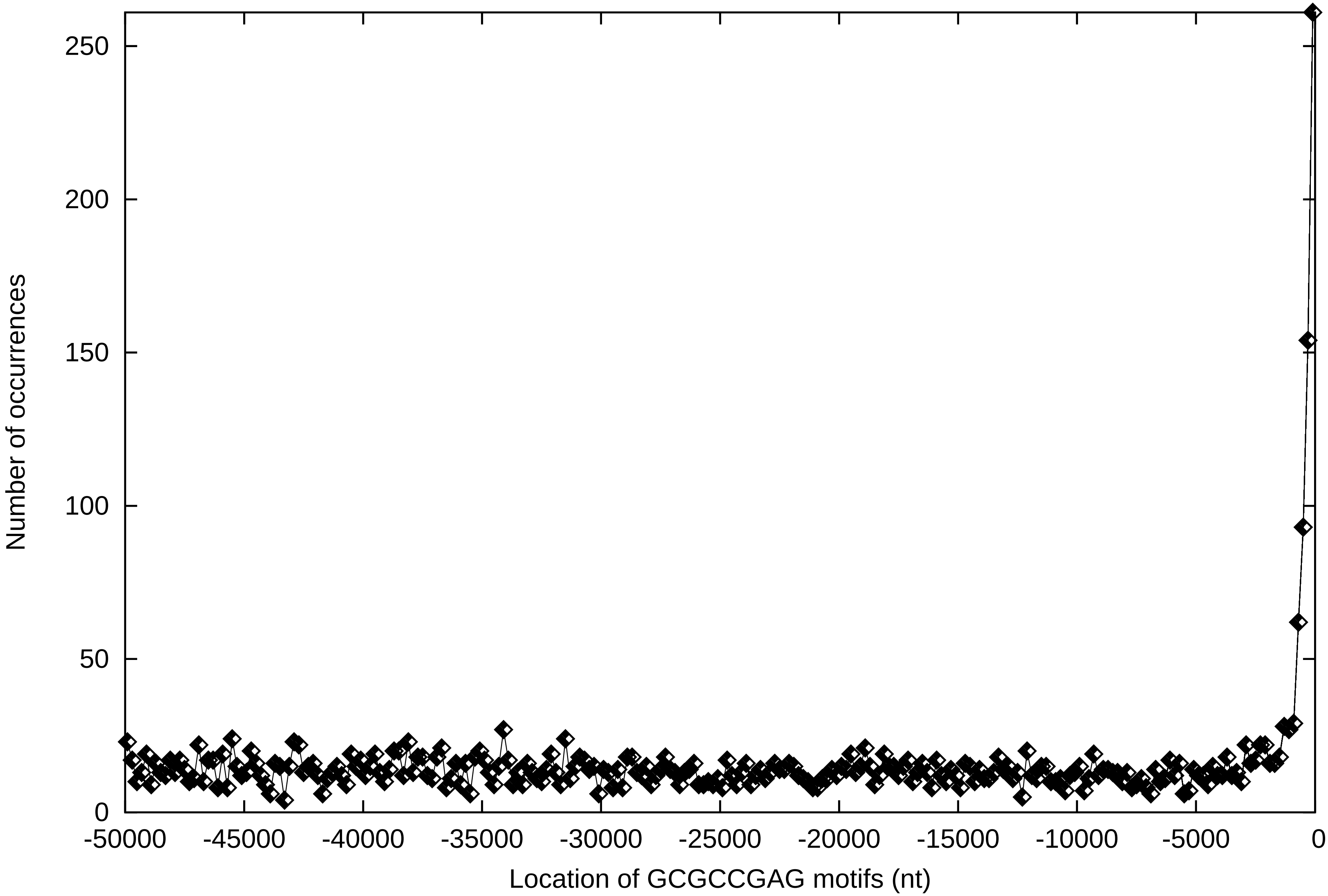

Number of occurrences
0
50
100
150
200
250
-50000
-45000
-40000
-35000
-30000
-25000
-20000
-15000
-10000
-5000
0
Location of GCGCCGAG motifs (nt)

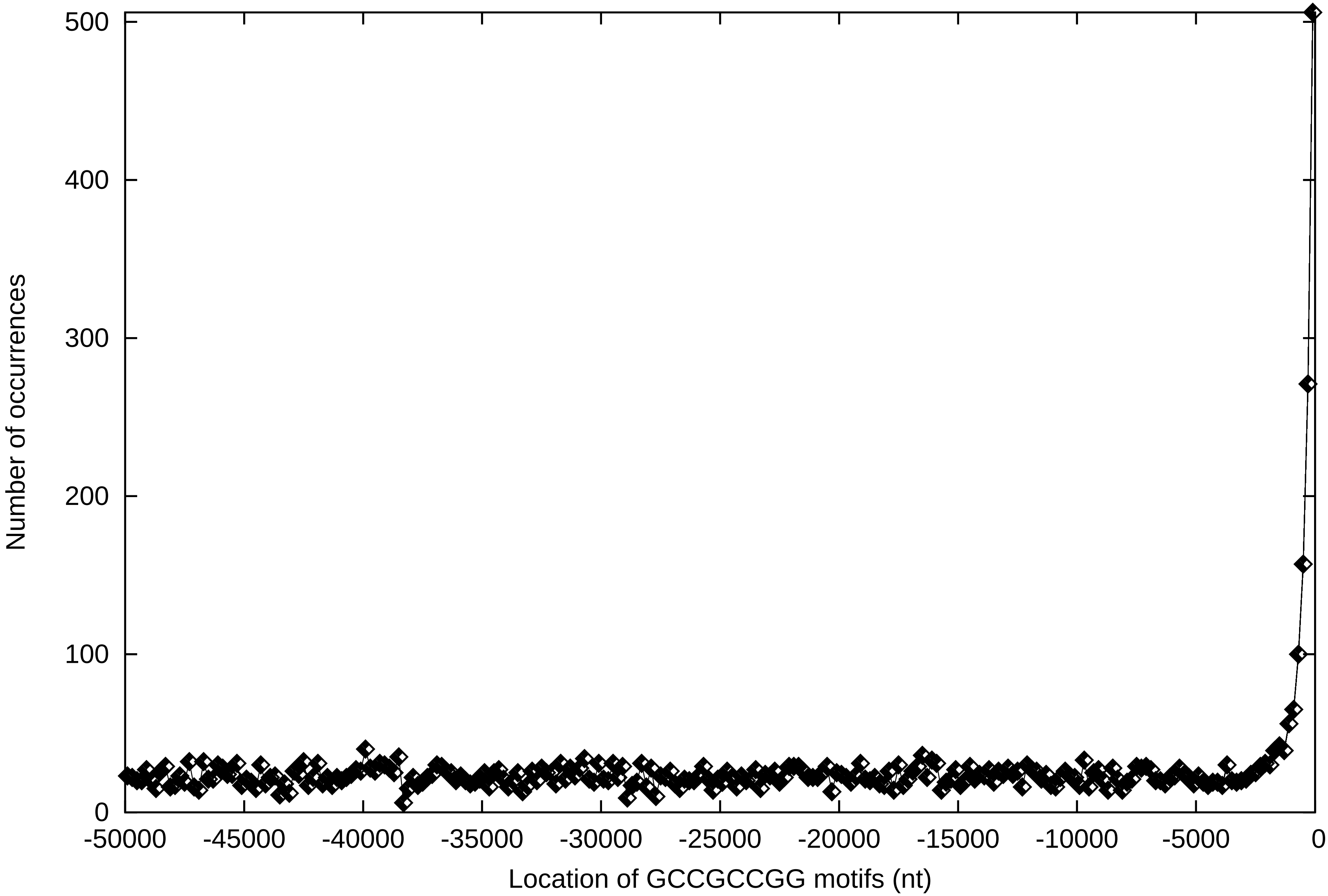

Number of occurrences
500
400
300
200
100
0
-50000
-45000
-40000
-35000
-30000
-25000
-20000
-15000
-10000
-5000
0
Location of GCCGCCGG motifs (nt)

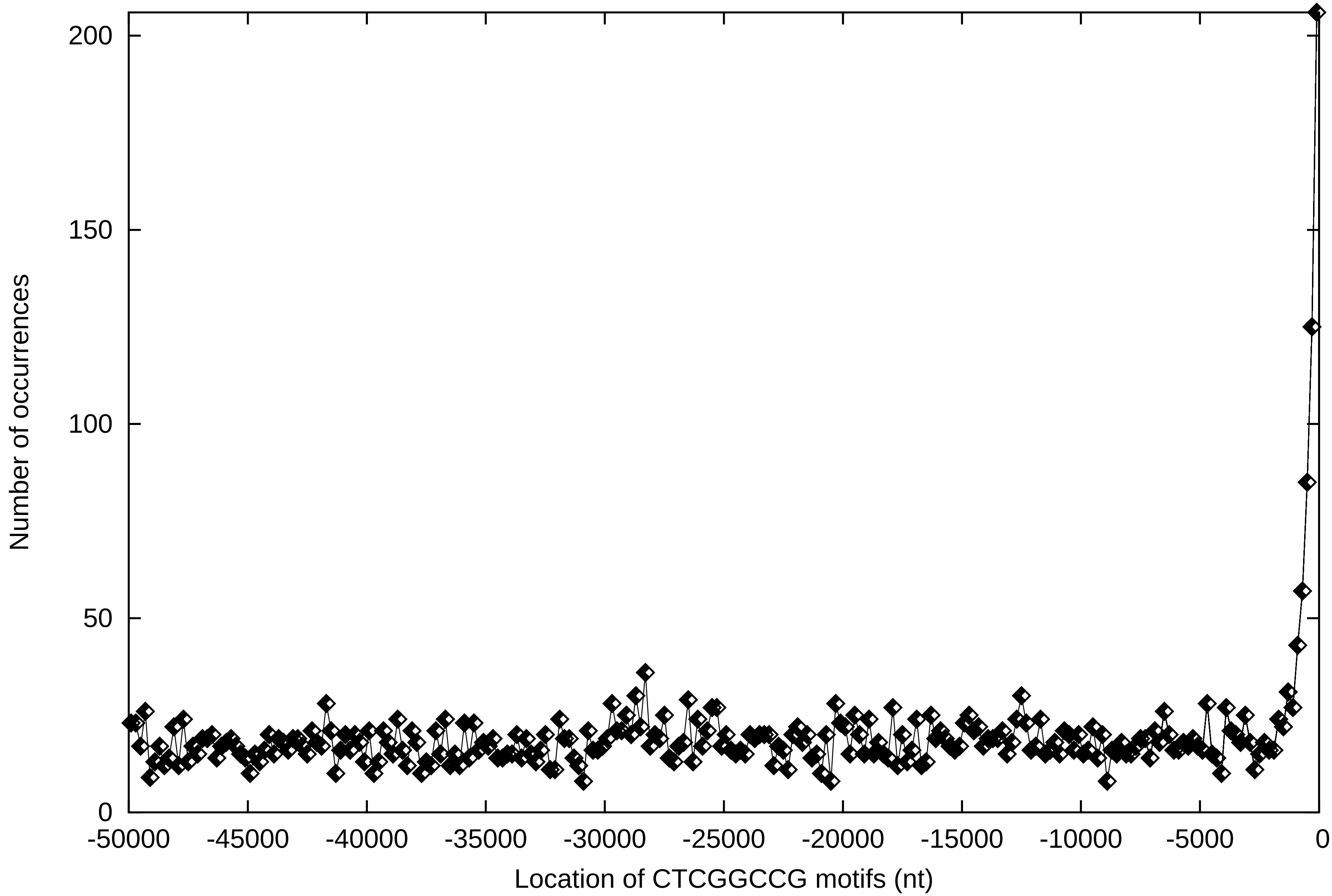

Number of occurrences
200
150
100
50
0
-50000
-45000
-40000
-35000
-30000
-25000
-20000
-15000
-10000
-5000
0
Location of CTCGGCCG motifs (nt)

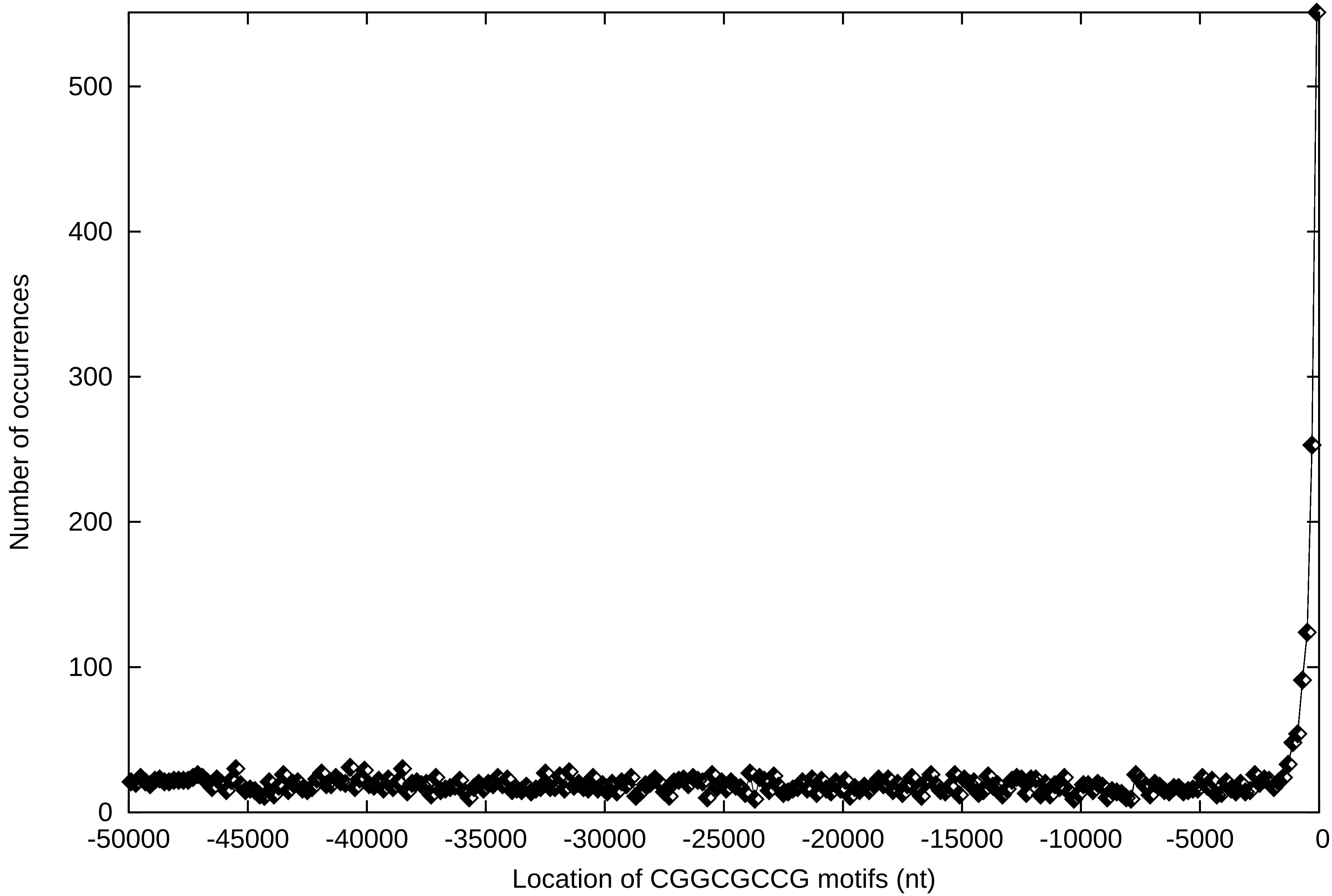
Number of occurrences
Location of CGGCGCCG motifs (nt)
0
100
200
300
400
500
-50000
-45000
-40000
-35000
-30000
-25000
-20000
-15000
-10000
-5000
0

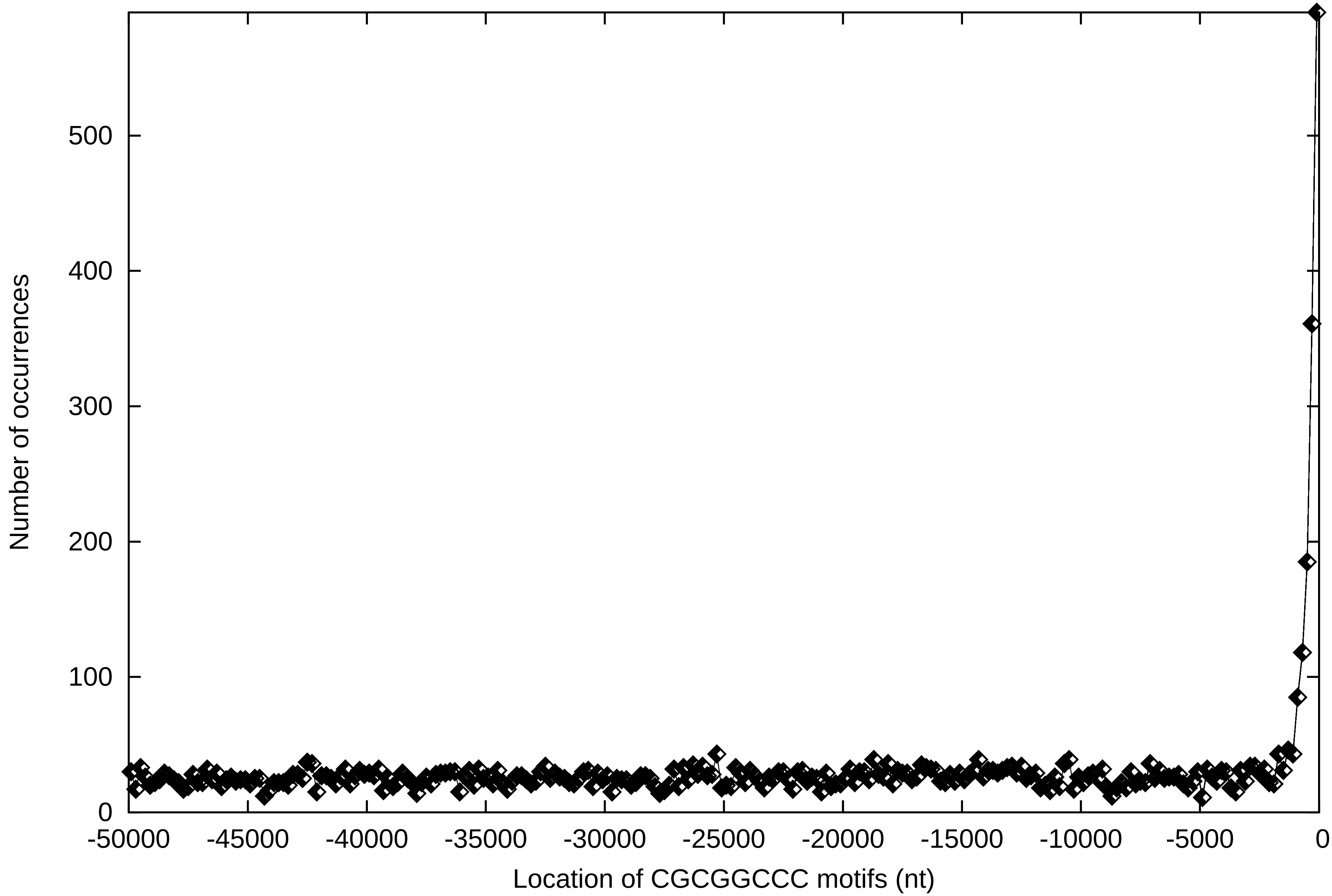

Number of occurrences
0
100
200
300
400
500
-50000
-45000
-40000
-35000
-30000
-25000
-20000
-15000
-10000
-5000
0
Location of CGCGGCCC motifs (nt)

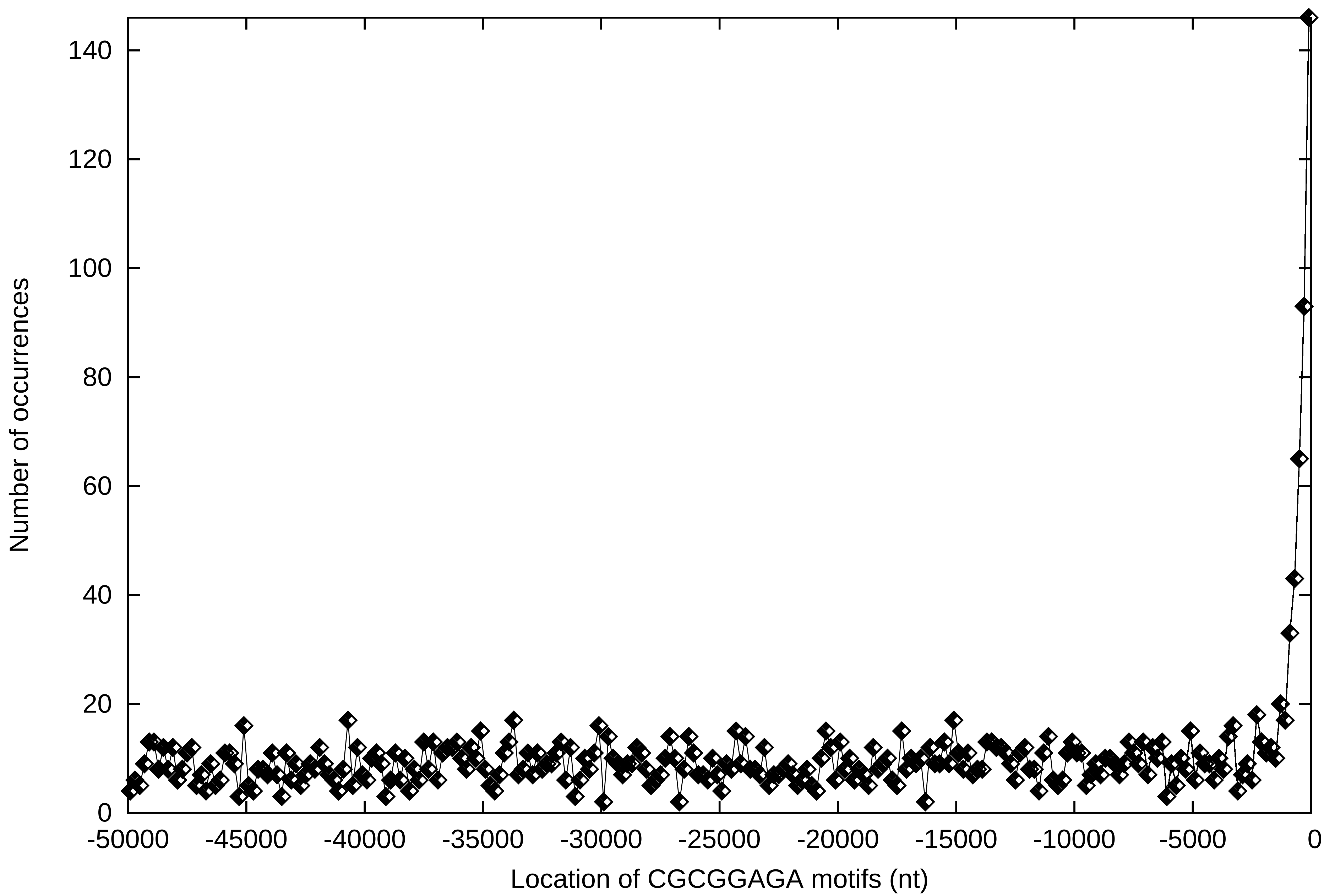

Number of occurrences
140
120
100
80
60
40
20
0
-50000
-45000
-40000
-35000
-30000
-25000
-20000
-15000
-10000
-5000
0
Location of CGCGGAGA motifs (nt)

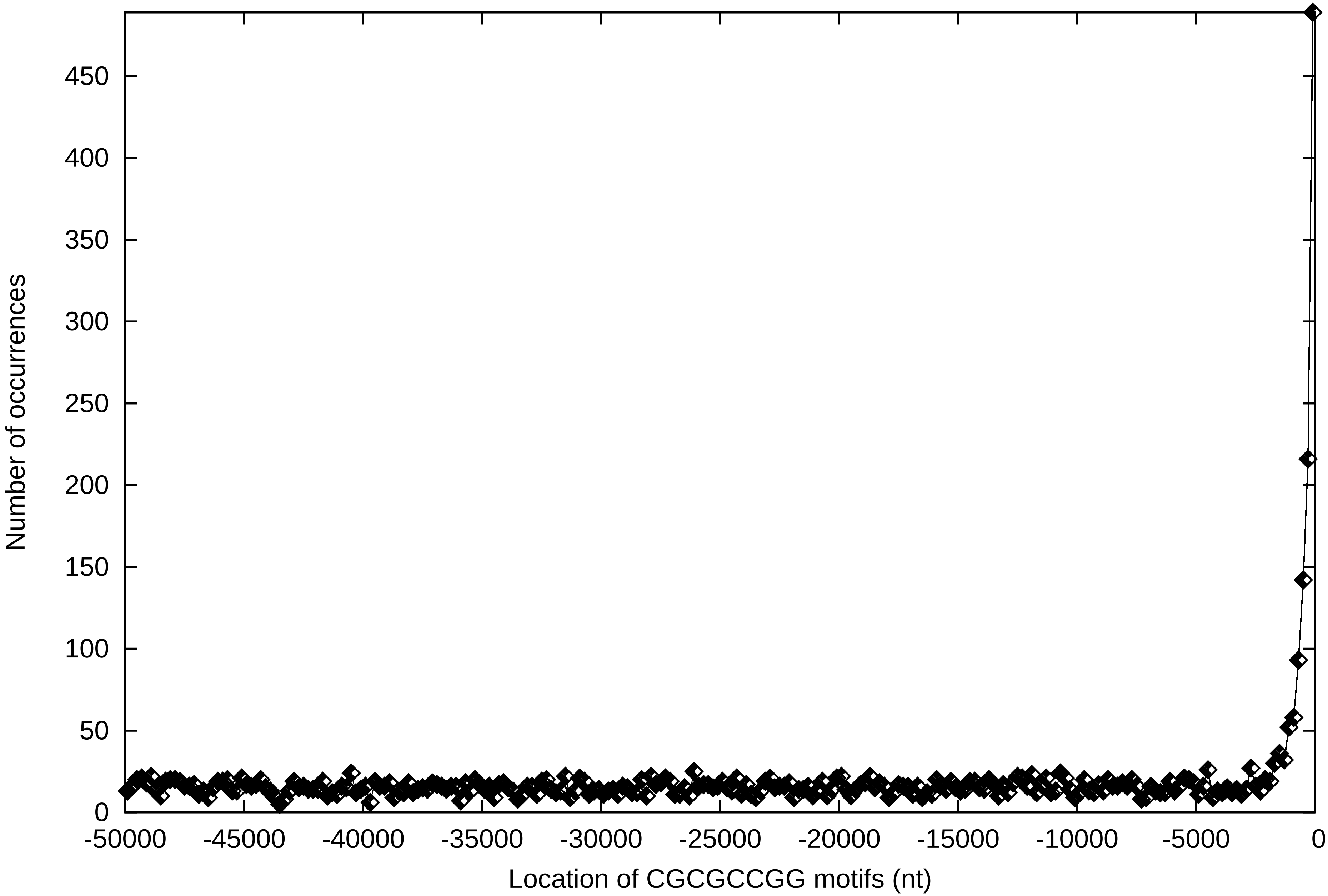

Number of occurrences
0
50
100
150
200
250
300
350
400
450
-50000
-45000
-40000
-35000
-30000
-25000
-20000
-15000
-10000
-5000
0
Location of CGCGCCGG motifs (nt)

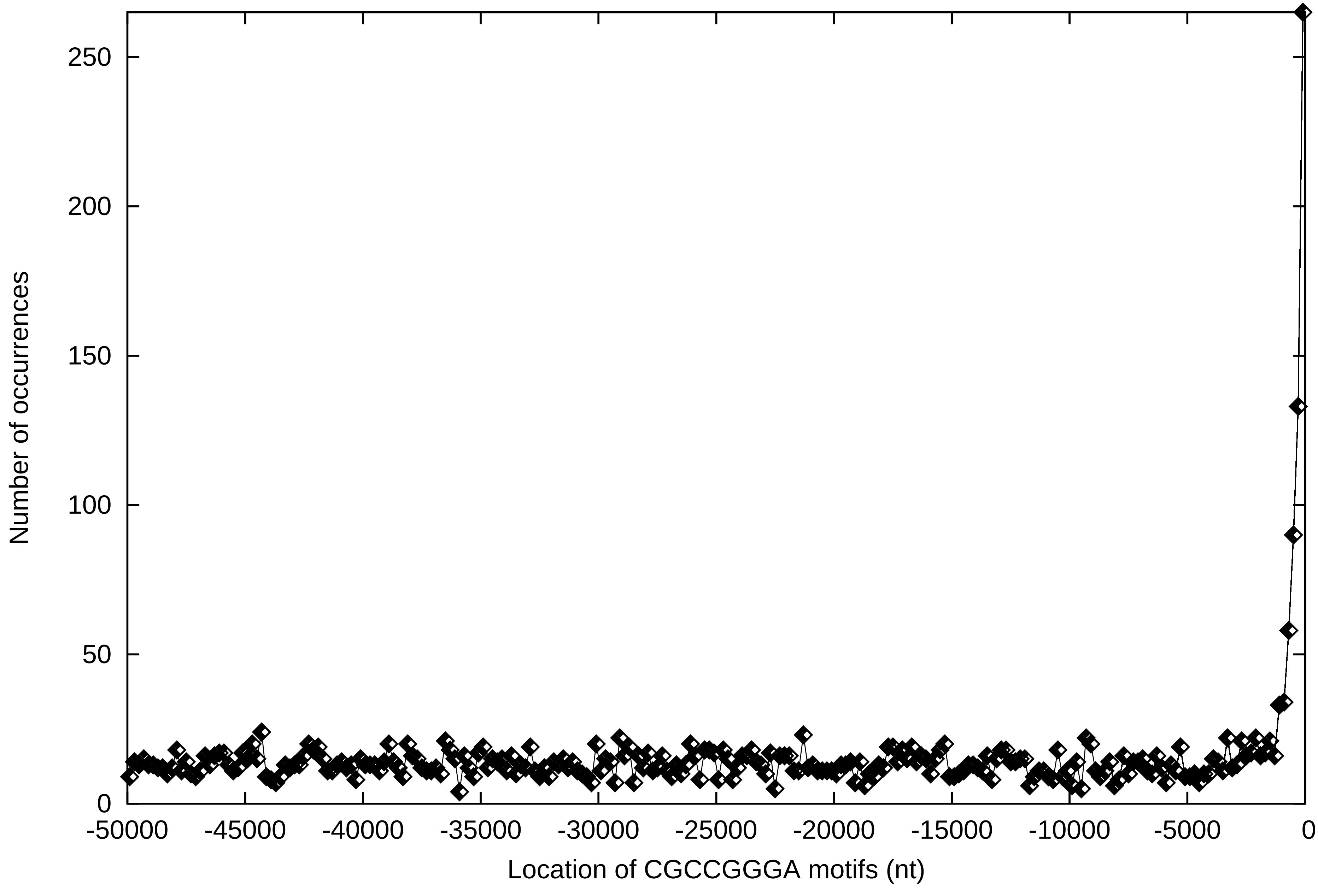
Number of occurrences
Location of CGCCGGGA motifs (nt)
0
50
100
150
200
250
-50000
-45000
-40000
-35000
-30000
-25000
-20000
-15000
-10000
-5000
0

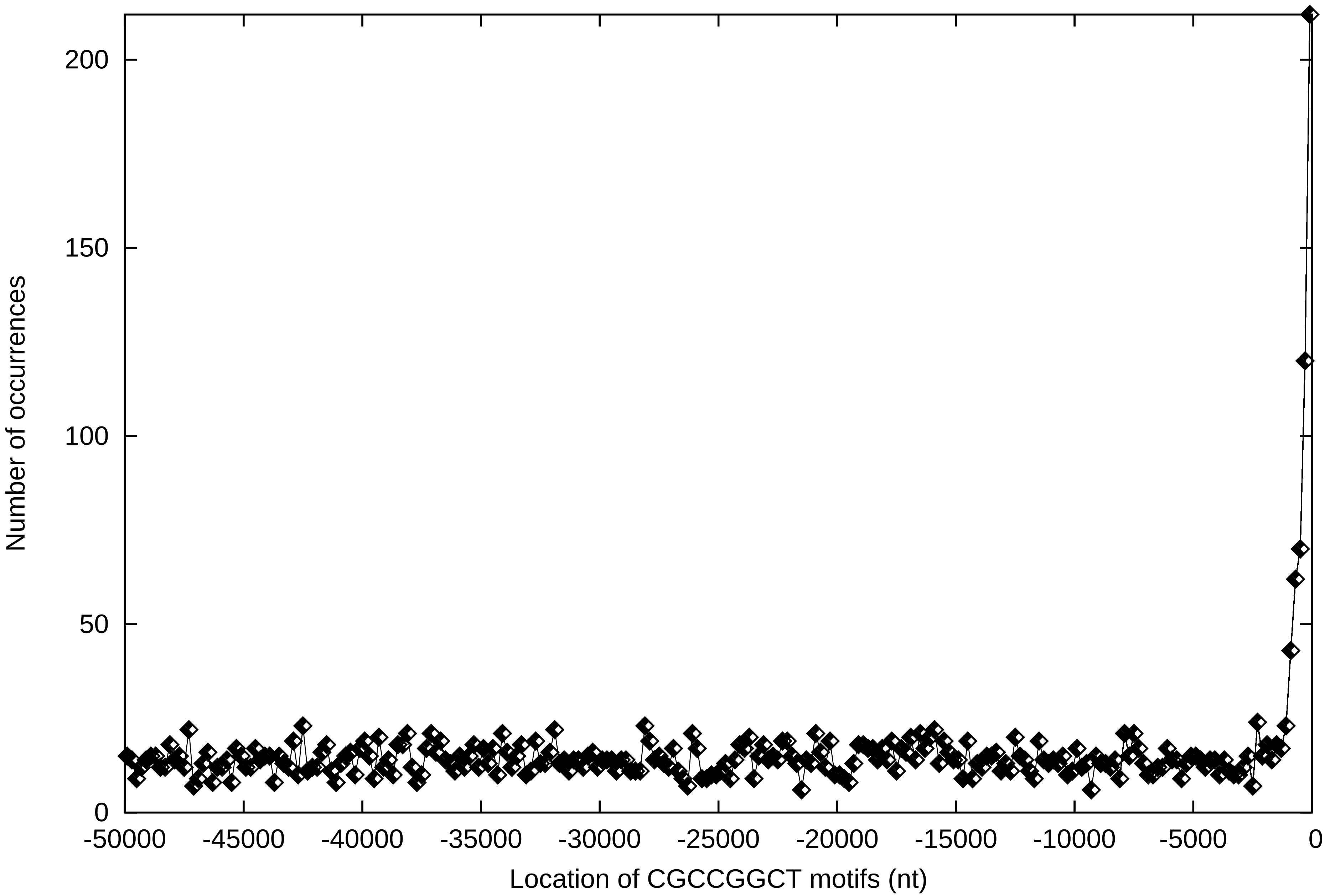

Number of occurrences
0
50
100
150
200
-50000
-45000
-40000
-35000
-30000
-25000
-20000
-15000
-10000
-5000
0
Location of CGCCGGCT motifs (nt)

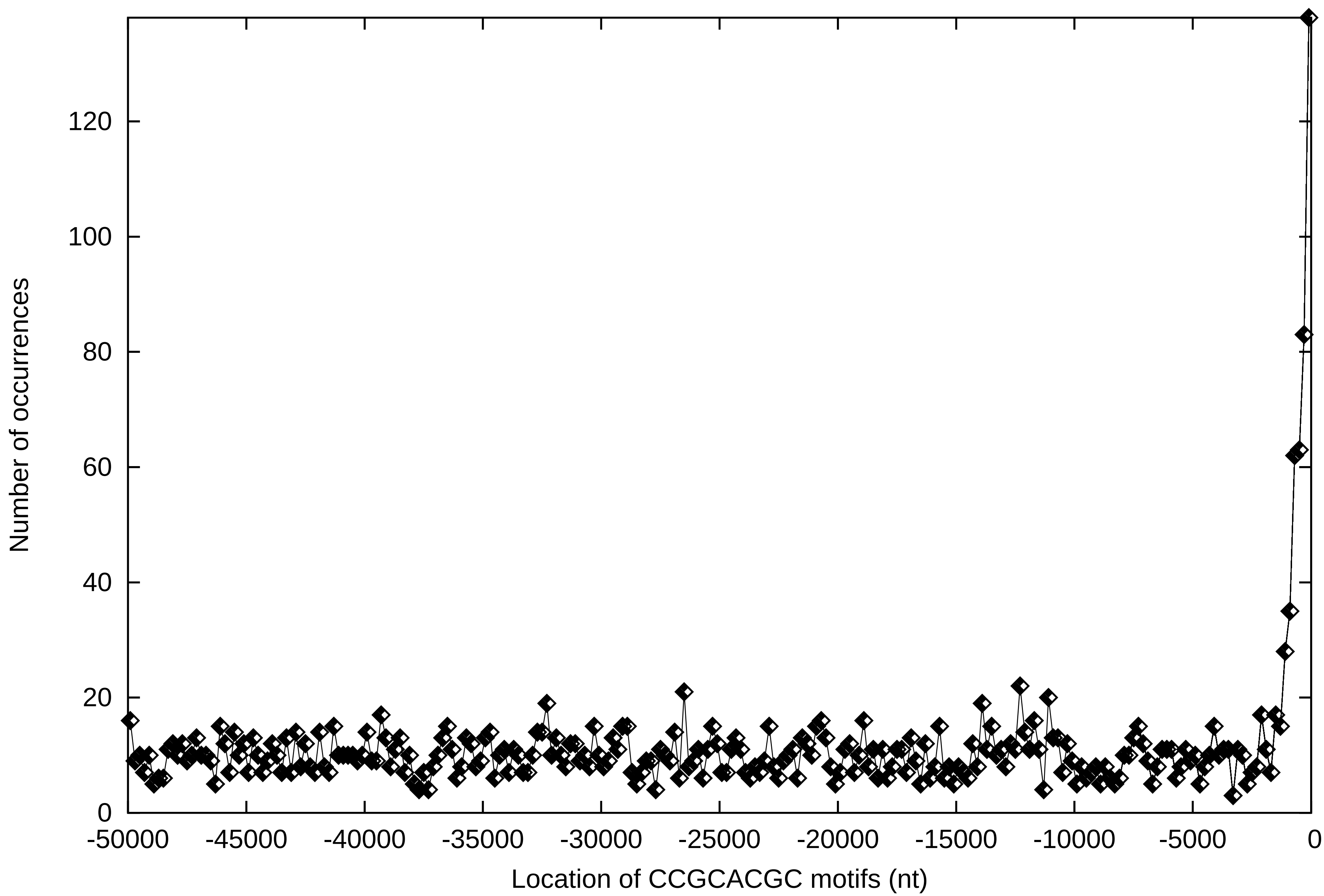

Number of occurrences
0
20
40
60
80
100
120
-50000
-45000
-40000
-35000
-30000
-25000
-20000
-15000
-10000
-5000
0
Location of CCGCACGC motifs (nt)

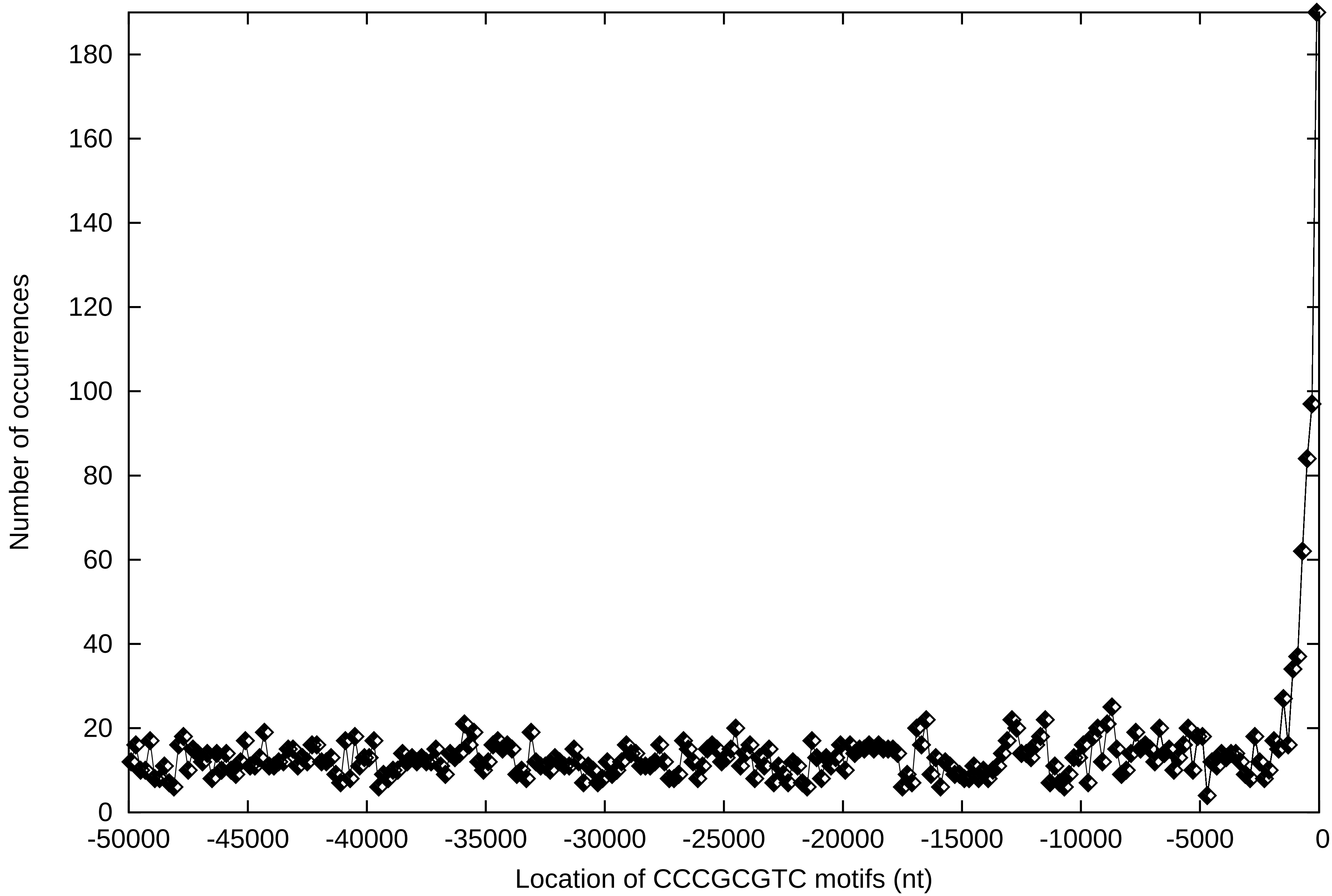

Number of occurrences
Location of CCCGCGTC motifs (nt)
0
20
40
60
80
100
120
140
160
180
-50000
-45000
-40000
-35000
-30000
-25000
-20000
-15000
-10000
-5000
0

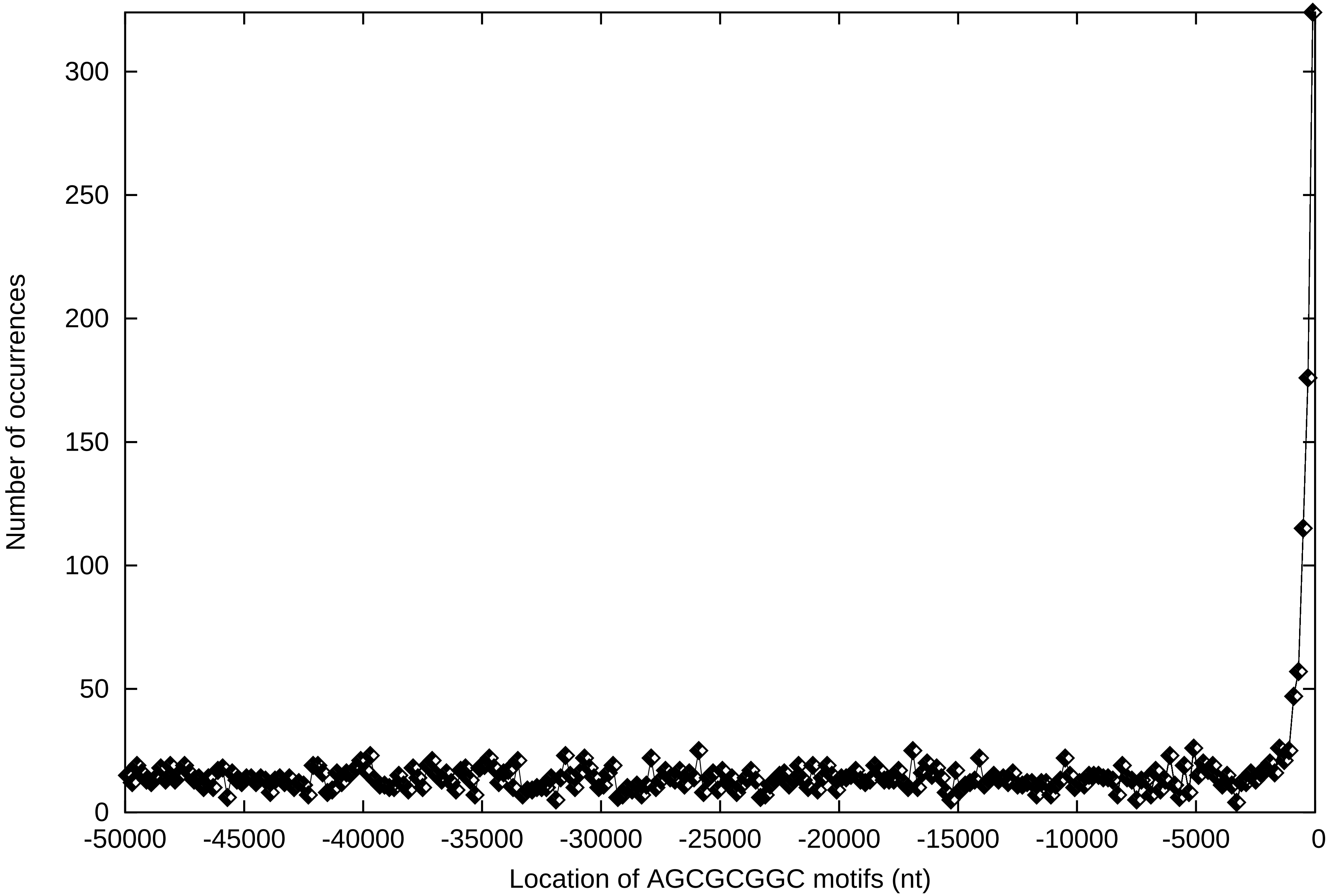

300
250
200
150
100
50
0
-50000
-45000
-40000
-35000
-30000
-25000
-20000
-15000
-10000
-5000
0
Number of occurrences
Location of AGCGCGGC motifs (nt)

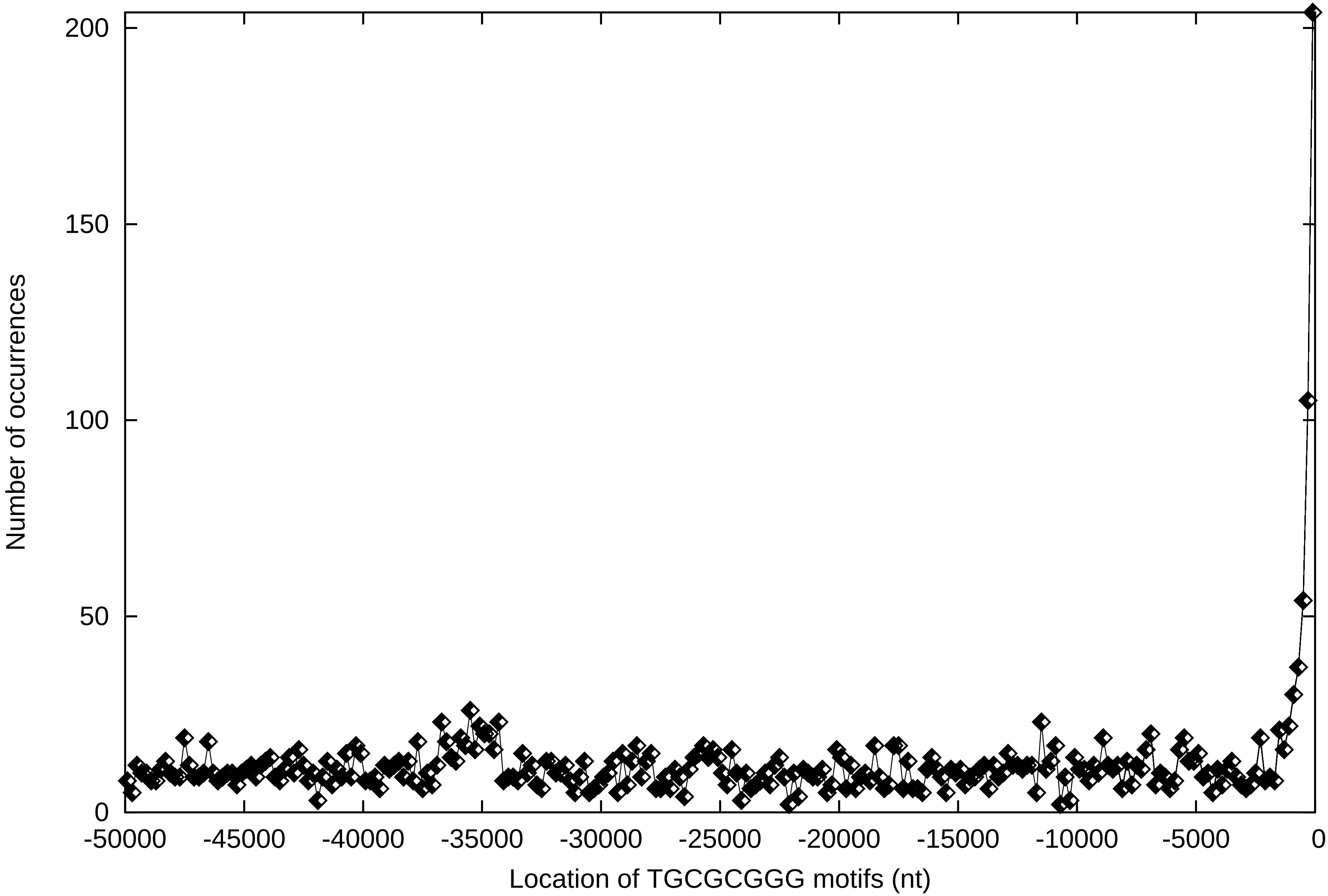

200
150
100
50
0
-50000
-45000
-40000
-35000
-30000
-25000
-20000
-15000
-10000
-5000
0
Number of occurrences
Location of TGCGCGGG motifs (nt)

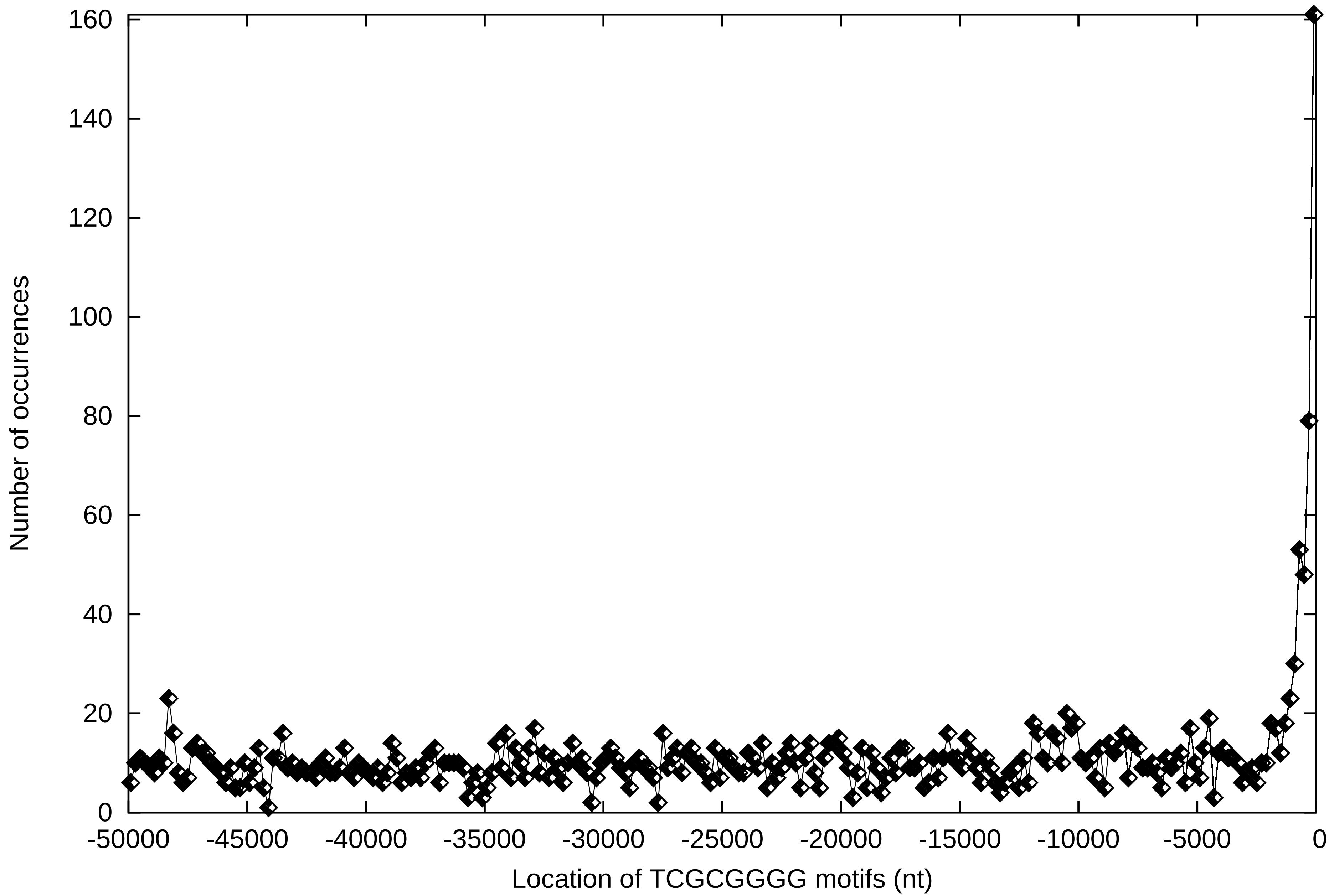

0
20
40
60
80
100
120
140
160
Number of occurrences
-50000
-45000
-40000
-35000
-30000
-25000
-20000
-15000
-10000
-5000
0
Location of TCGCGGGG motifs (nt)

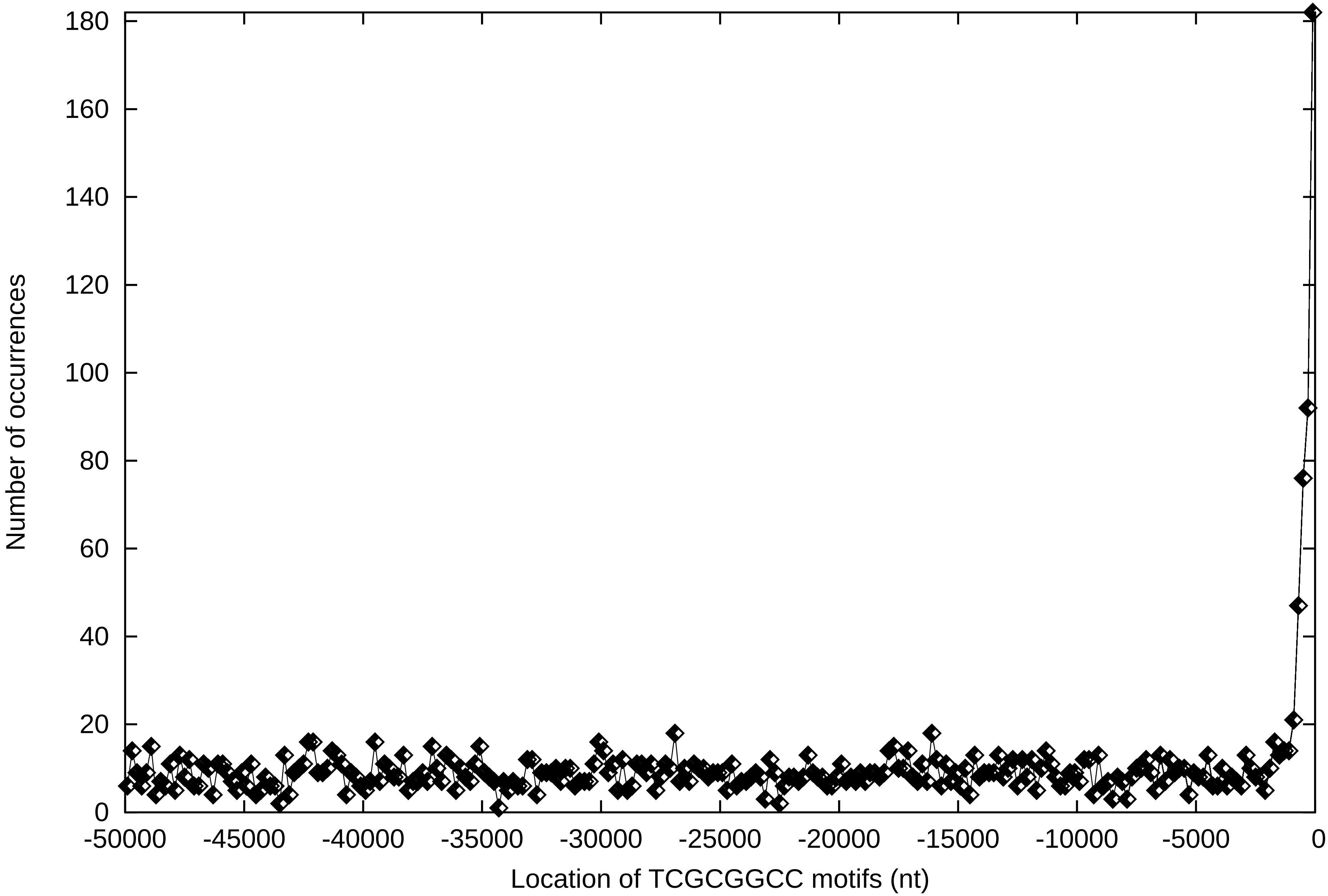

180
160
140
120
100
80
60
40
20
0
-50000
-45000
-40000
-35000
-30000
-25000
-20000
-15000
-10000
-5000
0
Number of occurrences
Location of TCGCGGCC motifs (nt)

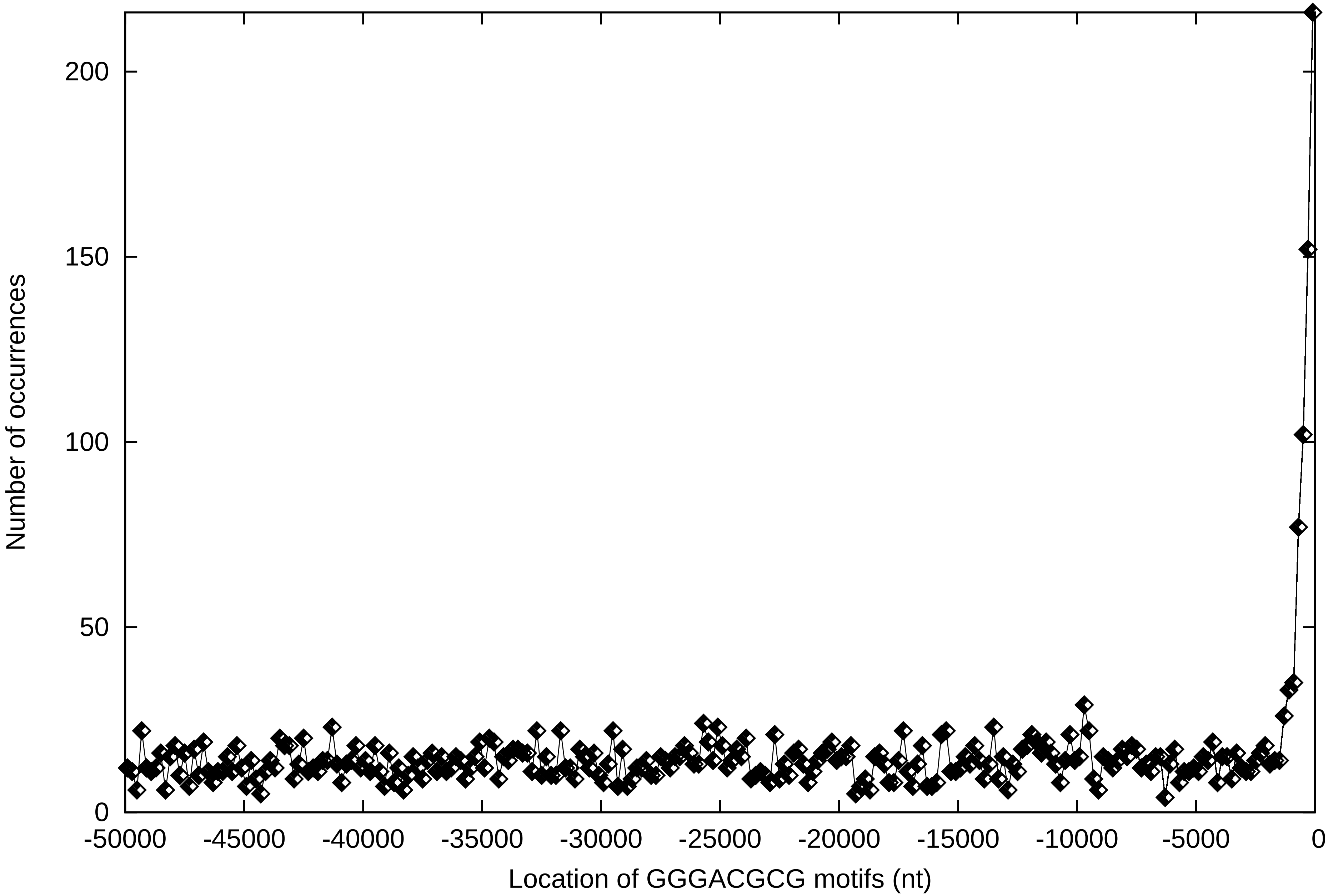

Number of occurrences
200
150
100
50
0
-50000
-45000
-40000
-35000
-30000
-25000
-20000
-15000
-10000
-5000
0
Location of GGGACGCG motifs (nt)

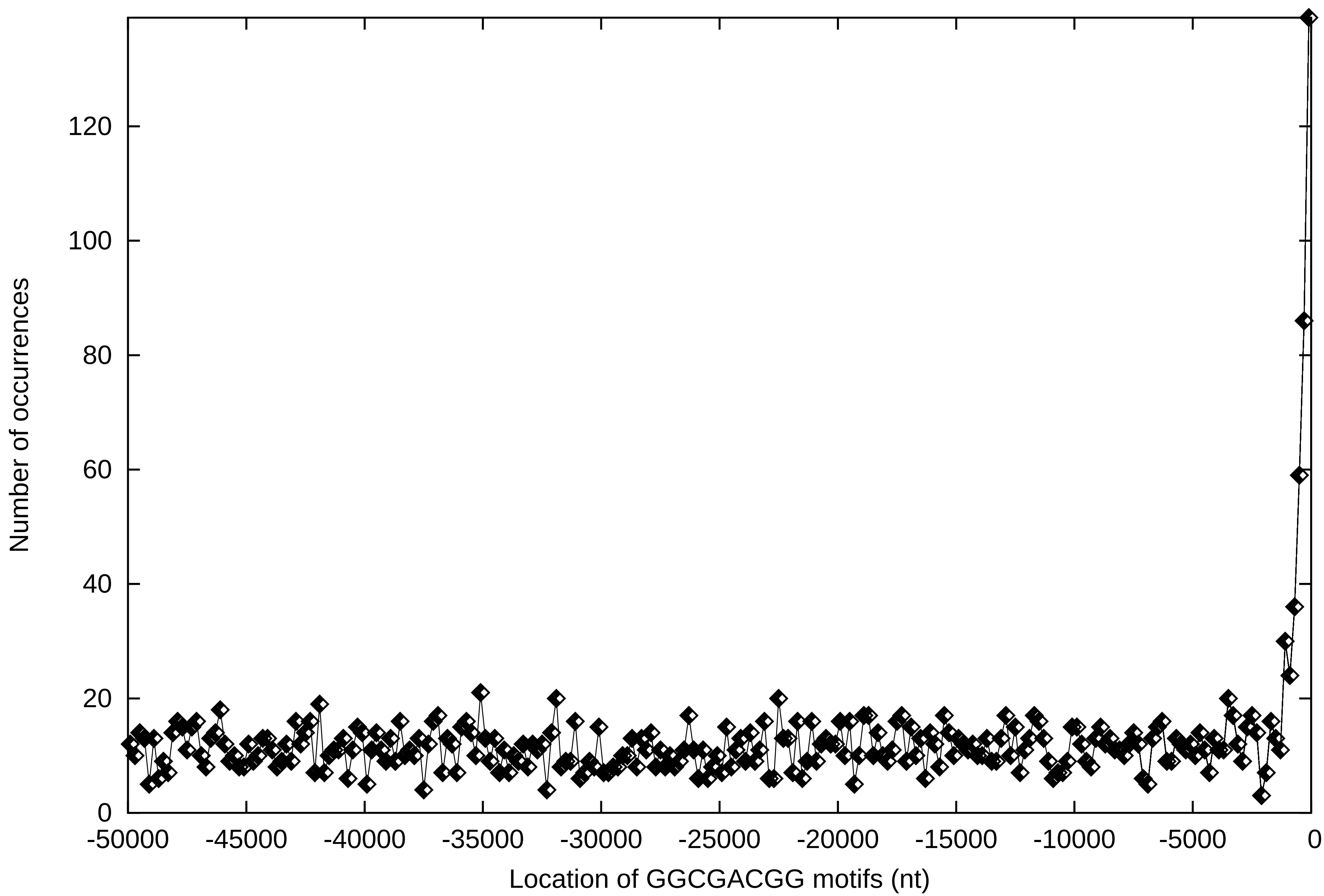

Number of occurrences
0
20
40
60
80
100
120
-50000
-45000
-40000
-35000
-30000
-25000
-20000
-15000
-10000
-5000
0
Location of GGCGACGG motifs (nt)

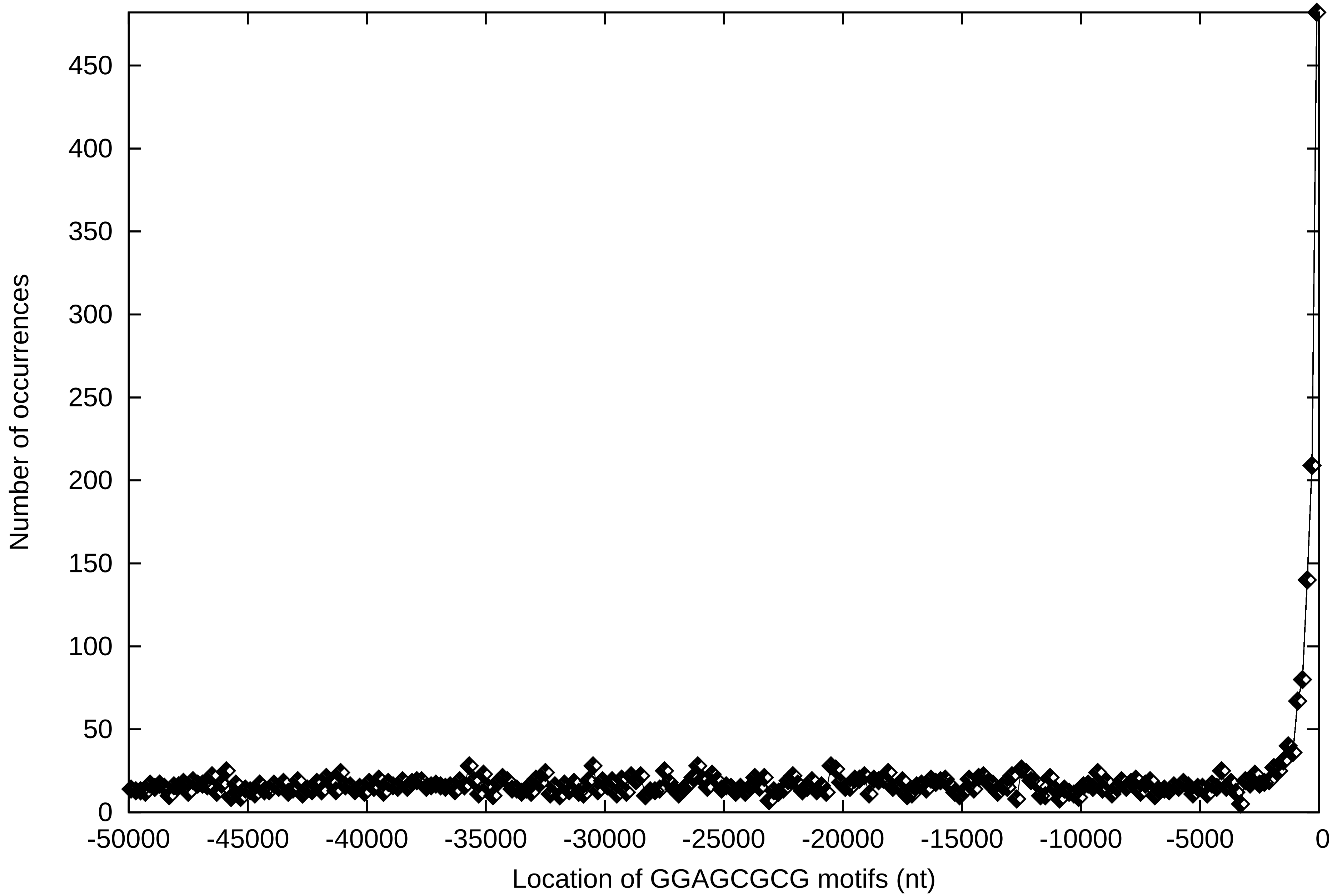

Number of occurrences
0
50
100
150
200
250
300
350
400
450
-50000
-45000
-40000
-35000
-30000
-25000
-20000
-15000
-10000
-5000
0
Location of GGAGCGCG motifs (nt)

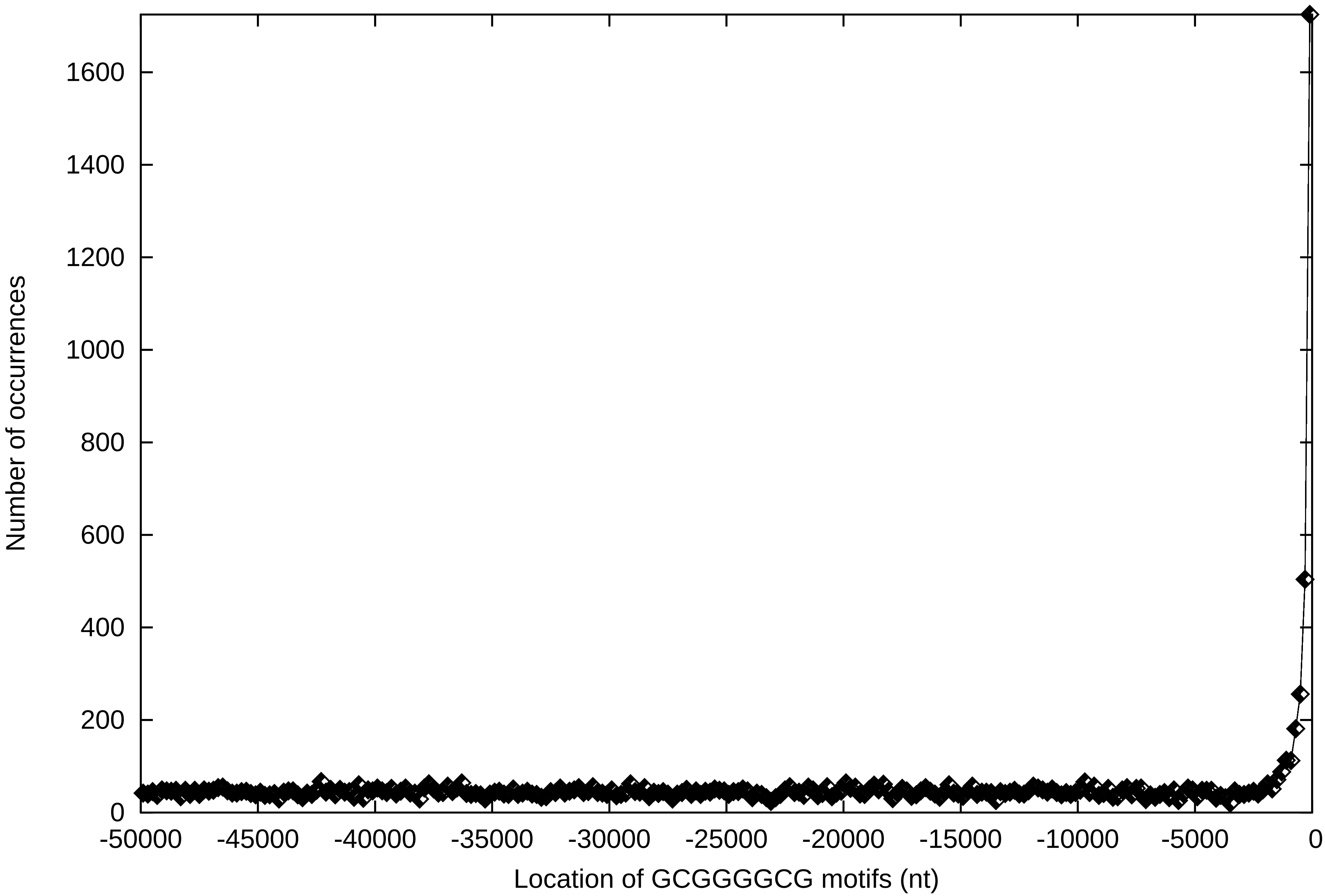

Number of occurrences
0
200
400
600
800
1000
1200
1400
1600
-50000
-45000
-40000
-35000
-30000
-25000
-20000
-15000
-10000
-5000
0
Location of GCGGGGCG motifs (nt)

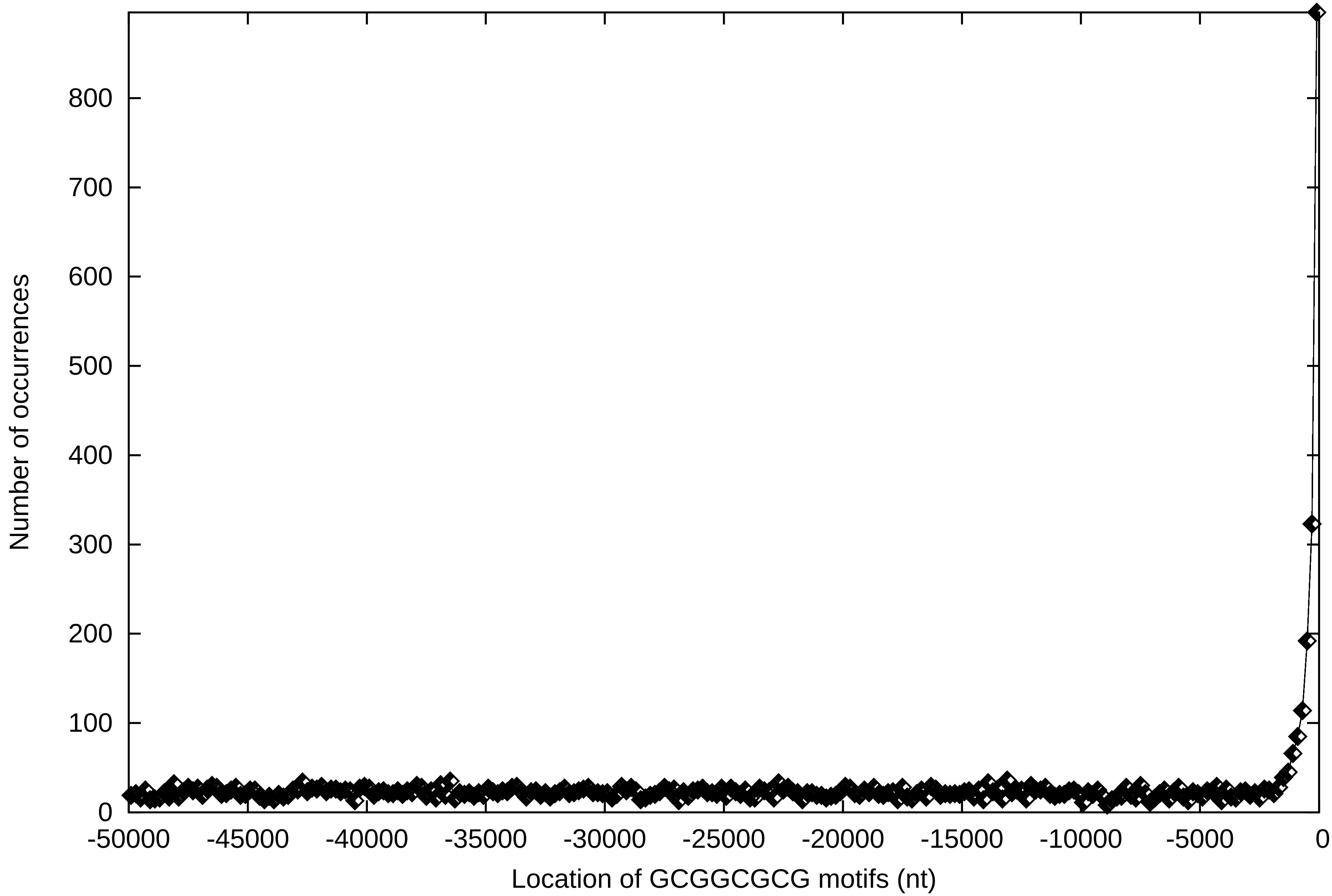
Number of occurrences
0
100
200
300
400
500
600
700
800
-50000
-45000
-40000
-35000
-30000
-25000
-20000
-15000
-10000
-5000
0
Location of GCGGCGCG motifs (nt)

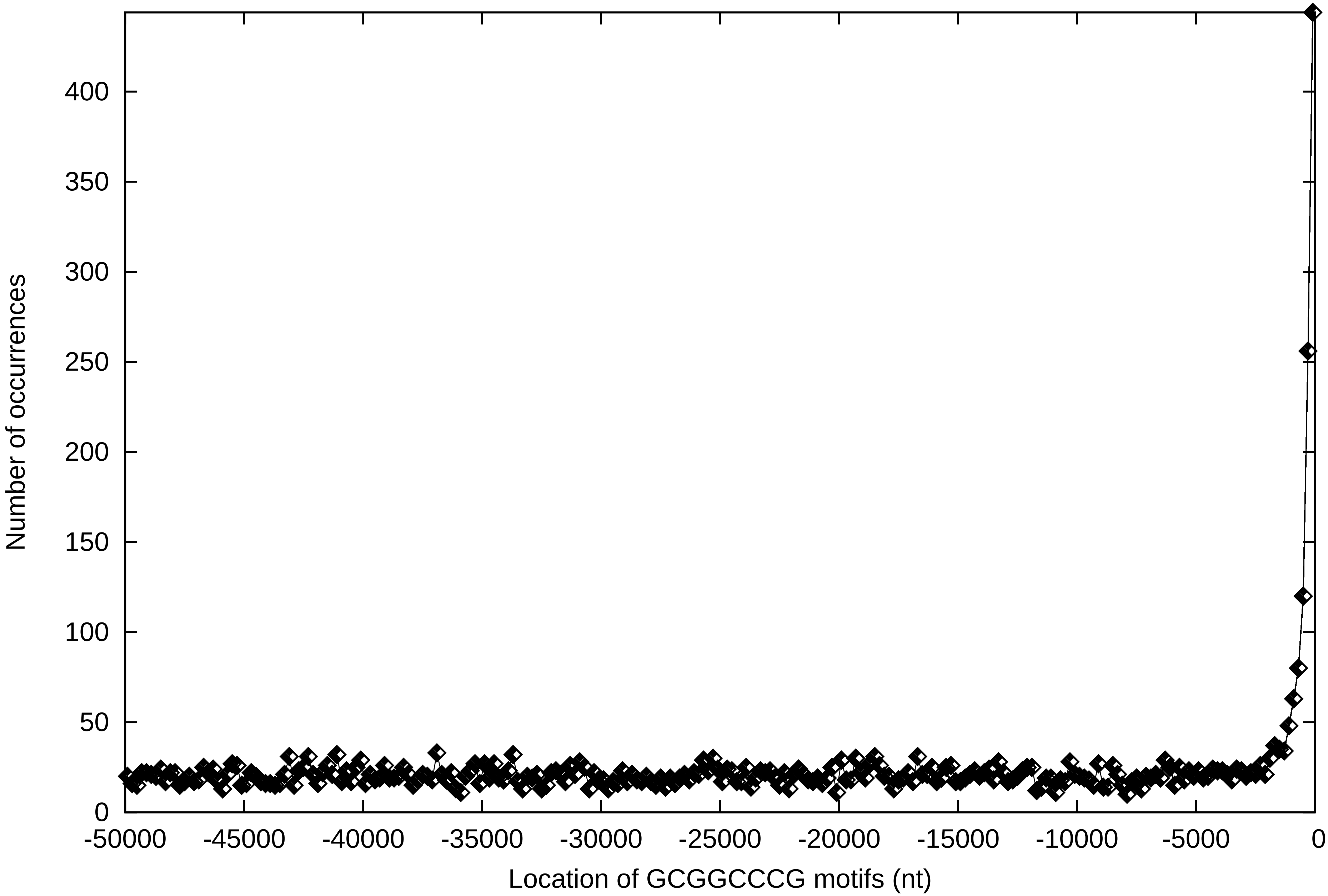

Number of occurrences
0
50
100
150
200
250
300
350
400
-50000
-45000
-40000
-35000
-30000
-25000
-20000
-15000
-10000
-5000
0
Location of GCGGCCCG motifs (nt)

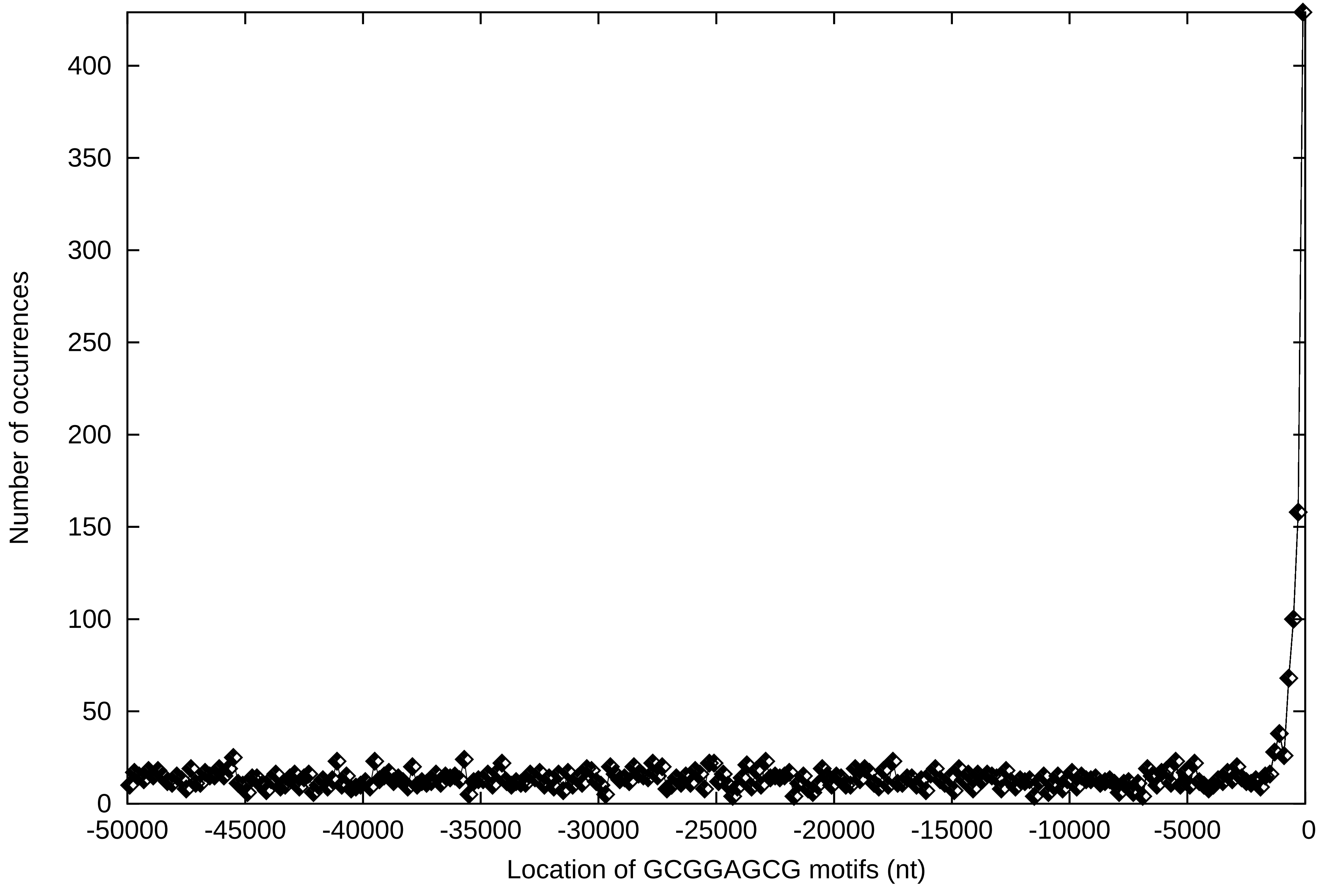

Number of occurrences
Location of GCGGAGCG motifs (nt)
0
50
100
150
200
250
300
350
400
-50000
-45000
-40000
-35000
-30000
-25000
-20000
-15000
-10000
-5000
0

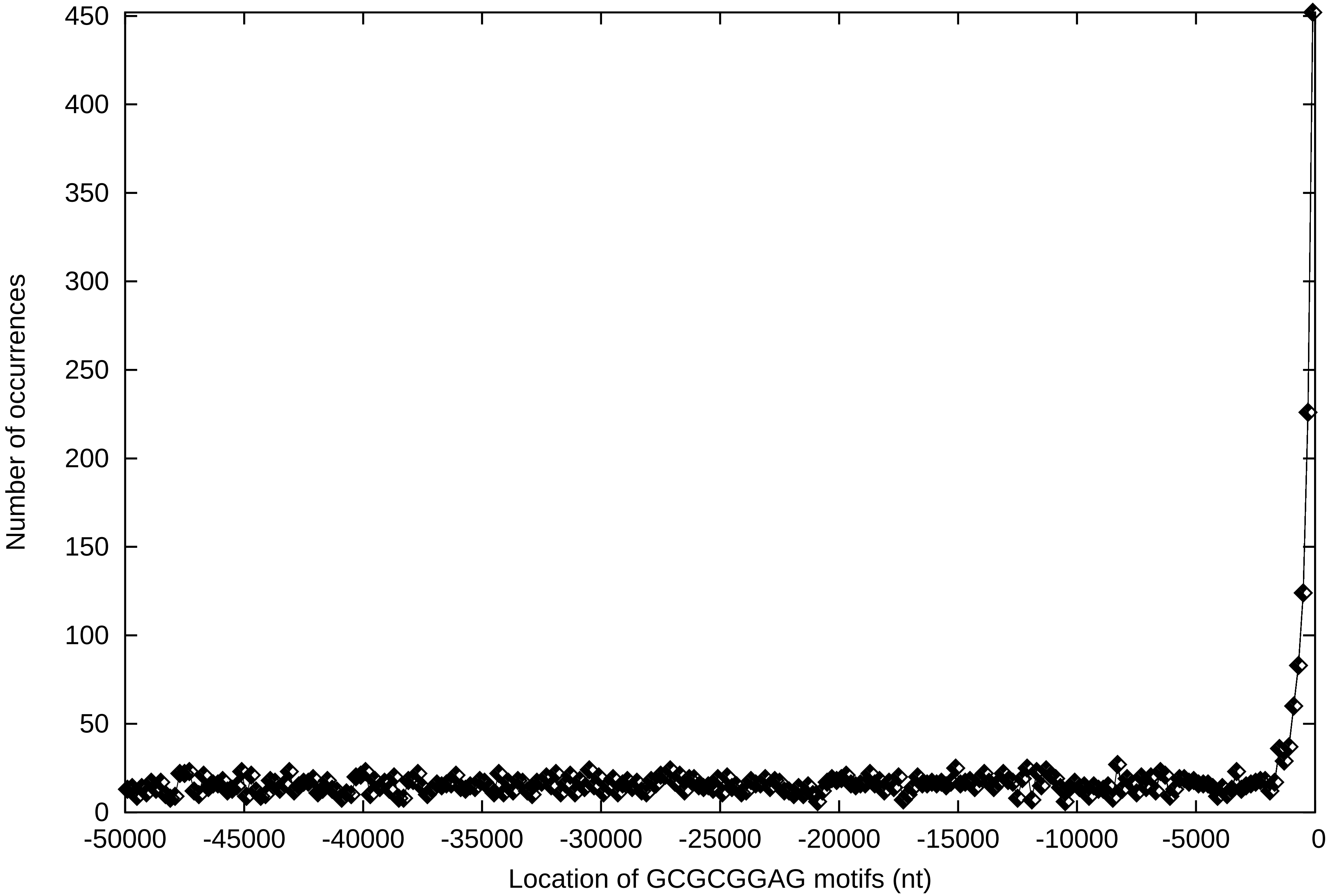

450
400
350
300
250
200
150
100
50
0
Number of occurrences
-50000
-45000
-40000
-35000
-30000
-25000
-20000
-15000
-10000
-5000
0
Location of GCGCGGAG motifs (nt)

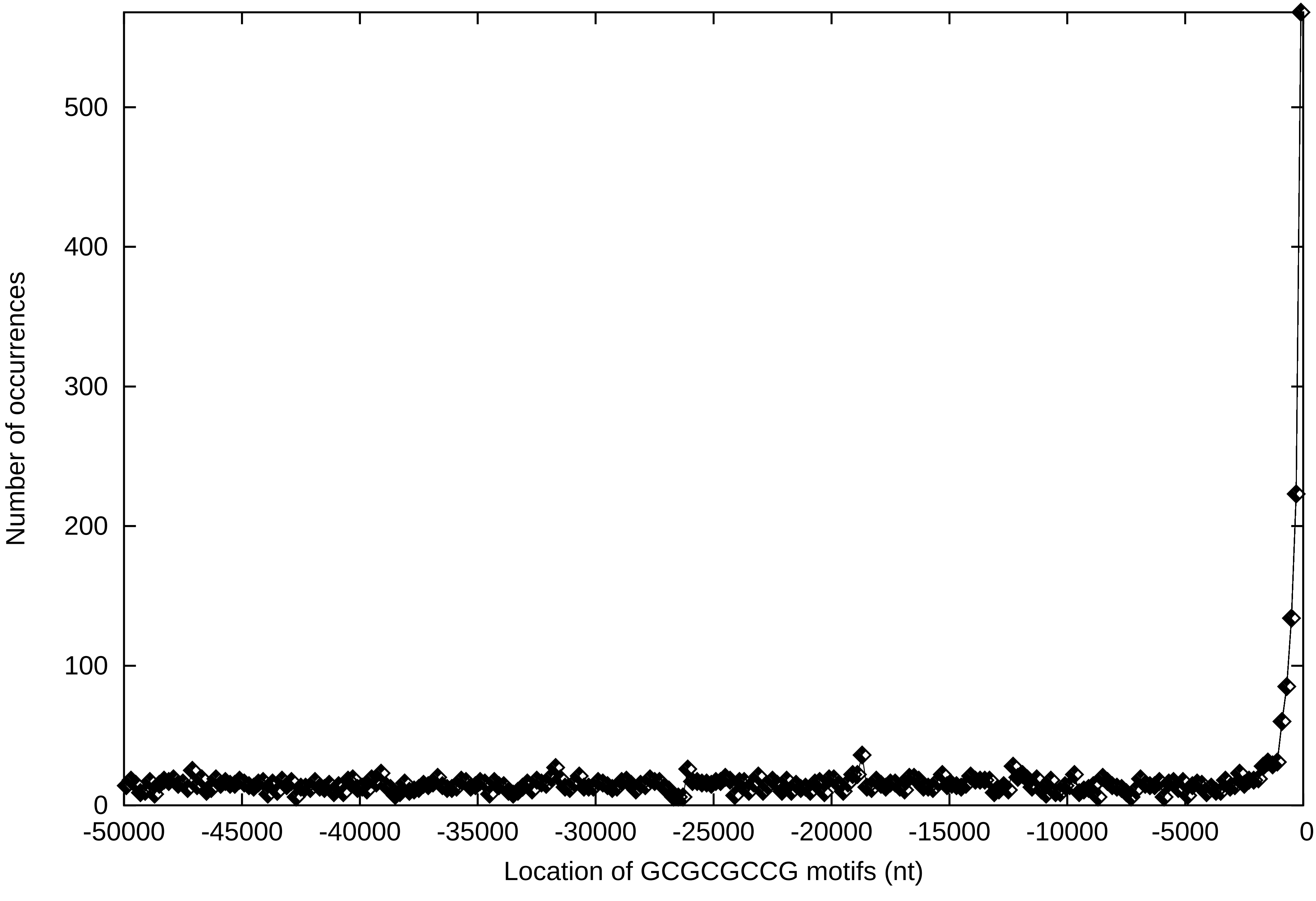

Number of occurrences
0
100
200
300
400
500
-50000
-45000
-40000
-35000
-30000
-25000
-20000
-15000
-10000
-5000
0
Location of GCGCGCCG motifs (nt)

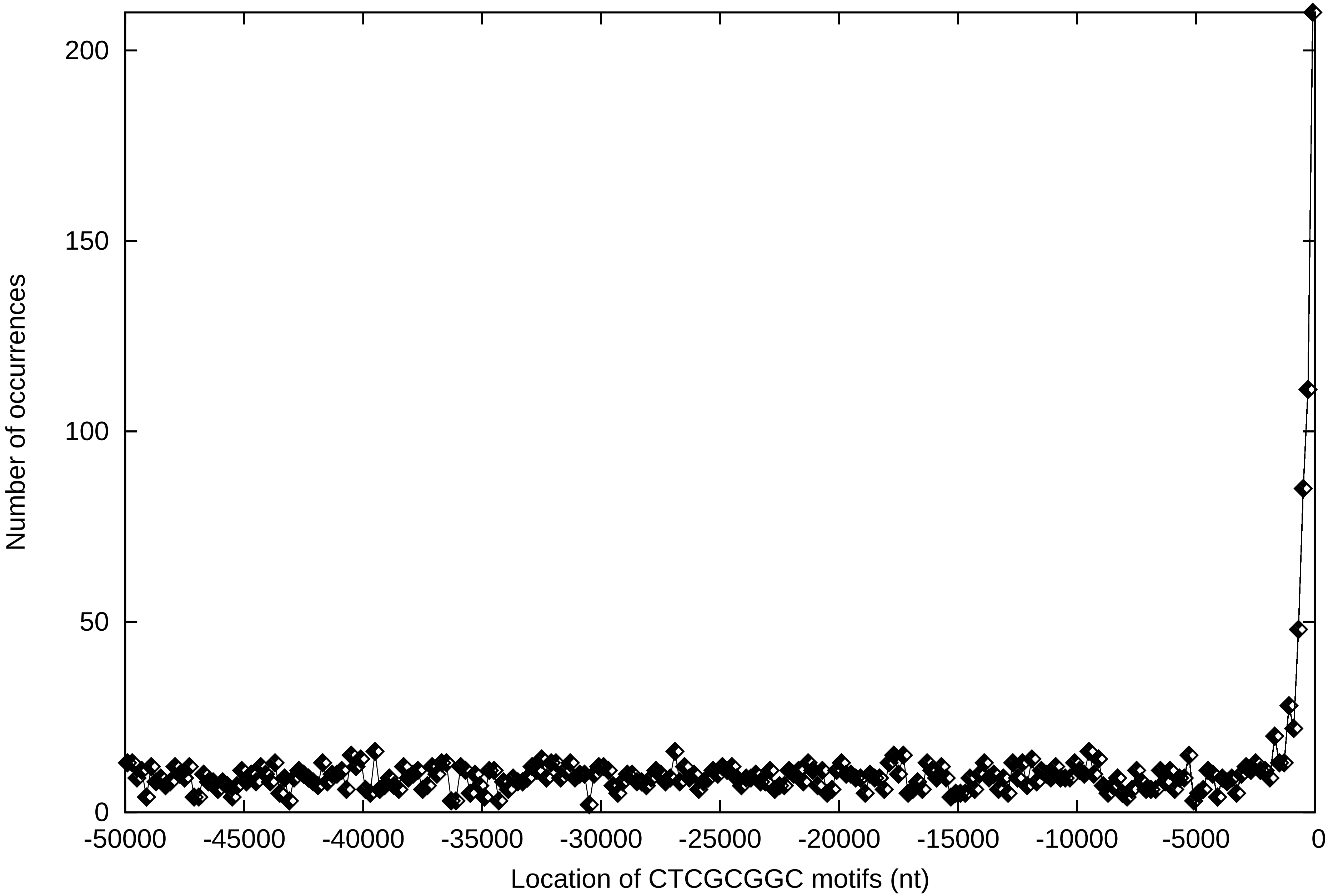

Number of occurrences
0
50
100
150
200
-50000
-45000
-40000
-35000
-30000
-25000
-20000
-15000
-10000
-5000
0
Location of CTCGCGGC motifs (nt)

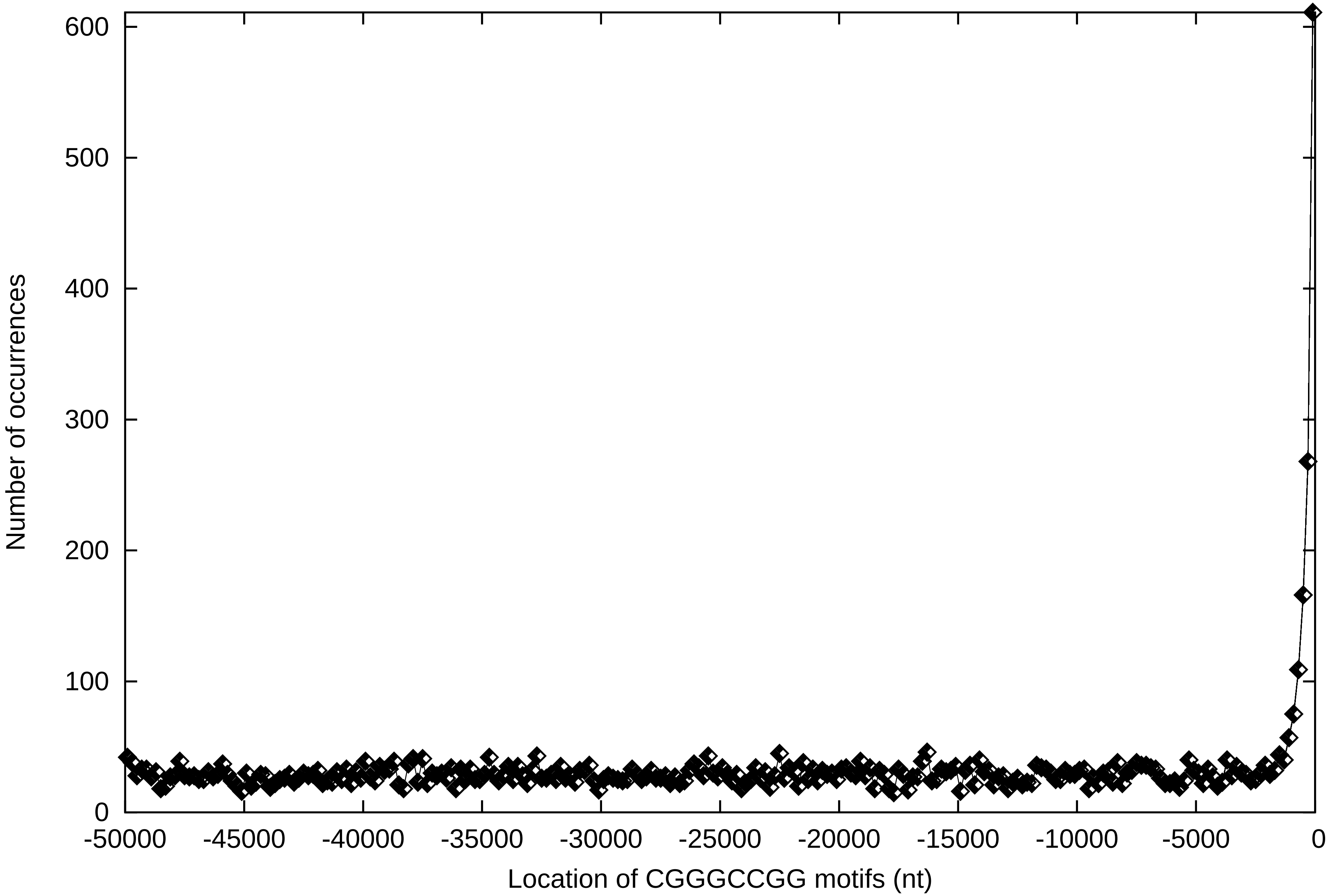

Number of occurrences
600
500
400
300
200
100
0
-50000
-45000
-40000
-35000
-30000
-25000
-20000
-15000
-10000
-5000
0
Location of CGGGCCGG motifs (nt)

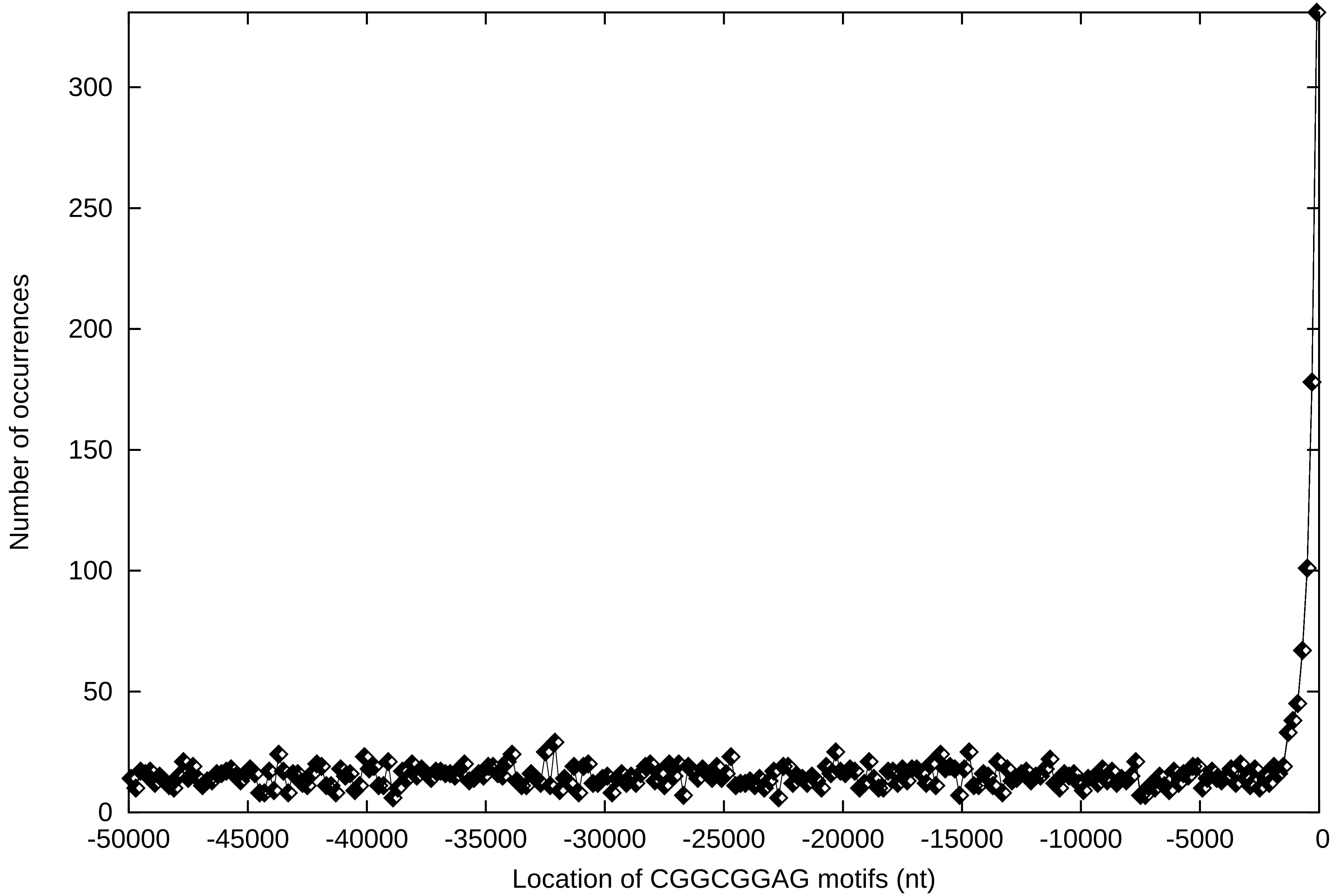

Number of occurrences
300
250
200
150
100
50
0
-50000
-45000
-40000
-35000
-30000
-25000
-20000
-15000
-10000
-5000
0
Location of CGGCGGAG motifs (nt)

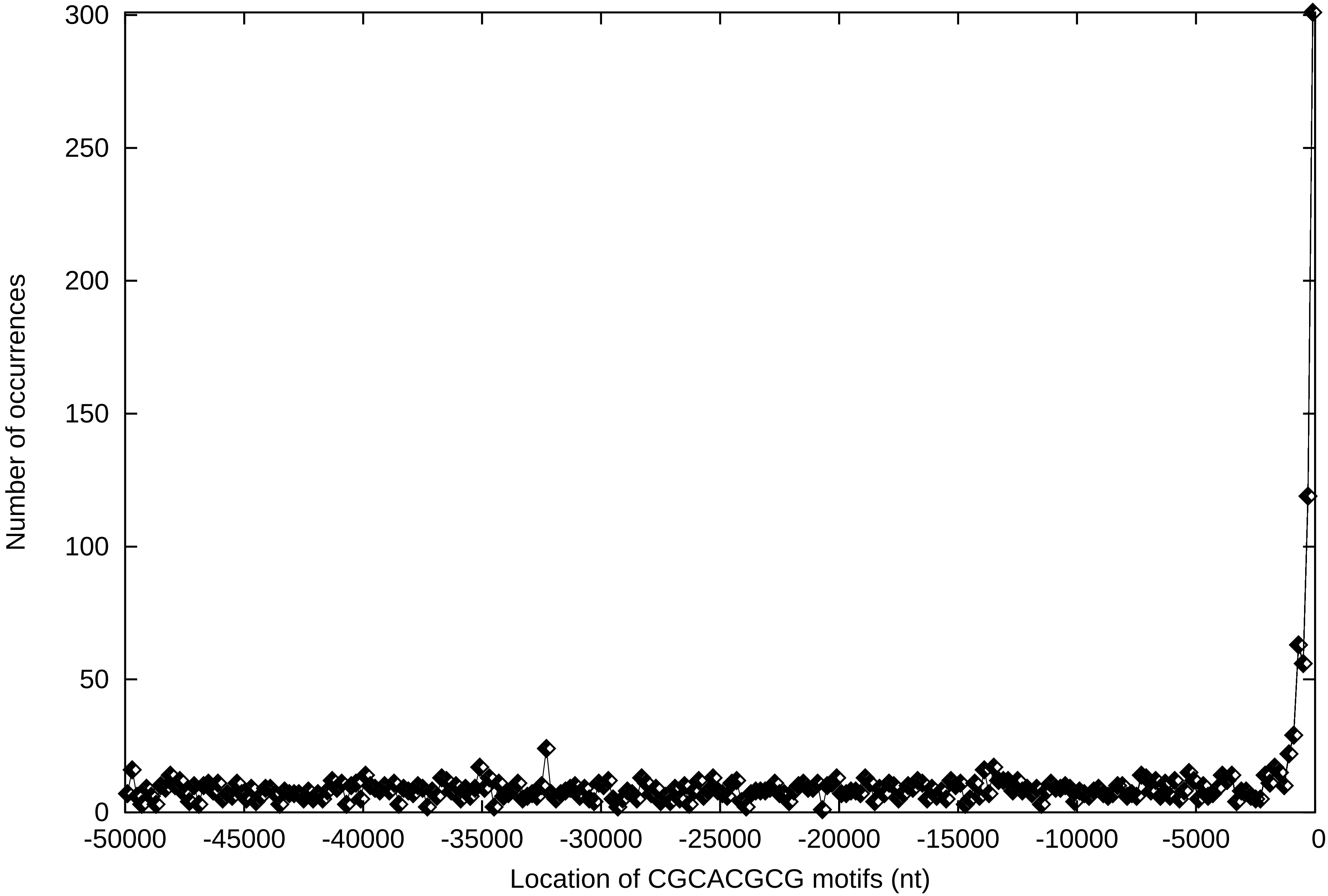

300
250
200
150
100
50
0
Number of occurrences
-50000
-45000
-40000
-35000
-30000
-25000
-20000
-15000
-10000
-5000
0
Location of CGCACGCG motifs (nt)

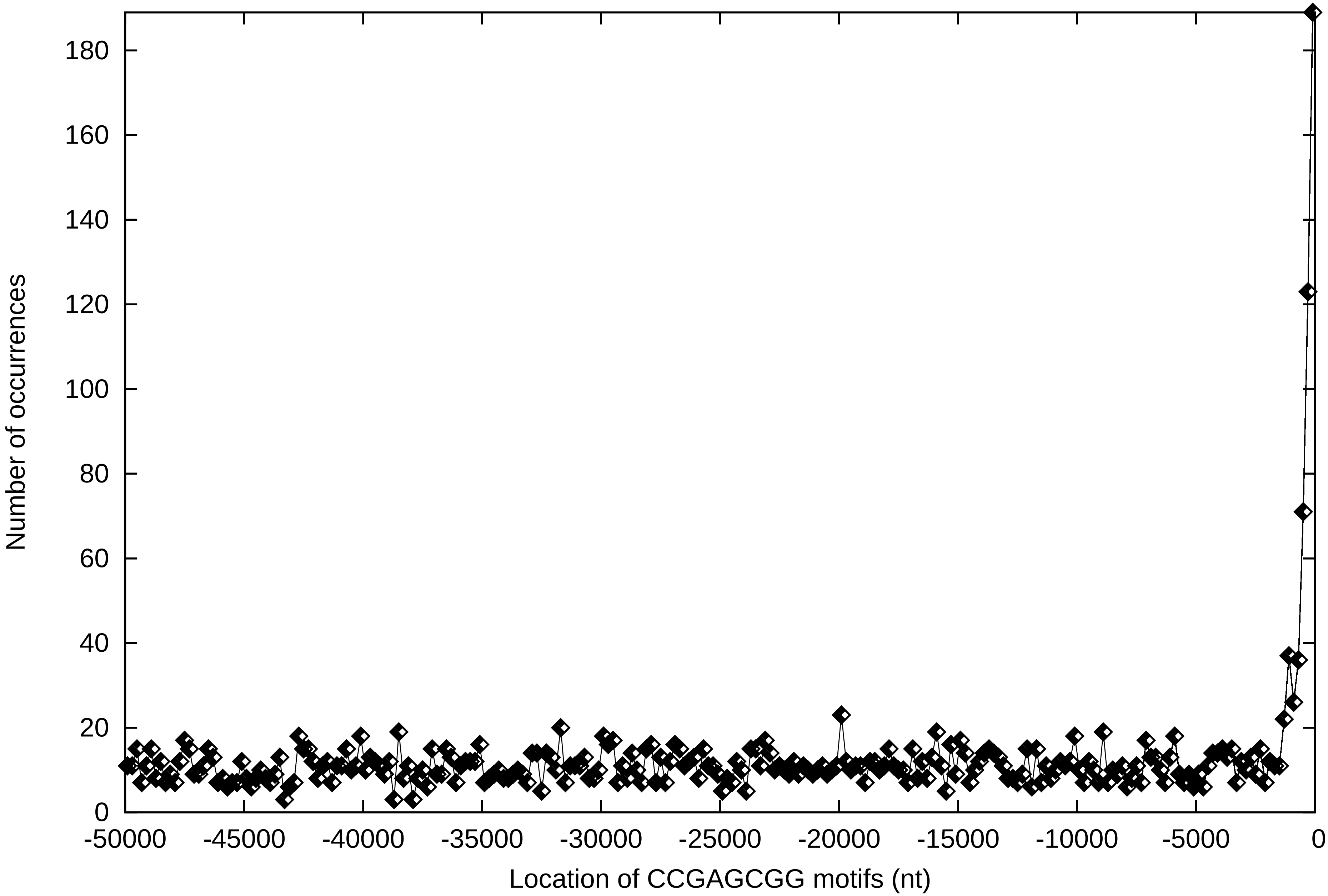

Number of occurrences
0
20
40
60
80
100
120
140
160
180
-50000
-45000
-40000
-35000
-30000
-25000
-20000
-15000
-10000
-5000
0
Location of CCGAGCGG motifs (nt)

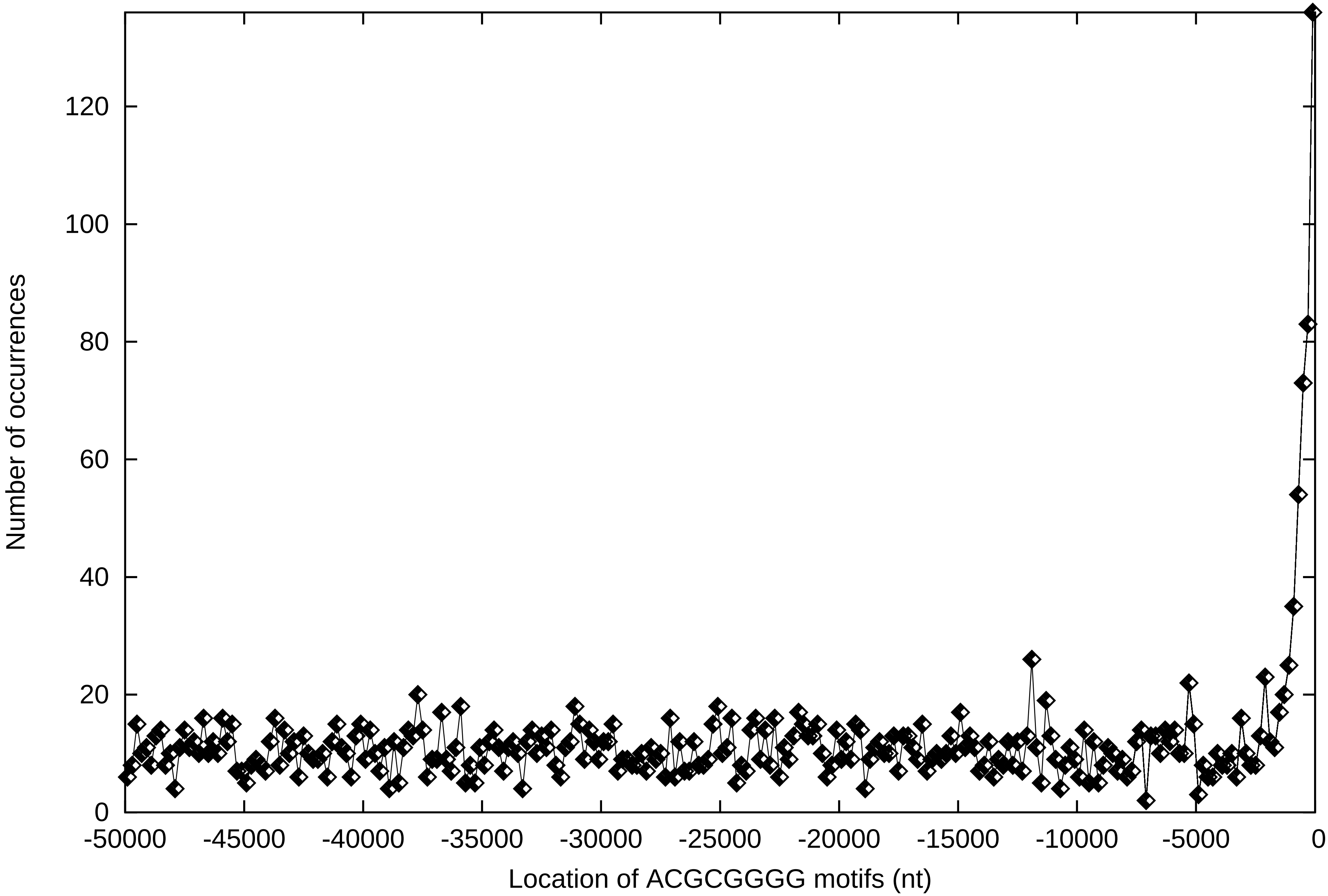

Number of occurrences
0
20
40
60
80
100
120
-50000
-45000
-40000
-35000
-30000
-25000
-20000
-15000
-10000
-5000
0
Location of ACGCGGGG motifs (nt)

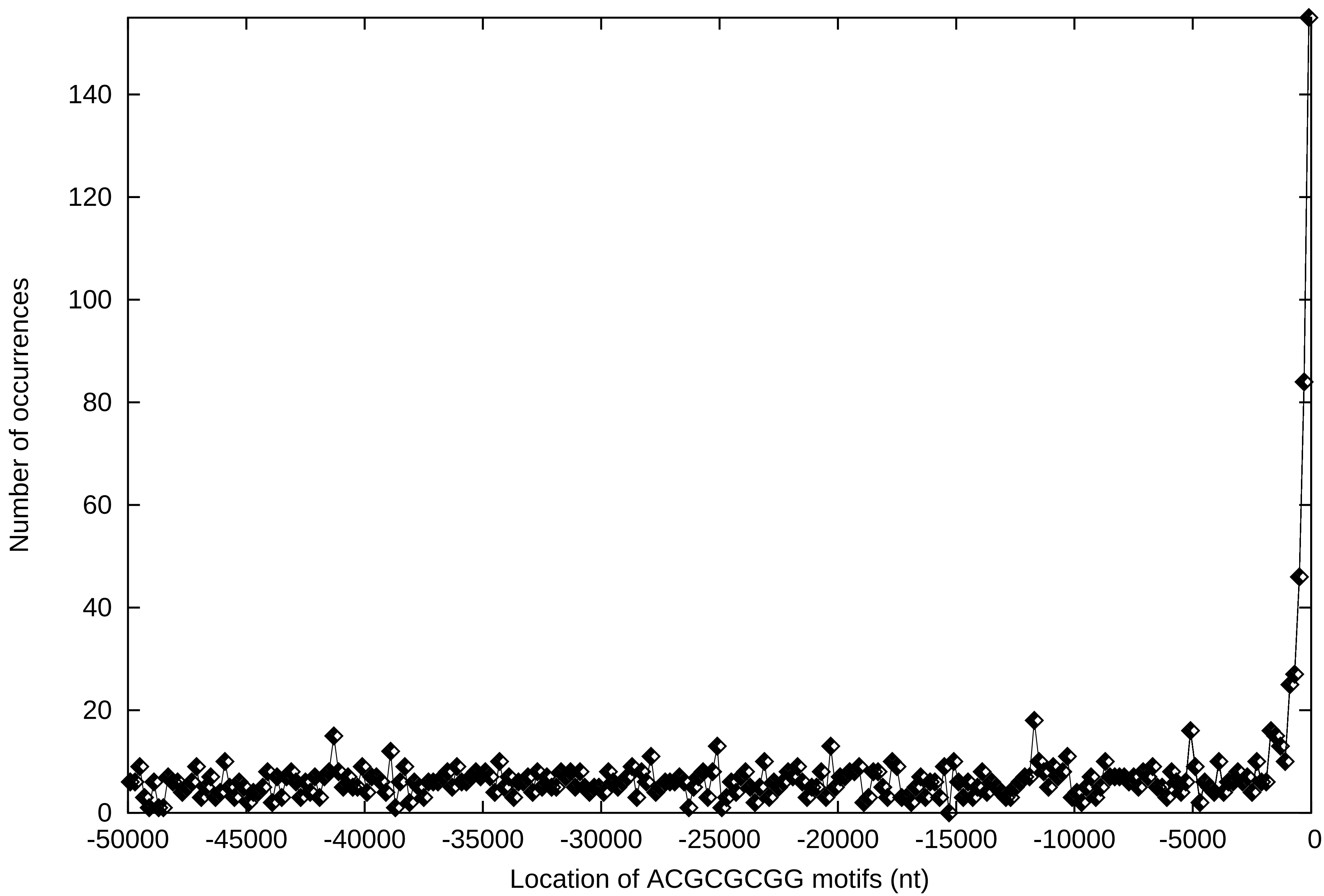

Number of occurrences
Location of ACGCGCGG motifs (nt)
0
20
40
60
80
100
120
140
-50000
-45000
-40000
-35000
-30000
-25000
-20000
-15000
-10000
-5000
0

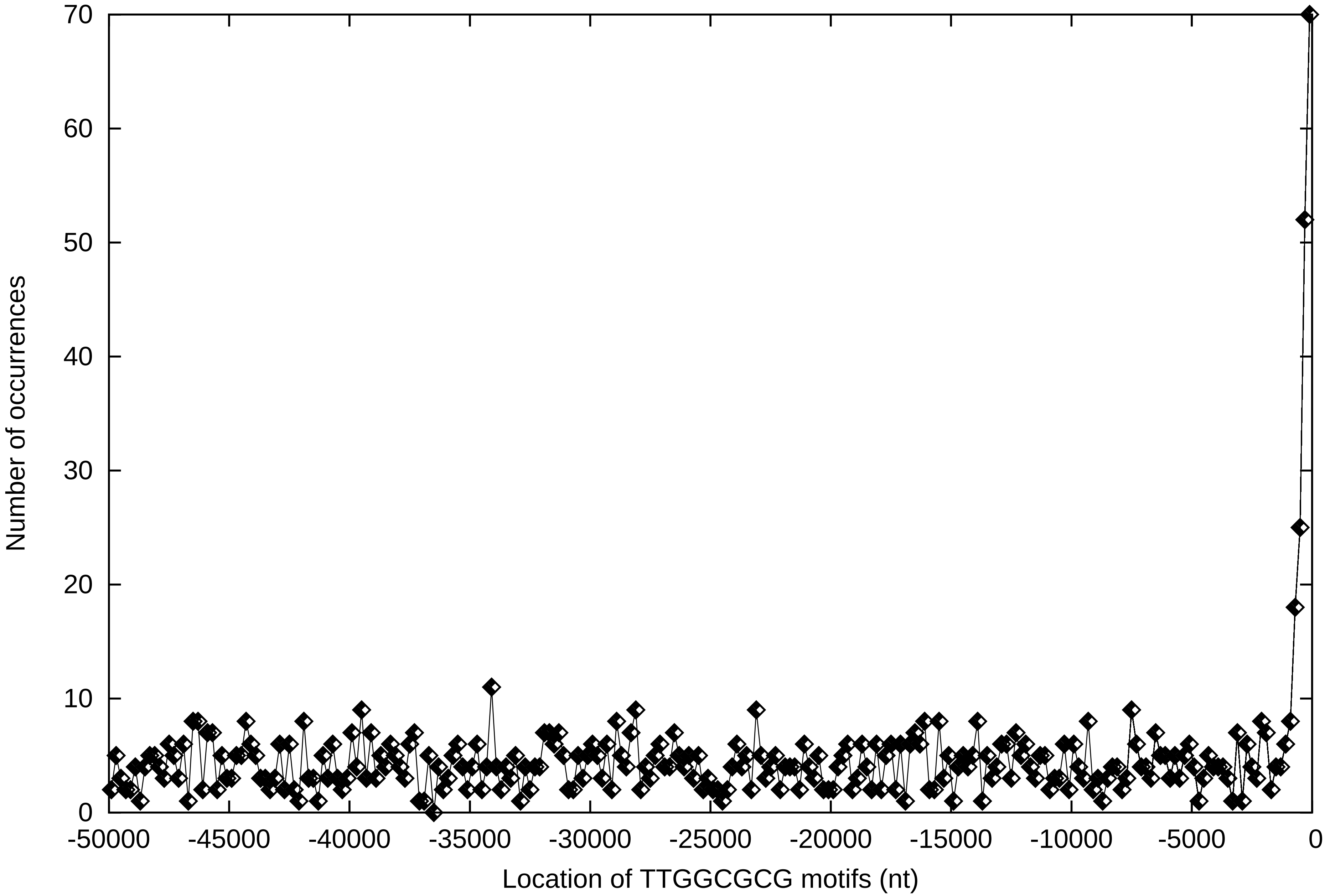

70
60
50
40
30
20
10
0
-50000
-45000
-40000
-35000
-30000
-25000
-20000
-15000
-10000
-5000
0
Number of occurrences
Location of TTGGCGCG motifs (nt)

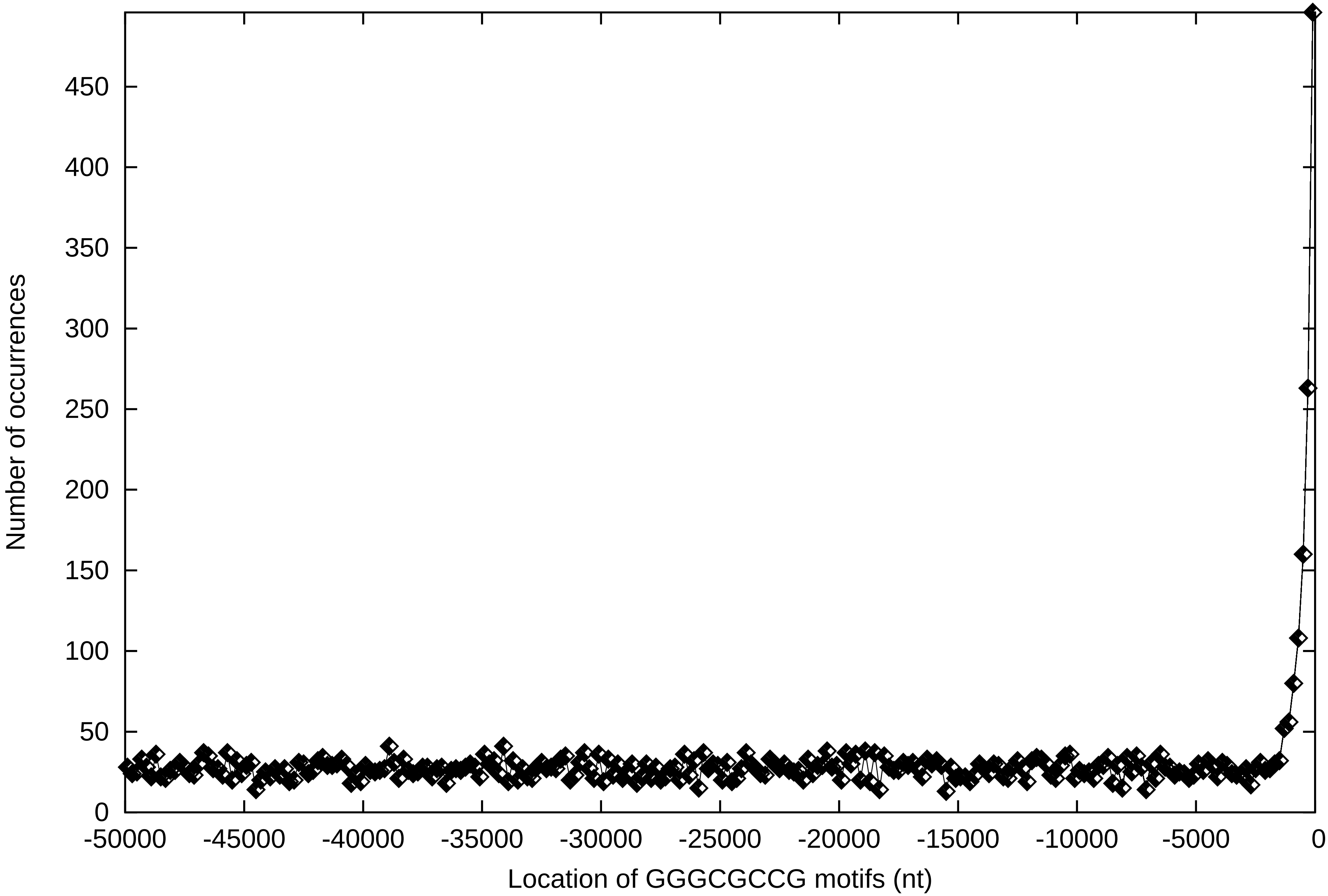

Number of occurrences
0
50
100
150
200
250
300
350
400
450
-50000
-45000
-40000
-35000
-30000
-25000
-20000
-15000
-10000
-5000
0
Location of GGGCGCCG motifs (nt)

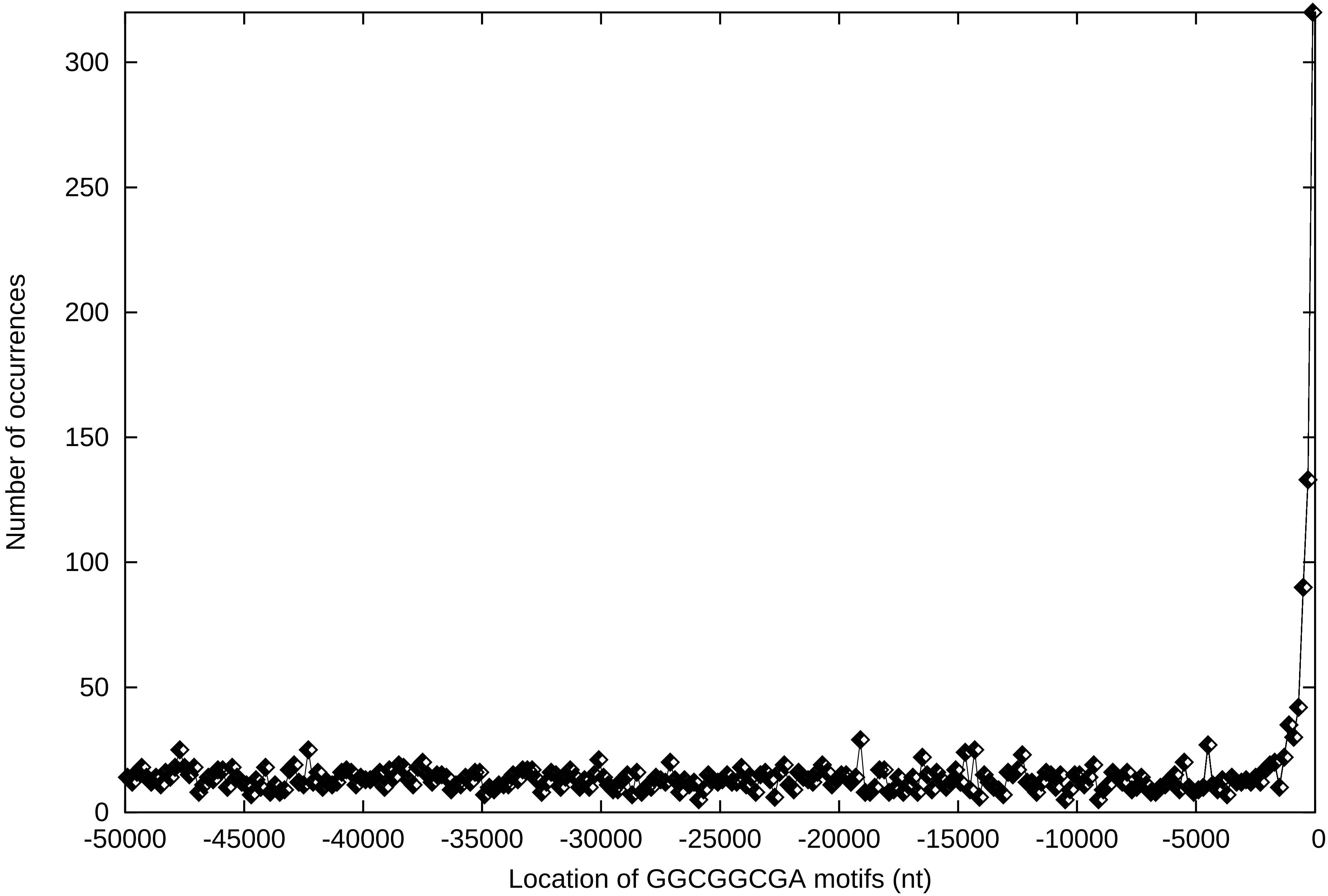

Number of occurrences
0
50
100
150
200
250
300
-50000
-45000
-40000
-35000
-30000
-25000
-20000
-15000
-10000
-5000
0
Location of GGCGGCGA motifs (nt)

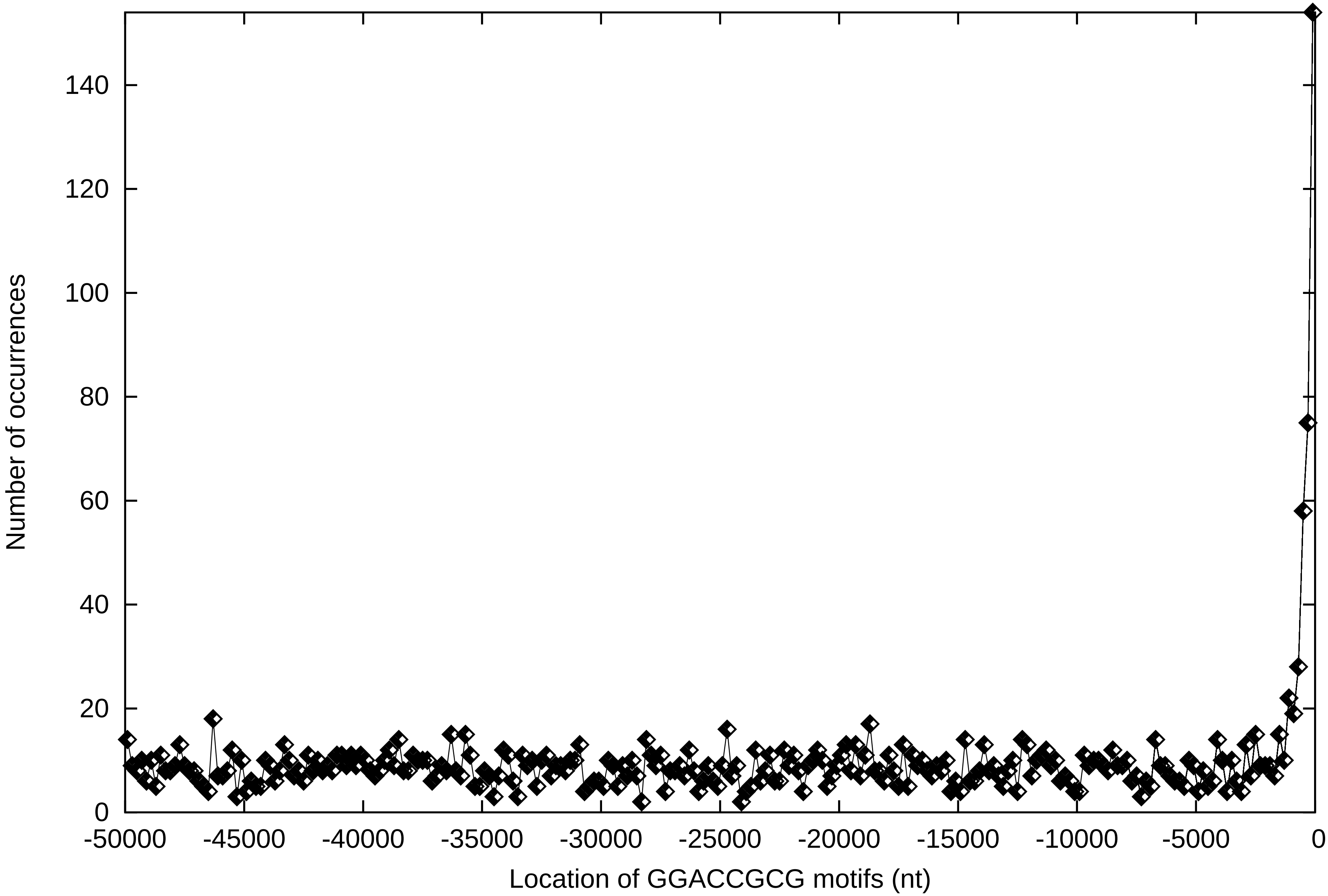

Number of occurrences
0
20
40
60
80
100
120
140
-50000
-45000
-40000
-35000
-30000
-25000
-20000
-15000
-10000
-5000
0
Location of GGACCGCG motifs (nt)

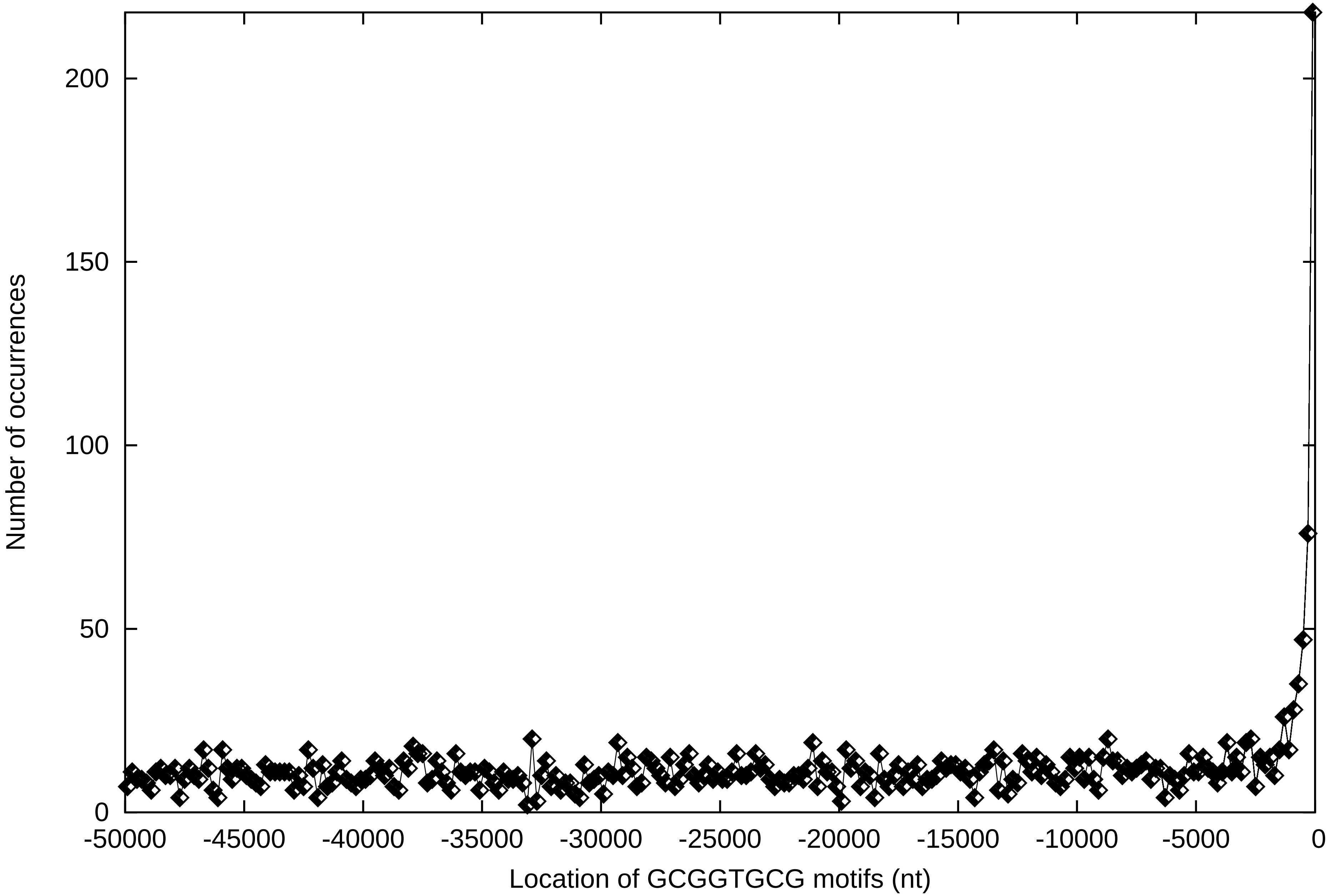

Number of occurrences
0
50
100
150
200
-50000
-45000
-40000
-35000
-30000
-25000
-20000
-15000
-10000
-5000
0
Location of GCGGTGCG motifs (nt)

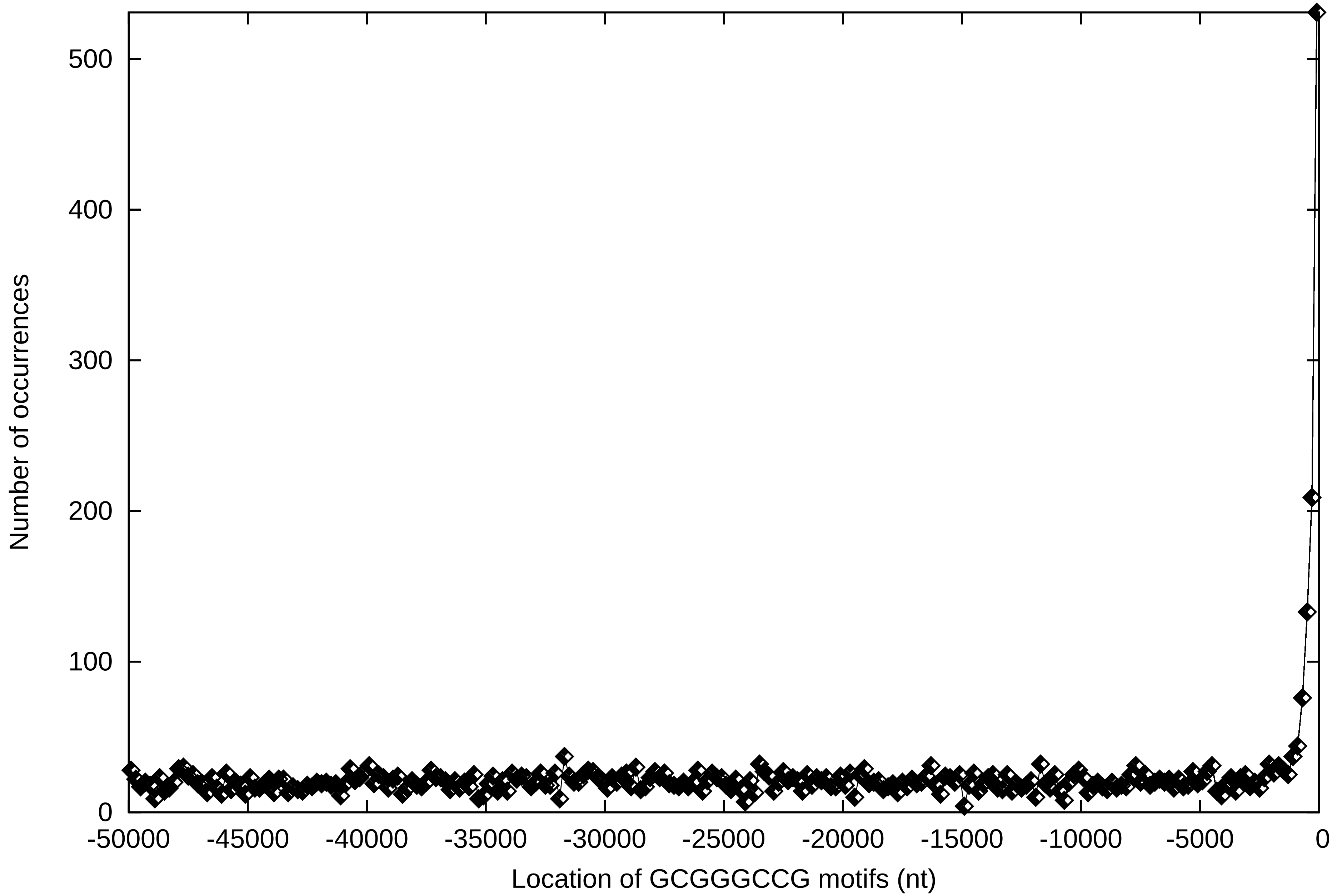
Number of occurrences
0
100
200
300
400
500
-50000
-45000
-40000
-35000
-30000
-25000
-20000
-15000
-10000
-5000
0
Location of GCGGGCCG motifs (nt)

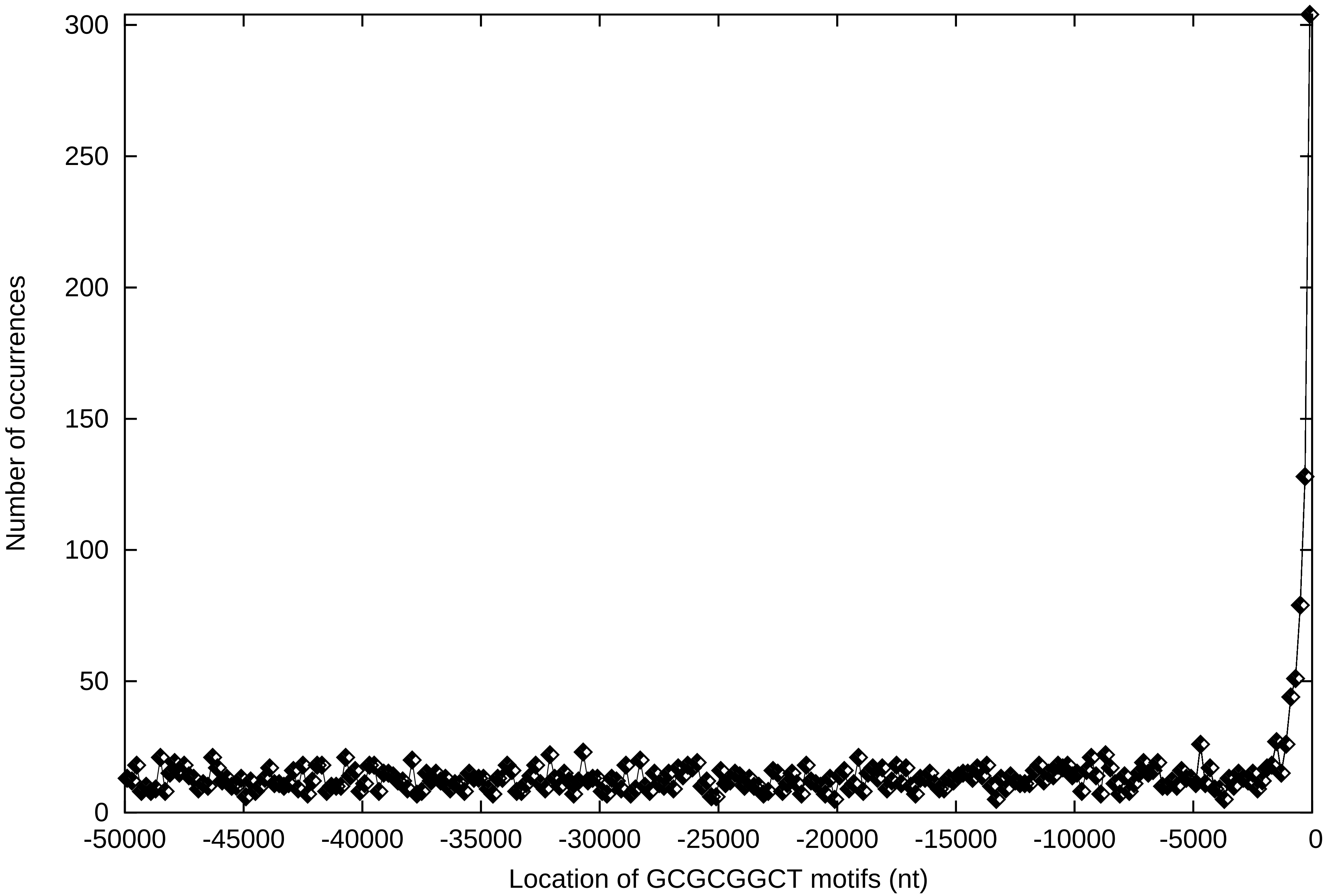

Number of occurrences
Location of GCGCGGCT motifs (nt)
300
250
200
150
100
50
0
-50000
-45000
-40000
-35000
-30000
-25000
-20000
-15000
-10000
-5000
0

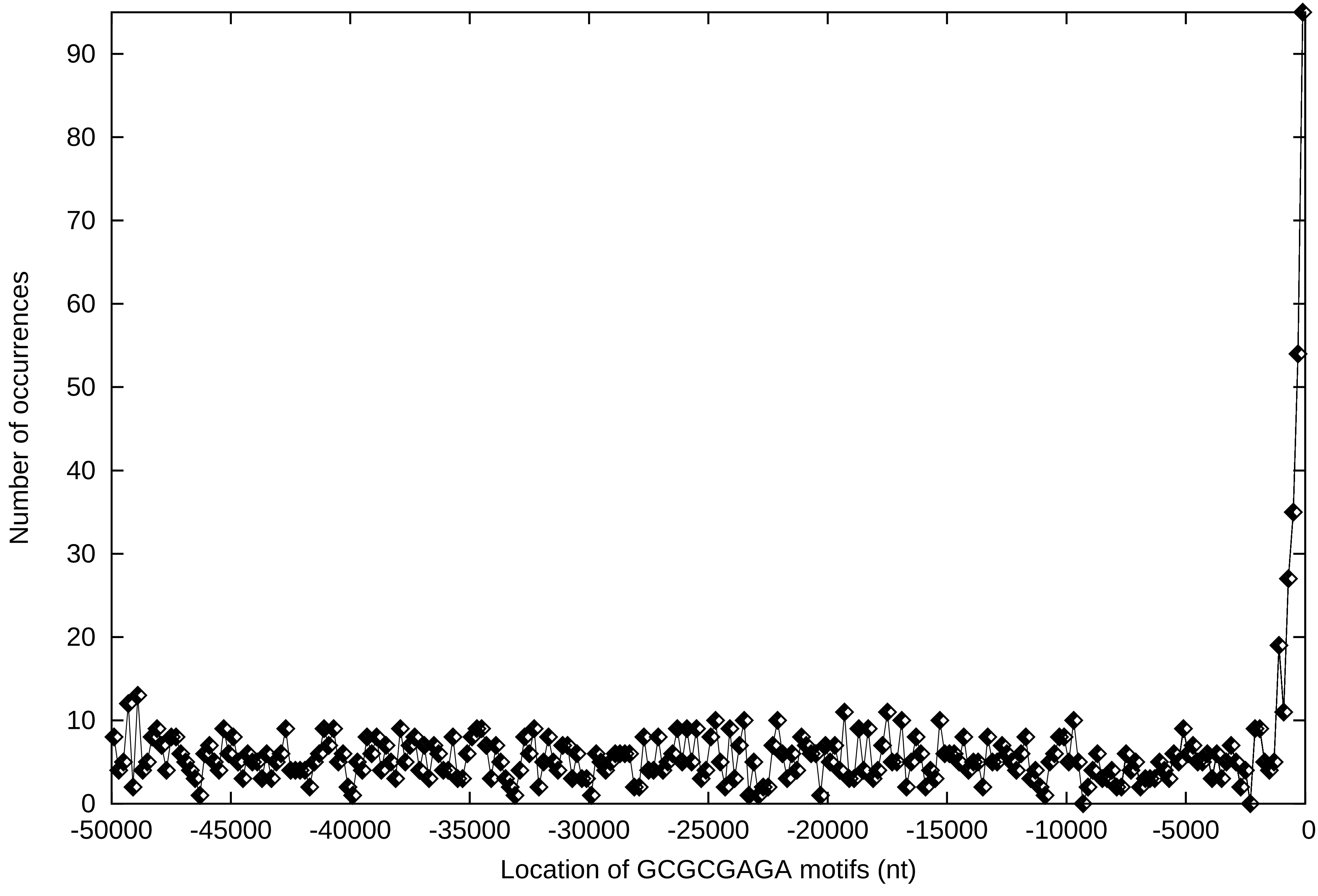
Number of occurrences
0
10
20
30
40
50
60
70
80
90
-50000
-45000
-40000
-35000
-30000
-25000
-20000
-15000
-10000
-5000
0
Location of GCGCGAGA motifs (nt)

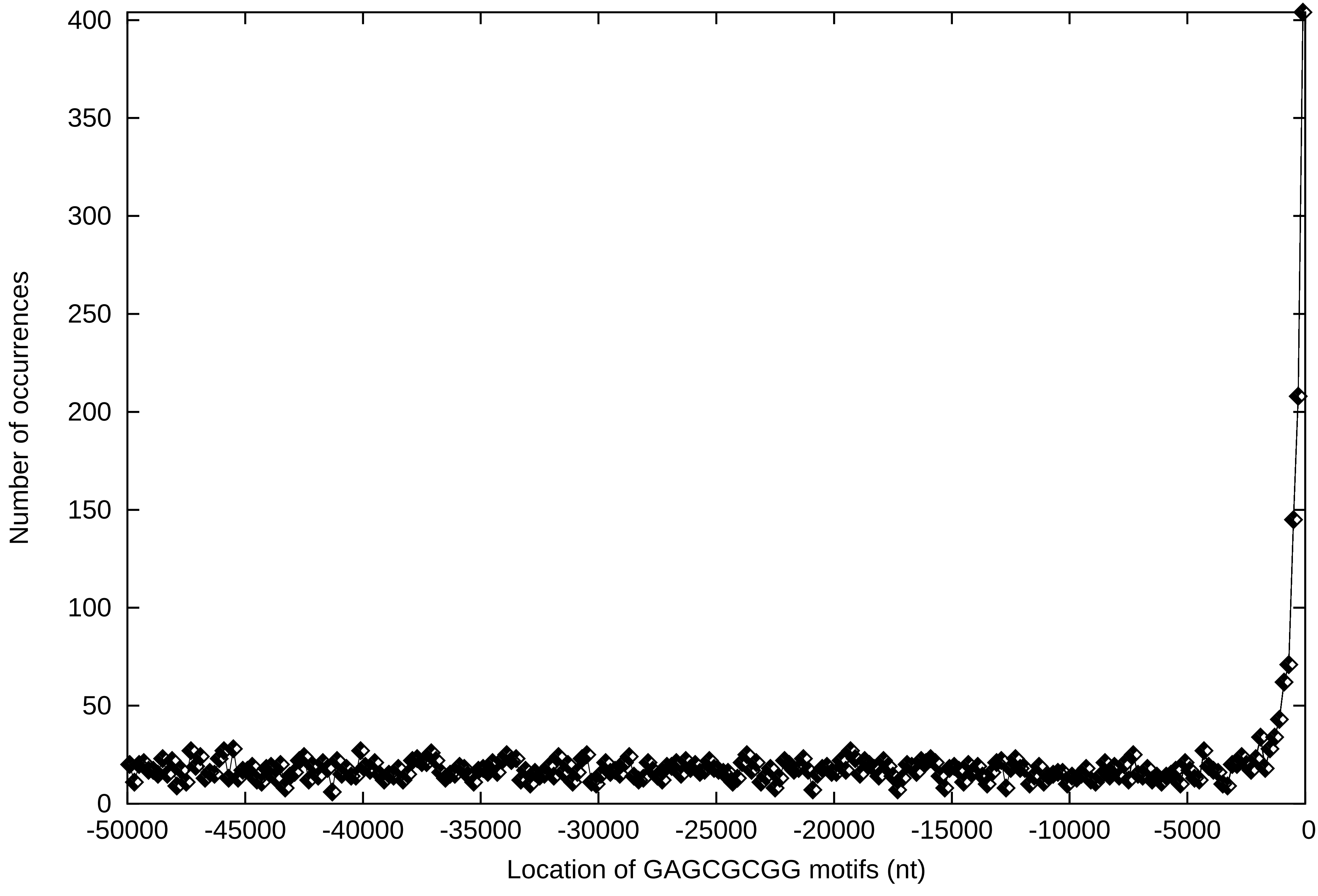

400
350
300
250
200
150
100
50
0
-50000
-45000
-40000
-35000
-30000
-25000
-20000
-15000
-10000
-5000
0
Number of occurrences
Location of GAGCGCGG motifs (nt)

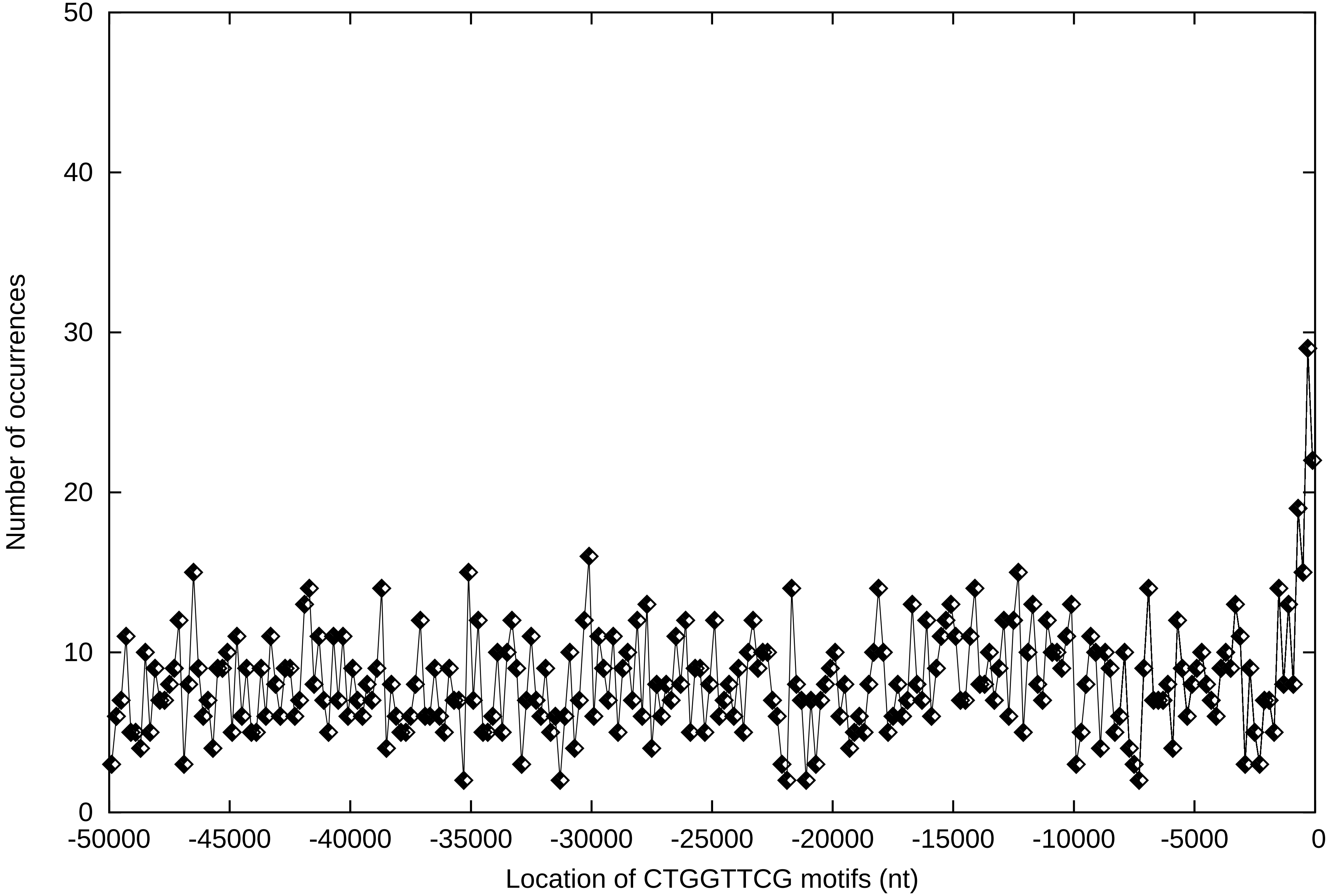
50
40
30
20
10
0
-50000
-45000
-40000
-35000
-30000
-25000
-20000
-15000
-10000
-5000
0
Number of occurrences
Location of CTGGTTCG motifs (nt)

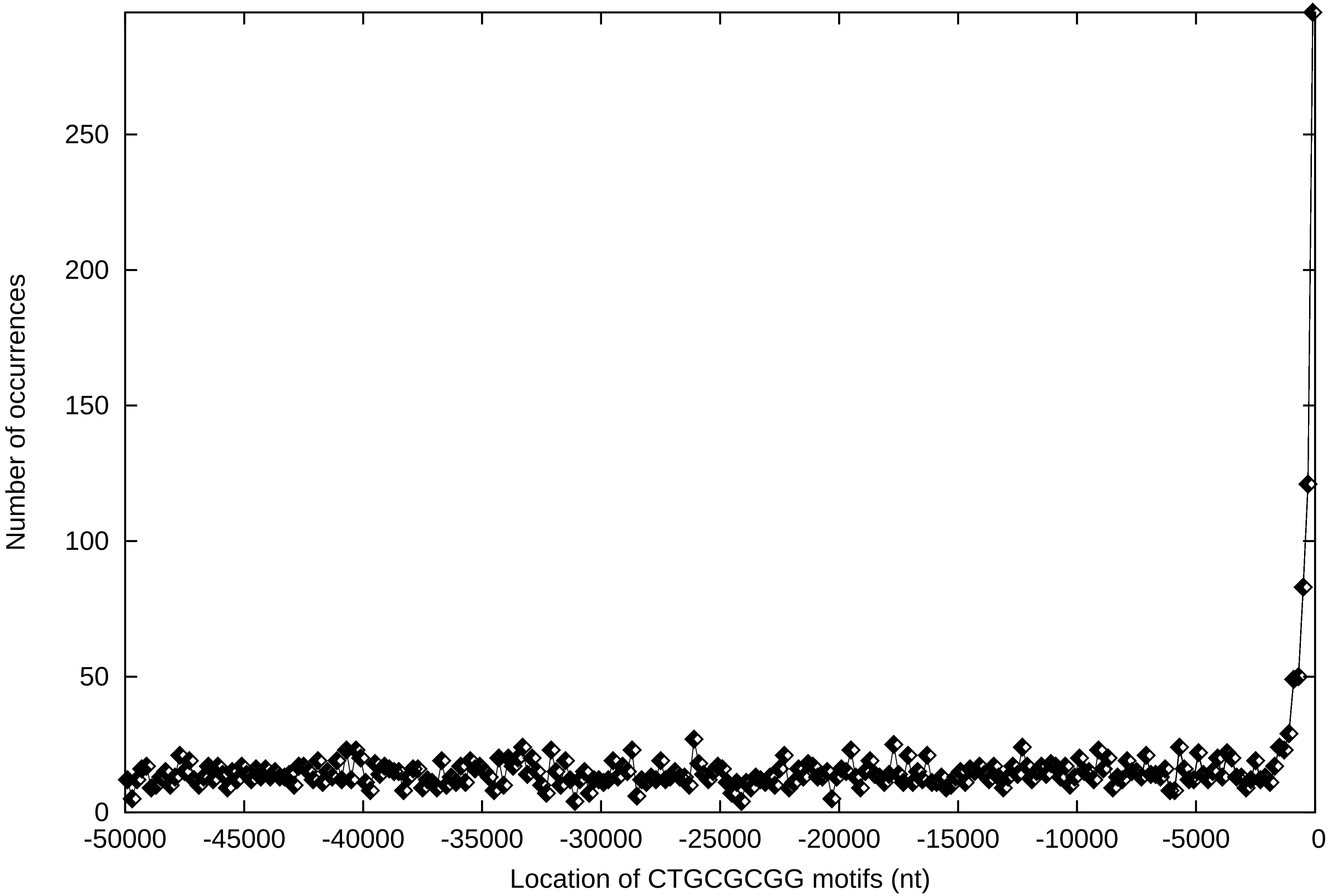

Number of occurrences
0
50
100
150
200
250
-50000
-45000
-40000
-35000
-30000
-25000
-20000
-15000
-10000
-5000
0
Location of CTGCGCGG motifs (nt)

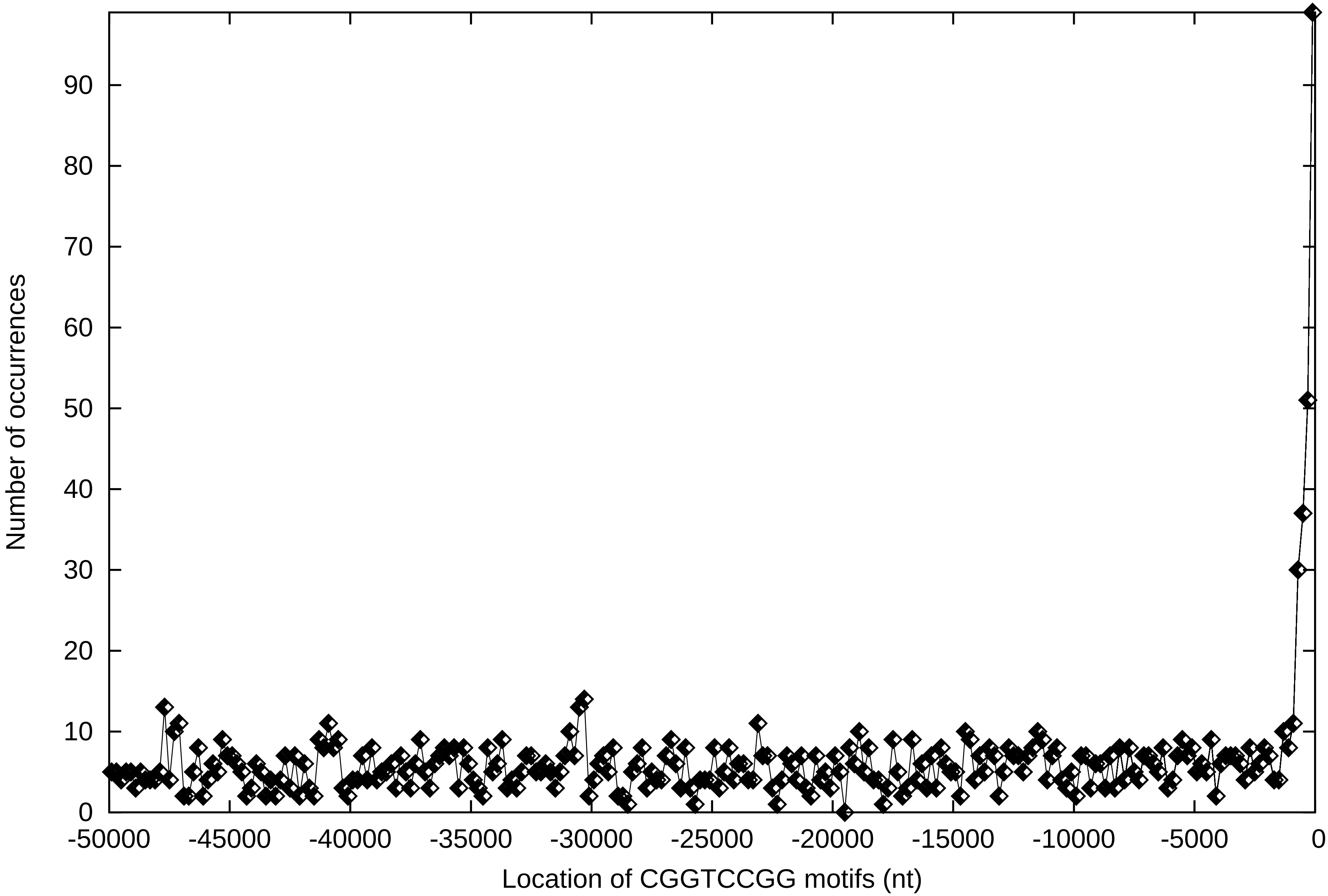

Number of occurrences
0
10
20
30
40
50
60
70
80
90
-50000
-45000
-40000
-35000
-30000
-25000
-20000
-15000
-10000
-5000
0
Location of CGGTCCGG motifs (nt)

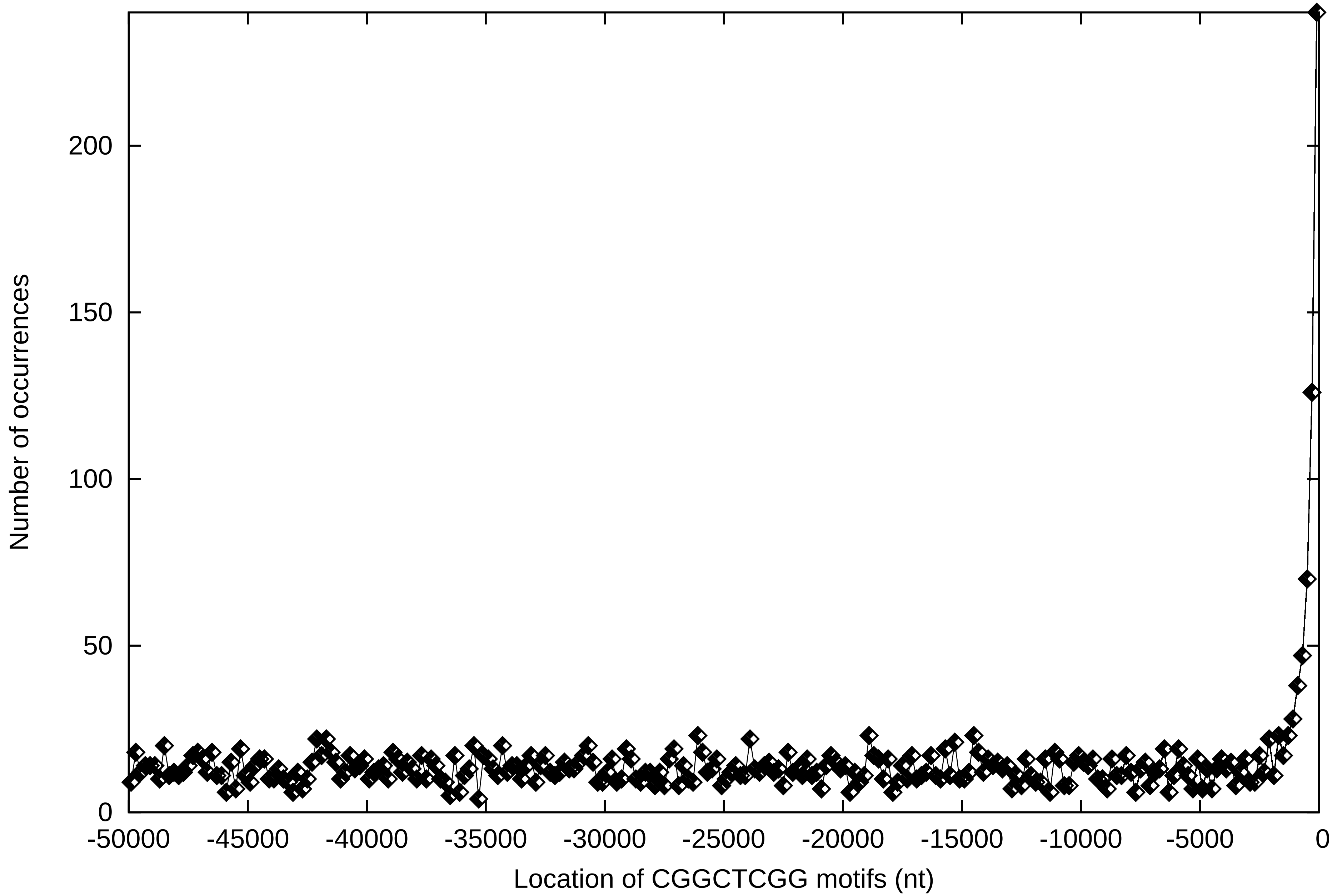

Number of occurrences
200
150
100
50
0
-50000
-45000
-40000
-35000
-30000
-25000
-20000
-15000
-10000
-5000
0
Location of CGGCTCGG motifs (nt)

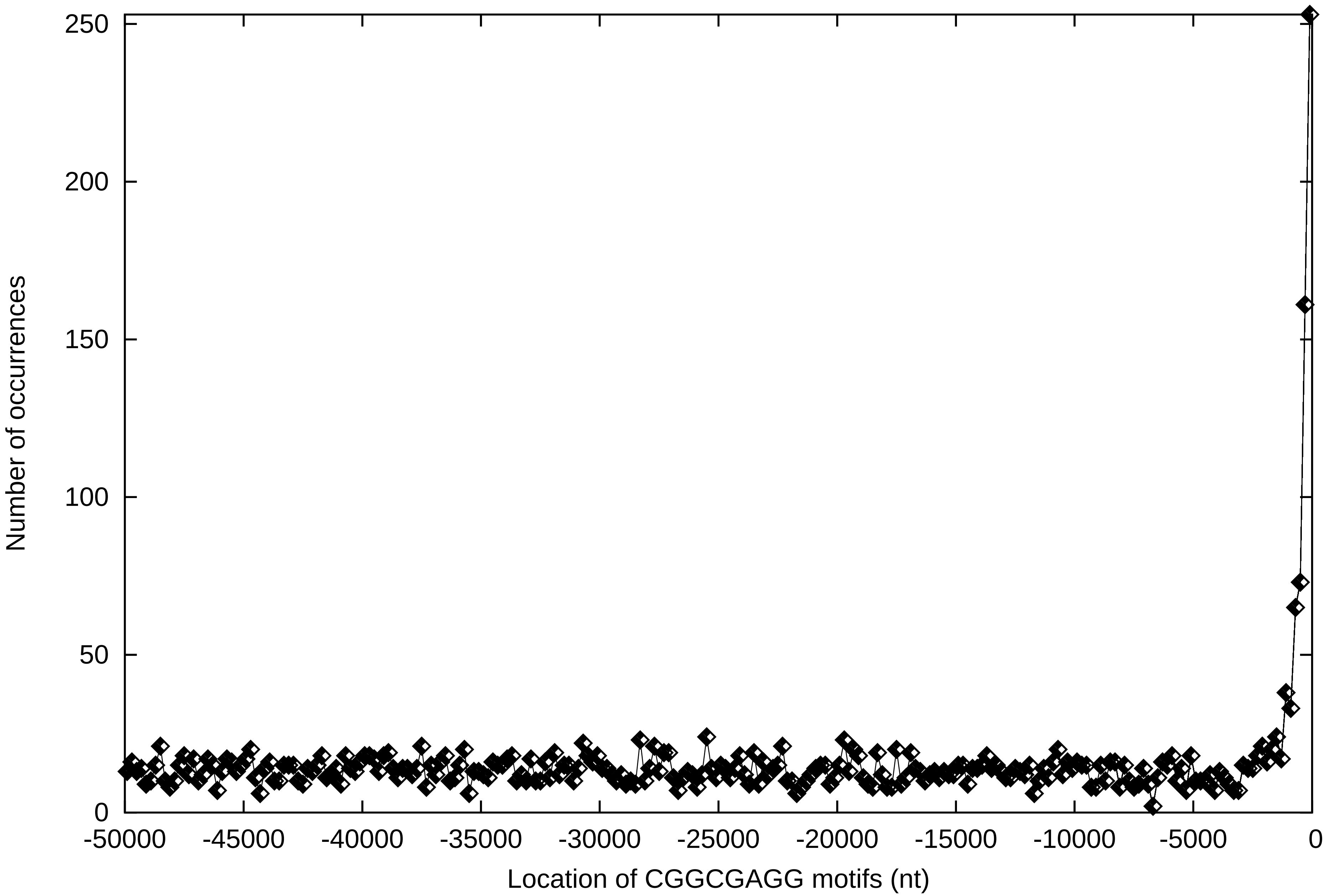

Number of occurrences
250
200
150
100
50
0
-50000
-45000
-40000
-35000
-30000
-25000
-20000
-15000
-10000
-5000
0
Location of CGGCGAGG motifs (nt)

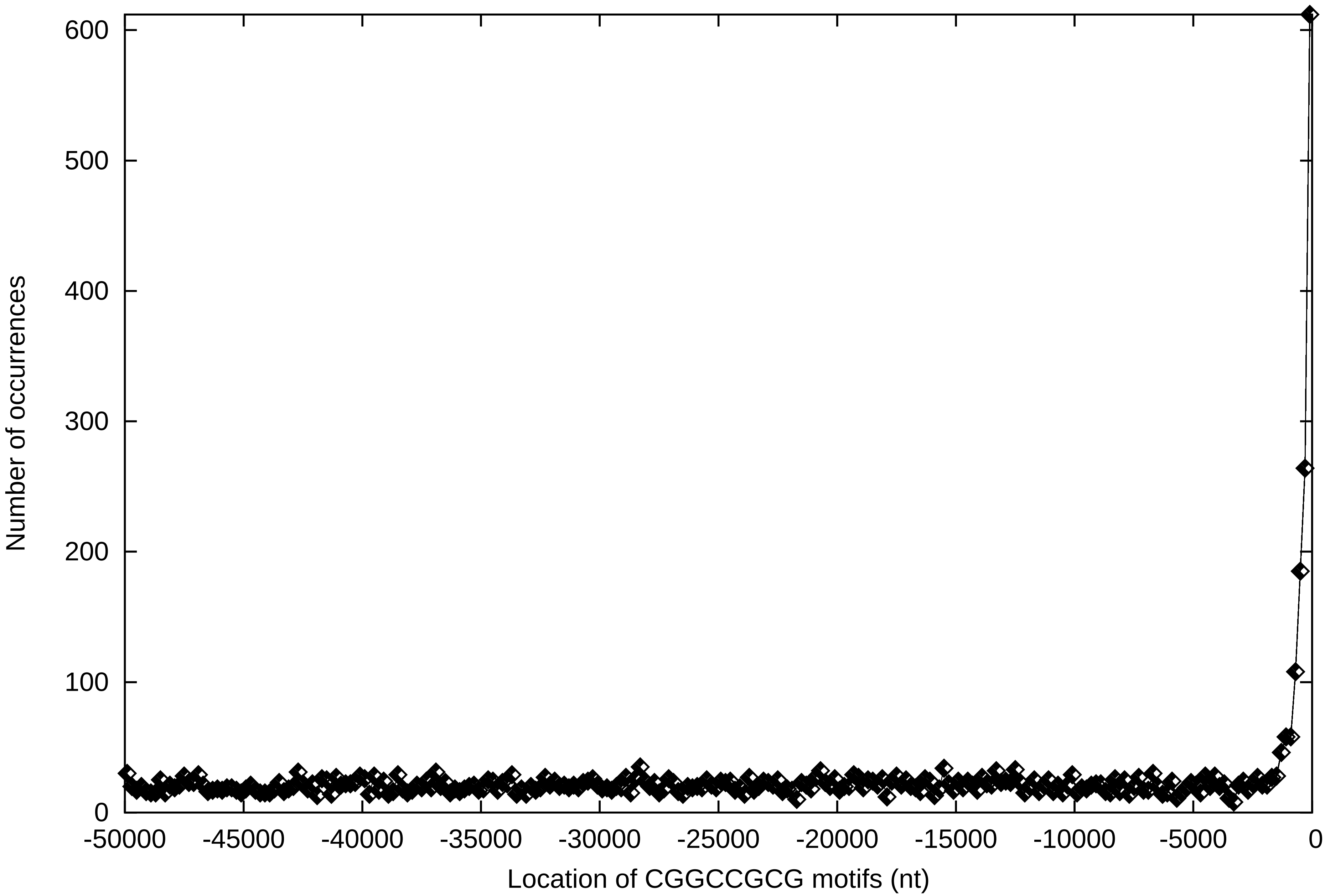

Number of occurrences
600
500
400
300
200
100
0
-50000
-45000
-40000
-35000
-30000
-25000
-20000
-15000
-10000
-5000
0
Location of CGGCCGCG motifs (nt)

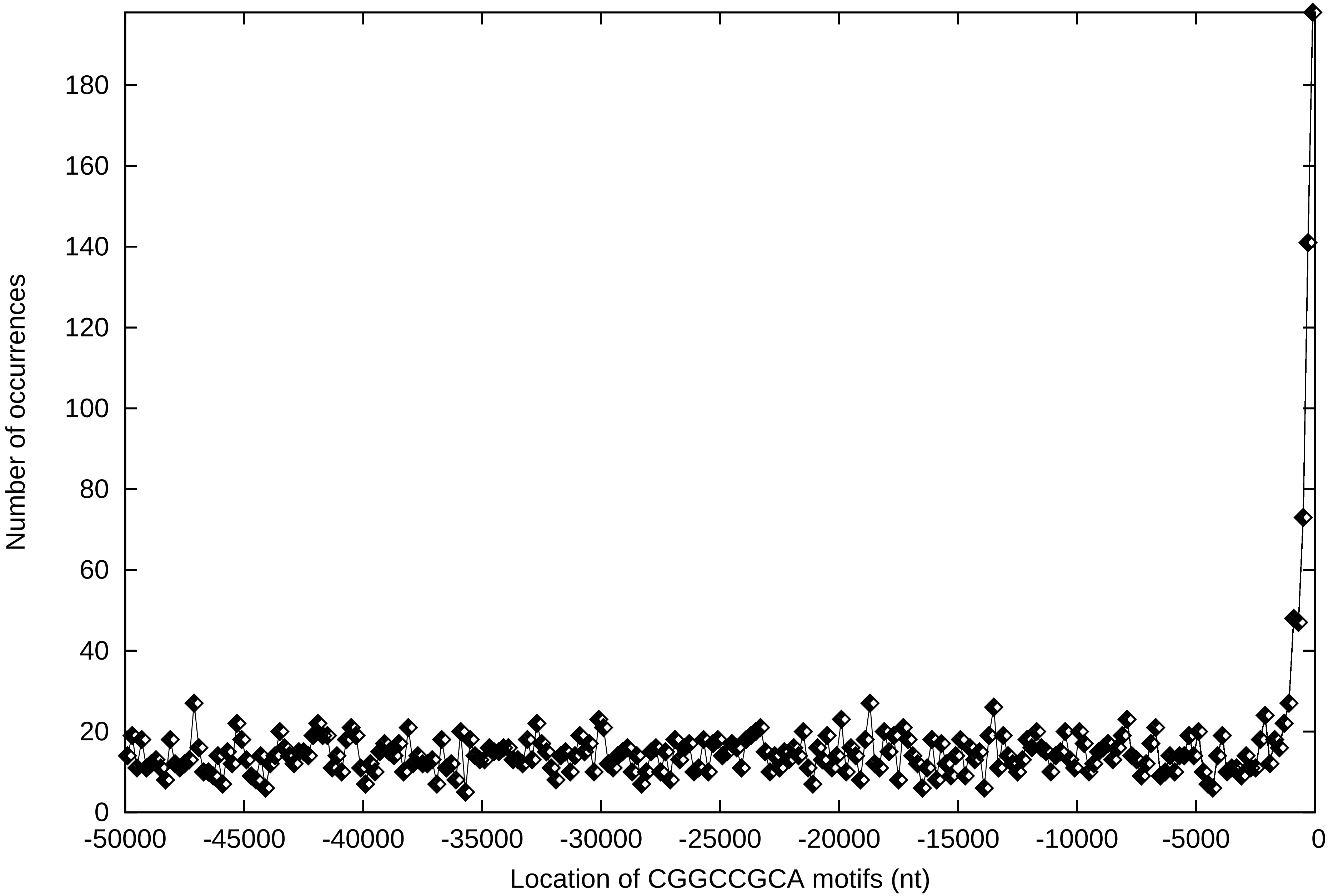

Number of occurrences
0
20
40
60
80
100
120
140
160
180
-50000
-45000
-40000
-35000
-30000
-25000
-20000
-15000
-10000
-5000
0
Location of CGGCCGCA motifs (nt)

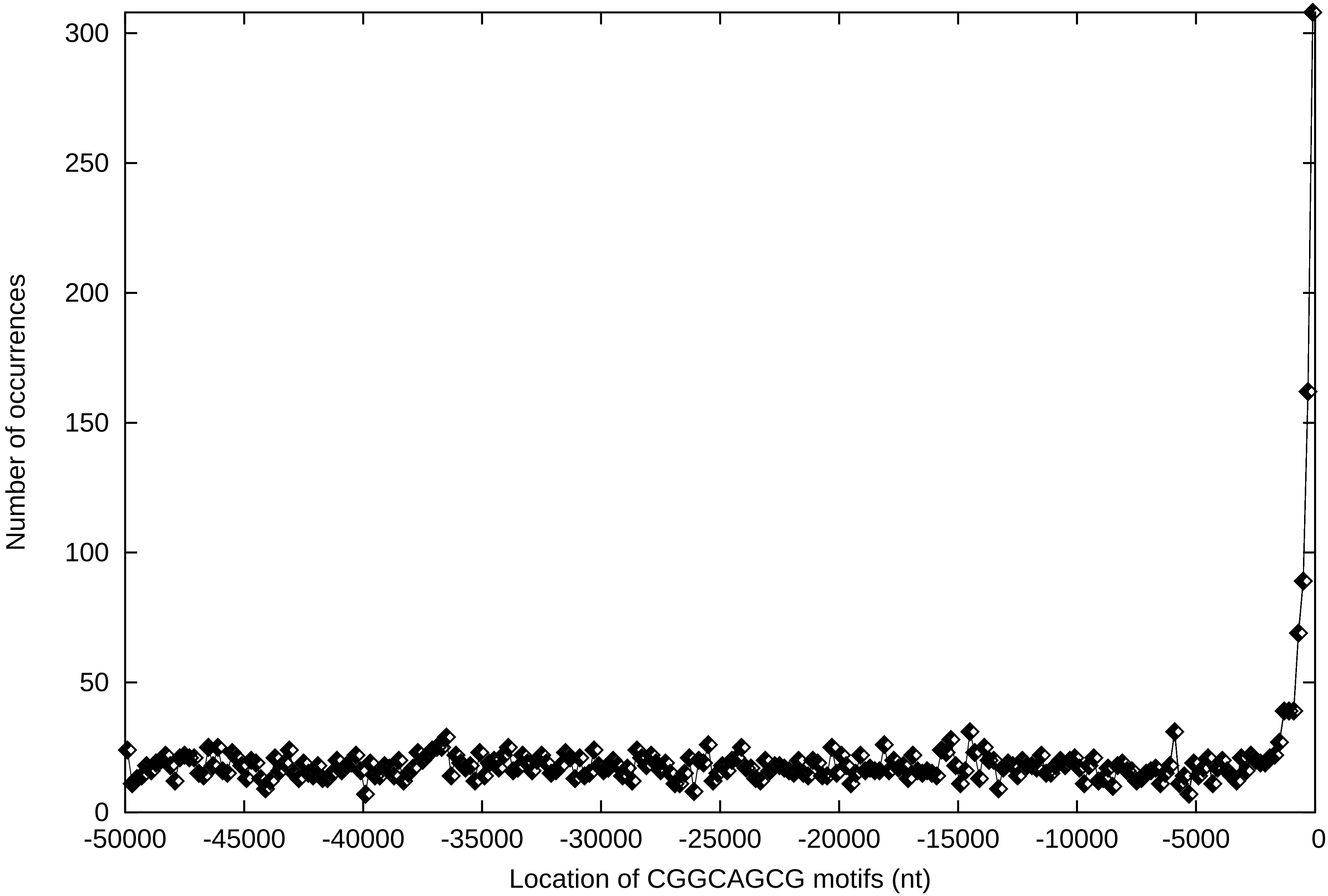

Number of occurrences
300
250
200
150
100
50
0
-50000
-45000
-40000
-35000
-30000
-25000
-20000
-15000
-10000
-5000
0
Location of CGGCAGCG motifs (nt)

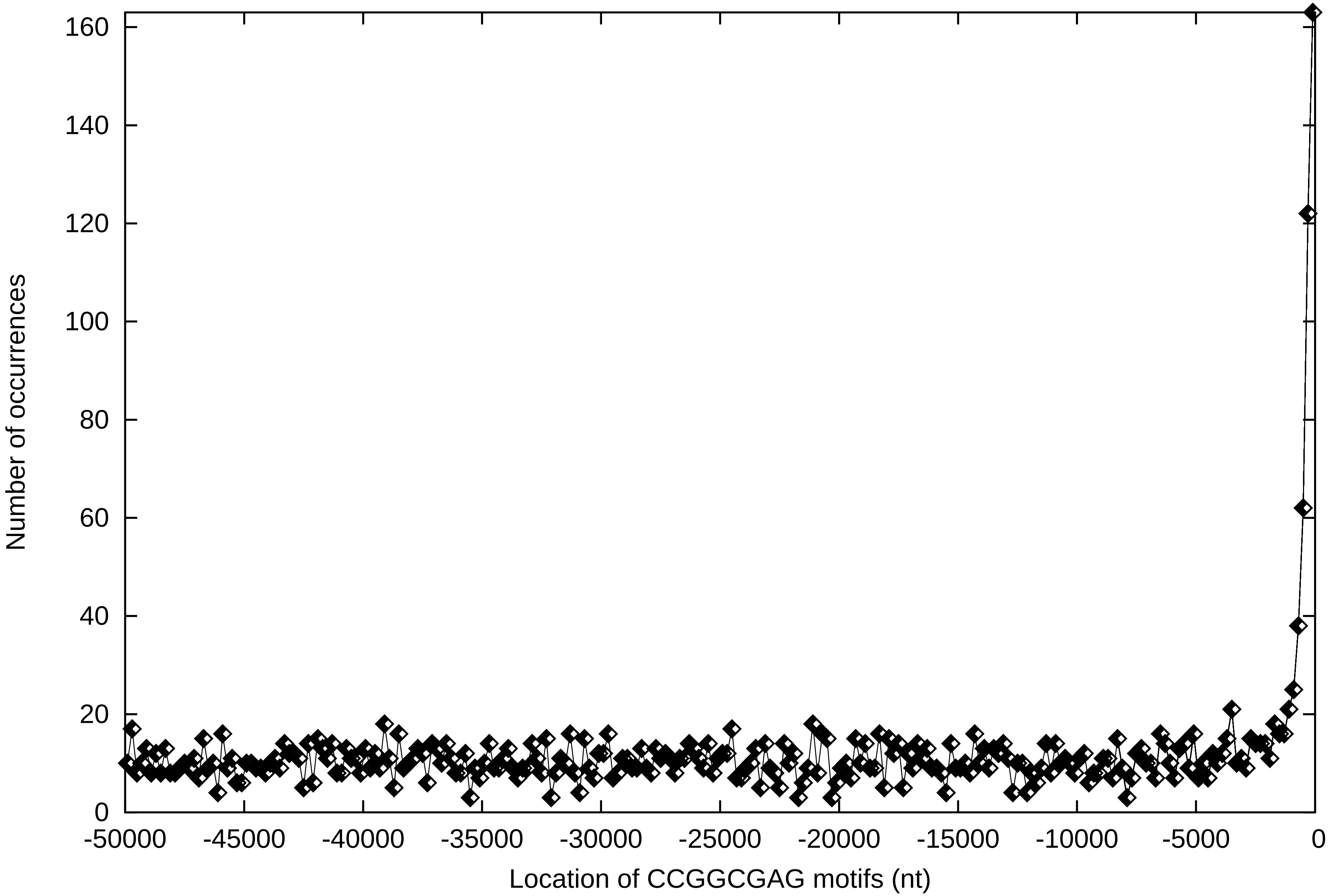

160
140
120
100
80
60
40
20
0
-50000
-45000
-40000
-35000
-30000
-25000
-20000
-15000
-10000
-5000
0
Number of occurrences
Location of CCGGCGAG motifs (nt)

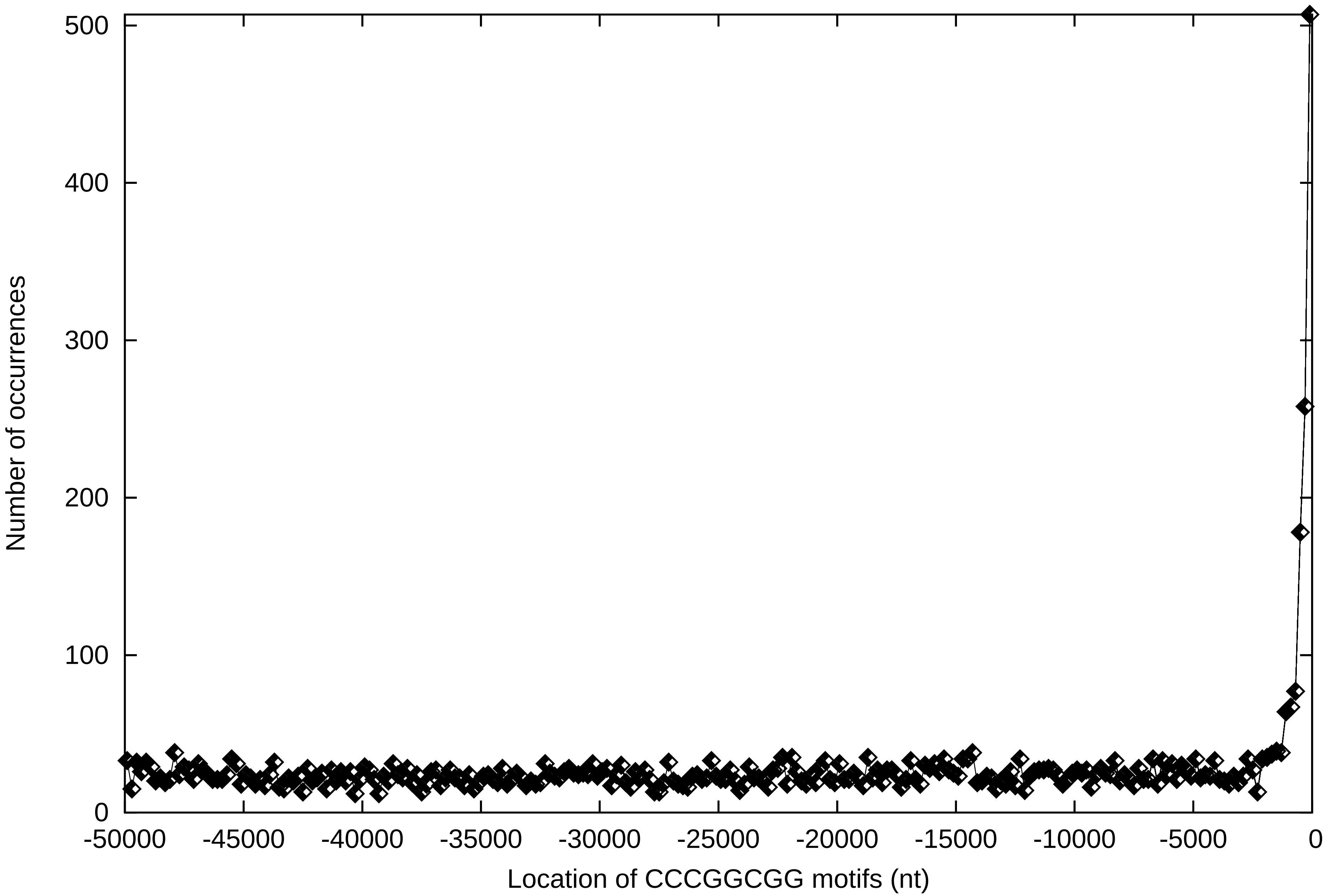

500
400
300
200
100
0
Number of occurrences
-50000
-45000
-40000
-35000
-30000
-25000
-20000
-15000
-10000
-5000
0
Location of CCCGGCGG motifs (nt)

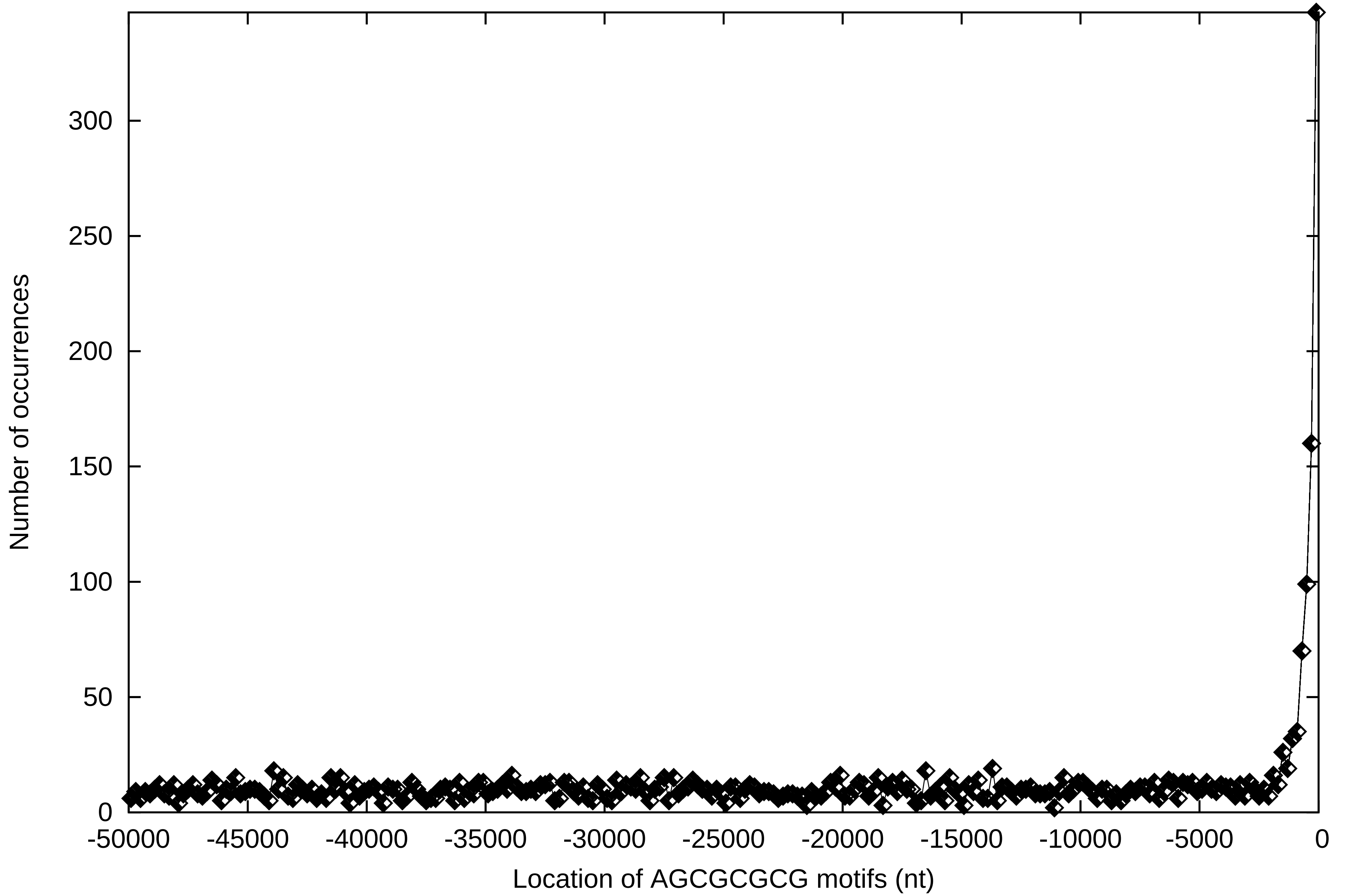

Number of occurrences
300
250
200
150
100
50
0
-50000
-45000
-40000
-35000
-30000
-25000
-20000
-15000
-10000
-5000
0
Location of AGCGCGCG motifs (nt)

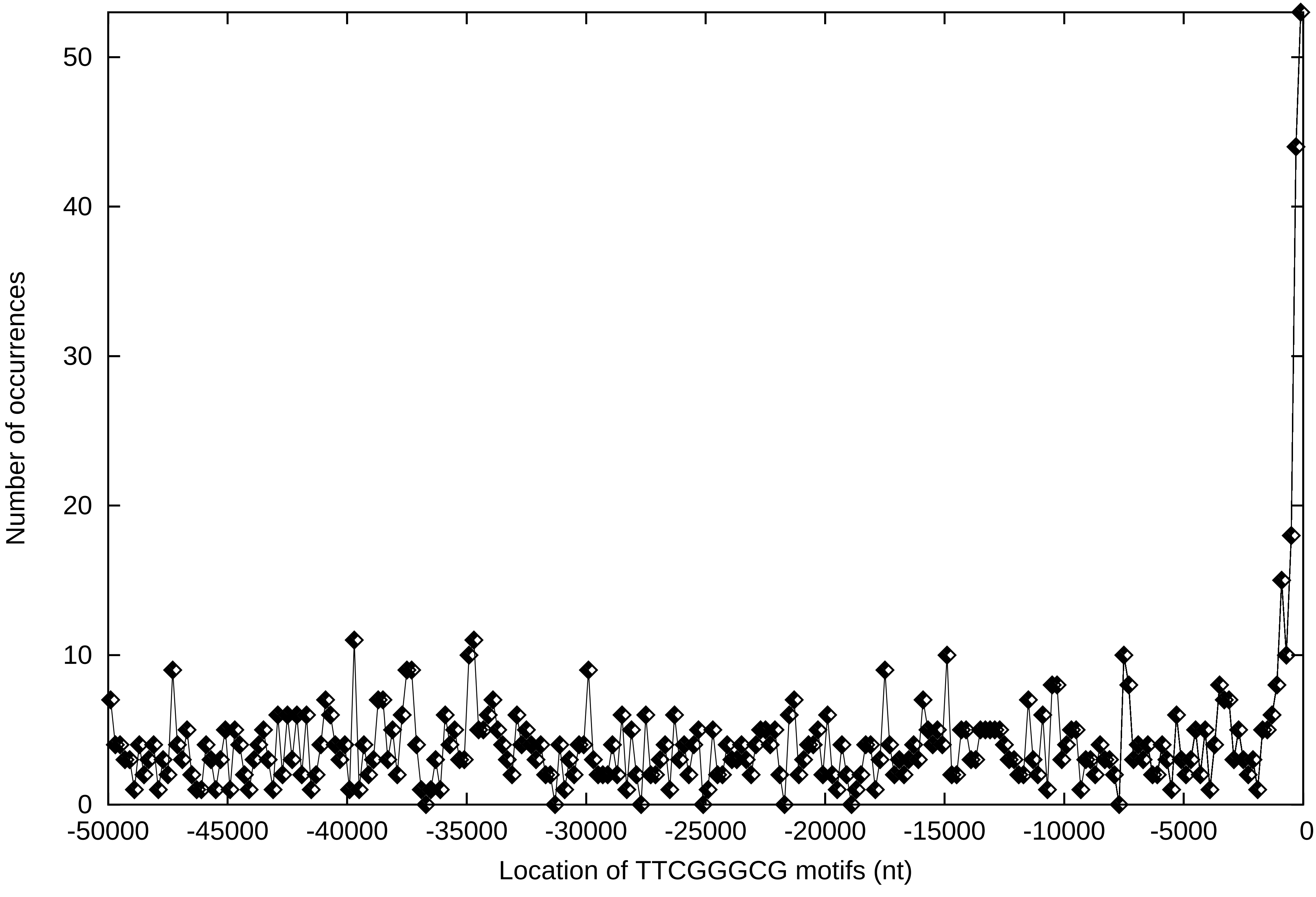

Number of occurrences
0
10
20
30
40
50
-50000
-45000
-40000
-35000
-30000
-25000
-20000
-15000
-10000
-5000
0
Location of TTCGGGCG motifs (nt)

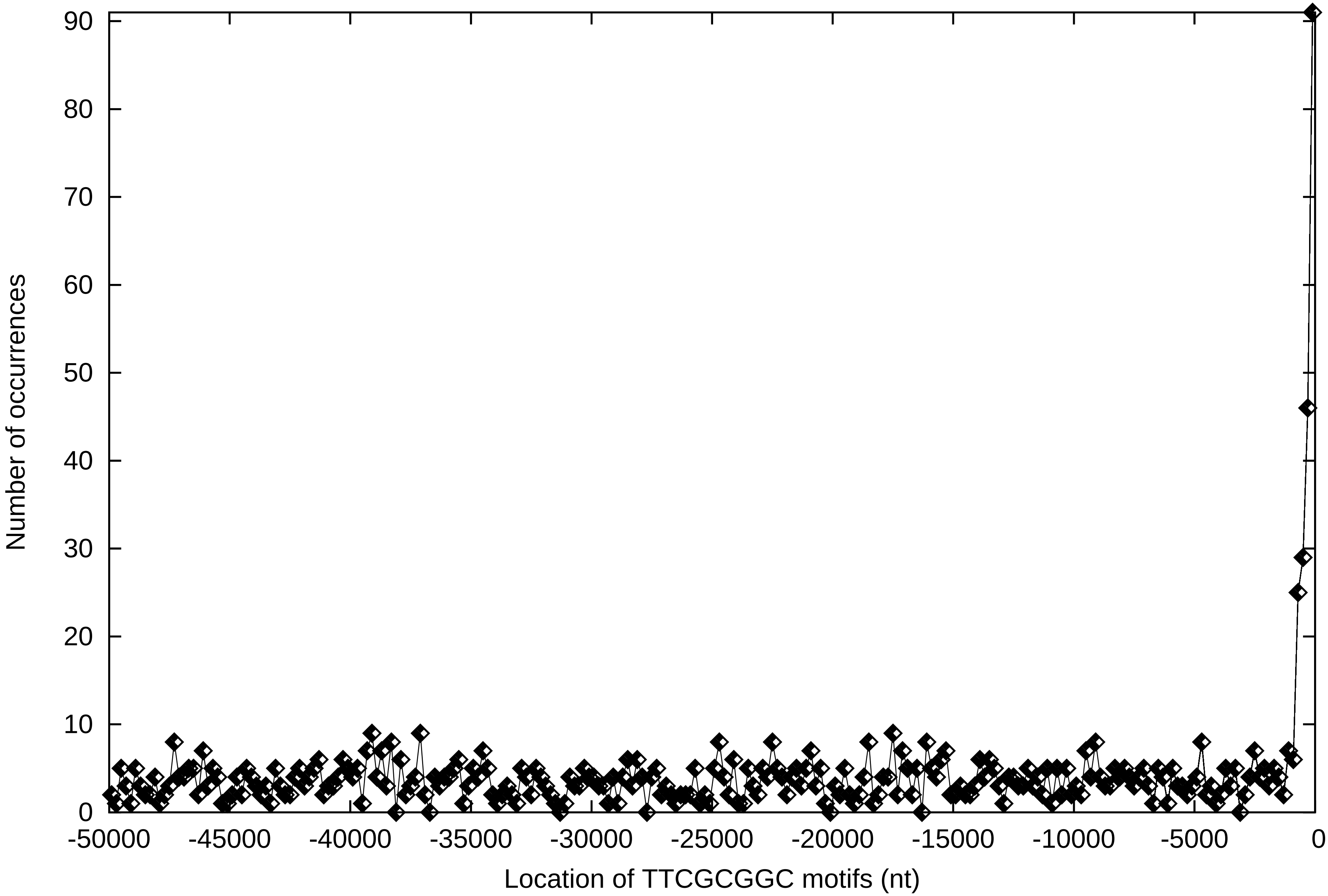
Number of occurrences
Location of TTCGCGGC motifs (nt)
0
10
20
30
40
50
60
70
80
90
-50000
-45000
-40000
-35000
-30000
-25000
-20000
-15000
-10000
-5000
0

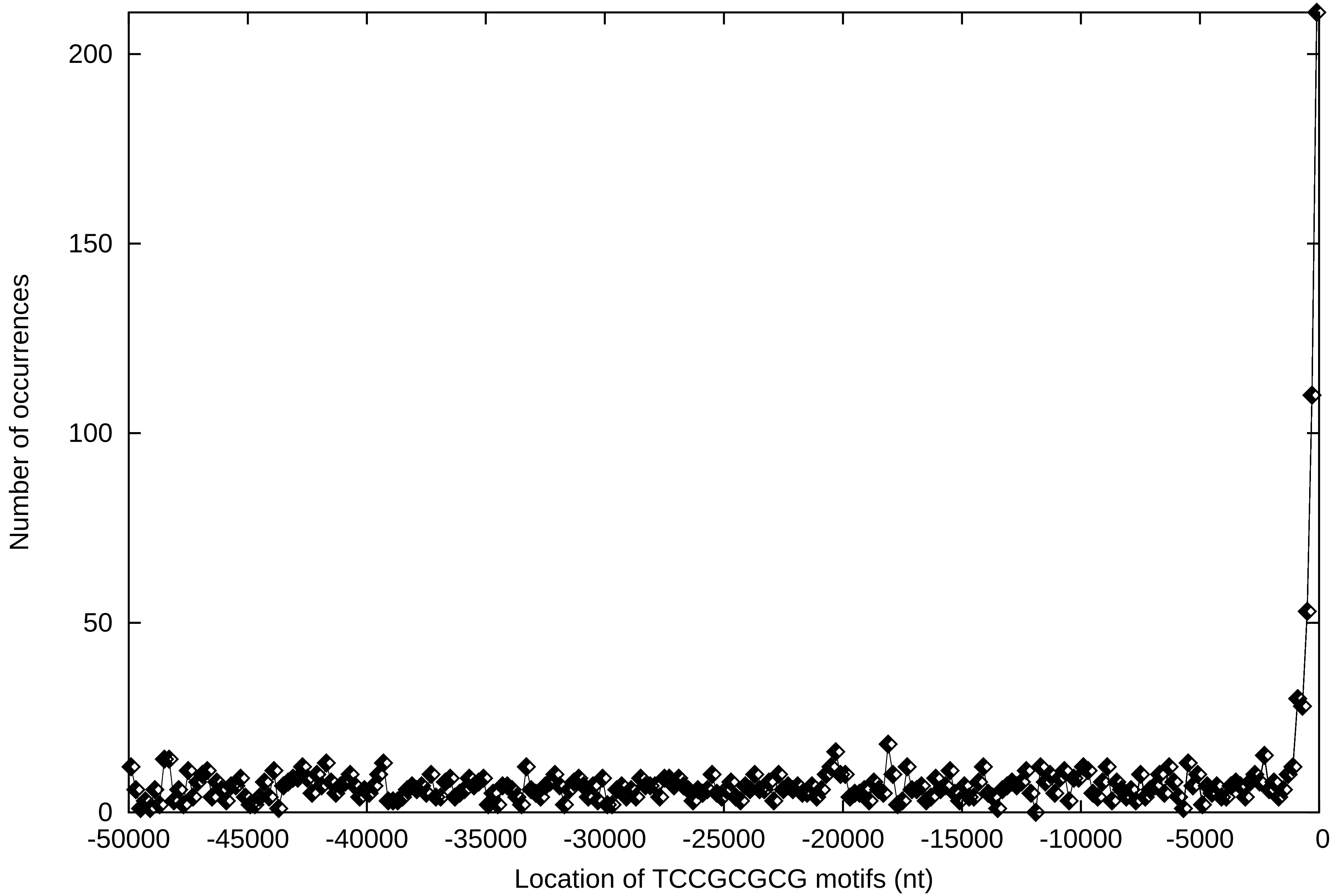

Number of occurrences
200
150
100
50
0
-50000
-45000
-40000
-35000
-30000
-25000
-20000
-15000
-10000
-5000
0
Location of TCCGCGCG motifs (nt)

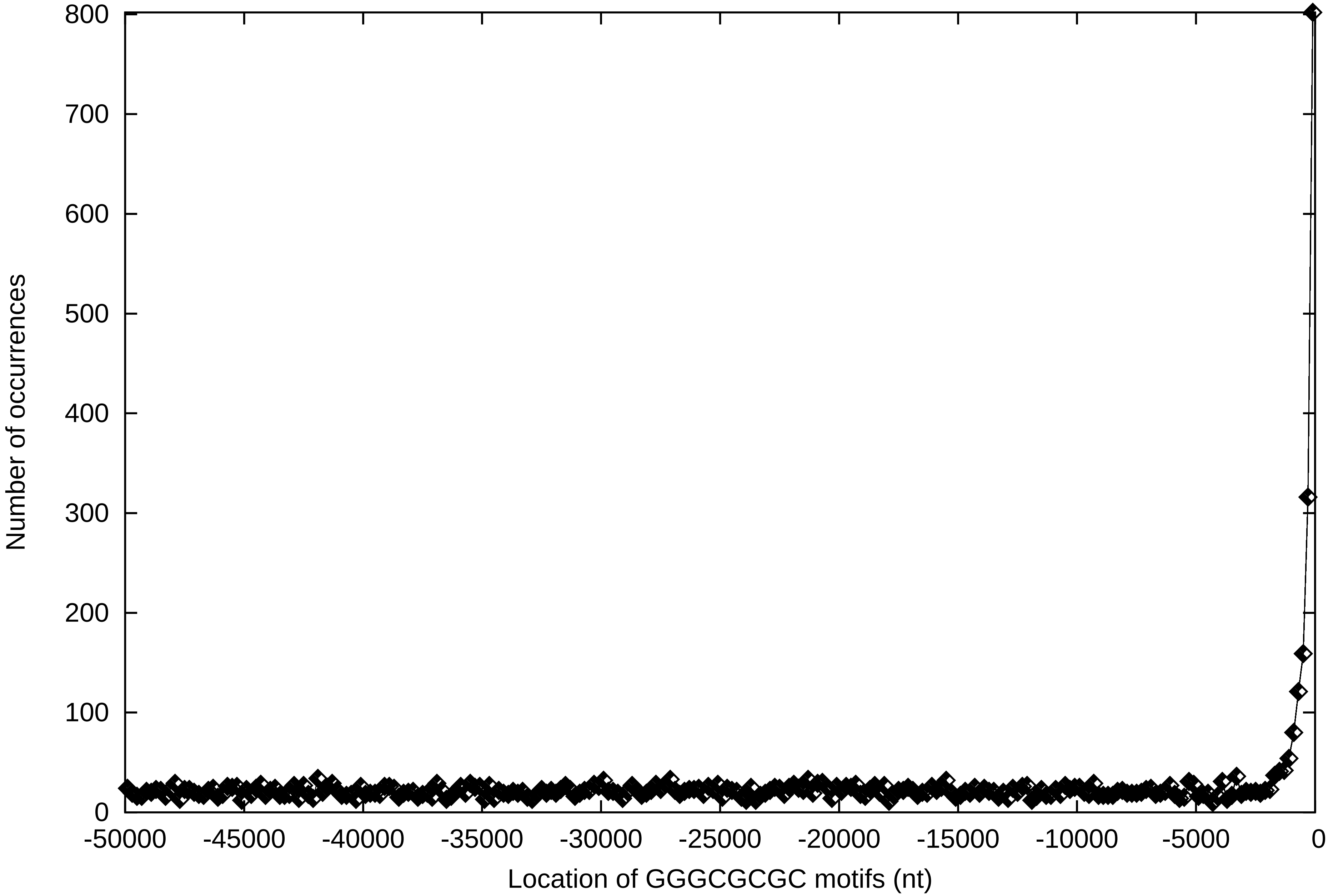

Number of occurrences
800
700
600
500
400
300
200
100
0
-50000
-45000
-40000
-35000
-30000
-25000
-20000
-15000
-10000
-5000
0
Location of GGGCGCGC motifs (nt)

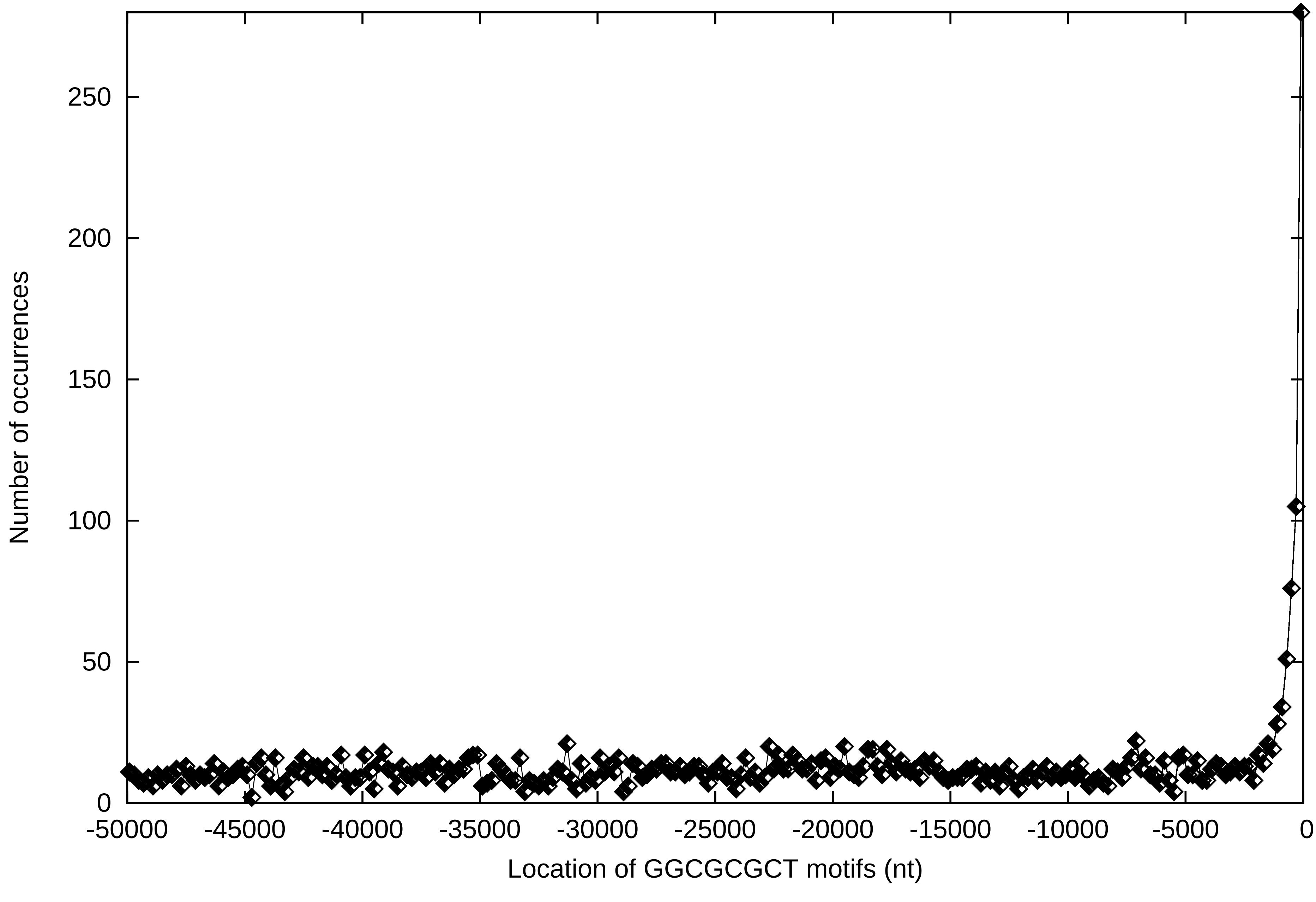
Number of occurrences
Location of GGCGCGCT motifs (nt)
0
50
100
150
200
250
-50000
-45000
-40000
-35000
-30000
-25000
-20000
-15000
-10000
-5000
0

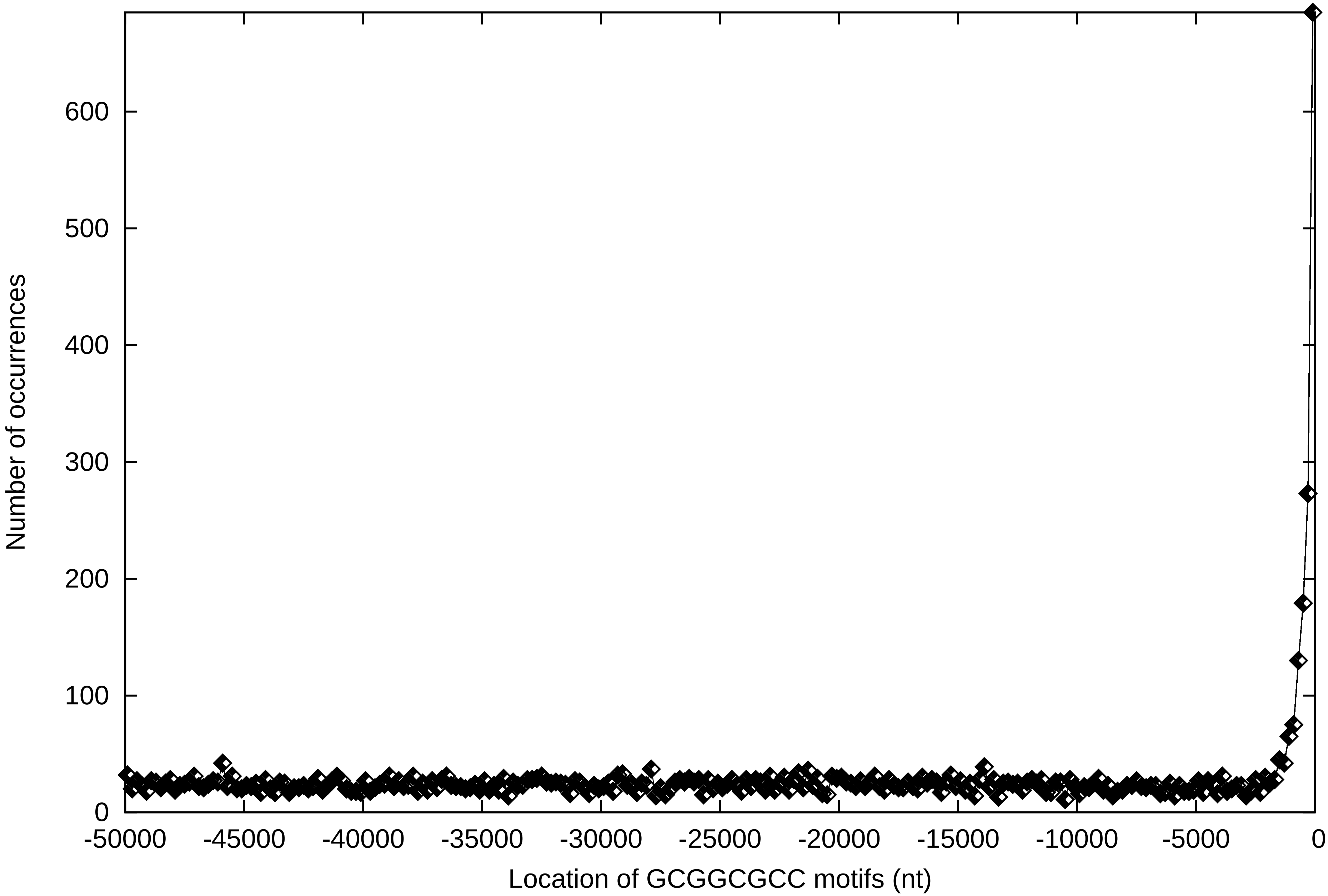

Number of occurrences
0
100
200
300
400
500
600
-50000
-45000
-40000
-35000
-30000
-25000
-20000
-15000
-10000
-5000
0
Location of GCGGCGCC motifs (nt)

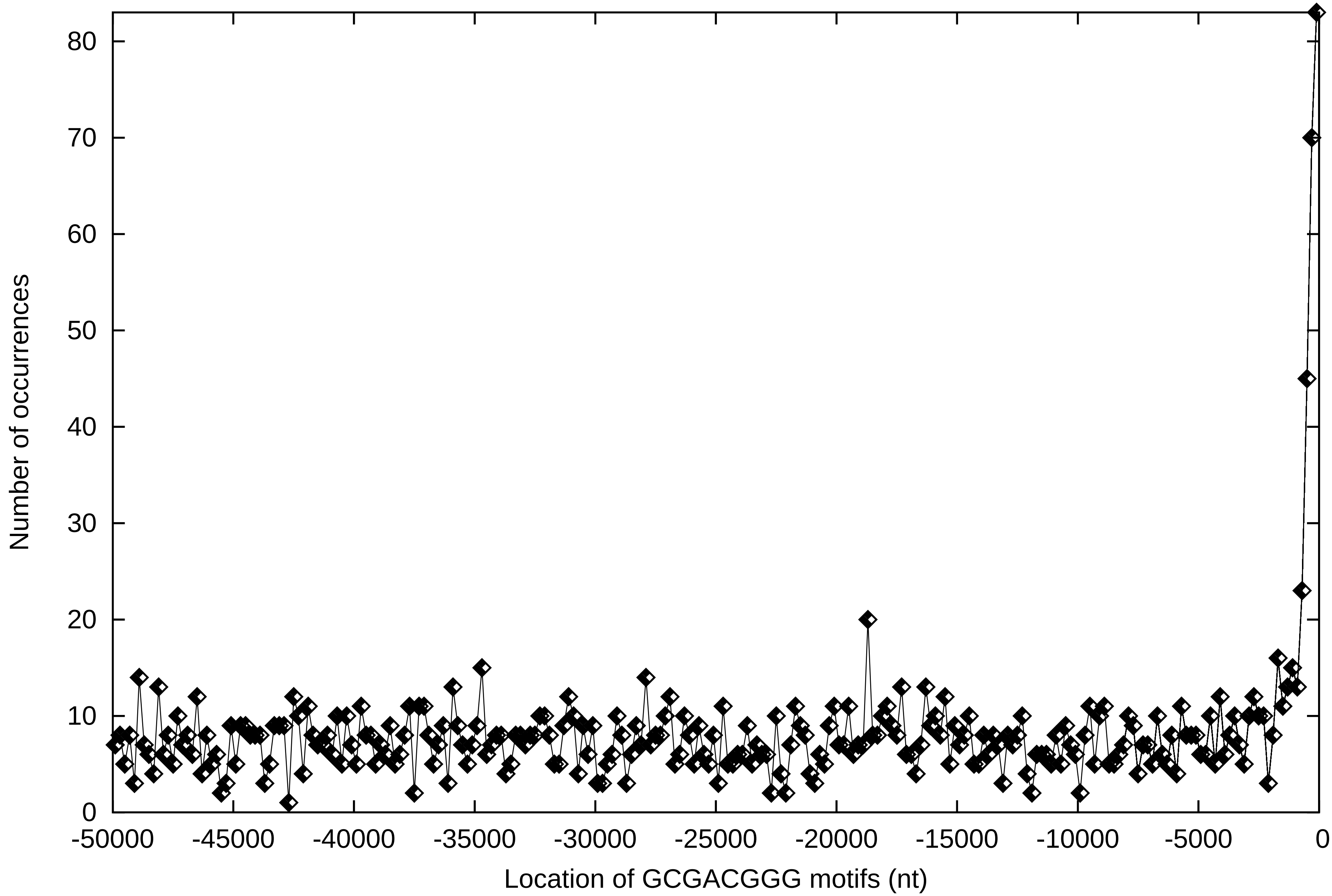

Number of occurrences
0
10
20
30
40
50
60
70
80
-50000
-45000
-40000
-35000
-30000
-25000
-20000
-15000
-10000
-5000
0
Location of GCGACGGG motifs (nt)

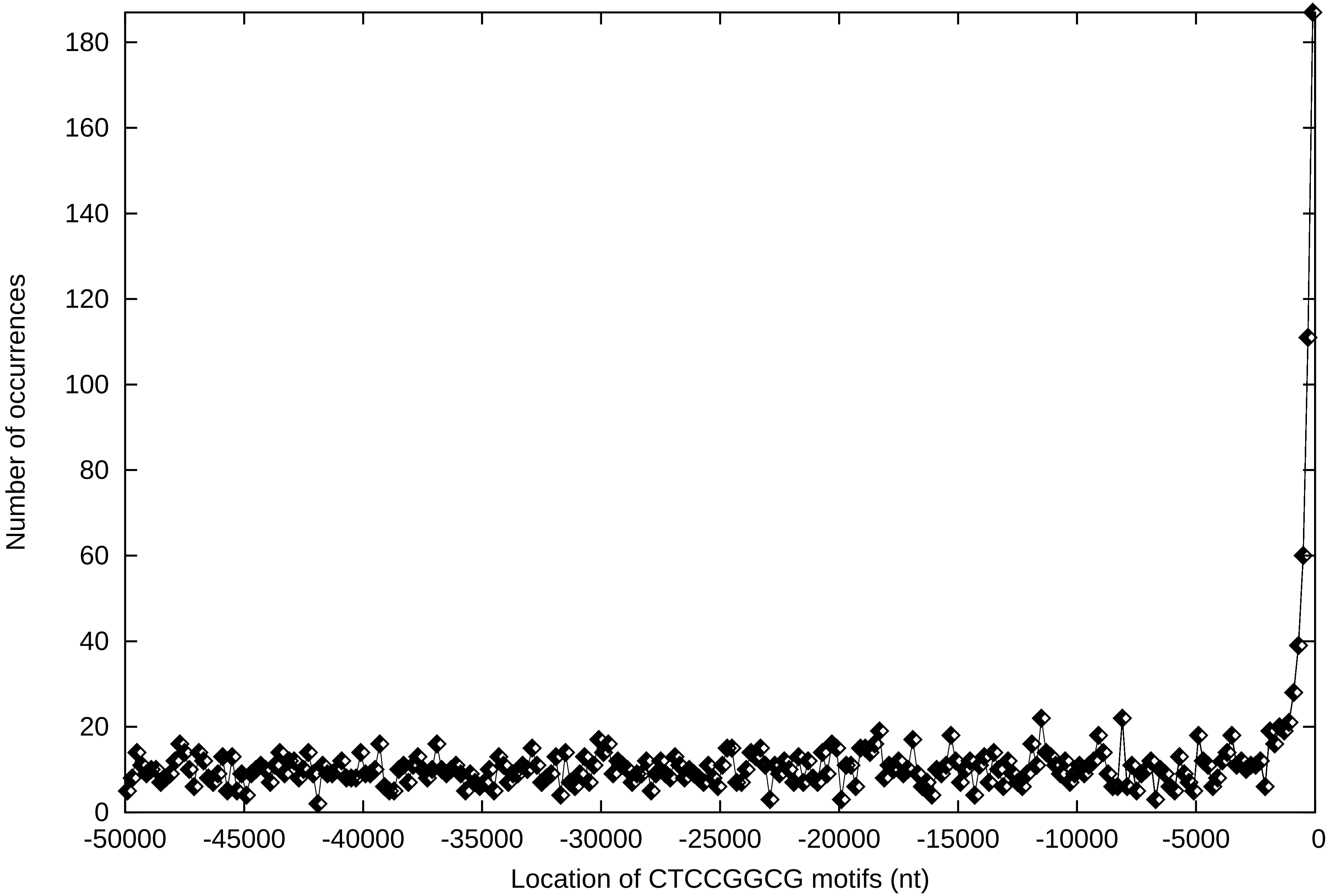

Number of occurrences
0
20
40
60
80
100
120
140
160
180
-50000
-45000
-40000
-35000
-30000
-25000
-20000
-15000
-10000
-5000
0
Location of CTCCGGCG motifs (nt)

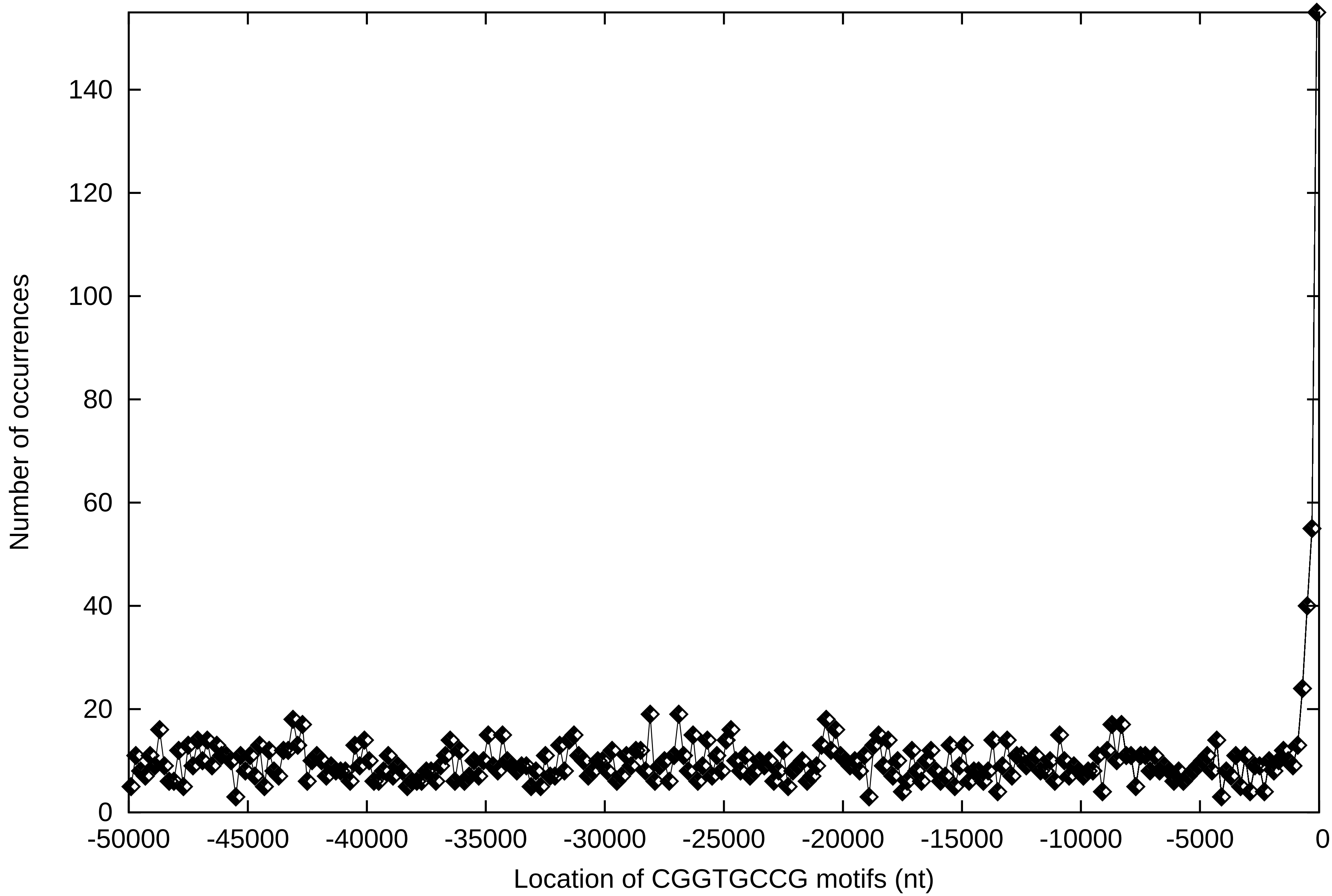

Number of occurrences
0
20
40
60
80
100
120
140
-50000
-45000
-40000
-35000
-30000
-25000
-20000
-15000
-10000
-5000
0
Location of CGGTGCCG motifs (nt)

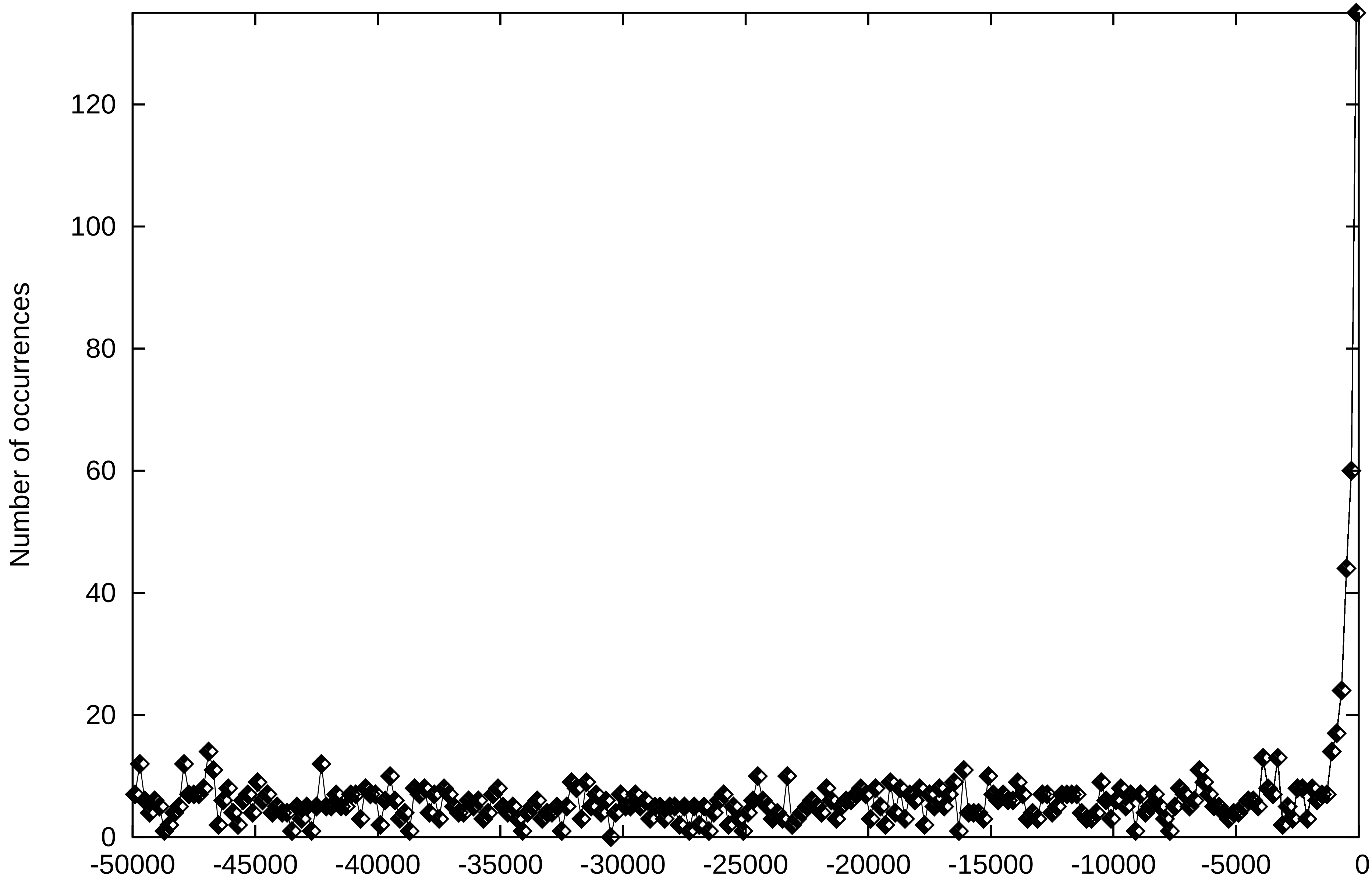

Number of occurrences
0
20
40
60
80
100
120
-50000
-45000
-40000
-35000
-30000
-25000
-20000
-15000
-10000
-5000
0
Location of CGGCGACG motifs (nt)

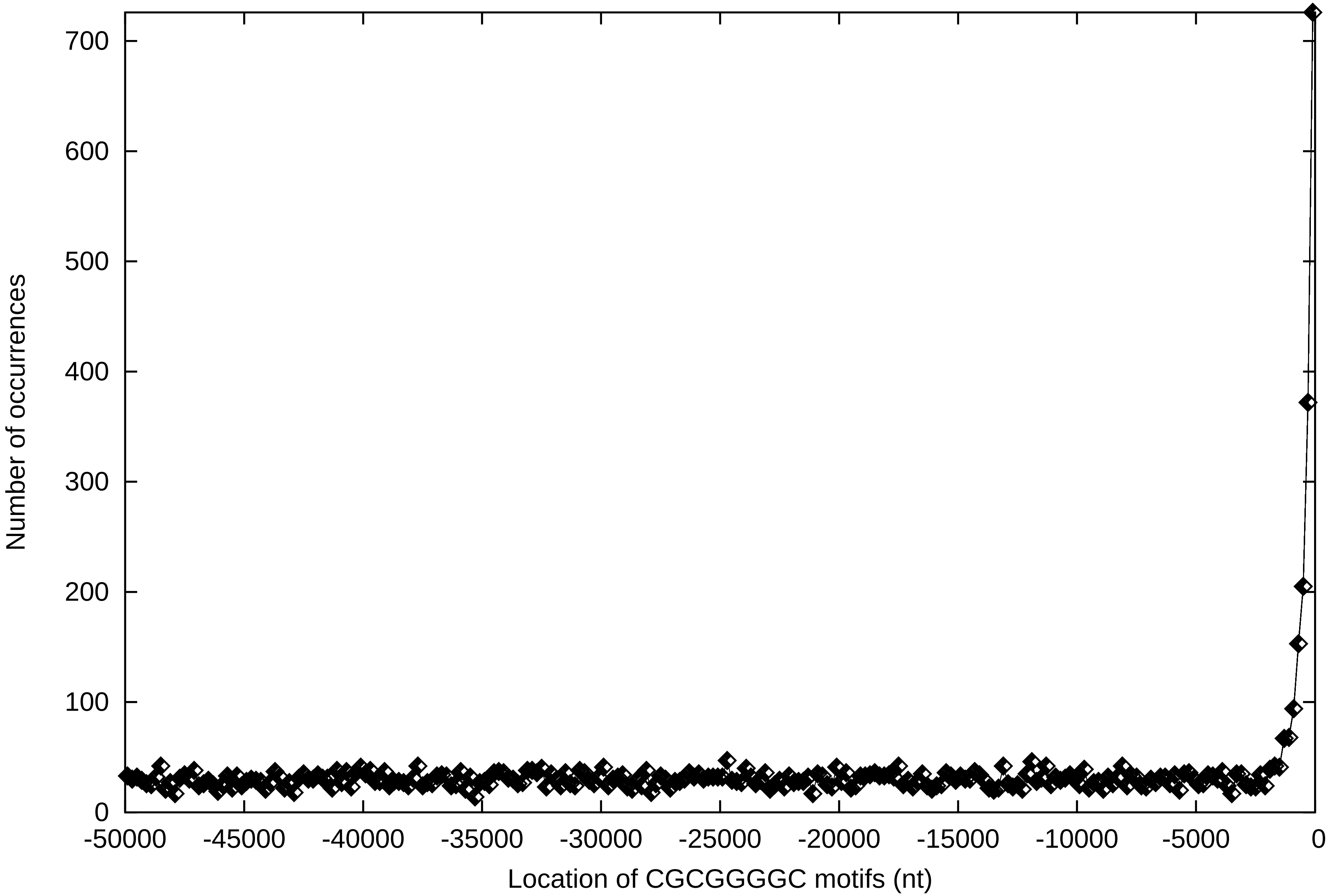

Number of occurrences
0
100
200
300
400
500
600
700
-50000
-45000
-40000
-35000
-30000
-25000
-20000
-15000
-10000
-5000
0
Location of CGCGGGGC motifs (nt)

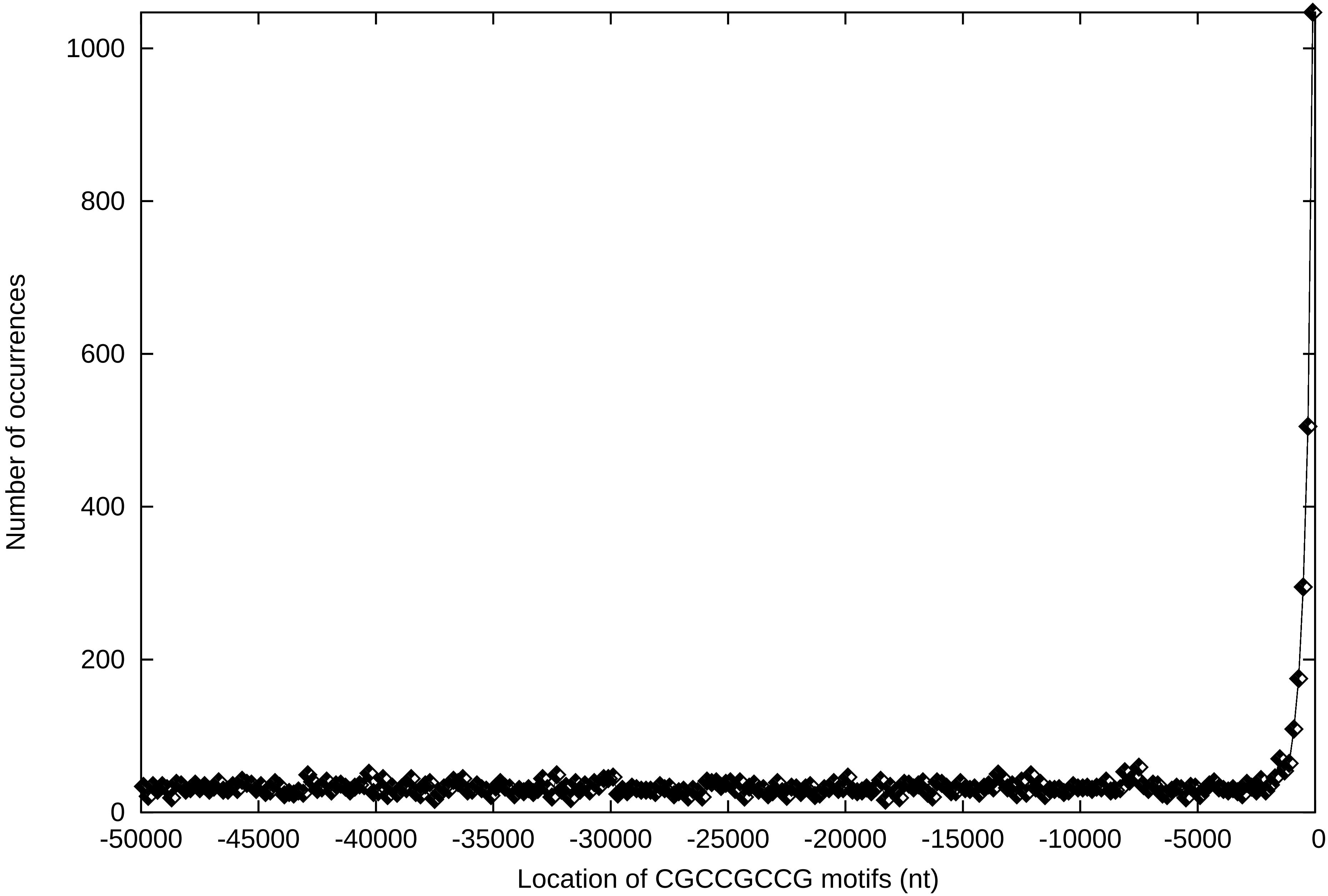

Number of occurrences
1000
800
600
400
200
0
-50000
-45000
-40000
-35000
-30000
-25000
-20000
-15000
-10000
-5000
0
Location of CGCCGCCG motifs (nt)

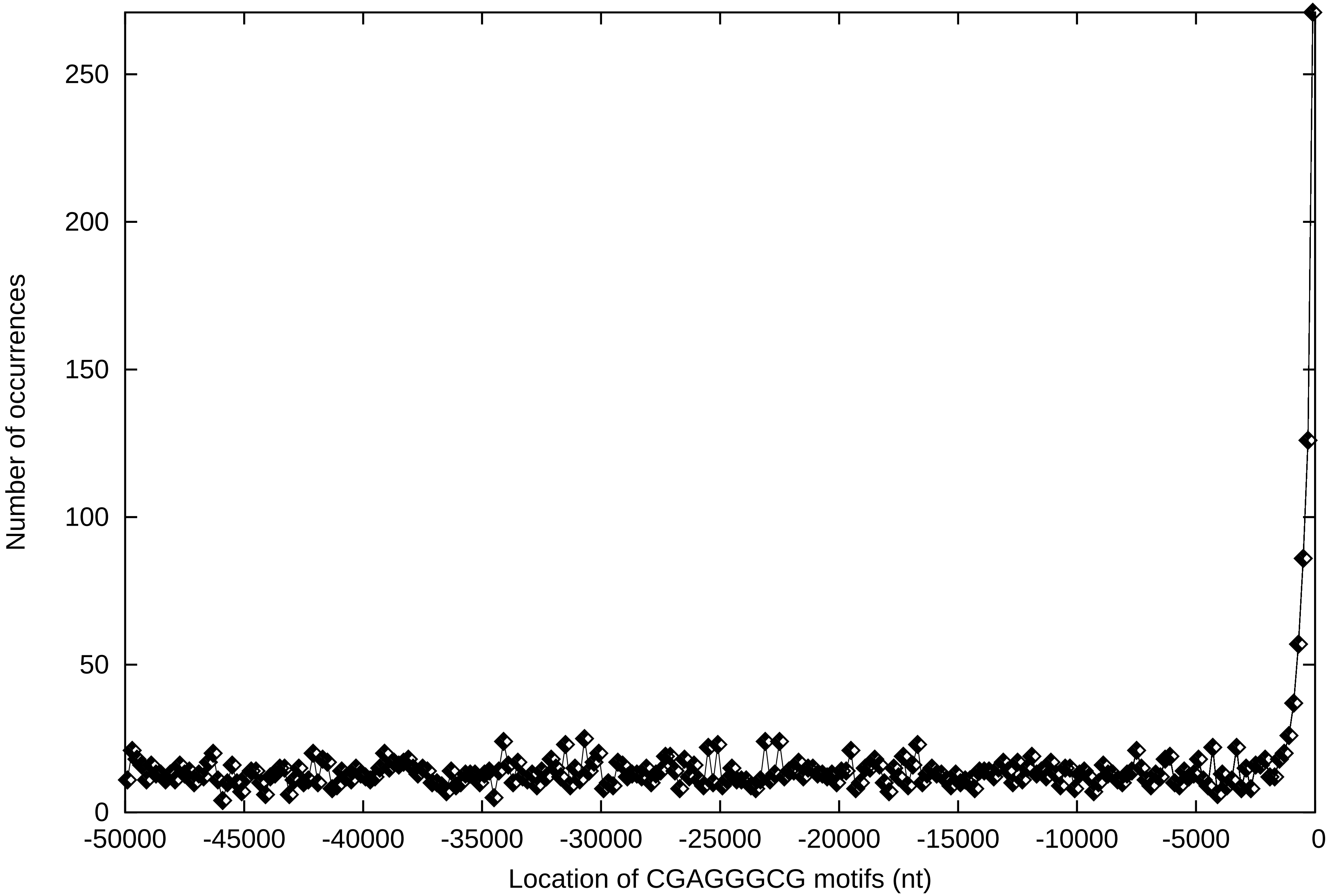

Number of occurrences
250
200
150
100
50
0
-50000
-45000
-40000
-35000
-30000
-25000
-20000
-15000
-10000
-5000
0
Location of CGAGGGCG motifs (nt)

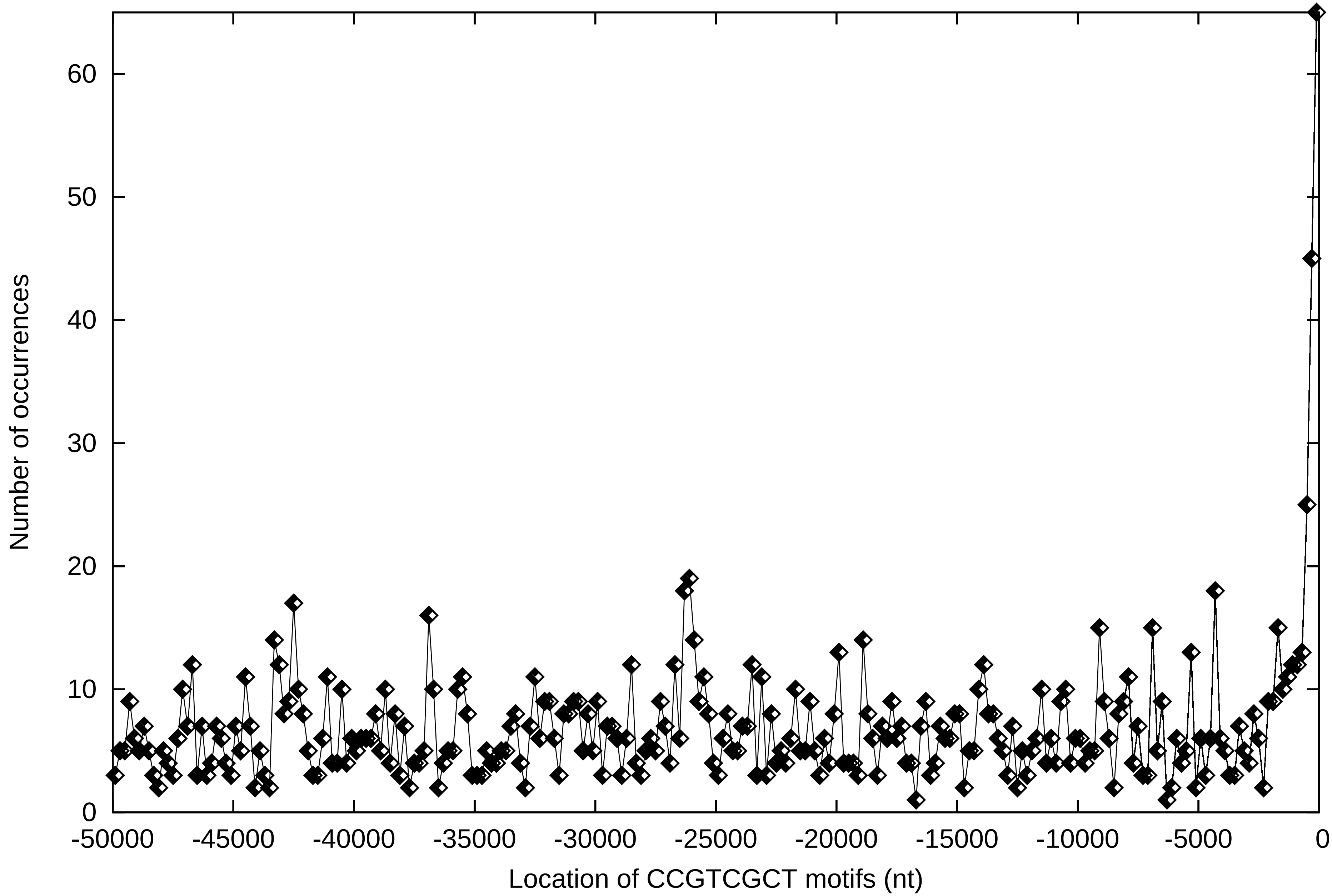

Number of occurrences
0
10
20
30
40
50
60
-50000
-45000
-40000
-35000
-30000
-25000
-20000
-15000
-10000
-5000
0
Location of CCGTCGCT motifs (nt)

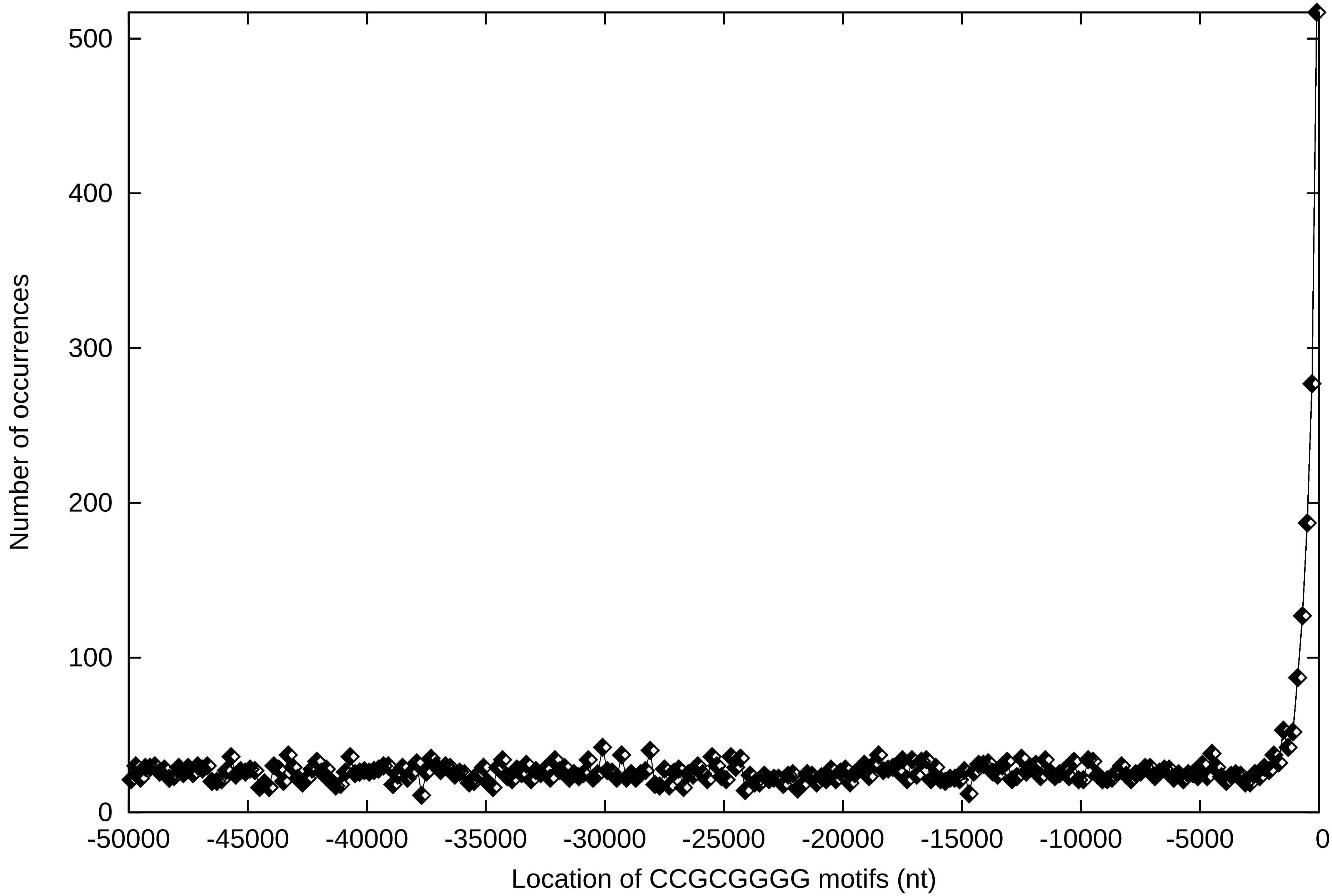

Number of occurrences
500
400
300
200
100
0
-50000
-45000
-40000
-35000
-30000
-25000
-20000
-15000
-10000
-5000
0
Location of CCGCGGGG motifs (nt)

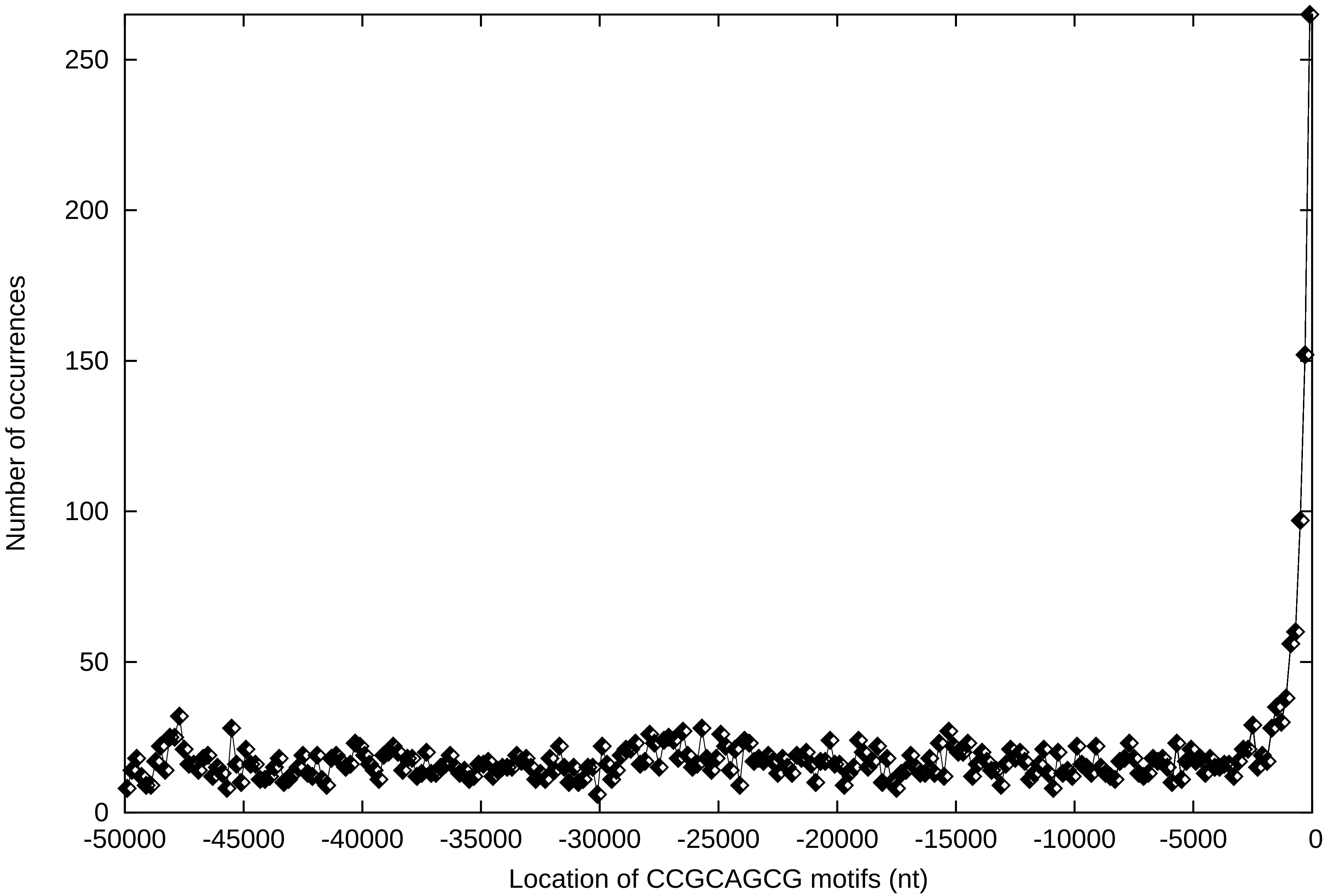

250
200
150
100
50
0
-50000
-45000
-40000
-35000
-30000
-25000
-20000
-15000
-10000
-5000
0
Number of occurrences
Location of CCGCAGCG motifs (nt)

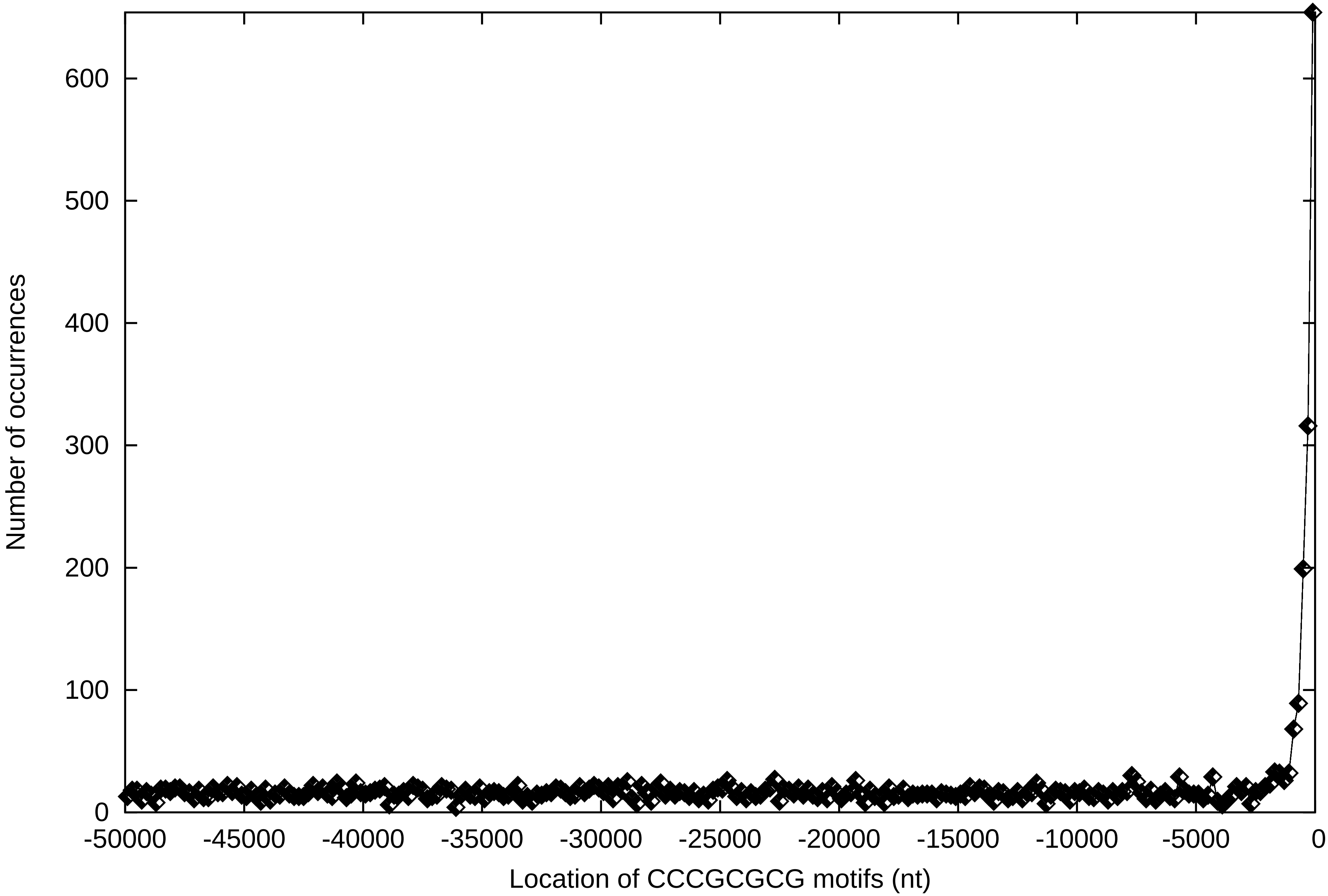
Number of occurrences
600
500
400
300
200
100
0
-50000
-45000
-40000
-35000
-30000
-25000
-20000
-15000
-10000
-5000
0
Location of CCCGCGCG motifs (nt)

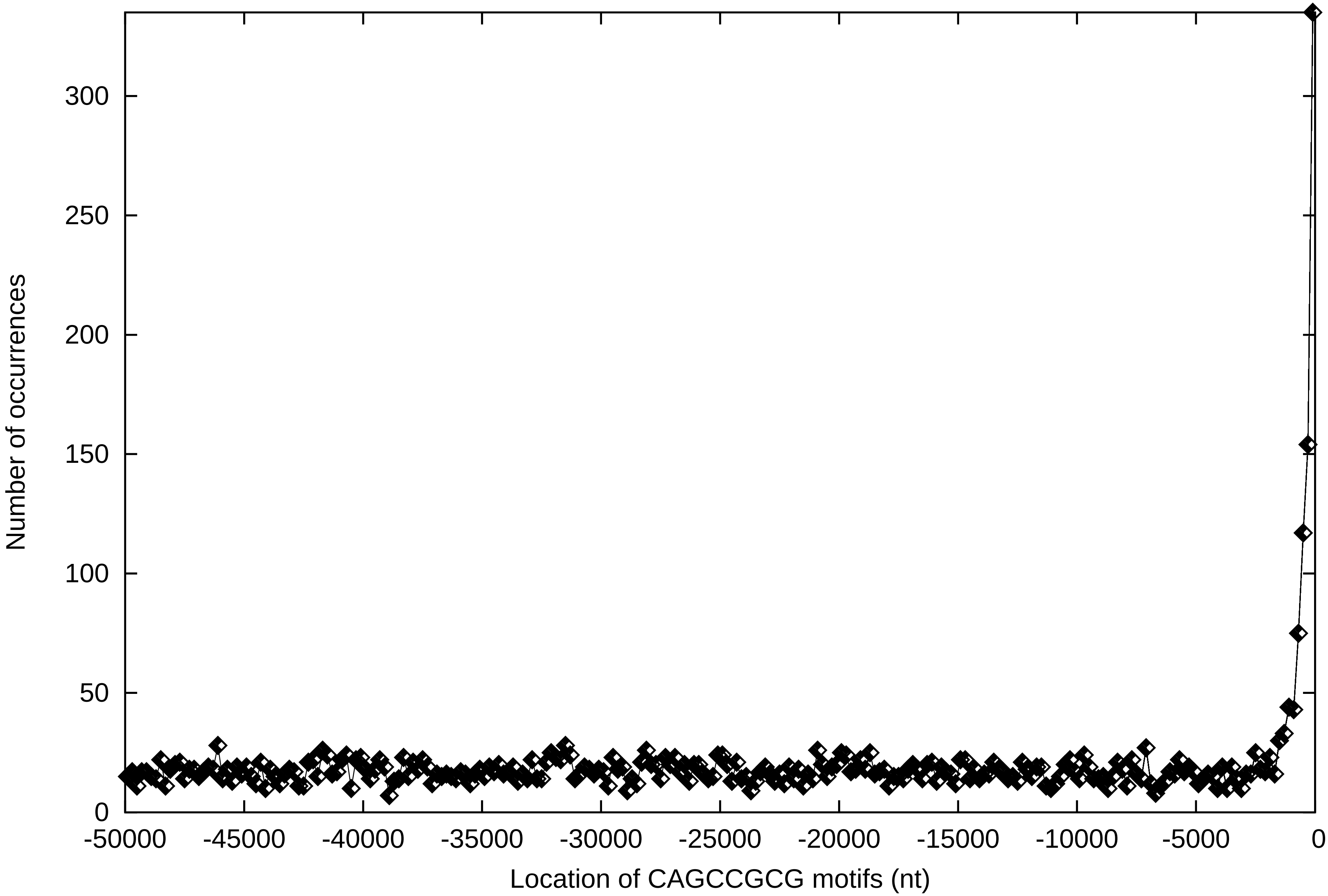

Number of occurrences
0
50
100
150
200
250
300
-50000
-45000
-40000
-35000
-30000
-25000
-20000
-15000
-10000
-5000
0
Location of CAGCCGCG motifs (nt)

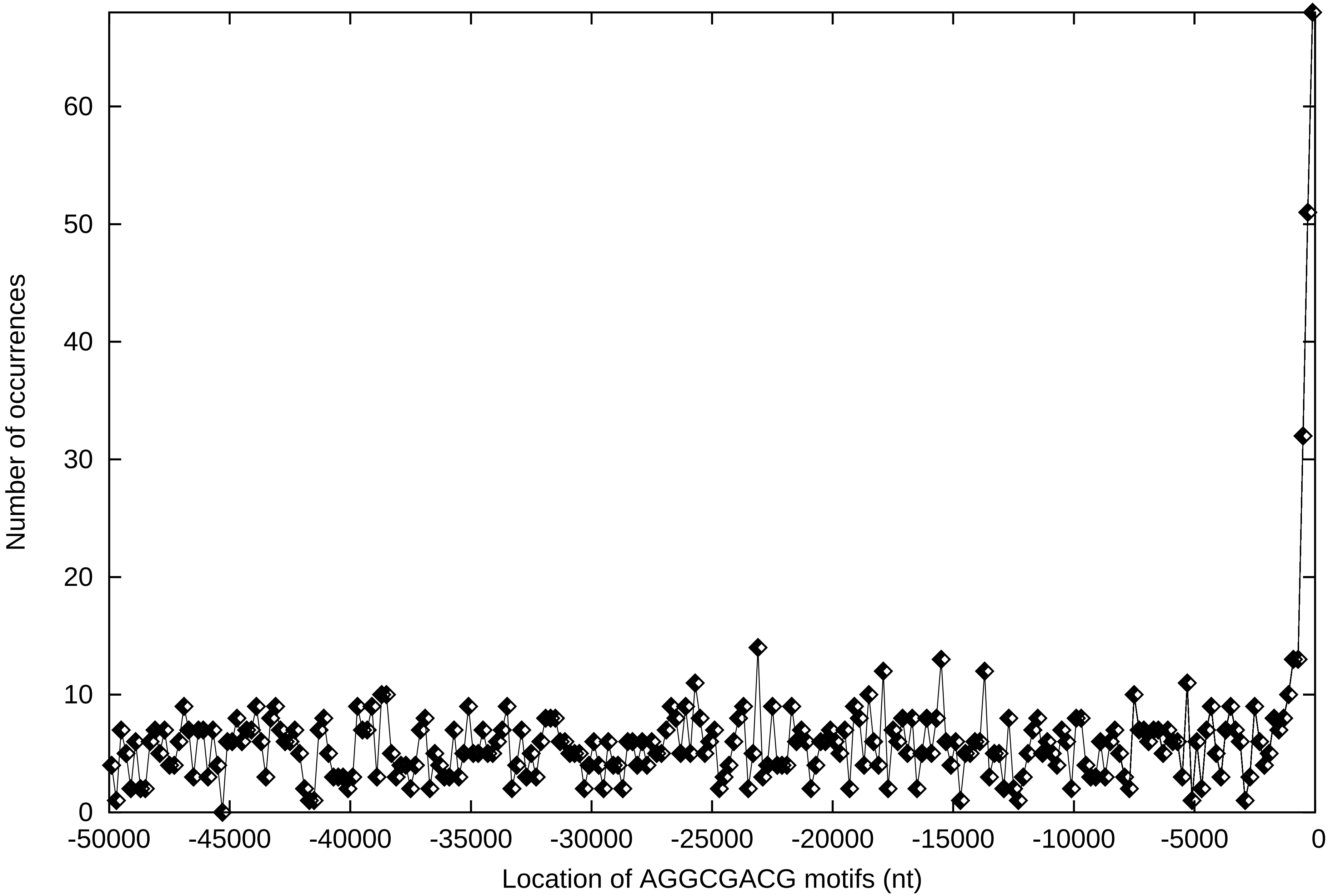

Number of occurrences
0
10
20
30
40
50
60
-50000
-45000
-40000
-35000
-30000
-25000
-20000
-15000
-10000
-5000
0
Location of AGGCGACG motifs (nt)

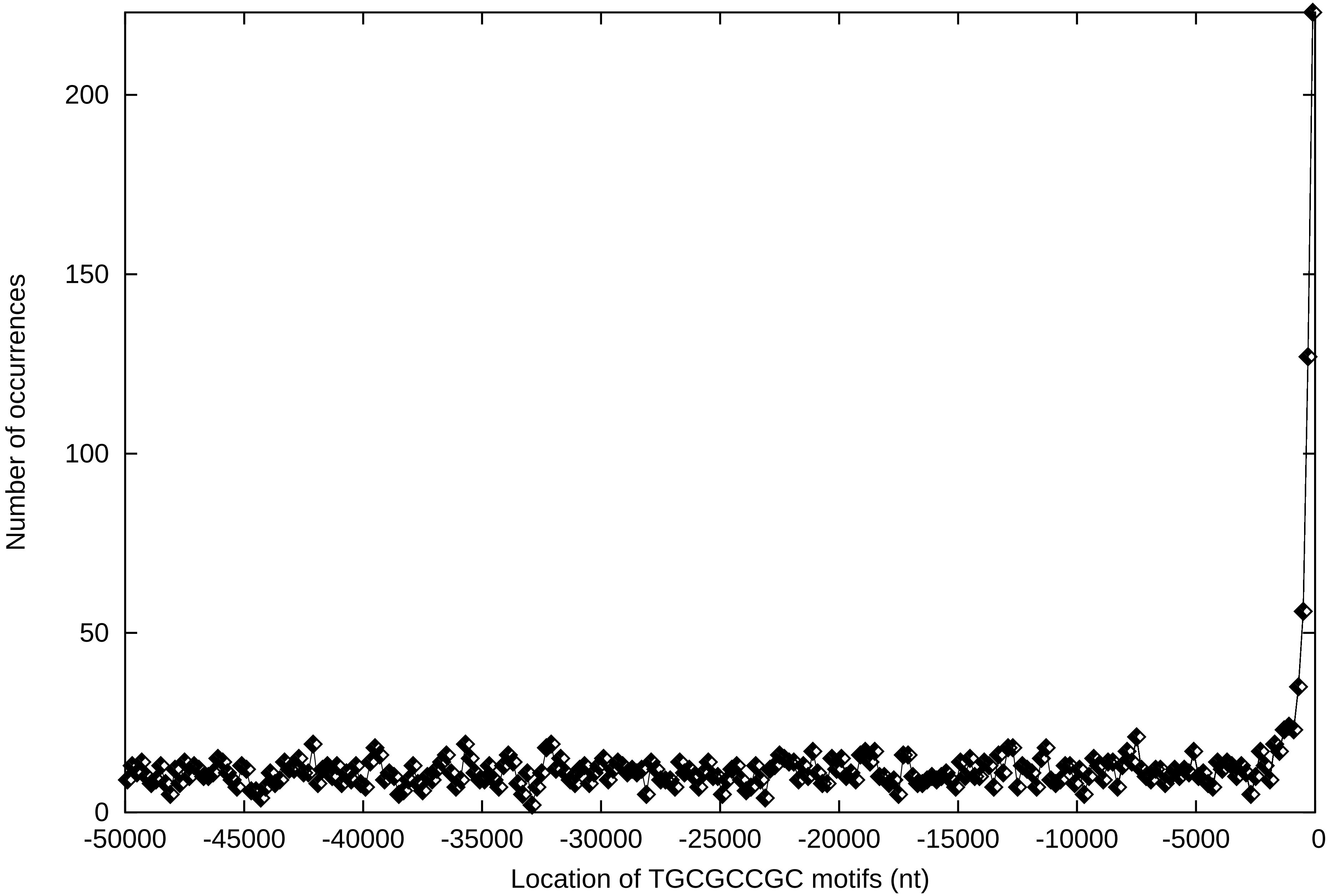

Number of occurrences
0
50
100
150
200
-50000
-45000
-40000
-35000
-30000
-25000
-20000
-15000
-10000
-5000
0
Location of TGCGCCGC motifs (nt)

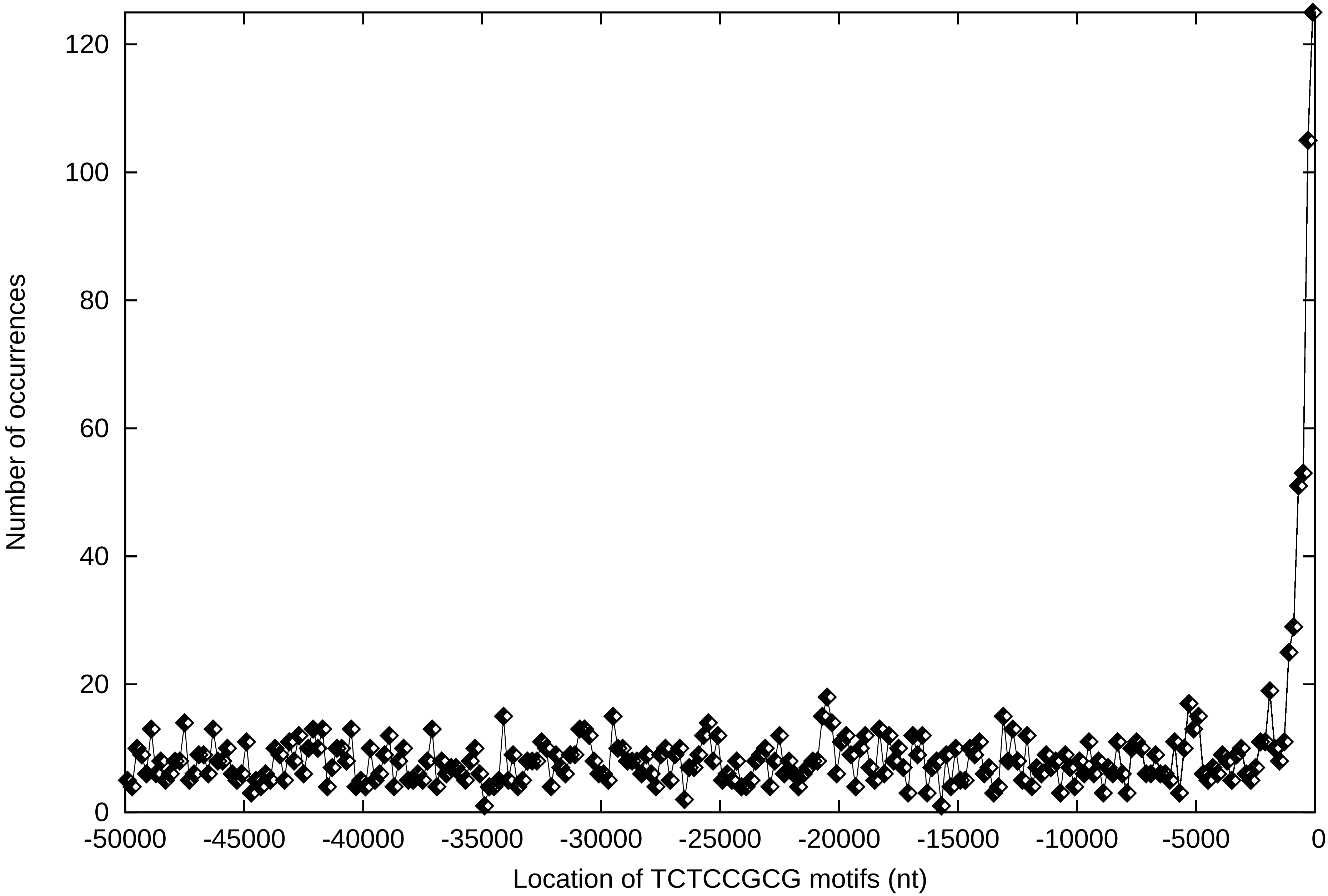

Number of occurrences
120
100
80
60
40
20
0
-50000
-45000
-40000
-35000
-30000
-25000
-20000
-15000
-10000
-5000
0
Location of TCTCCGCG motifs (nt)

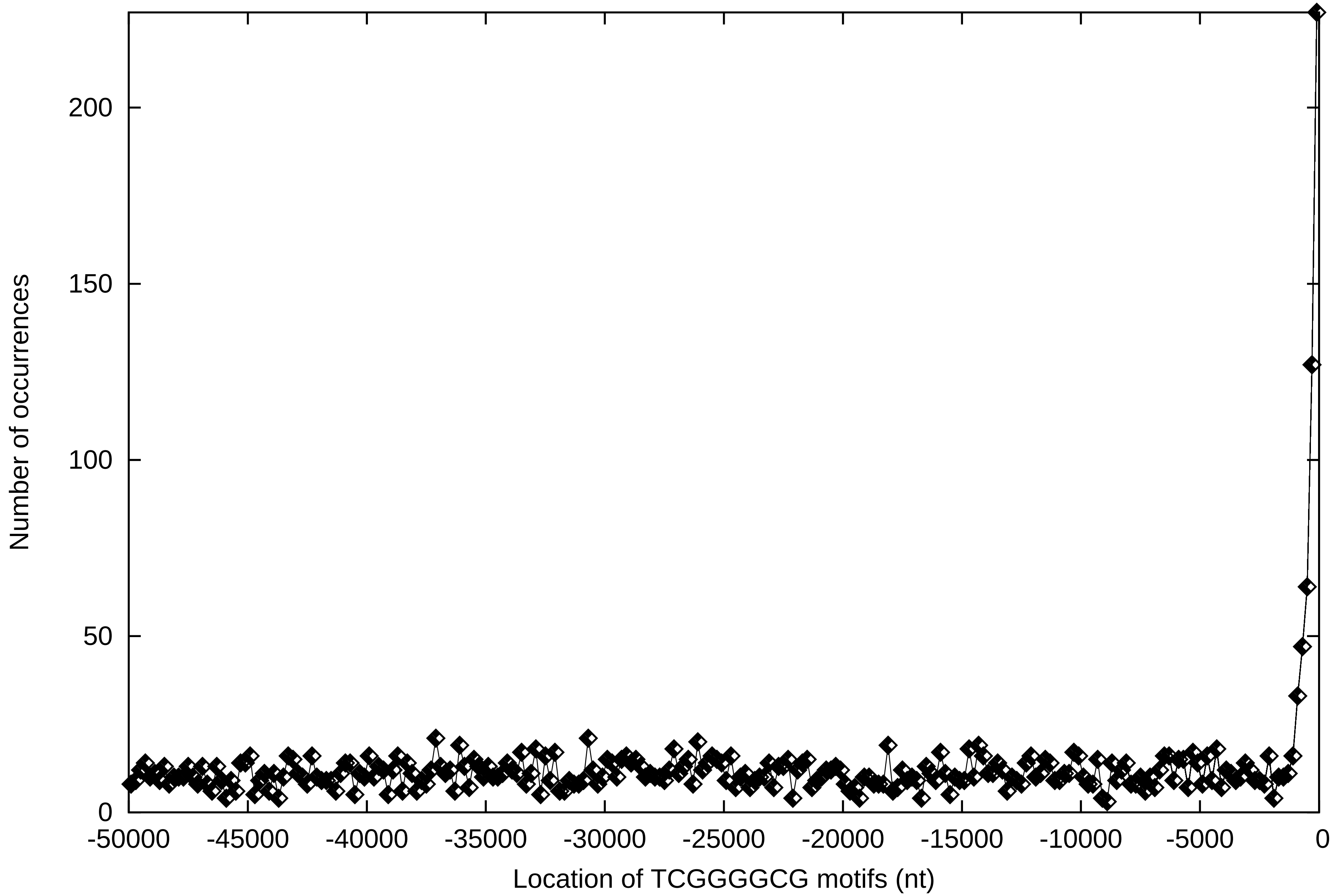

200
150
100
50
0
Number of occurrences
-50000
-45000
-40000
-35000
-30000
-25000
-20000
-15000
-10000
-5000
0
Location of TCGGGGCG motifs (nt)

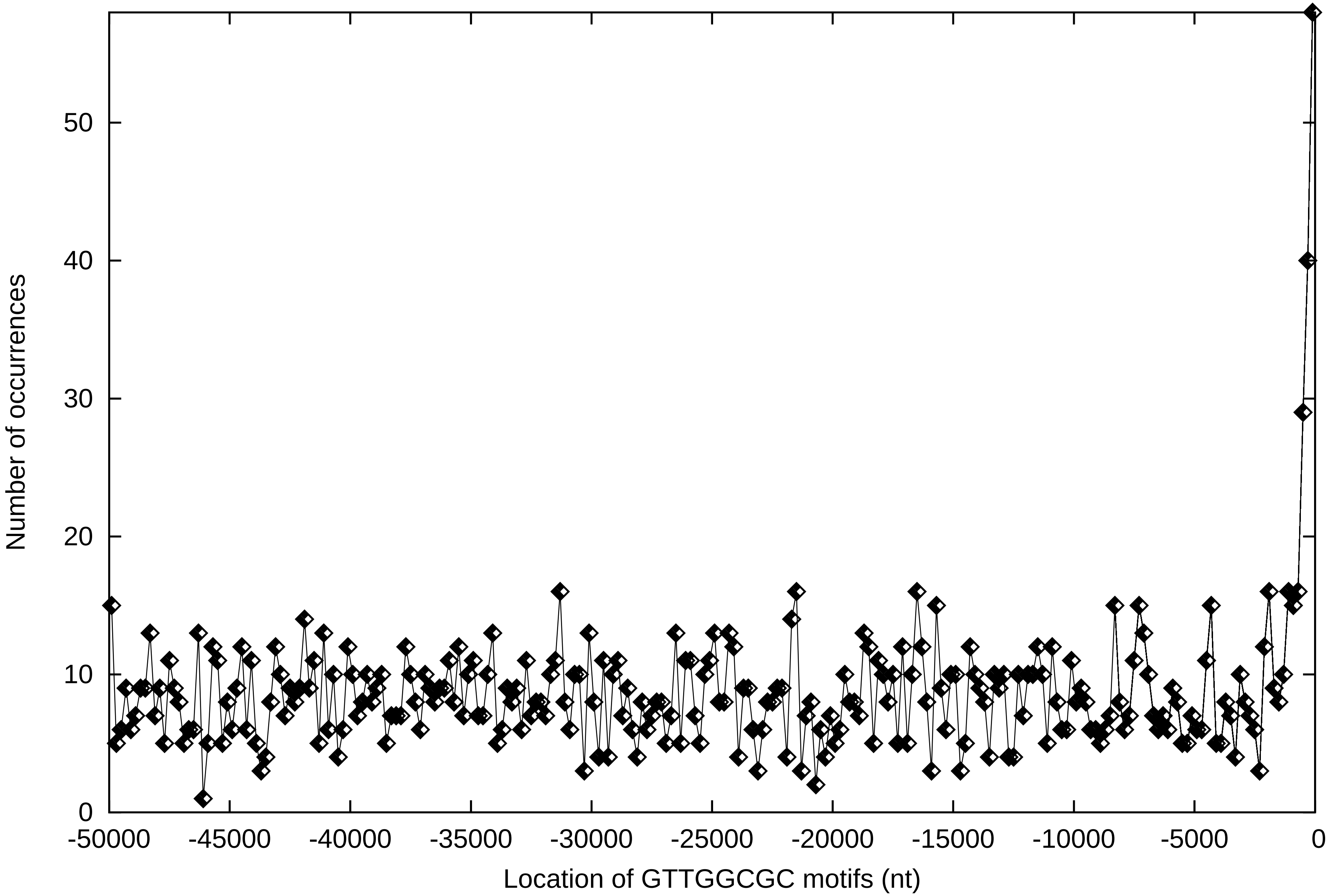

Number of occurrences
0
10
20
30
40
50
-50000
-45000
-40000
-35000
-30000
-25000
-20000
-15000
-10000
-5000
0
Location of GTTGGCGC motifs (nt)

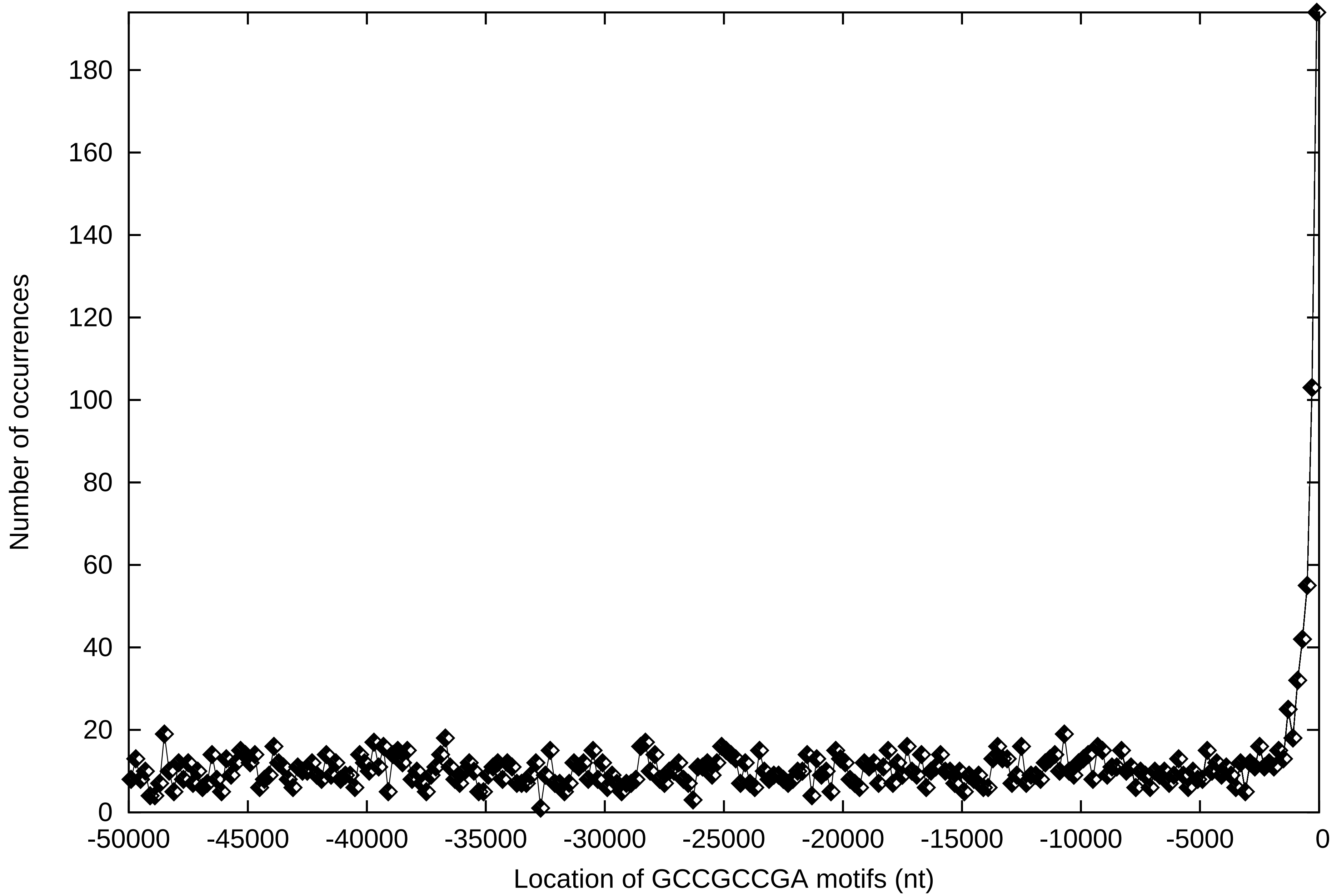
Number of occurrences
0
20
40
60
80
100
120
140
160
180
-50000
-45000
-40000
-35000
-30000
-25000
-20000
-15000
-10000
-5000
0
Location of GCCGCCGA motifs (nt)

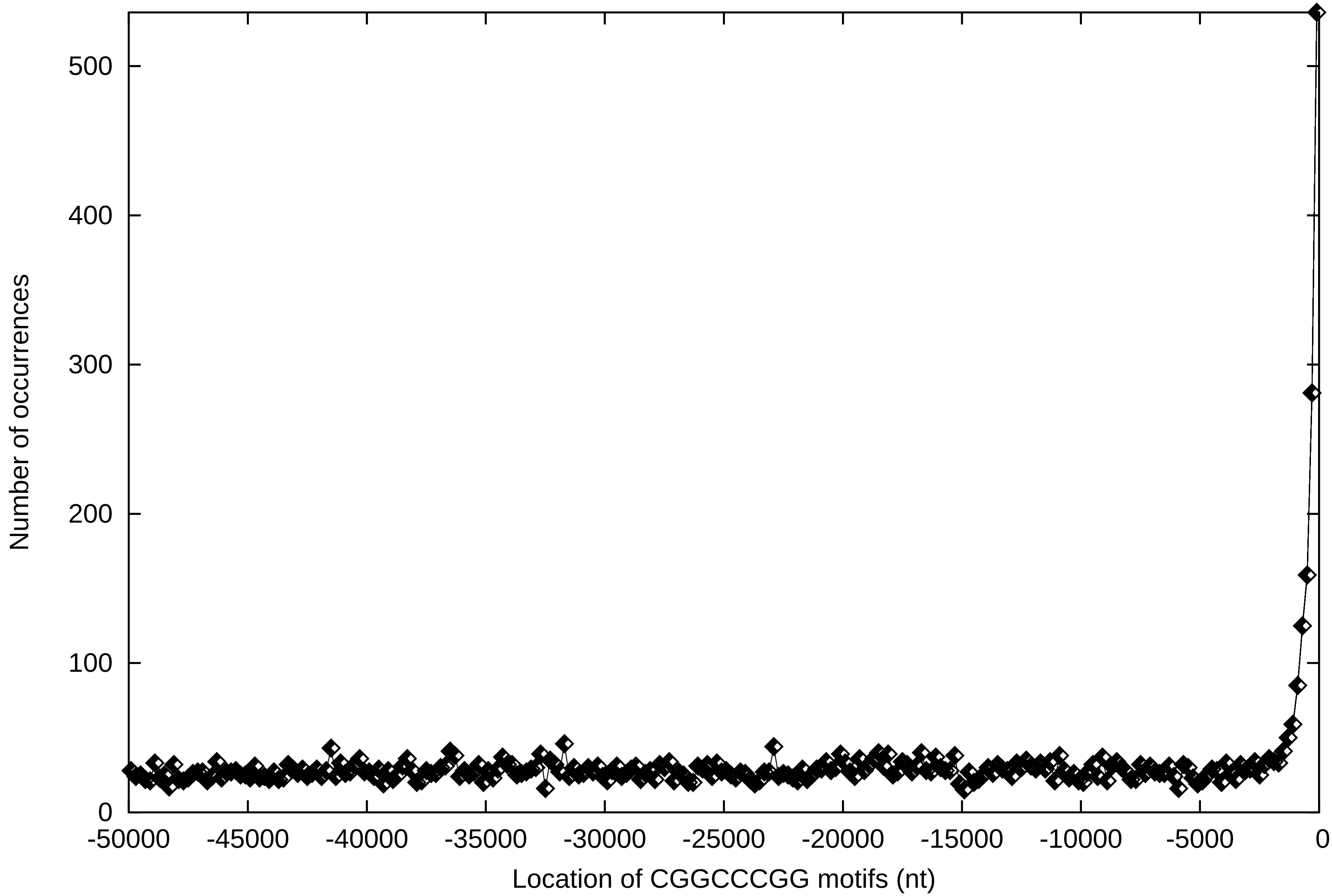

Number of occurrences
0
100
200
300
400
500
-50000
-45000
-40000
-35000
-30000
-25000
-20000
-15000
-10000
-5000
0
Location of CGGCCCGG motifs (nt)

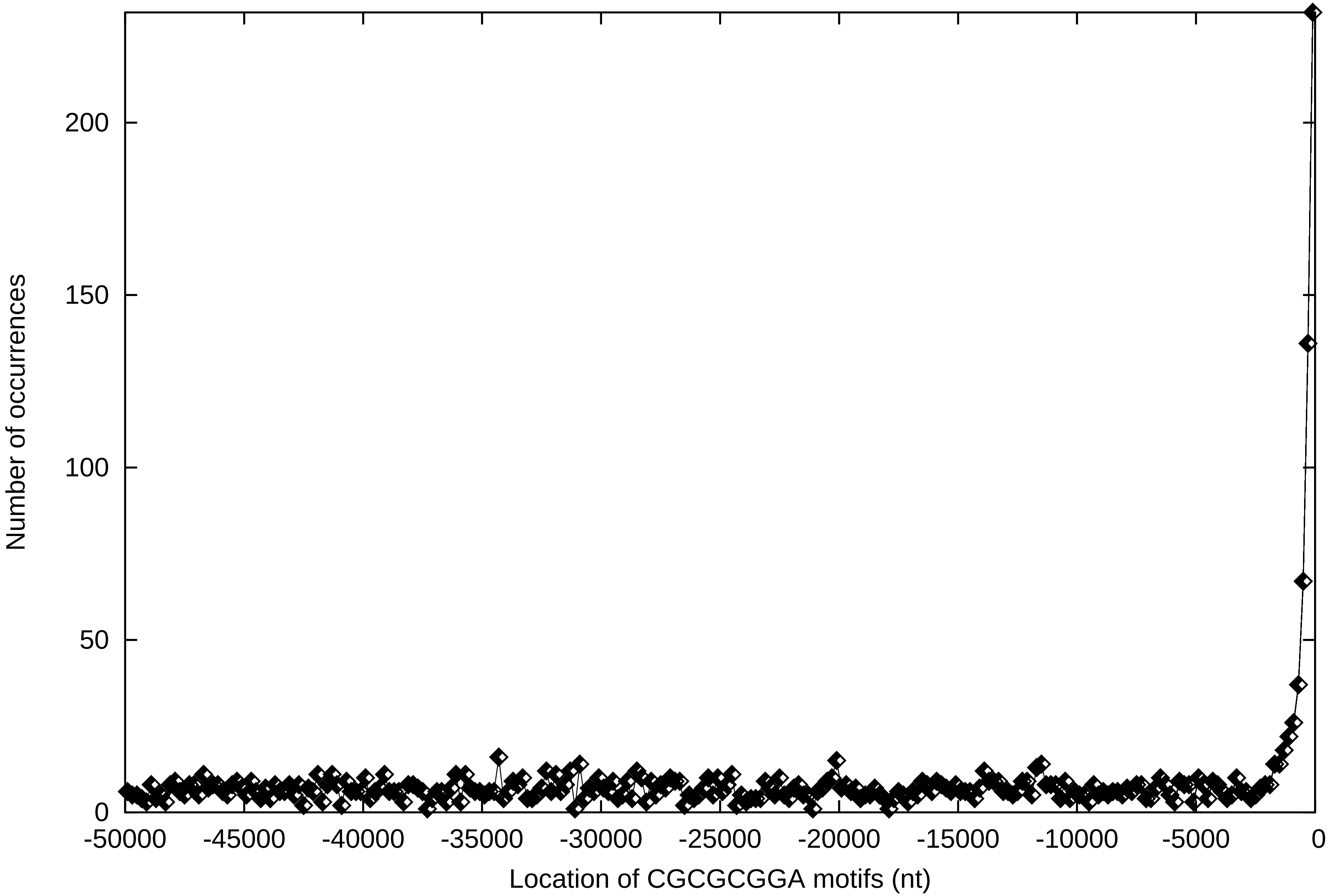

Number of occurrences
0
50
100
150
200
-50000
-45000
-40000
-35000
-30000
-25000
-20000
-15000
-10000
-5000
0
Location of CGCGCGGA motifs (nt)

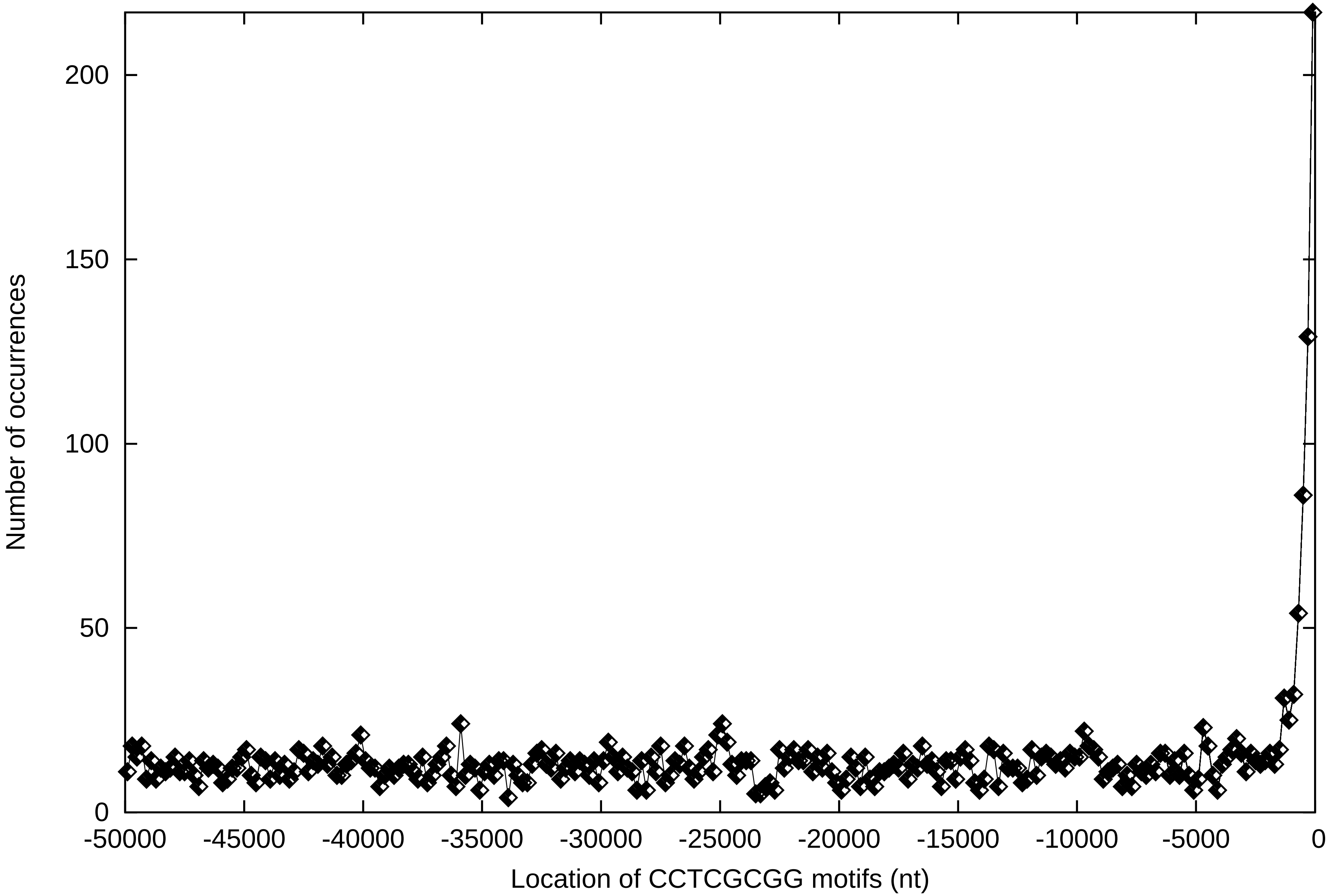

Number of occurrences
200
150
100
50
0
-50000
-45000
-40000
-35000
-30000
-25000
-20000
-15000
-10000
-5000
0
Location of CCTCGCGG motifs (nt)

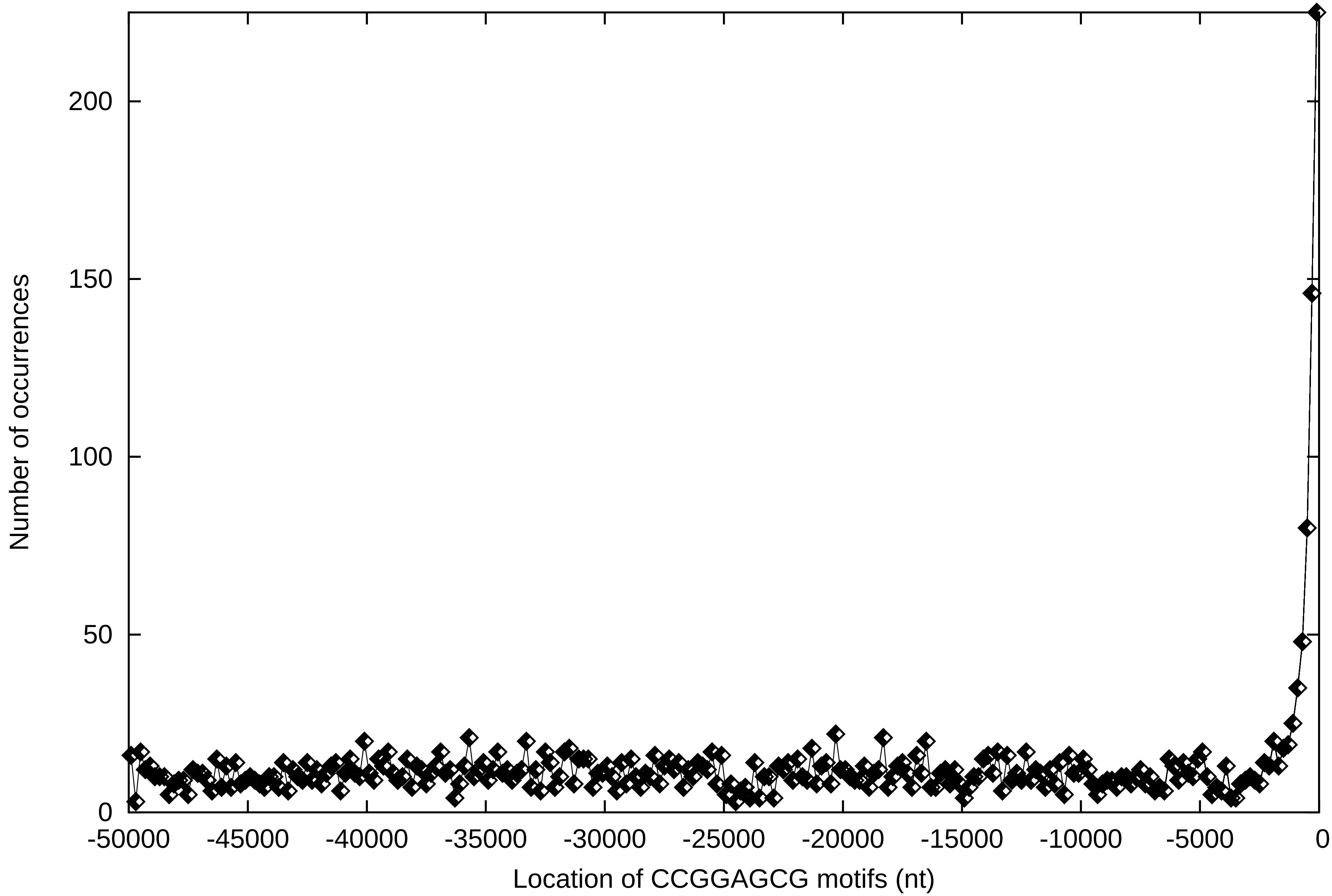
Number of occurrences
0
50
100
150
200
-50000
-45000
-40000
-35000
-30000
-25000
-20000
-15000
-10000
-5000
0
Location of CCGGAGCG motifs (nt)

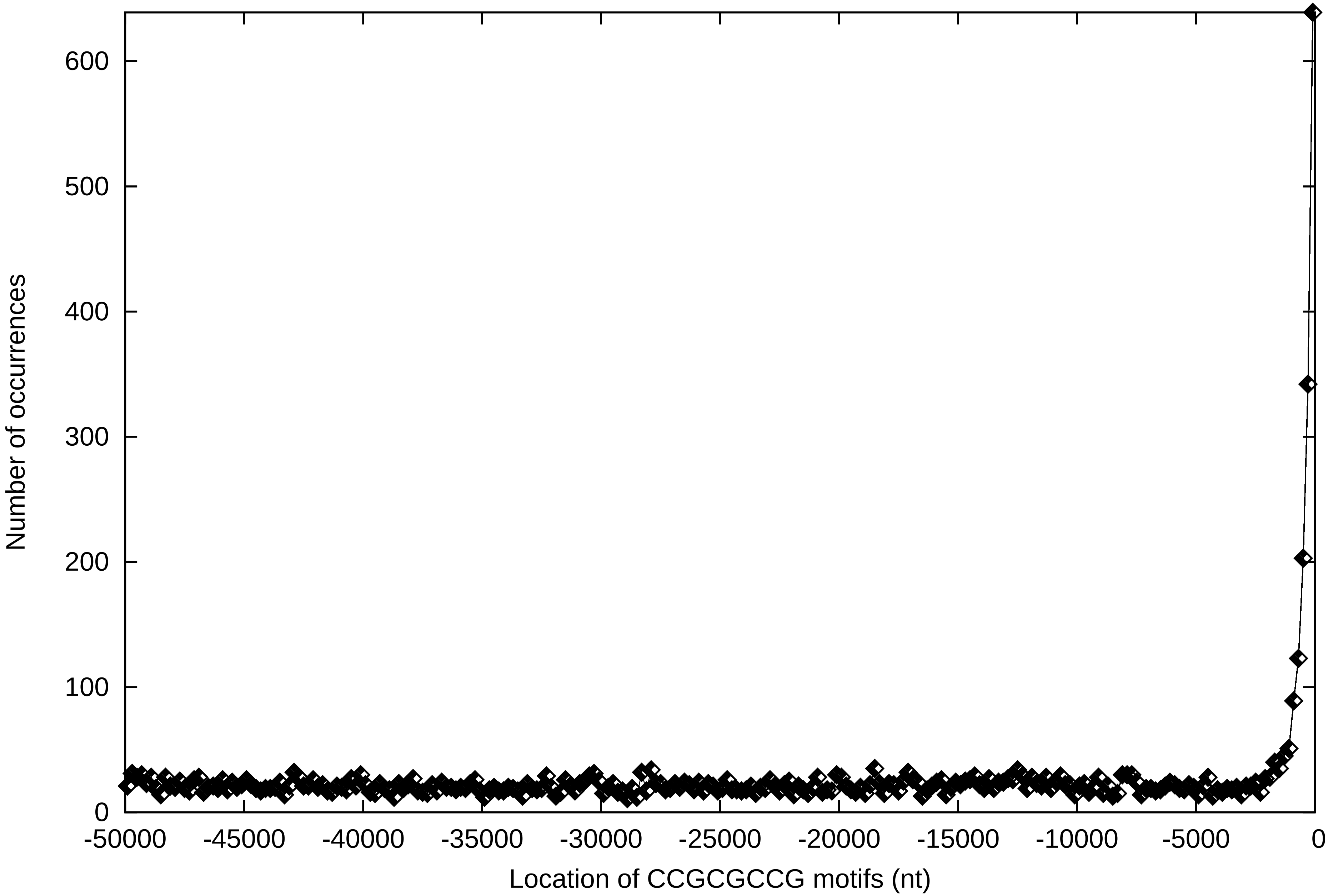
Number of occurrences
Location of CCGCGCCG motifs (nt)
0
100
200
300
400
500
600
-50000
-45000
-40000
-35000
-30000
-25000
-20000
-15000
-10000
-5000
0
