# Supplementary material for: Regulatory Circuit of Human MicroRNA Biogenesis
Source: PLoS Comput Biol. 2007 Apr 20;3(4):e67. doi: 10.1371/journal.pcbi.0030067 (PMC1853126; doi:10.1371/journal.pcbi.0030067)
Supplement: Supplementary file 3 [file pcbi.0030067.sg003.pdf]

The 100 top-predicted 7-mers are preferentially located upstream of protein-coding genes (pages 2-101). Each graph is a histogram that represents the number of occurrences of each motif (every 200 nt stretch) in the first 50 kb upstream region of protein-coding genes. The motifs preferentially occur within the first 500 nt region (-500 to 0 nt region) that is directly upstream of the protein-coding genes.

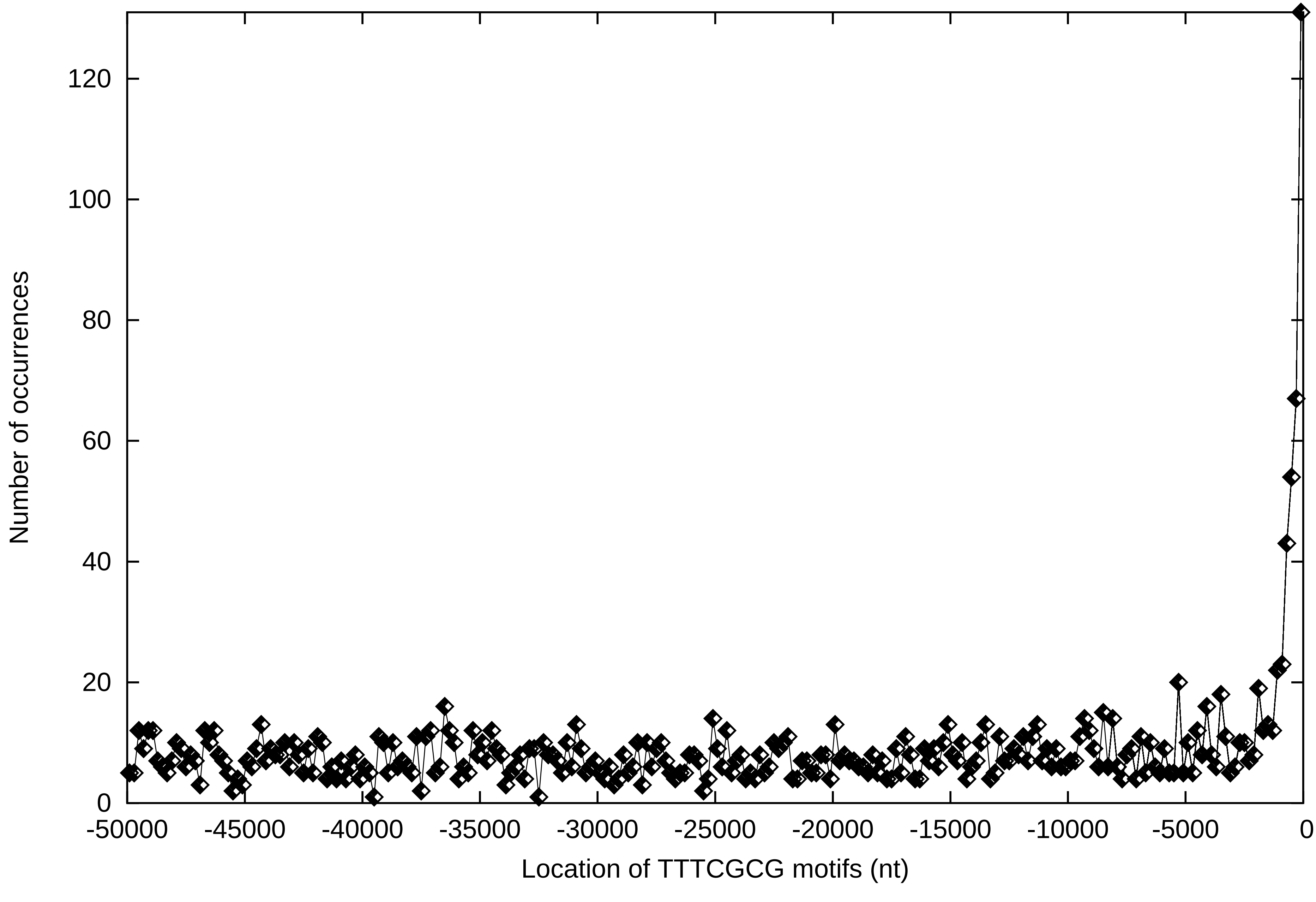

Number of occurrences
Location of TTTCGCG motifs (nt)
0
20
40
60
80
100
120
-50000
-45000
-40000
-35000
-30000
-25000
-20000
-15000
-10000
-5000
0

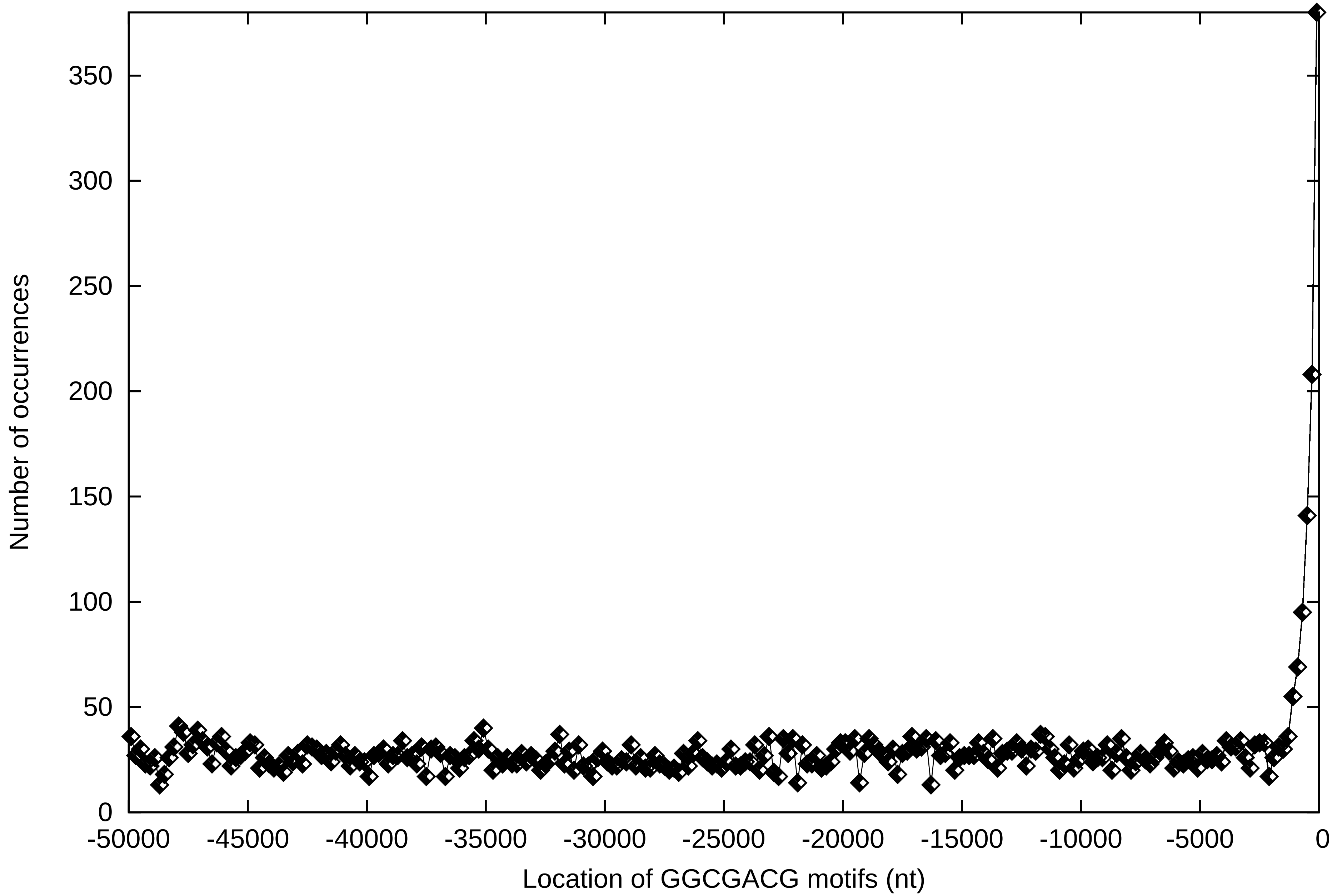

Number of occurrences
0
50
100
150
200
250
300
350
-50000
-45000
-40000
-35000
-30000
-25000
-20000
-15000
-10000
-5000
0
Location of GGCGACG motifs (nt)

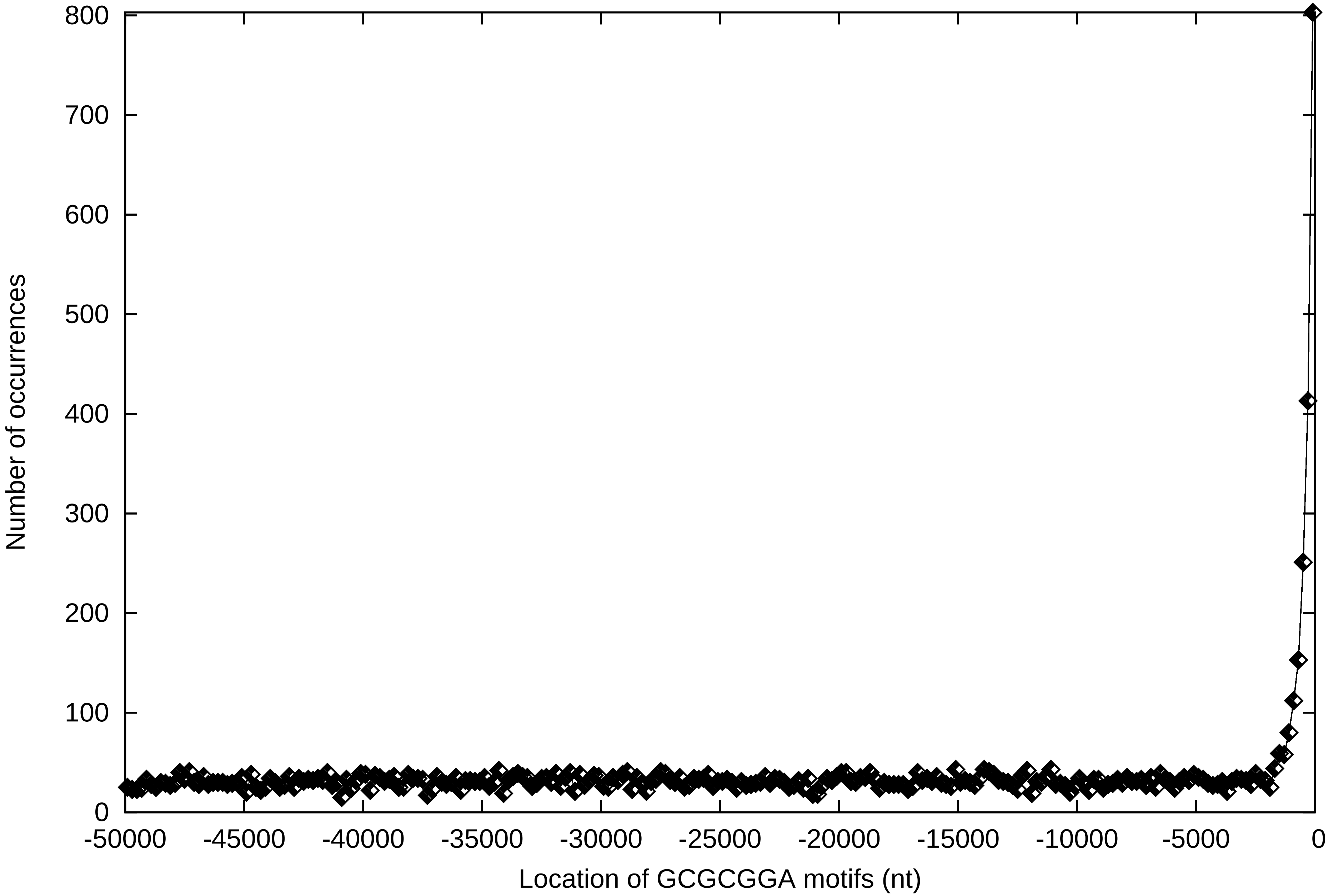

800
700
600
500
400
300
200
100
0
Number of occurrences
-50000
-45000
-40000
-35000
-30000
-25000
-20000
-15000
-10000
-5000
0
Location of GCGCGGA motifs (nt)

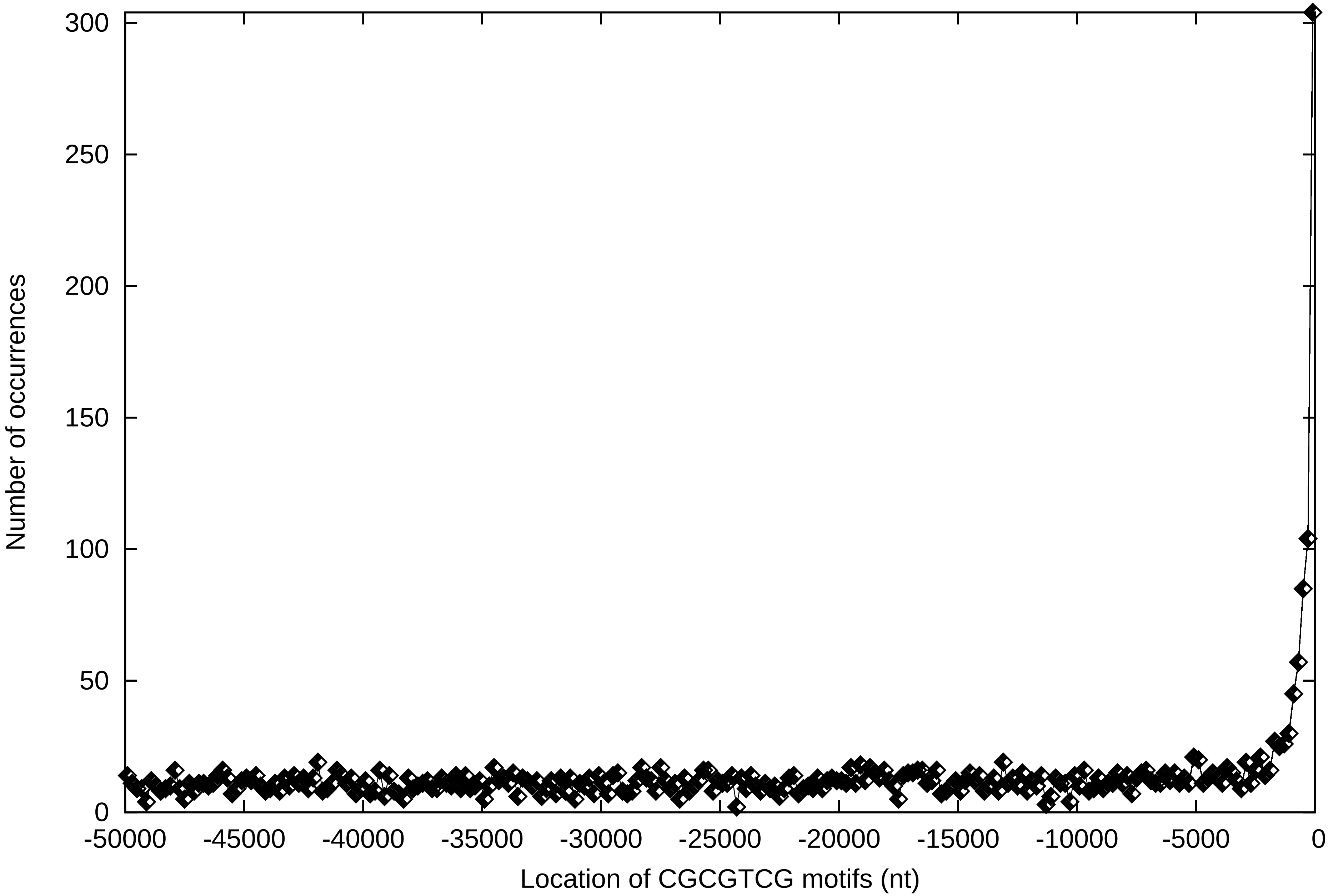

300
250
200
150
100
50
0
Number of occurrences
-50000
-45000
-40000
-35000
-30000
-25000
-20000
-15000
-10000
-5000
0
Location of CGCGTCG motifs (nt)

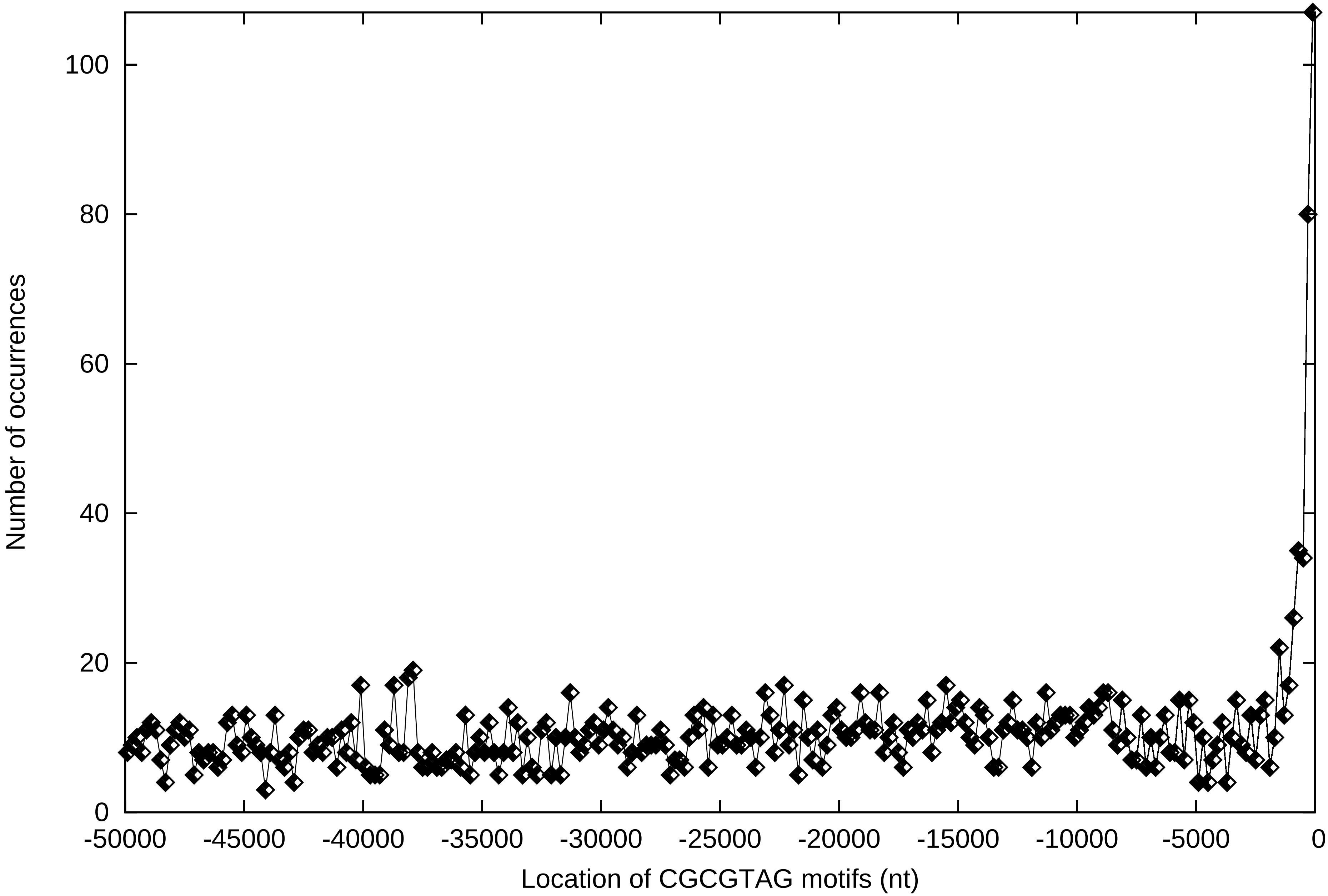

Number of occurrences
Location of CGCGTAG motifs (nt)
0
20
40
60
80
100
-50000
-45000
-40000
-35000
-30000
-25000
-20000
-15000
-10000
-5000
0

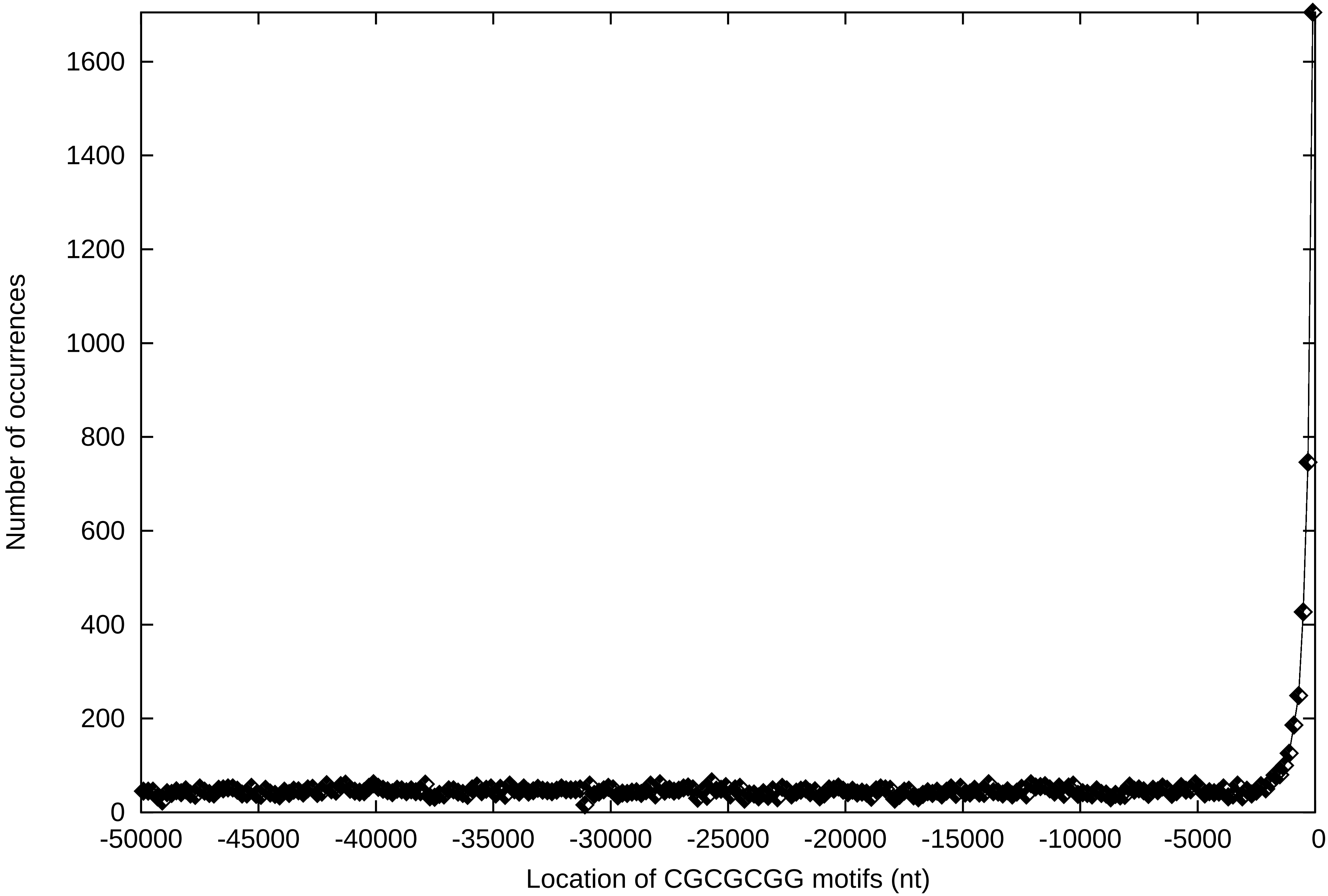
Number of occurrences
0
200
400
600
800
1000
1200
1400
1600
-50000
-45000
-40000
-35000
-30000
-25000
-20000
-15000
-10000
-5000
0
Location of CGCGCGG motifs (nt)

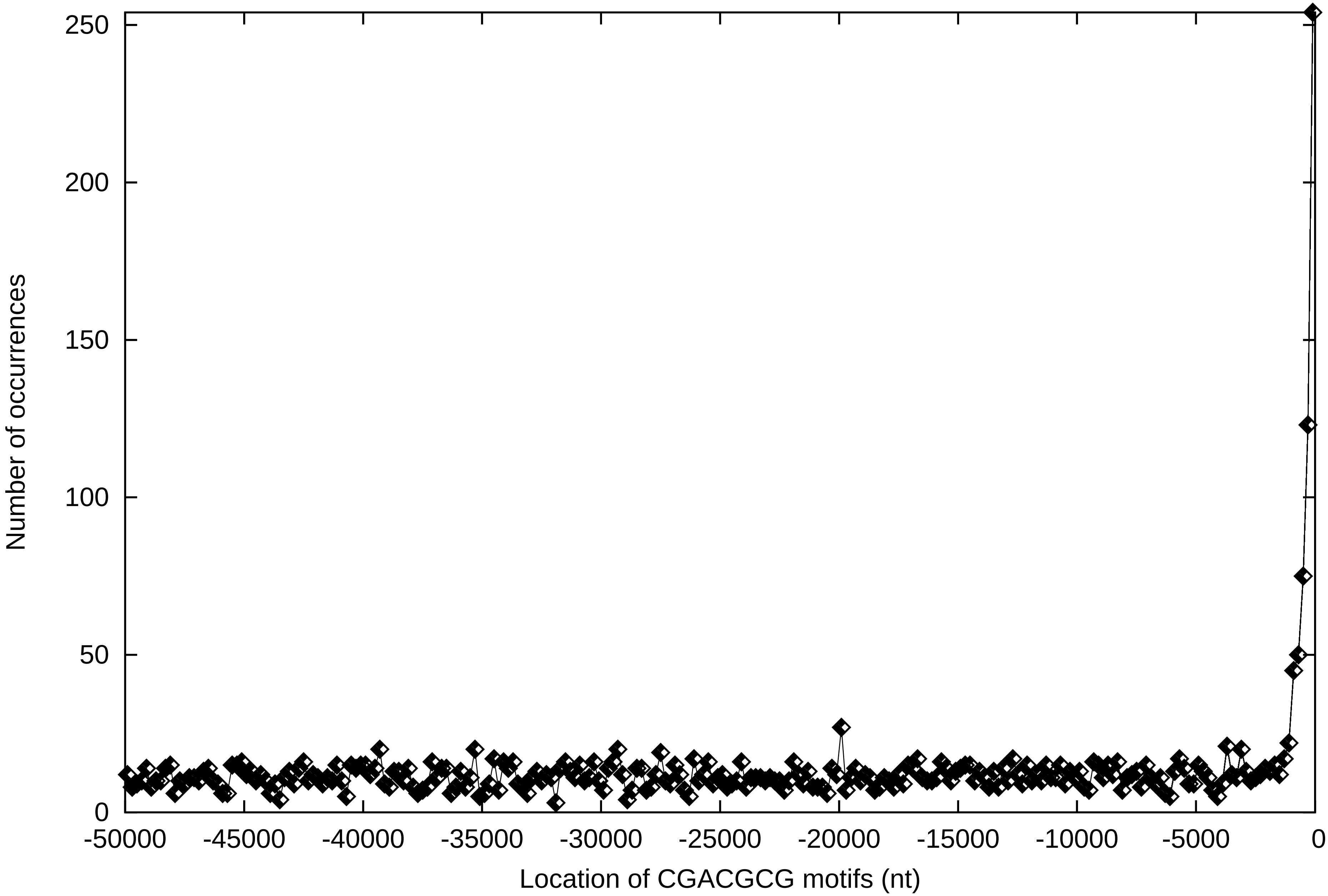

Number of occurrences
250
200
150
100
50
0
-50000
-45000
-40000
-35000
-30000
-25000
-20000
-15000
-10000
-5000
0
Location of CGACGCG motifs (nt)

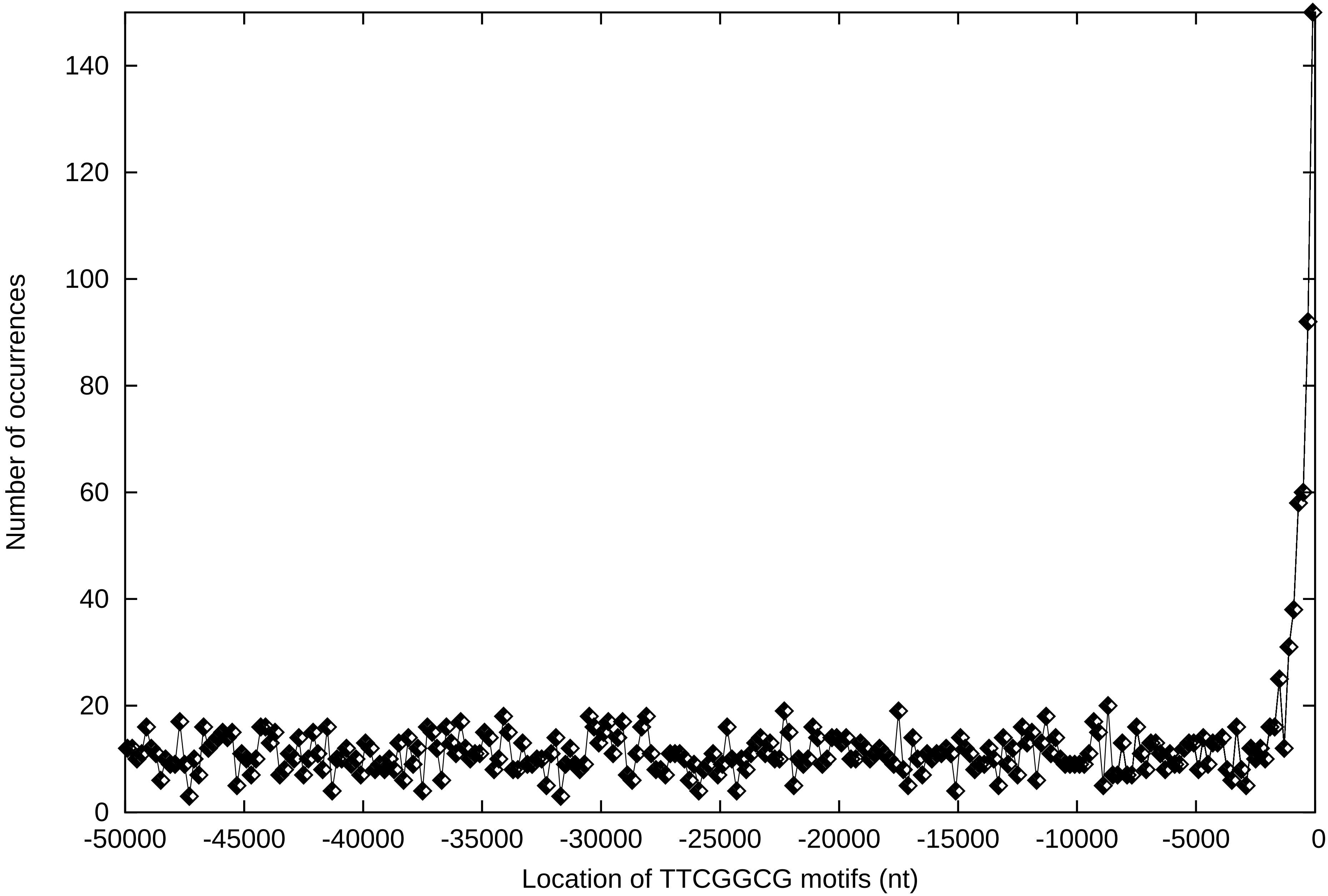

Number of occurrences
0
20
40
60
80
100
120
140
-50000
-45000
-40000
-35000
-30000
-25000
-20000
-15000
-10000
-5000
0
Location of TTCGGCG motifs (nt)

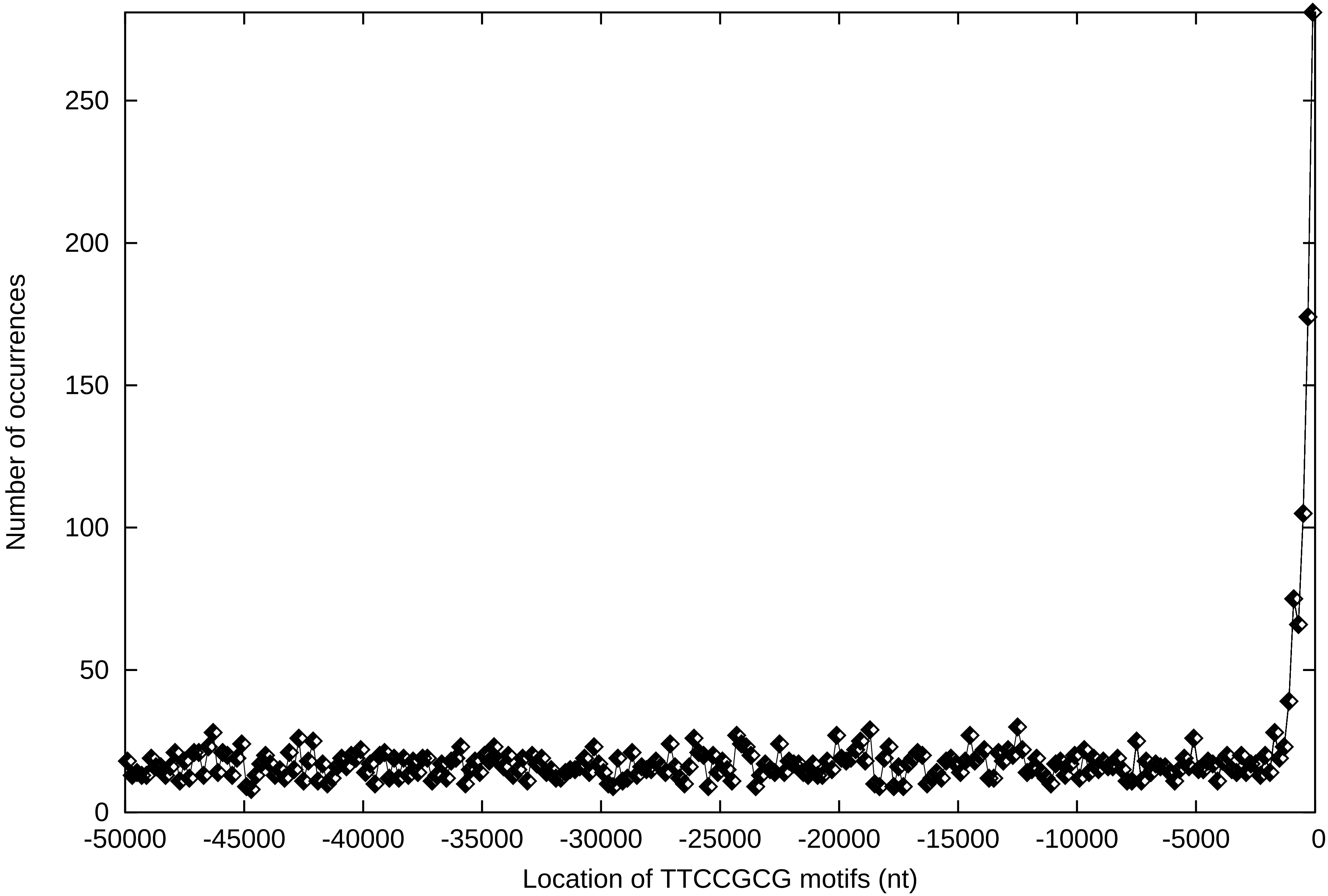

Number of occurrences
0
50
100
150
200
250
-50000
-45000
-40000
-35000
-30000
-25000
-20000
-15000
-10000
-5000
0
Location of TTCCGCG motifs (nt)

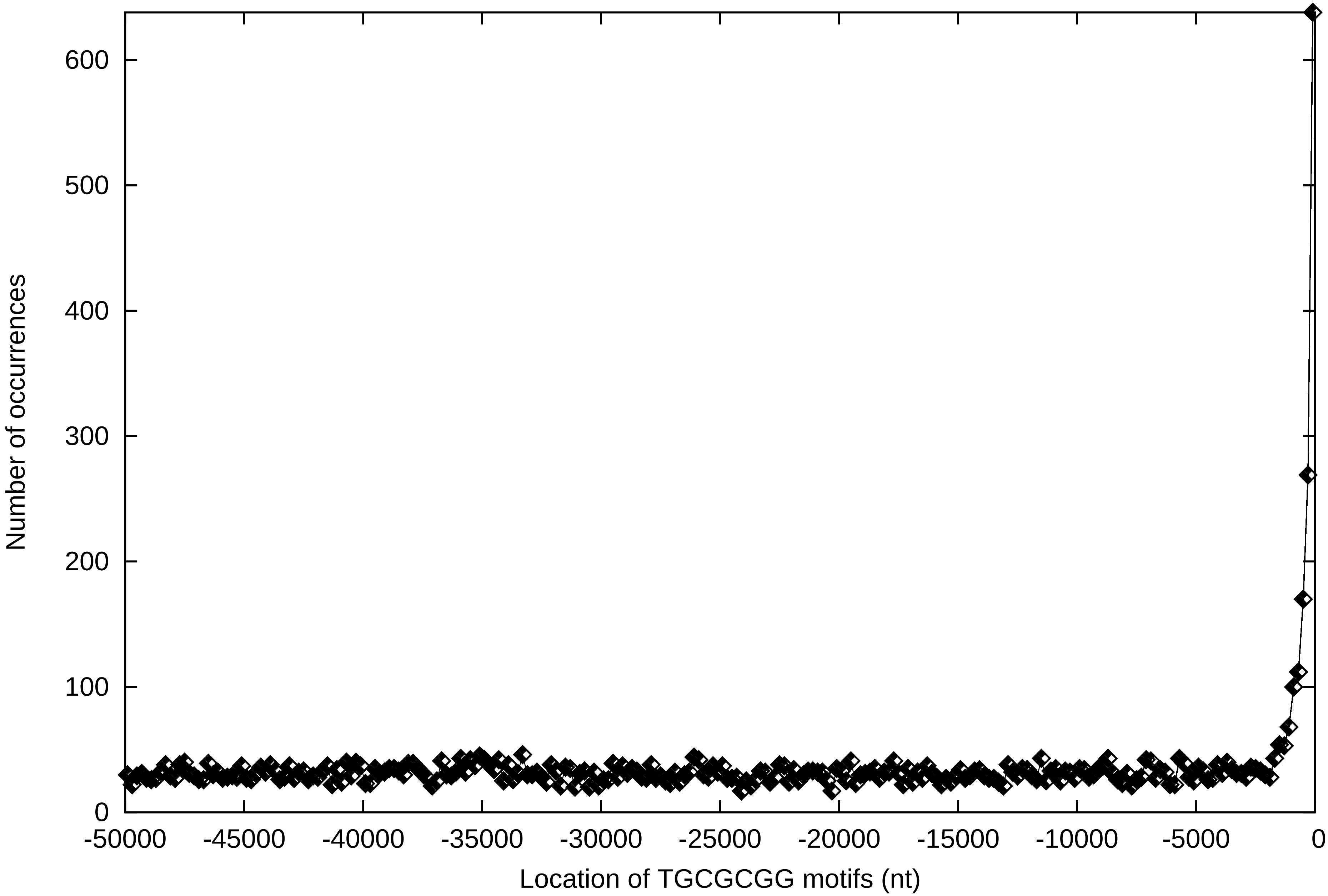

0
100
200
300
400
500
600
Number of occurrences
-50000
-45000
-40000
-35000
-30000
-25000
-20000
-15000
-10000
-5000
0
Location of TGCGCGG motifs (nt)

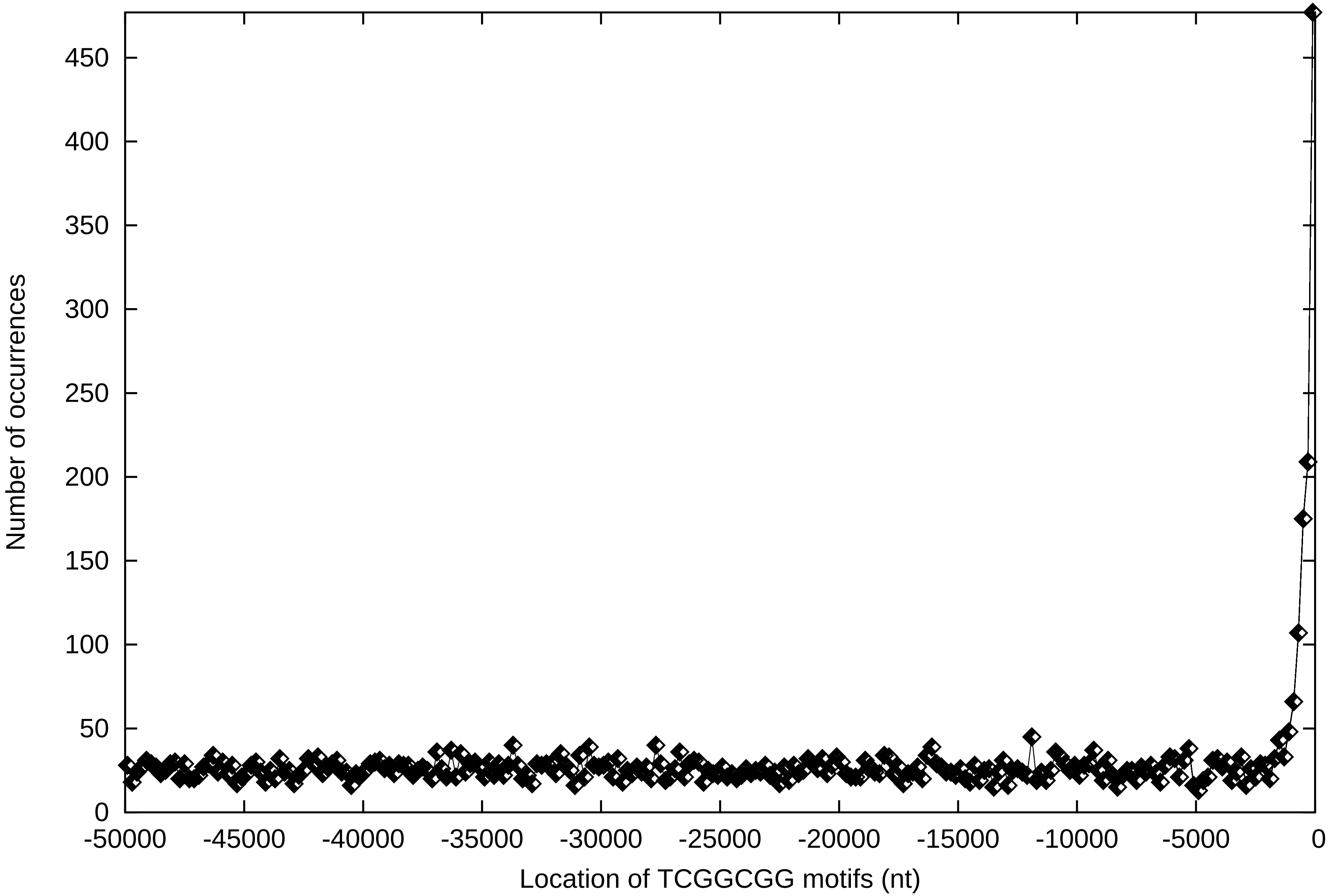

Number of occurrences
0
50
100
150
200
250
300
350
400
450
-50000
-45000
-40000
-35000
-30000
-25000
-20000
-15000
-10000
-5000
0
Location of TCGGCGG motifs (nt)

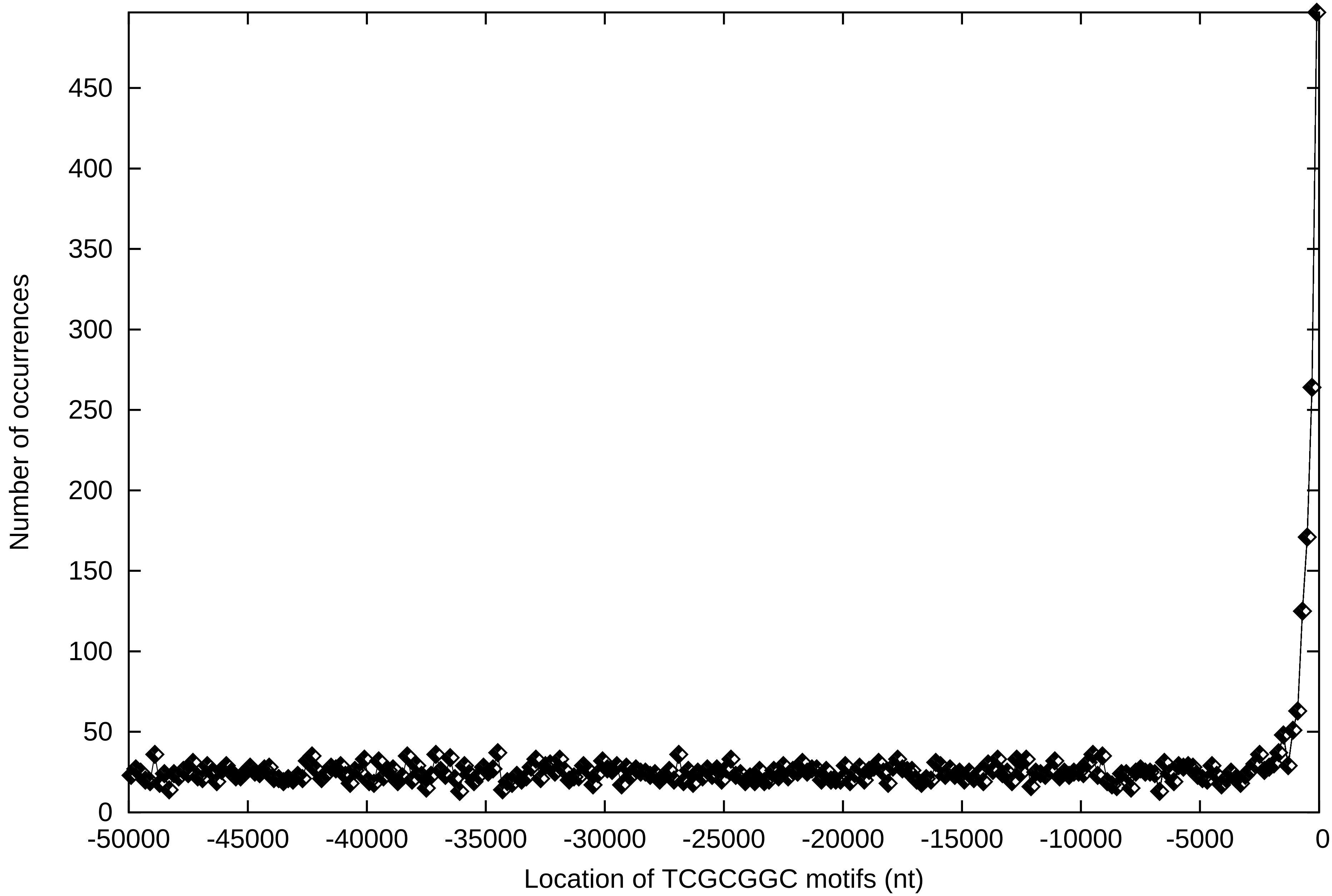

Number of occurrences
0
50
100
150
200
250
300
350
400
450
-50000
-45000
-40000
-35000
-30000
-25000
-20000
-15000
-10000
-5000
0
Location of TCGCGGC motifs (nt)

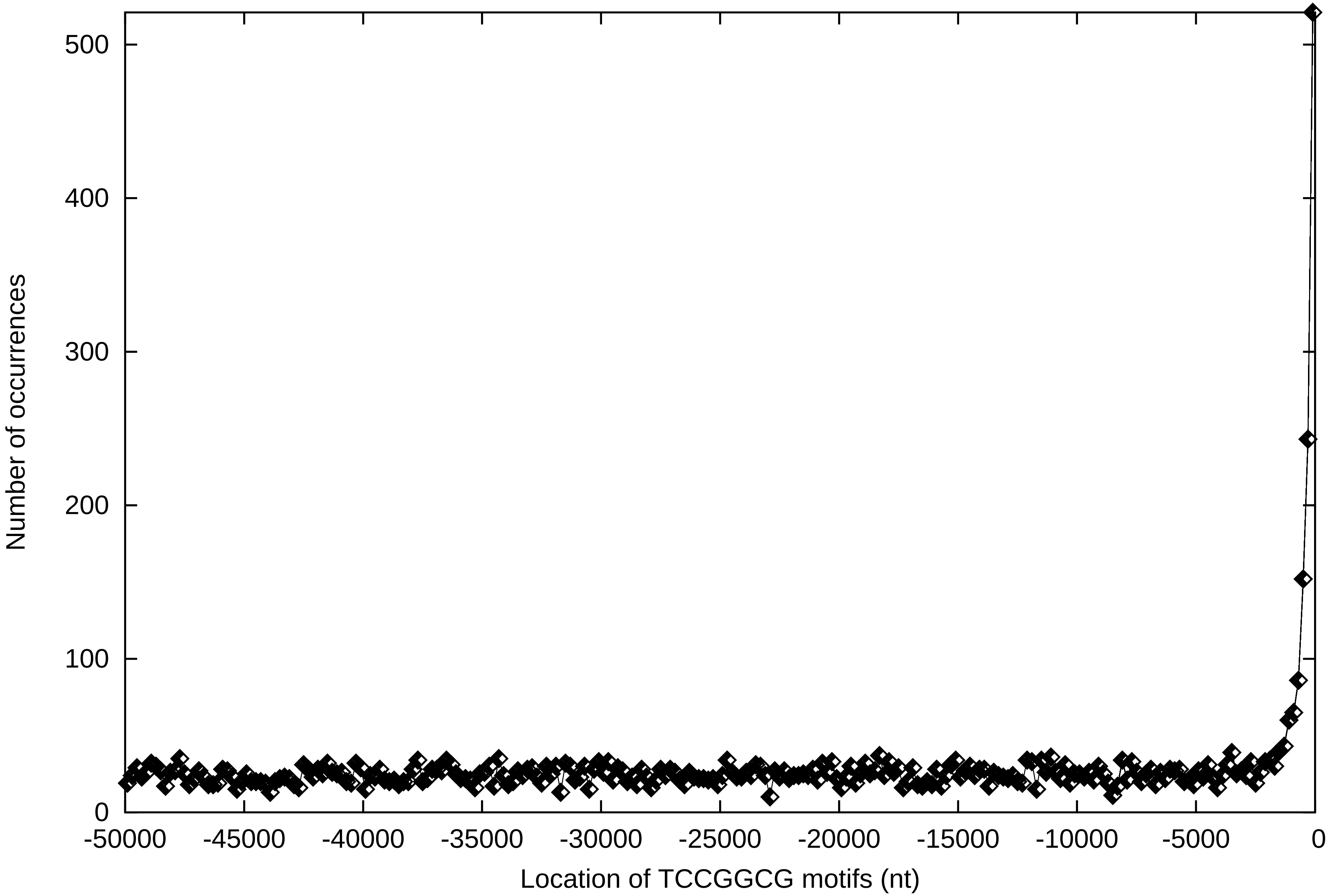
Number of occurrences
0
100
200
300
400
500
-50000
-45000
-40000
-35000
-30000
-25000
-20000
-15000
-10000
-5000
0
Location of TCCGGCG motifs (nt)

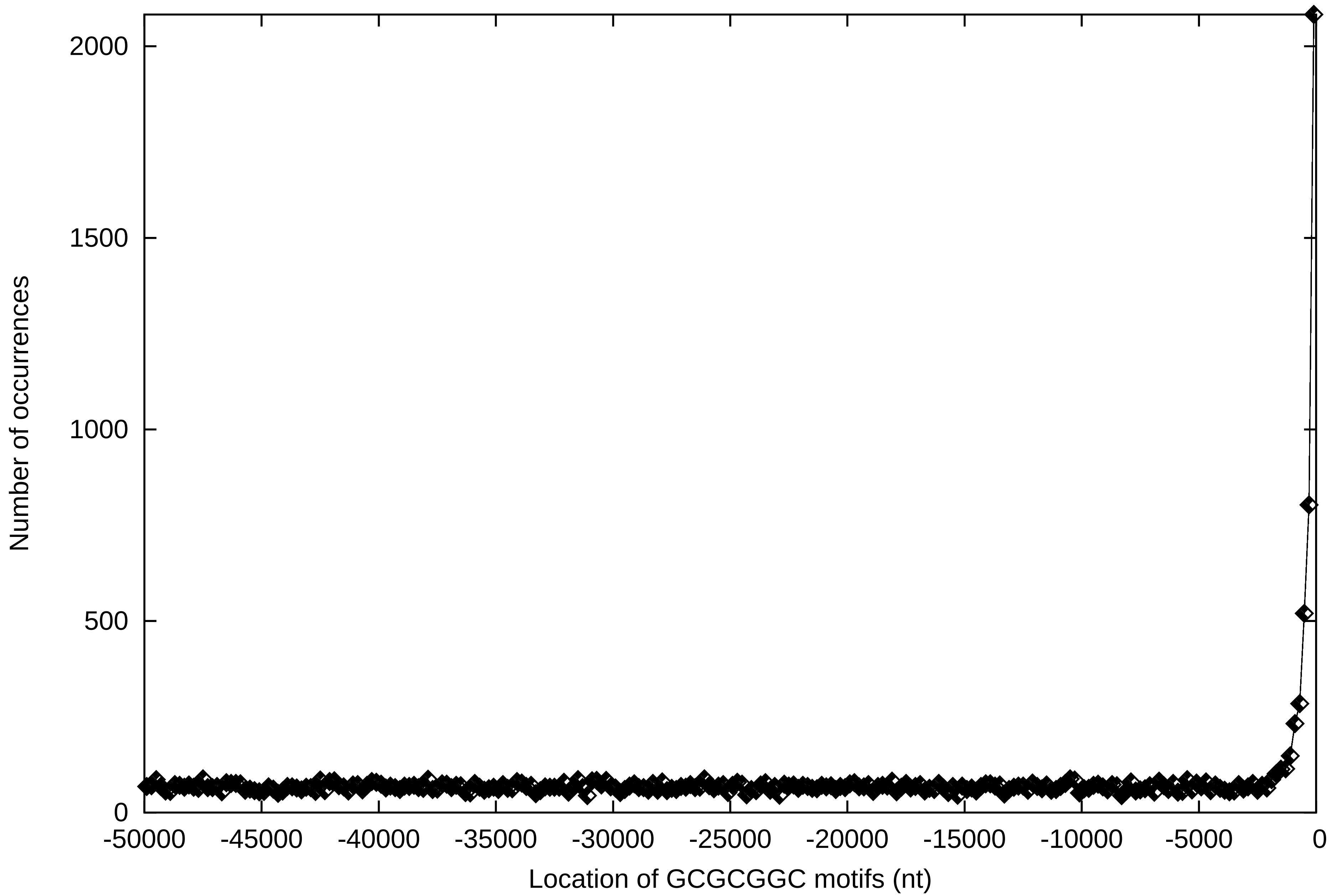

Number of occurrences
2000
1500
1000
500
0
-50000
-45000
-40000
-35000
-30000
-25000
-20000
-15000
-10000
-5000
0
Location of GCGCGGC motifs (nt)

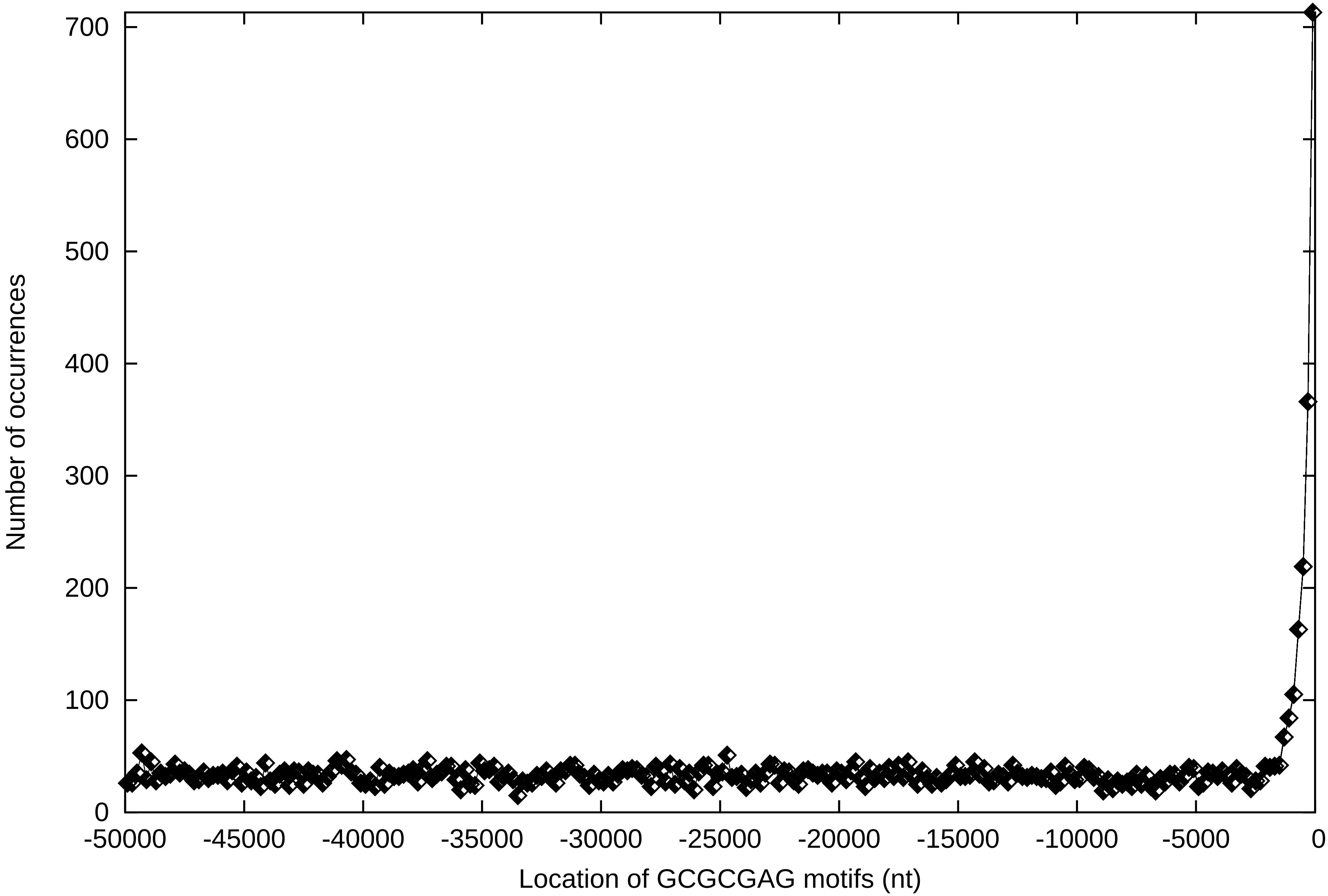

Number of occurrences
700
600
500
400
300
200
100
0
-50000
-45000
-40000
-35000
-30000
-25000
-20000
-15000
-10000
-5000
0
Location of GCGCGAG motifs (nt)

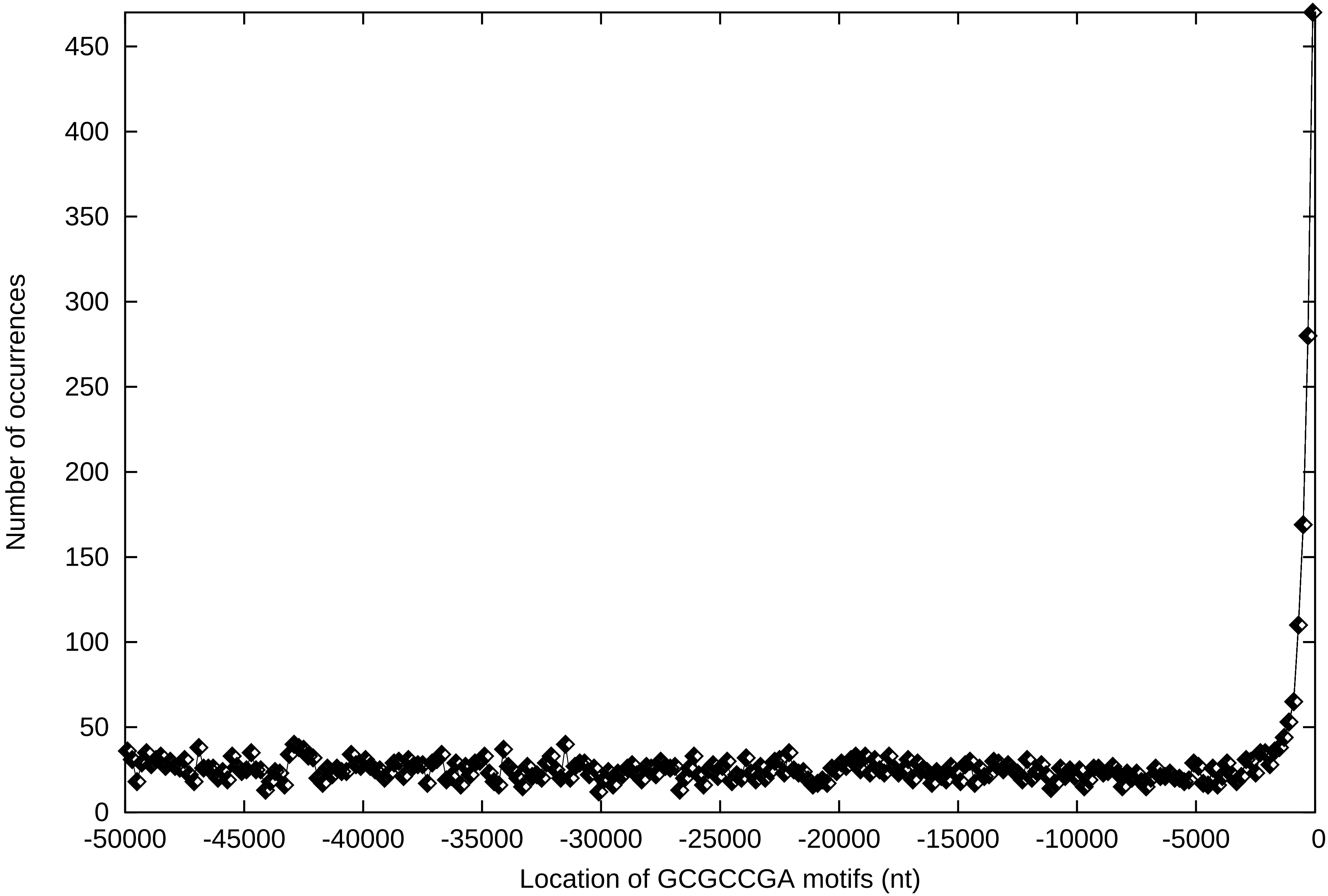

Number of occurrences
Location of GCGCCGA motifs (nt)
0
50
100
150
200
250
300
350
400
450
-50000
-45000
-40000
-35000
-30000
-25000
-20000
-15000
-10000
-5000
0

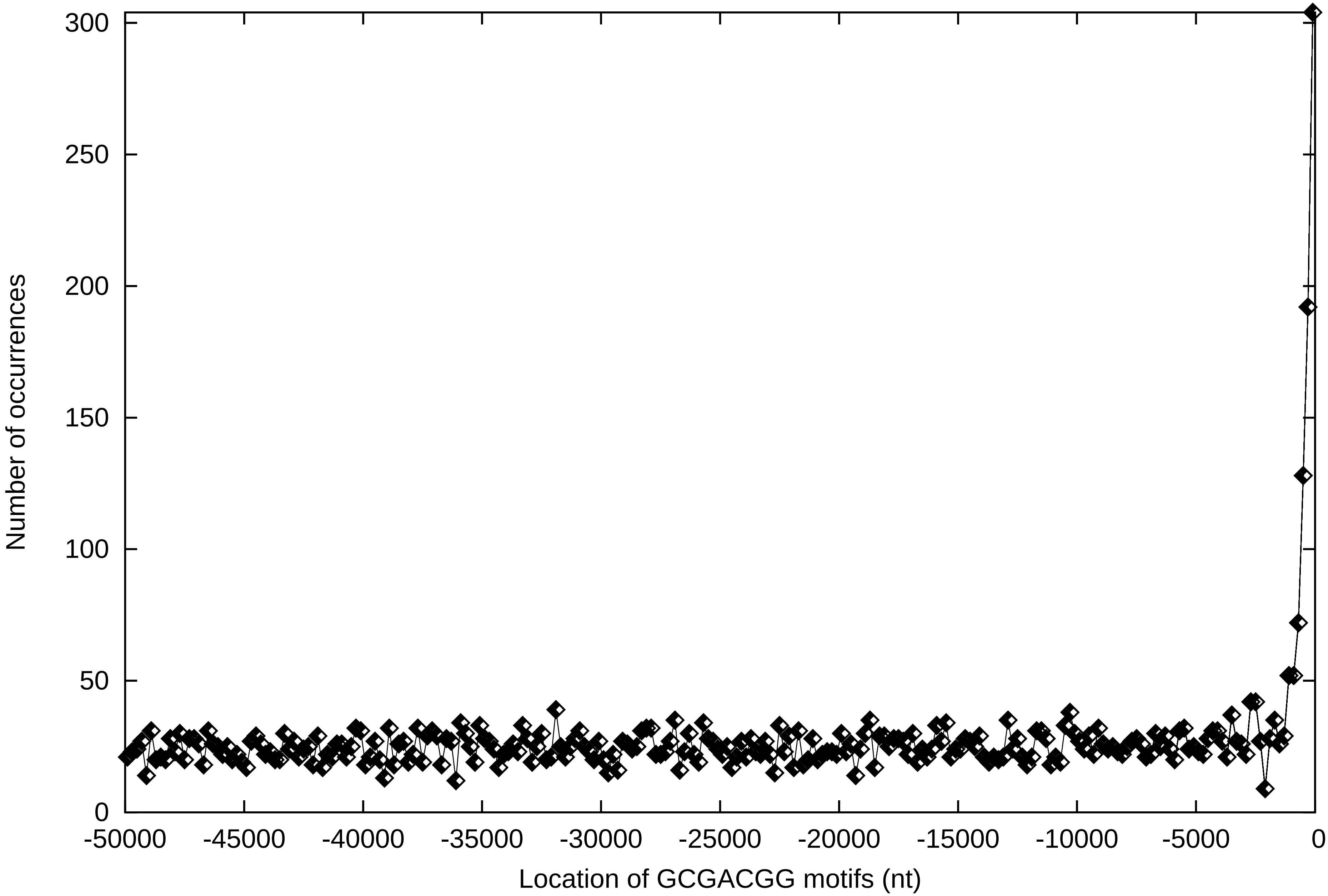
Number of occurrences
0
50
100
150
200
250
300
-50000
-45000
-40000
-35000
-30000
-25000
-20000
-15000
-10000
-5000
0
Location of GCGACGG motifs (nt)

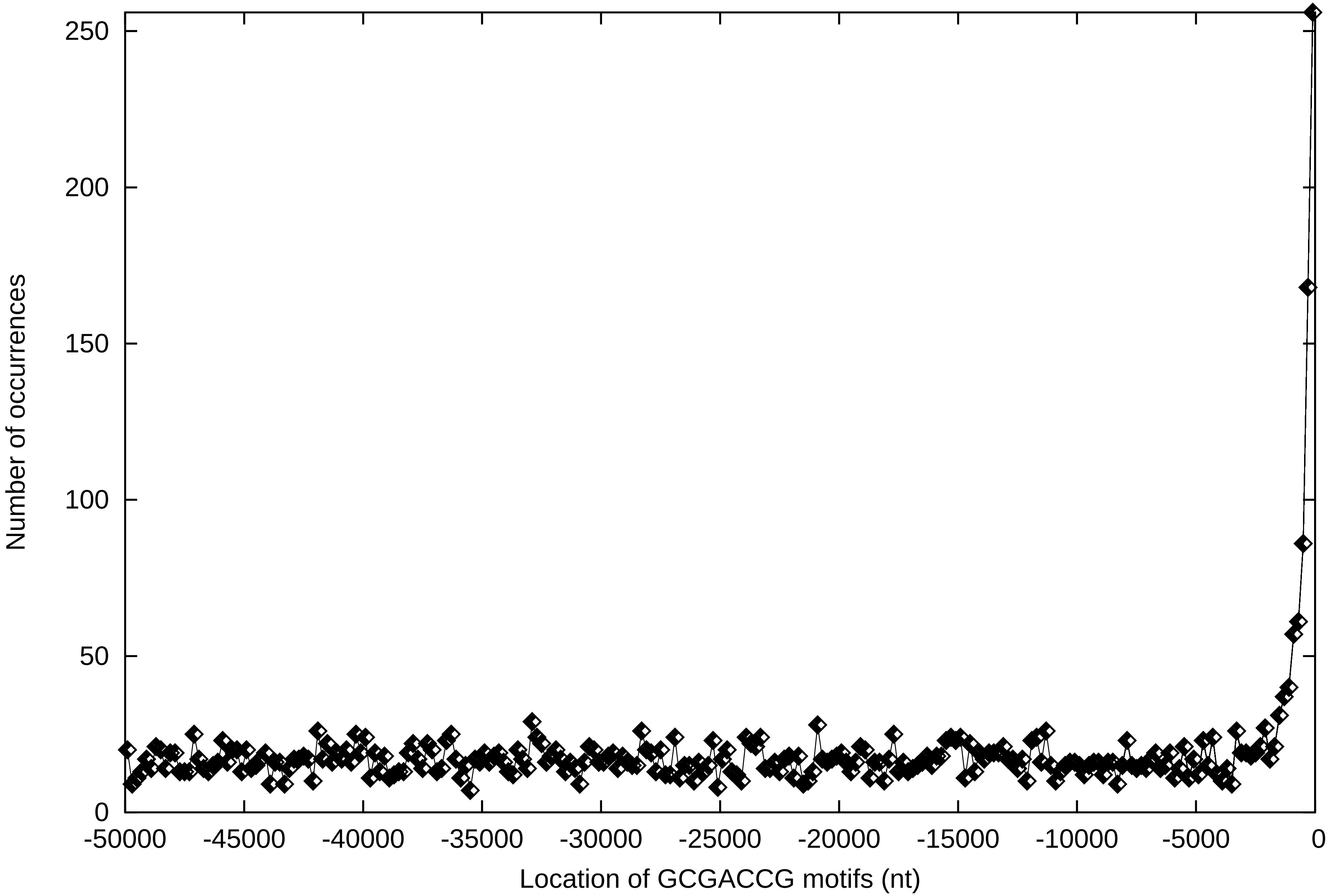

Number of occurrences
0
50
100
150
200
250
-50000
-45000
-40000
-35000
-30000
-25000
-20000
-15000
-10000
-5000
0
Location of GCGACCG motifs (nt)

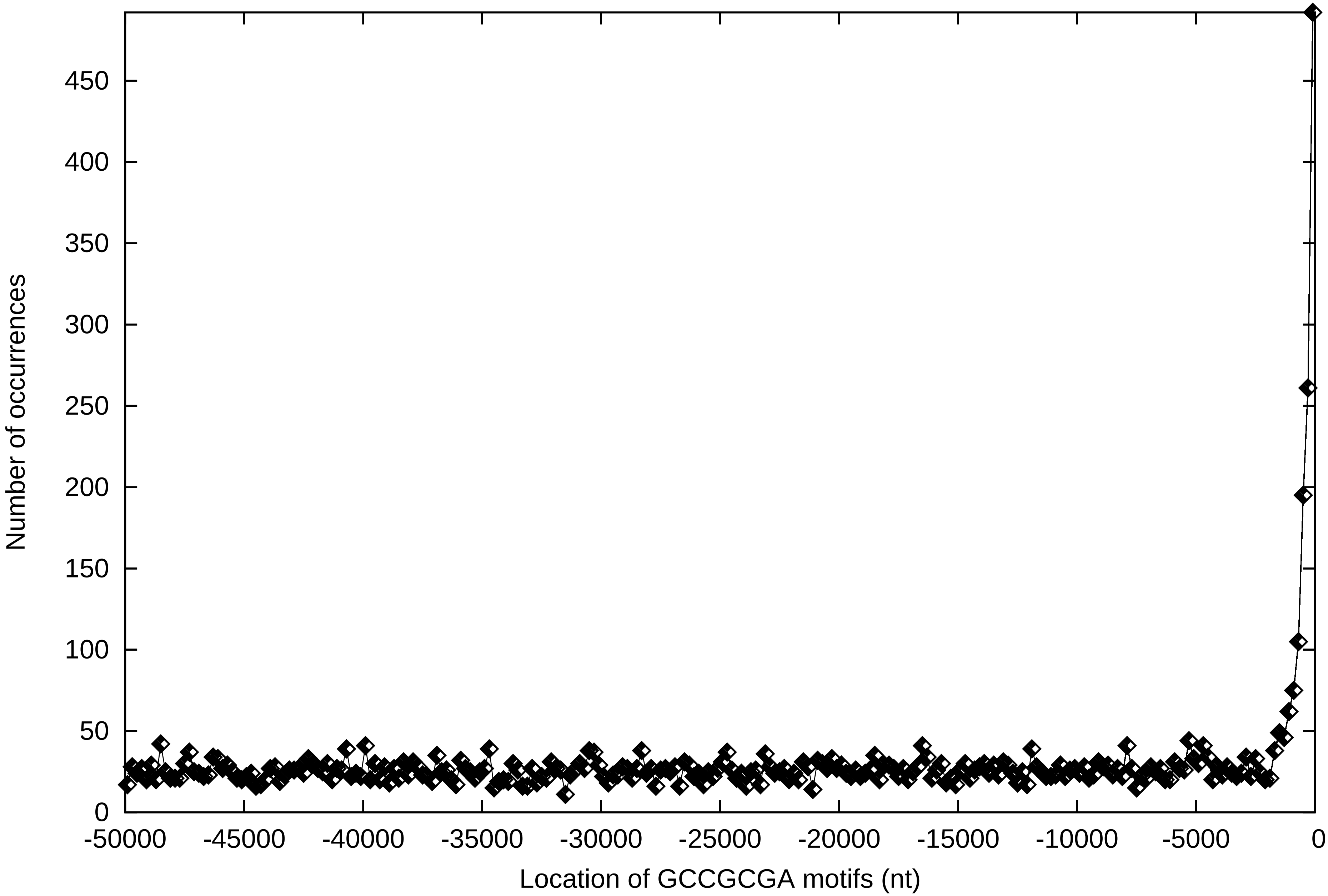

Number of occurrences
0
50
100
150
200
250
300
350
400
450
-50000
-45000
-40000
-35000
-30000
-25000
-20000
-15000
-10000
-5000
0
Location of GCCGCGA motifs (nt)

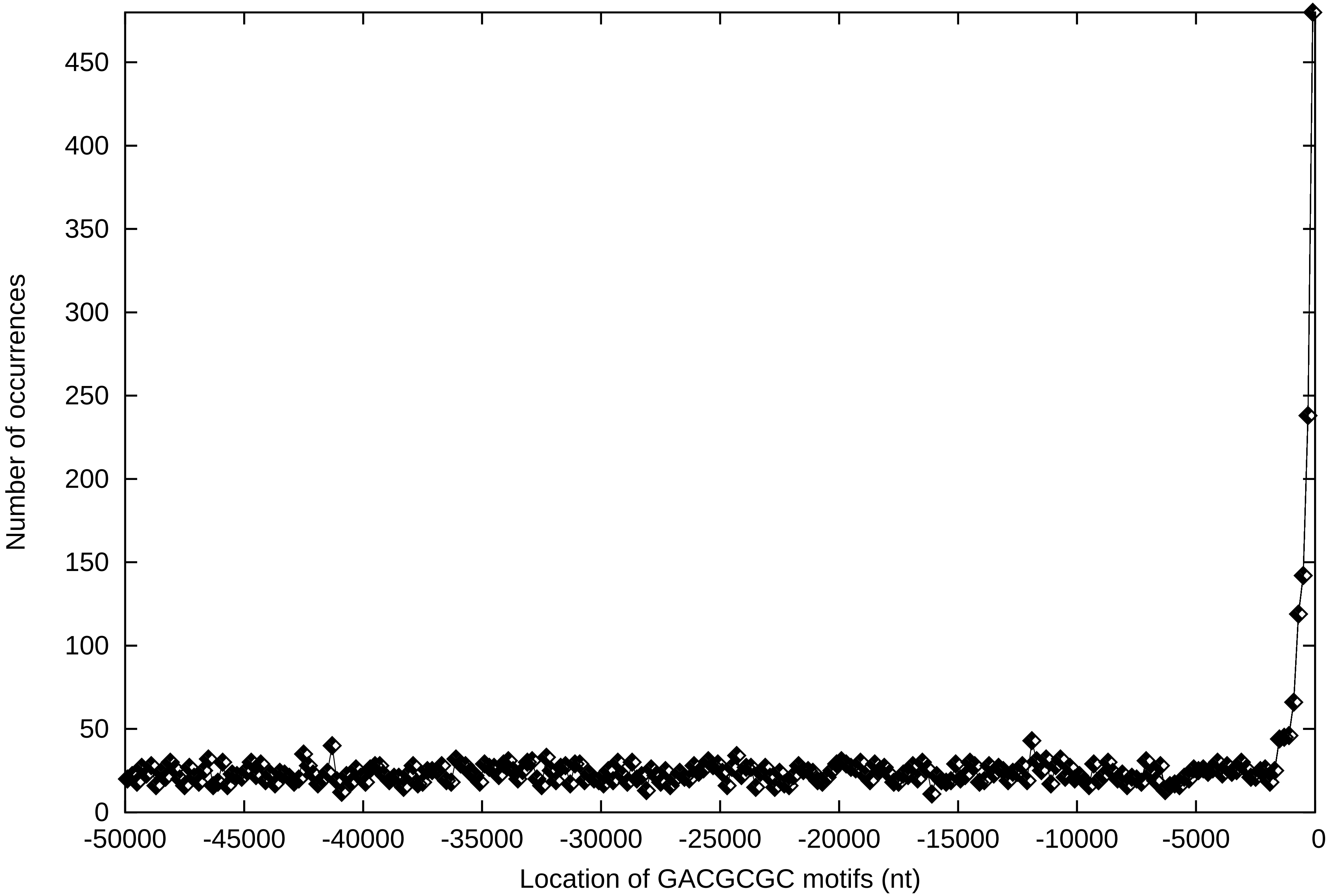

Number of occurrences
450
400
350
300
250
200
150
100
50
0
-50000
-45000
-40000
-35000
-30000
-25000
-20000
-15000
-10000
-5000
0
Location of GACGCGC motifs (nt)

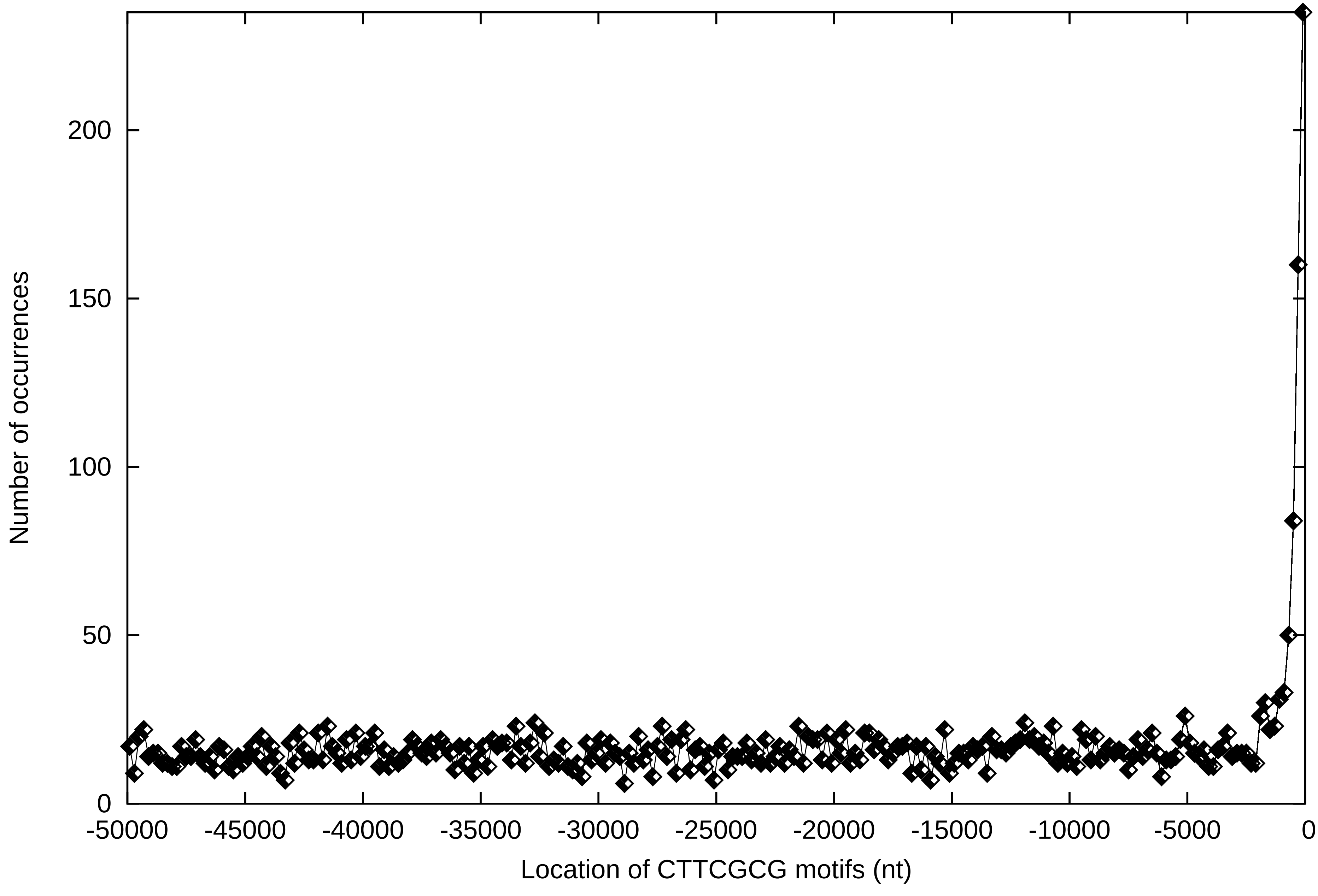

Number of occurrences
0
50
100
150
200
-50000
-45000
-40000
-35000
-30000
-25000
-20000
-15000
-10000
-5000
0
Location of CTTCGCG motifs (nt)

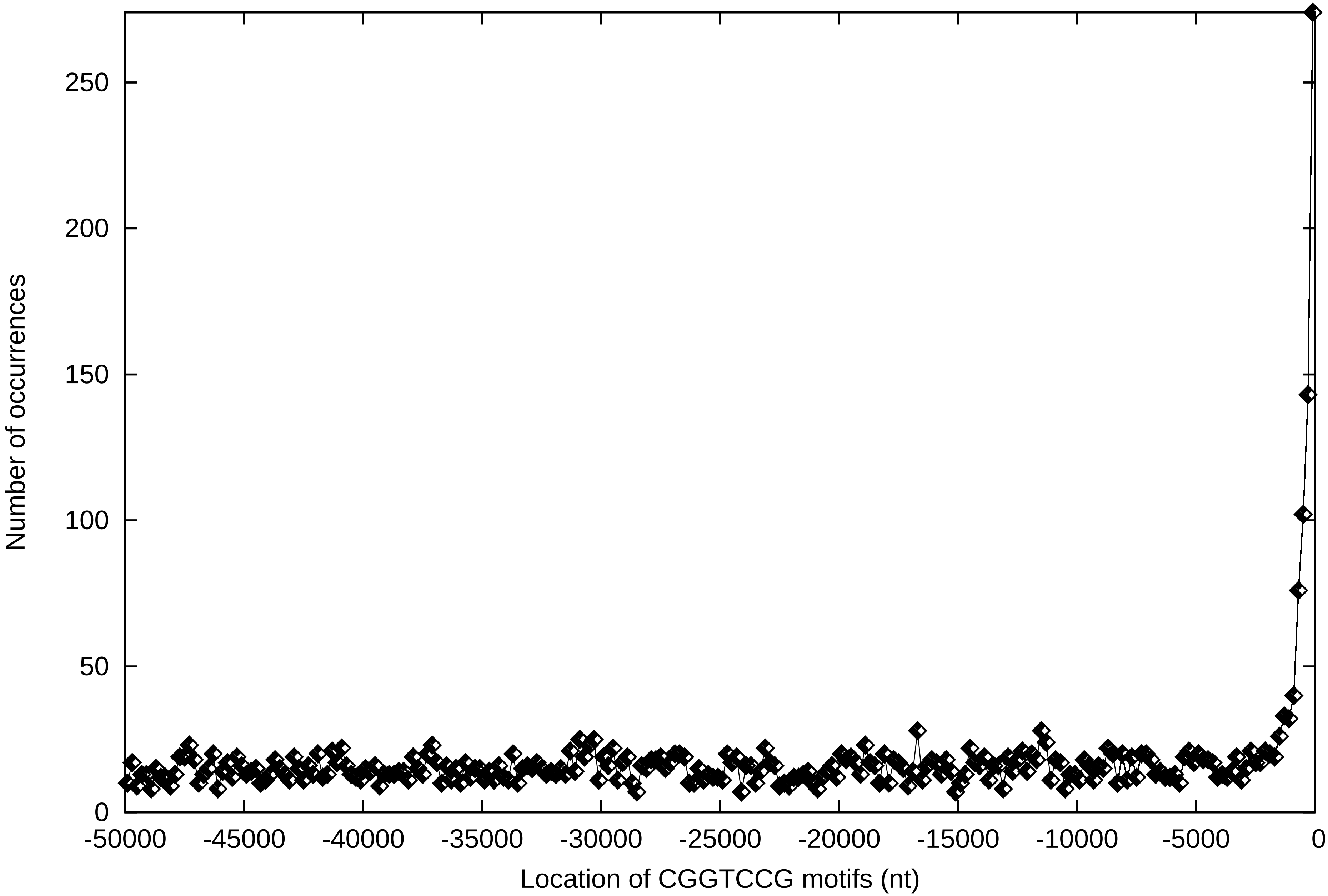
Number of occurrences
0
50
100
150
200
250
-50000
-45000
-40000
-35000
-30000
-25000
-20000
-15000
-10000
-5000
0
Location of CGGTCCG motifs (nt)

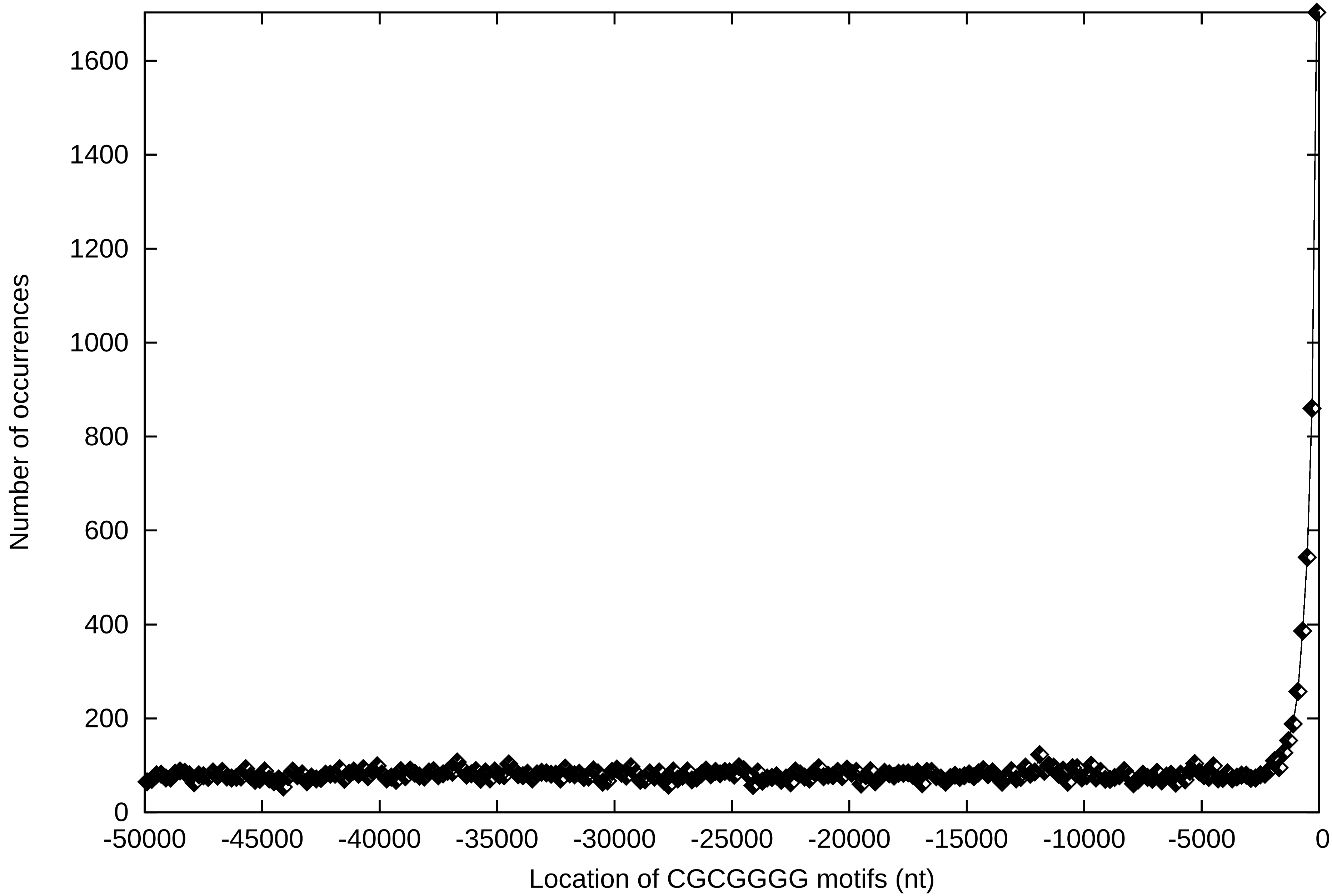

Number of occurrences
Location of CGCGGGG motifs (nt)
0
200
400
600
800
1000
1200
1400
1600
-50000
-45000
-40000
-35000
-30000
-25000
-20000
-15000
-10000
-5000
0

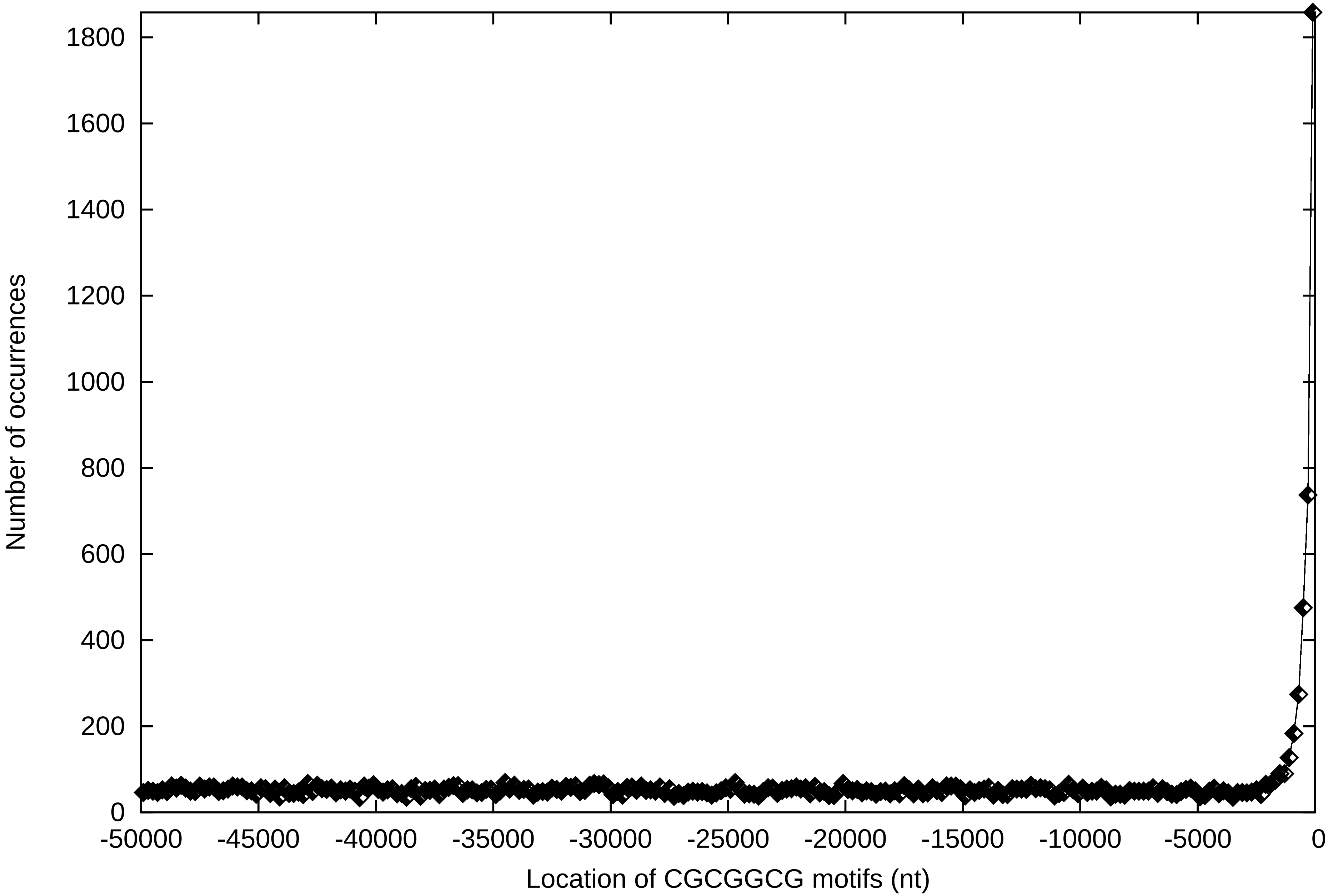

1800
1600
1400
1200
1000
800
600
400
200
0
-50000
-45000
-40000
-35000
-30000
-25000
-20000
-15000
-10000
-5000
0
Number of occurrences
Location of CGCGGCG motifs (nt)

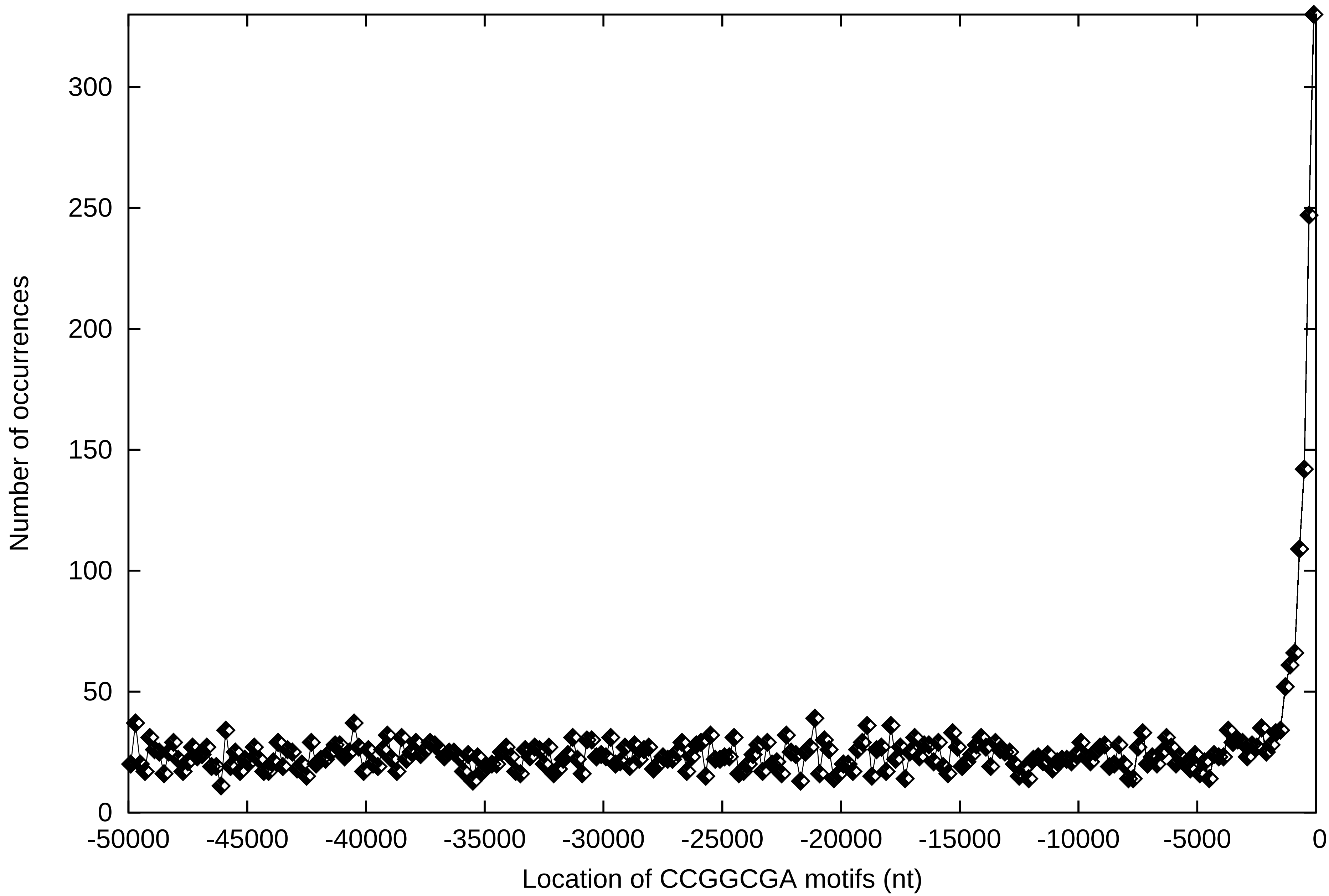

Number of occurrences
Location of CCGGCGA motifs (nt)
0
50
100
150
200
250
300
-50000
-45000
-40000
-35000
-30000
-25000
-20000
-15000
-10000
-5000
0

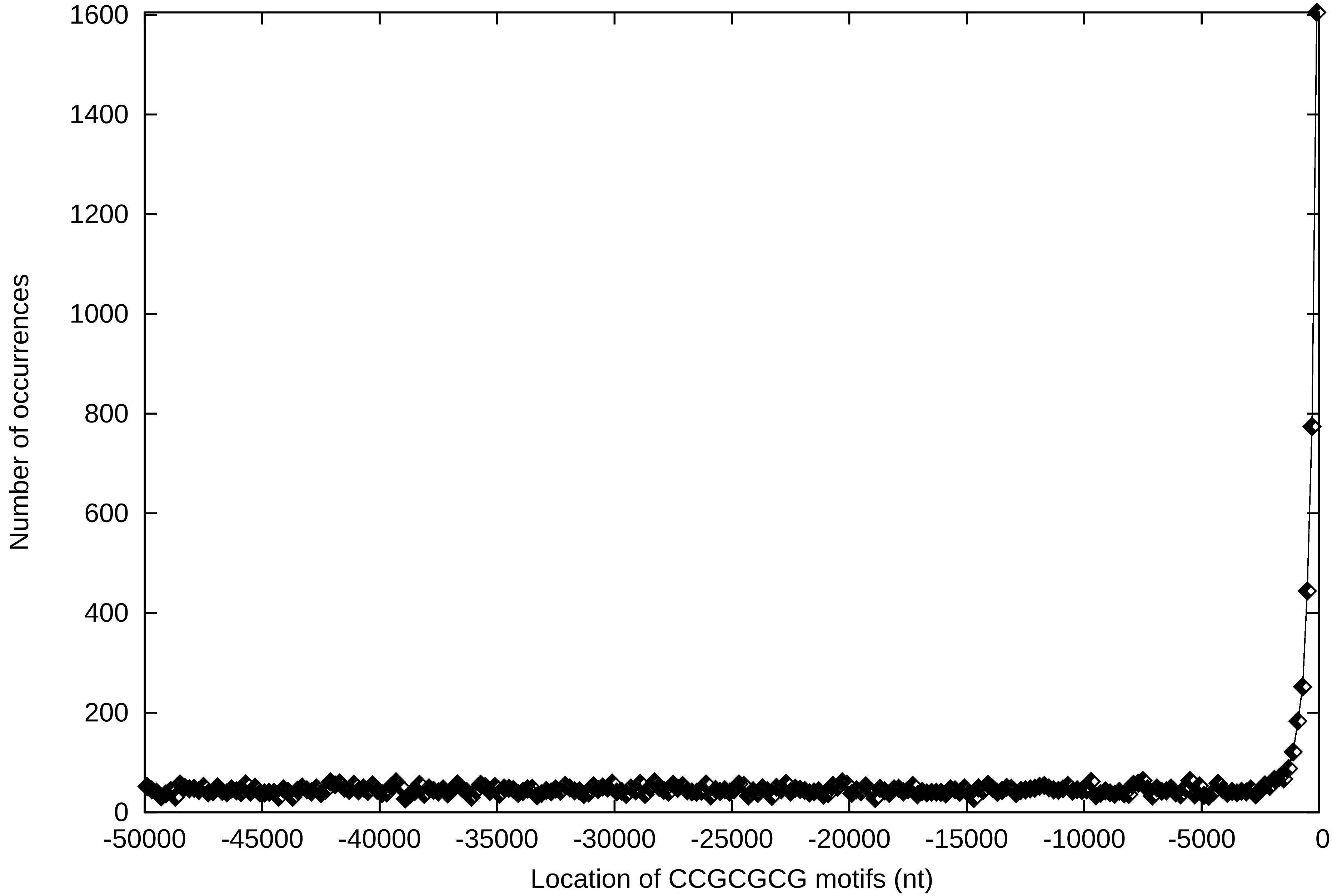

1600
1400
1200
1000
800
600
400
200
0
Number of occurrences
-50000
-45000
-40000
-35000
-30000
-25000
-20000
-15000
-10000
-5000
0
Location of CCGCGCG motifs (nt)

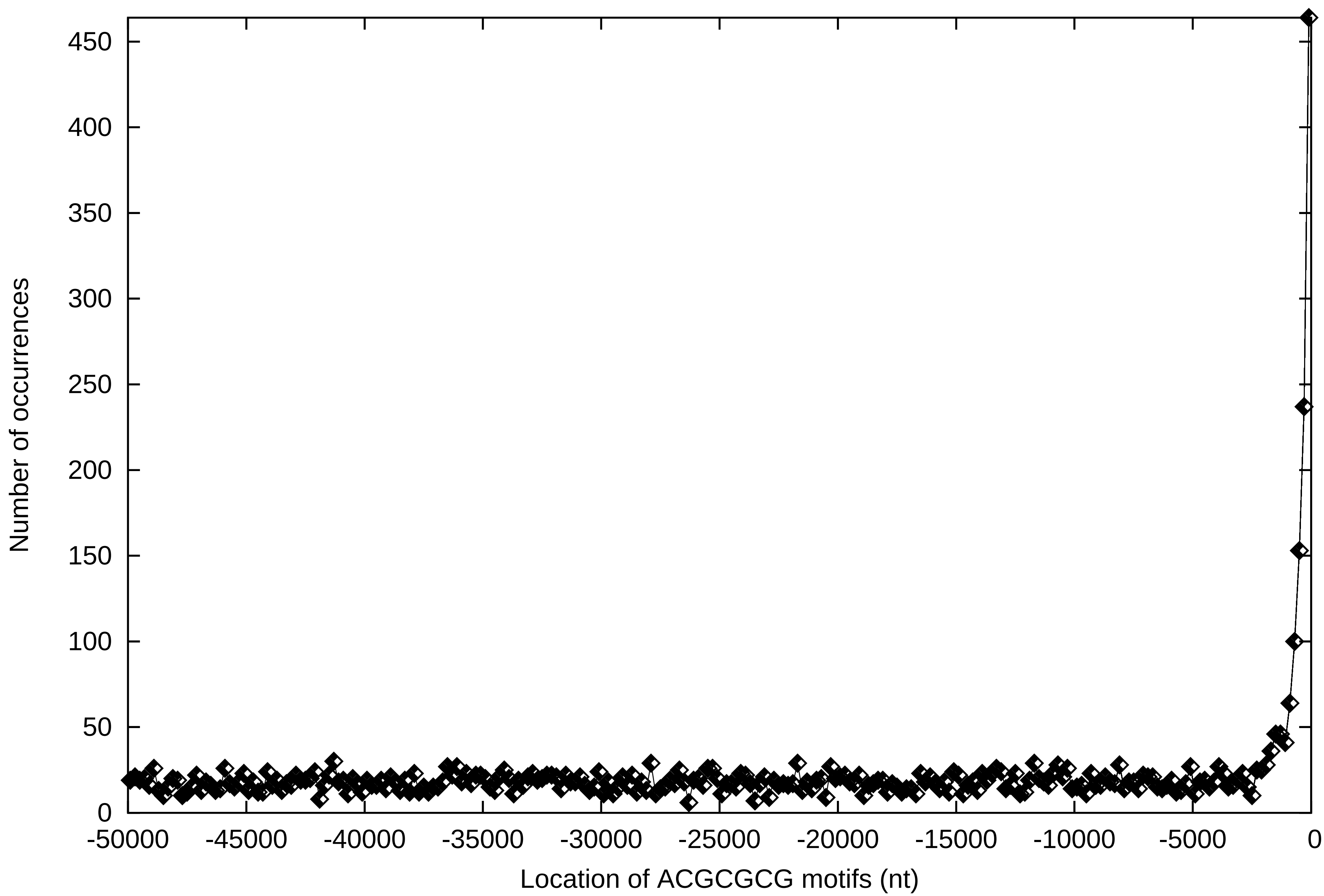

Number of occurrences
0
50
100
150
200
250
300
350
400
450
-50000
-45000
-40000
-35000
-30000
-25000
-20000
-15000
-10000
-5000
0
Location of ACGCGCG motifs (nt)

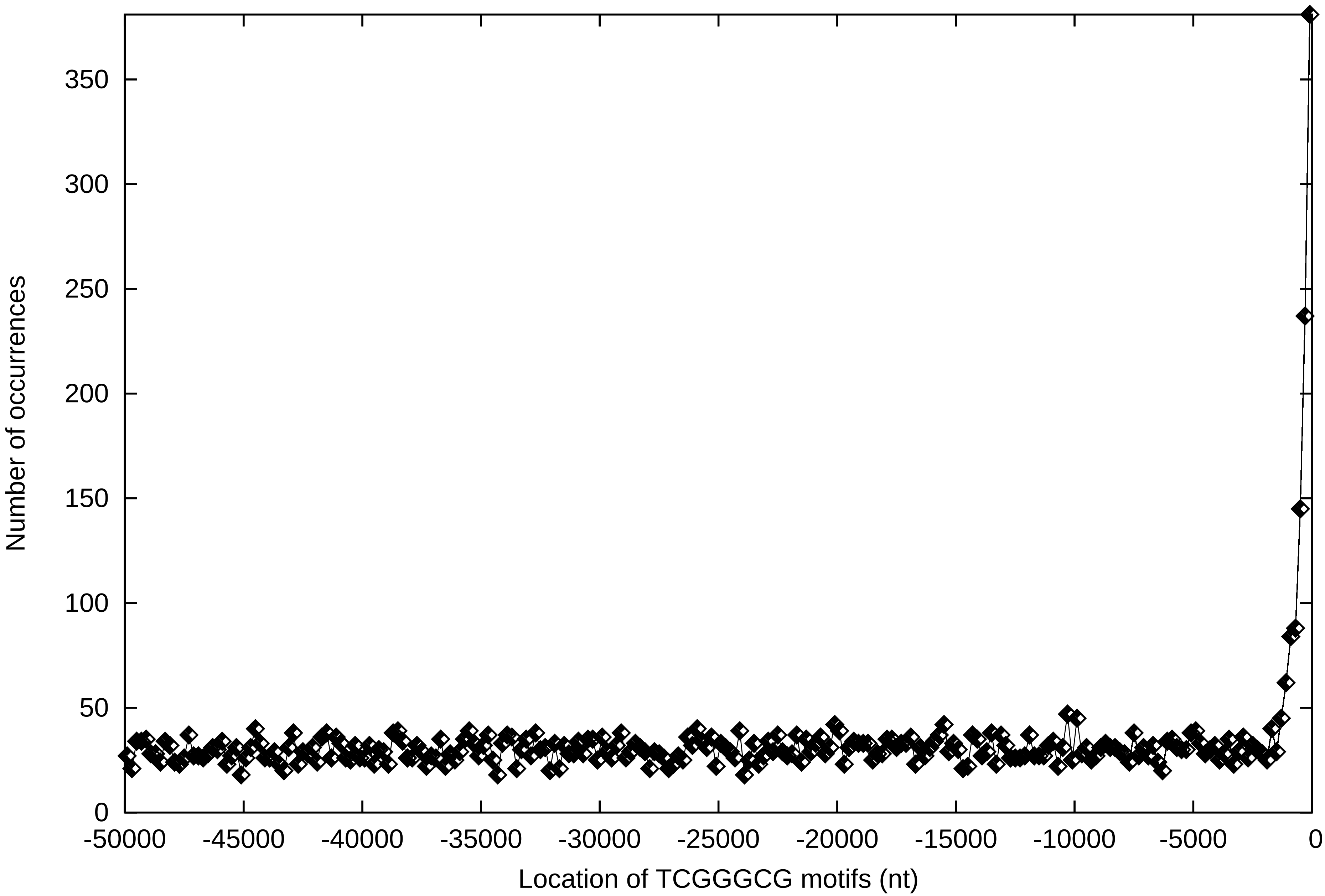
Number of occurrences
Location of TCGGGCG motifs (nt)
0
50
100
150
200
250
300
350
-50000
-45000
-40000
-35000
-30000
-25000
-20000
-15000
-10000
-5000
0

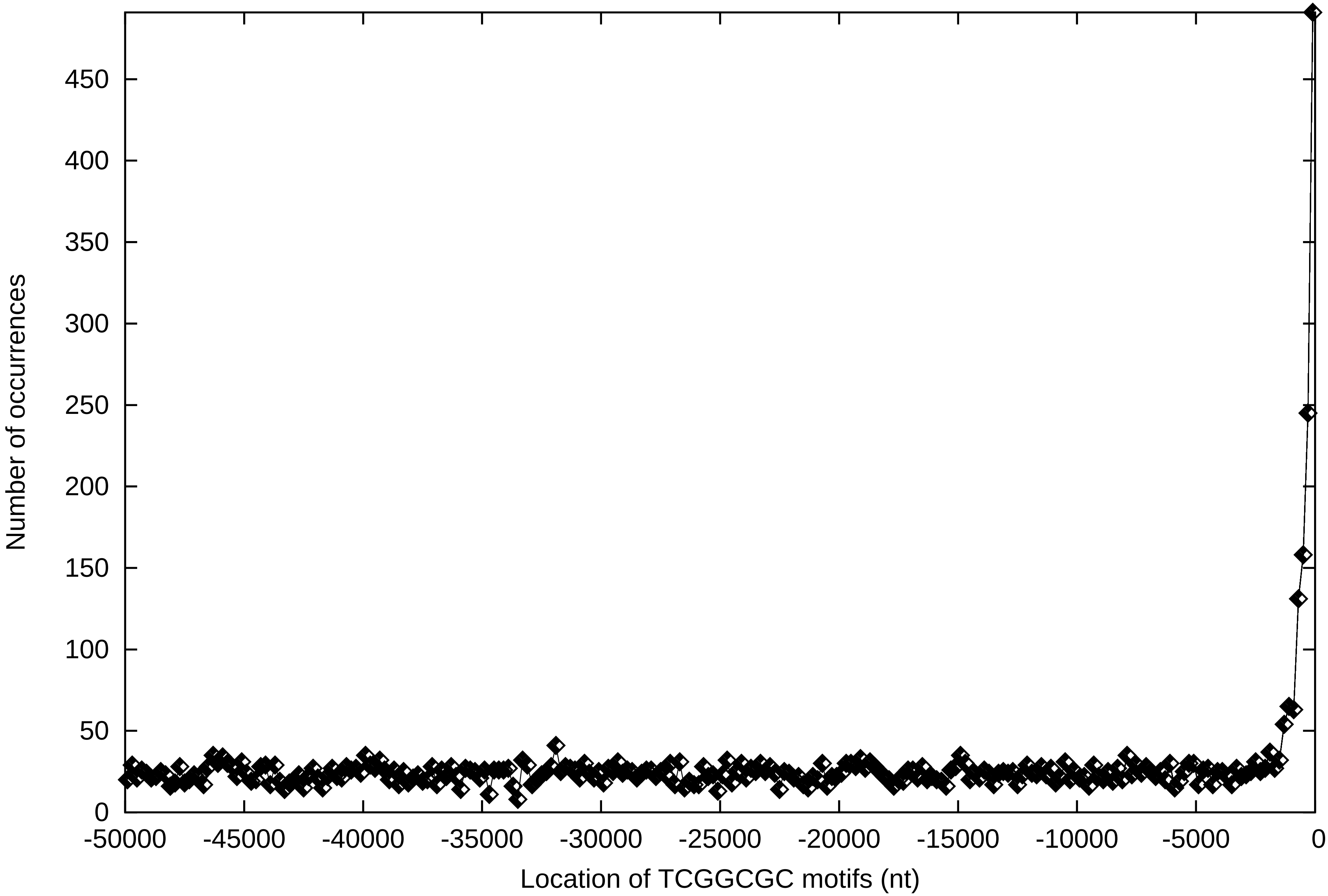

Number of occurrences
0
50
100
150
200
250
300
350
400
450
-50000
-45000
-40000
-35000
-30000
-25000
-20000
-15000
-10000
-5000
0
Location of TCGGCGC motifs (nt)

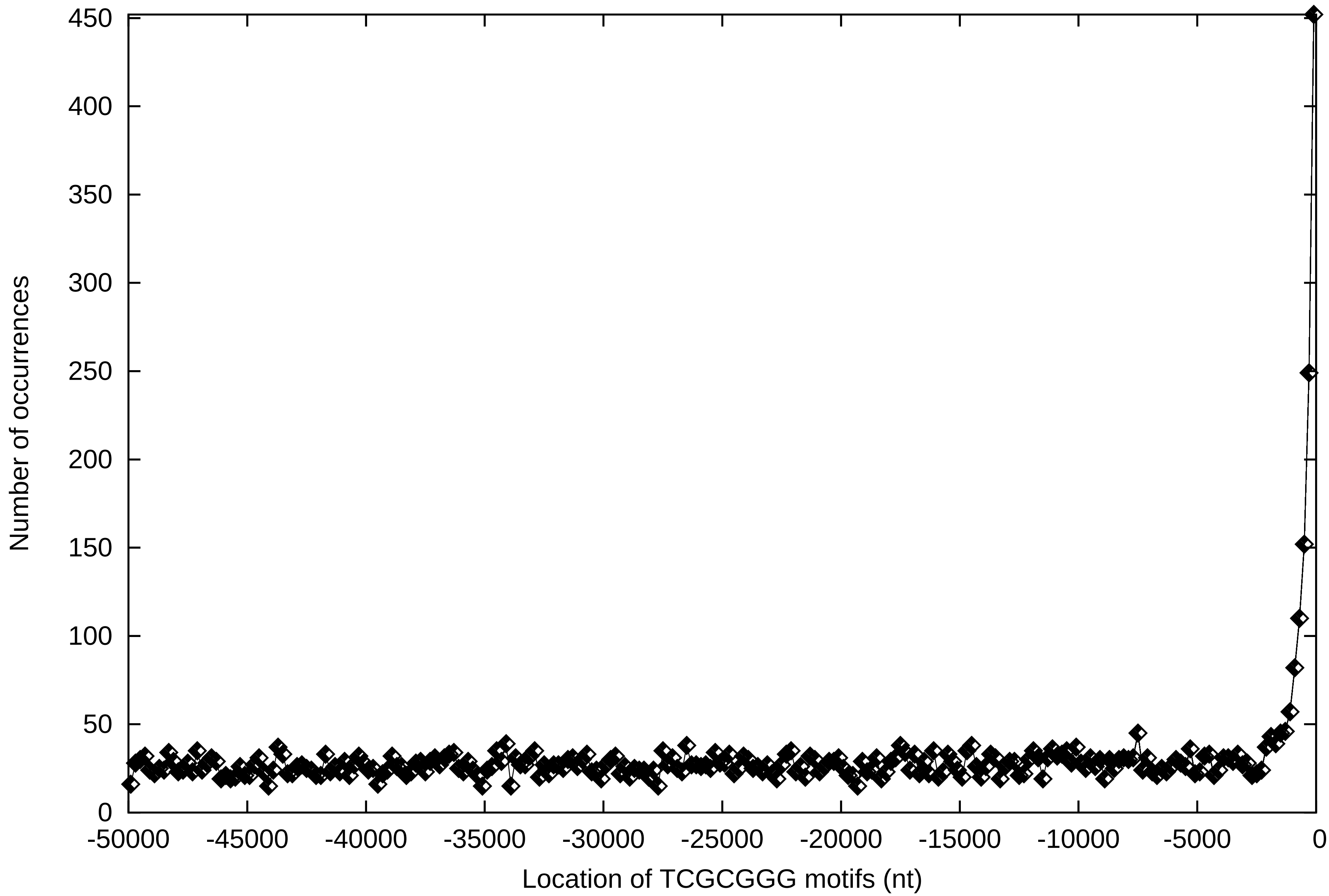

Number of occurrences
0
50
100
150
200
250
300
350
400
450
-50000
-45000
-40000
-35000
-30000
-25000
-20000
-15000
-10000
-5000
0
Location of TCGCGGG motifs (nt)

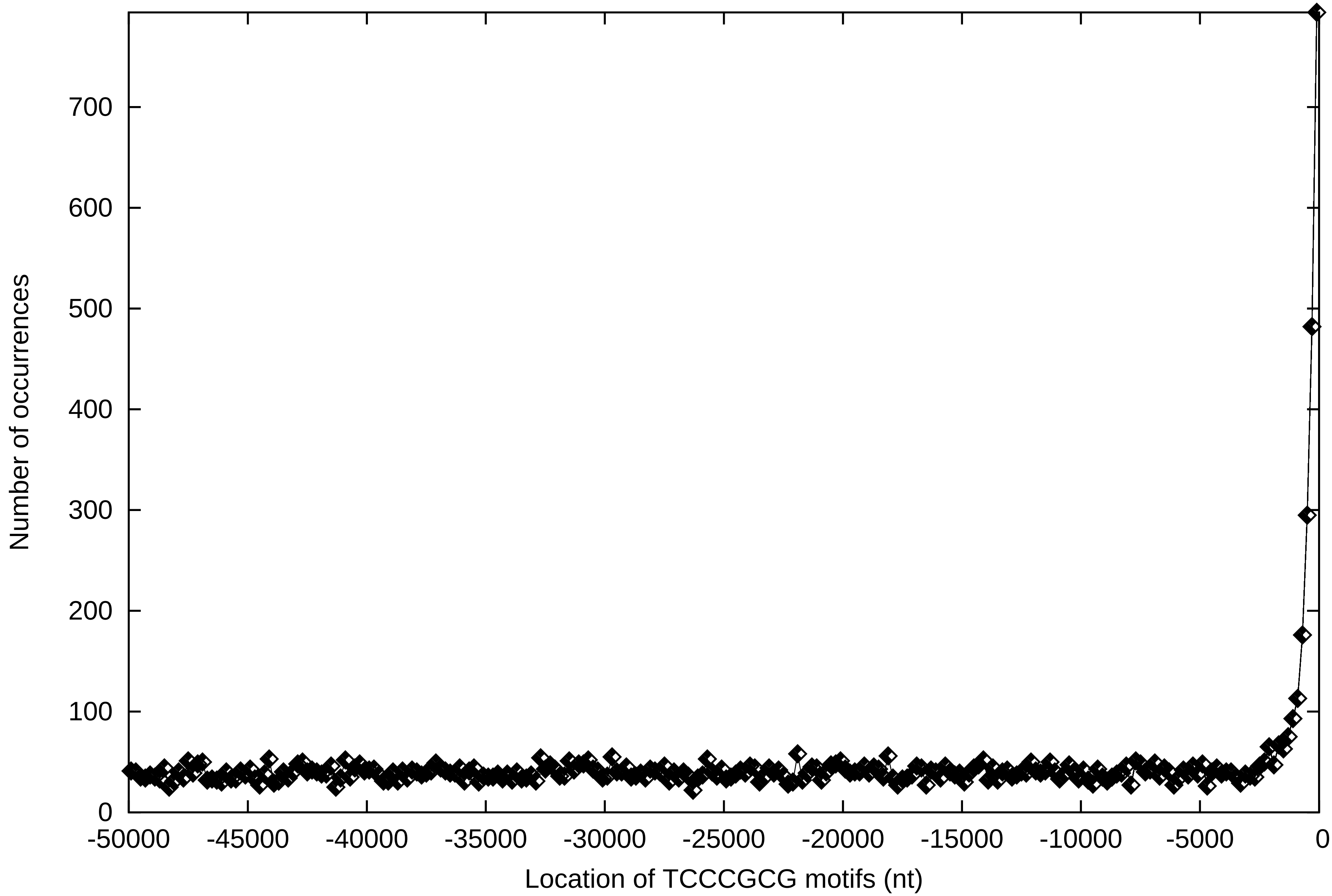

Number of occurrences
0
100
200
300
400
500
600
700
-50000
-45000
-40000
-35000
-30000
-25000
-20000
-15000
-10000
-5000
0
Location of TCCCGCG motifs (nt)

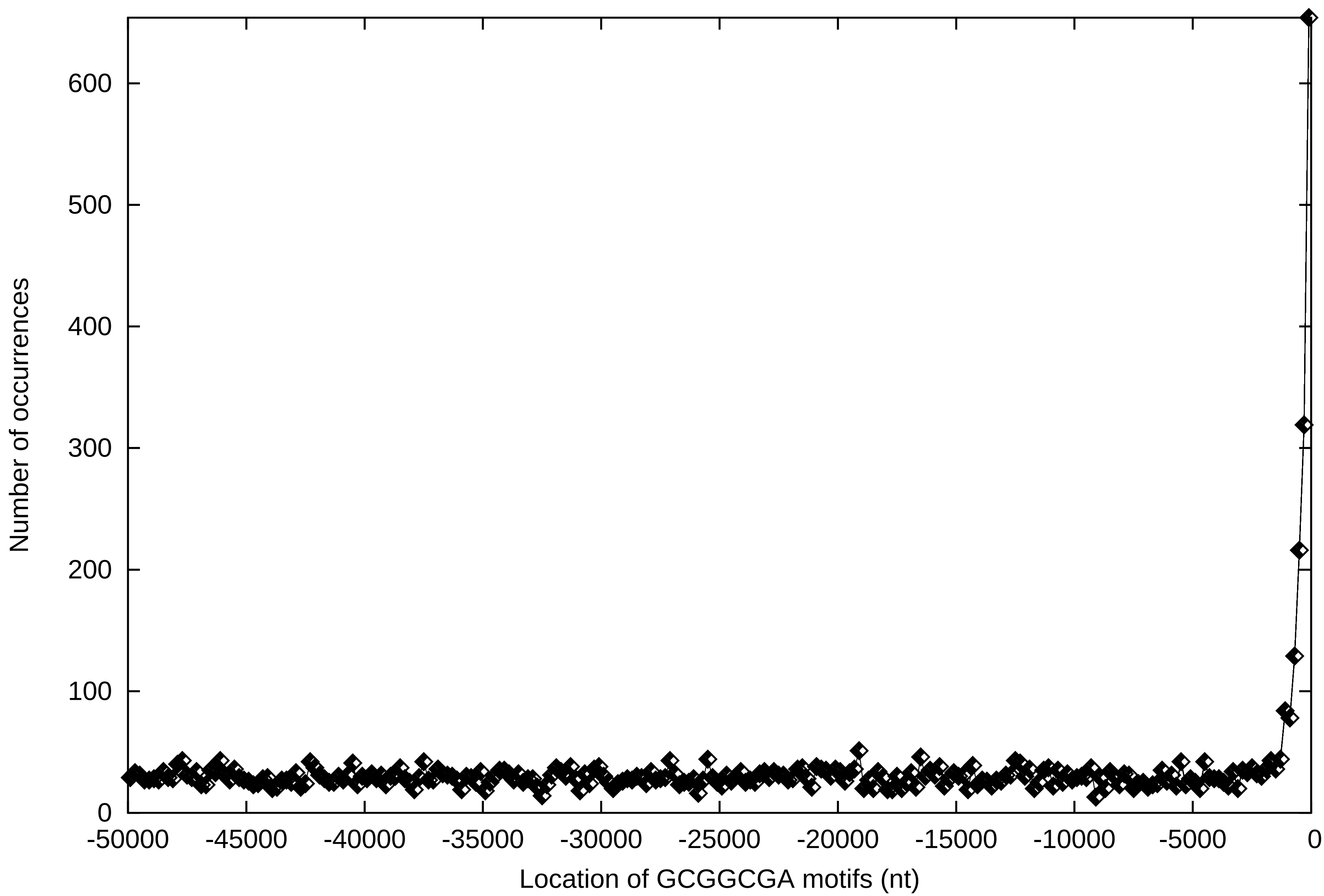
Number of occurrences
0
100
200
300
400
500
600
-50000
-45000
-40000
-35000
-30000
-25000
-20000
-15000
-10000
-5000
0
Location of GCGGCGA motifs (nt)

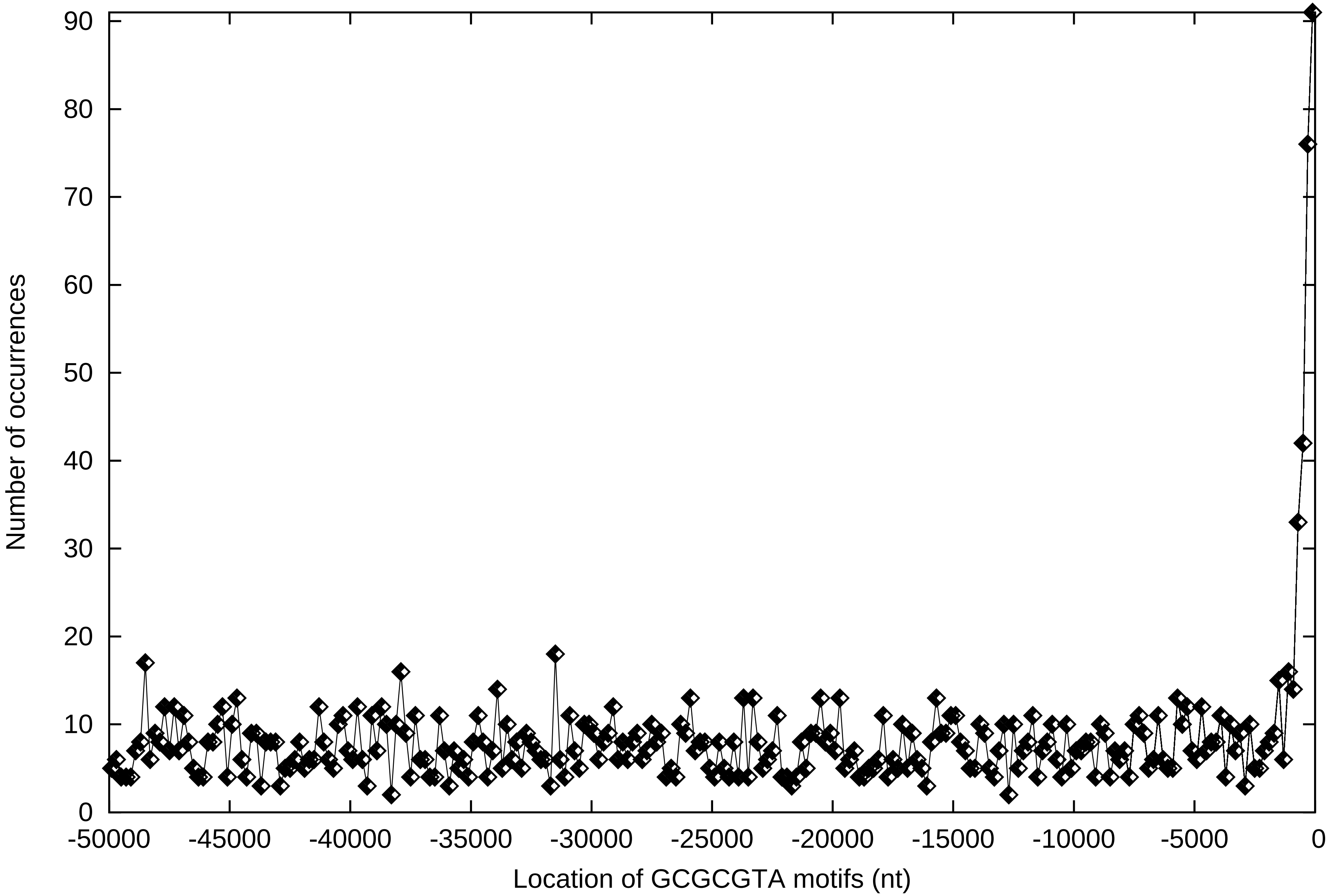
Number of occurrences
0
10
20
30
40
50
60
70
80
90
-50000
-45000
-40000
-35000
-30000
-25000
-20000
-15000
-10000
-5000
0
Location of GCGCGTA motifs (nt)

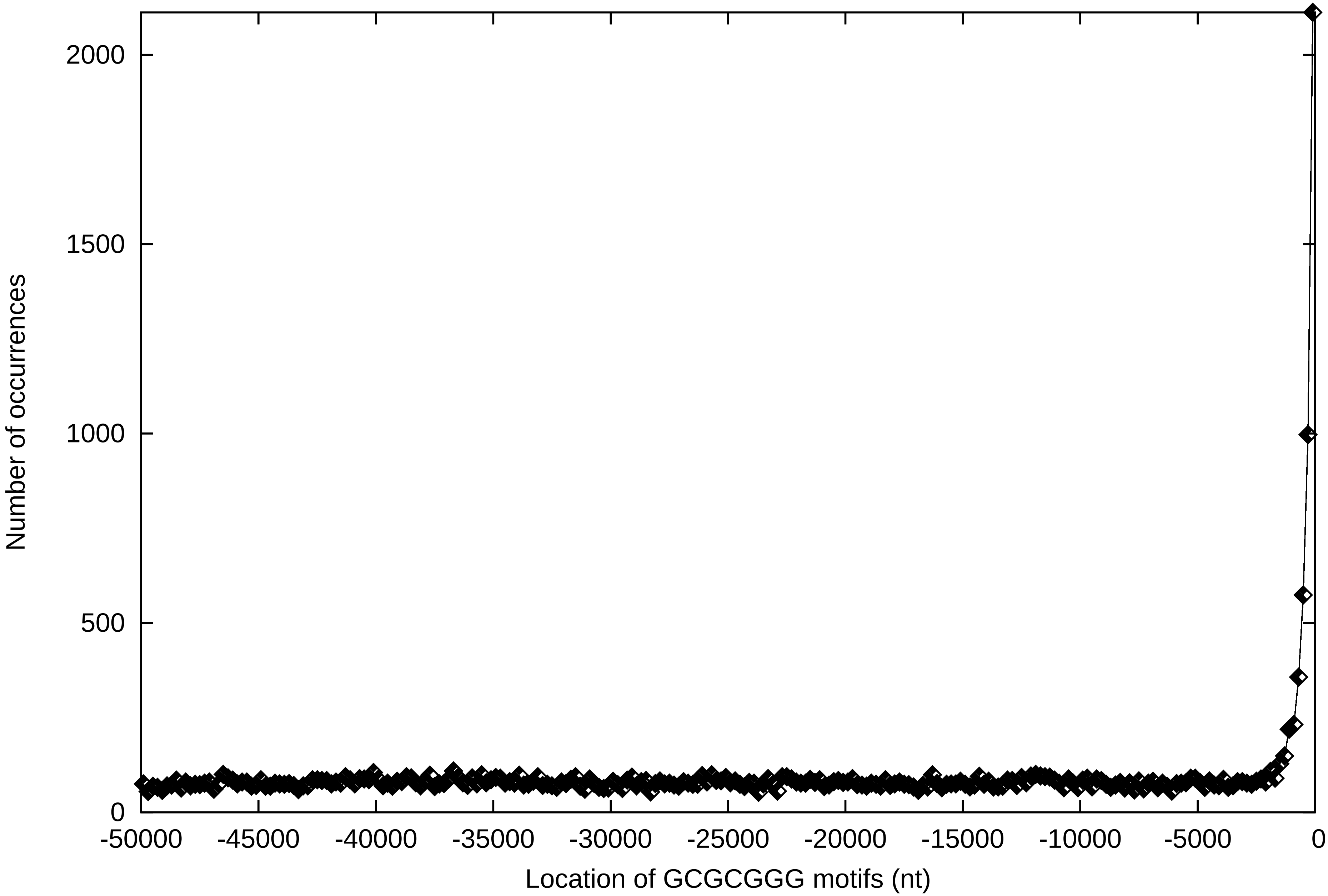

Number of occurrences
2000
1500
1000
500
0
-50000
-45000
-40000
-35000
-30000
-25000
-20000
-15000
-10000
-5000
0
Location of GCGCGGG motifs (nt)

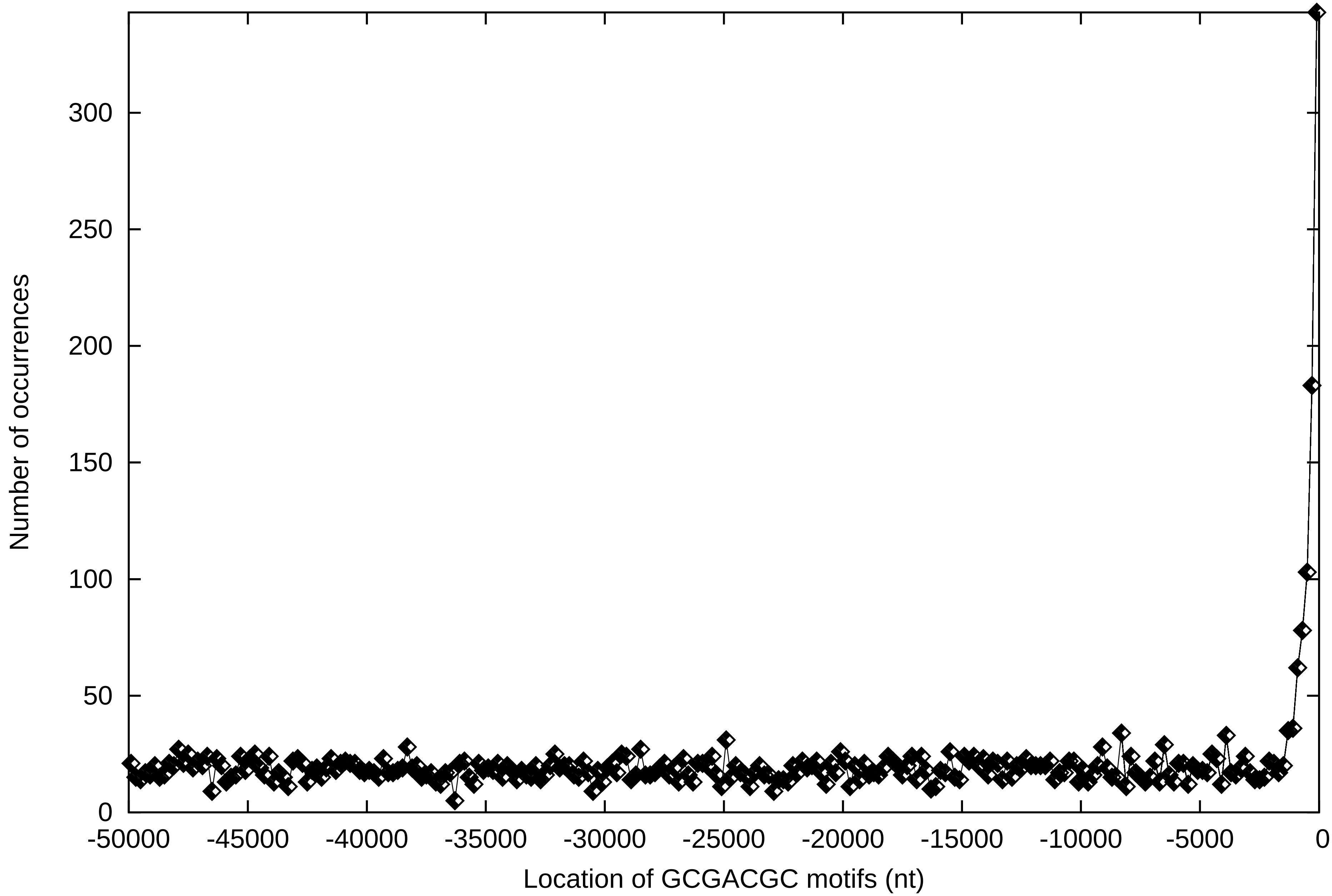

Number of occurrences
Location of GCGACGC motifs (nt)
0
50
100
150
200
250
300
-50000
-45000
-40000
-35000
-30000
-25000
-20000
-15000
-10000
-5000
0

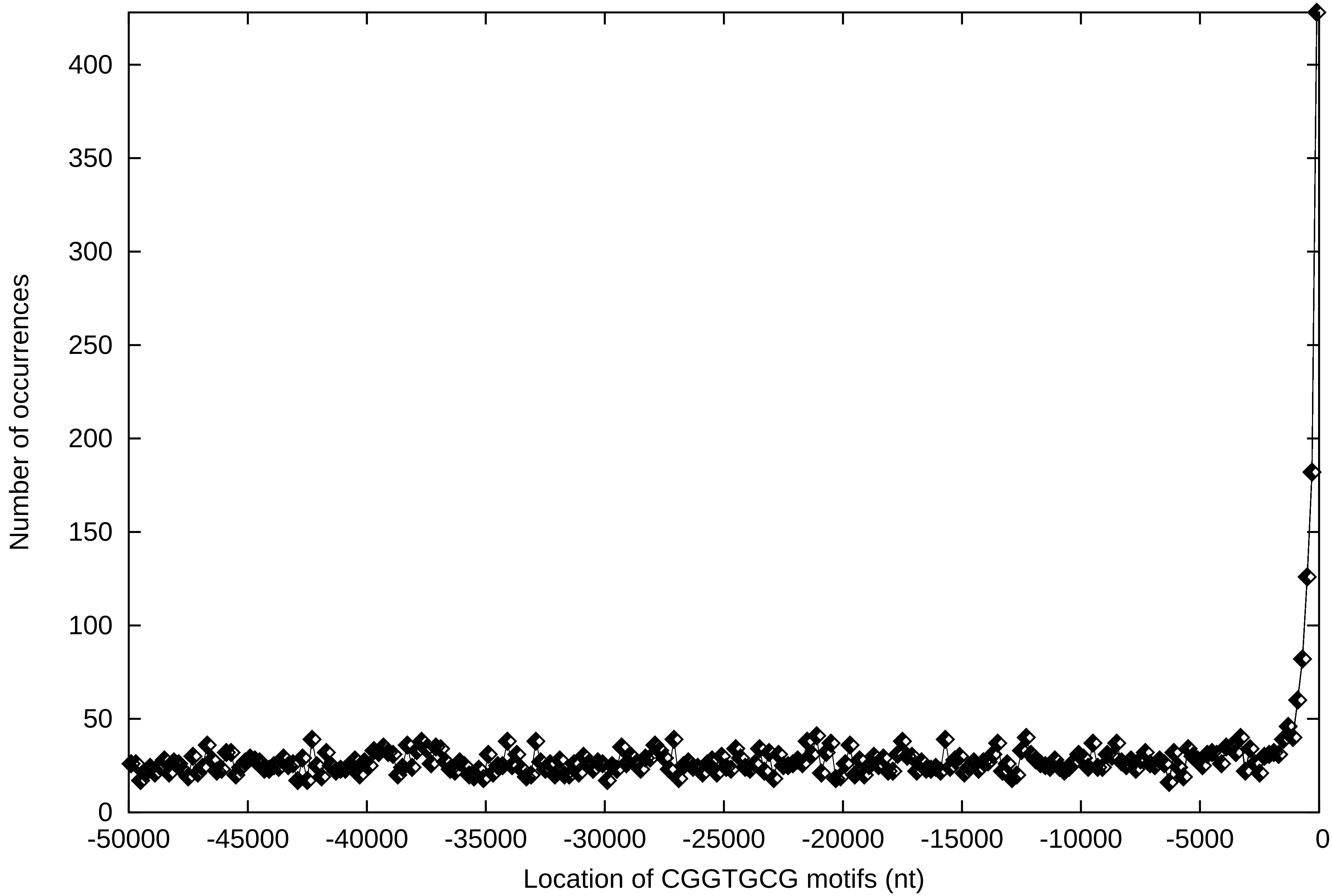
Number of occurrences
0
50
100
150
200
250
300
350
400
-50000
-45000
-40000
-35000
-30000
-25000
-20000
-15000
-10000
-5000
0
Location of CGGTGCG motifs (nt)

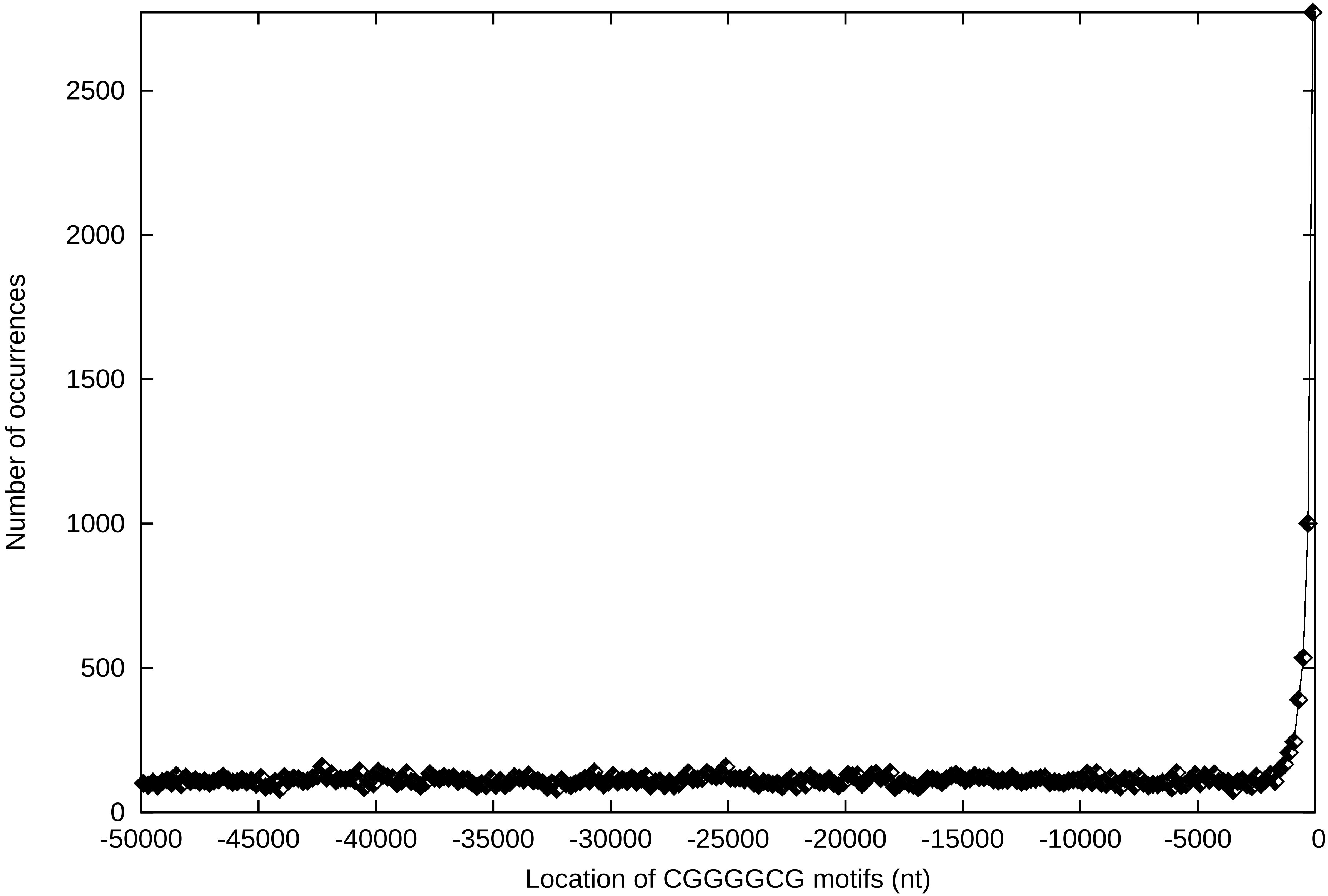

Number of occurrences
Location of CGGGGCG motifs (nt)
0
500
1000
1500
2000
2500
-50000
-45000
-40000
-35000
-30000
-25000
-20000
-15000
-10000
-5000
0

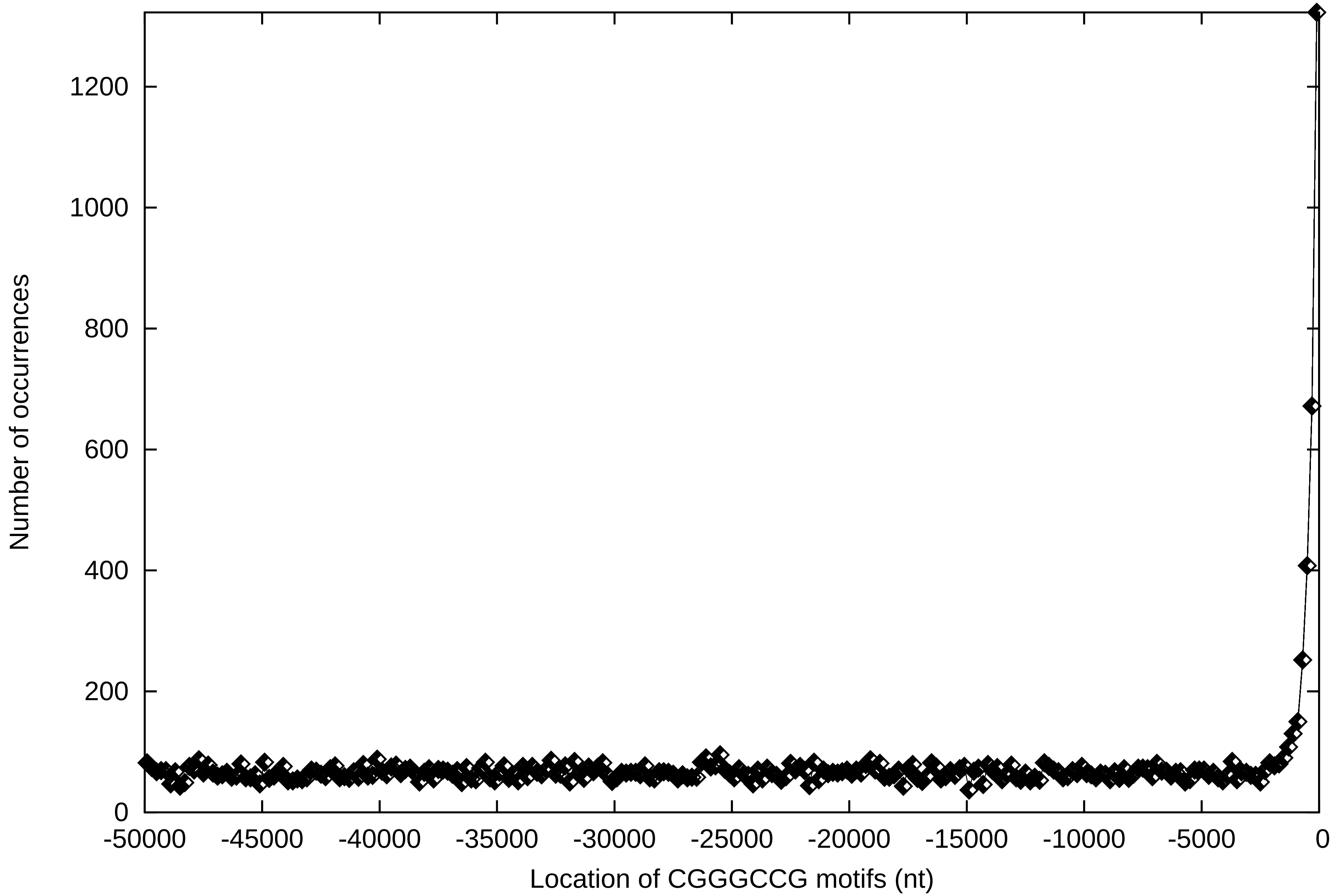

1200
1000
800
600
400
200
0
-50000
-45000
-40000
-35000
-30000
-25000
-20000
-15000
-10000
-5000
0
Number of occurrences
Location of CGGGCCG motifs (nt)

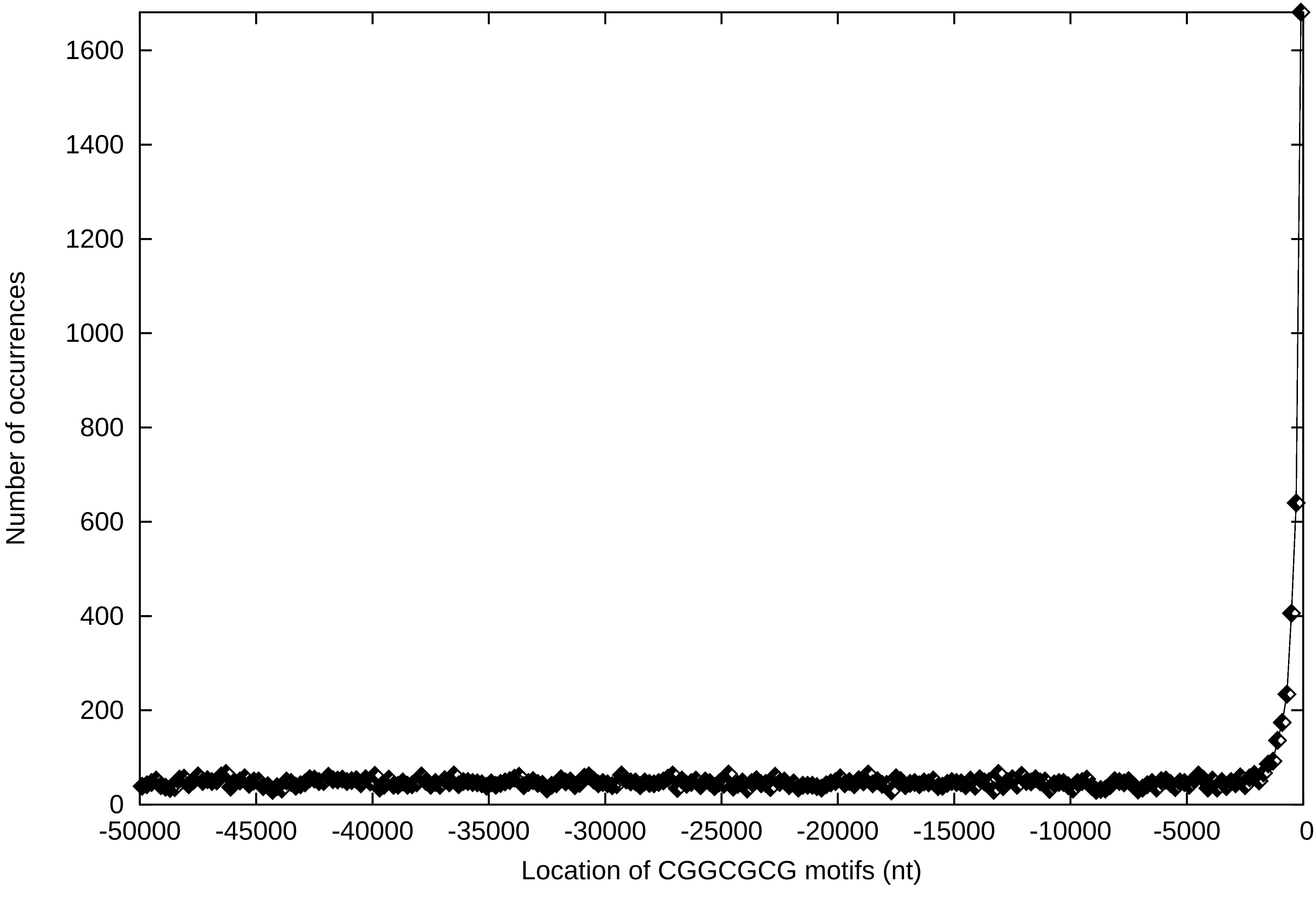

1600
1400
1200
1000
800
600
400
200
0
Number of occurrences
-50000
-45000
-40000
-35000
-30000
-25000
-20000
-15000
-10000
-5000
0
Location of CGGCGCG motifs (nt)

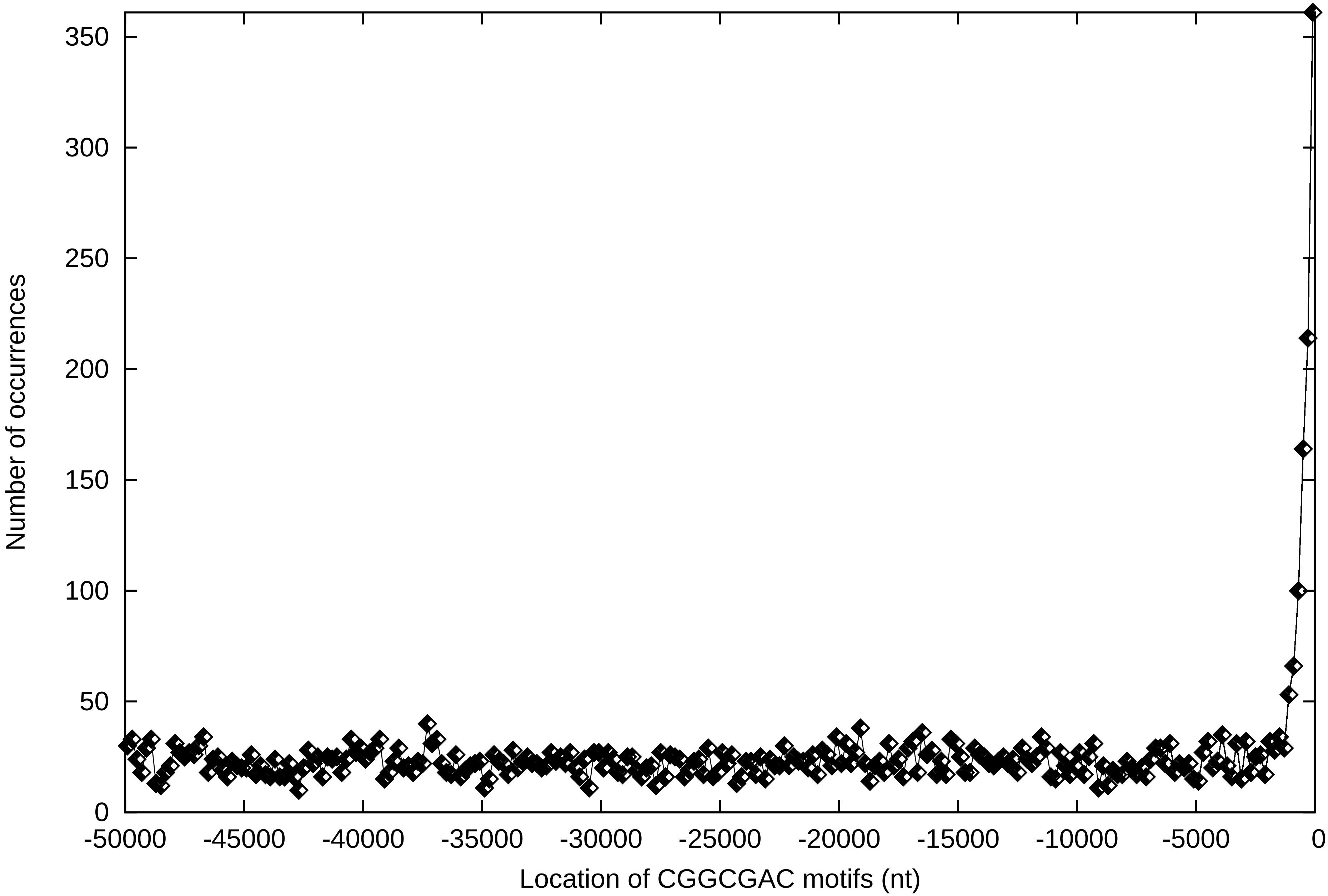
Number of occurrences
0
50
100
150
200
250
300
350
-50000
-45000
-40000
-35000
-30000
-25000
-20000
-15000
-10000
-5000
0
Location of CGGCGAC motifs (nt)

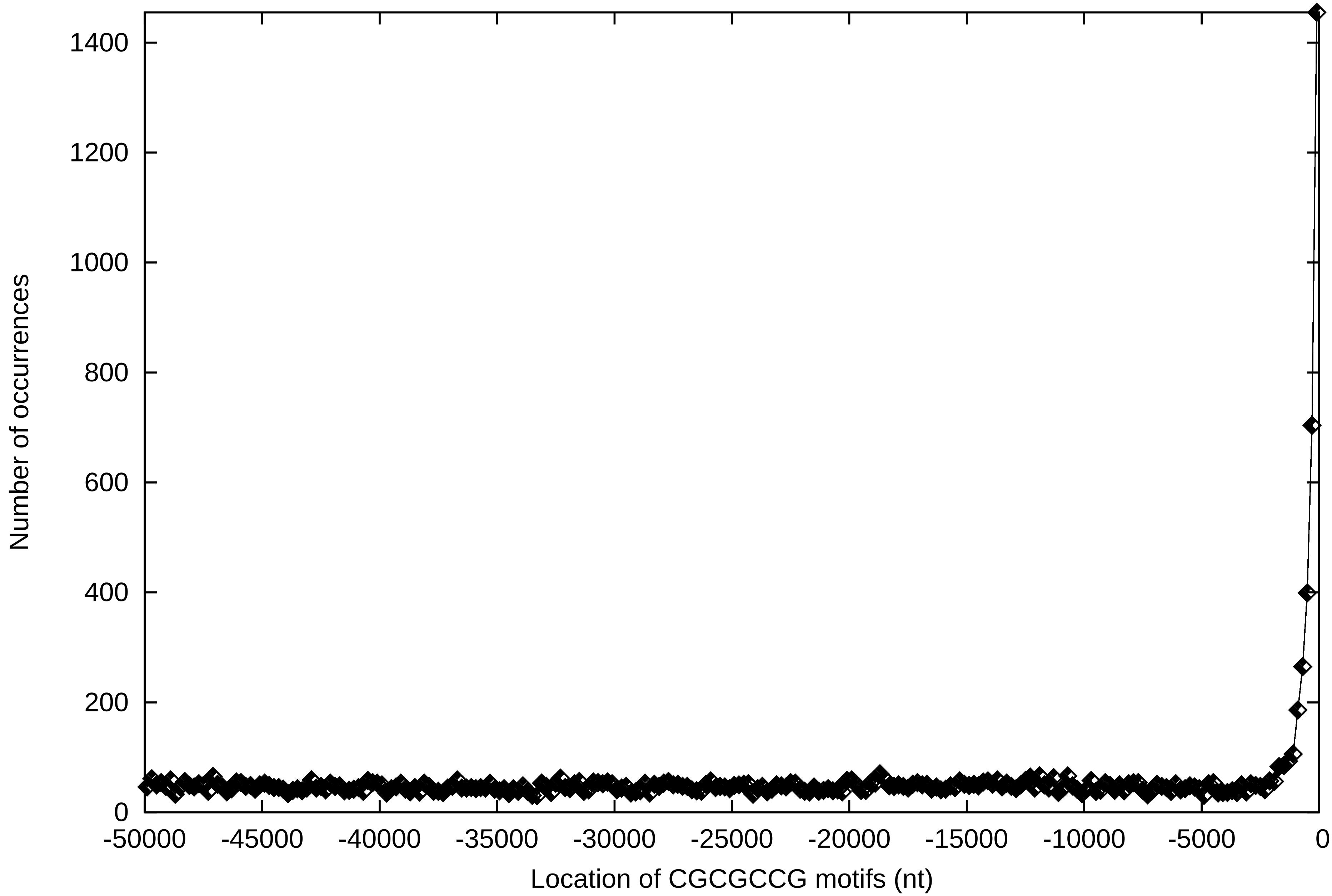

Number of occurrences
1400
1200
1000
800
600
400
200
0
-50000
-45000
-40000
-35000
-30000
-25000
-20000
-15000
-10000
-5000
0
Location of CGCGCCG motifs (nt)

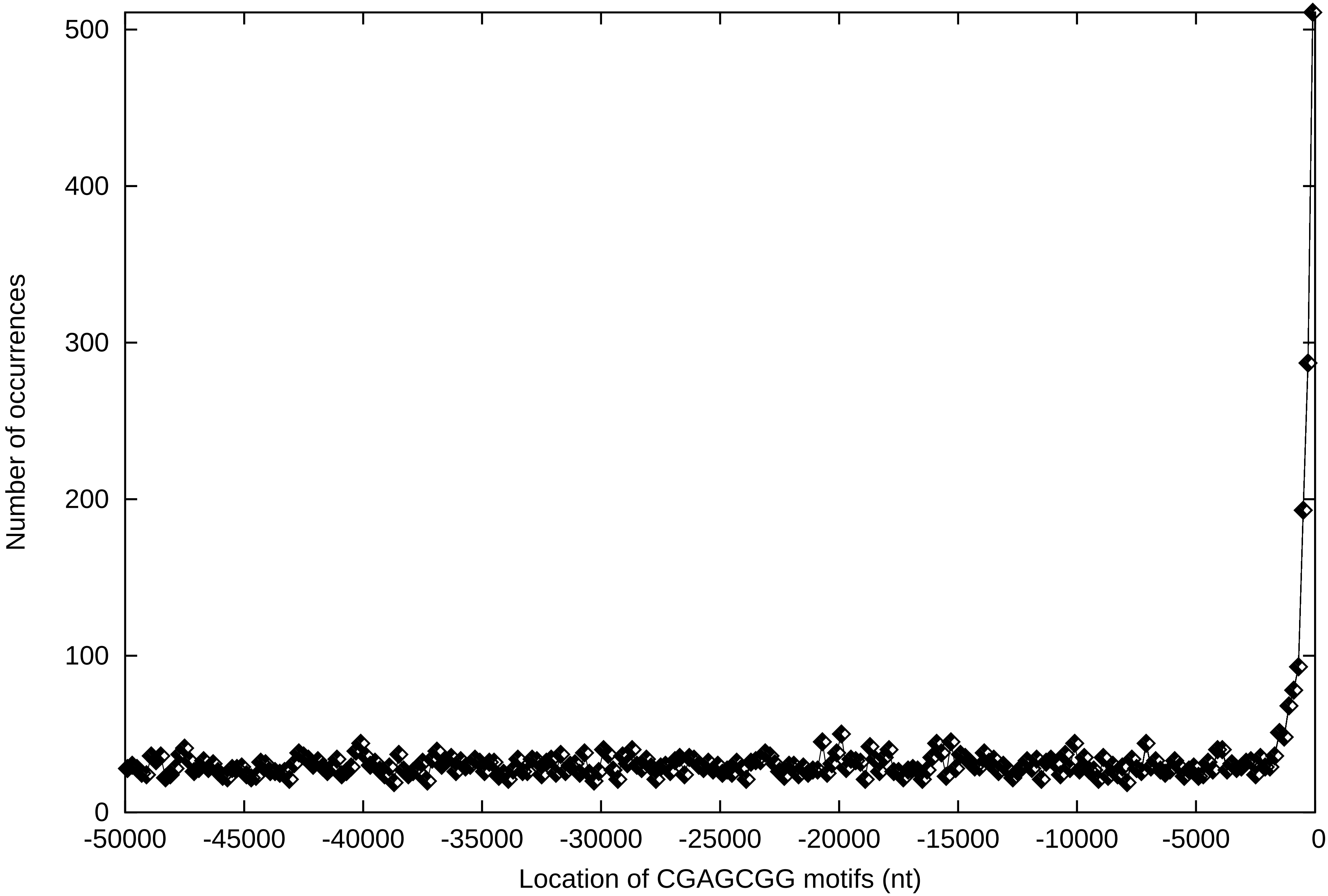

500
400
300
200
100
0
-50000
-45000
-40000
-35000
-30000
-25000
-20000
-15000
-10000
-5000
0
Number of occurrences
Location of CGAGCGG motifs (nt)

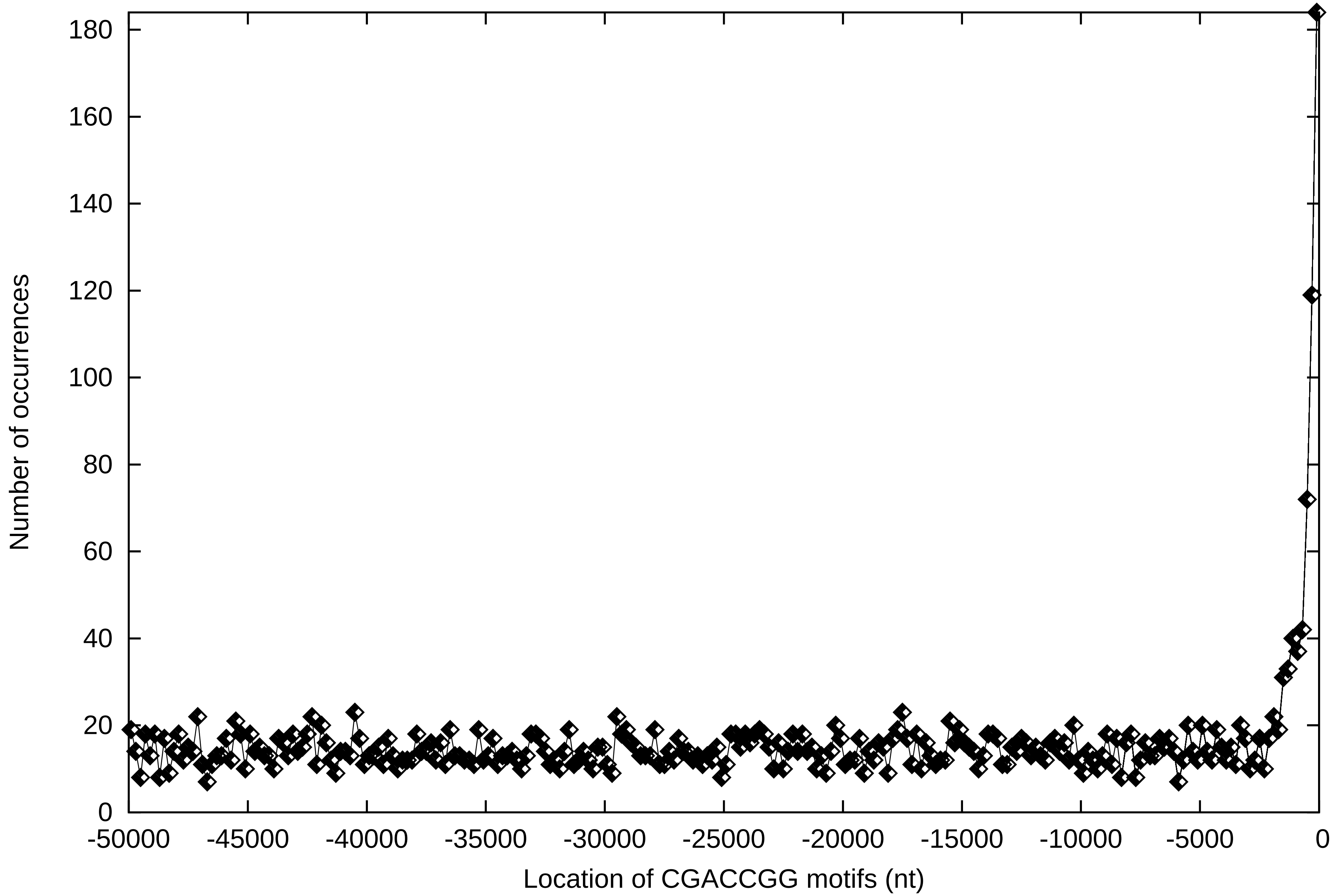
Number of occurrences
Location of CGACCGG motifs (nt)
0
20
40
60
80
100
120
140
160
180
-50000
-45000
-40000
-35000
-30000
-25000
-20000
-15000
-10000
-5000
0

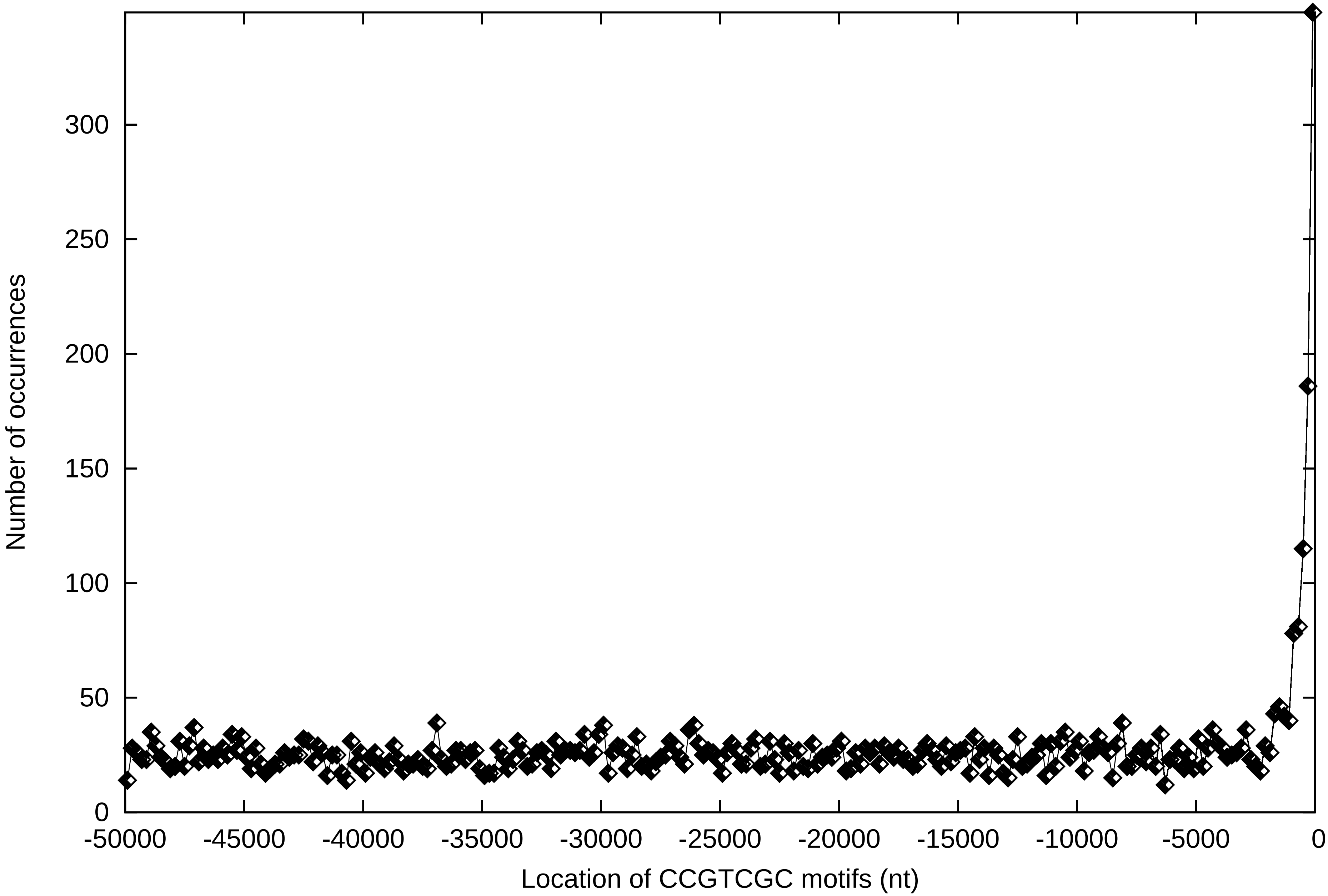
Number of occurrences
Location of CCGTCGC motifs (nt)
0
50
100
150
200
250
300
-50000
-45000
-40000
-35000
-30000
-25000
-20000
-15000
-10000
-5000
0

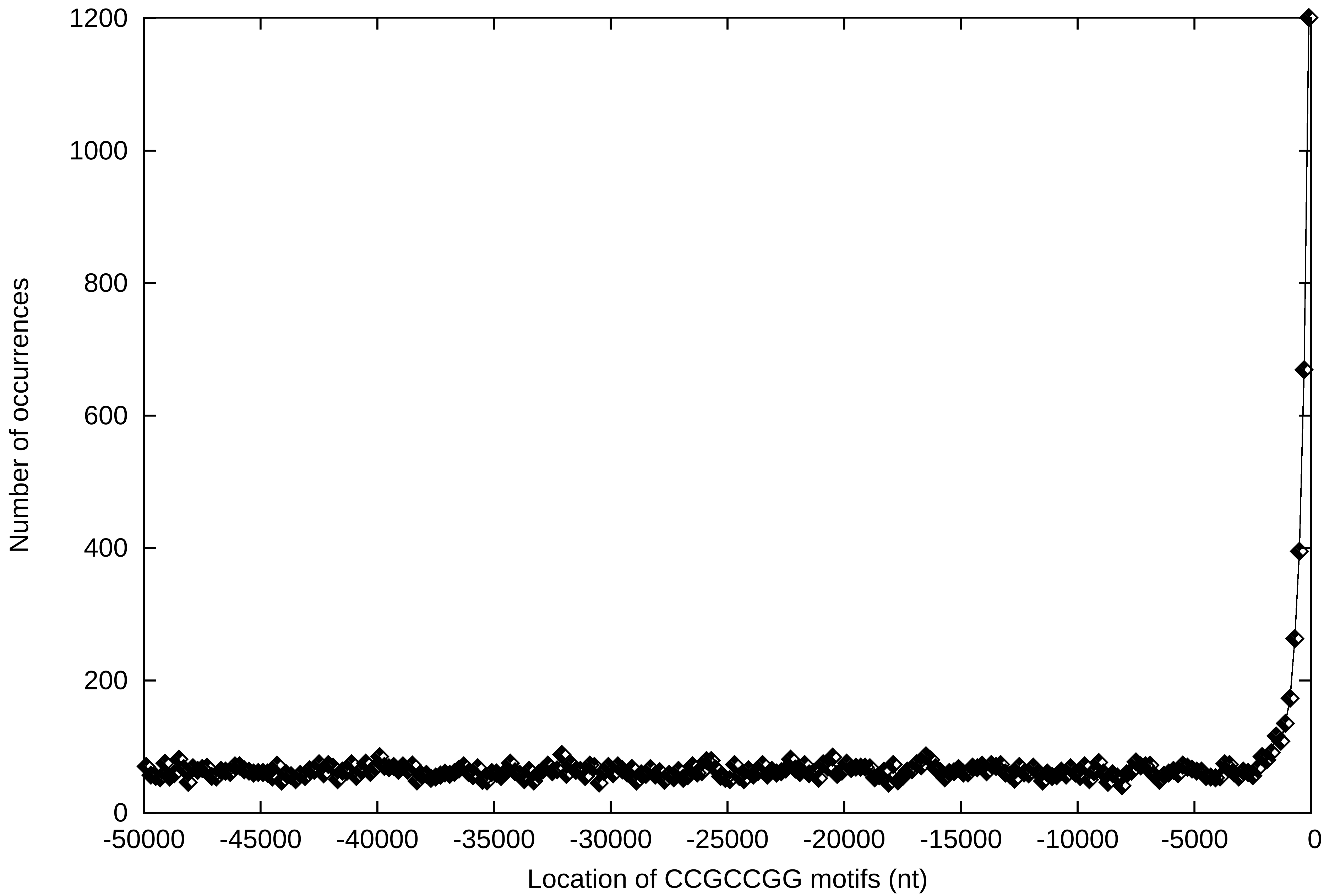

Number of occurrences
Location of CCGCCGG motifs (nt)
0
200
400
600
800
1000
1200
-50000
-45000
-40000
-35000
-30000
-25000
-20000
-15000
-10000
-5000
0

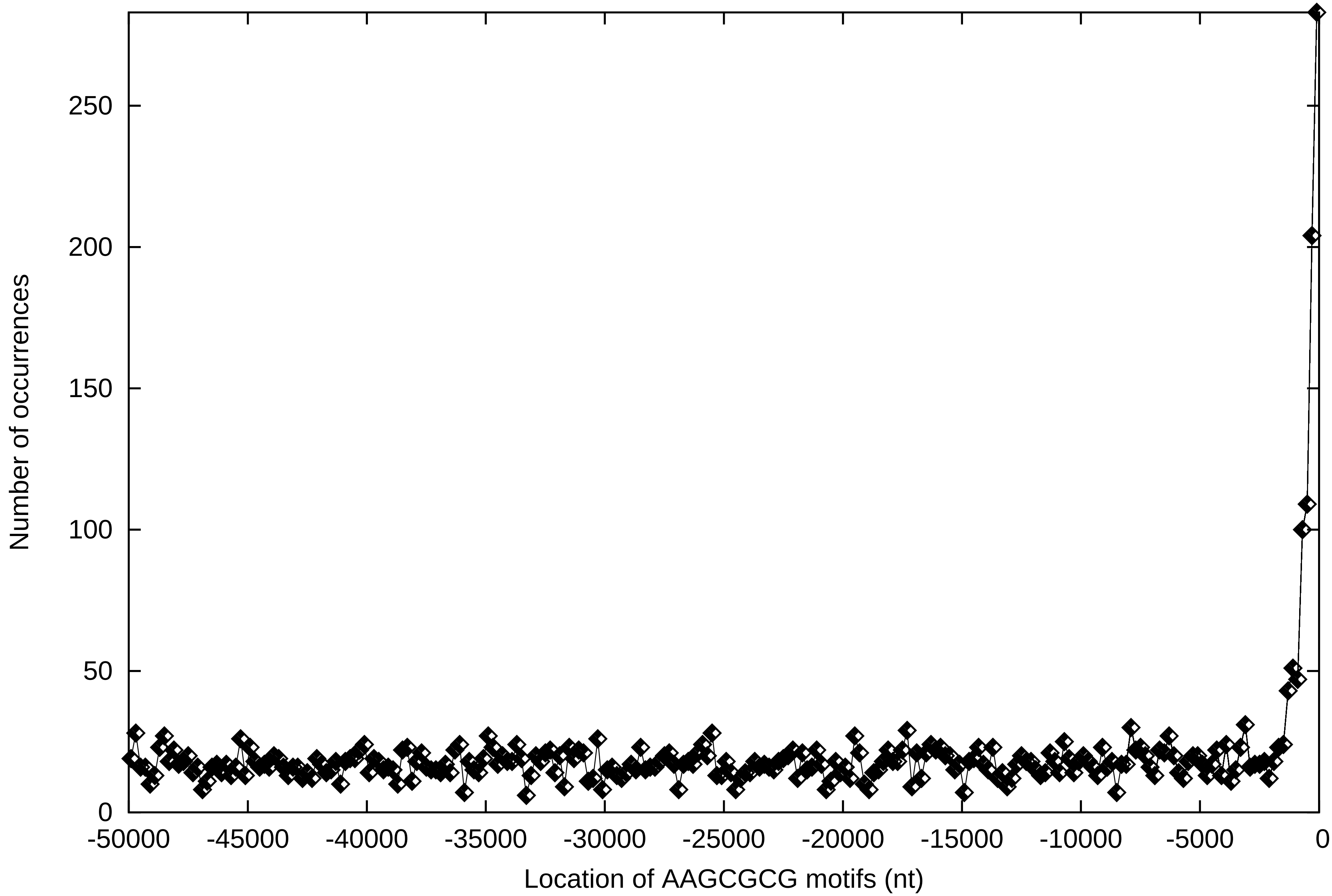

Number of occurrences
Location of AAGCGCG motifs (nt)
0
50
100
150
200
250
-50000
-45000
-40000
-35000
-30000
-25000
-20000
-15000
-10000
-5000
0

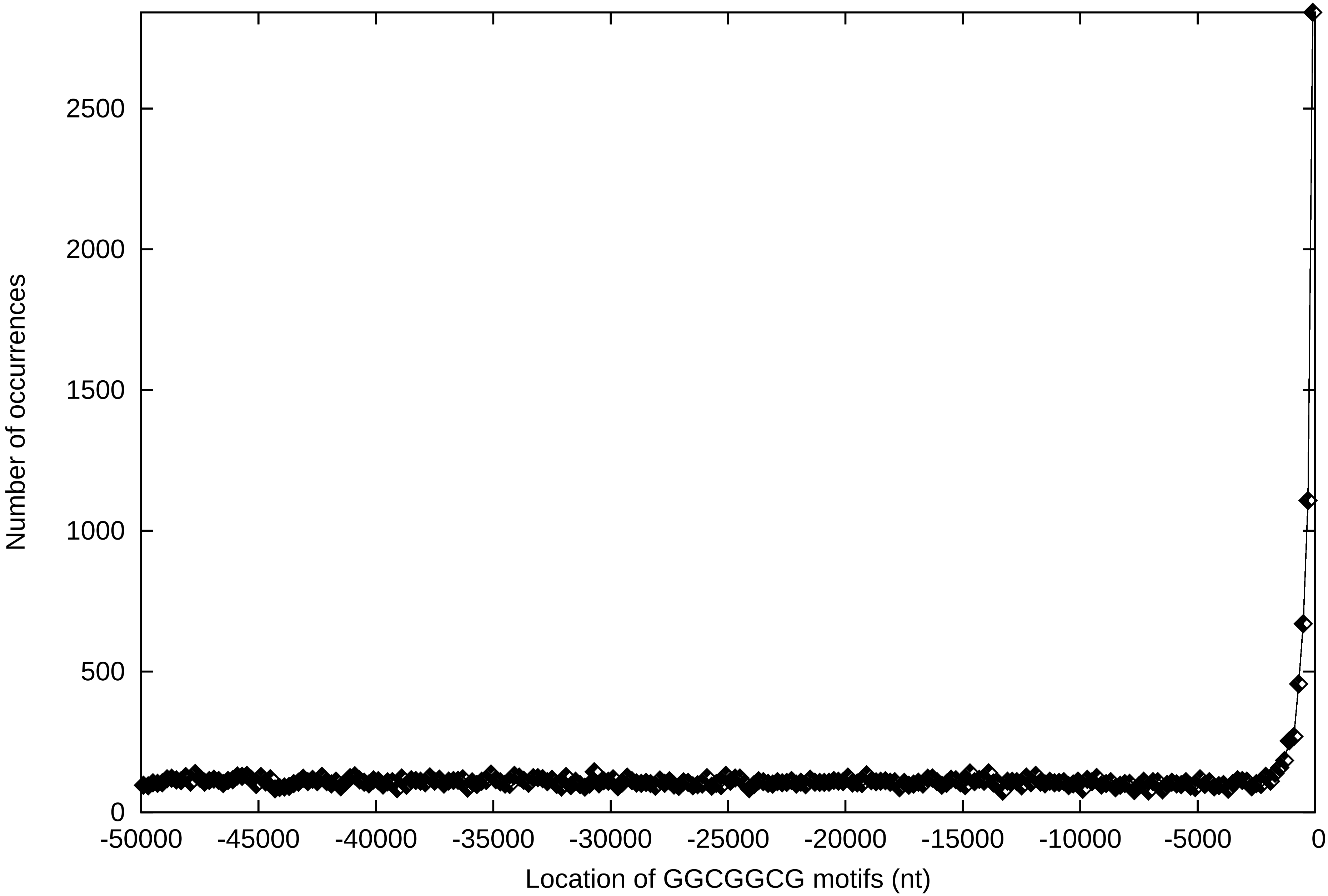
Number of occurrences
0
500
1000
1500
2000
2500
-50000
-45000
-40000
-35000
-30000
-25000
-20000
-15000
-10000
-5000
0
Location of GGCGGCG motifs (nt)

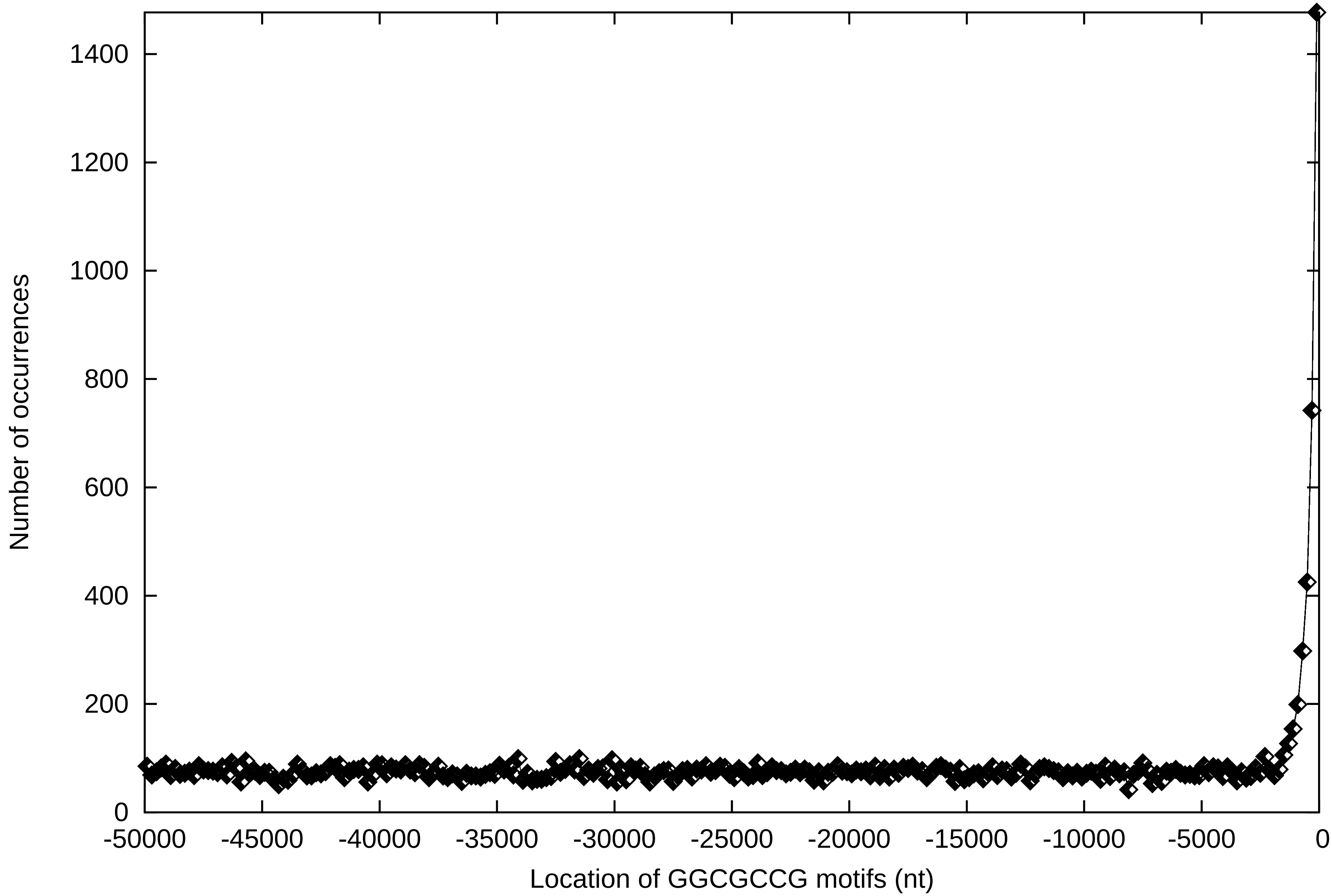

1400
1200
1000
800
600
400
200
0
-50000
-45000
-40000
-35000
-30000
-25000
-20000
-15000
-10000
-5000
0
Number of occurrences
Location of GGCGCCG motifs (nt)

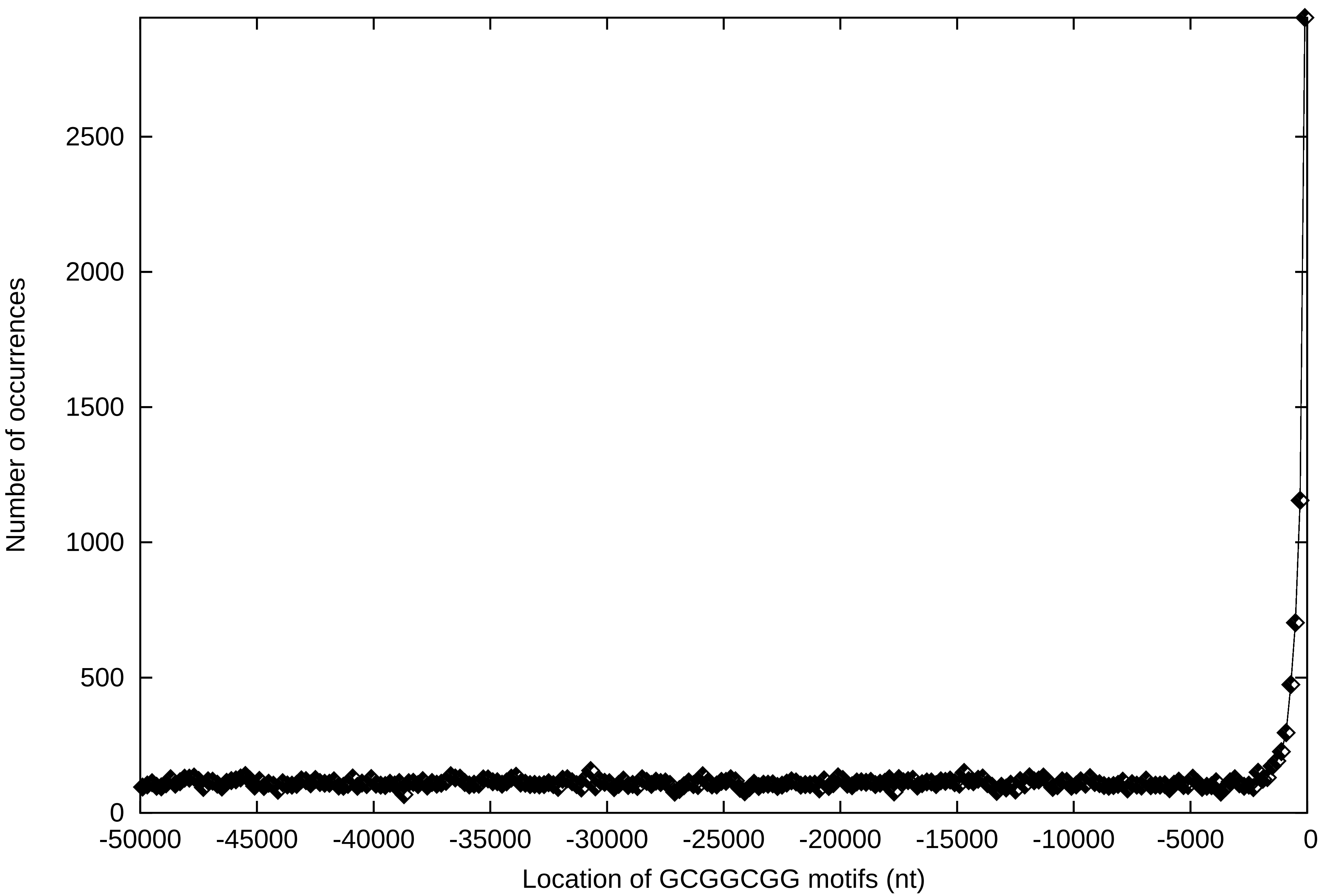

Number of occurrences
0
500
1000
1500
2000
2500
-50000
-45000
-40000
-35000
-30000
-25000
-20000
-15000
-10000
-5000
0
Location of GCGGCGG motifs (nt)

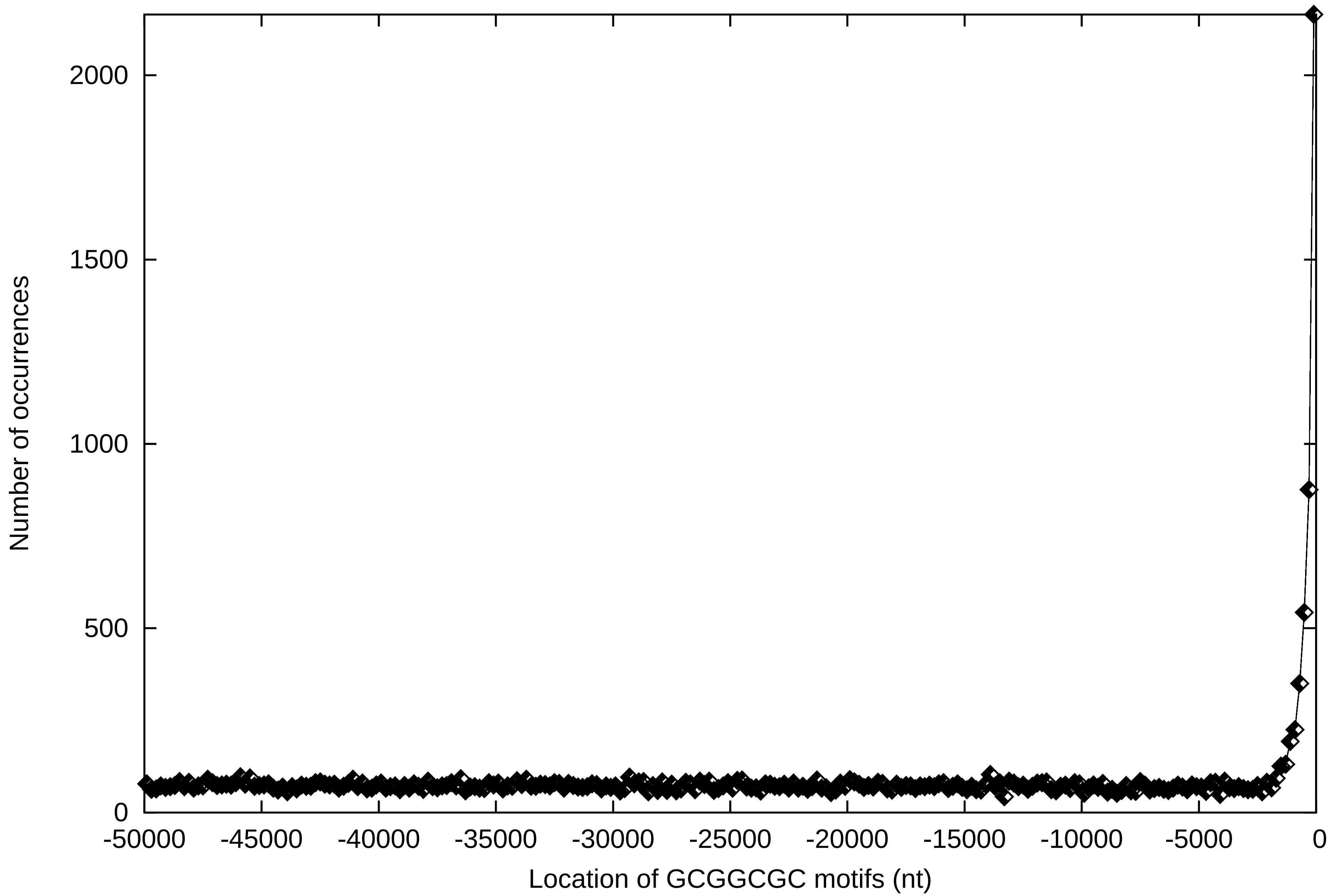

Number of occurrences
2000
1500
1000
500
0
-50000
-45000
-40000
-35000
-30000
-25000
-20000
-15000
-10000
-5000
0
Location of GCGGCGC motifs (nt)

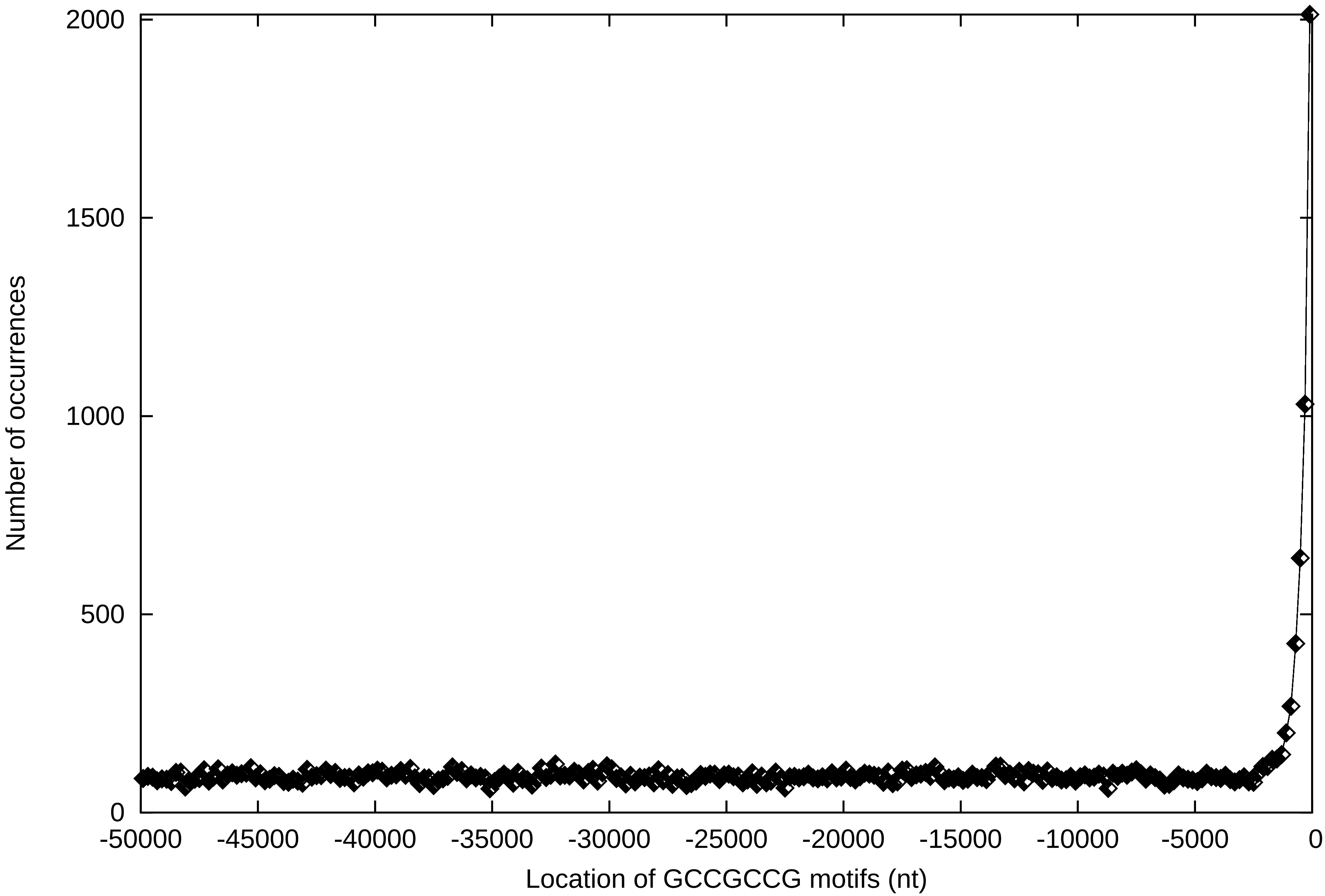
Number of occurrences
2000
1500
1000
500
0
-50000
-45000
-40000
-35000
-30000
-25000
-20000
-15000
-10000
-5000
0
Location of GCCGCCG motifs (nt)

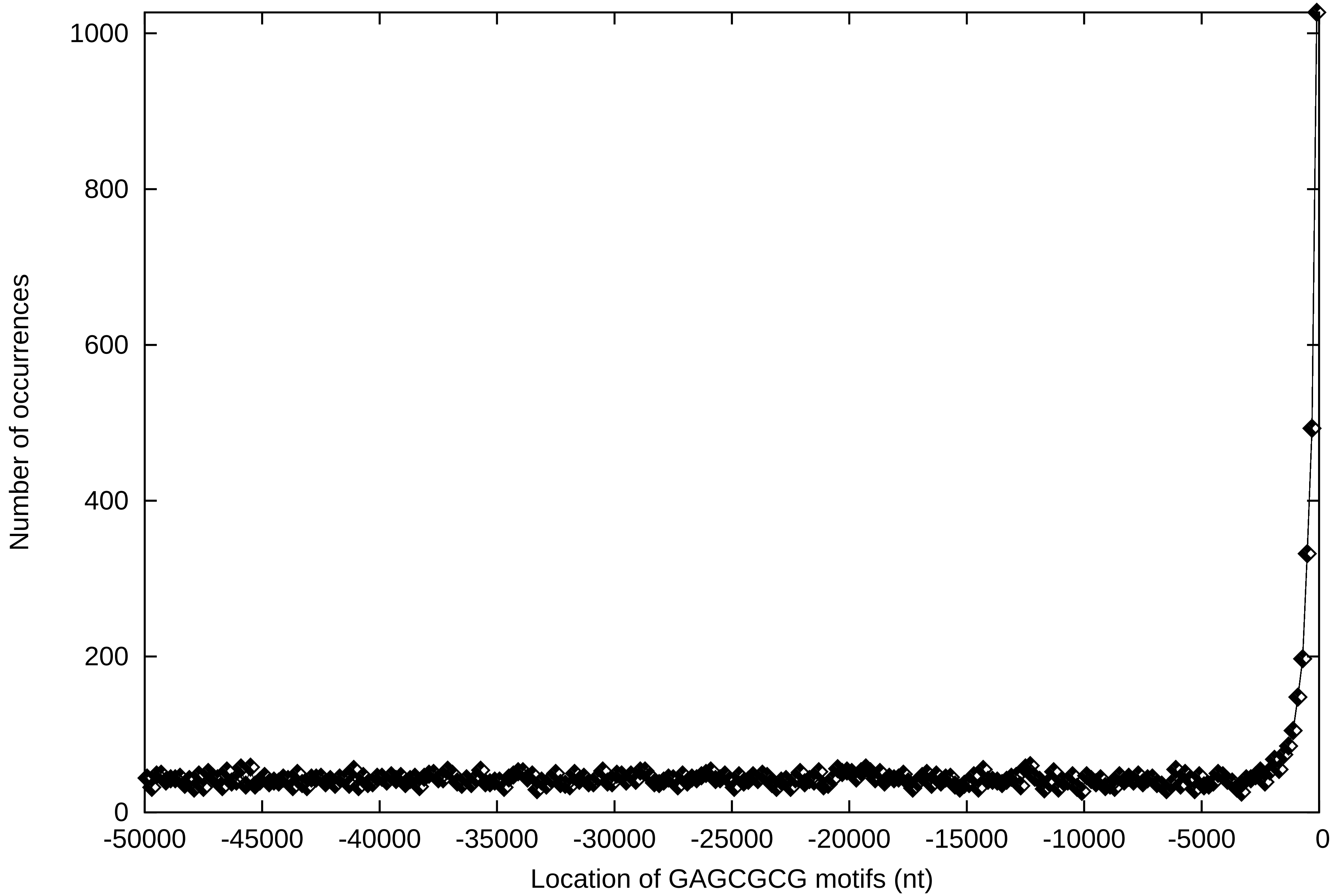
1000
800
600
400
200
0
-50000
-45000
-40000
-35000
-30000
-25000
-20000
-15000
-10000
-5000
0
Number of occurrences
Location of GAGCGCG motifs (nt)

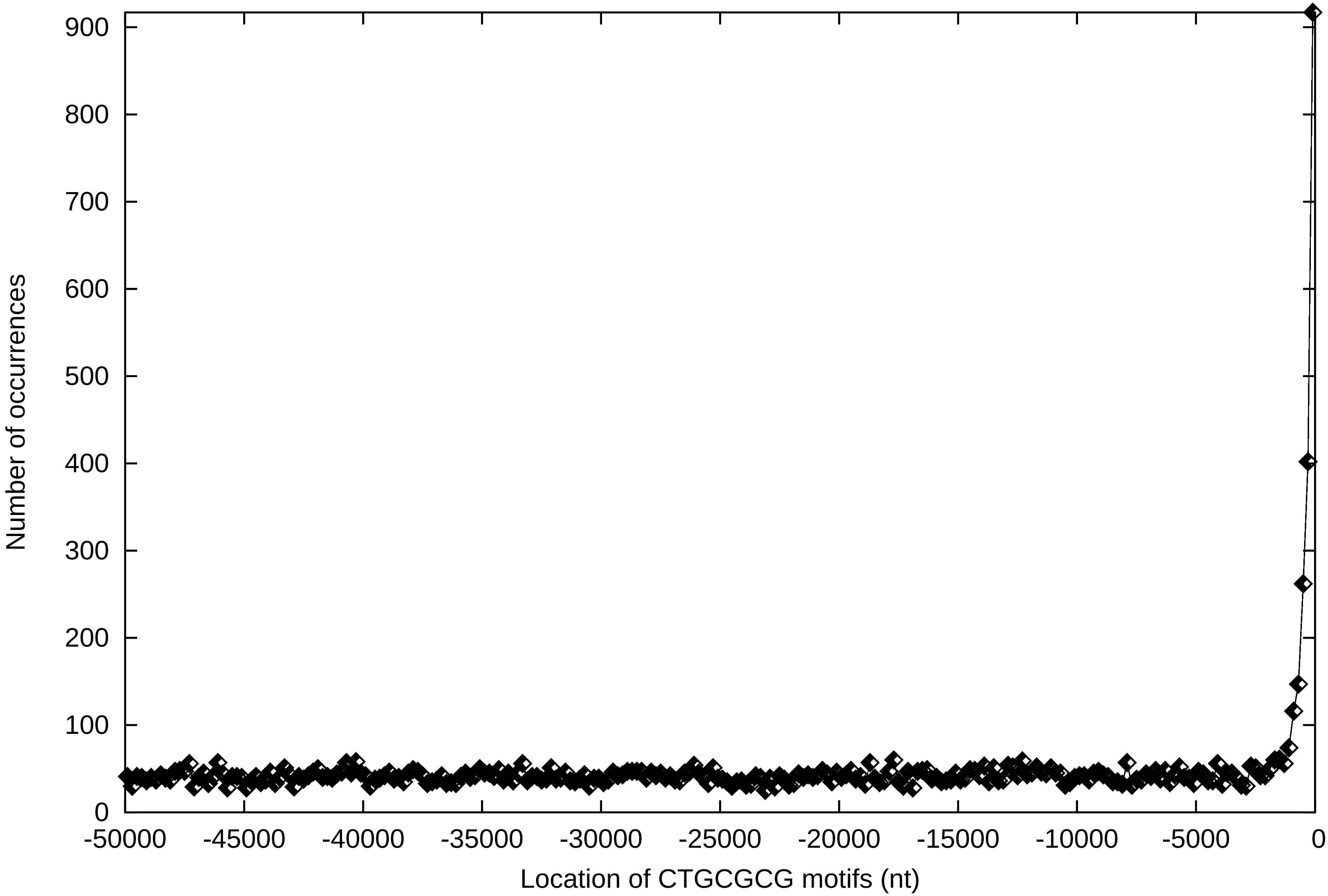

Number of occurrences
900
800
700
600
500
400
300
200
100
0
-50000
-45000
-40000
-35000
-30000
-25000
-20000
-15000
-10000
-5000
0
Location of CTGCGCG motifs (nt)

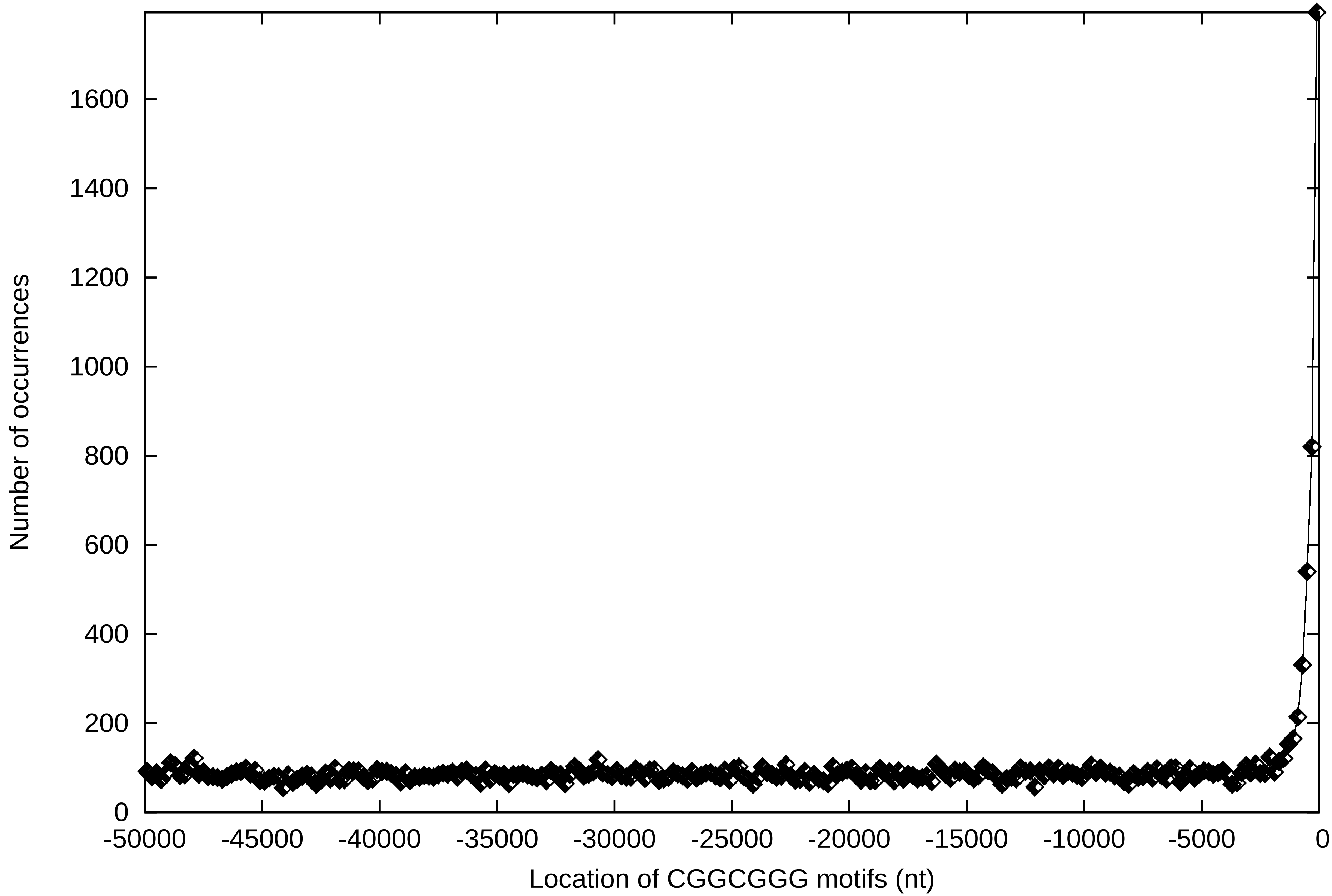

Number of occurrences
0
200
400
600
800
1000
1200
1400
1600
-50000
-45000
-40000
-35000
-30000
-25000
-20000
-15000
-10000
-5000
0
Location of CGGCGGG motifs (nt)

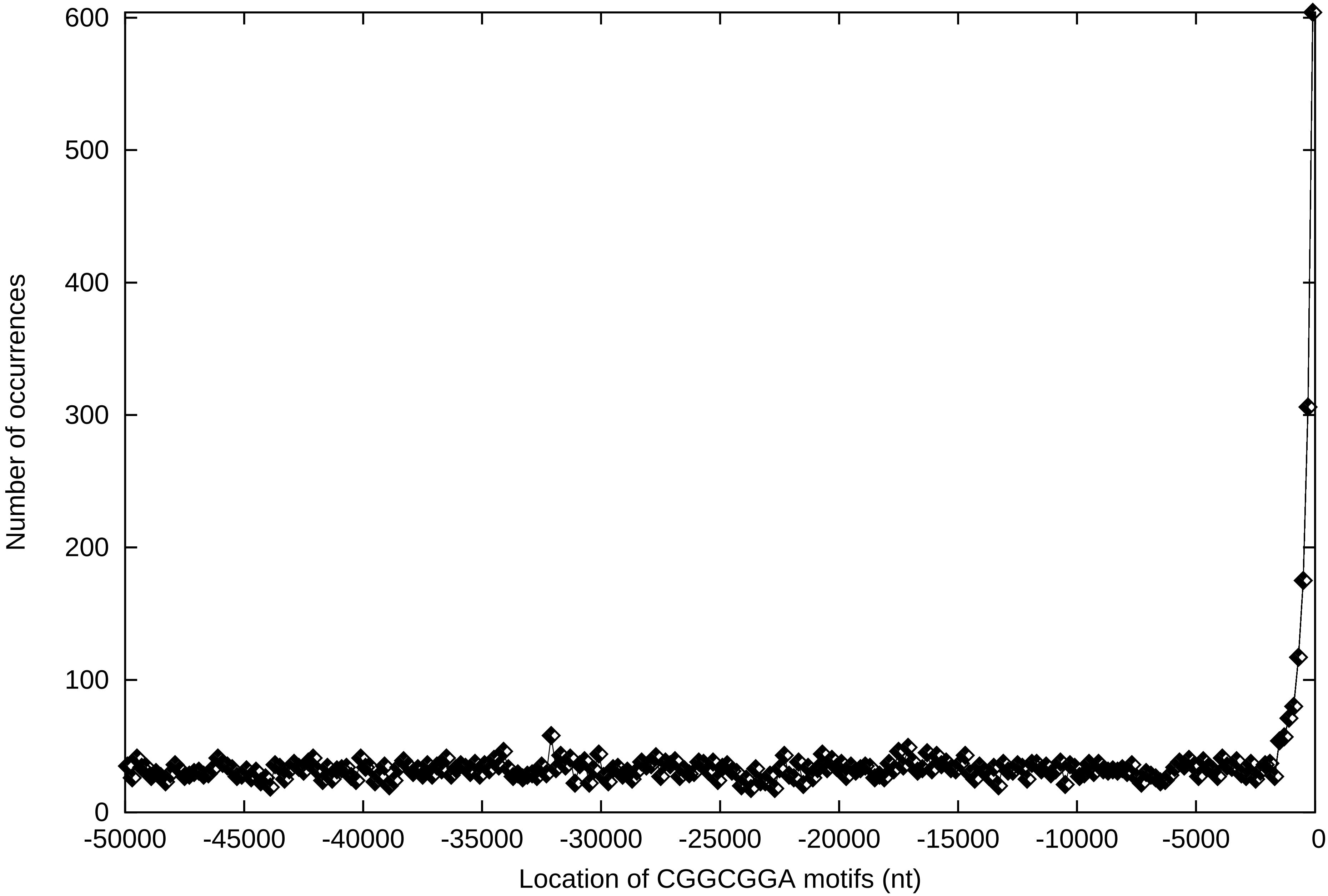

600
500
400
300
200
100
0
Number of occurrences
-50000
-45000
-40000
-35000
-30000
-25000
-20000
-15000
-10000
-5000
0
Location of CGGCGGA motifs (nt)

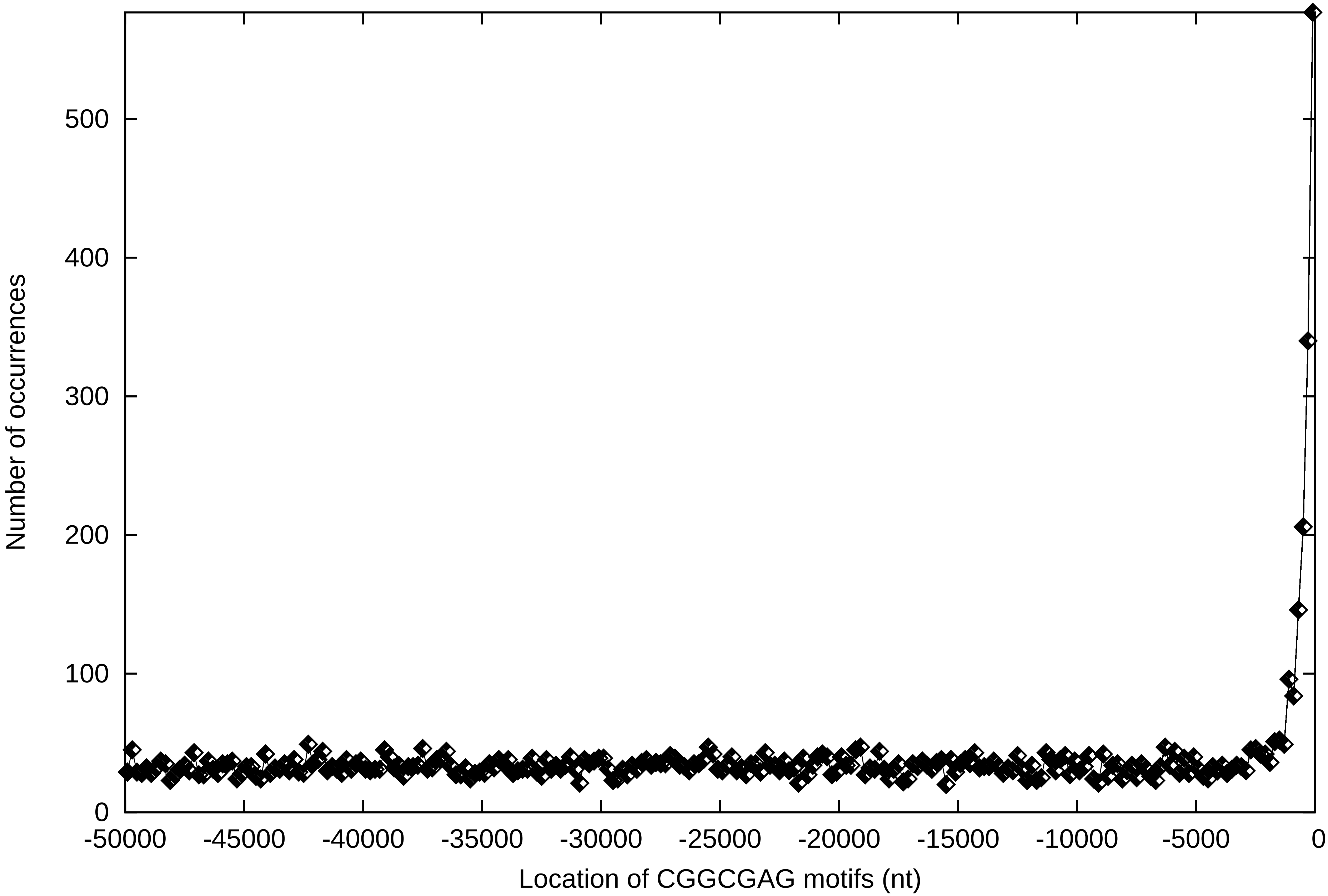

Number of occurrences
0
100
200
300
400
500
-50000
-45000
-40000
-35000
-30000
-25000
-20000
-15000
-10000
-5000
0
Location of CGGCGAG motifs (nt)

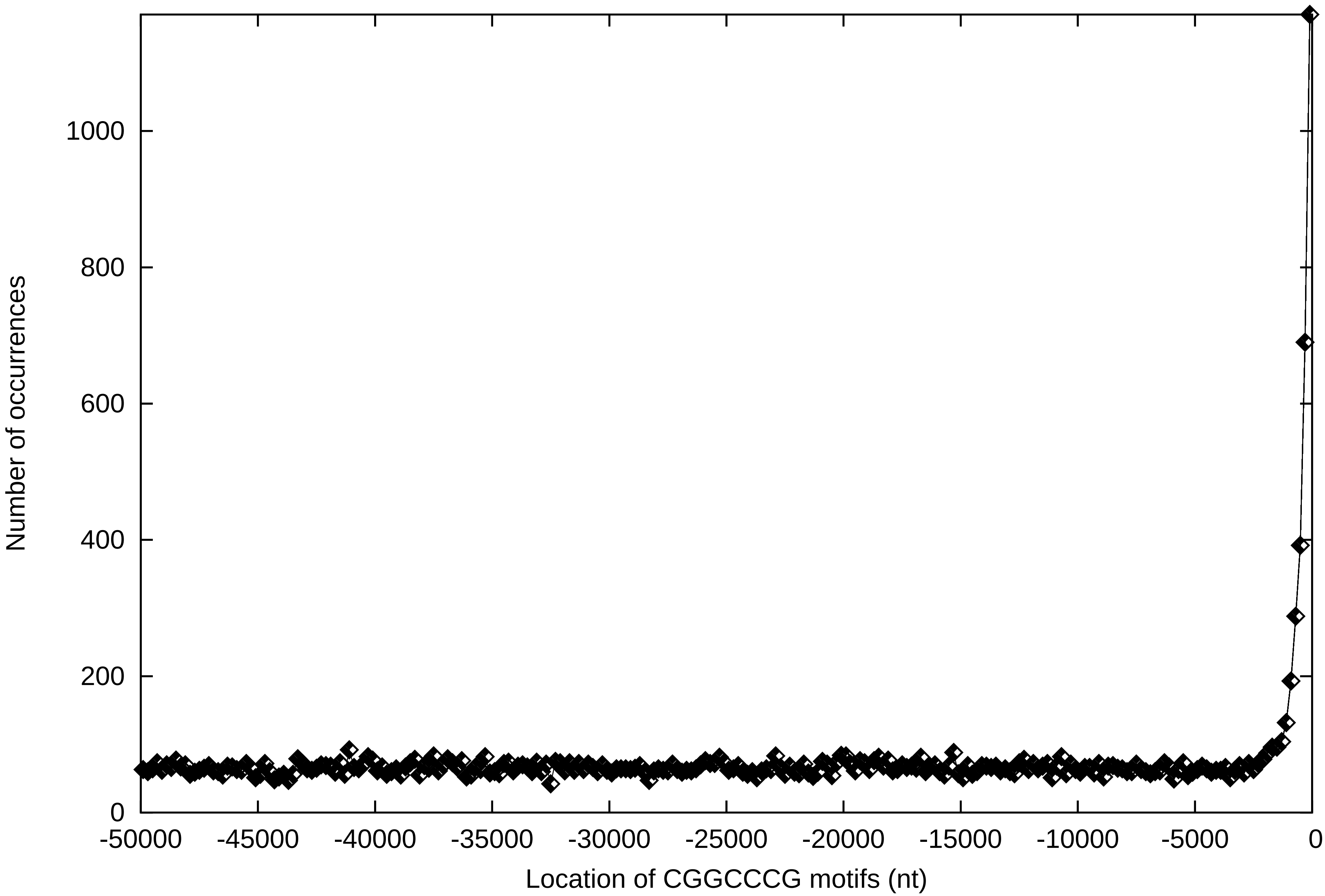

Number of occurrences
0
200
400
600
800
1000
-50000
-45000
-40000
-35000
-30000
-25000
-20000
-15000
-10000
-5000
0
Location of CGGCCCG motifs (nt)

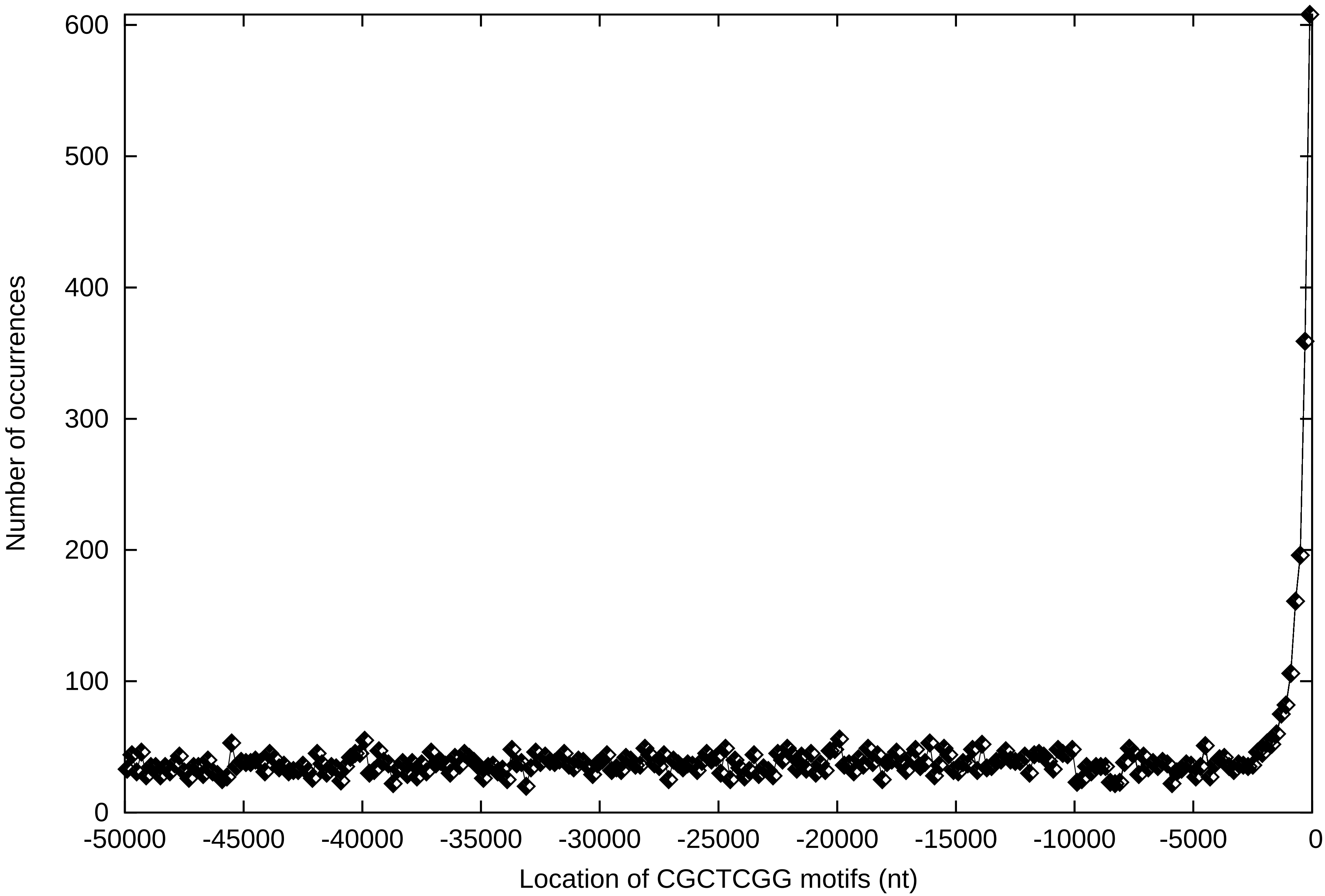

Number of occurrences
0
100
200
300
400
500
600
-50000
-45000
-40000
-35000
-30000
-25000
-20000
-15000
-10000
-5000
0
Location of CGCTCGG motifs (nt)

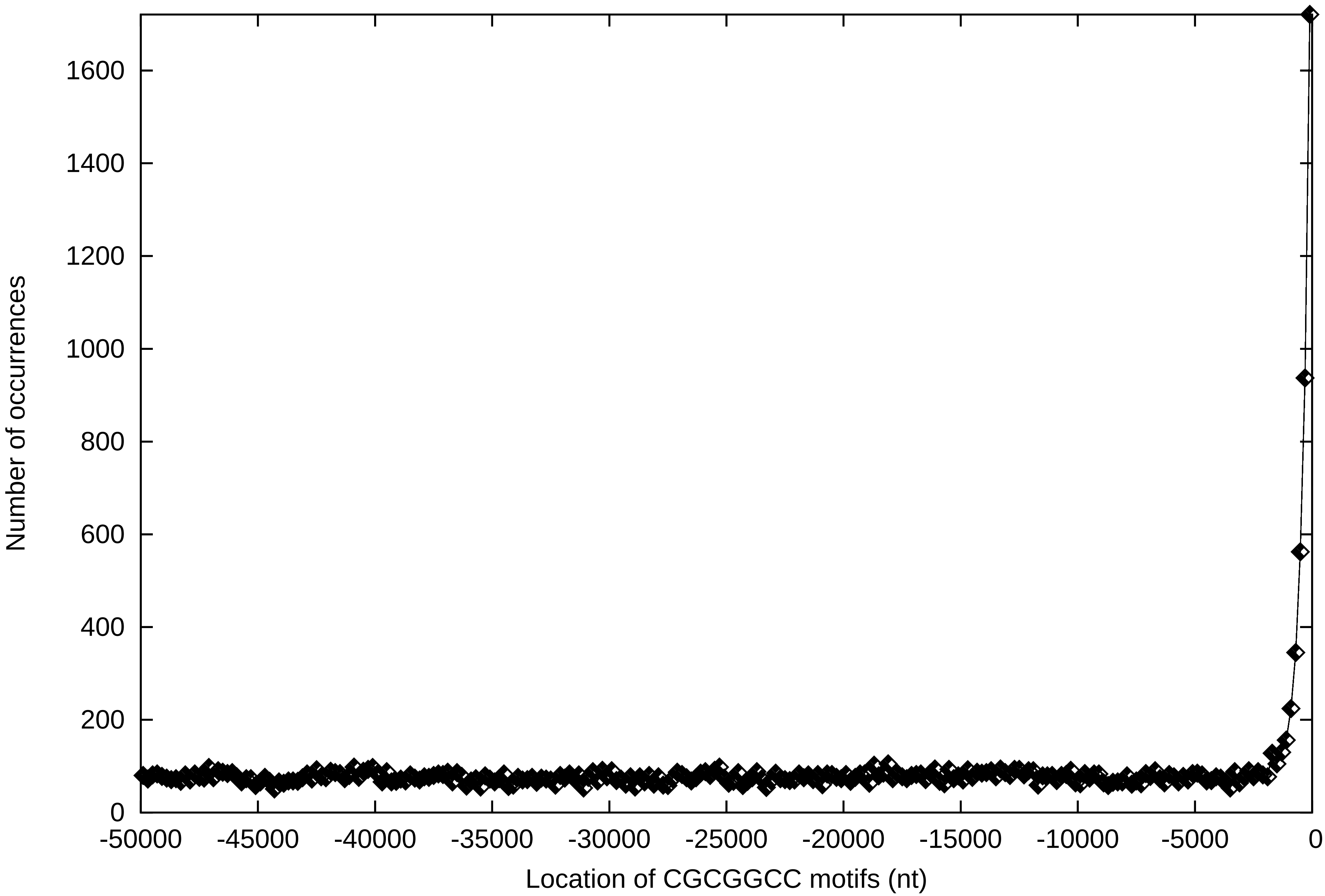

1600
1400
1200
1000
800
600
400
200
0
Number of occurrences
-50000
-45000
-40000
-35000
-30000
-25000
-20000
-15000
-10000
-5000
0
Location of CGCGGCC motifs (nt)

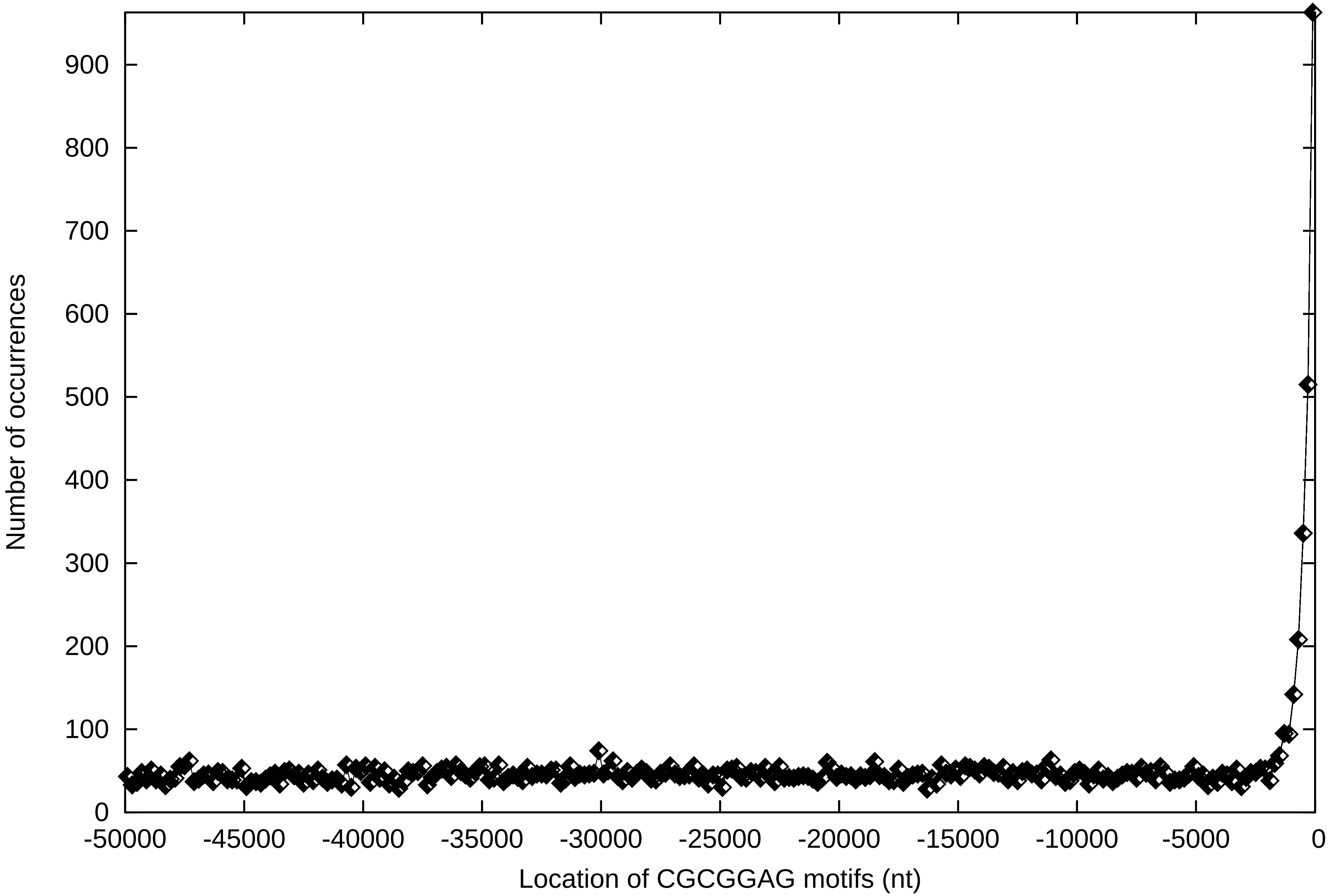
Number of occurrences
900
800
700
600
500
400
300
200
100
0
-50000
-45000
-40000
-35000
-30000
-25000
-20000
-15000
-10000
-5000
0
Location of CGCGGAG motifs (nt)

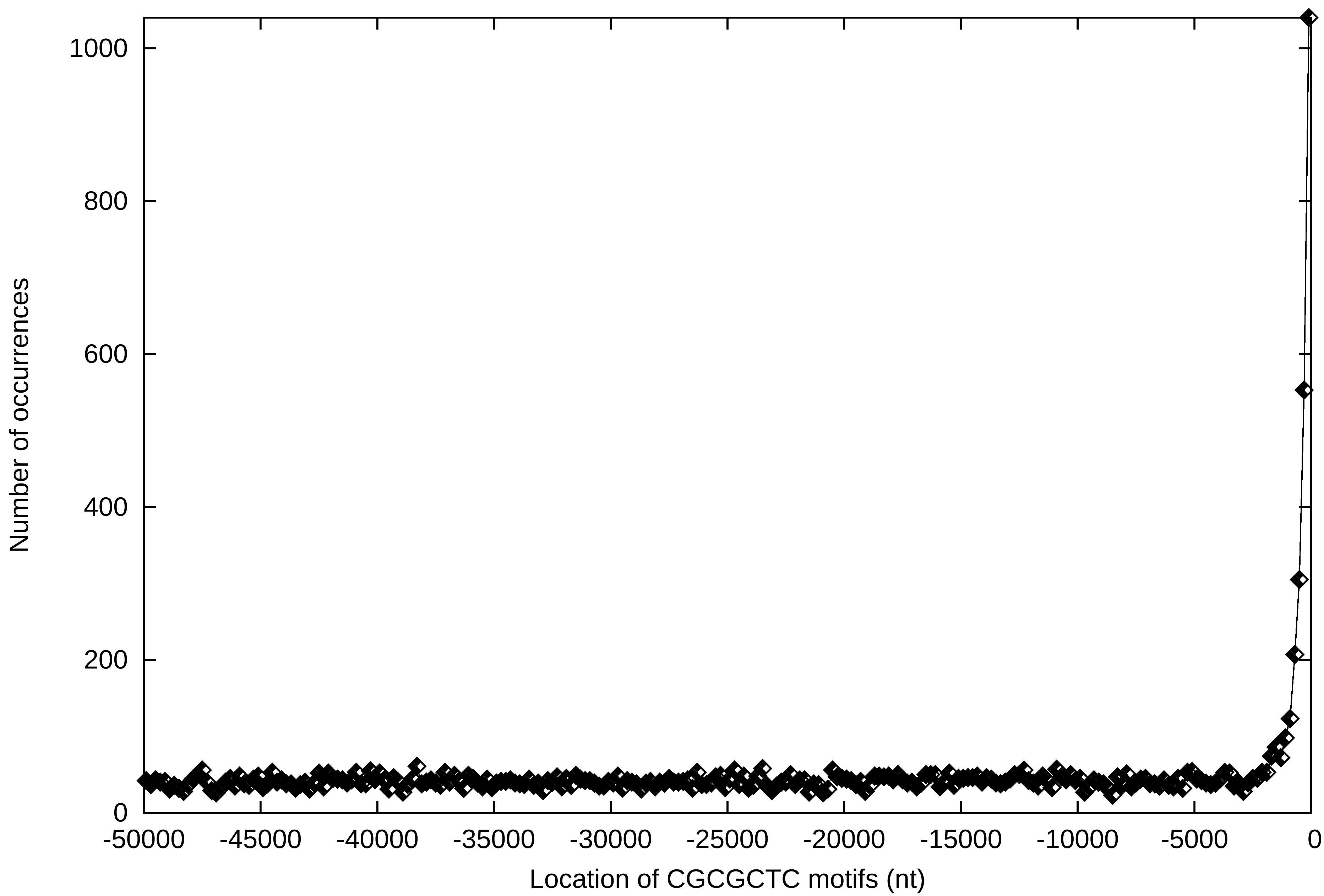

1000
800
600
400
200
0
Number of occurrences
-50000
-45000
-40000
-35000
-30000
-25000
-20000
-15000
-10000
-5000
0
Location of CGCGCTC motifs (nt)

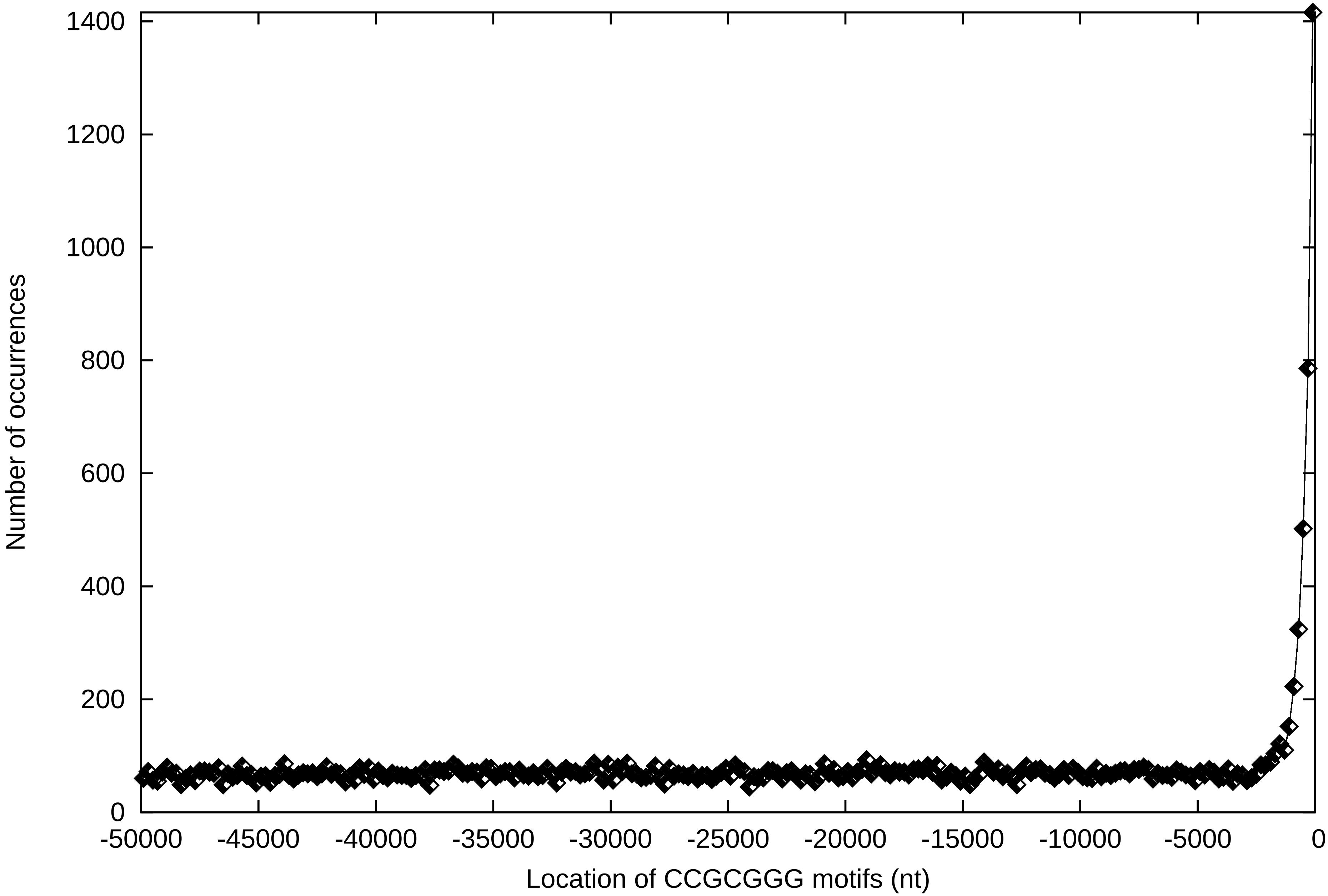

Number of occurrences
1400
1200
1000
800
600
400
200
0
-50000
-45000
-40000
-35000
-30000
-25000
-20000
-15000
-10000
-5000
0
Location of CCGCGGG motifs (nt)

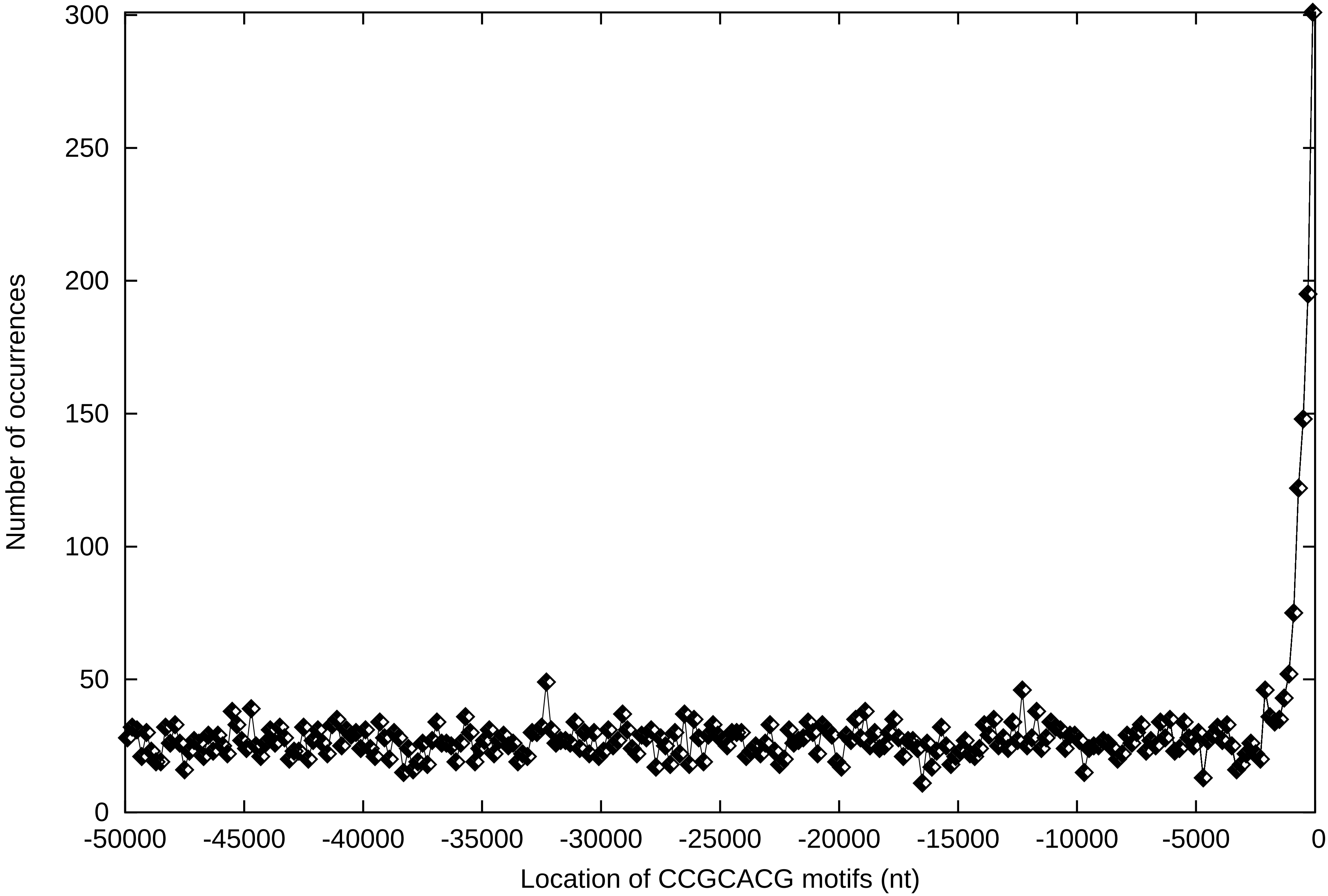

300
250
200
150
100
50
0
-50000
-45000
-40000
-35000
-30000
-25000
-20000
-15000
-10000
-5000
0
Number of occurrences
Location of CCGCACG motifs (nt)

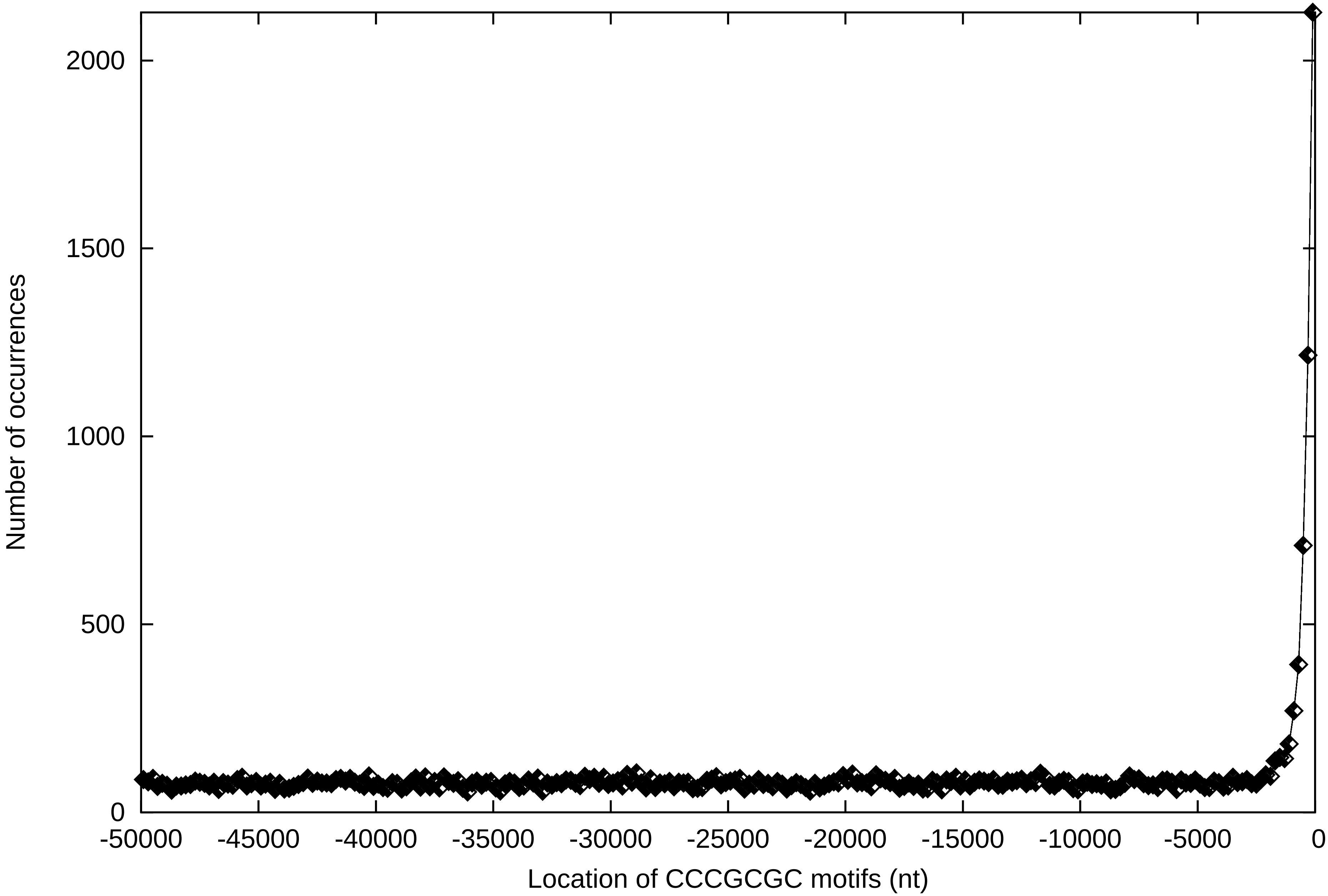

Number of occurrences
2000
1500
1000
500
0
-50000
-45000
-40000
-35000
-30000
-25000
-20000
-15000
-10000
-5000
0
Location of CCCGCGC motifs (nt)

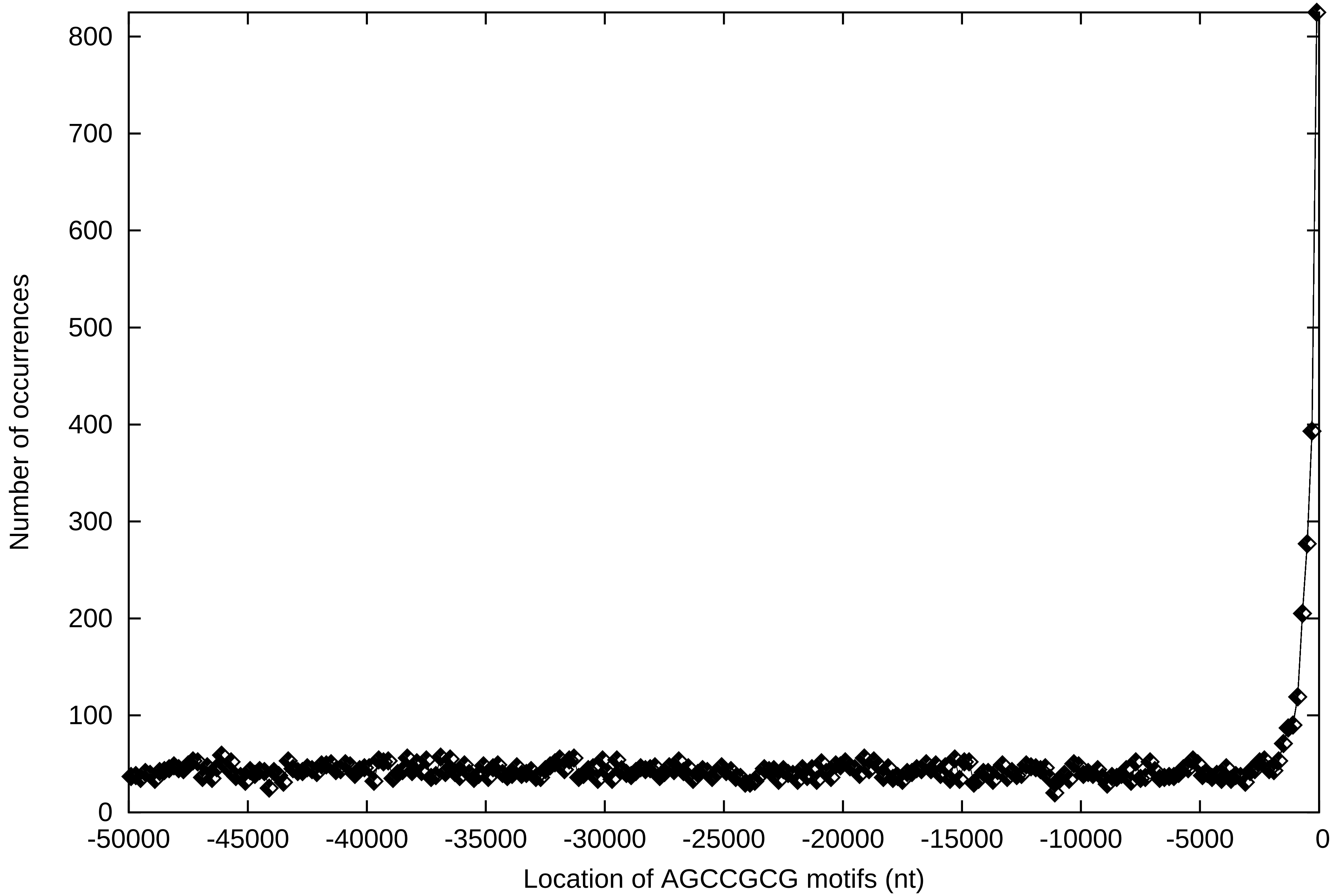

Number of occurrences
0
100
200
300
400
500
600
700
800
-50000
-45000
-40000
-35000
-30000
-25000
-20000
-15000
-10000
-5000
0
Location of AGCCGCG motifs (nt)

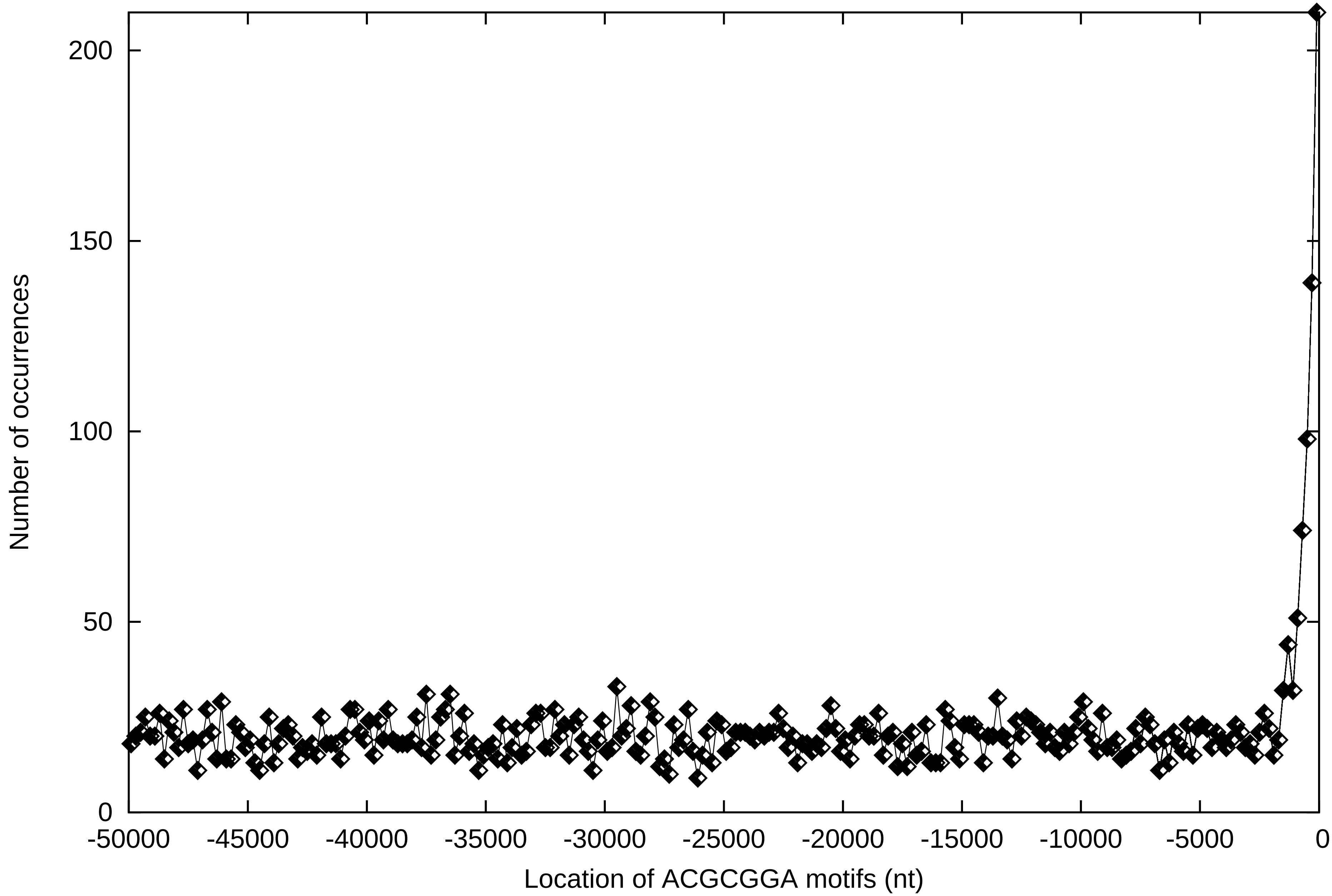

Number of occurrences
0
50
100
150
200
-50000
-45000
-40000
-35000
-30000
-25000
-20000
-15000
-10000
-5000
0
Location of ACGCGGA motifs (nt)

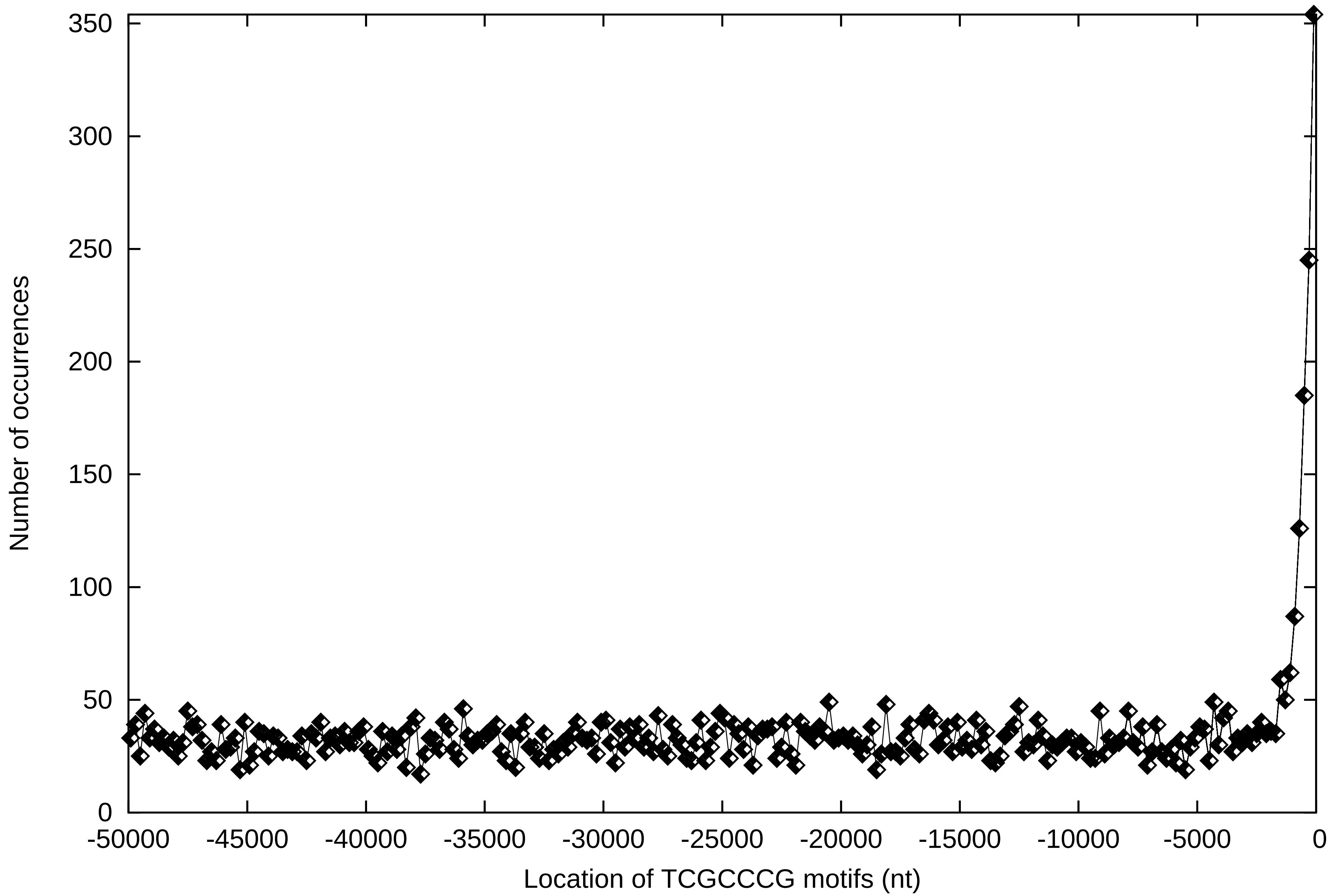
350
300
250
200
150
100
50
0
-50000
-45000
-40000
-35000
-30000
-25000
-20000
-15000
-10000
-5000
0
Number of occurrences
Location of TCGCCCG motifs (nt)

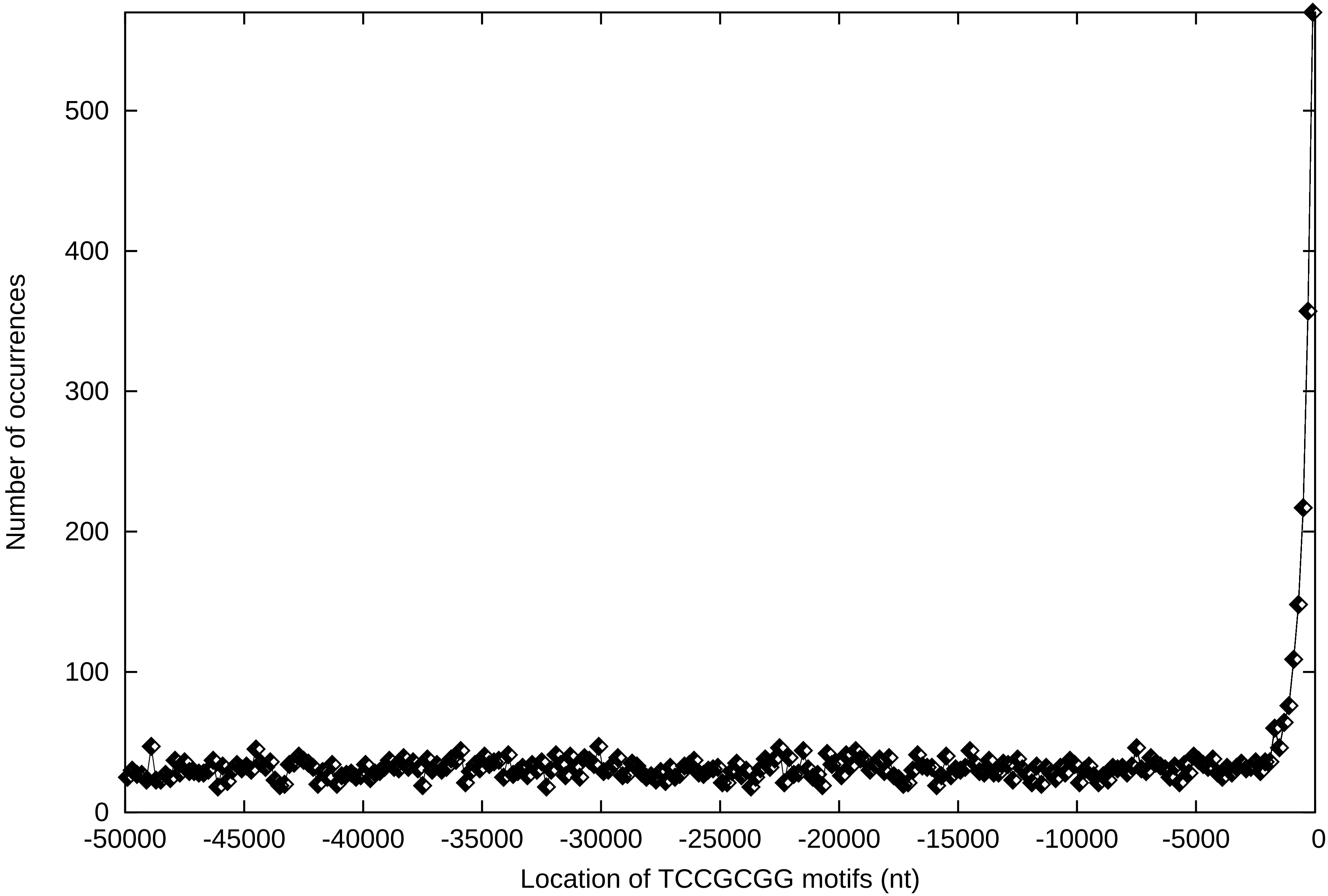

Number of occurrences
0
100
200
300
400
500
-50000
-45000
-40000
-35000
-30000
-25000
-20000
-15000
-10000
-5000
0
Location of TCCGCGG motifs (nt)

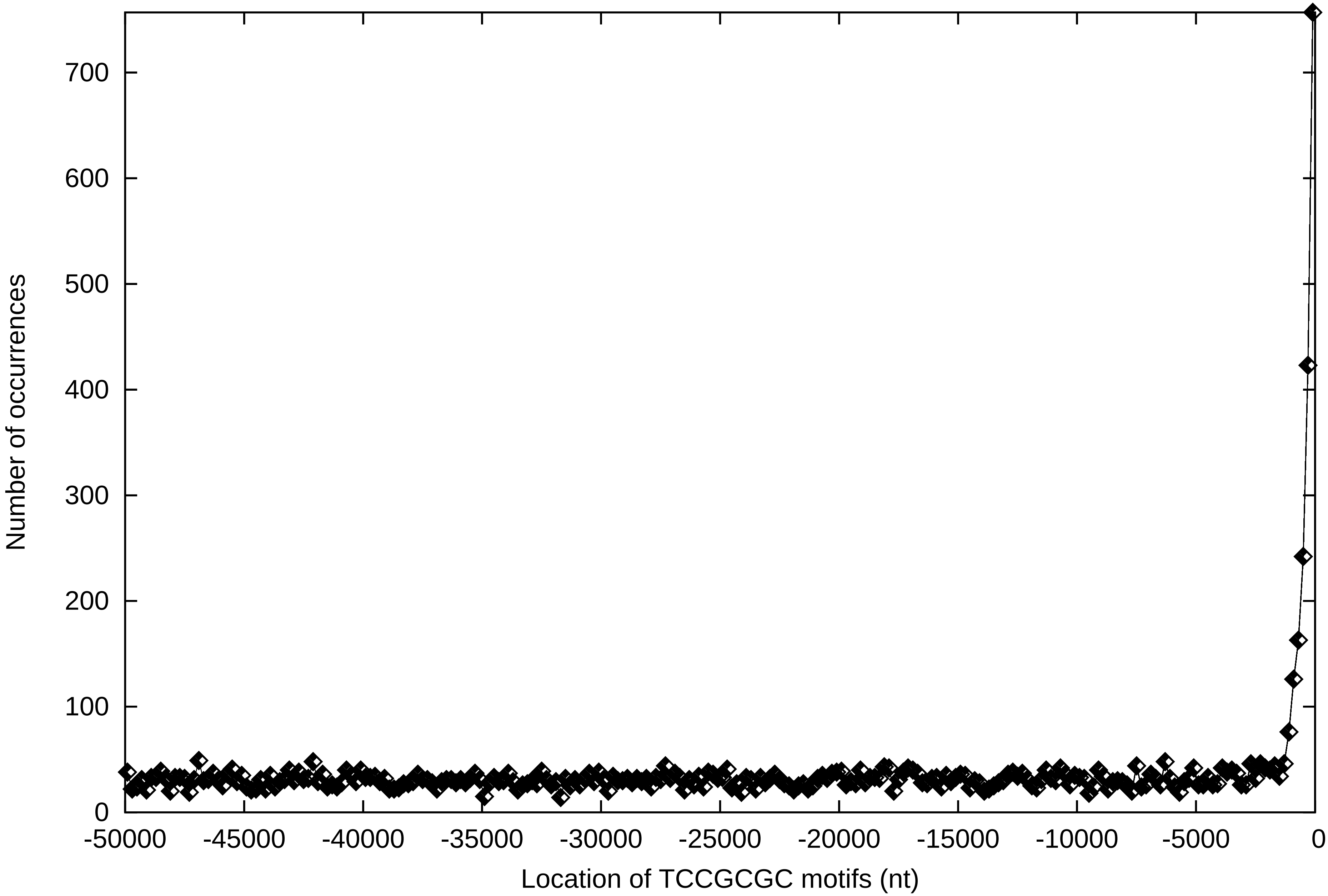
Number of occurrences
0
100
200
300
400
500
600
700
-50000
-45000
-40000
-35000
-30000
-25000
-20000
-15000
-10000
-5000
0
Location of TCCGCGC motifs (nt)

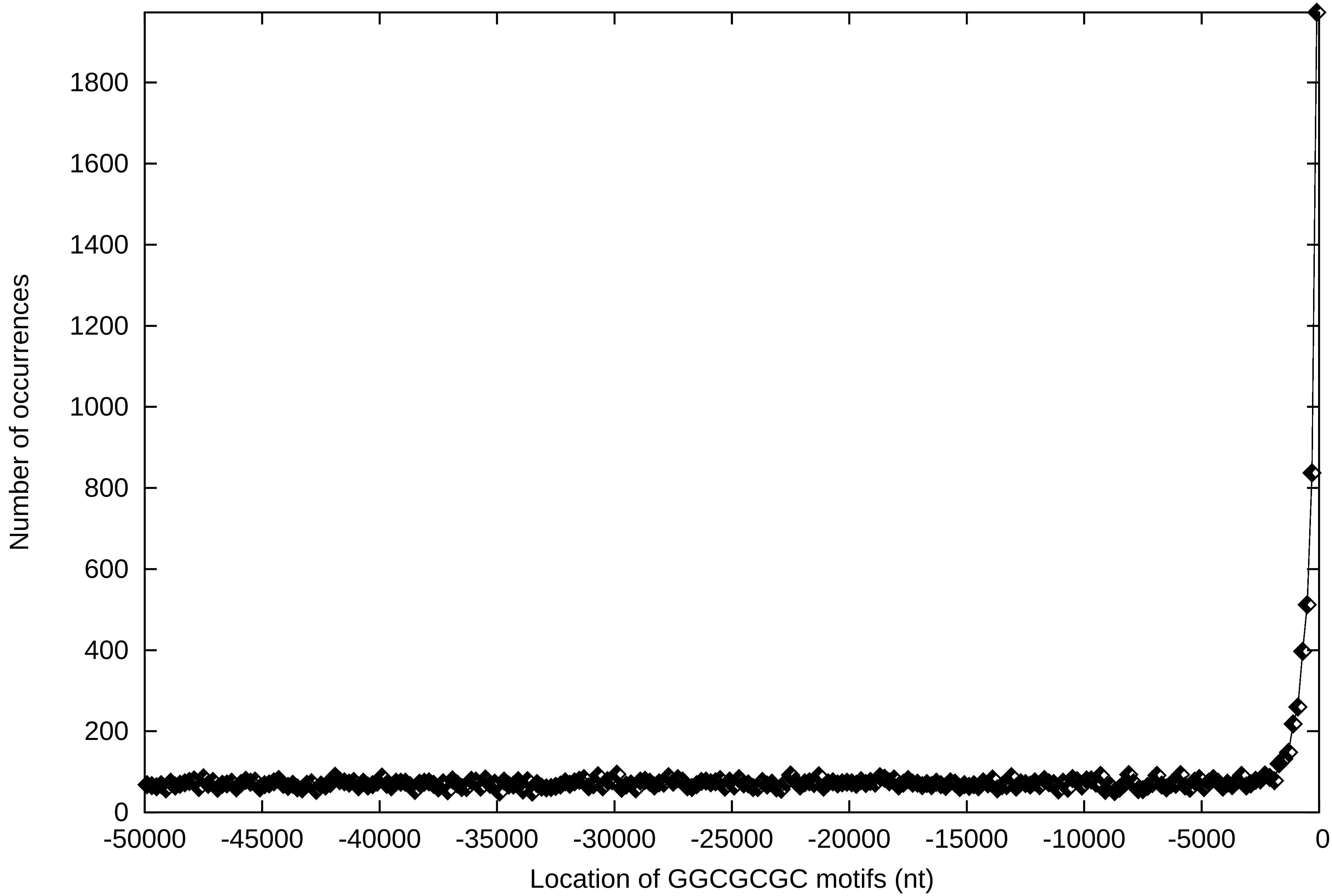
Number of occurrences
0
200
400
600
800
1000
1200
1400
1600
1800
-50000
-45000
-40000
-35000
-30000
-25000
-20000
-15000
-10000
-5000
0
Location of GGCGCGC motifs (nt)

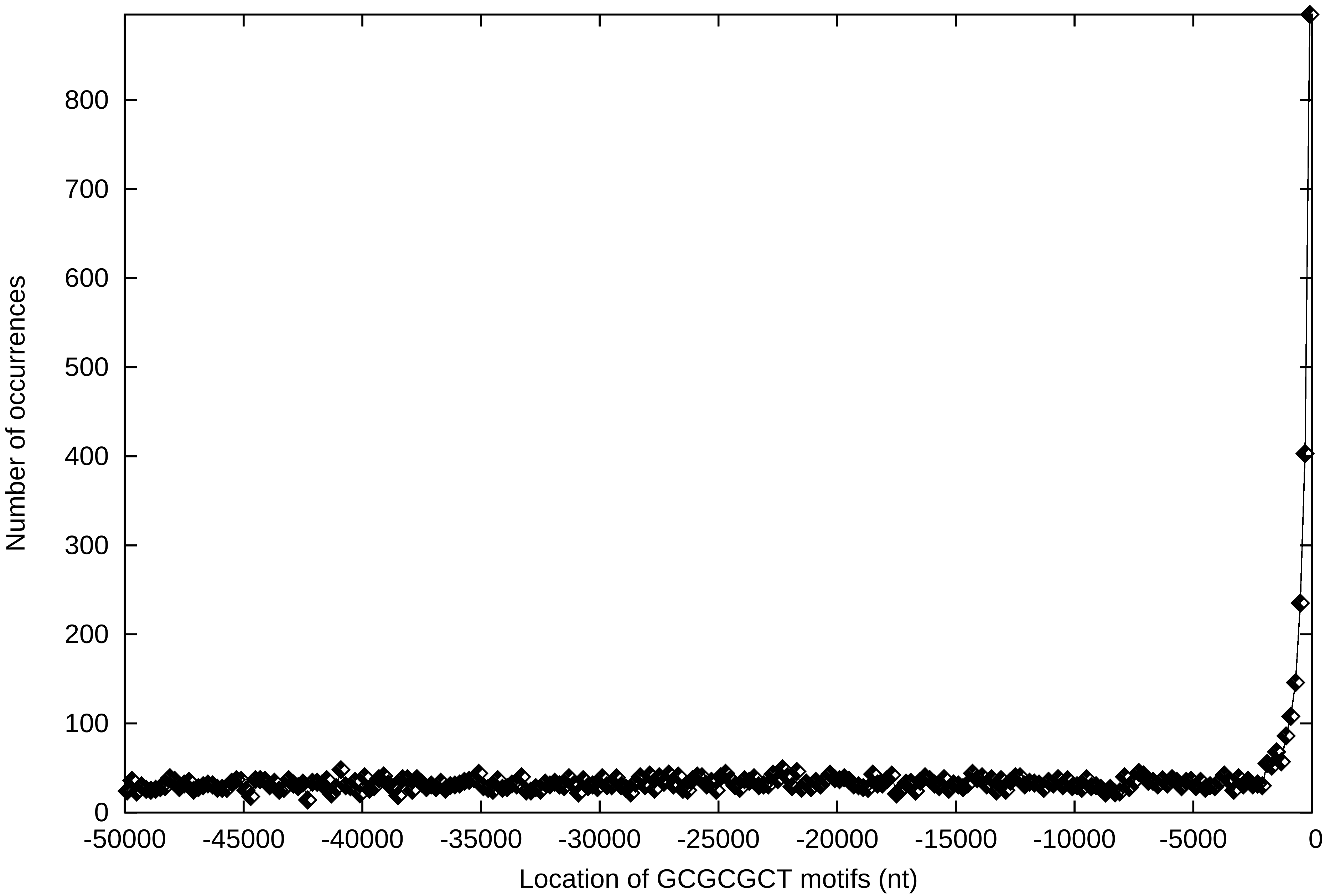

Number of occurrences
800
700
600
500
400
300
200
100
0
-50000
-45000
-40000
-35000
-30000
-25000
-20000
-15000
-10000
-5000
0
Location of GCGCGCT motifs (nt)

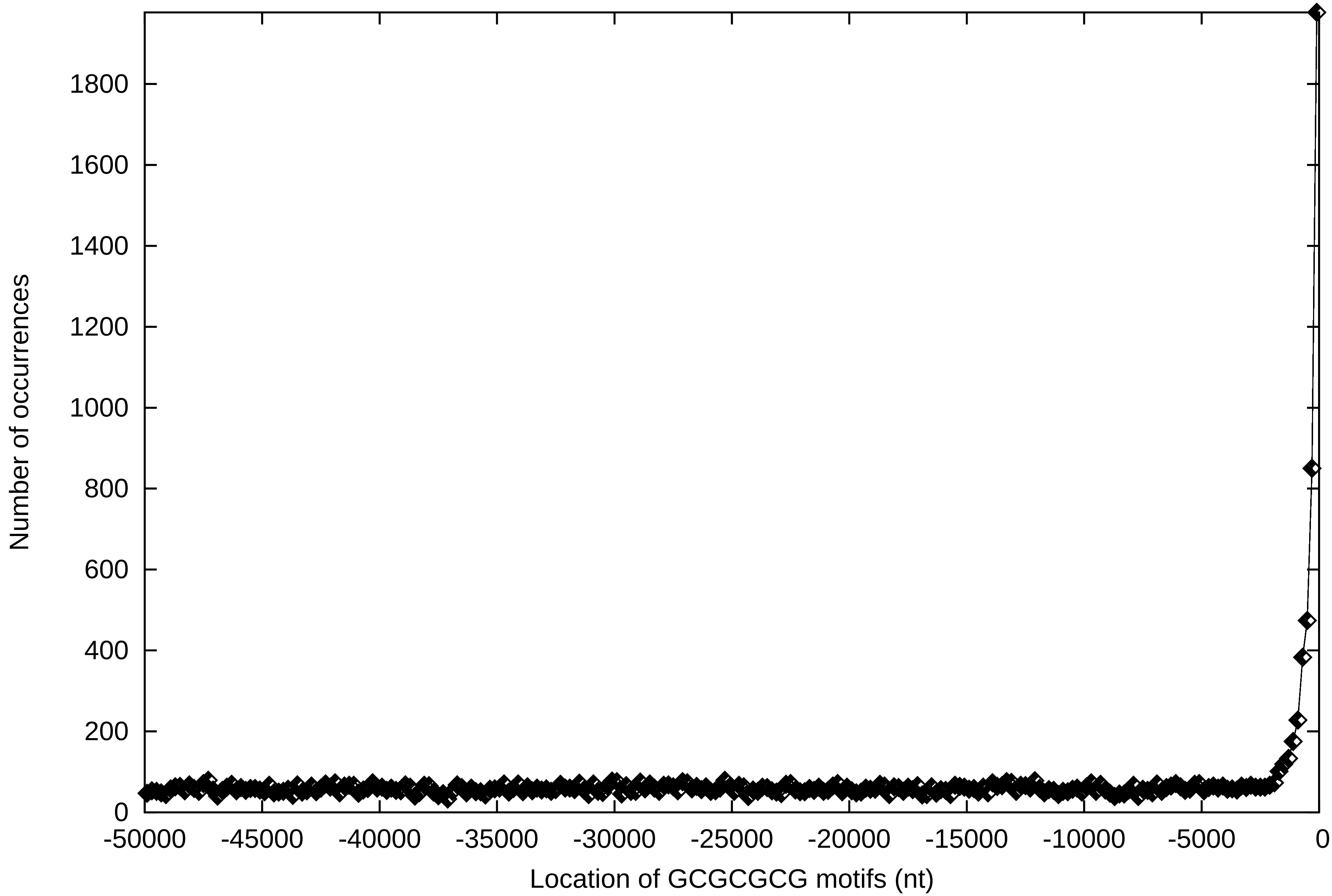

1800
1600
1400
1200
1000
800
600
400
200
0
-50000
-45000
-40000
-35000
-30000
-25000
-20000
-15000
-10000
-5000
0
Number of occurrences
Location of GCGCGCG motifs (nt)

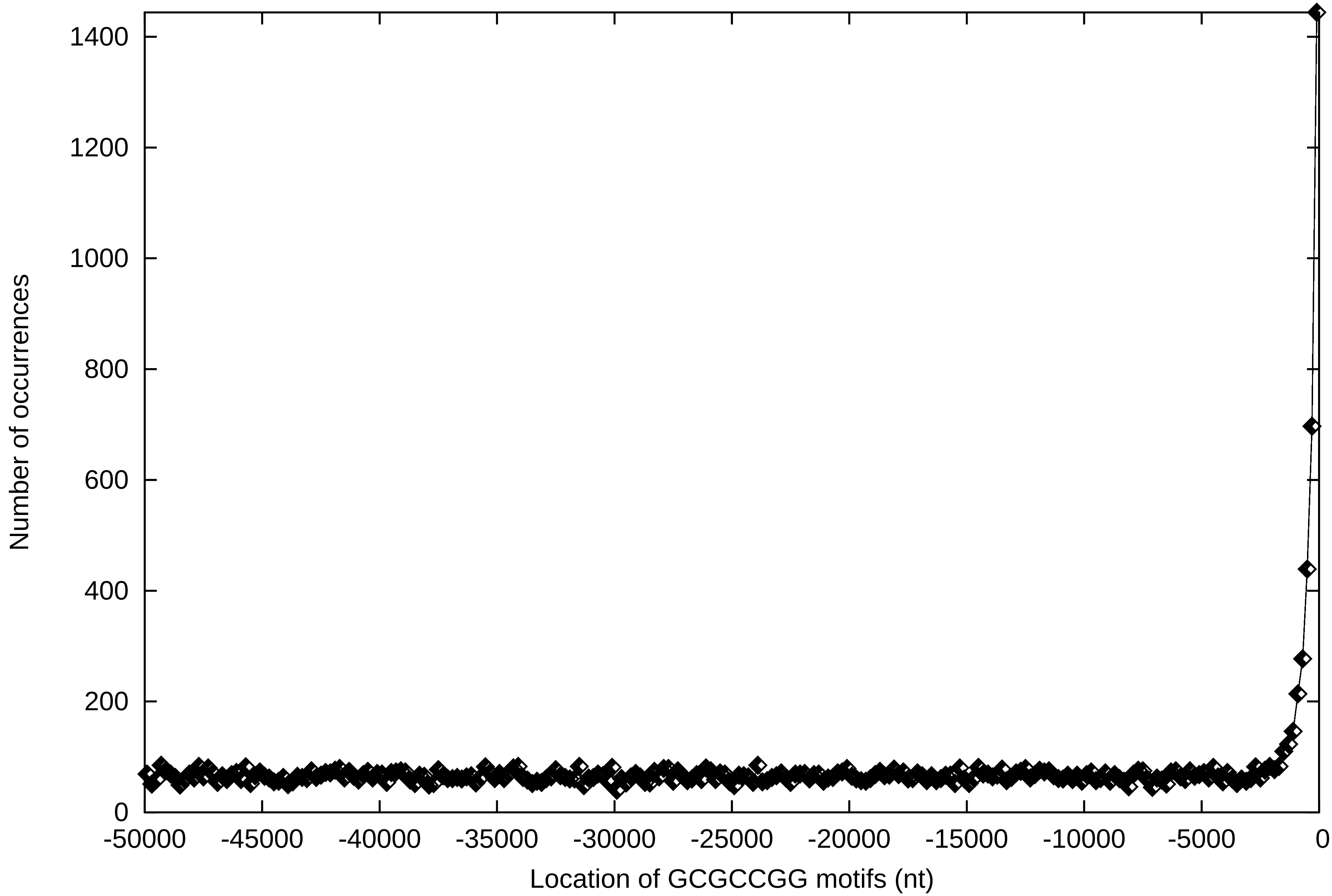
Number of occurrences
0
200
400
600
800
1000
1200
1400
-50000
-45000
-40000
-35000
-30000
-25000
-20000
-15000
-10000
-5000
0
Location of GCGCCGG motifs (nt)

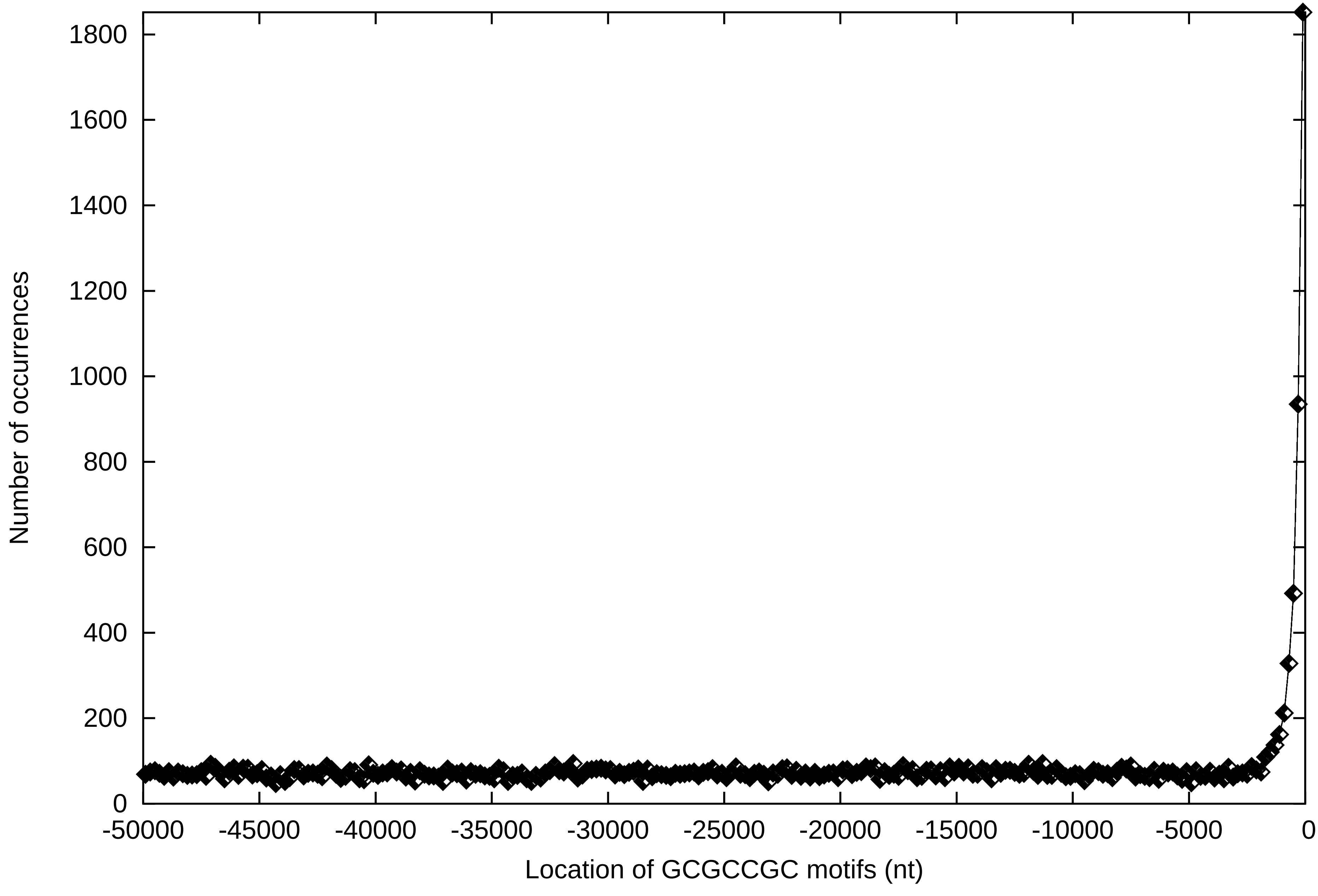

Number of occurrences
0
200
400
600
800
1000
1200
1400
1600
1800
-50000
-45000
-40000
-35000
-30000
-25000
-20000
-15000
-10000
-5000
0
Location of GCGCCGC motifs (nt)

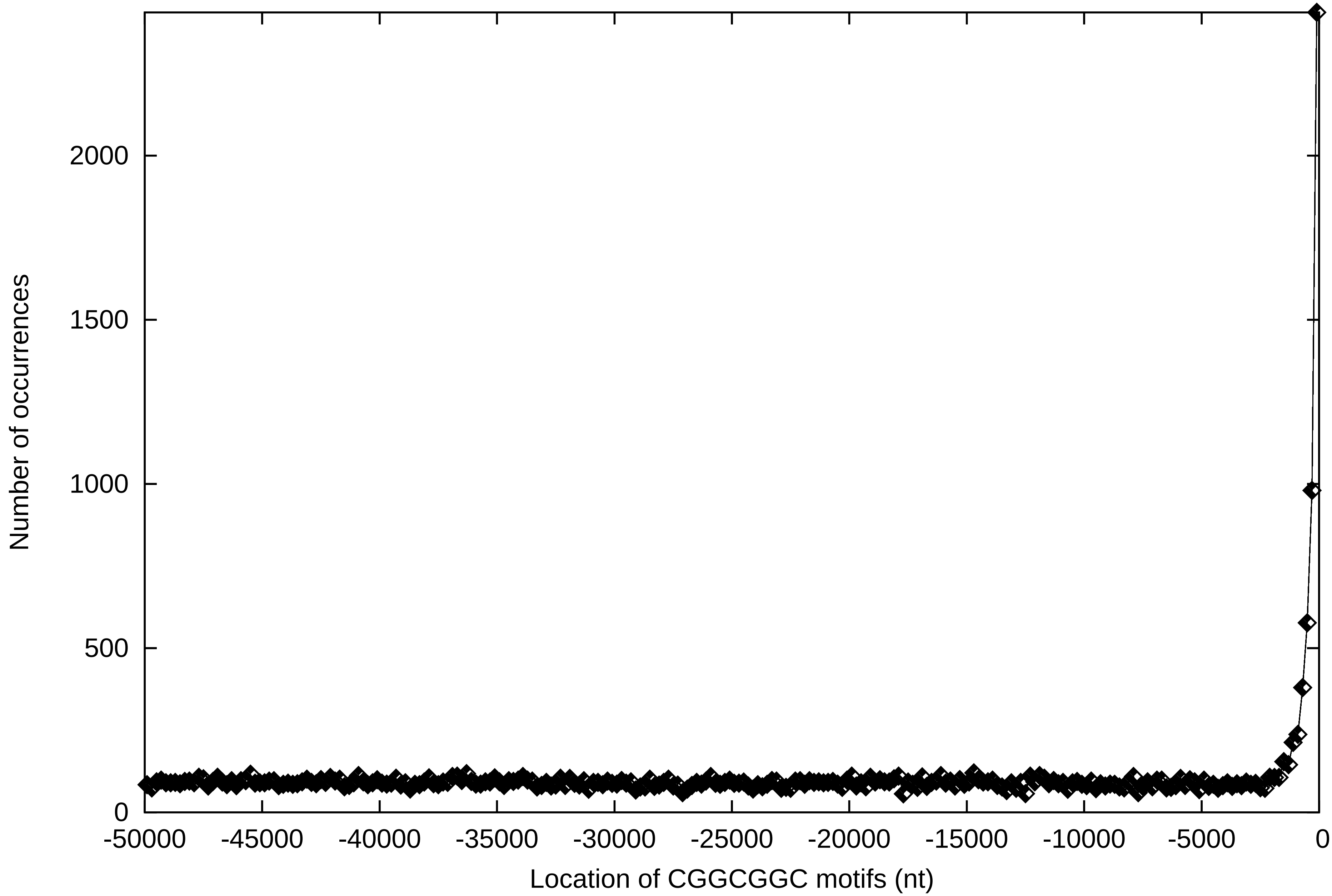

Number of occurrences
2000
1500
1000
500
0
-50000
-45000
-40000
-35000
-30000
-25000
-20000
-15000
-10000
-5000
0
Location of CGGCGGC motifs (nt)

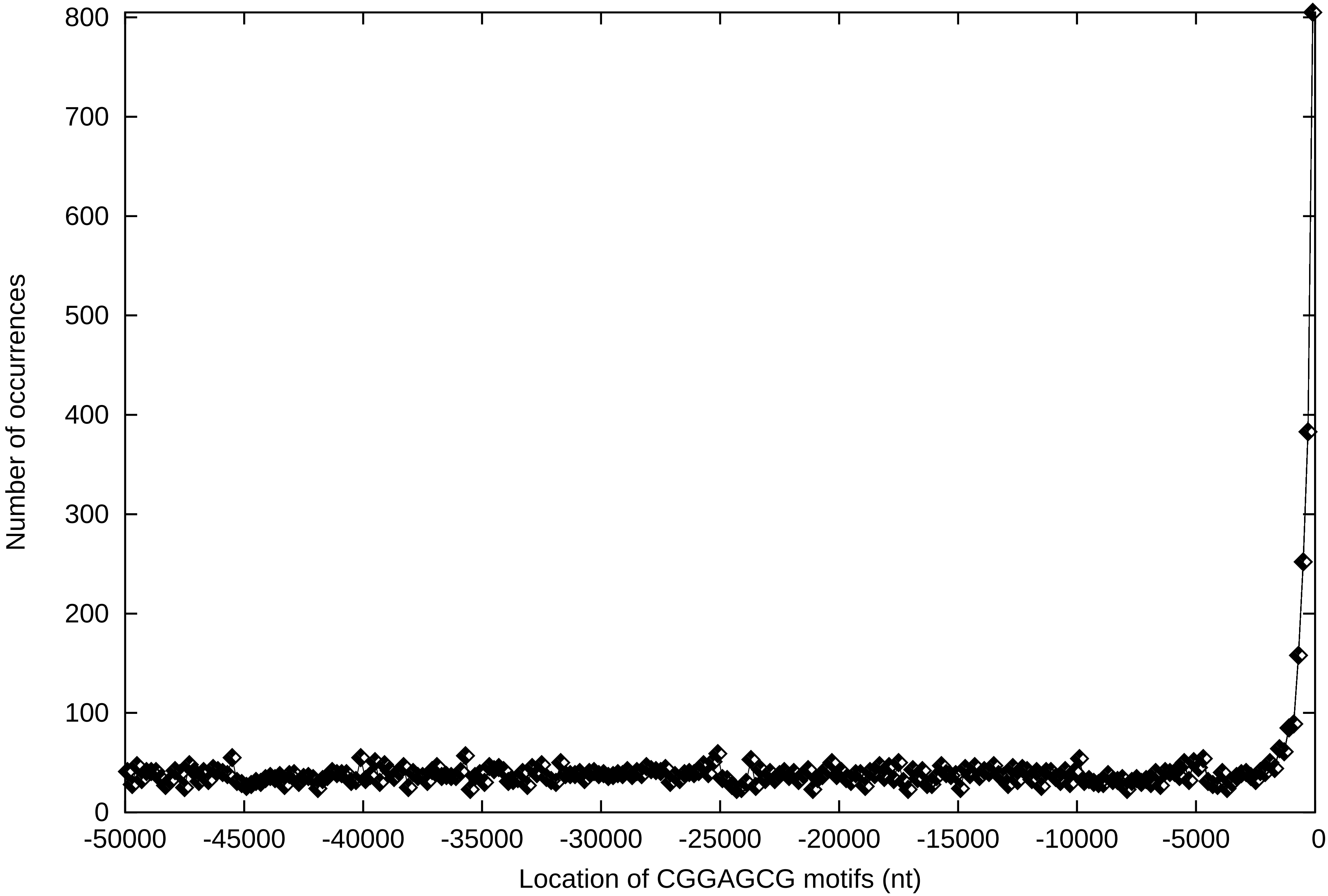

Number of occurrences
800
700
600
500
400
300
200
100
0
-50000
-45000
-40000
-35000
-30000
-25000
-20000
-15000
-10000
-5000
0
Location of CGGAGCG motifs (nt)

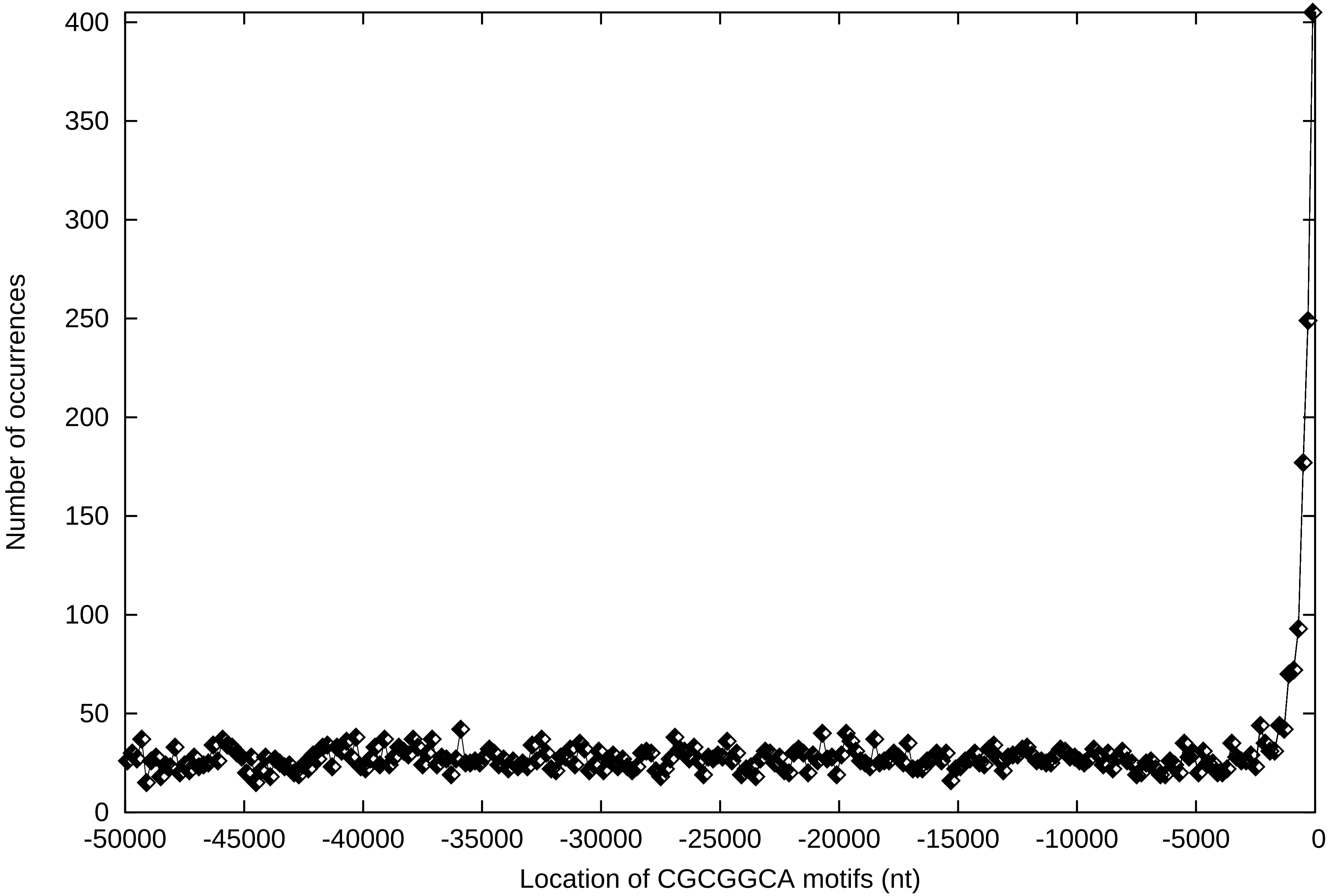
400
350
300
250
200
150
100
50
0
-50000
-45000
-40000
-35000
-30000
-25000
-20000
-15000
-10000
-5000
0
Number of occurrences
Location of CGCGGCA motifs (nt)

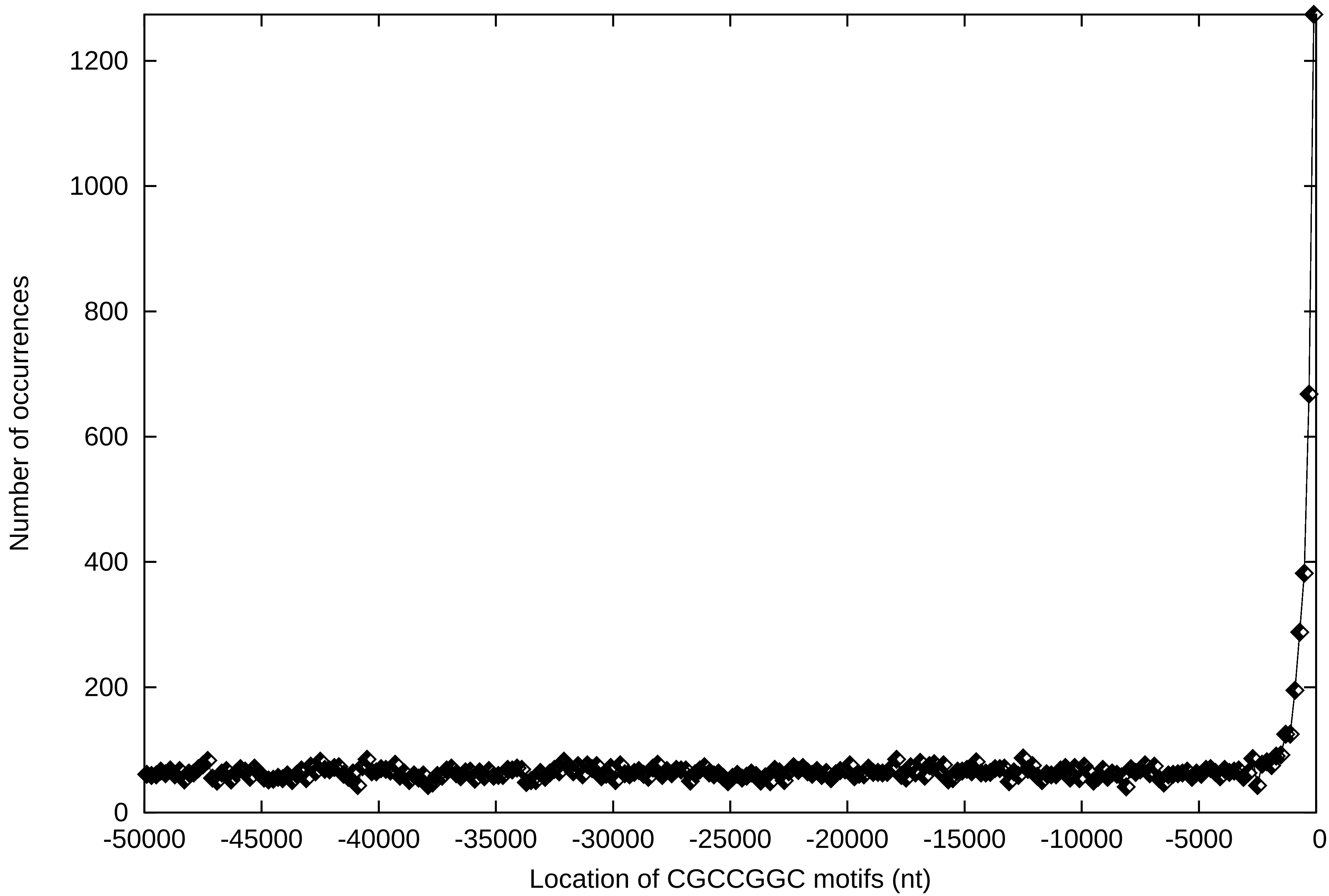
Number of occurrences
0
200
400
600
800
1000
1200
-50000
-45000
-40000
-35000
-30000
-25000
-20000
-15000
-10000
-5000
0
Location of CGCCGGC motifs (nt)

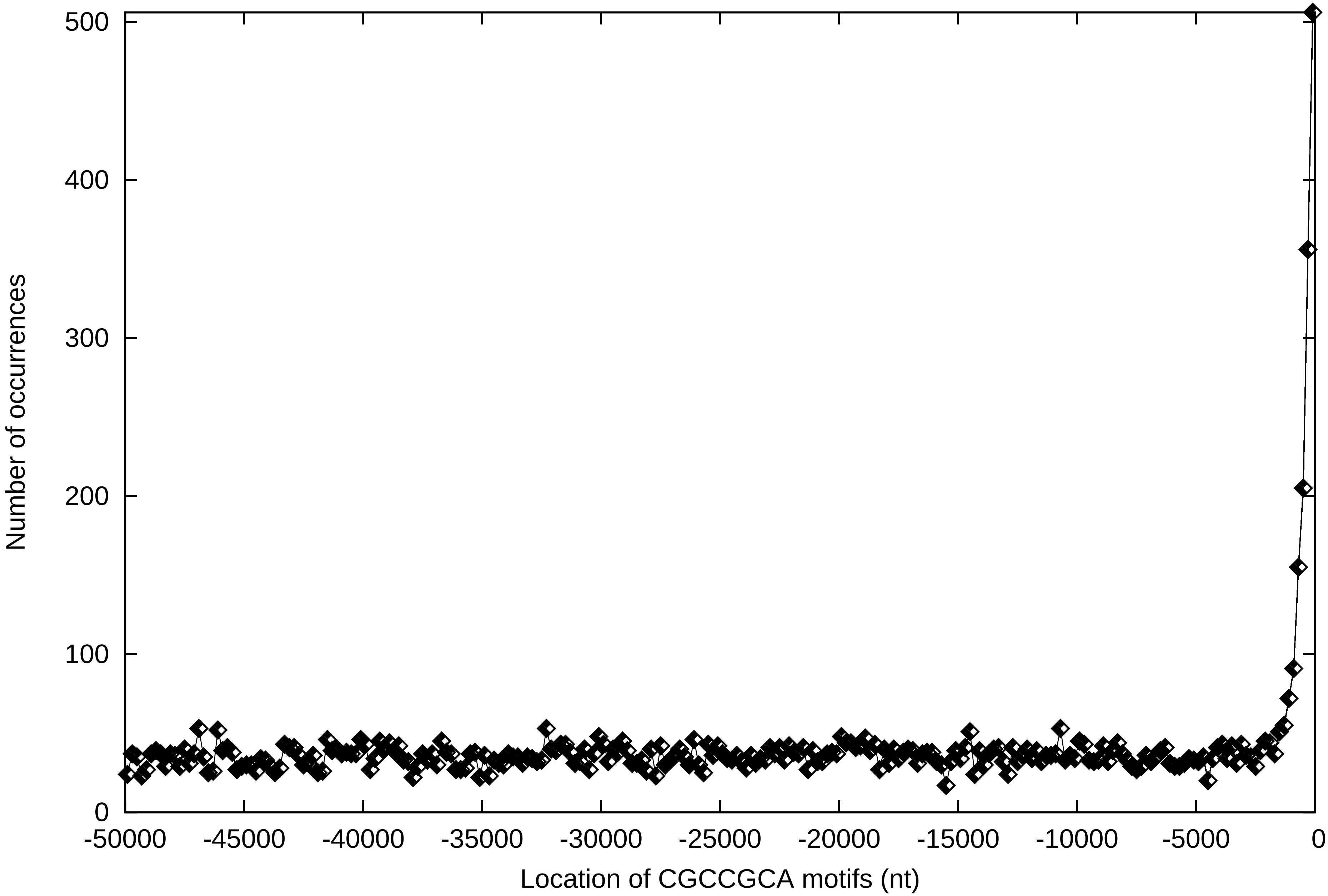

Number of occurrences
0
100
200
300
400
500
-50000
-45000
-40000
-35000
-30000
-25000
-20000
-15000
-10000
-5000
0
Location of CGCCGCA motifs (nt)

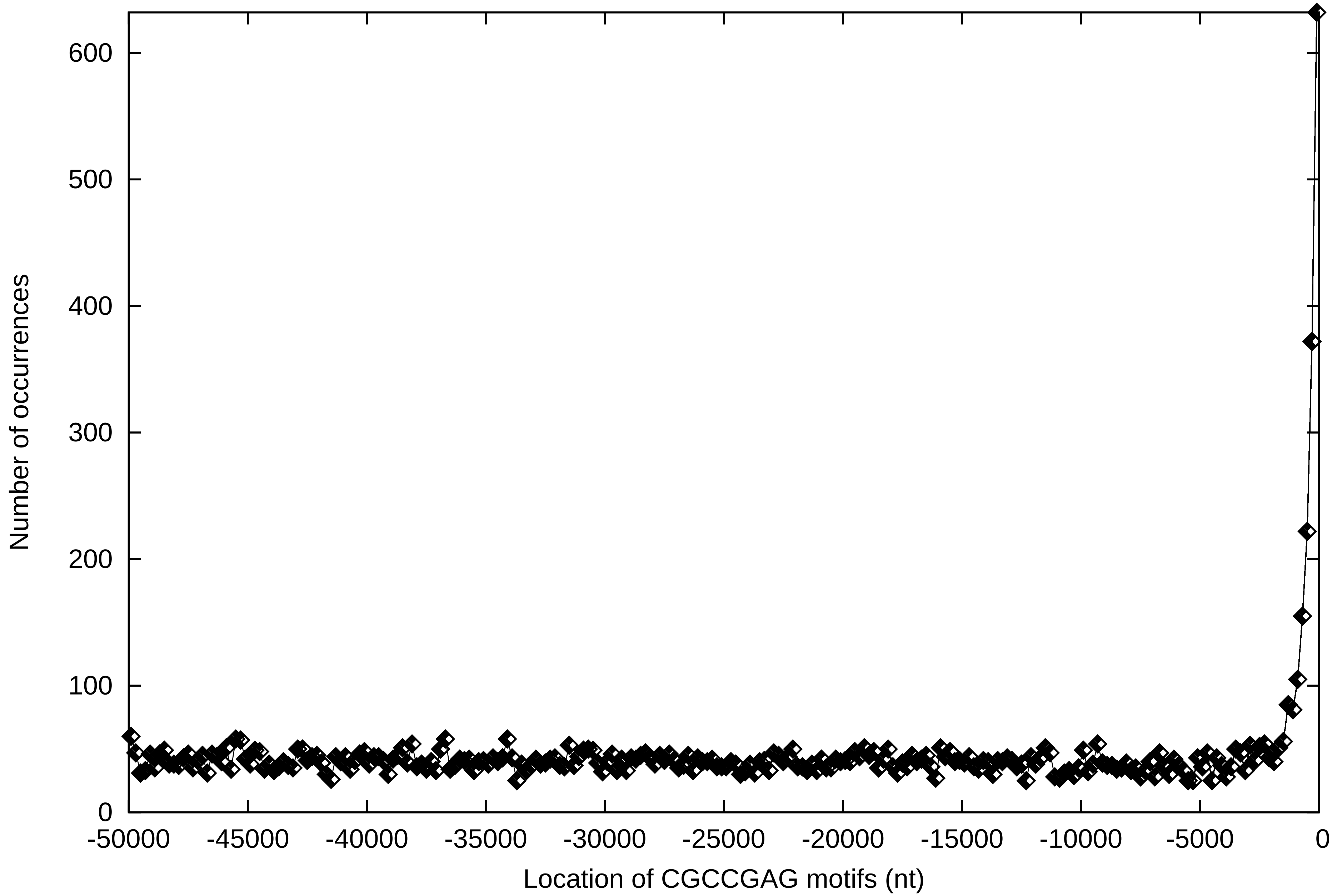
Number of occurrences
Location of CGCCGAG motifs (nt)
0
100
200
300
400
500
600
-50000
-45000
-40000
-35000
-30000
-25000
-20000
-15000
-10000
-5000
0

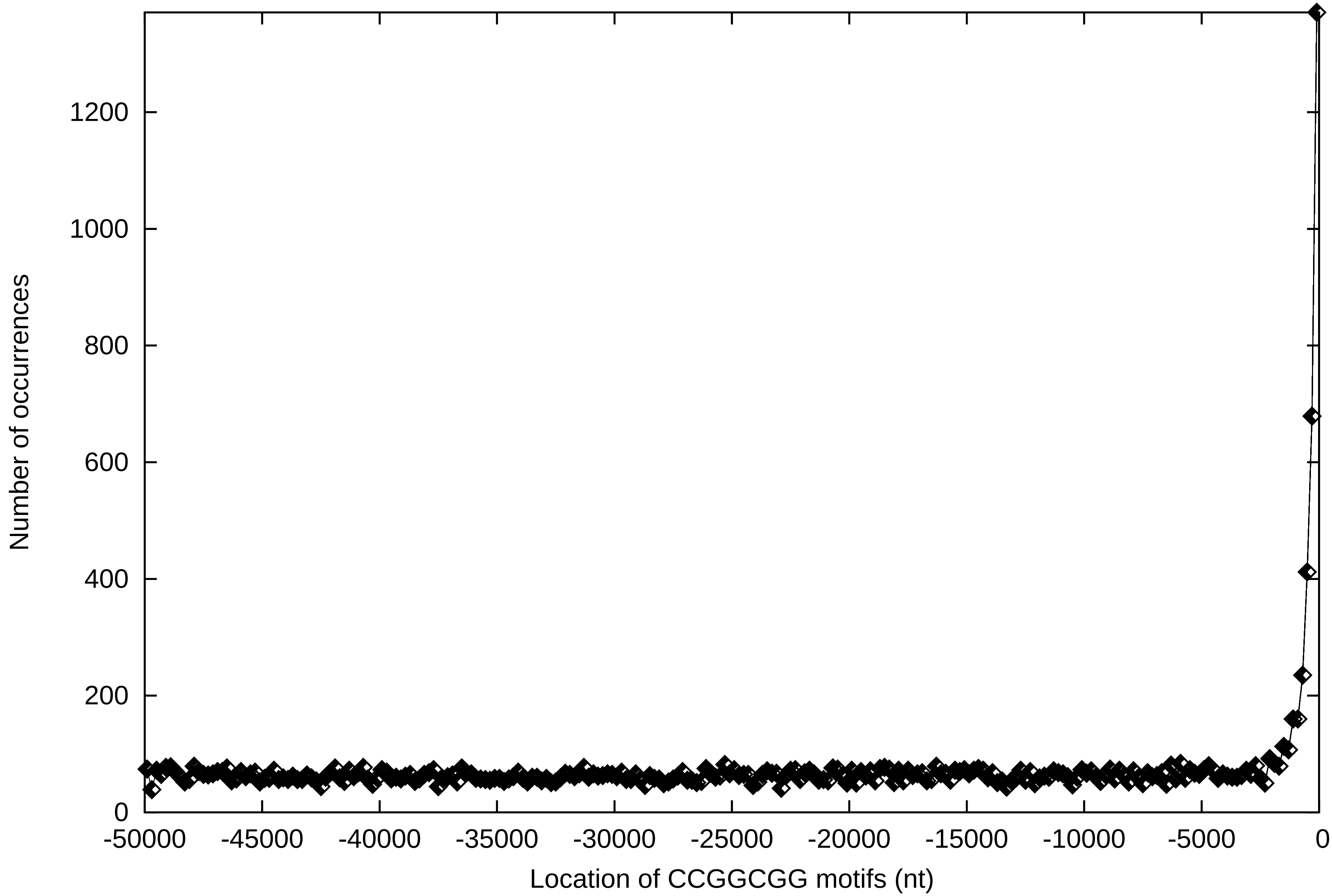

Number of occurrences
0
200
400
600
800
1000
1200
-50000
-45000
-40000
-35000
-30000
-25000
-20000
-15000
-10000
-5000
0
Location of CCGGCGG motifs (nt)

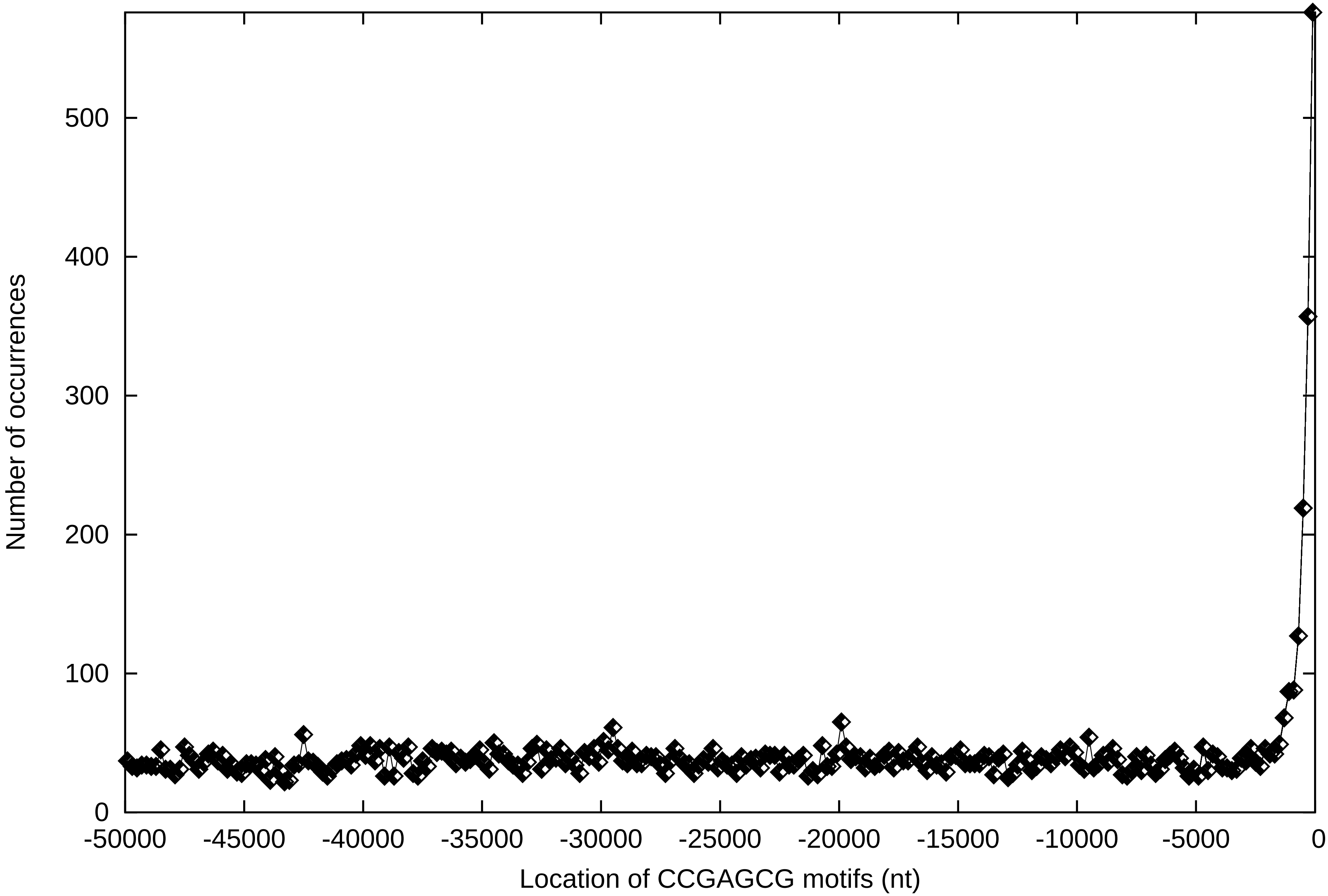

Number of occurrences
0
100
200
300
400
500
-50000
-45000
-40000
-35000
-30000
-25000
-20000
-15000
-10000
-5000
0
Location of CCGAGCG motifs (nt)

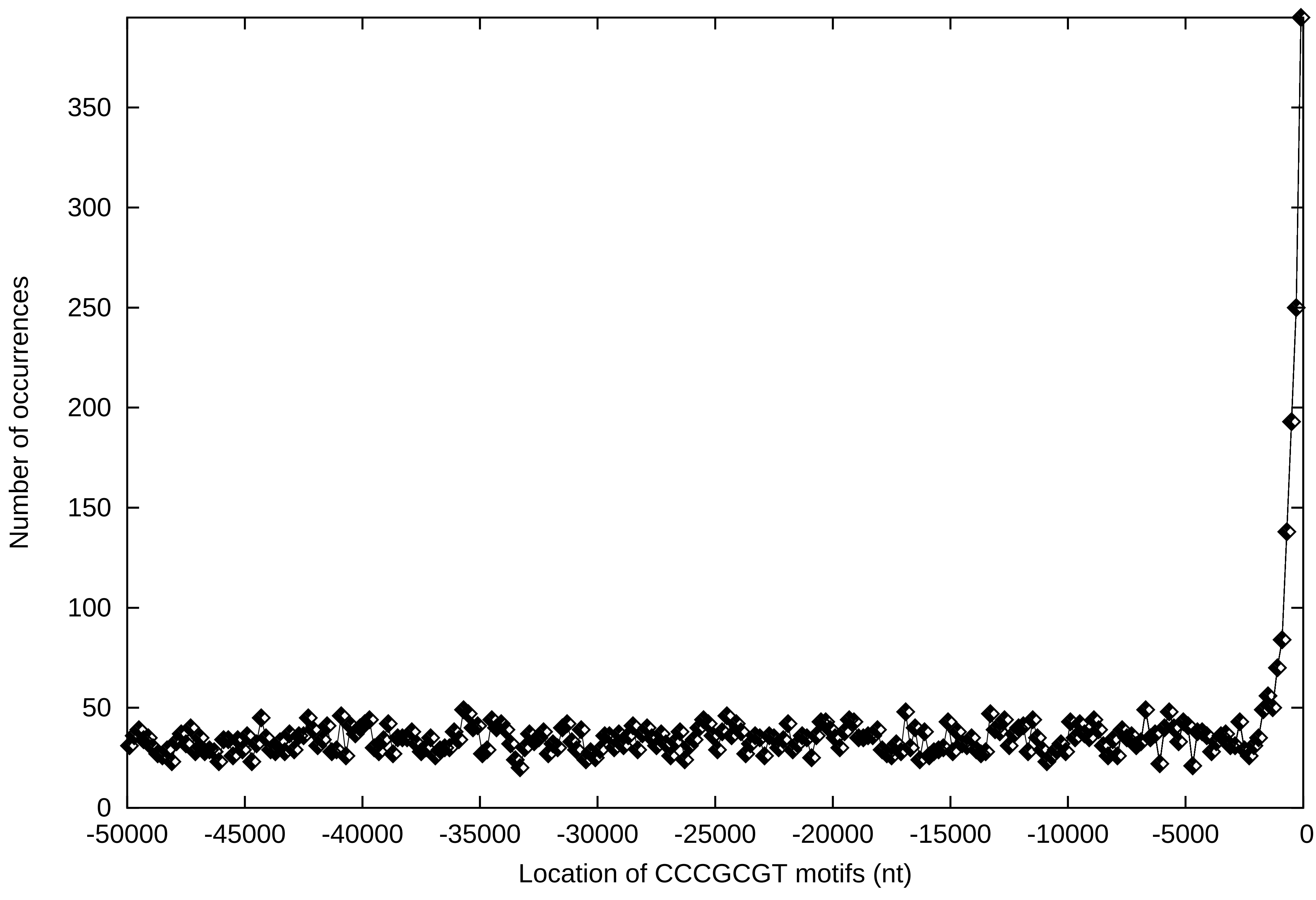

Number of occurrences
350
300
250
200
150
100
50
0
-50000
-45000
-40000
-35000
-30000
-25000
-20000
-15000
-10000
-5000
0
Location of CCCGCGT motifs (nt)

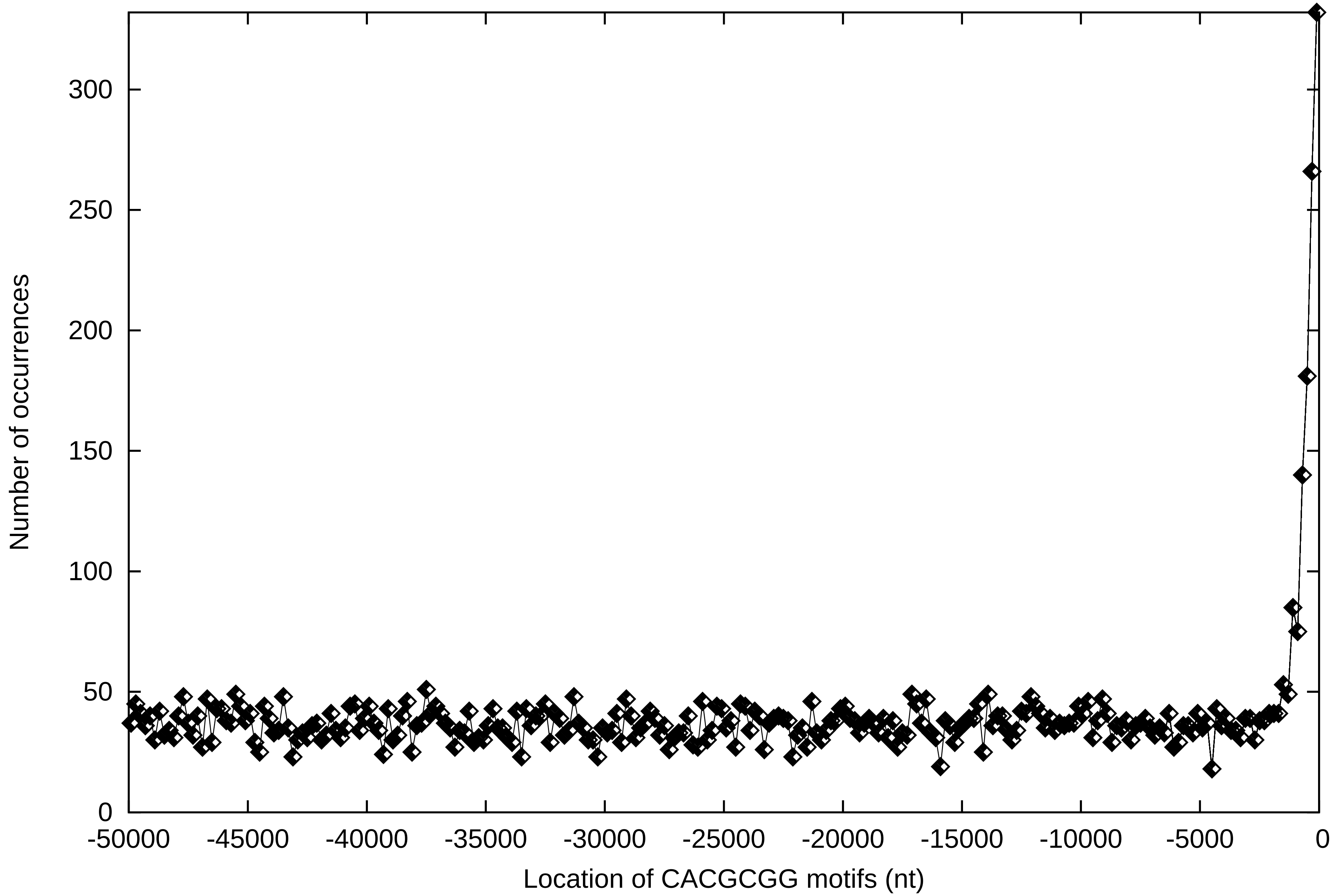

Number of occurrences
Location of CACGCGG motifs (nt)
0
50
100
150
200
250
300
-50000
-45000
-40000
-35000
-30000
-25000
-20000
-15000
-10000
-5000
0

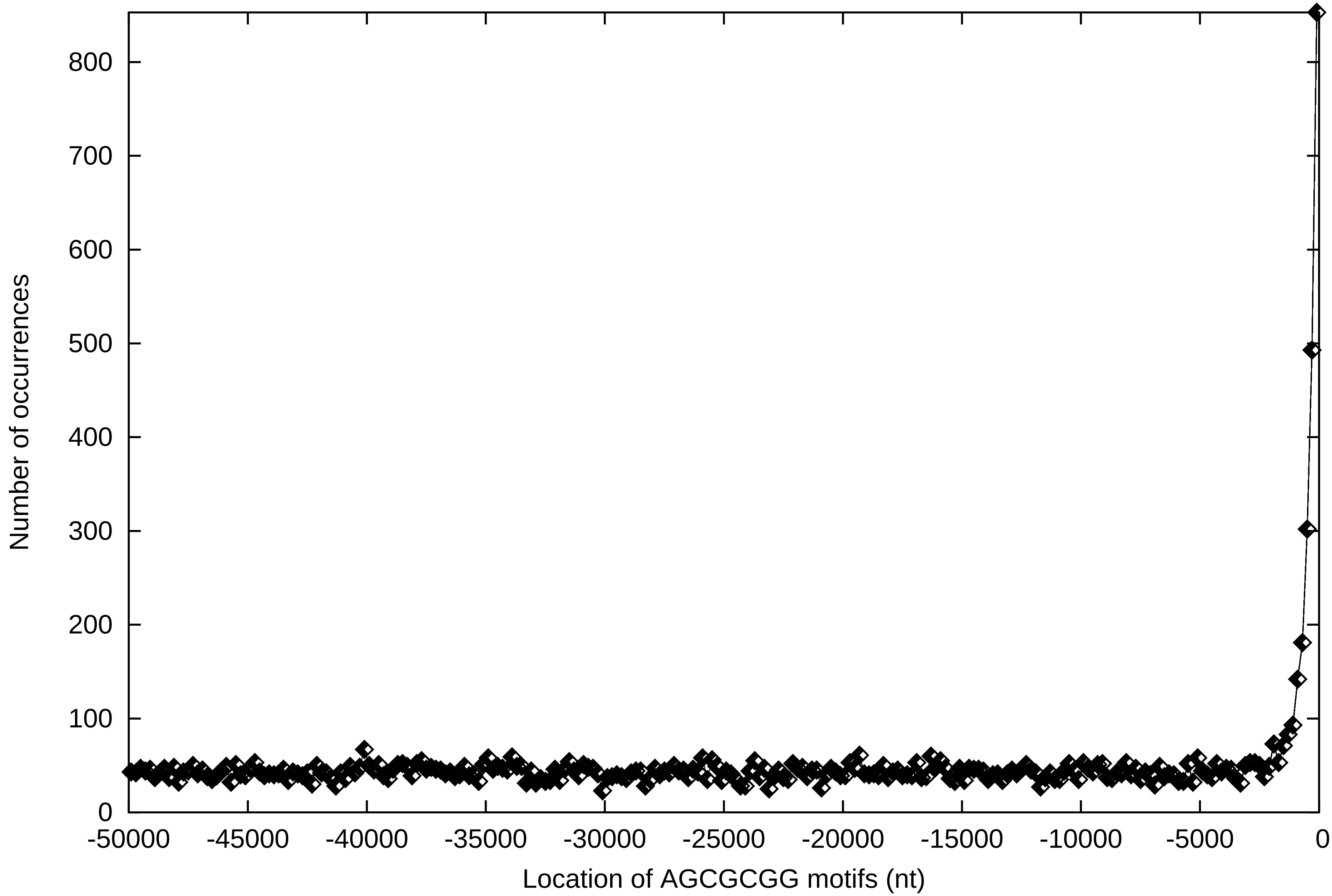

0
100
200
300
400
500
600
700
800
Number of occurrences
-50000
-45000
-40000
-35000
-30000
-25000
-20000
-15000
-10000
-5000
0
Location of AGCGCGG motifs (nt)

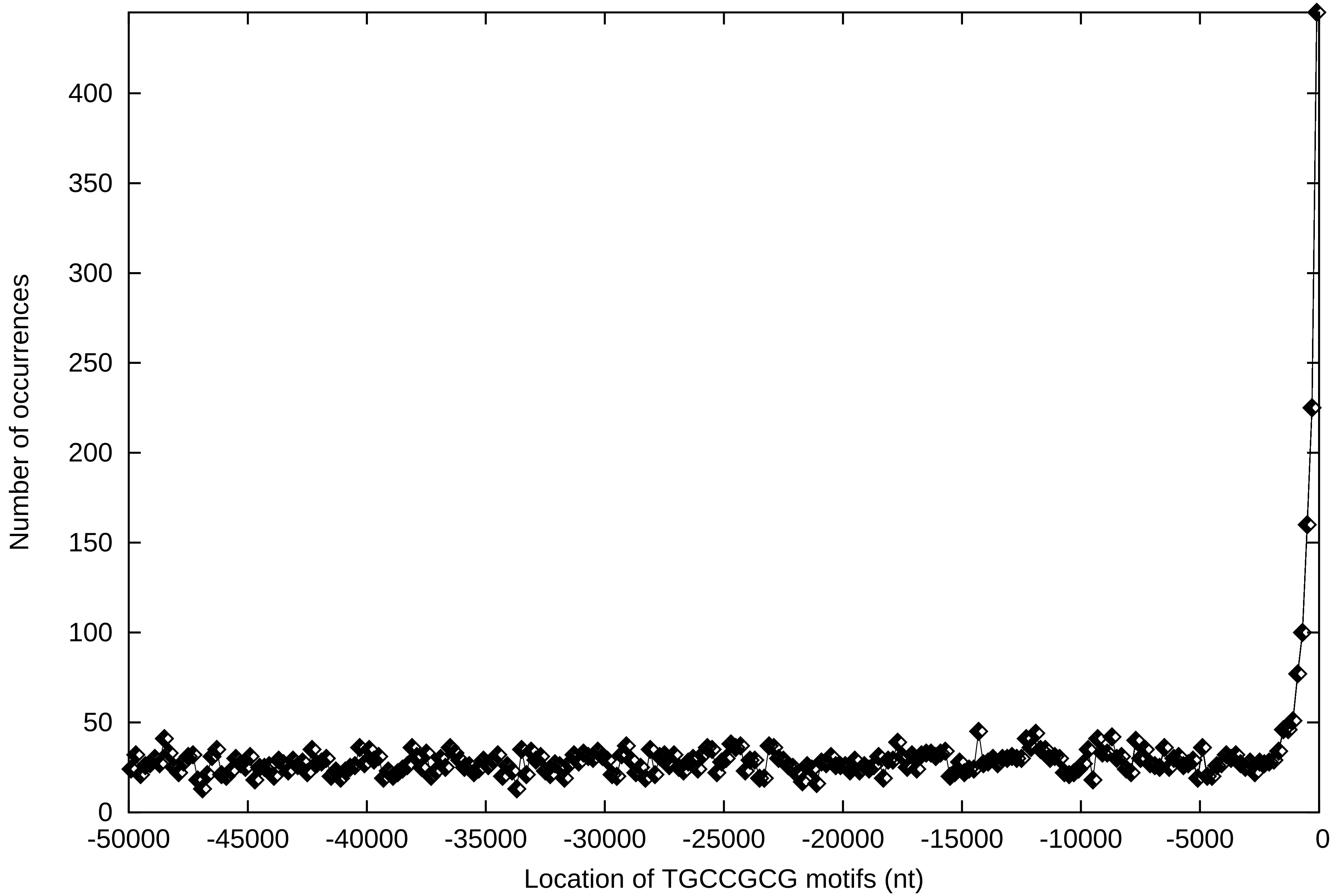
Number of occurrences
400
350
300
250
200
150
100
50
0
-50000
-45000
-40000
-35000
-30000
-25000
-20000
-15000
-10000
-5000
0
Location of TGCCGCG motifs (nt)

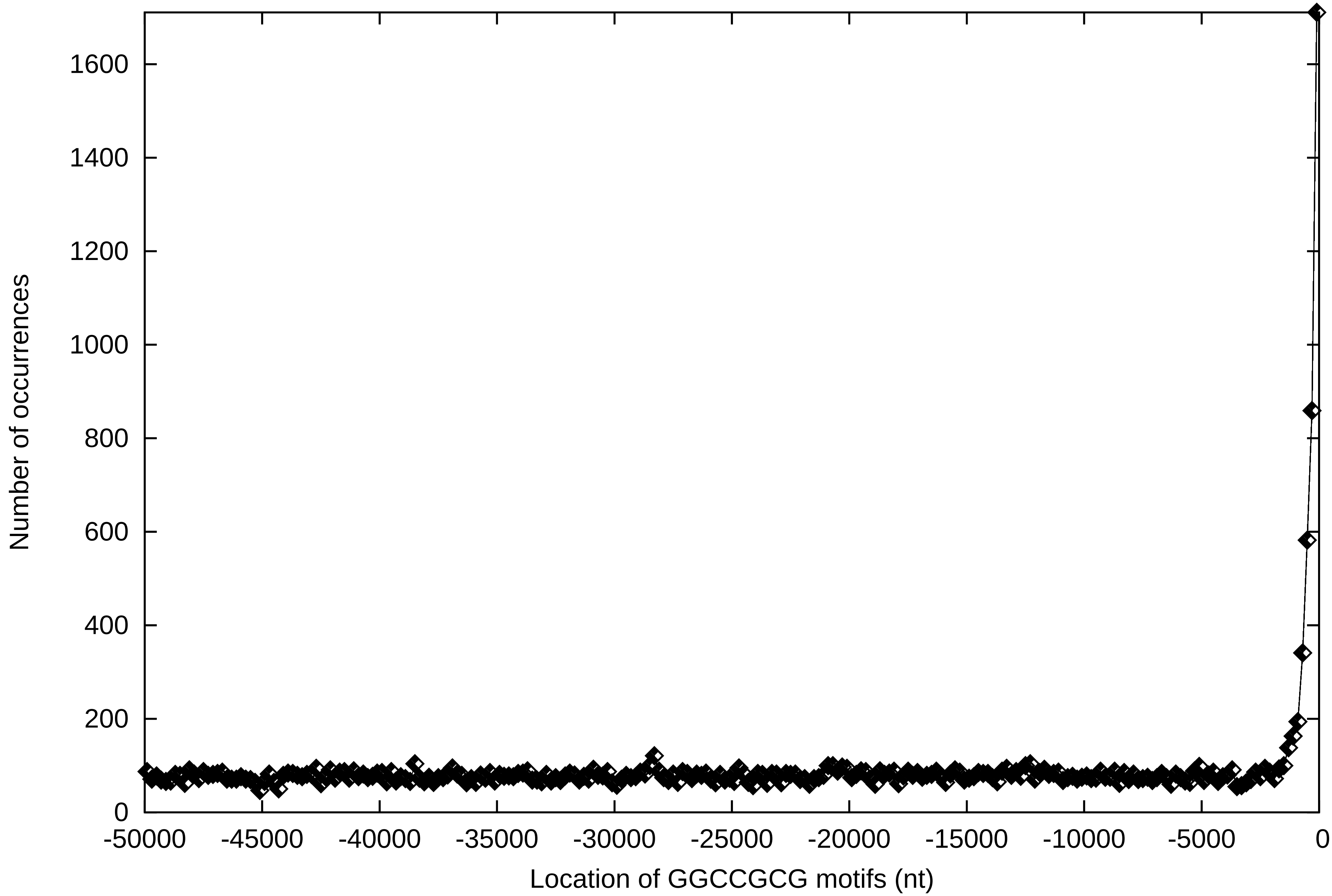

Number of occurrences
0
200
400
600
800
1000
1200
1400
1600
-50000
-45000
-40000
-35000
-30000
-25000
-20000
-15000
-10000
-5000
0
Location of GGCCGCG motifs (nt)

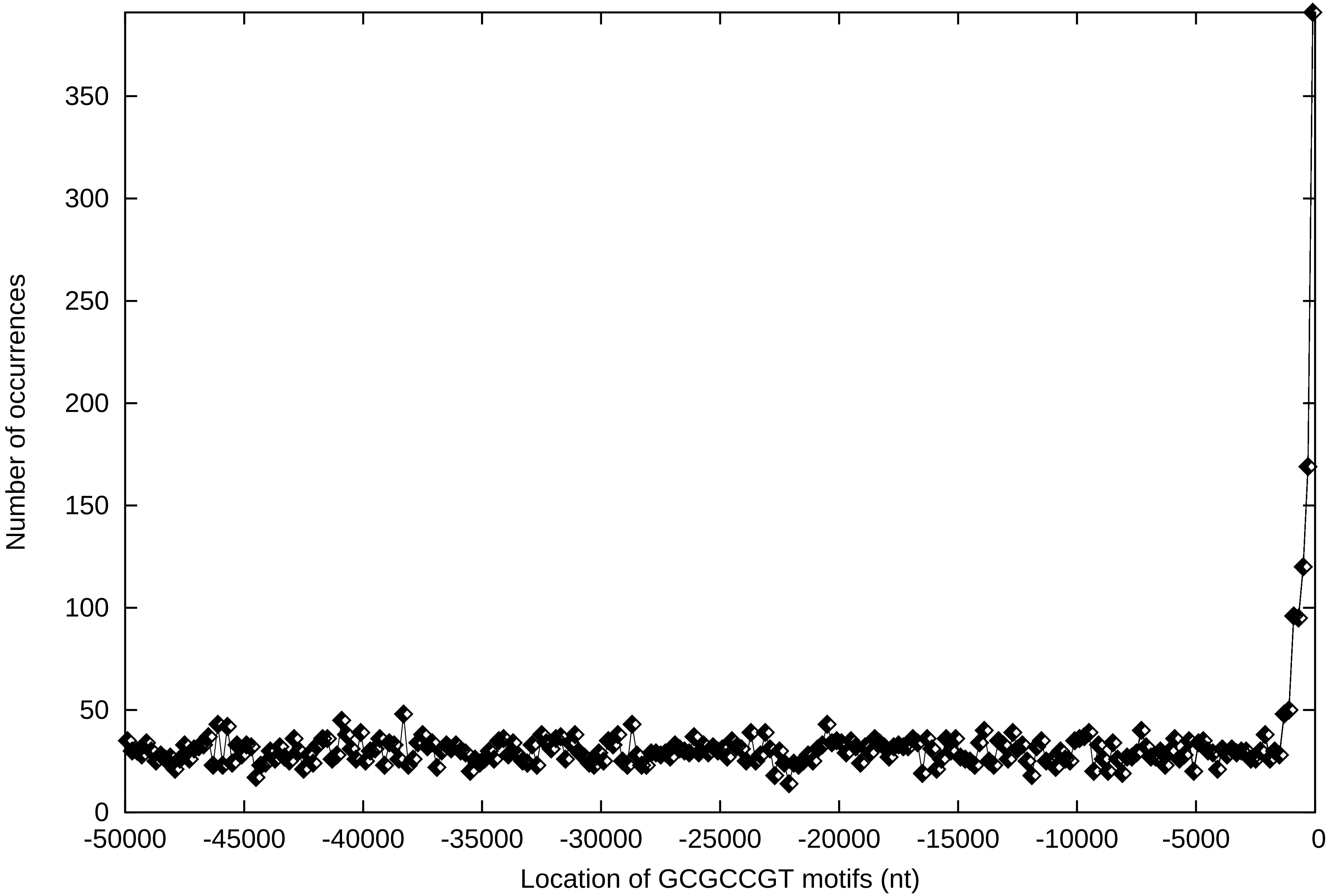

Number of occurrences
Location of GCGCCGT motifs (nt)
0
50
100
150
200
250
300
350
-50000
-45000
-40000
-35000
-30000
-25000
-20000
-15000
-10000
-5000
0

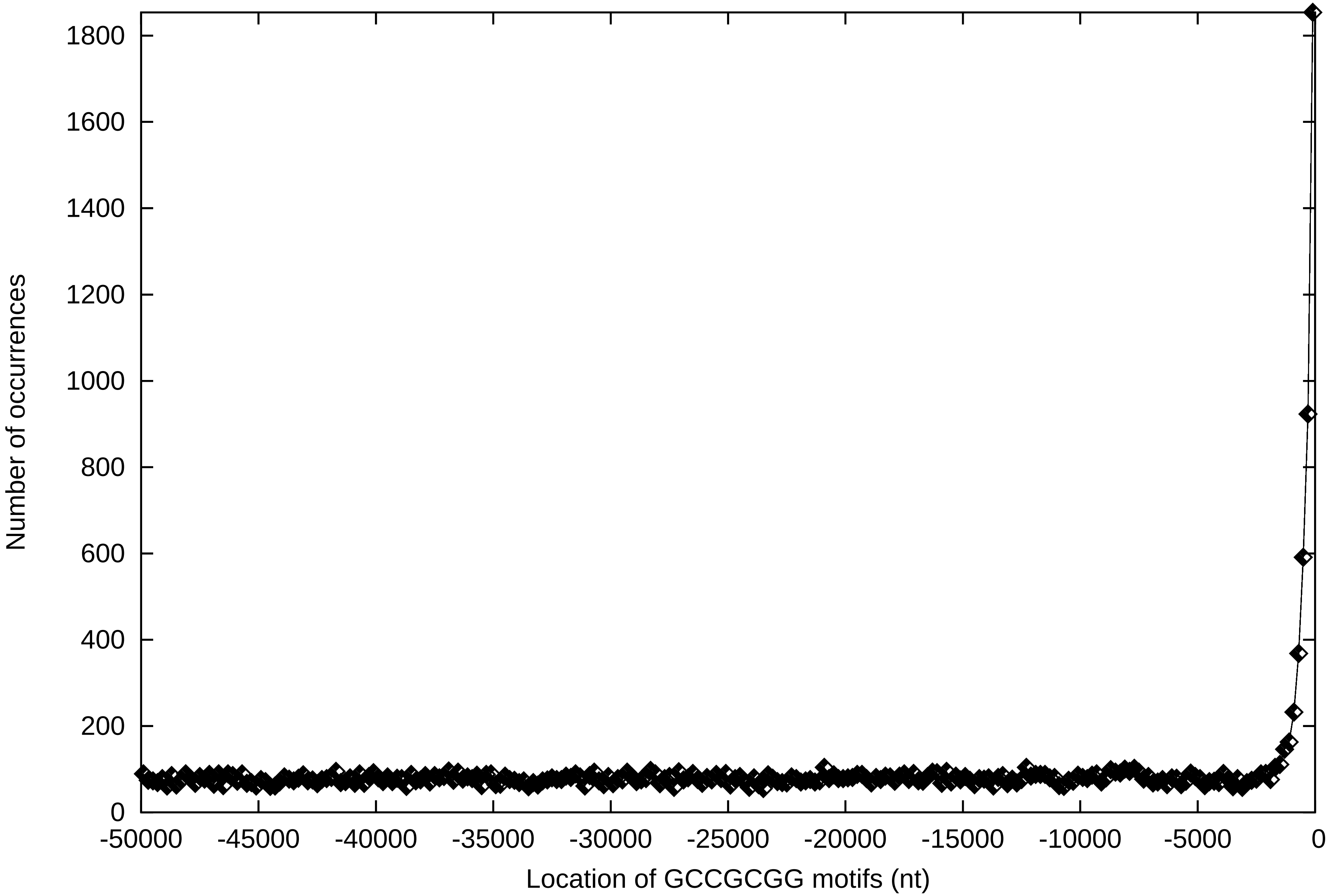

Number of occurrences
0
200
400
600
800
1000
1200
1400
1600
1800
-50000
-45000
-40000
-35000
-30000
-25000
-20000
-15000
-10000
-5000
0
Location of GCCGCGG motifs (nt)

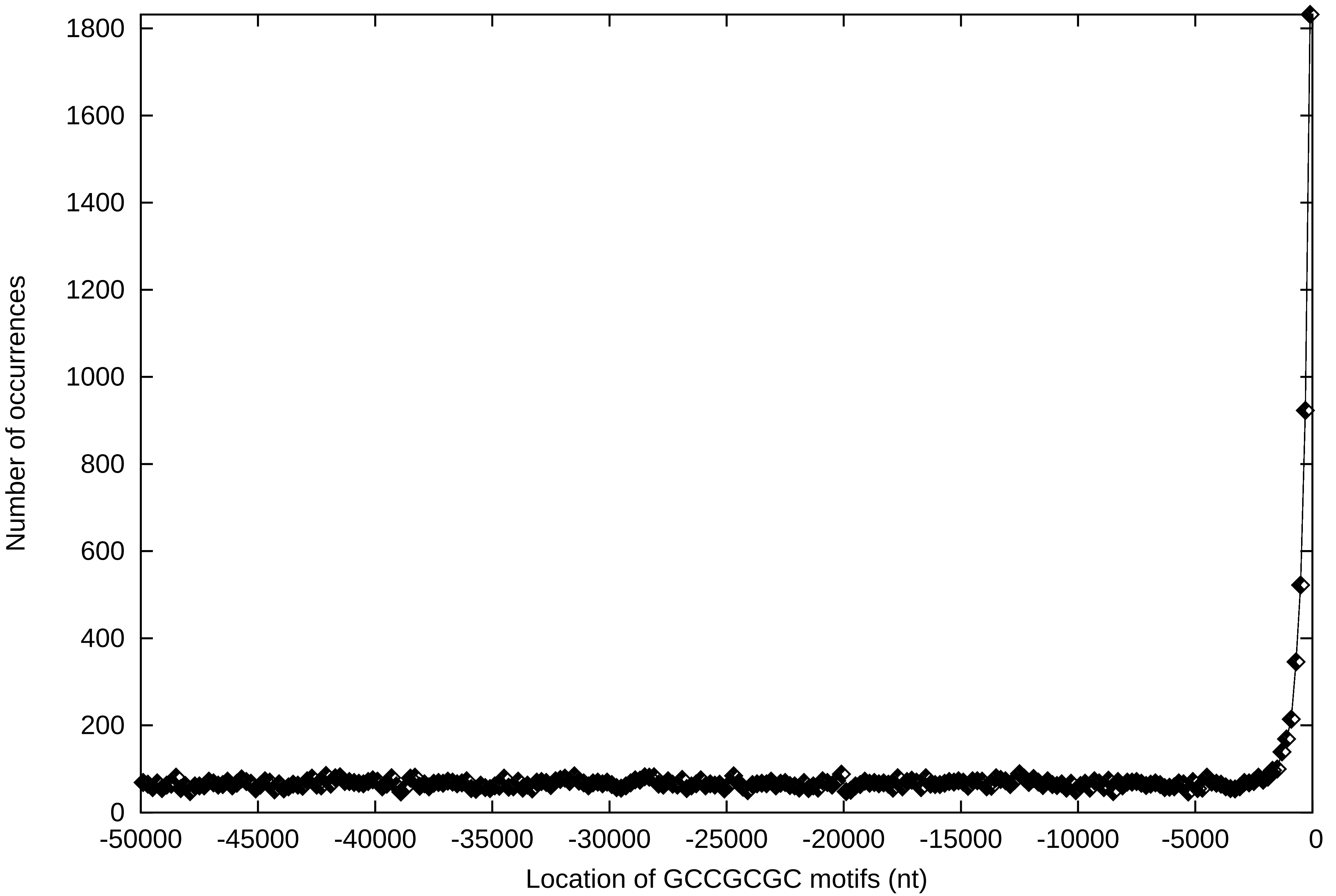

Number of occurrences
0
200
400
600
800
1000
1200
1400
1600
1800
-50000
-45000
-40000
-35000
-30000
-25000
-20000
-15000
-10000
-5000
0
Location of GCCGCGC motifs (nt)

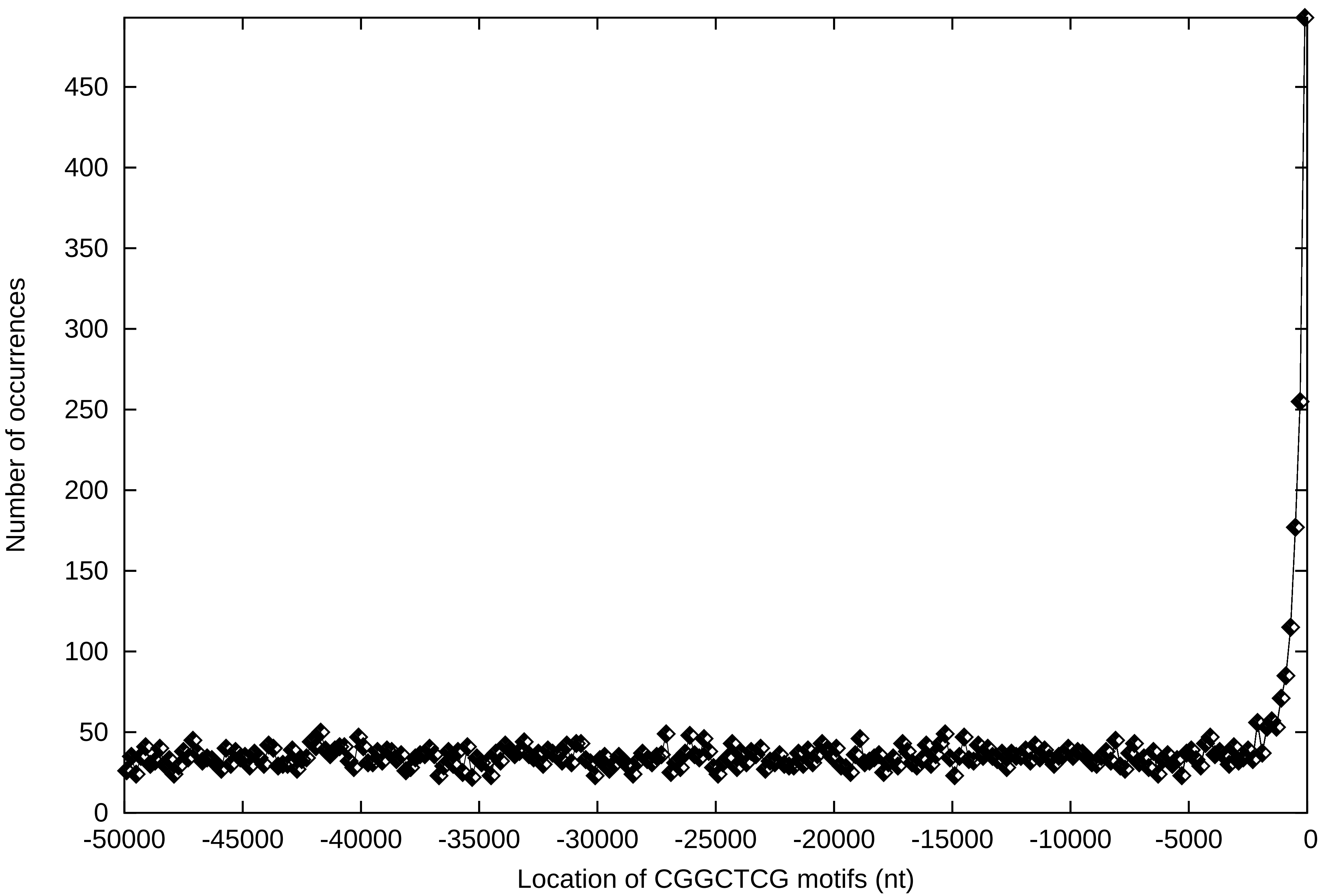

Number of occurrences
0
50
100
150
200
250
300
350
400
450
-50000
-45000
-40000
-35000
-30000
-25000
-20000
-15000
-10000
-5000
0
Location of CGGCTCG motifs (nt)

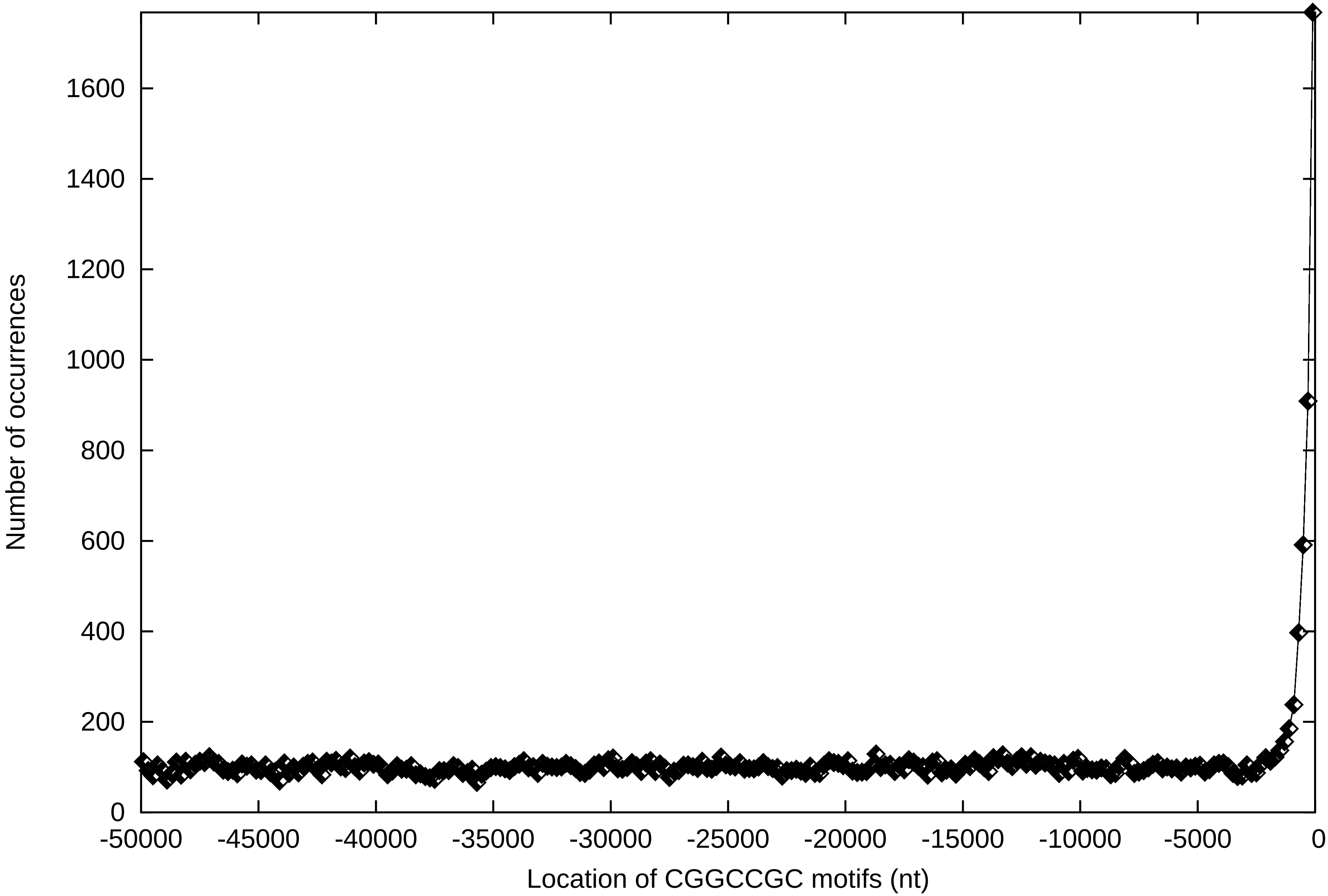

Number of occurrences
1600
1400
1200
1000
800
600
400
200
0
-50000
-45000
-40000
-35000
-30000
-25000
-20000
-15000
-10000
-5000
0
Location of CGGCCGC motifs (nt)

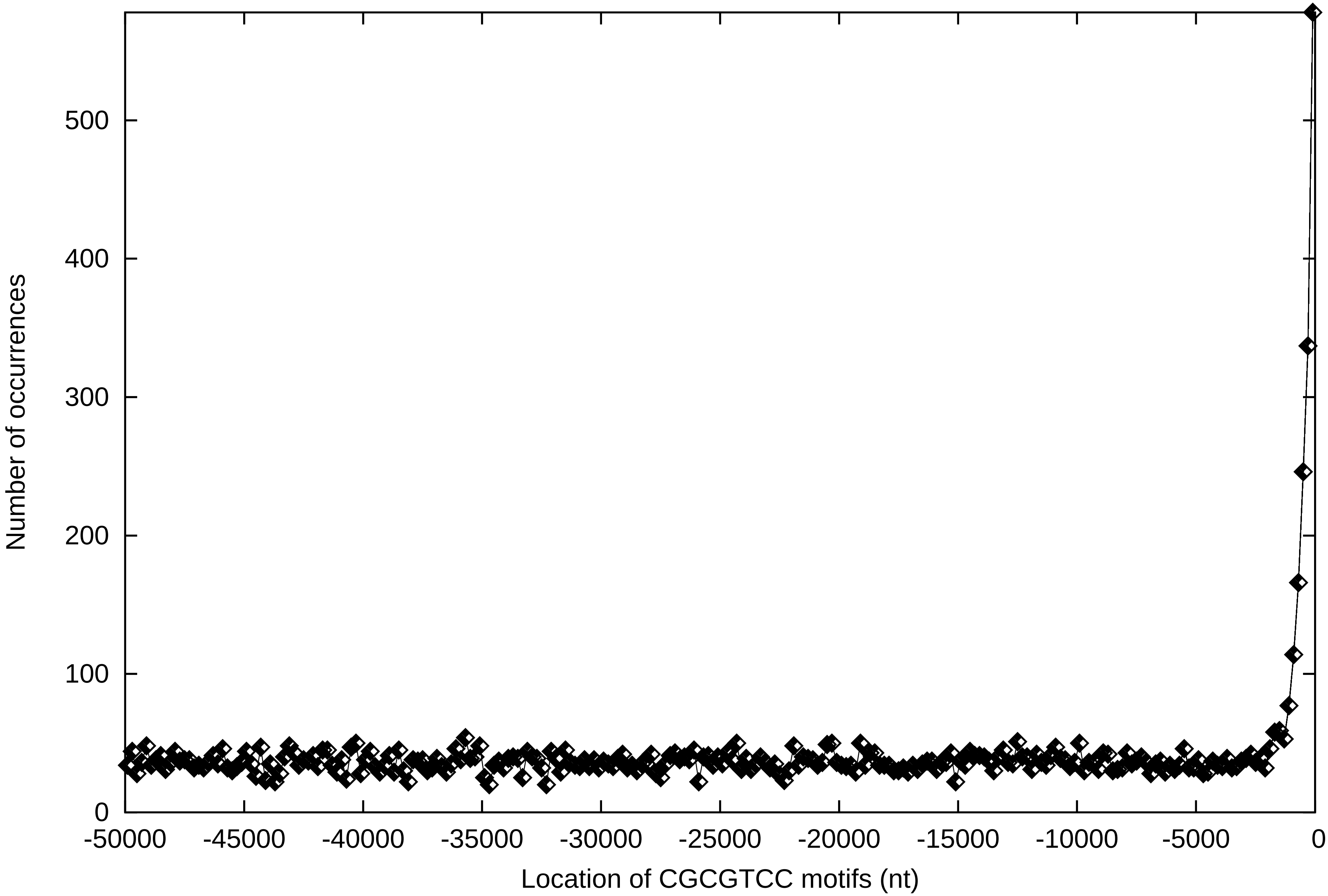

Number of occurrences
500
400
300
200
100
0
-50000
-45000
-40000
-35000
-30000
-25000
-20000
-15000
-10000
-5000
0
Location of CGCGTCC motifs (nt)

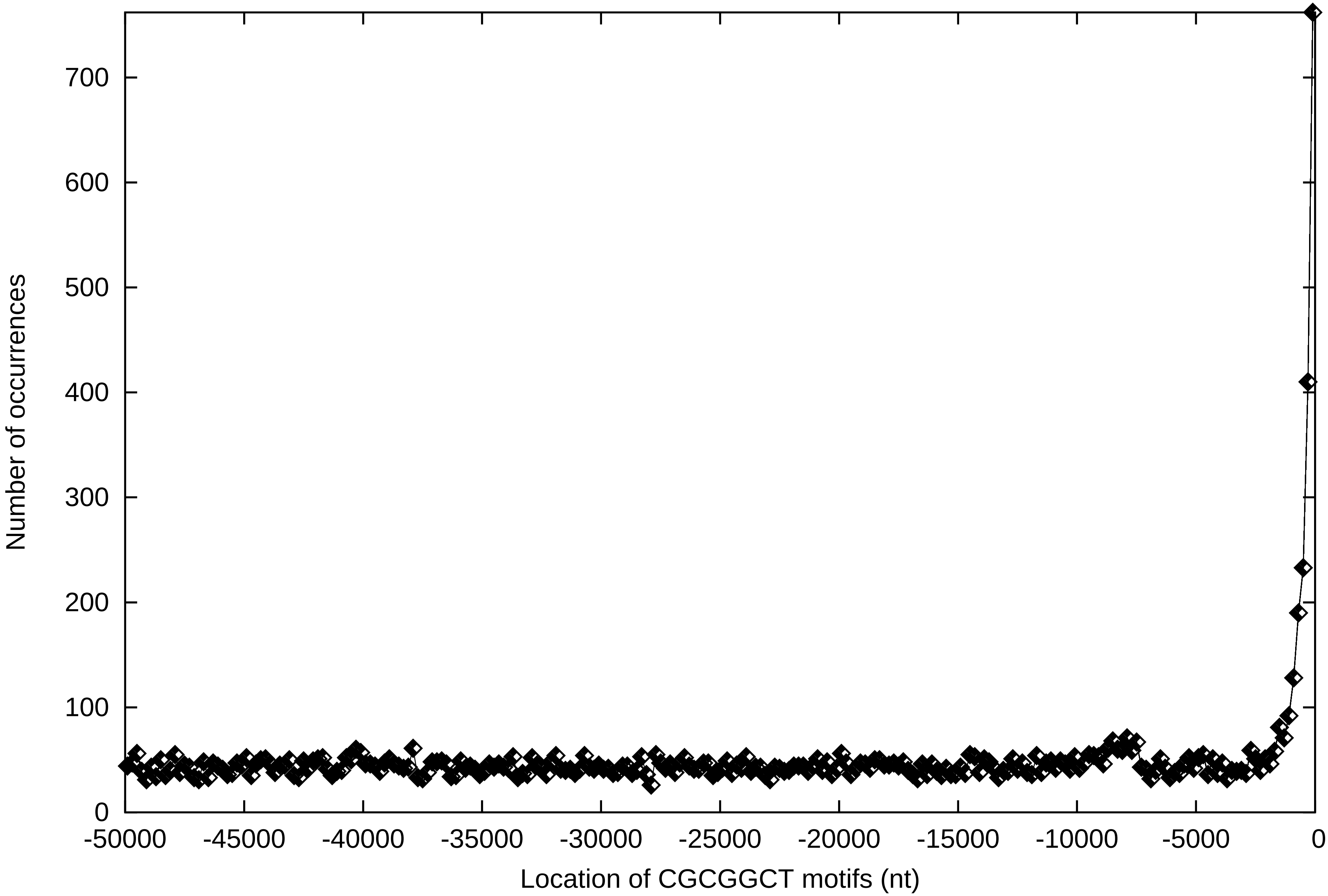
Number of occurrences
700
600
500
400
300
200
100
0
-50000
-45000
-40000
-35000
-30000
-25000
-20000
-15000
-10000
-5000
0
Location of CGCGGCT motifs (nt)

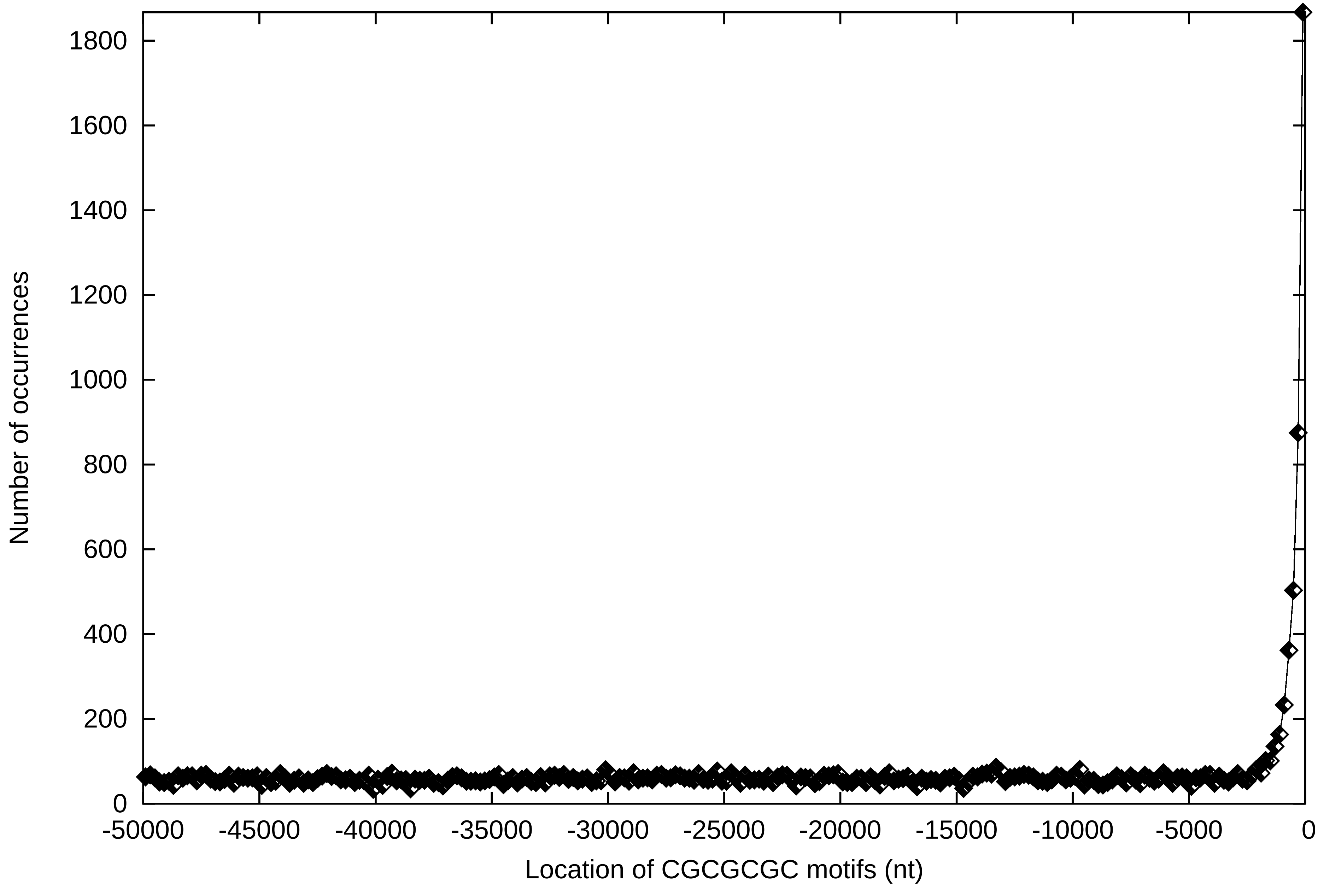

Number of occurrences
1800
1600
1400
1200
1000
800
600
400
200
0
-50000
-45000
-40000
-35000
-30000
-25000
-20000
-15000
-10000
-5000
0
Location of CGCGCGC motifs (nt)

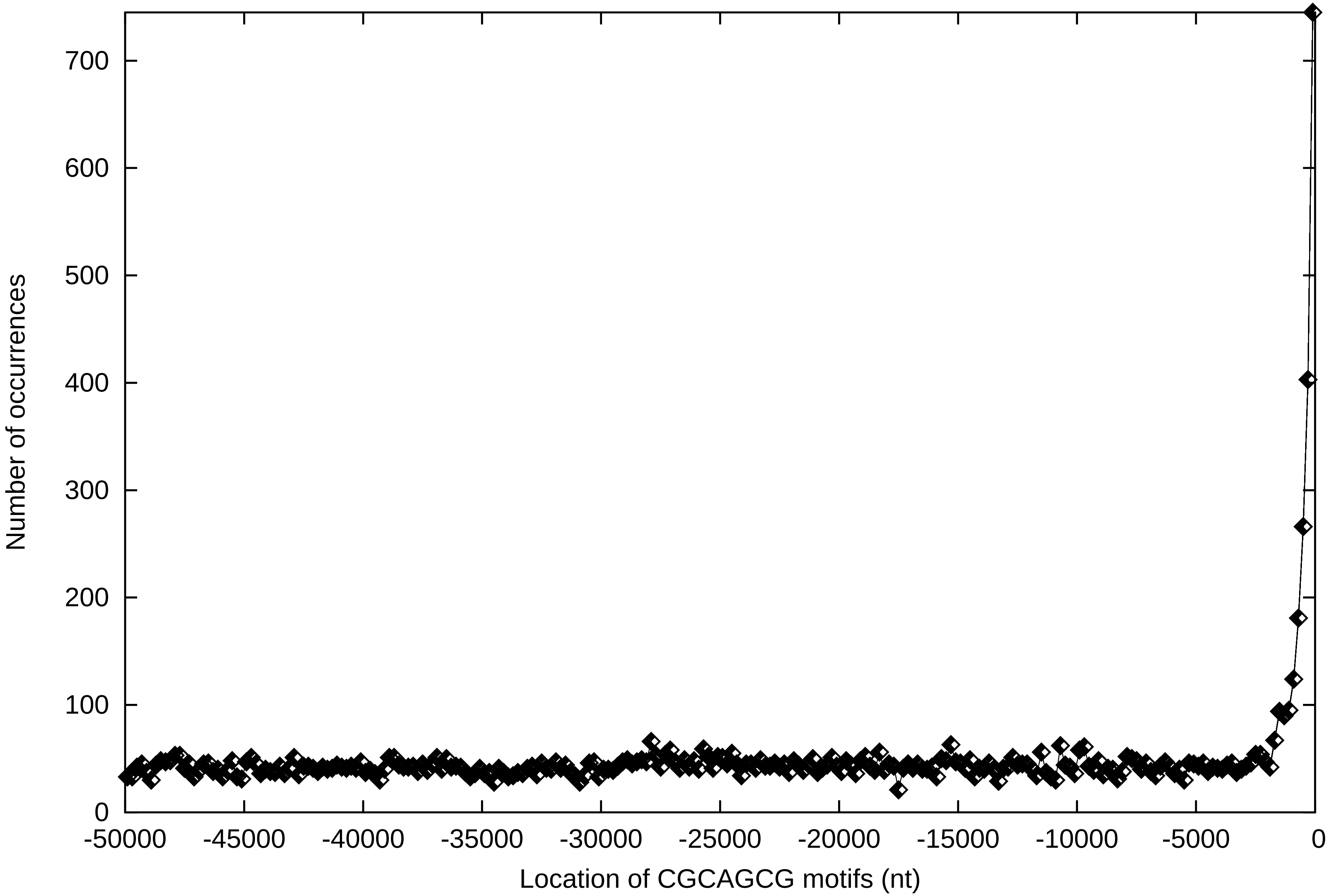

700
600
500
400
300
200
100
0
Number of occurrences
-50000
-45000
-40000
-35000
-30000
-25000
-20000
-15000
-10000
-5000
0
Location of CGCAGCG motifs (nt)

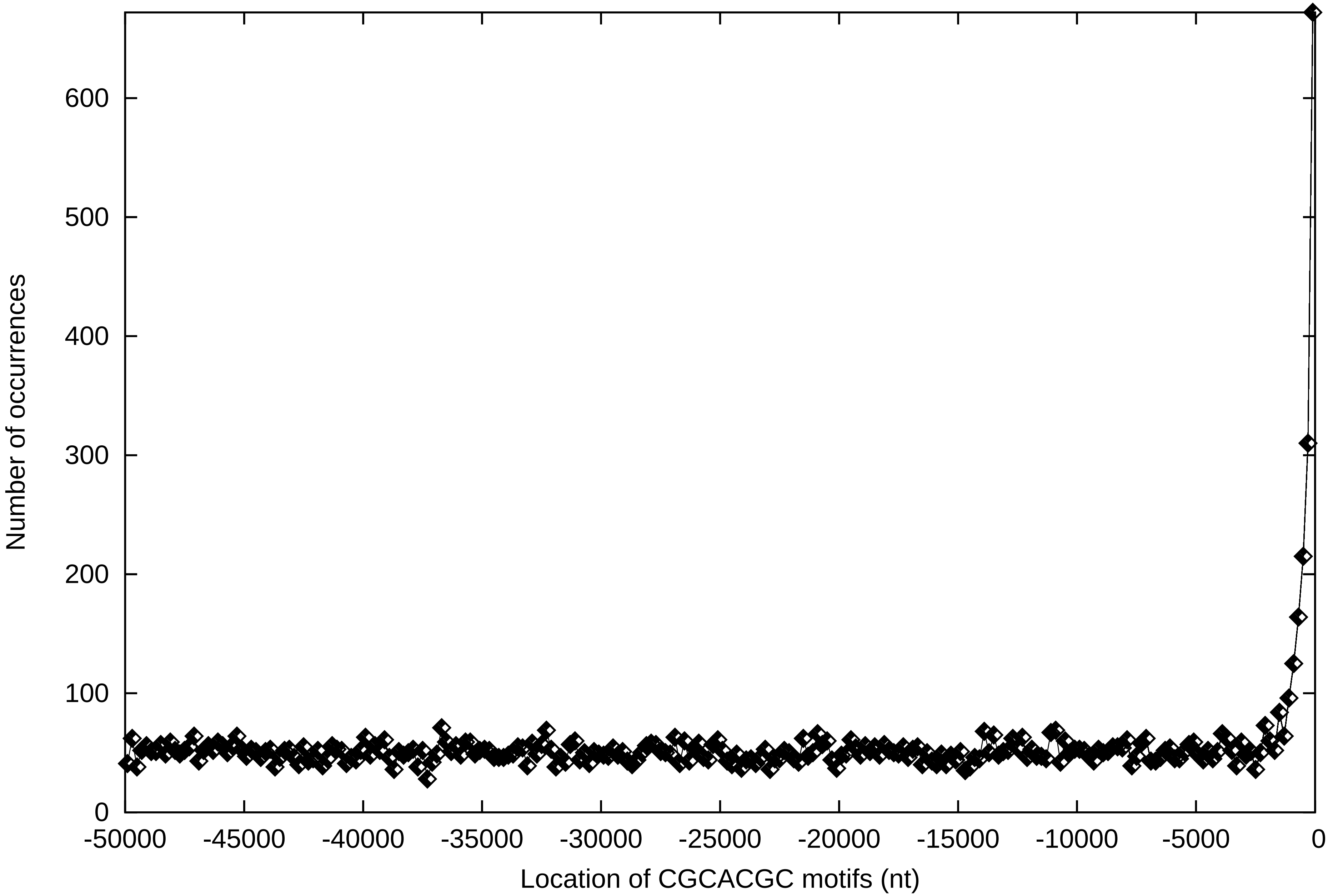
600
500
400
300
200
100
0
Number of occurrences
-50000
-45000
-40000
-35000
-30000
-25000
-20000
-15000
-10000
-5000
0
Location of CGCACGC motifs (nt)

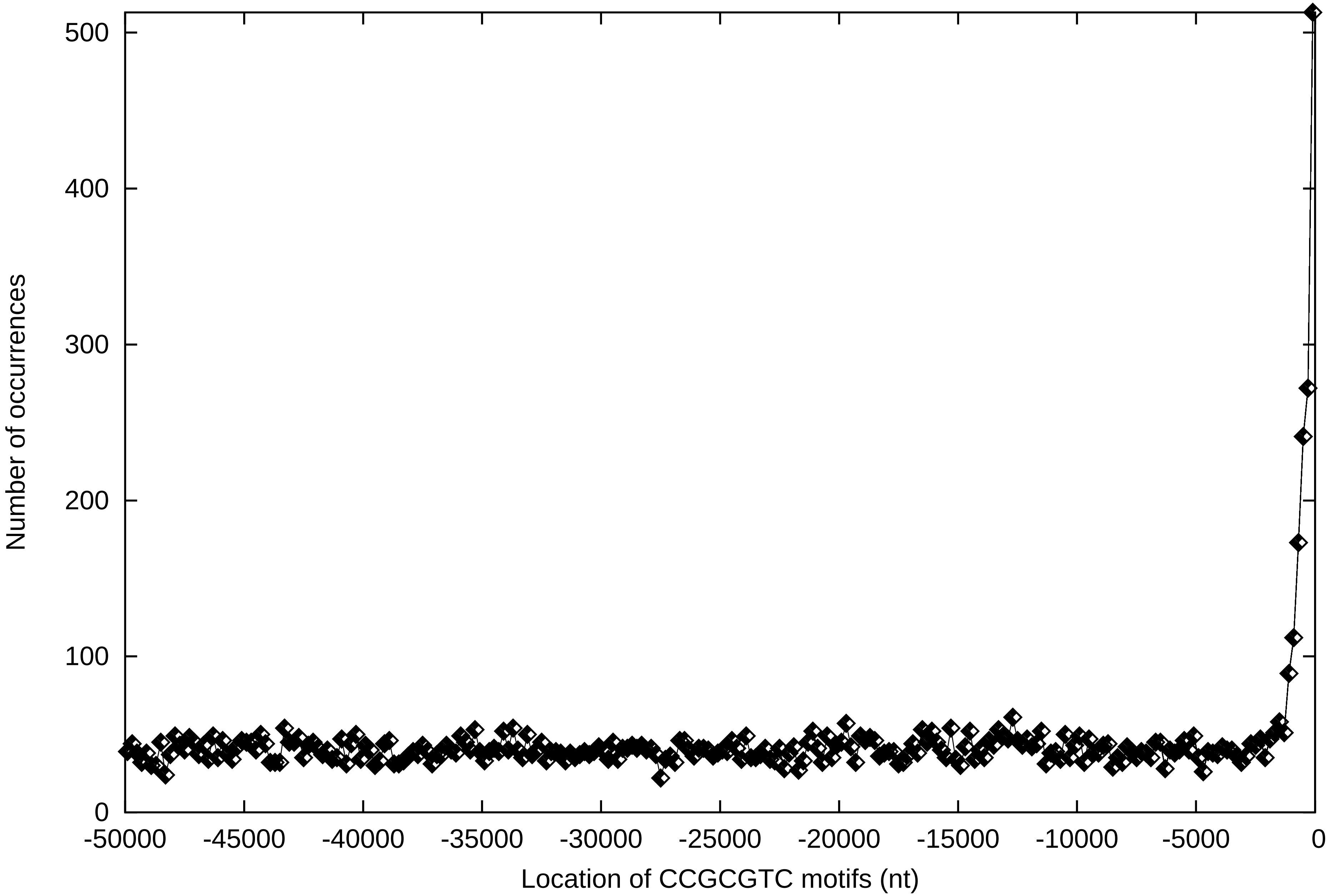
Number of occurrences
0
100
200
300
400
500
-50000
-45000
-40000
-35000
-30000
-25000
-20000
-15000
-10000
-5000
0
Location of CCGCGTC motifs (nt)

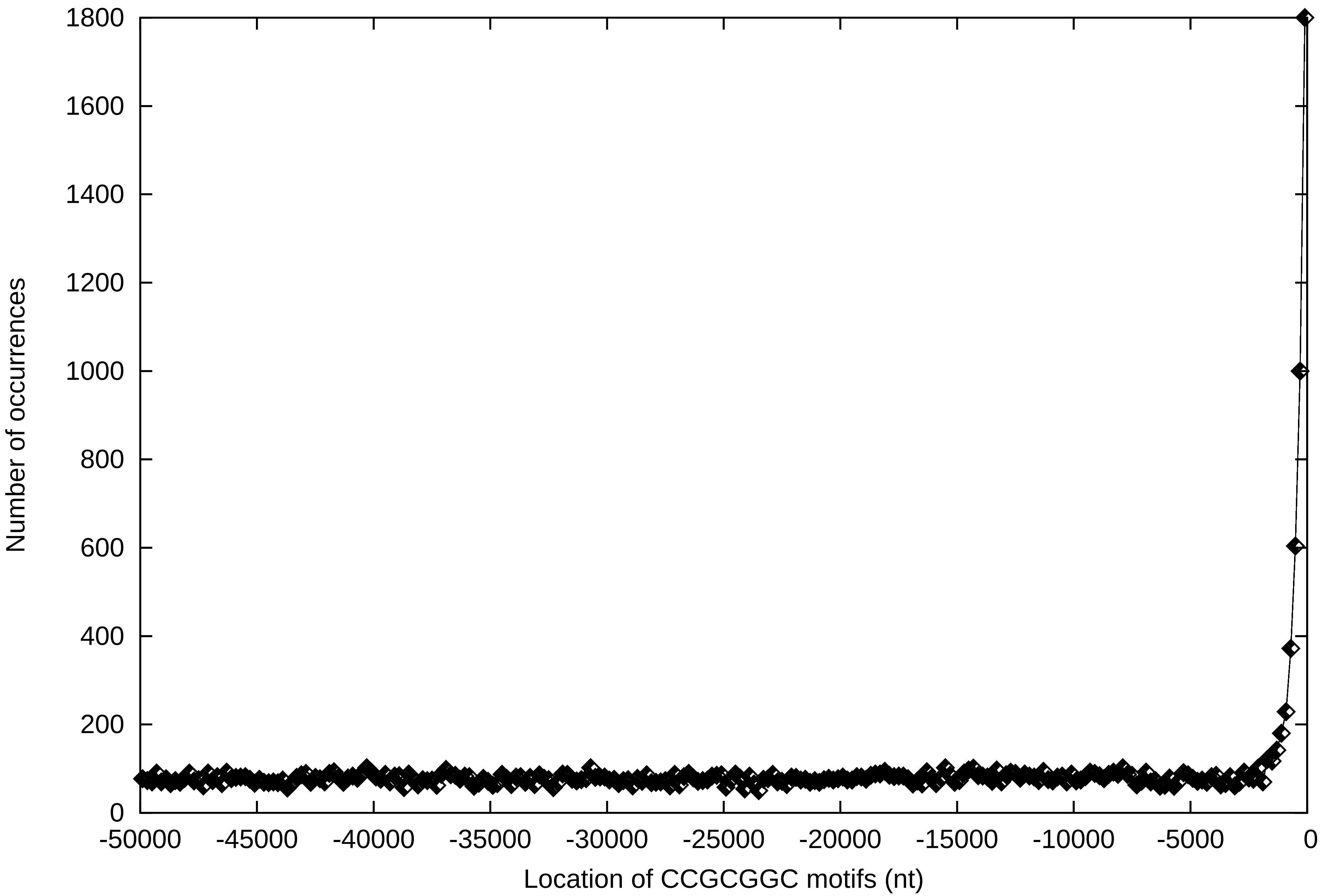

1800
1600
1400
1200
1000
800
600
400
200
0
Number of occurrences
-50000
-45000
-40000
-35000
-30000
-25000
-20000
-15000
-10000
-5000
0
Location of CCGCGGC motifs (nt)

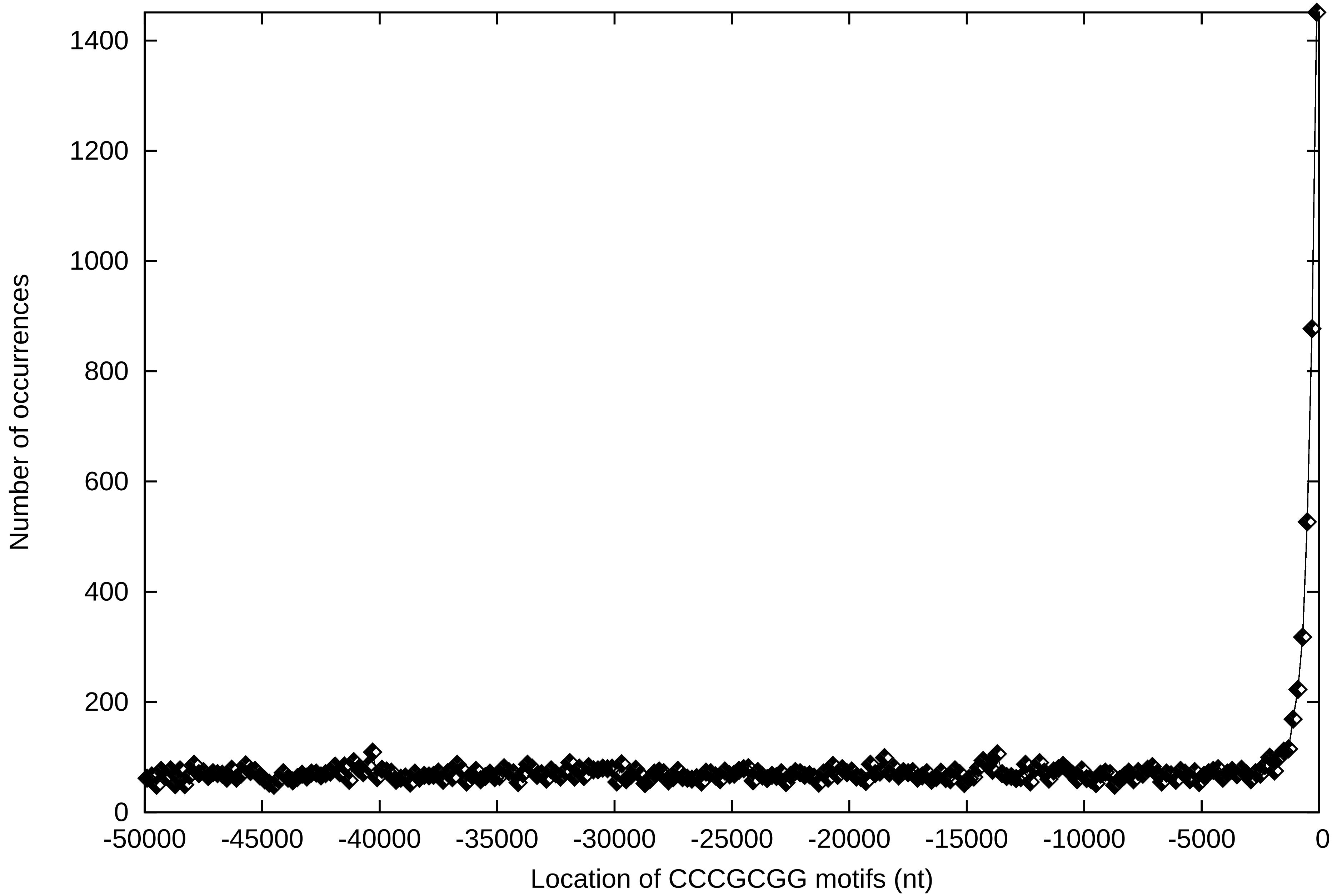

1400
1200
1000
800
600
400
200
0
Number of occurrences
-50000
-45000
-40000
-35000
-30000
-25000
-20000
-15000
-10000
-5000
0
Location of CCCGCGG motifs (nt)
