# Supplementary material for: Regulatory Circuit of Human MicroRNA Biogenesis
Source: PLoS Comput Biol. 2007 Apr 20;3(4):e67. doi: 10.1371/journal.pcbi.0030067 (PMC1853126; doi:10.1371/journal.pcbi.0030067)
Supplement: Supplementary file 4 [file pcbi.0030067.sg004.pdf]

The 100 top-predicted 6-mers are preferentially located upstream of protein-coding genes (pages 2-101). Each graph is a histogram that represents the number of occurrences of each motif (every 200 nt stretch) in the first 50 kb upstream region of protein-coding genes. The motifs preferentially occur within the first 500 nt region (-500 to 0 nt region) that is directly upstream of the protein-coding genes.

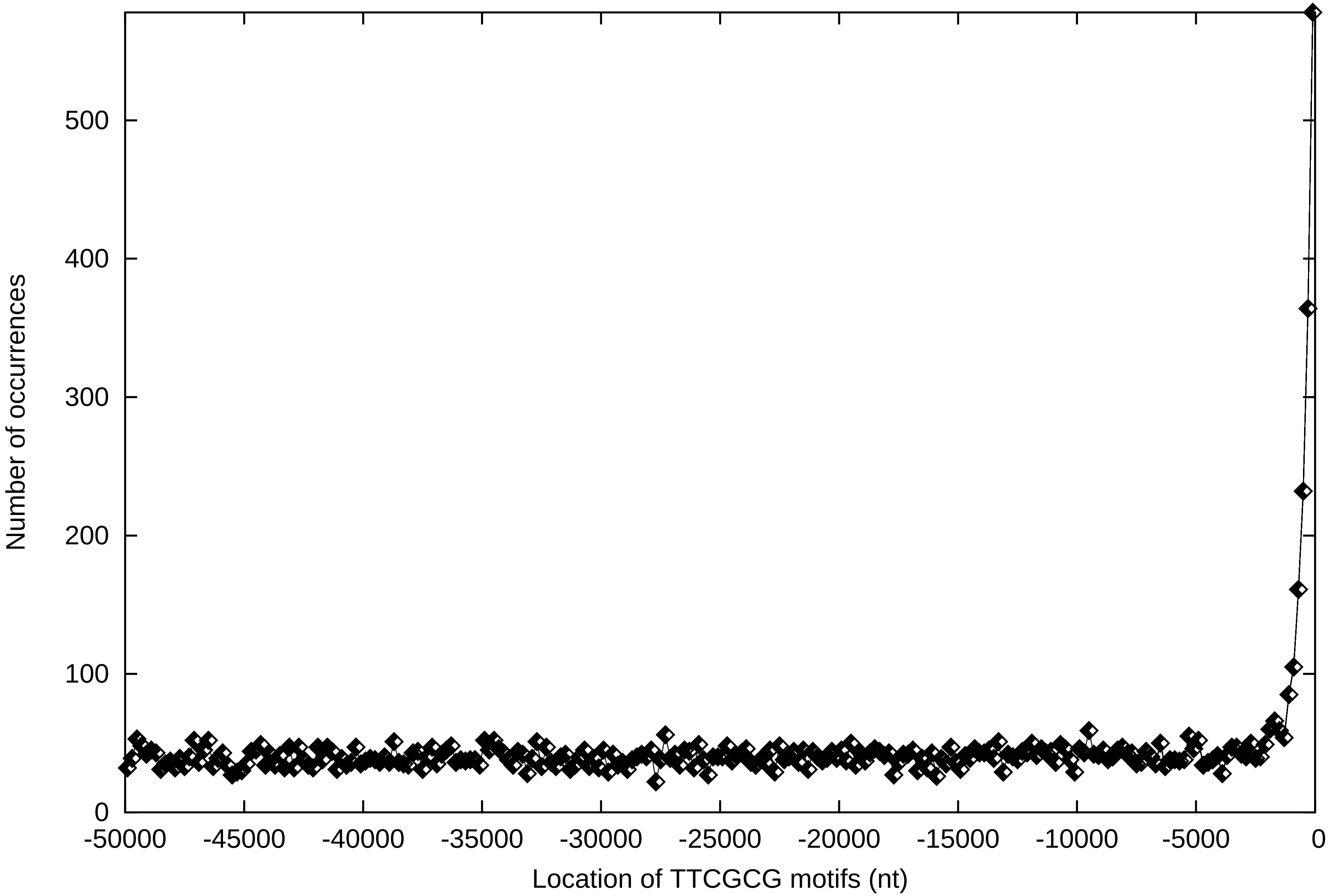
Number of occurrences
0
100
200
300
400
500
-50000
-45000
-40000
-35000
-30000
-25000
-20000
-15000
-10000
-5000
0
Location of TTCGCG motifs (nt)

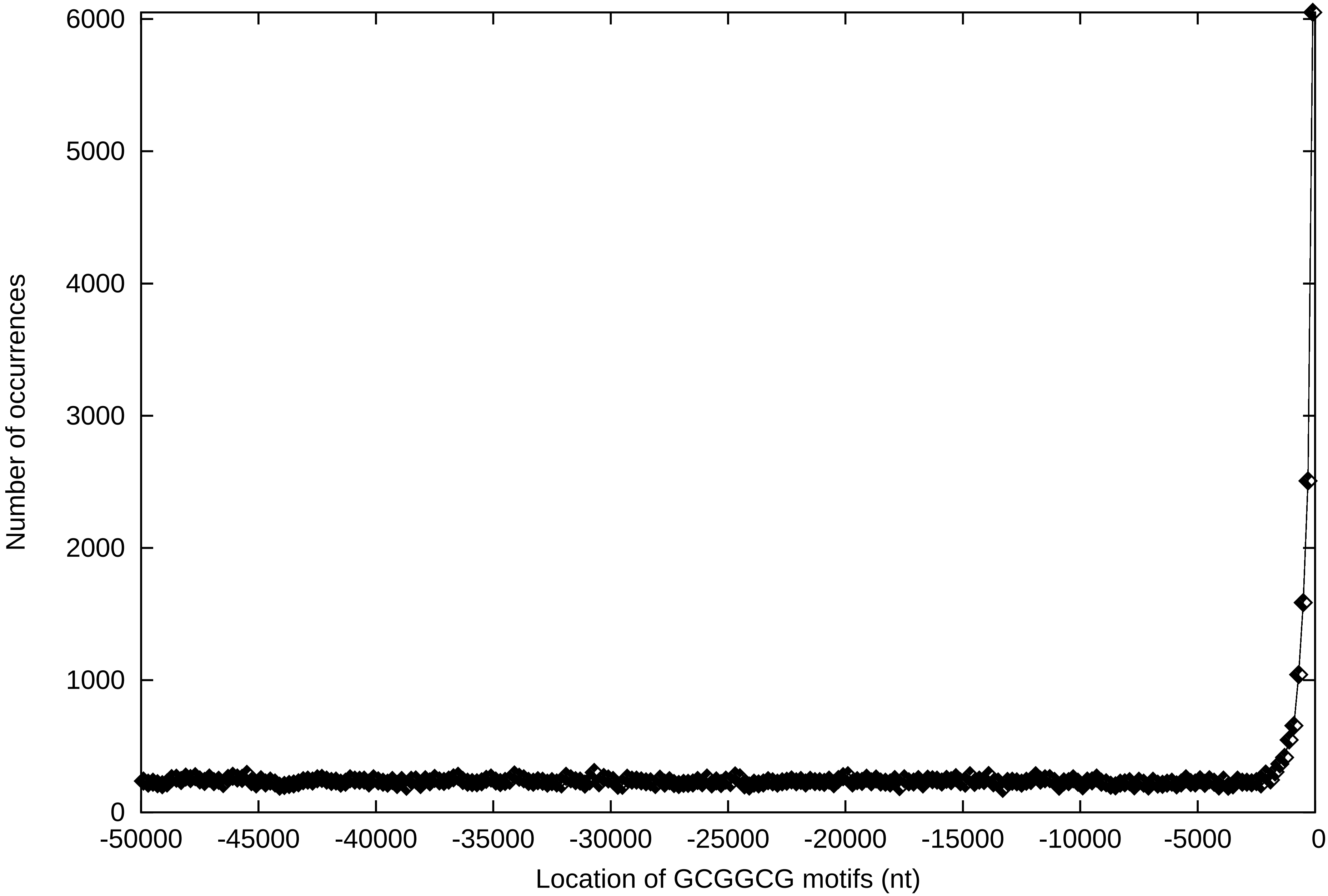

6000
5000
4000
3000
2000
1000
0
Number of occurrences
-50000
-45000
-40000
-35000
-30000
-25000
-20000
-15000
-10000
-5000
0
Location of GCGGCG motifs (nt)

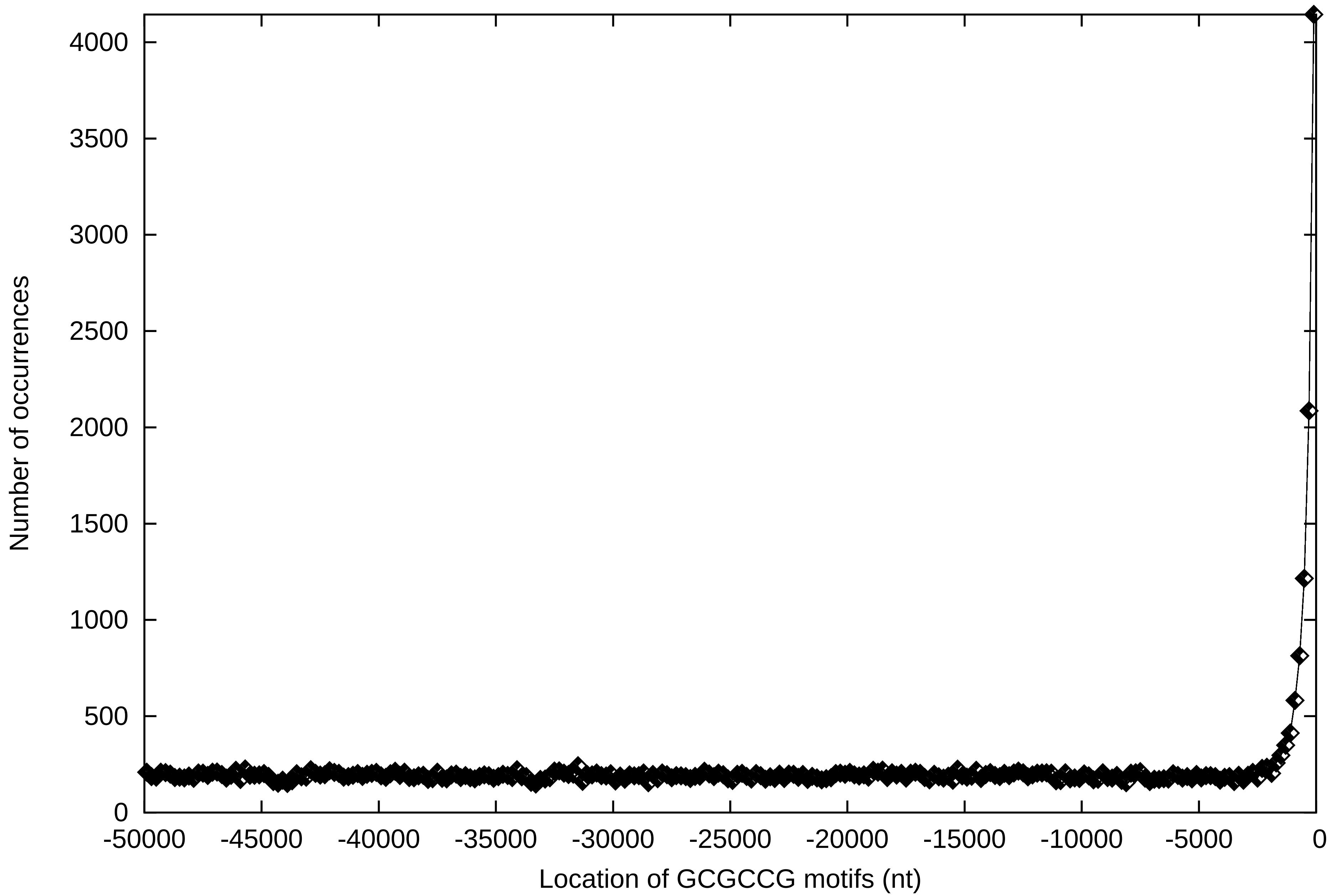

4000
3500
3000
2500
2000
1500
1000
500
0
Number of occurrences
-50000
-45000
-40000
-35000
-30000
-25000
-20000
-15000
-10000
-5000
0
Location of GCGCCG motifs (nt)

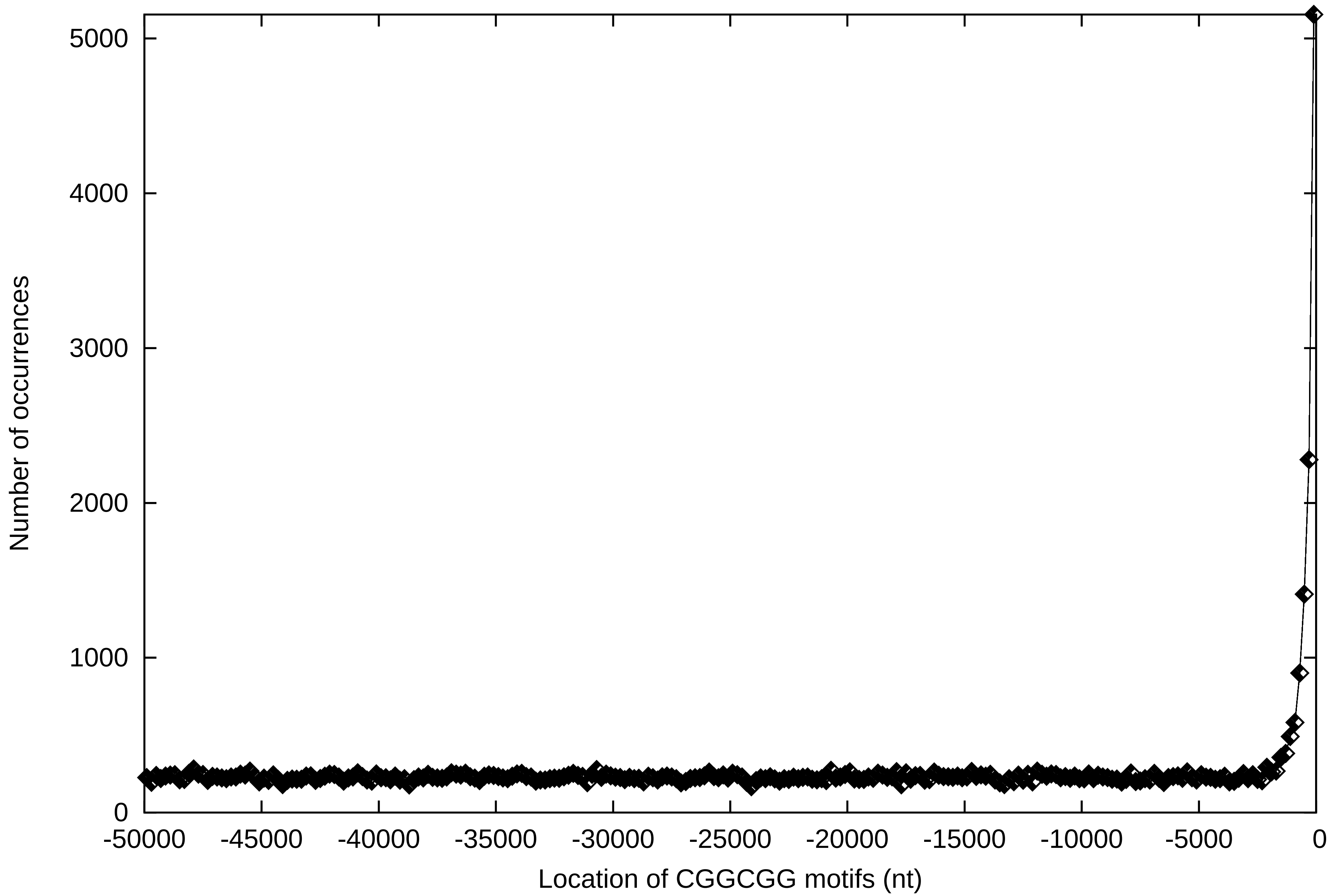

5000
4000
3000
2000
1000
0
-50000
-45000
-40000
-35000
-30000
-25000
-20000
-15000
-10000
-5000
0
Number of occurrences
Location of CGGCGG motifs (nt)

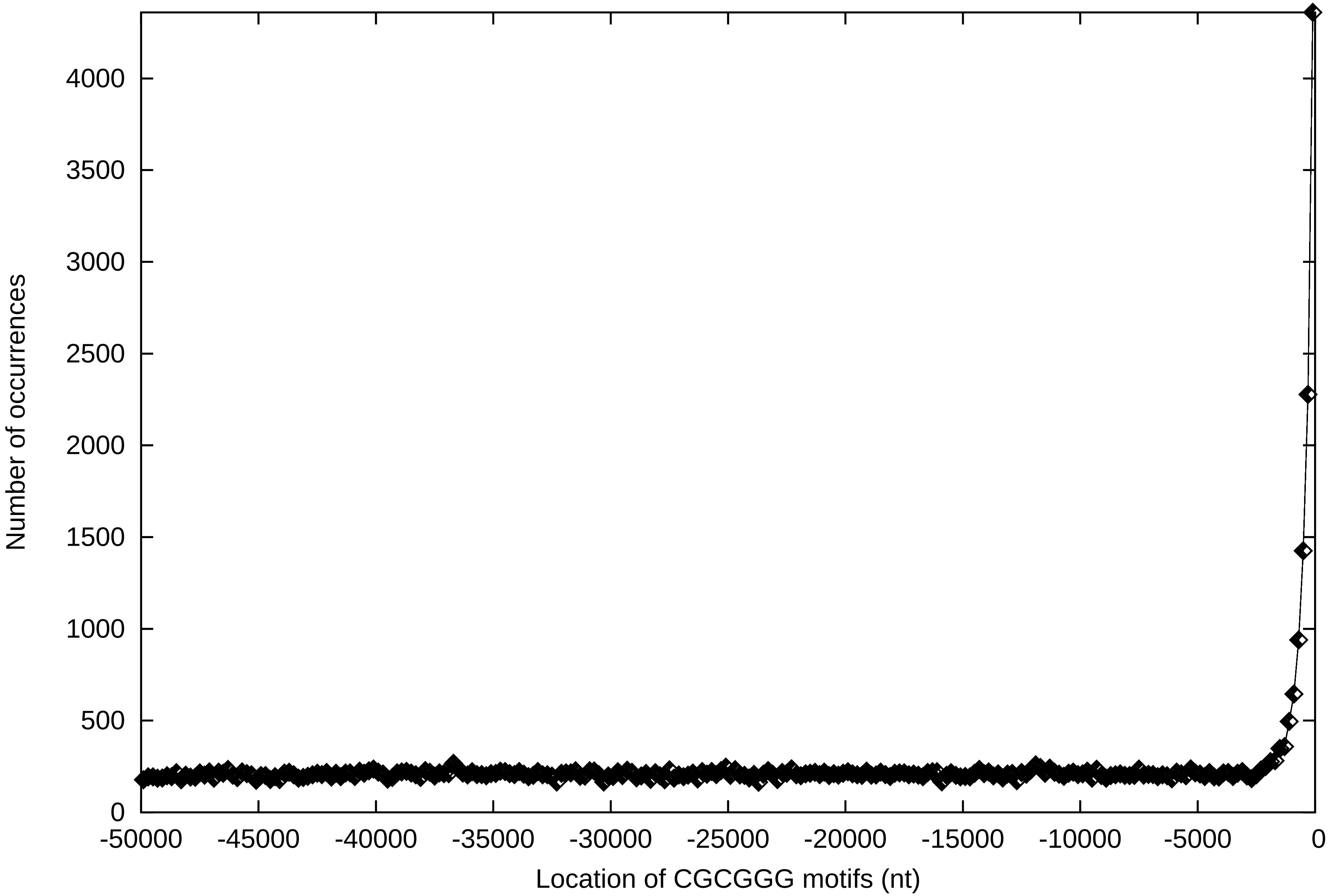

Number of occurrences
0
500
1000
1500
2000
2500
3000
3500
4000
-50000
-45000
-40000
-35000
-30000
-25000
-20000
-15000
-10000
-5000
0
Location of CGCGGG motifs (nt)

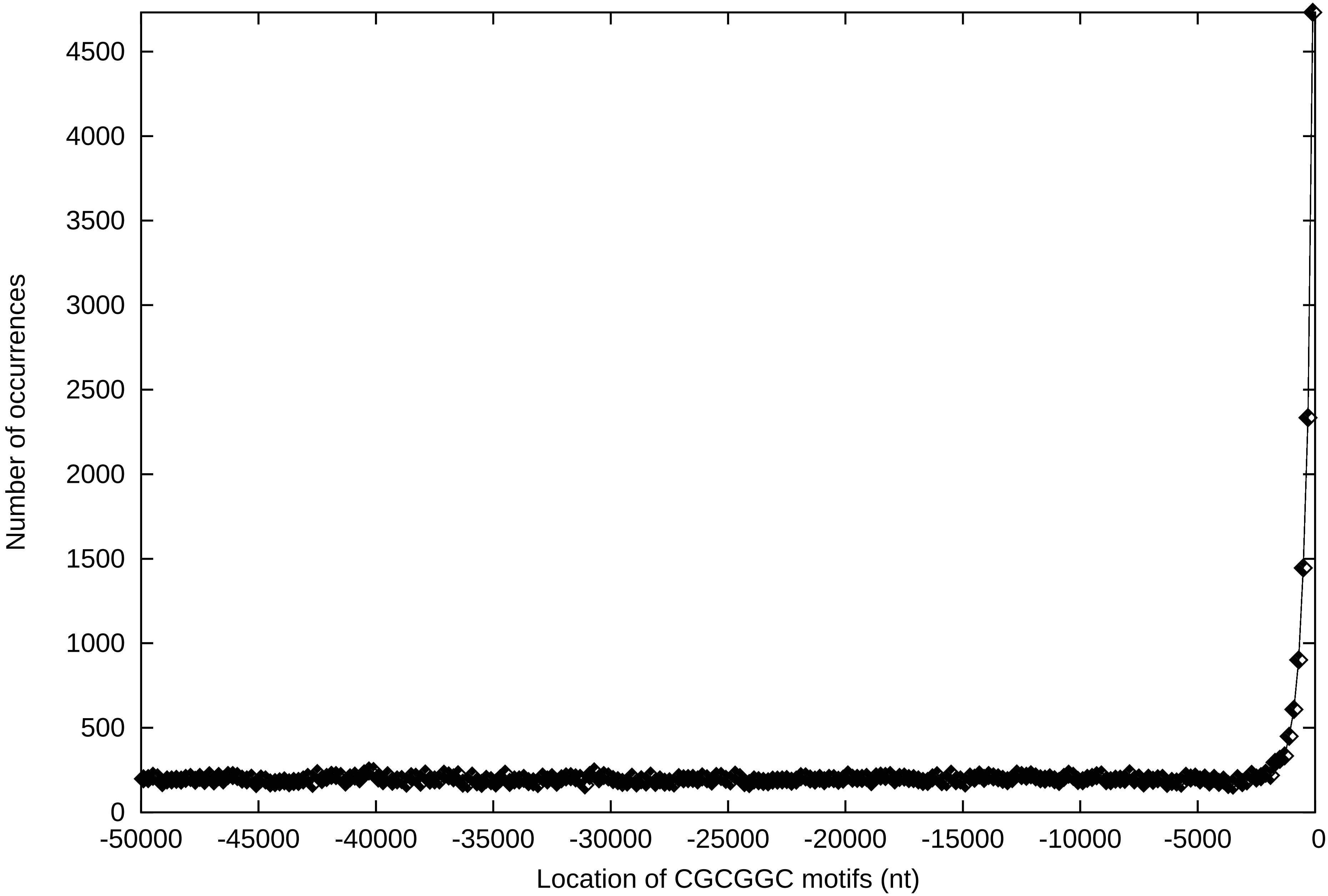

Number of occurrences
Location of CGCGGC motifs (nt)
0
500
1000
1500
2000
2500
3000
3500
4000
4500
-50000
-45000
-40000
-35000
-30000
-25000
-20000
-15000
-10000
-5000
0

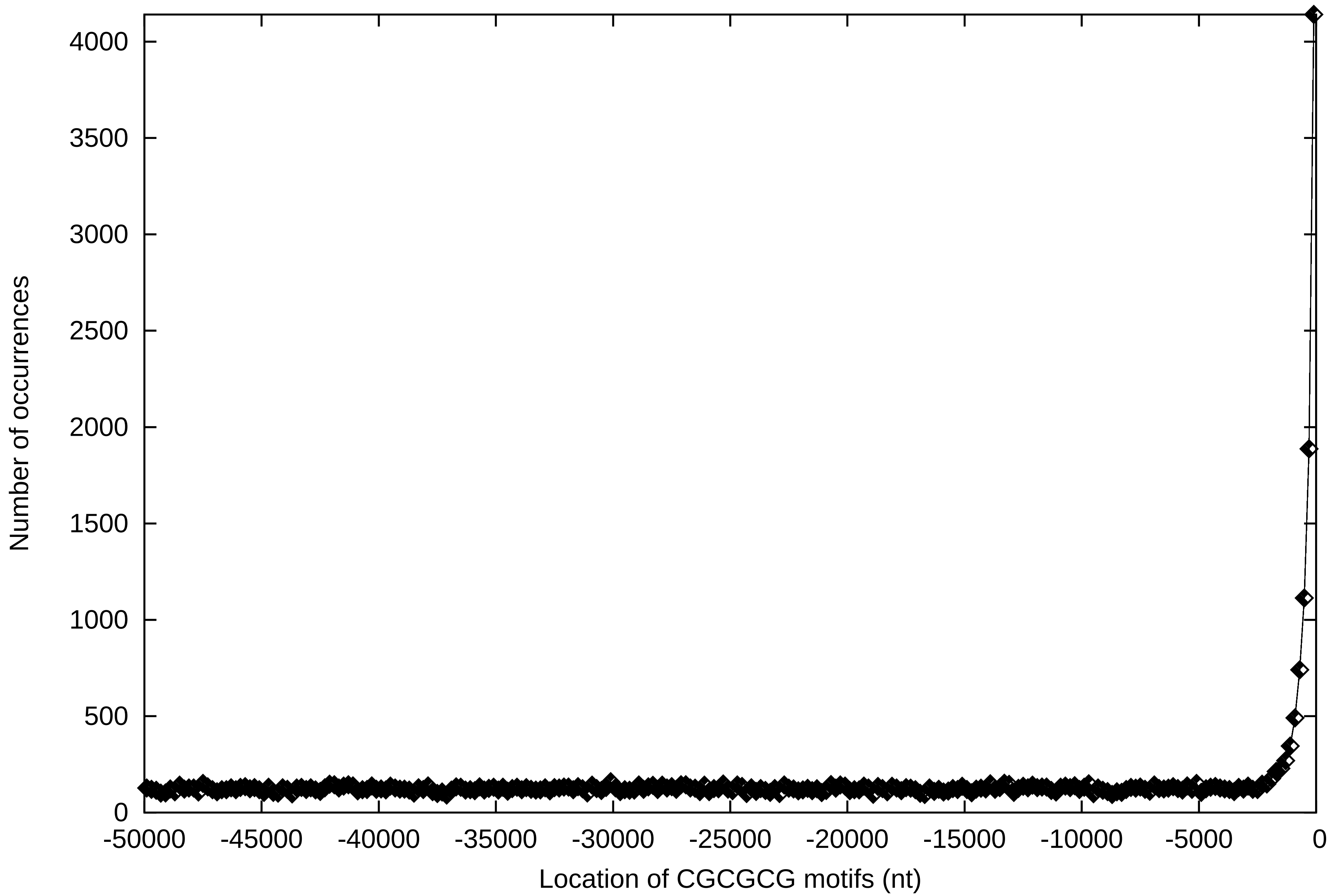

4000
3500
3000
2500
2000
1500
1000
500
0
Number of occurrences
-50000
-45000
-40000
-35000
-30000
-25000
-20000
-15000
-10000
-5000
0
Location of CGCGCG motifs (nt)

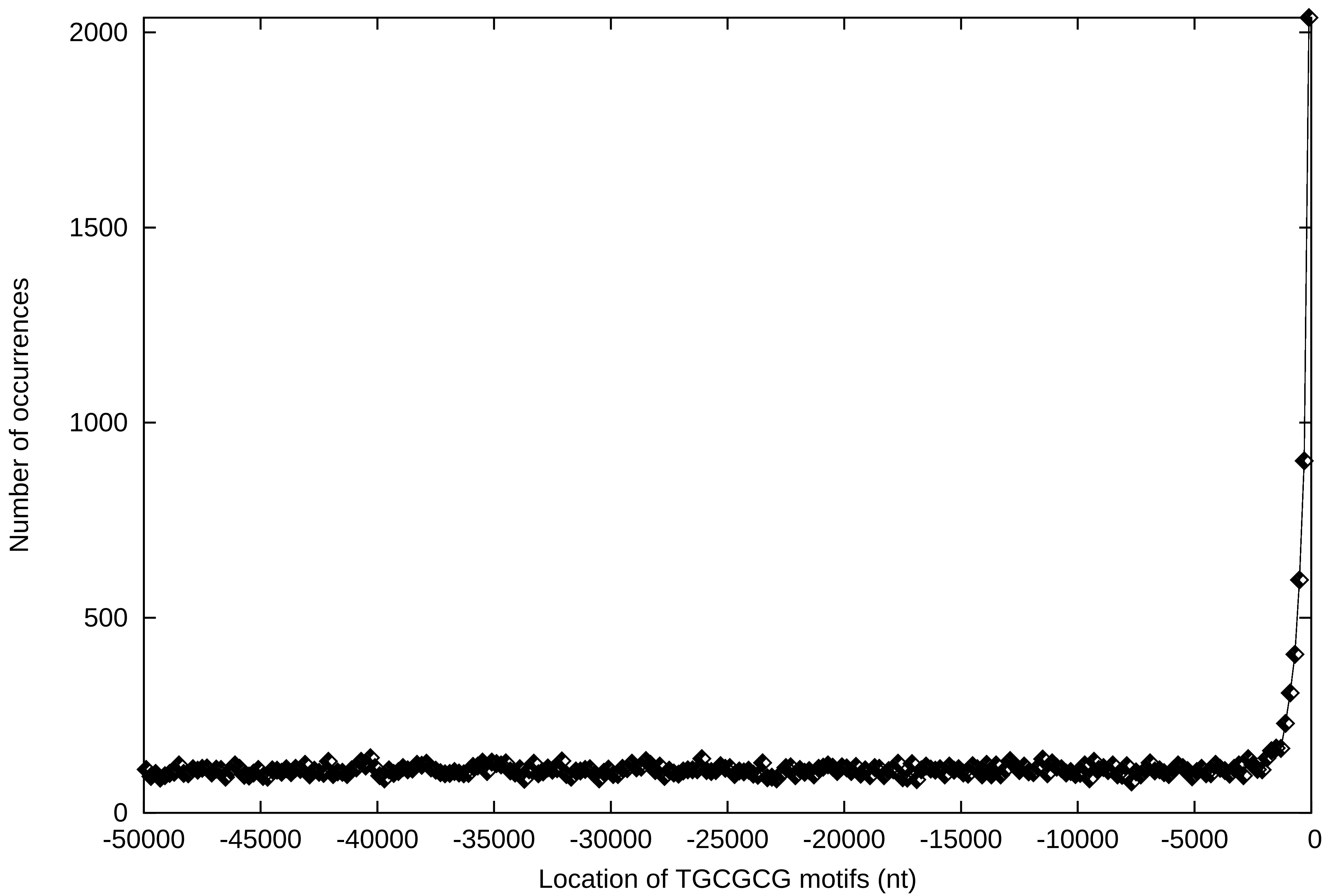

Number of occurrences
Location of TGCGCG motifs (nt)
0
500
1000
1500
2000
-50000
-45000
-40000
-35000
-30000
-25000
-20000
-15000
-10000
-5000
0

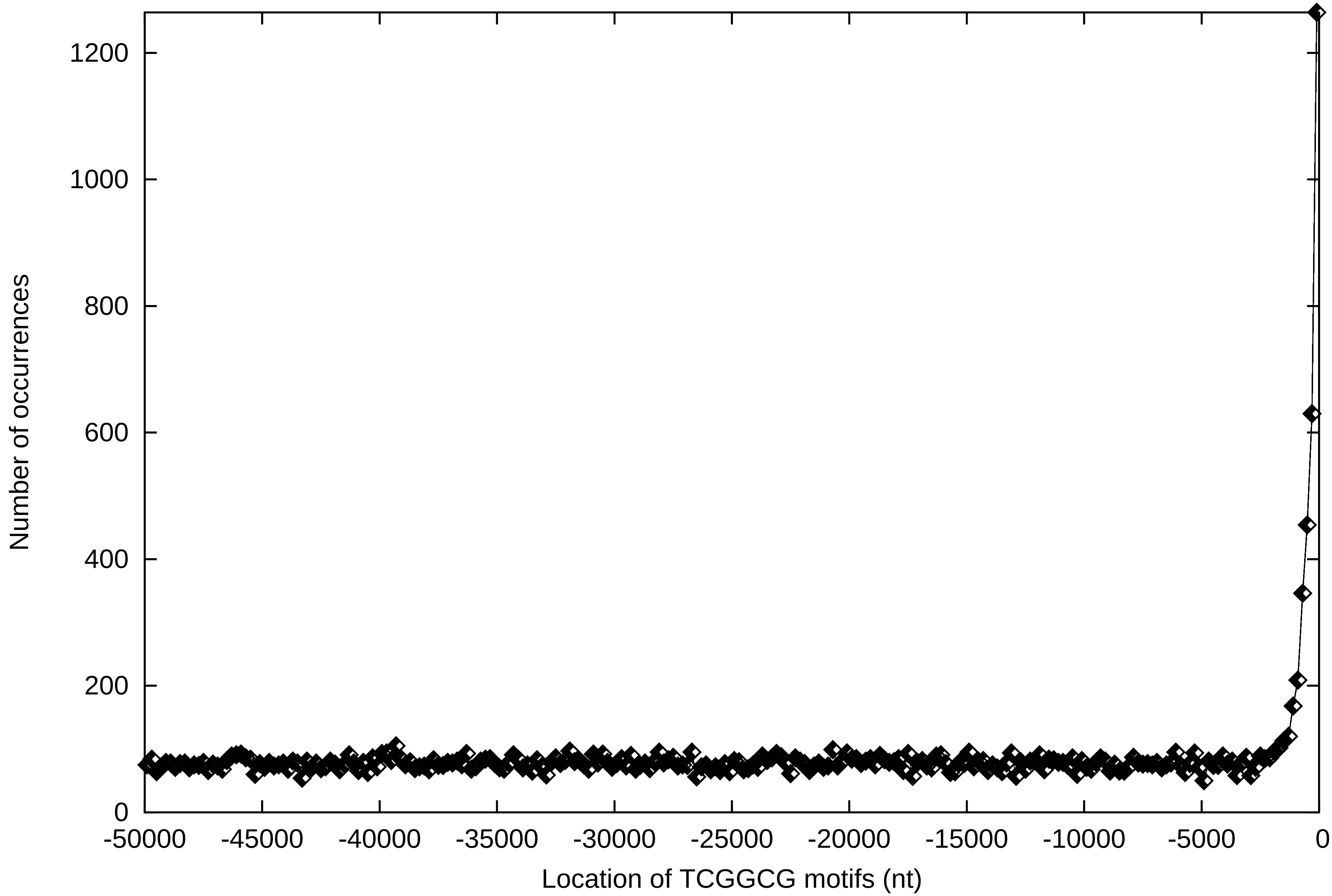

Number of occurrences
0
200
400
600
800
1000
1200
-50000
-45000
-40000
-35000
-30000
-25000
-20000
-15000
-10000
-5000
0
Location of TCGGCG motifs (nt)

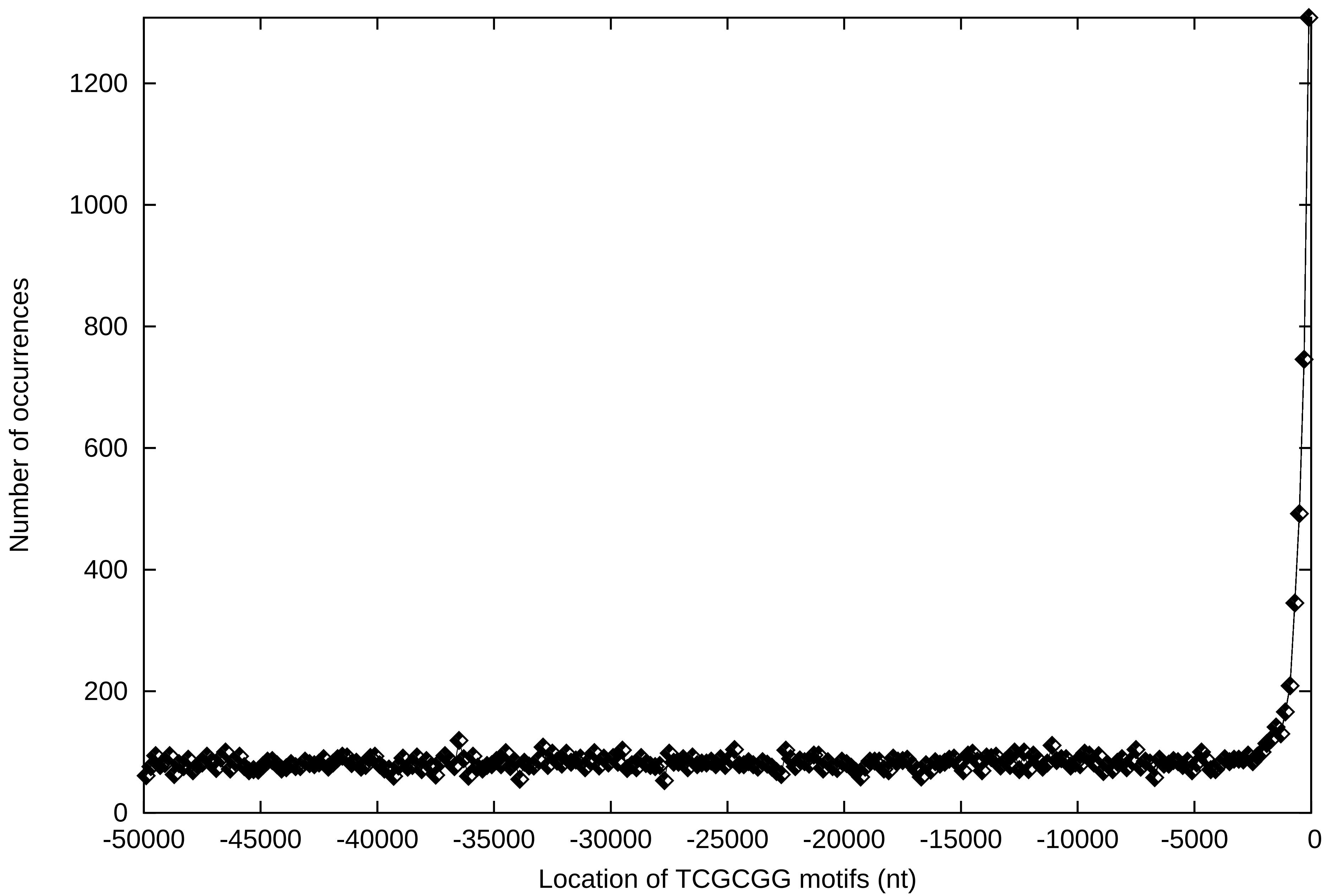
Number of occurrences
0
200
400
600
800
1000
1200
-50000
-45000
-40000
-35000
-30000
-25000
-20000
-15000
-10000
-5000
0
Location of TCGCGG motifs (nt)

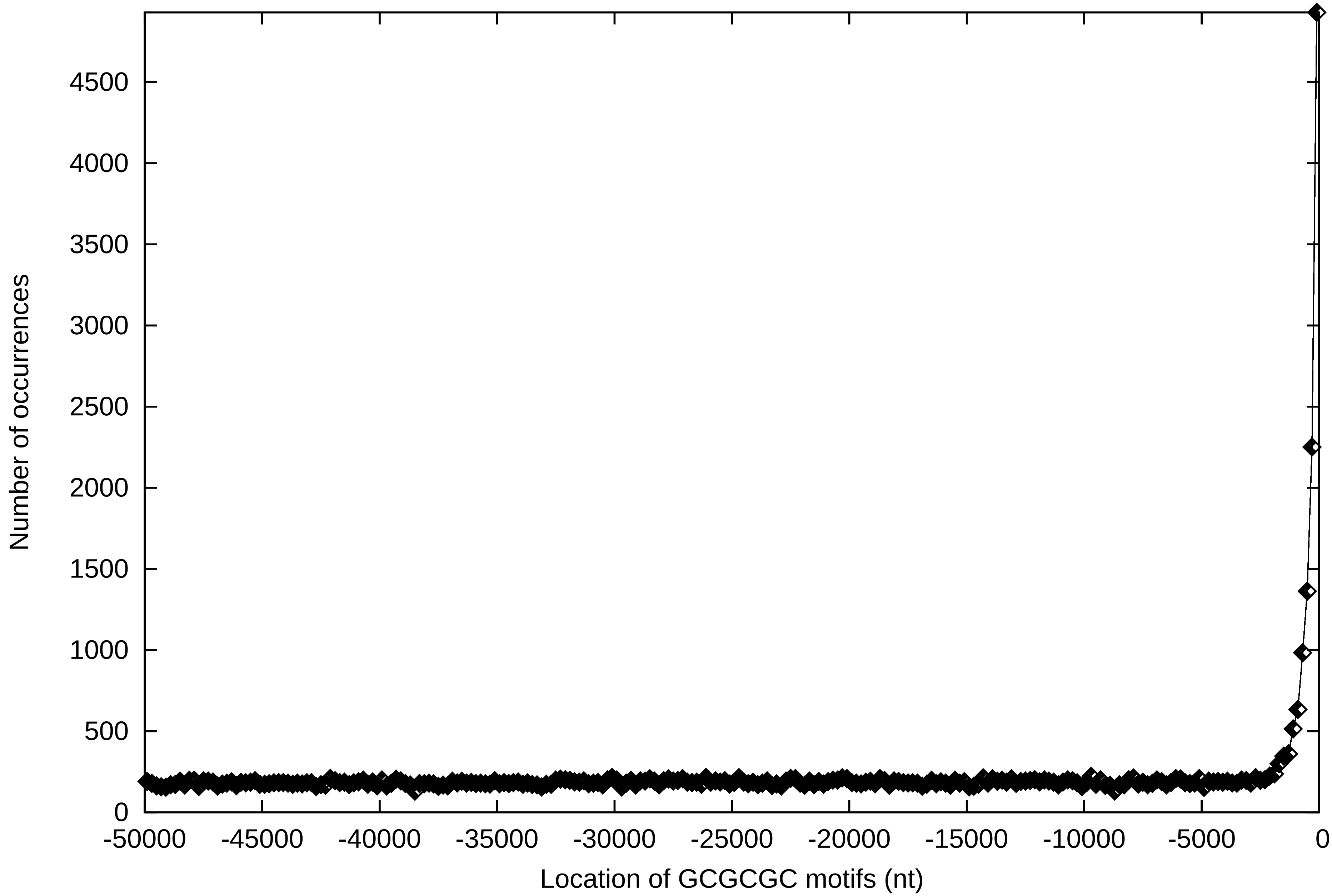

Number of occurrences
4500
4000
3500
3000
2500
2000
1500
1000
500
0
-50000
-45000
-40000
-35000
-30000
-25000
-20000
-15000
-10000
-5000
0
Location of GCGCGC motifs (nt)

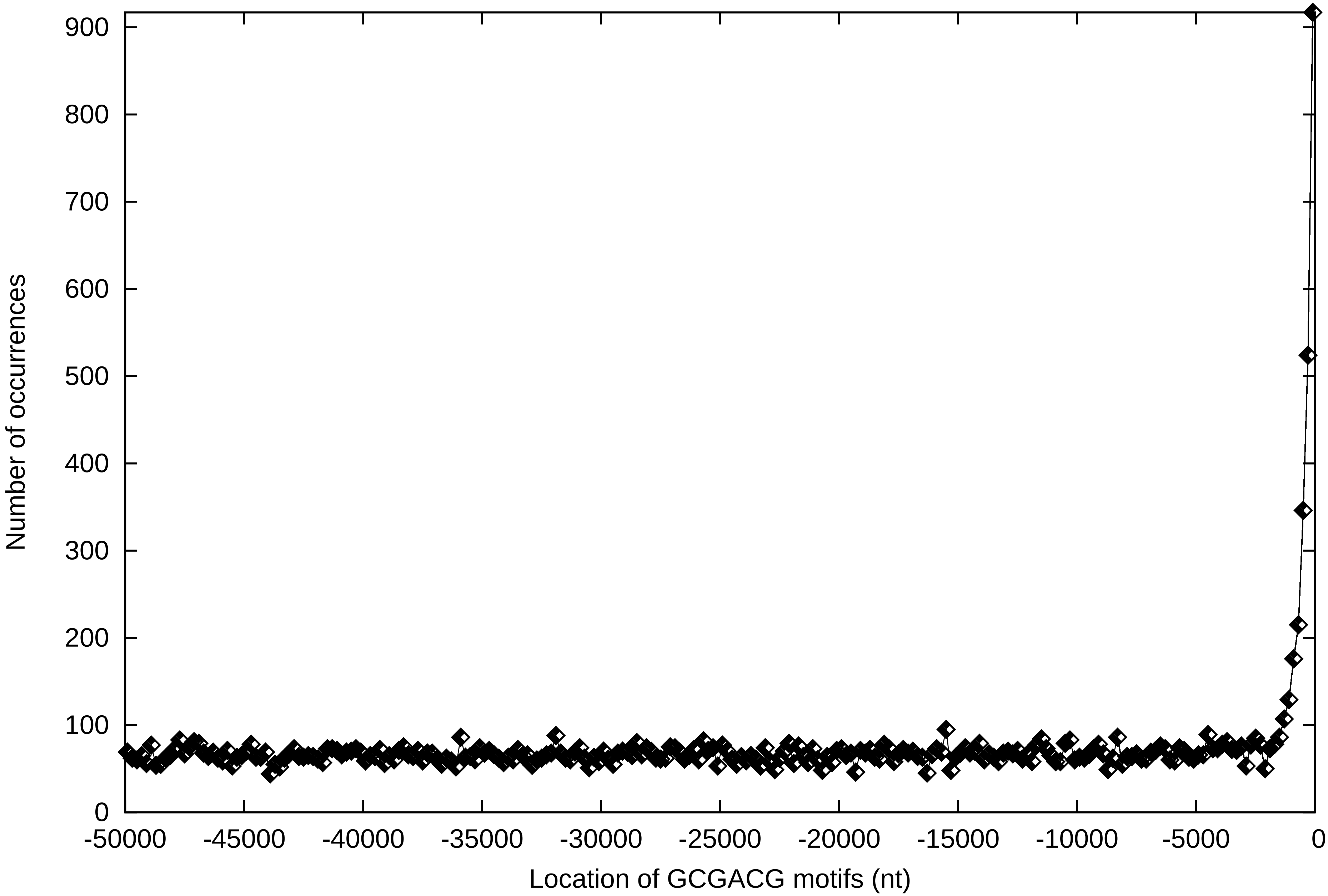

900
800
700
600
500
400
300
200
100
0
-50000
-45000
-40000
-35000
-30000
-25000
-20000
-15000
-10000
-5000
0
Number of occurrences
Location of GCGACG motifs (nt)

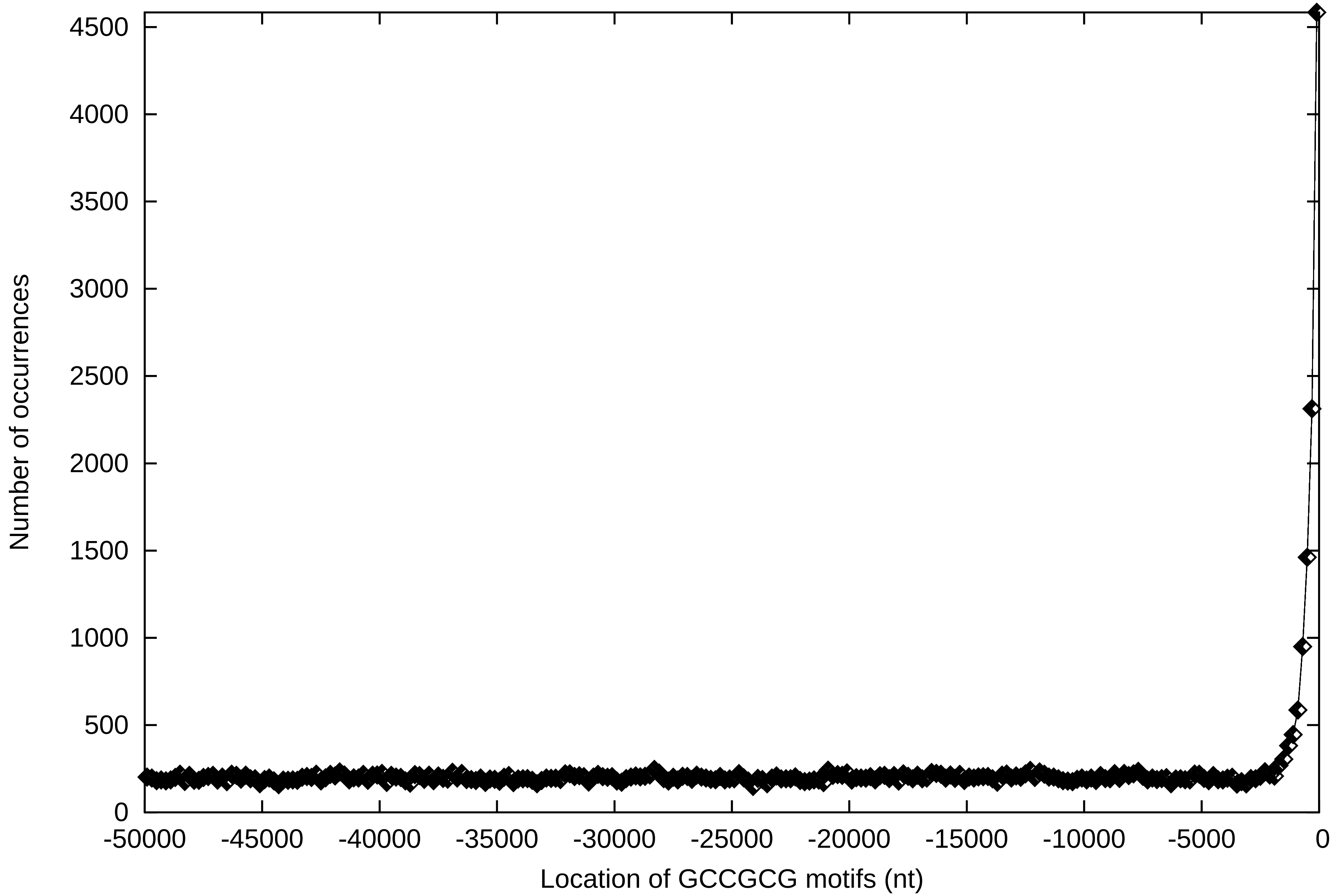
Number of occurrences
Location of GCCGCG motifs (nt)
4500
4000
3500
3000
2500
2000
1500
1000
500
0
-50000
-45000
-40000
-35000
-30000
-25000
-20000
-15000
-10000
-5000
0

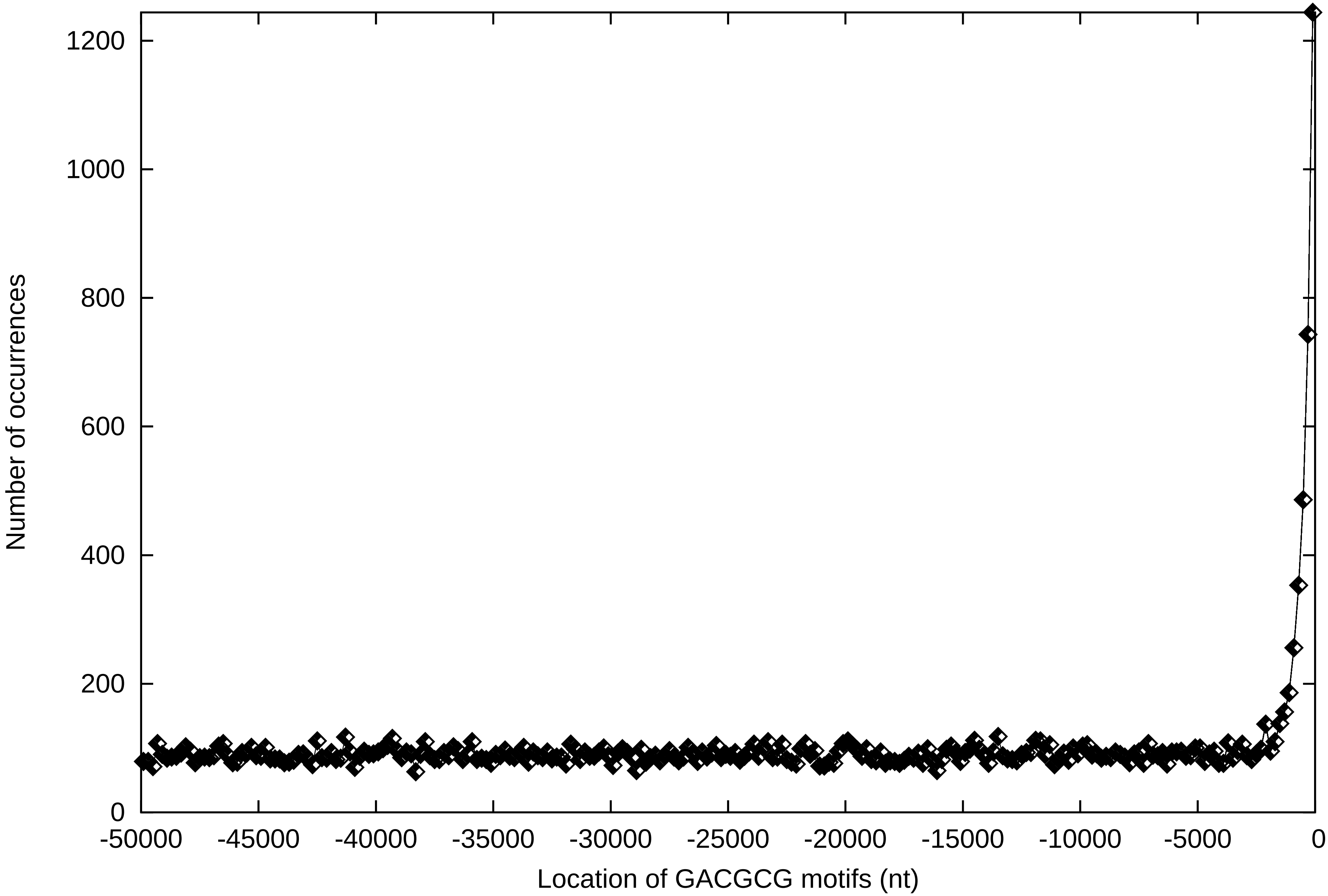

Number of occurrences
0
200
400
600
800
1000
1200
-50000
-45000
-40000
-35000
-30000
-25000
-20000
-15000
-10000
-5000
0
Location of GACGCG motifs (nt)

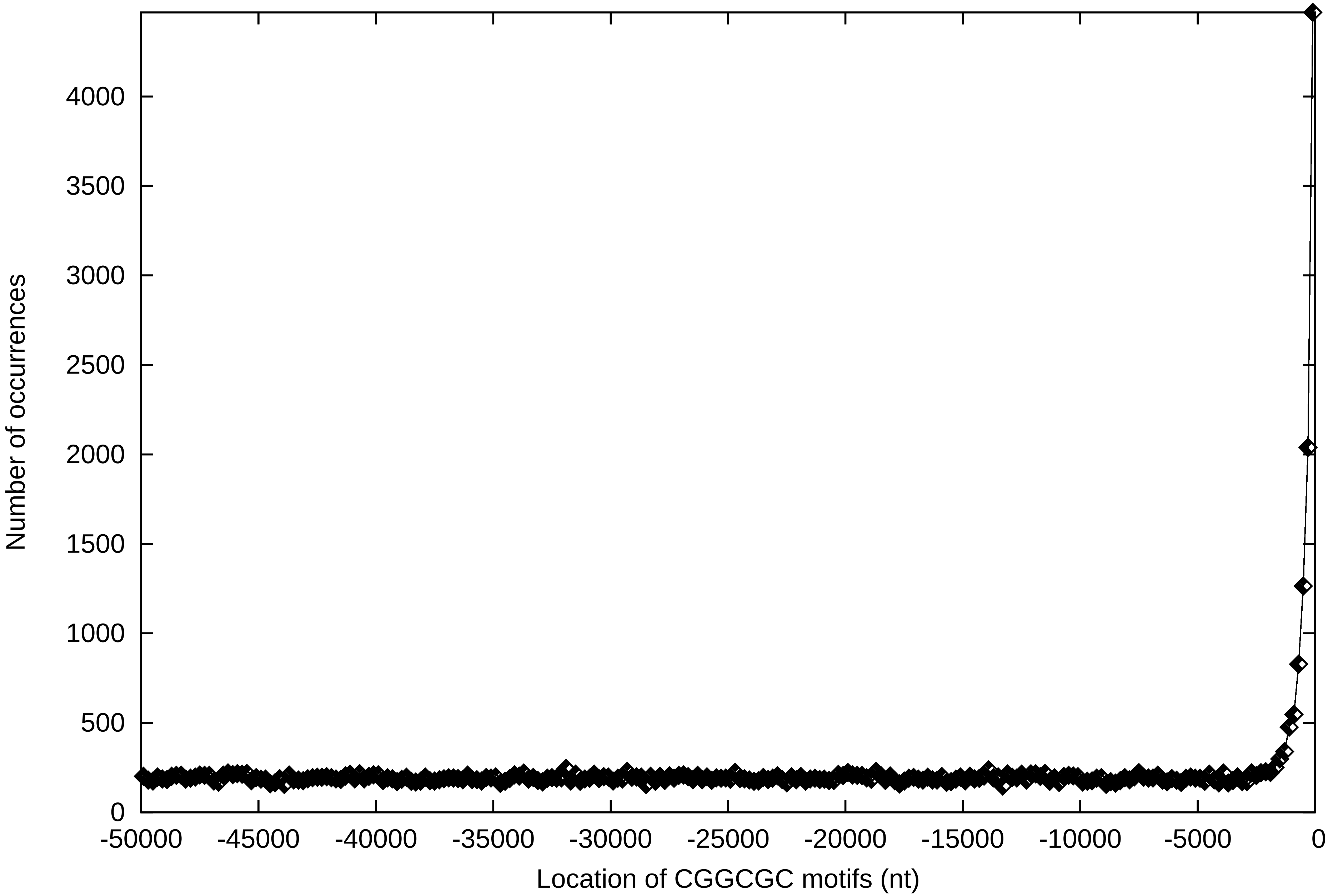

4000
3500
3000
2500
2000
1500
1000
500
0
-50000
-45000
-40000
-35000
-30000
-25000
-20000
-15000
-10000
-5000
0
Number of occurrences
Location of CGGCGC motifs (nt)

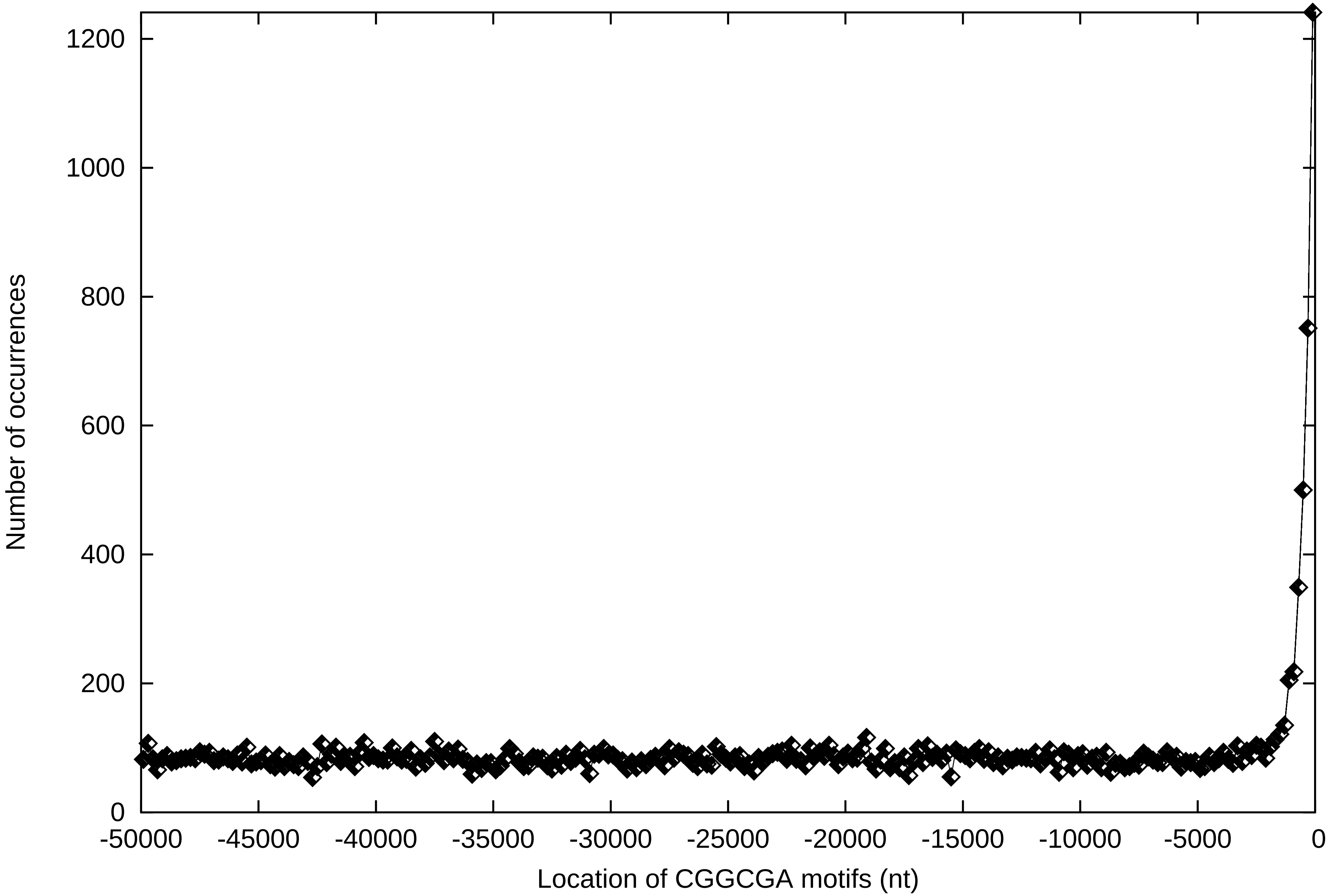

Number of occurrences
1200
1000
800
600
400
200
0
-50000
-45000
-40000
-35000
-30000
-25000
-20000
-15000
-10000
-5000
0
Location of CGGCGA motifs (nt)

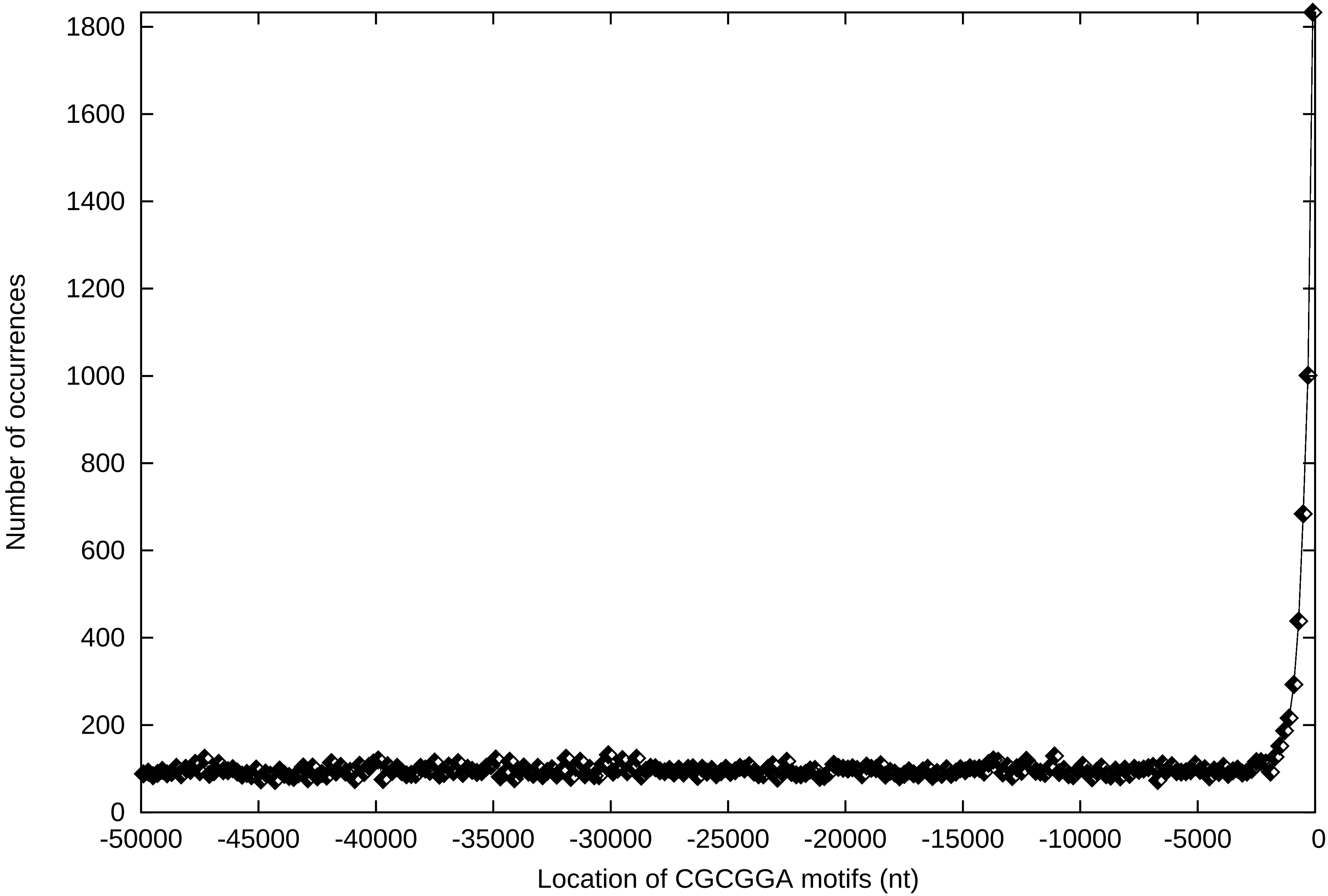

Number of occurrences
1800
1600
1400
1200
1000
800
600
400
200
0
-50000
-45000
-40000
-35000
-30000
-25000
-20000
-15000
-10000
-5000
0
Location of CGCGGA motifs (nt)

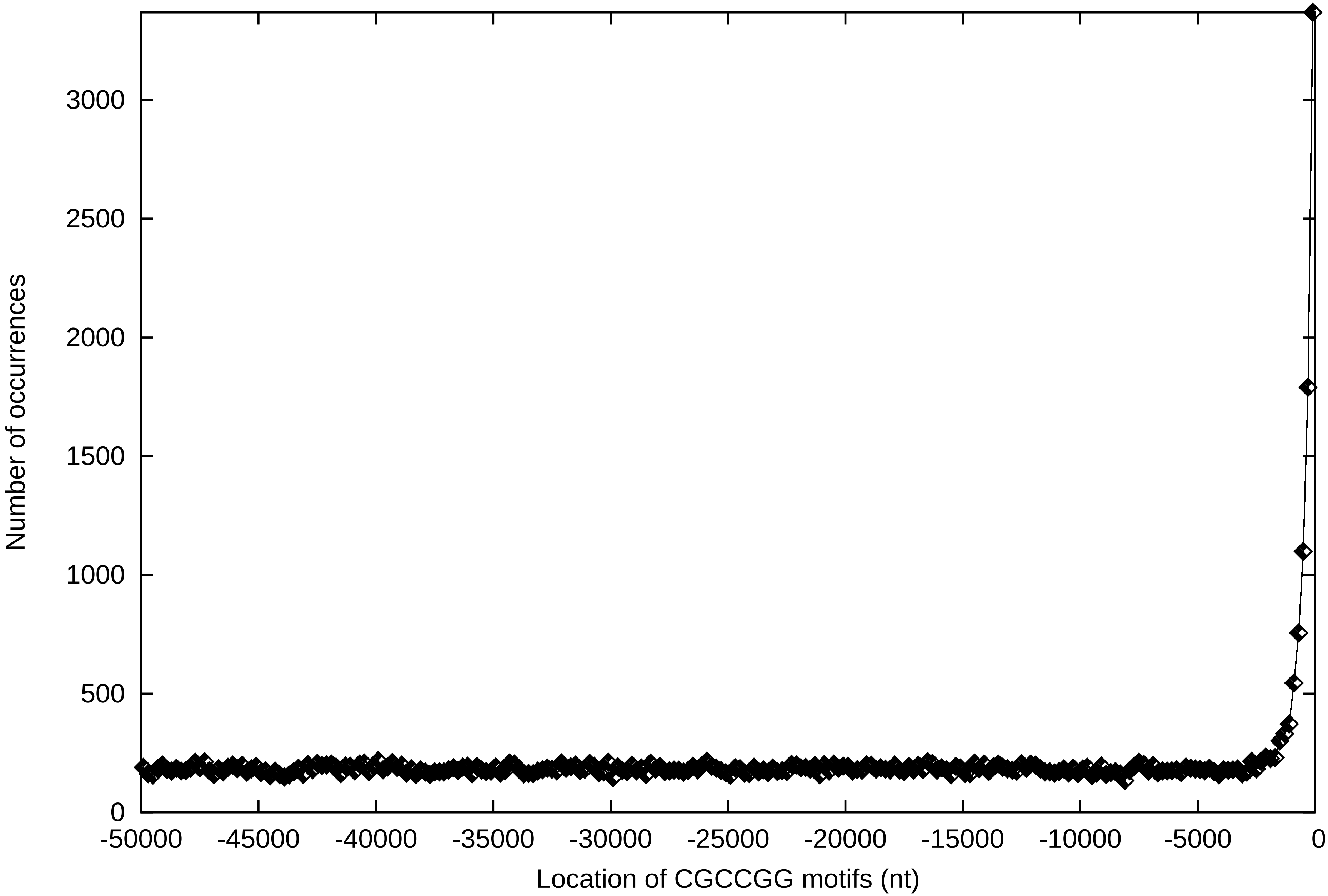

Number of occurrences
0
500
1000
1500
2000
2500
3000
-50000
-45000
-40000
-35000
-30000
-25000
-20000
-15000
-10000
-5000
0
Location of CGCCGG motifs (nt)

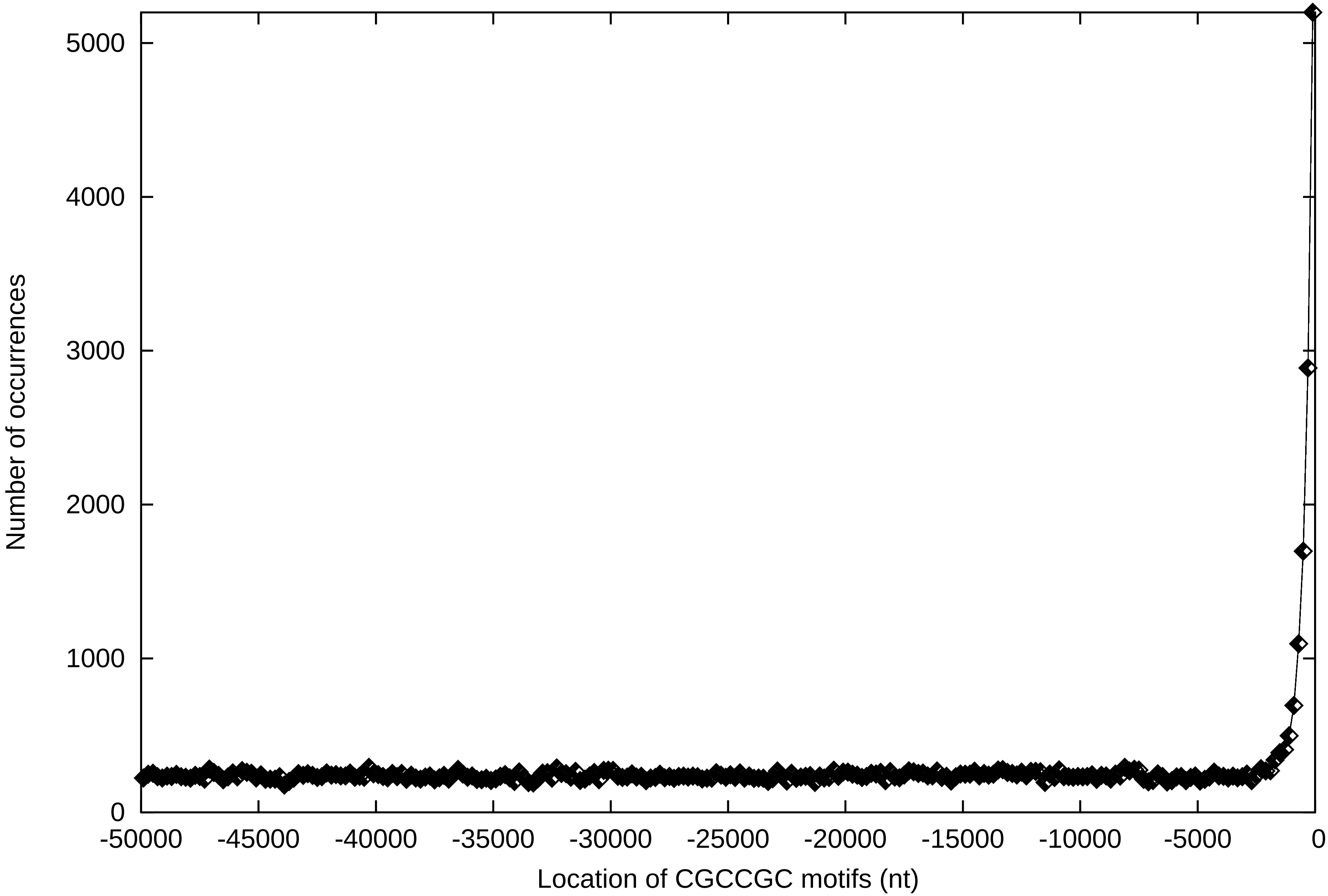

Number of occurrences
5000
4000
3000
2000
1000
0
-50000
-45000
-40000
-35000
-30000
-25000
-20000
-15000
-10000
-5000
0
Location of CGCCGC motifs (nt)

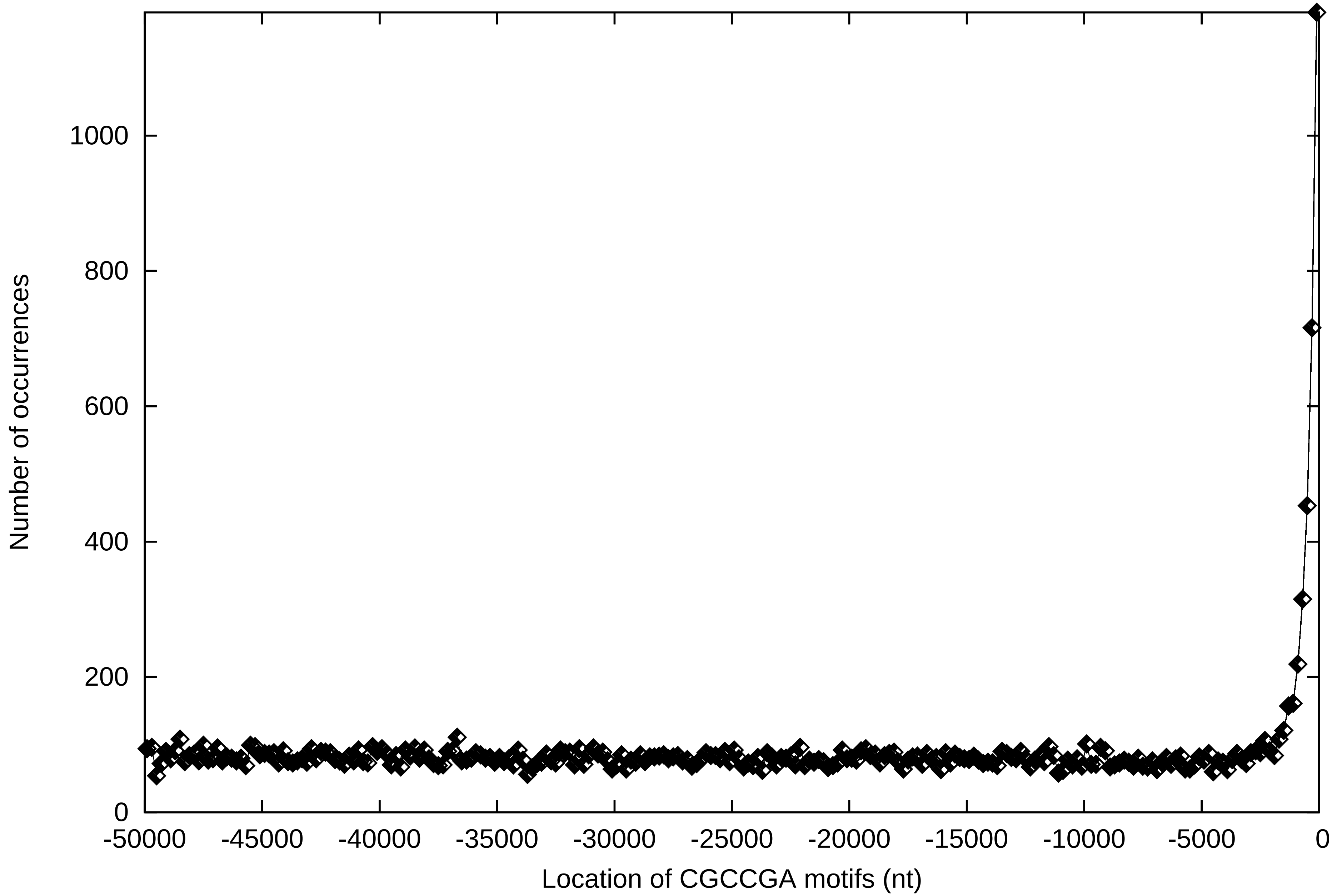
Number of occurrences
0
200
400
600
800
1000
-50000
-45000
-40000
-35000
-30000
-25000
-20000
-15000
-10000
-5000
0
Location of CGCCGA motifs (nt)

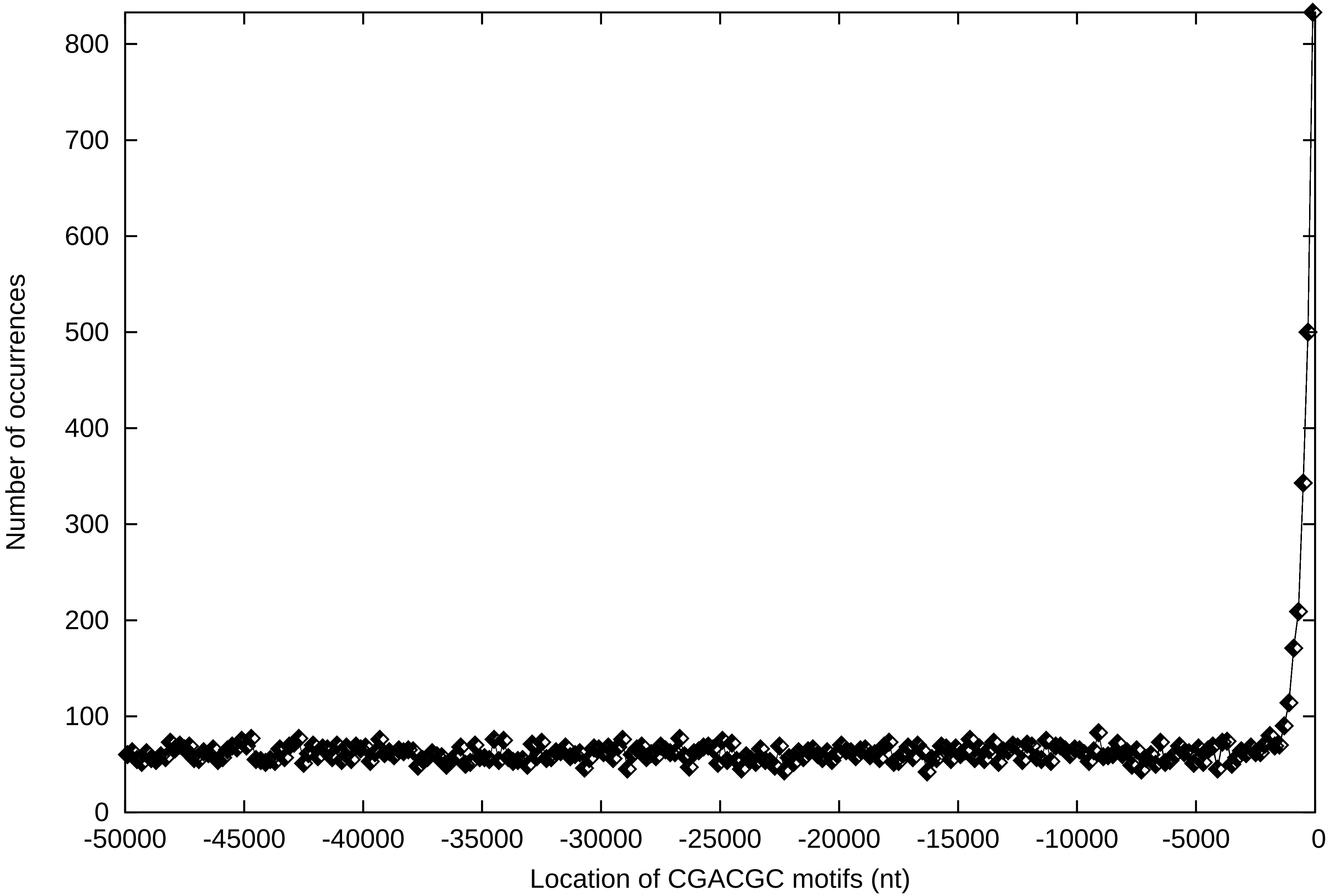

Number of occurrences
800
700
600
500
400
300
200
100
0
-50000
-45000
-40000
-35000
-30000
-25000
-20000
-15000
-10000
-5000
0
Location of CGACGC motifs (nt)

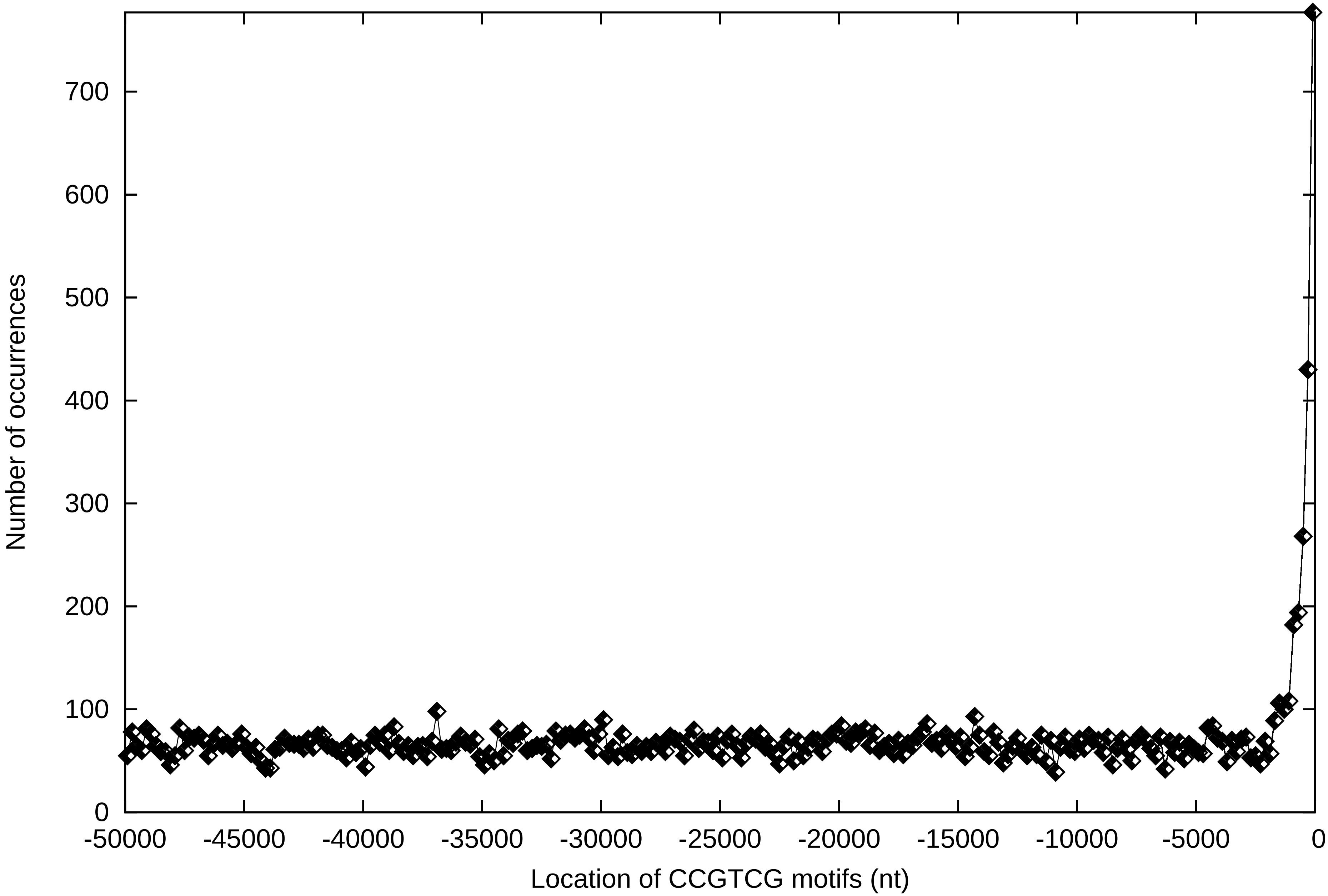

Number of occurrences
0
100
200
300
400
500
600
700
-50000
-45000
-40000
-35000
-30000
-25000
-20000
-15000
-10000
-5000
0
Location of CCGTCG motifs (nt)

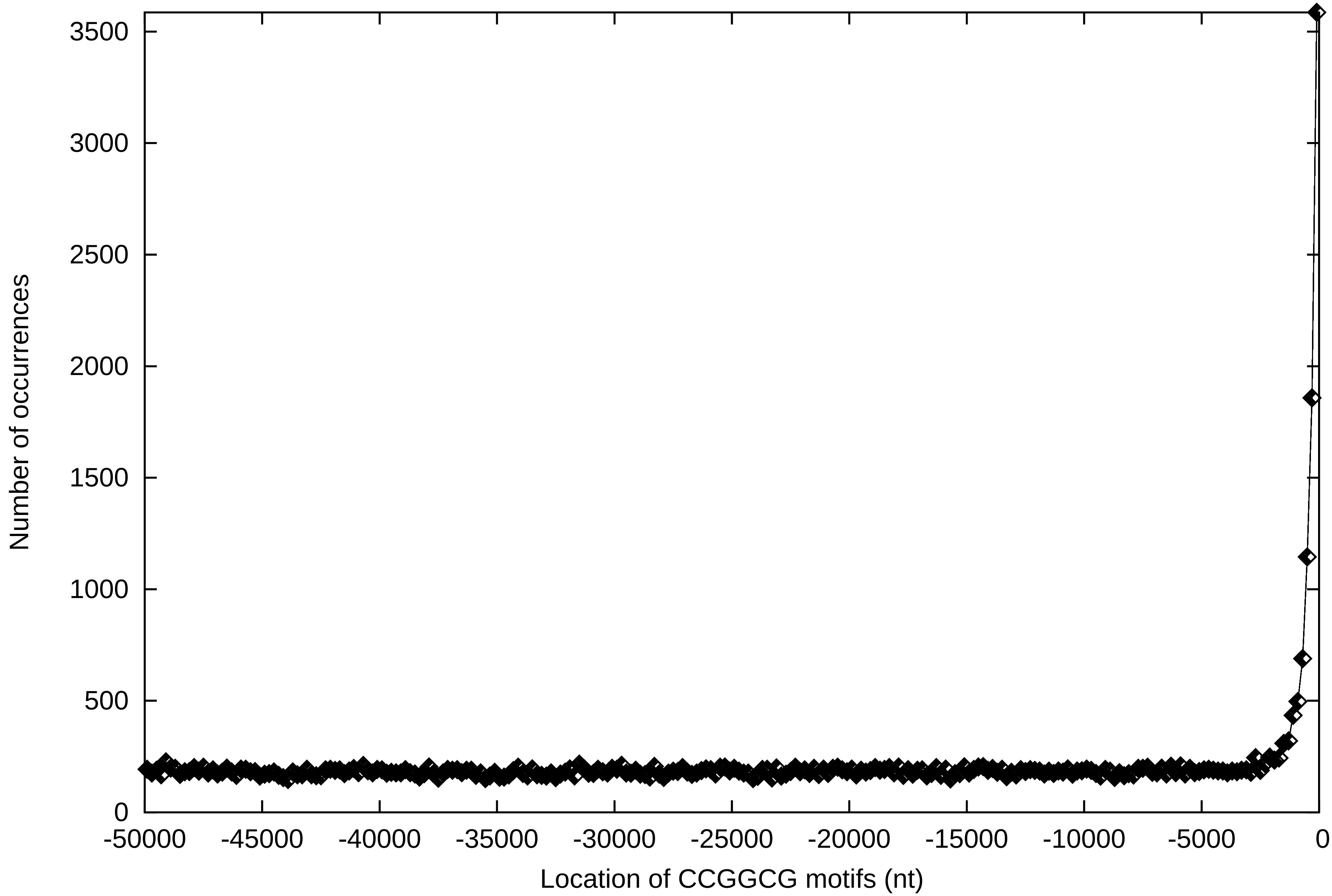

3500
3000
2500
2000
1500
1000
500
0
-50000
-45000
-40000
-35000
-30000
-25000
-20000
-15000
-10000
-5000
0
Number of occurrences
Location of CCGGCG motifs (nt)

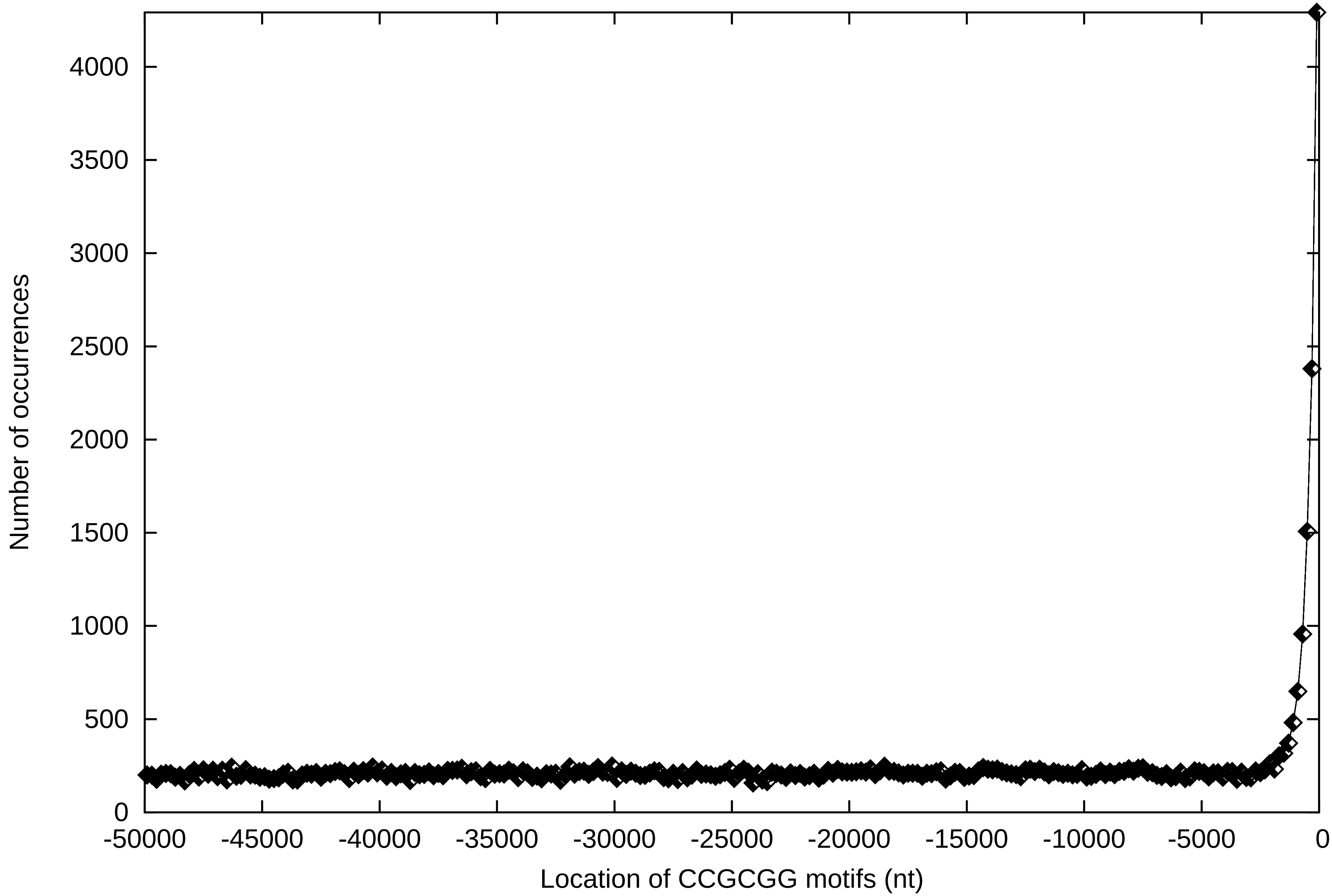

Number of occurrences
0
500
1000
1500
2000
2500
3000
3500
4000
-50000
-45000
-40000
-35000
-30000
-25000
-20000
-15000
-10000
-5000
0
Location of CCGCGG motifs (nt)

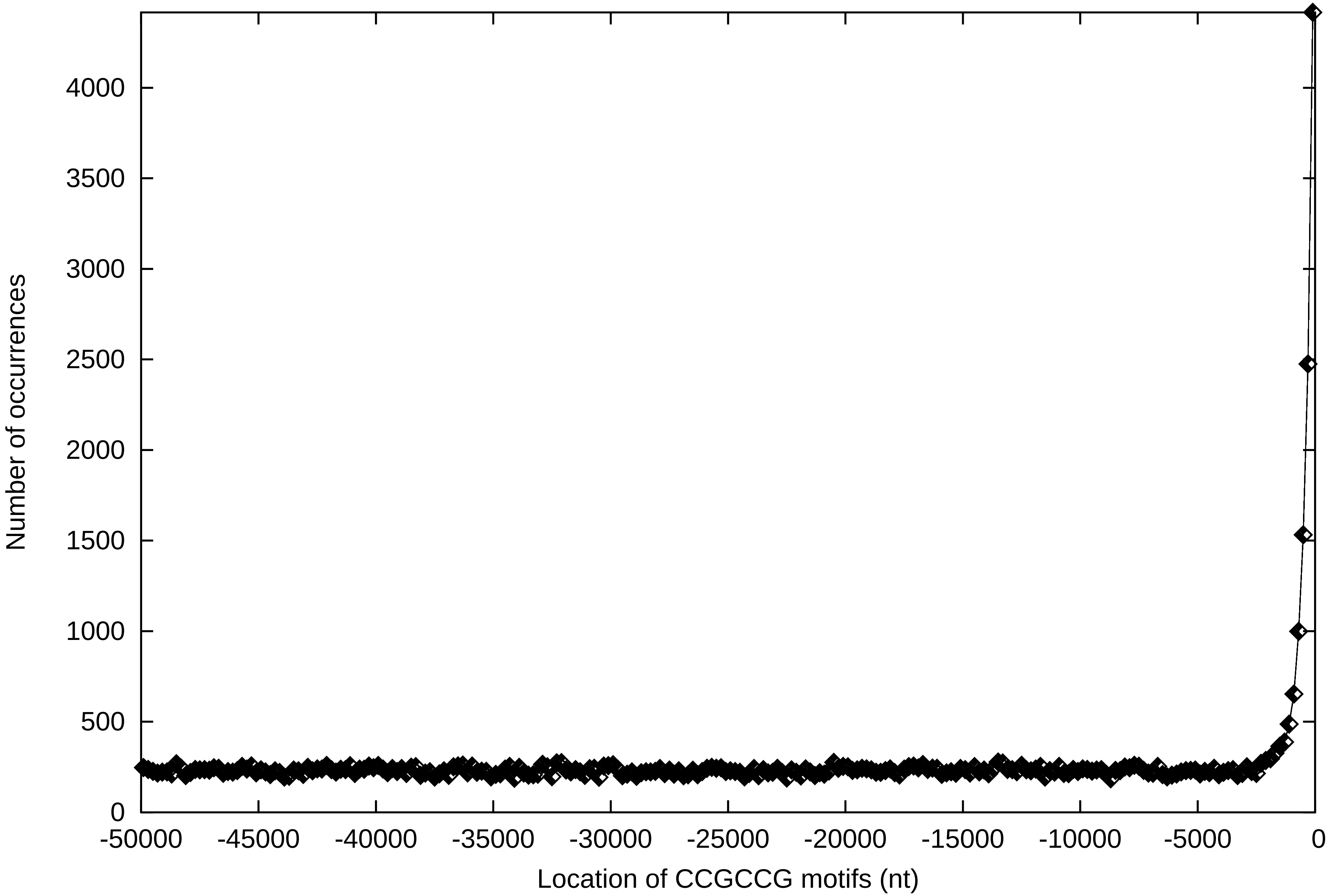

4000
3500
3000
2500
2000
1500
1000
500
0
-50000
-45000
-40000
-35000
-30000
-25000
-20000
-15000
-10000
-5000
0
Number of occurrences
Location of CCGCCG motifs (nt)

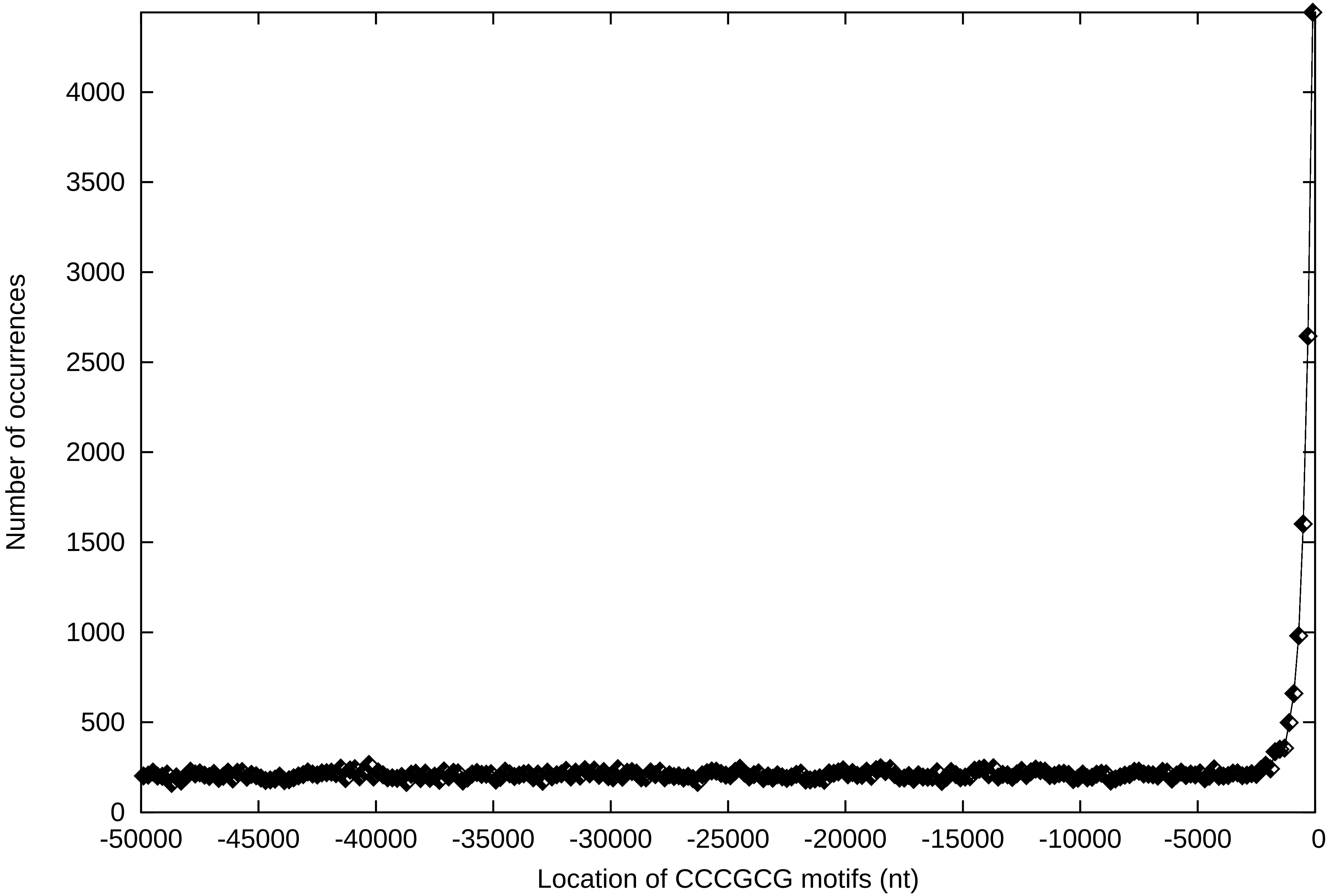

Number of occurrences
0
500
1000
1500
2000
2500
3000
3500
4000
-50000
-45000
-40000
-35000
-30000
-25000
-20000
-15000
-10000
-5000
0
Location of CCCGCG motifs (nt)

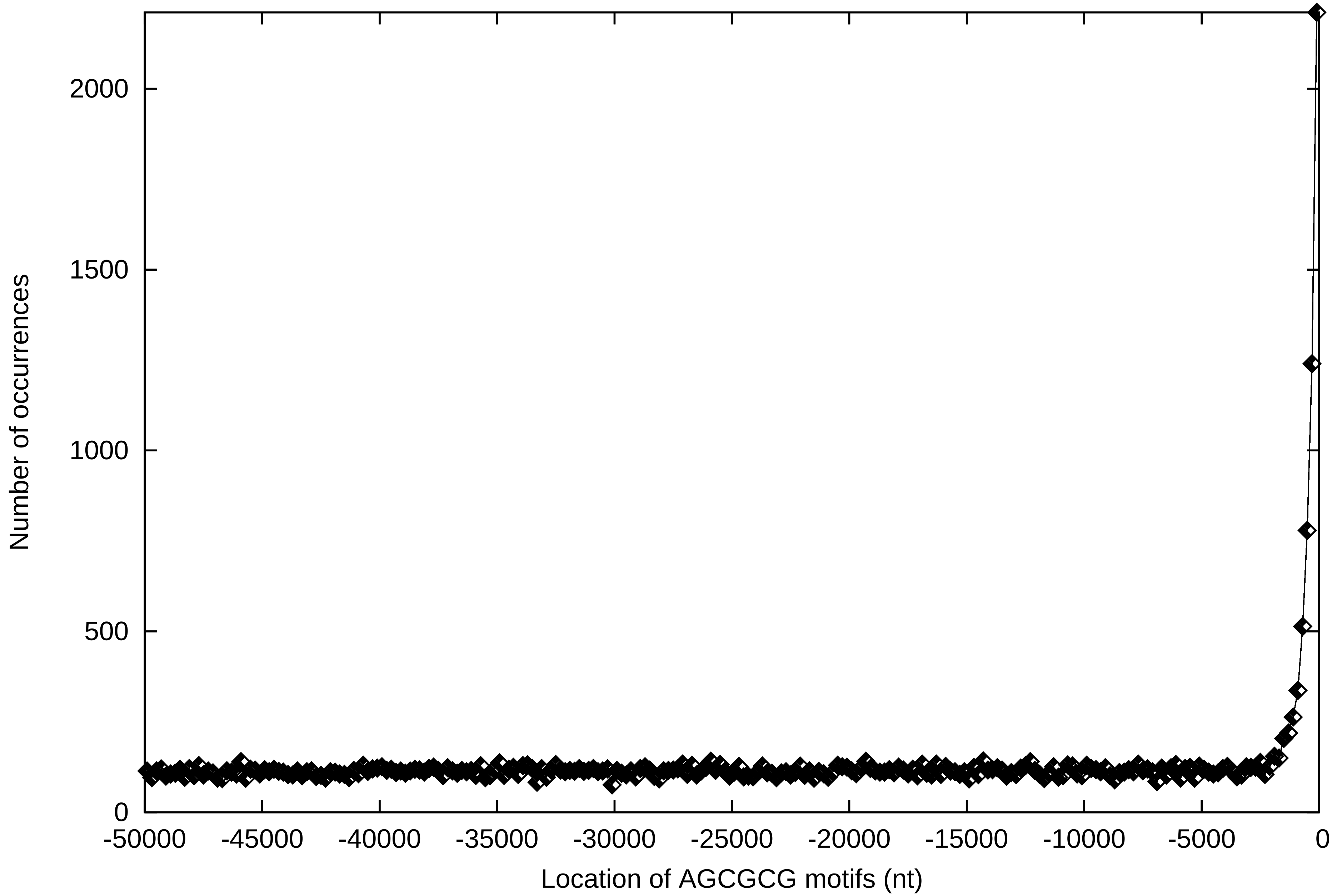

Number of occurrences
0
500
1000
1500
2000
-50000
-45000
-40000
-35000
-30000
-25000
-20000
-15000
-10000
-5000
0
Location of AGCGCG motifs (nt)

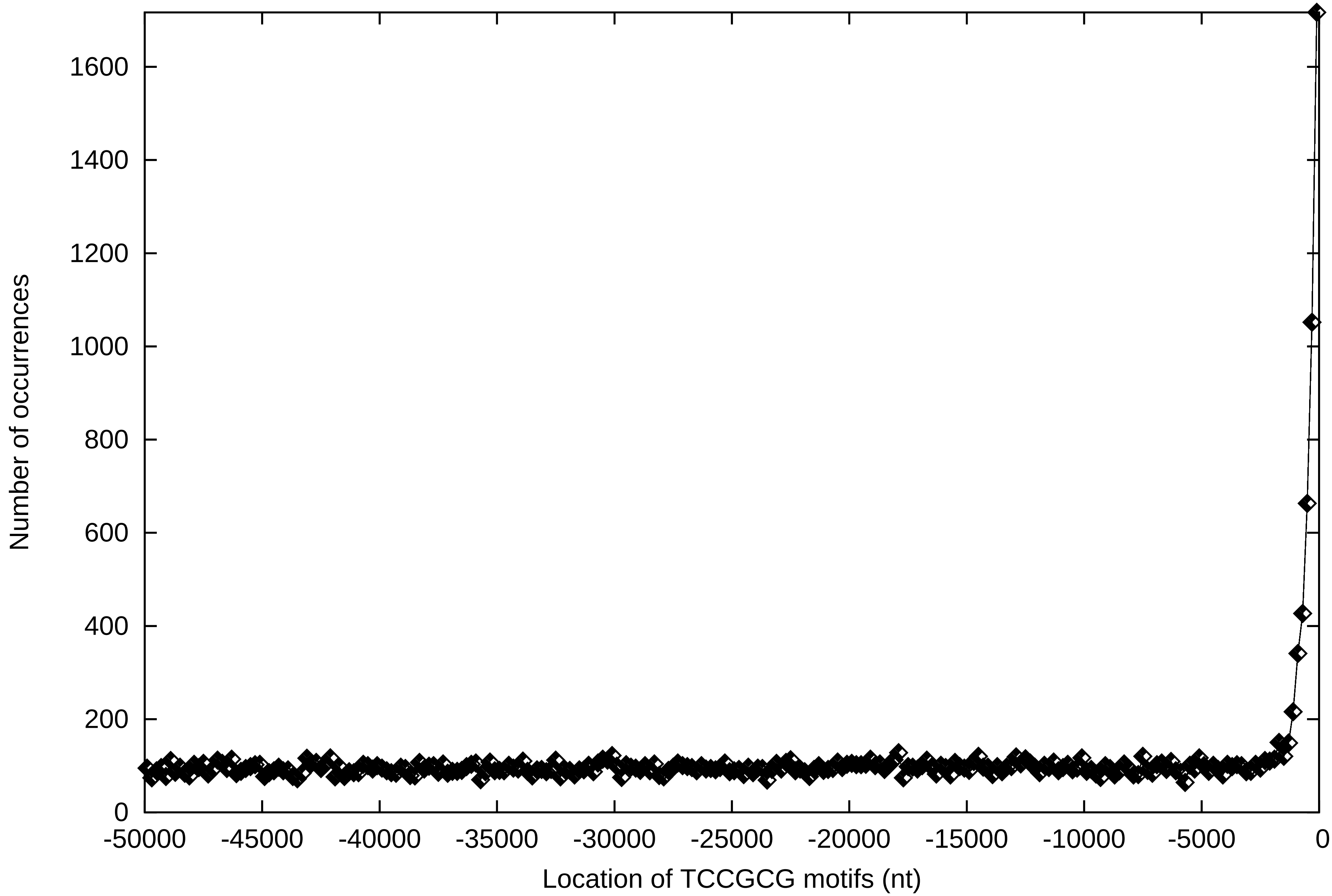

Number of occurrences
0
200
400
600
800
1000
1200
1400
1600
-50000
-45000
-40000
-35000
-30000
-25000
-20000
-15000
-10000
-5000
0
Location of TCCGCG motifs (nt)

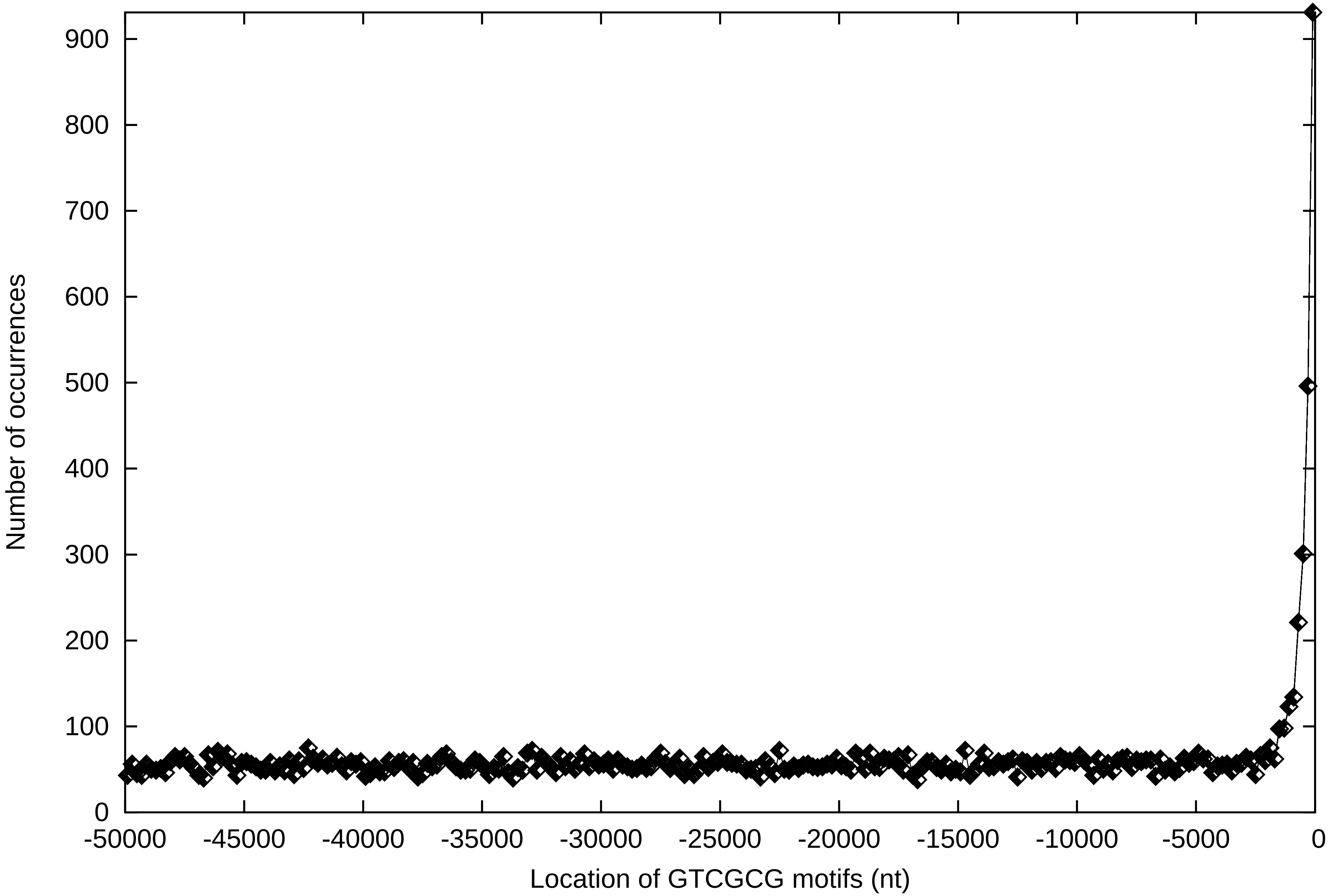

Number of occurrences
0
100
200
300
400
500
600
700
800
900
-50000
-45000
-40000
-35000
-30000
-25000
-20000
-15000
-10000
-5000
0
Location of GTCGCG motifs (nt)

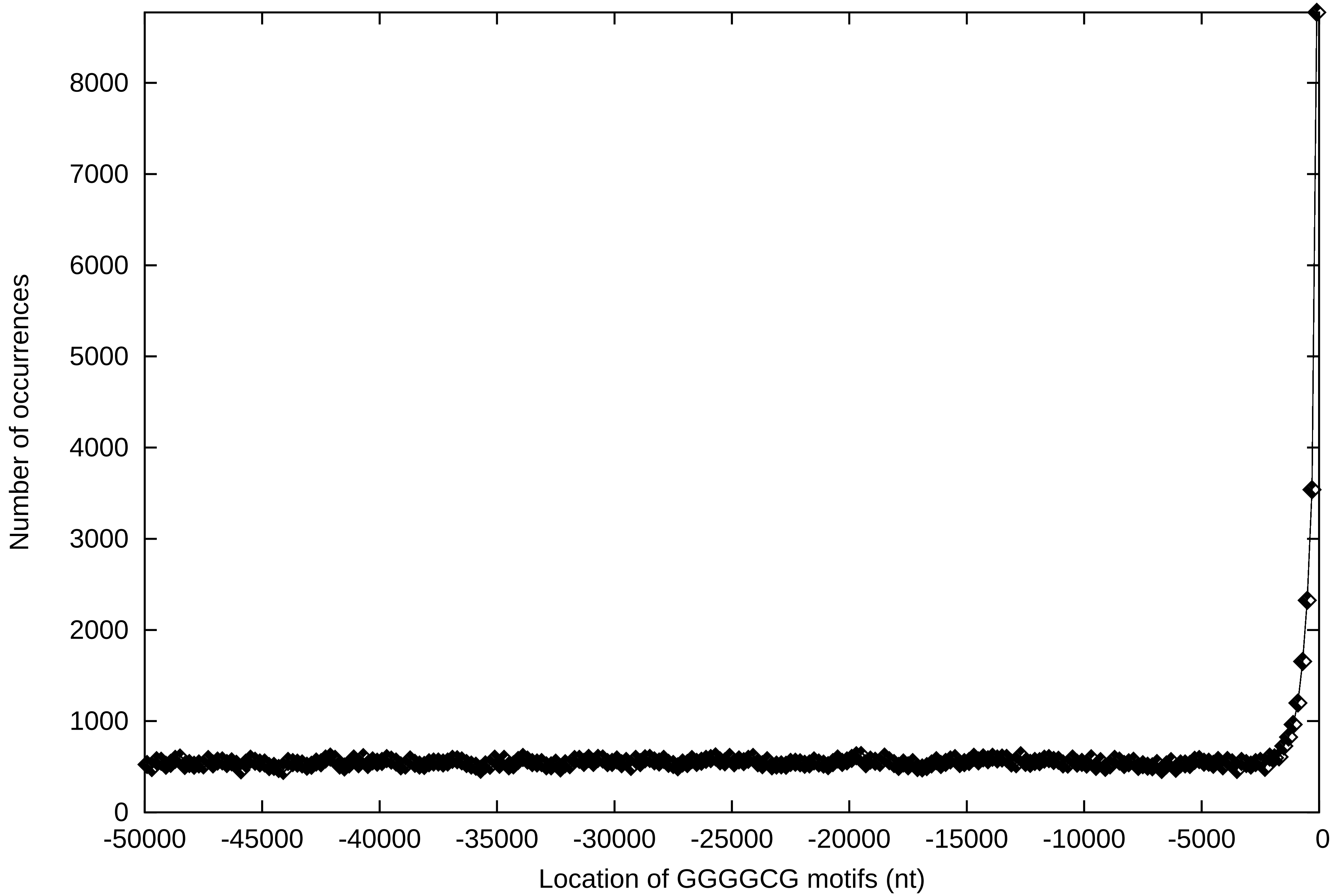

Number of occurrences
0
1000
2000
3000
4000
5000
6000
7000
8000
-50000
-45000
-40000
-35000
-30000
-25000
-20000
-15000
-10000
-5000
0
Location of GGGGCG motifs (nt)

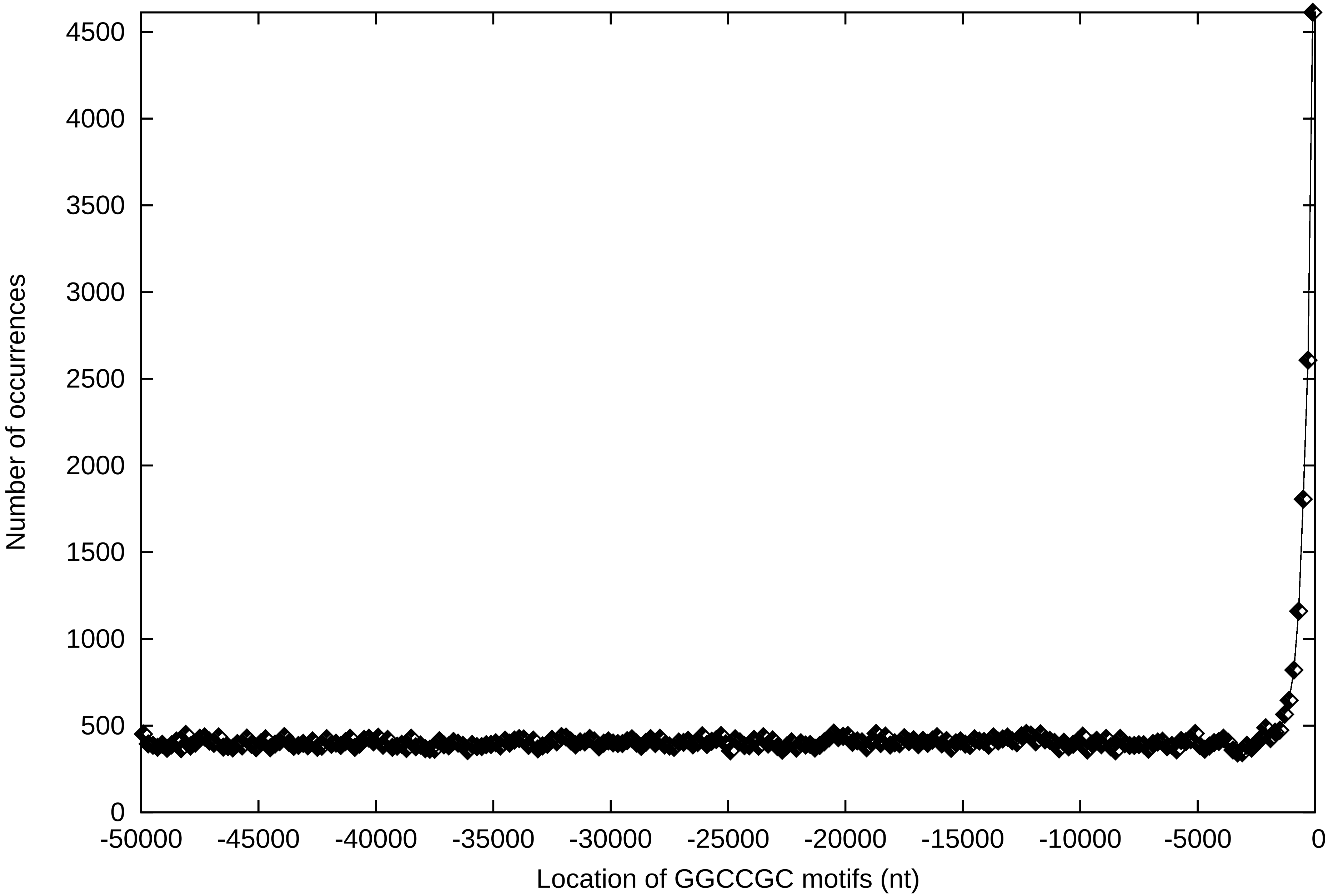

Number of occurrences
Location of GGCCGC motifs (nt)
0
500
1000
1500
2000
2500
3000
3500
4000
4500
-50000
-45000
-40000
-35000
-30000
-25000
-20000
-15000
-10000
-5000
0

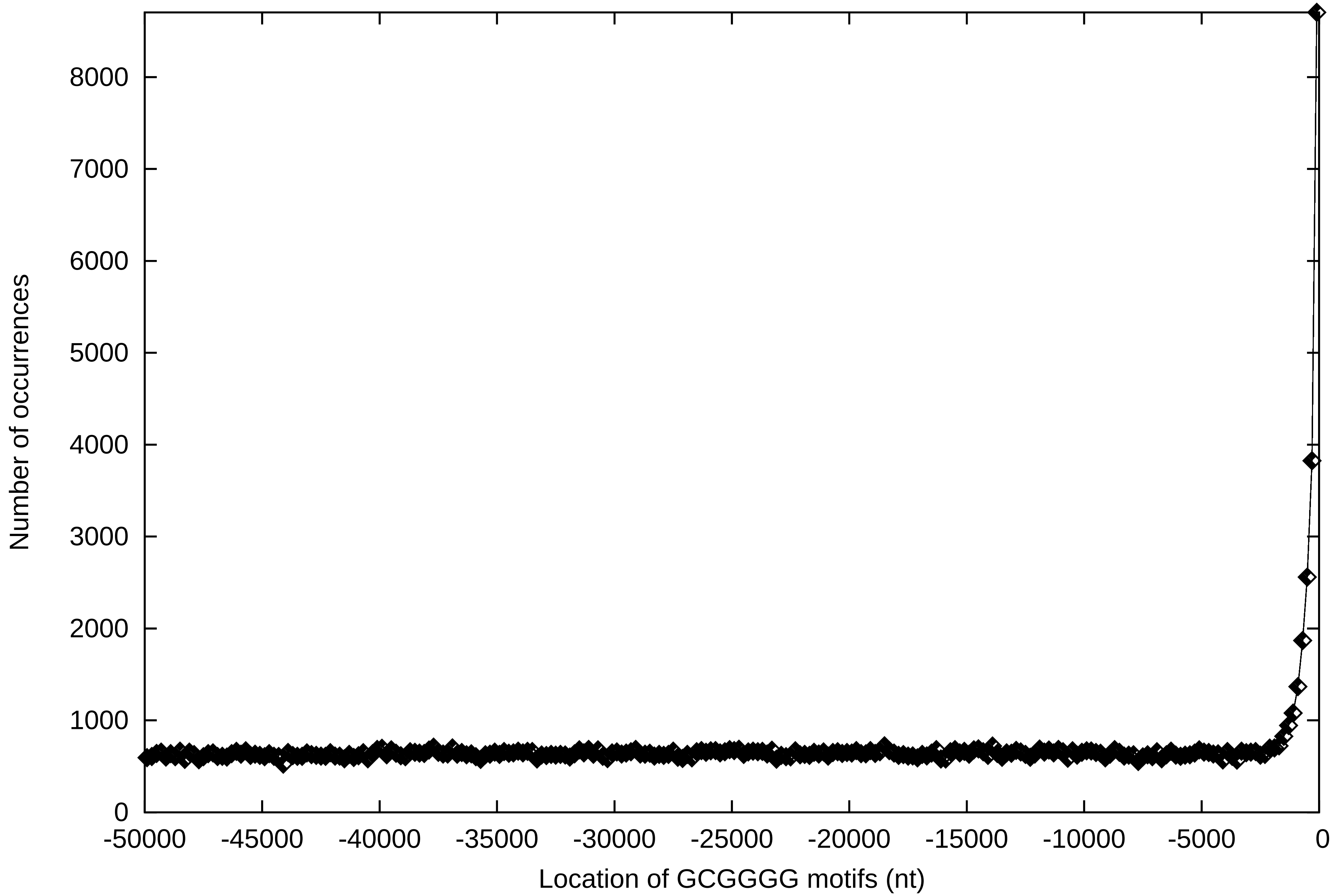
Number of occurrences
Location of GCGGGG motifs (nt)
0
1000
2000
3000
4000
5000
6000
7000
8000
-50000
-45000
-40000
-35000
-30000
-25000
-20000
-15000
-10000
-5000
0

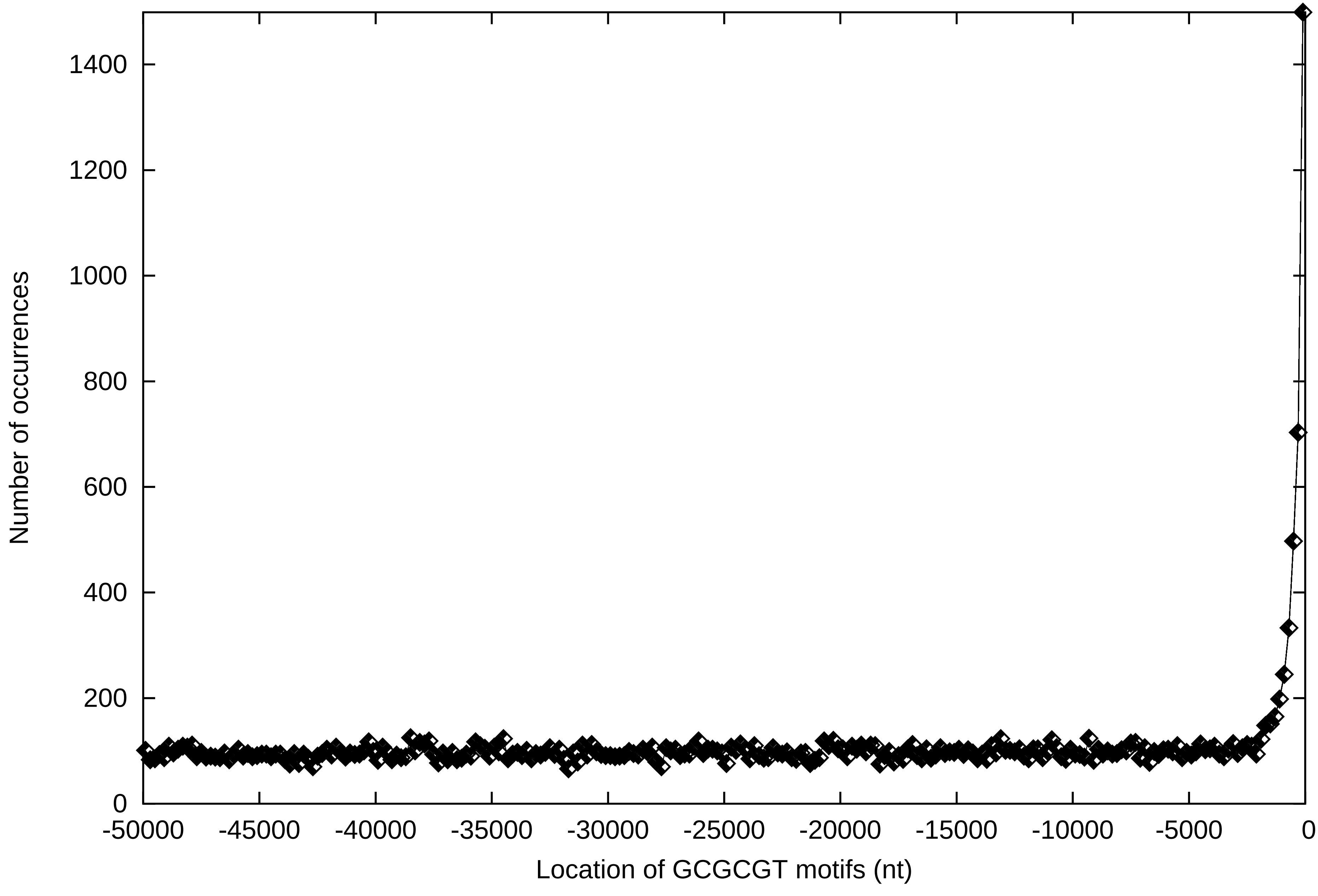
1400
1200
1000
800
600
400
200
0
Number of occurrences
-50000
-45000
-40000
-35000
-30000
-25000
-20000
-15000
-10000
-5000
0
Location of GCGCGT motifs (nt)

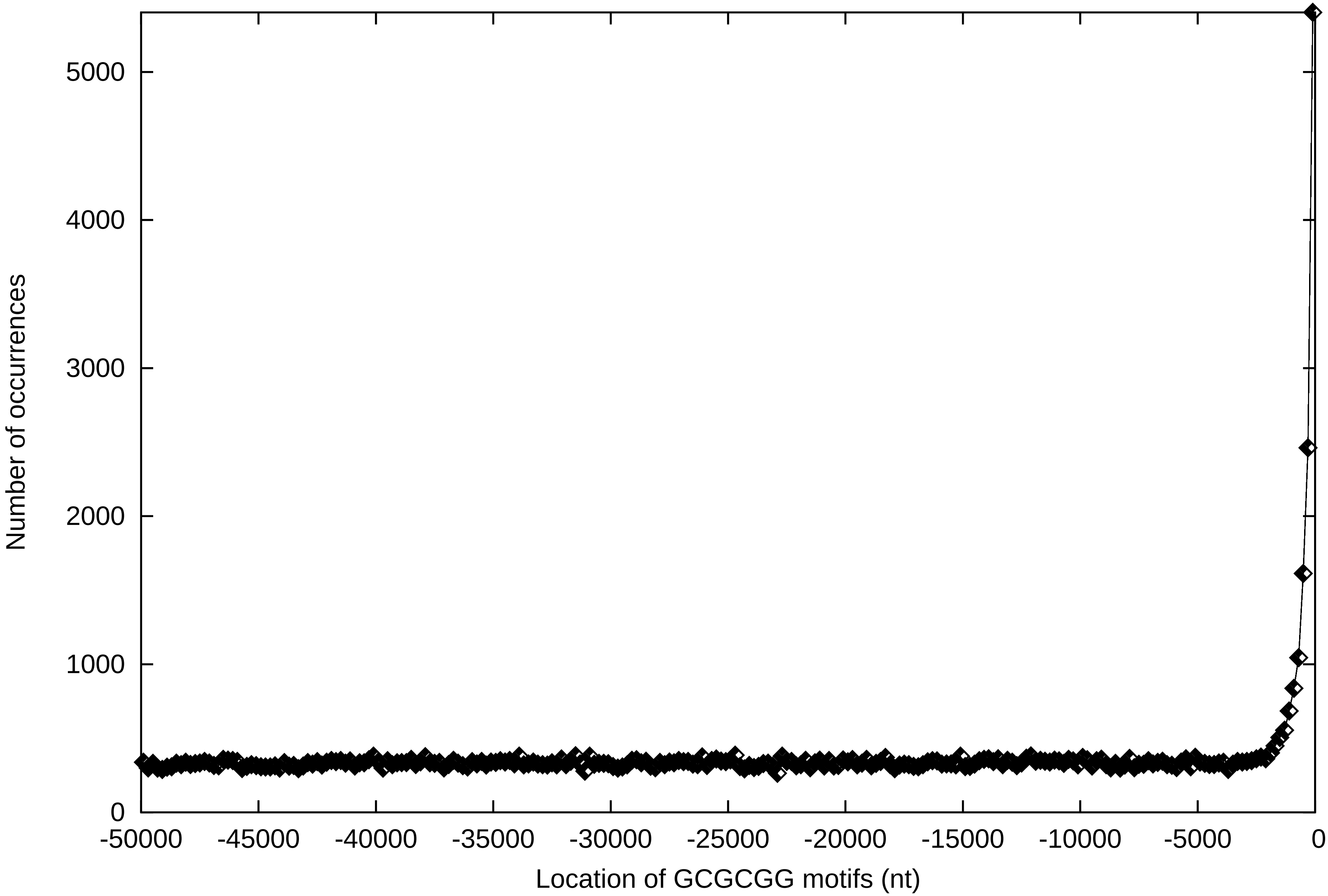
Number of occurrences
5000
4000
3000
2000
1000
0
-50000
-45000
-40000
-35000
-30000
-25000
-20000
-15000
-10000
-5000
0
Location of GCGCGG motifs (nt)

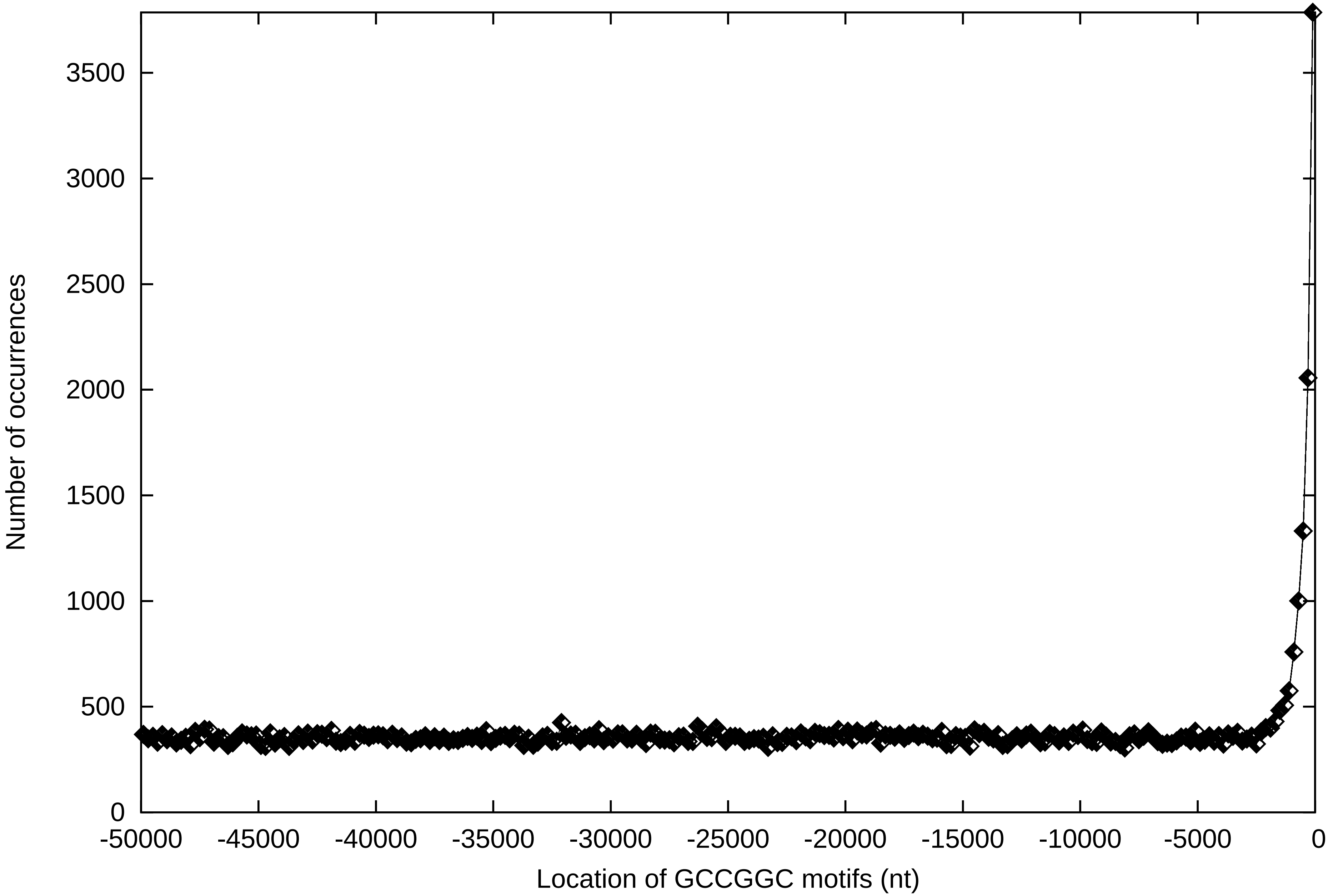

Number of occurrences
3500
3000
2500
2000
1500
1000
500
0
-50000
-45000
-40000
-35000
-30000
-25000
-20000
-15000
-10000
-5000
0
Location of GCCGGC motifs (nt)

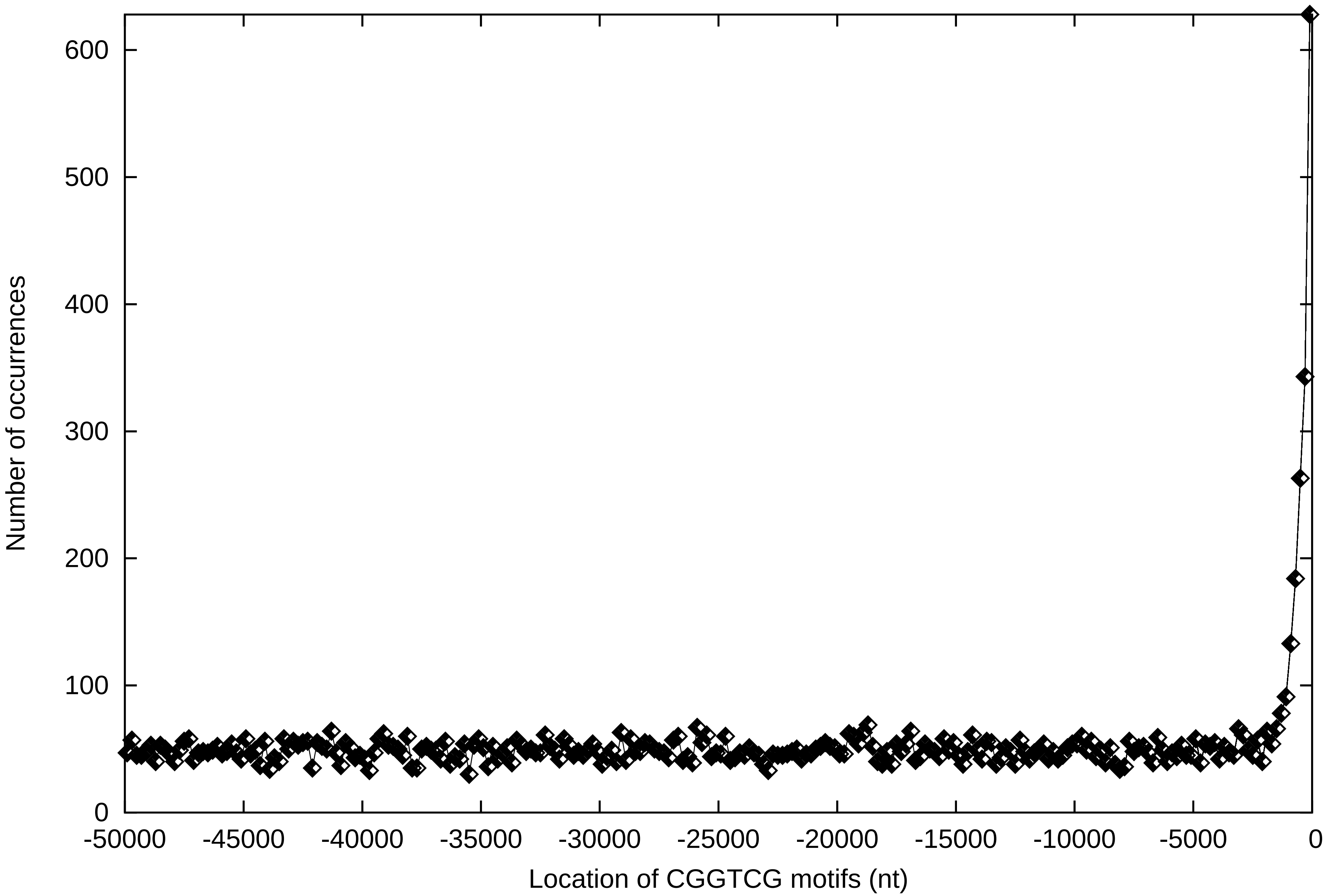

Number of occurrences
Location of CGGTCG motifs (nt)
0
100
200
300
400
500
600
-50000
-45000
-40000
-35000
-30000
-25000
-20000
-15000
-10000
-5000
0

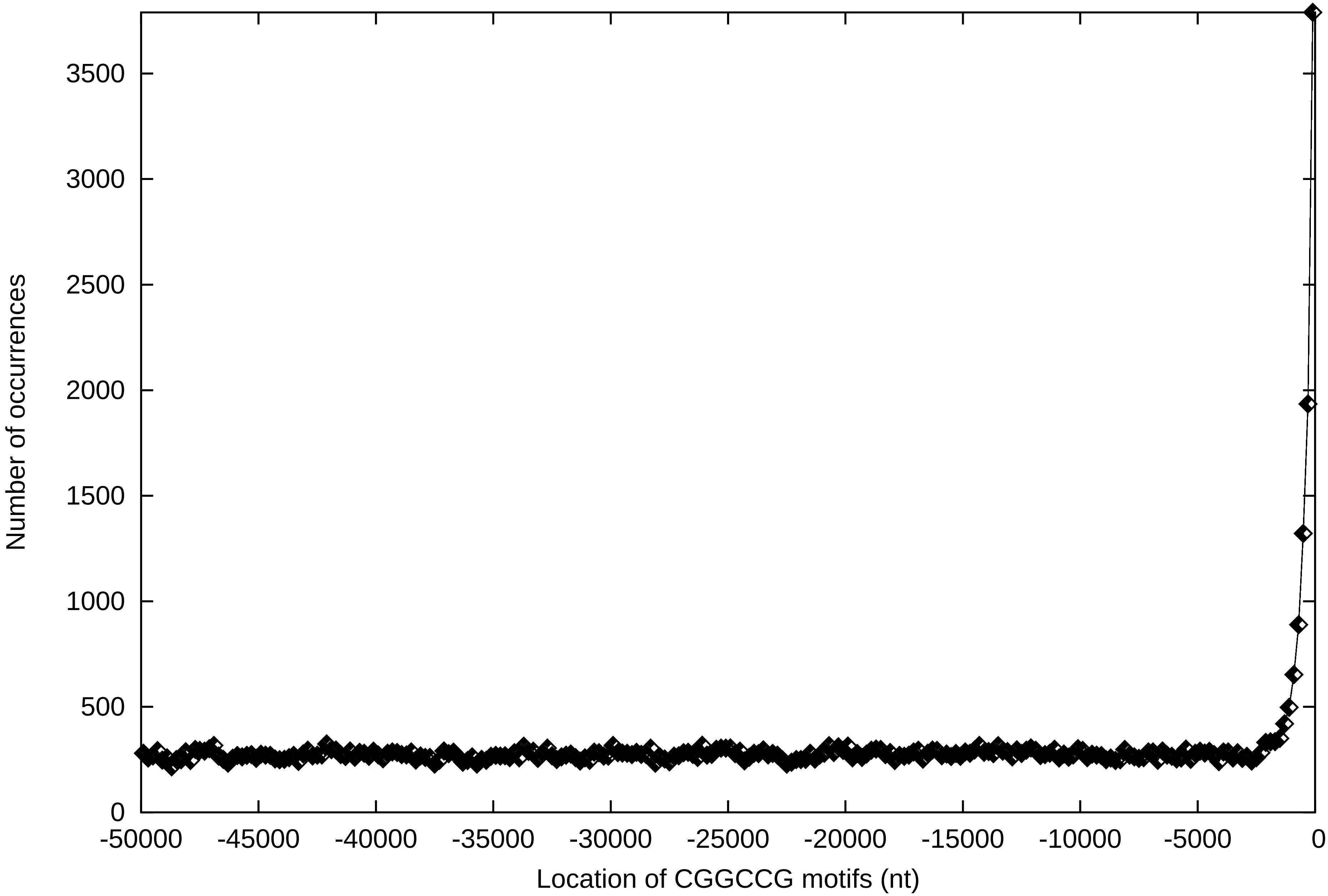

Number of occurrences
0
500
1000
1500
2000
2500
3000
3500
-50000
-45000
-40000
-35000
-30000
-25000
-20000
-15000
-10000
-5000
0
Location of CGGCCG motifs (nt)

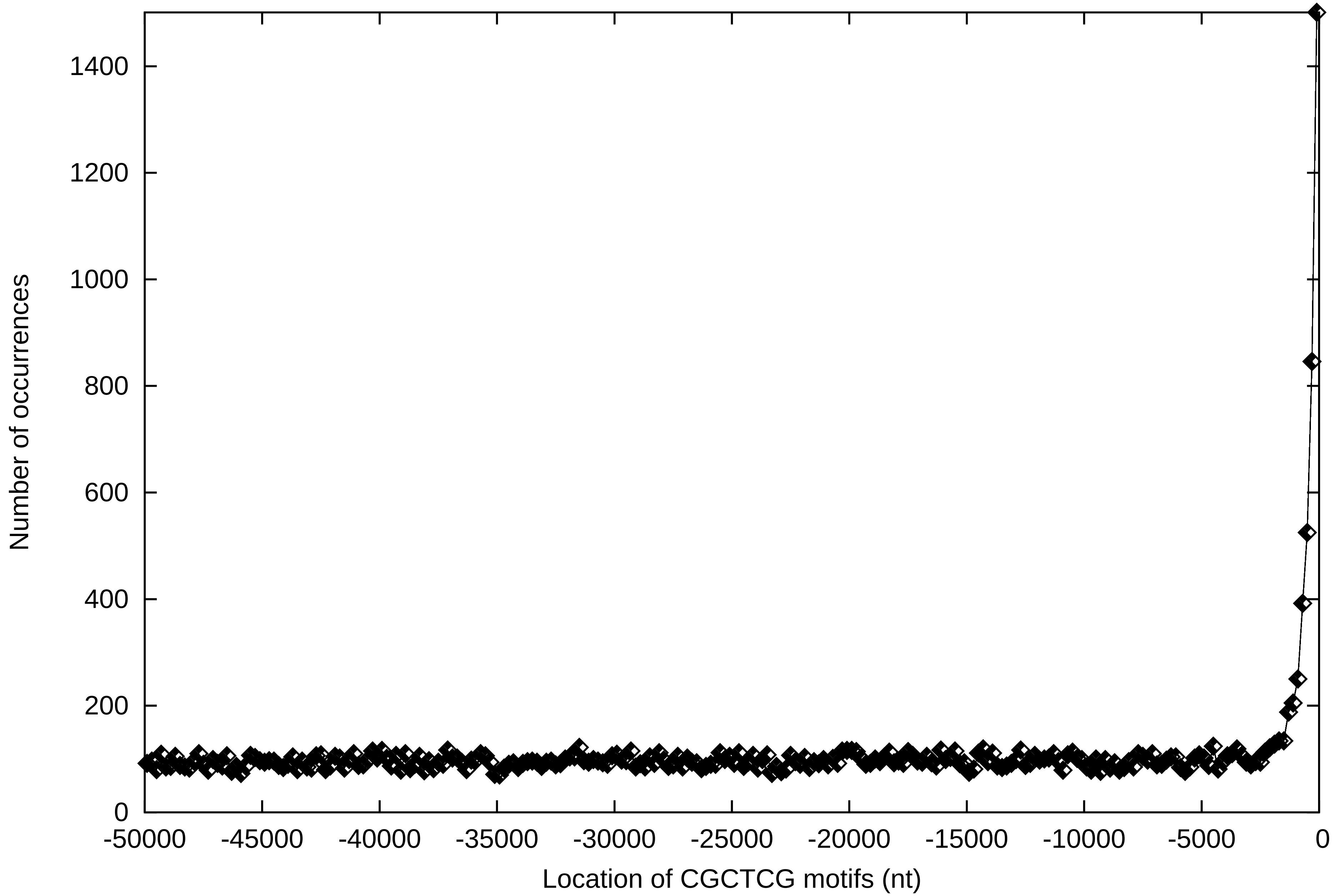

Number of occurrences
0
200
400
600
800
1000
1200
1400
-50000
-45000
-40000
-35000
-30000
-25000
-20000
-15000
-10000
-5000
0
Location of CGCTCG motifs (nt)

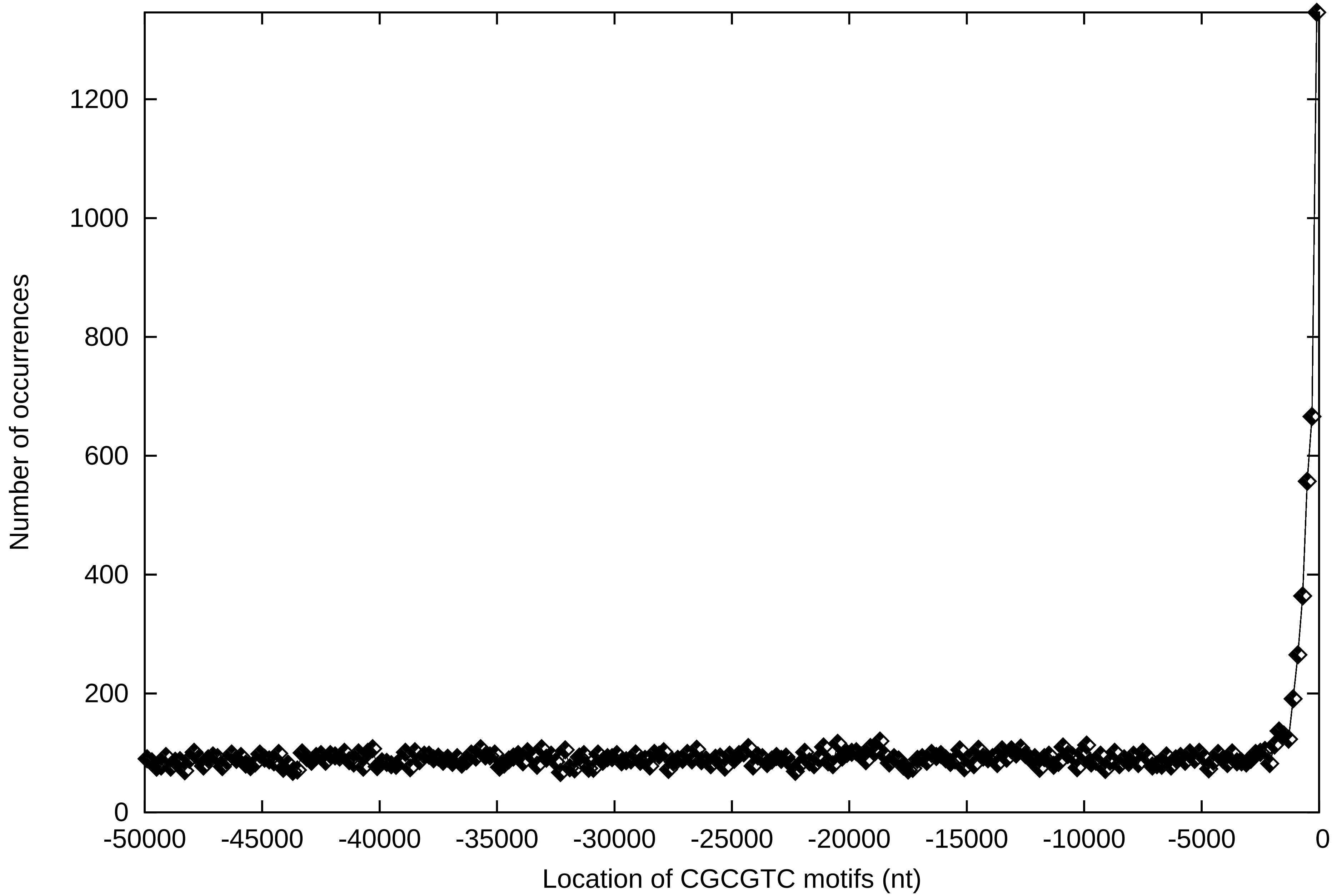

0
200
400
600
800
1000
1200
Number of occurrences
-50000
-45000
-40000
-35000
-30000
-25000
-20000
-15000
-10000
-5000
0
Location of CGCGTC motifs (nt)

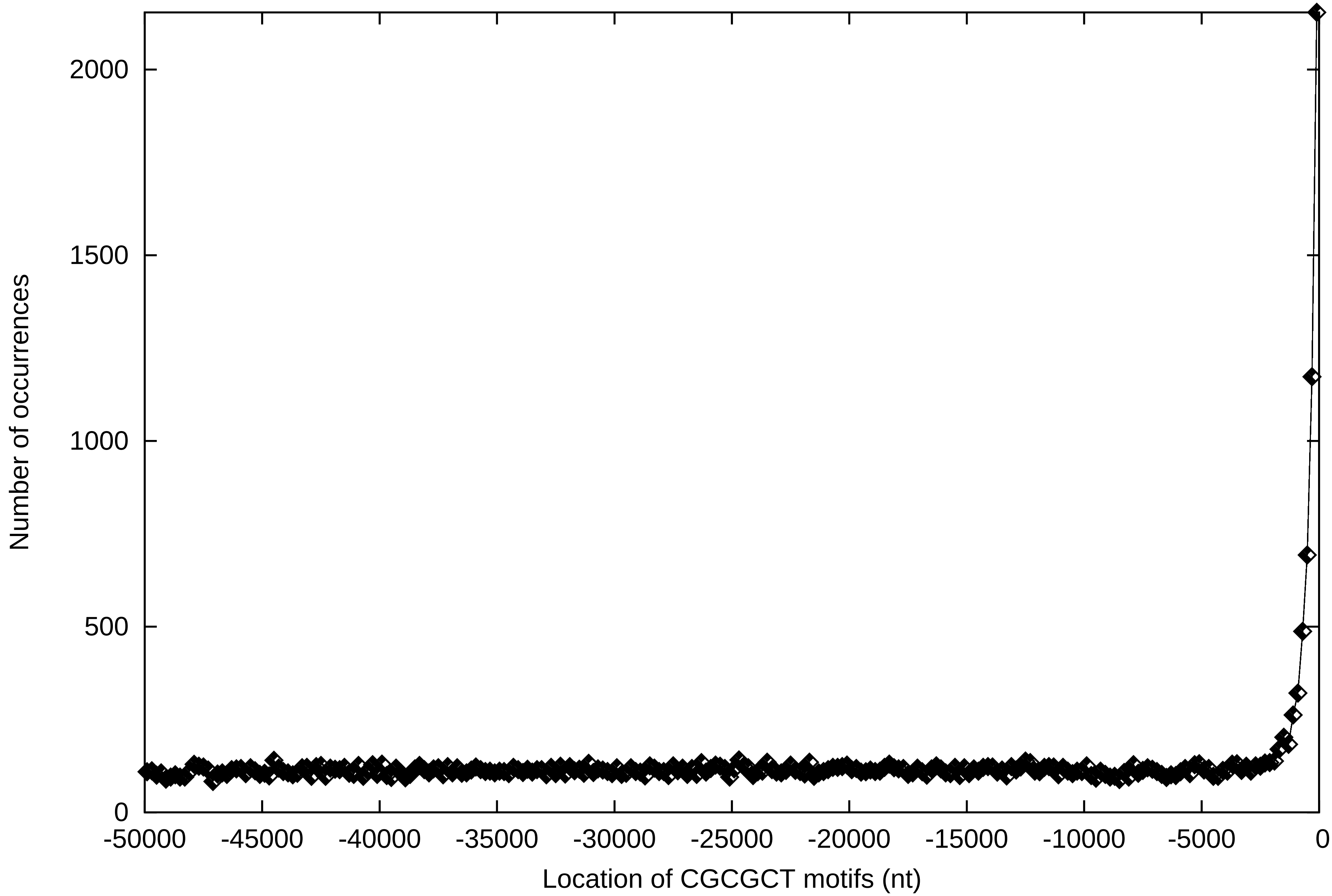

Number of occurrences
0
500
1000
1500
2000
-50000
-45000
-40000
-35000
-30000
-25000
-20000
-15000
-10000
-5000
0
Location of CGCGCT motifs (nt)

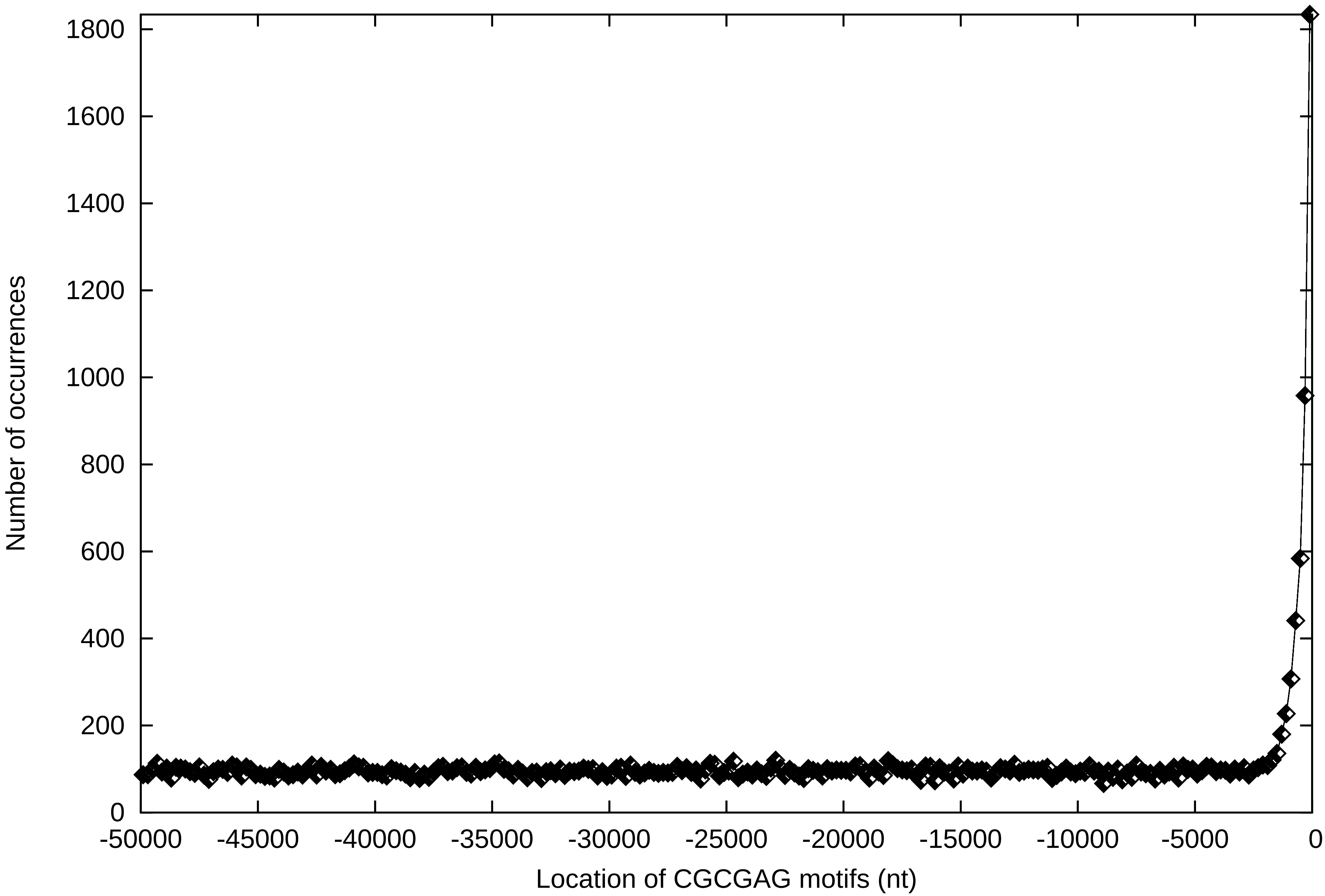

1800
1600
1400
1200
1000
800
600
400
200
0
Number of occurrences
-50000
-45000
-40000
-35000
-30000
-25000
-20000
-15000
-10000
-5000
0
Location of CGCGAG motifs (nt)

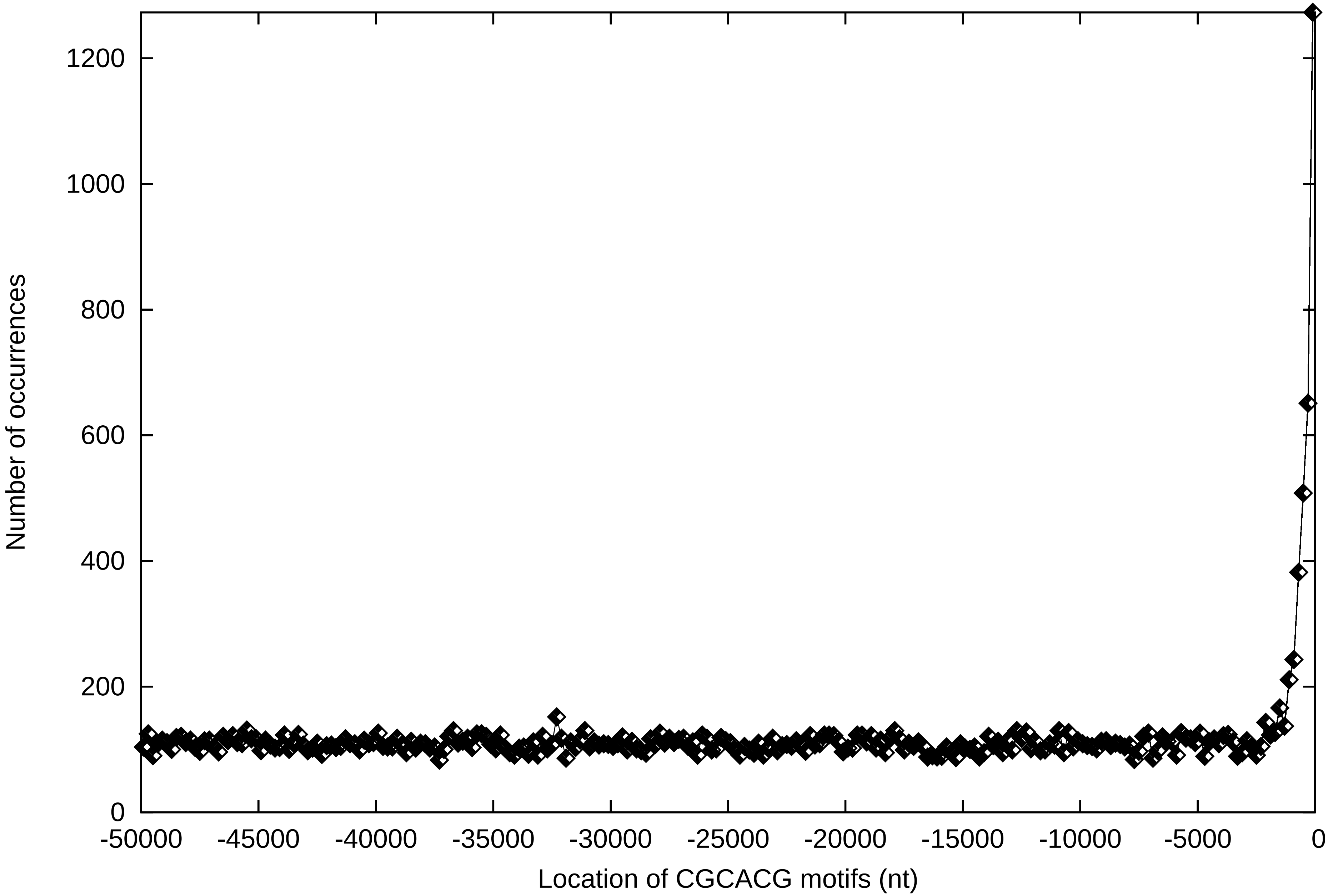

Number of occurrences
0
200
400
600
800
1000
1200
-50000
-45000
-40000
-35000
-30000
-25000
-20000
-15000
-10000
-5000
0
Location of CGCACG motifs (nt)

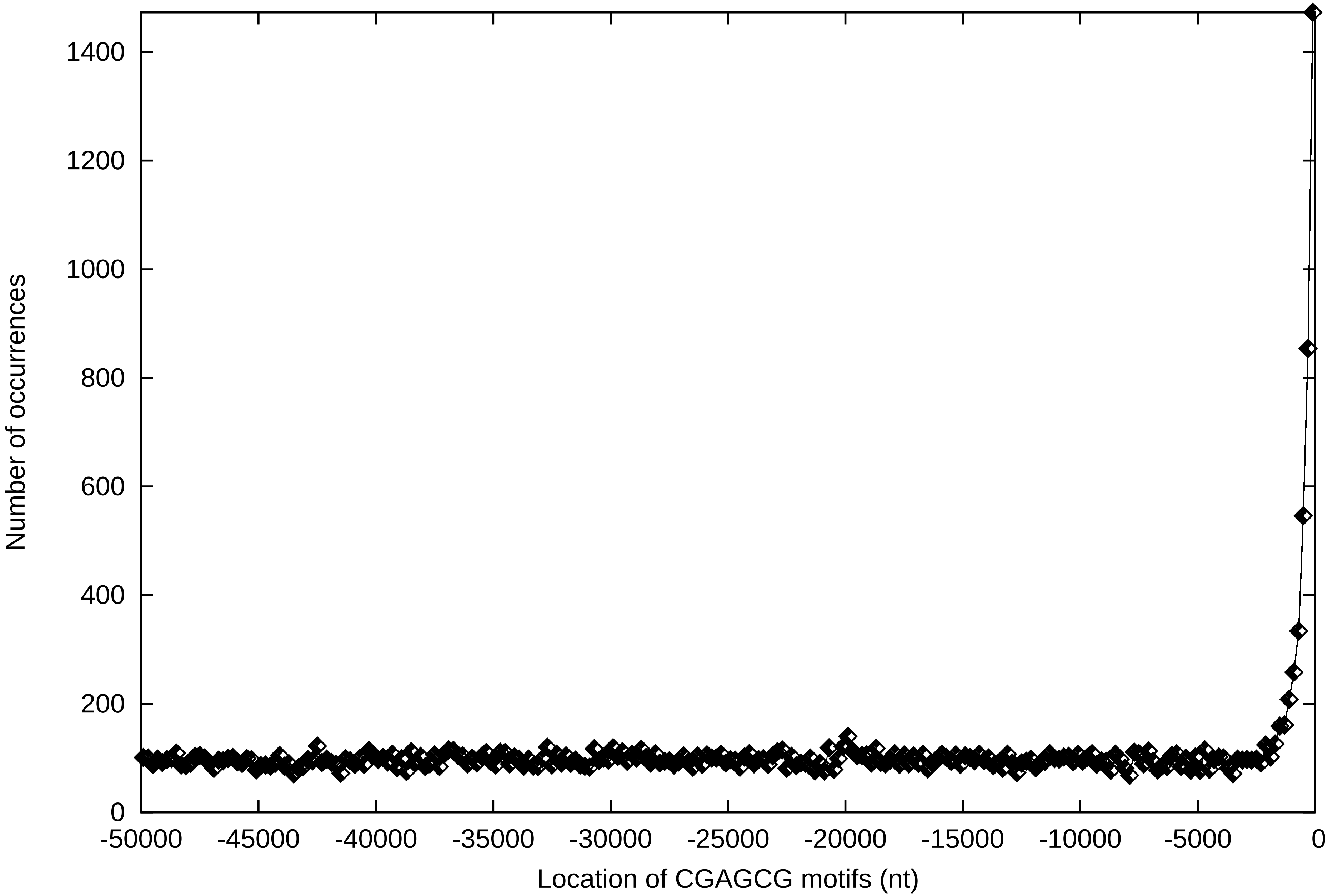
Number of occurrences
0
200
400
600
800
1000
1200
1400
-50000
-45000
-40000
-35000
-30000
-25000
-20000
-15000
-10000
-5000
0
Location of CGAGCG motifs (nt)

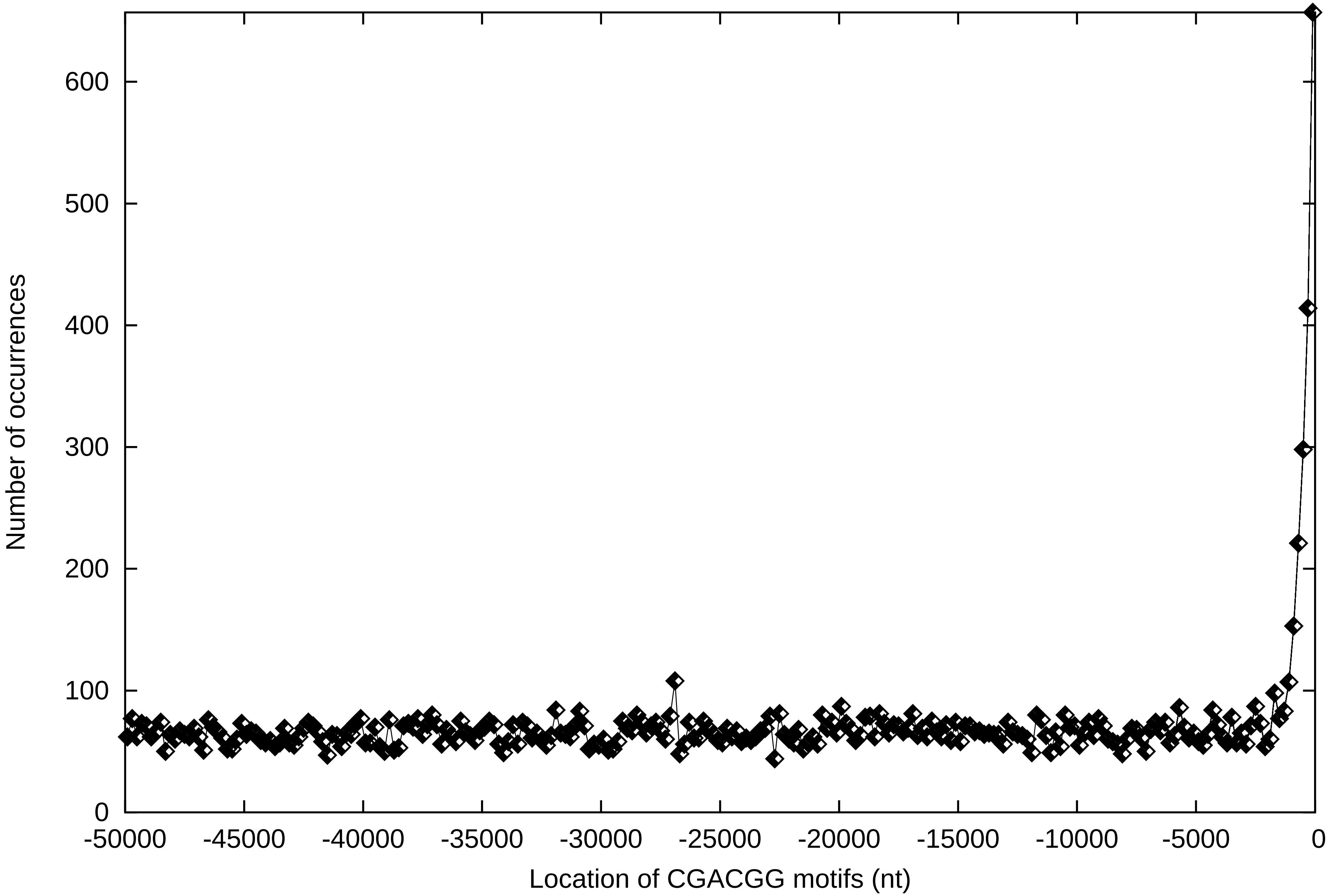
Number of occurrences
0
100
200
300
400
500
600
-50000
-45000
-40000
-35000
-30000
-25000
-20000
-15000
-10000
-5000
0
Location of CGACGG motifs (nt)

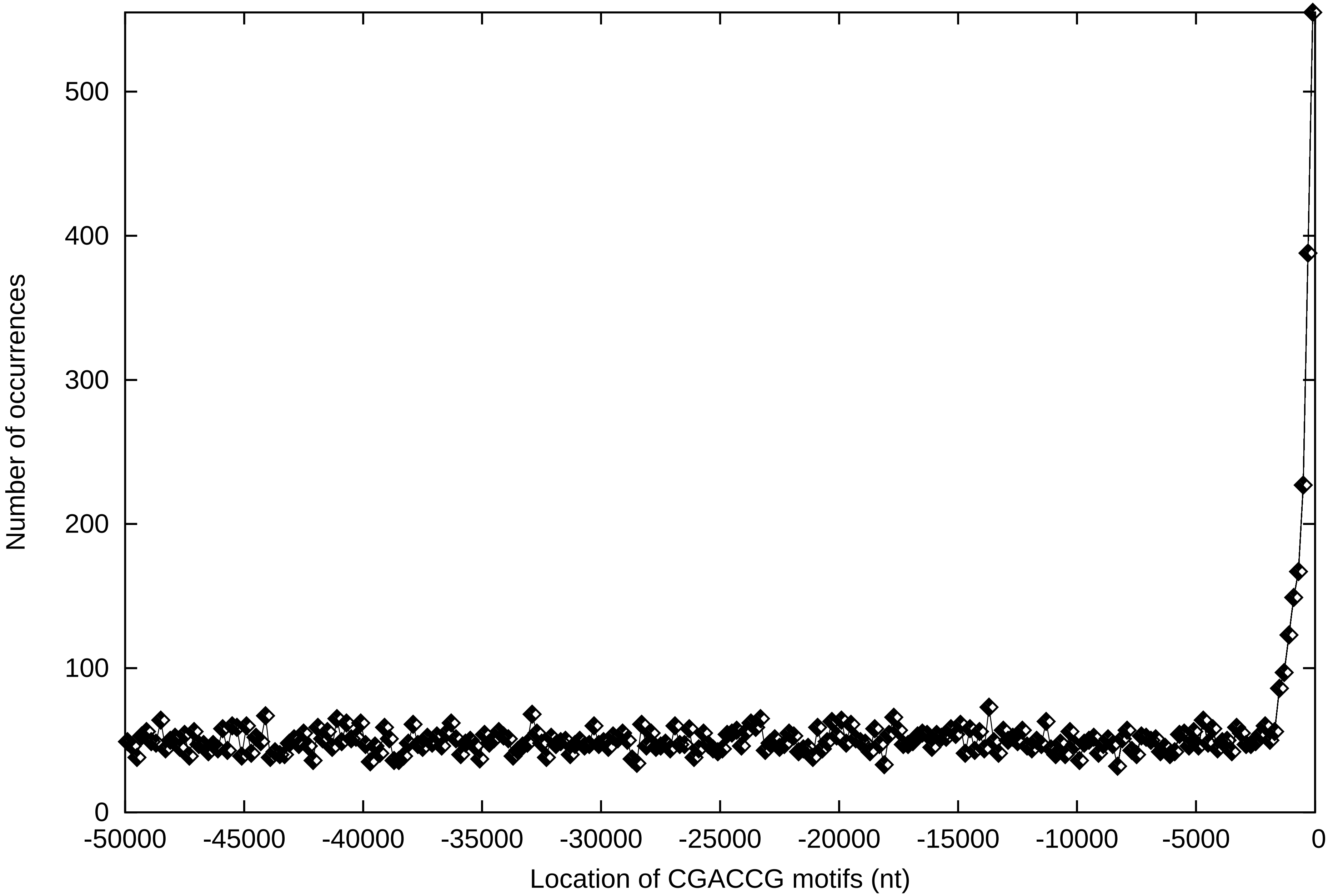

Number of occurrences
0
100
200
300
400
500
-50000
-45000
-40000
-35000
-30000
-25000
-20000
-15000
-10000
-5000
0
Location of CGACCG motifs (nt)

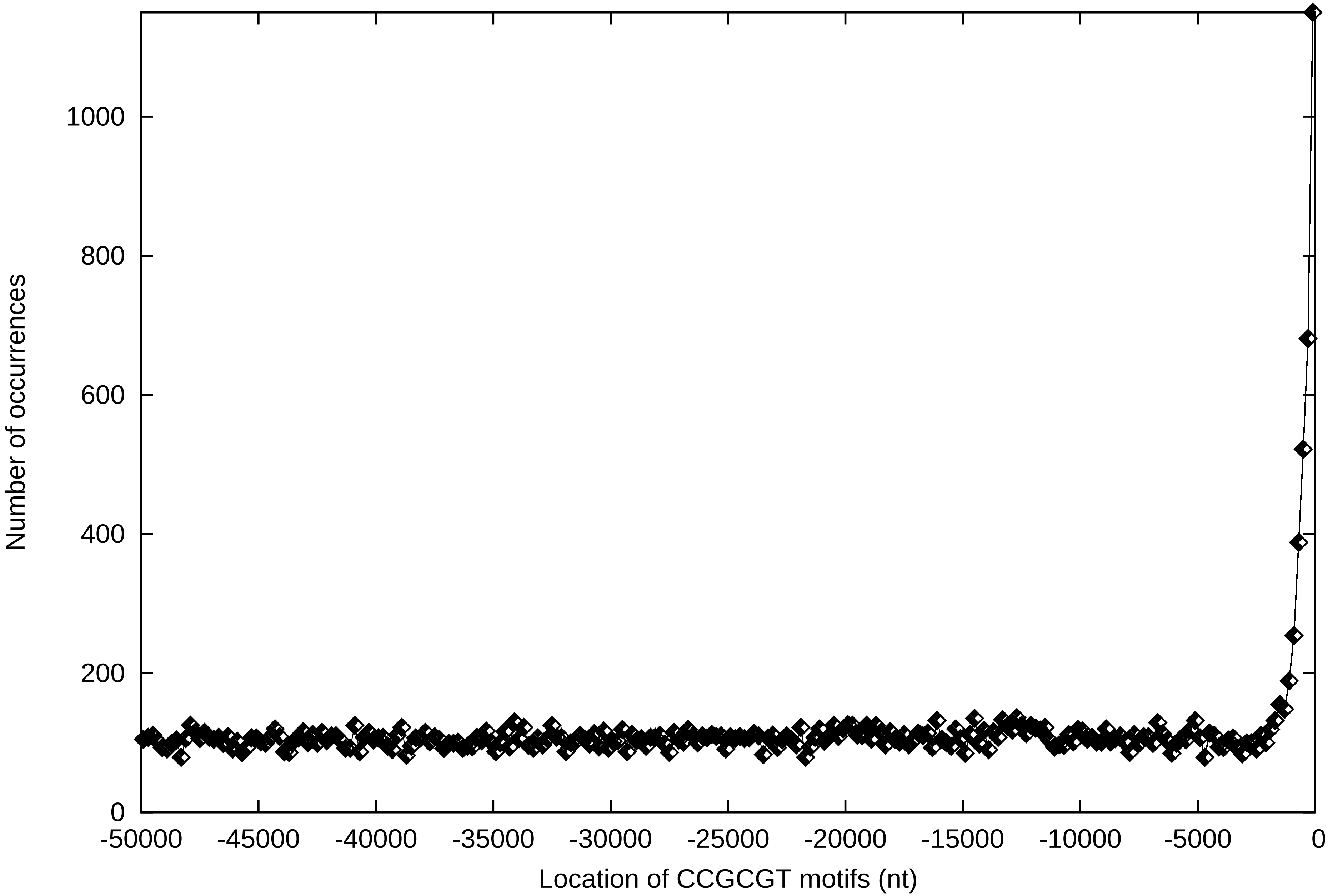

Number of occurrences
1000
800
600
400
200
0
-50000
-45000
-40000
-35000
-30000
-25000
-20000
-15000
-10000
-5000
0
Location of CCGCGT motifs (nt)

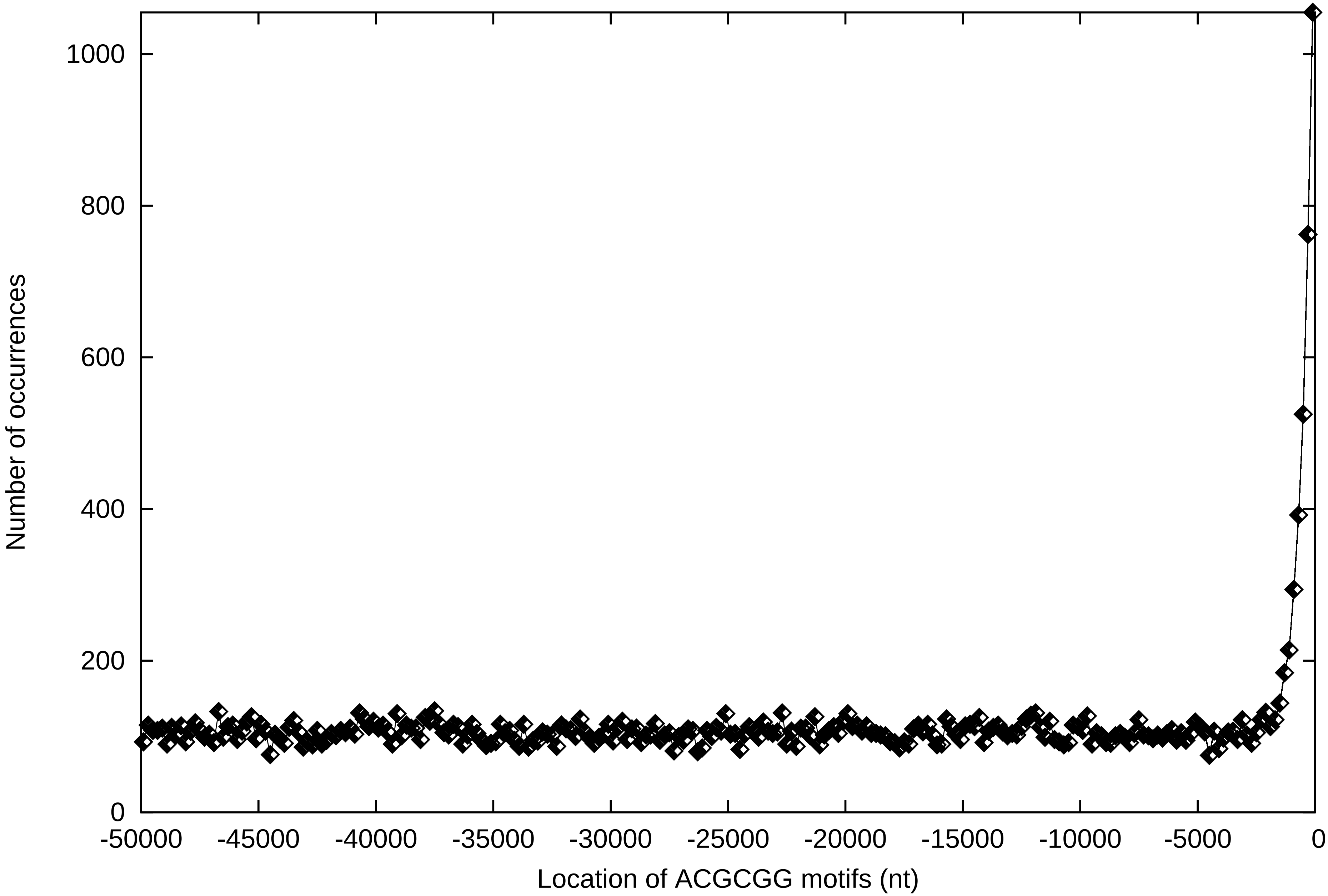

Number of occurrences
0
200
400
600
800
1000
-50000
-45000
-40000
-35000
-30000
-25000
-20000
-15000
-10000
-5000
0
Location of ACGCGG motifs (nt)

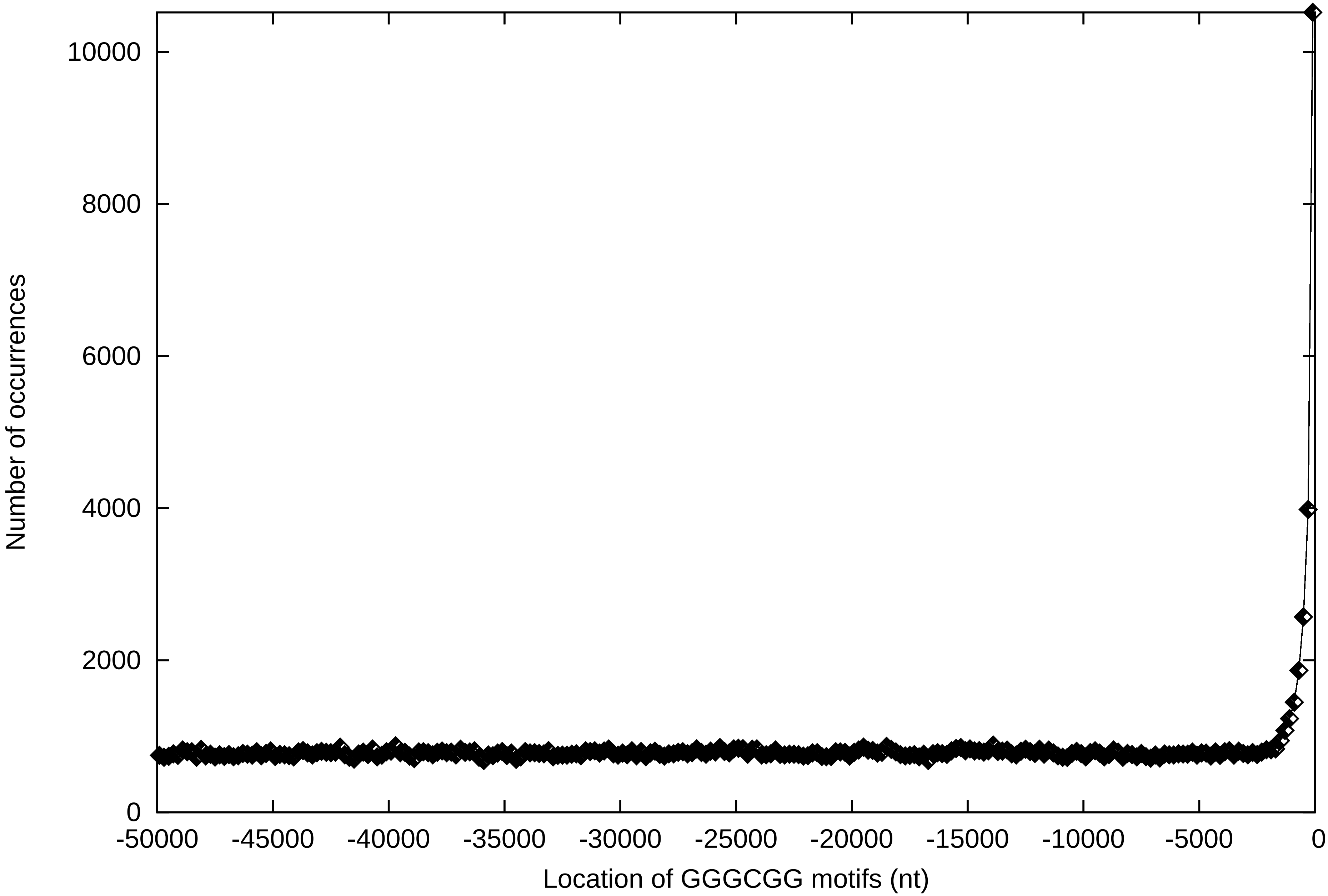

Number of occurrences
0
2000
4000
6000
8000
10000
-50000
-45000
-40000
-35000
-30000
-25000
-20000
-15000
-10000
-5000
0
Location of GGGCGG motifs (nt)

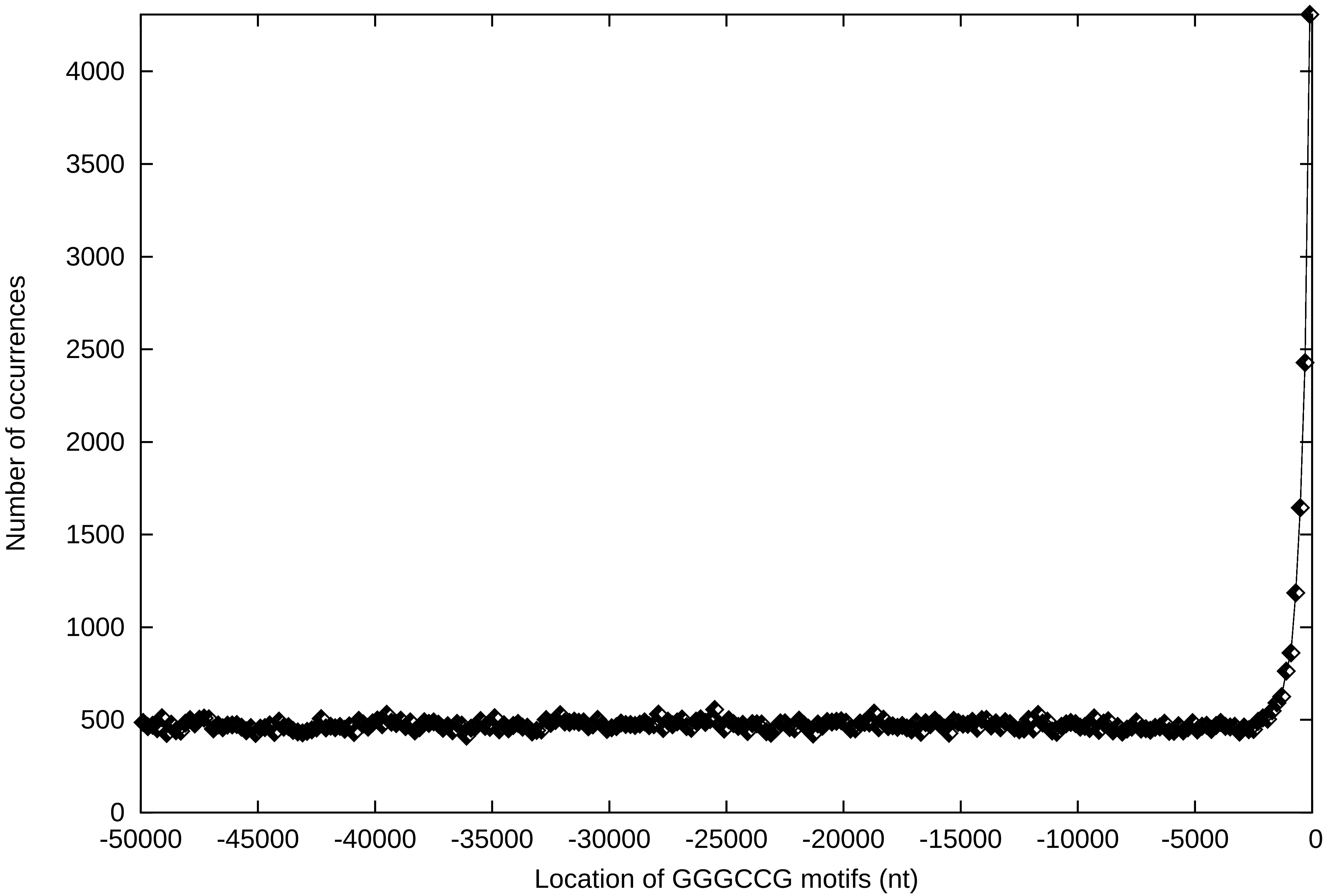

Number of occurrences
0
500
1000
1500
2000
2500
3000
3500
4000
-50000
-45000
-40000
-35000
-30000
-25000
-20000
-15000
-10000
-5000
0
Location of GGGCCG motifs (nt)

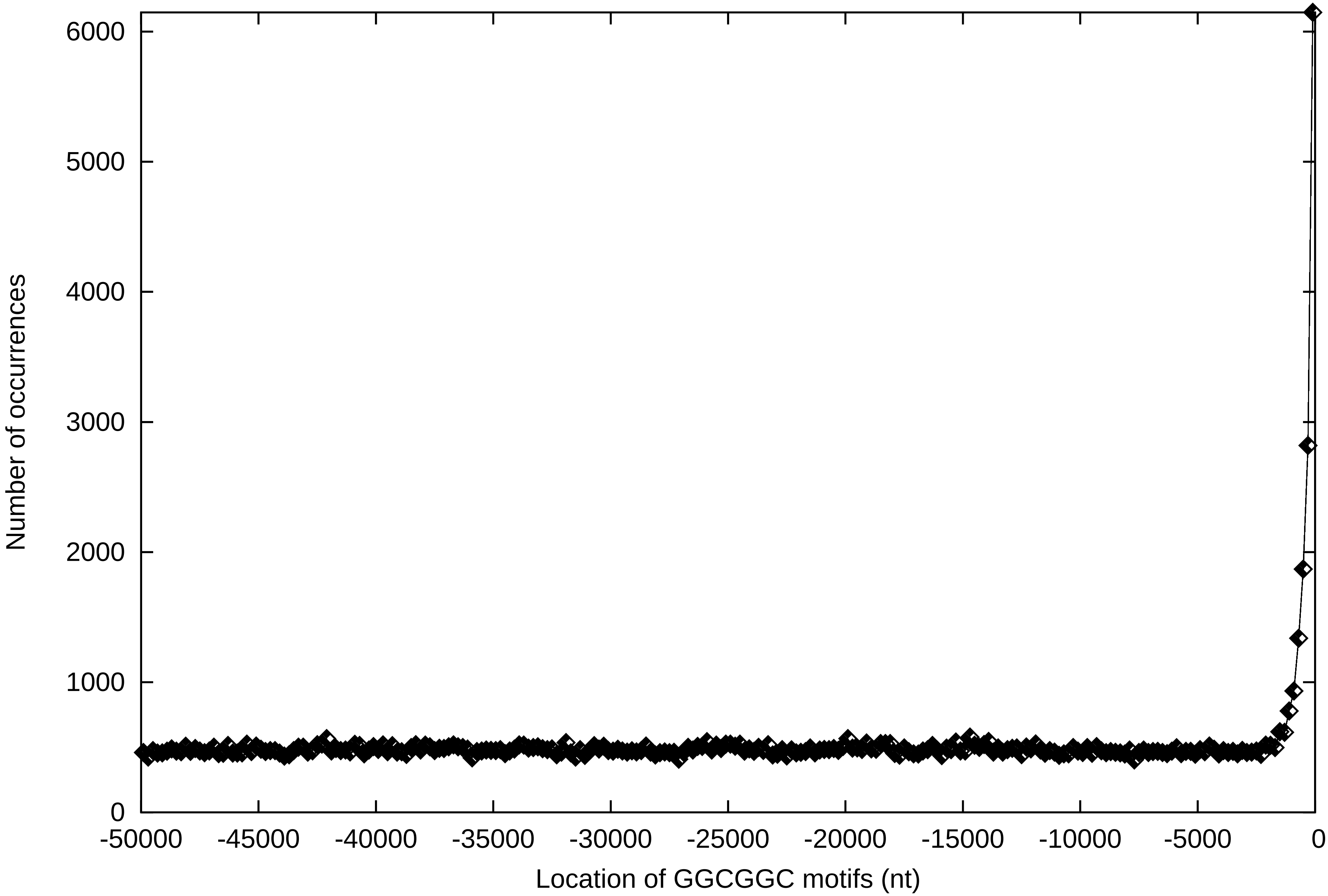

Number of occurrences
0
1000
2000
3000
4000
5000
6000
-50000
-45000
-40000
-35000
-30000
-25000
-20000
-15000
-10000
-5000
0
Location of GGCGGC motifs (nt)

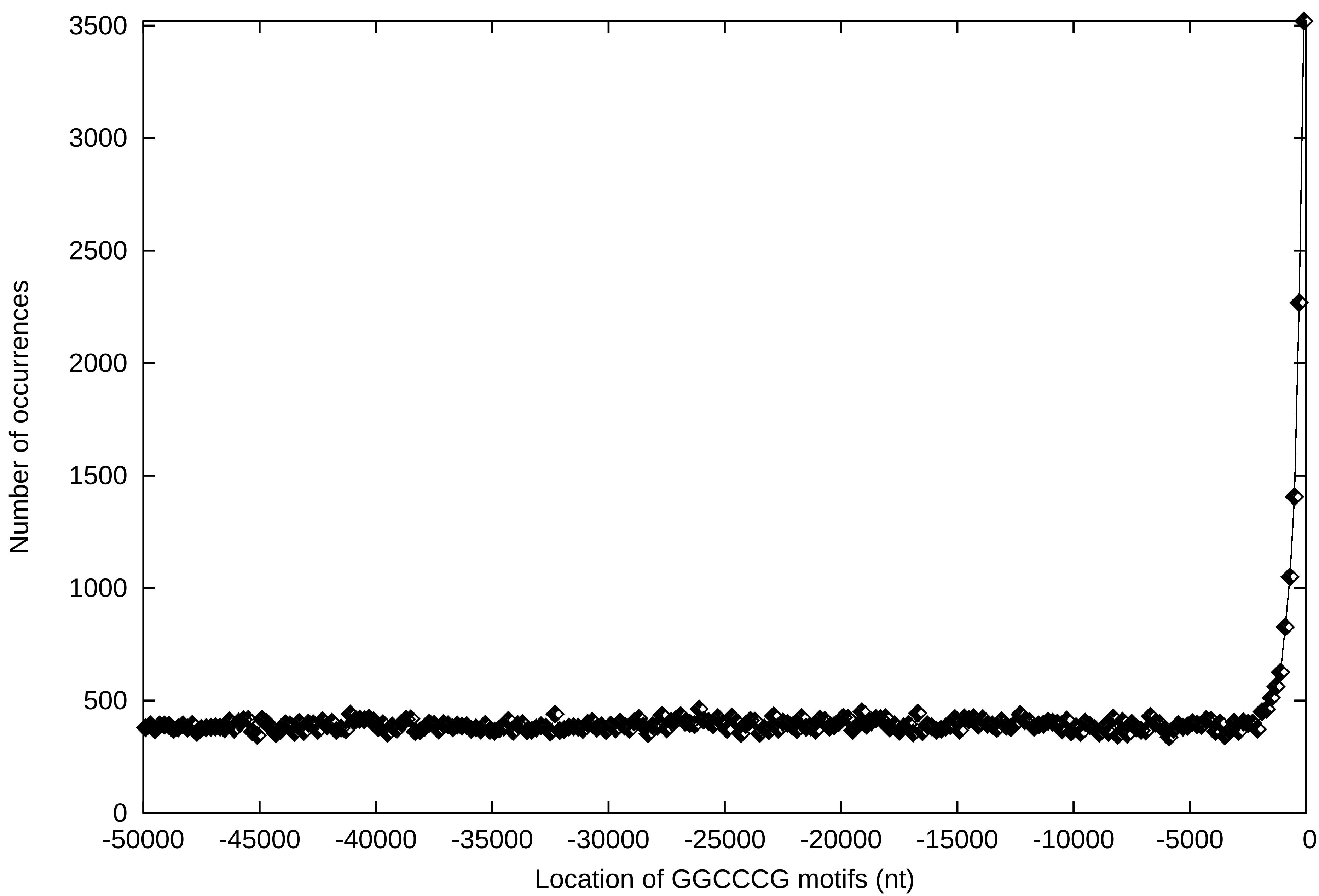

3500
3000
2500
2000
1500
1000
500
0
-50000
-45000
-40000
-35000
-30000
-25000
-20000
-15000
-10000
-5000
0
Number of occurrences
Location of GGCCCG motifs (nt)

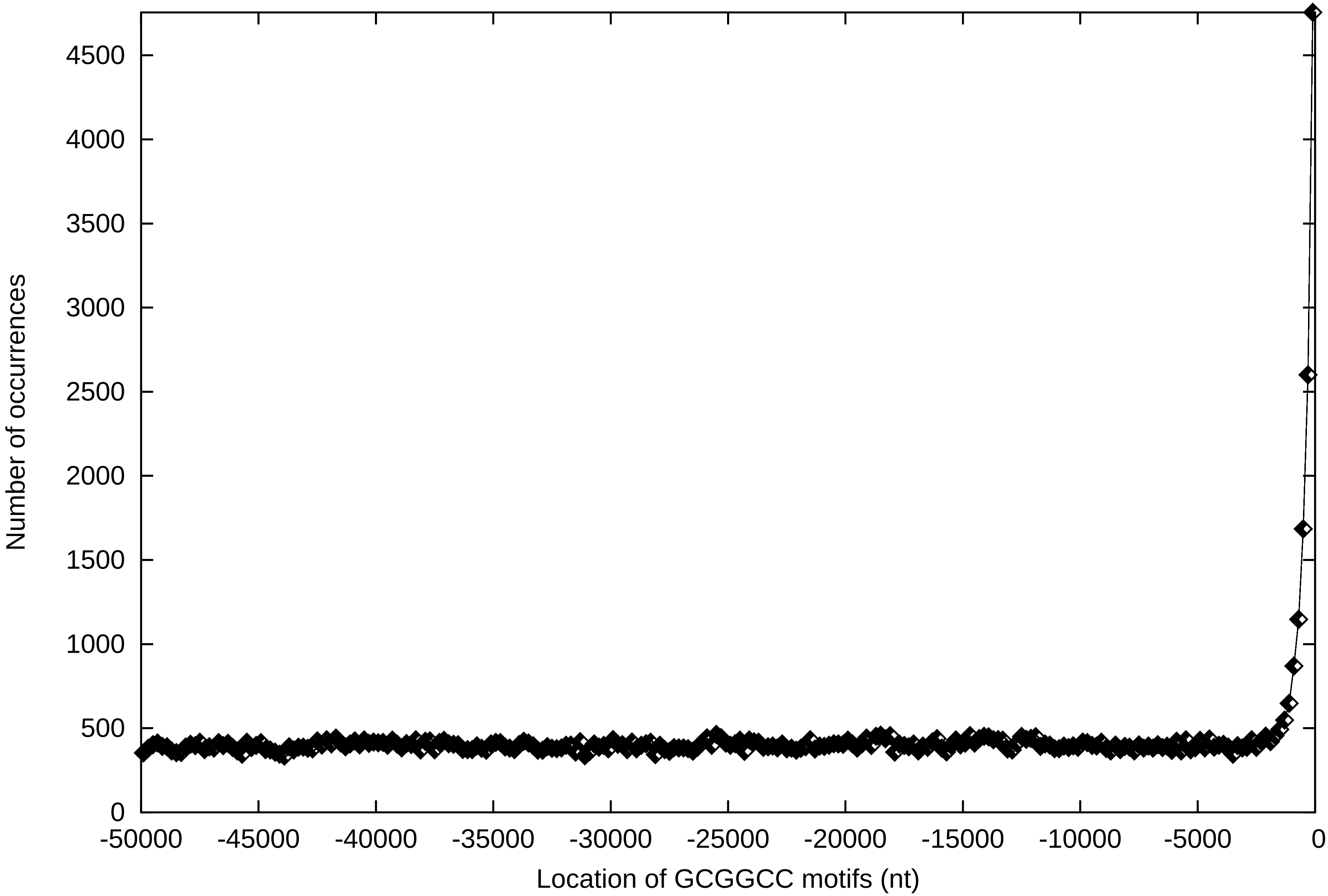

Number of occurrences
0
500
1000
1500
2000
2500
3000
3500
4000
4500
-50000
-45000
-40000
-35000
-30000
-25000
-20000
-15000
-10000
-5000
0
Location of GCGGCC motifs (nt)

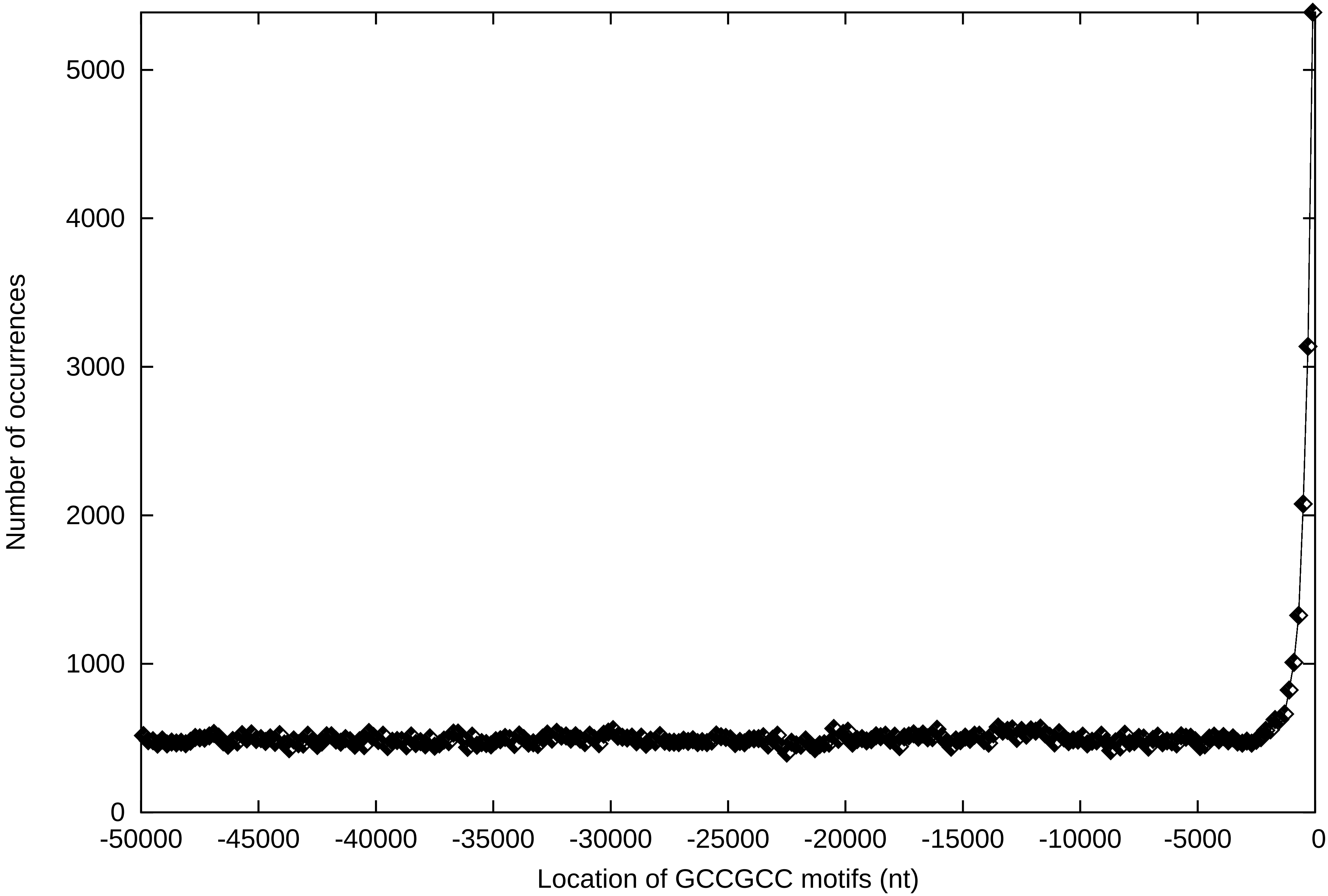

Number of occurrences
5000
4000
3000
2000
1000
0
-50000
-45000
-40000
-35000
-30000
-25000
-20000
-15000
-10000
-5000
0
Location of GCCGCC motifs (nt)

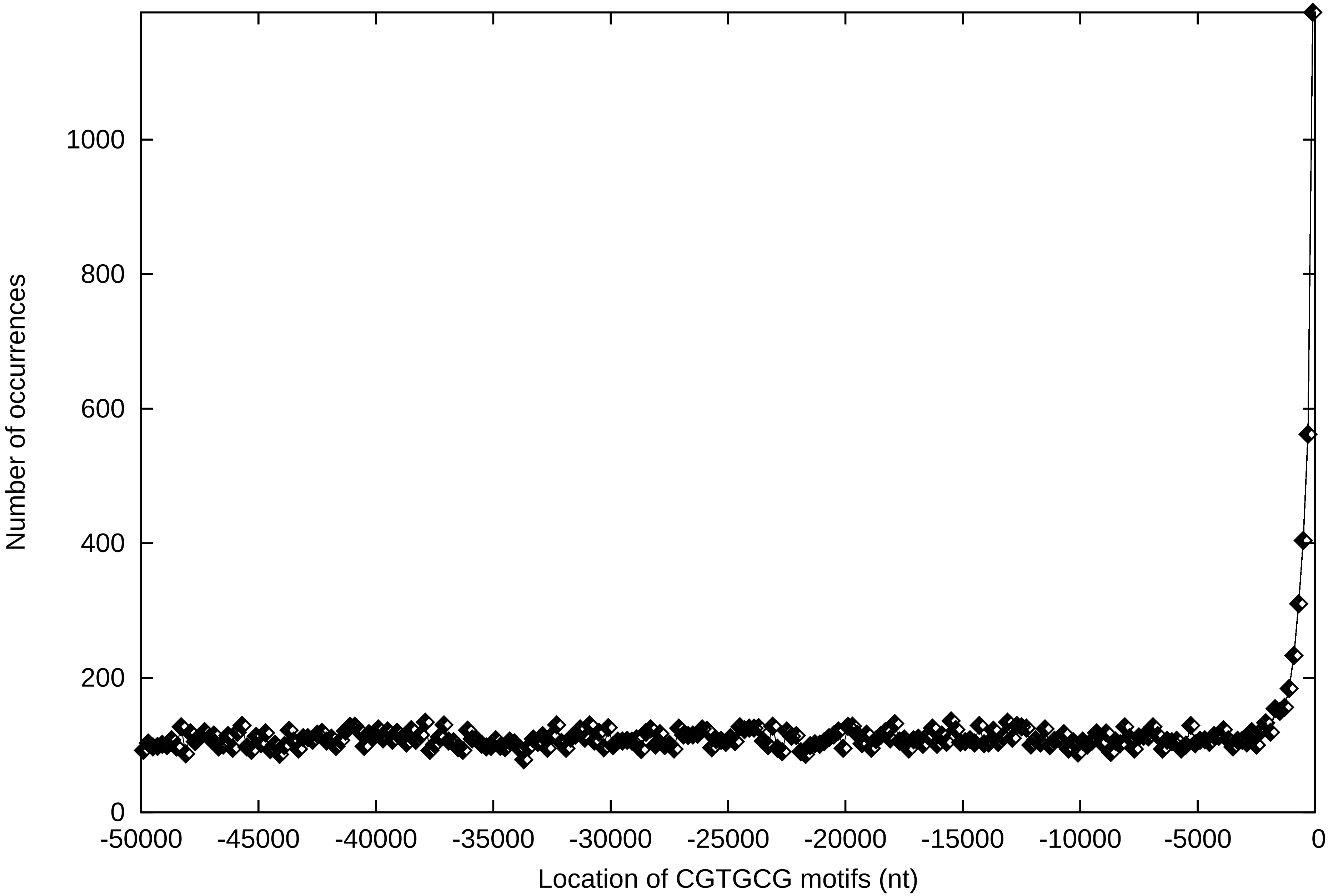
Number of occurrences
1000
800
600
400
200
0
-50000
-45000
-40000
-35000
-30000
-25000
-20000
-15000
-10000
-5000
0
Location of CGTGCG motifs (nt)

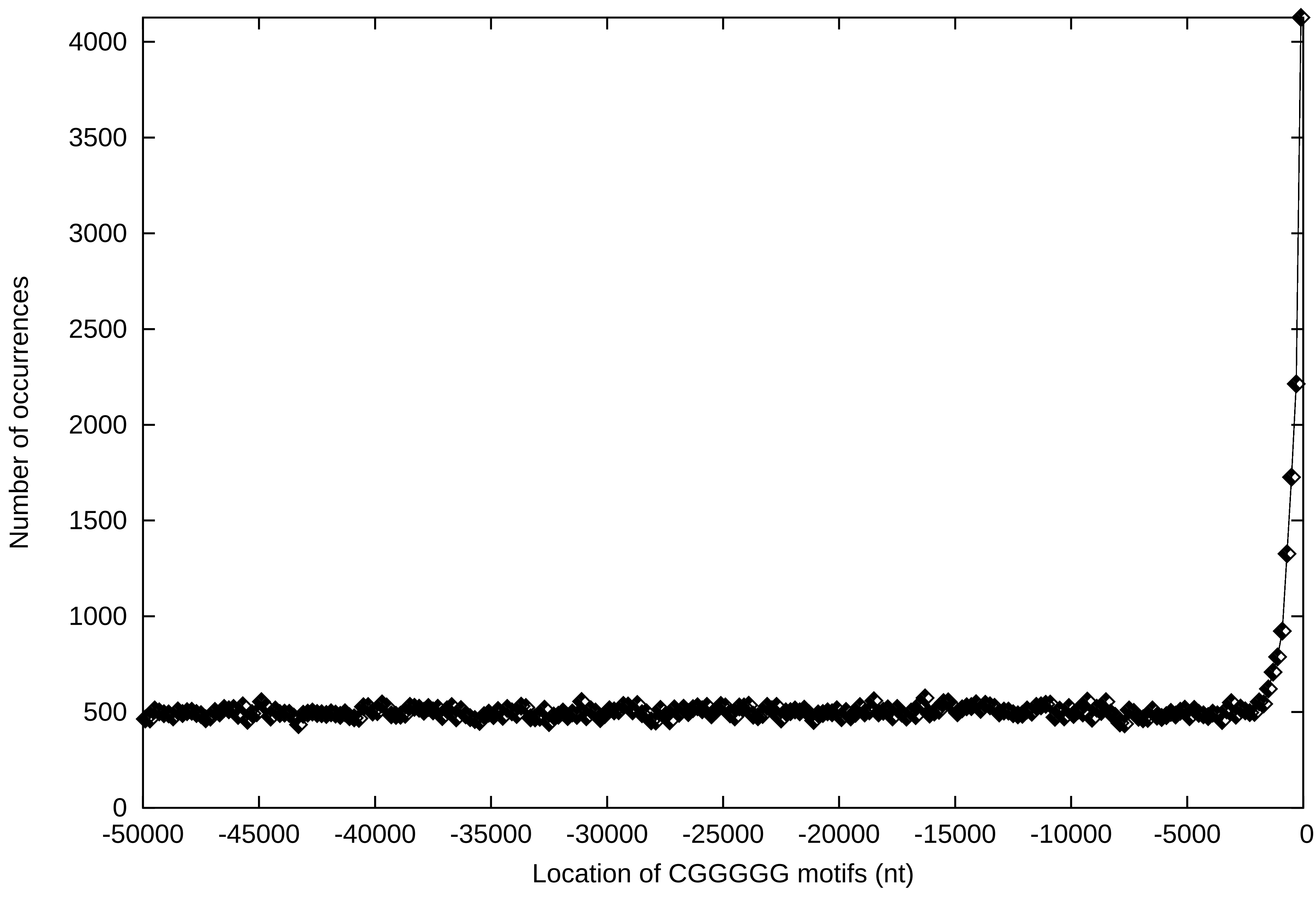
Number of occurrences
0
500
1000
1500
2000
2500
3000
3500
4000
-50000
-45000
-40000
-35000
-30000
-25000
-20000
-15000
-10000
-5000
0
Location of CGGGGG motifs (nt)

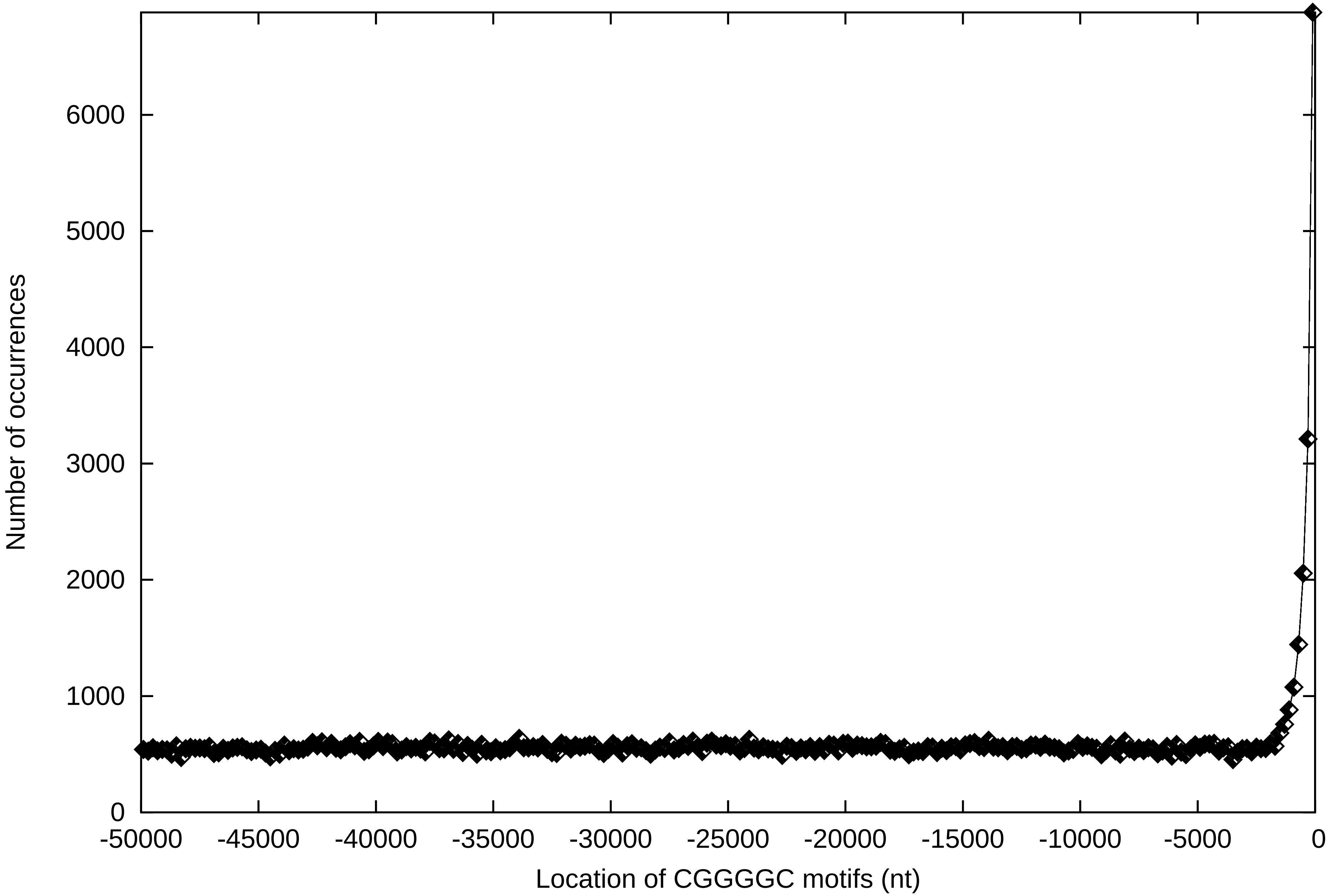

Number of occurrences
0
1000
2000
3000
4000
5000
6000
-50000
-45000
-40000
-35000
-30000
-25000
-20000
-15000
-10000
-5000
0
Location of CGGGGC motifs (nt)

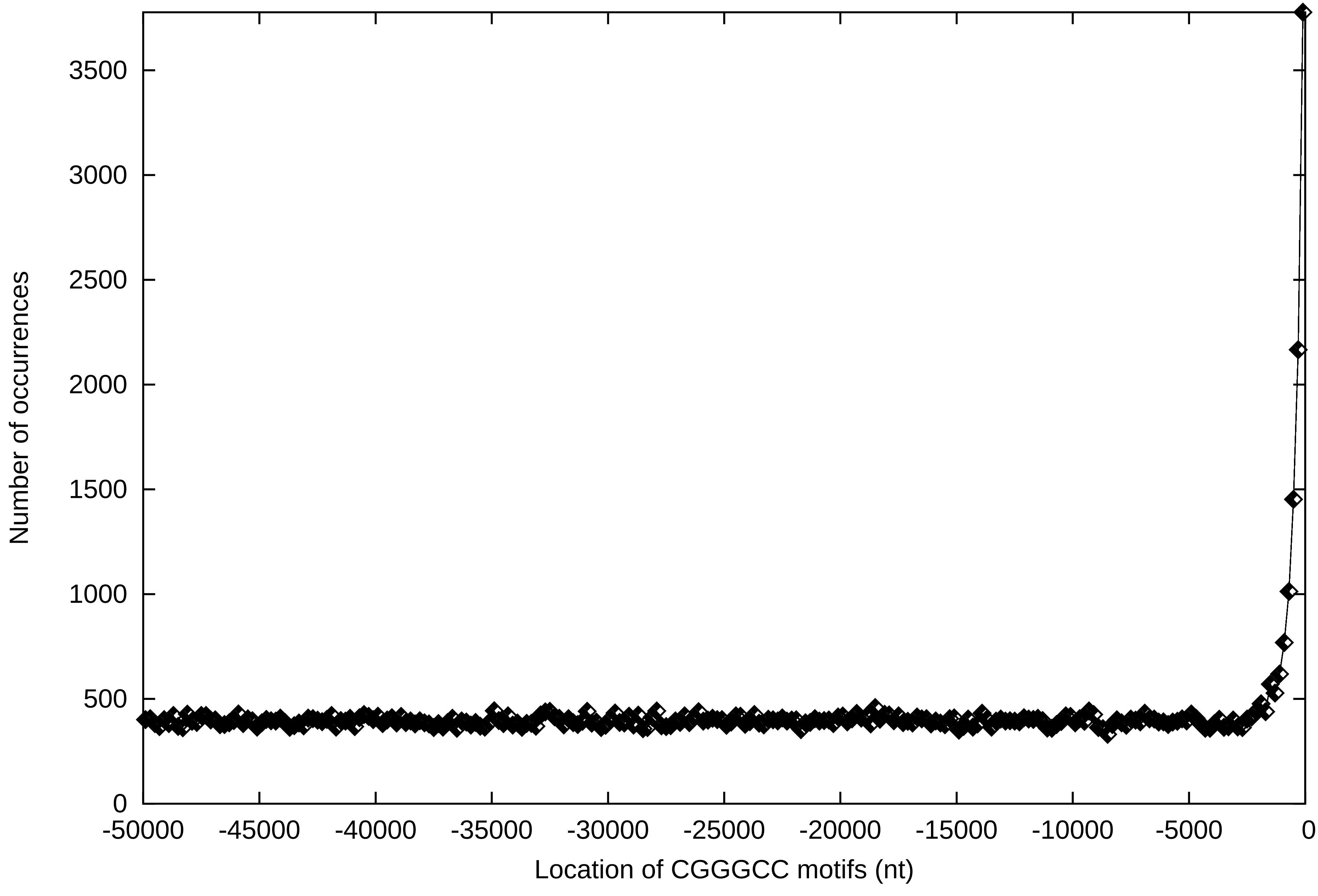

3500
3000
2500
2000
1500
1000
500
0
Number of occurrences
-50000
-45000
-40000
-35000
-30000
-25000
-20000
-15000
-10000
-5000
0
Location of CGGGCC motifs (nt)

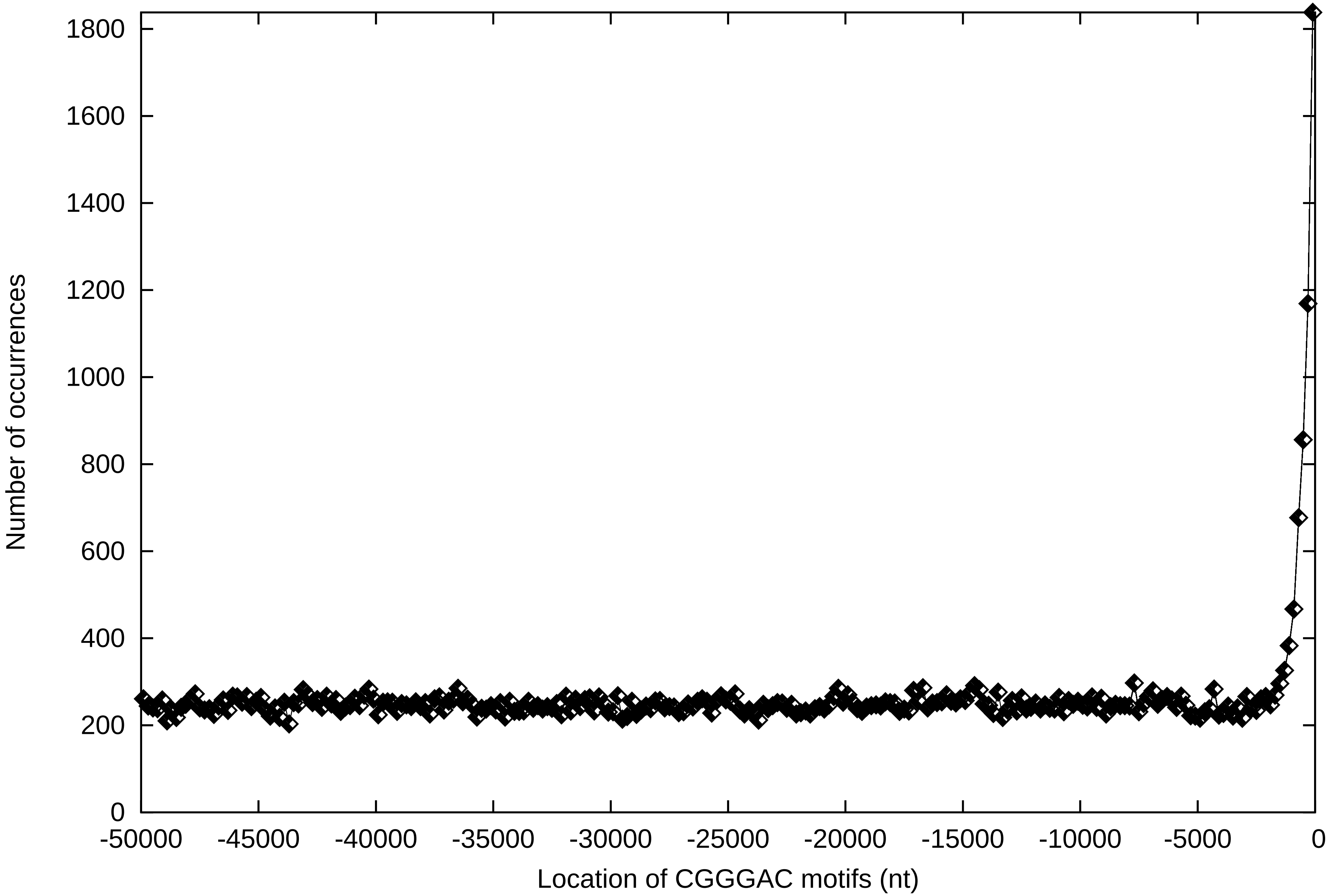

Number of occurrences
1800
1600
1400
1200
1000
800
600
400
200
0
-50000
-45000
-40000
-35000
-30000
-25000
-20000
-15000
-10000
-5000
0
Location of CGGGAC motifs (nt)

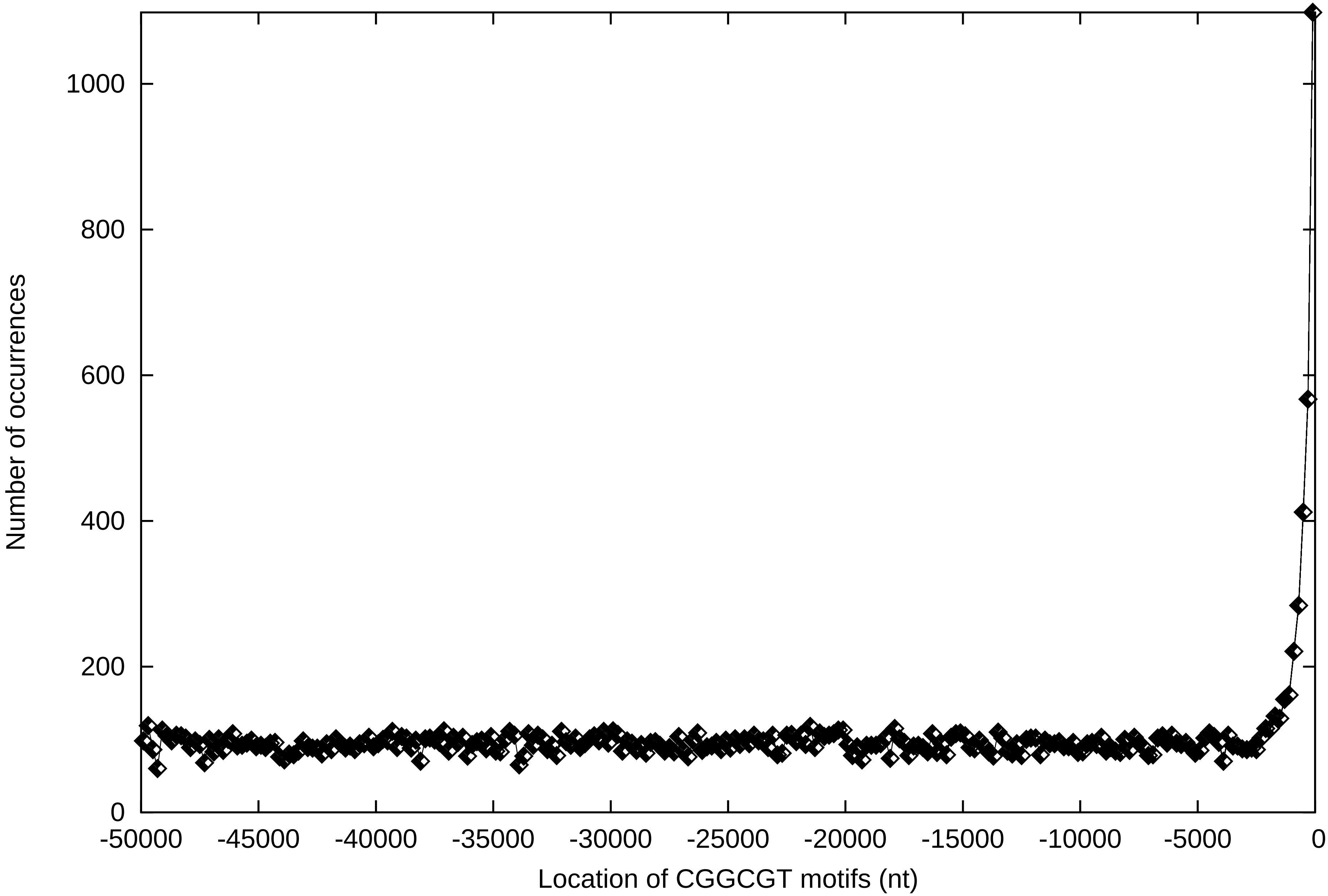
1000
800
600
400
200
0
-50000
-45000
-40000
-35000
-30000
-25000
-20000
-15000
-10000
-5000
0
Number of occurrences
Location of CGGCGT motifs (nt)

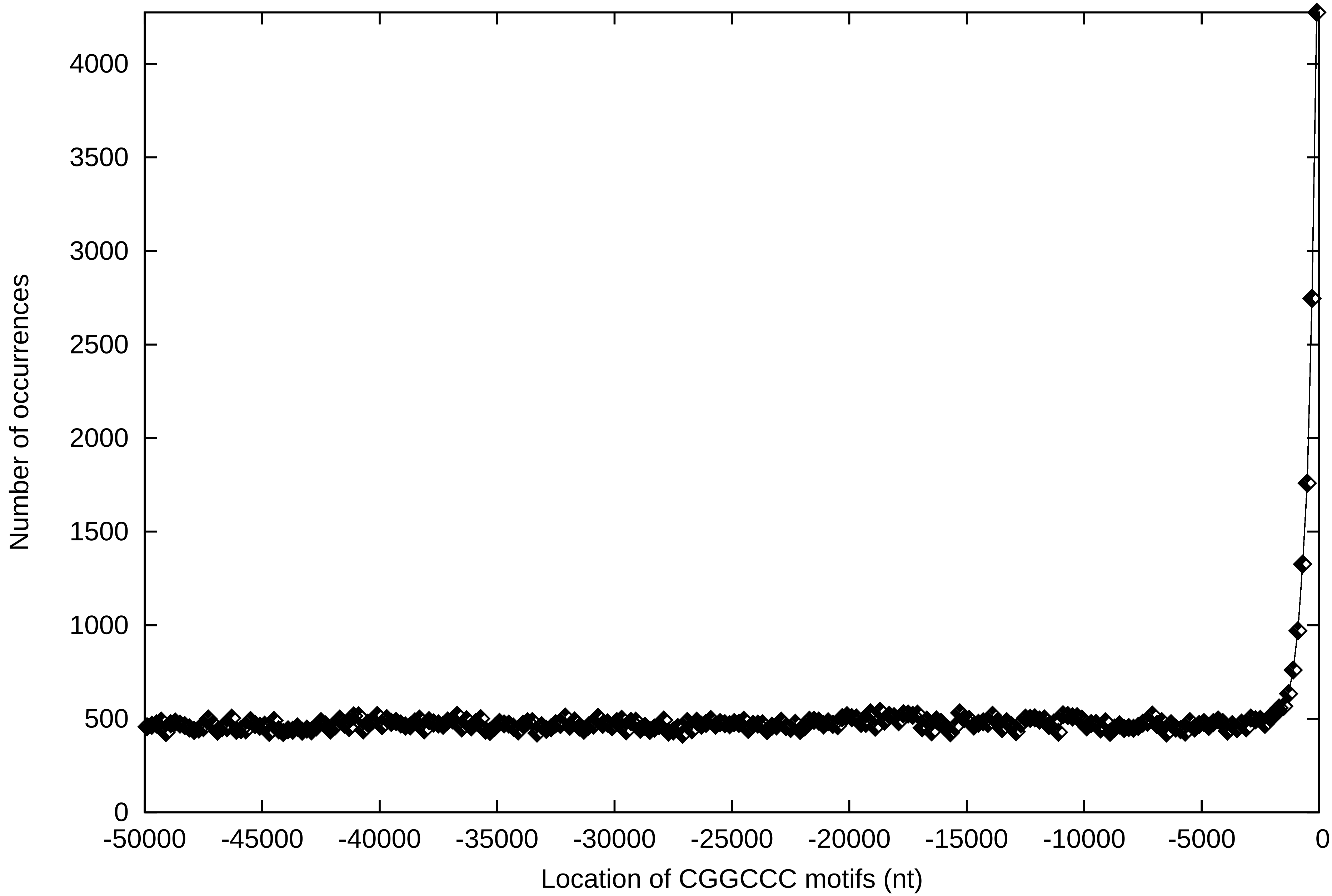

Number of occurrences
Location of CGGCCC motifs (nt)
0
500
1000
1500
2000
2500
3000
3500
4000
-50000
-45000
-40000
-35000
-30000
-25000
-20000
-15000
-10000
-5000
0

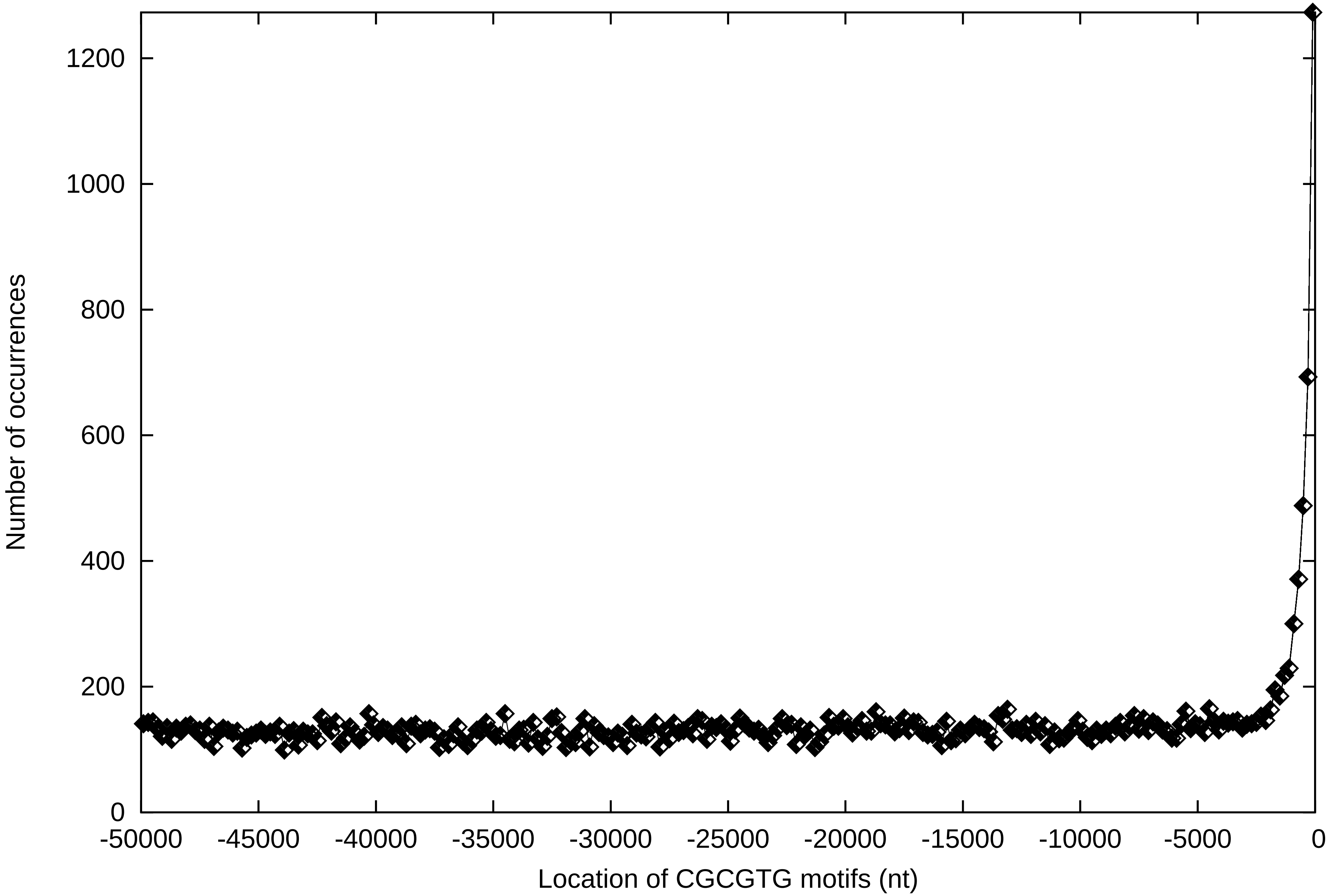
Number of occurrences
0
200
400
600
800
1000
1200
-50000
-45000
-40000
-35000
-30000
-25000
-20000
-15000
-10000
-5000
0
Location of CGCGTG motifs (nt)

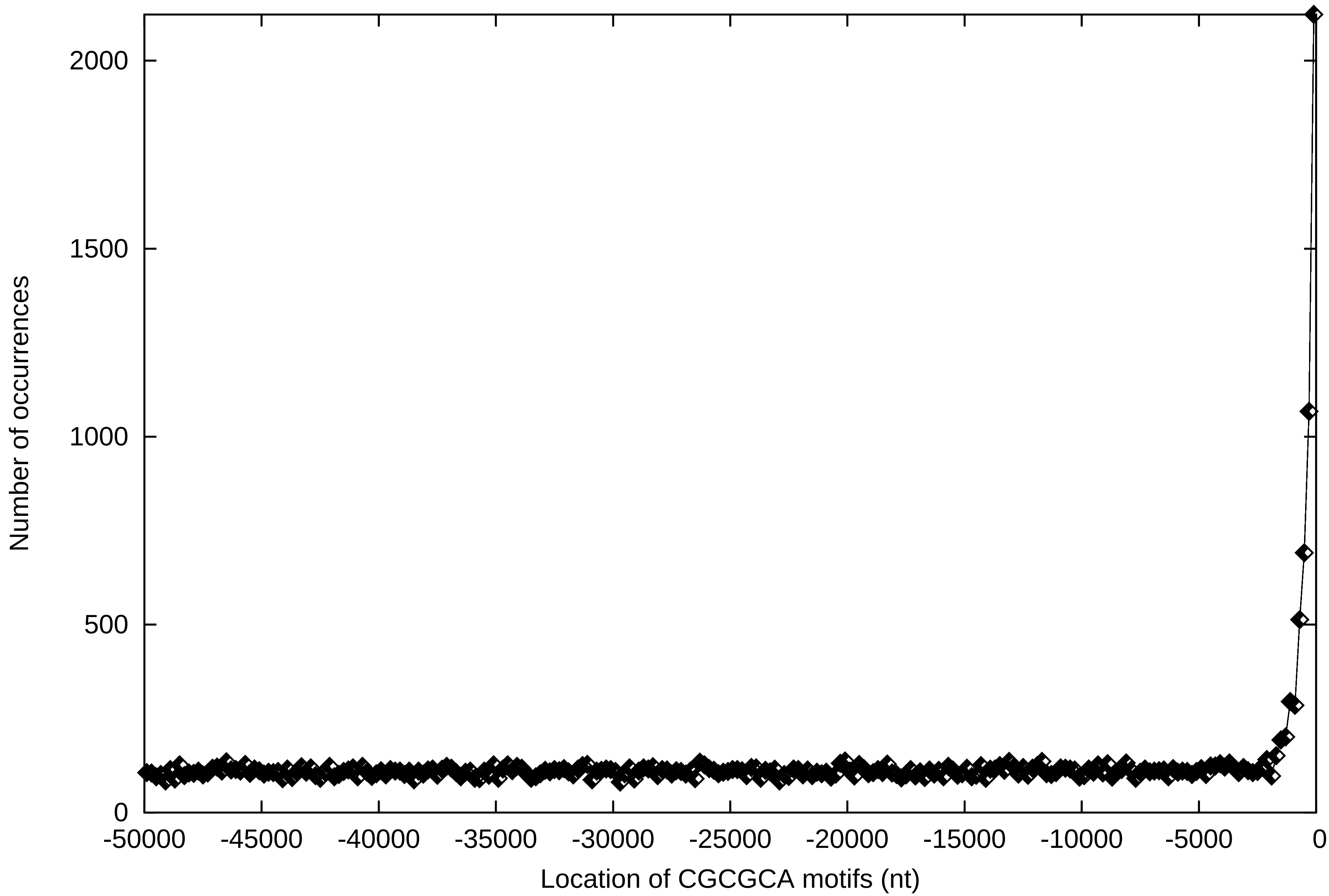

Number of occurrences
2000
1500
1000
500
0
-50000
-45000
-40000
-35000
-30000
-25000
-20000
-15000
-10000
-5000
0
Location of CGCGCA motifs (nt)

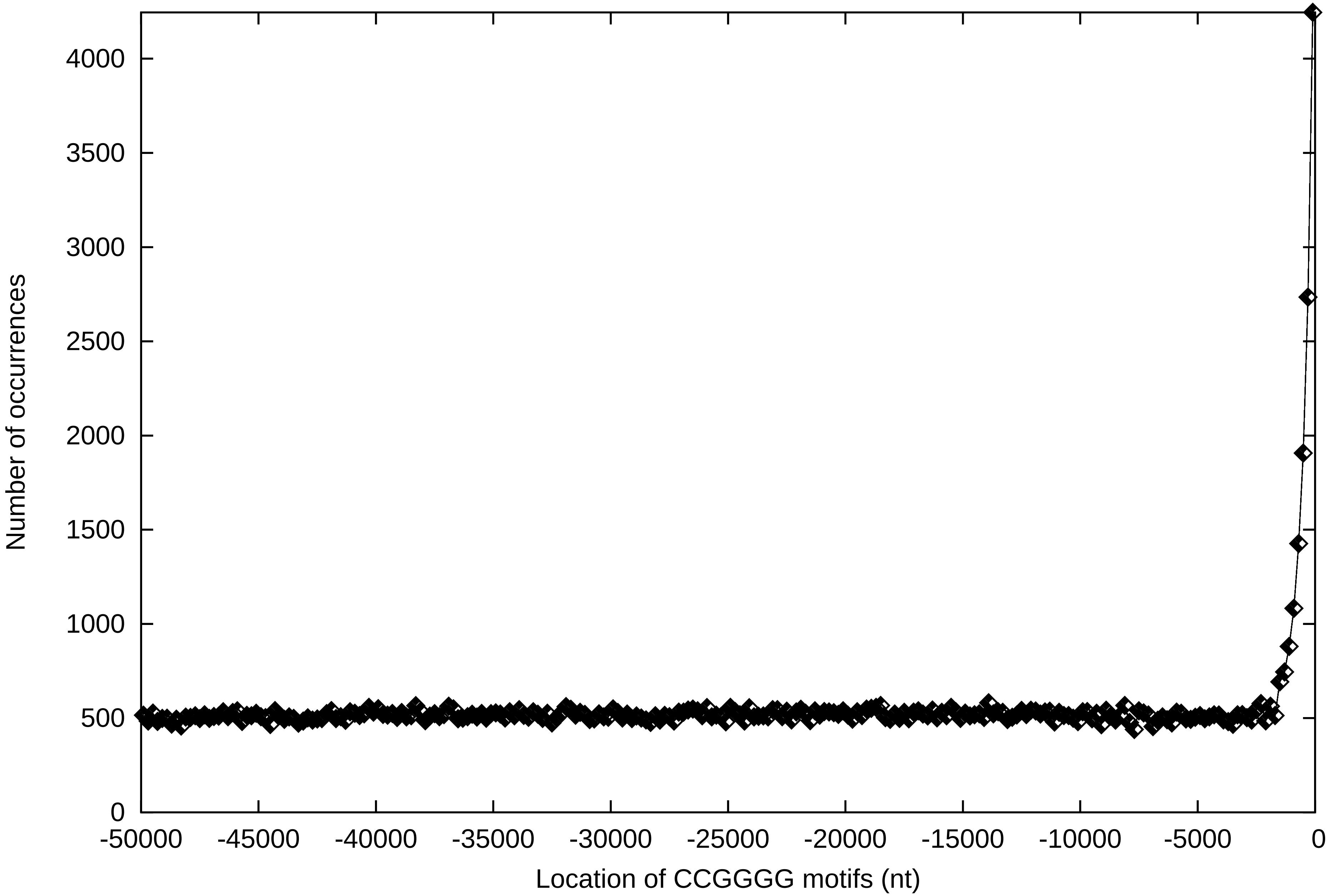

Number of occurrences
0
500
1000
1500
2000
2500
3000
3500
4000
-50000
-45000
-40000
-35000
-30000
-25000
-20000
-15000
-10000
-5000
0
Location of CCGGGG motifs (nt)

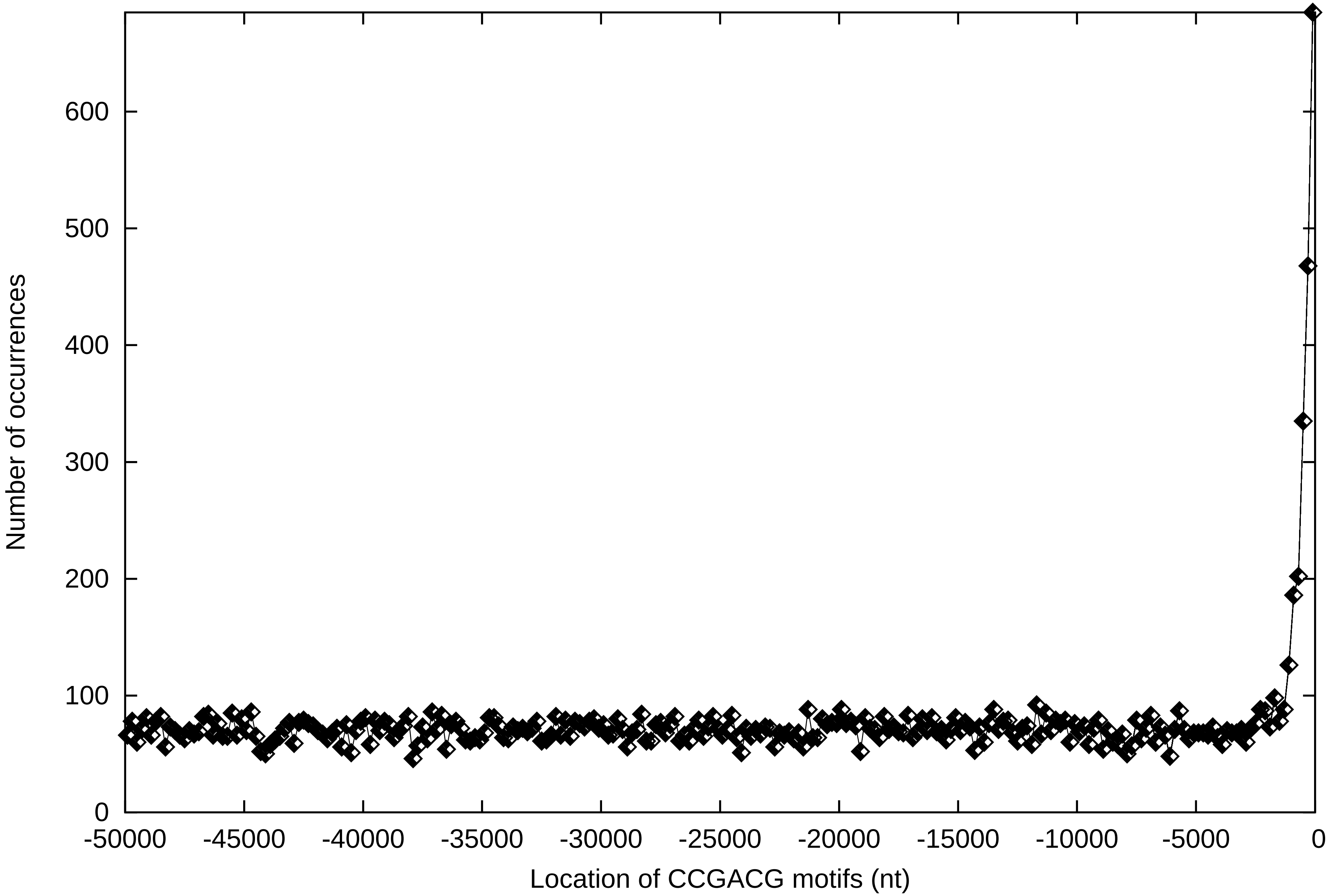

Number of occurrences
0
100
200
300
400
500
600
-50000
-45000
-40000
-35000
-30000
-25000
-20000
-15000
-10000
-5000
0
Location of CCGACG motifs (nt)

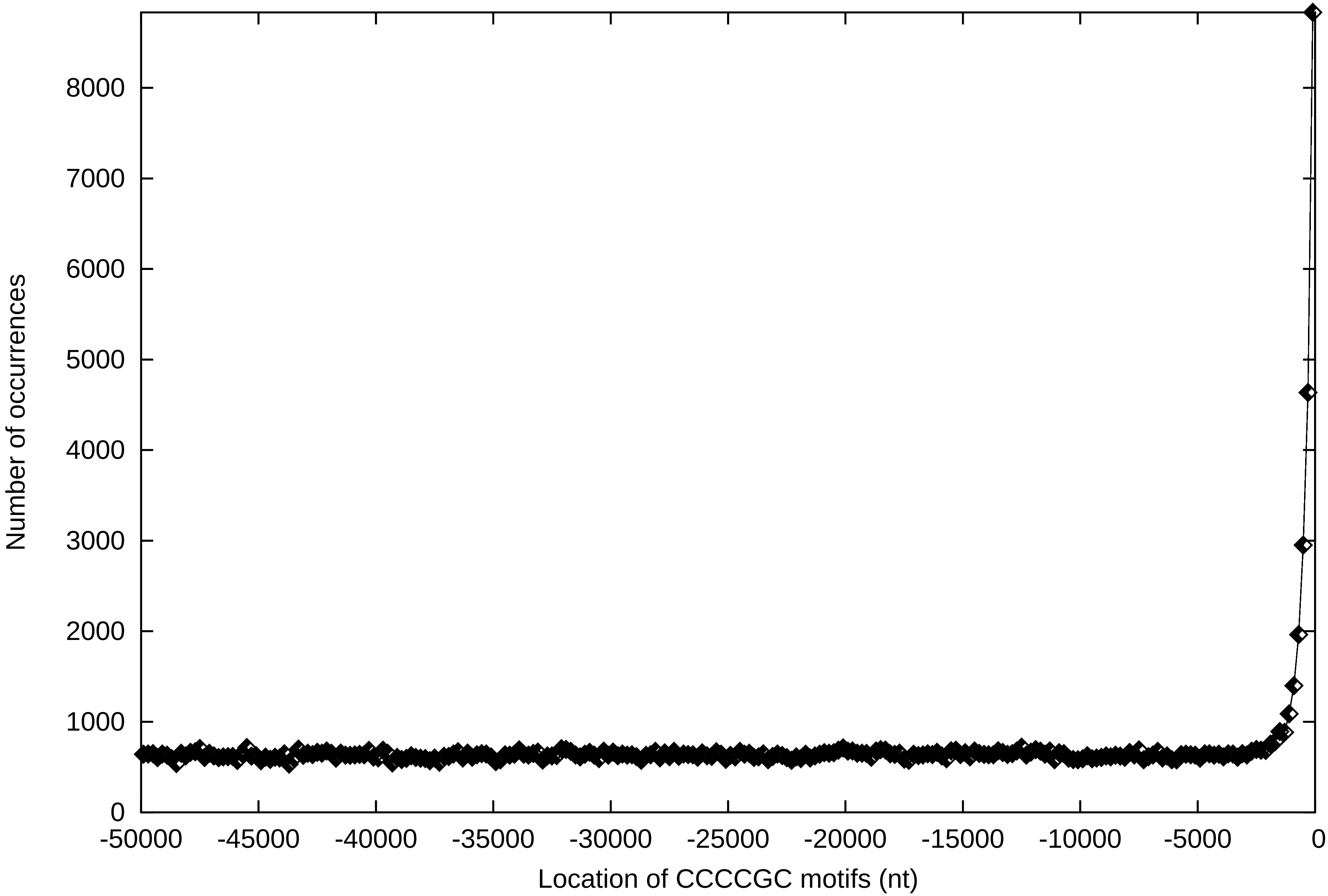

Number of occurrences
0
1000
2000
3000
4000
5000
6000
7000
8000
-50000
-45000
-40000
-35000
-30000
-25000
-20000
-15000
-10000
-5000
0
Location of CCCCGC motifs (nt)

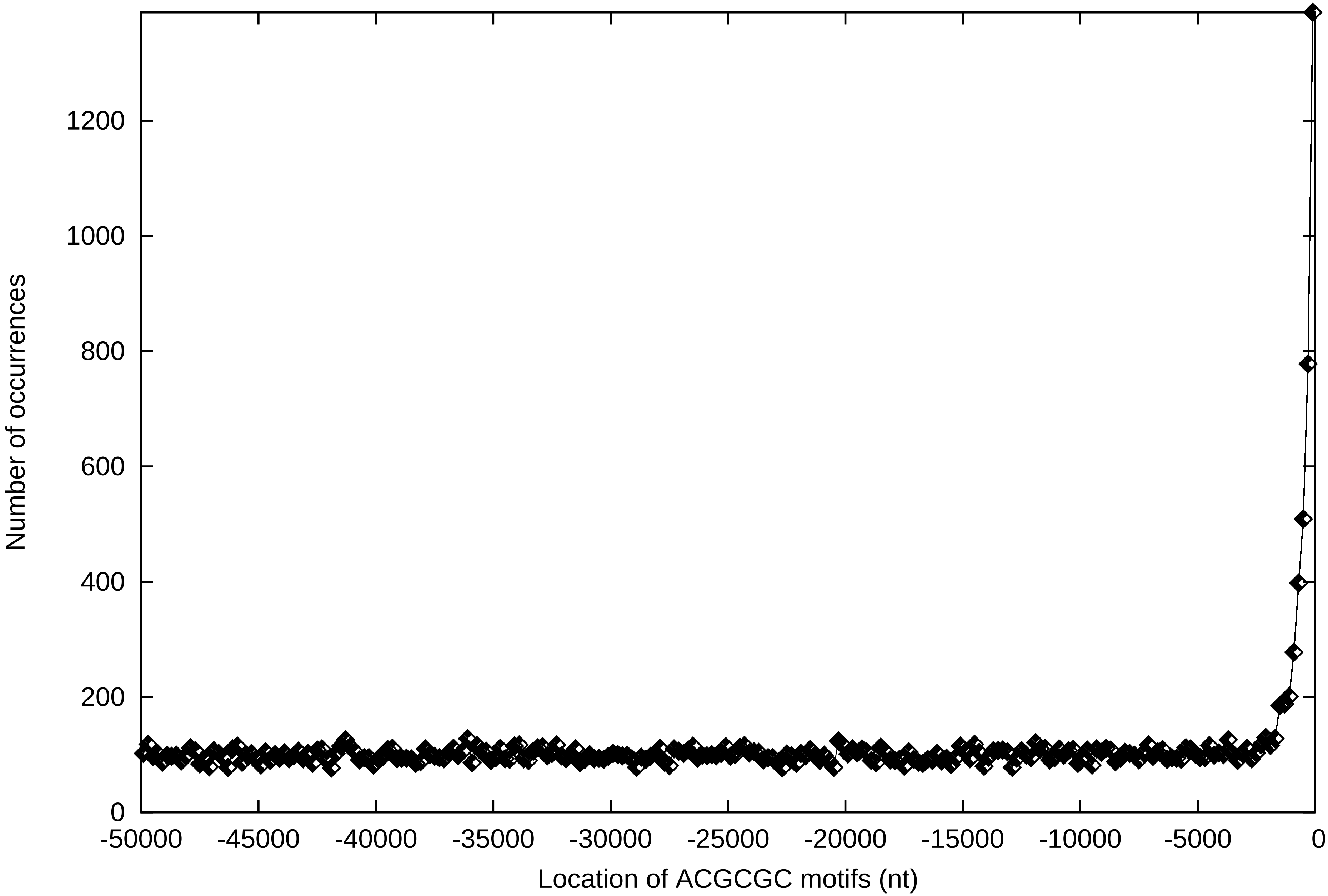

Number of occurrences
0
200
400
600
800
1000
1200
-50000
-45000
-40000
-35000
-30000
-25000
-20000
-15000
-10000
-5000
0
Location of ACGCGC motifs (nt)

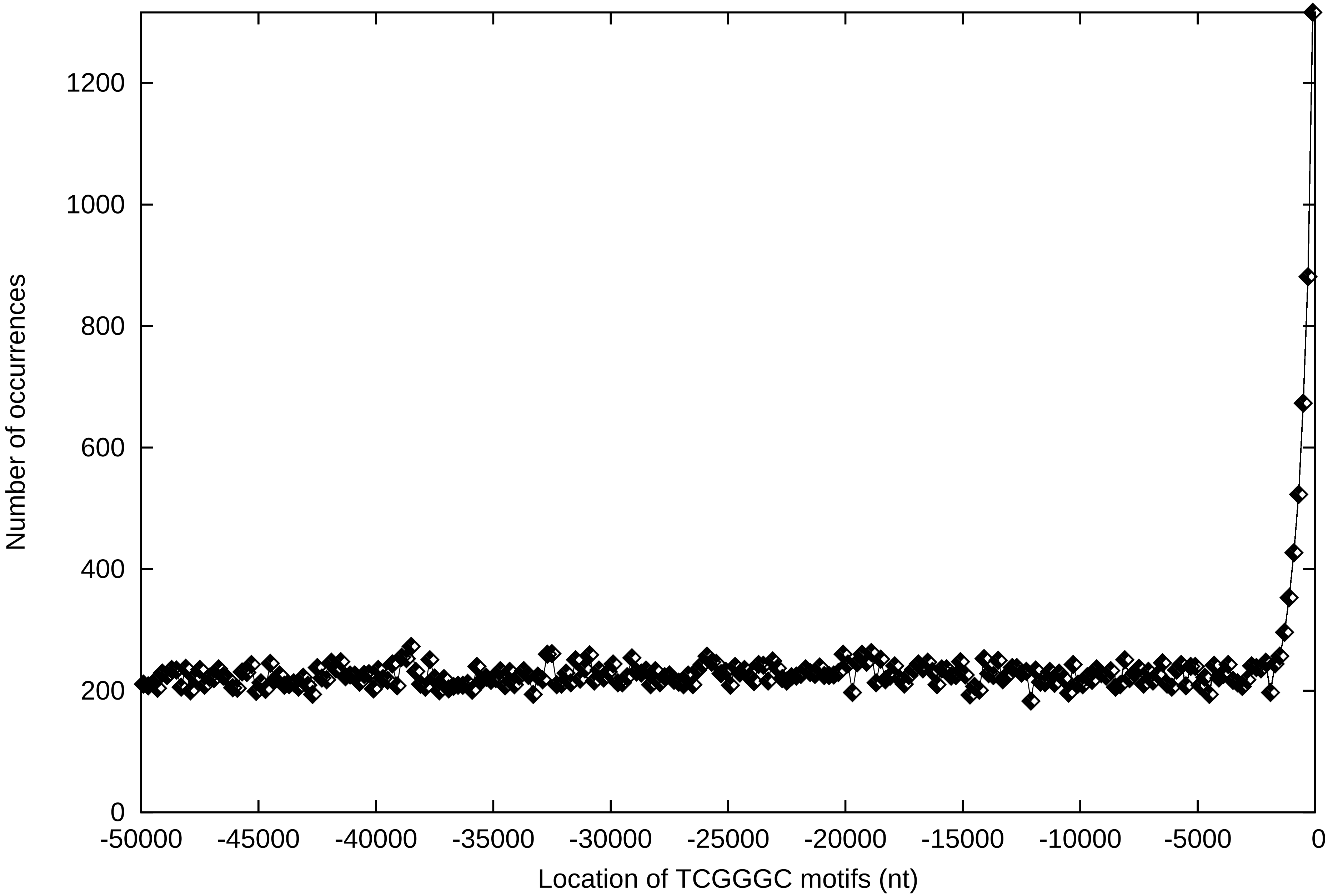

Number of occurrences
0
200
400
600
800
1000
1200
-50000
-45000
-40000
-35000
-30000
-25000
-20000
-15000
-10000
-5000
0
Location of TCGGGC motifs (nt)

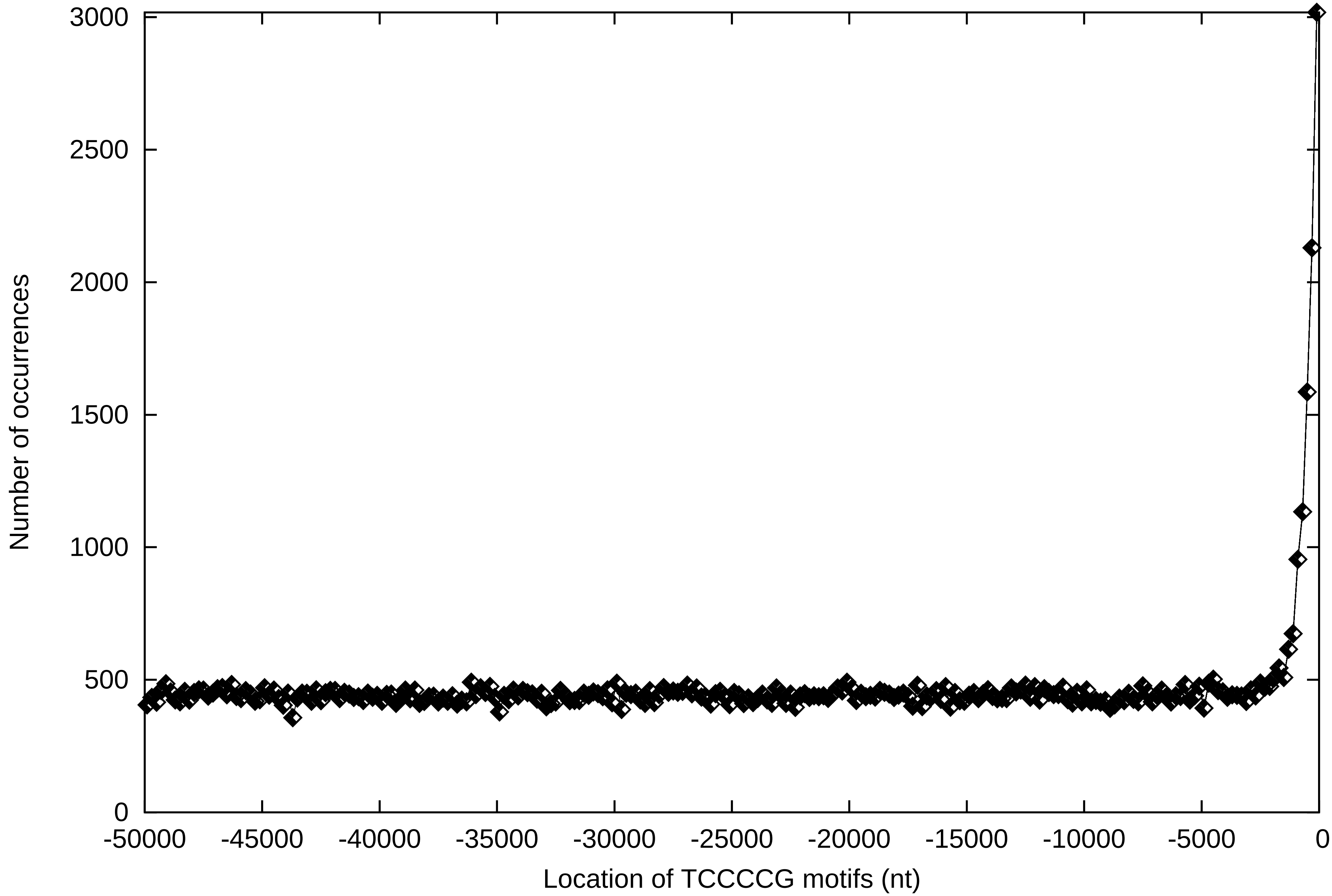
Number of occurrences
0
500
1000
1500
2000
2500
3000
-50000
-45000
-40000
-35000
-30000
-25000
-20000
-15000
-10000
-5000
0
Location of TCCCCG motifs (nt)

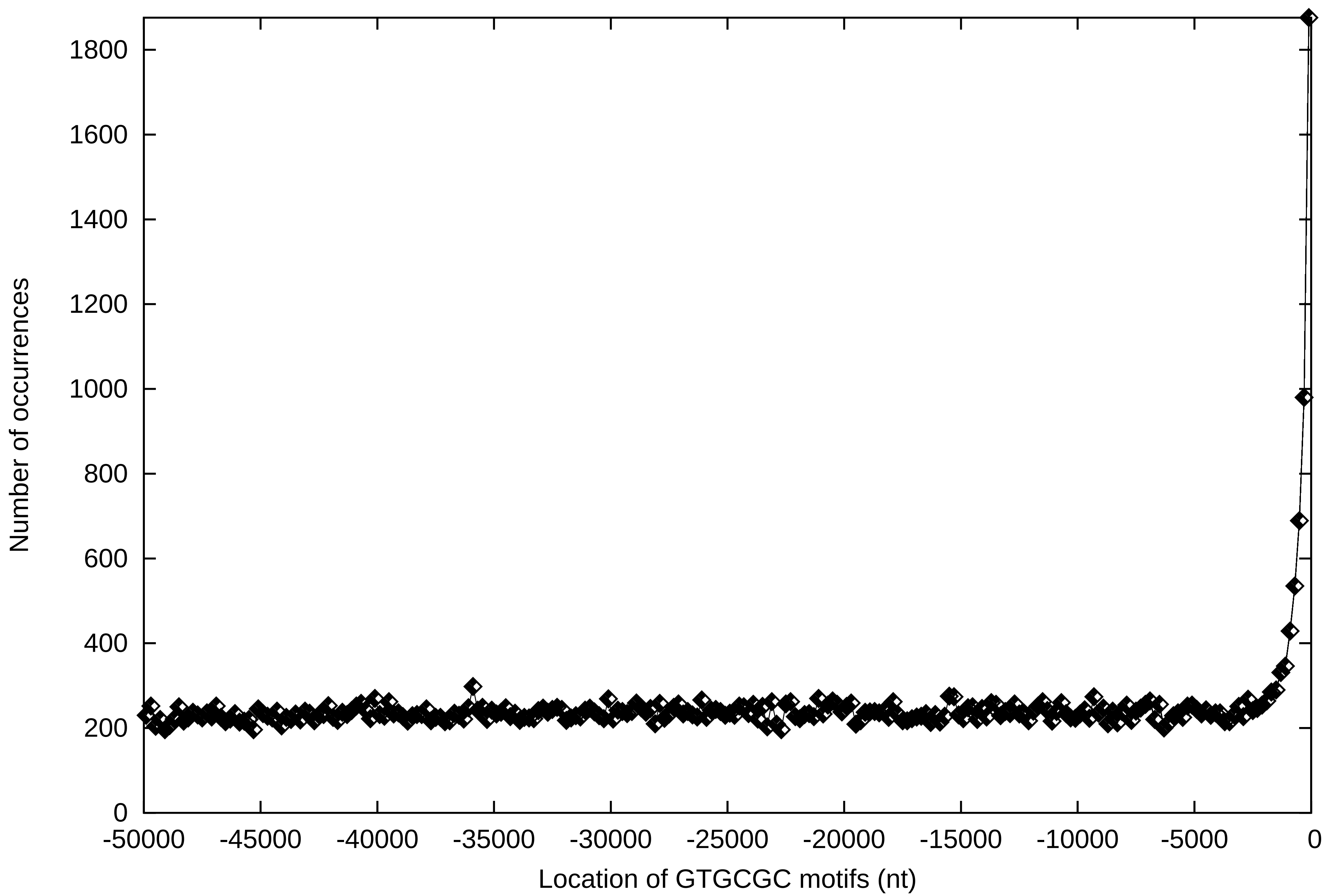

Number of occurrences
0
200
400
600
800
1000
1200
1400
1600
1800
-50000
-45000
-40000
-35000
-30000
-25000
-20000
-15000
-10000
-5000
0
Location of GTGCGC motifs (nt)

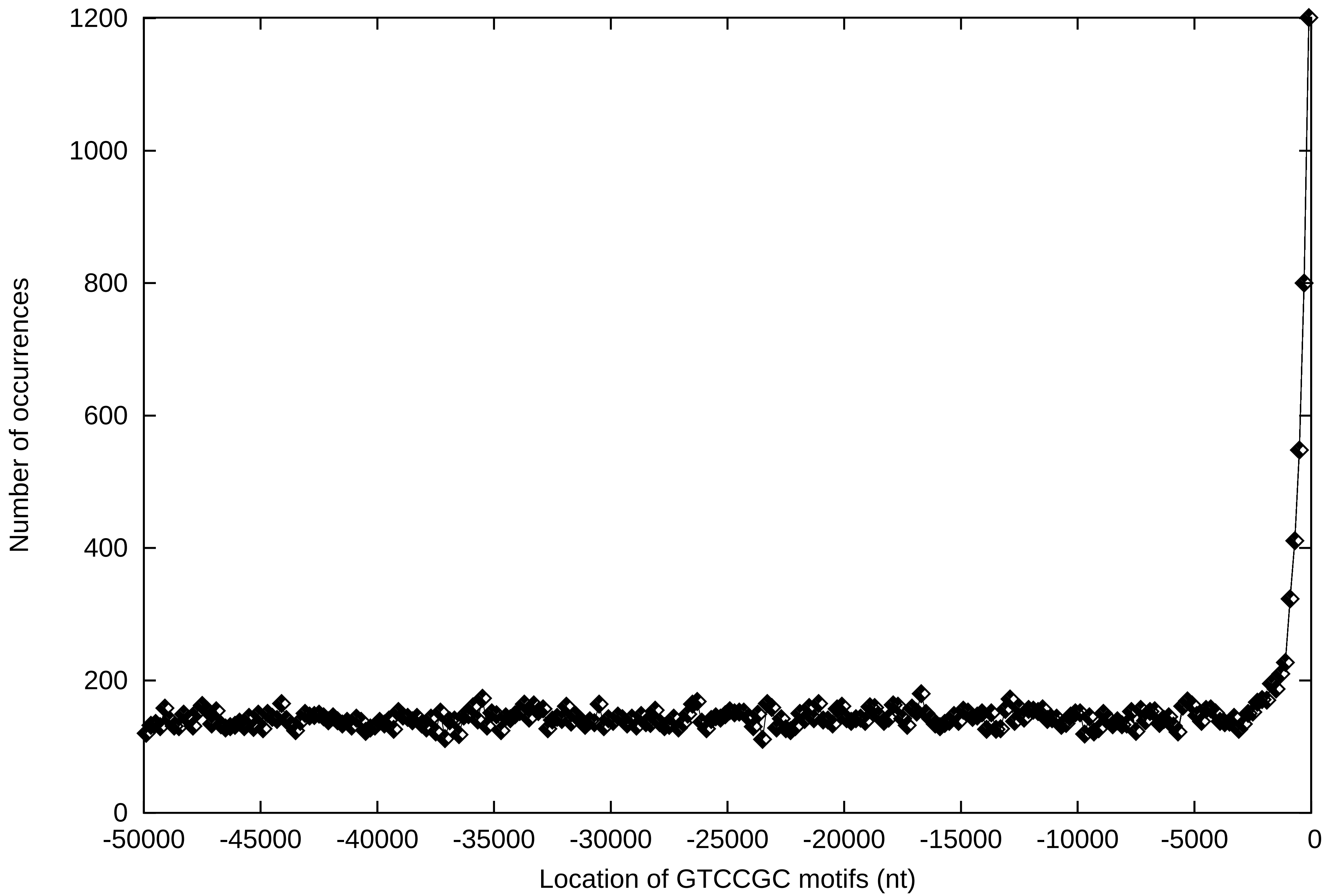

1200
1000
800
600
400
200
0
Number of occurrences
-50000
-45000
-40000
-35000
-30000
-25000
-20000
-15000
-10000
-5000
0
Location of GTCCGC motifs (nt)

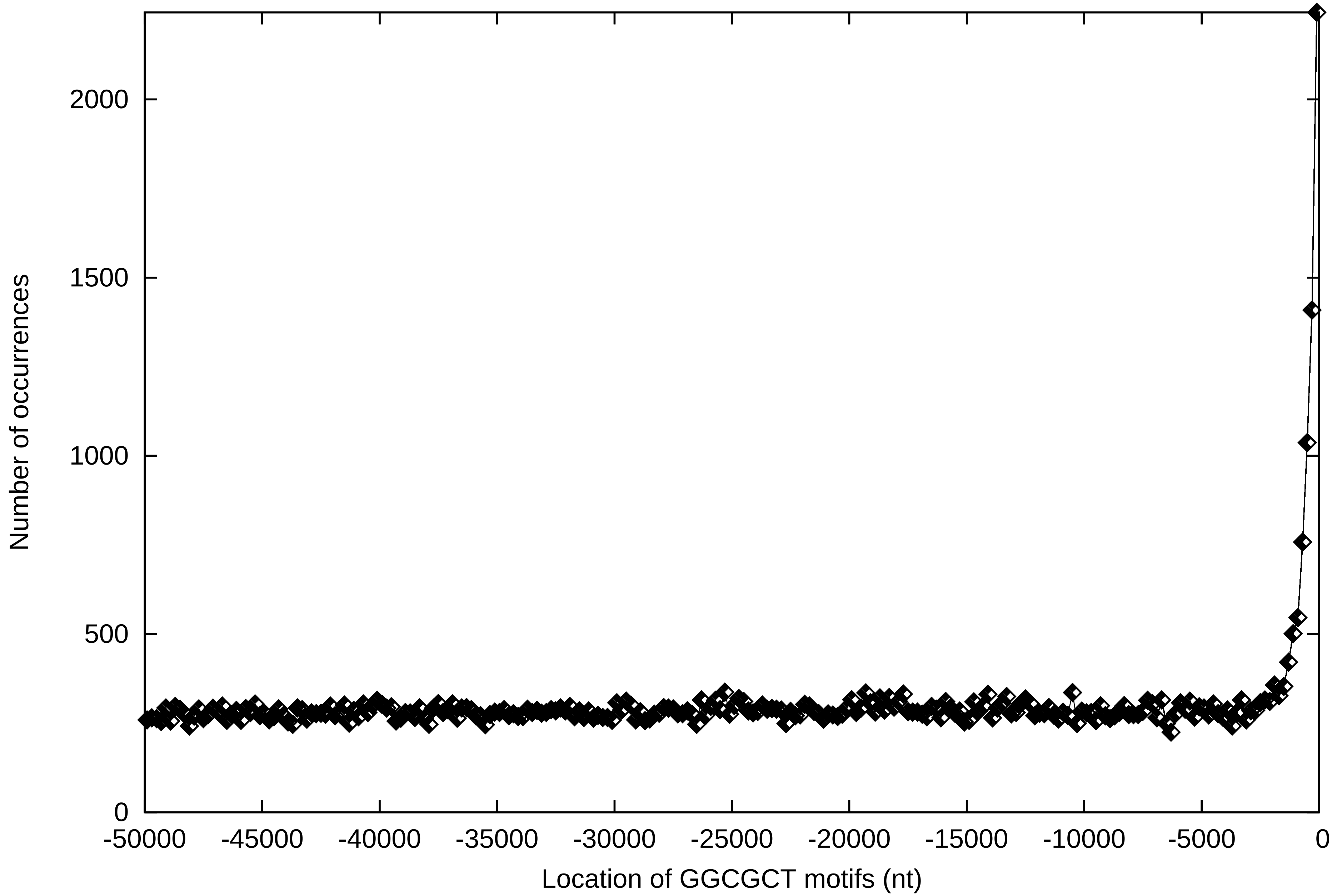

Number of occurrences
2000
1500
1000
500
0
-50000
-45000
-40000
-35000
-30000
-25000
-20000
-15000
-10000
-5000
0
Location of GGCGCT motifs (nt)

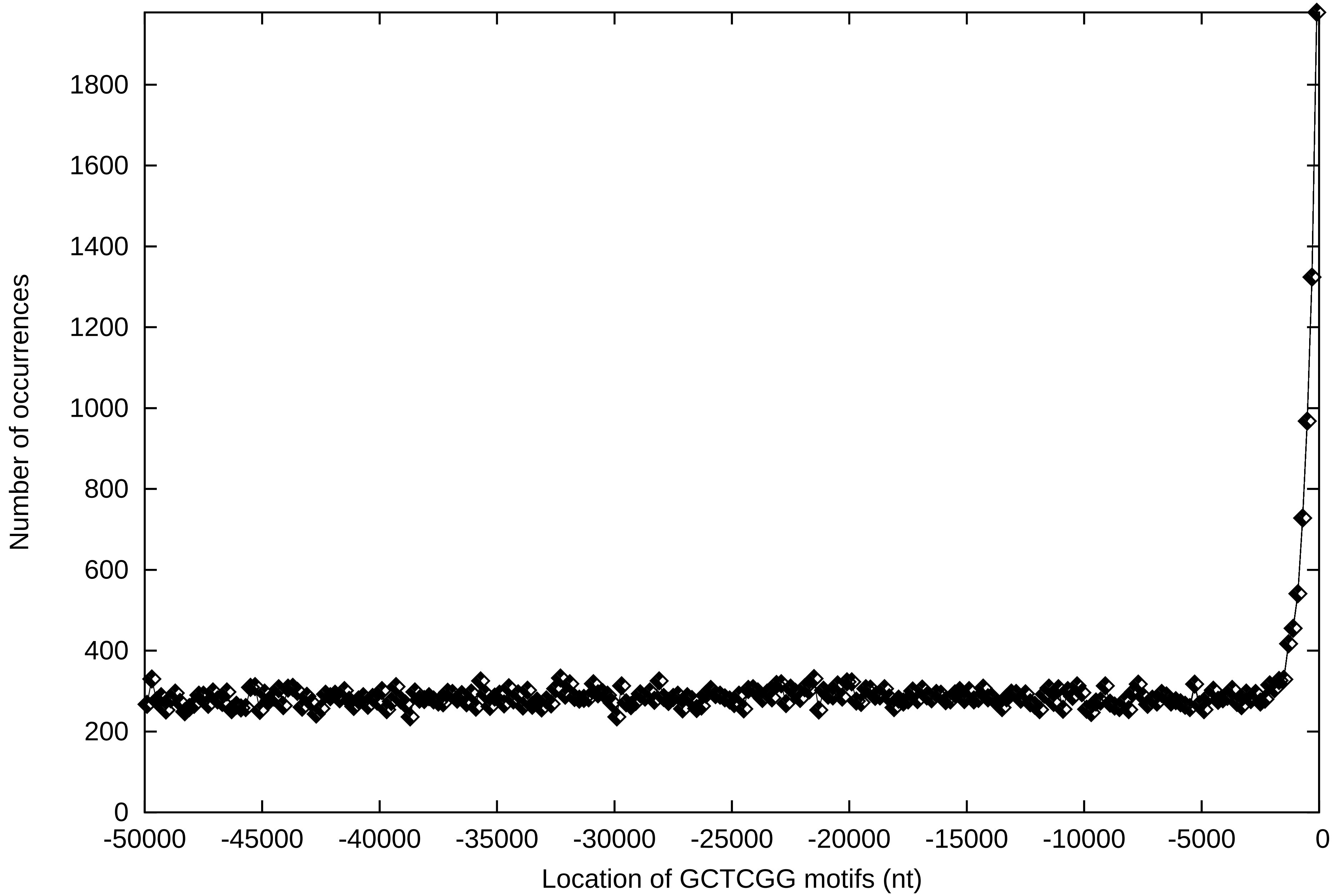

Number of occurrences
0
200
400
600
800
1000
1200
1400
1600
1800
-50000
-45000
-40000
-35000
-30000
-25000
-20000
-15000
-10000
-5000
0
Location of GCTCGG motifs (nt)

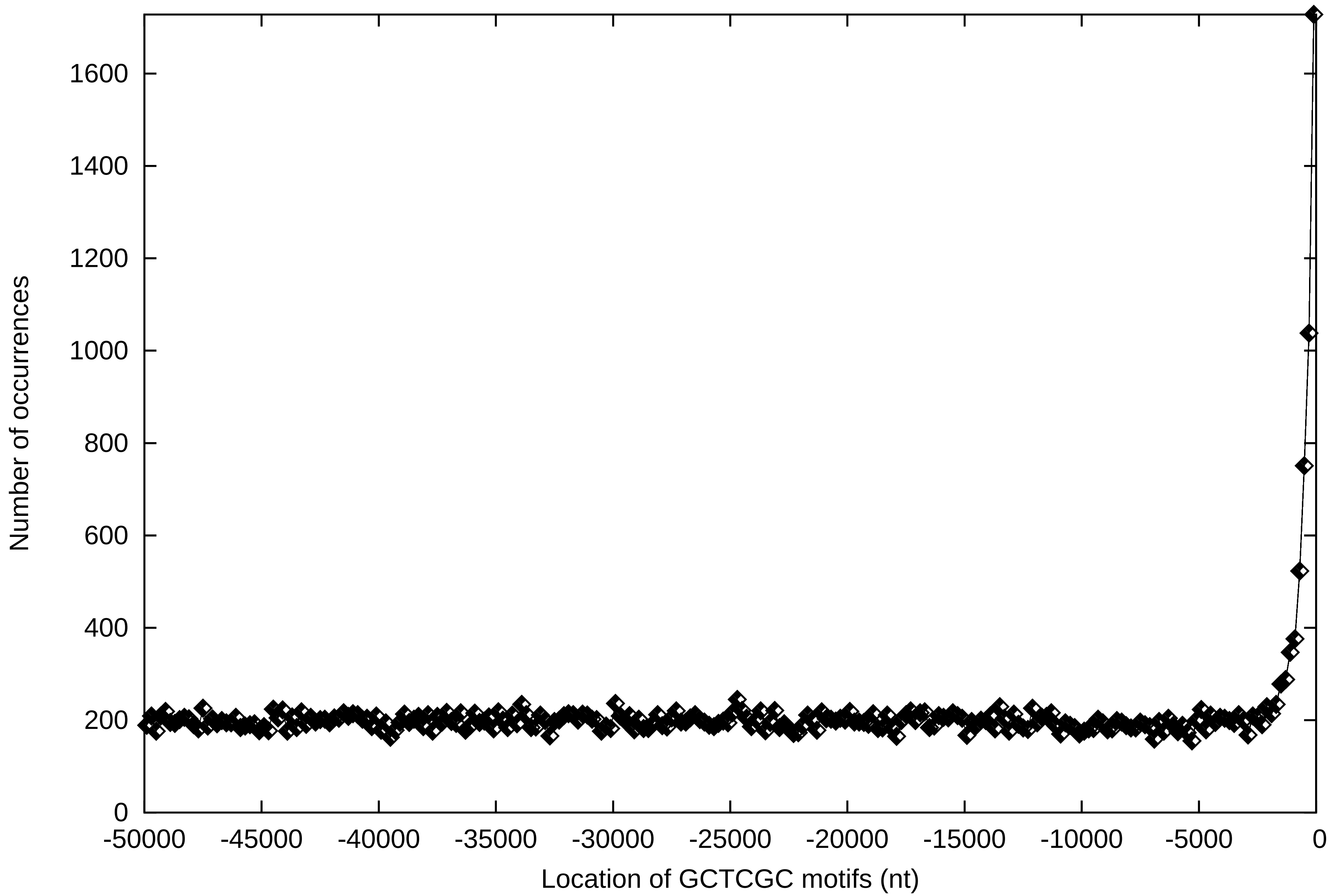

Number of occurrences
0
200
400
600
800
1000
1200
1400
1600
-50000
-45000
-40000
-35000
-30000
-25000
-20000
-15000
-10000
-5000
0
Location of GCTCGC motifs (nt)

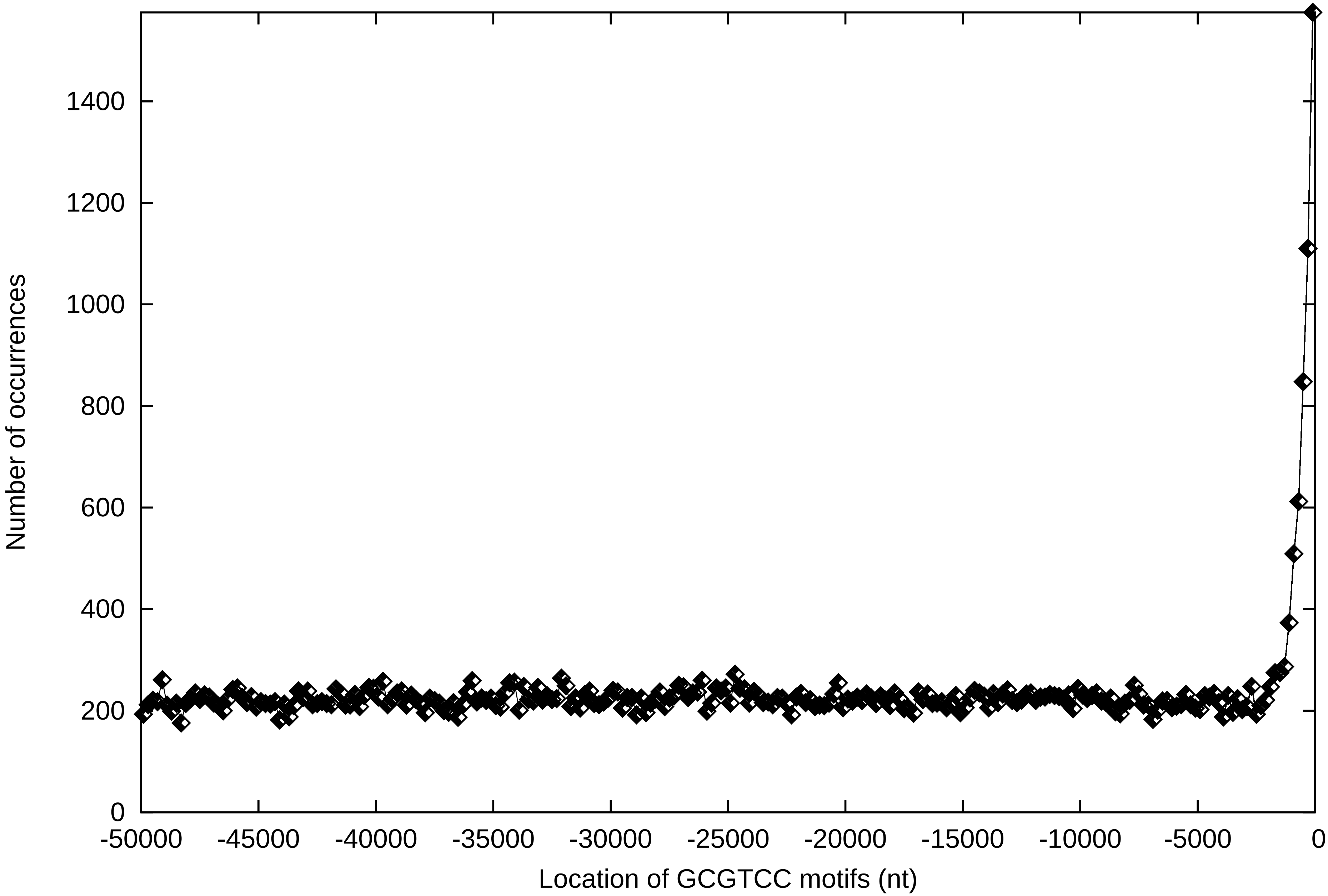

Number of occurrences
0
200
400
600
800
1000
1200
1400
-50000
-45000
-40000
-35000
-30000
-25000
-20000
-15000
-10000
-5000
0
Location of GCGTCC motifs (nt)

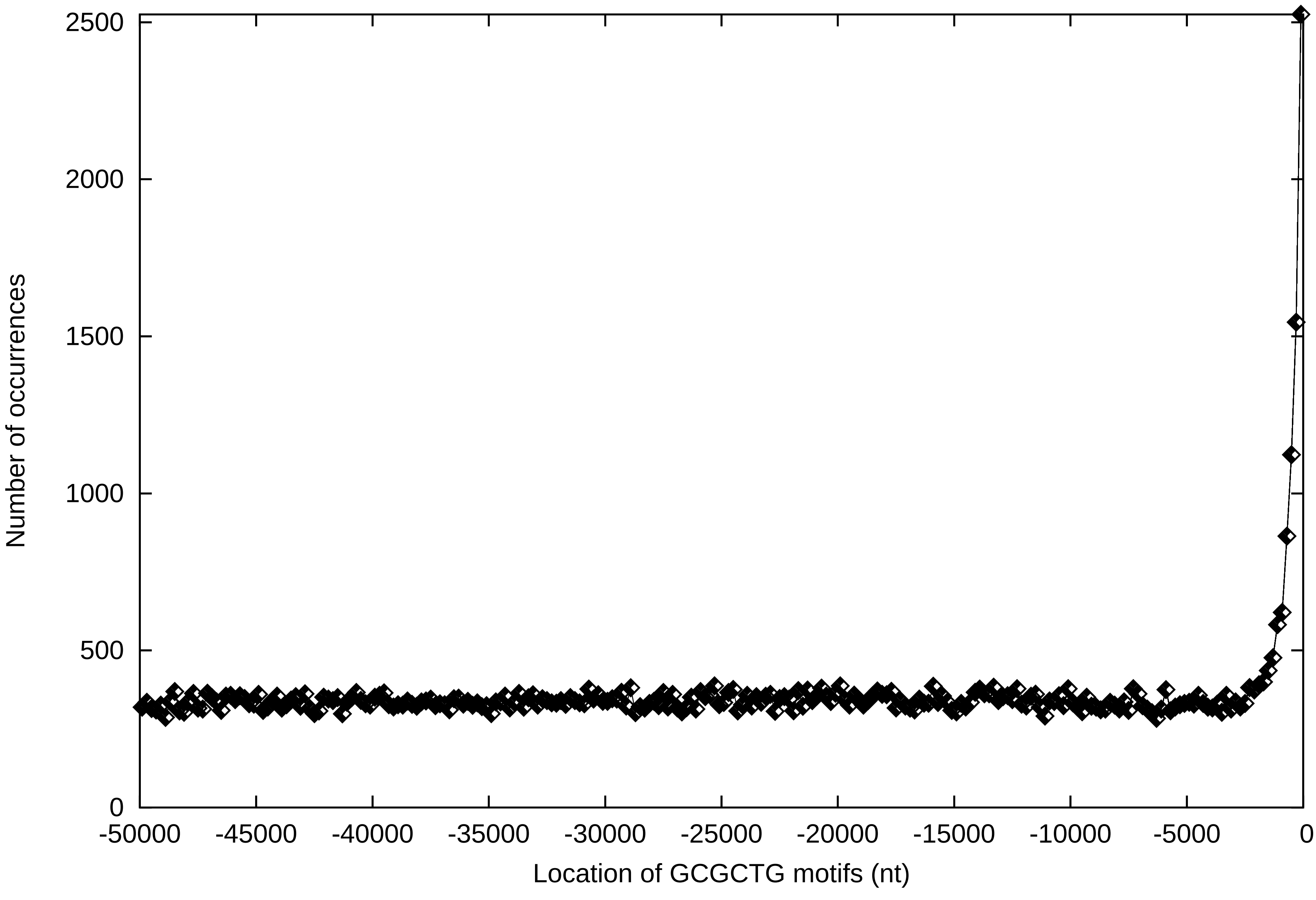
Number of occurrences
2500
2000
1500
1000
500
0
-50000
-45000
-40000
-35000
-30000
-25000
-20000
-15000
-10000
-5000
0
Location of GCGCTG motifs (nt)

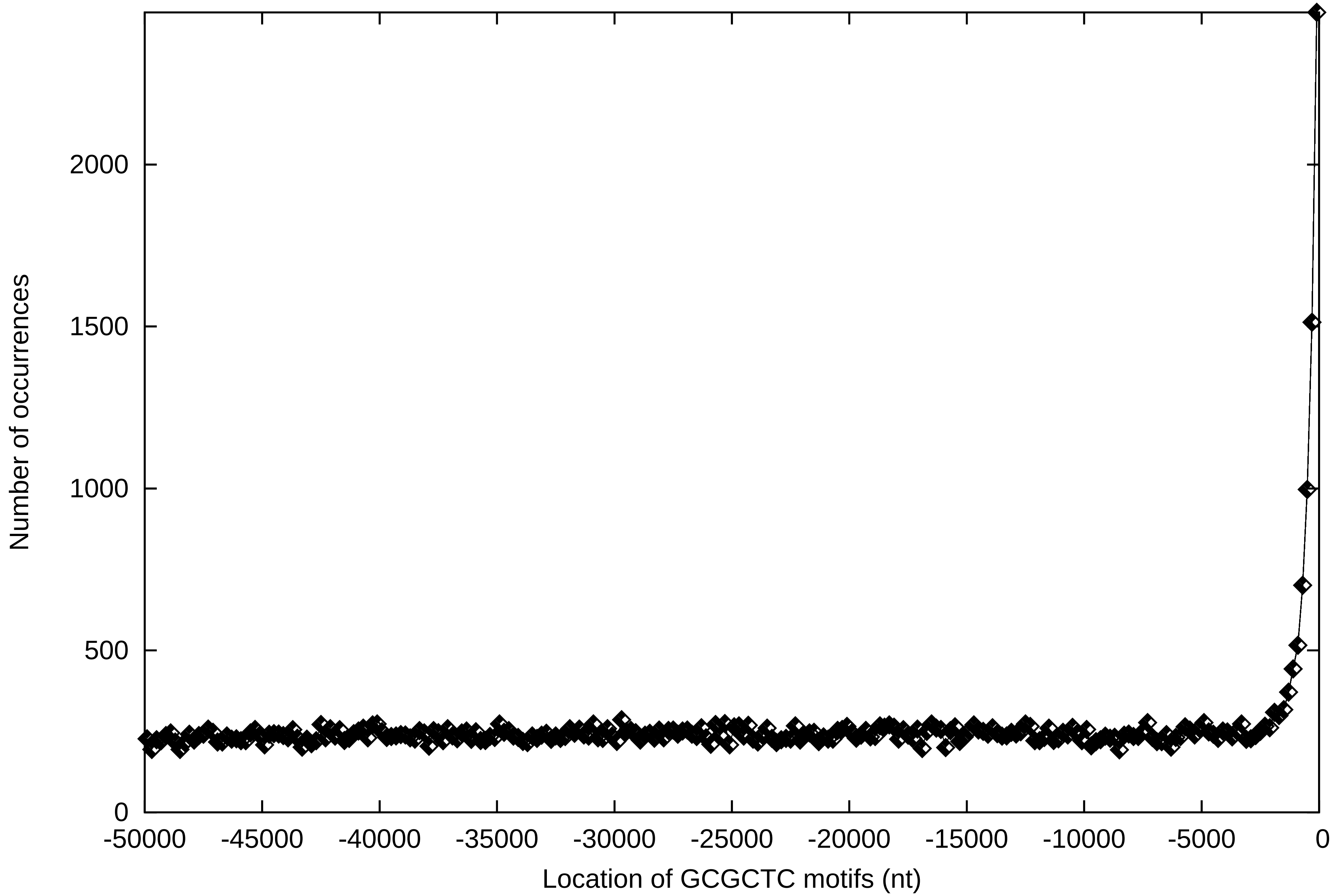

Number of occurrences
0
500
1000
1500
2000
-50000
-45000
-40000
-35000
-30000
-25000
-20000
-15000
-10000
-5000
0
Location of GCGCTC motifs (nt)

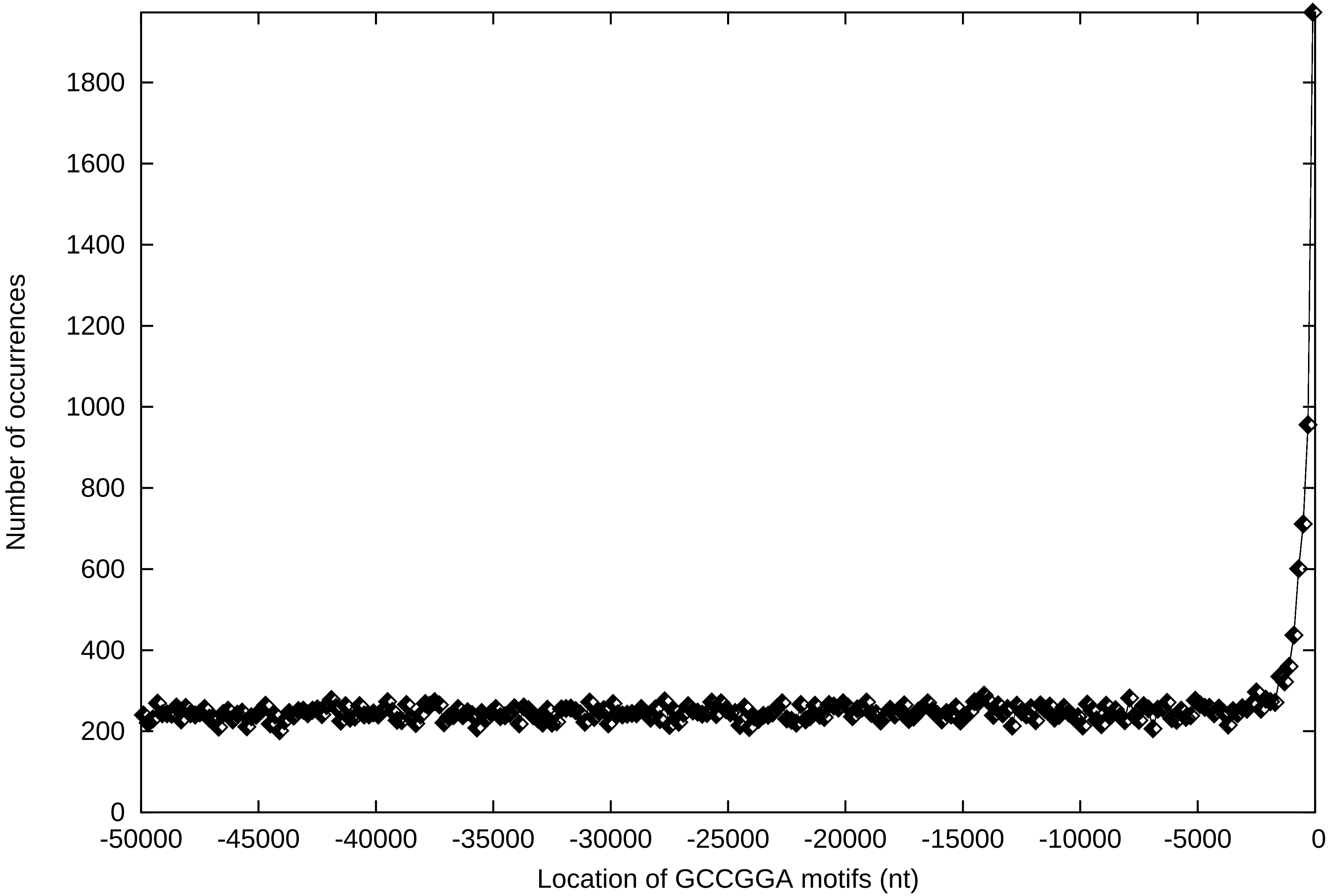
Number of occurrences
0
200
400
600
800
1000
1200
1400
1600
1800
-50000
-45000
-40000
-35000
-30000
-25000
-20000
-15000
-10000
-5000
0
Location of GCCGGA motifs (nt)

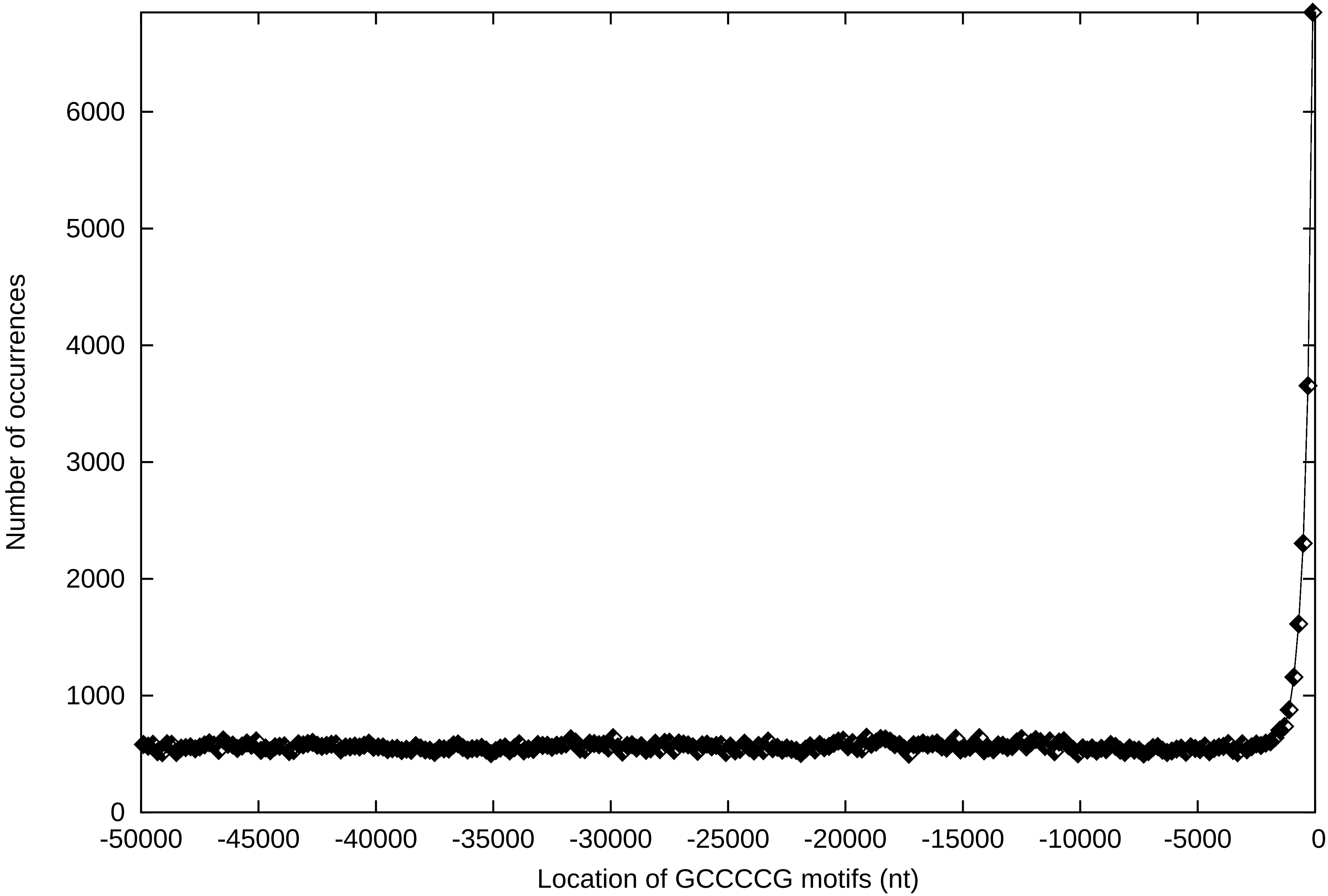

Number of occurrences
0
1000
2000
3000
4000
5000
6000
-50000
-45000
-40000
-35000
-30000
-25000
-20000
-15000
-10000
-5000
0
Location of GCCCCG motifs (nt)

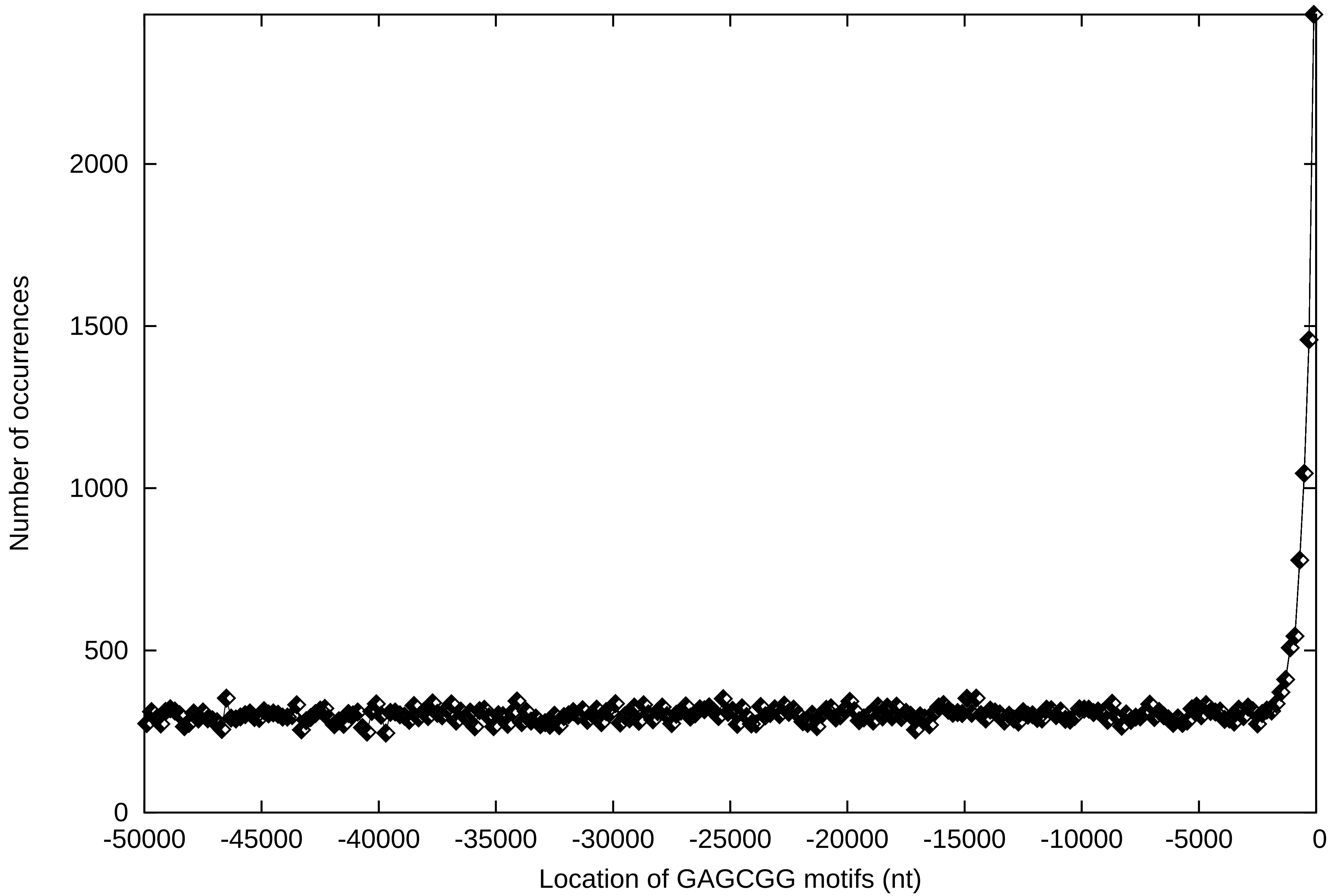
Number of occurrences
0
500
1000
1500
2000
-50000
-45000
-40000
-35000
-30000
-25000
-20000
-15000
-10000
-5000
0
Location of GAGCGG motifs (nt)

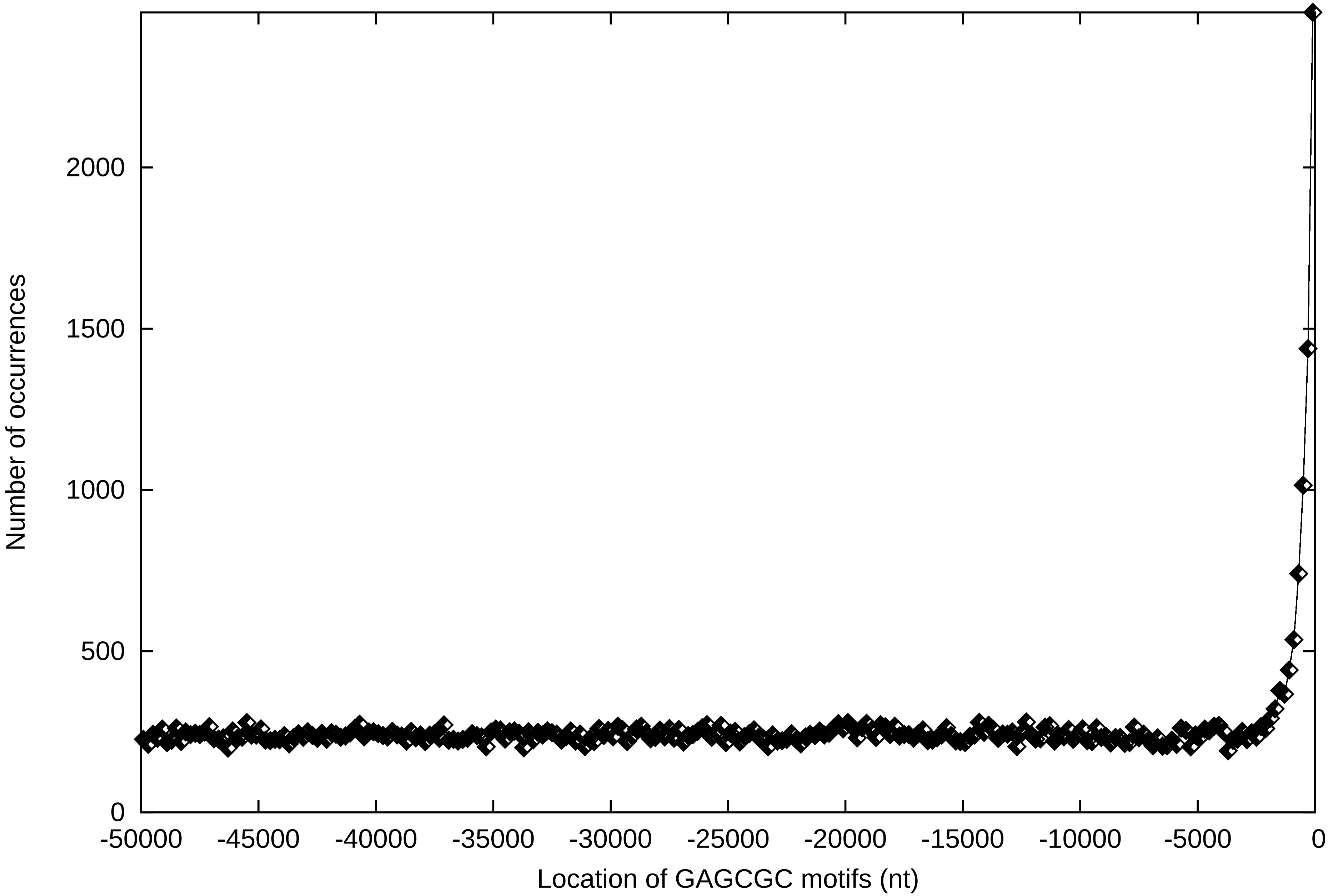
Number of occurrences
0
500
1000
1500
2000
-50000
-45000
-40000
-35000
-30000
-25000
-20000
-15000
-10000
-5000
0
Location of GAGCGC motifs (nt)

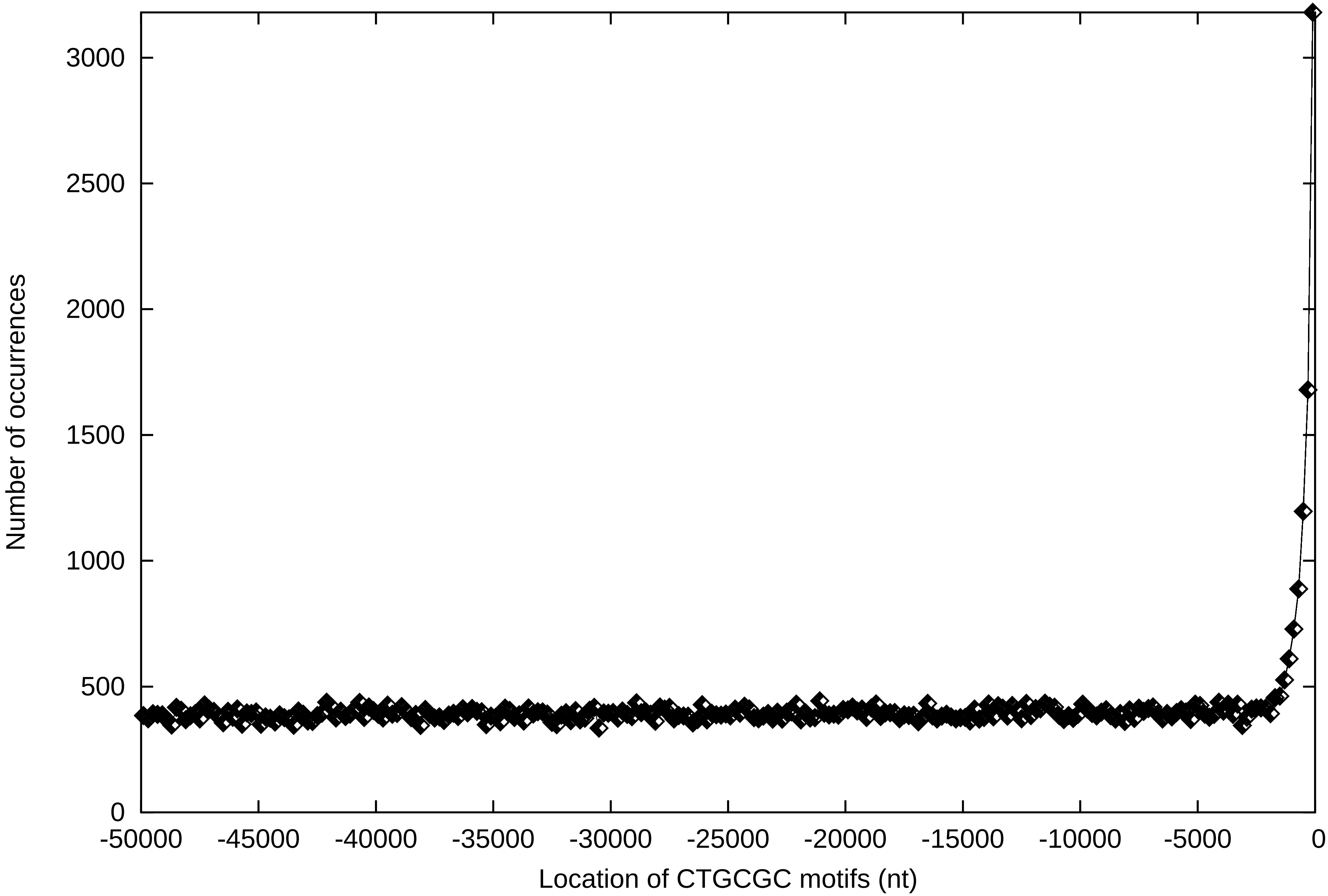

Number of occurrences
3000
2500
2000
1500
1000
500
0
-50000
-45000
-40000
-35000
-30000
-25000
-20000
-15000
-10000
-5000
0
Location of CTGCGC motifs (nt)

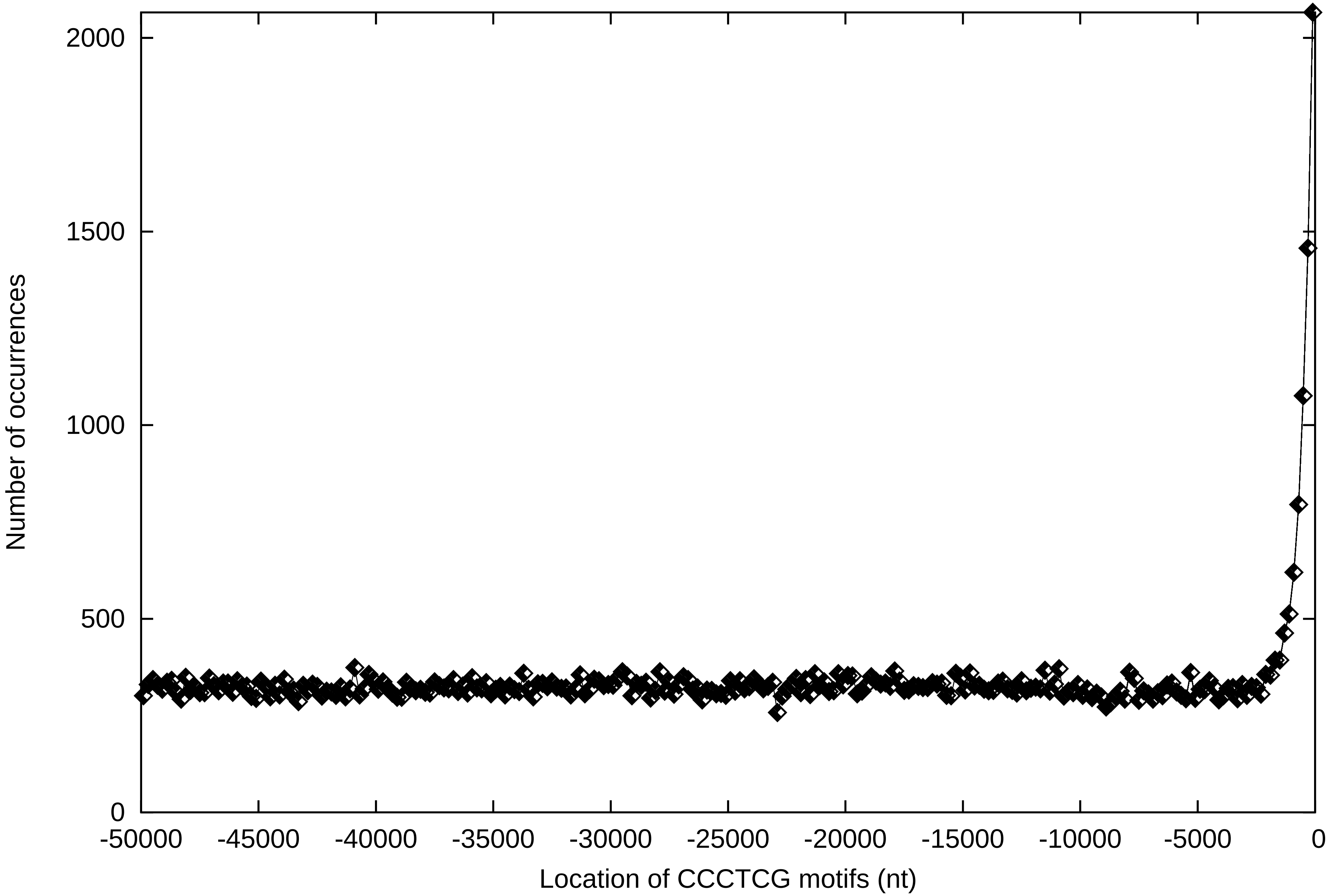

Number of occurrences
0
500
1000
1500
2000
-50000
-45000
-40000
-35000
-30000
-25000
-20000
-15000
-10000
-5000
0
Location of CCCTCG motifs (nt)

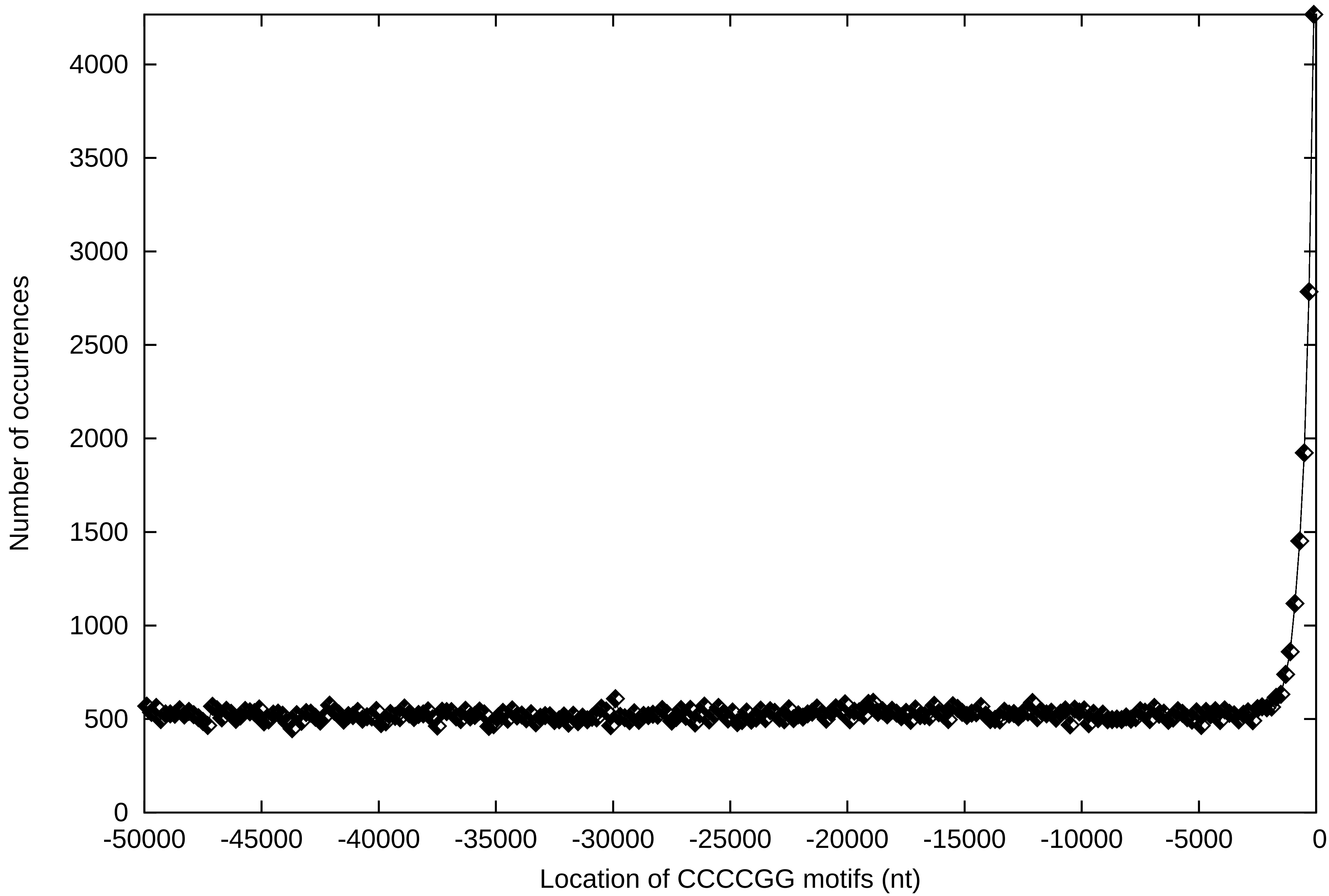

Number of occurrences
4000
3500
3000
2500
2000
1500
1000
500
0
-50000
-45000
-40000
-35000
-30000
-25000
-20000
-15000
-10000
-5000
0
Location of CCCCGG motifs (nt)

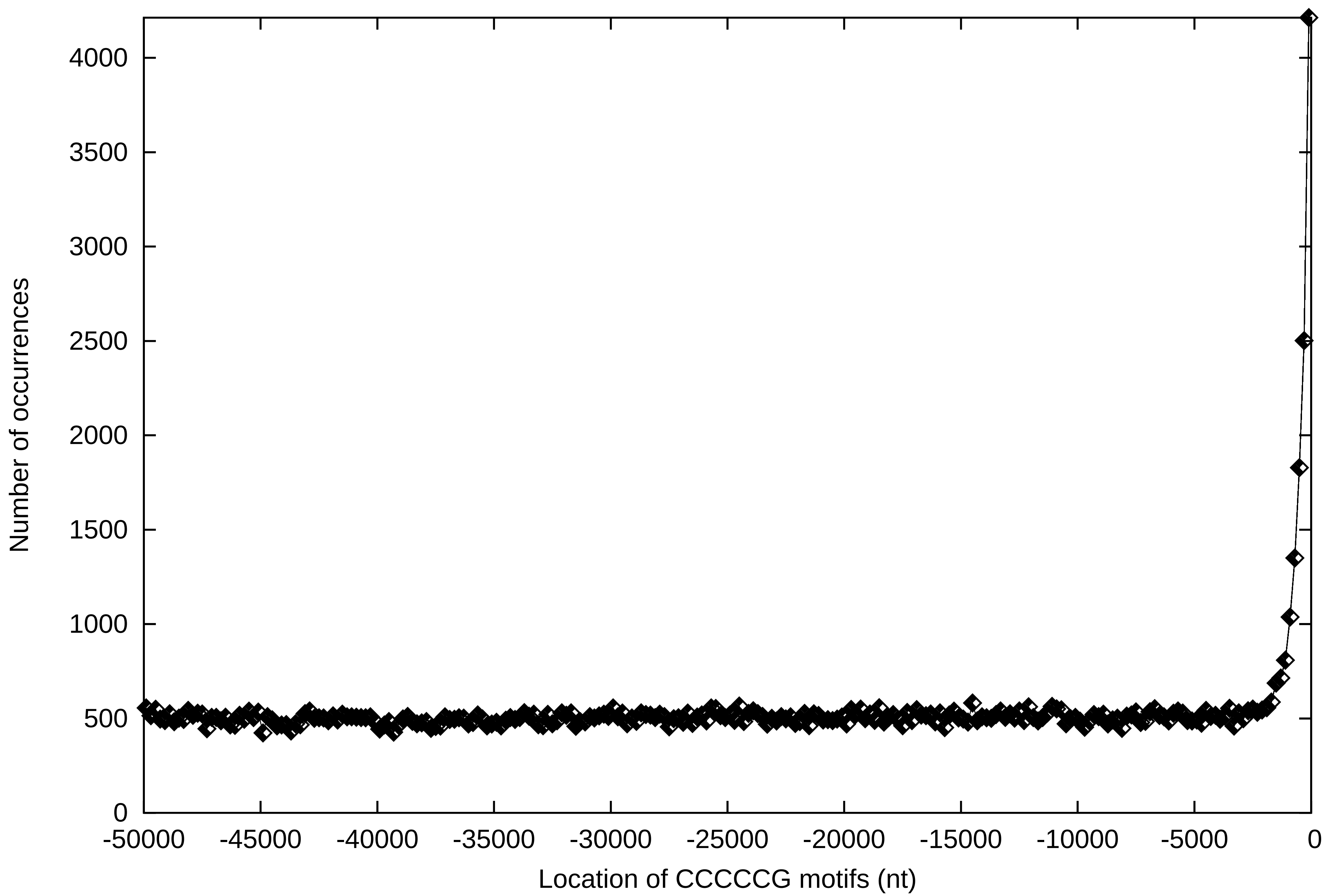

Number of occurrences
0
500
1000
1500
2000
2500
3000
3500
4000
-50000
-45000
-40000
-35000
-30000
-25000
-20000
-15000
-10000
-5000
0
Location of CCCCCG motifs (nt)

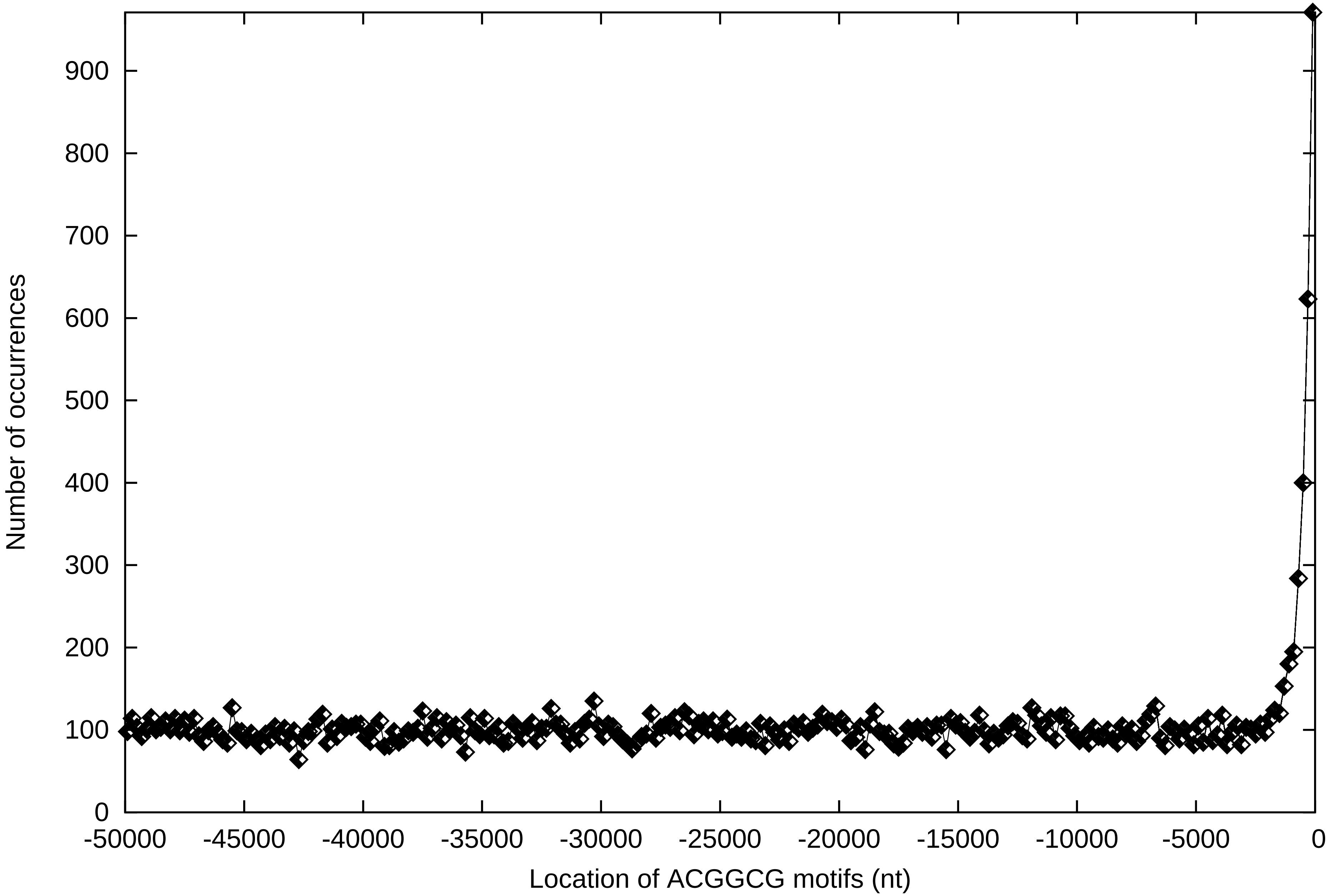

Number of occurrences
0
100
200
300
400
500
600
700
800
900
-50000
-45000
-40000
-35000
-30000
-25000
-20000
-15000
-10000
-5000
0
Location of ACGGCG motifs (nt)

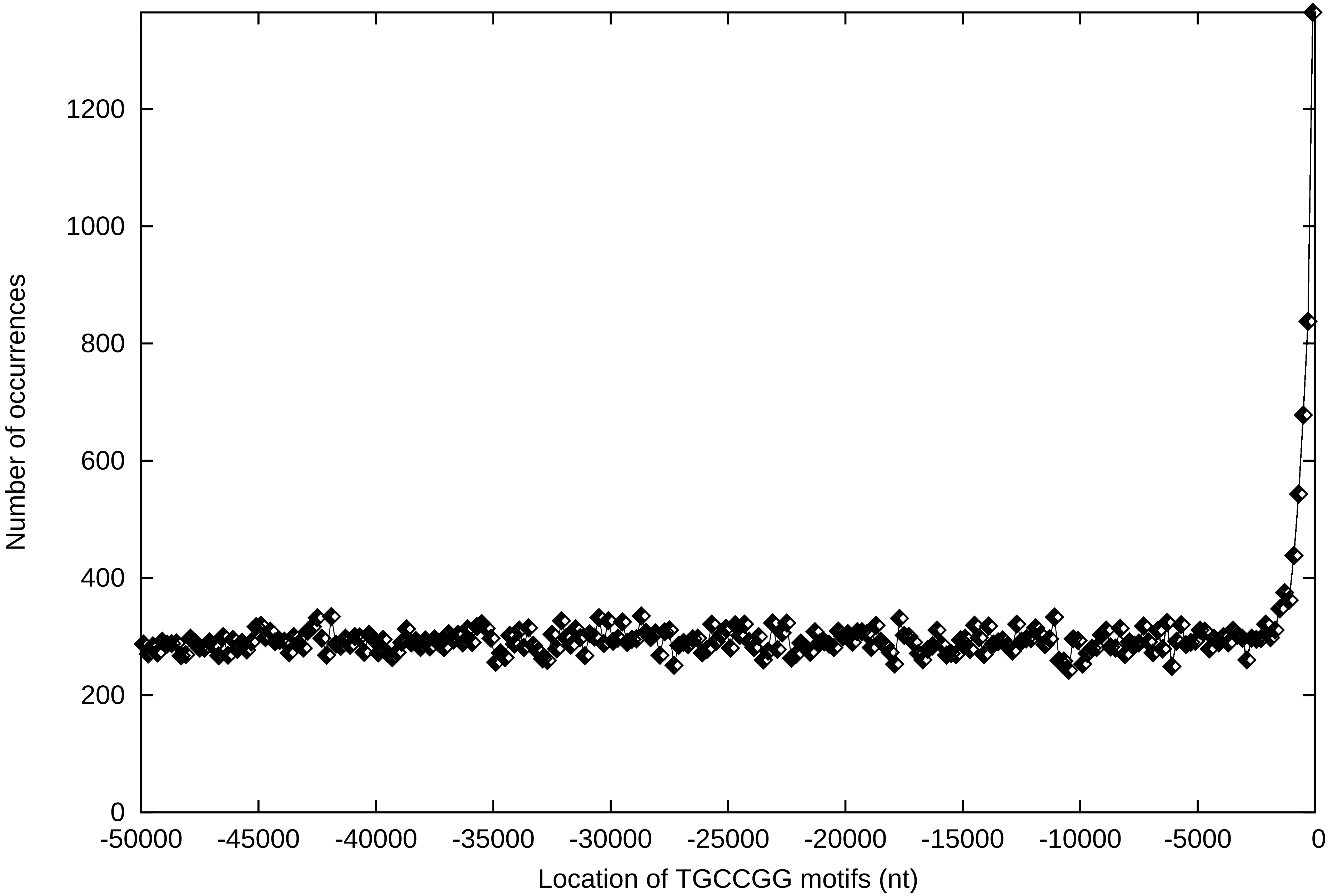
Number of occurrences
Location of TGCCGG motifs (nt)
0
200
400
600
800
1000
1200
-50000
-45000
-40000
-35000
-30000
-25000
-20000
-15000
-10000
-5000
0

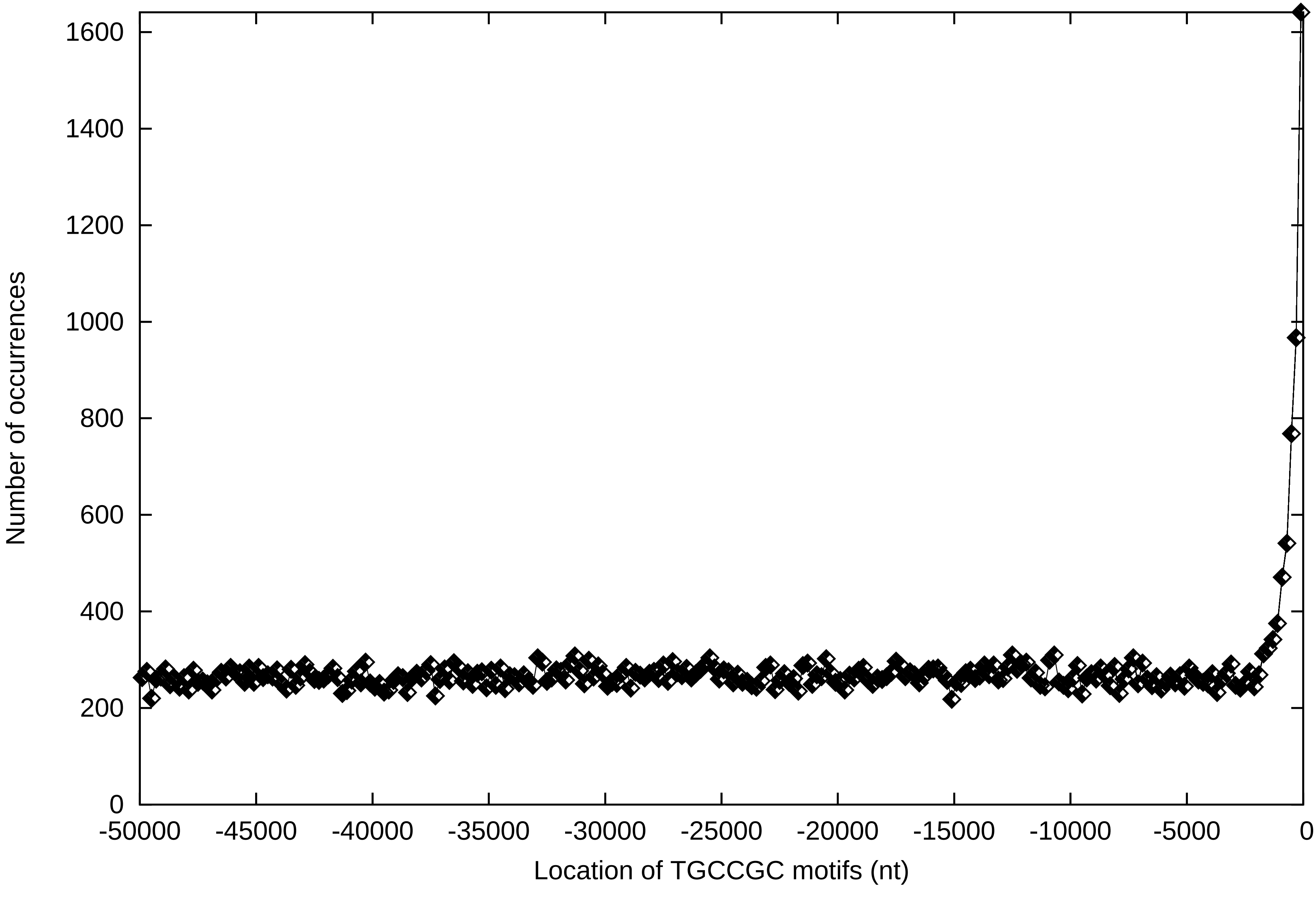

Number of occurrences
1600
1400
1200
1000
800
600
400
200
0
-50000
-45000
-40000
-35000
-30000
-25000
-20000
-15000
-10000
-5000
0
Location of TGCCGC motifs (nt)

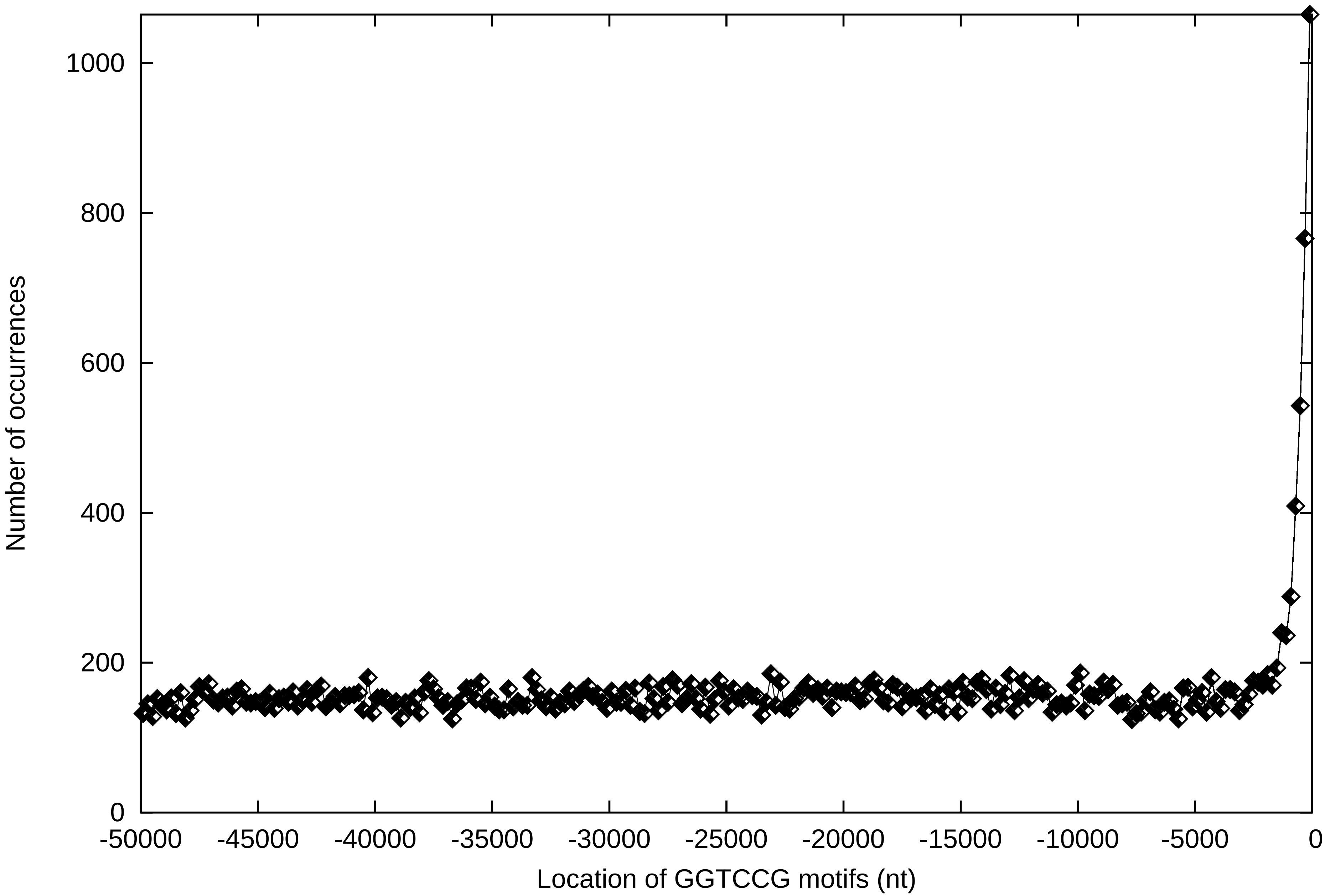

Number of occurrences
1000
800
600
400
200
0
-50000
-45000
-40000
-35000
-30000
-25000
-20000
-15000
-10000
-5000
0
Location of GGTCCG motifs (nt)

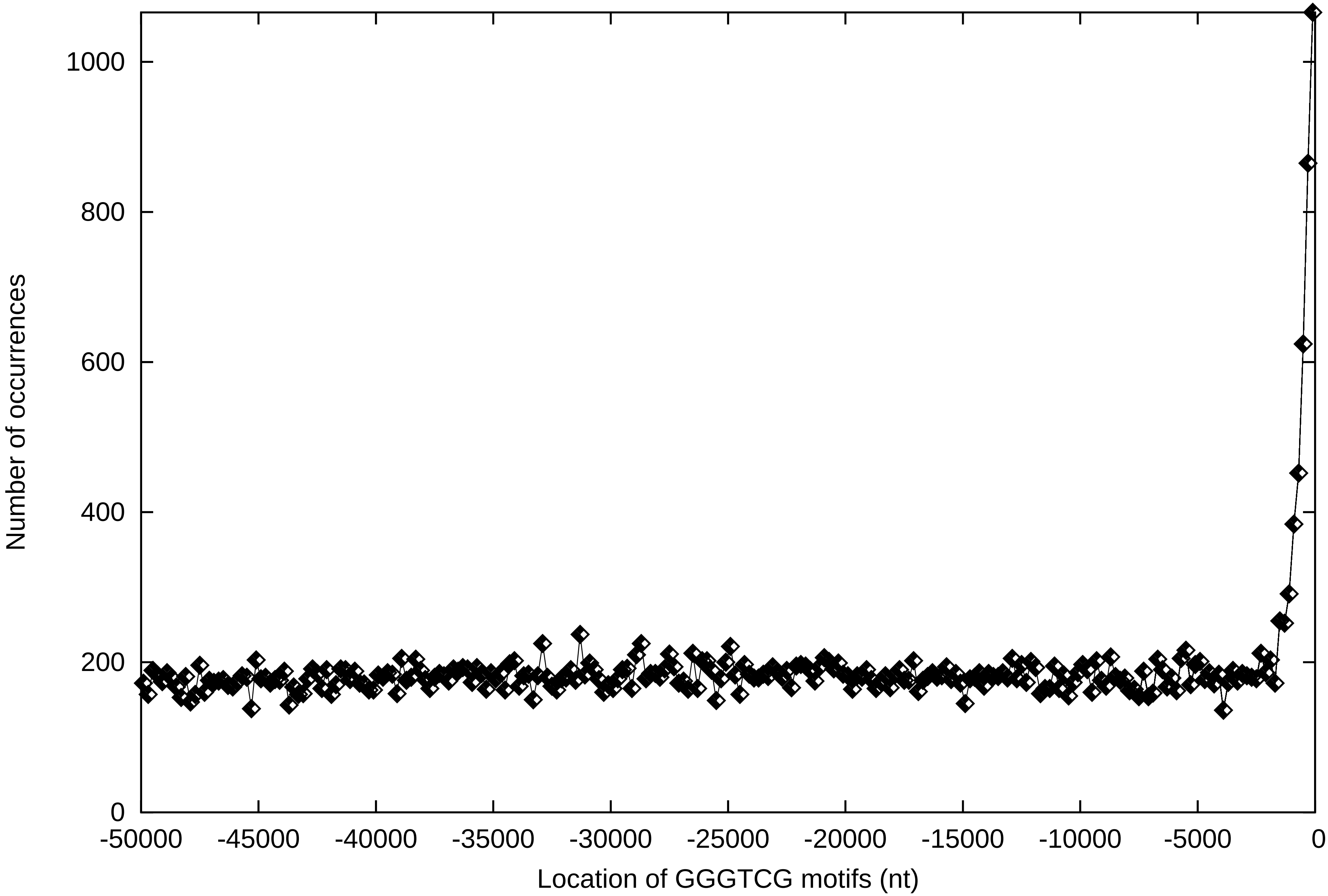

Number of occurrences
0
200
400
600
800
1000
-50000
-45000
-40000
-35000
-30000
-25000
-20000
-15000
-10000
-5000
0
Location of GGGTCG motifs (nt)

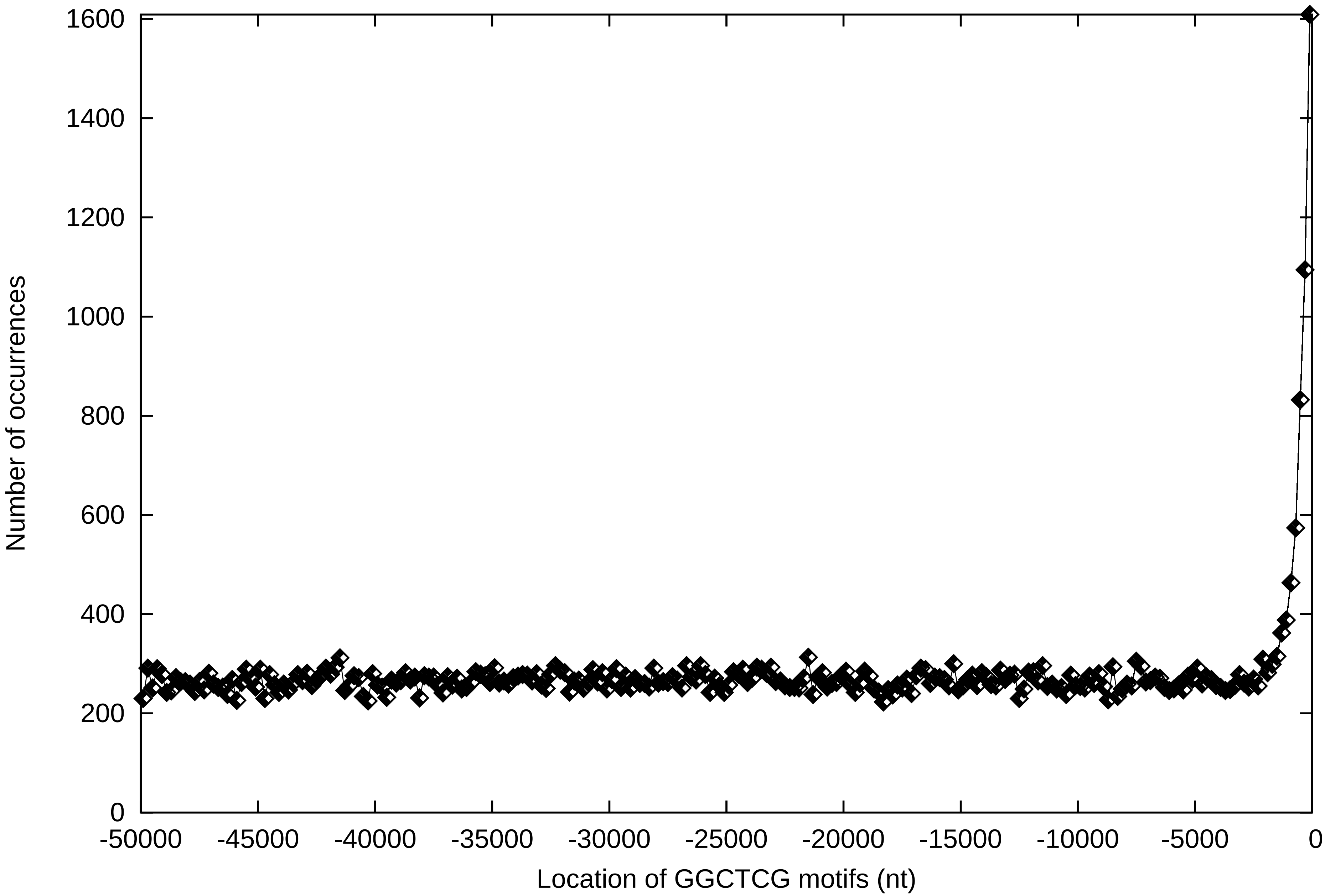
1600
1400
1200
1000
800
600
400
200
0
Number of occurrences
-50000
-45000
-40000
-35000
-30000
-25000
-20000
-15000
-10000
-5000
0
Location of GGCTCG motifs (nt)

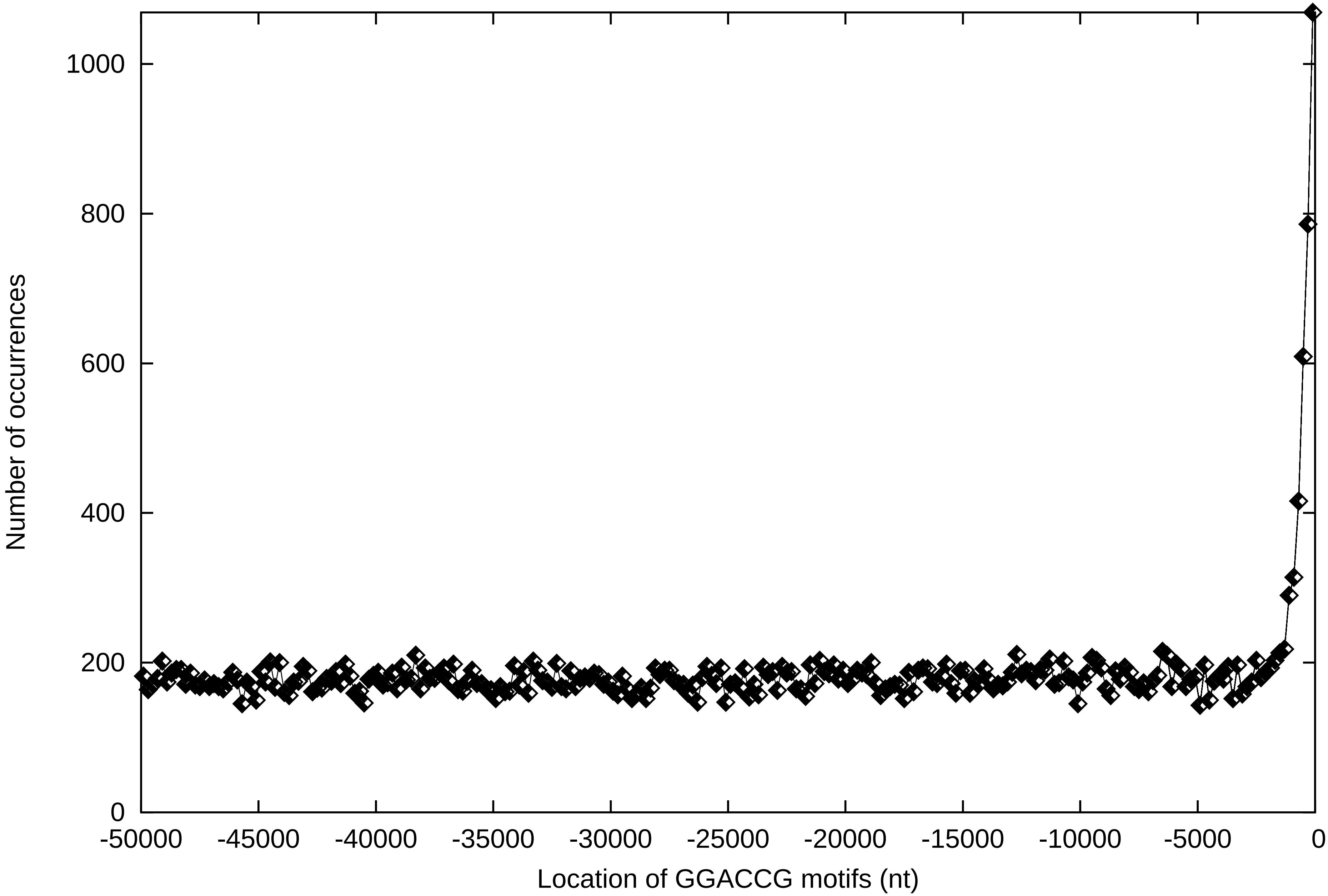

Number of occurrences
0
200
400
600
800
1000
-50000
-45000
-40000
-35000
-30000
-25000
-20000
-15000
-10000
-5000
0
Location of GGACCG motifs (nt)

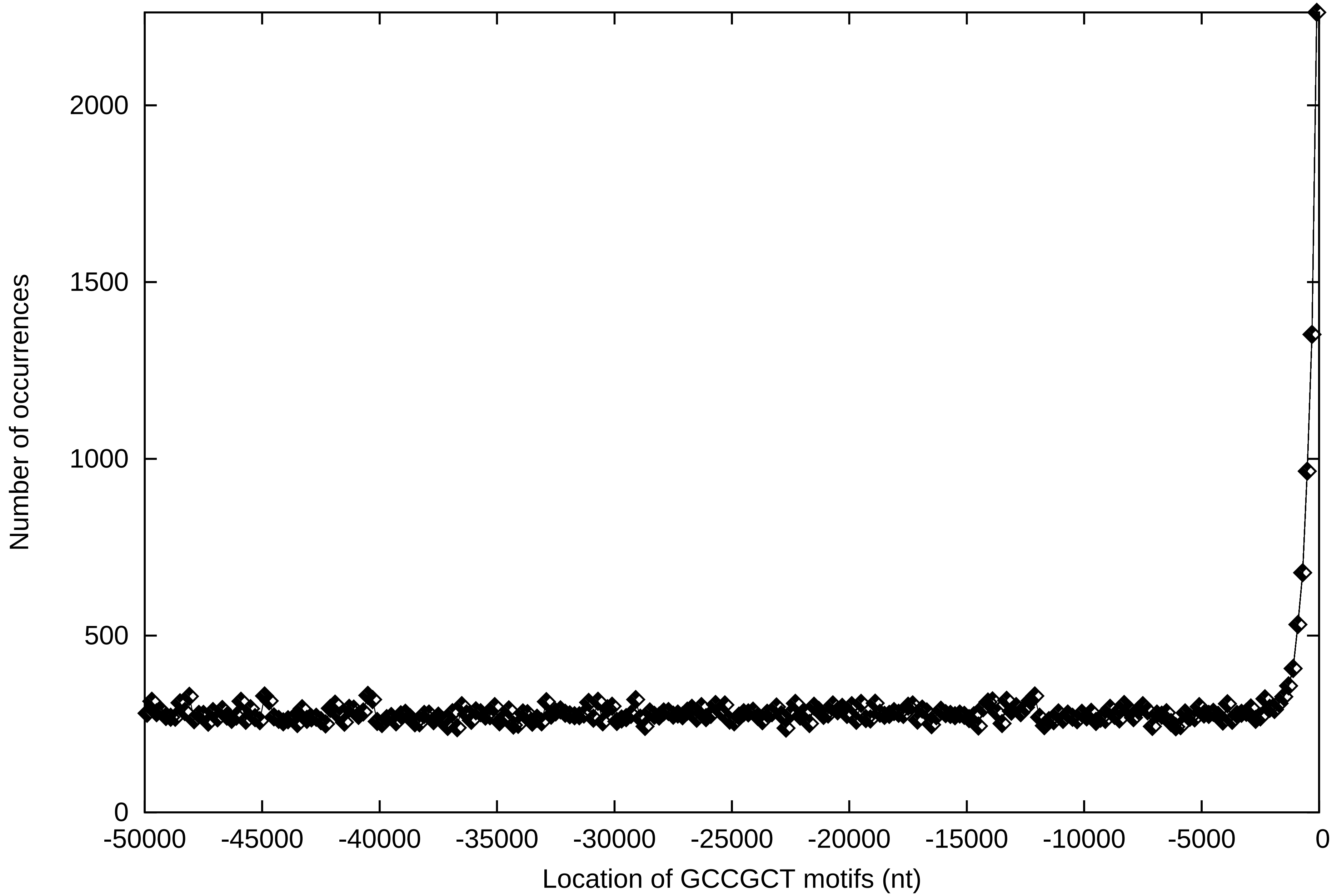

Number of occurrences
2000
1500
1000
500
0
-50000
-45000
-40000
-35000
-30000
-25000
-20000
-15000
-10000
-5000
0
Location of GCCGCT motifs (nt)

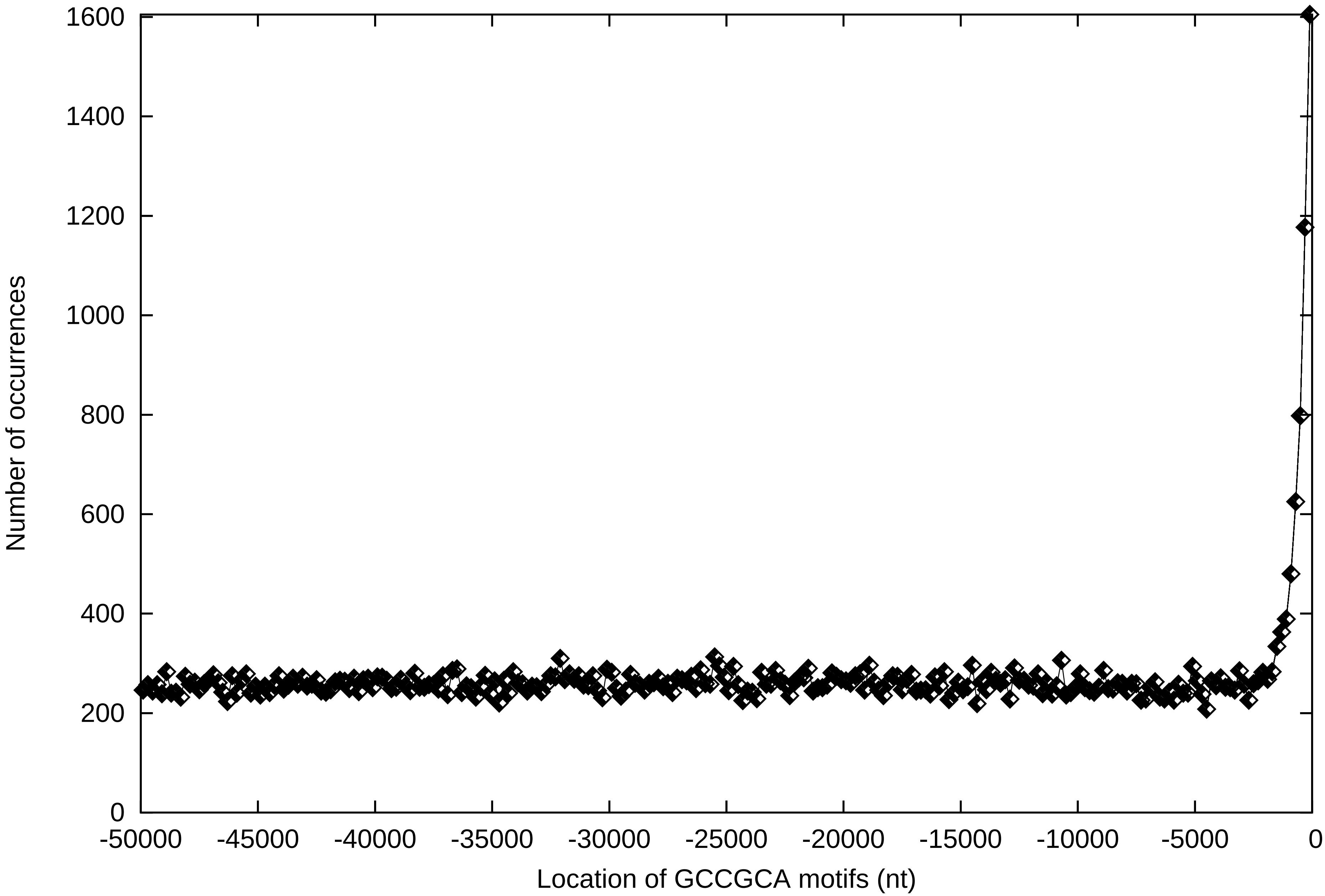

1600
1400
1200
1000
800
600
400
200
0
Number of occurrences
-50000
-45000
-40000
-35000
-30000
-25000
-20000
-15000
-10000
-5000
0
Location of GCCGCA motifs (nt)

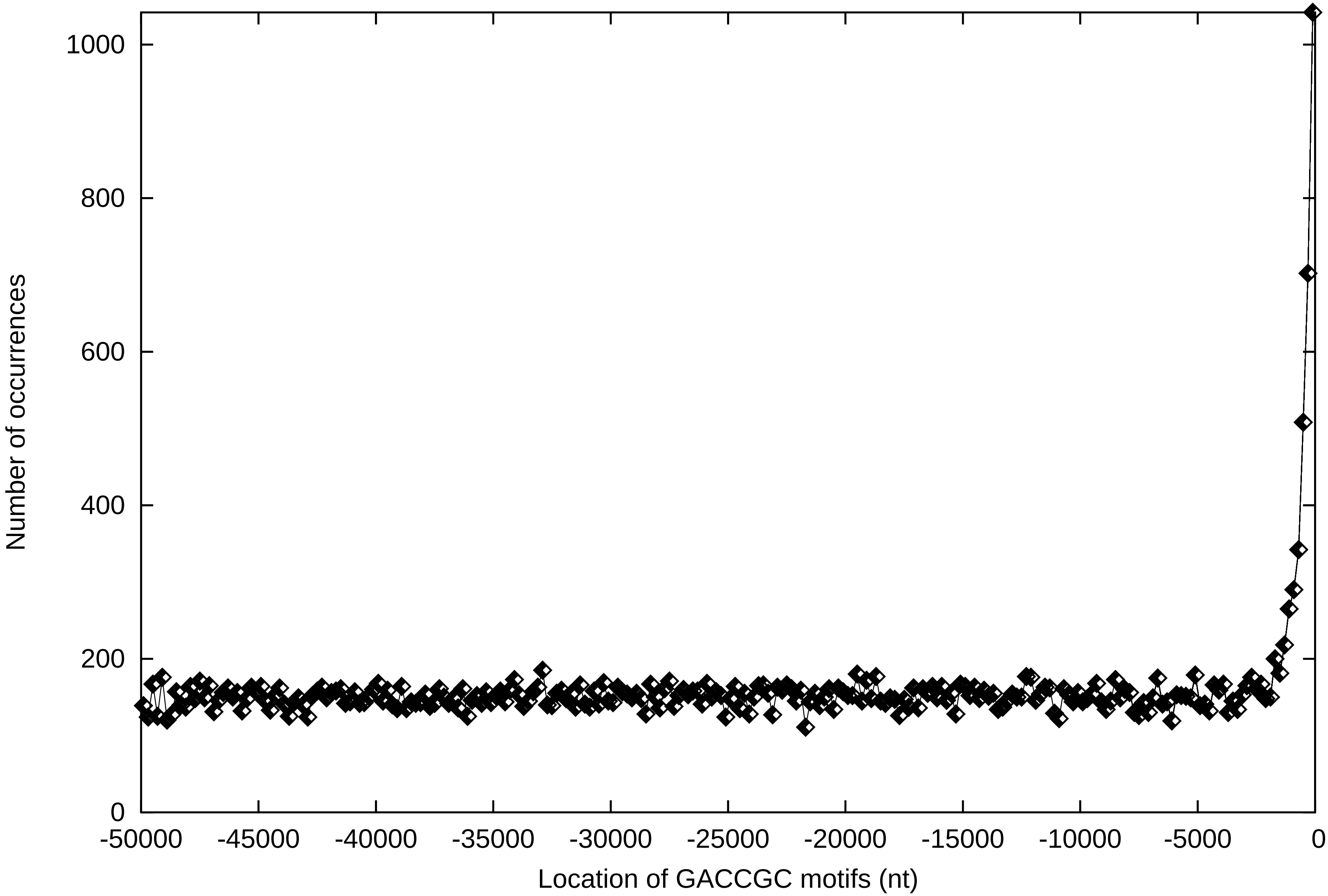

Number of occurrences
0
200
400
600
800
1000
-50000
-45000
-40000
-35000
-30000
-25000
-20000
-15000
-10000
-5000
0
Location of GACCGC motifs (nt)

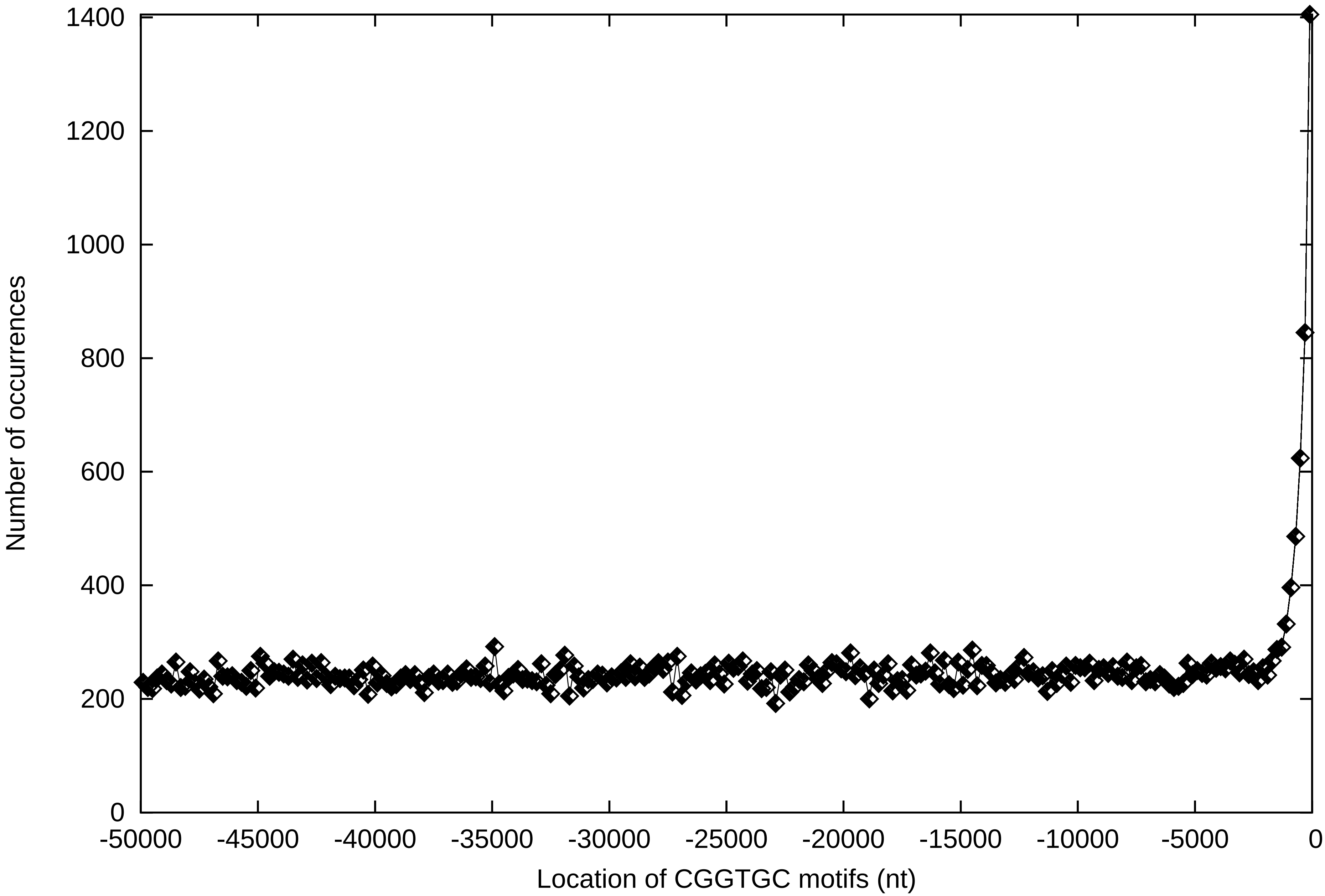
Number of occurrences
1400
1200
1000
800
600
400
200
0
-50000
-45000
-40000
-35000
-30000
-25000
-20000
-15000
-10000
-5000
0
Location of CGGTGC motifs (nt)

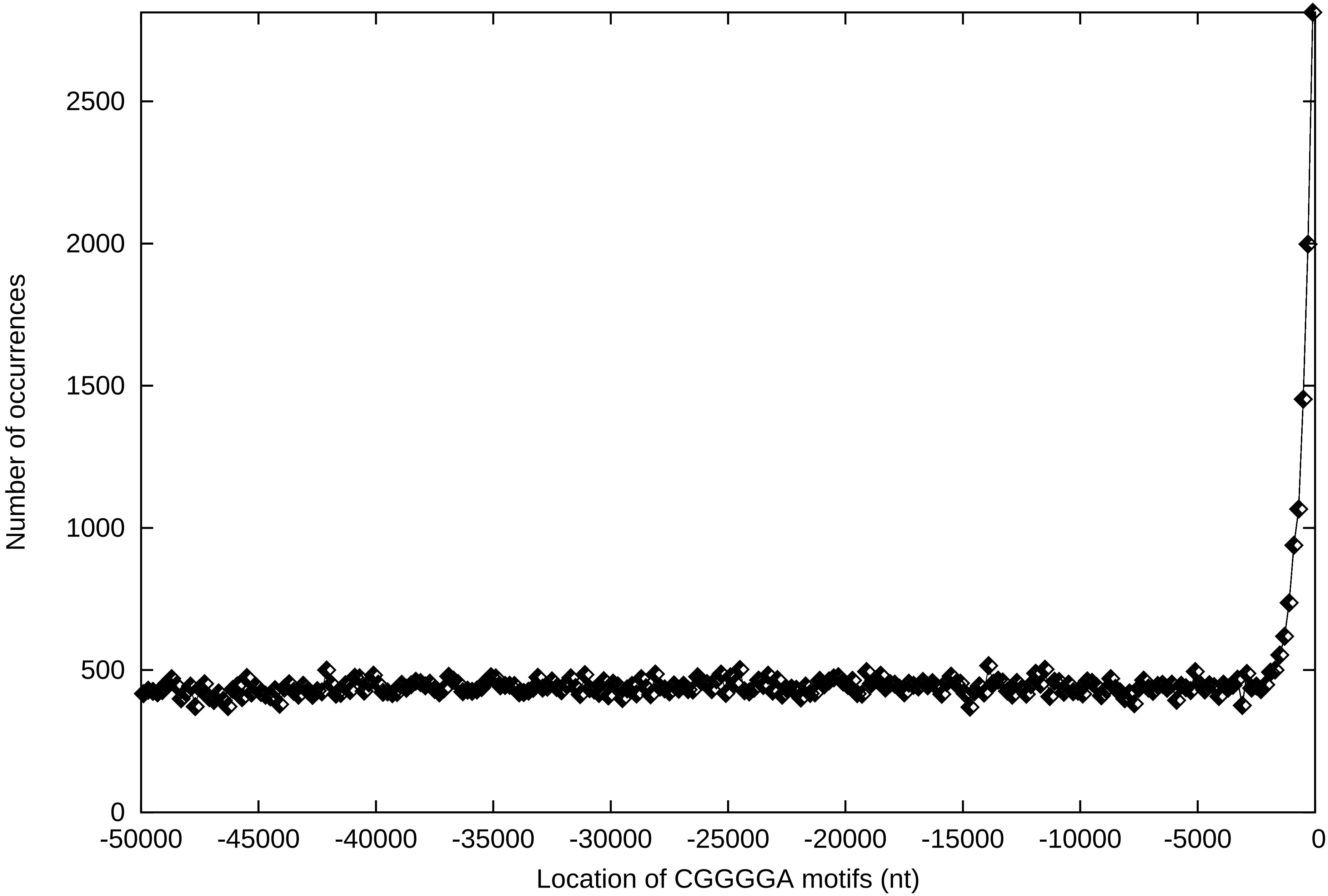
Number of occurrences
0
500
1000
1500
2000
2500
-50000
-45000
-40000
-35000
-30000
-25000
-20000
-15000
-10000
-5000
0
Location of CGGGGA motifs (nt)

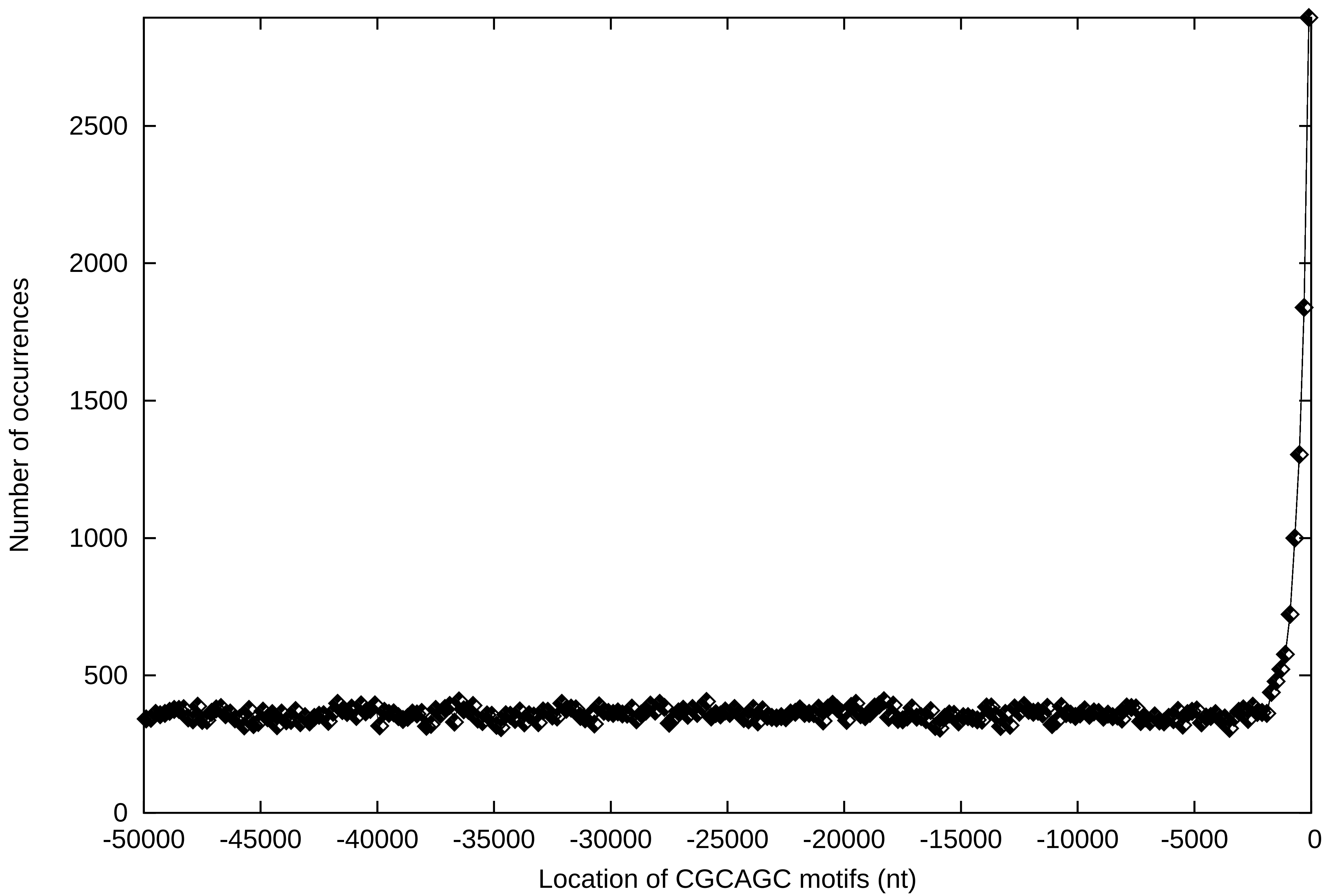
Number of occurrences
0
500
1000
1500
2000
2500
-50000
-45000
-40000
-35000
-30000
-25000
-20000
-15000
-10000
-5000
0
Location of CGCAGC motifs (nt)

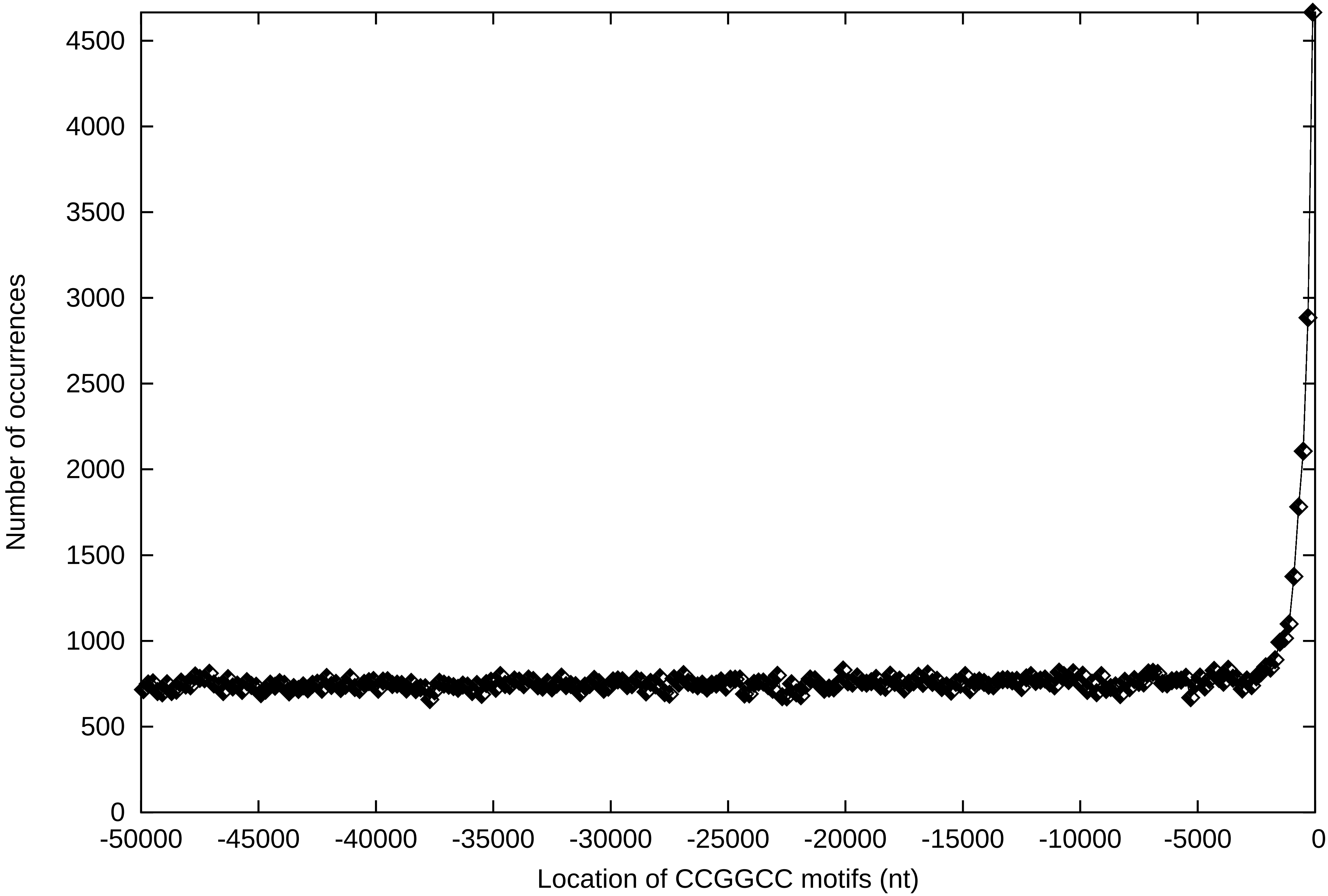
Number of occurrences
0
500
1000
1500
2000
2500
3000
3500
4000
4500
-50000
-45000
-40000
-35000
-30000
-25000
-20000
-15000
-10000
-5000
0
Location of CCGGCC motifs (nt)

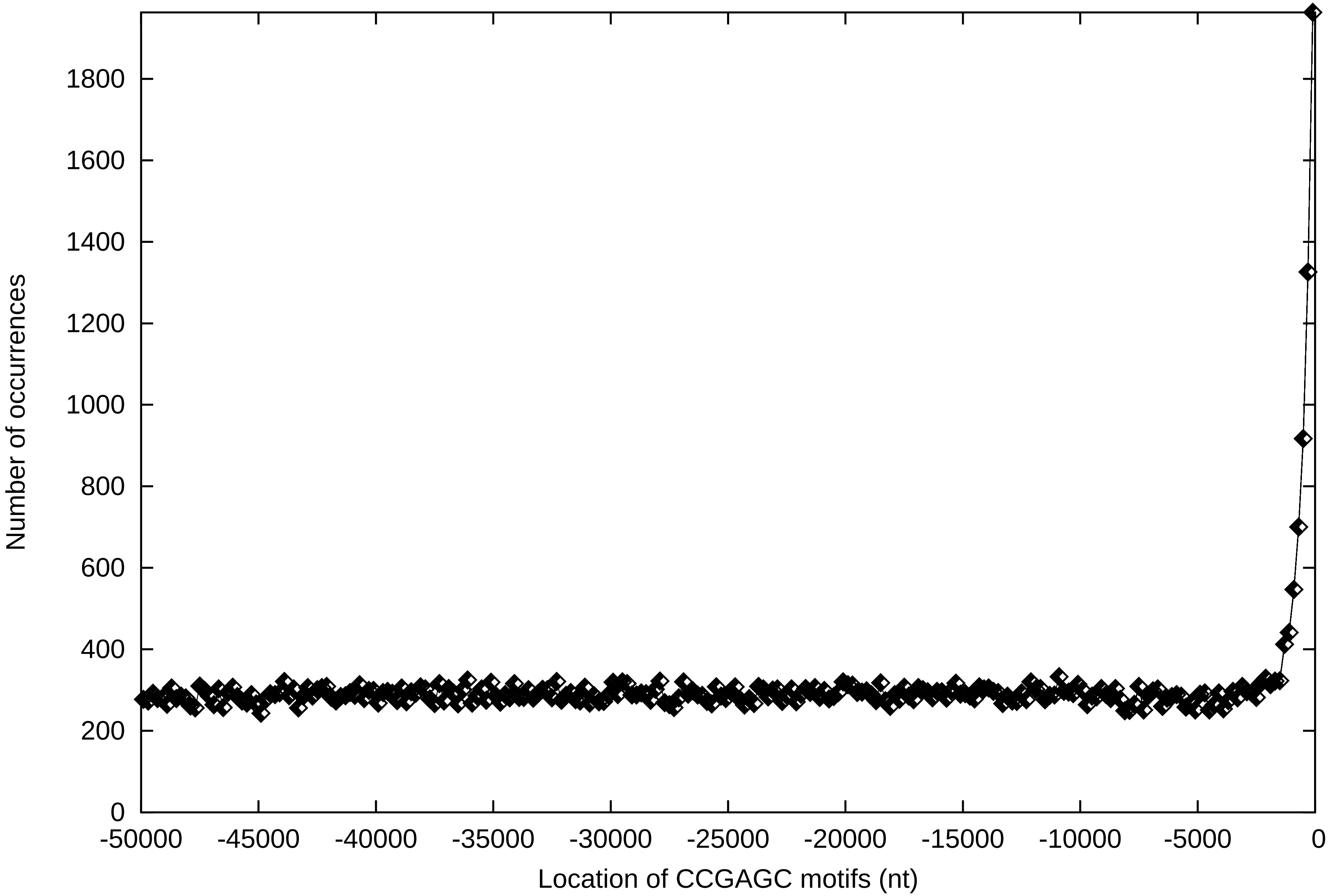

Number of occurrences
0
200
400
600
800
1000
1200
1400
1600
1800
-50000
-45000
-40000
-35000
-30000
-25000
-20000
-15000
-10000
-5000
0
Location of CCGAGC motifs (nt)

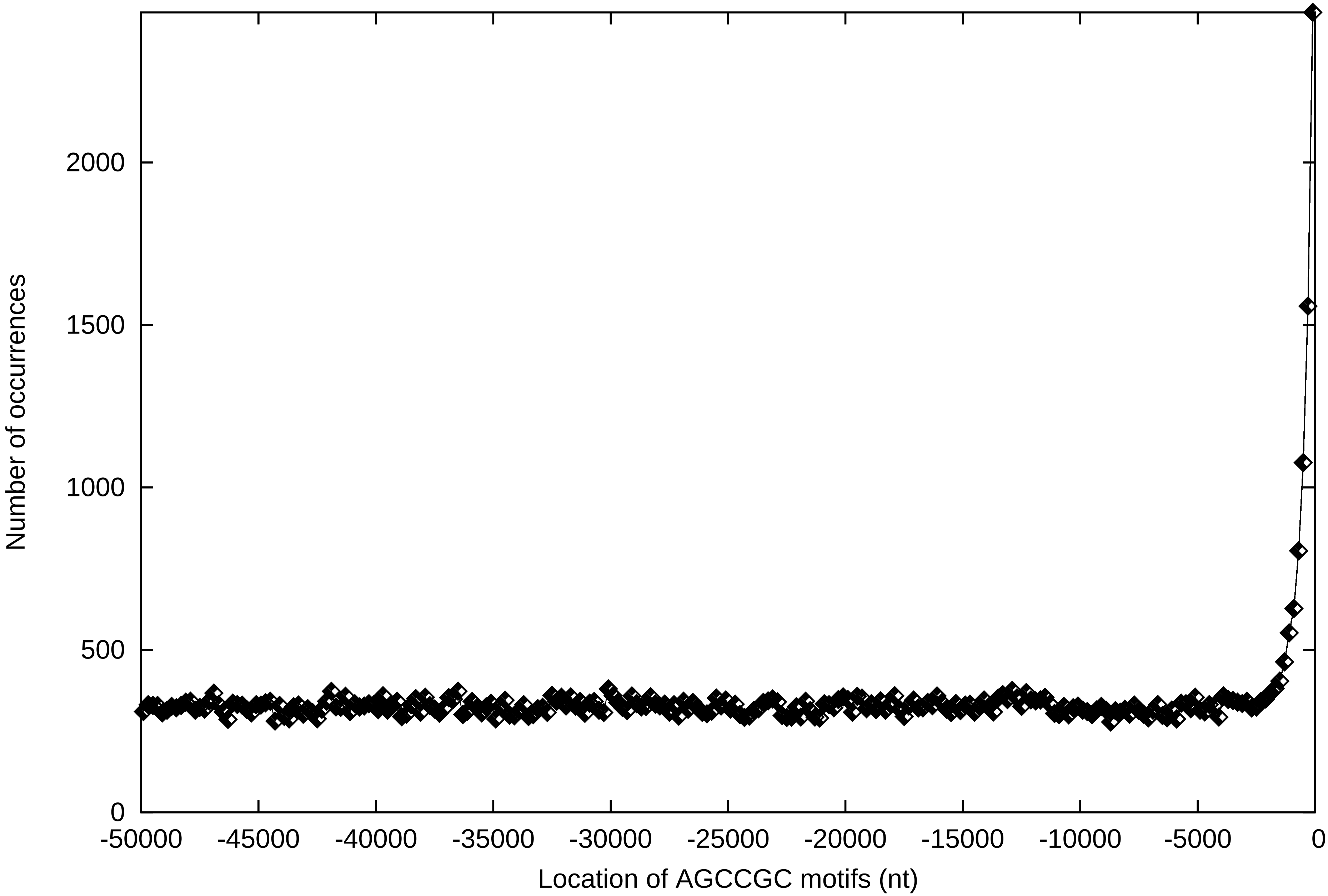

Number of occurrences
2000
1500
1000
500
0
-50000
-45000
-40000
-35000
-30000
-25000
-20000
-15000
-10000
-5000
0
Location of AGCCGC motifs (nt)
