# Supplementary material for: Regulatory Circuit of Human MicroRNA Biogenesis
Source: PLoS Comput Biol. 2007 Apr 20;3(4):e67. doi: 10.1371/journal.pcbi.0030067 (PMC1853126; doi:10.1371/journal.pcbi.0030067)
Supplement: Supplementary file 5 [file pcbi.0030067.sg005.pdf]

The 14 top-predicted 5-mers are preferentially located upstream of protein-coding genes (pages 2-15). Each graph is a histogram that represents the number of occurrences of each motif (every 200 nt stretch) in the first 50 kb upstream region of protein-coding genes. The motifs preferentially occur within the first 500 nt region (-500 to 0 nt region) that is directly upstream of the protein-coding genes.

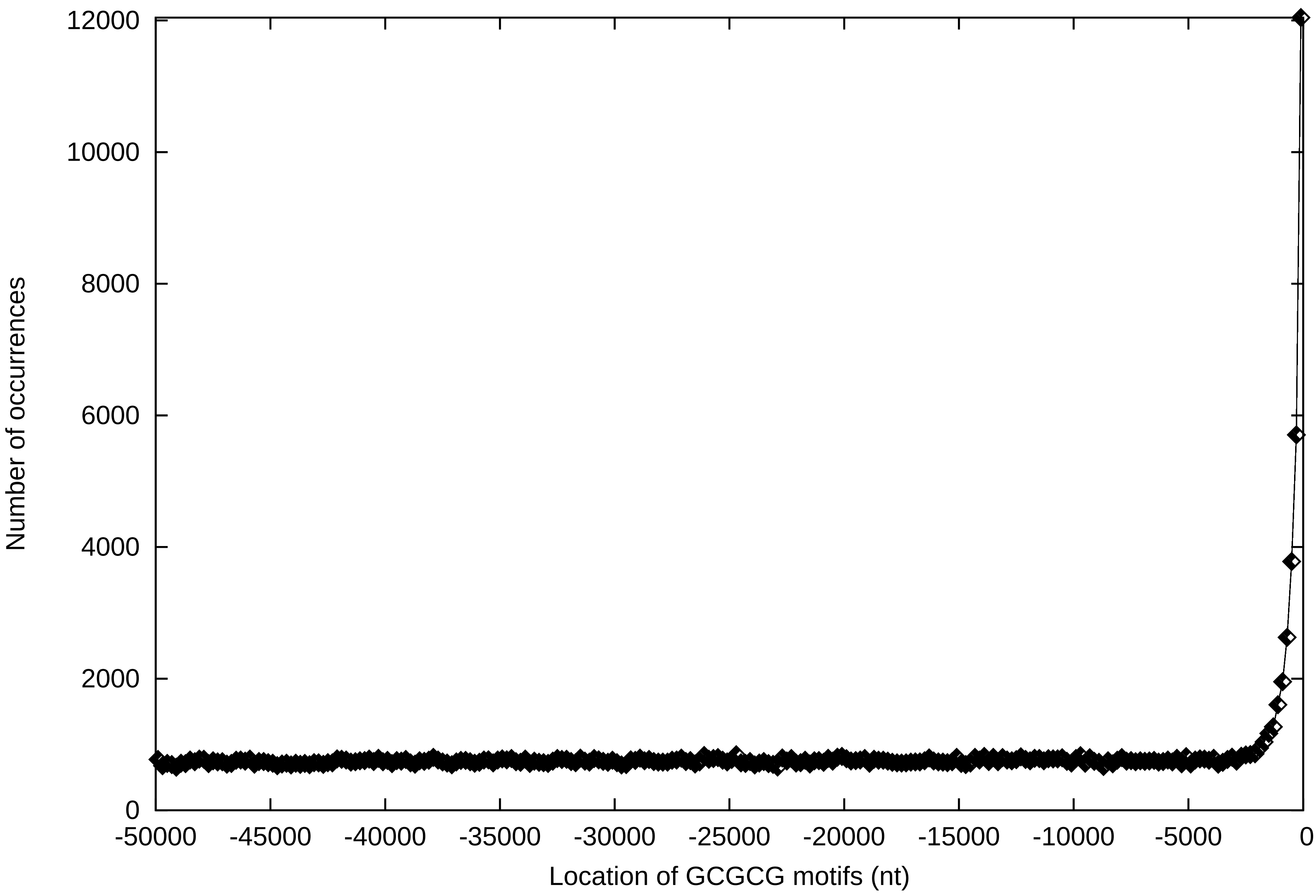

Number of occurrences
12000
10000
8000
6000
4000
2000
0
-50000
-45000
-40000
-35000
-30000
-25000
-20000
-15000
-10000
-5000
0
Location of GCGCG motifs (nt)

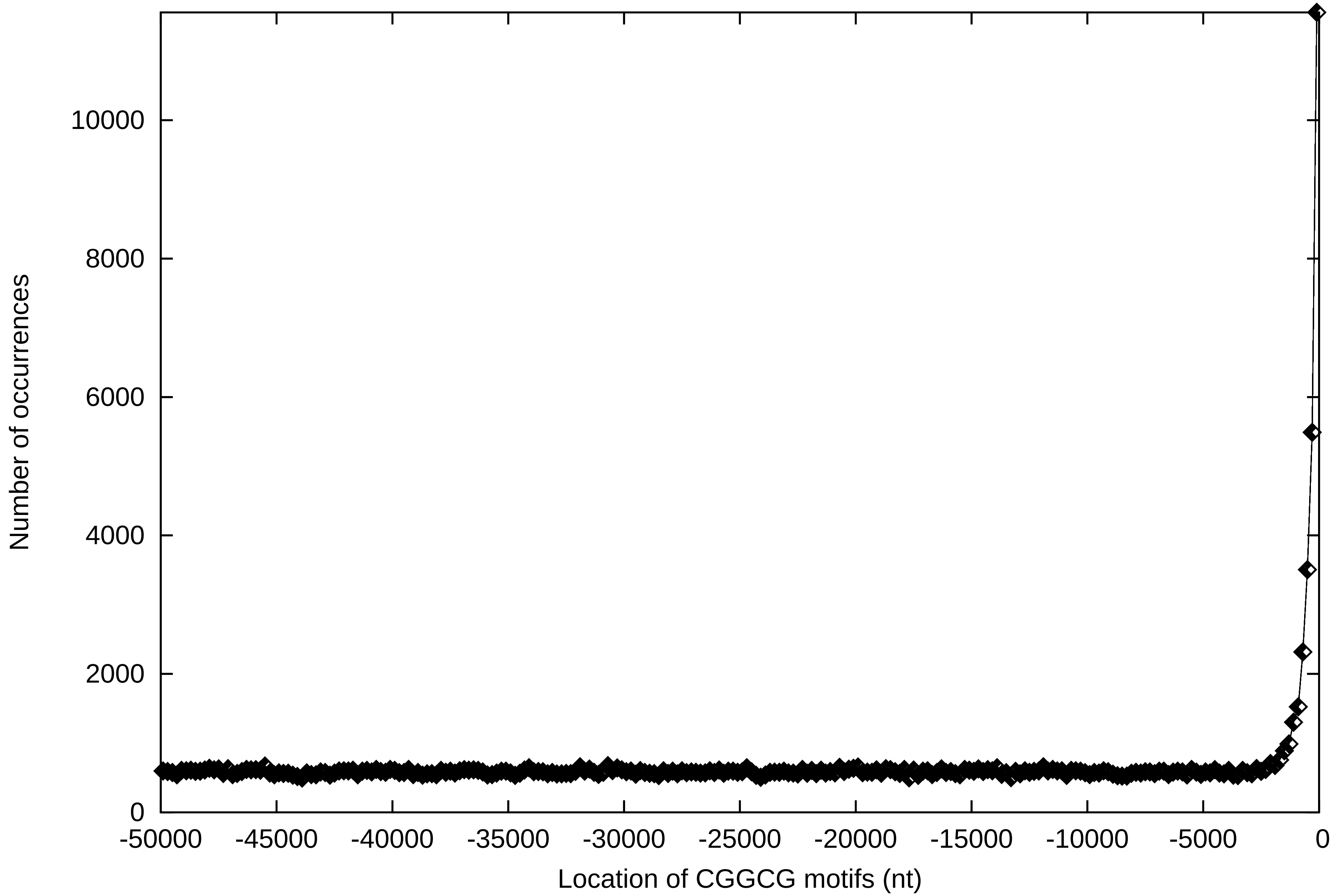

Number of occurrences
0
2000
4000
6000
8000
10000
-50000
-45000
-40000
-35000
-30000
-25000
-20000
-15000
-10000
-5000
0
Location of CGGCG motifs (nt)

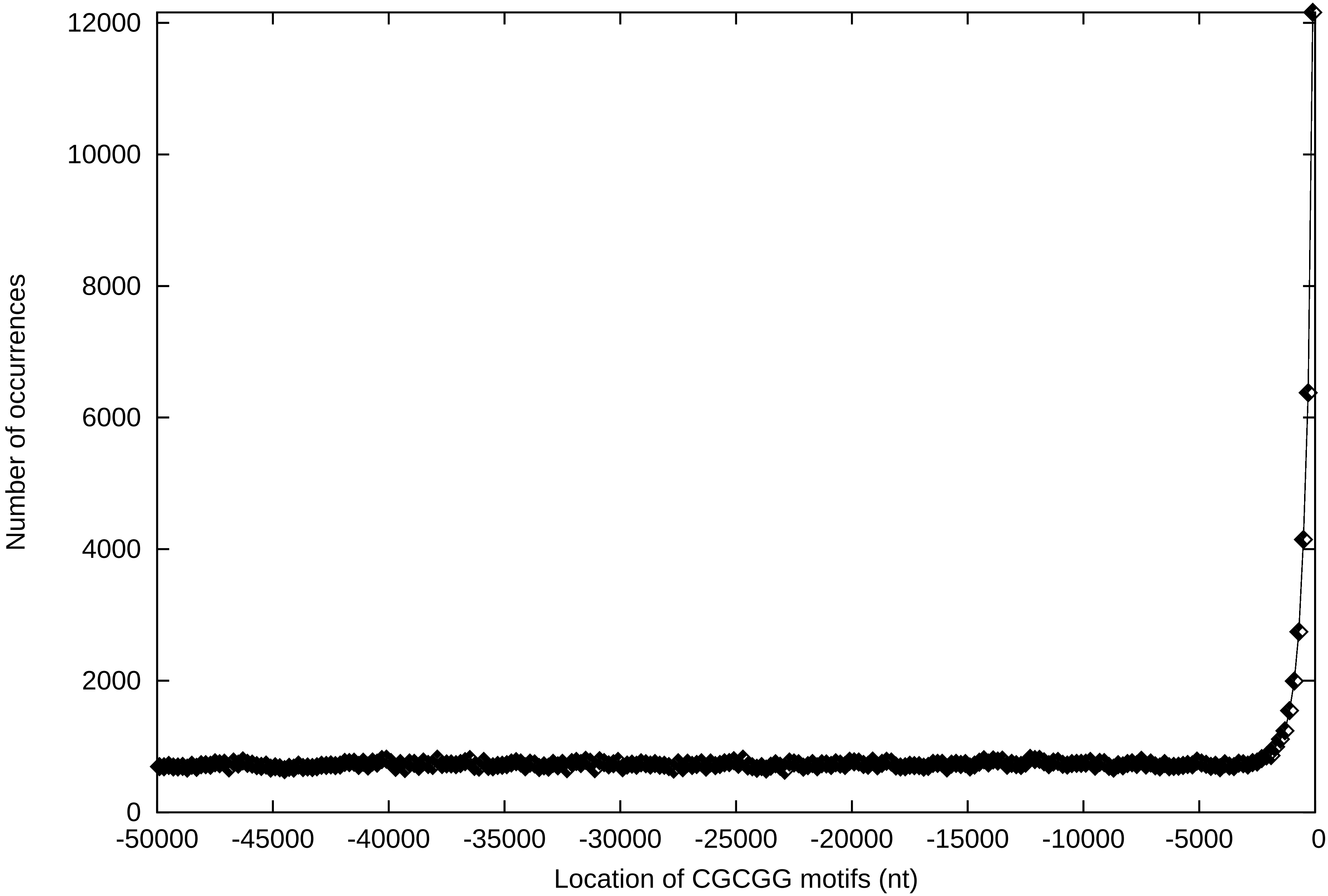

Number of occurrences
Location of CGCGG motifs (nt)
0
2000
4000
6000
8000
10000
12000
-50000
-45000
-40000
-35000
-30000
-25000
-20000
-15000
-10000
-5000
0

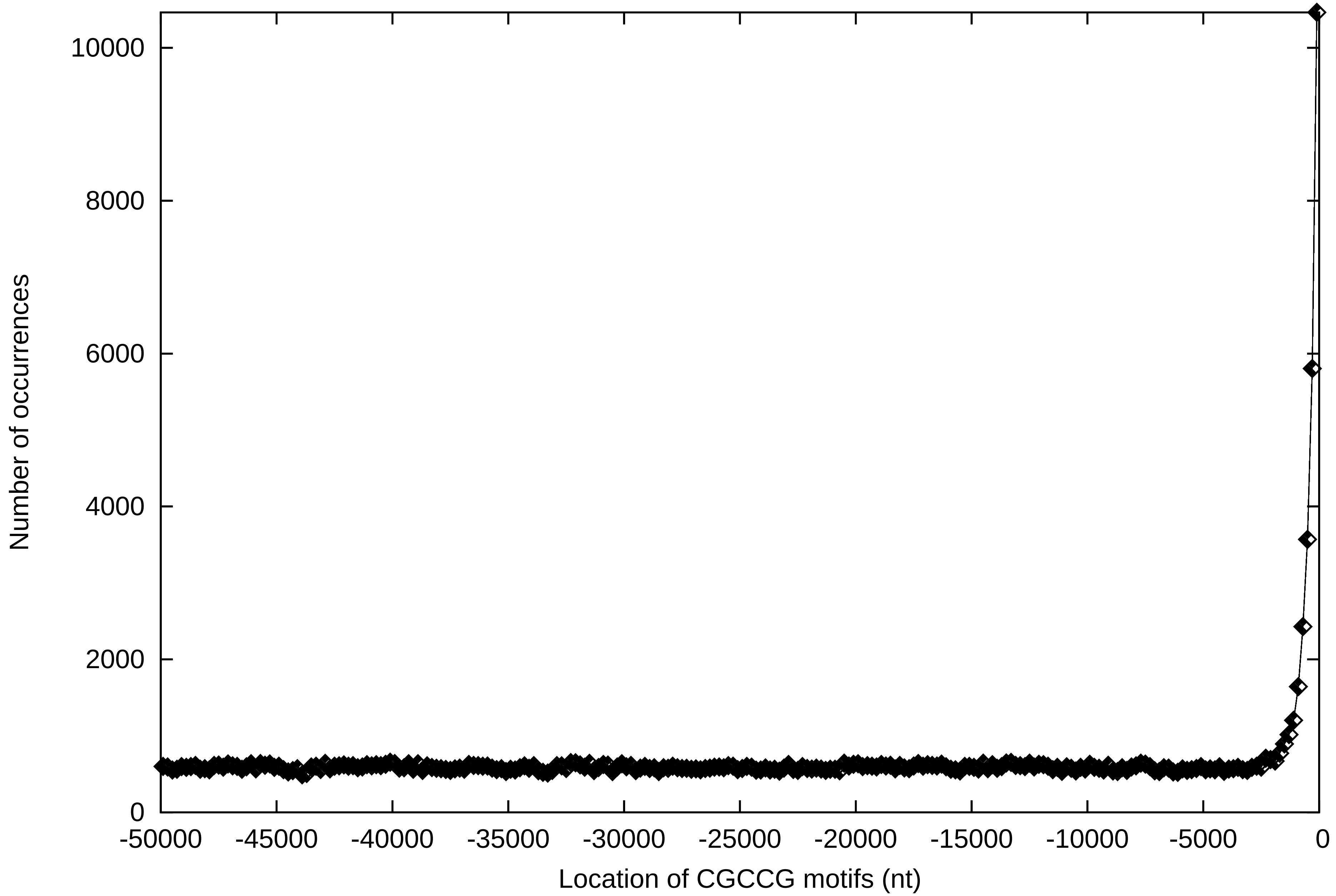

Number of occurrences
10000
8000
6000
4000
2000
0
-50000
-45000
-40000
-35000
-30000
-25000
-20000
-15000
-10000
-5000
0
Location of CGCCG motifs (nt)

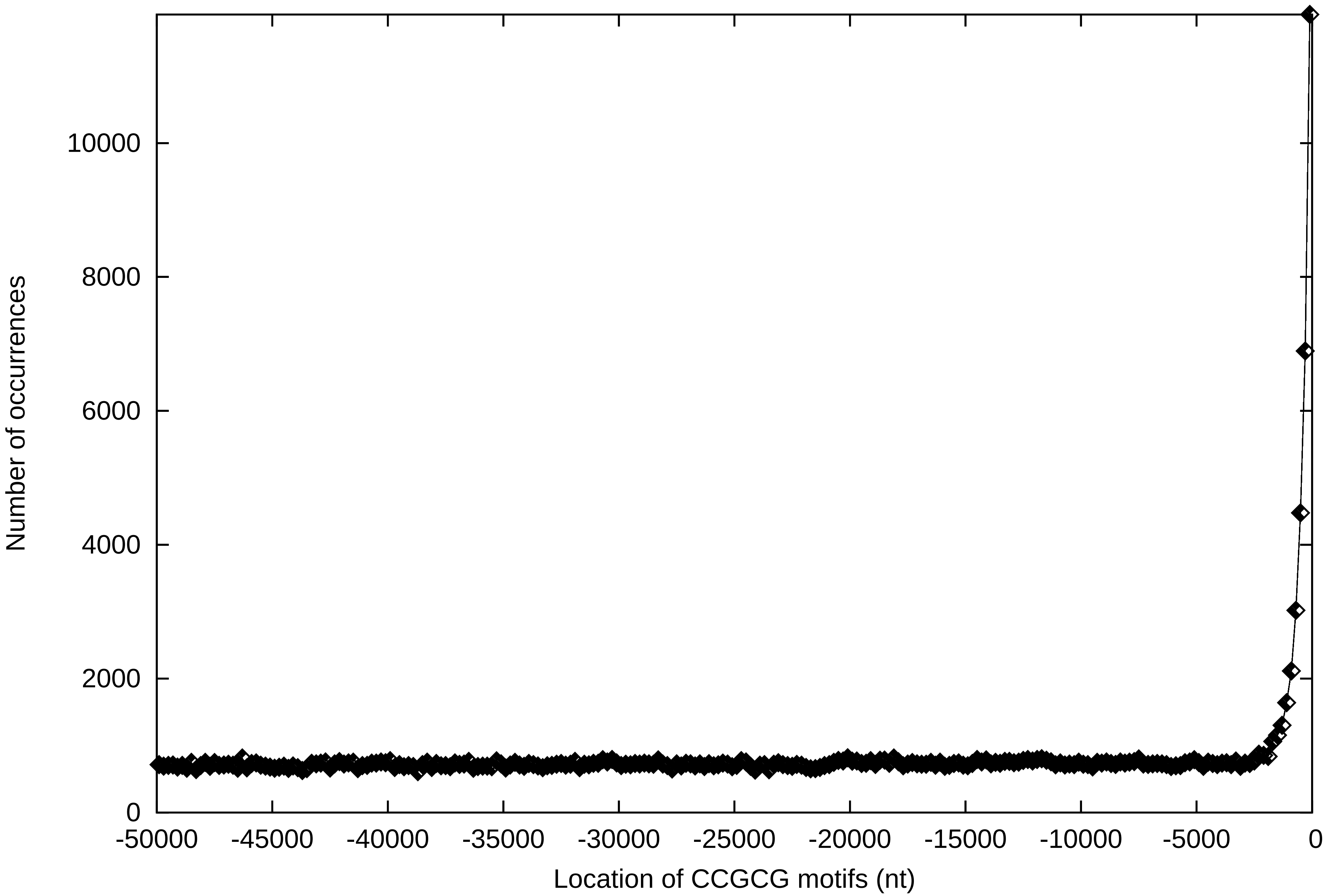

10000
8000
6000
4000
2000
0
Number of occurrences
-50000
-45000
-40000
-35000
-30000
-25000
-20000
-15000
-10000
-5000
0
Location of CCGCG motifs (nt)

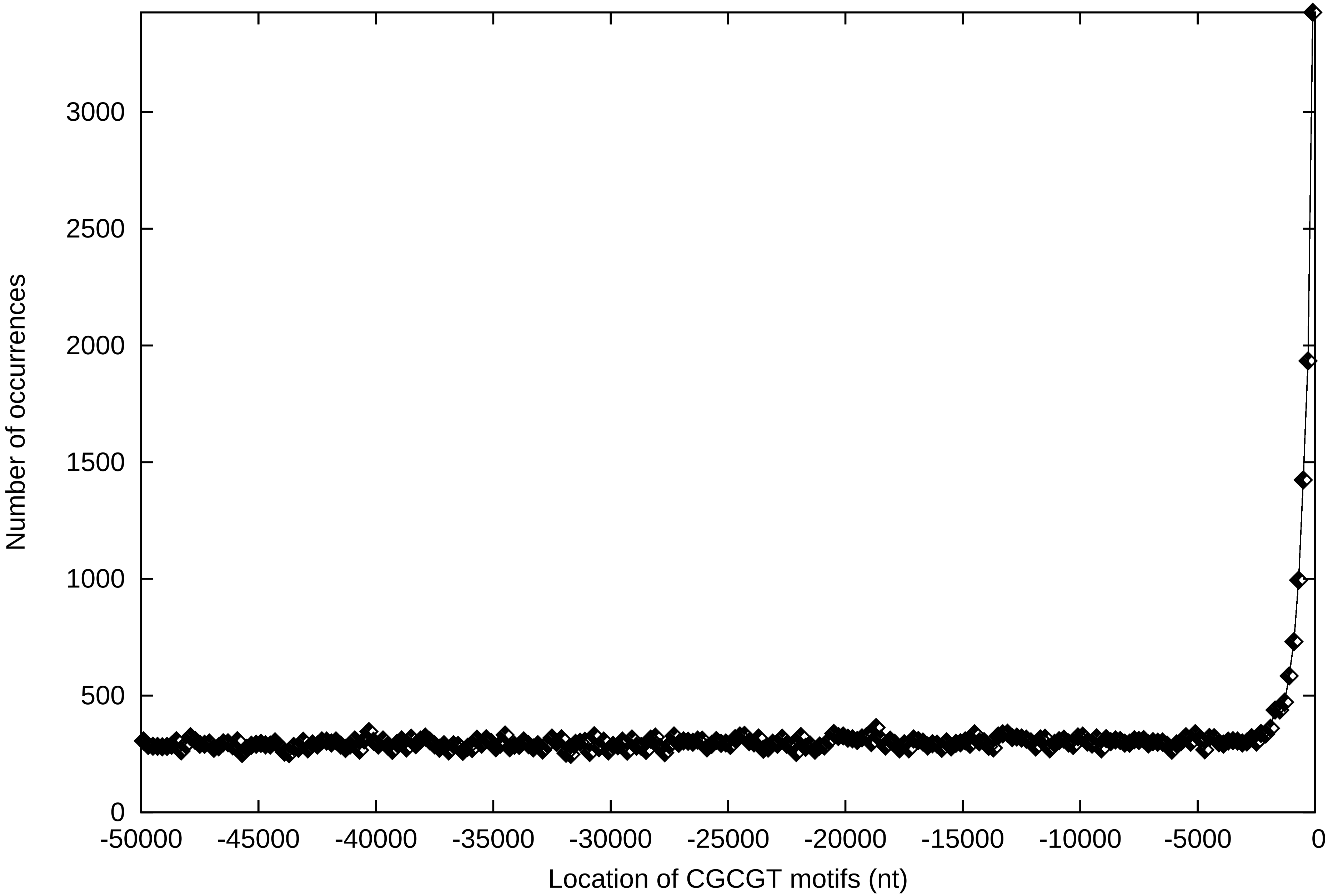

Number of occurrences
0
500
1000
1500
2000
2500
3000
-50000
-45000
-40000
-35000
-30000
-25000
-20000
-15000
-10000
-5000
0
Location of CGCGT motifs (nt)

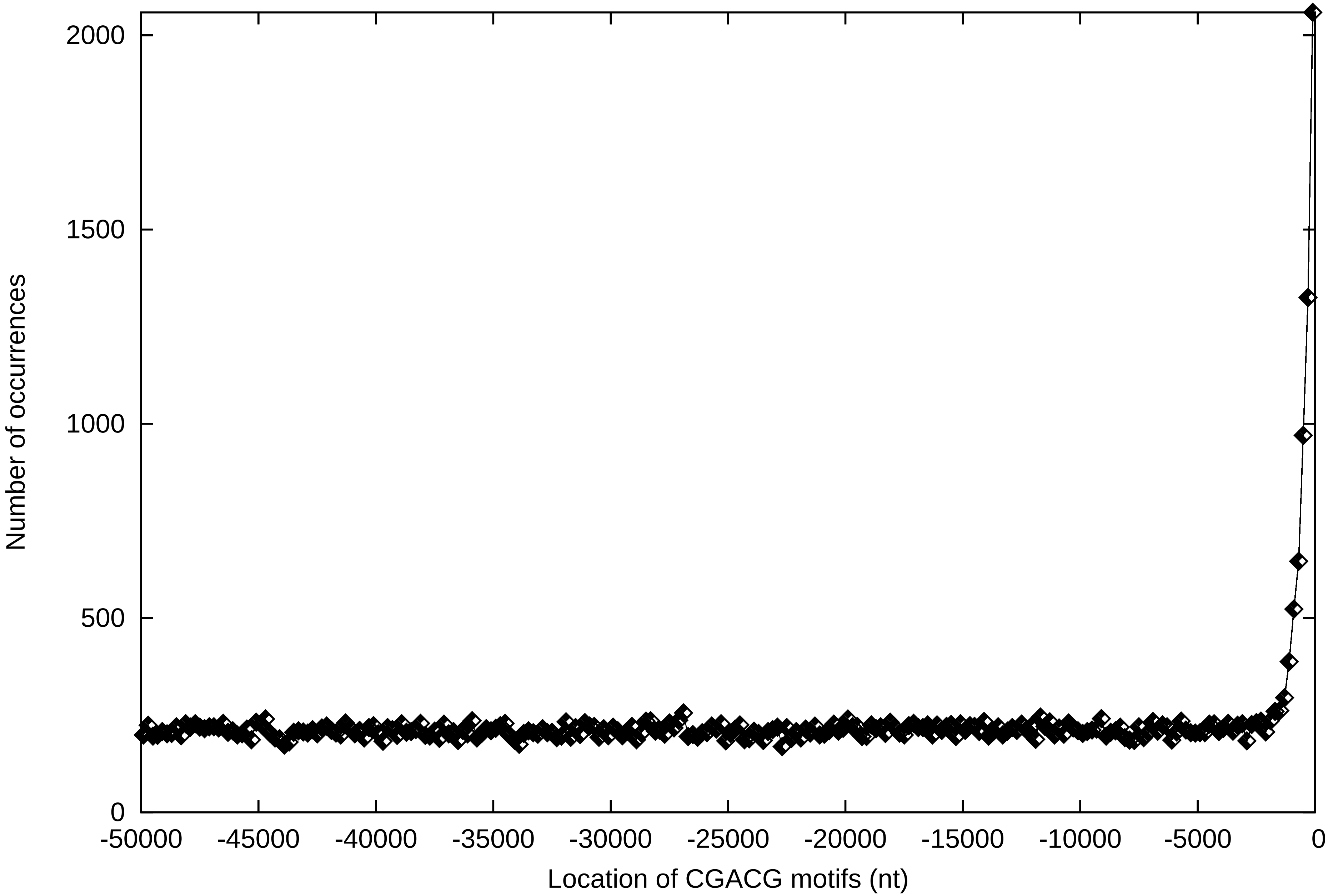

Number of occurrences
Location of CGACG motifs (nt)
2000
1500
1000
500
0
-50000
-45000
-40000
-35000
-30000
-25000
-20000
-15000
-10000
-5000
0

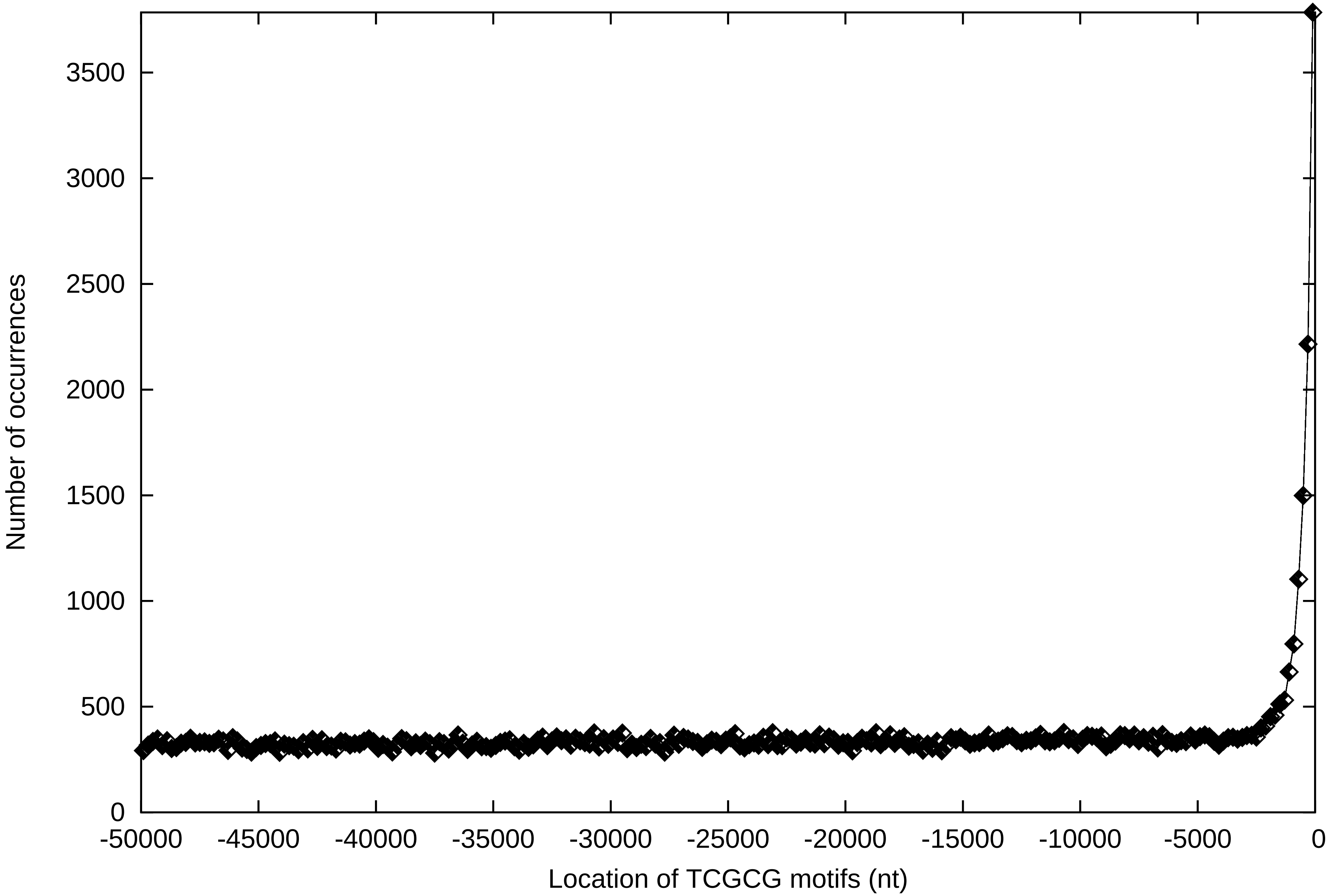

Number of occurrences
0
500
1000
1500
2000
2500
3000
3500
-50000
-45000
-40000
-35000
-30000
-25000
-20000
-15000
-10000
-5000
0
Location of TCGCG motifs (nt)

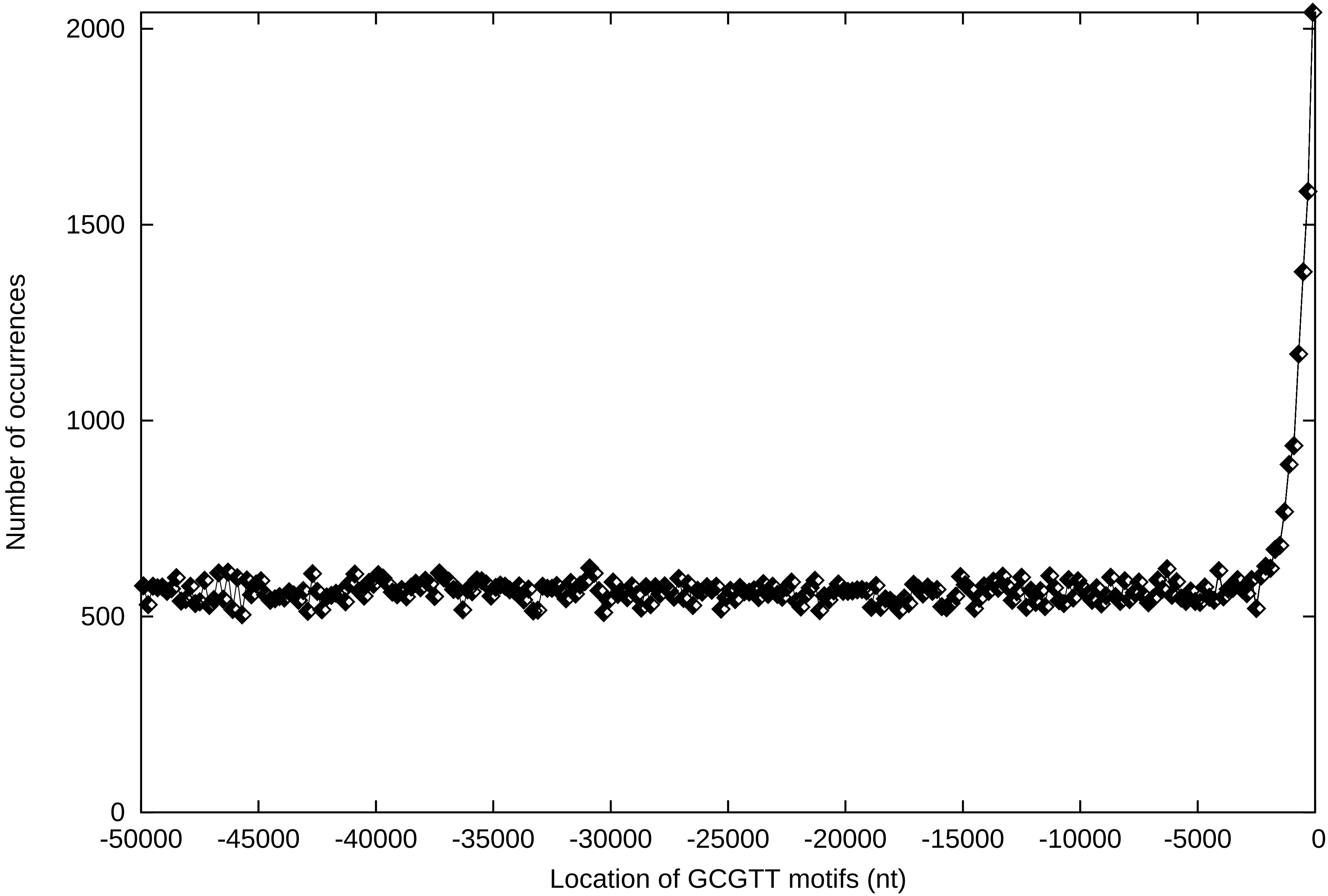

Number of occurrences
2000
1500
1000
500
0
-50000
-45000
-40000
-35000
-30000
-25000
-20000
-15000
-10000
-5000
0
Location of GCGTT motifs (nt)

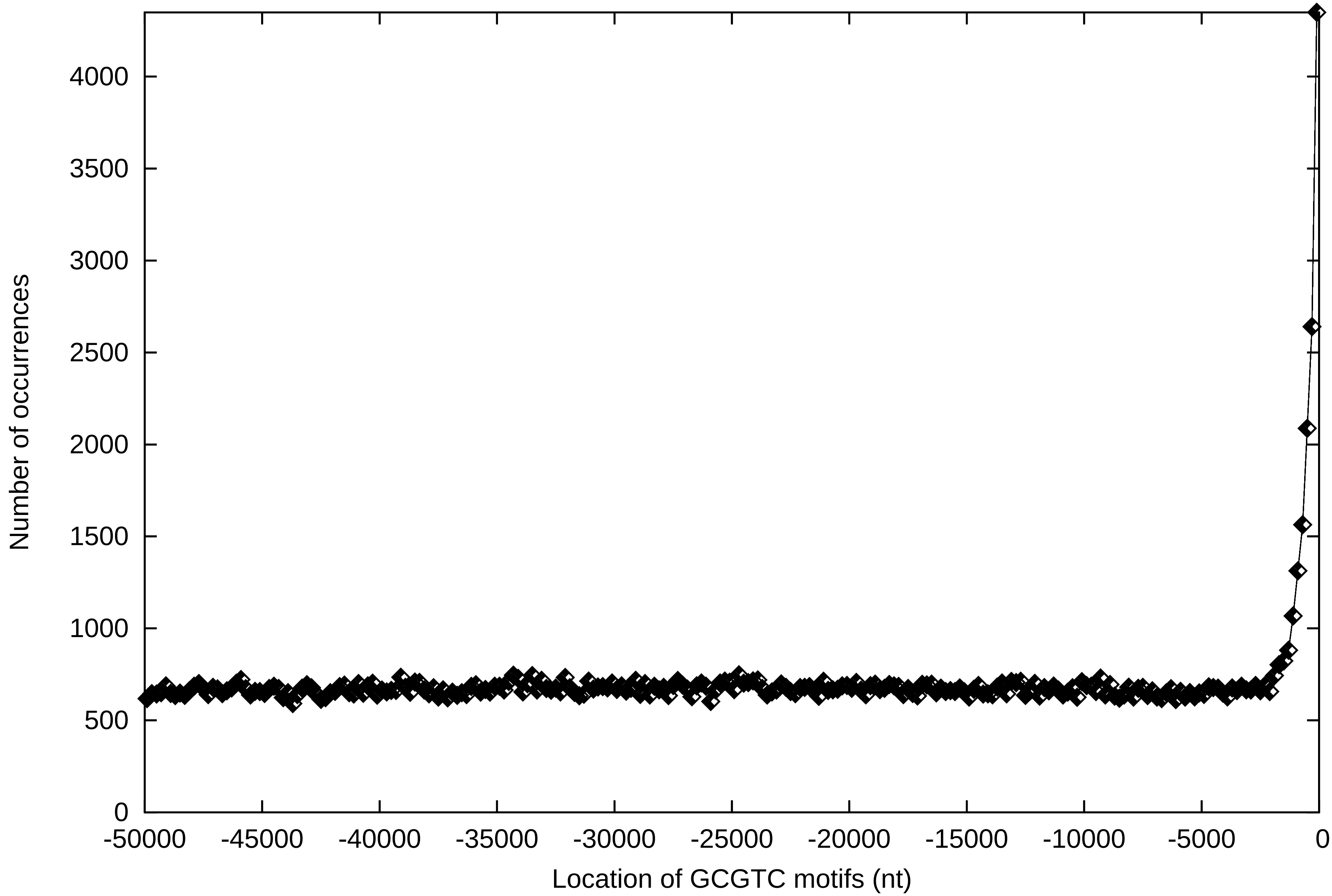
Number of occurrences
0
500
1000
1500
2000
2500
3000
3500
4000
-50000
-45000
-40000
-35000
-30000
-25000
-20000
-15000
-10000
-5000
0
Location of GCGTC motifs (nt)

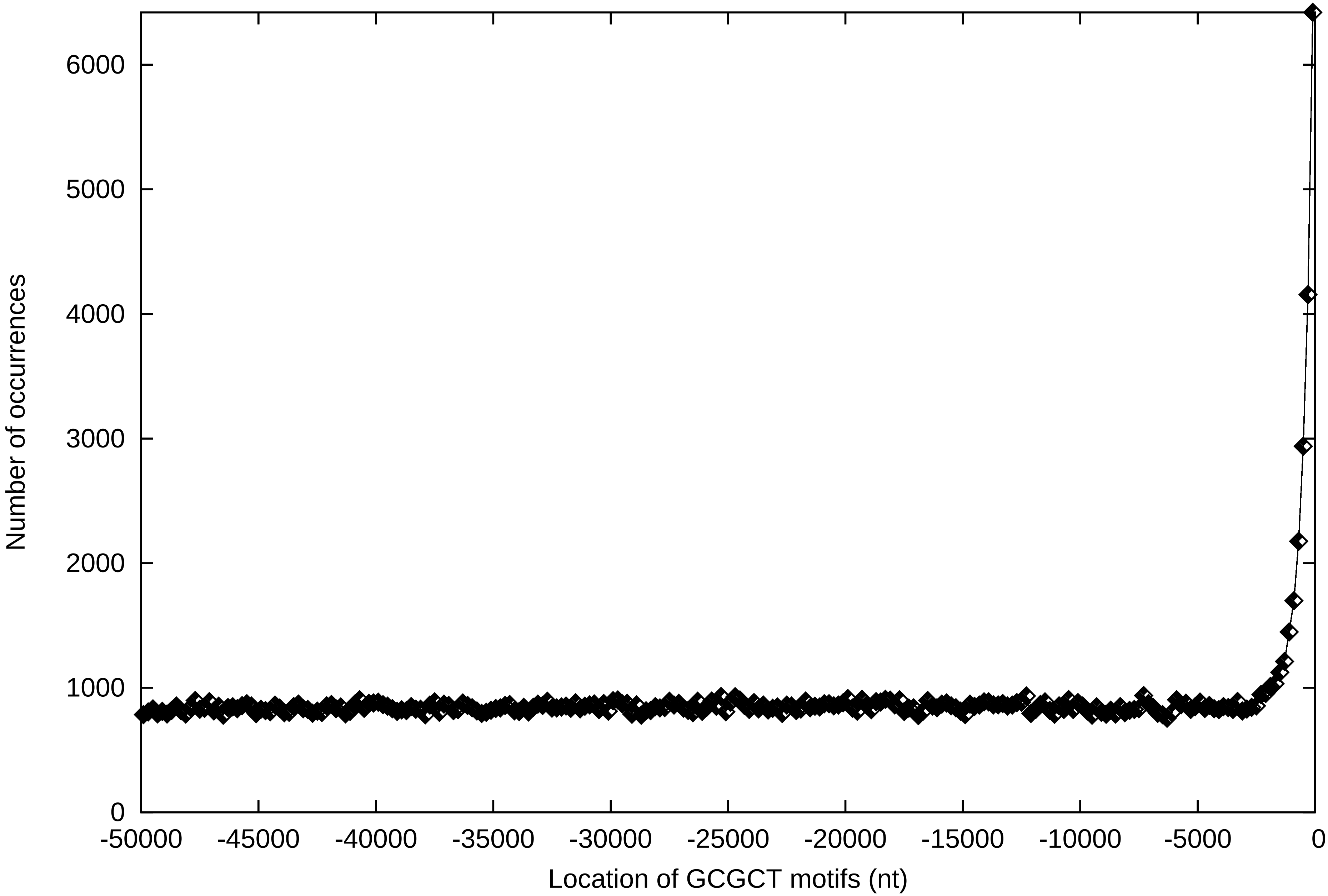

Number of occurrences
6000
5000
4000
3000
2000
1000
0
-50000
-45000
-40000
-35000
-30000
-25000
-20000
-15000
-10000
-5000
0
Location of GCGCT motifs (nt)

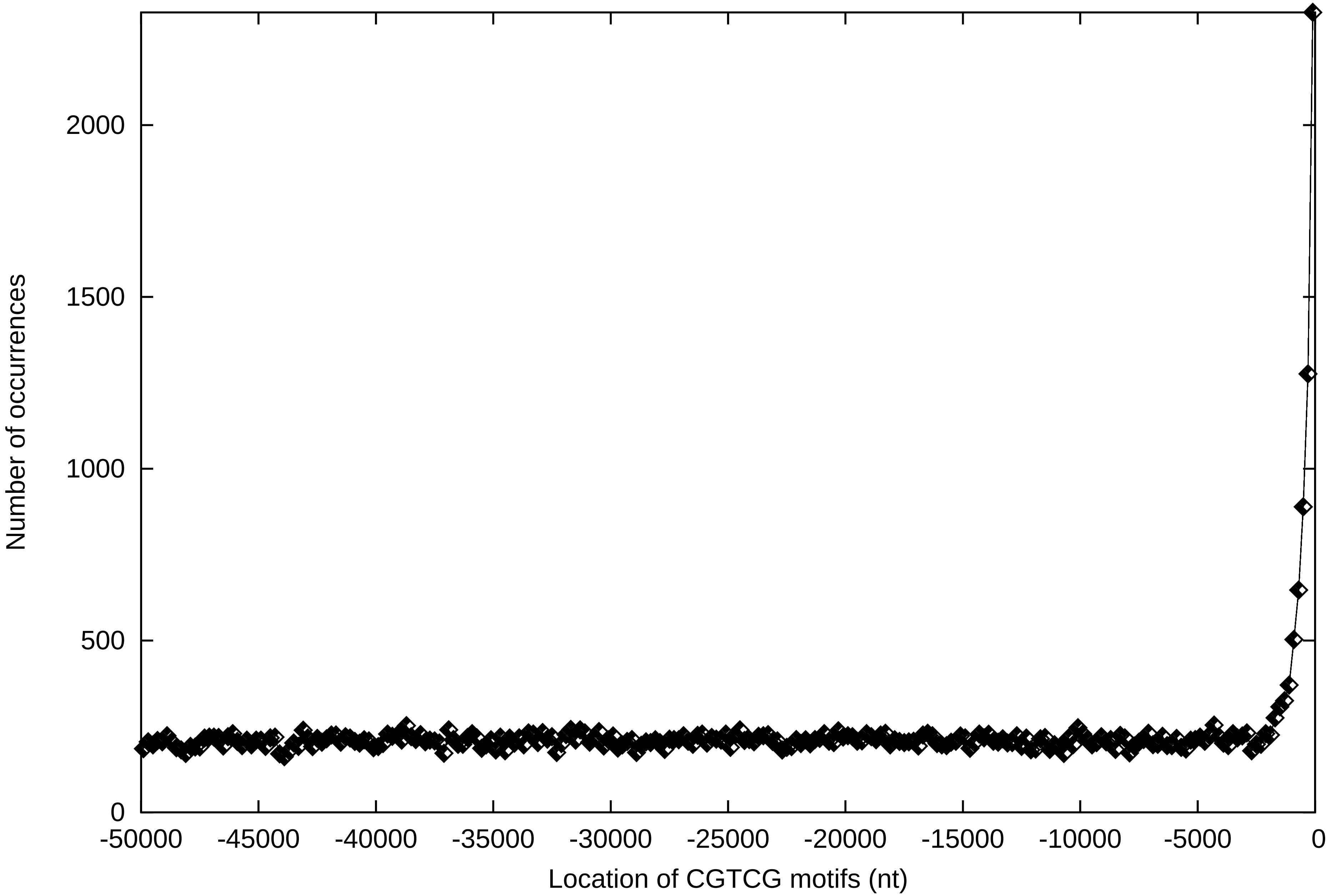

Number of occurrences
2000
1500
1000
500
0
-50000
-45000
-40000
-35000
-30000
-25000
-20000
-15000
-10000
-5000
0
Location of CGTCG motifs (nt)

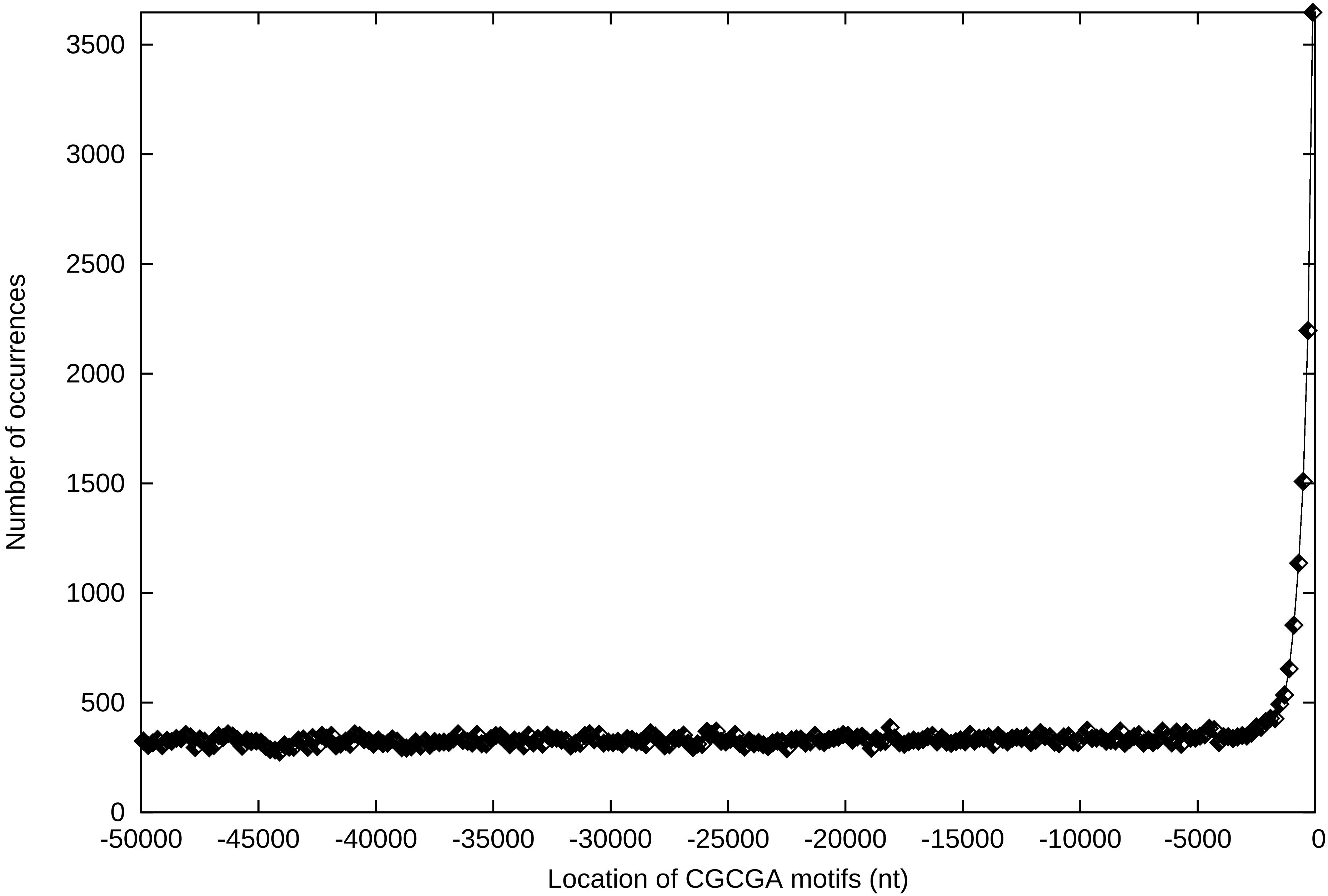

Number of occurrences
3500
3000
2500
2000
1500
1000
500
0
-50000
-45000
-40000
-35000
-30000
-25000
-20000
-15000
-10000
-5000
0
Location of CGCGA motifs (nt)

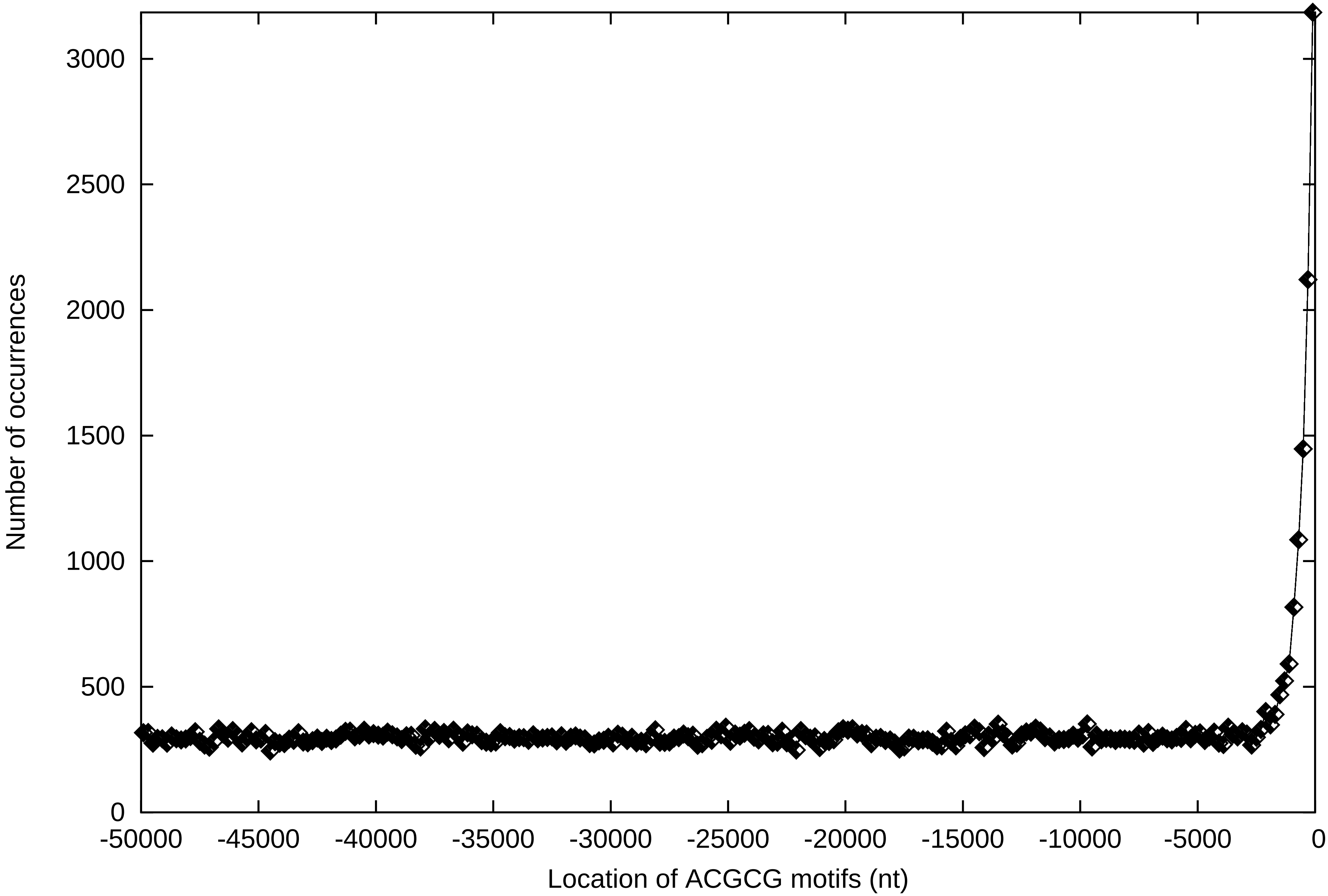

3000
2500
2000
1500
1000
500
0
Number of occurrences
-50000
-45000
-40000
-35000
-30000
-25000
-20000
-15000
-10000
-5000
0
Location of ACGCG motifs (nt)
