# Supplementary material for: Regulatory Circuit of Human MicroRNA Biogenesis
Source: PLoS Comput Biol. 2007 Apr 20;3(4):e67. doi: 10.1371/journal.pcbi.0030067 (PMC1853126; doi:10.1371/journal.pcbi.0030067)
Supplement: Supplementary file 6 [file pcbi.0030067.sg006.pdf]

Distance distribution of randomly selected 9 mers (pages 2-101). Each graph is a histogram that represents the number of occurrences of each motif (every 200 nt stretch) in the first 50 kb upstream region of protein-coding genes. In stark contrast to the top-predicted motifs, the control motifs do not have a strong preference to occur in the neighbor hood of the transcription start sites of protein-coding genes.

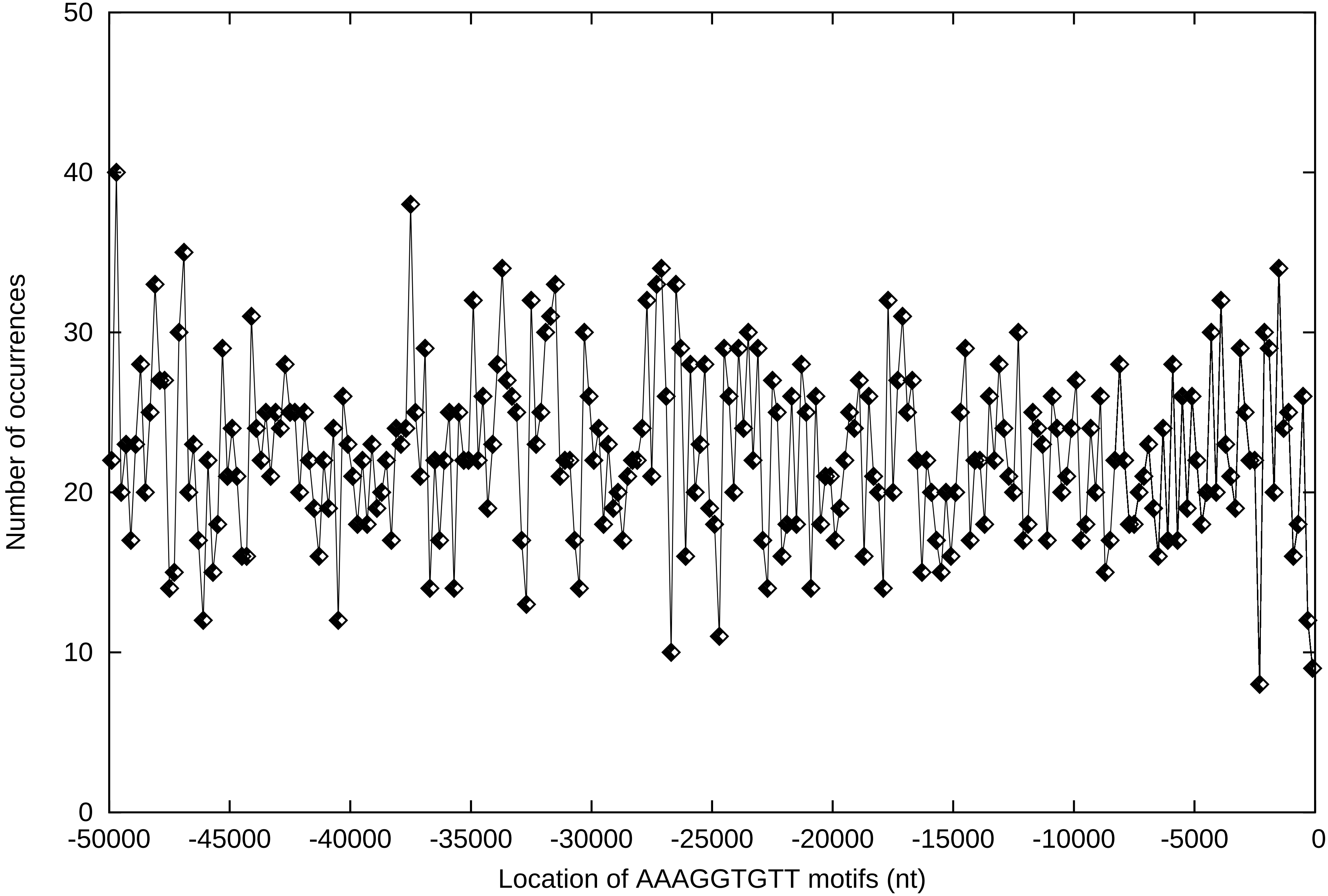

Number of occurrences
Location of AAAGGTGTT motifs (nt)
0
10
20
30
40
50
-50000
-45000
-40000
-35000
-30000
-25000
-20000
-15000
-10000
-5000
0

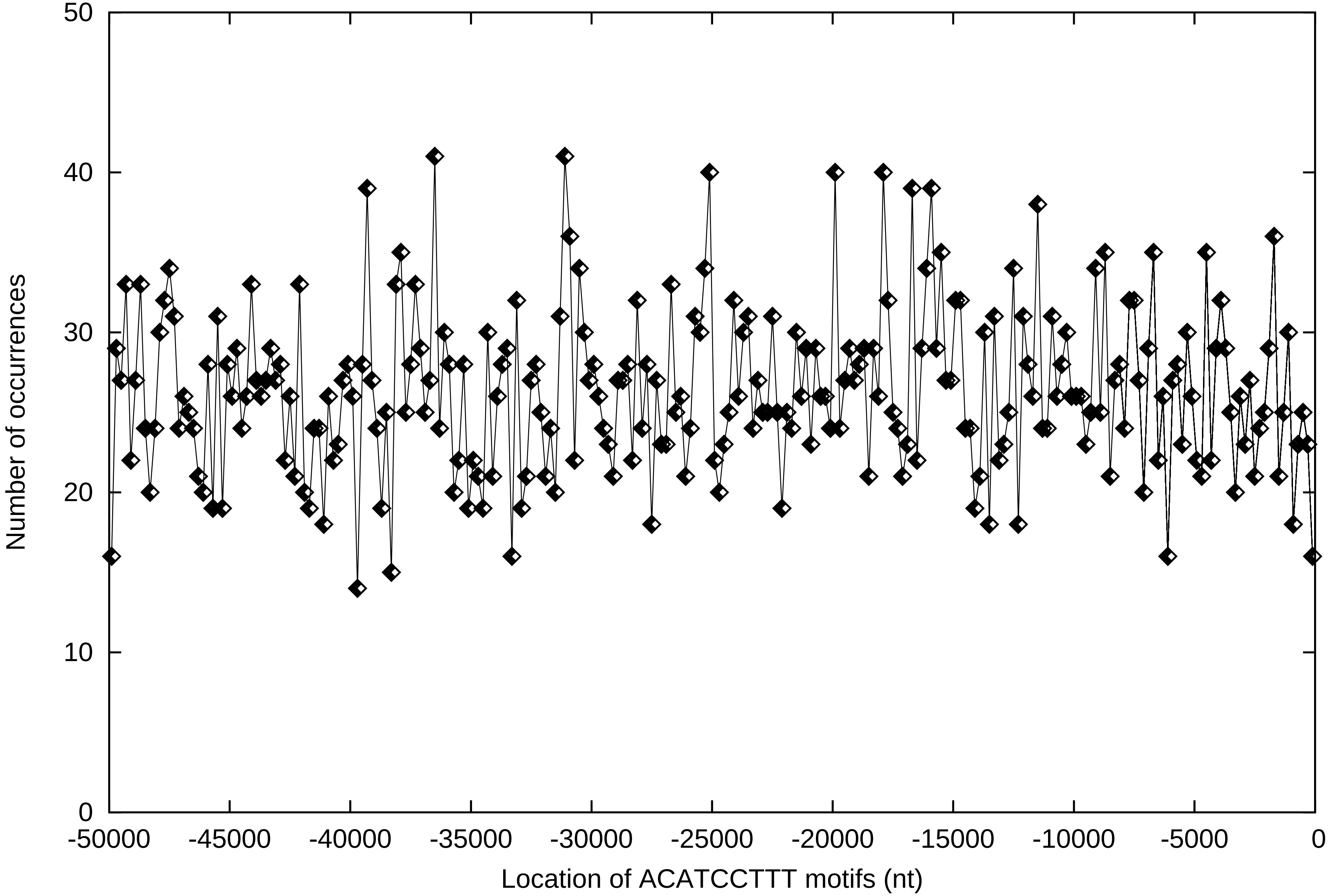

50
40
30
20
10
0
-50000
-45000
-40000
-35000
-30000
-25000
-20000
-15000
-10000
-5000
0
Number of occurrences
Location of ACATCCTTT motifs (nt)

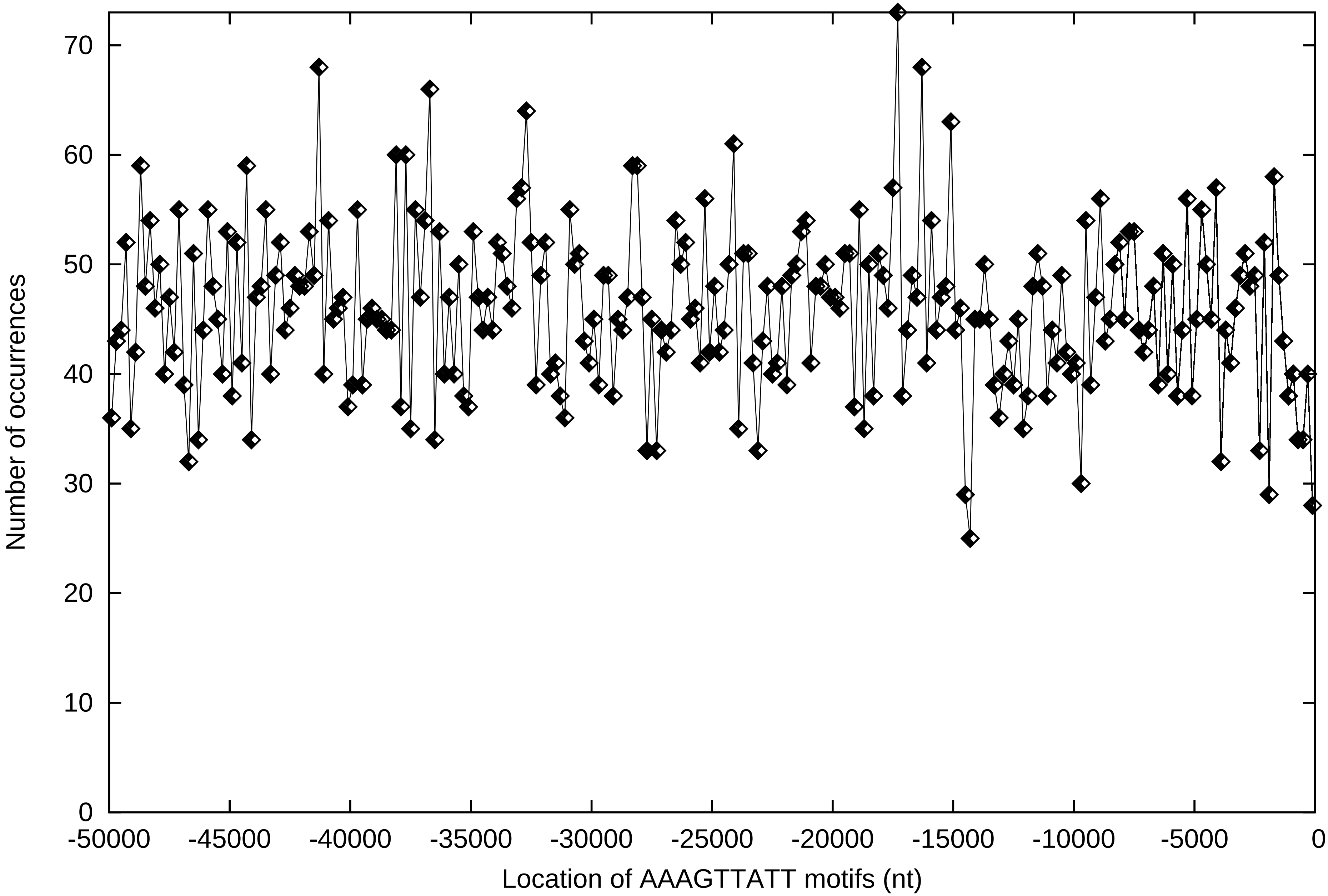

Number of occurrences
Location of AAAGTTATT motifs (nt)
0
10
20
30
40
50
60
70
-50000
-45000
-40000
-35000
-30000
-25000
-20000
-15000
-10000
-5000
0

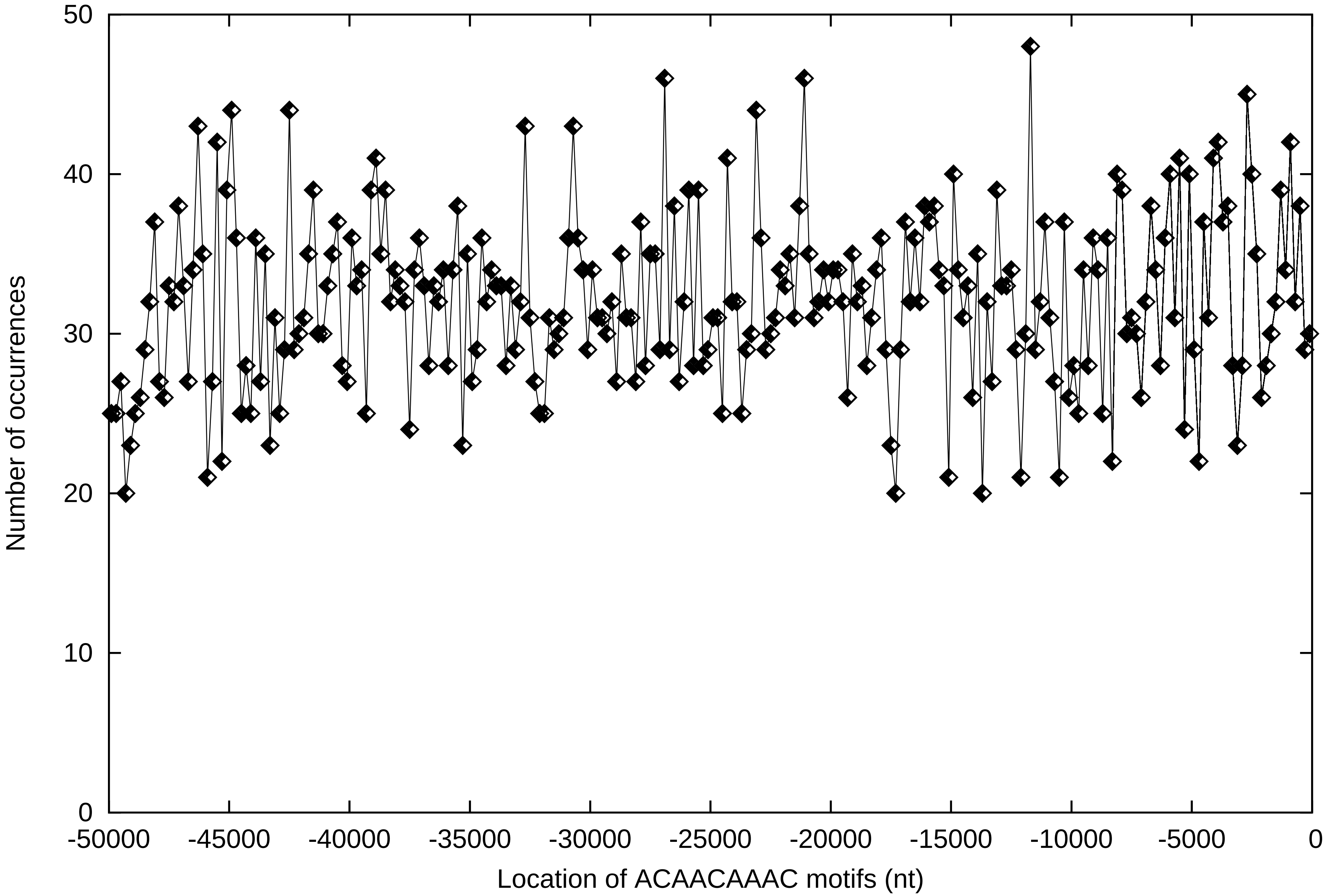

50
40
30
20
10
0
-50000
-45000
-40000
-35000
-30000
-25000
-20000
-15000
-10000
-5000
0
Number of occurrences
Location of ACAACAAAC motifs (nt)

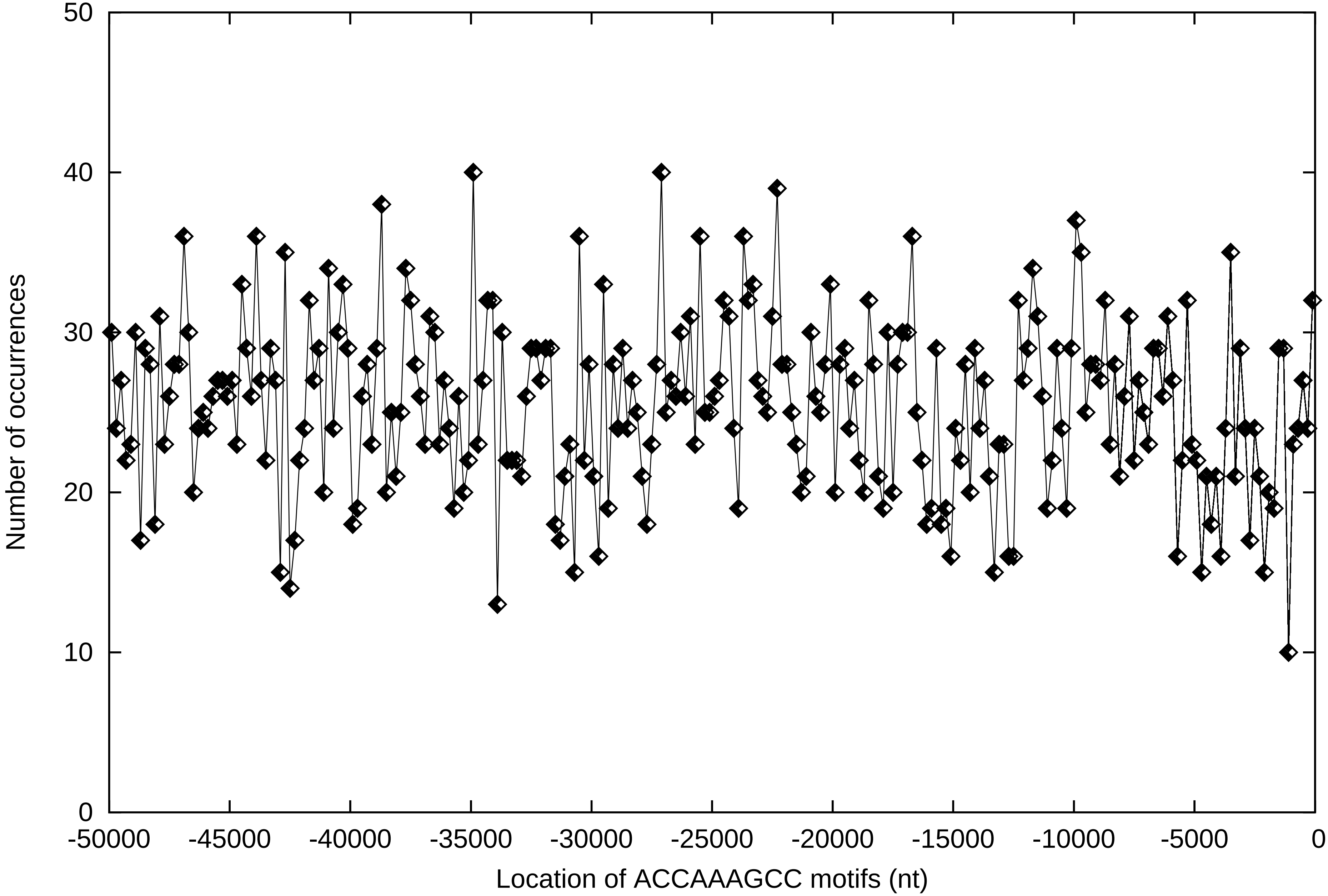

50
40
30
20
10
0
-50000
-45000
-40000
-35000
-30000
-25000
-20000
-15000
-10000
-5000
0
Number of occurrences
Location of ACCAAAGCC motifs (nt)

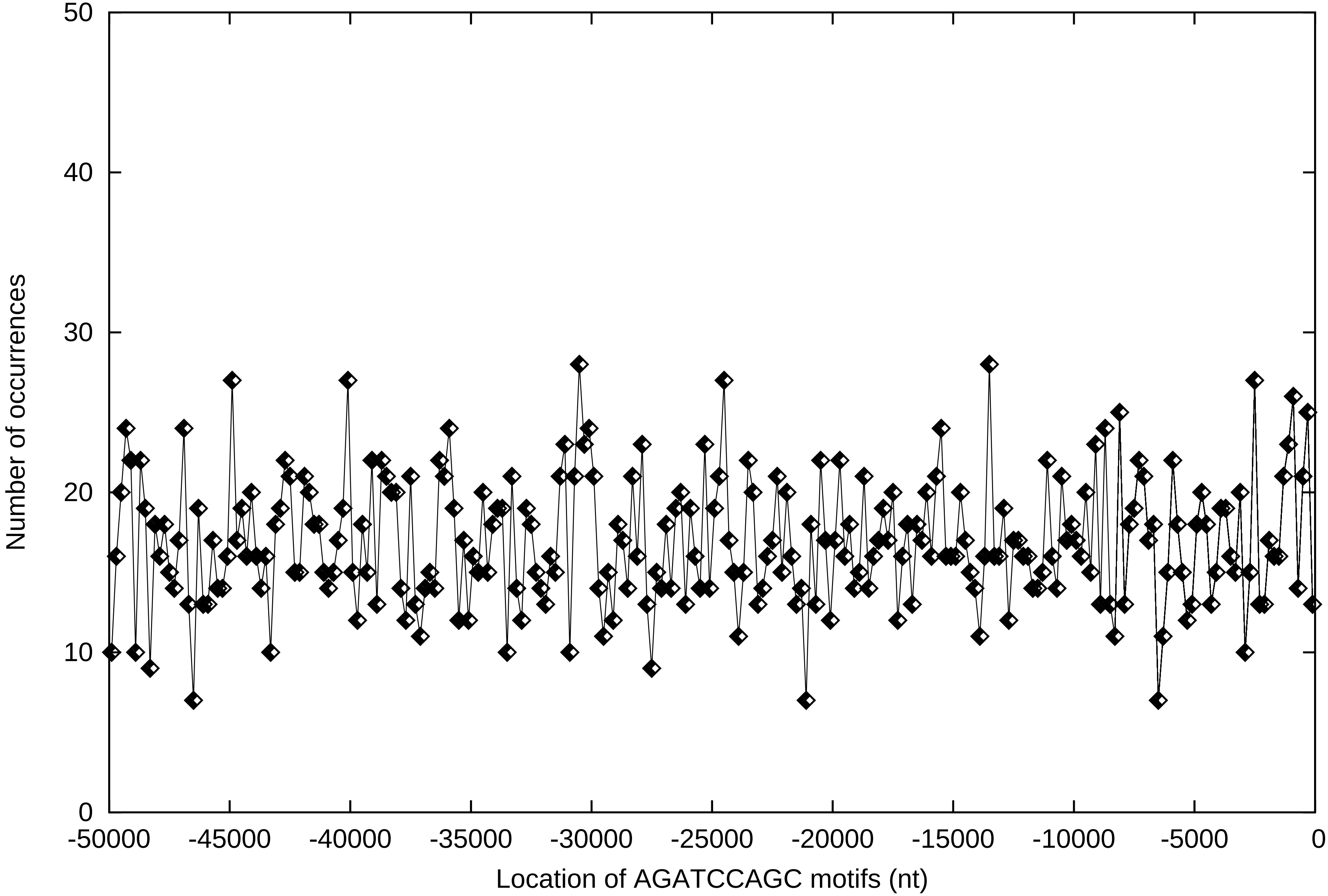

Number of occurrences
Location of AGATCCAGC motifs (nt)
0
10
20
30
40
50
-50000
-45000
-40000
-35000
-30000
-25000
-20000
-15000
-10000
-5000
0

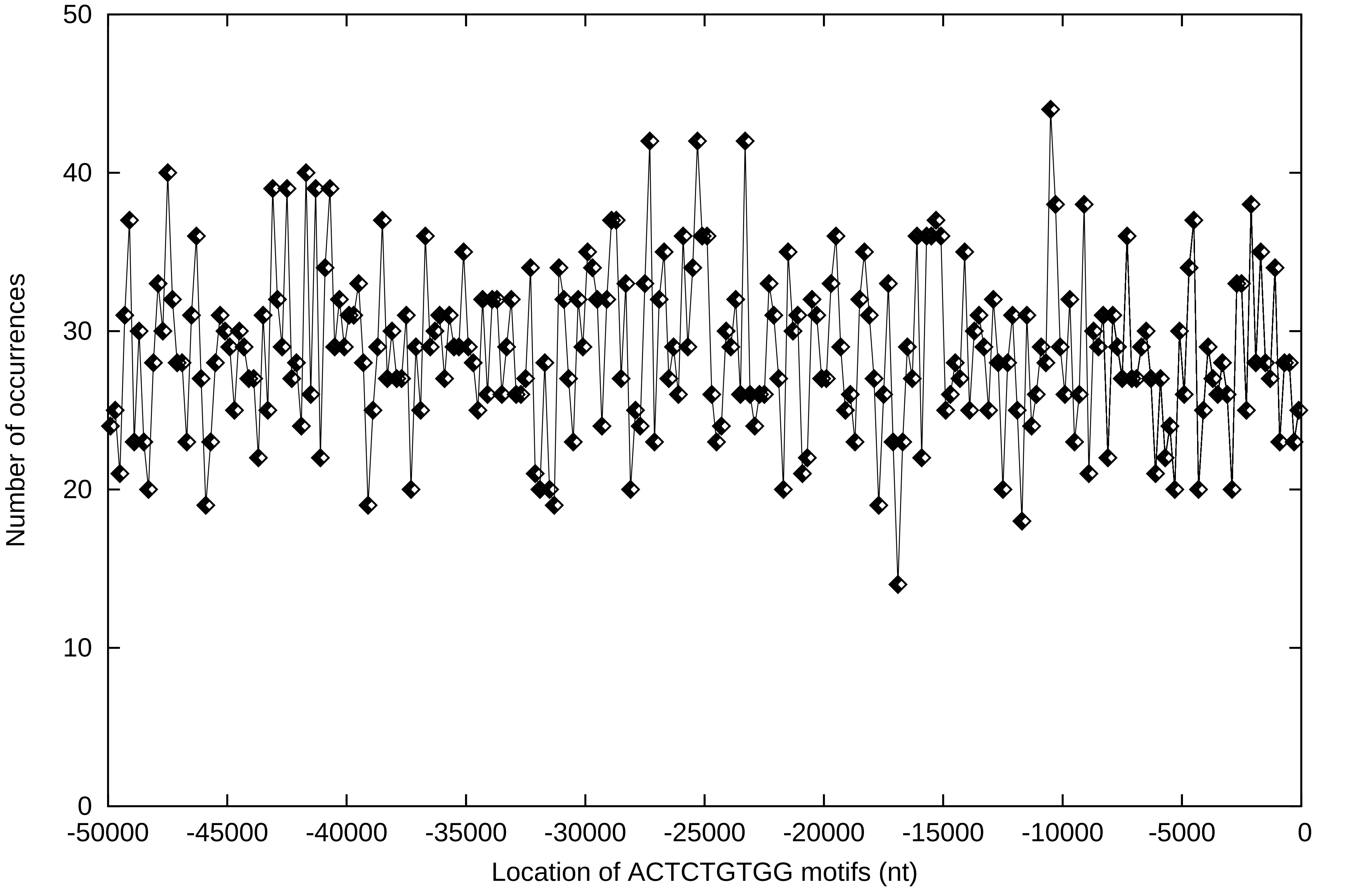

50
40
30
20
10
0
-50000
-45000
-40000
-35000
-30000
-25000
-20000
-15000
-10000
-5000
0
Number of occurrences
Location of ACTCTGTGG motifs (nt)

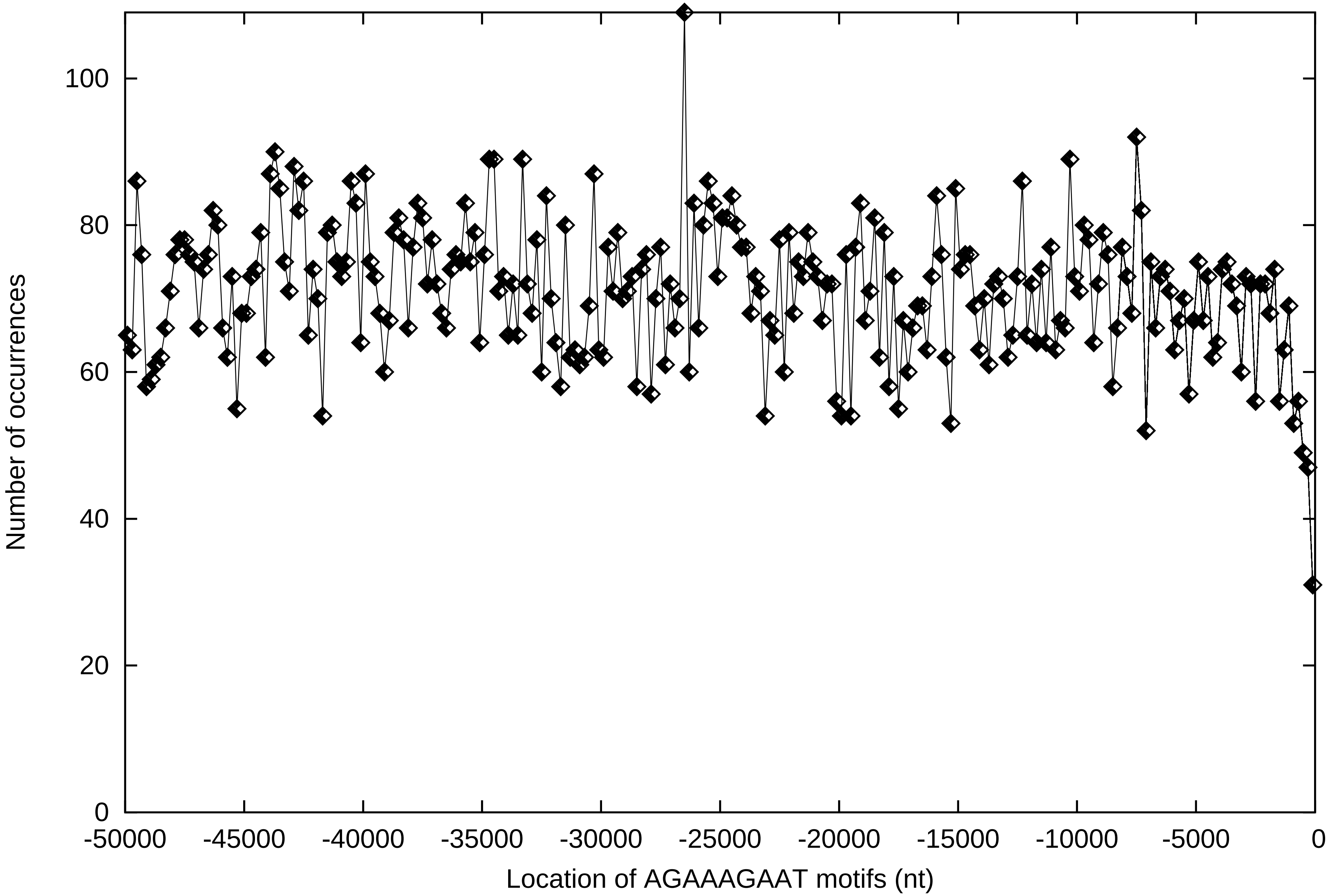

Number of occurrences
Location of AGAAAGAAT motifs (nt)
0
20
40
60
80
100
-50000
-45000
-40000
-35000
-30000
-25000
-20000
-15000
-10000
-5000
0

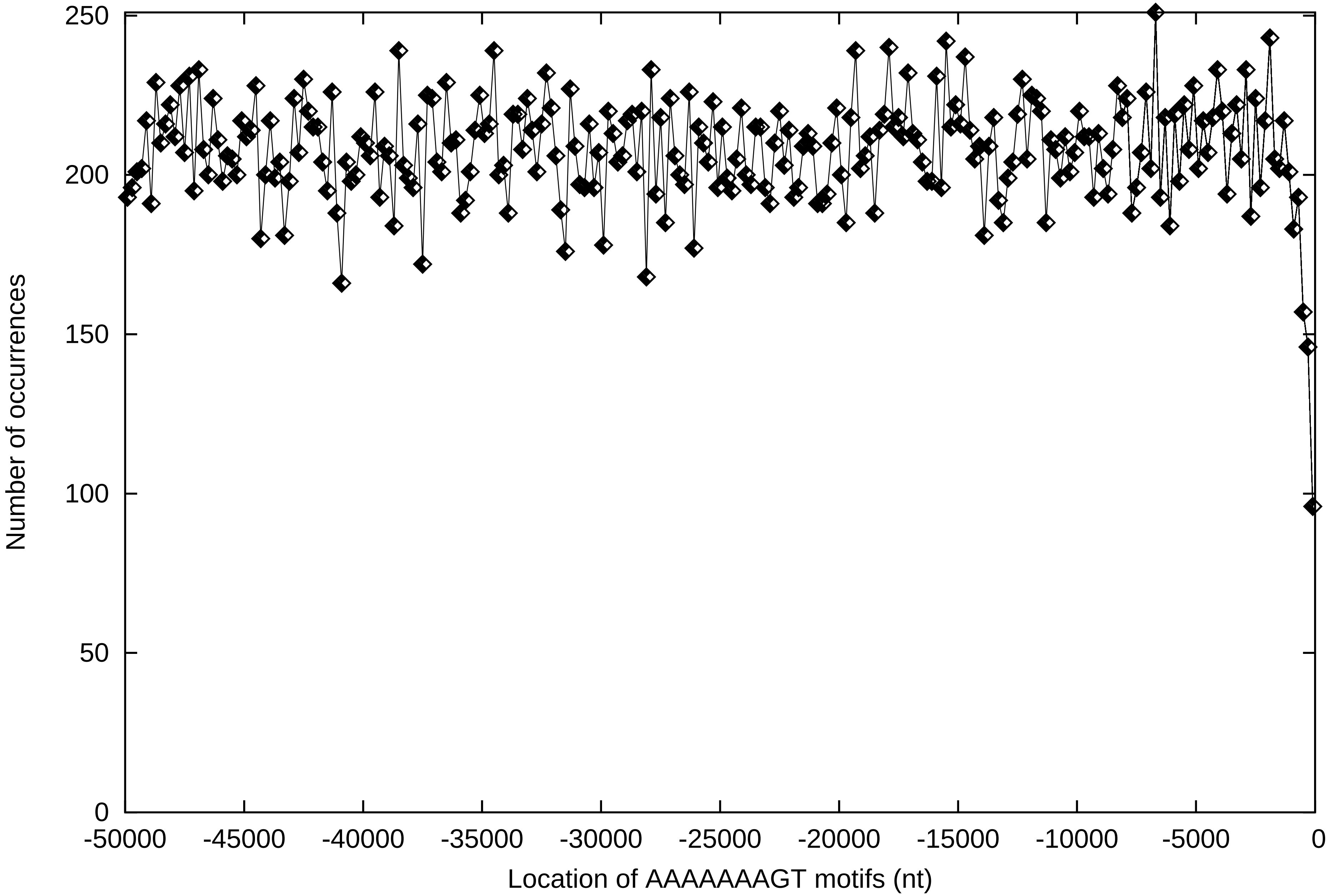

250
200
150
100
50
0
-50000
-45000
-40000
-35000
-30000
-25000
-20000
-15000
-10000
-5000
0
Number of occurrences
Location of AAAAAAAGT motifs (nt)

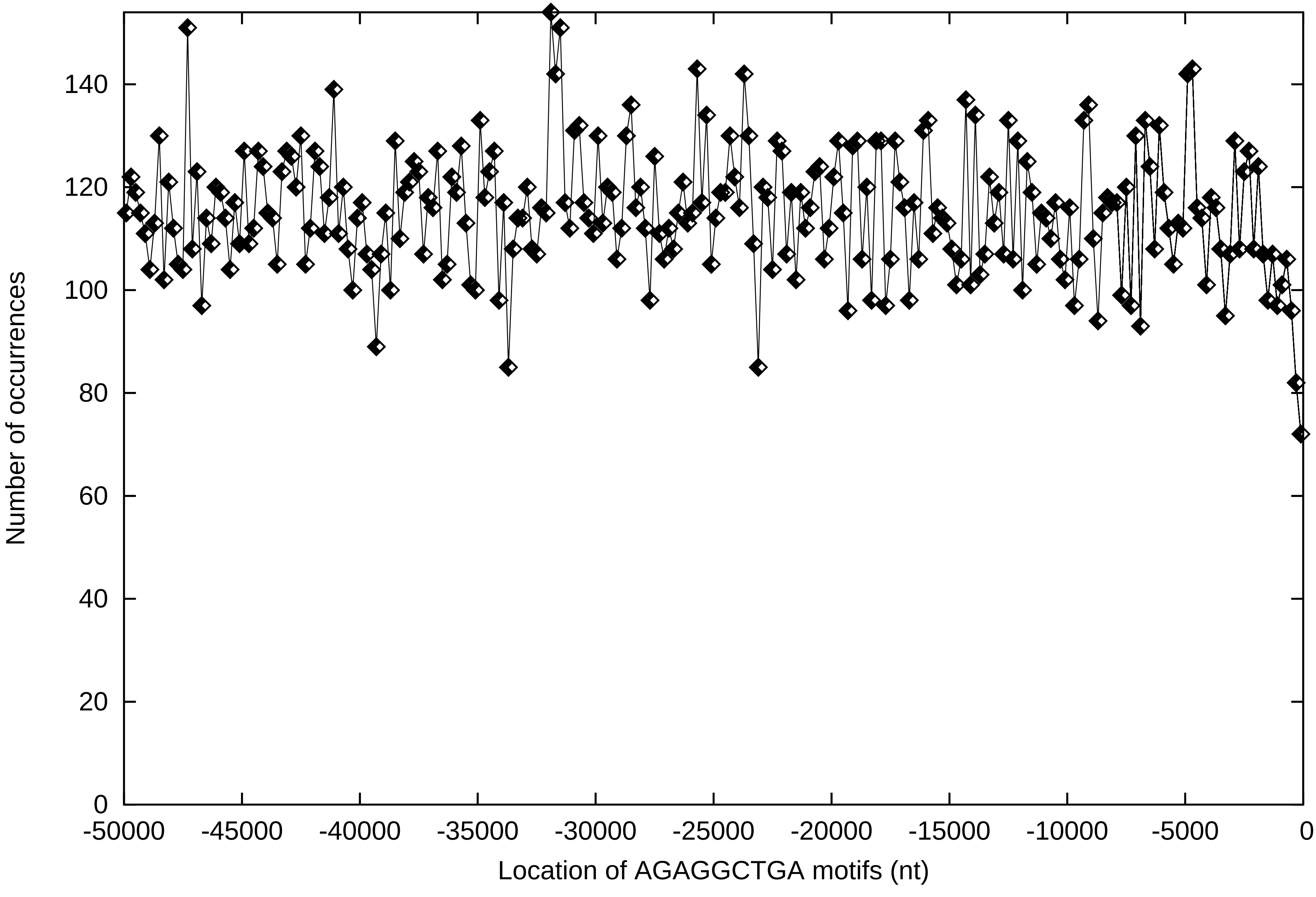

Number of occurrences
0
20
40
60
80
100
120
140
-50000
-45000
-40000
-35000
-30000
-25000
-20000
-15000
-10000
-5000
0
Location of AGAGGCTGA motifs (nt)

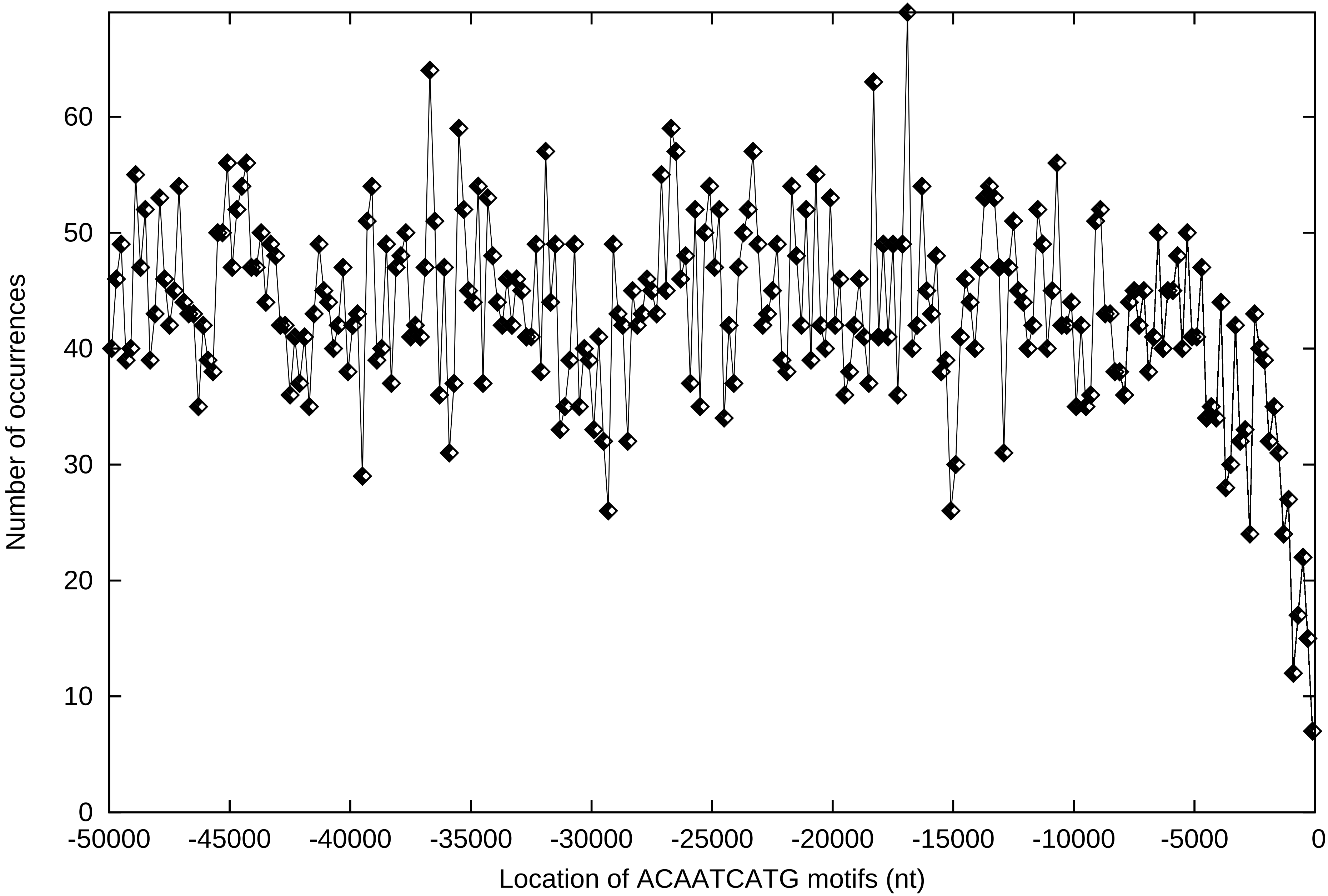
Number of occurrences
Location of ACAATCATG motifs (nt)
0
10
20
30
40
50
60
-50000
-45000
-40000
-35000
-30000
-25000
-20000
-15000
-10000
-5000
0

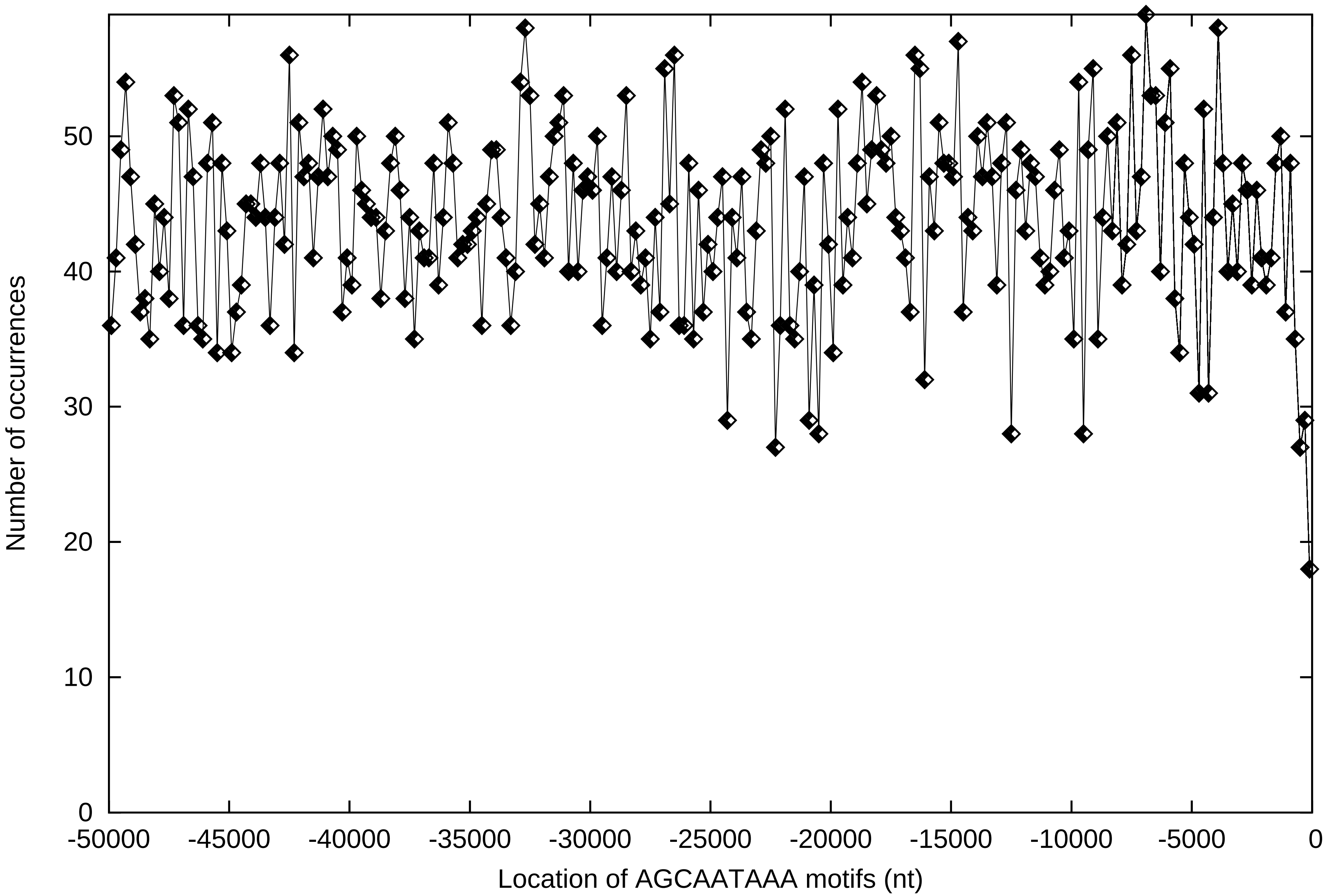
Number of occurrences
0
10
20
30
40
50
-50000
-45000
-40000
-35000
-30000
-25000
-20000
-15000
-10000
-5000
0
Location of AGCAATAAA motifs (nt)

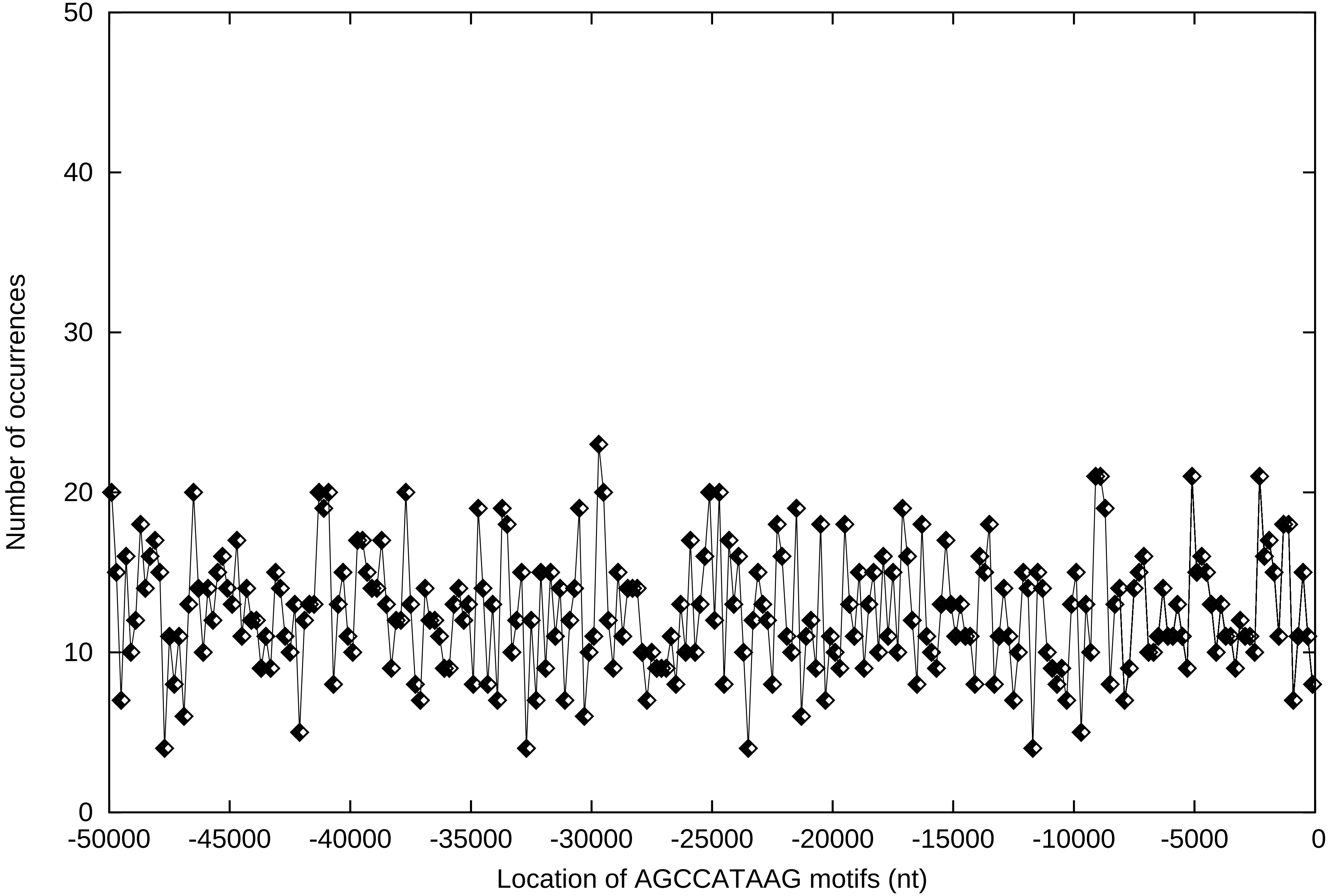
50
40
30
20
10
0
Number of occurrences
-50000
-45000
-40000
-35000
-30000
-25000
-20000
-15000
-10000
-5000
0
Location of AGCCATAAG motifs (nt)

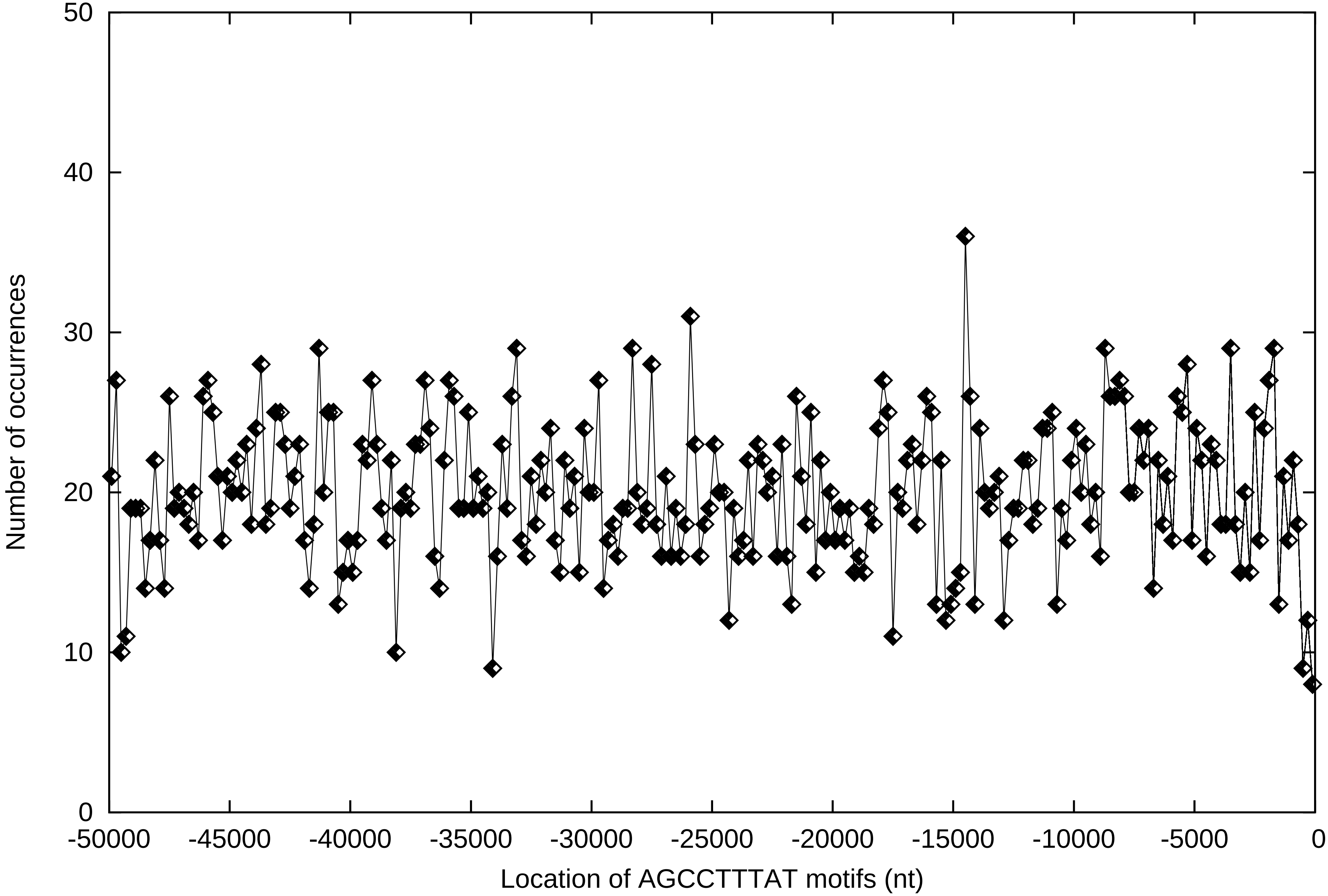

50
40
30
20
10
0
-50000
-45000
-40000
-35000
-30000
-25000
-20000
-15000
-10000
-5000
0
Number of occurrences
Location of AGCCTTTAT motifs (nt)

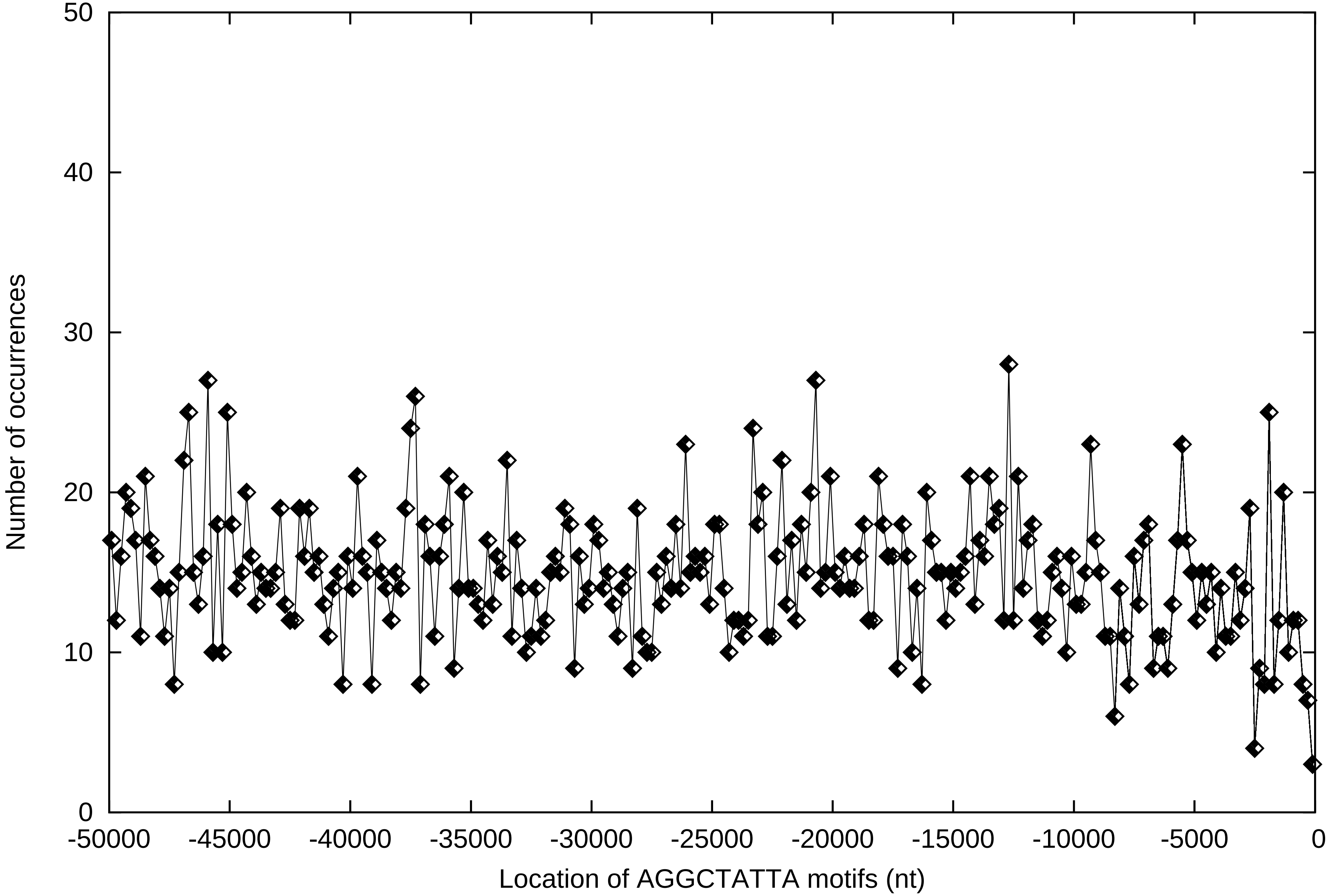

Number of occurrences
Location of AGGCTATTA motifs (nt)
0
10
20
30
40
50
-50000
-45000
-40000
-35000
-30000
-25000
-20000
-15000
-10000
-5000
0

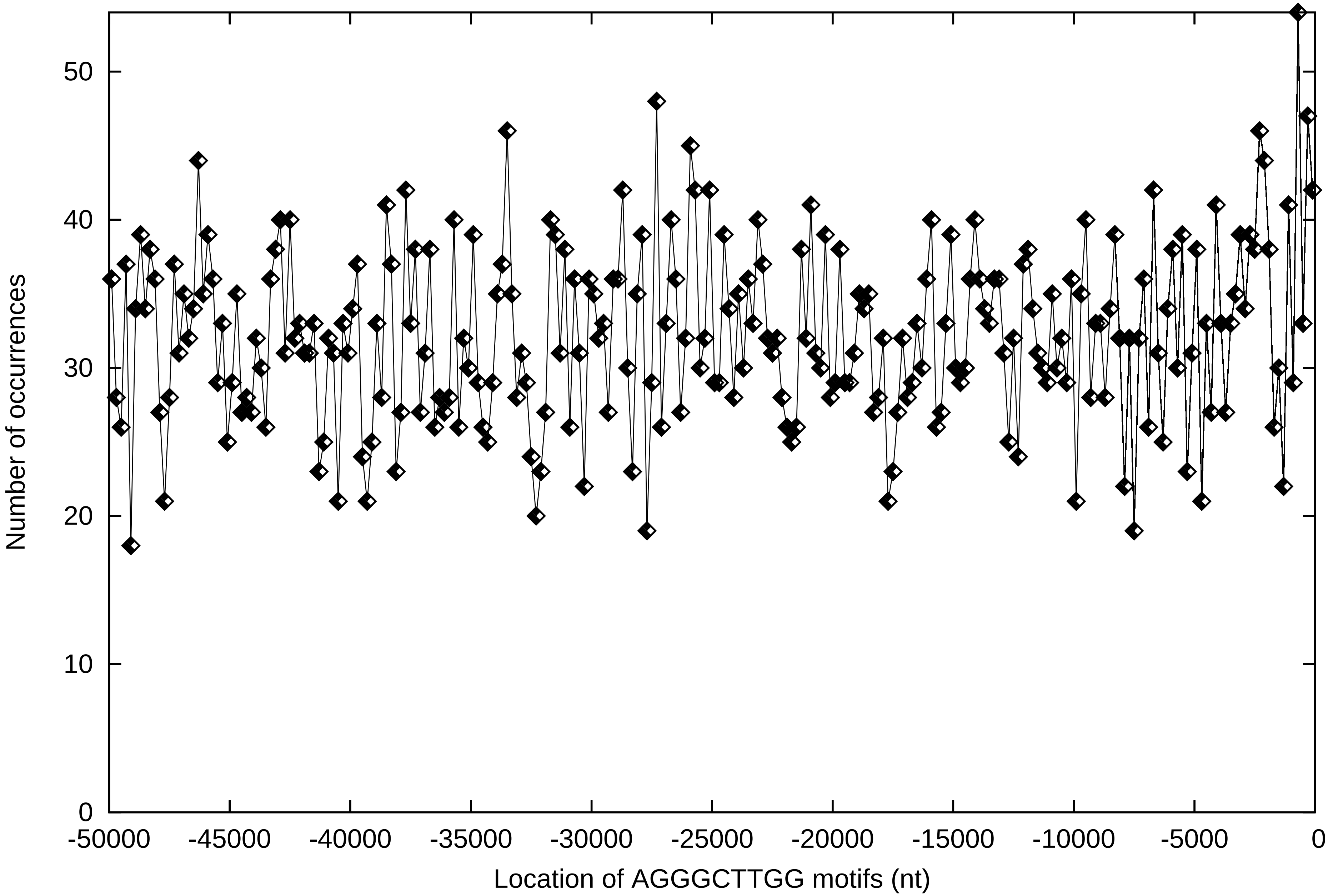

Number of occurrences
0
10
20
30
40
50
-50000
-45000
-40000
-35000
-30000
-25000
-20000
-15000
-10000
-5000
0
Location of AGGGCTTGG motifs (nt)

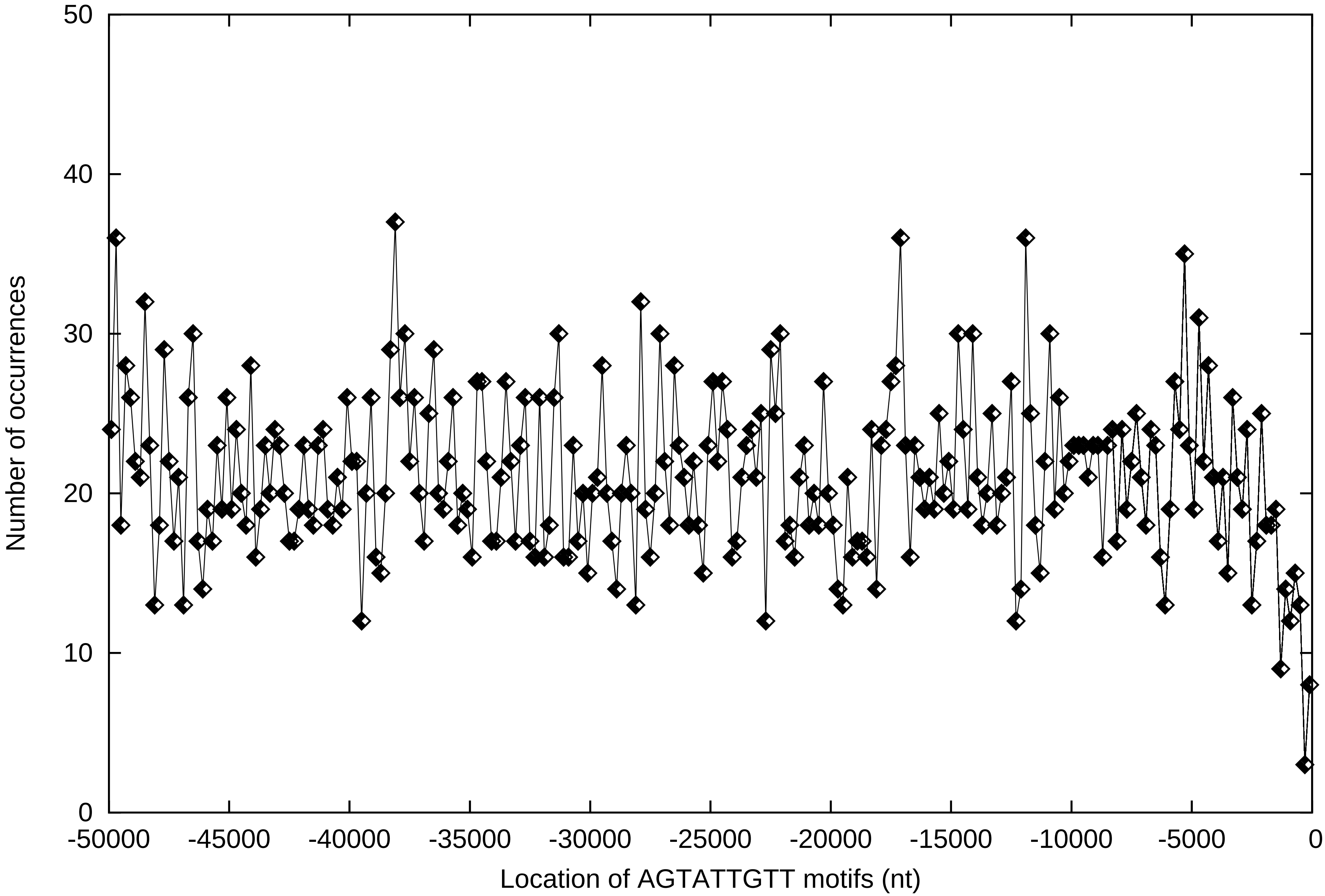

50
40
30
20
10
0
-50000
-45000
-40000
-35000
-30000
-25000
-20000
-15000
-10000
-5000
0
Number of occurrences
Location of AGTATTGTT motifs (nt)

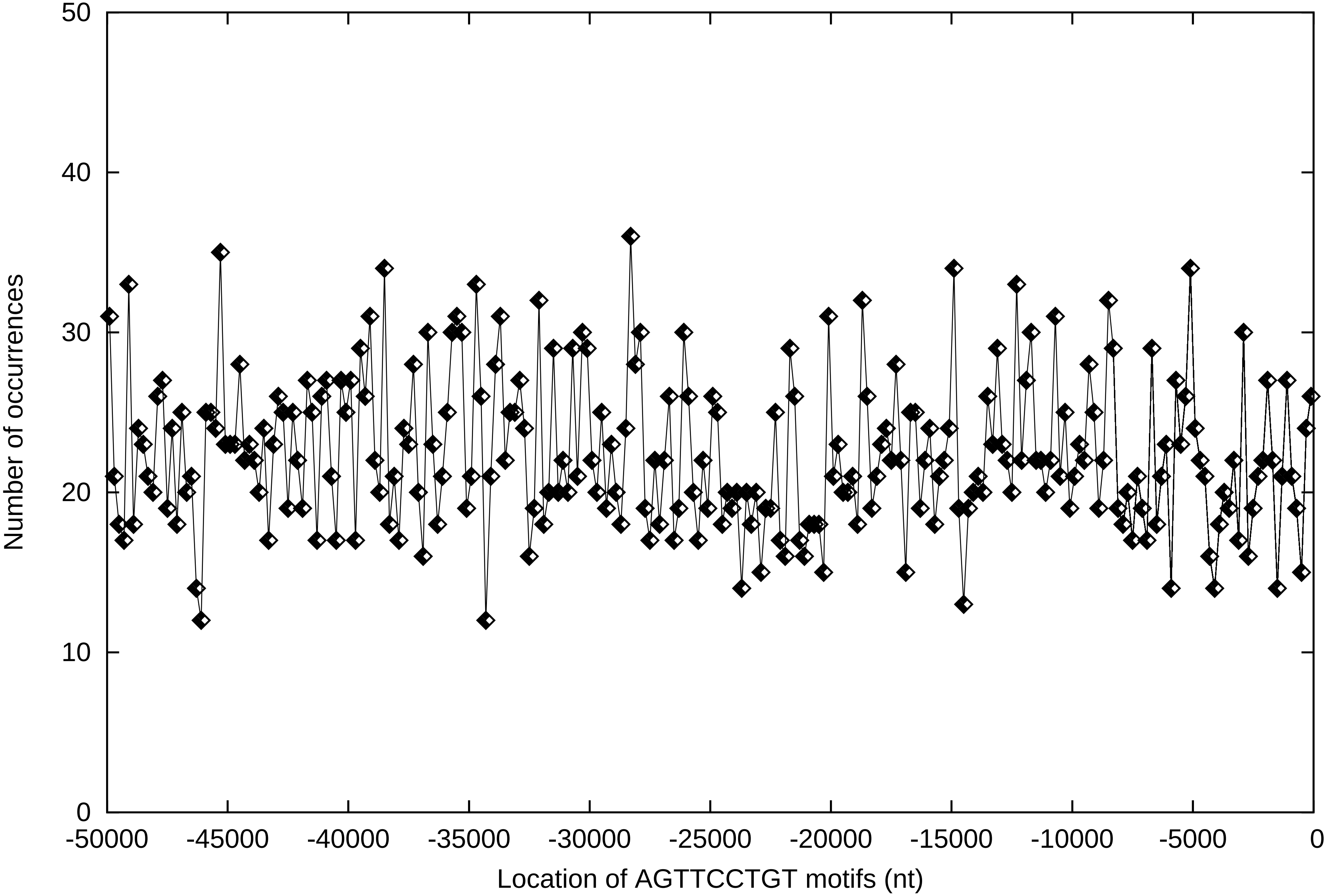

Number of occurrences
Location of AGTTCCTGT motifs (nt)
0
10
20
30
40
50
-50000
-45000
-40000
-35000
-30000
-25000
-20000
-15000
-10000
-5000
0

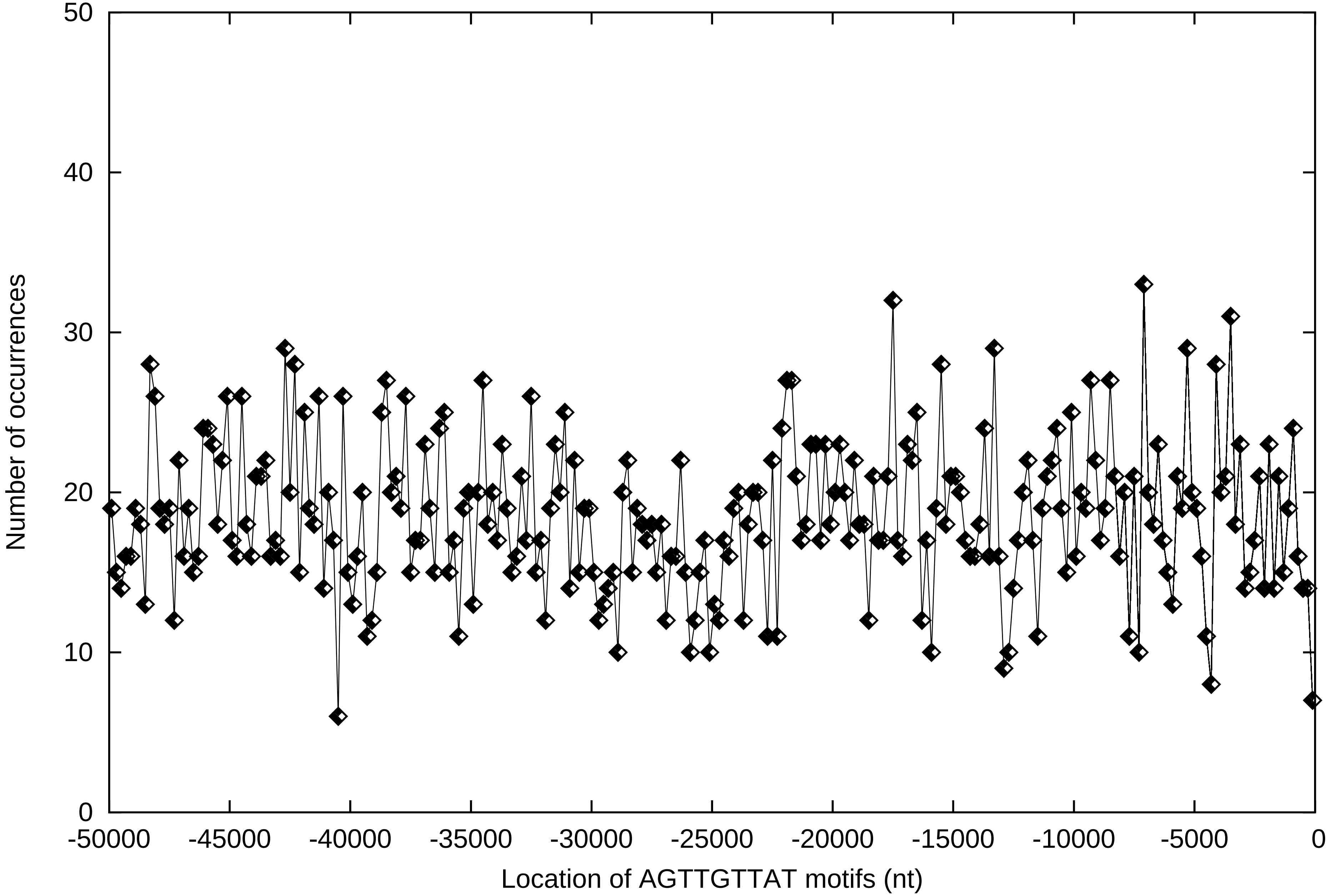

50
40
30
20
10
0
-50000
-45000
-40000
-35000
-30000
-25000
-20000
-15000
-10000
-5000
0
Number of occurrences
Location of AGTTGTTAT motifs (nt)

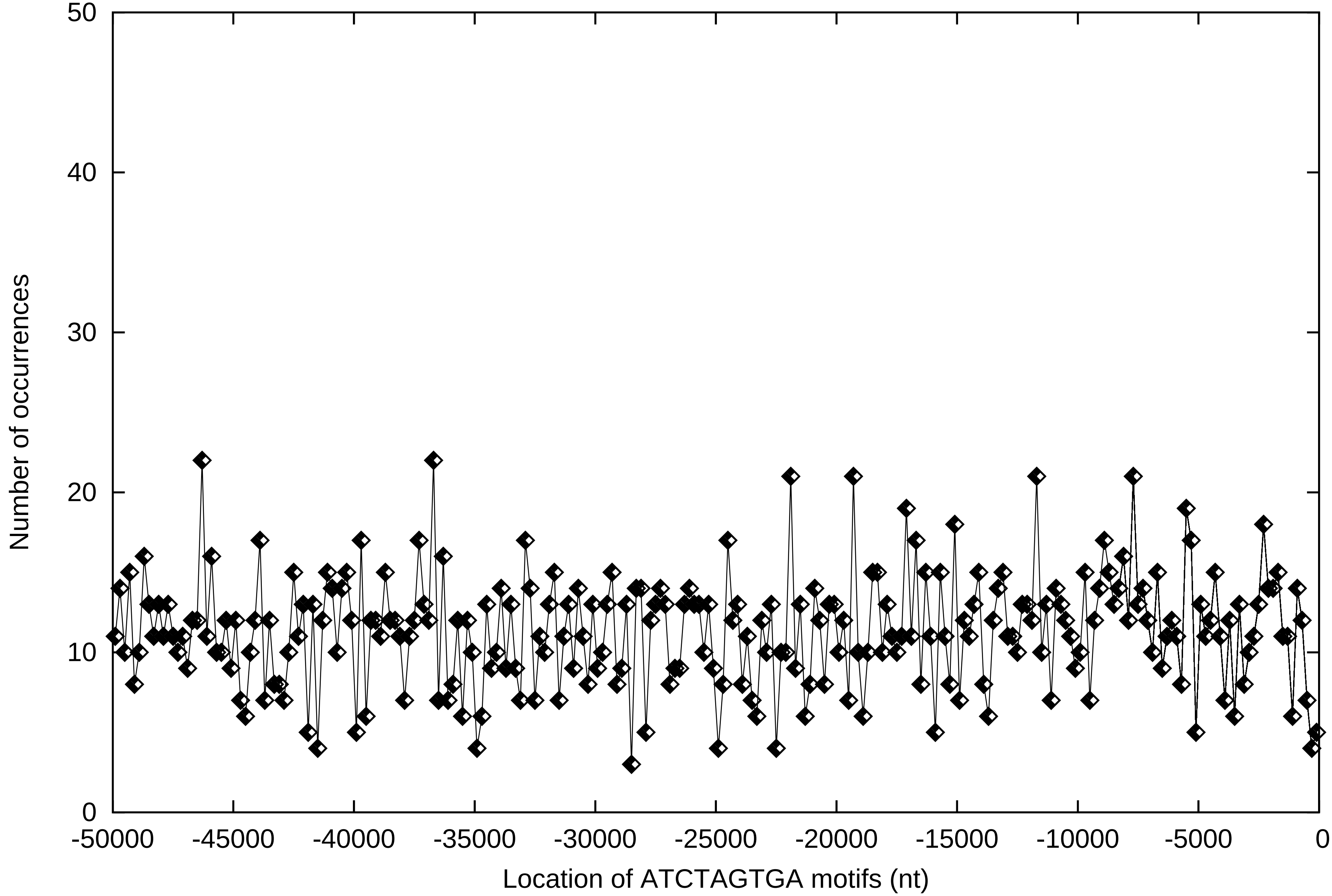

50
40
30
20
10
0
-50000
-45000
-40000
-35000
-30000
-25000
-20000
-15000
-10000
-5000
0
Number of occurrences
Location of ATCTAGTGA motifs (nt)

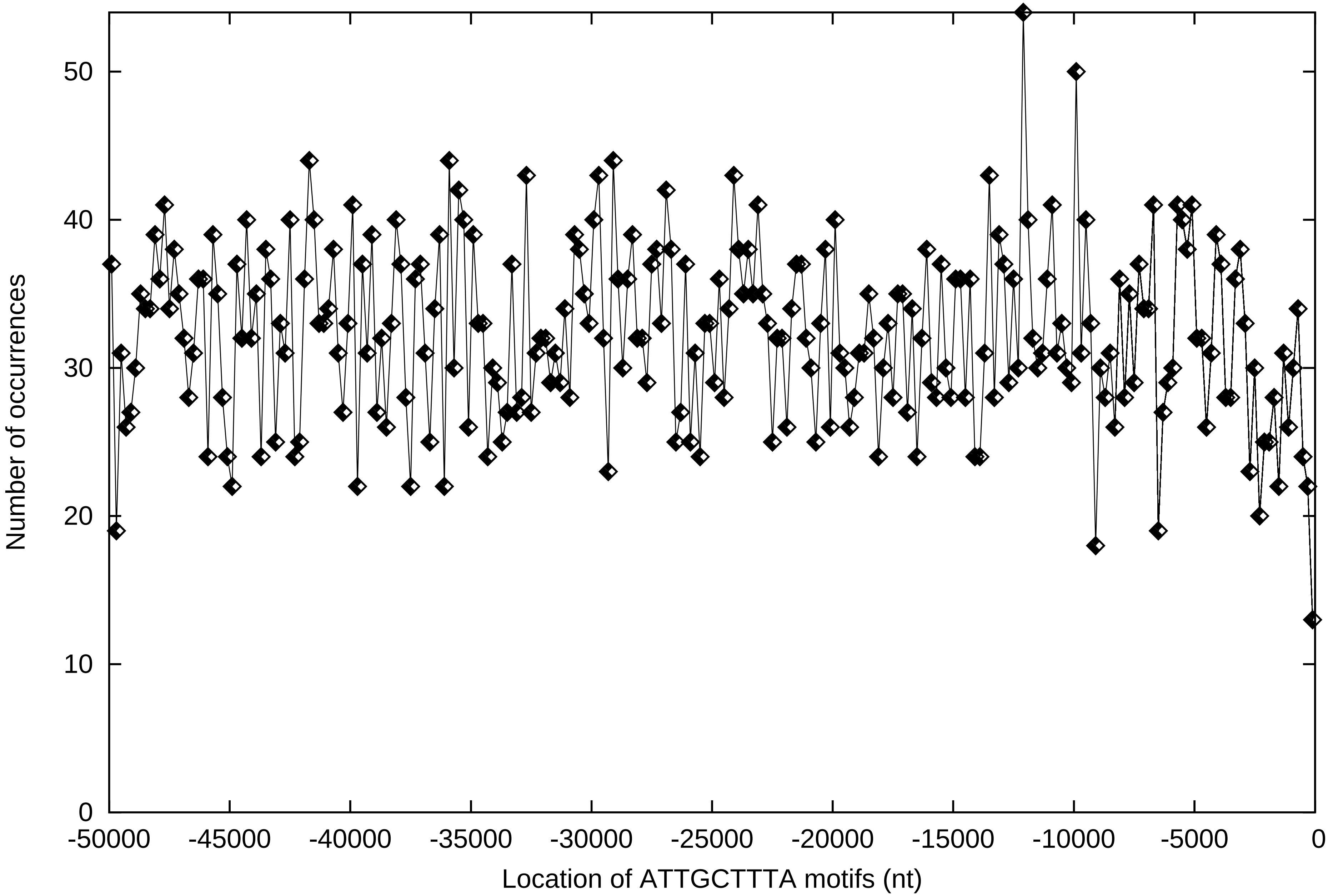

Number of occurrences
Location of ATTGCTTTA motifs (nt)
0
10
20
30
40
50
-50000
-45000
-40000
-35000
-30000
-25000
-20000
-15000
-10000
-5000
0

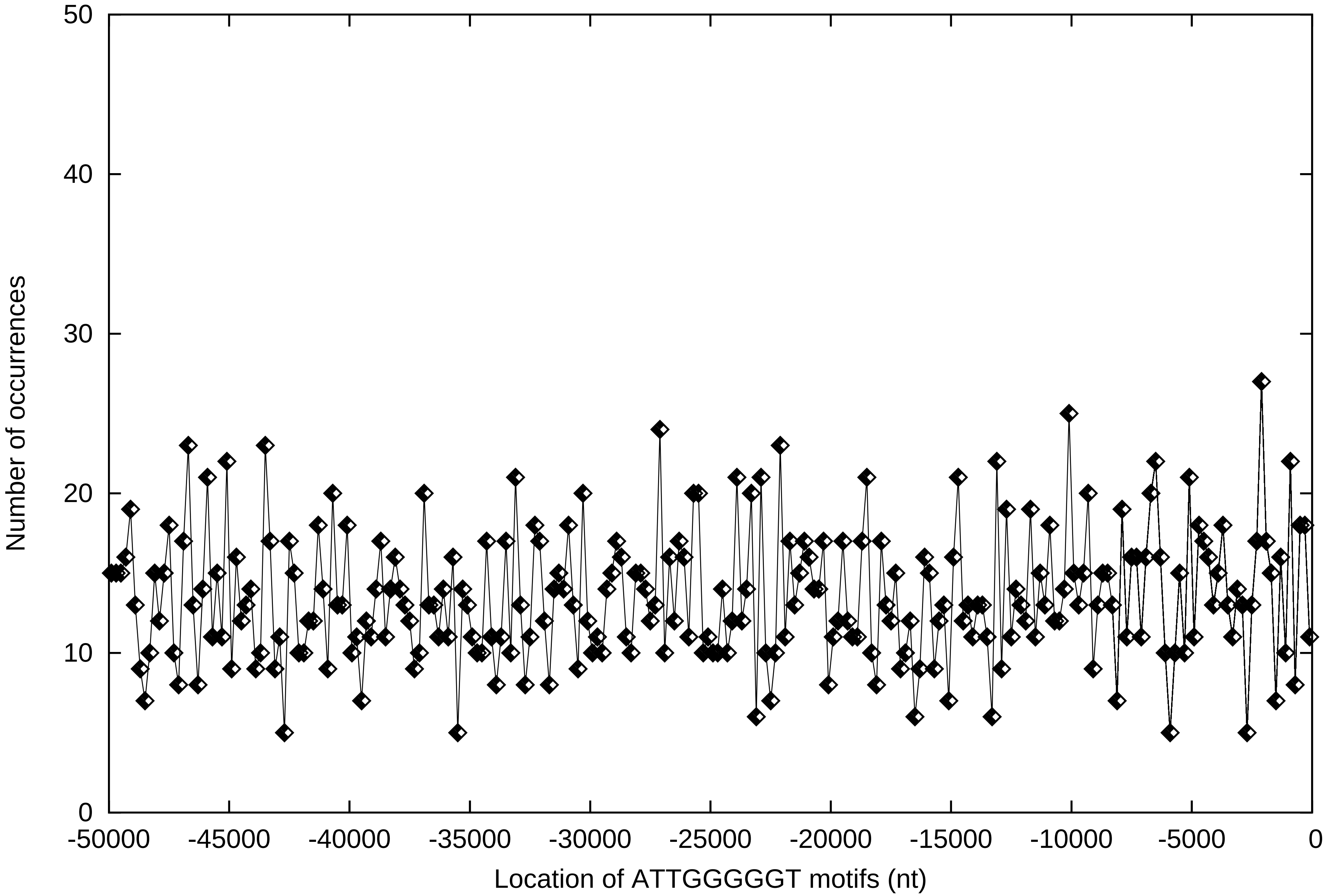

Number of occurrences
Location of ATTGGGGGT motifs (nt)
0
10
20
30
40
50
-50000
-45000
-40000
-35000
-30000
-25000
-20000
-15000
-10000
-5000
0

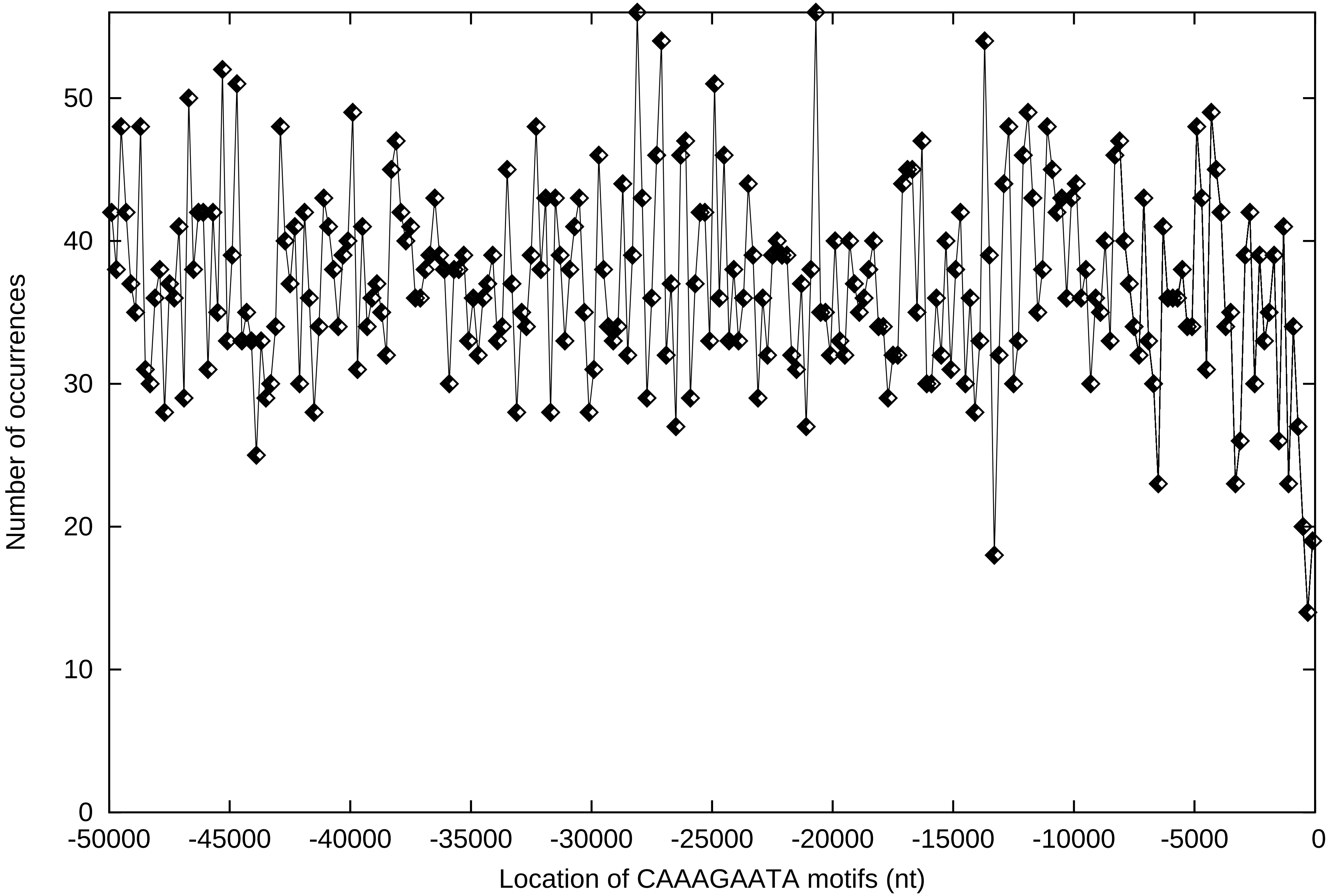

Number of occurrences
0
10
20
30
40
50
-50000
-45000
-40000
-35000
-30000
-25000
-20000
-15000
-10000
-5000
0
Location of CAAAGAATA motifs (nt)

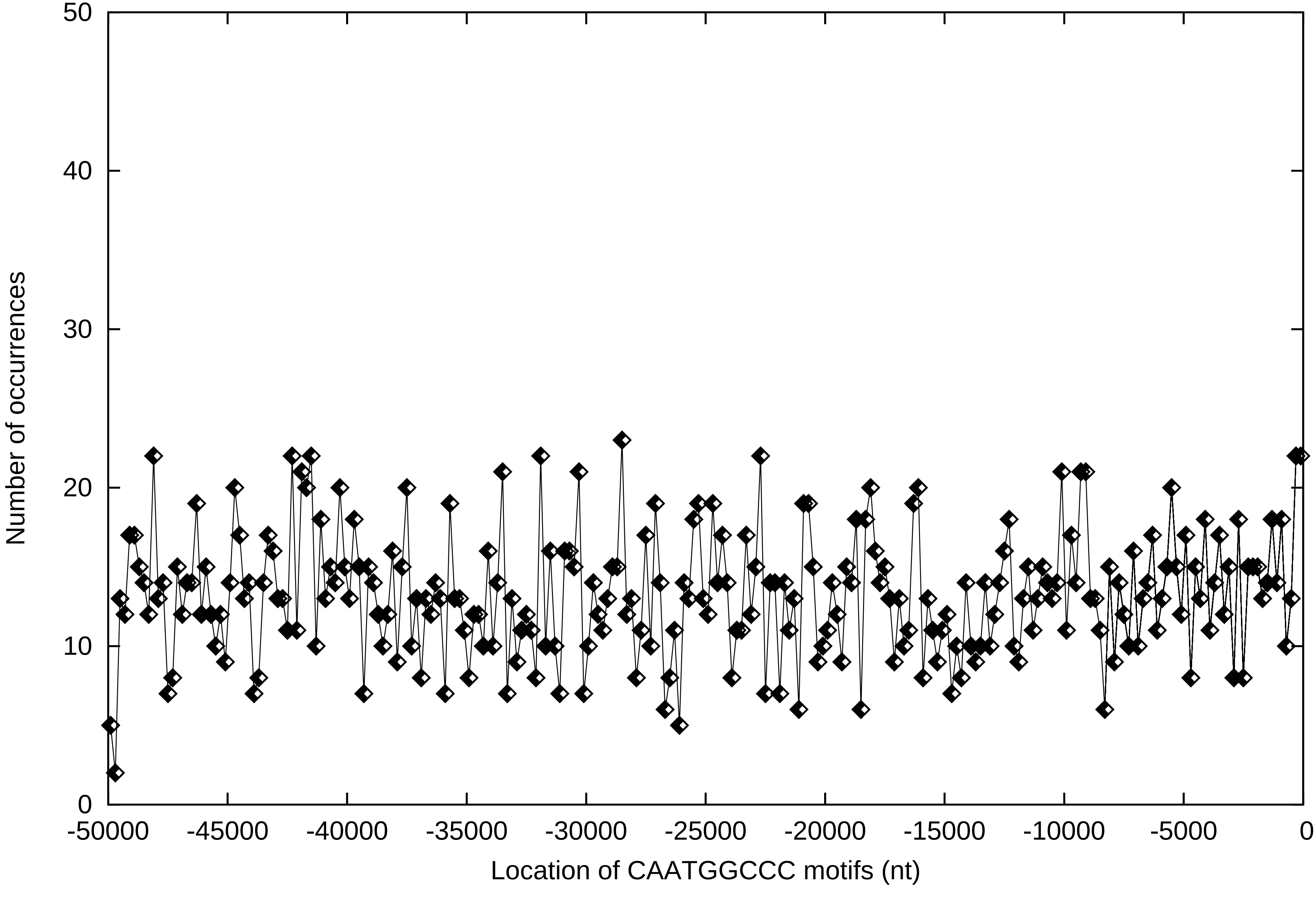

Number of occurrences
50
40
30
20
10
0
-50000
-45000
-40000
-35000
-30000
-25000
-20000
-15000
-10000
-5000
0
Location of CAATGGCCC motifs (nt)

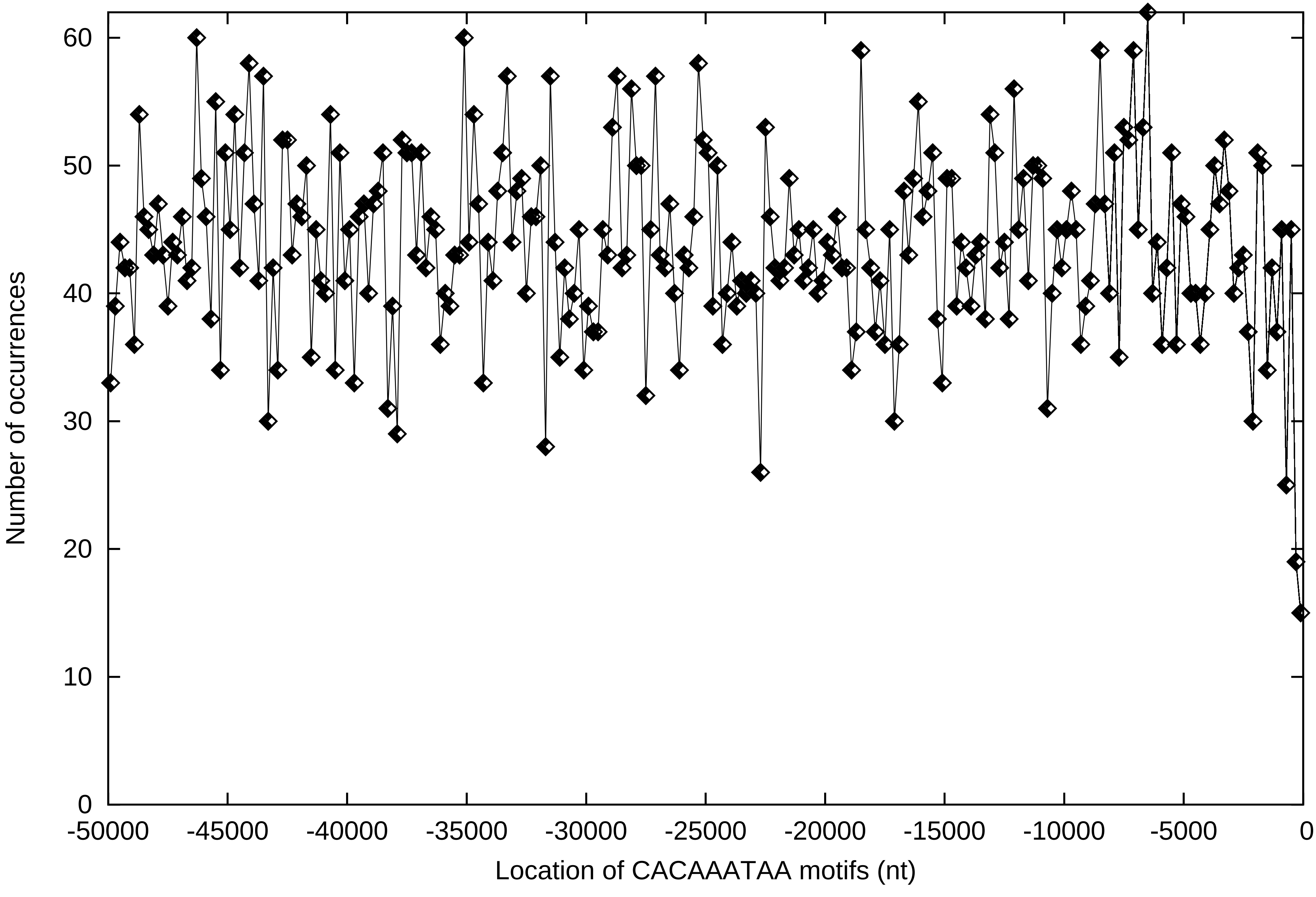

Number of occurrences
Location of CACAAATAA motifs (nt)
0
10
20
30
40
50
60
-50000
-45000
-40000
-35000
-30000
-25000
-20000
-15000
-10000
-5000
0

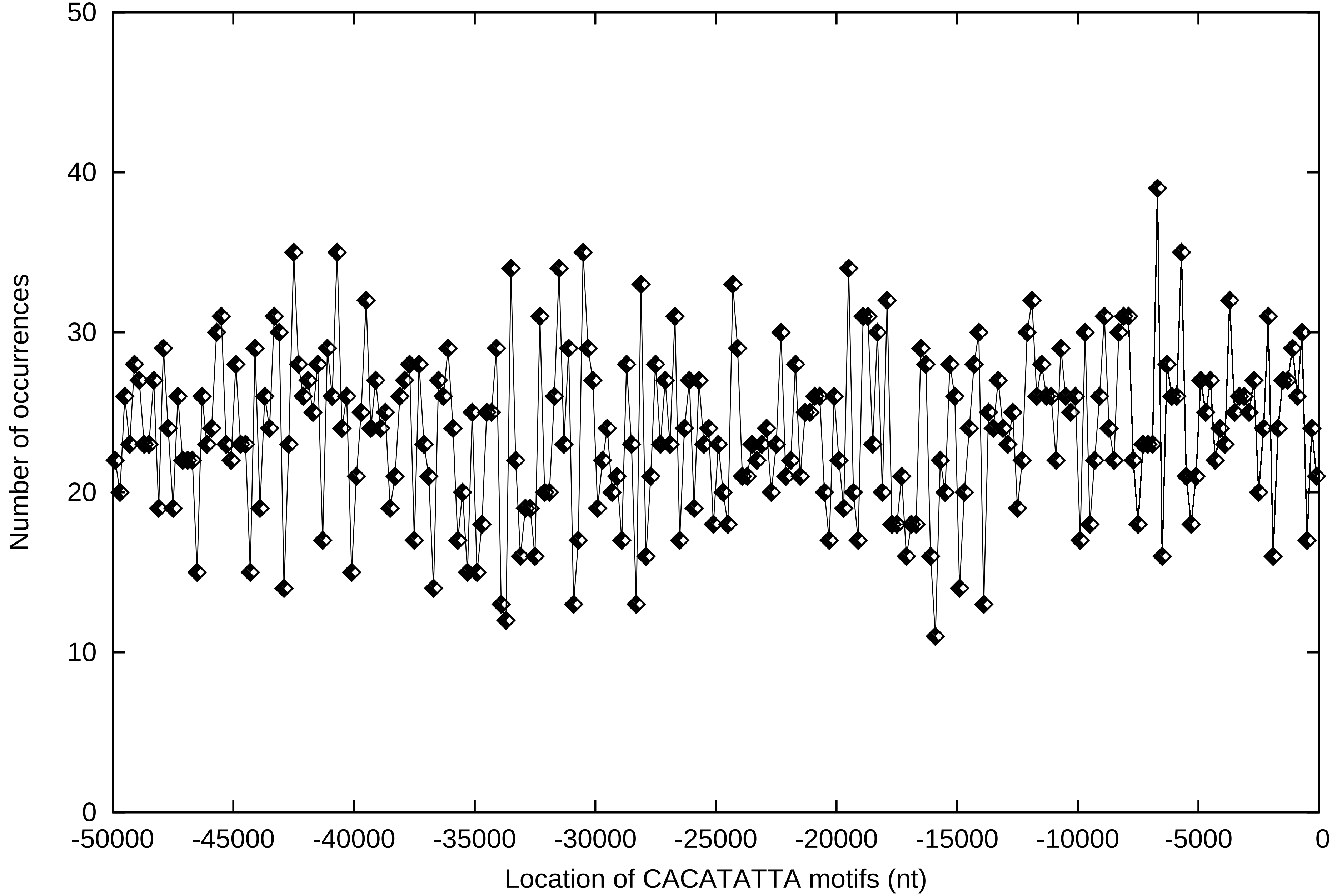
Number of occurrences
0
10
20
30
40
50
-50000
-45000
-40000
-35000
-30000
-25000
-20000
-15000
-10000
-5000
0
Location of CACATATTA motifs (nt)

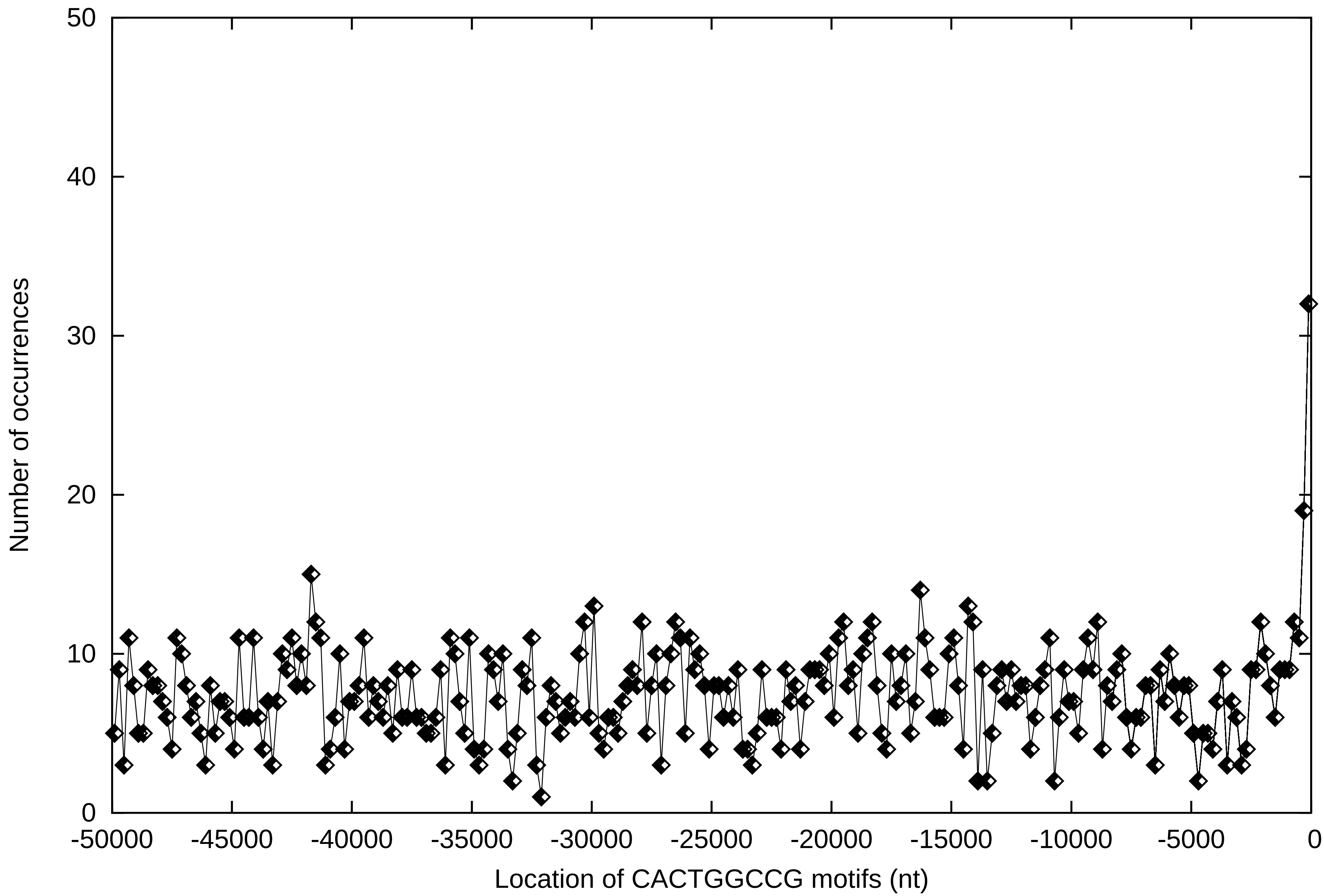
50
40
30
20
10
0
Number of occurrences
-50000
-45000
-40000
-35000
-30000
-25000
-20000
-15000
-10000
-5000
0
Location of CACTGGCCG motifs (nt)

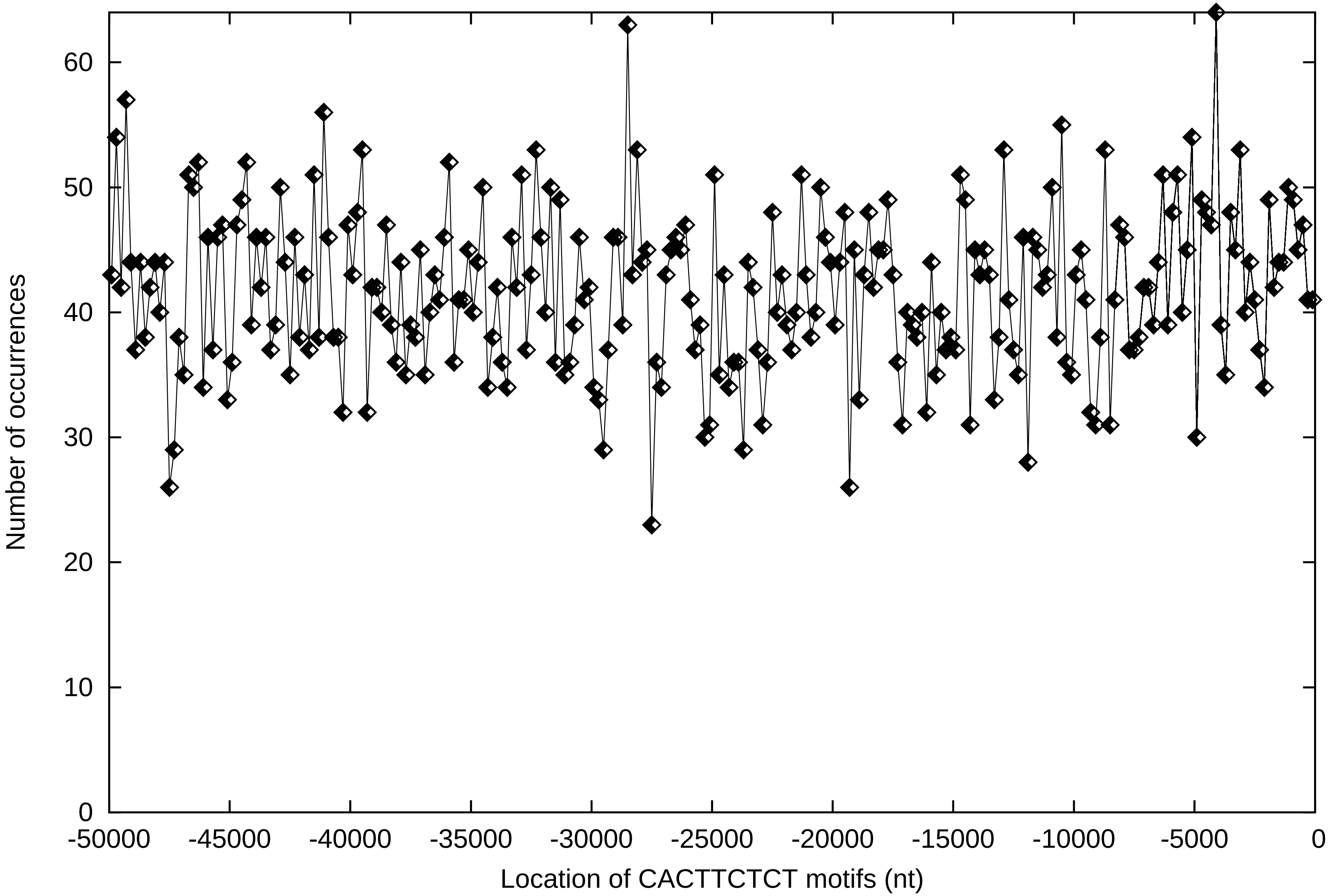
Number of occurrences
Location of CACTTCTCT motifs (nt)
0
10
20
30
40
50
60
-50000
-45000
-40000
-35000
-30000
-25000
-20000
-15000
-10000
-5000
0

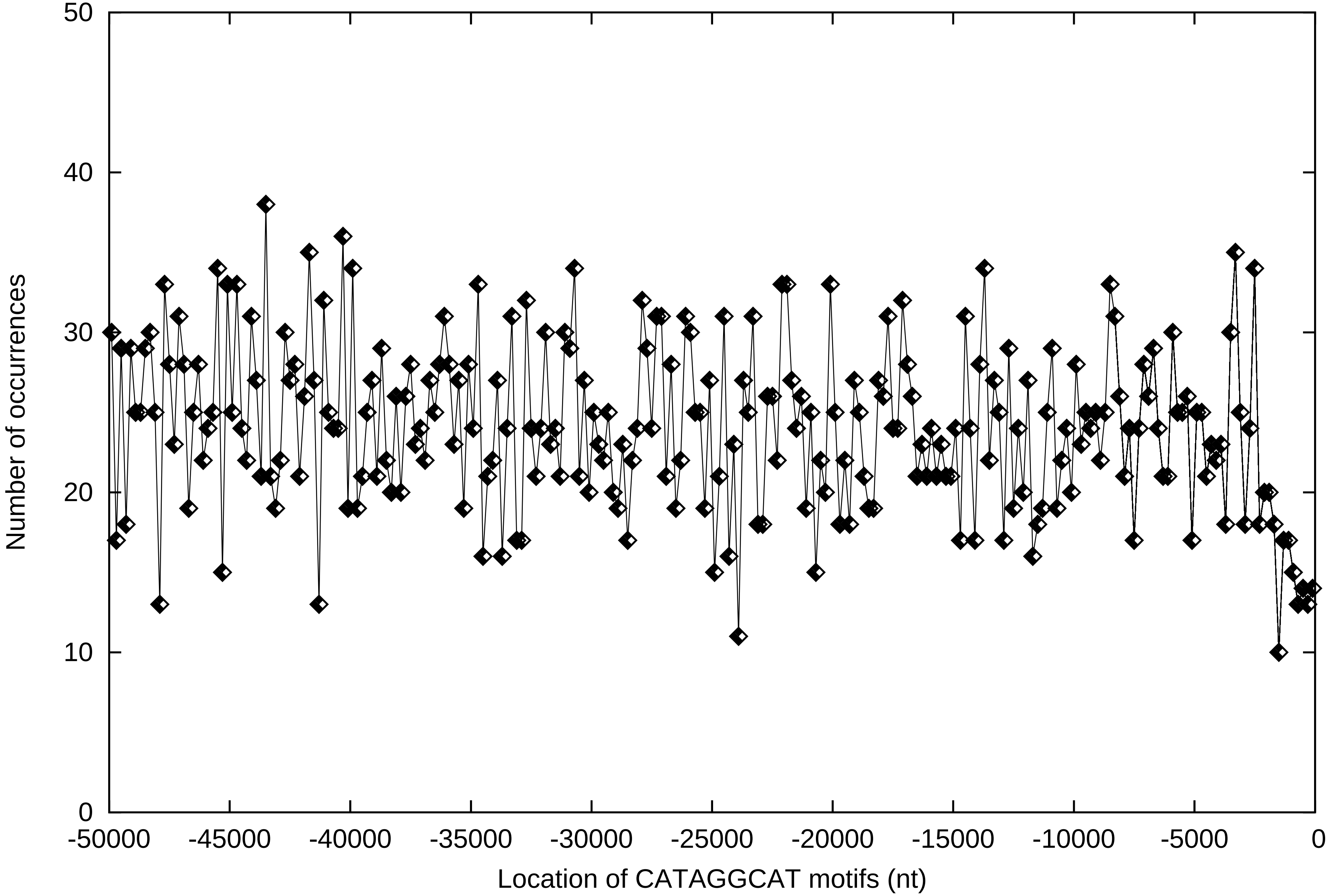

50
40
30
20
10
0
Number of occurrences
-50000
-45000
-40000
-35000
-30000
-25000
-20000
-15000
-10000
-5000
0
Location of CATAGGCAT motifs (nt)

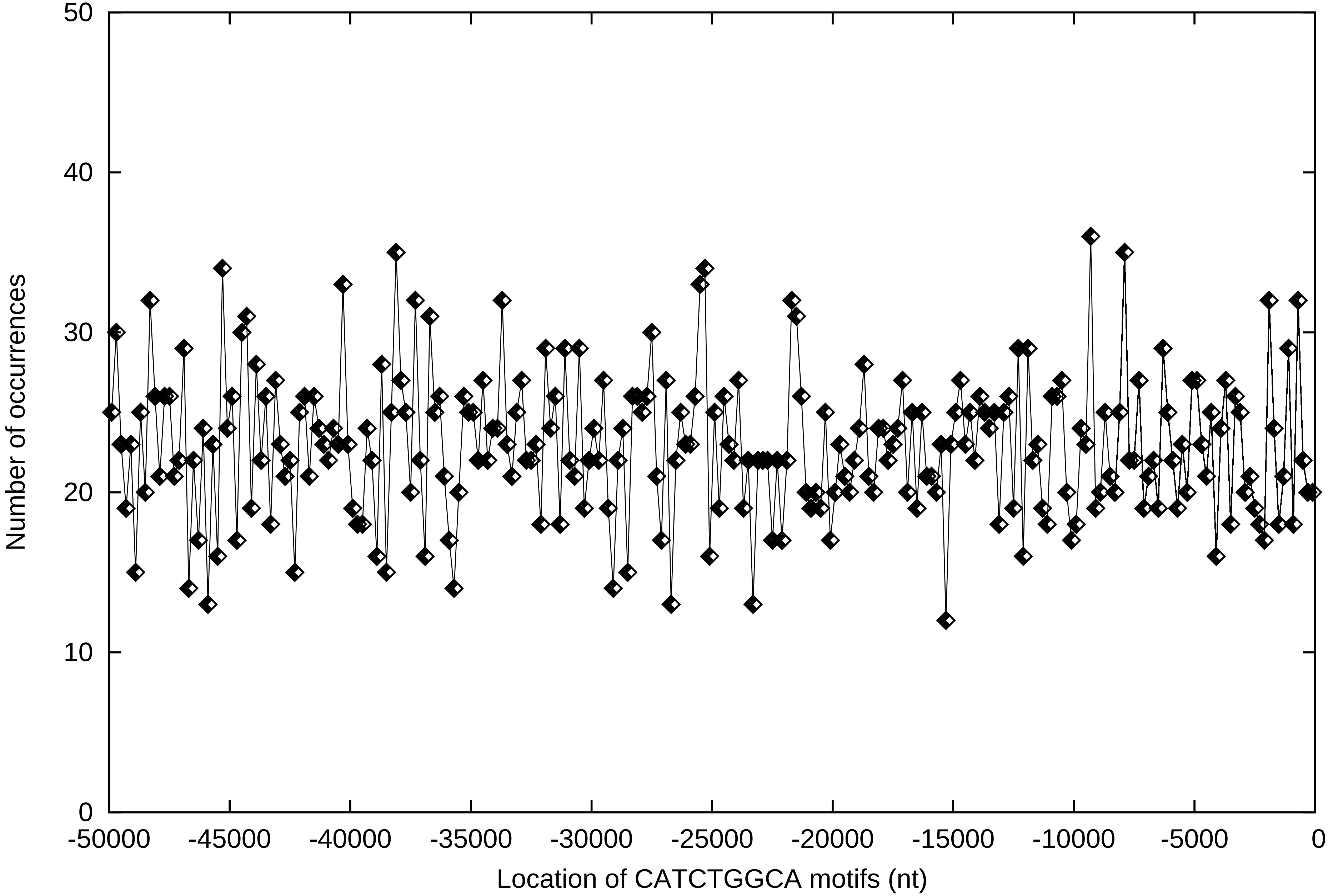

50
40
30
20
10
0
-50000
-45000
-40000
-35000
-30000
-25000
-20000
-15000
-10000
-5000
0
Number of occurrences
Location of CATCTGGCA motifs (nt)

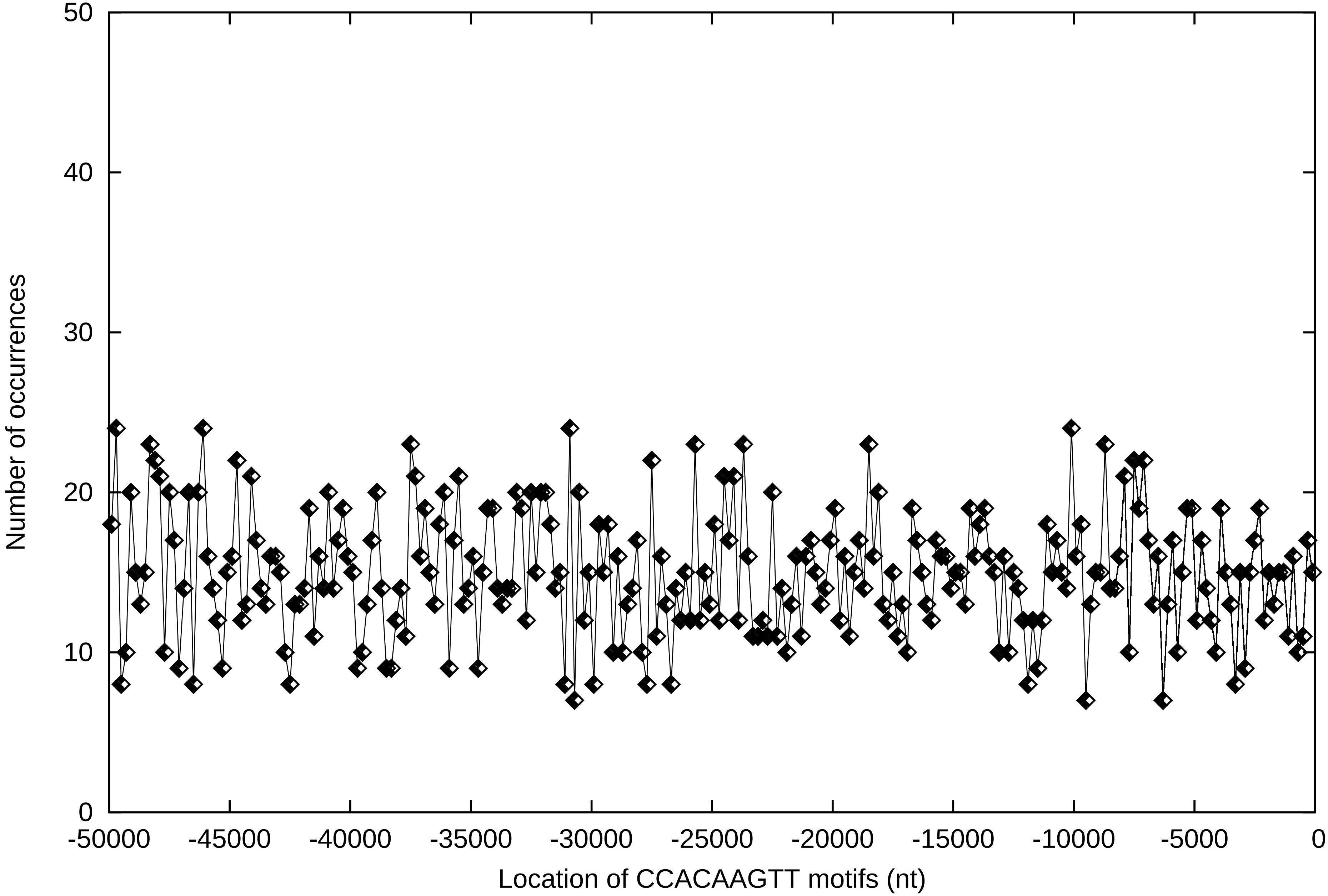

Number of occurrences
50
40
30
20
10
0
-50000
-45000
-40000
-35000
-30000
-25000
-20000
-15000
-10000
-5000
0
Location of CCACAAGTT motifs (nt)

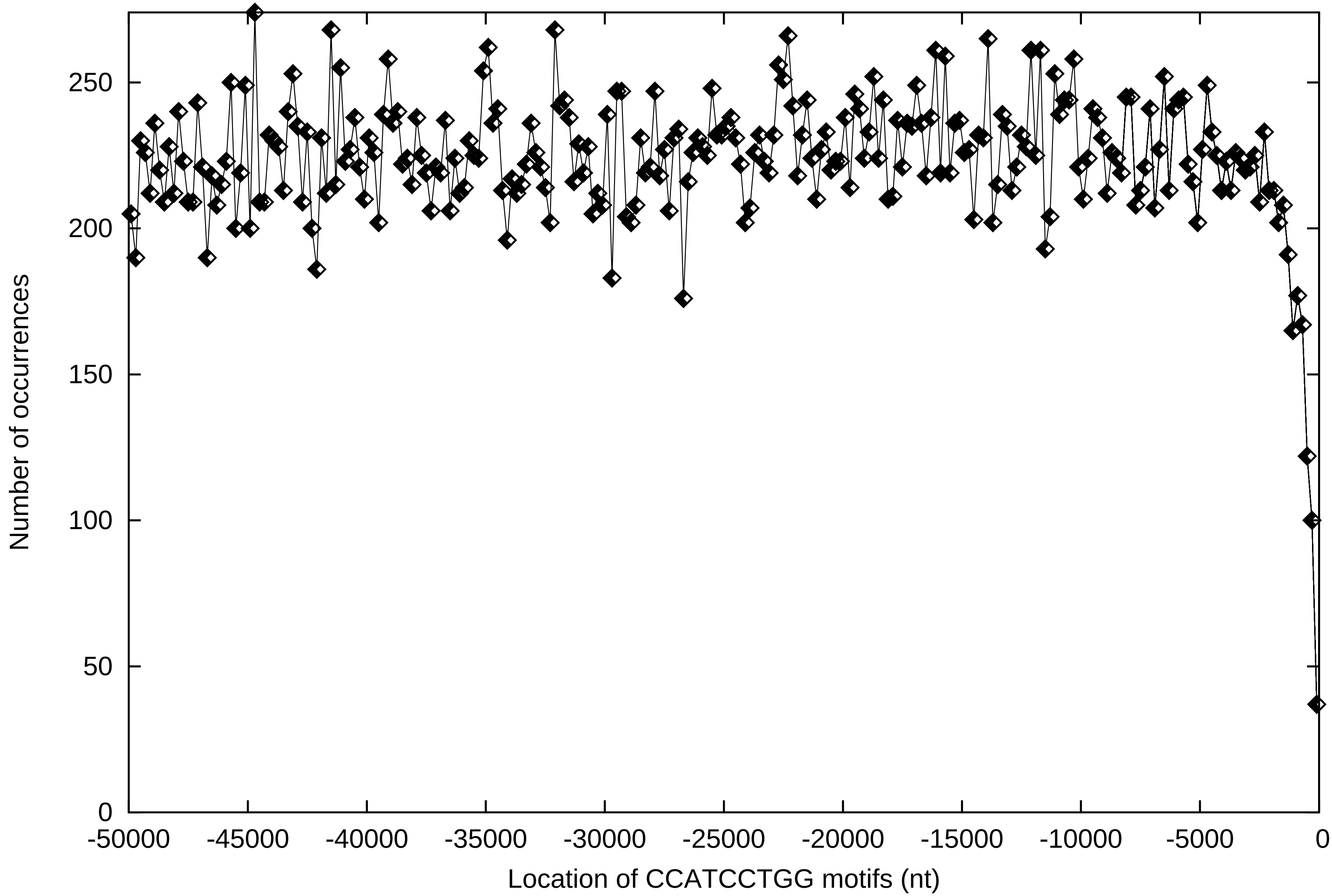

Number of occurrences
Location of CCATCCTGG motifs (nt)
0
50
100
150
200
250
-50000
-45000
-40000
-35000
-30000
-25000
-20000
-15000
-10000
-5000
0

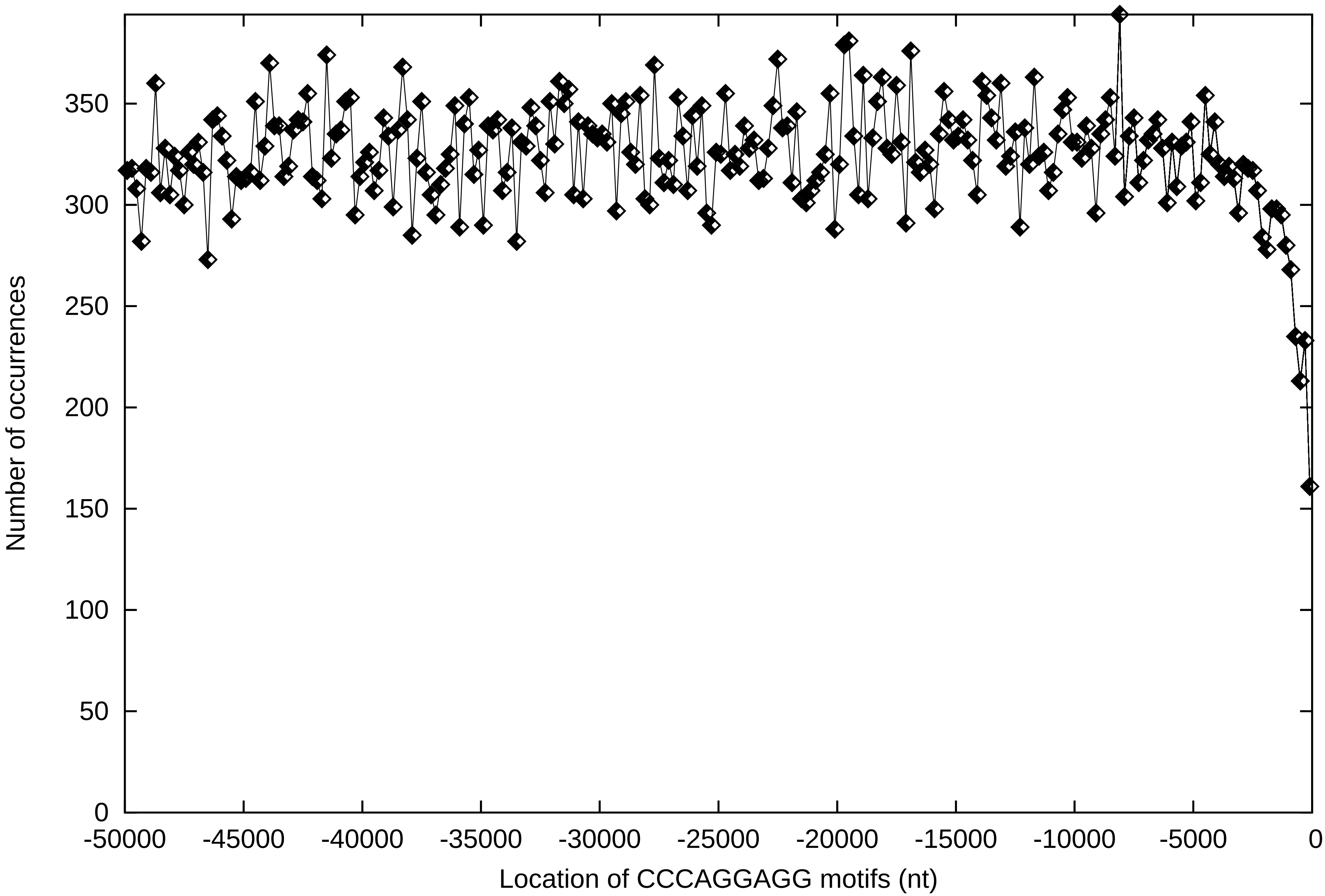

Number of occurrences
0
50
100
150
200
250
300
350
-50000
-45000
-40000
-35000
-30000
-25000
-20000
-15000
-10000
-5000
0
Location of CCCAGGAGG motifs (nt)

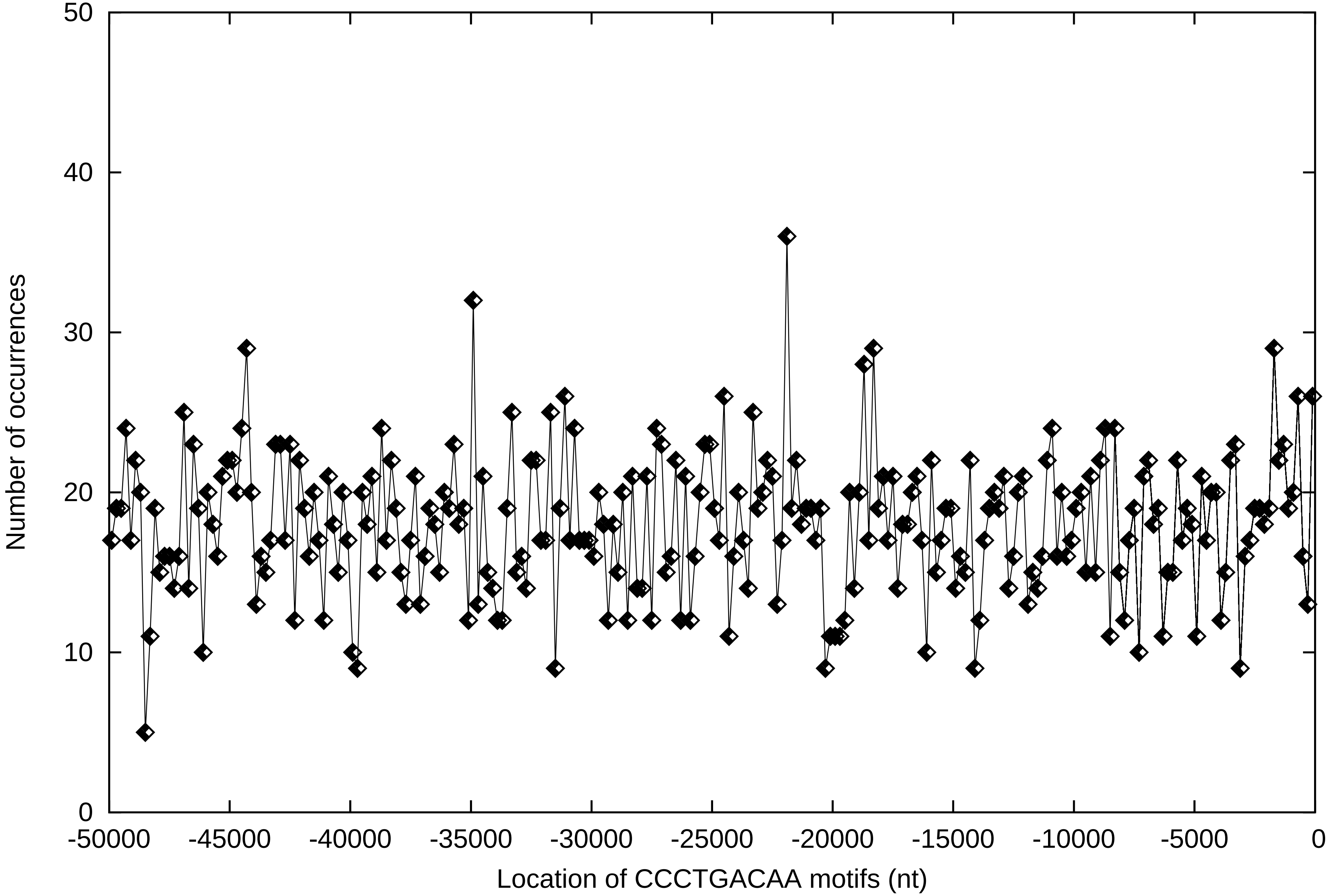

50
40
30
20
10
0
-50000
-45000
-40000
-35000
-30000
-25000
-20000
-15000
-10000
-5000
0
Number of occurrences
Location of CCCTGACAA motifs (nt)

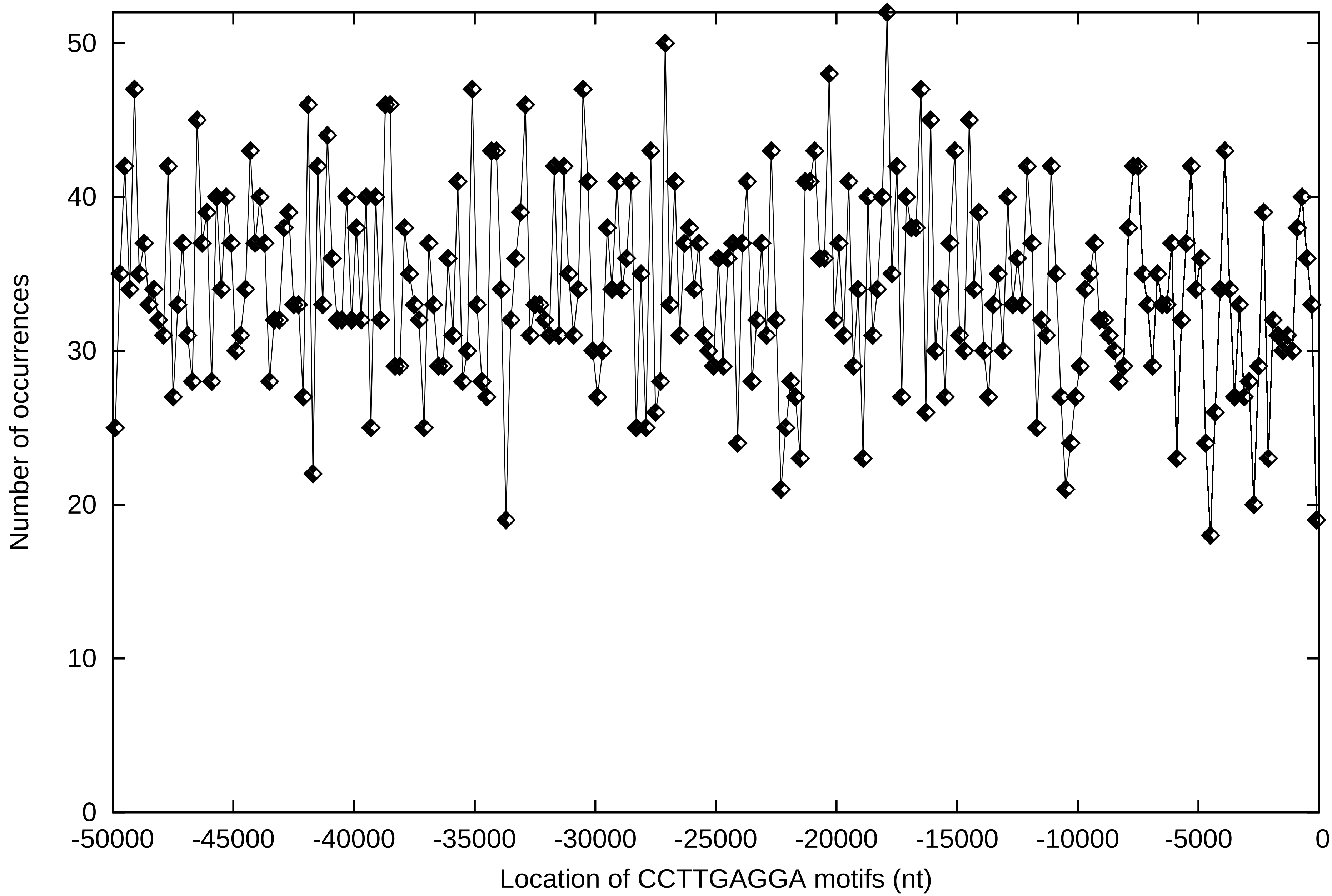
Number of occurrences
Location of CCTTGAGGA motifs (nt)
0
10
20
30
40
50
-50000
-45000
-40000
-35000
-30000
-25000
-20000
-15000
-10000
-5000
0

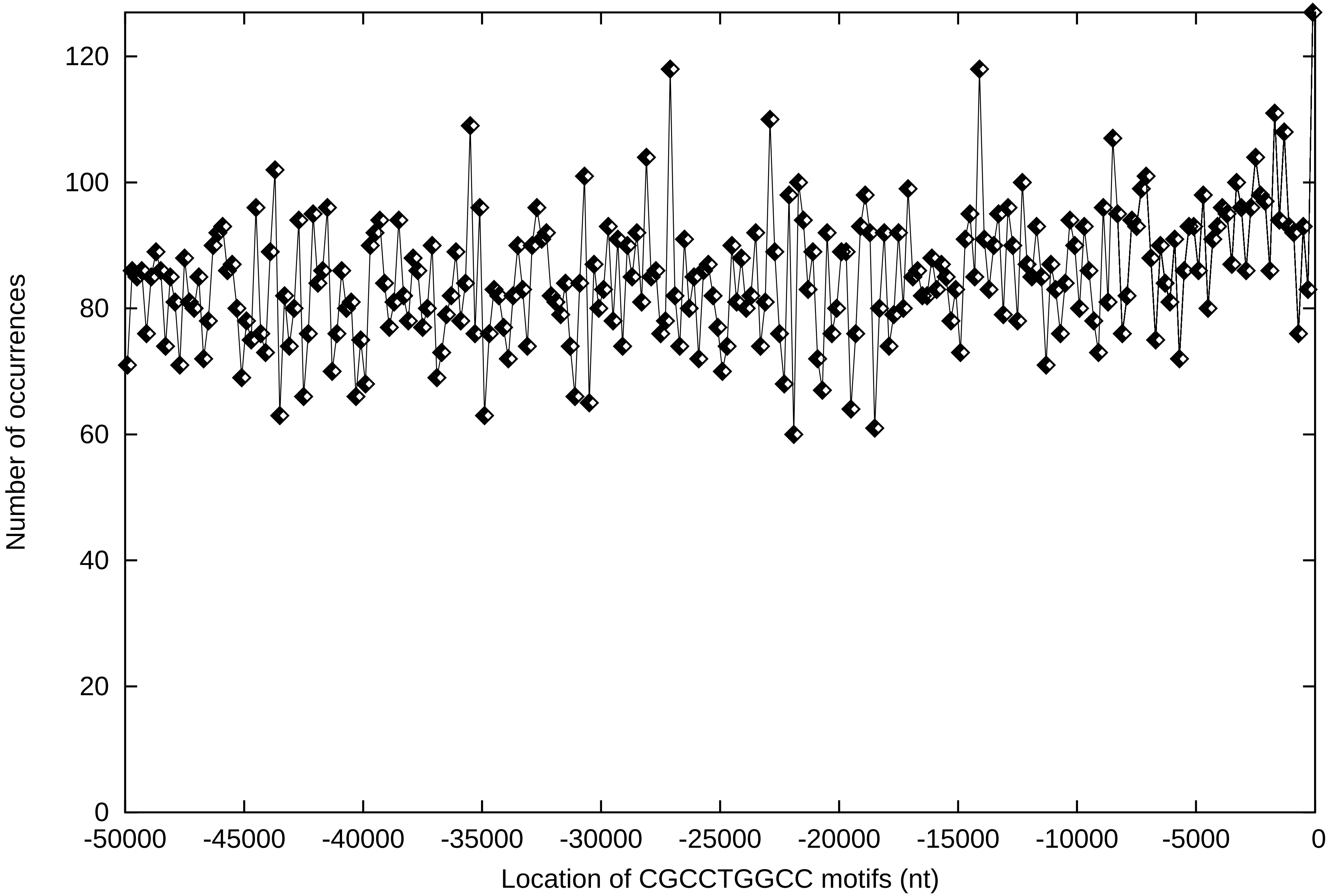

Number of occurrences
Location of CGCCTGGCC motifs (nt)
0
20
40
60
80
100
120
-50000
-45000
-40000
-35000
-30000
-25000
-20000
-15000
-10000
-5000
0

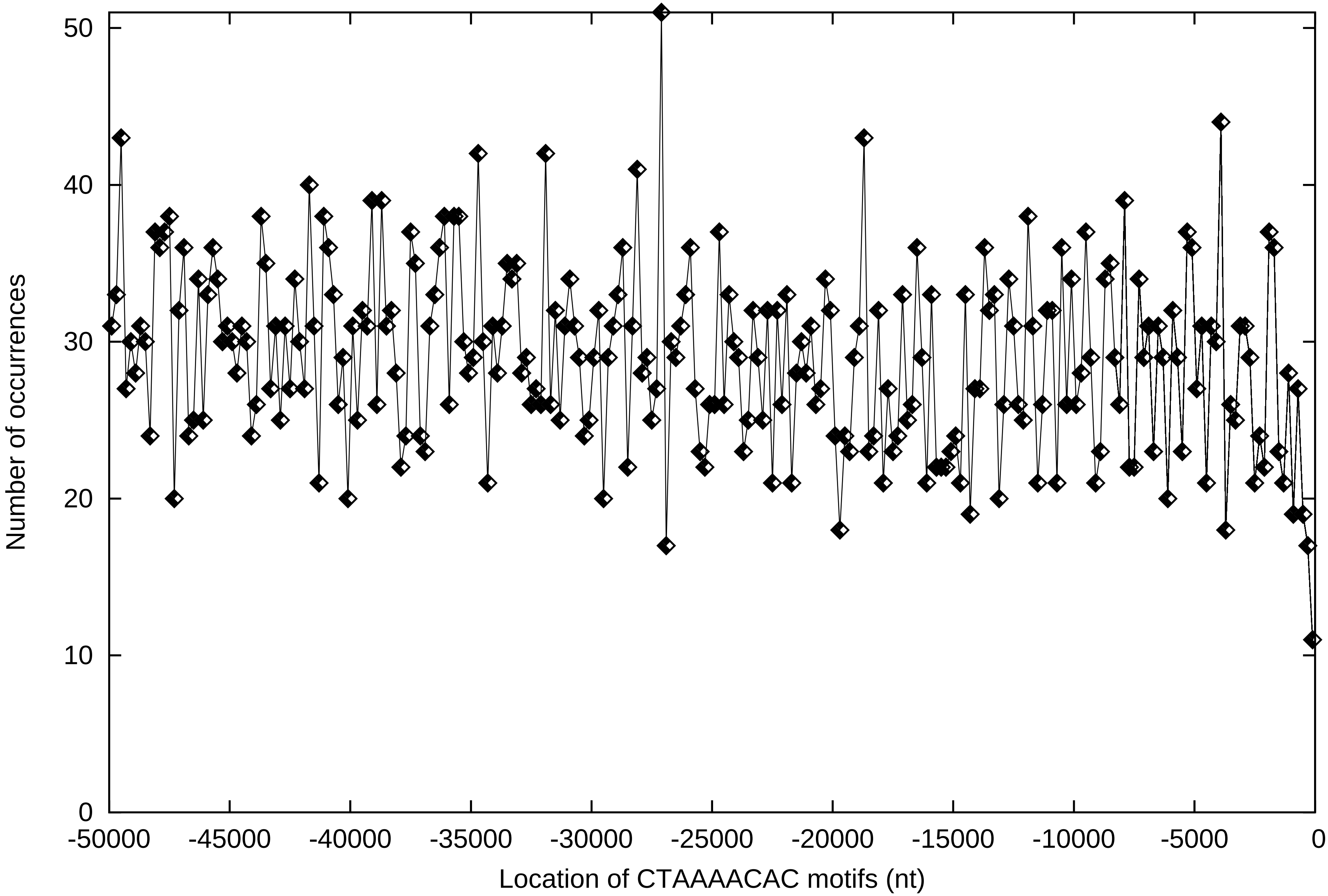

Number of occurrences
Location of CTAAAACAC motifs (nt)
0
10
20
30
40
50
-50000
-45000
-40000
-35000
-30000
-25000
-20000
-15000
-10000
-5000
0

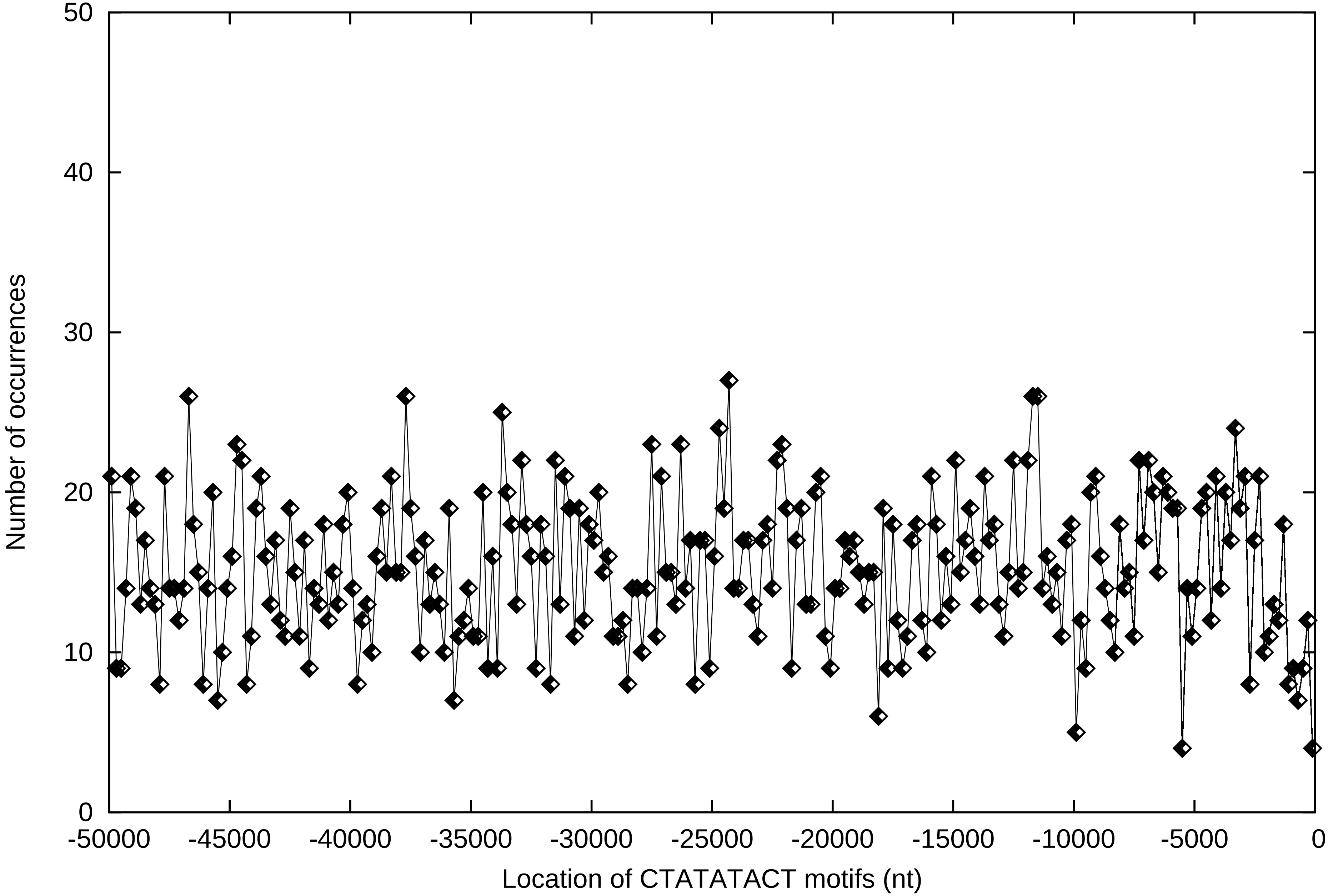

50
40
30
20
10
0
Number of occurrences
-50000
-45000
-40000
-35000
-30000
-25000
-20000
-15000
-10000
-5000
0
Location of CTATATACT motifs (nt)

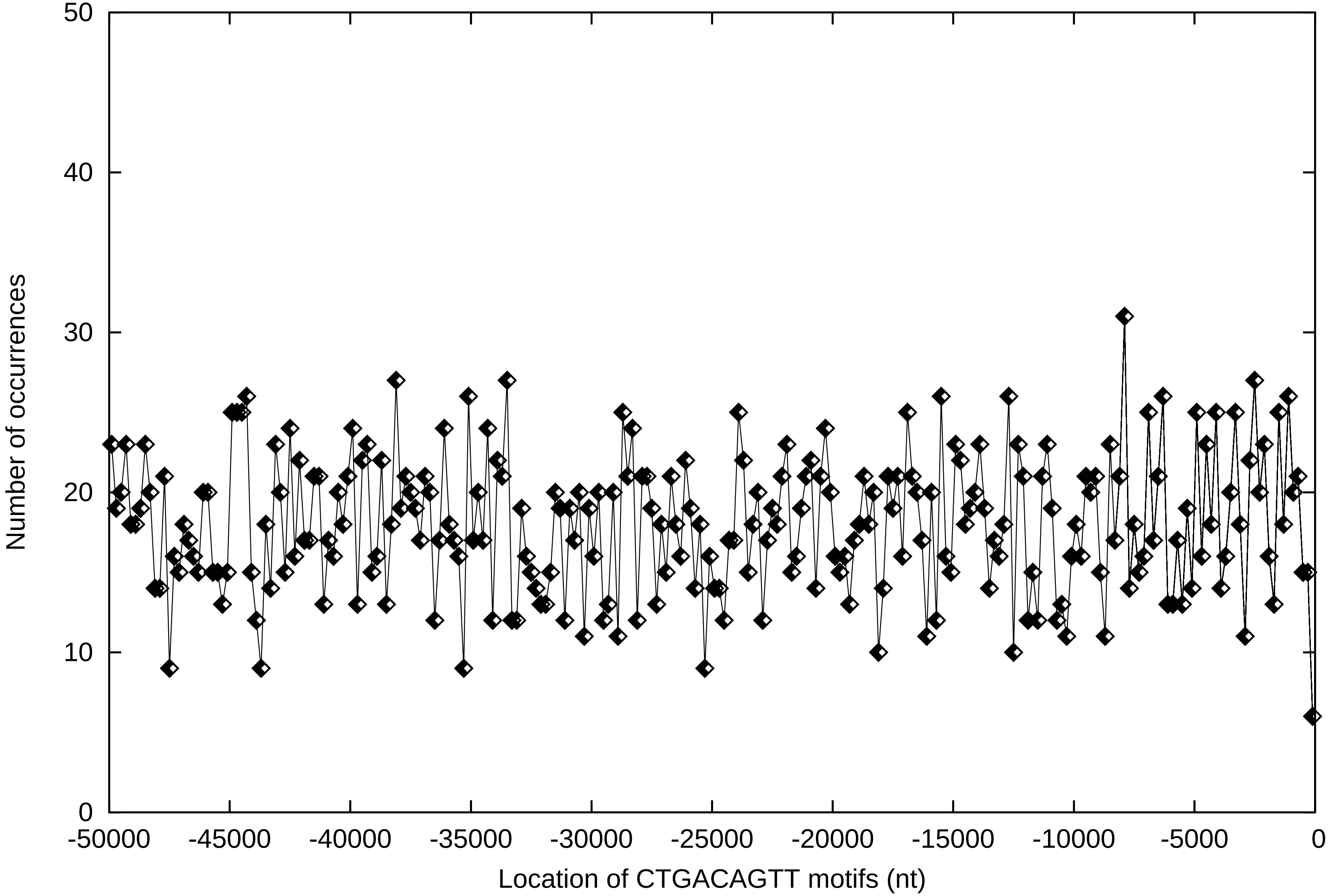

50
40
30
20
10
0
-50000
-45000
-40000
-35000
-30000
-25000
-20000
-15000
-10000
-5000
0
Number of occurrences
Location of CTGACAGTT motifs (nt)

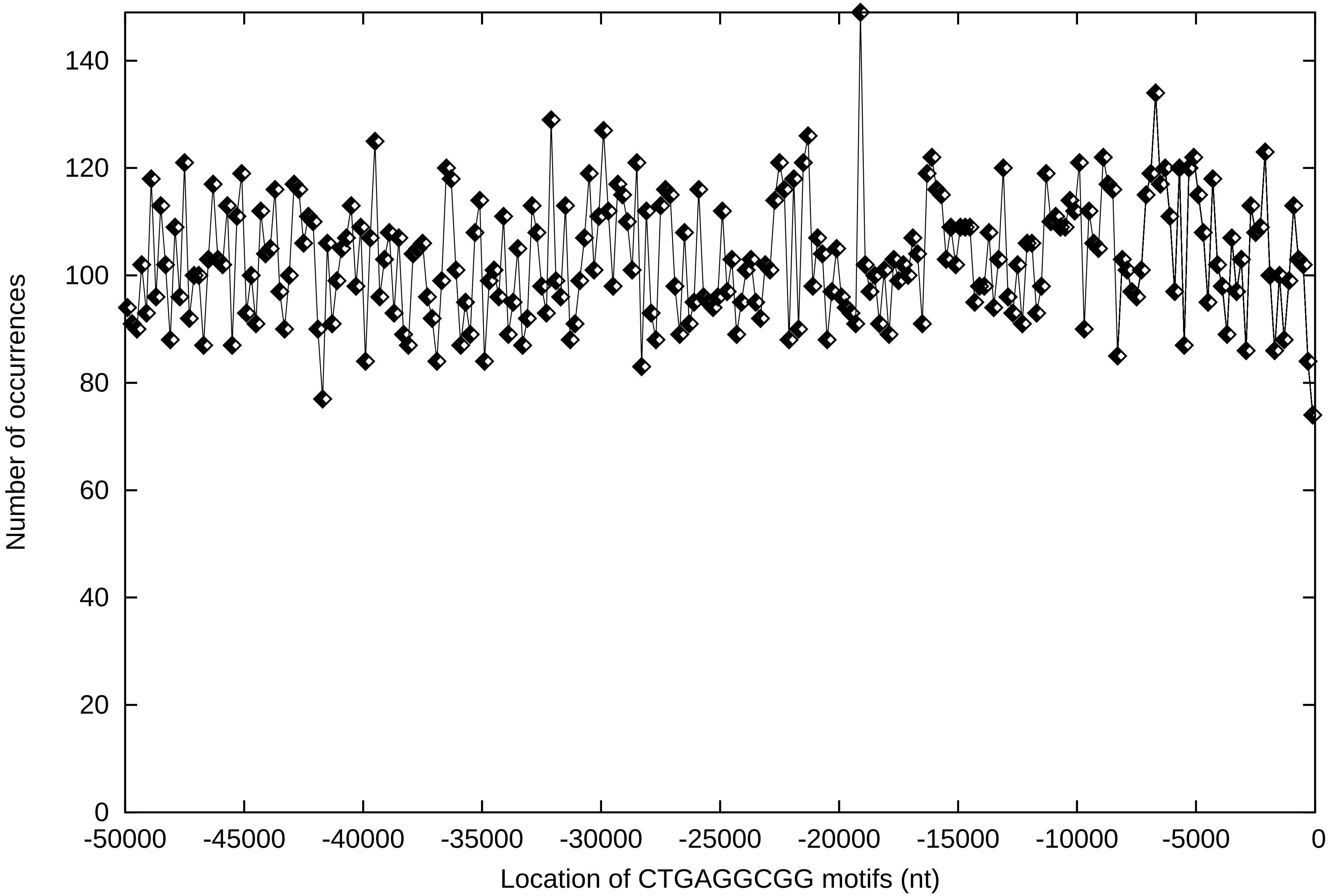

Number of occurrences
0
20
40
60
80
100
120
140
-50000
-45000
-40000
-35000
-30000
-25000
-20000
-15000
-10000
-5000
0
Location of CTGAGGCGG motifs (nt)

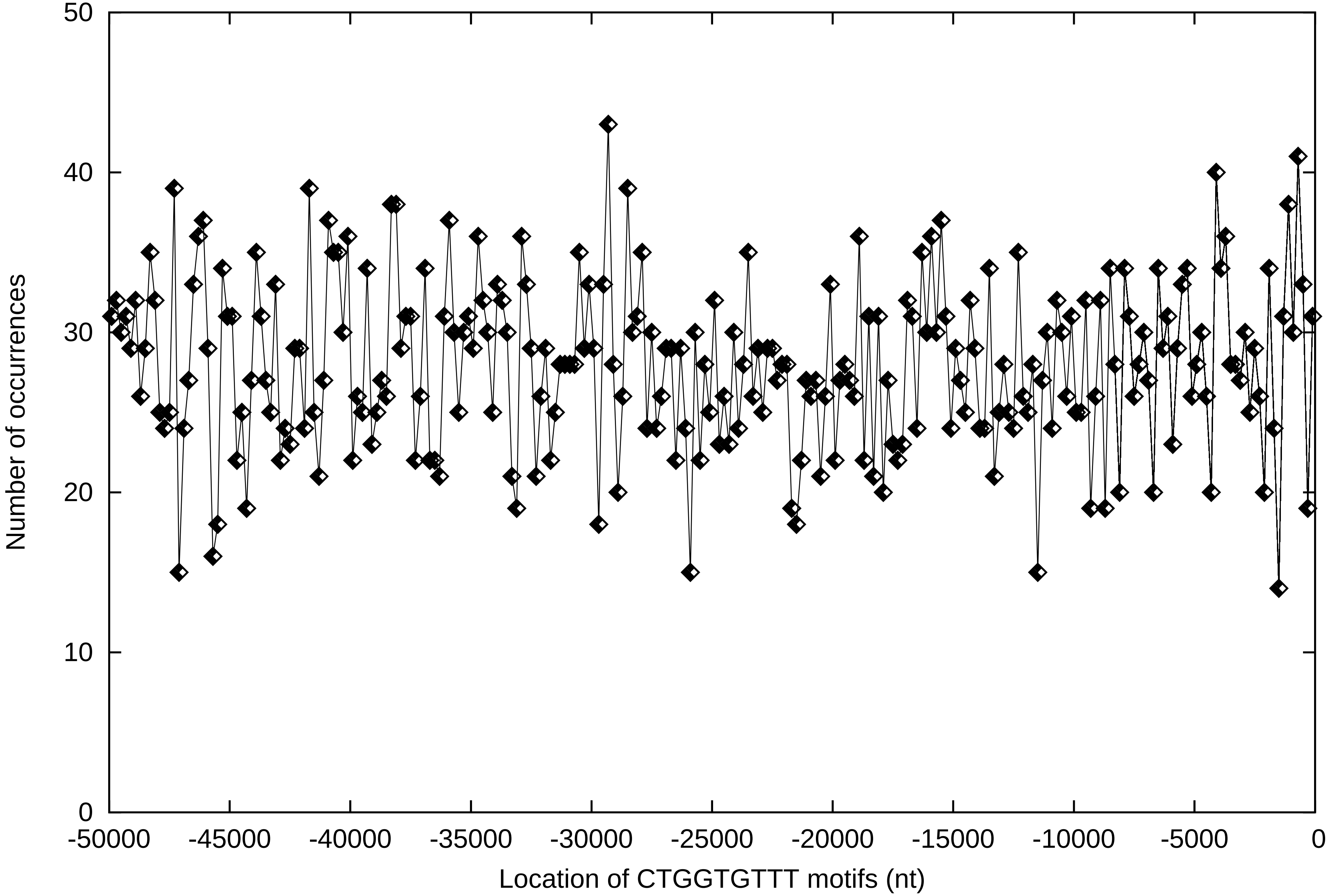

50
40
30
20
10
0
-50000
-45000
-40000
-35000
-30000
-25000
-20000
-15000
-10000
-5000
0
Number of occurrences
Location of CTGGTGTTT motifs (nt)

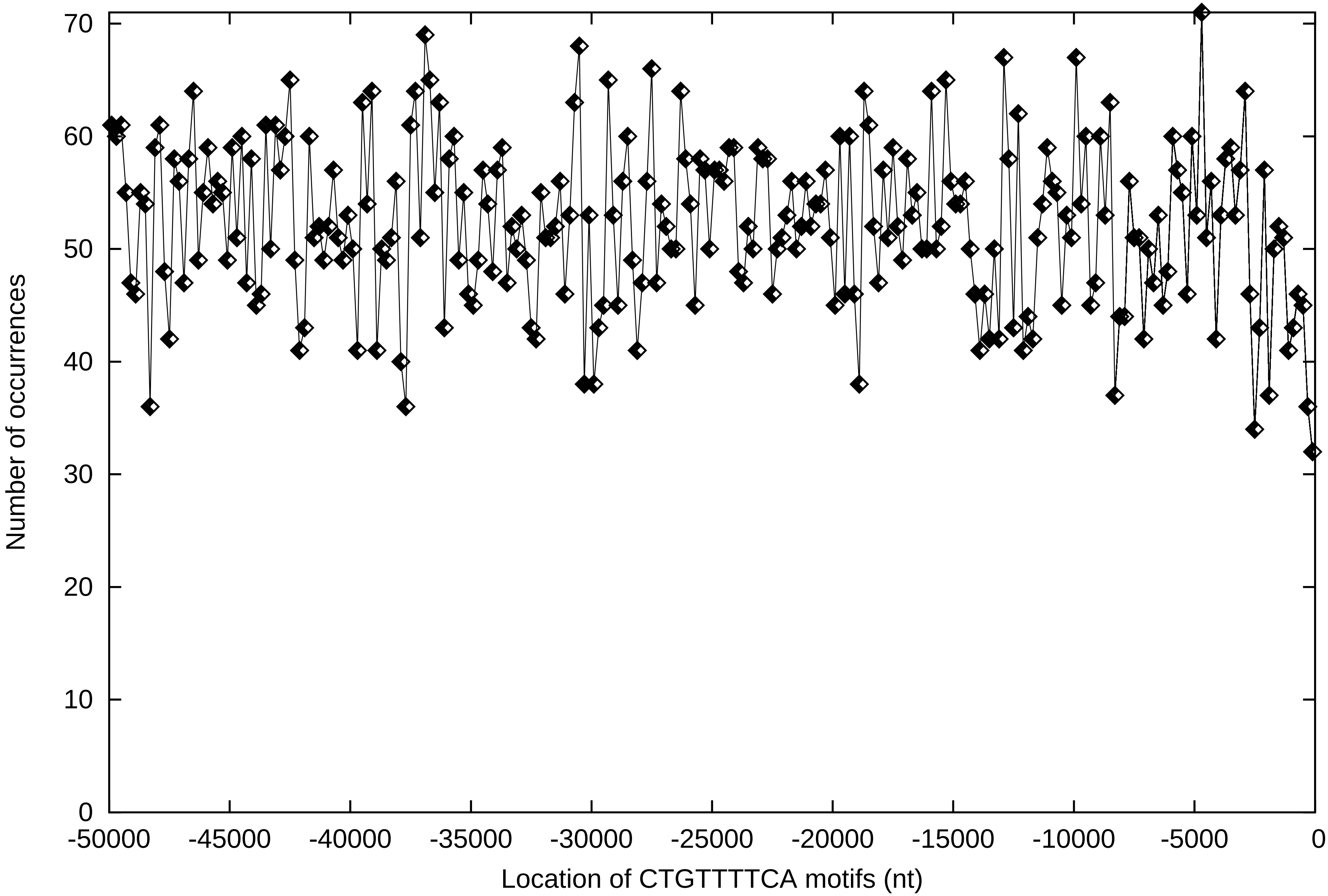

Number of occurrences
Location of CTGTTTTCA motifs (nt)
0
10
20
30
40
50
60
70
-50000
-45000
-40000
-35000
-30000
-25000
-20000
-15000
-10000
-5000
0

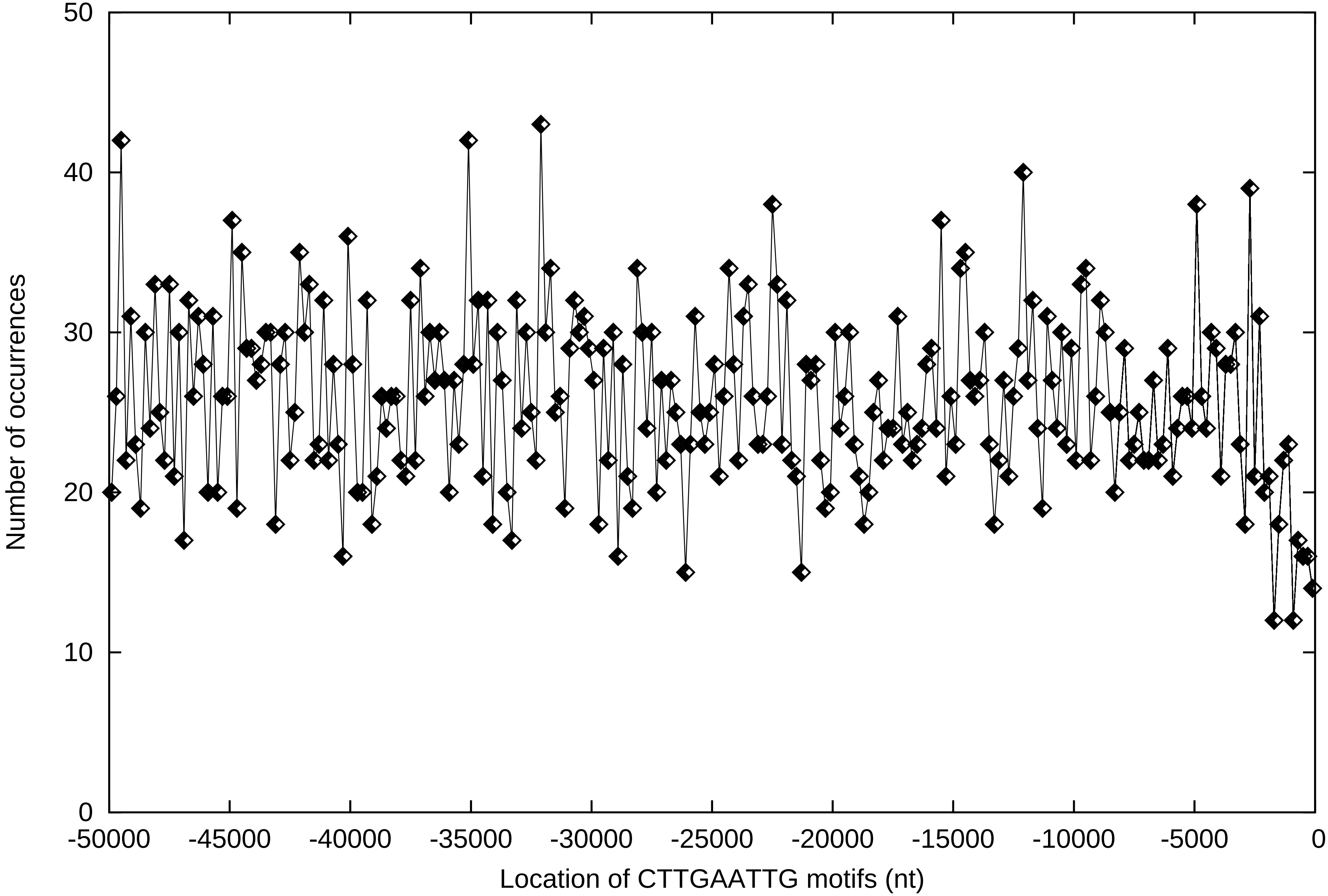

Number of occurrences
Location of CTTGAATTG motifs (nt)
0
10
20
30
40
50
-50000
-45000
-40000
-35000
-30000
-25000
-20000
-15000
-10000
-5000
0

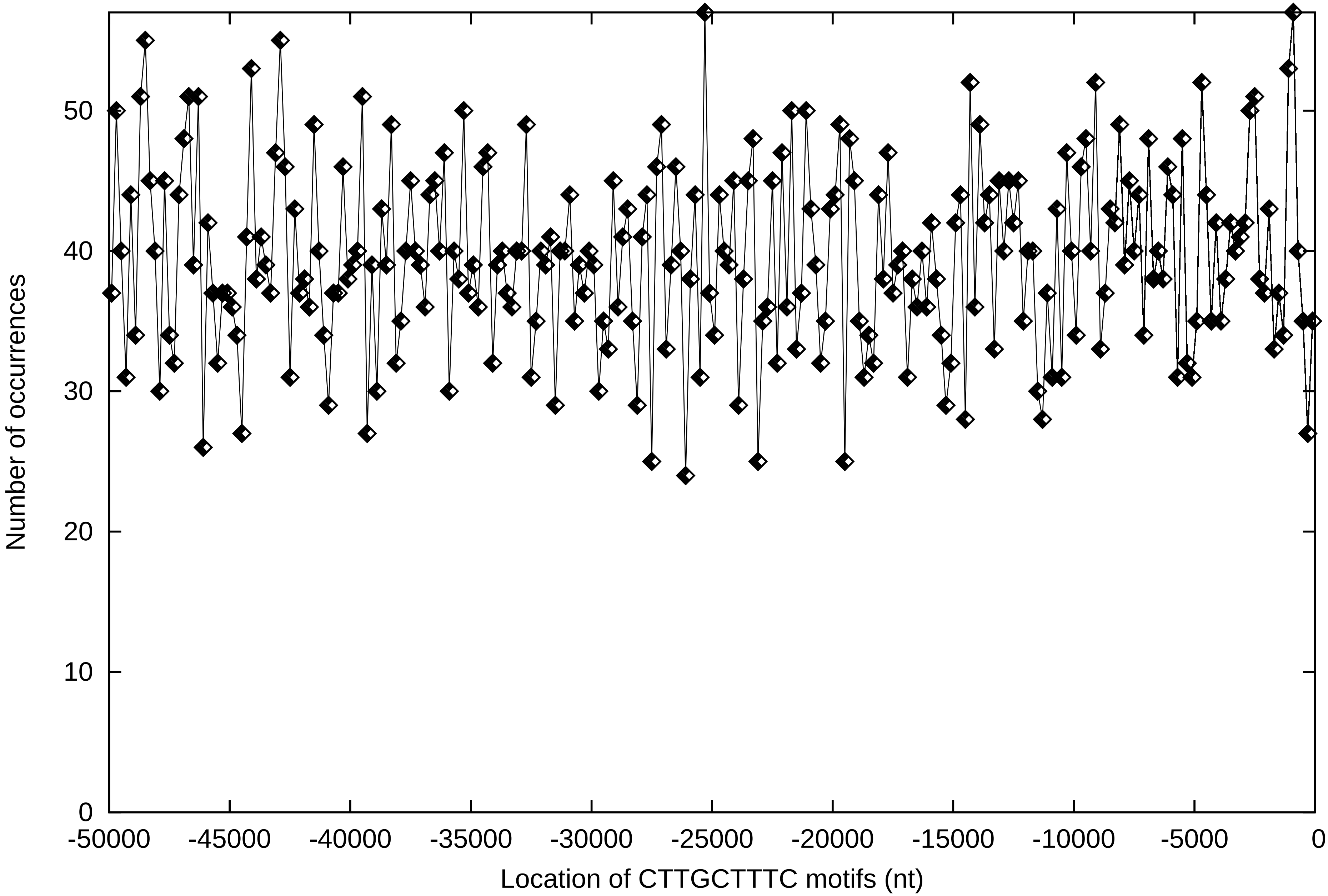

0
10
20
30
40
50
Number of occurrences
-50000
-45000
-40000
-35000
-30000
-25000
-20000
-15000
-10000
-5000
0
Location of CTTGCTTTC motifs (nt)

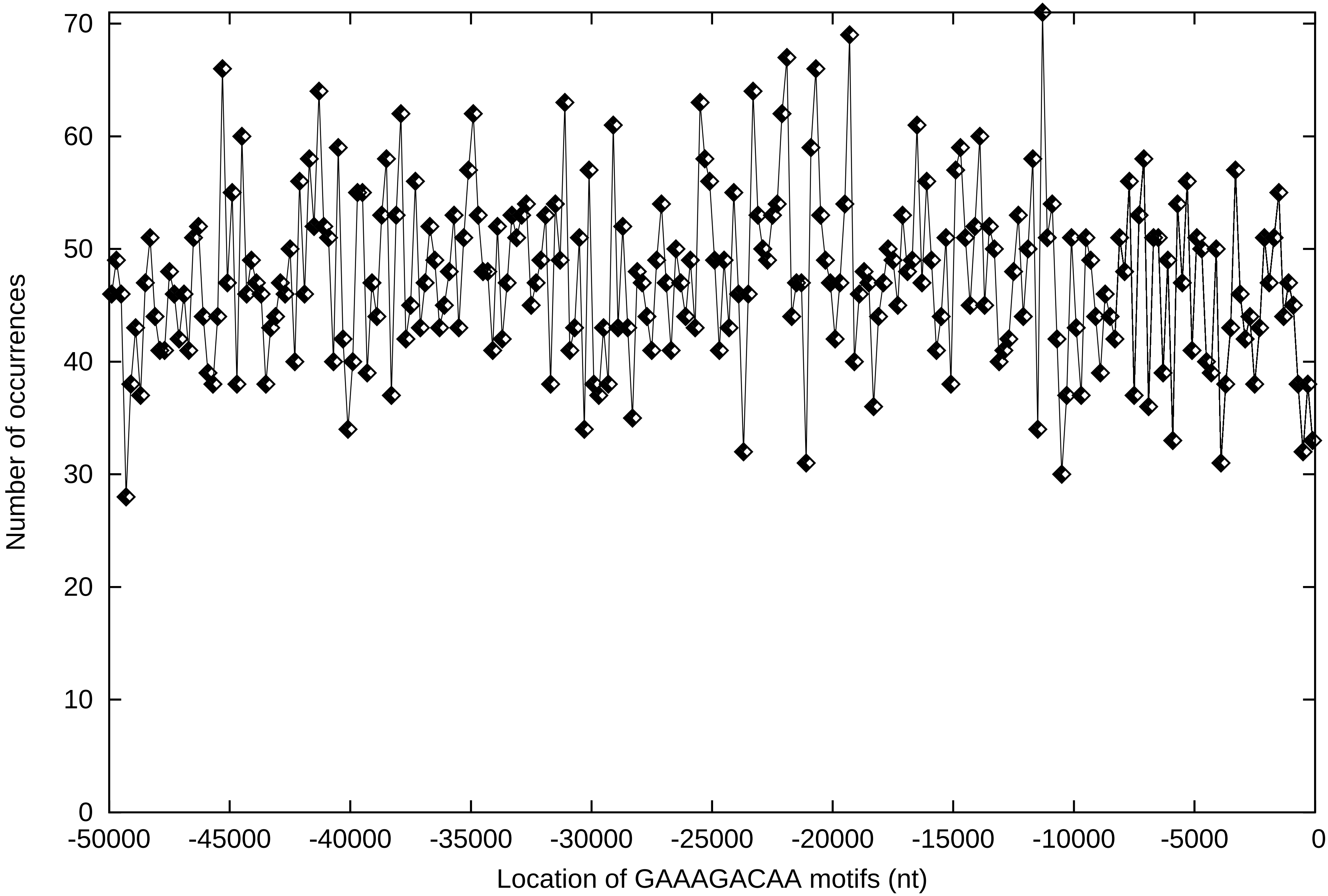
Number of occurrences
Location of GAAAGACAA motifs (nt)
0
10
20
30
40
50
60
70
-50000
-45000
-40000
-35000
-30000
-25000
-20000
-15000
-10000
-5000
0

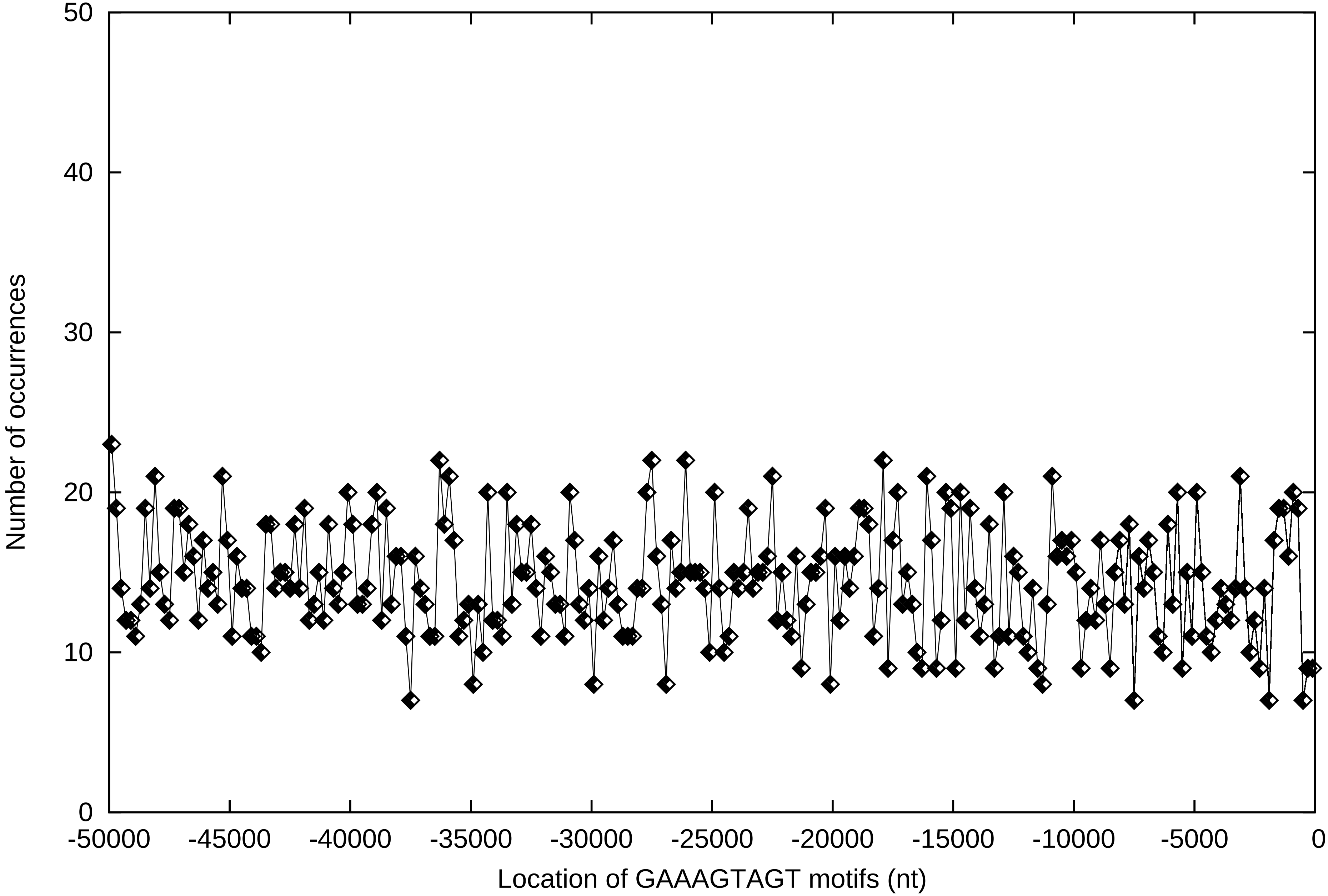

50
40
30
20
10
0
Number of occurrences
-50000
-45000
-40000
-35000
-30000
-25000
-20000
-15000
-10000
-5000
0
Location of GAAAGTAGT motifs (nt)

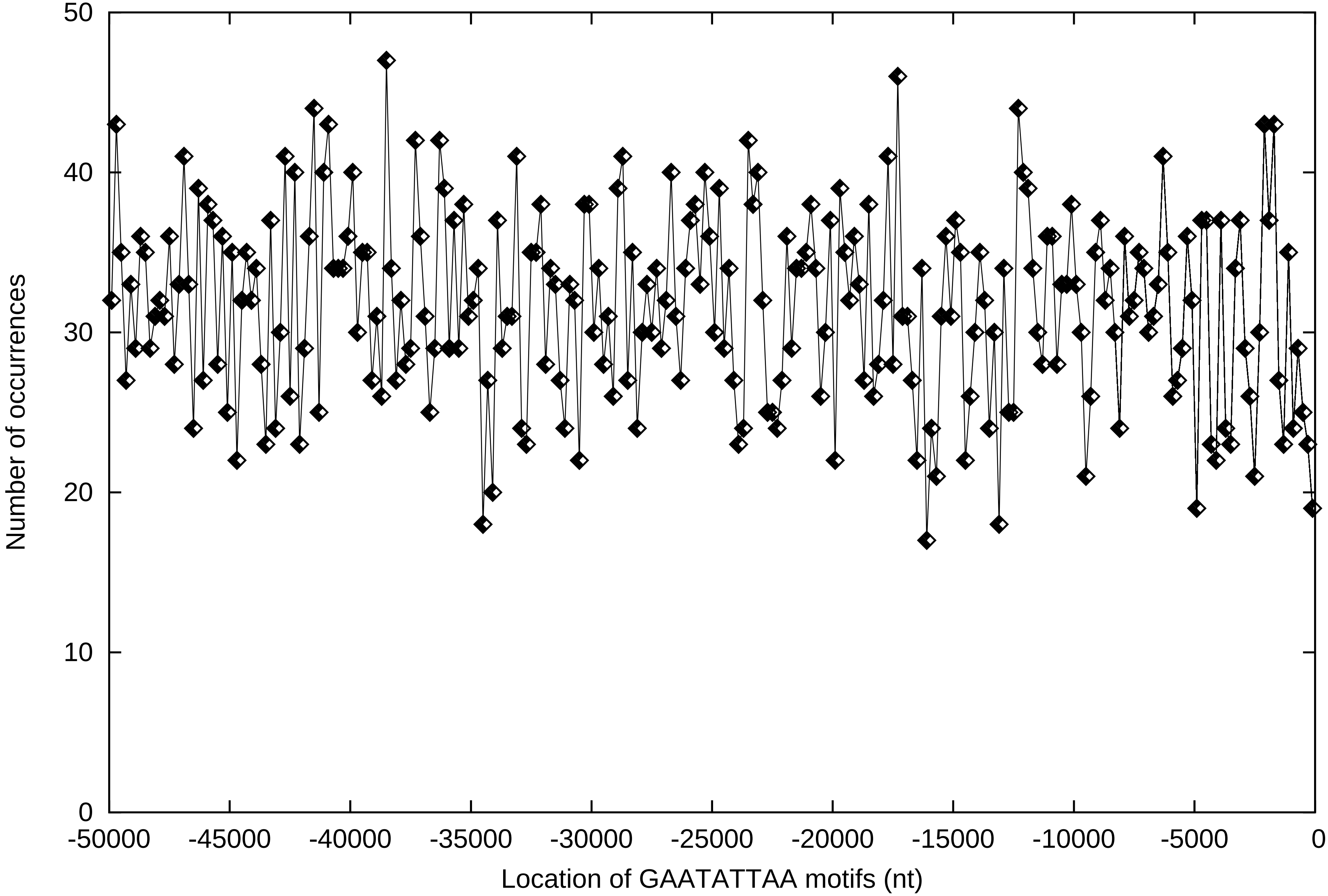

50
40
30
20
10
0
-50000
-45000
-40000
-35000
-30000
-25000
-20000
-15000
-10000
-5000
0
Number of occurrences
Location of GAATATTAA motifs (nt)

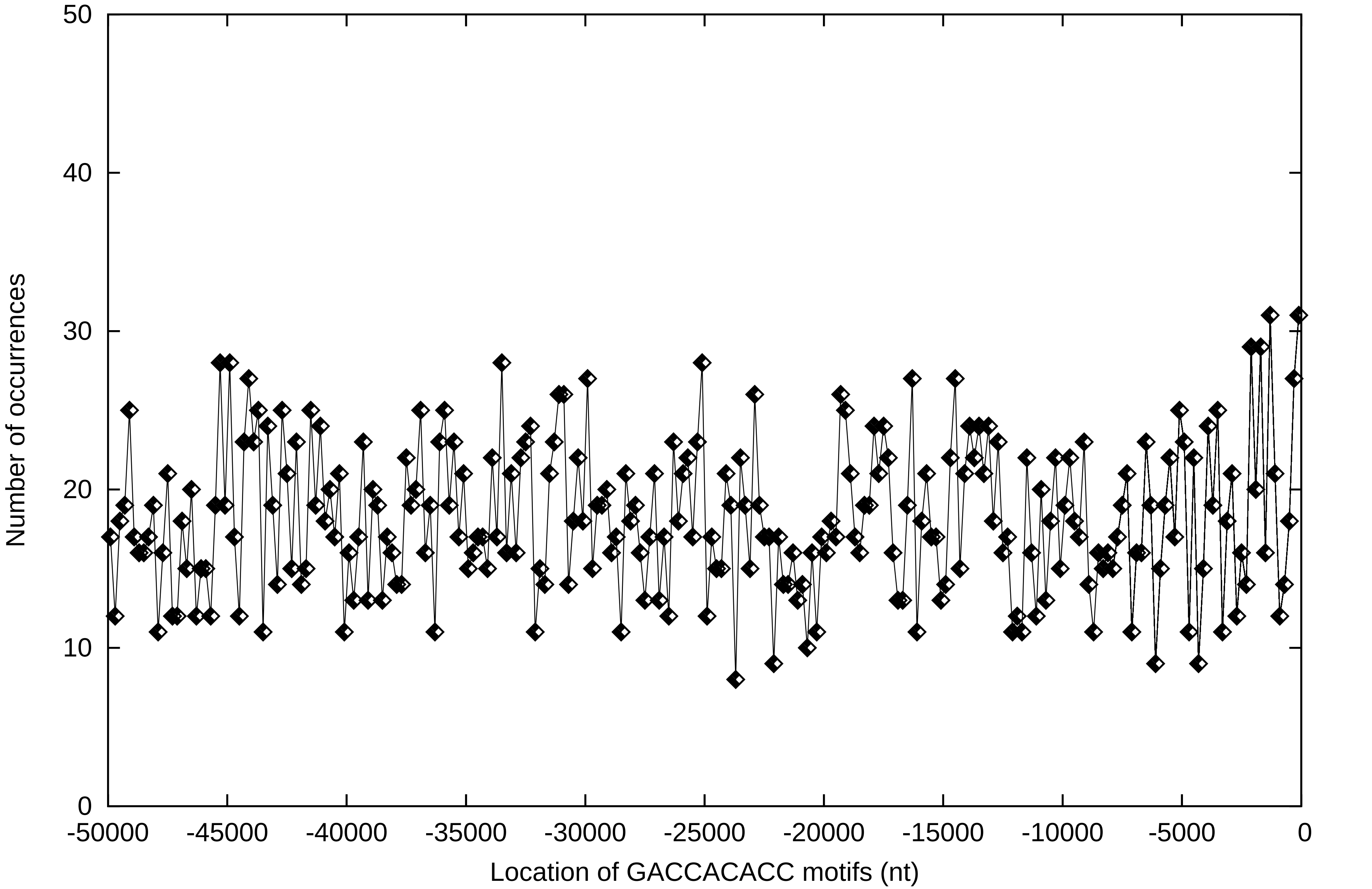

Number of occurrences
0
10
20
30
40
50
-50000
-45000
-40000
-35000
-30000
-25000
-20000
-15000
-10000
-5000
0
Location of GACCACACC motifs (nt)

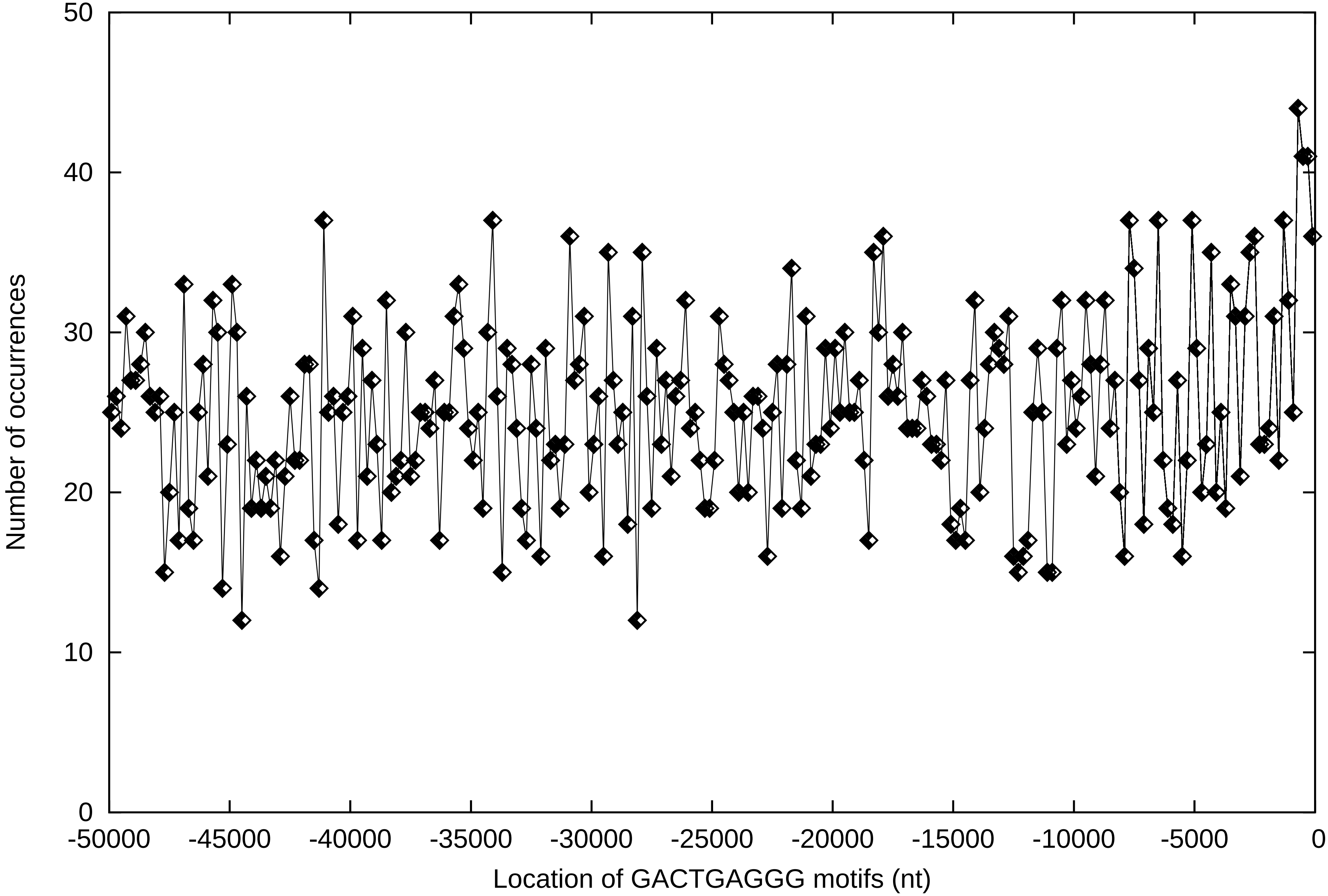

50
40
30
20
10
0
-50000
-45000
-40000
-35000
-30000
-25000
-20000
-15000
-10000
-5000
0
Number of occurrences
Location of GACTGAGGG motifs (nt)

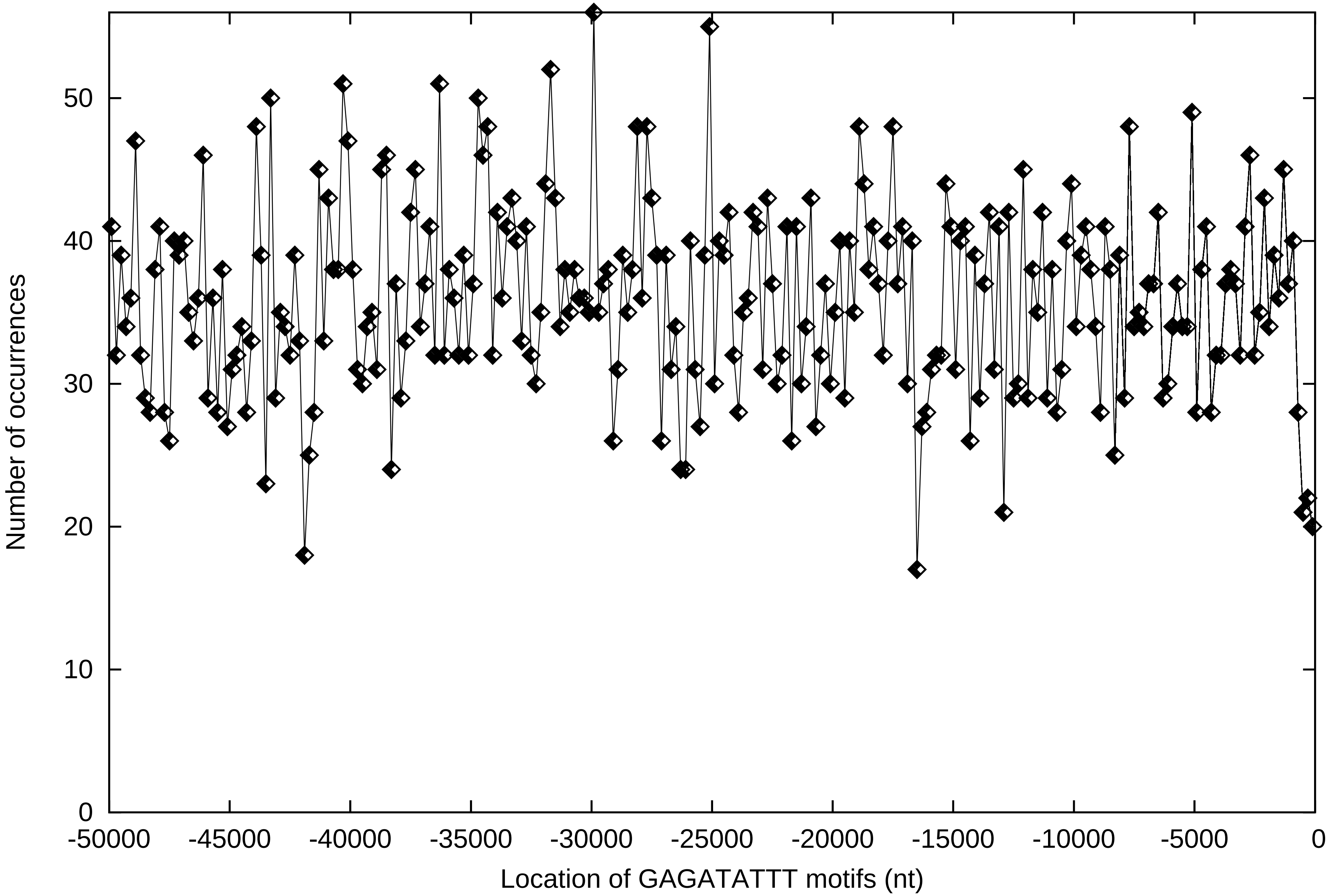

Number of occurrences
0
10
20
30
40
50
-50000
-45000
-40000
-35000
-30000
-25000
-20000
-15000
-10000
-5000
0
Location of GAGATATTT motifs (nt)

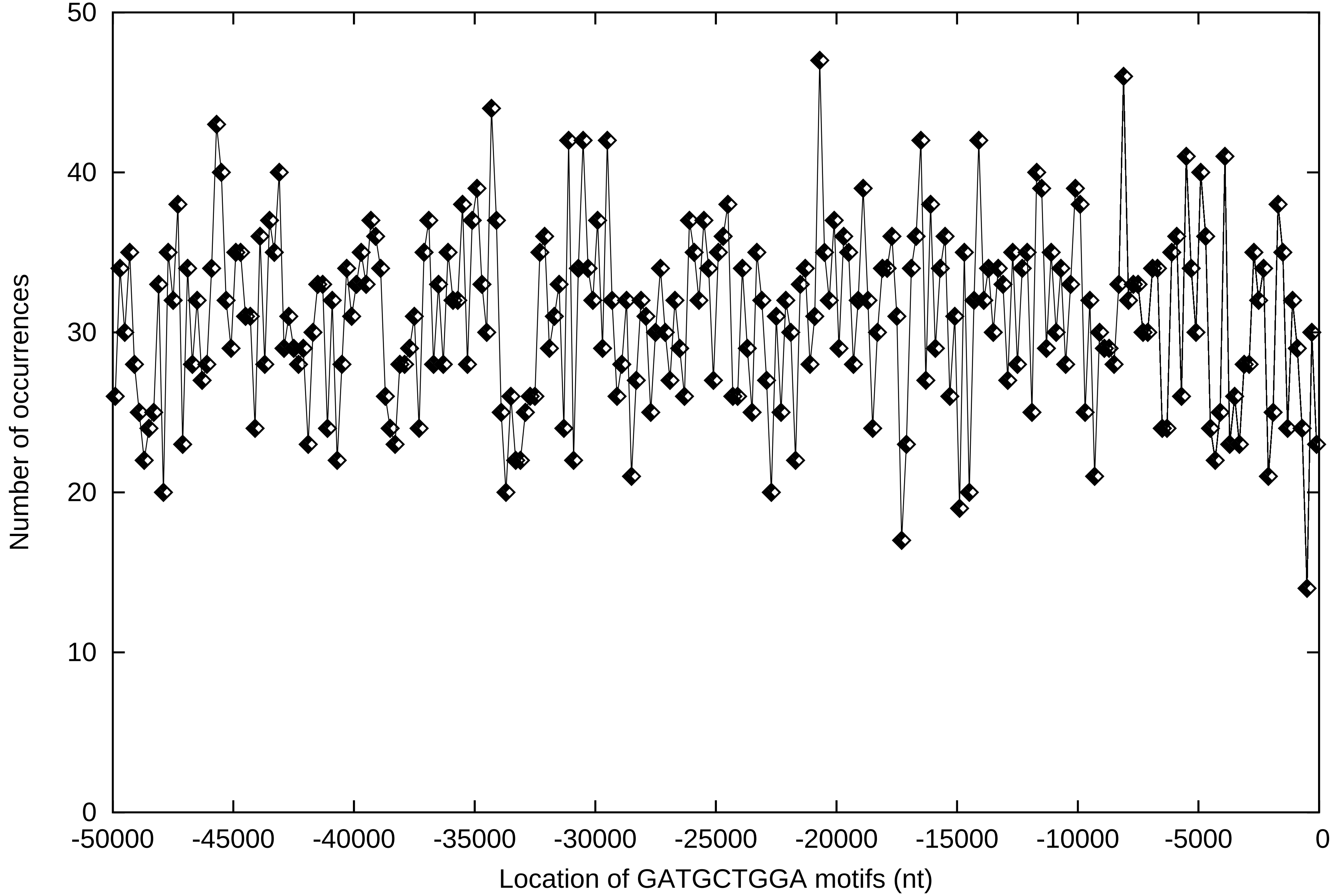

Number of occurrences
Location of GATGCTGGA motifs (nt)
0
10
20
30
40
50
-50000
-45000
-40000
-35000
-30000
-25000
-20000
-15000
-10000
-5000
0

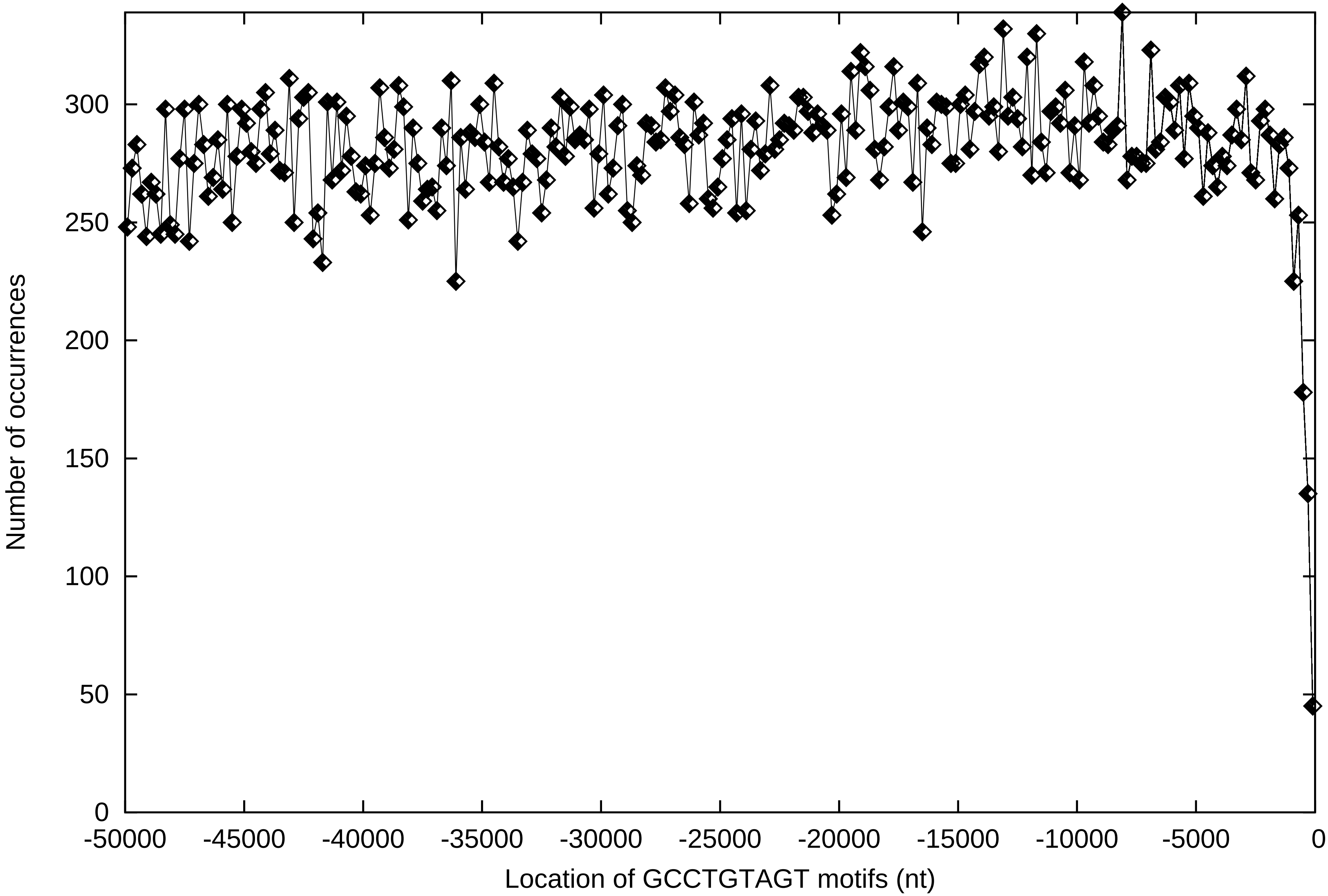

Number of occurrences
0
50
100
150
200
250
300
-50000
-45000
-40000
-35000
-30000
-25000
-20000
-15000
-10000
-5000
0
Location of GCCTGTAGT motifs (nt)

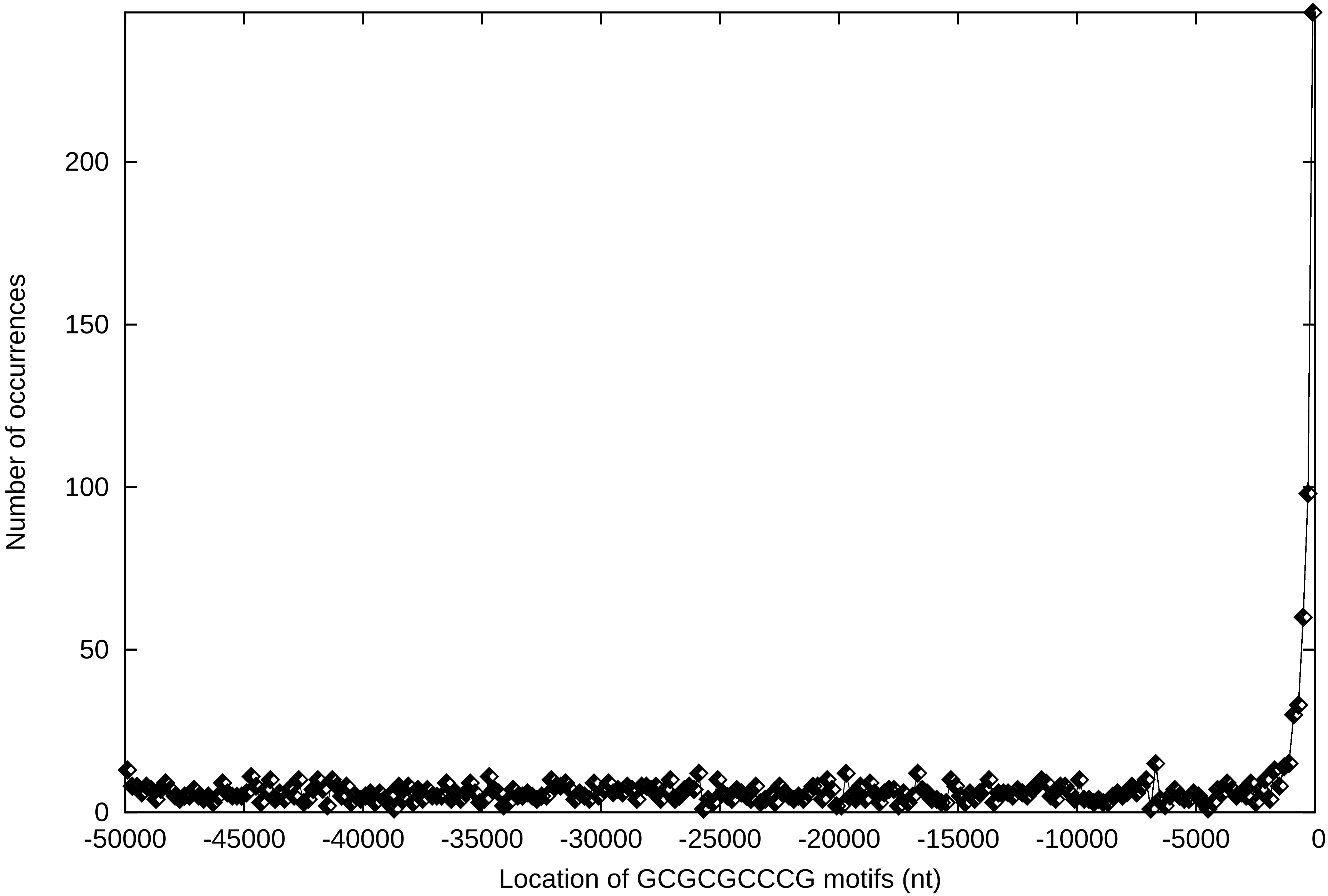

Number of occurrences
200
150
100
50
0
-50000
-45000
-40000
-35000
-30000
-25000
-20000
-15000
-10000
-5000
0
Location of GCGCGCCCG motifs (nt)

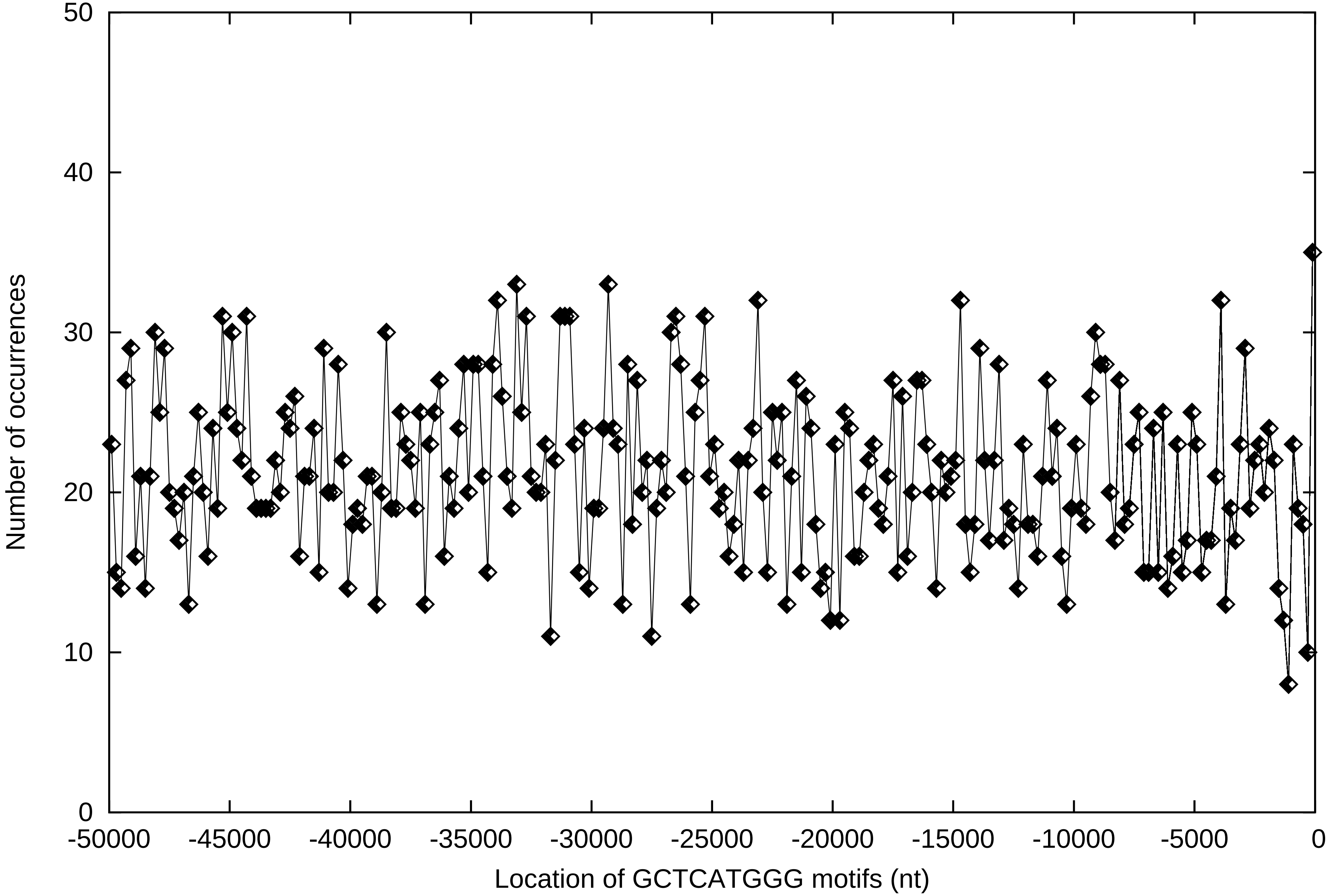

50
40
30
20
10
0
-50000
-45000
-40000
-35000
-30000
-25000
-20000
-15000
-10000
-5000
0
Number of occurrences
Location of GCTCATGGG motifs (nt)

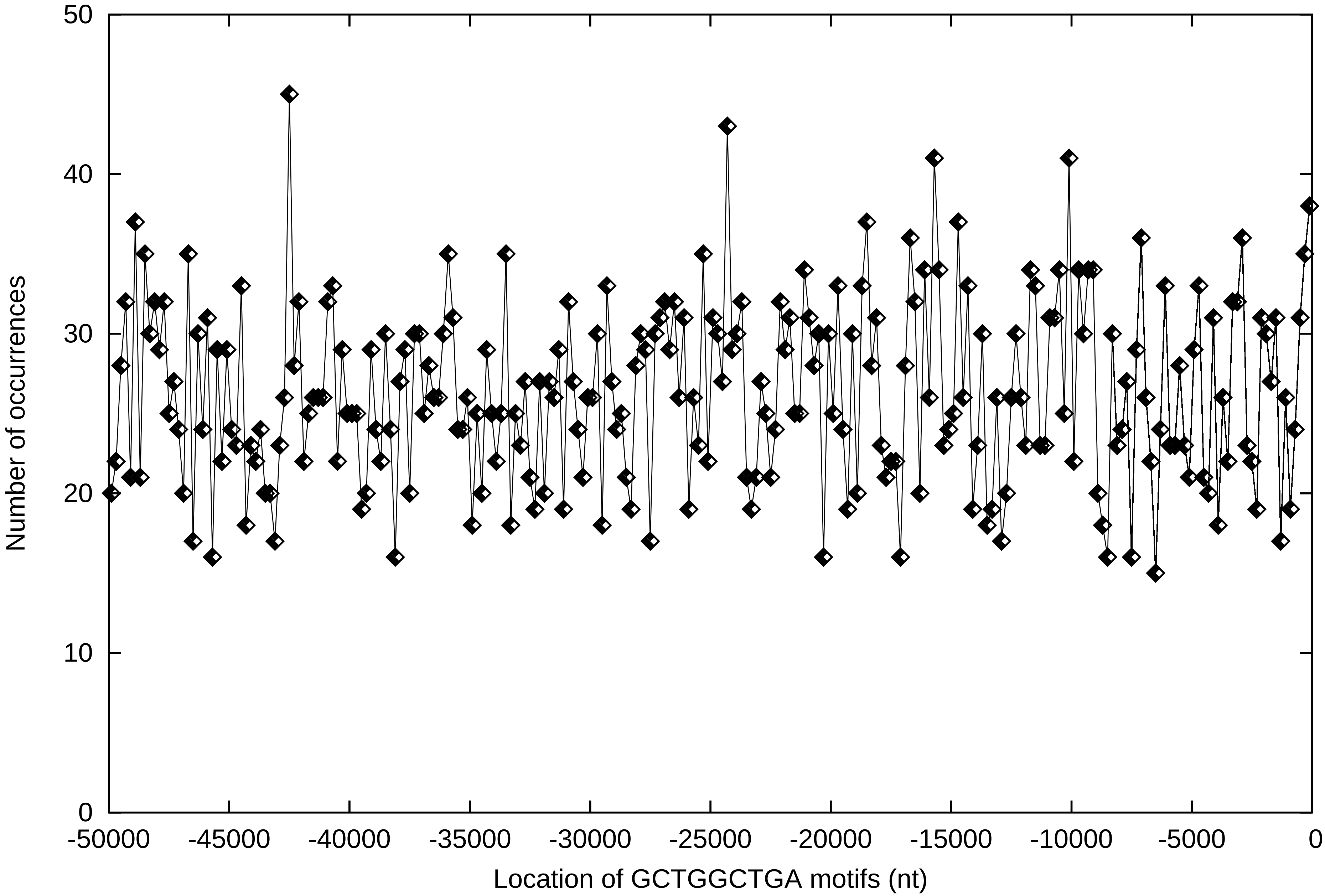

50
40
30
20
10
0
-50000
-45000
-40000
-35000
-30000
-25000
-20000
-15000
-10000
-5000
0
Number of occurrences
Location of GCTGGCTGA motifs (nt)

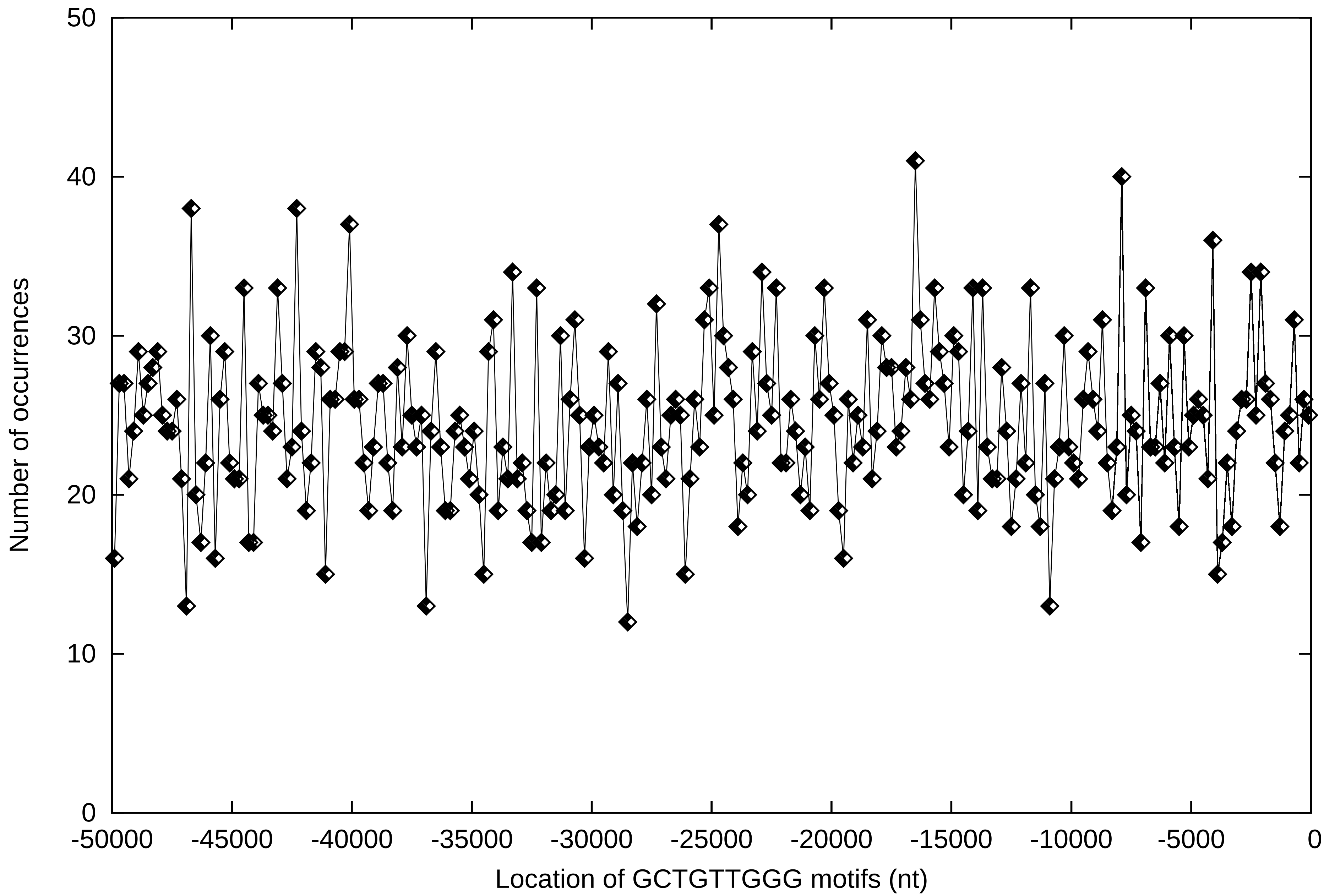

50
40
30
20
10
0
Number of occurrences
-50000
-45000
-40000
-35000
-30000
-25000
-20000
-15000
-10000
-5000
0
Location of GCTGTTGGG motifs (nt)

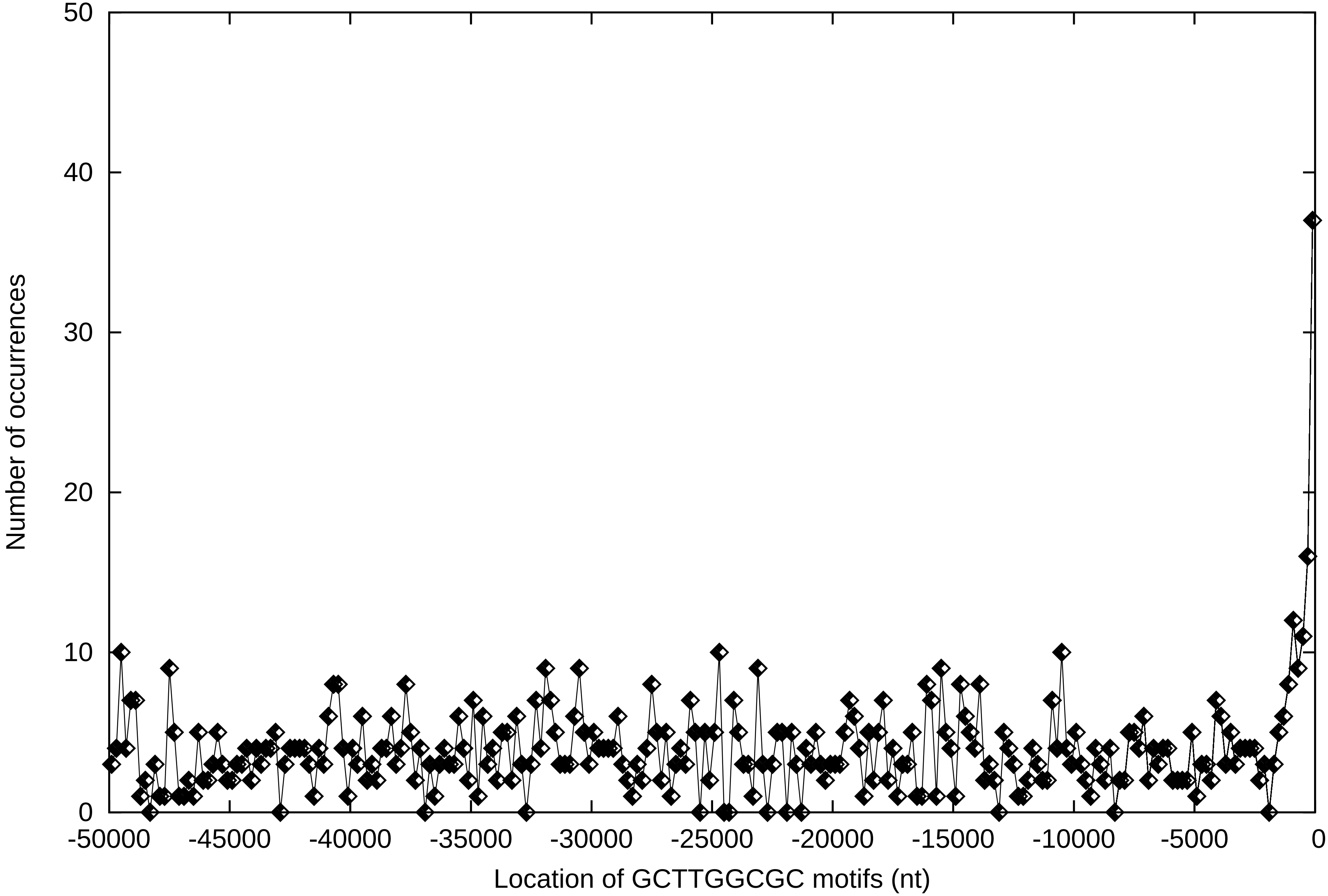

Number of occurrences
Location of GCTTGGCGC motifs (nt)
0
10
20
30
40
50
-50000
-45000
-40000
-35000
-30000
-25000
-20000
-15000
-10000
-5000
0

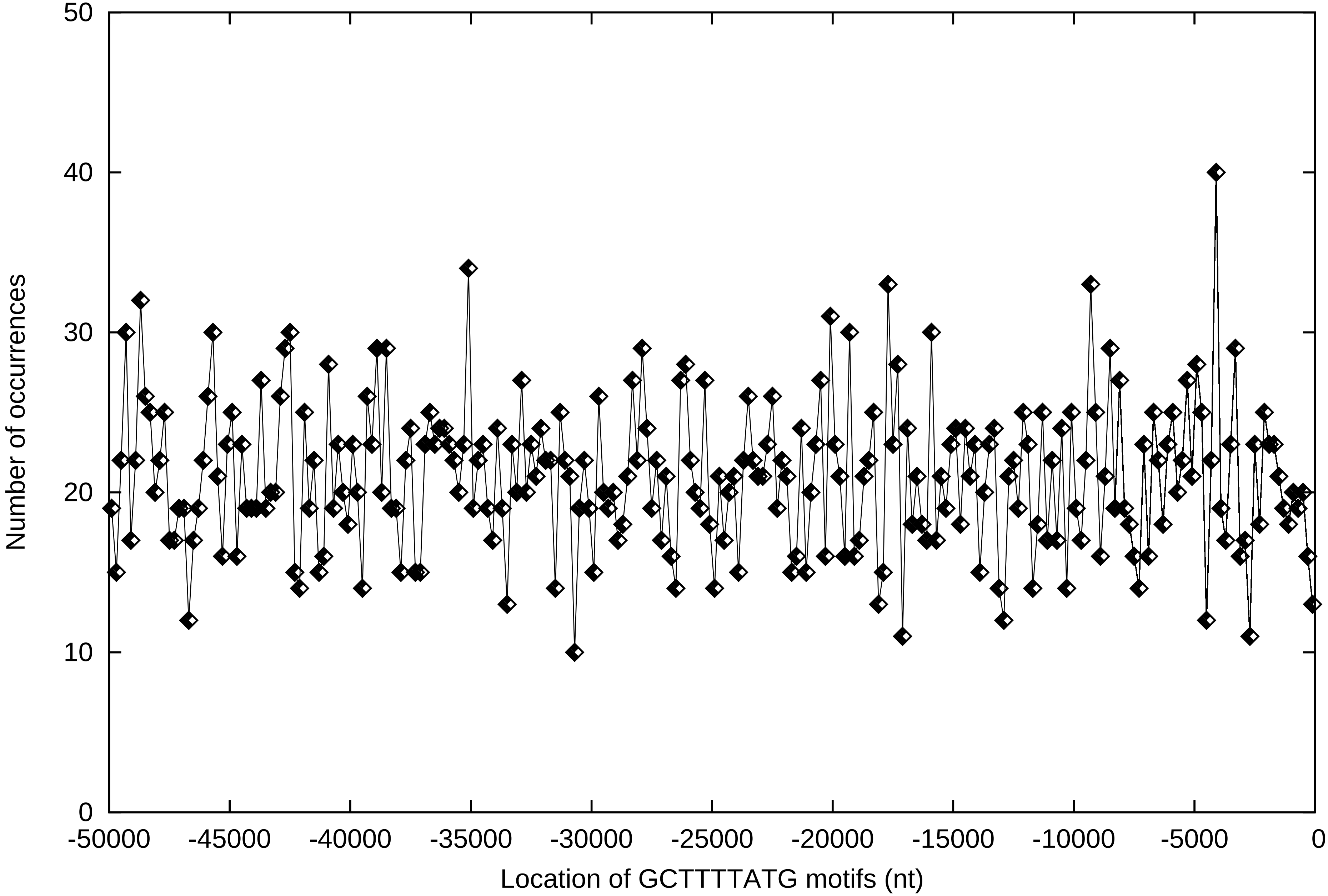

50
40
30
20
10
0
-50000
-45000
-40000
-35000
-30000
-25000
-20000
-15000
-10000
-5000
0
Number of occurrences
Location of GCTTTTATG motifs (nt)

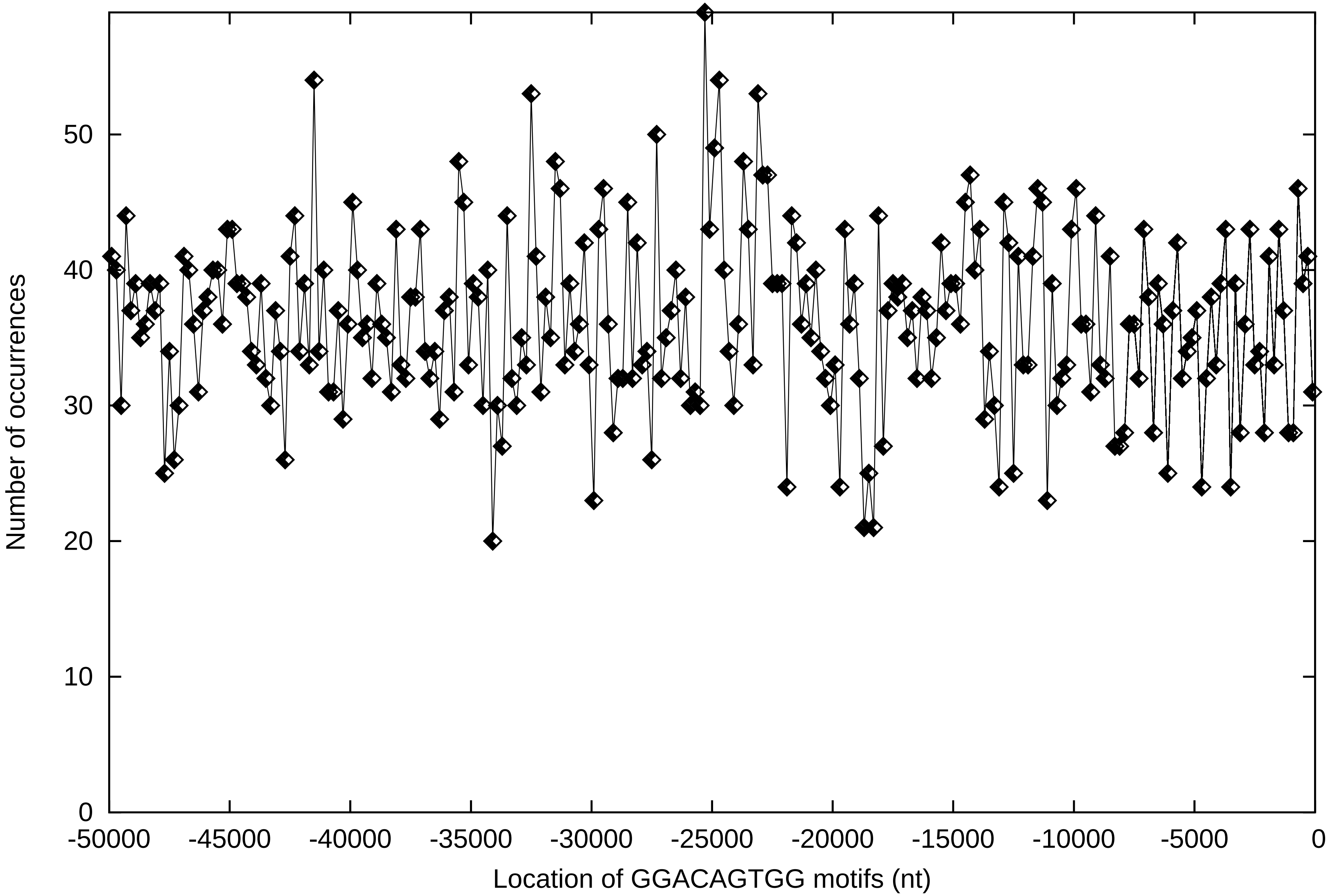

Number of occurrences
Location of GGACAGTGG motifs (nt)
0
10
20
30
40
50
-50000
-45000
-40000
-35000
-30000
-25000
-20000
-15000
-10000
-5000
0

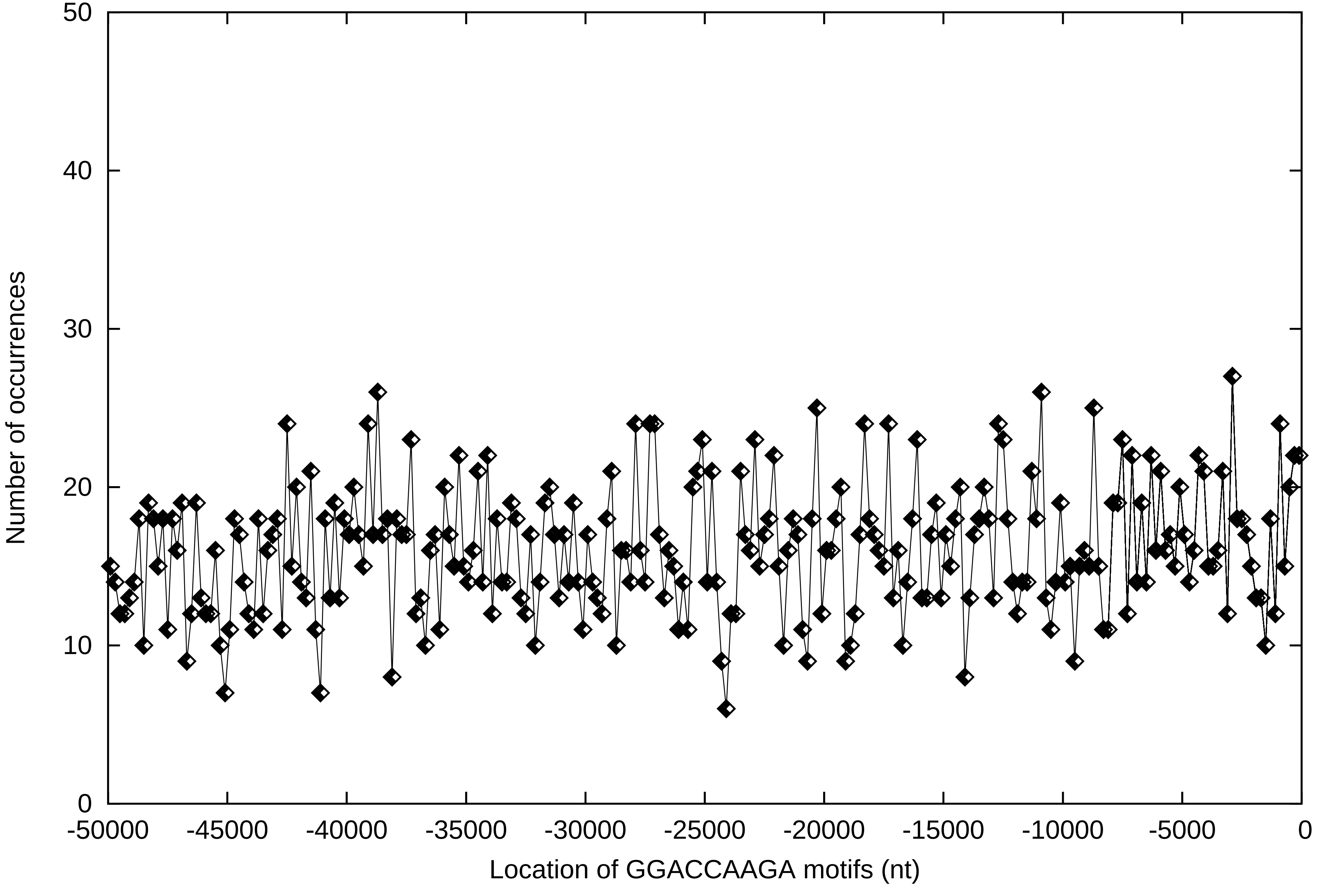

50
40
30
20
10
0
-50000
-45000
-40000
-35000
-30000
-25000
-20000
-15000
-10000
-5000
0
Number of occurrences
Location of GGACCAAGA motifs (nt)

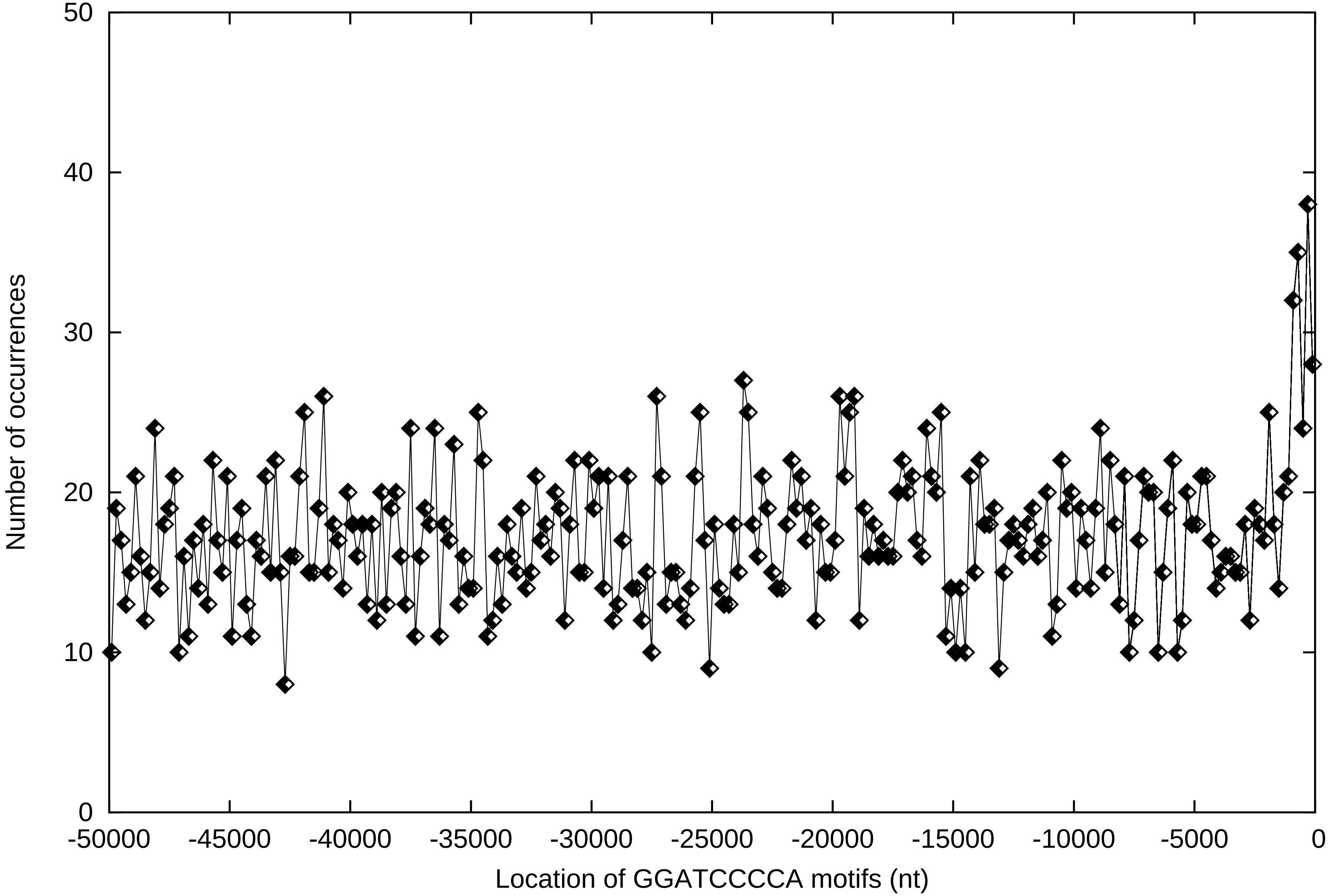

50
40
30
20
10
0
Number of occurrences
-50000
-45000
-40000
-35000
-30000
-25000
-20000
-15000
-10000
-5000
0
Location of GGATCCCCA motifs (nt)

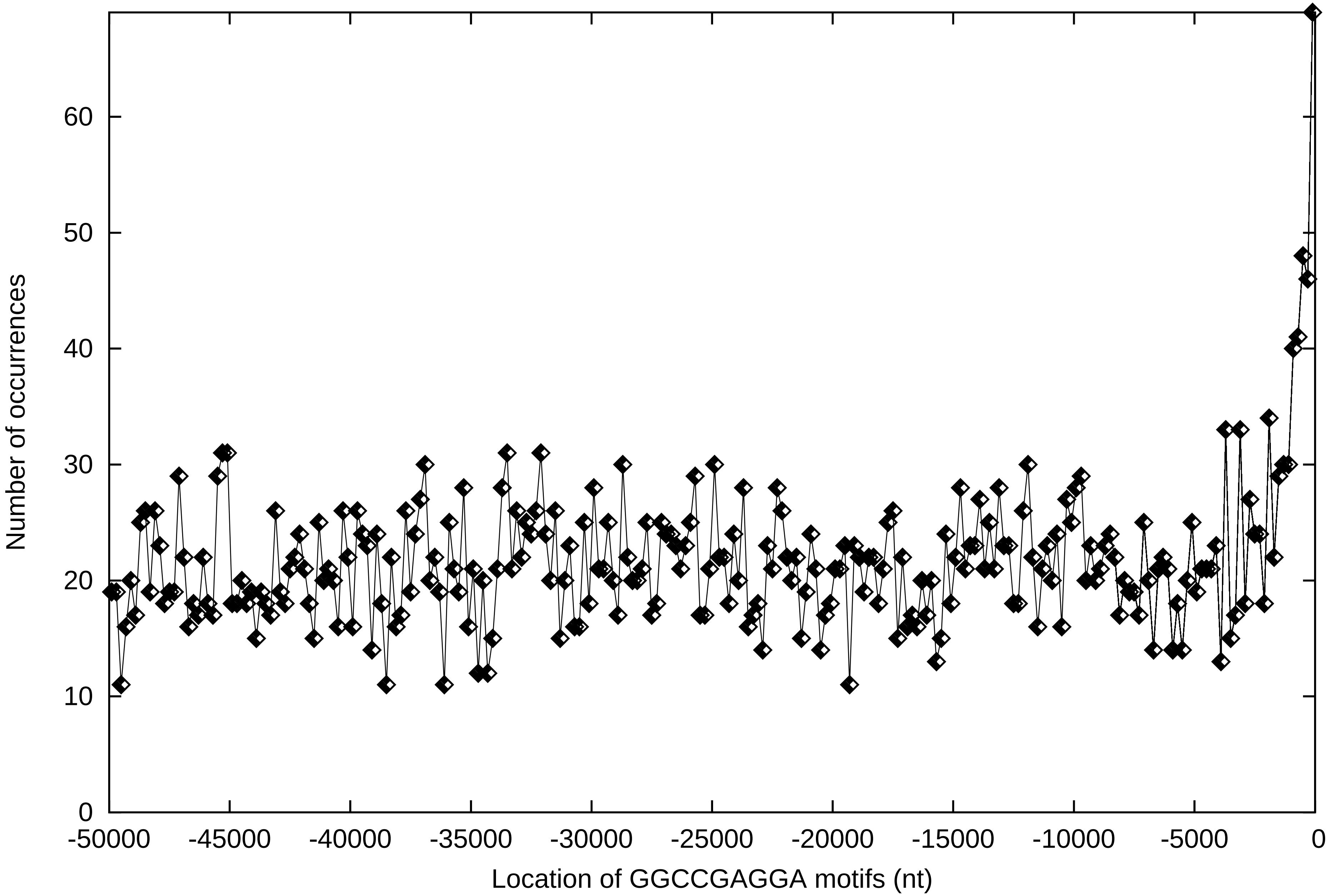

Number of occurrences
Location of GGCCGAGGA motifs (nt)
0
10
20
30
40
50
60
-50000
-45000
-40000
-35000
-30000
-25000
-20000
-15000
-10000
-5000
0

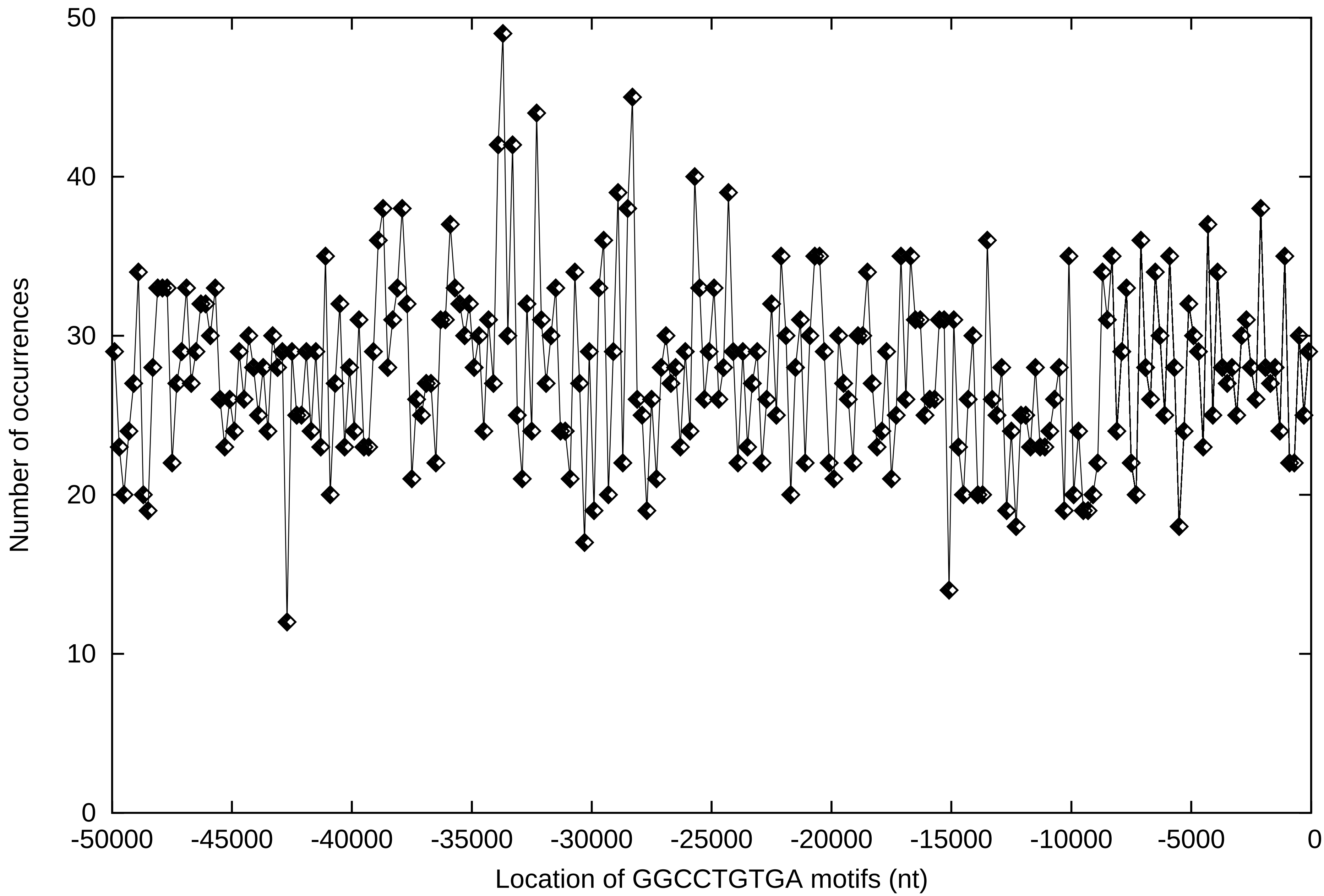

Number of occurrences
Location of GGCCTGTGA motifs (nt)
50
40
30
20
10
0
-50000
-45000
-40000
-35000
-30000
-25000
-20000
-15000
-10000
-5000
0

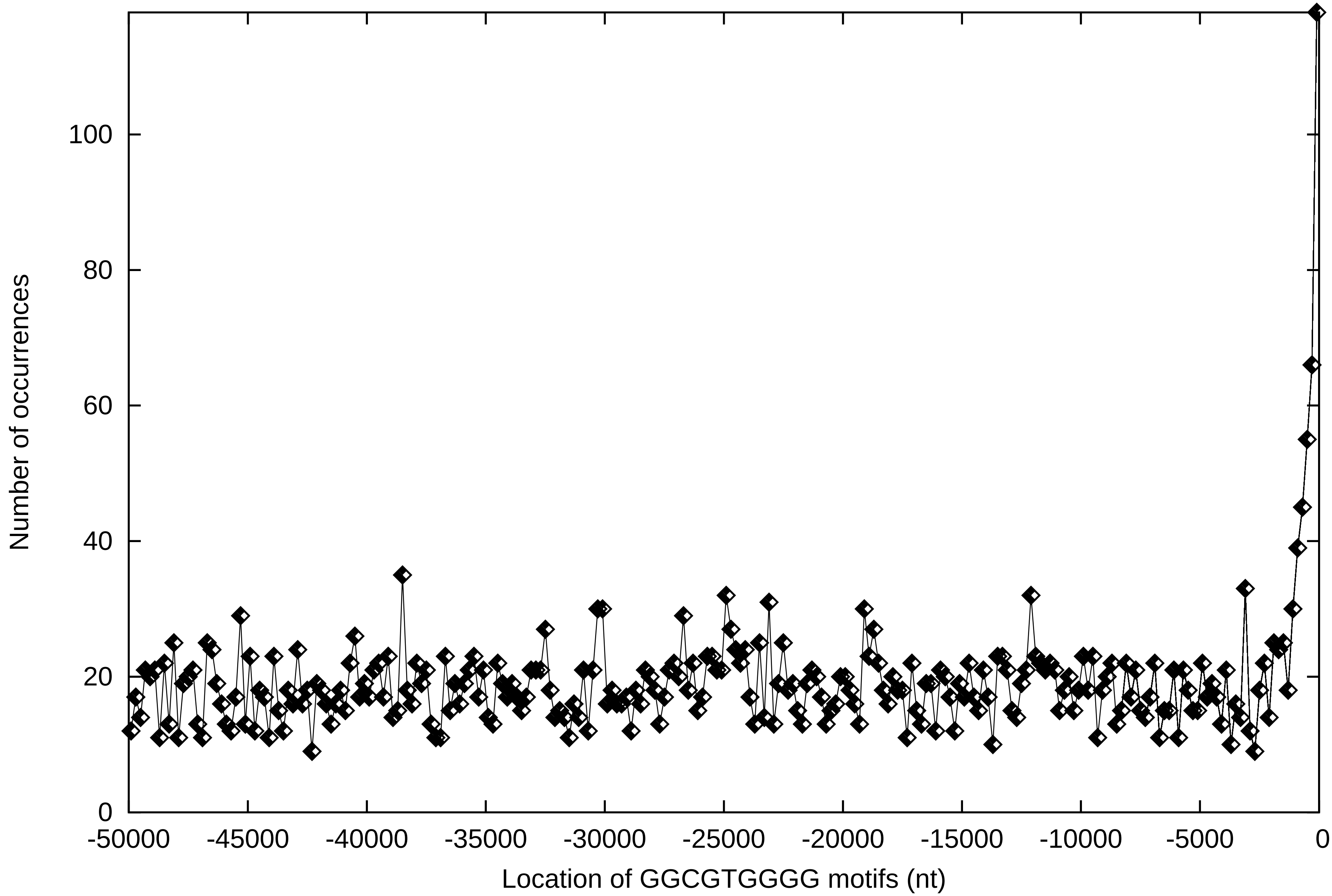

Number of occurrences
0
20
40
60
80
100
-50000
-45000
-40000
-35000
-30000
-25000
-20000
-15000
-10000
-5000
0
Location of GGCGTGGGG motifs (nt)

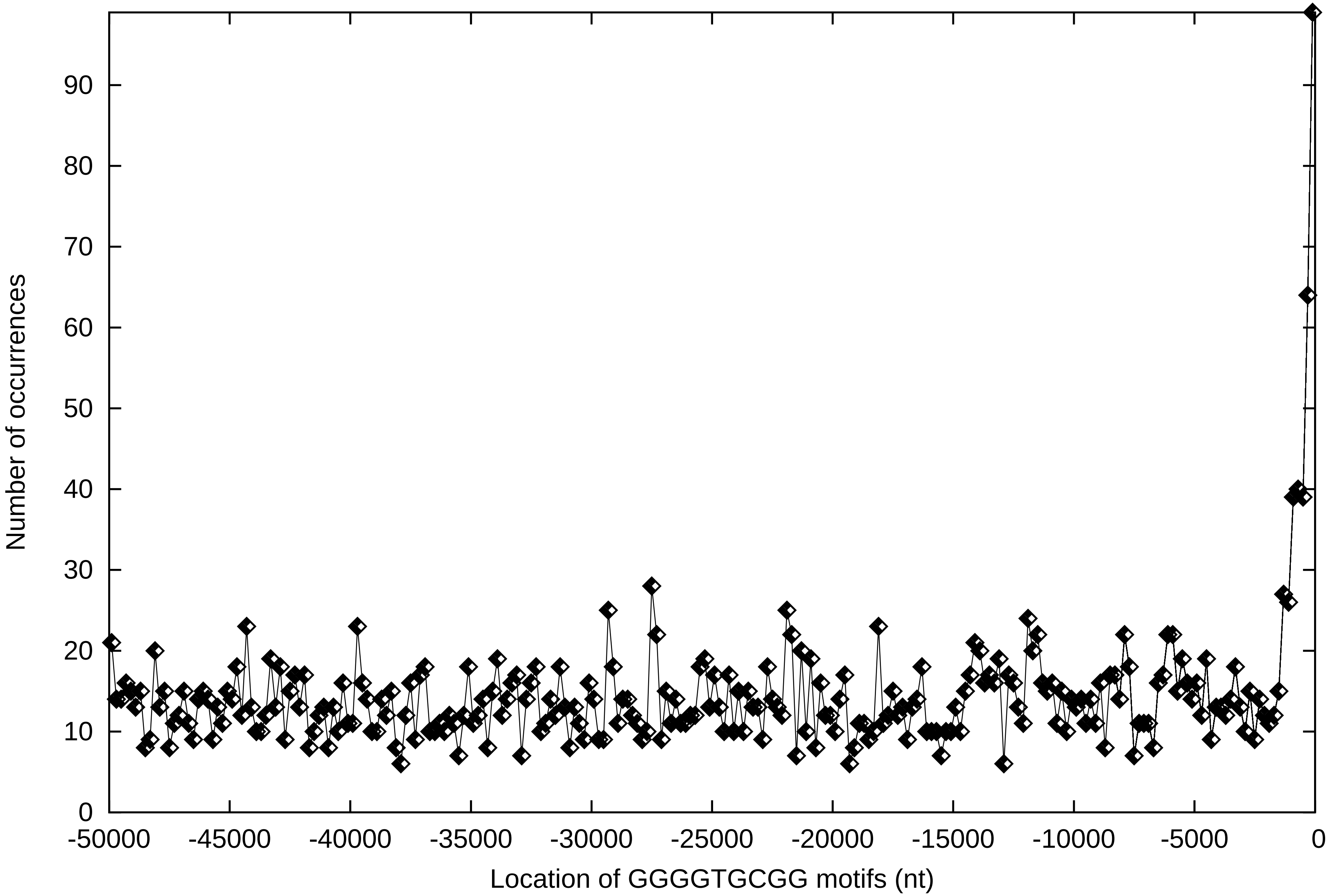

Number of occurrences
0
10
20
30
40
50
60
70
80
90
-50000
-45000
-40000
-35000
-30000
-25000
-20000
-15000
-10000
-5000
0
Location of GGGGTGCGG motifs (nt)

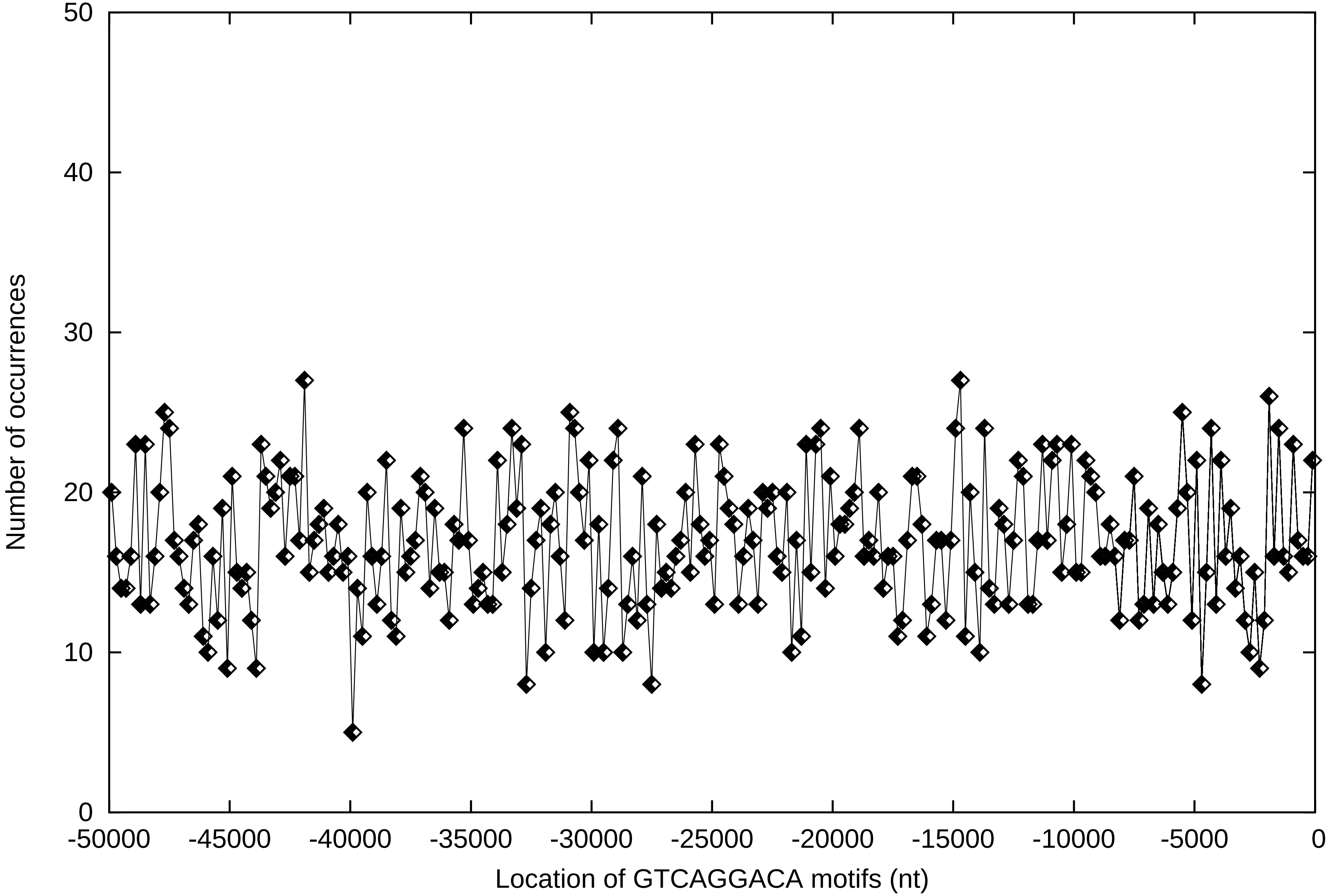

Number of occurrences
0
10
20
30
40
50
-50000
-45000
-40000
-35000
-30000
-25000
-20000
-15000
-10000
-5000
0
Location of GTCAGGACA motifs (nt)

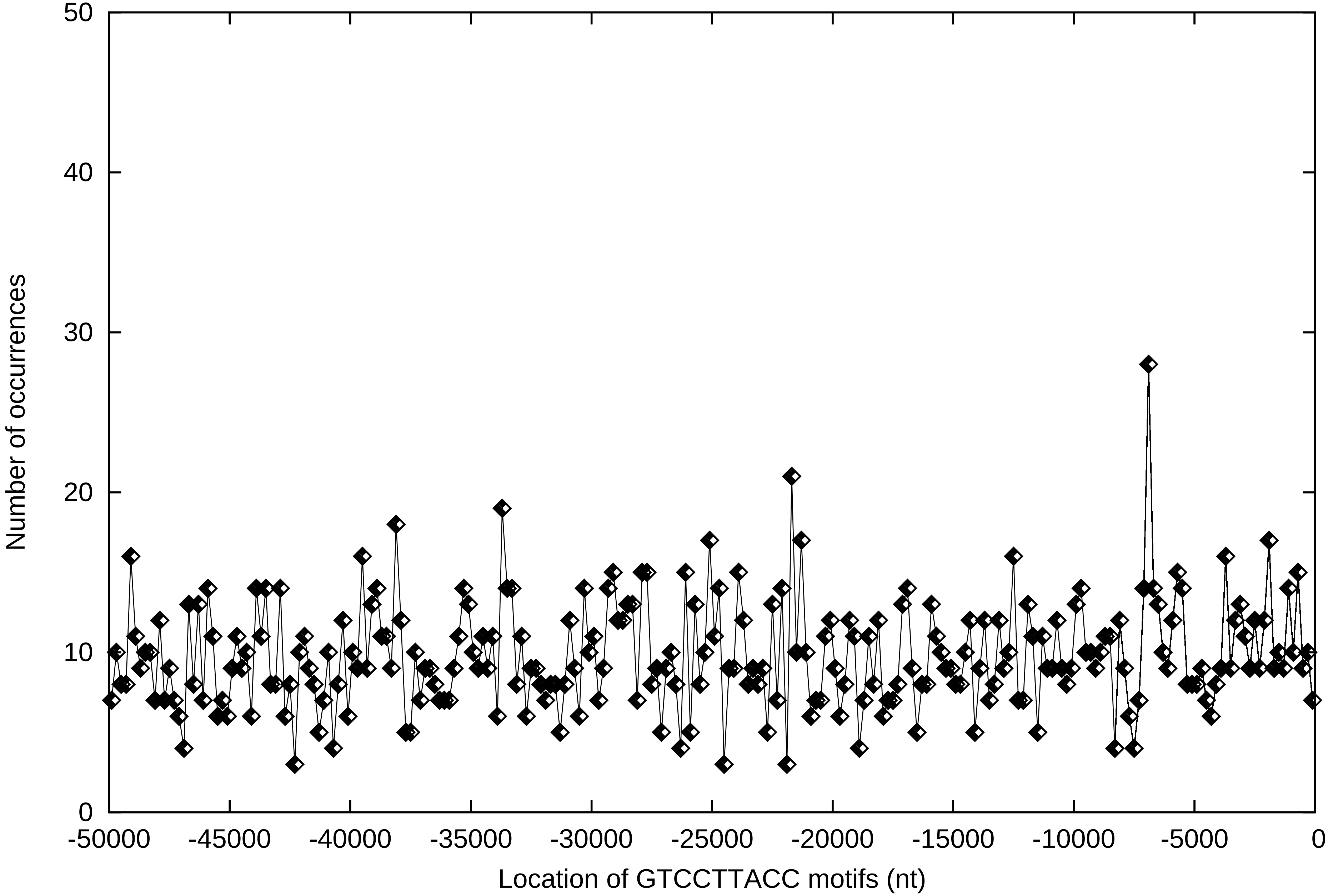

50
40
30
20
10
0
-50000
-45000
-40000
-35000
-30000
-25000
-20000
-15000
-10000
-5000
0
Number of occurrences
Location of GTCCTTACC motifs (nt)

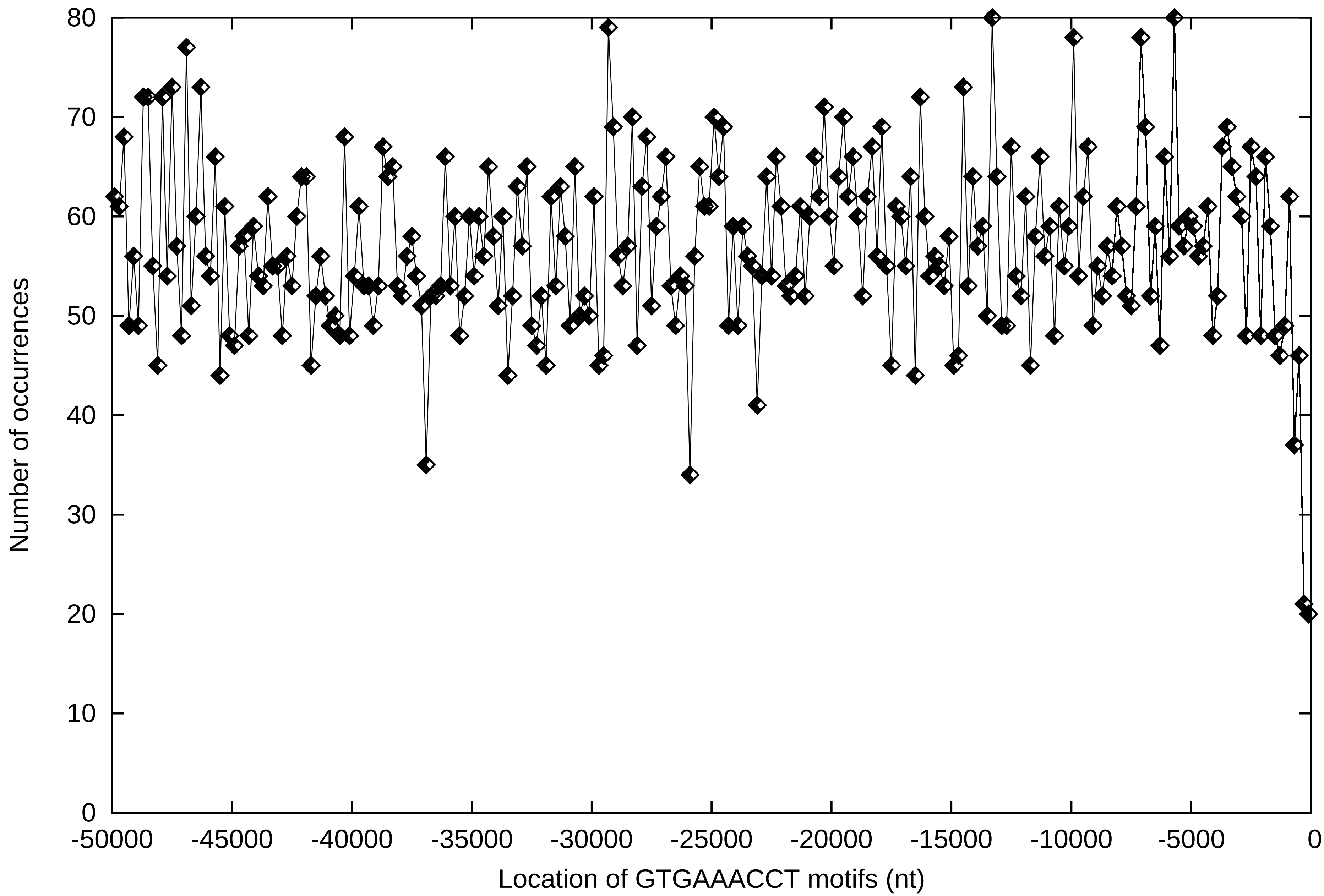

80
70
60
50
40
30
20
10
0
Number of occurrences
-50000
-45000
-40000
-35000
-30000
-25000
-20000
-15000
-10000
-5000
0
Location of GTGAAACCT motifs (nt)

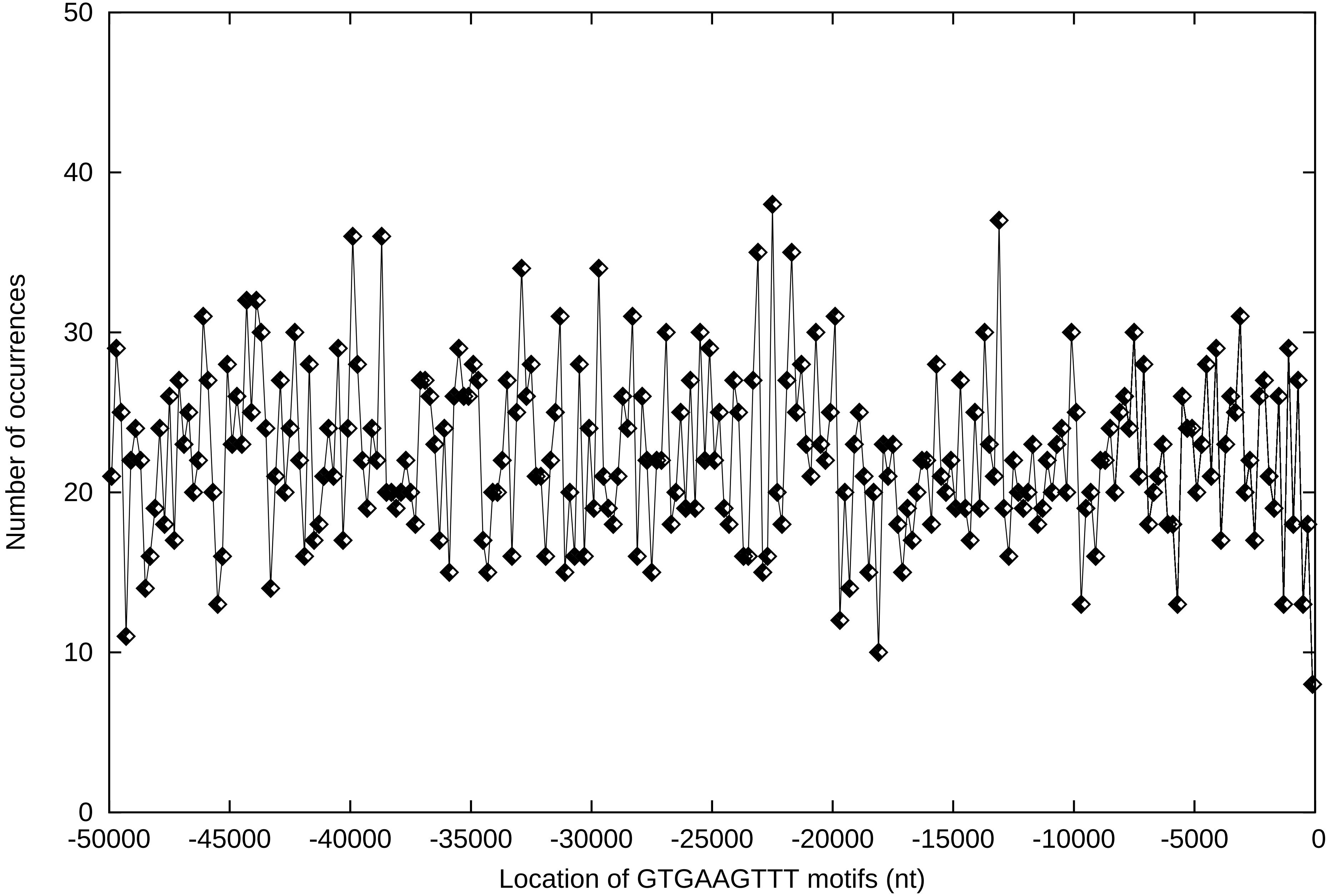

Number of occurrences
Location of GTGAAGTTT motifs (nt)
50
40
30
20
10
0
-50000
-45000
-40000
-35000
-30000
-25000
-20000
-15000
-10000
-5000
0

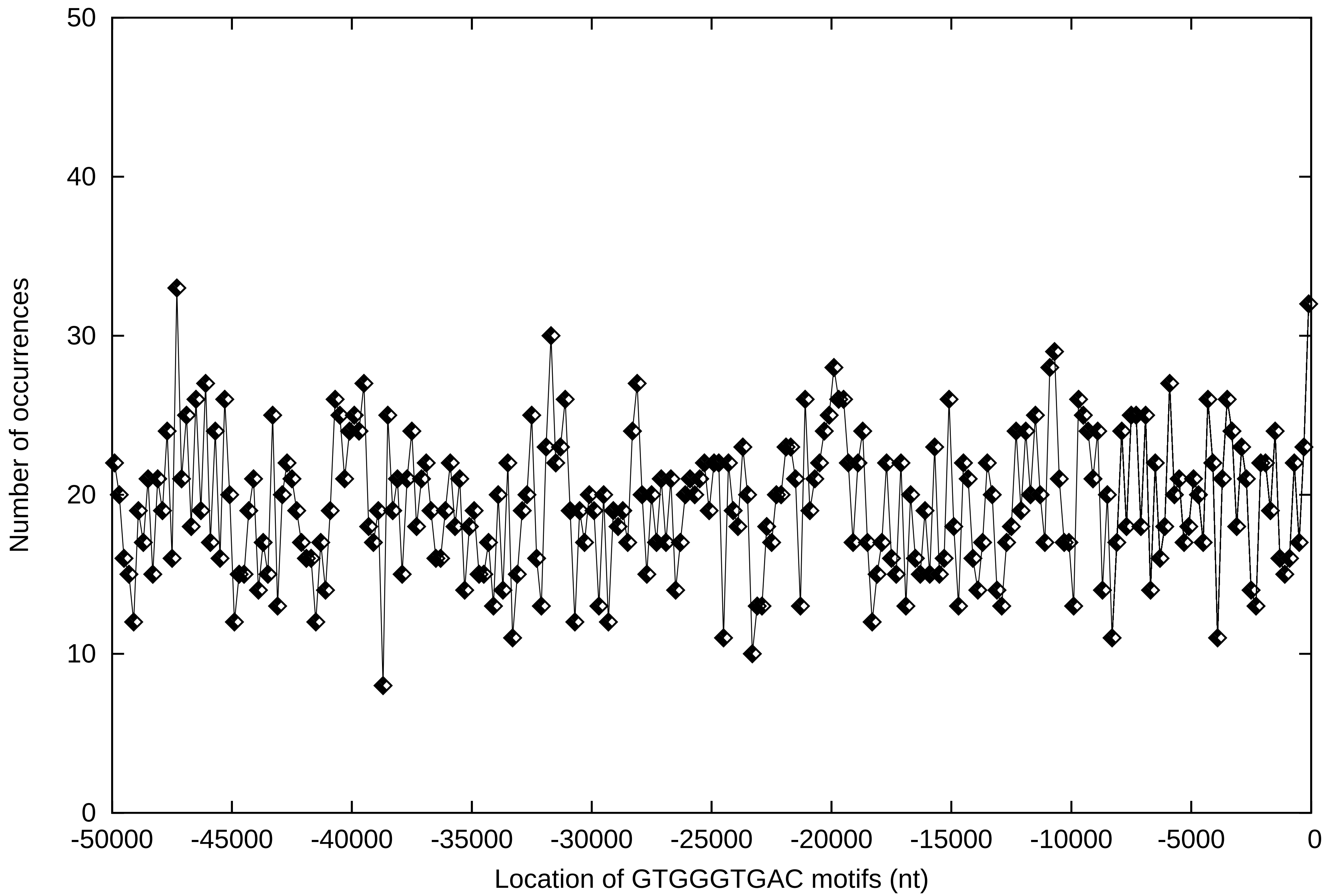

Number of occurrences
0
10
20
30
40
50
-50000
-45000
-40000
-35000
-30000
-25000
-20000
-15000
-10000
-5000
0
Location of GTGGGTGAC motifs (nt)

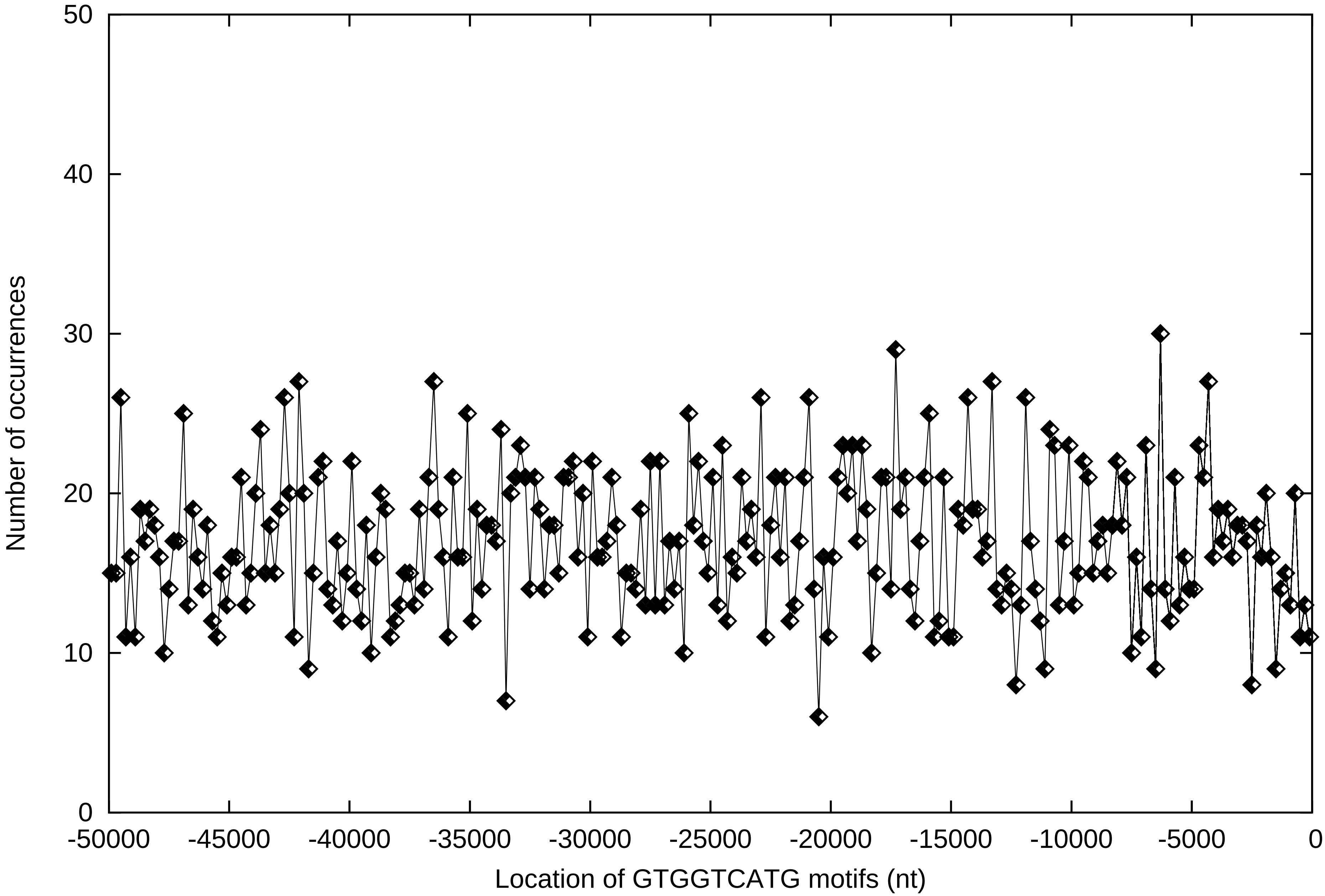

50
40
30
20
10
0
Number of occurrences
-50000
-45000
-40000
-35000
-30000
-25000
-20000
-15000
-10000
-5000
0
Location of GTGGTCATG motifs (nt)

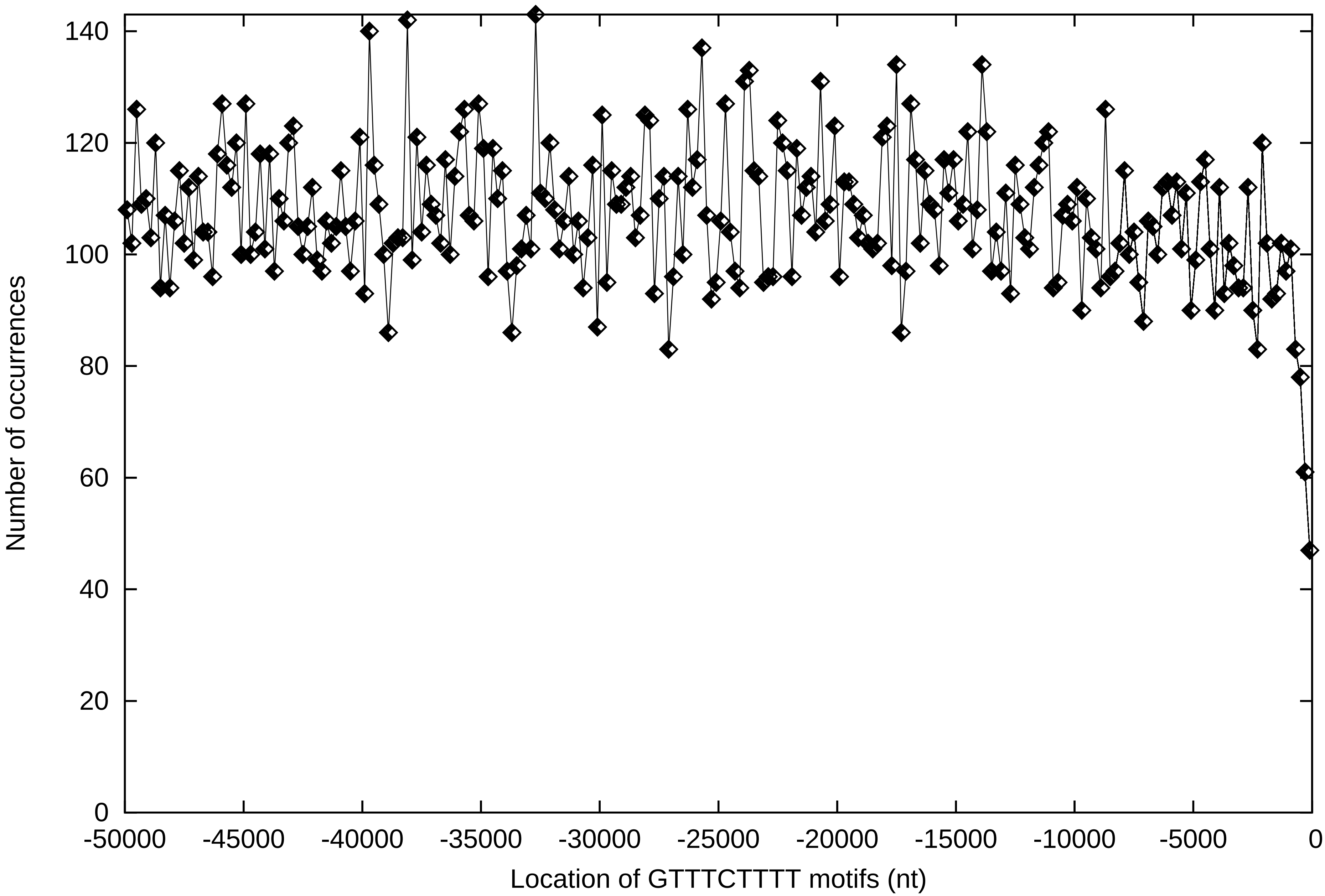

Number of occurrences
Location of GTTTCTTTT motifs (nt)
0
20
40
60
80
100
120
140
-50000
-45000
-40000
-35000
-30000
-25000
-20000
-15000
-10000
-5000
0

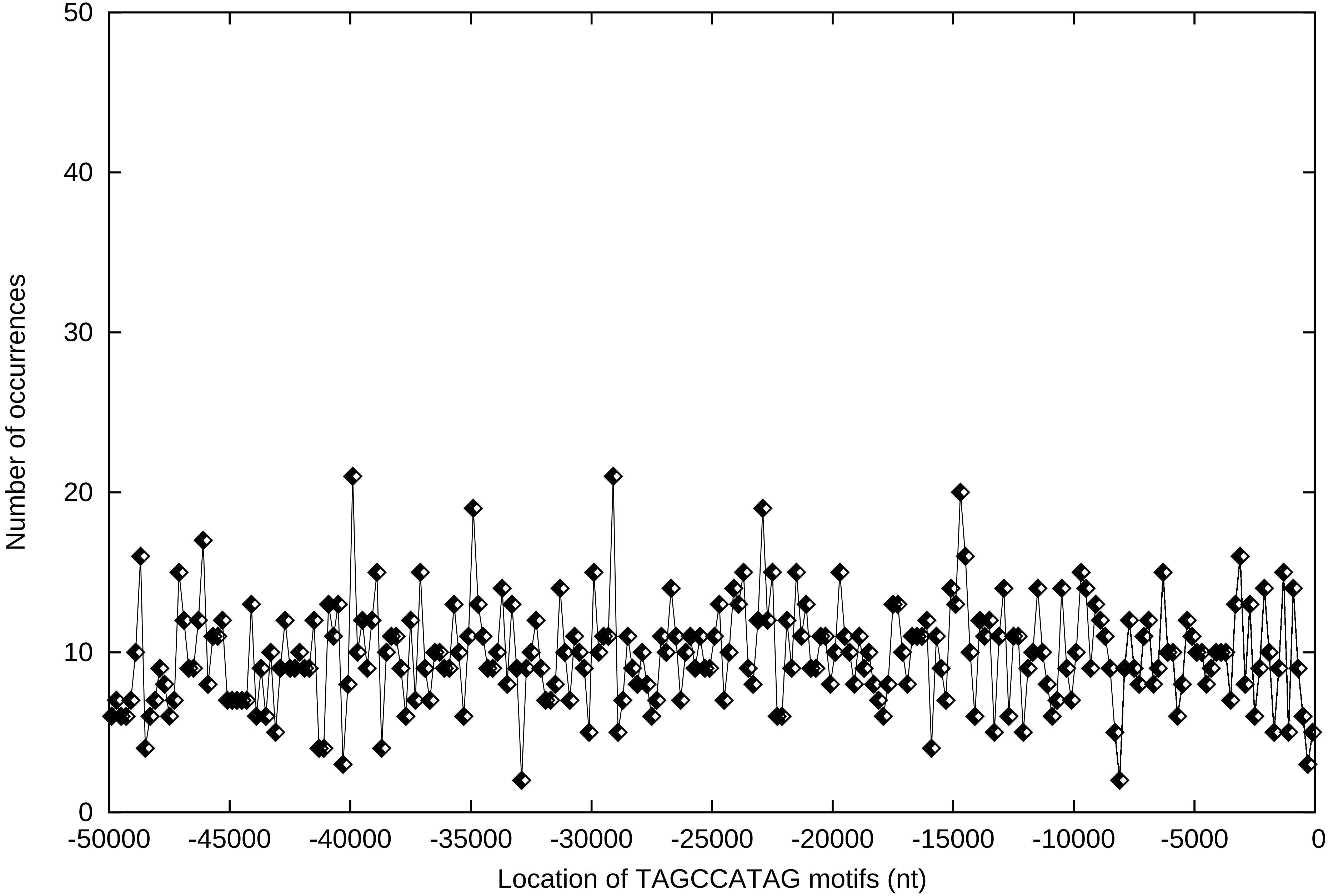

50
40
30
20
10
0
-50000
-45000
-40000
-35000
-30000
-25000
-20000
-15000
-10000
-5000
0
Number of occurrences
Location of TAGCCATAG motifs (nt)

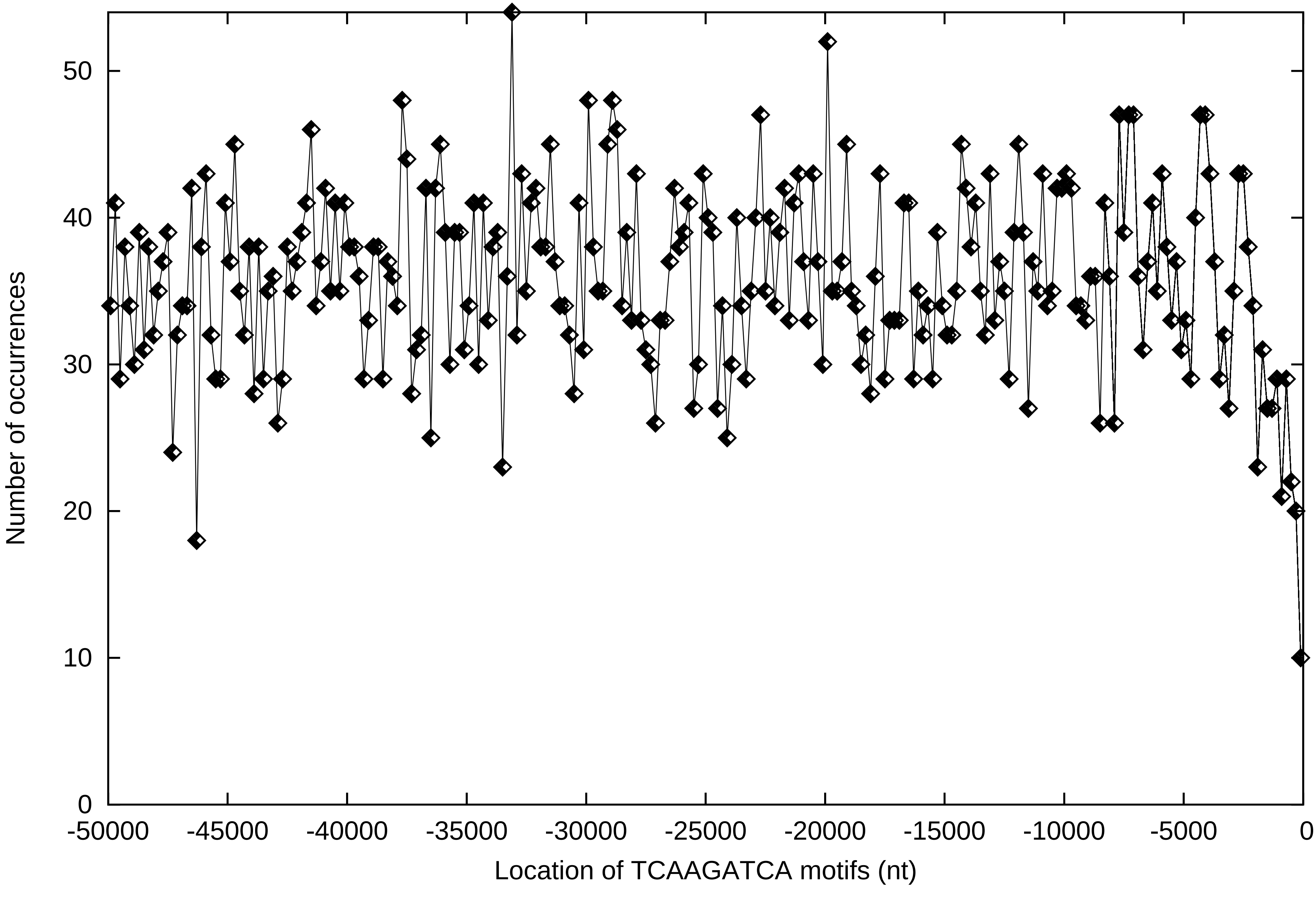

Number of occurrences
0
10
20
30
40
50
-50000
-45000
-40000
-35000
-30000
-25000
-20000
-15000
-10000
-5000
0
Location of TCAAGATCA motifs (nt)

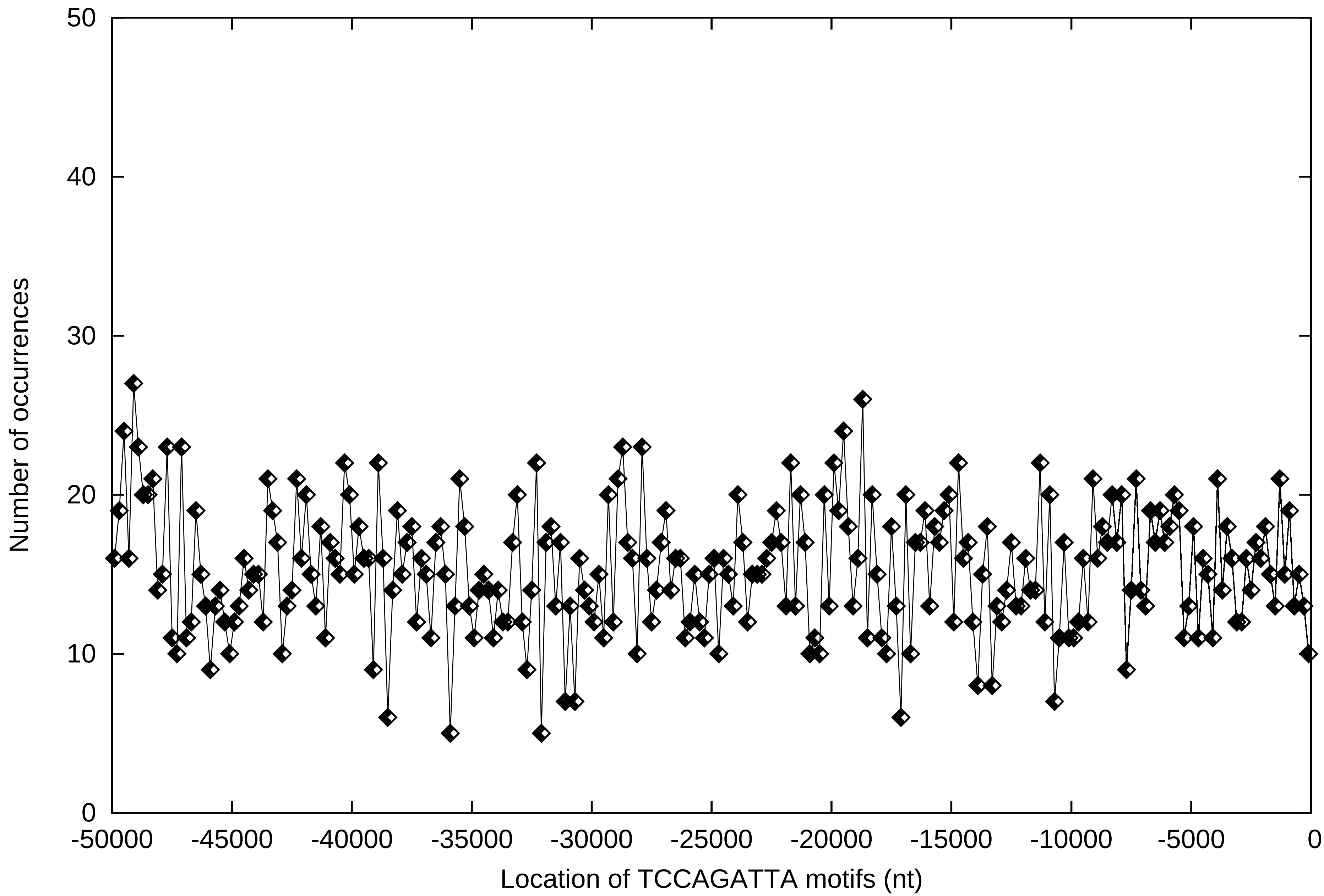
50
40
30
20
10
0
-50000
-45000
-40000
-35000
-30000
-25000
-20000
-15000
-10000
-5000
0
Number of occurrences
Location of TCCAGATTA motifs (nt)

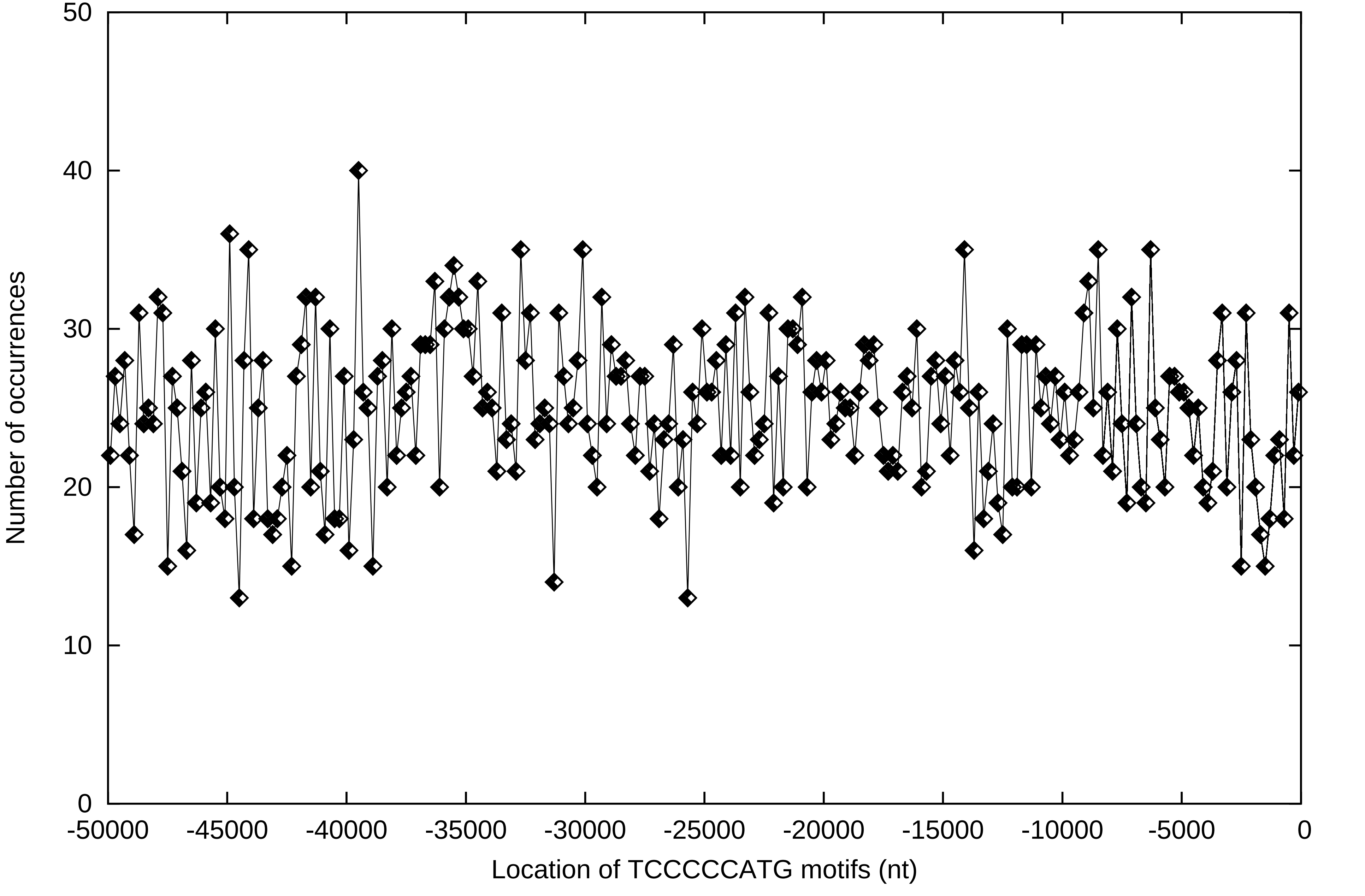

50
40
30
20
10
0
-50000
-45000
-40000
-35000
-30000
-25000
-20000
-15000
-10000
-5000
0
Number of occurrences
Location of TCCCCCATG motifs (nt)

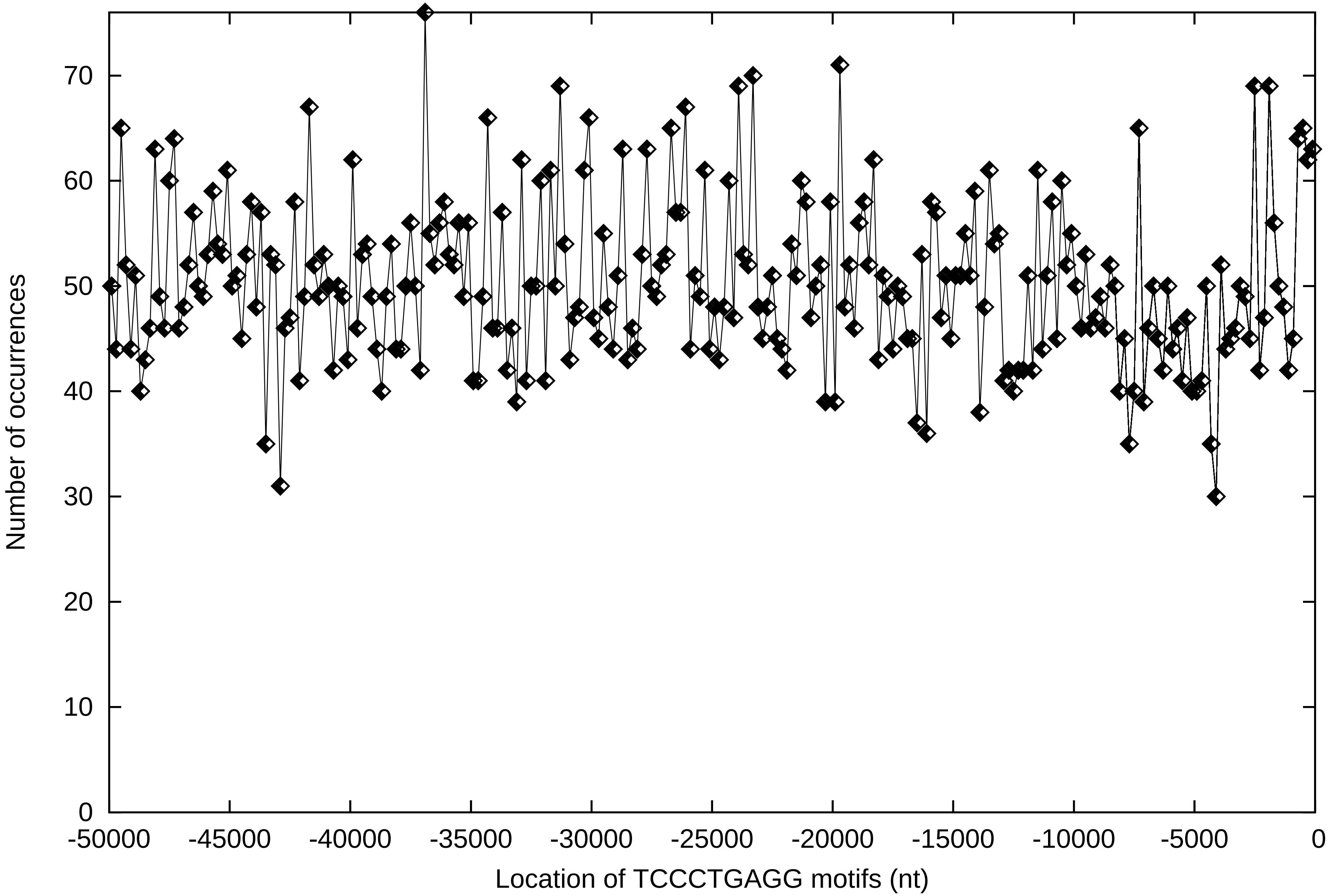

Number of occurrences
Location of TCCCTGAGG motifs (nt)
0
10
20
30
40
50
60
70
-50000
-45000
-40000
-35000
-30000
-25000
-20000
-15000
-10000
-5000
0

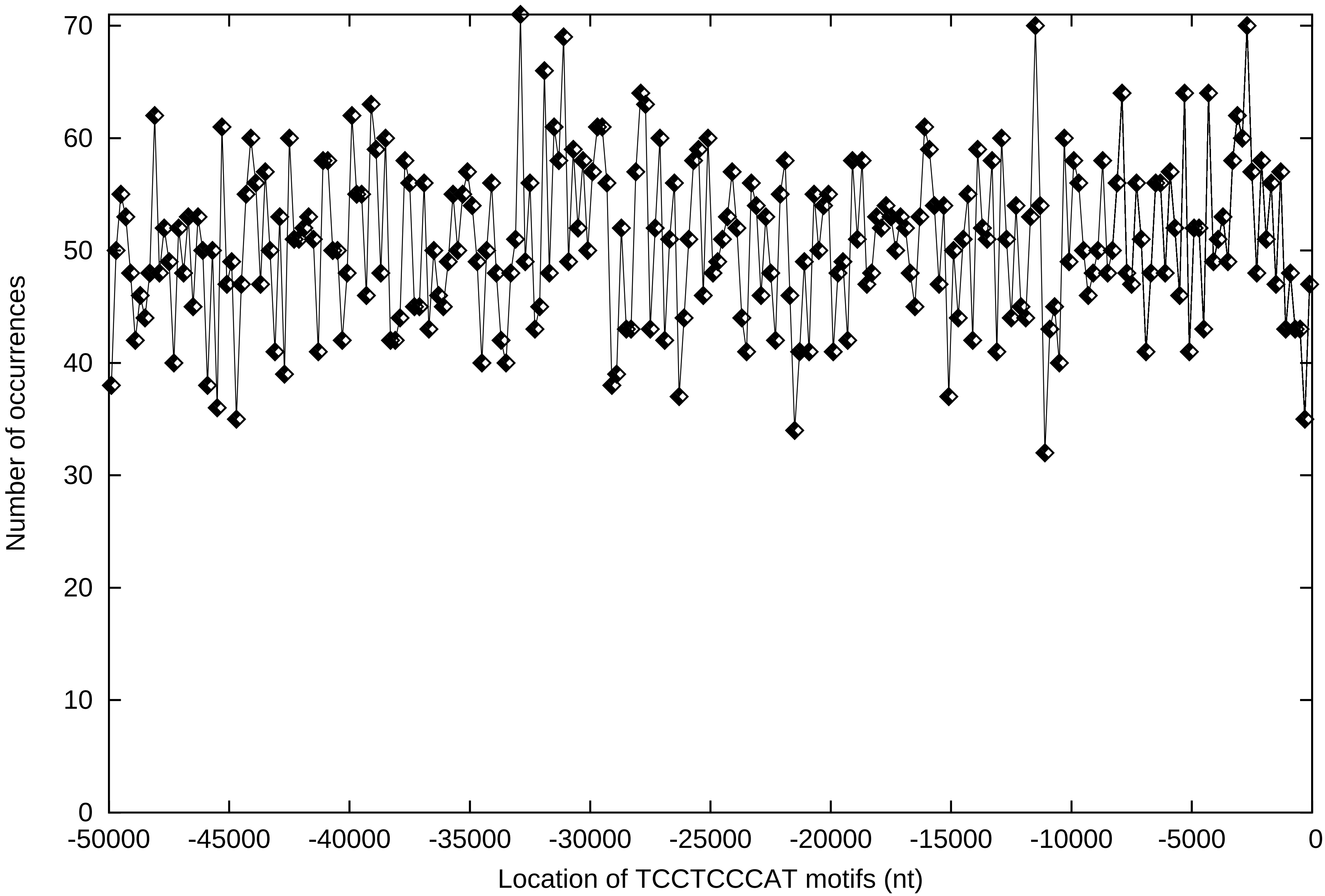

Number of occurrences
70
60
50
40
30
20
10
0
-50000
-45000
-40000
-35000
-30000
-25000
-20000
-15000
-10000
-5000
0
Location of TCCTCCCAT motifs (nt)

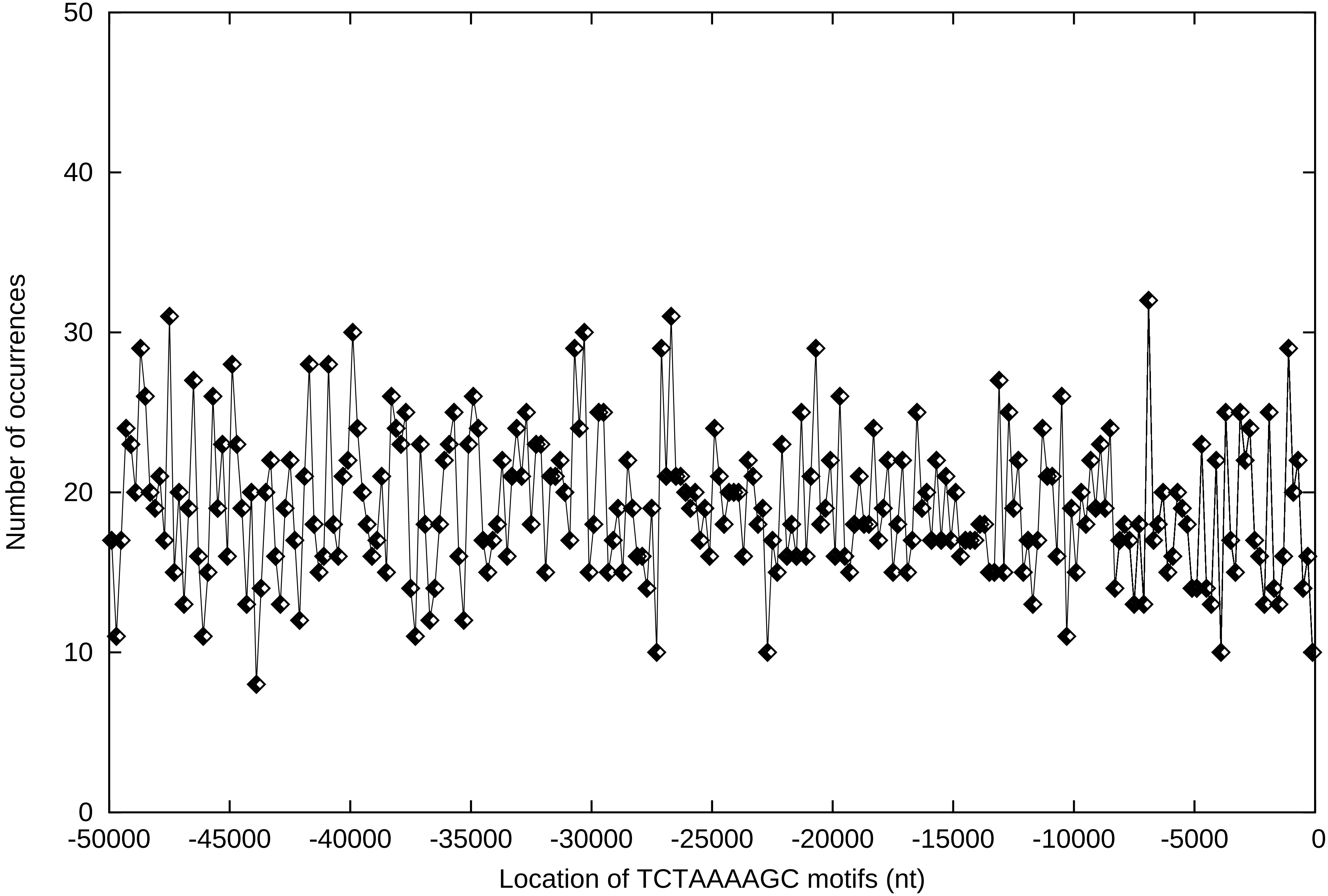

50
40
30
20
10
0
-50000
-45000
-40000
-35000
-30000
-25000
-20000
-15000
-10000
-5000
0
Number of occurrences
Location of TCTAAAAGC motifs (nt)

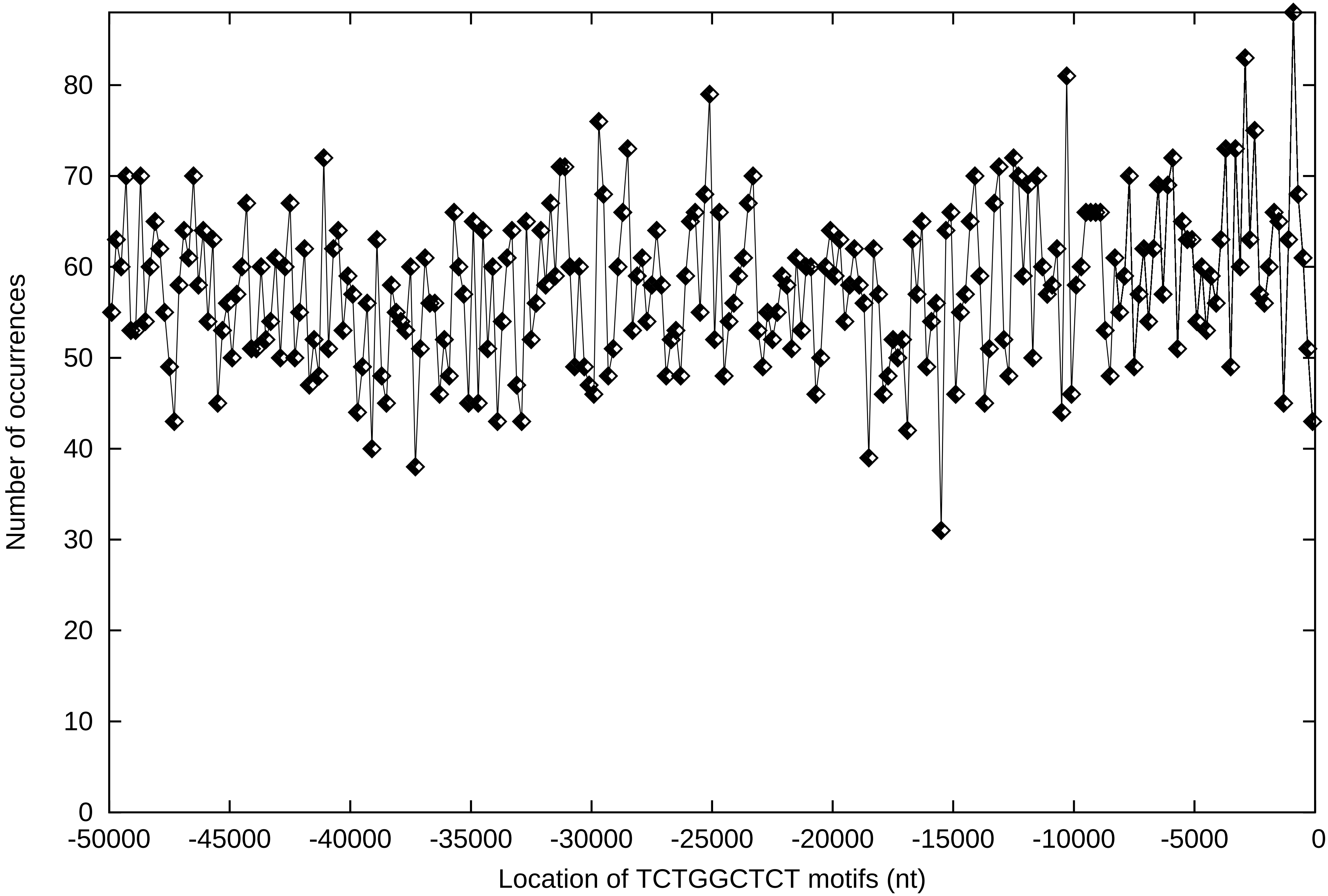

Number of occurrences
0
10
20
30
40
50
60
70
80
-50000
-45000
-40000
-35000
-30000
-25000
-20000
-15000
-10000
-5000
0
Location of TCTGGCTCT motifs (nt)

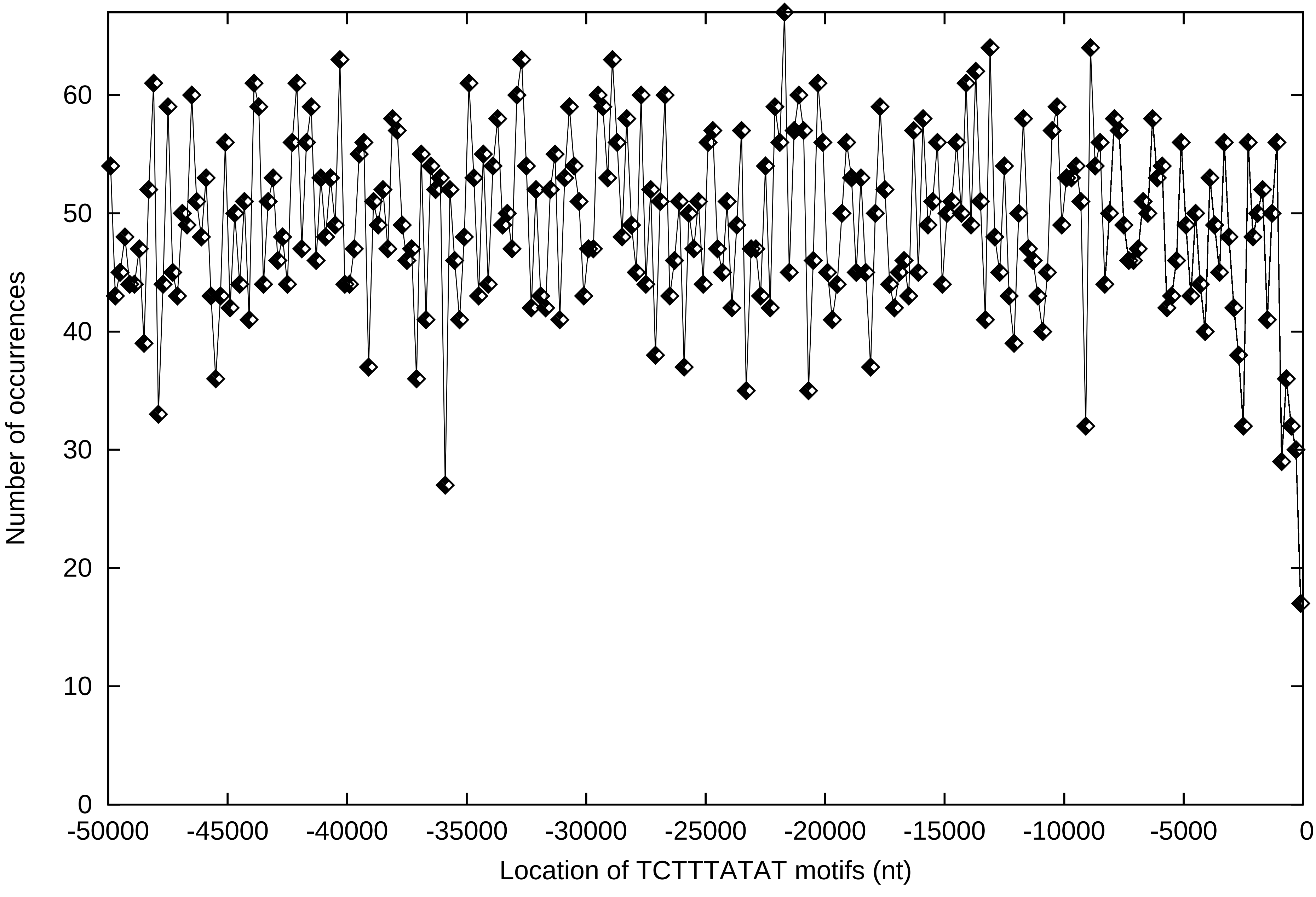

Number of occurrences
0
10
20
30
40
50
60
-50000
-45000
-40000
-35000
-30000
-25000
-20000
-15000
-10000
-5000
0
Location of TCTTTATAT motifs (nt)

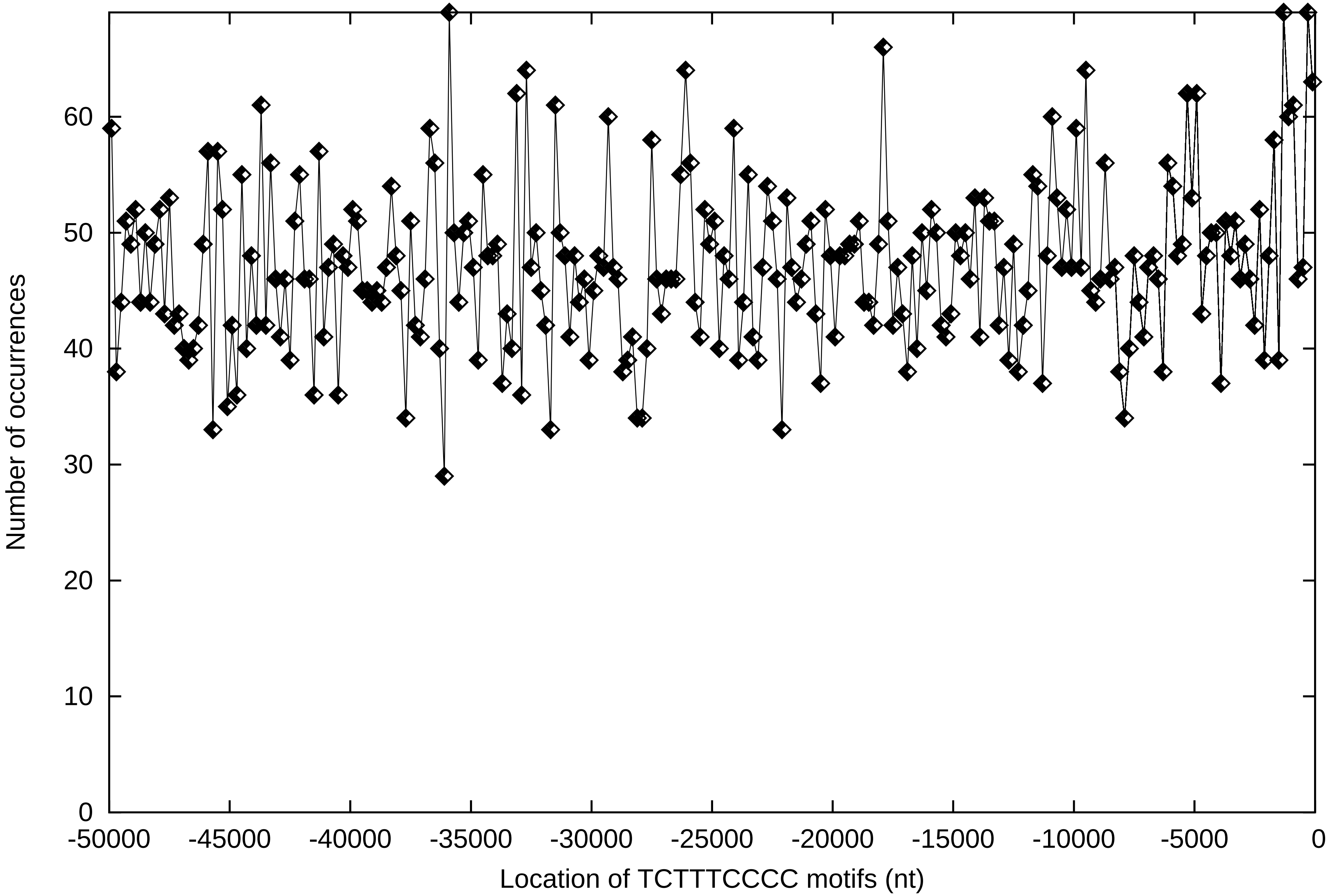

Number of occurrences
0
10
20
30
40
50
60
-50000
-45000
-40000
-35000
-30000
-25000
-20000
-15000
-10000
-5000
0
Location of TCTTTCCCC motifs (nt)

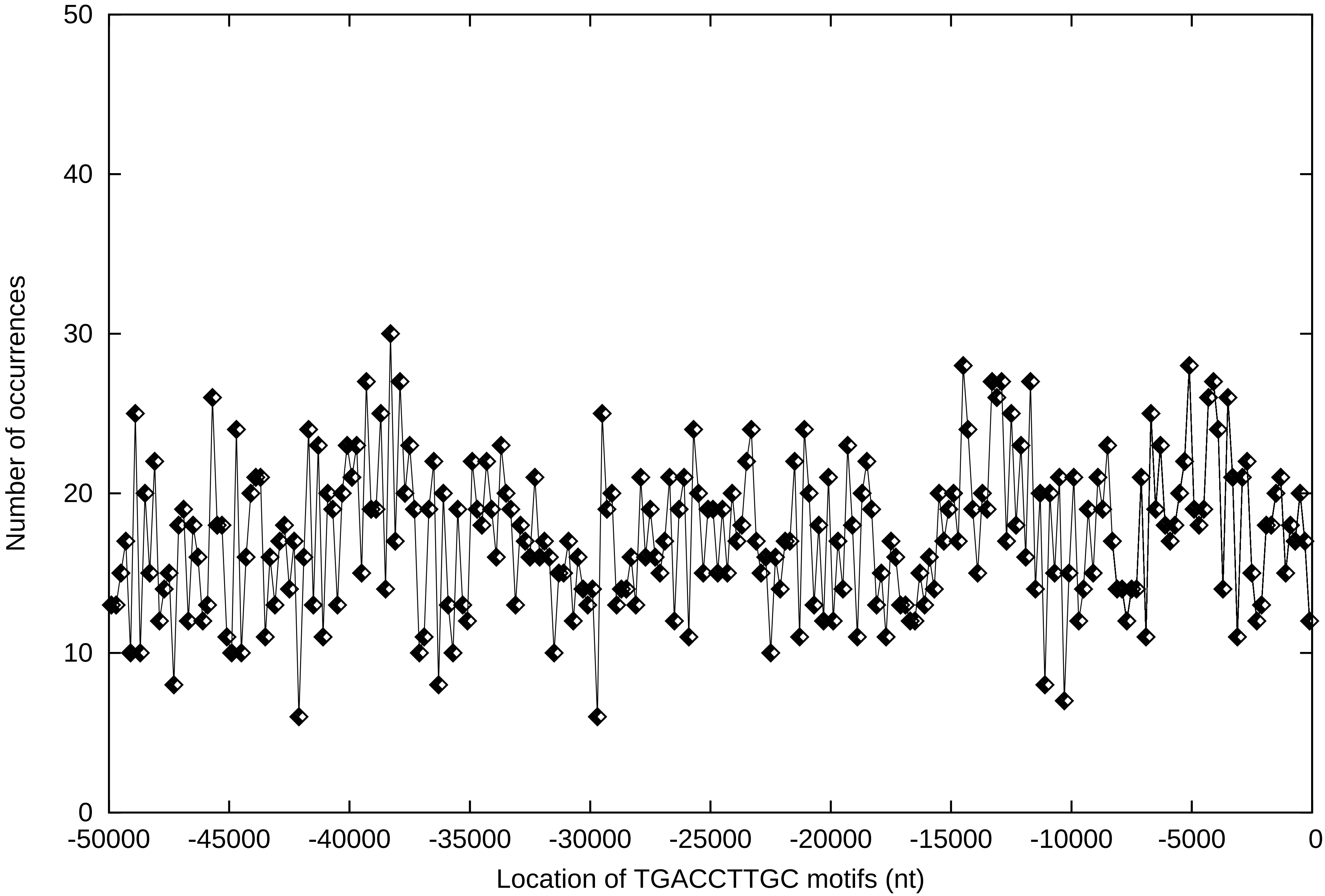

50
40
30
20
10
0
-50000
-45000
-40000
-35000
-30000
-25000
-20000
-15000
-10000
-5000
0
Number of occurrences
Location of TGACCTTGC motifs (nt)

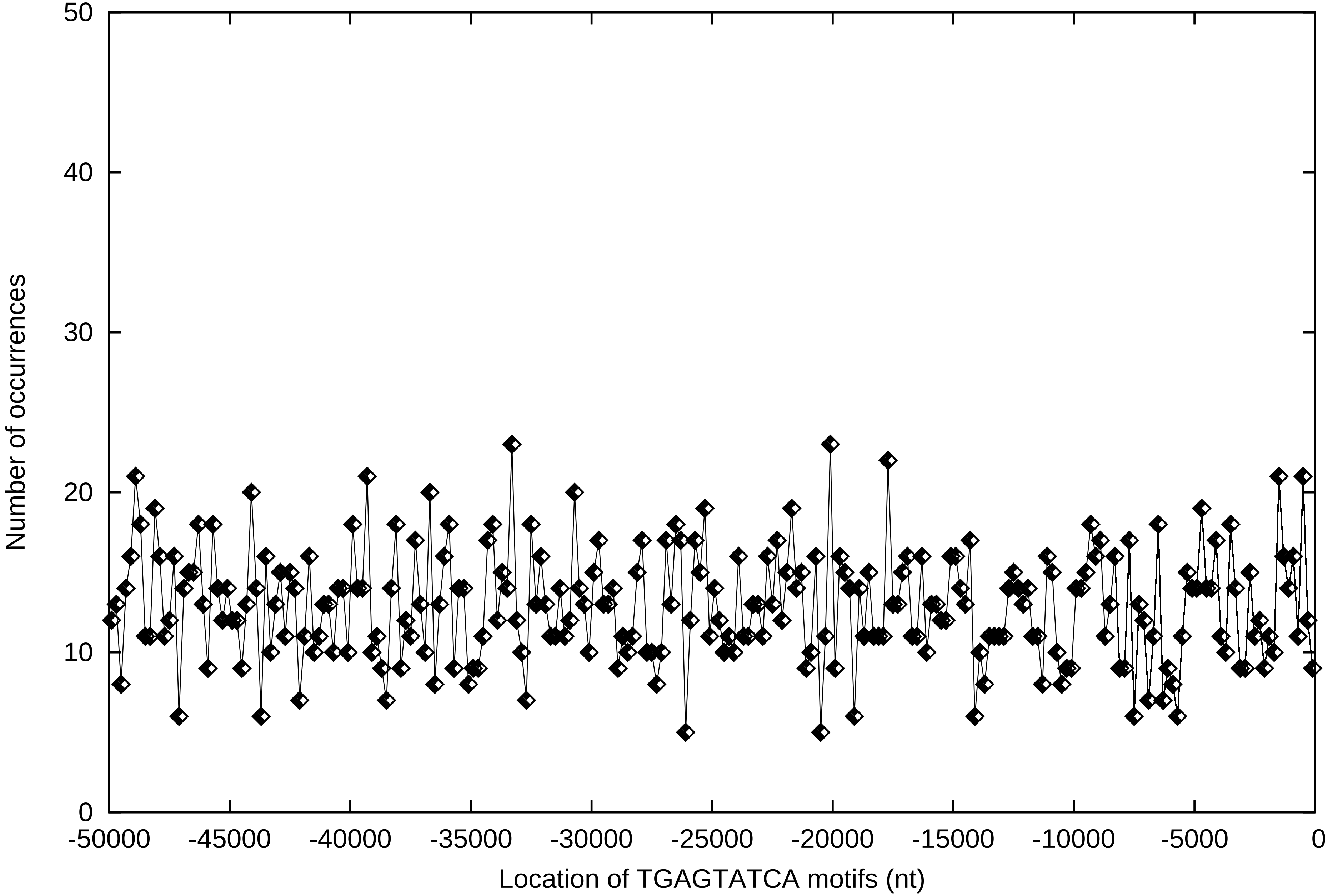

50
40
30
20
10
0
-50000
-45000
-40000
-35000
-30000
-25000
-20000
-15000
-10000
-5000
0
Number of occurrences
Location of TGAGTATCA motifs (nt)

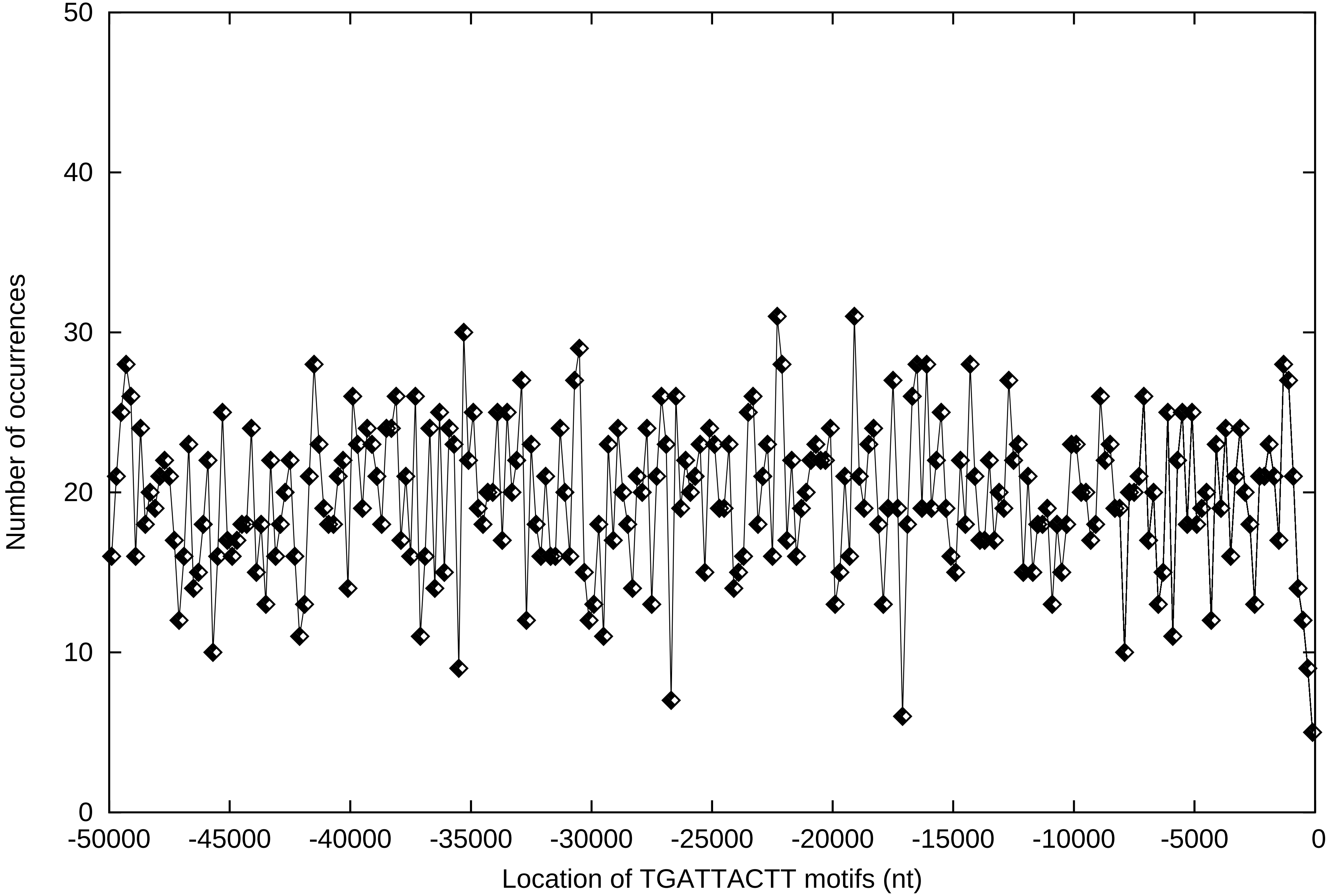

50
40
30
20
10
0
Number of occurrences
-50000
-45000
-40000
-35000
-30000
-25000
-20000
-15000
-10000
-5000
0
Location of TGATTACTT motifs (nt)

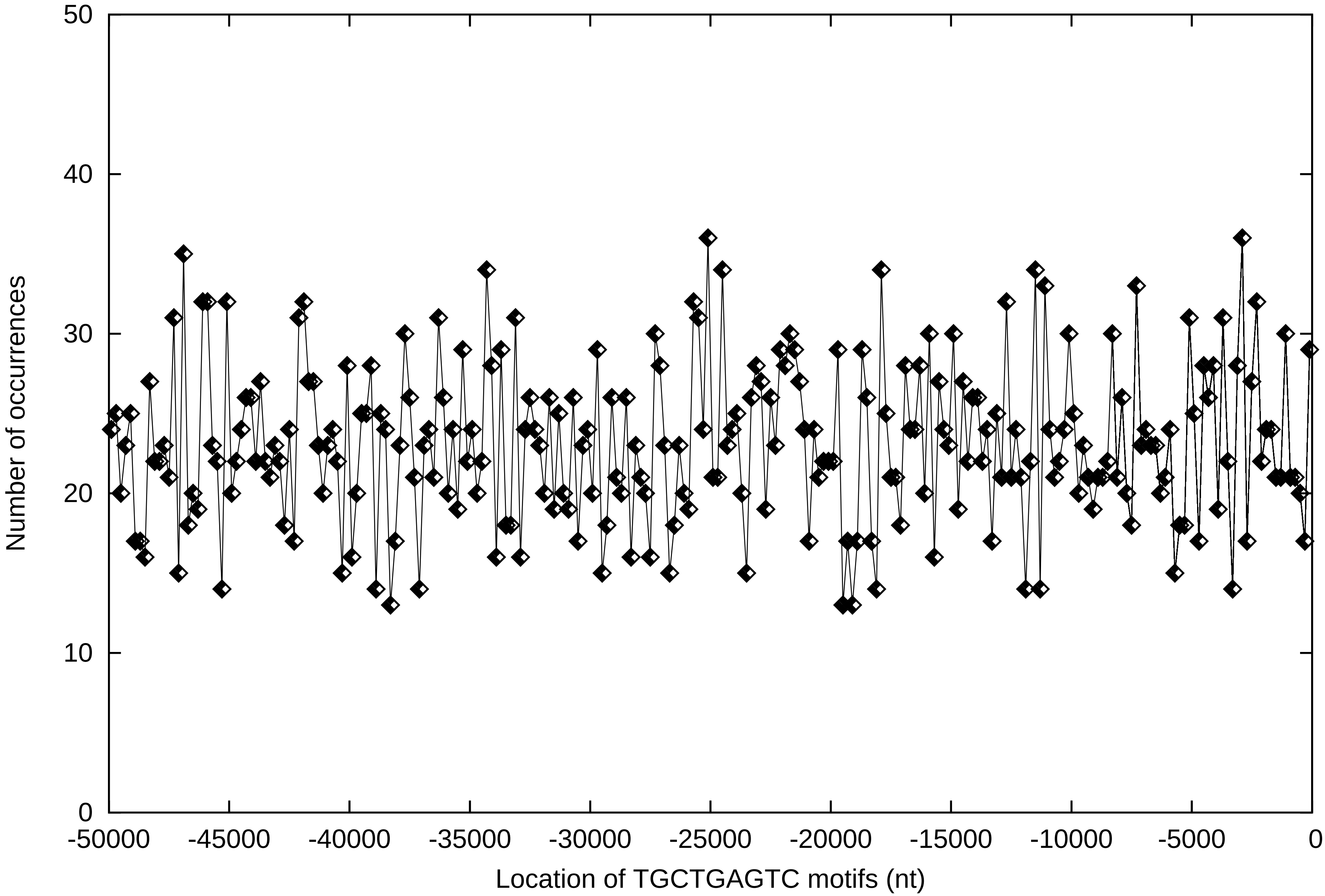

50
40
30
20
10
0
-50000
-45000
-40000
-35000
-30000
-25000
-20000
-15000
-10000
-5000
0
Number of occurrences
Location of TGCTGAGTC motifs (nt)

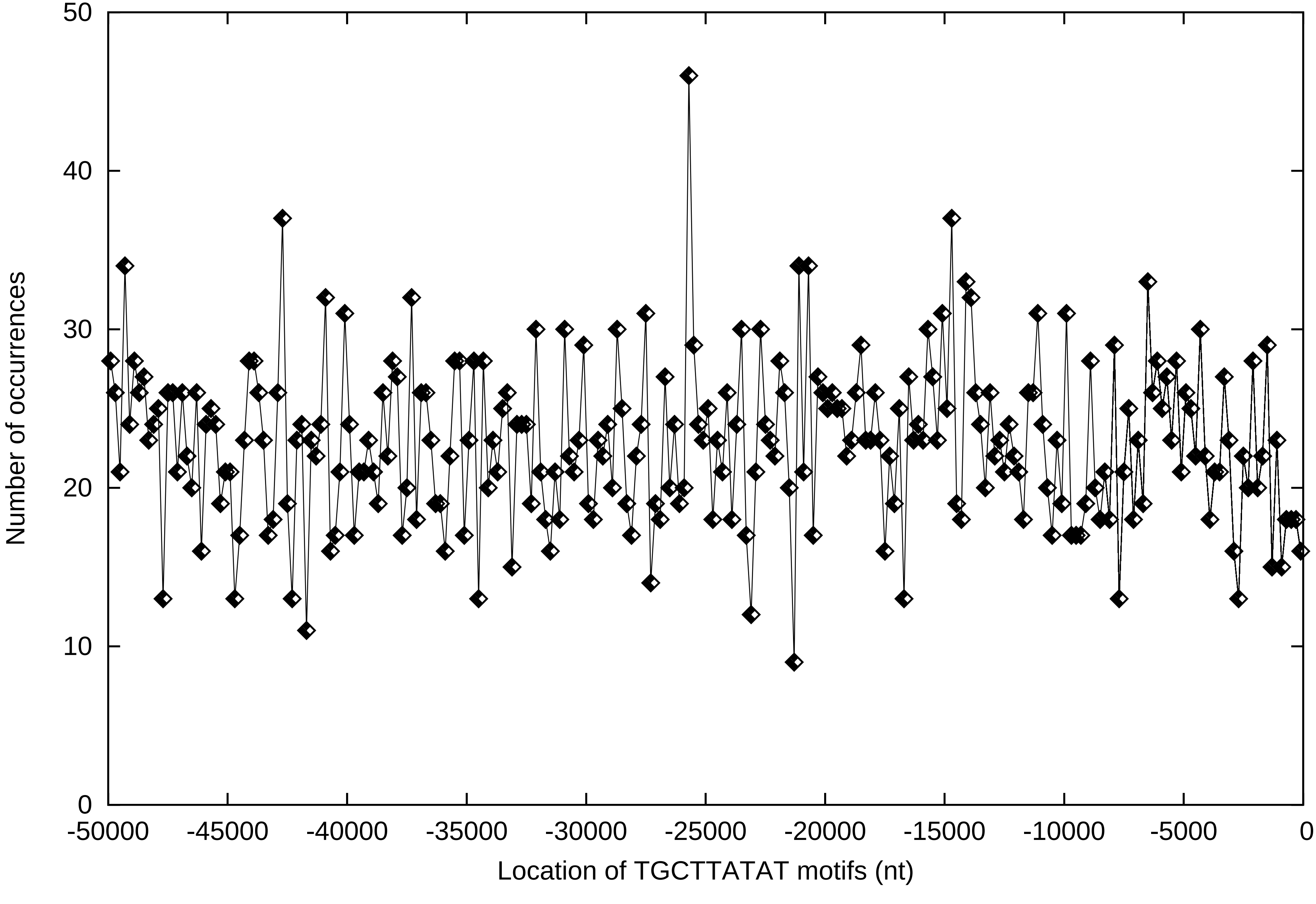

Number of occurrences
50
40
30
20
10
0
-50000
-45000
-40000
-35000
-30000
-25000
-20000
-15000
-10000
-5000
0
Location of TGCTTATAT motifs (nt)

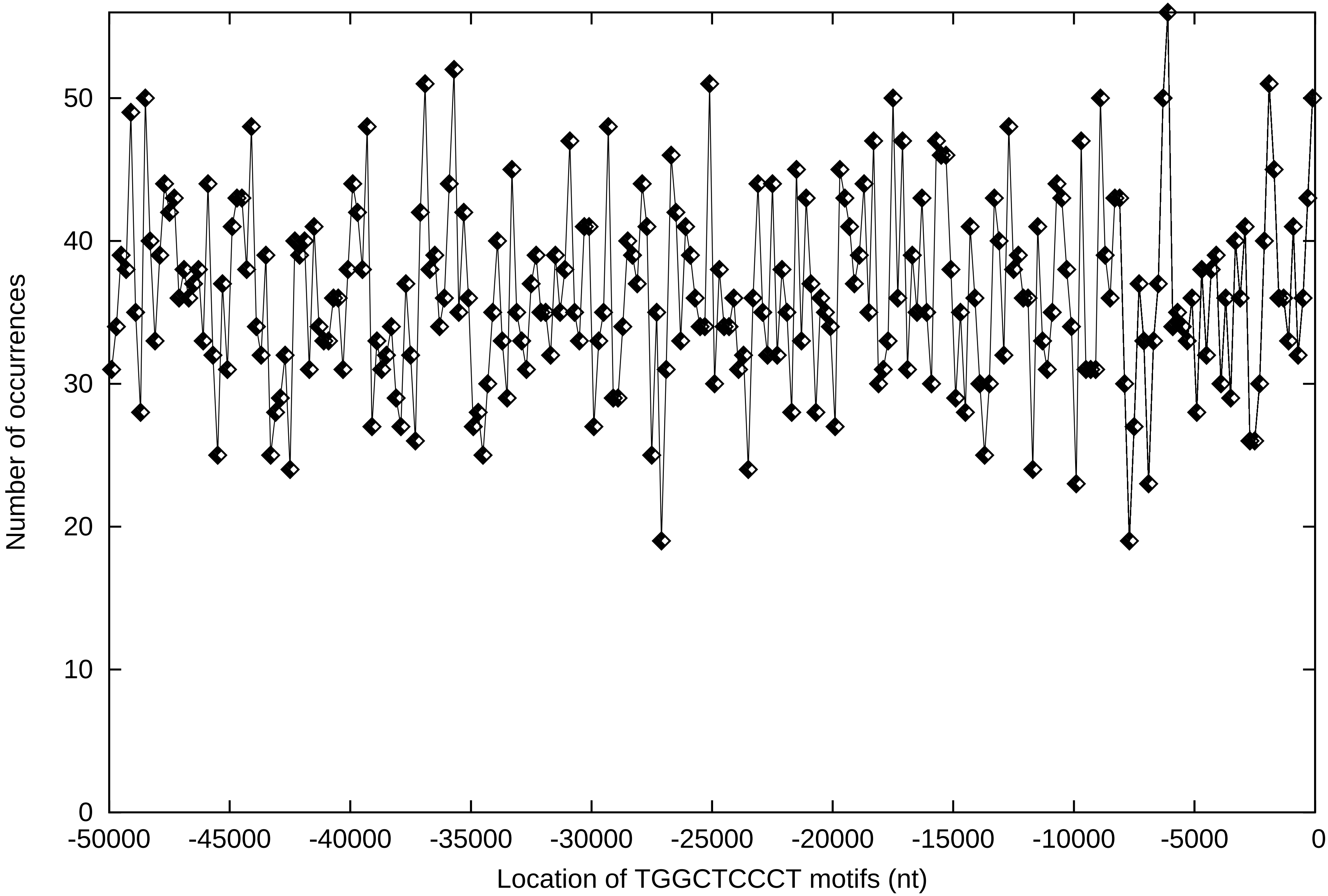

Number of occurrences
0
10
20
30
40
50
-50000
-45000
-40000
-35000
-30000
-25000
-20000
-15000
-10000
-5000
0
Location of TGGCTCCCT motifs (nt)

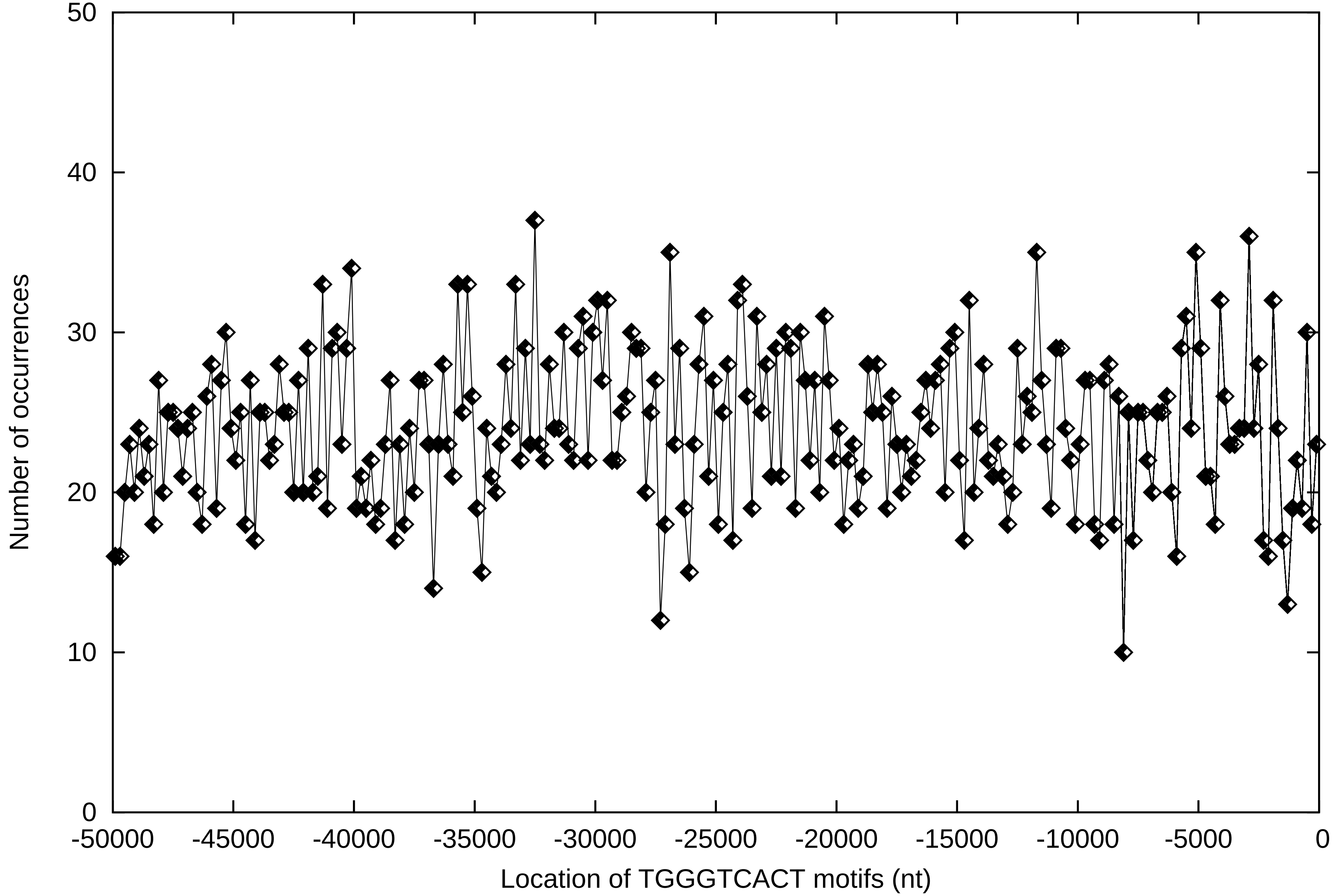
50
40
30
20
10
0
-50000
-45000
-40000
-35000
-30000
-25000
-20000
-15000
-10000
-5000
0
Number of occurrences
Location of TGGGTCACT motifs (nt)

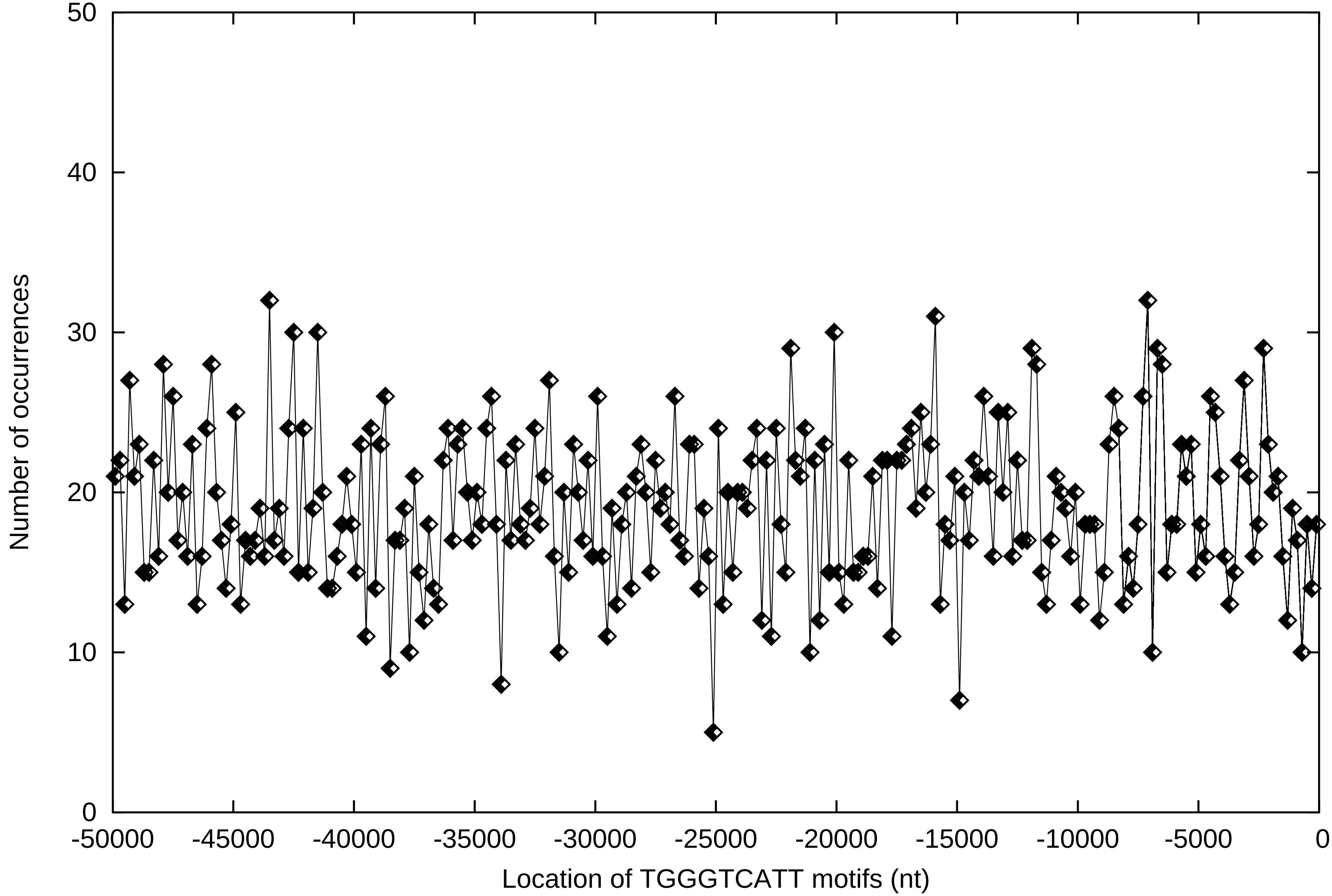

50
40
30
20
10
0
-50000
-45000
-40000
-35000
-30000
-25000
-20000
-15000
-10000
-5000
0
Number of occurrences
Location of TGGGTCATT motifs (nt)

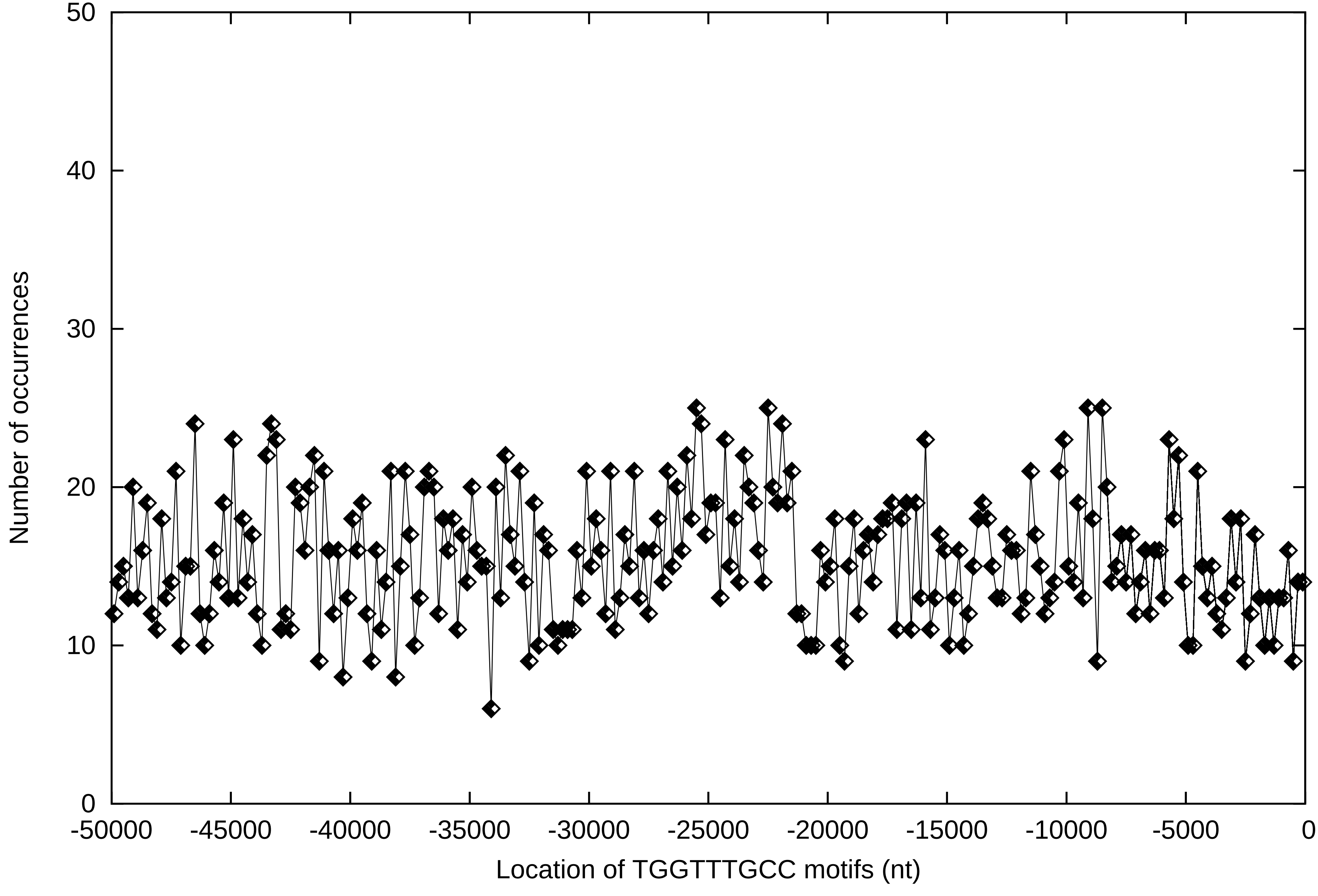

50
40
30
20
10
0
Number of occurrences
-50000
-45000
-40000
-35000
-30000
-25000
-20000
-15000
-10000
-5000
0
Location of TGGTTTGCC motifs (nt)

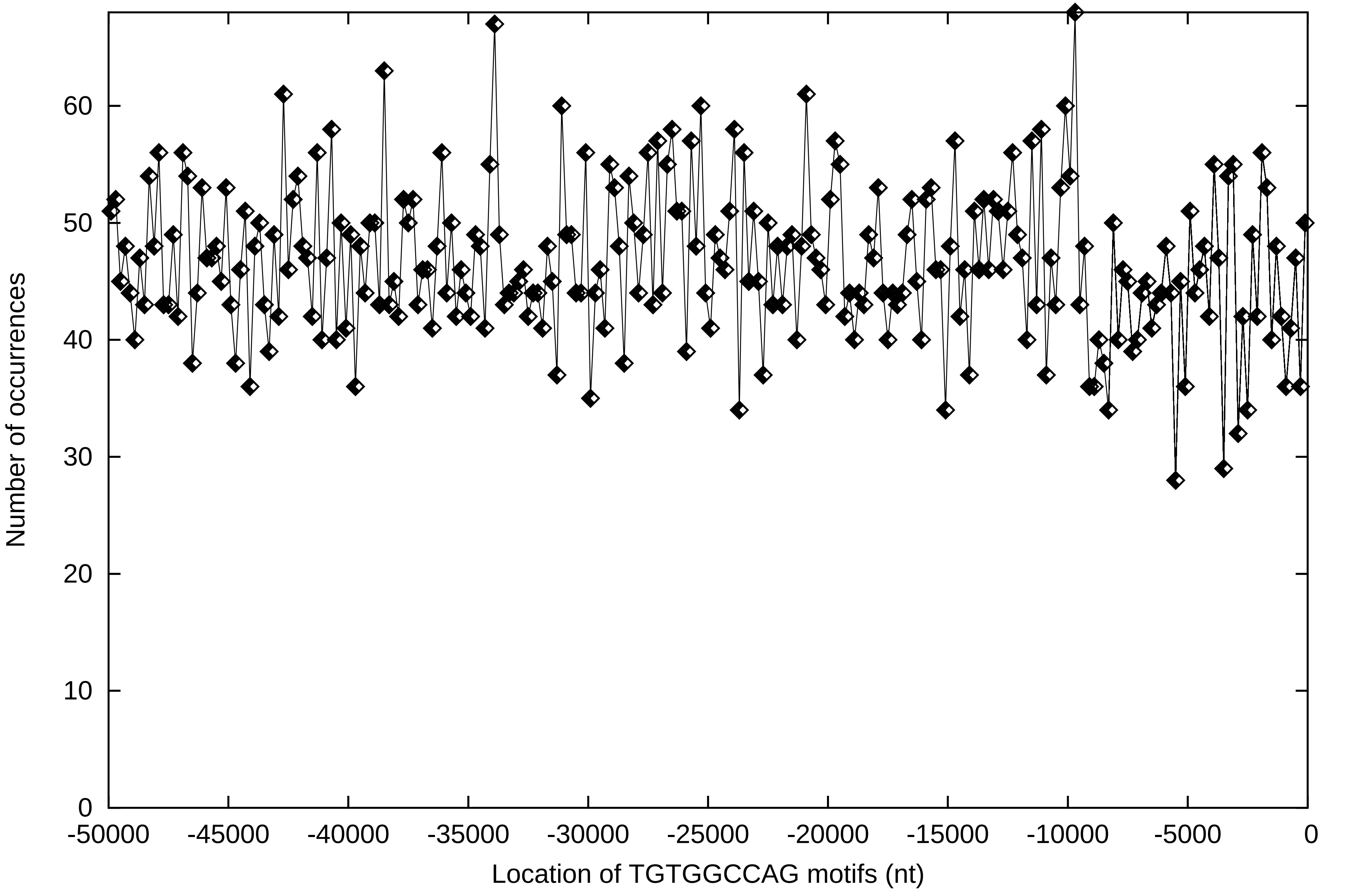
Number of occurrences
Location of TGTGGCCAG motifs (nt)
0
10
20
30
40
50
60
-50000
-45000
-40000
-35000
-30000
-25000
-20000
-15000
-10000
-5000
0

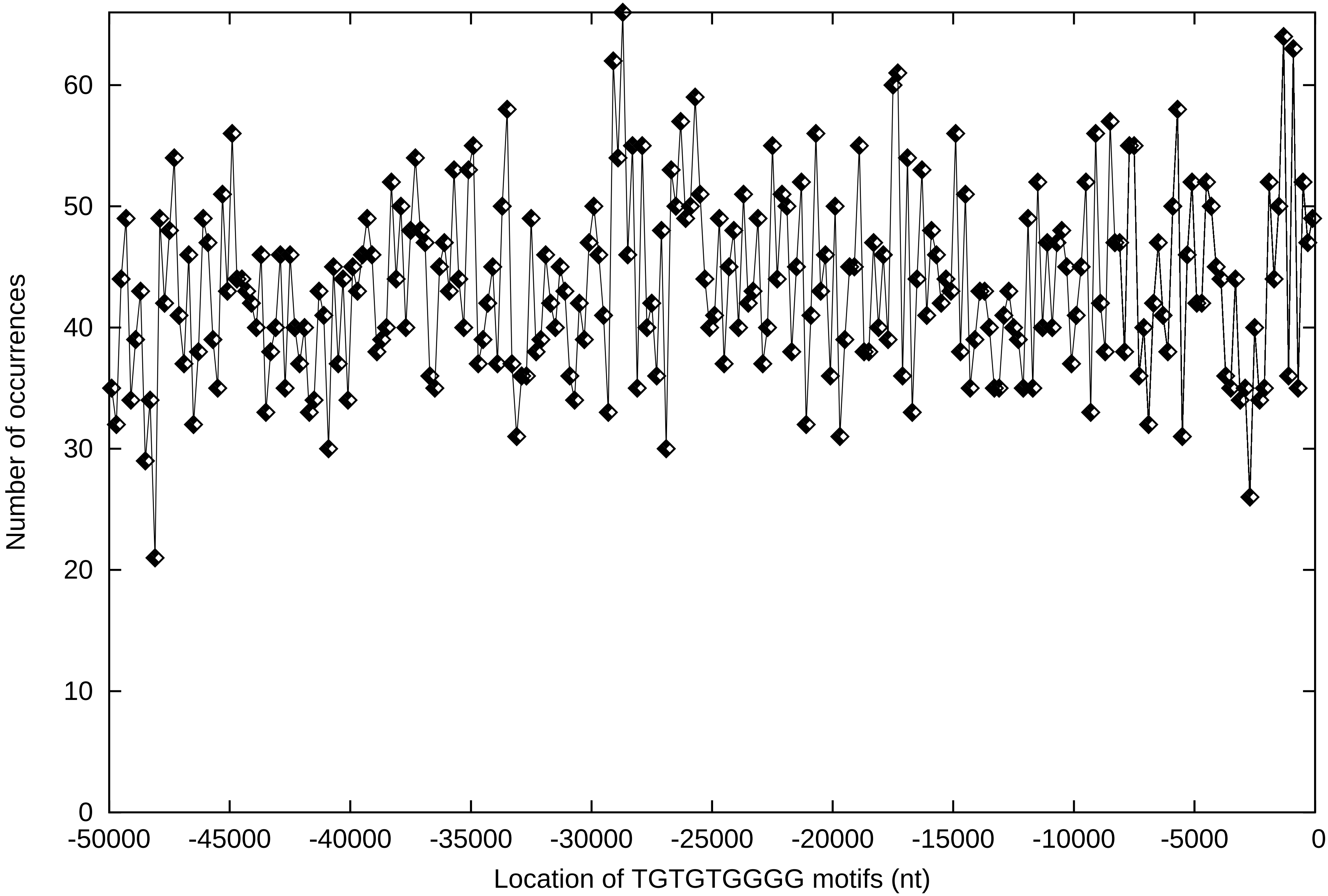
Number of occurrences
0
10
20
30
40
50
60
-50000
-45000
-40000
-35000
-30000
-25000
-20000
-15000
-10000
-5000
0
Location of TGTGTGGGG motifs (nt)

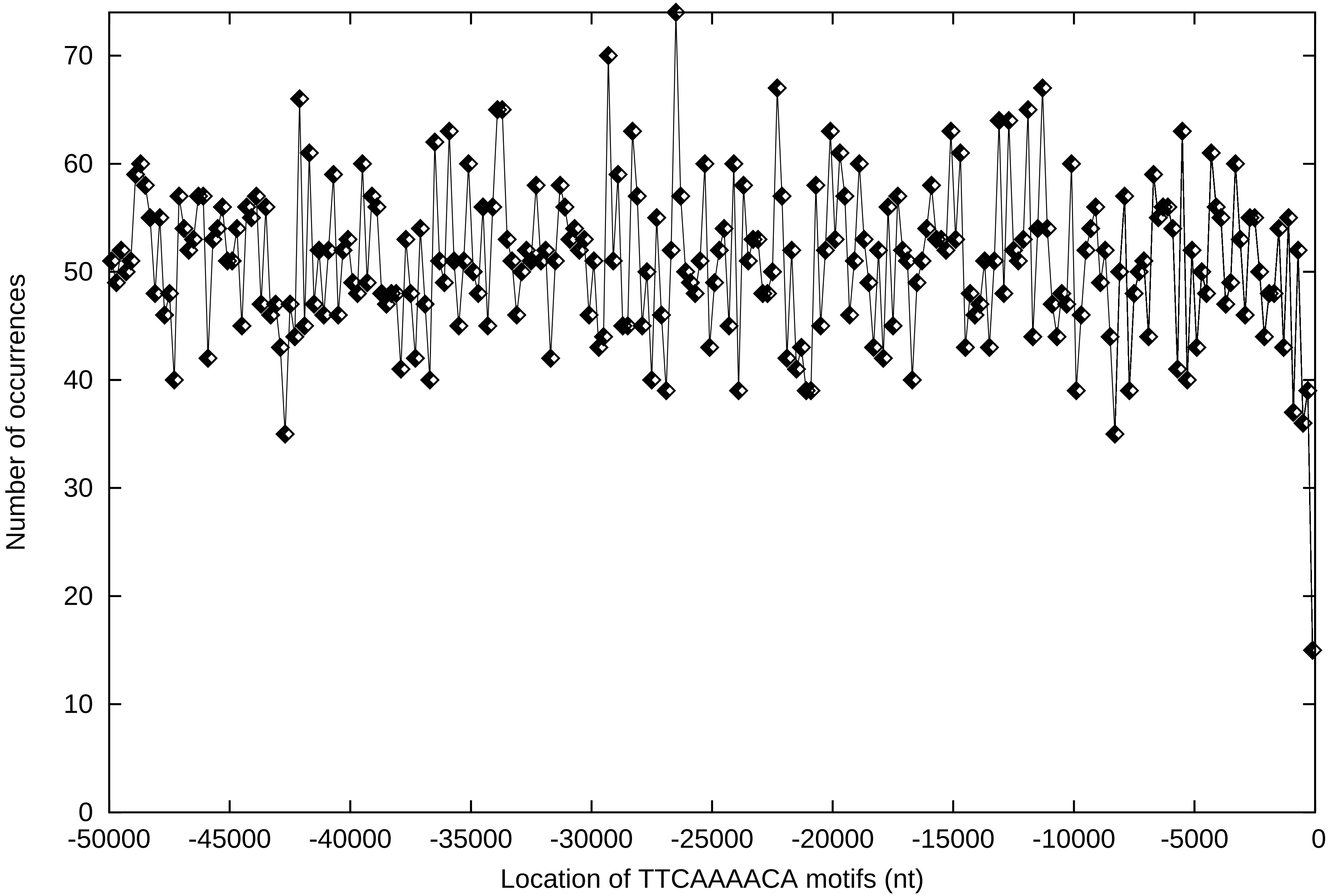

Number of occurrences
0
10
20
30
40
50
60
70
-50000
-45000
-40000
-35000
-30000
-25000
-20000
-15000
-10000
-5000
0
Location of TTCAAAACA motifs (nt)

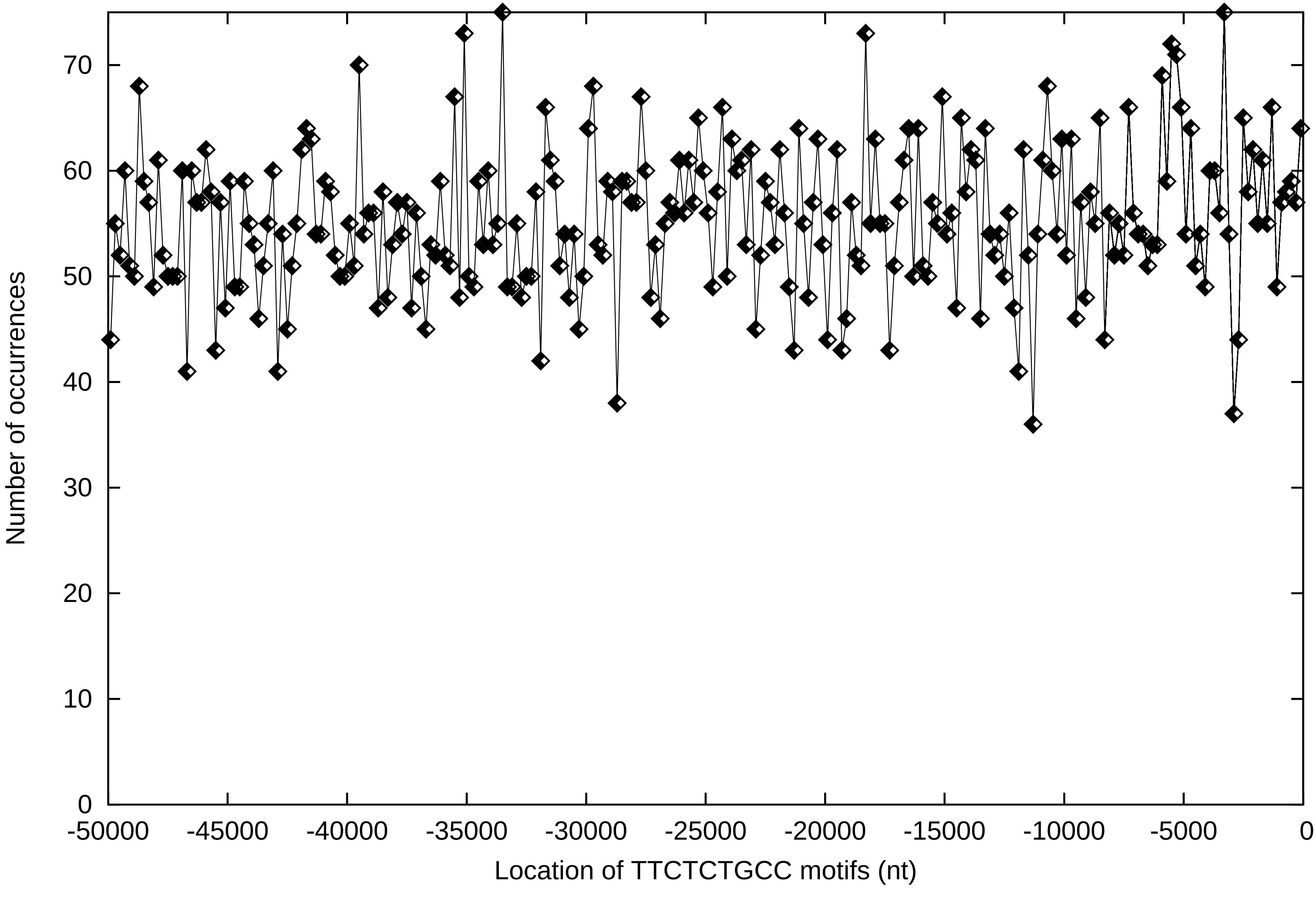
Number of occurrences
0
10
20
30
40
50
60
70
-50000
-45000
-40000
-35000
-30000
-25000
-20000
-15000
-10000
-5000
0
Location of TTCTCTGCC motifs (nt)

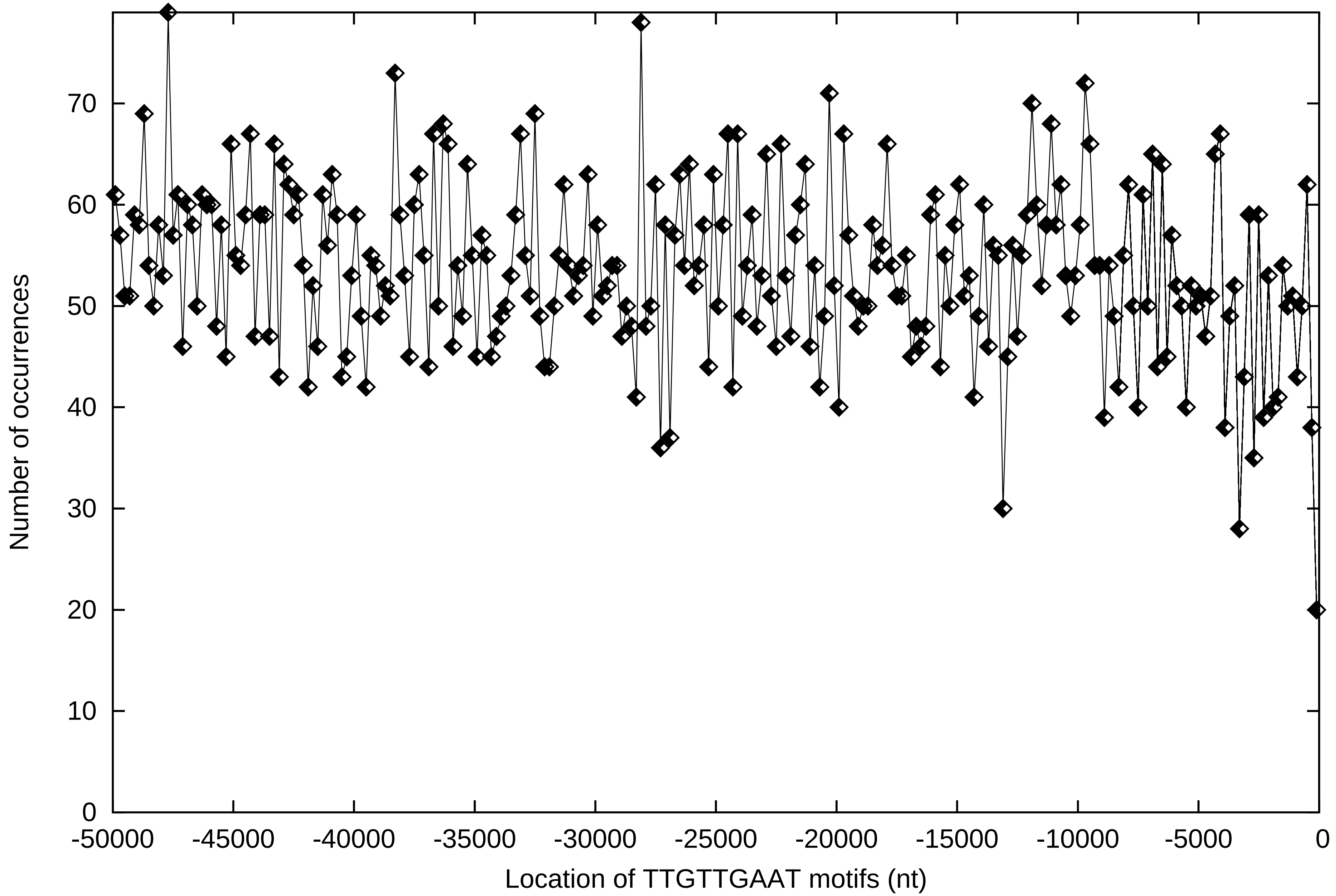

Number of occurrences
0
10
20
30
40
50
60
70
-50000
-45000
-40000
-35000
-30000
-25000
-20000
-15000
-10000
-5000
0
Location of TTGTTGAAT motifs (nt)

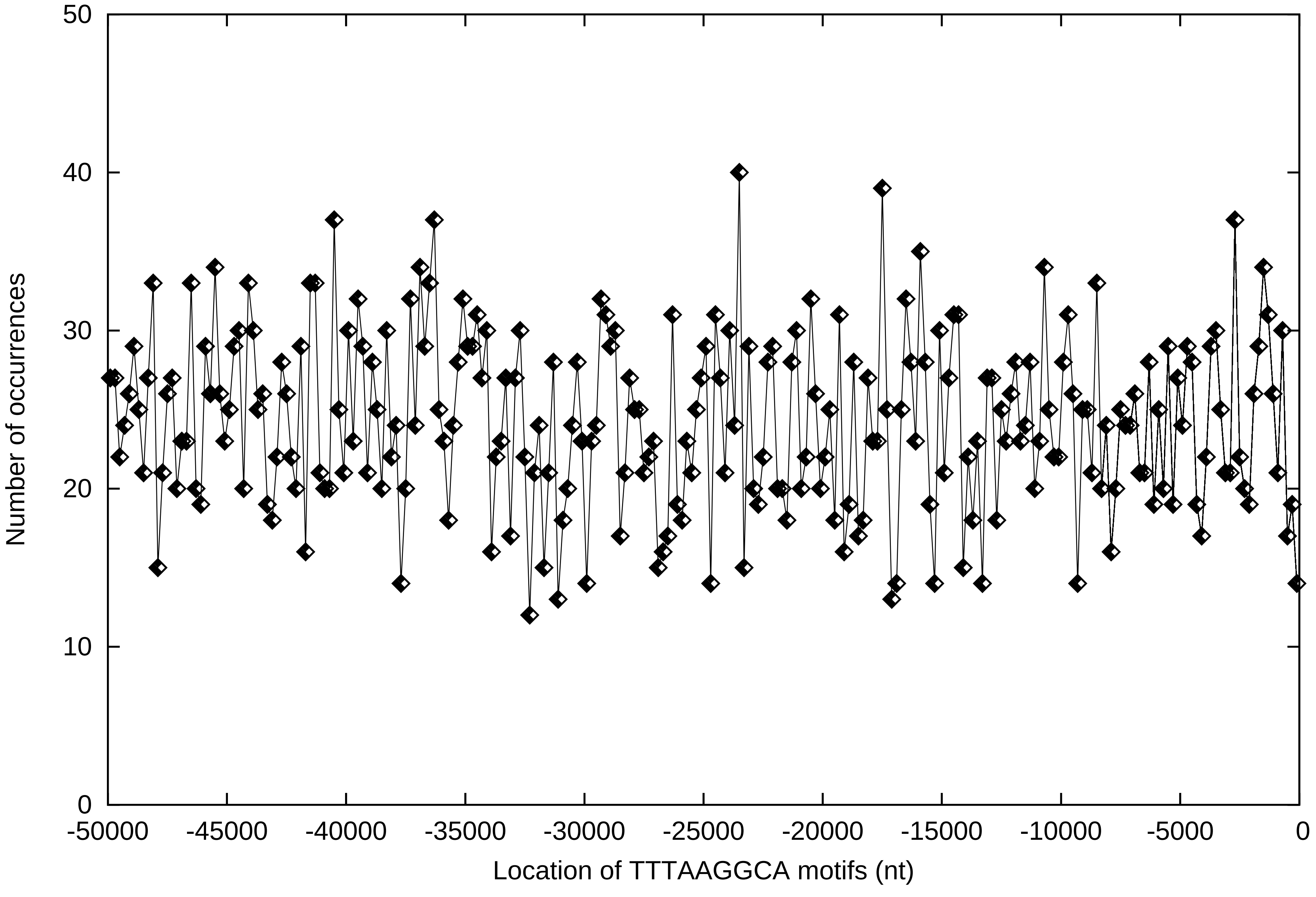
Number of occurrences
Location of TTTAAGGCA motifs (nt)
0
10
20
30
40
50
-50000
-45000
-40000
-35000
-30000
-25000
-20000
-15000
-10000
-5000
0

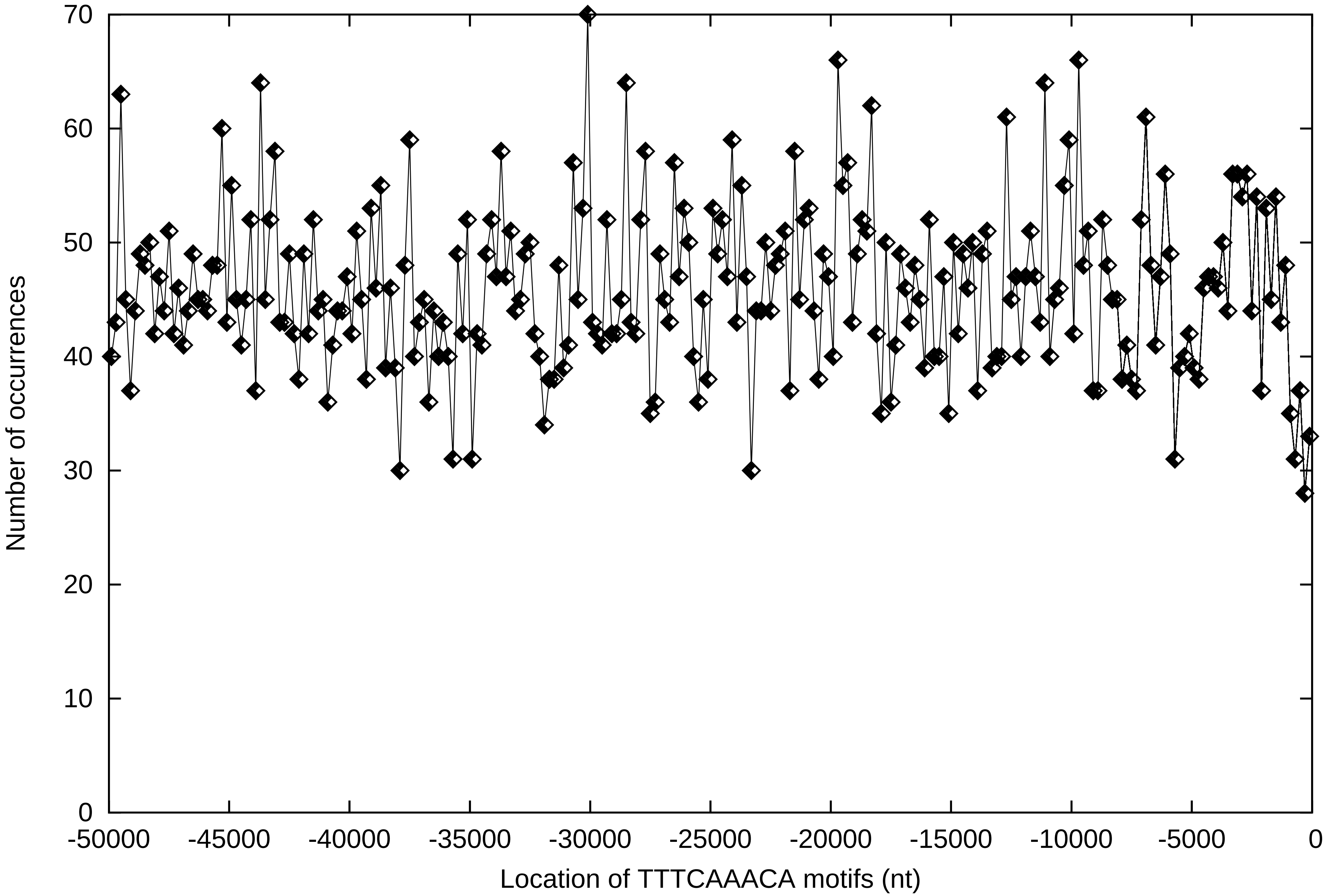

Number of occurrences
Location of TTTCAAACA motifs (nt)
0
10
20
30
40
50
60
70
-50000
-45000
-40000
-35000
-30000
-25000
-20000
-15000
-10000
-5000
0

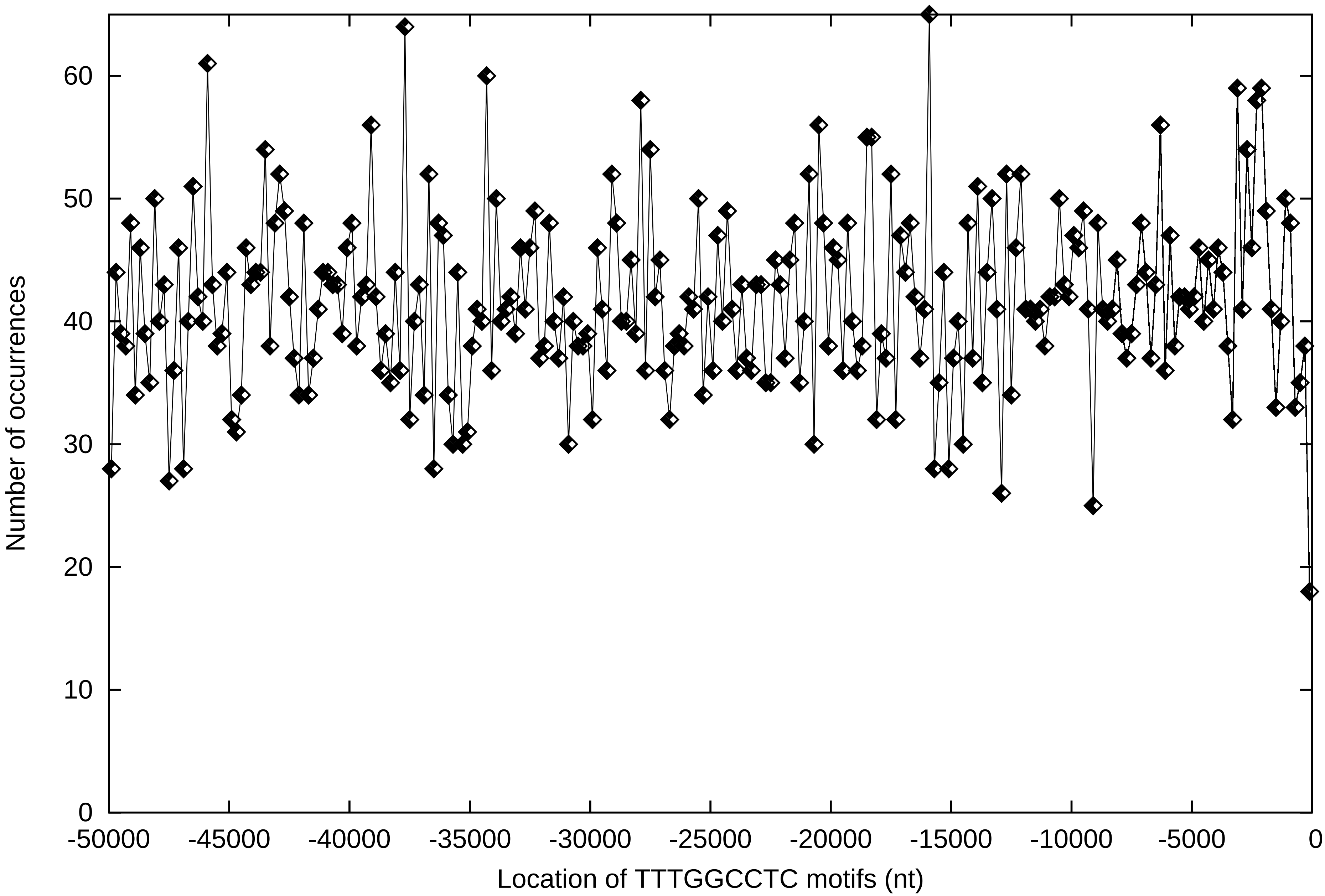
Number of occurrences
Location of TTTGGCCTC motifs (nt)
0
10
20
30
40
50
60
-50000
-45000
-40000
-35000
-30000
-25000
-20000
-15000
-10000
-5000
0

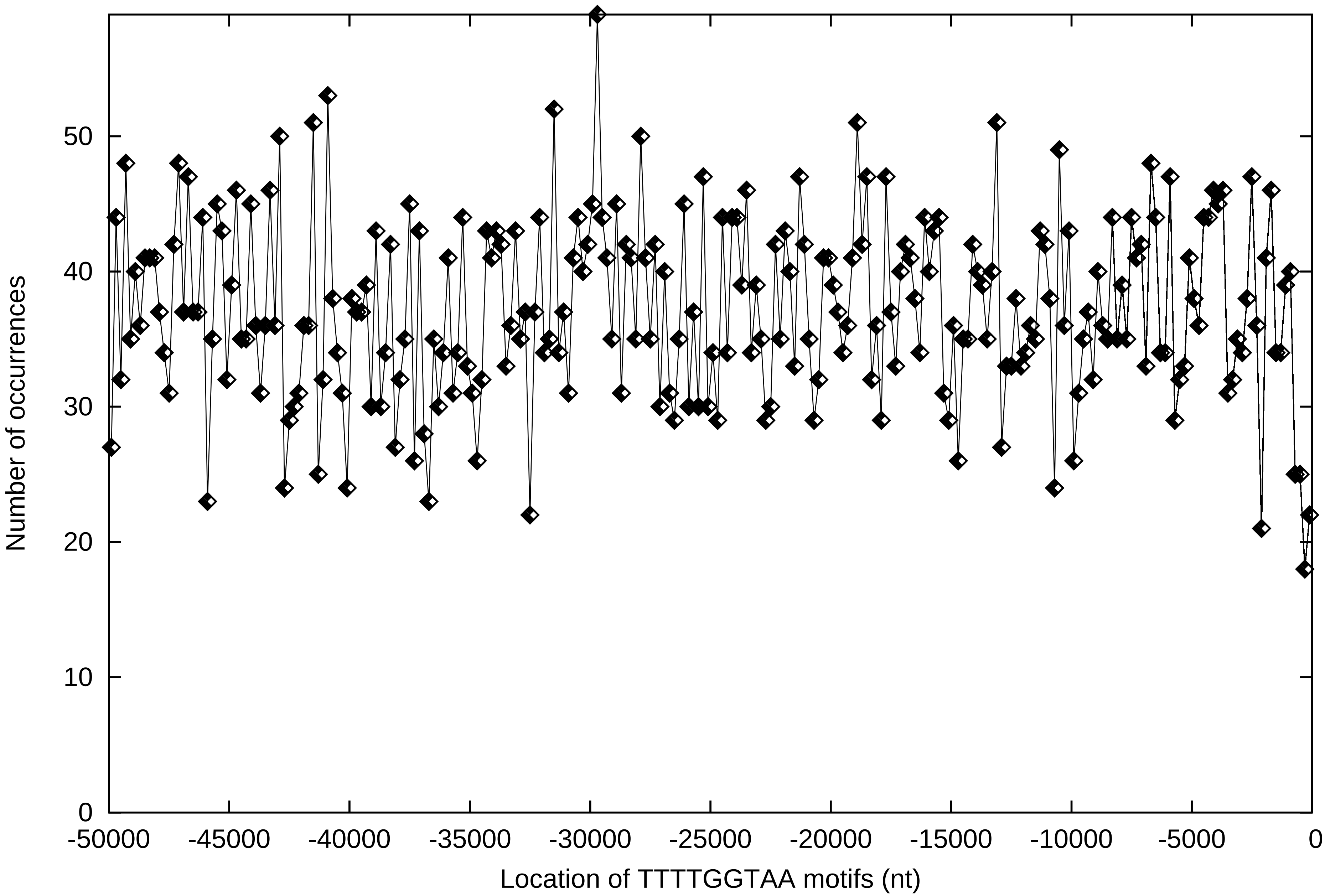
Number of occurrences
Location of TTTTGGTAA motifs (nt)
0
10
20
30
40
50
-50000
-45000
-40000
-35000
-30000
-25000
-20000
-15000
-10000
-5000
0
