# Supplementary material for: Regulatory Circuit of Human MicroRNA Biogenesis
Source: PLoS Comput Biol. 2007 Apr 20;3(4):e67. doi: 10.1371/journal.pcbi.0030067 (PMC1853126; doi:10.1371/journal.pcbi.0030067)
Supplement: Supplementary file 7 [file pcbi.0030067.sg007.pdf]

Distance distribution of randomly selected 8 mers (pages 2-101). Each graph is a histogram that represents the number of occurrences of each motif (every 200 nt stretch) in the first 50 kb upstream region of protein-coding genes. In stark contrast to the top-predicted motifs, the control motifs do not have a strong preference to occur in the neighbor hood of the transcription start sites of protein-coding genes.

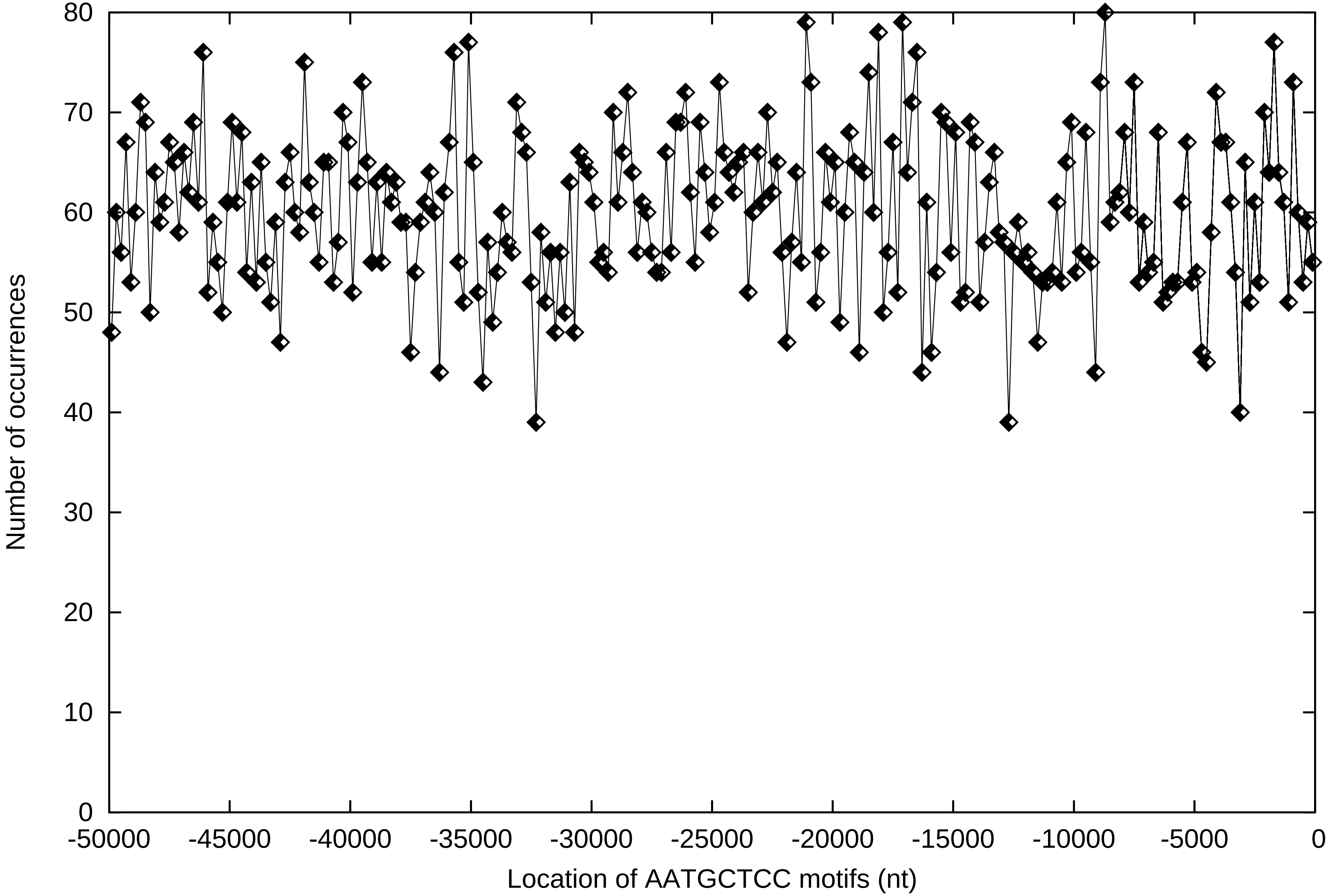

Number of occurrences
80
70
60
50
40
30
20
10
0
-50000
-45000
-40000
-35000
-30000
-25000
-20000
-15000
-10000
-5000
0
Location of AATGCTCC motifs (nt)

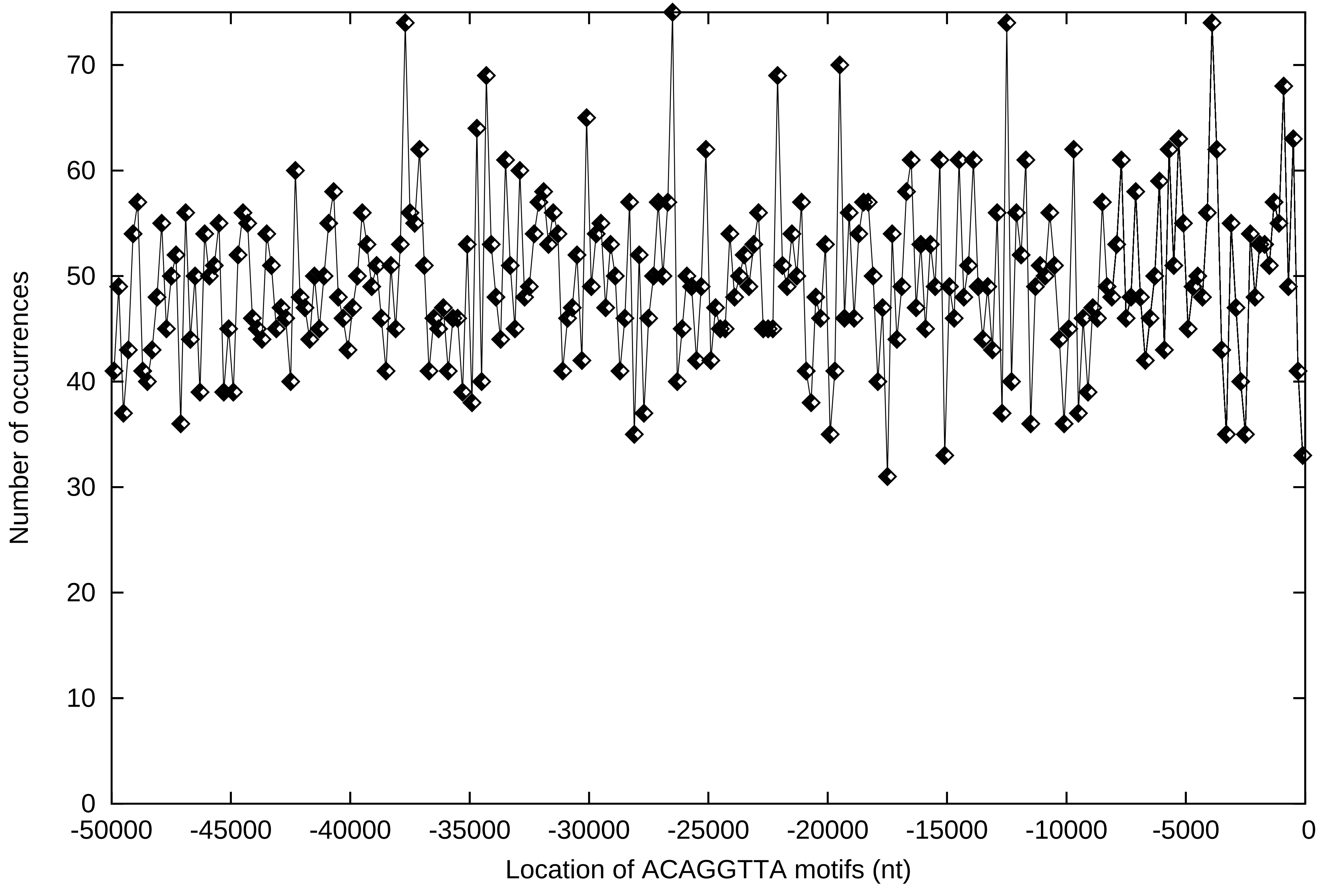
Number of occurrences
Location of ACAGGTTA motifs (nt)
0
10
20
30
40
50
60
70
-50000
-45000
-40000
-35000
-30000
-25000
-20000
-15000
-10000
-5000
0

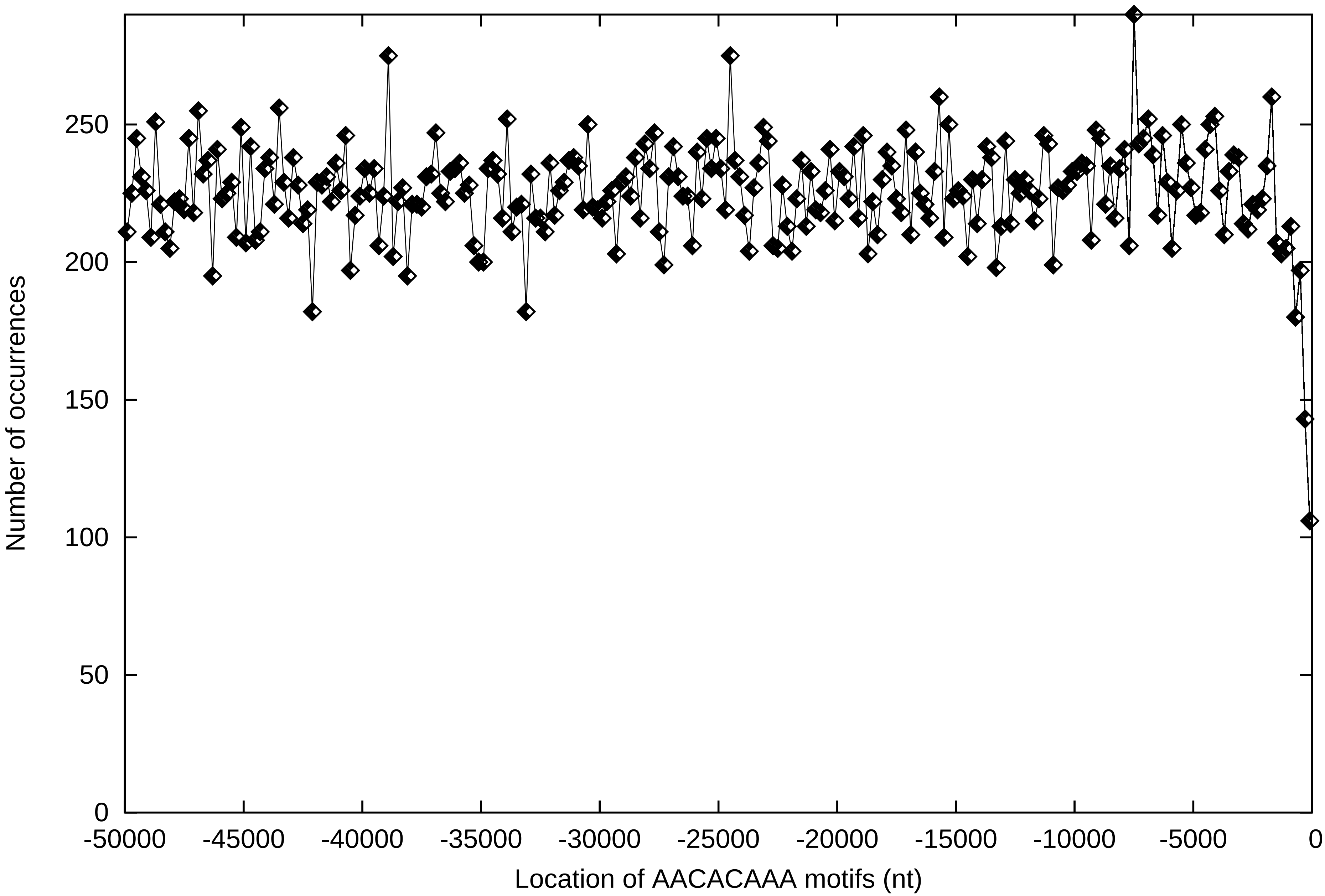

Number of occurrences
0
50
100
150
200
250
-50000
-45000
-40000
-35000
-30000
-25000
-20000
-15000
-10000
-5000
0
Location of AACACAAA motifs (nt)

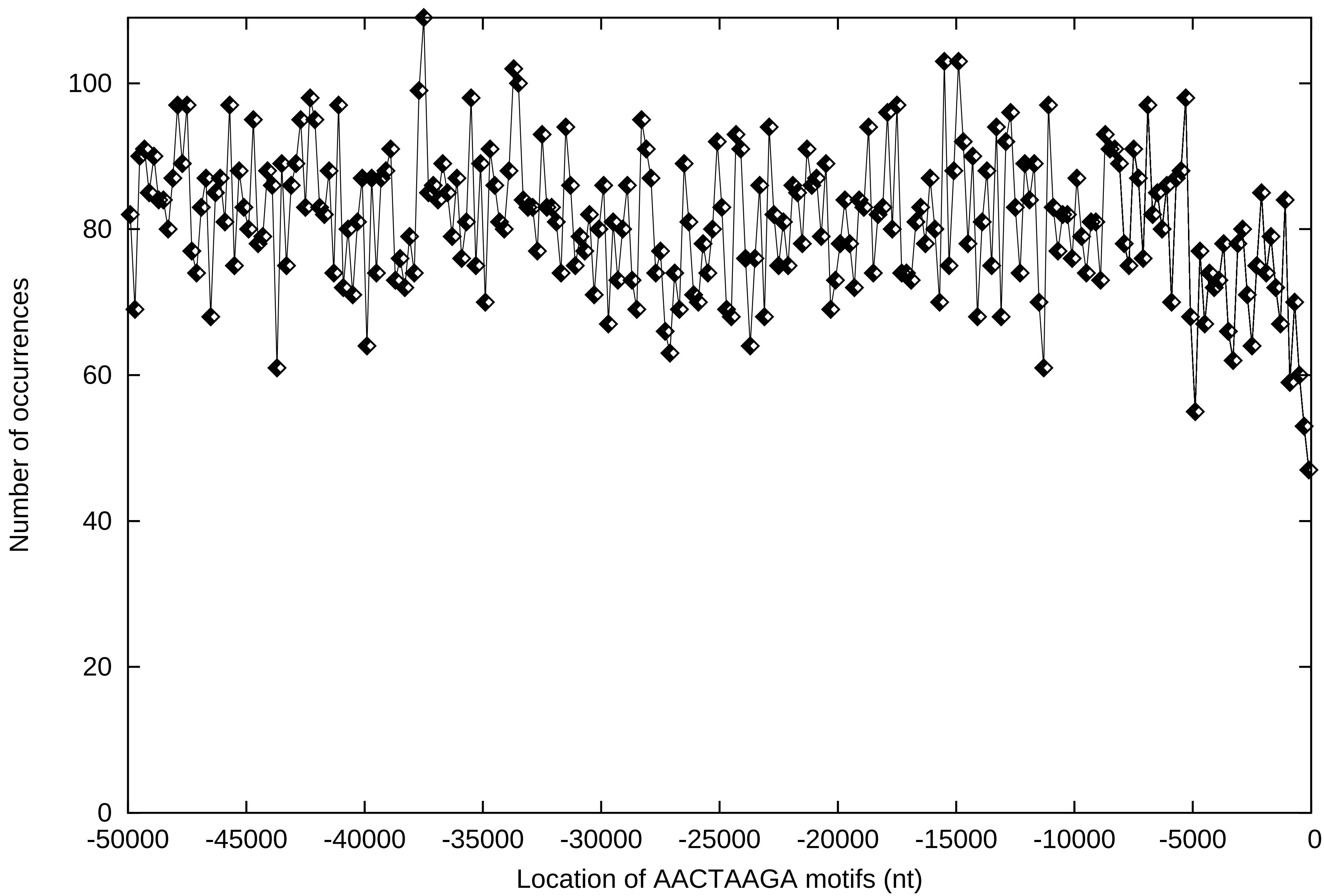

Number of occurrences
Location of AACTAAGA motifs (nt)
0
20
40
60
80
100
-50000
-45000
-40000
-35000
-30000
-25000
-20000
-15000
-10000
-5000
0

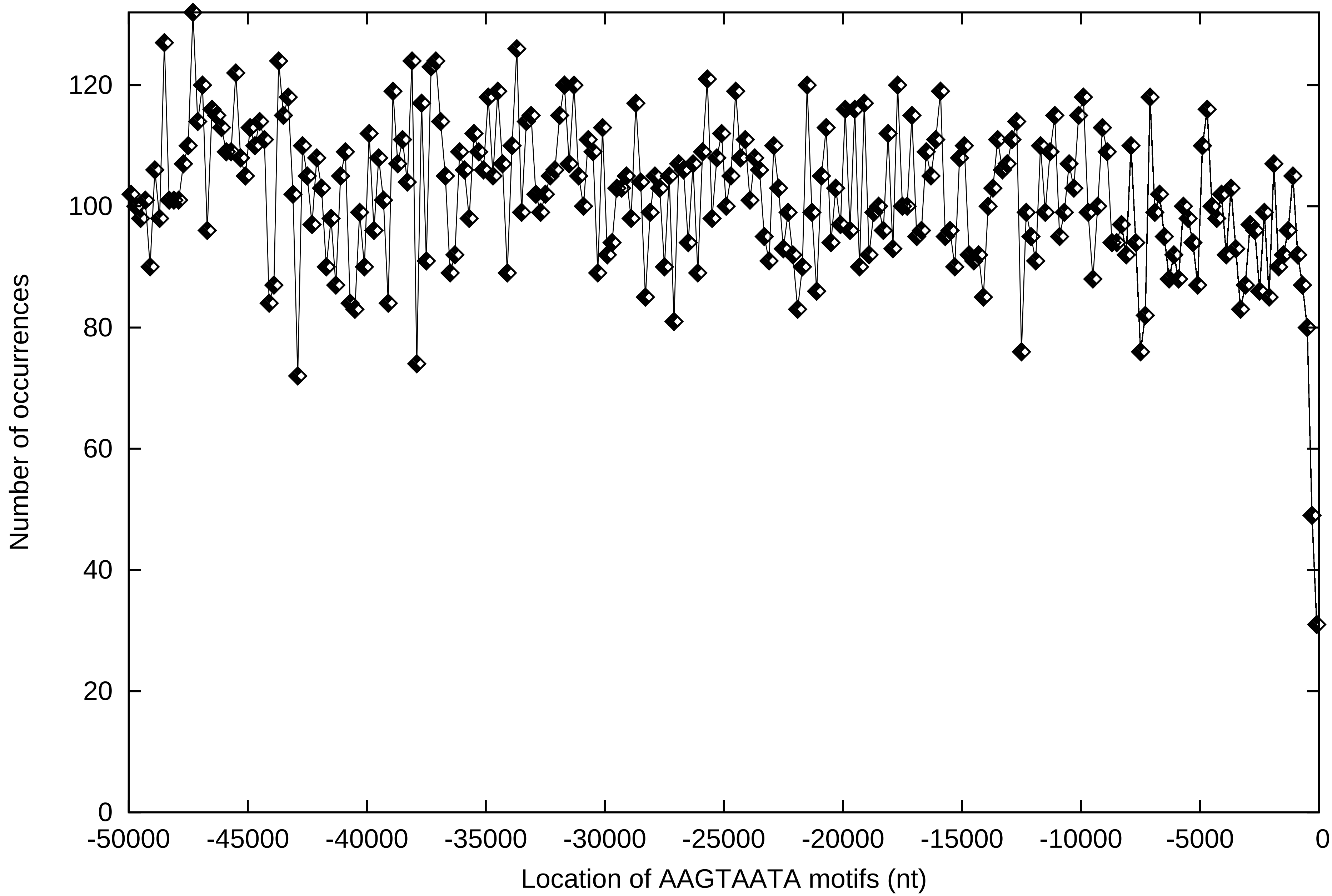

0
20
40
60
80
100
120
-50000
-45000
-40000
-35000
-30000
-25000
-20000
-15000
-10000
-5000
0
Number of occurrences
Location of AAGTAATA motifs (nt)

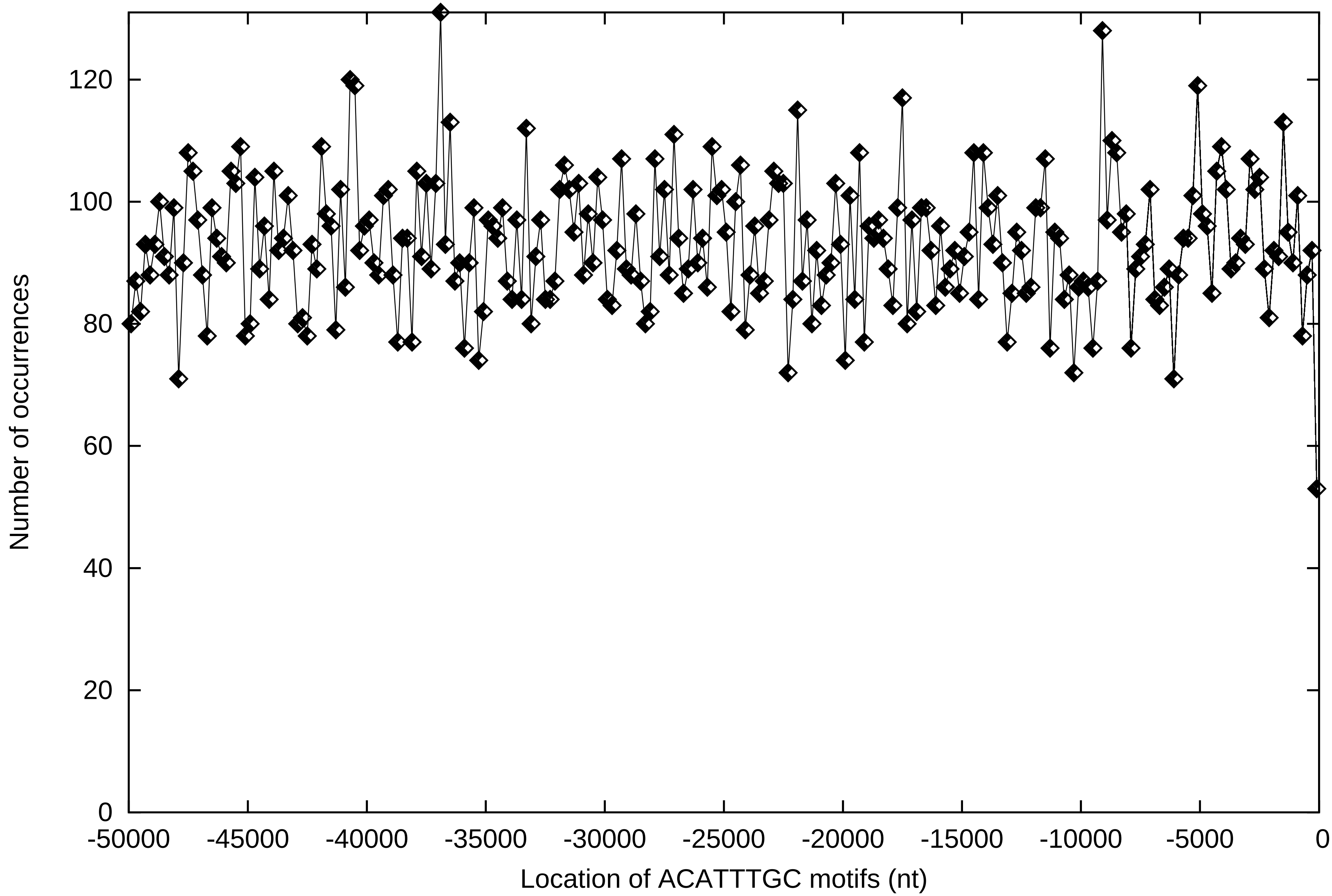

Number of occurrences
Location of ACATTTGC motifs (nt)
0
20
40
60
80
100
120
-50000
-45000
-40000
-35000
-30000
-25000
-20000
-15000
-10000
-5000
0

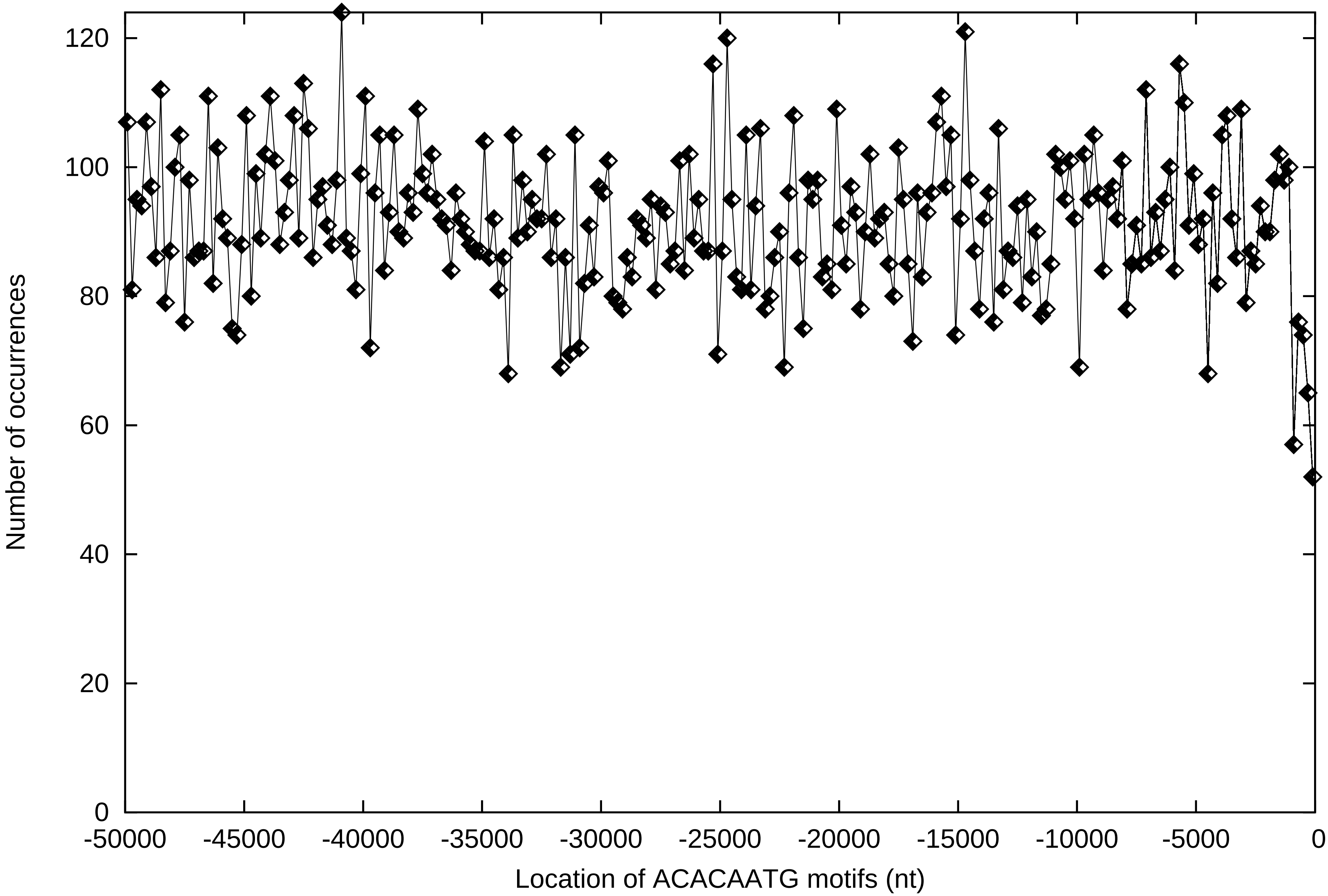

Number of occurrences
Location of ACACAATG motifs (nt)
0
20
40
60
80
100
120
-50000
-45000
-40000
-35000
-30000
-25000
-20000
-15000
-10000
-5000
0

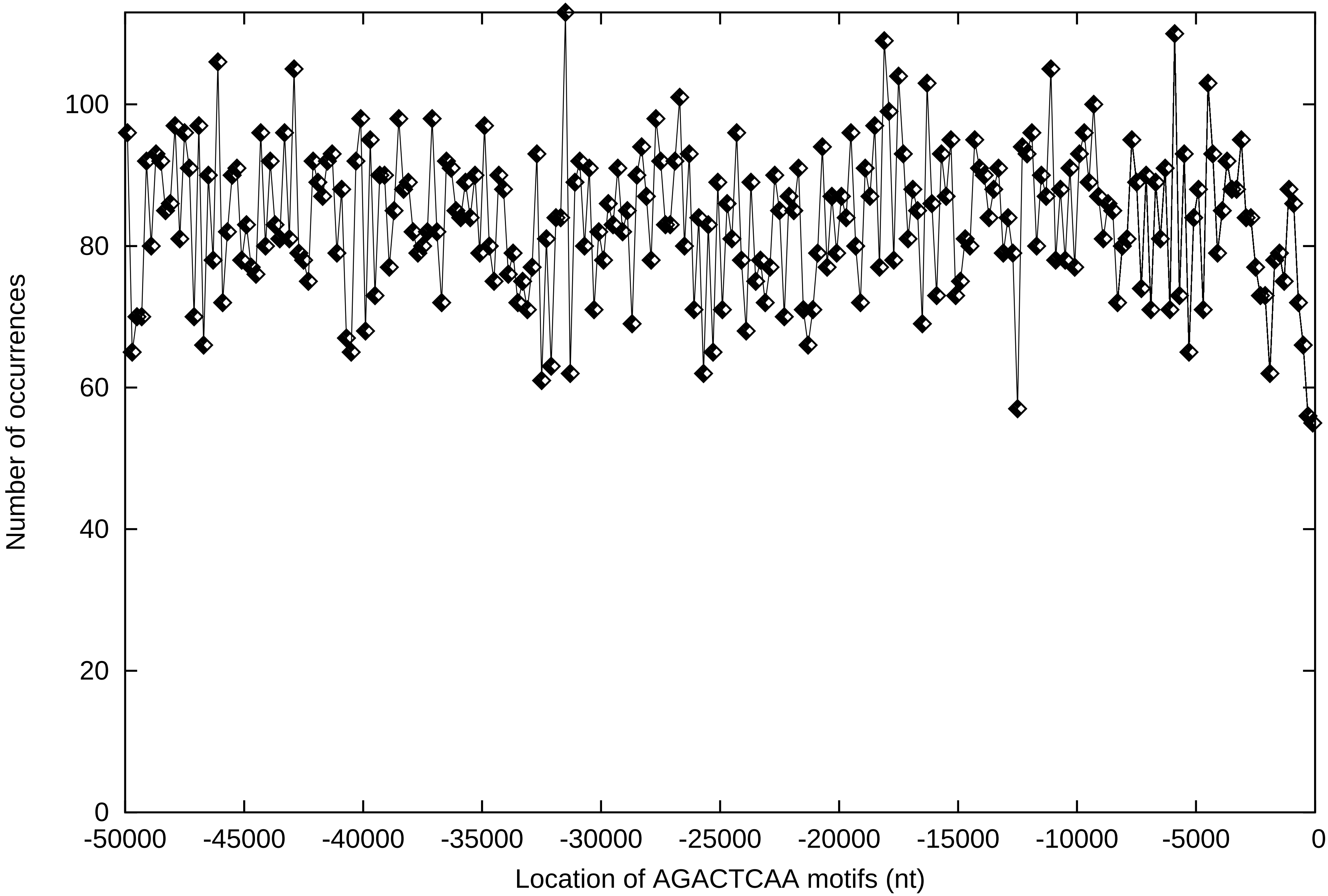

Number of occurrences
0
20
40
60
80
100
-50000
-45000
-40000
-35000
-30000
-25000
-20000
-15000
-10000
-5000
0
Location of AGACTCAA motifs (nt)

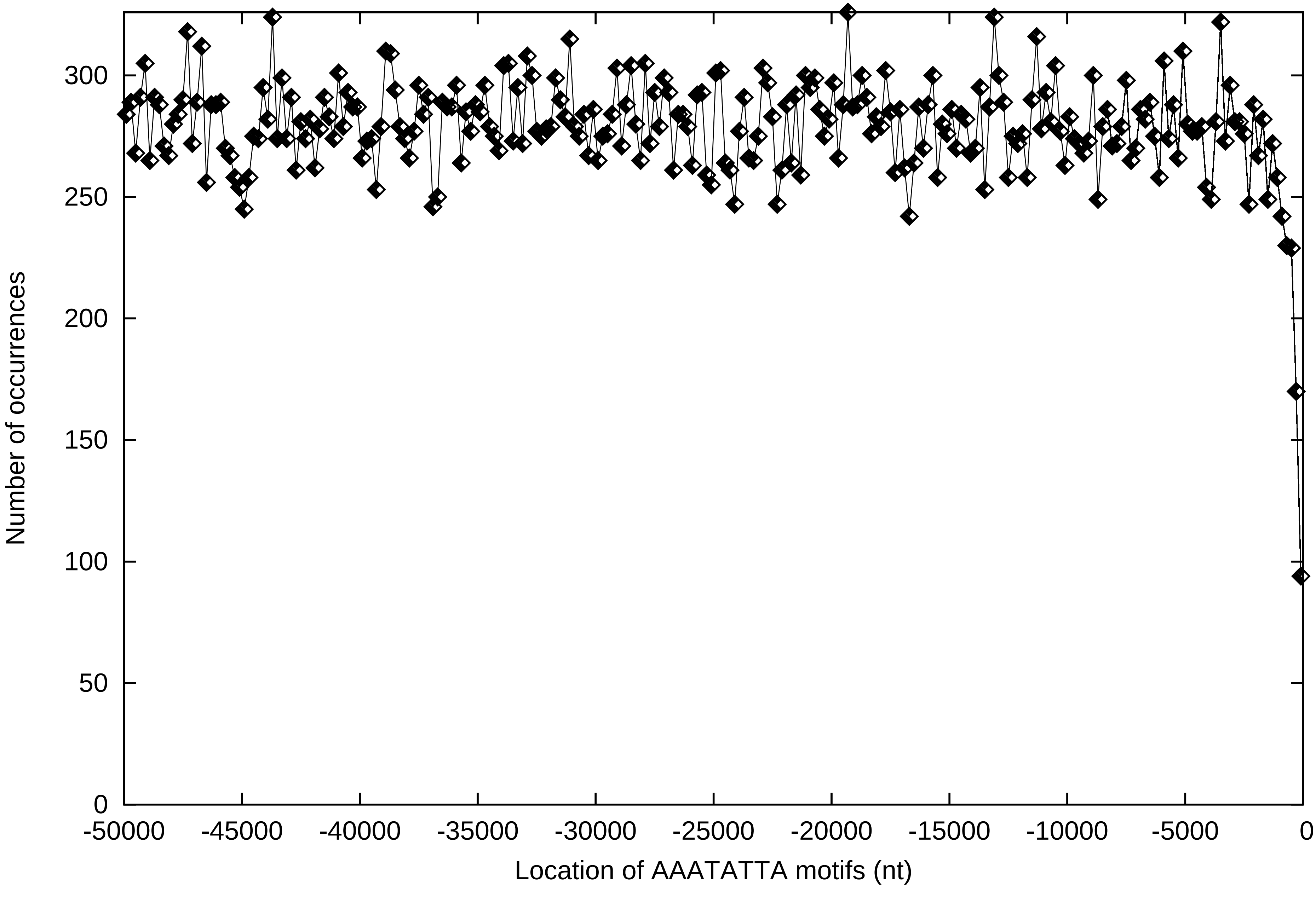

Number of occurrences
0
50
100
150
200
250
300
-50000
-45000
-40000
-35000
-30000
-25000
-20000
-15000
-10000
-5000
0
Location of AAATATTA motifs (nt)

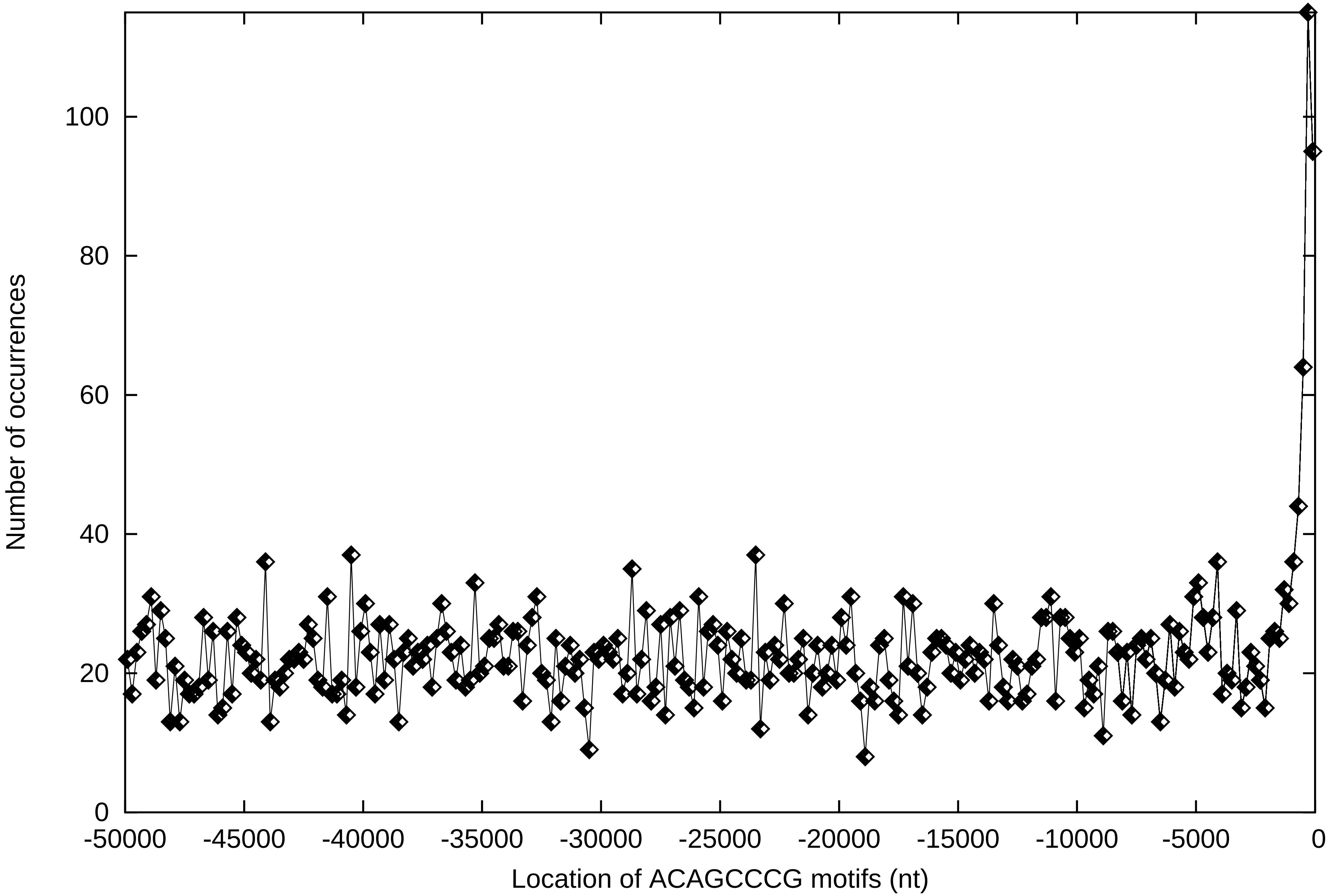

Number of occurrences
Location of ACAGCCCG motifs (nt)
0
20
40
60
80
100
-50000
-45000
-40000
-35000
-30000
-25000
-20000
-15000
-10000
-5000
0

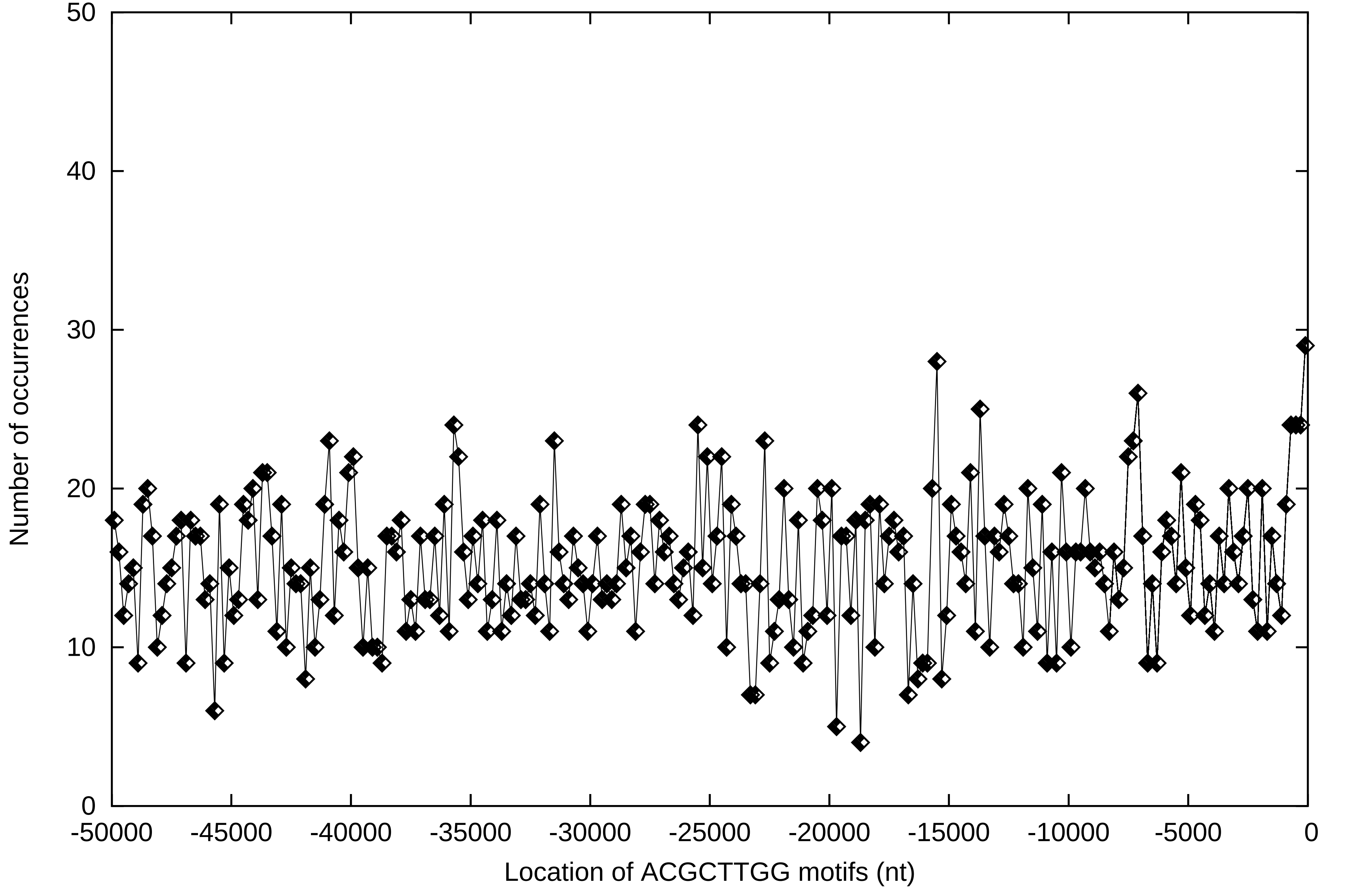

Number of occurrences
0
10
20
30
40
50
-50000
-45000
-40000
-35000
-30000
-25000
-20000
-15000
-10000
-5000
0
Location of ACGCTTGG motifs (nt)

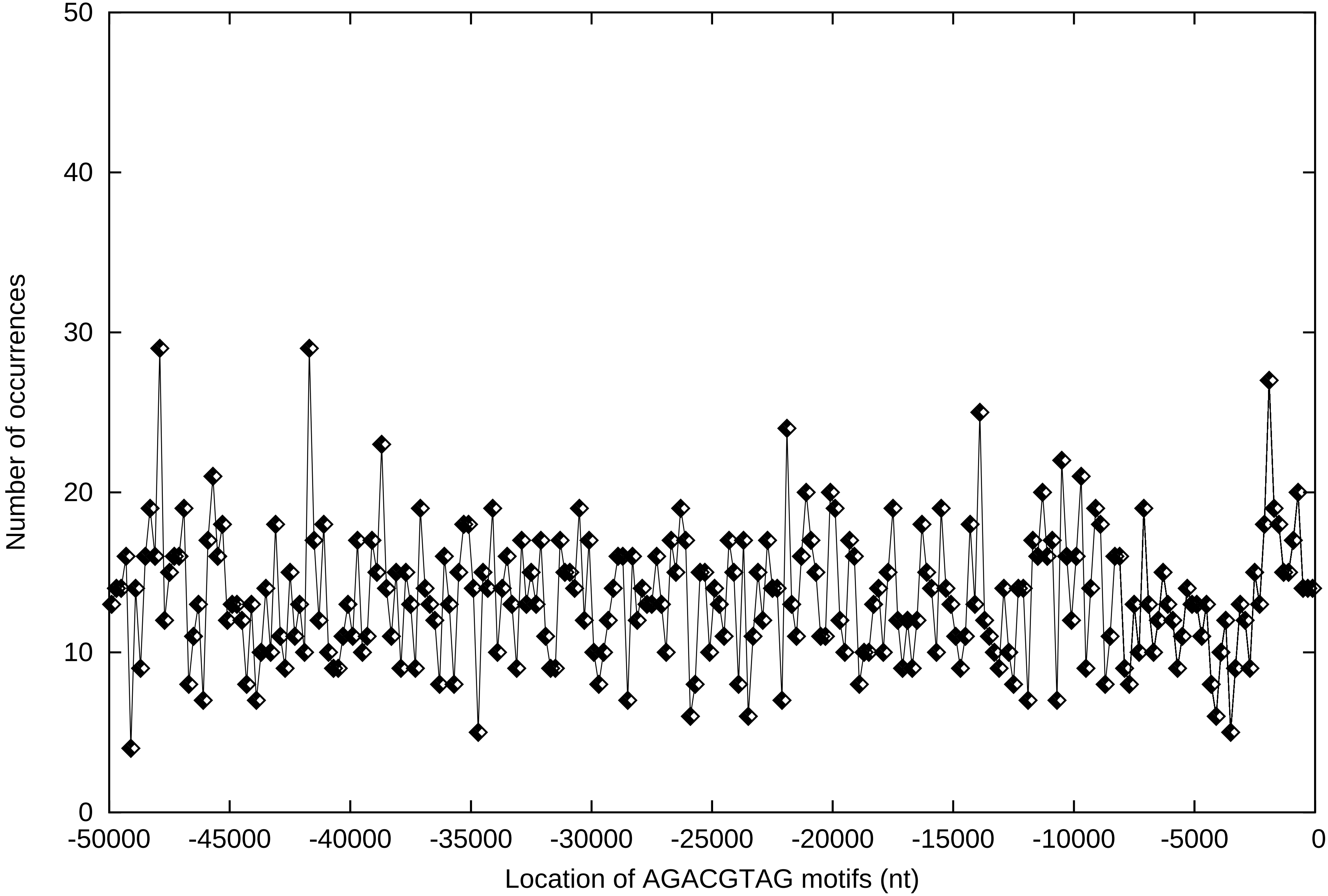

Number of occurrences
Location of AGACGTAG motifs (nt)
0
10
20
30
40
50
-50000
-45000
-40000
-35000
-30000
-25000
-20000
-15000
-10000
-5000
0

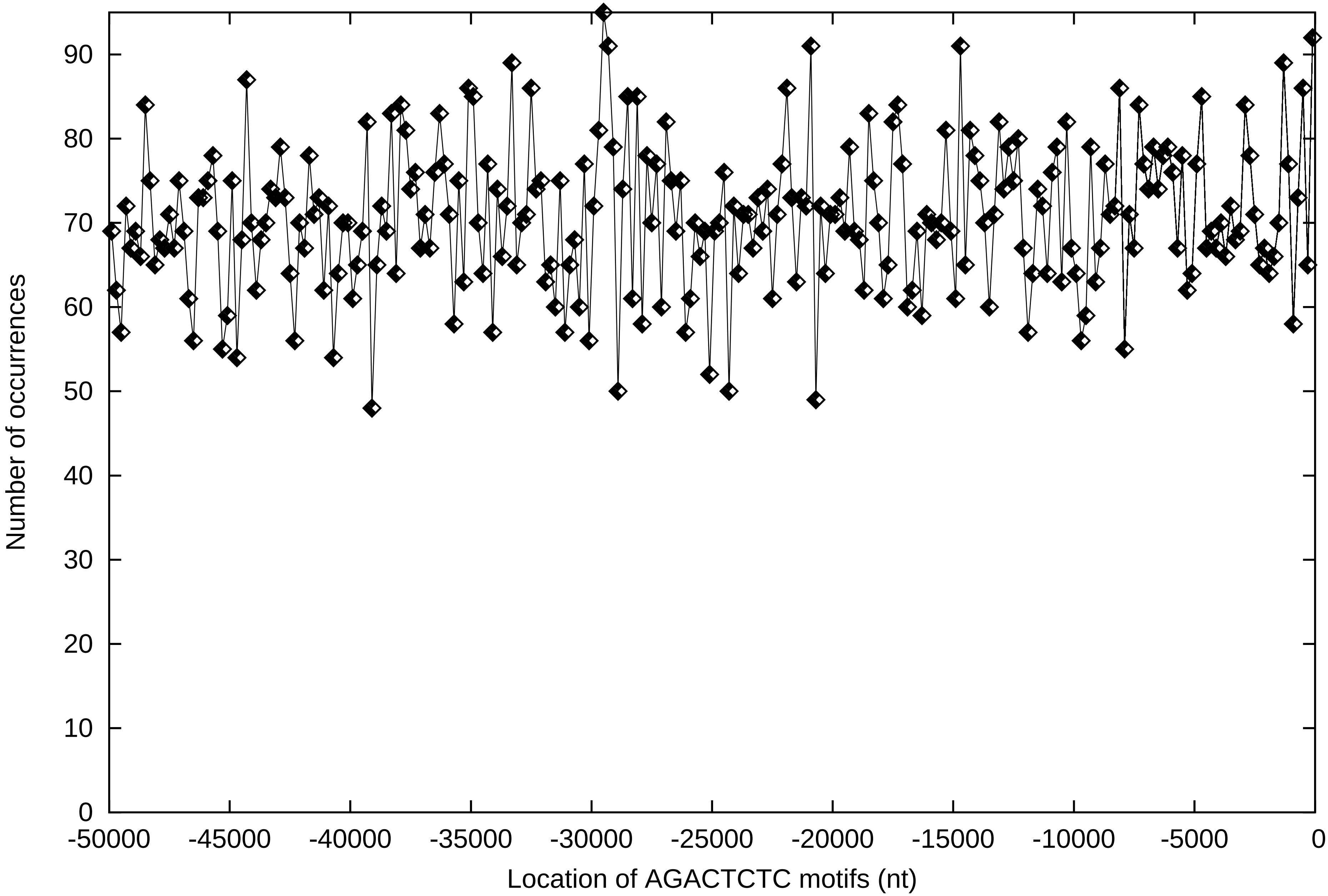

Number of occurrences
0
10
20
30
40
50
60
70
80
90
-50000
-45000
-40000
-35000
-30000
-25000
-20000
-15000
-10000
-5000
0
Location of AGACTCTC motifs (nt)

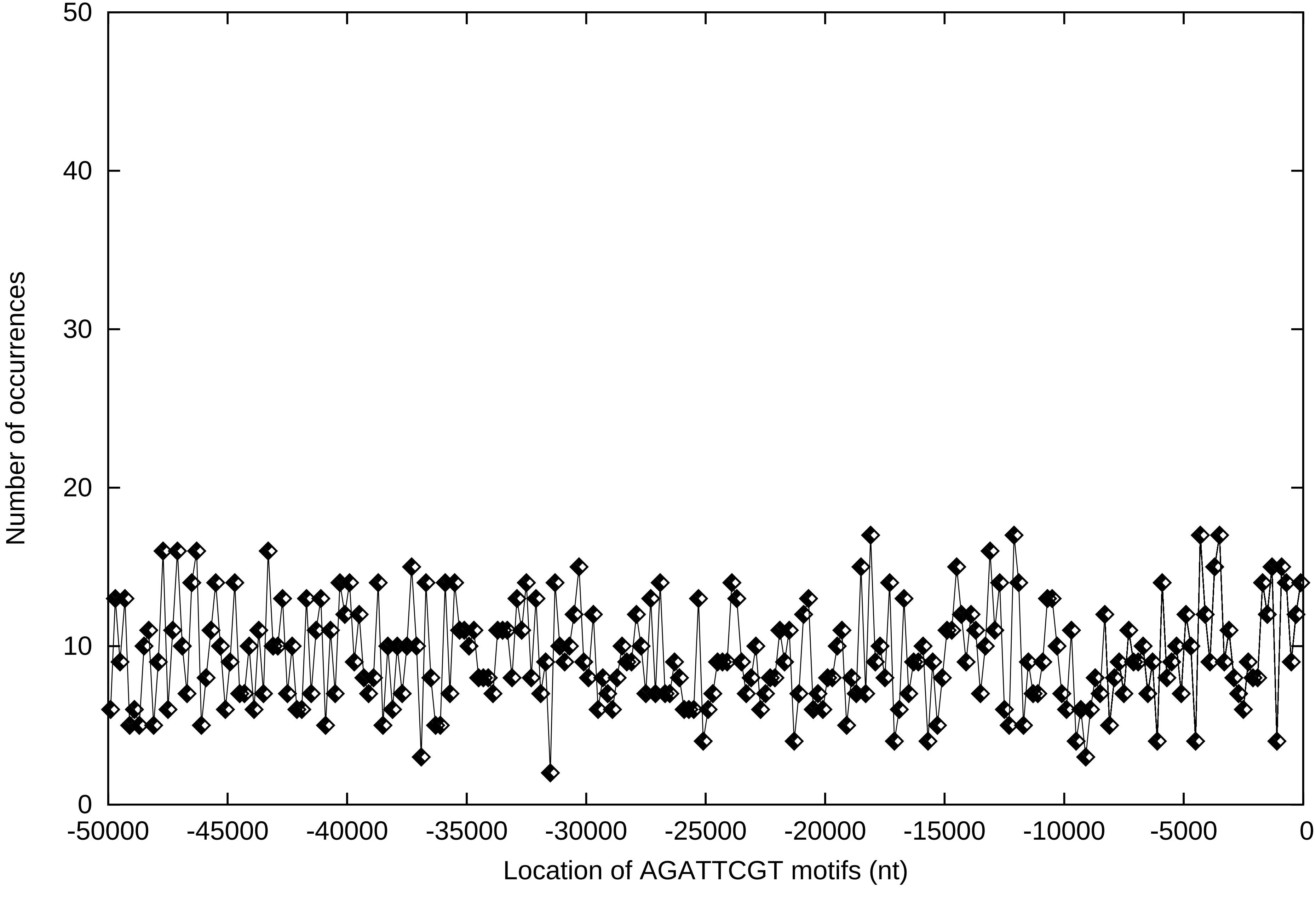

50
40
30
20
10
0
-50000
-45000
-40000
-35000
-30000
-25000
-20000
-15000
-10000
-5000
0
Number of occurrences
Location of AGATTCGT motifs (nt)

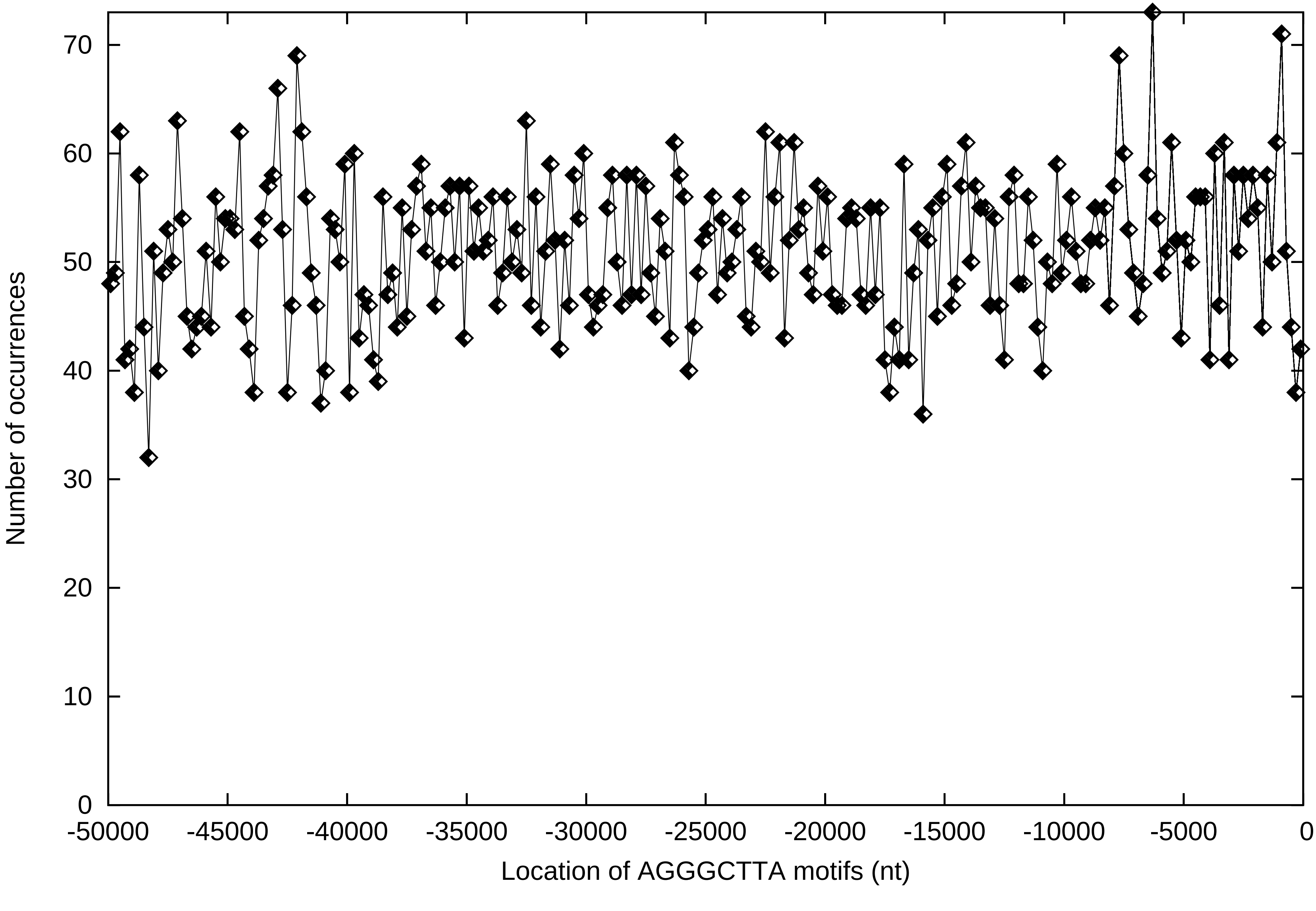

Number of occurrences
0
10
20
30
40
50
60
70
-50000
-45000
-40000
-35000
-30000
-25000
-20000
-15000
-10000
-5000
0
Location of AGGGCTTA motifs (nt)

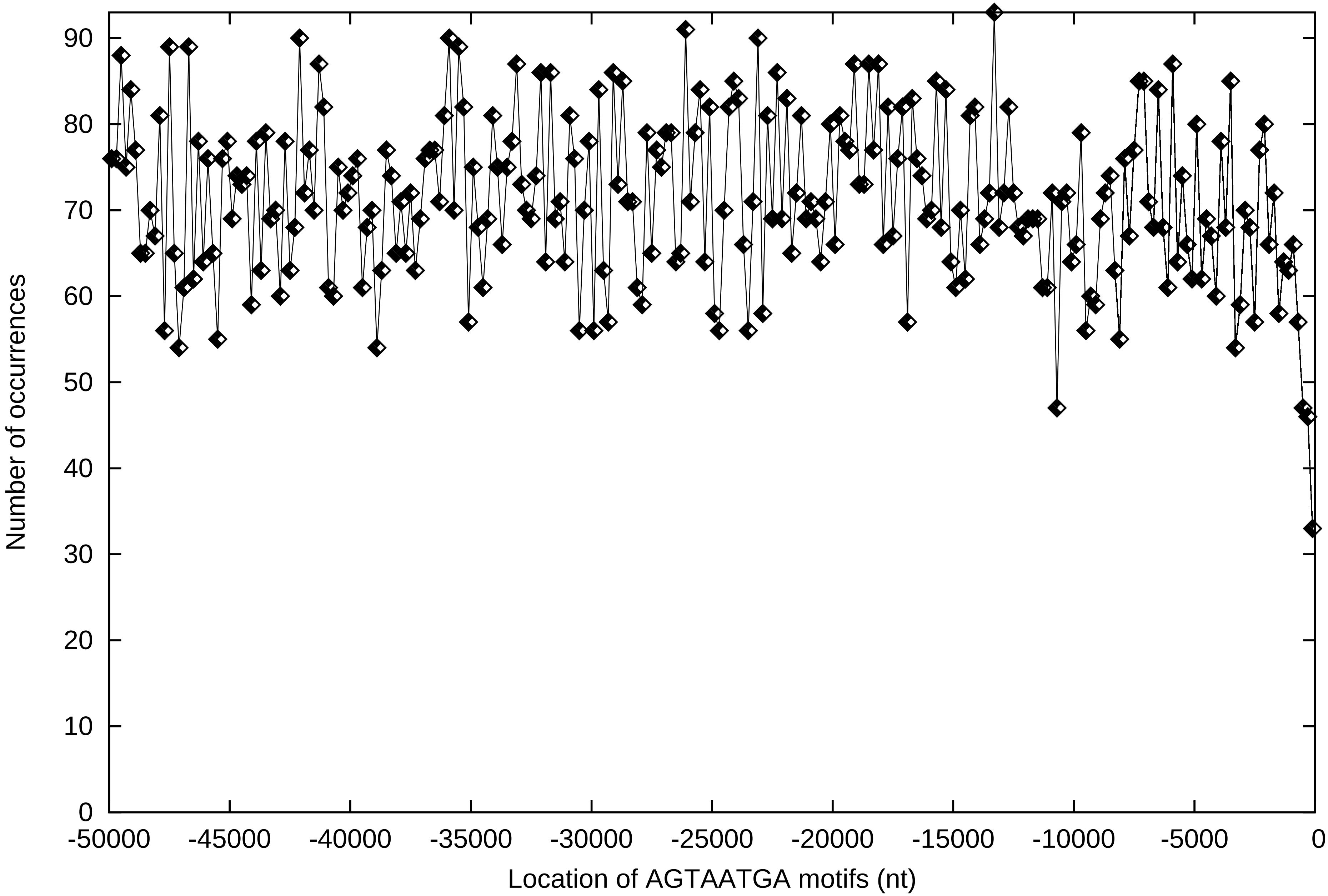
Number of occurrences
0
10
20
30
40
50
60
70
80
90
-50000
-45000
-40000
-35000
-30000
-25000
-20000
-15000
-10000
-5000
0
Location of AGTAATGA motifs (nt)

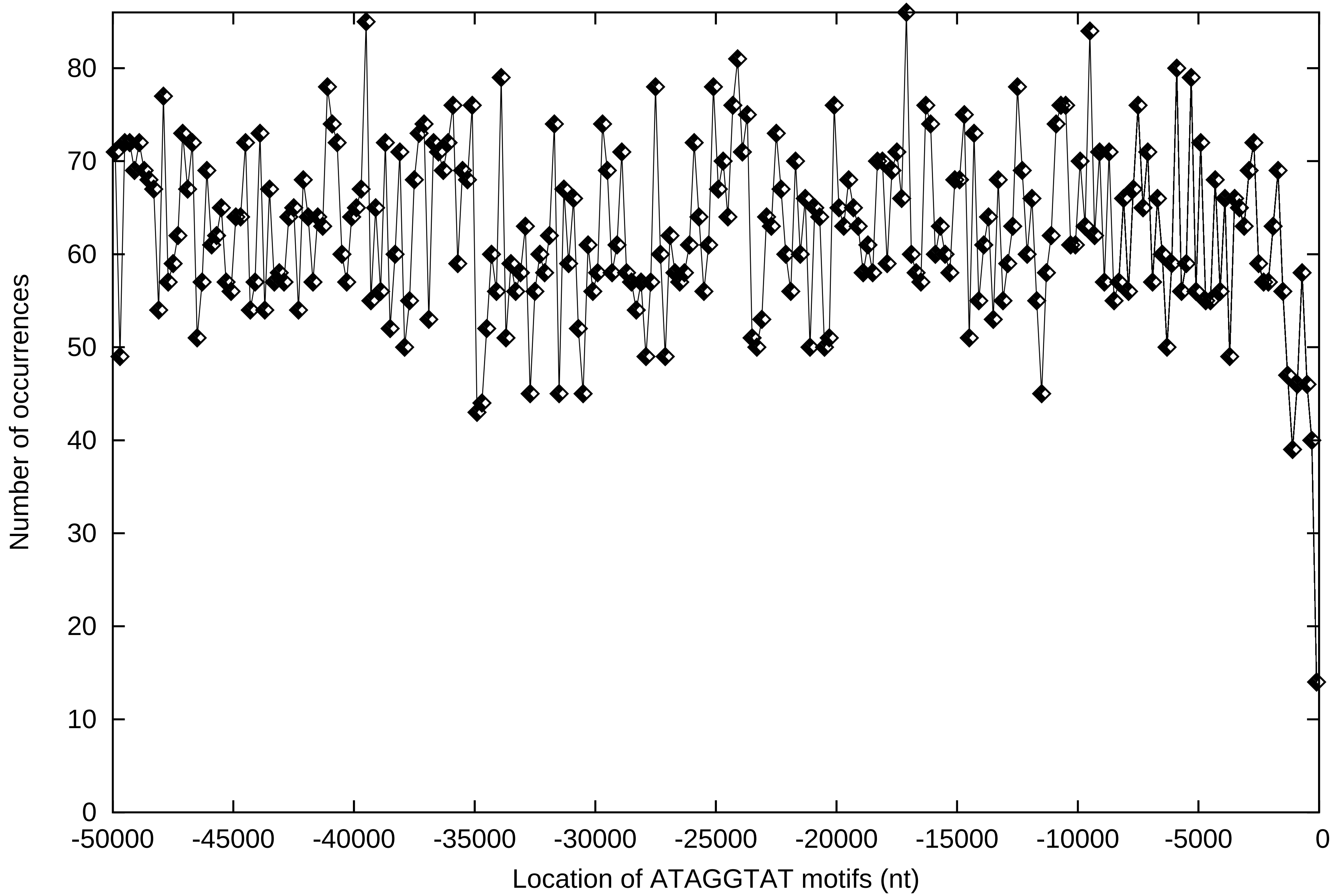

Number of occurrences
Location of ATAGGTAT motifs (nt)
0
10
20
30
40
50
60
70
80
-50000
-45000
-40000
-35000
-30000
-25000
-20000
-15000
-10000
-5000
0

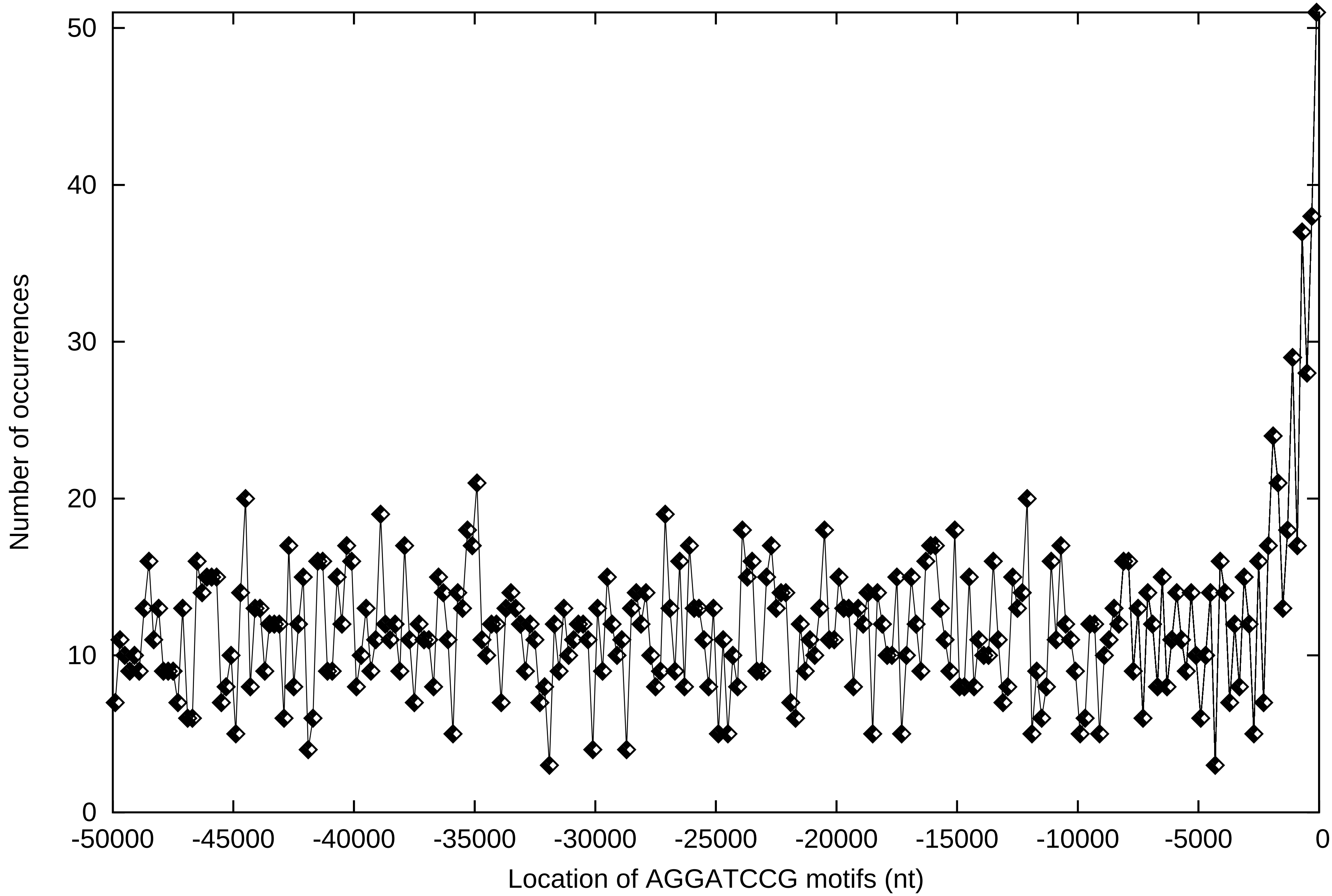

Number of occurrences
Location of AGGATCCG motifs (nt)
0
10
20
30
40
50
-50000
-45000
-40000
-35000
-30000
-25000
-20000
-15000
-10000
-5000
0

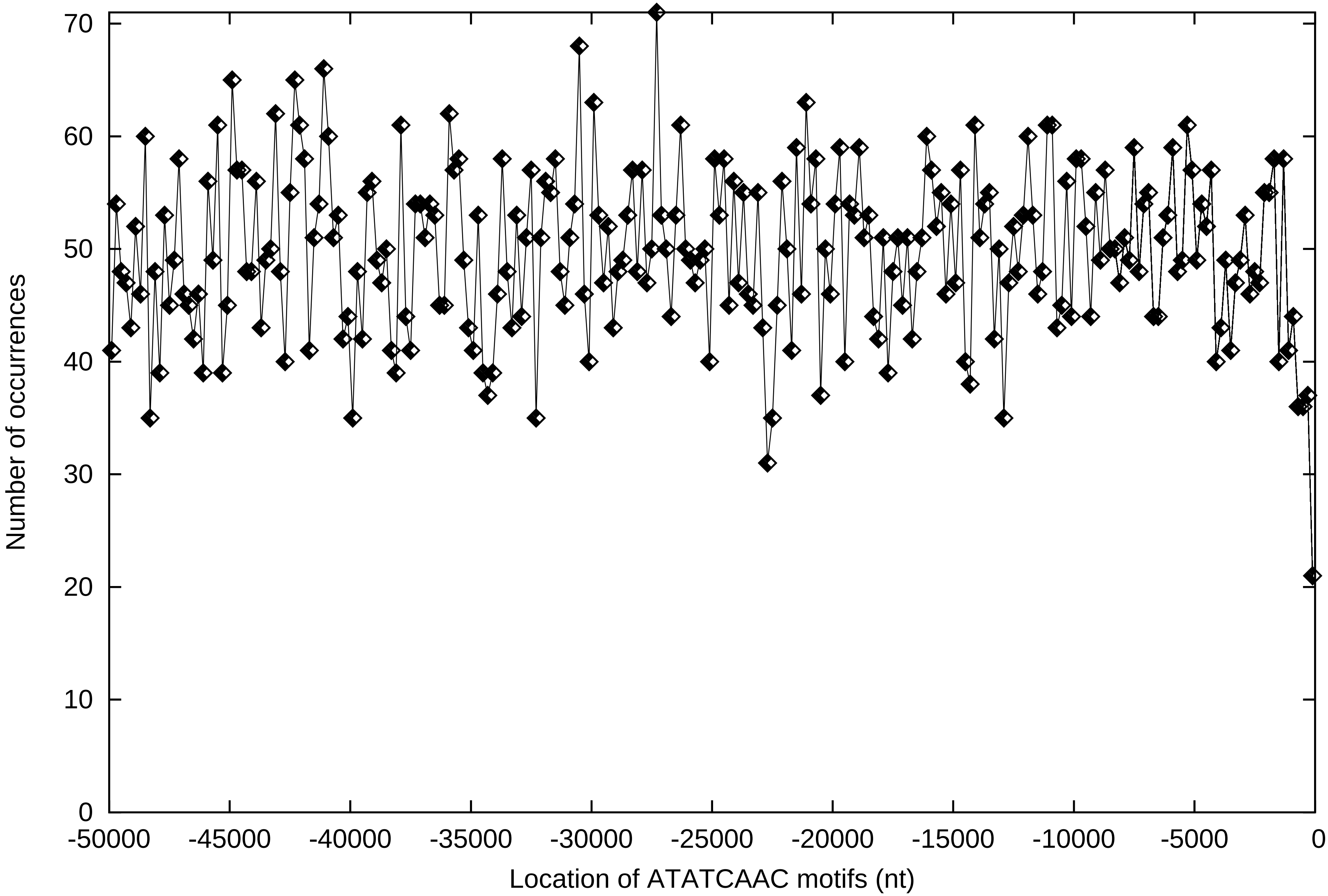

70
60
50
40
30
20
10
0
-50000
-45000
-40000
-35000
-30000
-25000
-20000
-15000
-10000
-5000
0
Number of occurrences
Location of ATATCAAC motifs (nt)

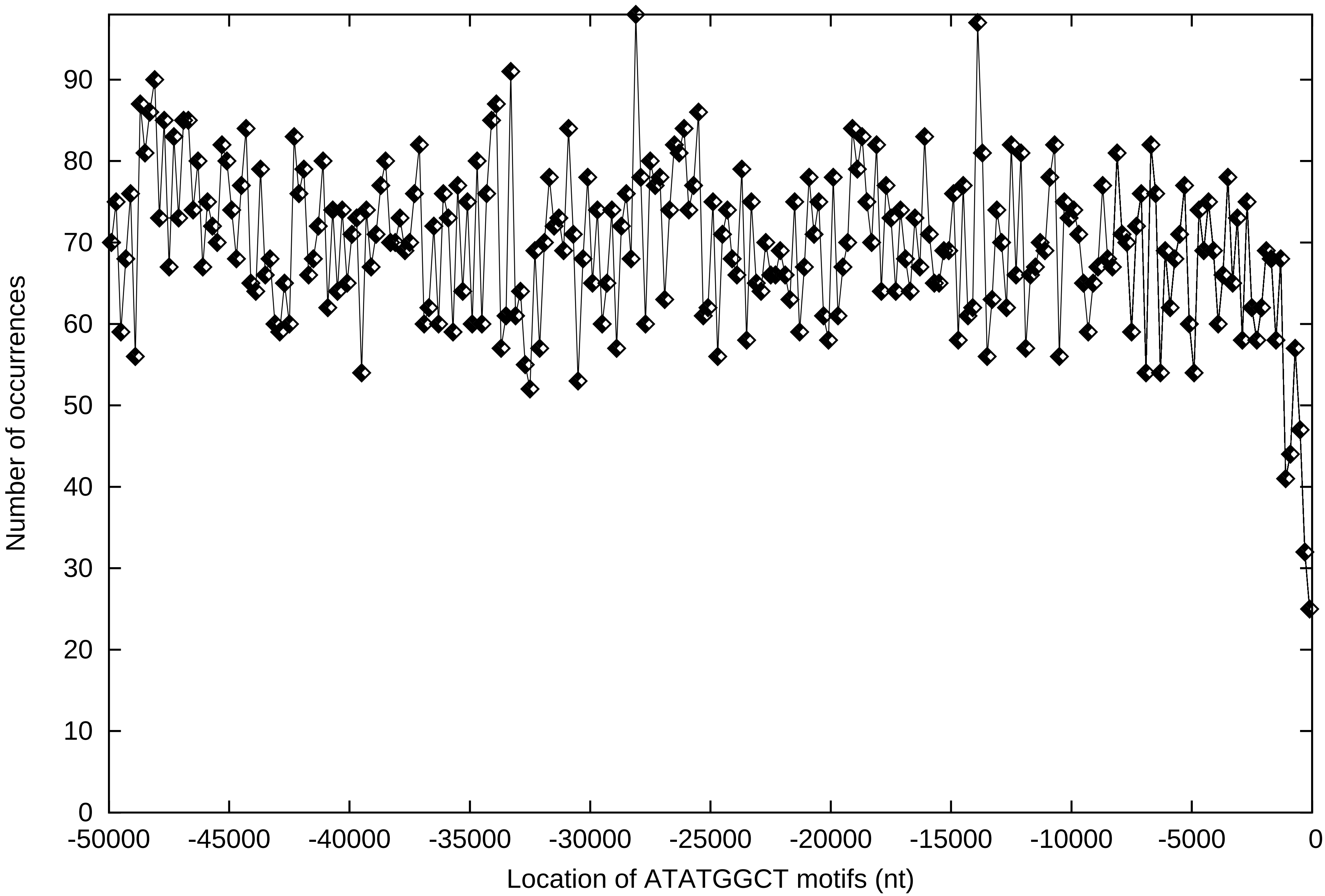

Number of occurrences
0
10
20
30
40
50
60
70
80
90
-50000
-45000
-40000
-35000
-30000
-25000
-20000
-15000
-10000
-5000
0
Location of ATATGGCT motifs (nt)

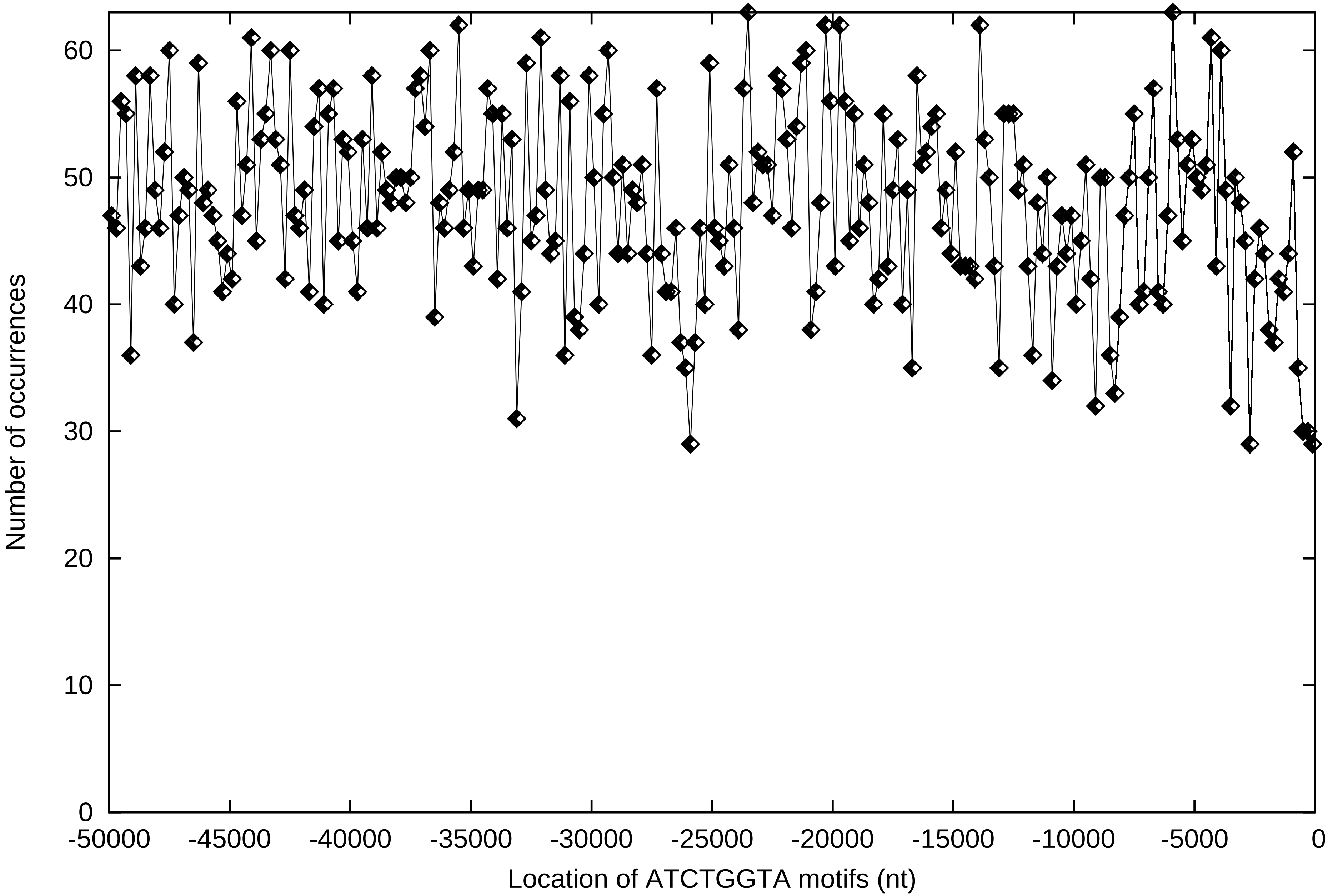

Number of occurrences
0
10
20
30
40
50
60
-50000
-45000
-40000
-35000
-30000
-25000
-20000
-15000
-10000
-5000
0
Location of ATCTGGTA motifs (nt)

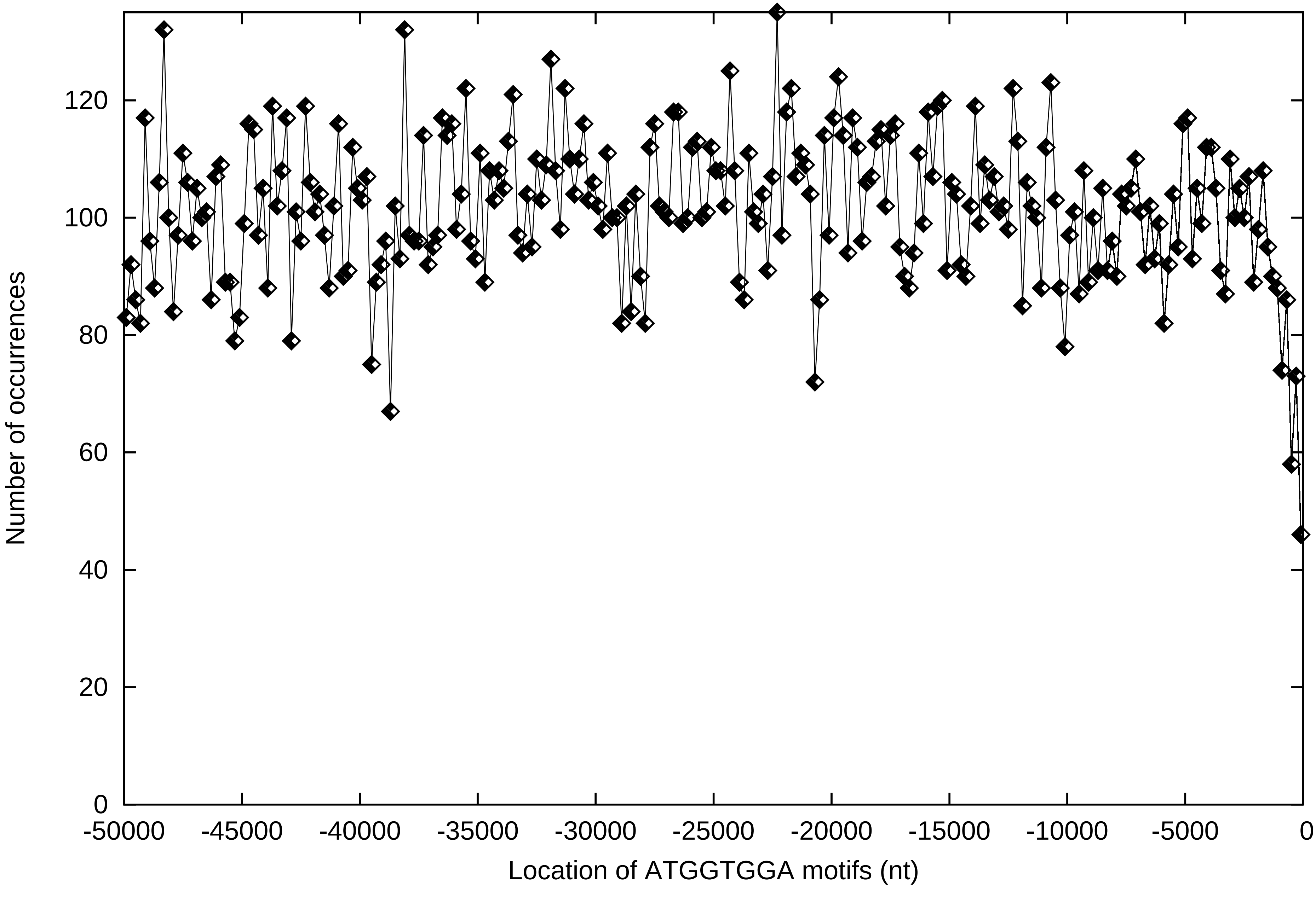

Number of occurrences
0
20
40
60
80
100
120
-50000
-45000
-40000
-35000
-30000
-25000
-20000
-15000
-10000
-5000
0
Location of ATGGTGGA motifs (nt)

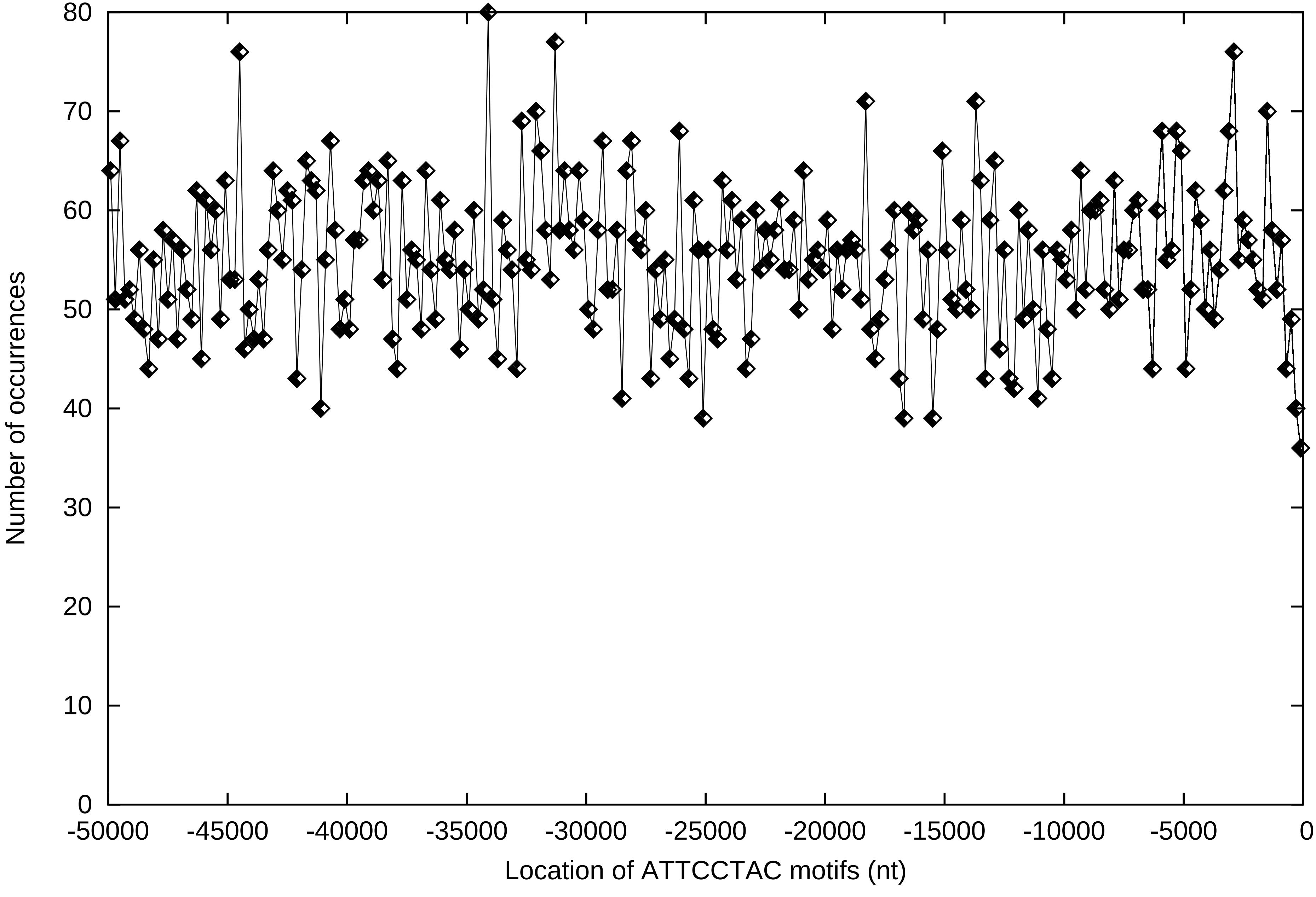

80
70
60
50
40
30
20
10
0
Number of occurrences
-50000
-45000
-40000
-35000
-30000
-25000
-20000
-15000
-10000
-5000
0
Location of ATTCCTAC motifs (nt)

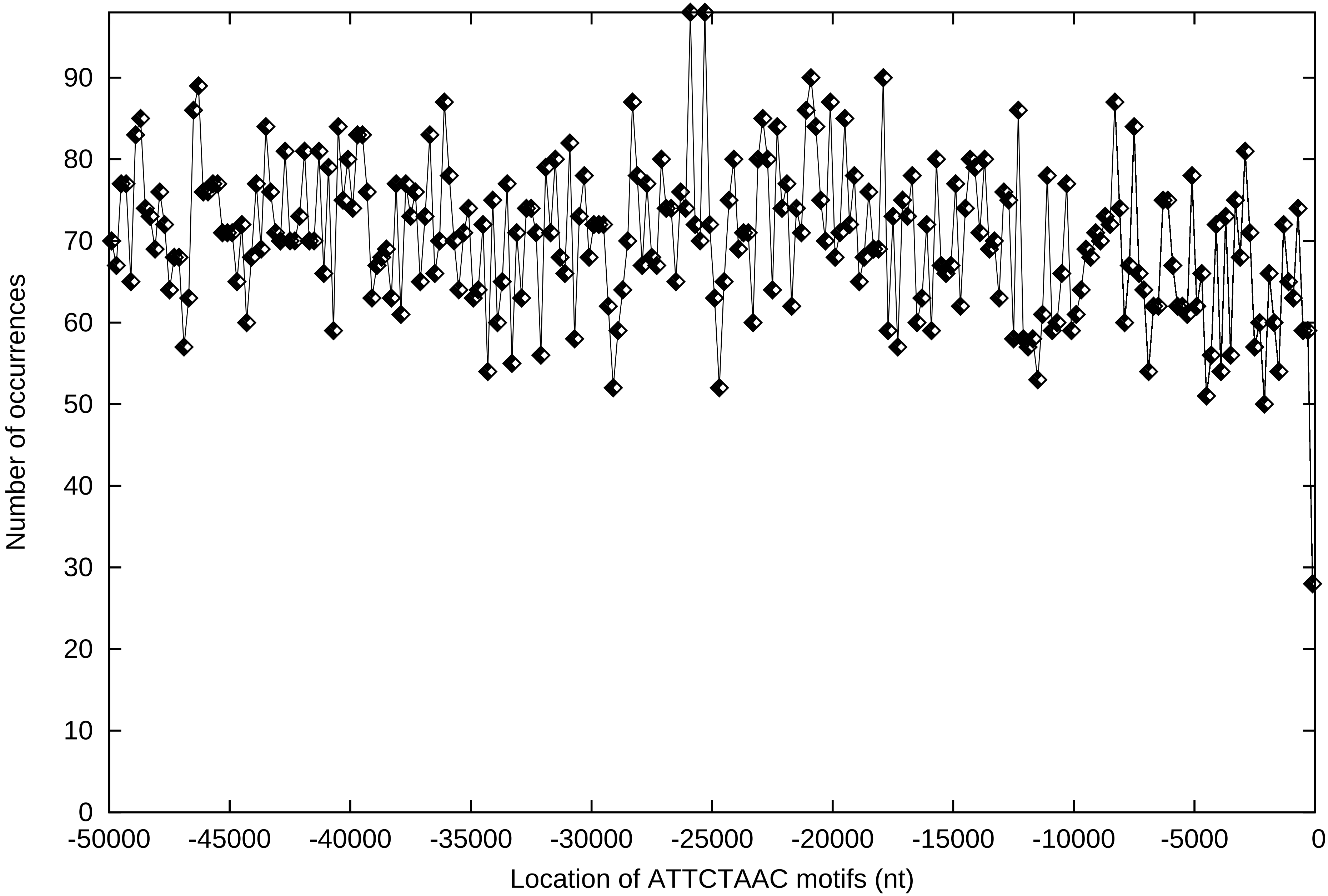

Number of occurrences
Location of ATTCTAAC motifs (nt)
0
10
20
30
40
50
60
70
80
90
-50000
-45000
-40000
-35000
-30000
-25000
-20000
-15000
-10000
-5000
0

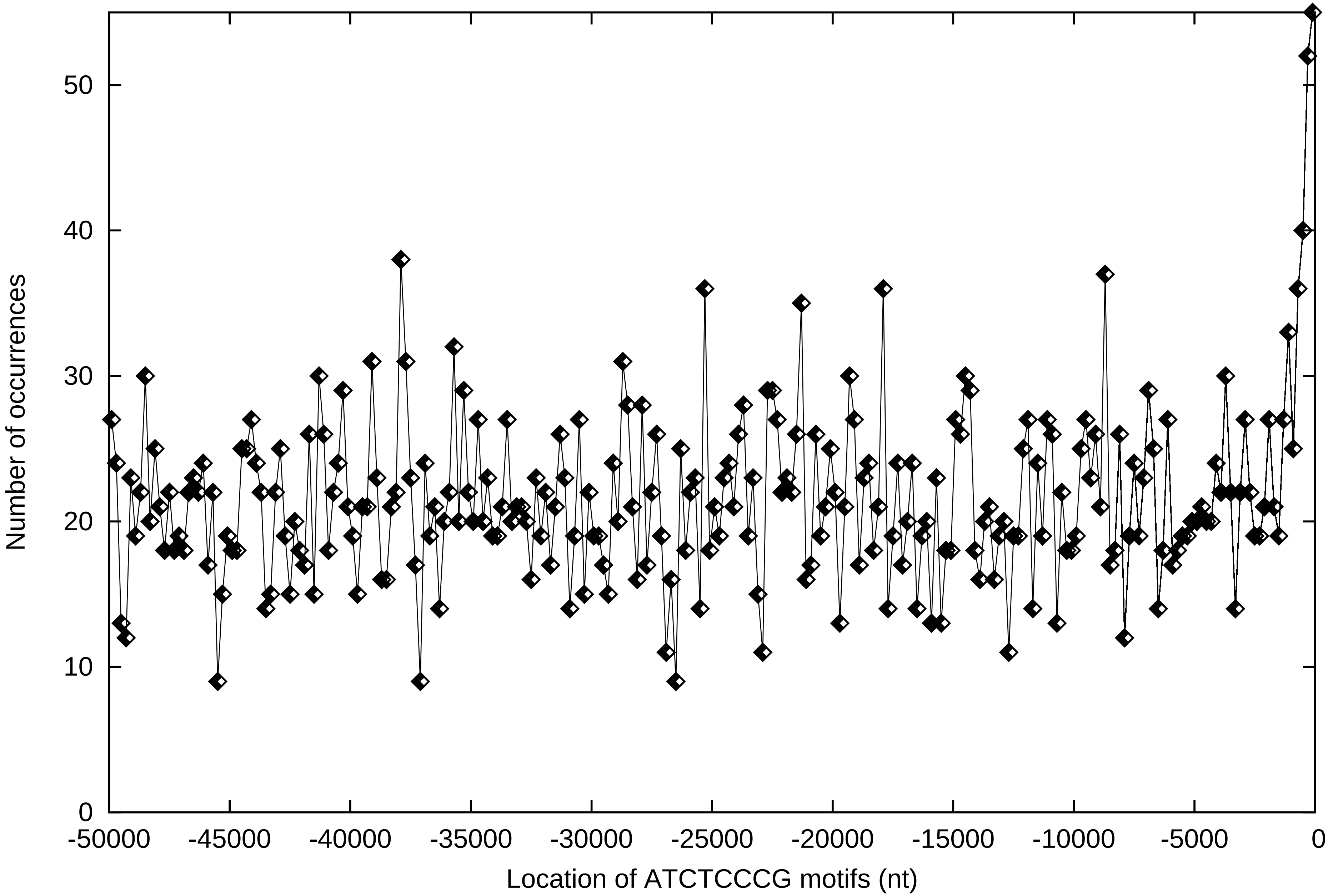

Number of occurrences
0
10
20
30
40
50
-50000
-45000
-40000
-35000
-30000
-25000
-20000
-15000
-10000
-5000
0
Location of ATCTCCCG motifs (nt)

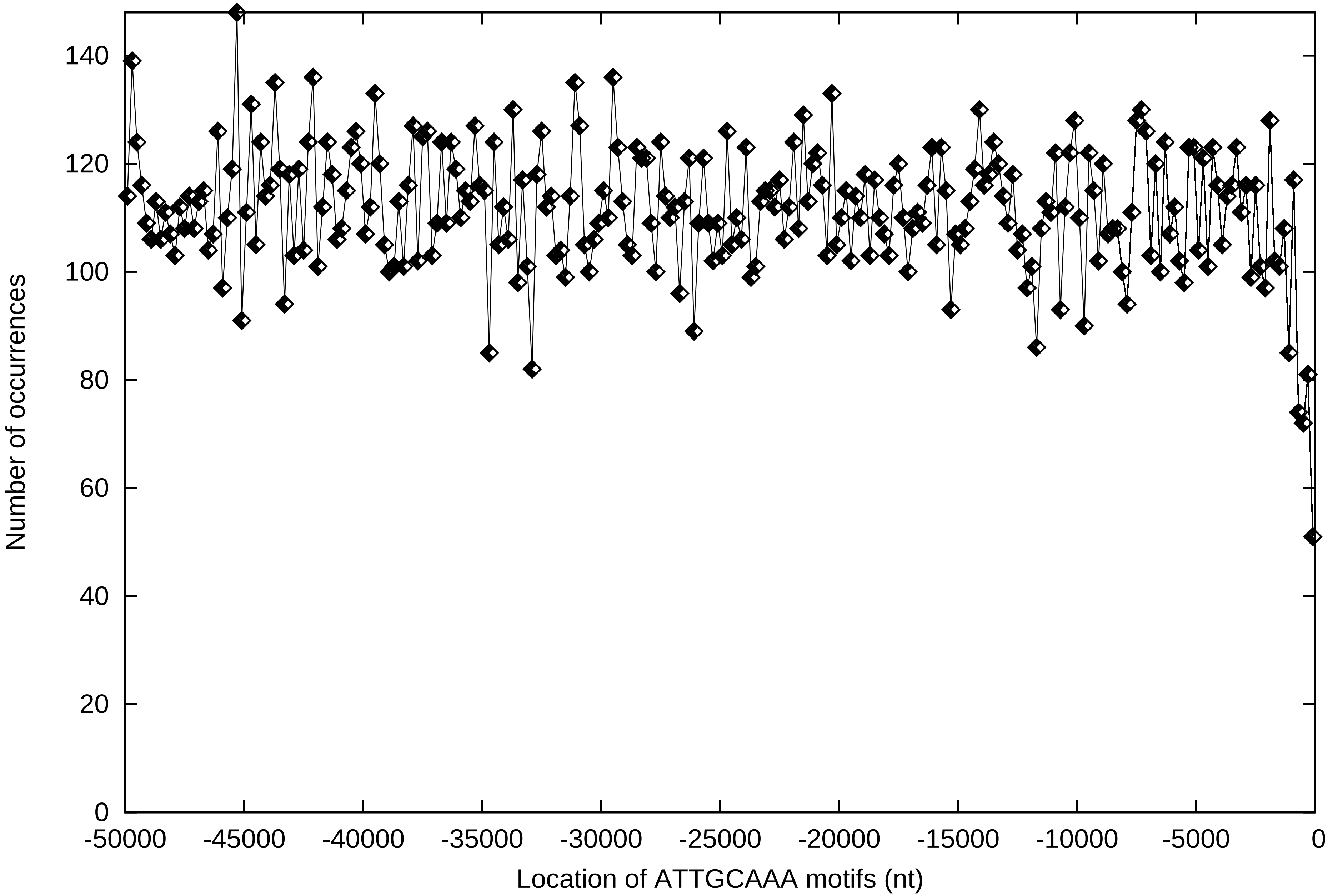

Number of occurrences
0
20
40
60
80
100
120
140
-50000
-45000
-40000
-35000
-30000
-25000
-20000
-15000
-10000
-5000
0
Location of ATTGCAAA motifs (nt)

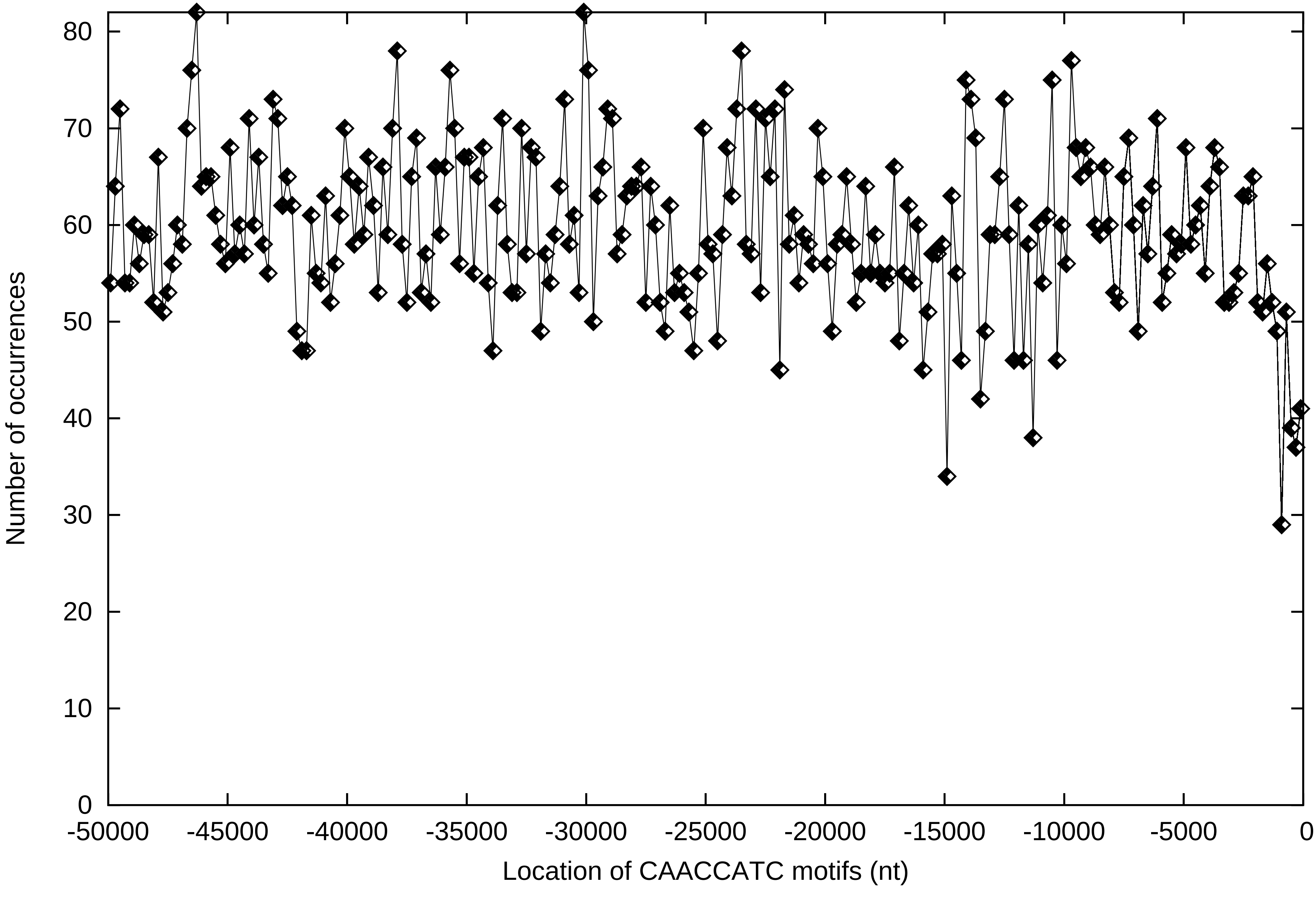

Number of occurrences
0
10
20
30
40
50
60
70
80
-50000
-45000
-40000
-35000
-30000
-25000
-20000
-15000
-10000
-5000
0
Location of CAACCATC motifs (nt)

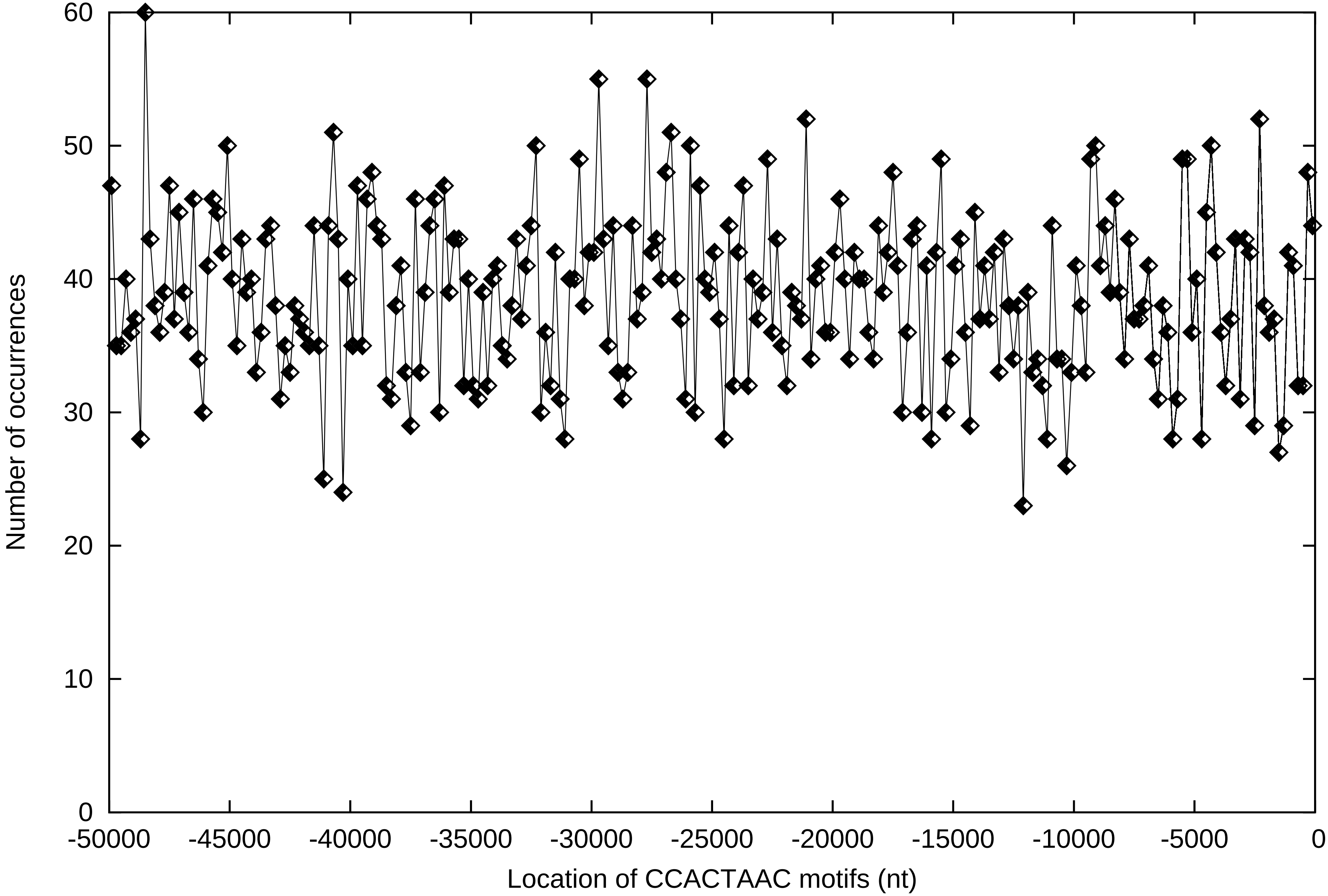

60
50
40
30
20
10
0
Number of occurrences
-50000
-45000
-40000
-35000
-30000
-25000
-20000
-15000
-10000
-5000
0
Location of CCACTAAC motifs (nt)

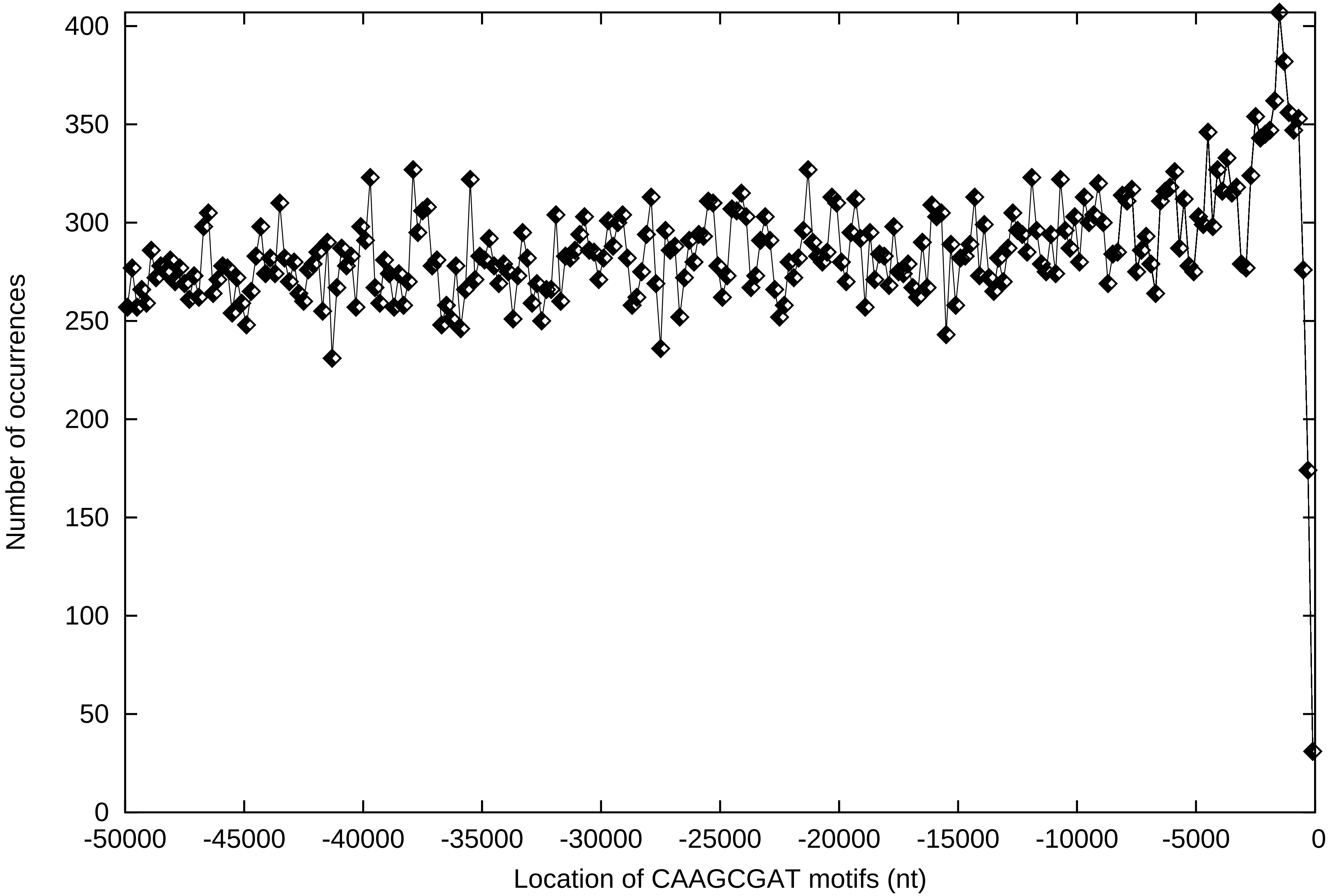
Number of occurrences
Location of CAAGCGAT motifs (nt)
400
350
300
250
200
150
100
50
0
-50000
-45000
-40000
-35000
-30000
-25000
-20000
-15000
-10000
-5000
0

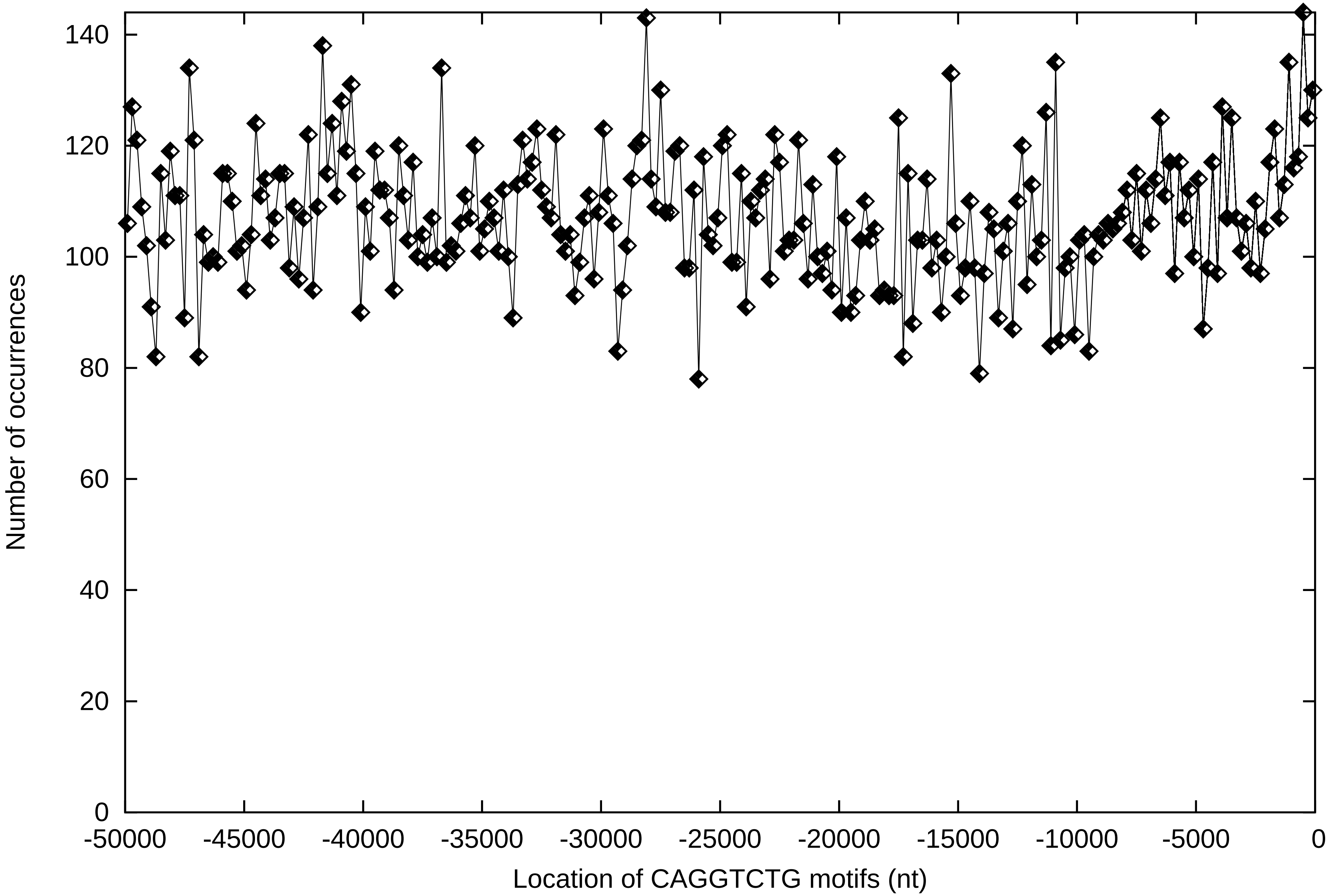

Number of occurrences
0
20
40
60
80
100
120
140
-50000
-45000
-40000
-35000
-30000
-25000
-20000
-15000
-10000
-5000
0
Location of CAGGTCTG motifs (nt)

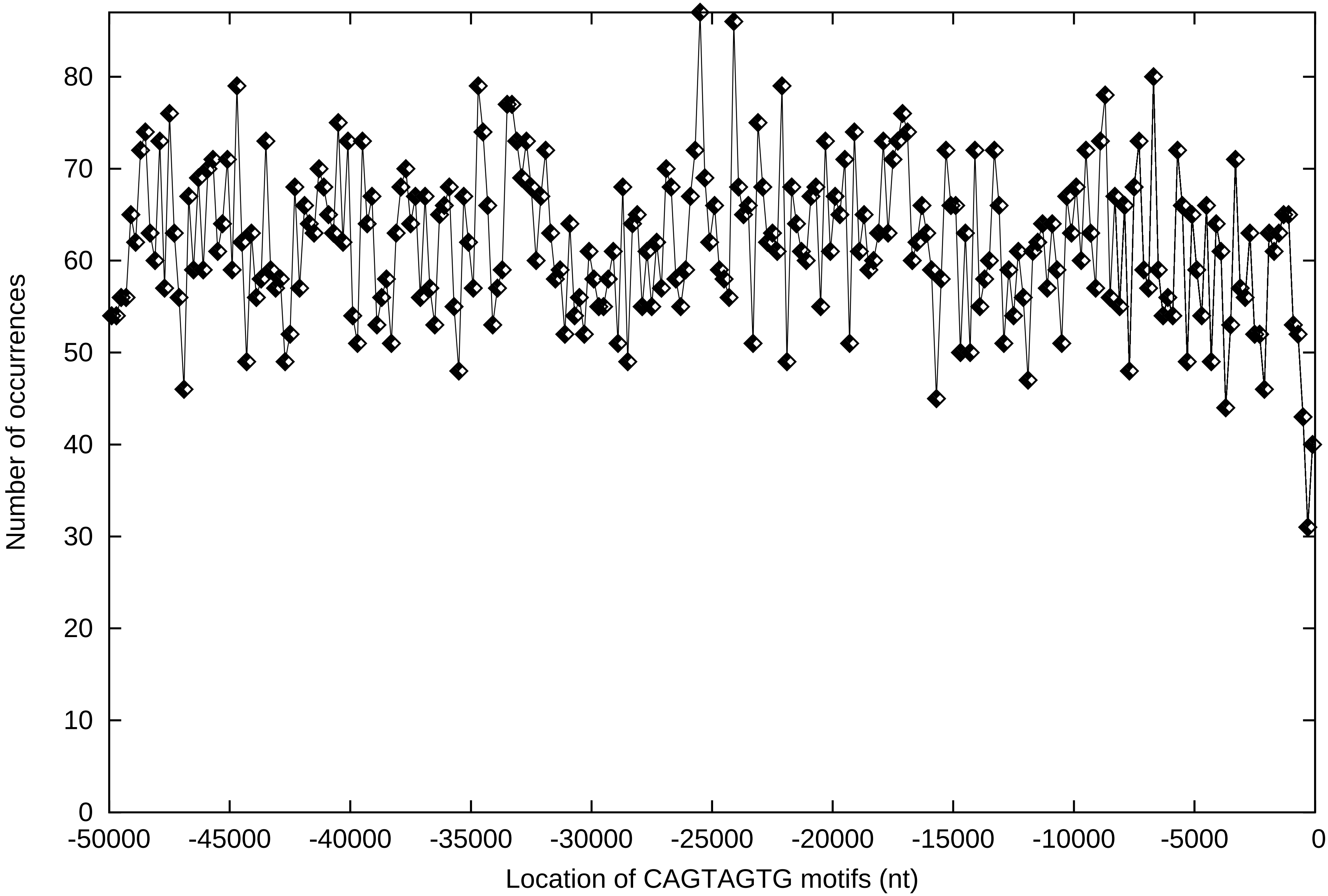

Number of occurrences
0
10
20
30
40
50
60
70
80
-50000
-45000
-40000
-35000
-30000
-25000
-20000
-15000
-10000
-5000
0
Location of CAGTAGTG motifs (nt)

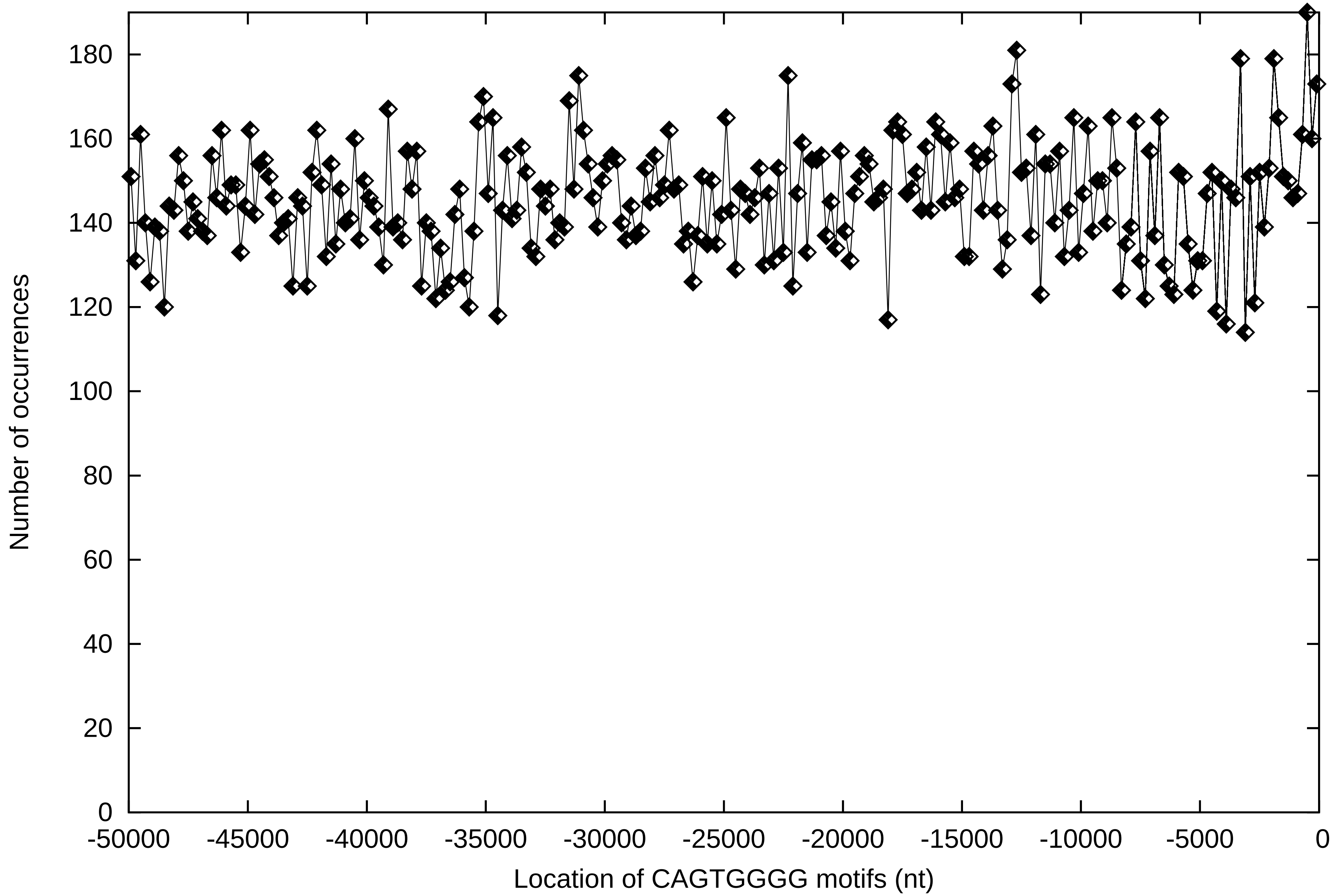

Number of occurrences
0
20
40
60
80
100
120
140
160
180
-50000
-45000
-40000
-35000
-30000
-25000
-20000
-15000
-10000
-5000
0
Location of CAGTGGGG motifs (nt)

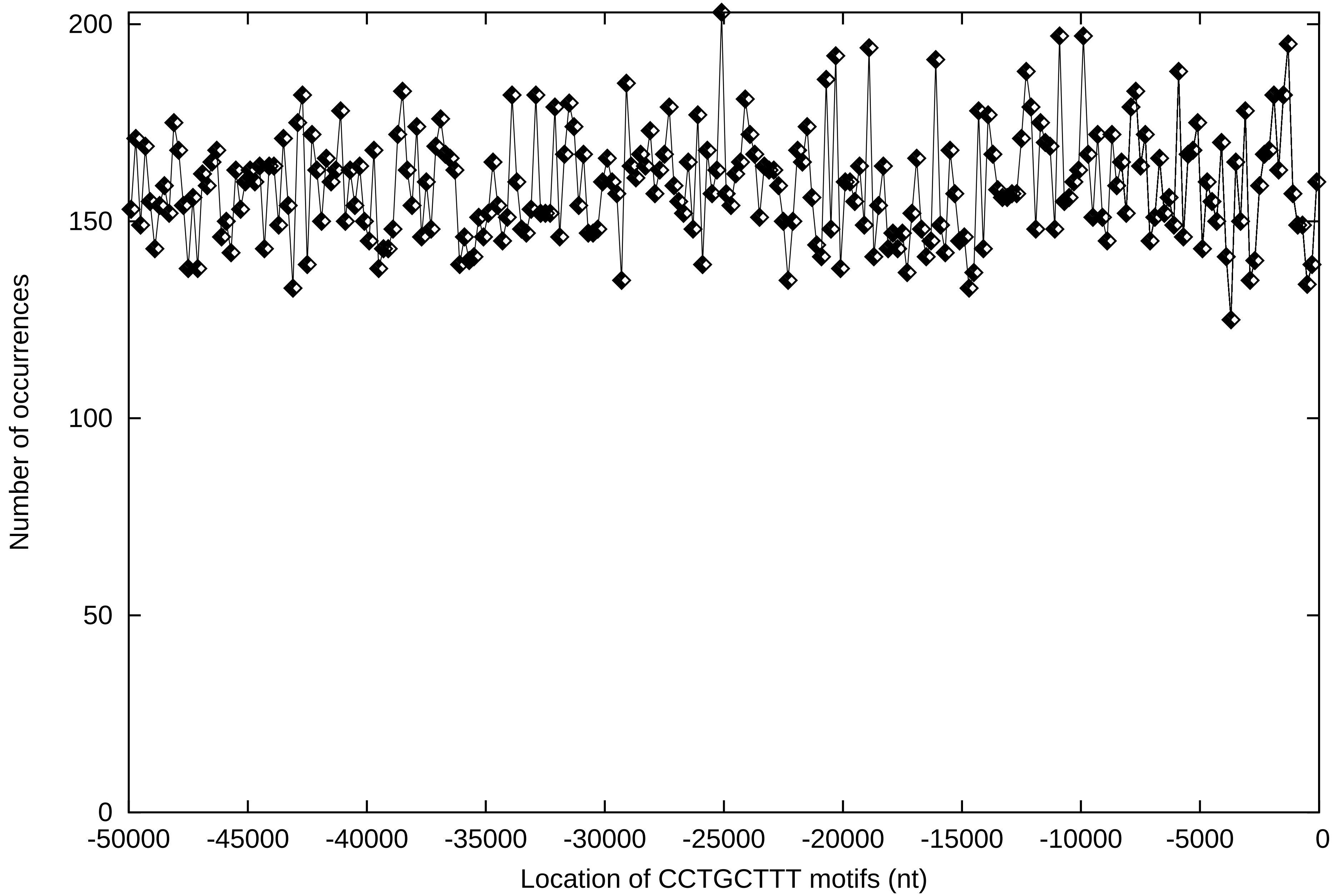

Number of occurrences
200
150
100
50
0
-50000
-45000
-40000
-35000
-30000
-25000
-20000
-15000
-10000
-5000
0
Location of CCTGCTTT motifs (nt)

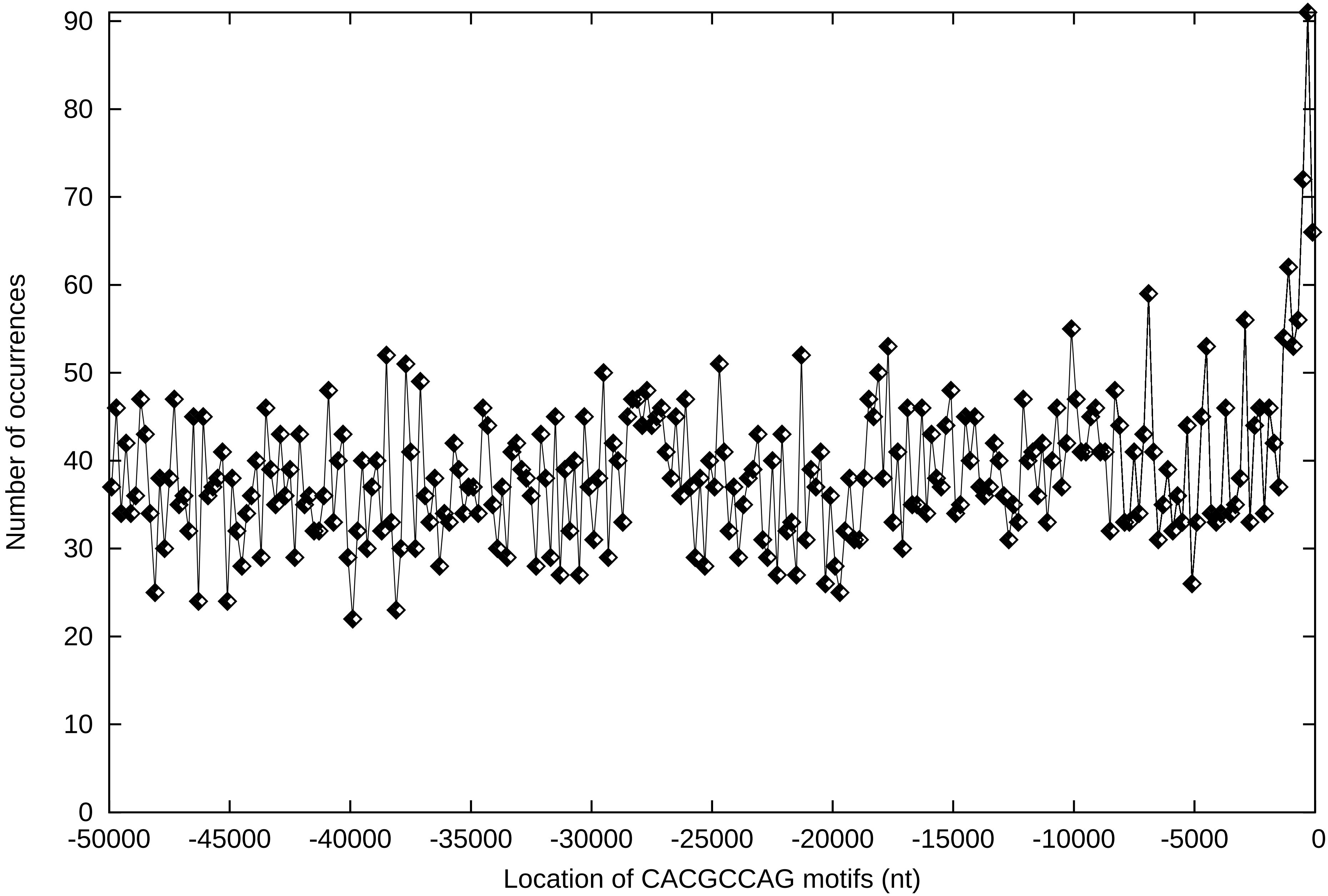
Number of occurrences
Location of CACGCCAG motifs (nt)
0
10
20
30
40
50
60
70
80
90
-50000
-45000
-40000
-35000
-30000
-25000
-20000
-15000
-10000
-5000
0

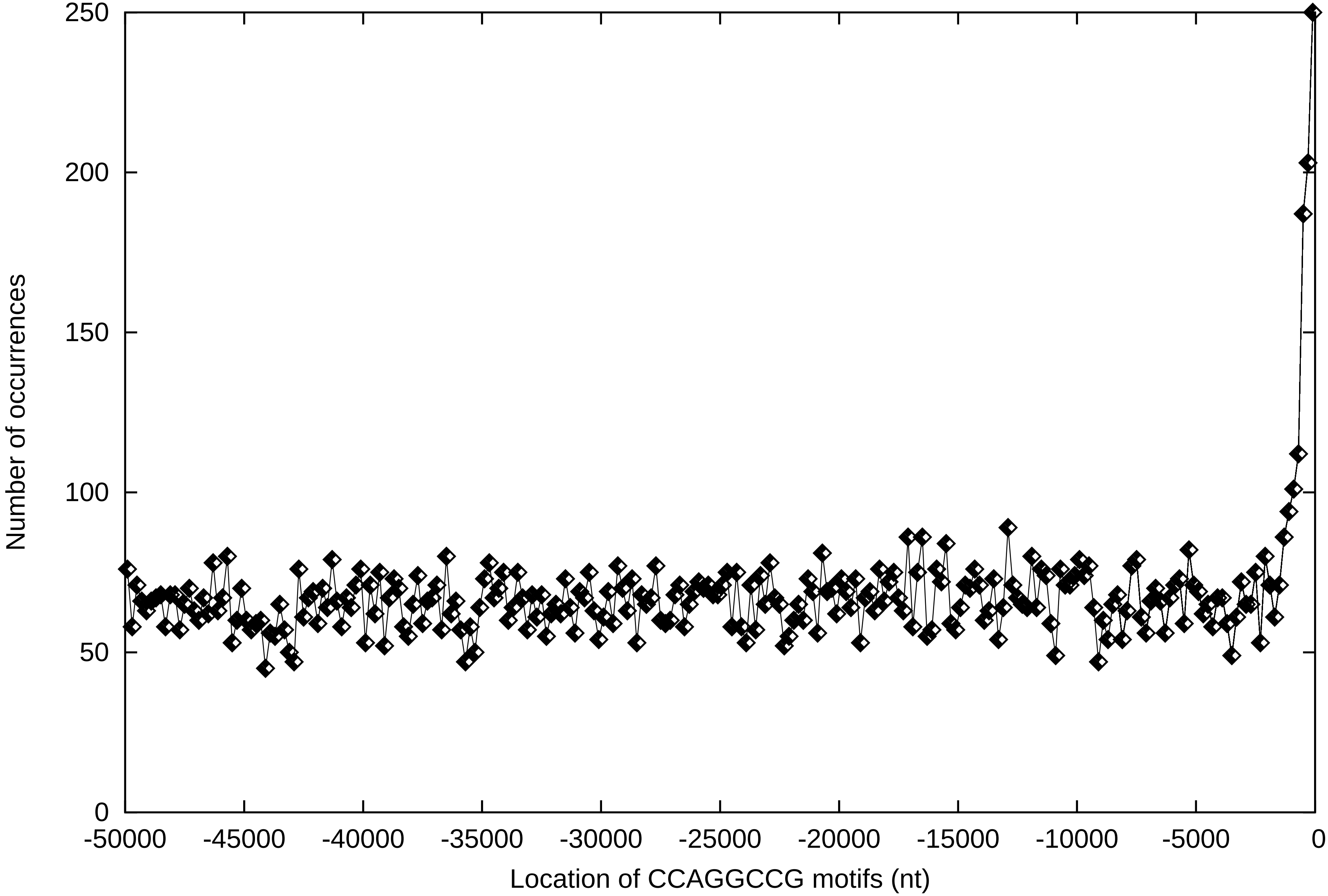
Number of occurrences
250
200
150
100
50
0
-50000
-45000
-40000
-35000
-30000
-25000
-20000
-15000
-10000
-5000
0
Location of CCAGGCCG motifs (nt)

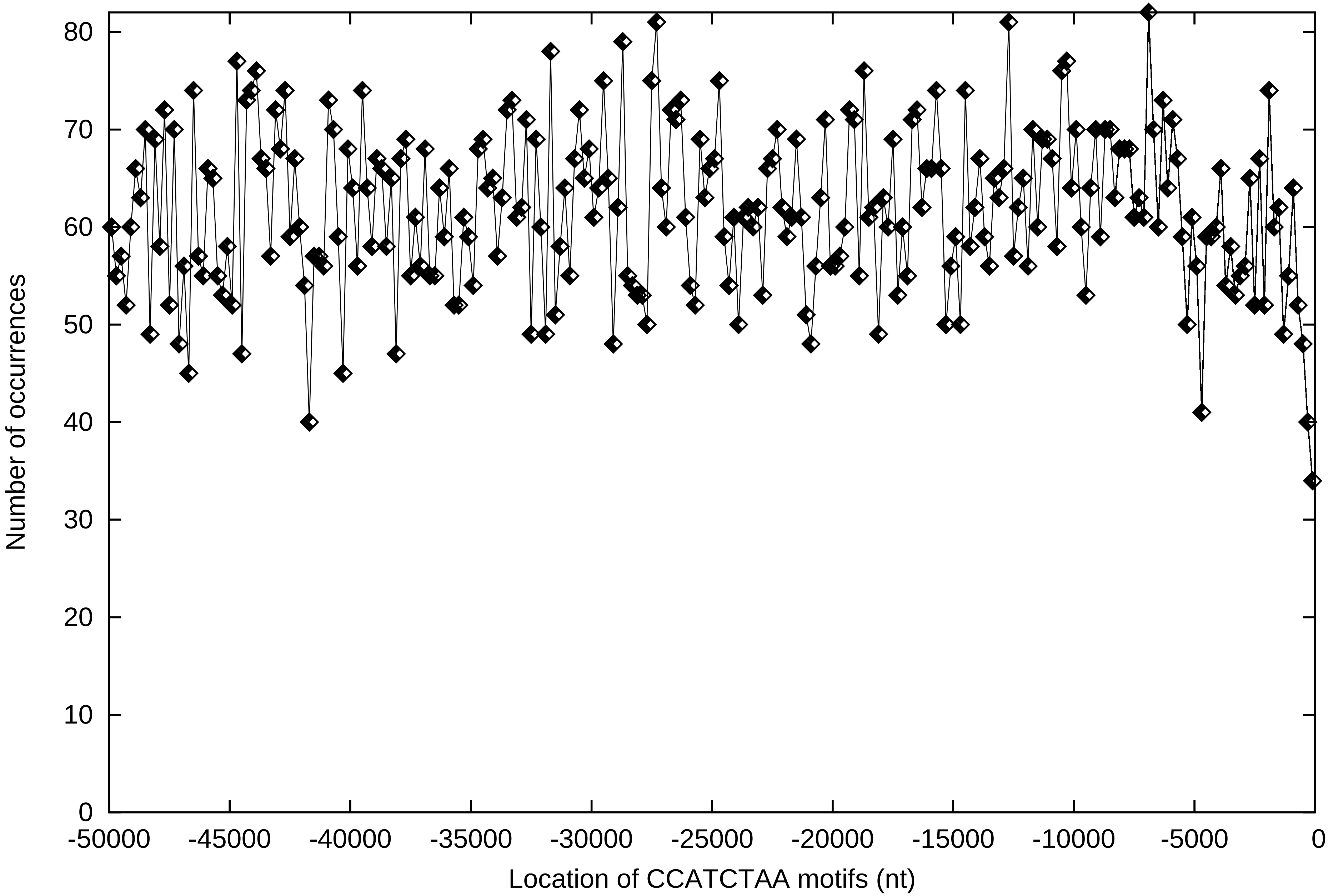

Number of occurrences
Location of CCATCTAA motifs (nt)
0
10
20
30
40
50
60
70
80
-50000
-45000
-40000
-35000
-30000
-25000
-20000
-15000
-10000
-5000
0

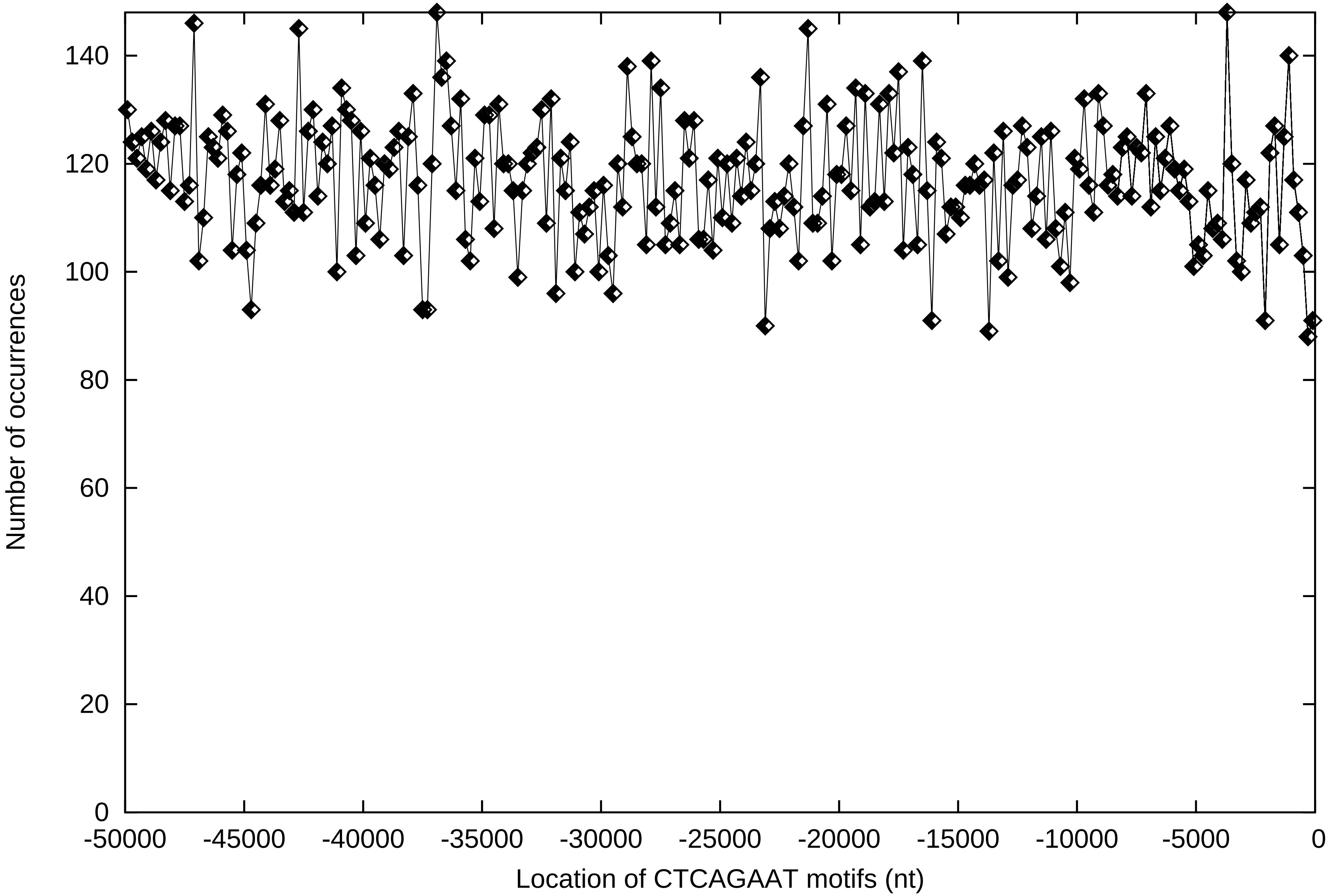

Number of occurrences
0
20
40
60
80
100
120
140
-50000
-45000
-40000
-35000
-30000
-25000
-20000
-15000
-10000
-5000
0
Location of CTCAGAAT motifs (nt)

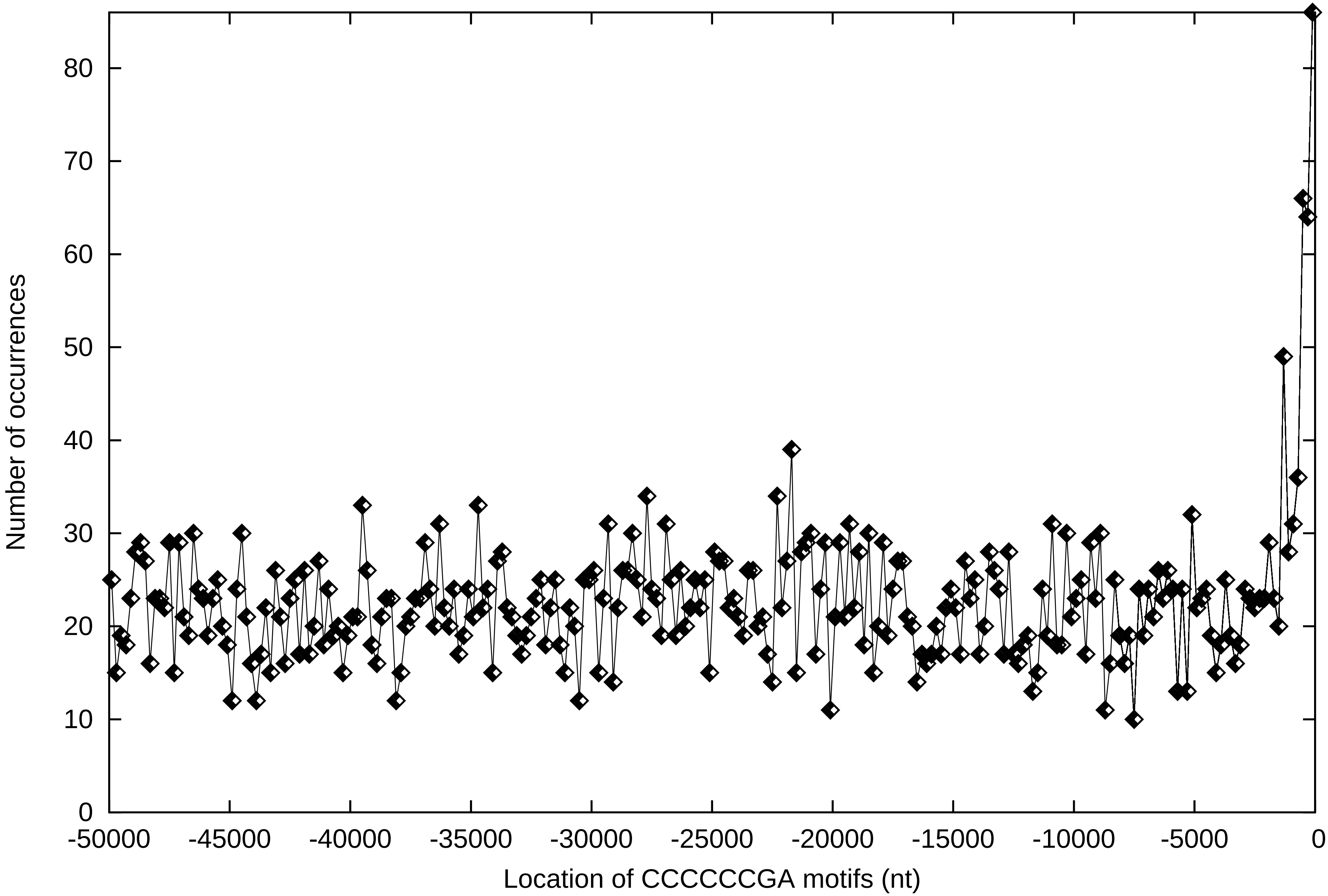

Number of occurrences
Location of CCCCCCGA motifs (nt)
0
10
20
30
40
50
60
70
80
-50000
-45000
-40000
-35000
-30000
-25000
-20000
-15000
-10000
-5000
0

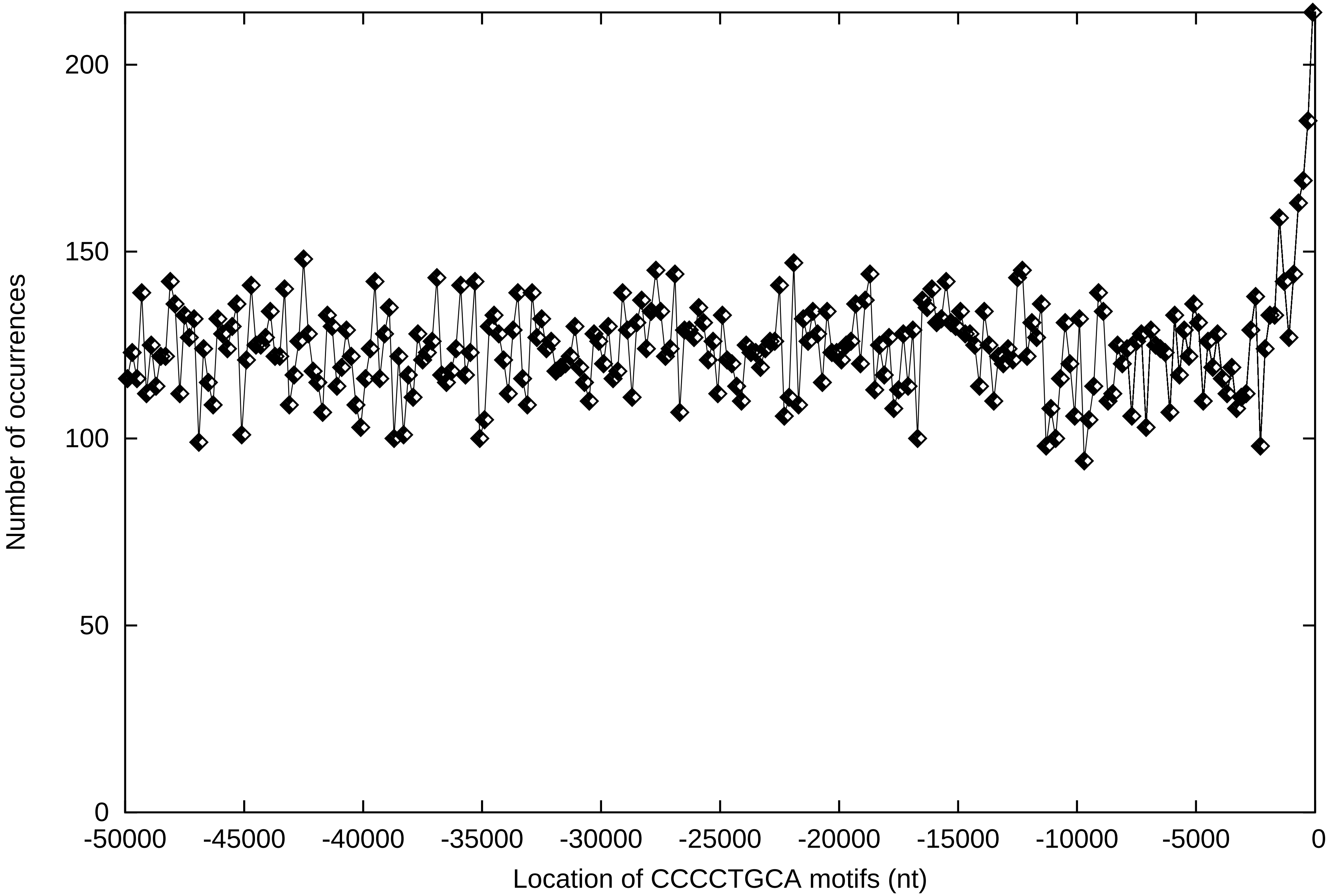

Number of occurrences
Location of CCCCTGCA motifs (nt)
0
50
100
150
200
-50000
-45000
-40000
-35000
-30000
-25000
-20000
-15000
-10000
-5000
0

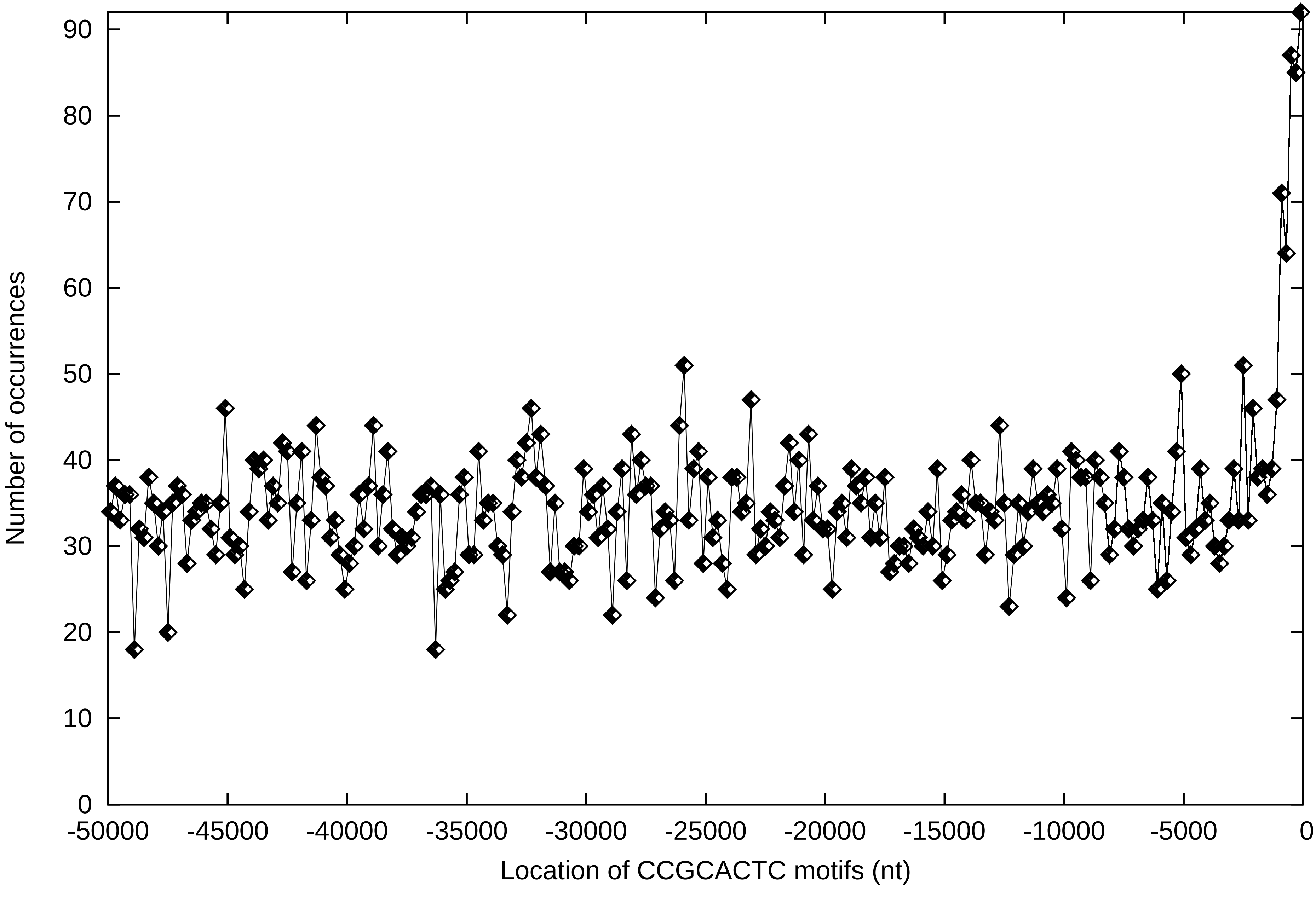

Number of occurrences
0
10
20
30
40
50
60
70
80
90
-50000
-45000
-40000
-35000
-30000
-25000
-20000
-15000
-10000
-5000
0
Location of CCGCACTC motifs (nt)

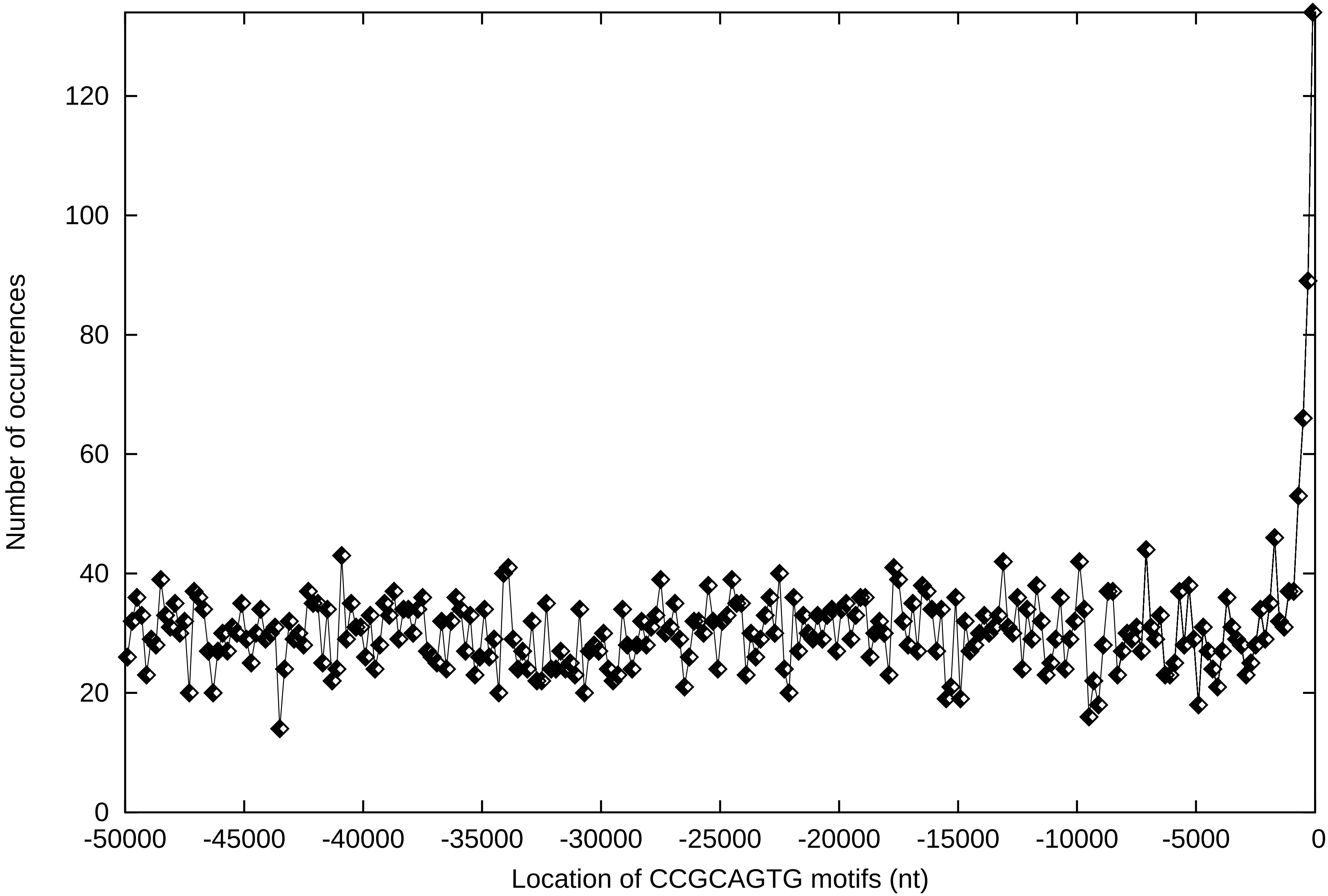

Number of occurrences
0
20
40
60
80
100
120
-50000
-45000
-40000
-35000
-30000
-25000
-20000
-15000
-10000
-5000
0
Location of CCGCAGTG motifs (nt)

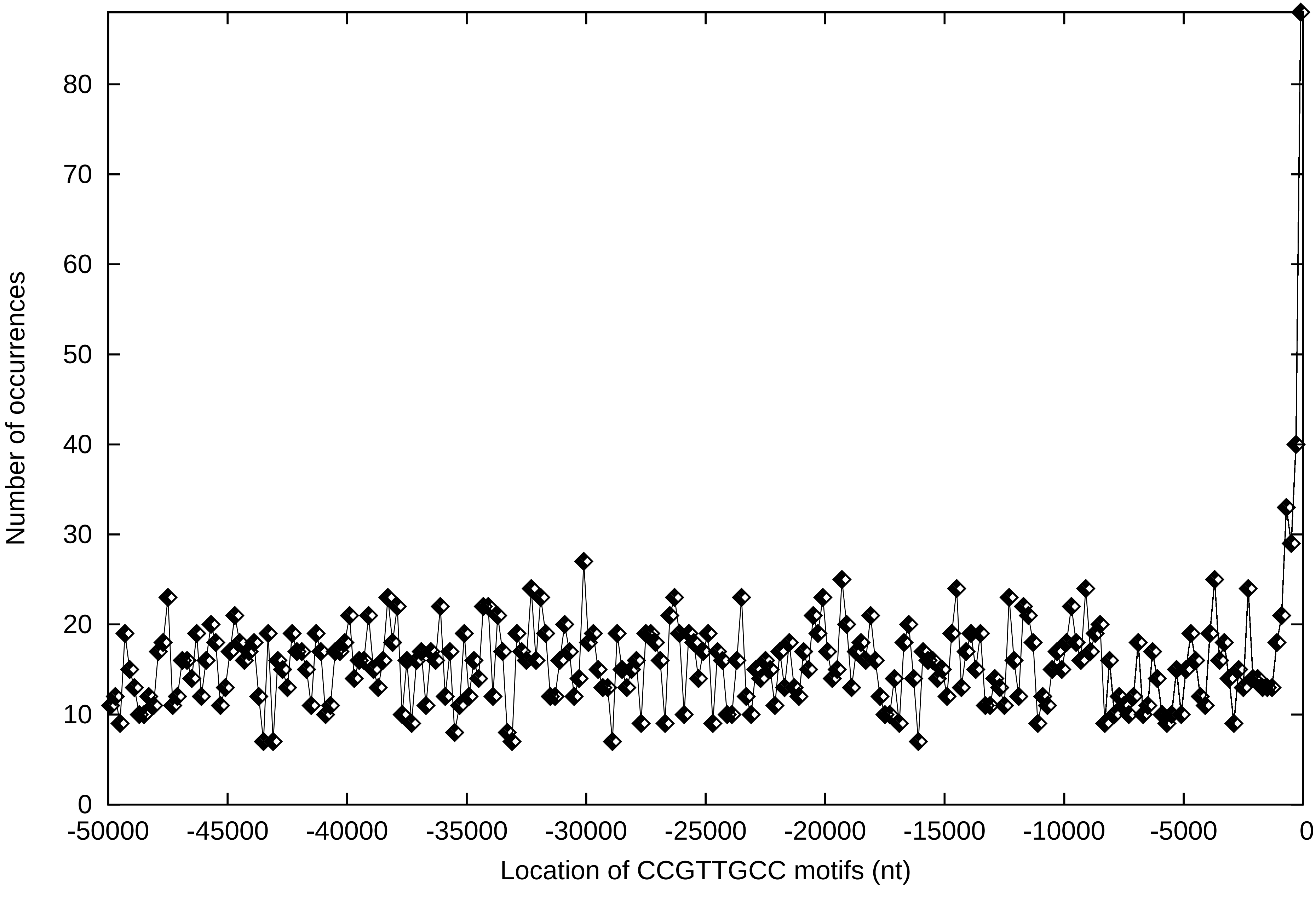

Number of occurrences
Location of CCGTTGCC motifs (nt)
0
10
20
30
40
50
60
70
80
-50000
-45000
-40000
-35000
-30000
-25000
-20000
-15000
-10000
-5000
0

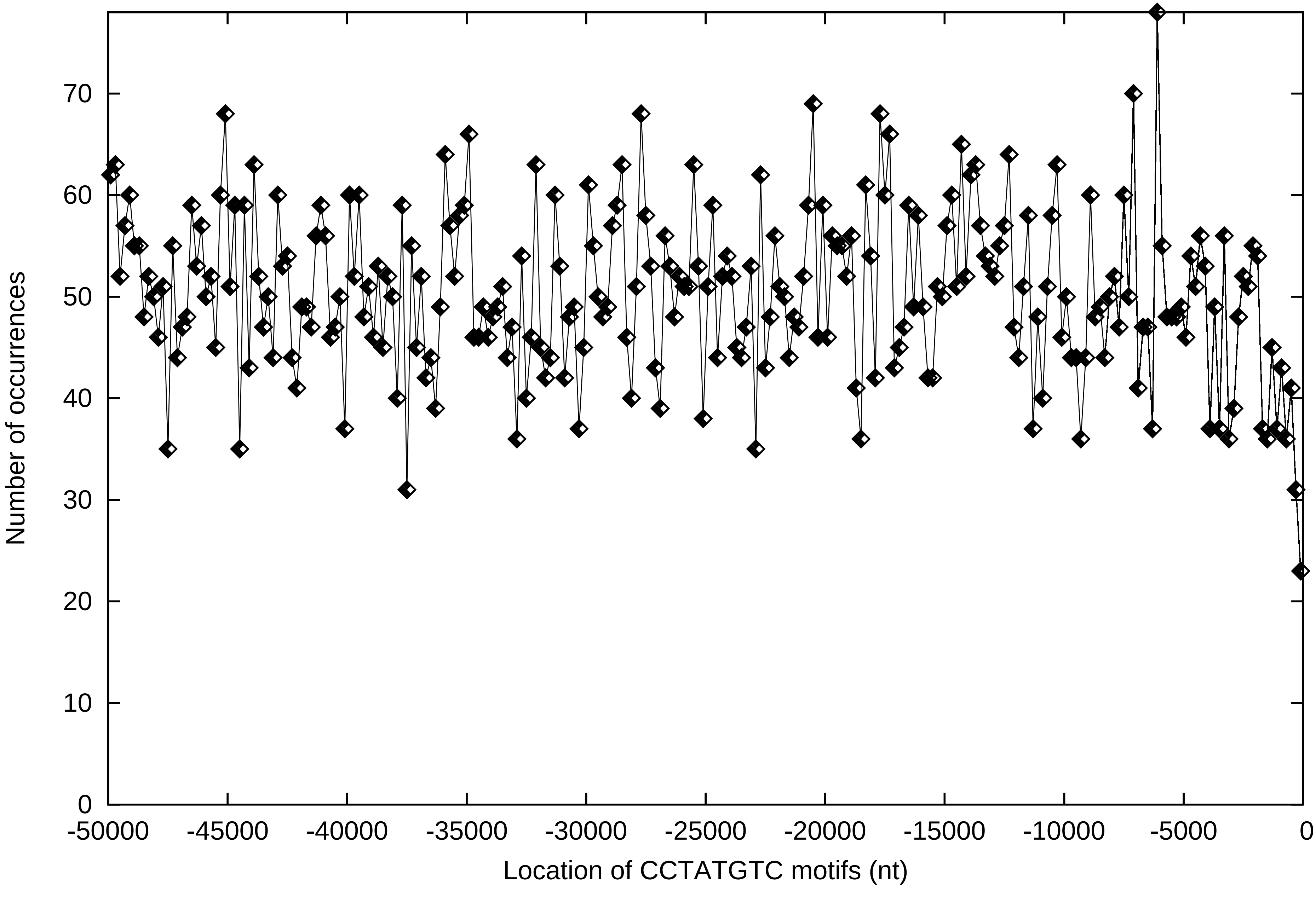
Number of occurrences
Location of CCTATGTC motifs (nt)
0
10
20
30
40
50
60
70
-50000
-45000
-40000
-35000
-30000
-25000
-20000
-15000
-10000
-5000
0

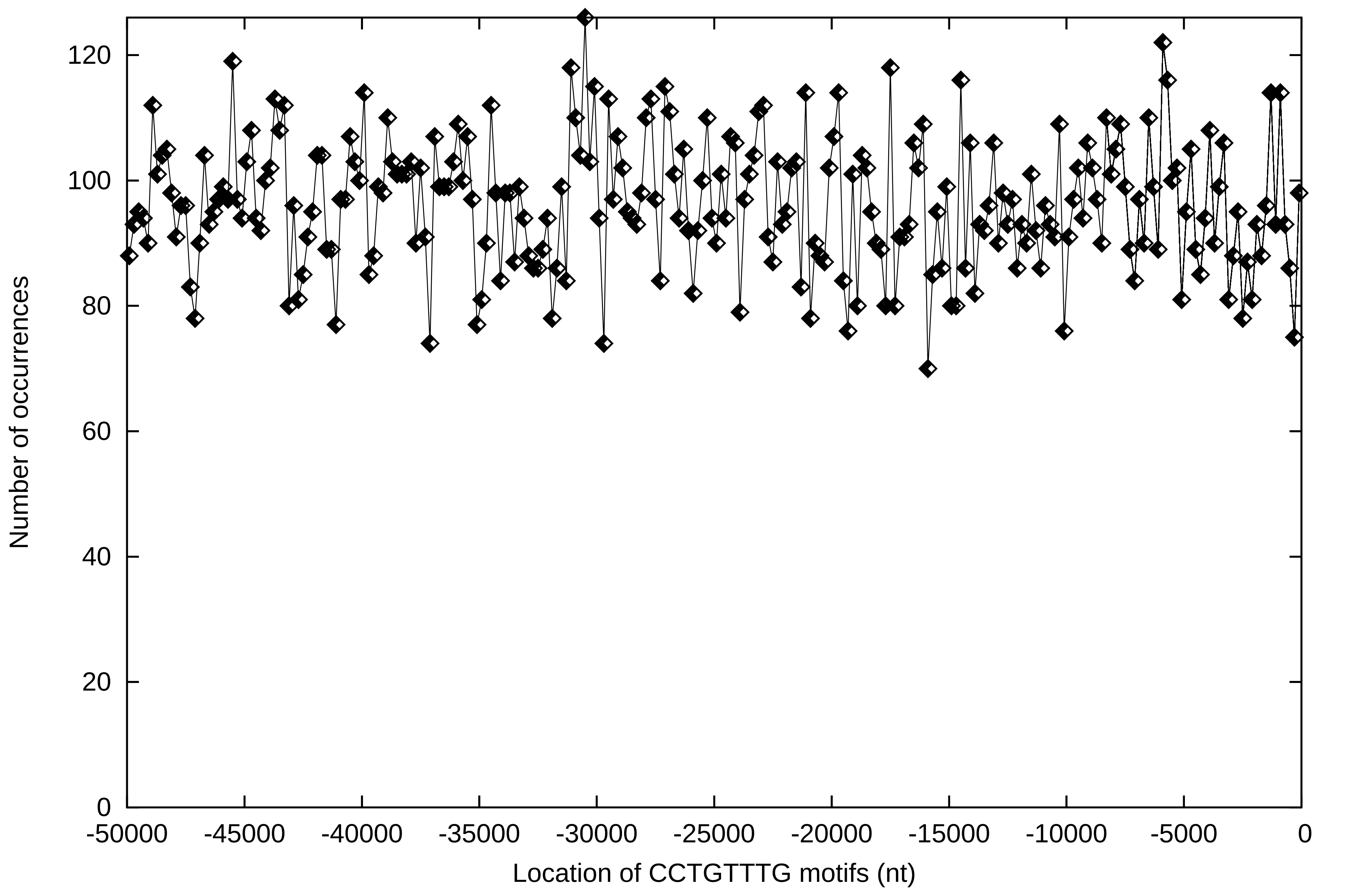

Number of occurrences
0
20
40
60
80
100
120
-50000
-45000
-40000
-35000
-30000
-25000
-20000
-15000
-10000
-5000
0
Location of CCTGTTTG motifs (nt)

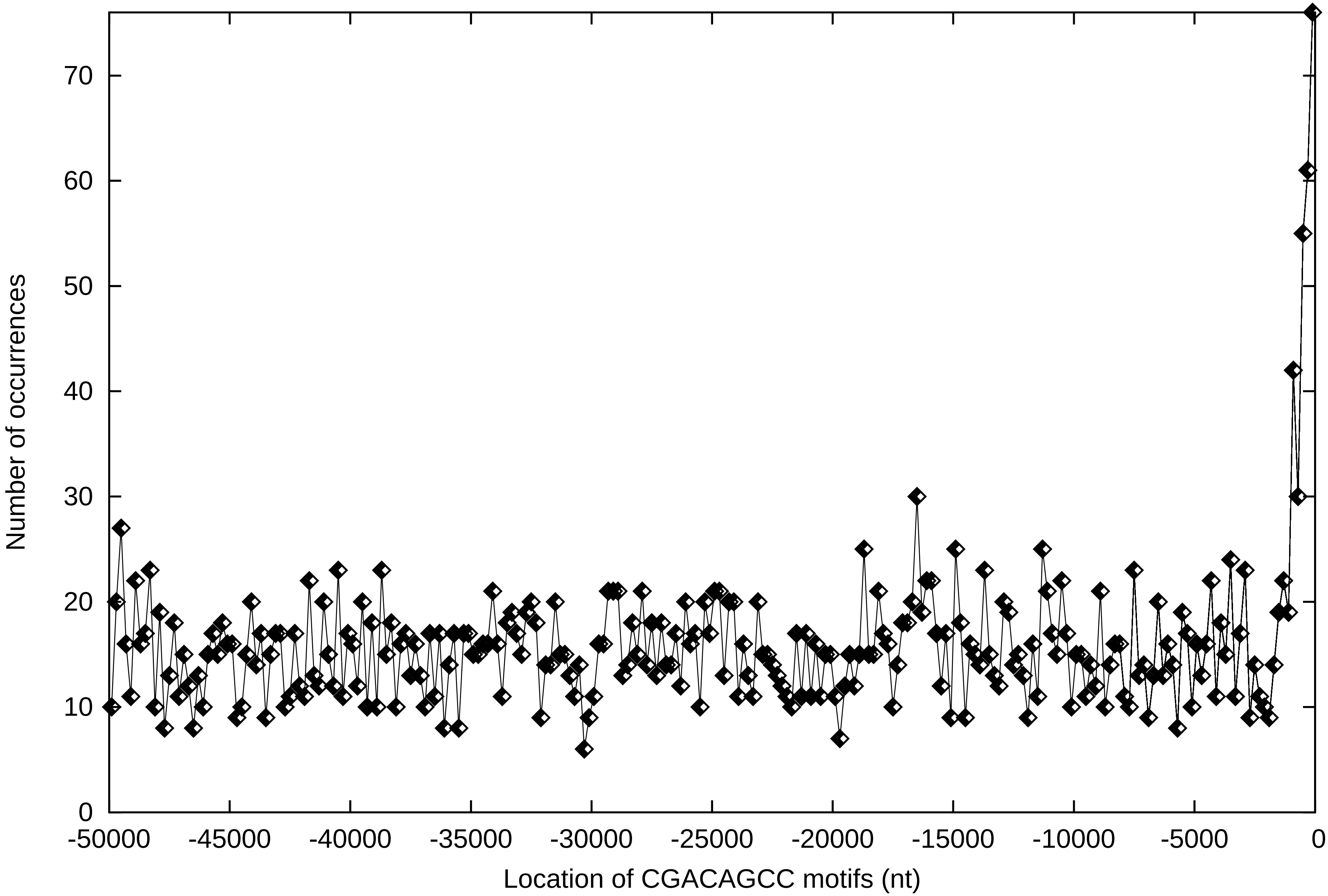

Number of occurrences
Location of CGACAGCC motifs (nt)
0
10
20
30
40
50
60
70
-50000
-45000
-40000
-35000
-30000
-25000
-20000
-15000
-10000
-5000
0

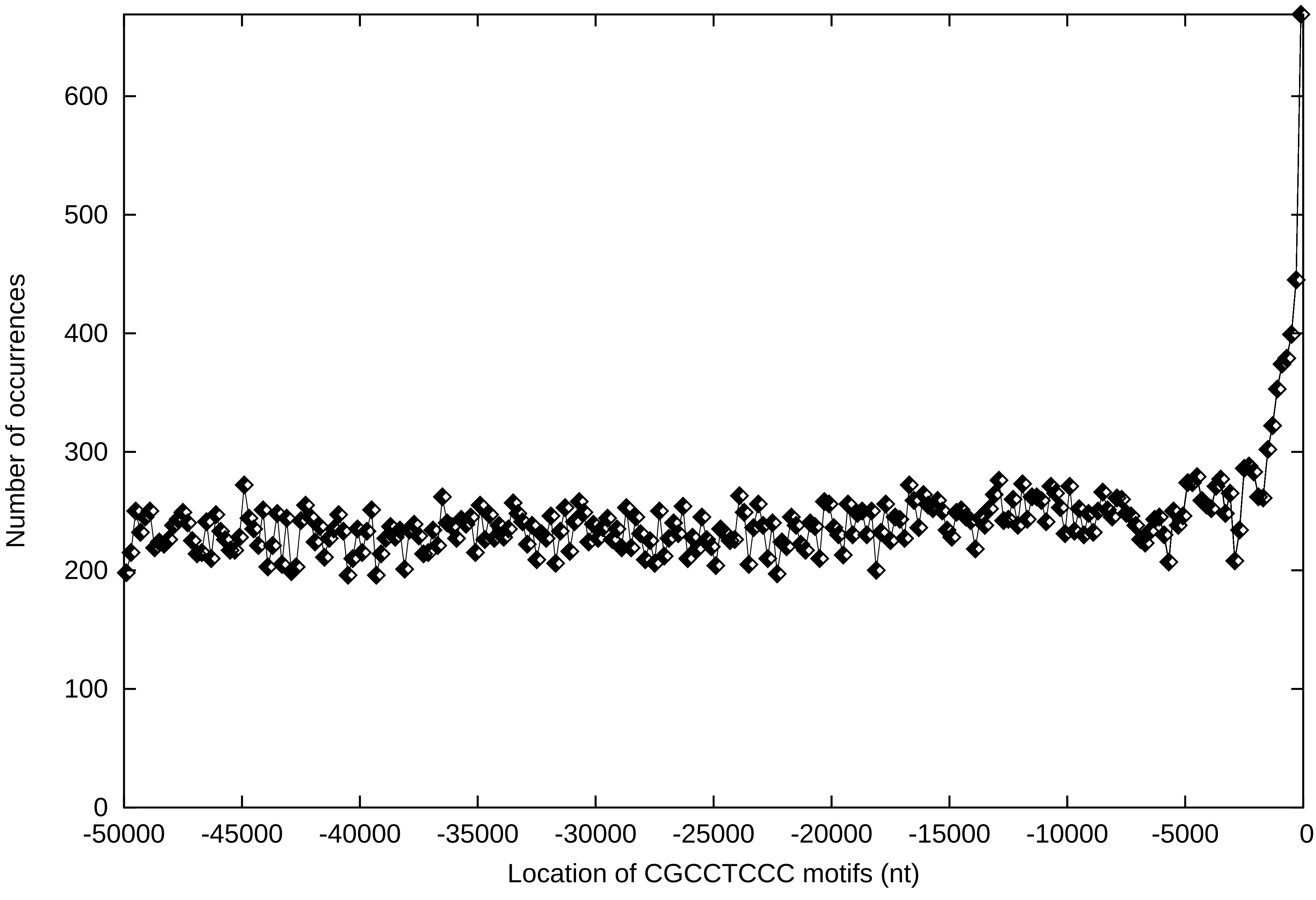

Number of occurrences
Location of CGCCTCCC motifs (nt)
0
100
200
300
400
500
600
-50000
-45000
-40000
-35000
-30000
-25000
-20000
-15000
-10000
-5000
0

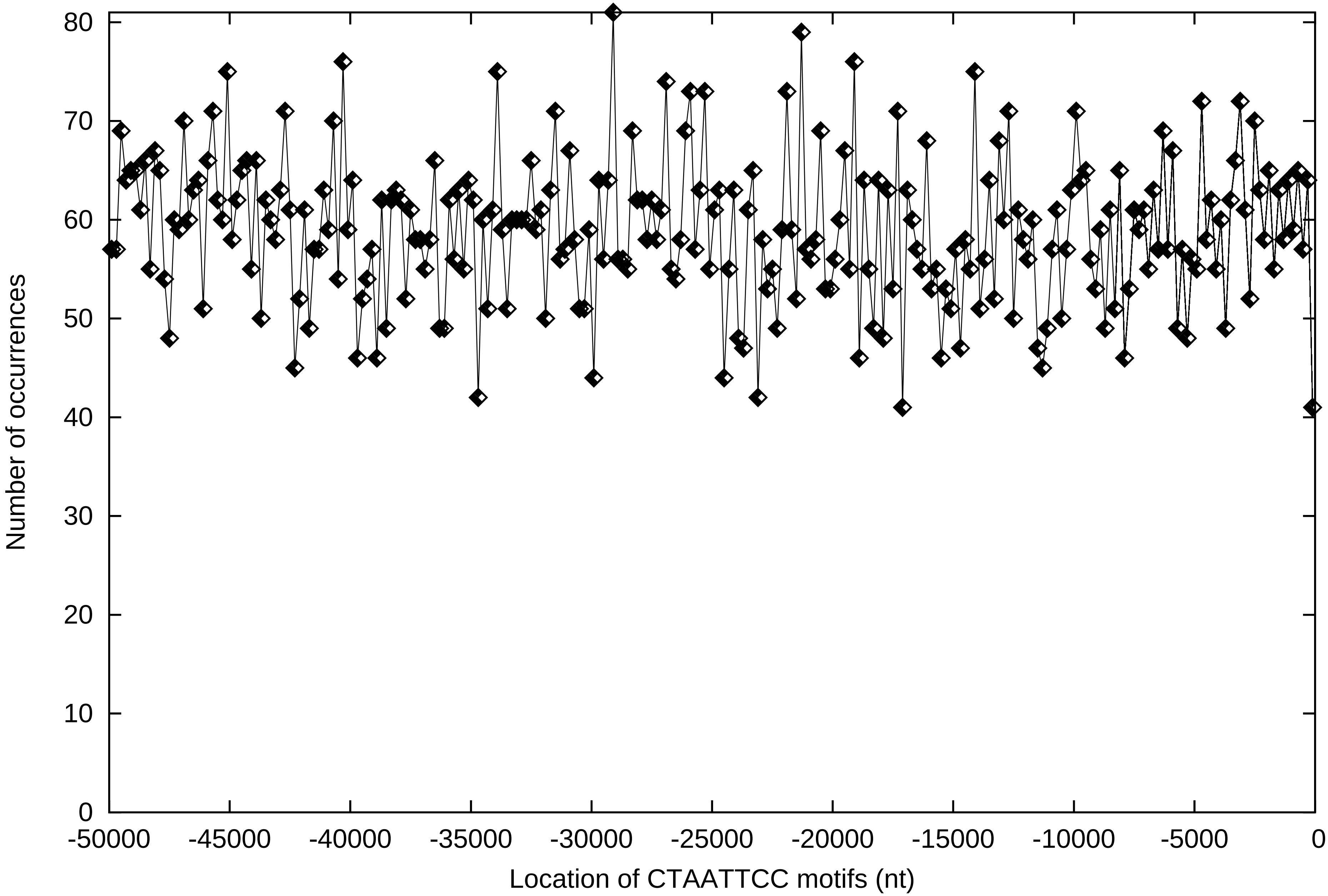

Number of occurrences
Location of CTAATTCC motifs (nt)
0
10
20
30
40
50
60
70
80
-50000
-45000
-40000
-35000
-30000
-25000
-20000
-15000
-10000
-5000
0

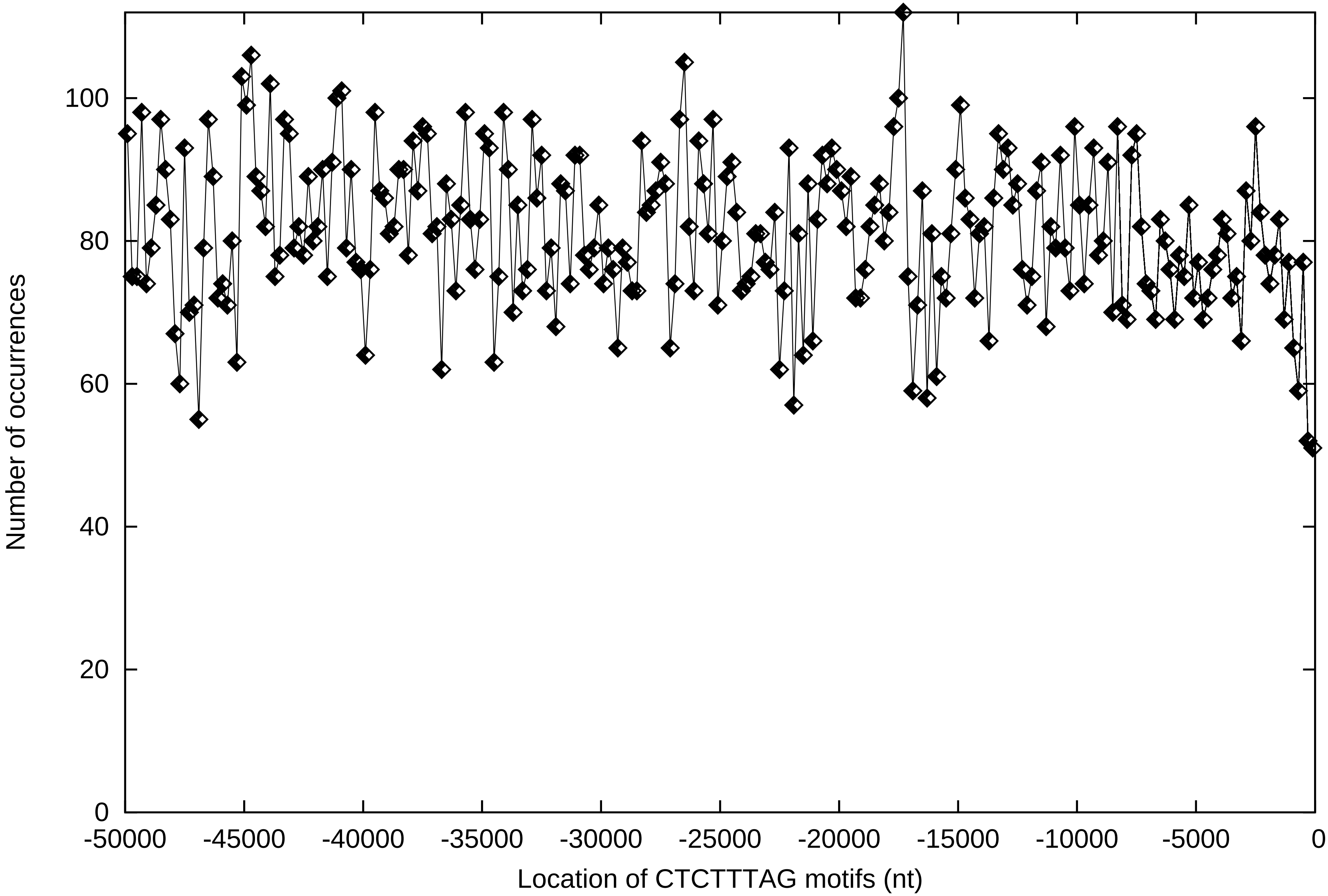

Number of occurrences
0
20
40
60
80
100
-50000
-45000
-40000
-35000
-30000
-25000
-20000
-15000
-10000
-5000
0
Location of CTCTTTAG motifs (nt)

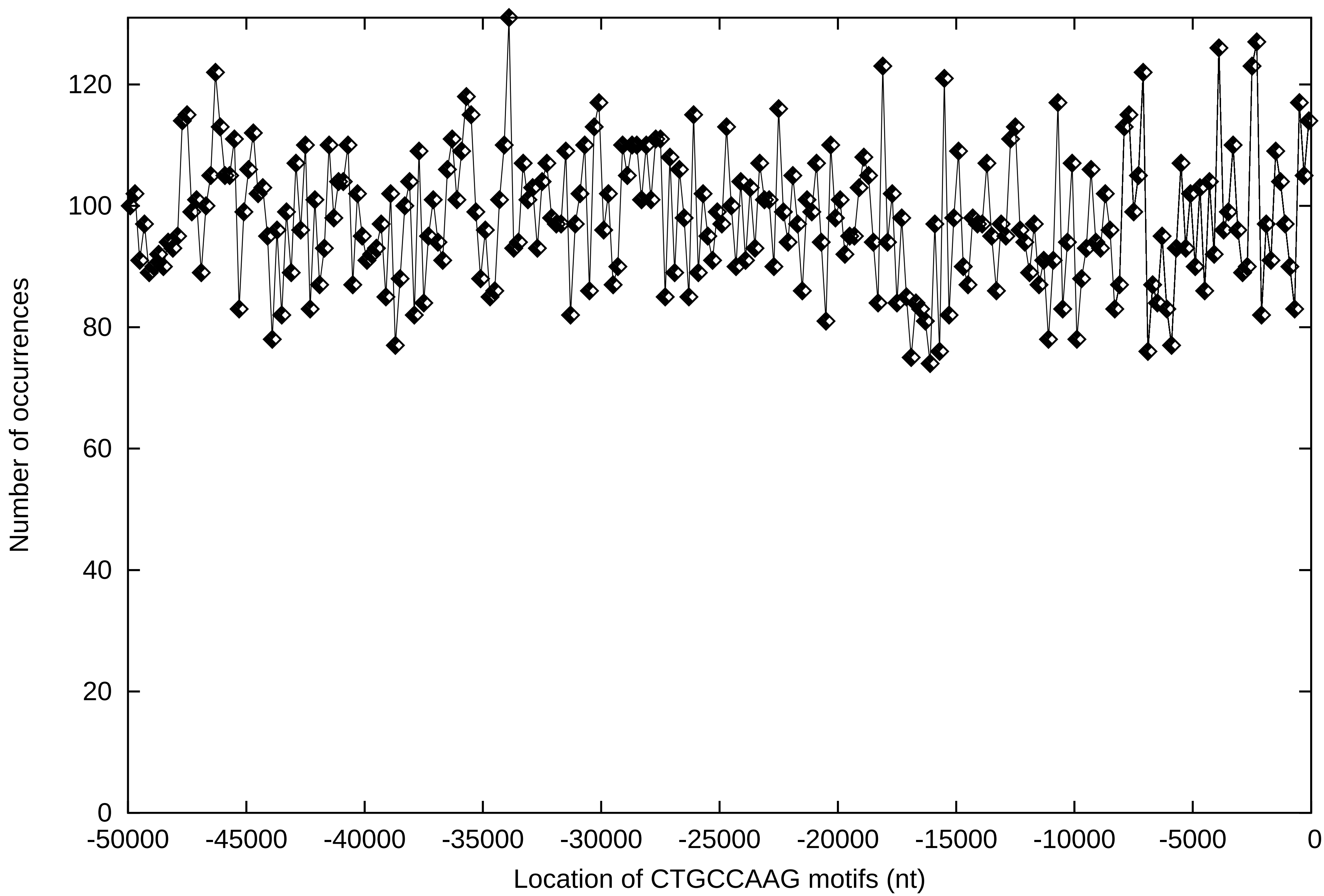

Number of occurrences
0
20
40
60
80
100
120
-50000
-45000
-40000
-35000
-30000
-25000
-20000
-15000
-10000
-5000
0
Location of CTGCCAAG motifs (nt)

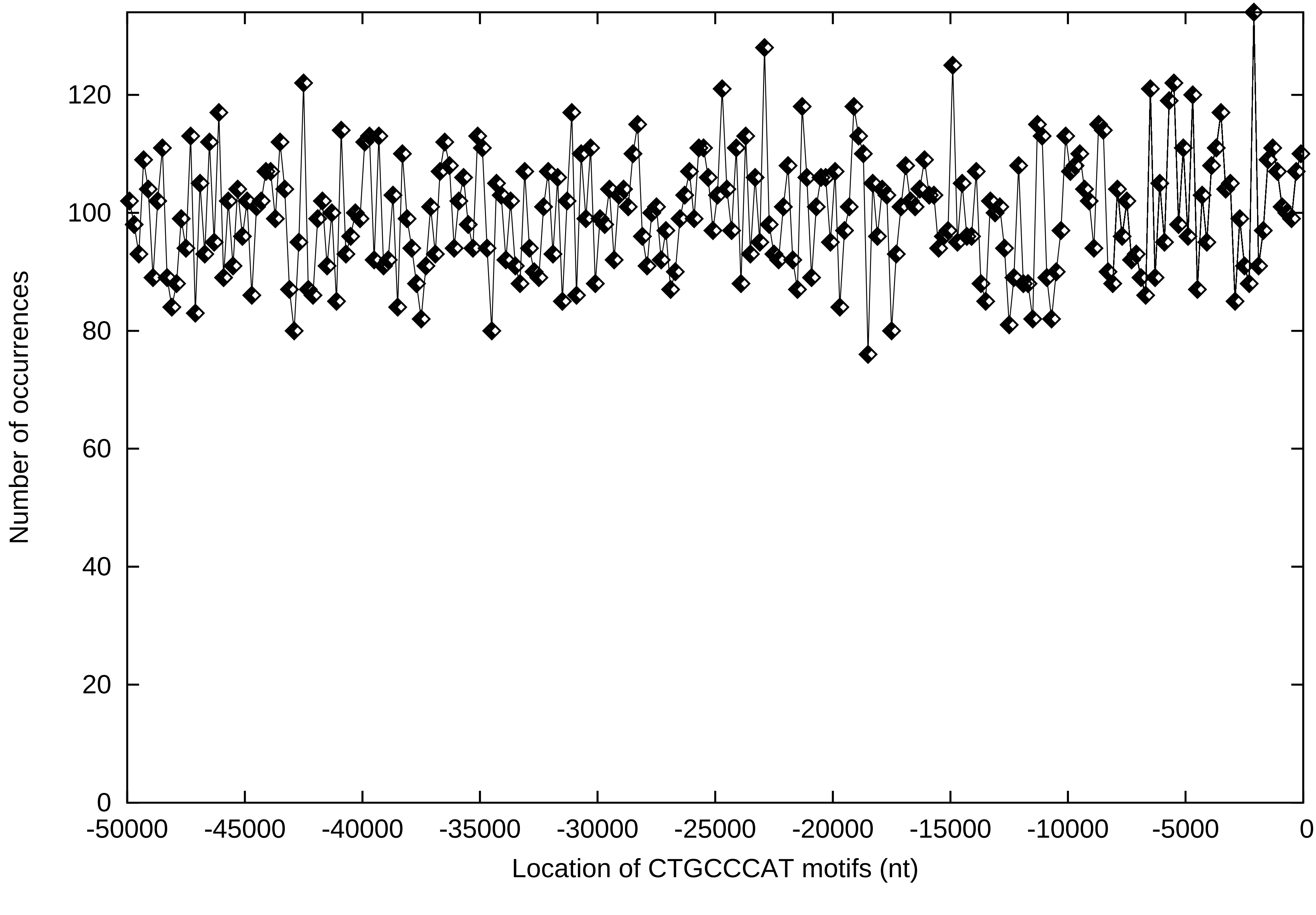

Number of occurrences
0
20
40
60
80
100
120
-50000
-45000
-40000
-35000
-30000
-25000
-20000
-15000
-10000
-5000
0
Location of CTGCCCAT motifs (nt)

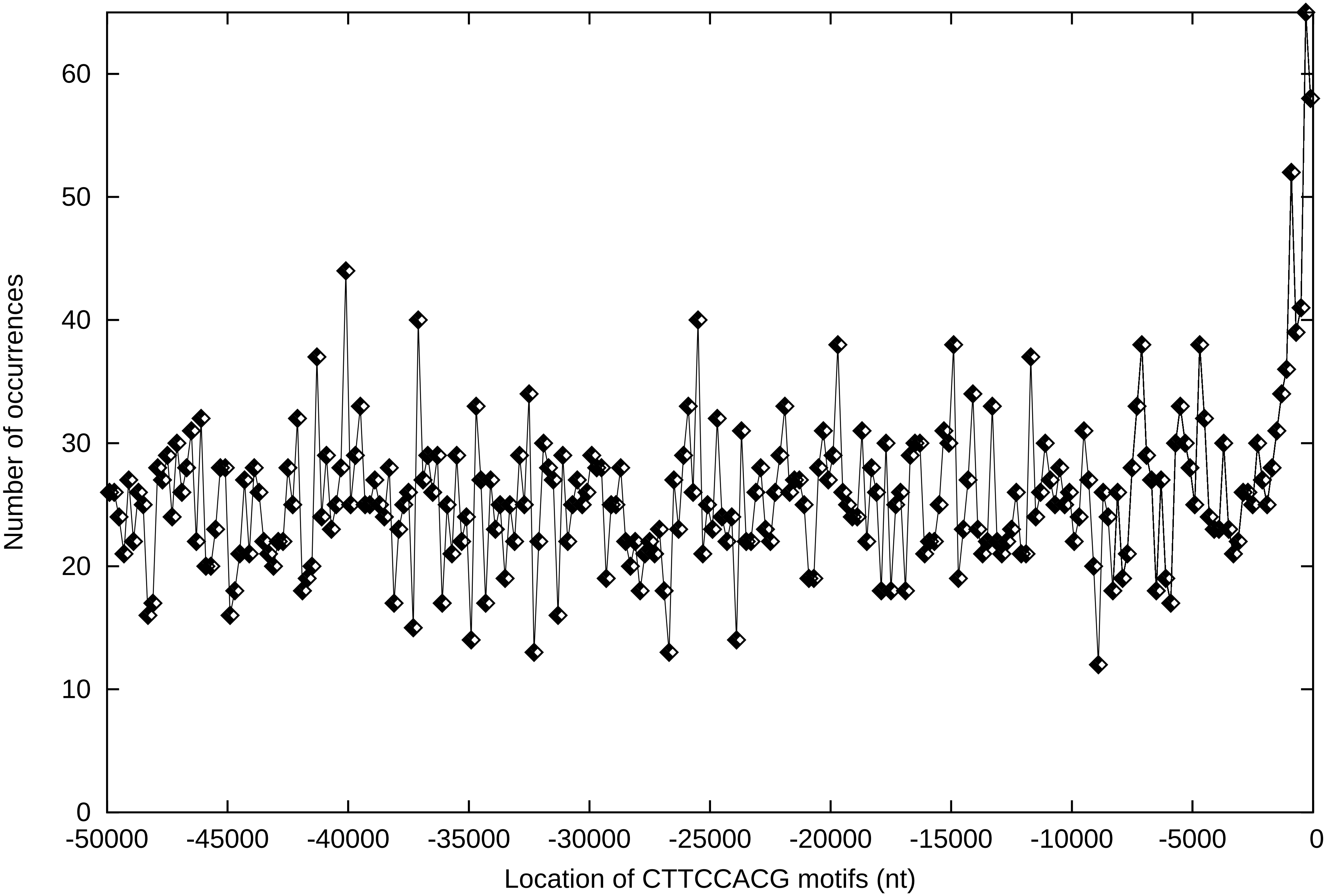

Number of occurrences
Location of CTTCCACG motifs (nt)
0
10
20
30
40
50
60
-50000
-45000
-40000
-35000
-30000
-25000
-20000
-15000
-10000
-5000
0

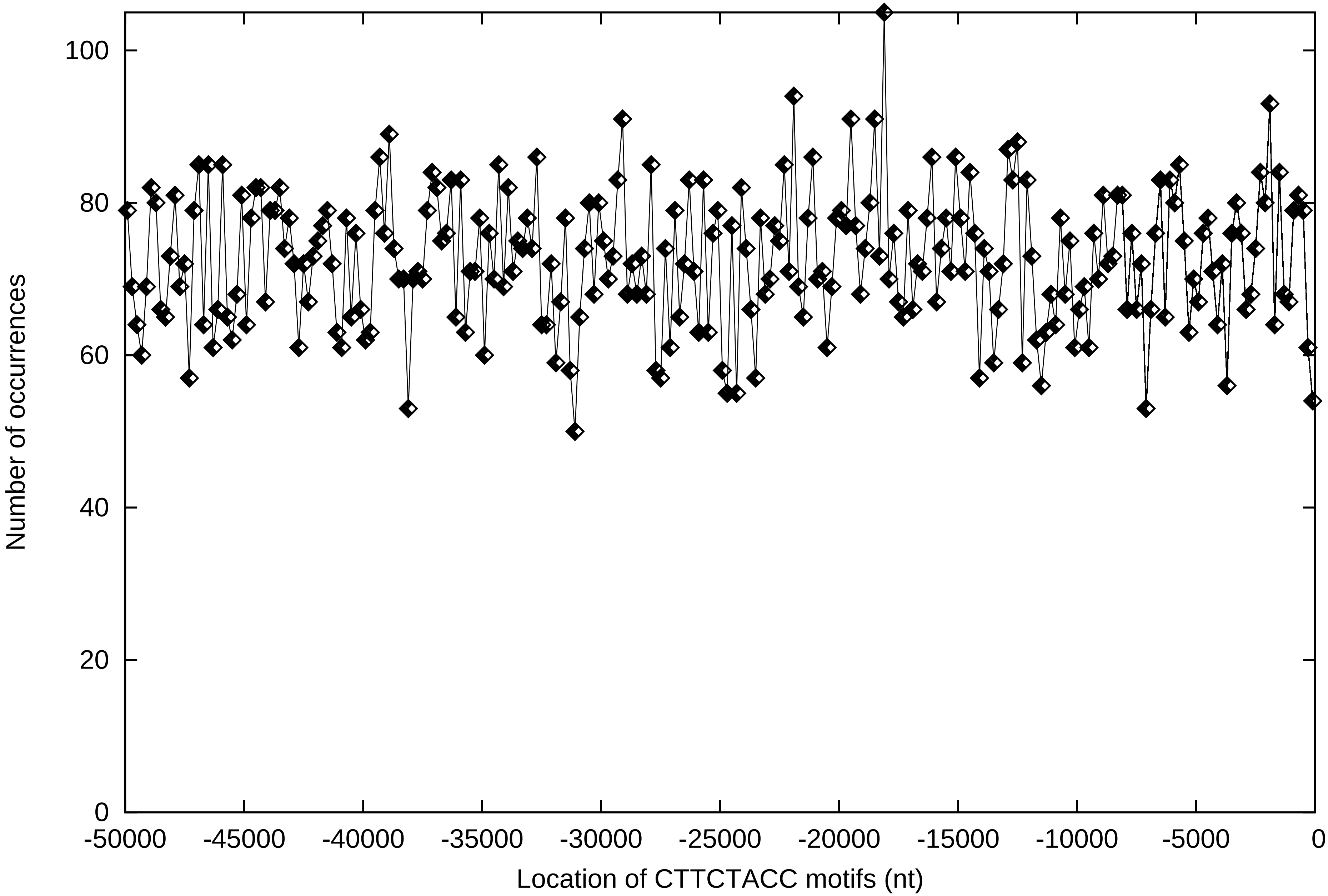

Number of occurrences
0
20
40
60
80
100
-50000
-45000
-40000
-35000
-30000
-25000
-20000
-15000
-10000
-5000
0
Location of CTTCTACC motifs (nt)

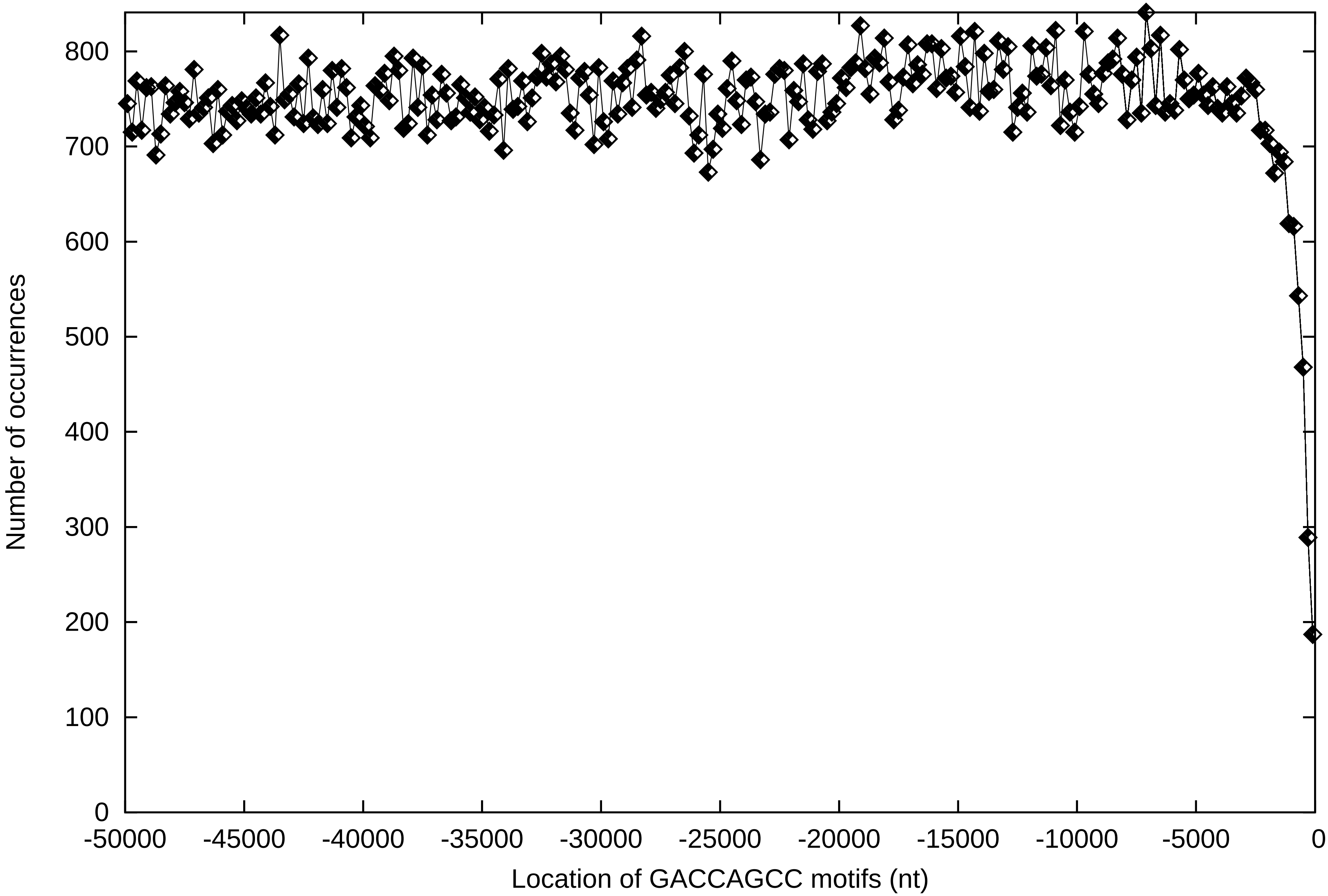

Number of occurrences
0
100
200
300
400
500
600
700
800
-50000
-45000
-40000
-35000
-30000
-25000
-20000
-15000
-10000
-5000
0
Location of GACCAGCC motifs (nt)

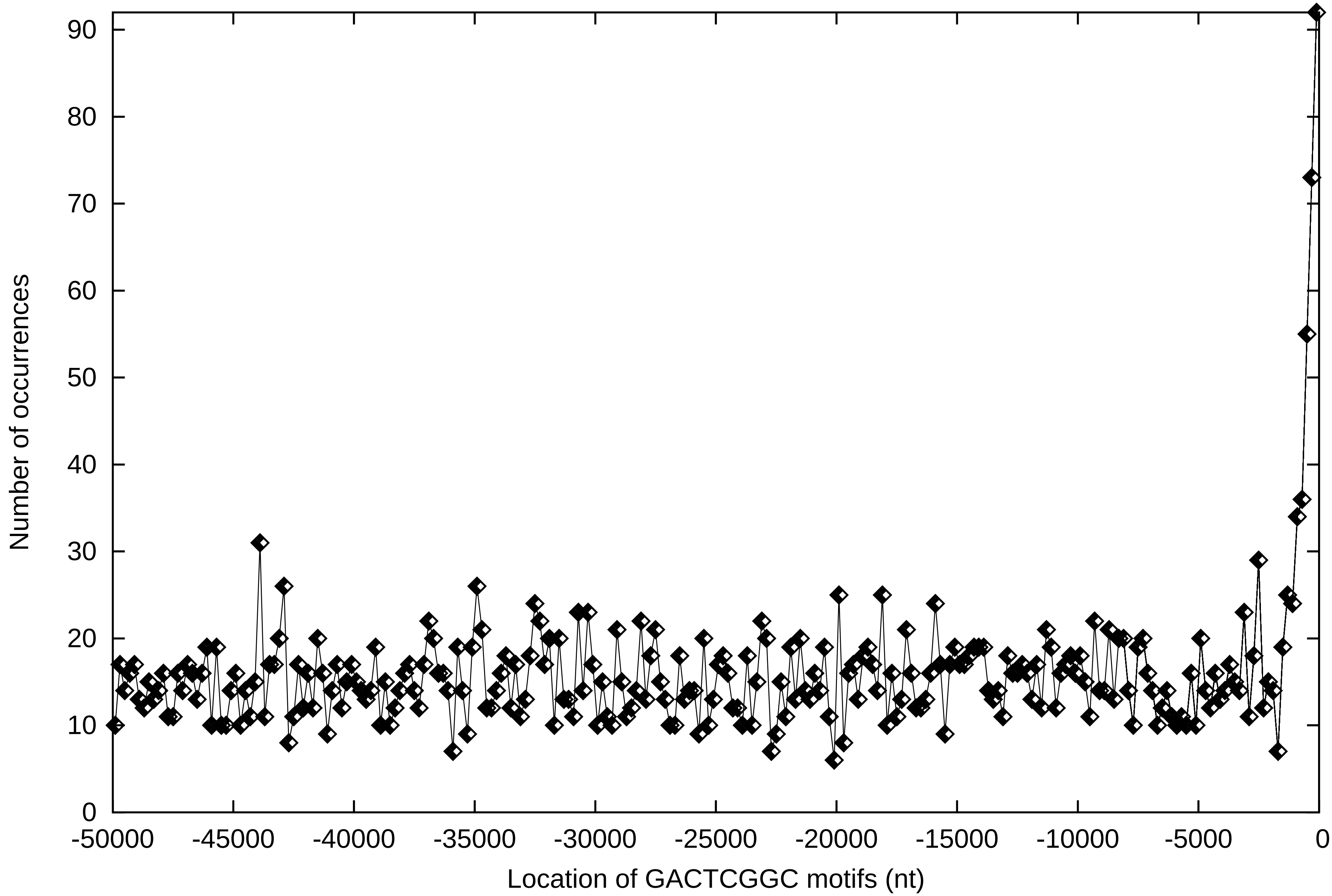

Number of occurrences
90
80
70
60
50
40
30
20
10
0
-50000
-45000
-40000
-35000
-30000
-25000
-20000
-15000
-10000
-5000
0
Location of GACTCGGC motifs (nt)

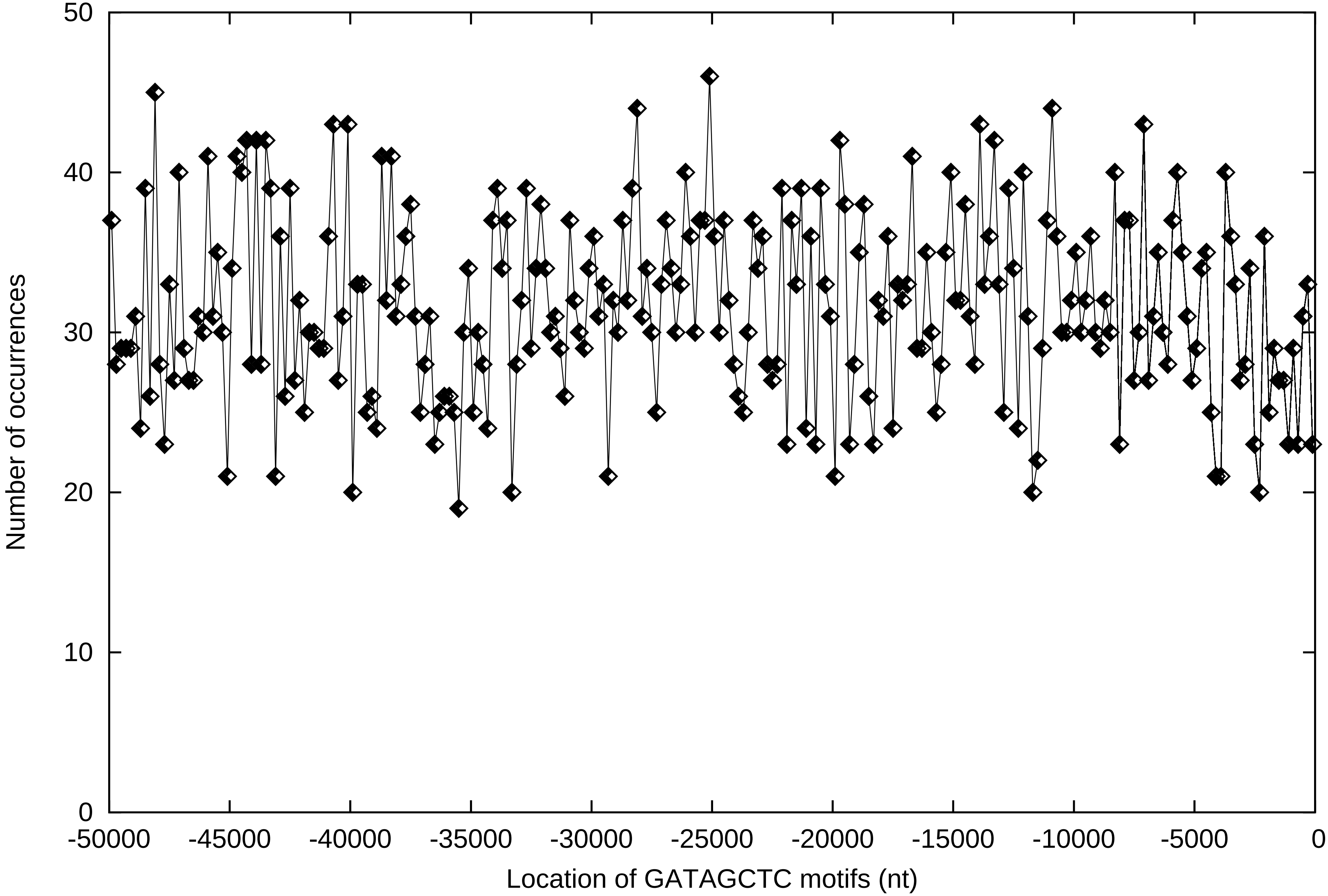

Number of occurrences
Location of GATAGCTC motifs (nt)
0
10
20
30
40
50
-50000
-45000
-40000
-35000
-30000
-25000
-20000
-15000
-10000
-5000
0

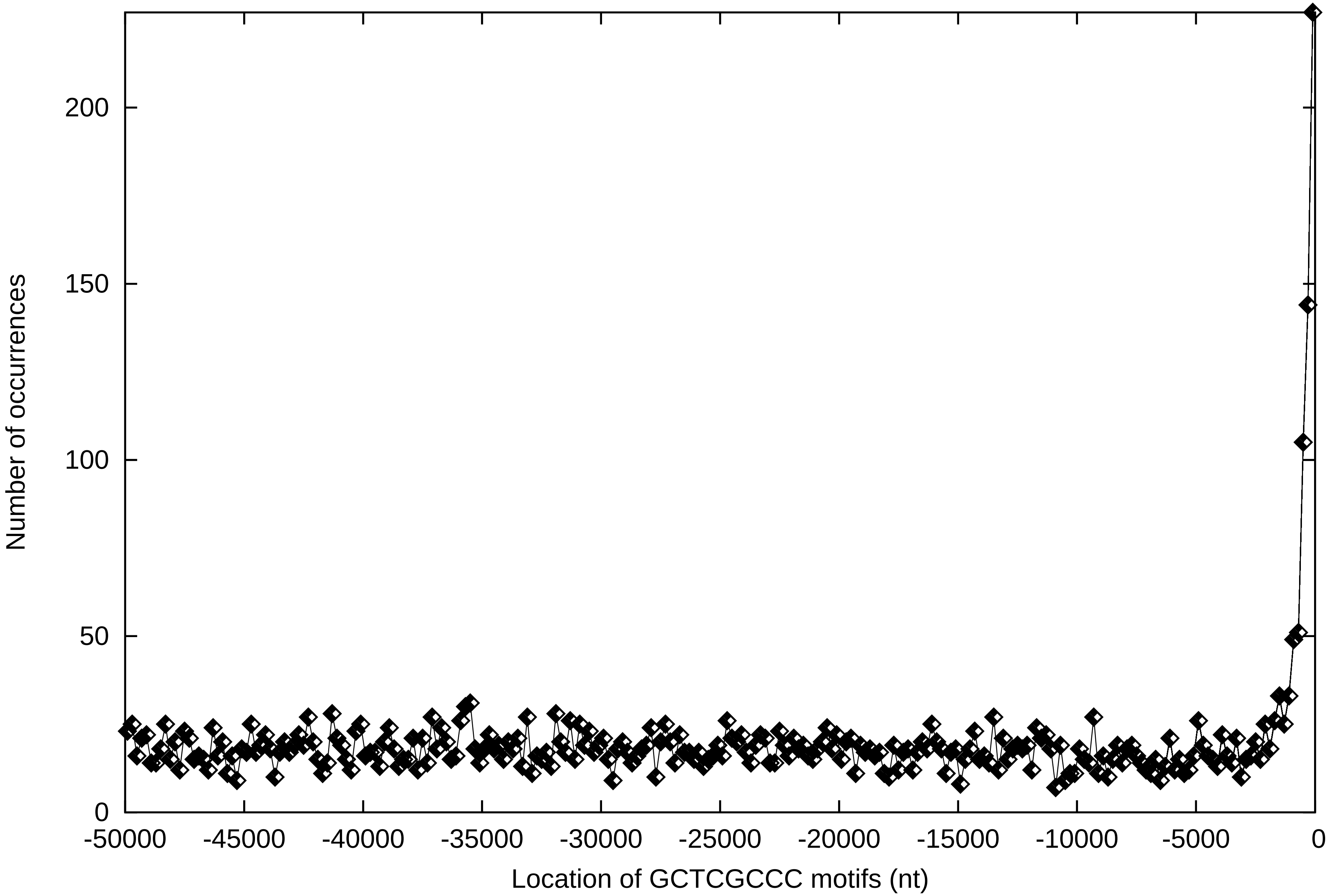

Number of occurrences
0
50
100
150
200
-50000
-45000
-40000
-35000
-30000
-25000
-20000
-15000
-10000
-5000
0
Location of GCTCGCCC motifs (nt)

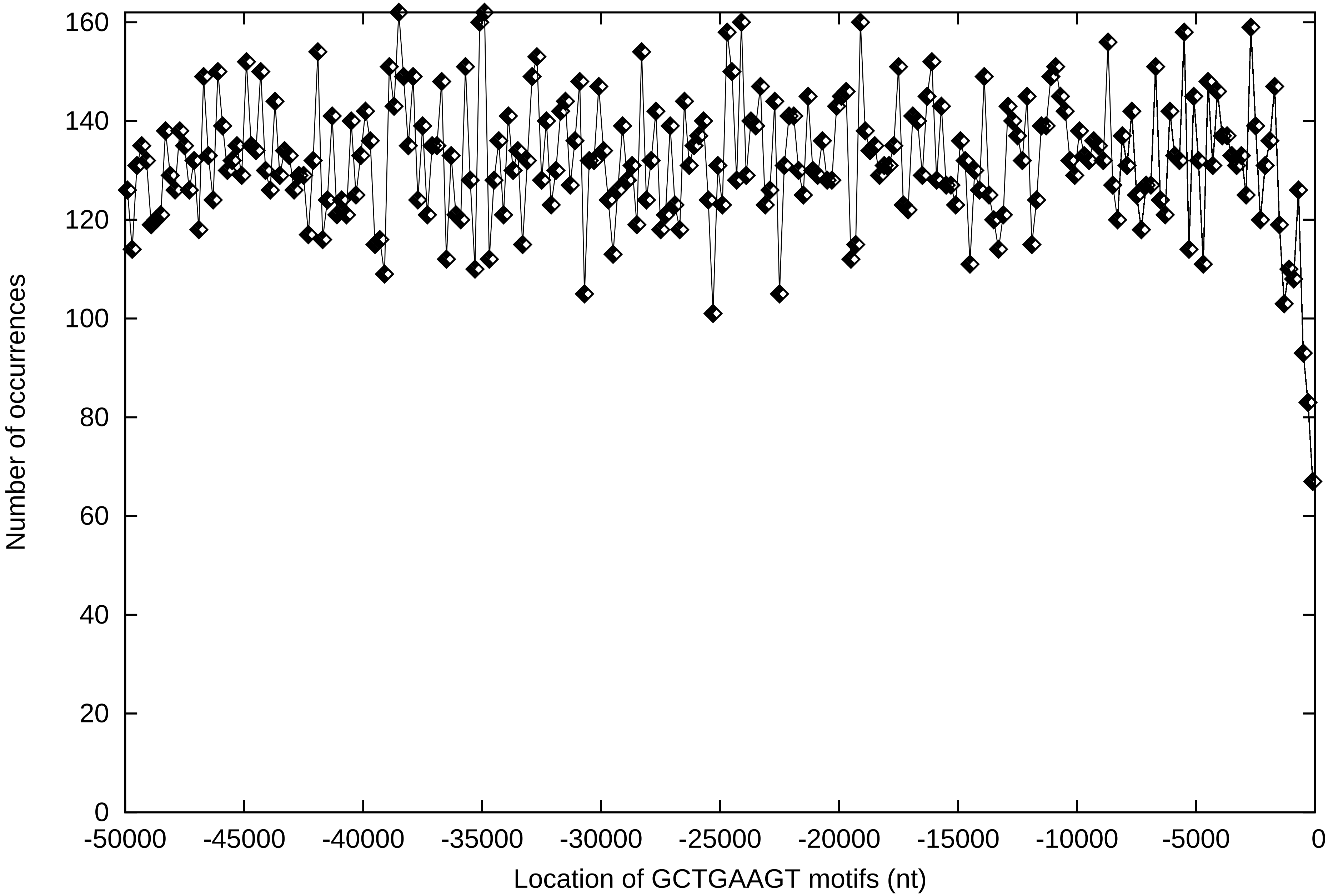

Number of occurrences
Location of GCTGAAGT motifs (nt)
0
20
40
60
80
100
120
140
160
-50000
-45000
-40000
-35000
-30000
-25000
-20000
-15000
-10000
-5000
0

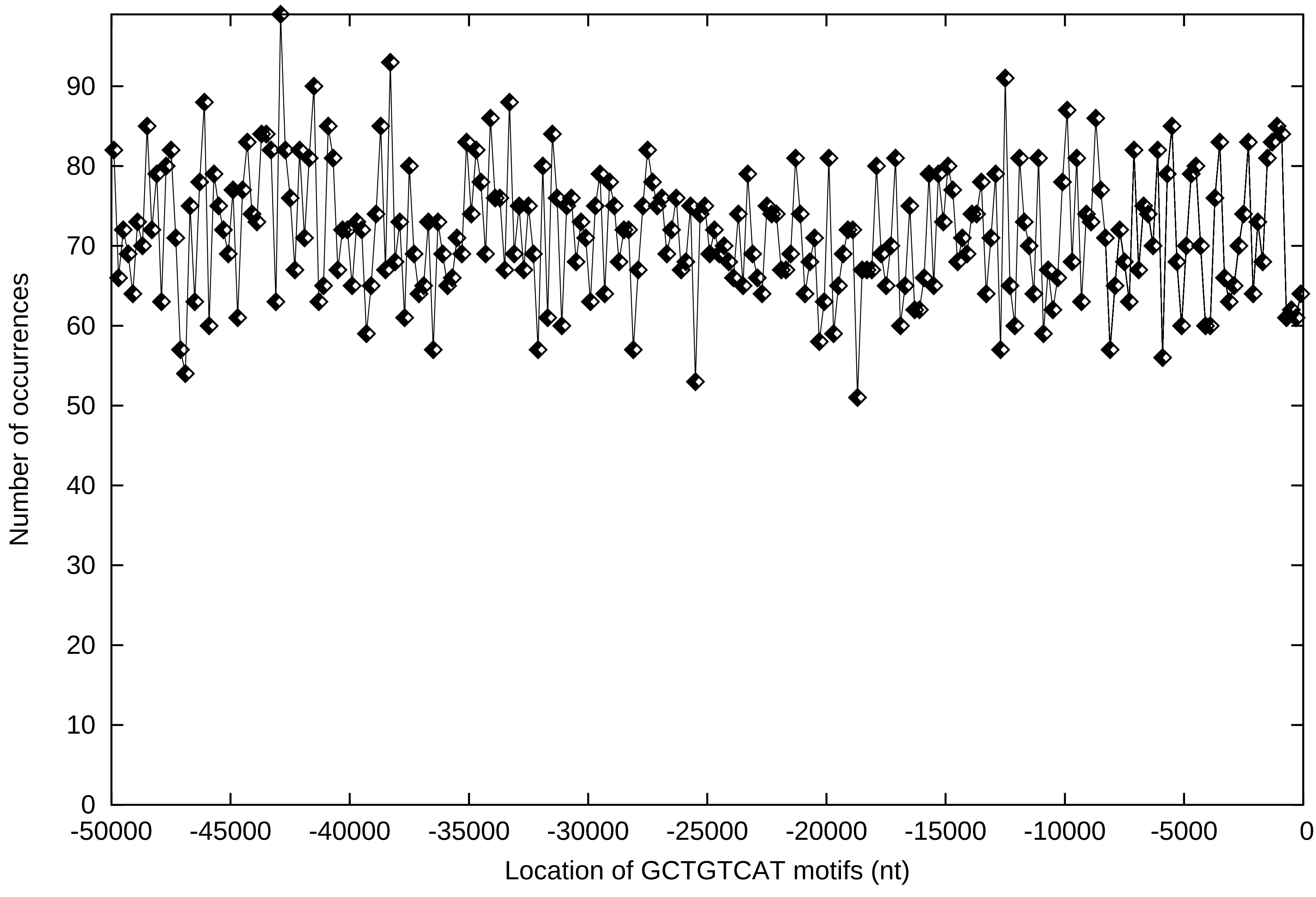

Number of occurrences
0
10
20
30
40
50
60
70
80
90
-50000
-45000
-40000
-35000
-30000
-25000
-20000
-15000
-10000
-5000
0
Location of GCTGTCAT motifs (nt)

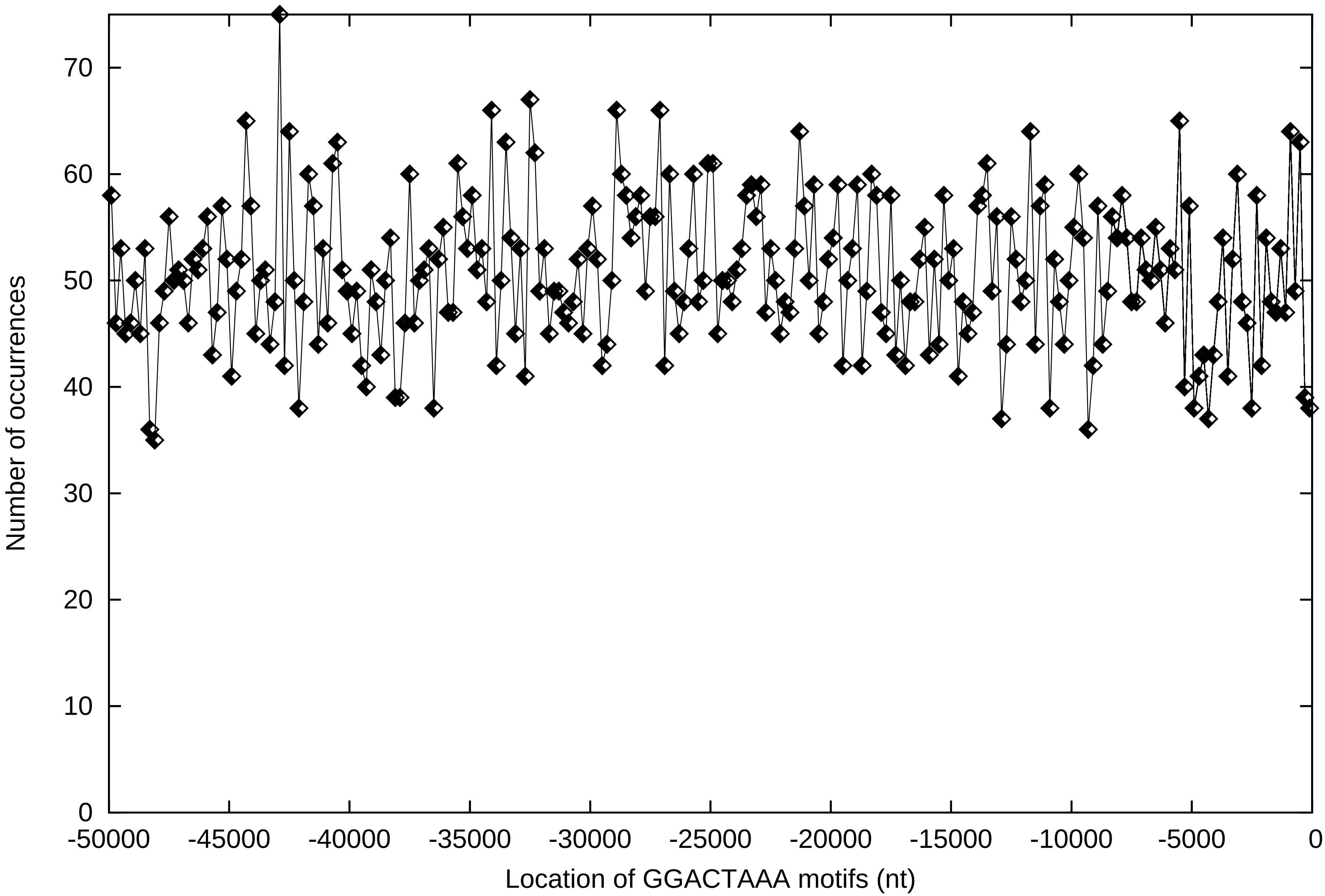

Number of occurrences
Location of GGACTAAA motifs (nt)
0
10
20
30
40
50
60
70
-50000
-45000
-40000
-35000
-30000
-25000
-20000
-15000
-10000
-5000
0

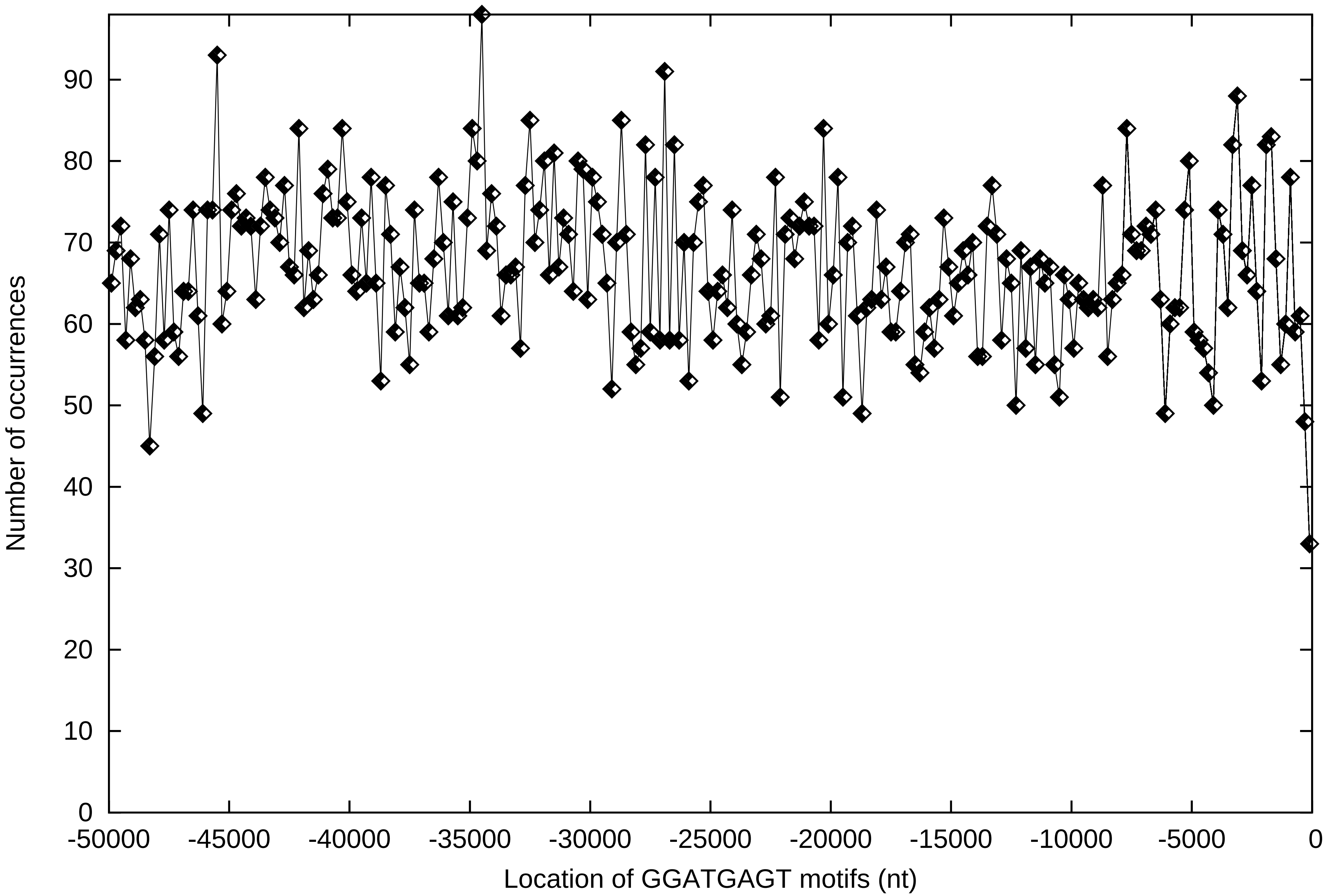
Number of occurrences
0
10
20
30
40
50
60
70
80
90
-50000
-45000
-40000
-35000
-30000
-25000
-20000
-15000
-10000
-5000
0
Location of GGATGAGT motifs (nt)

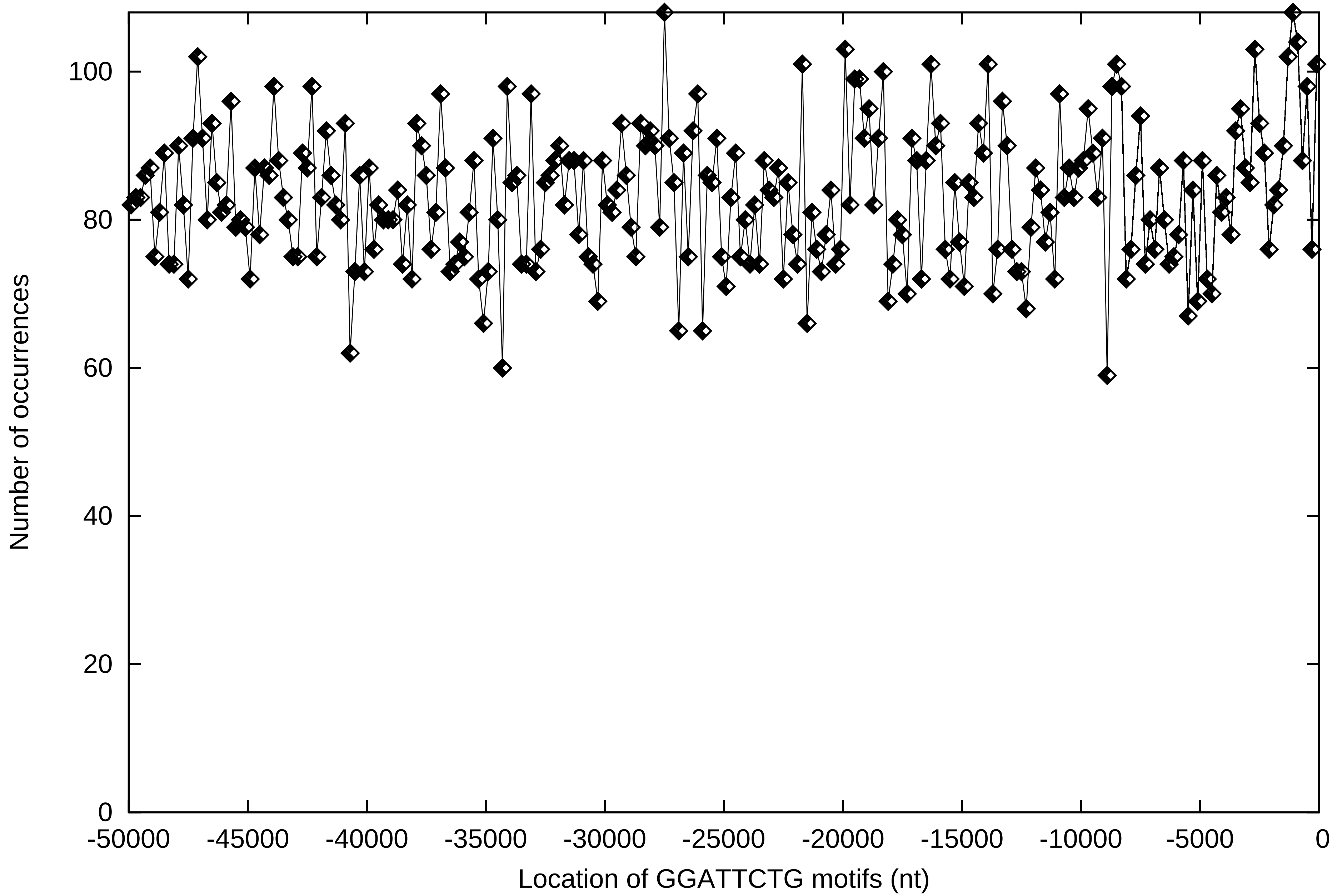

Number of occurrences
0
20
40
60
80
100
-50000
-45000
-40000
-35000
-30000
-25000
-20000
-15000
-10000
-5000
0
Location of GGATTCTG motifs (nt)

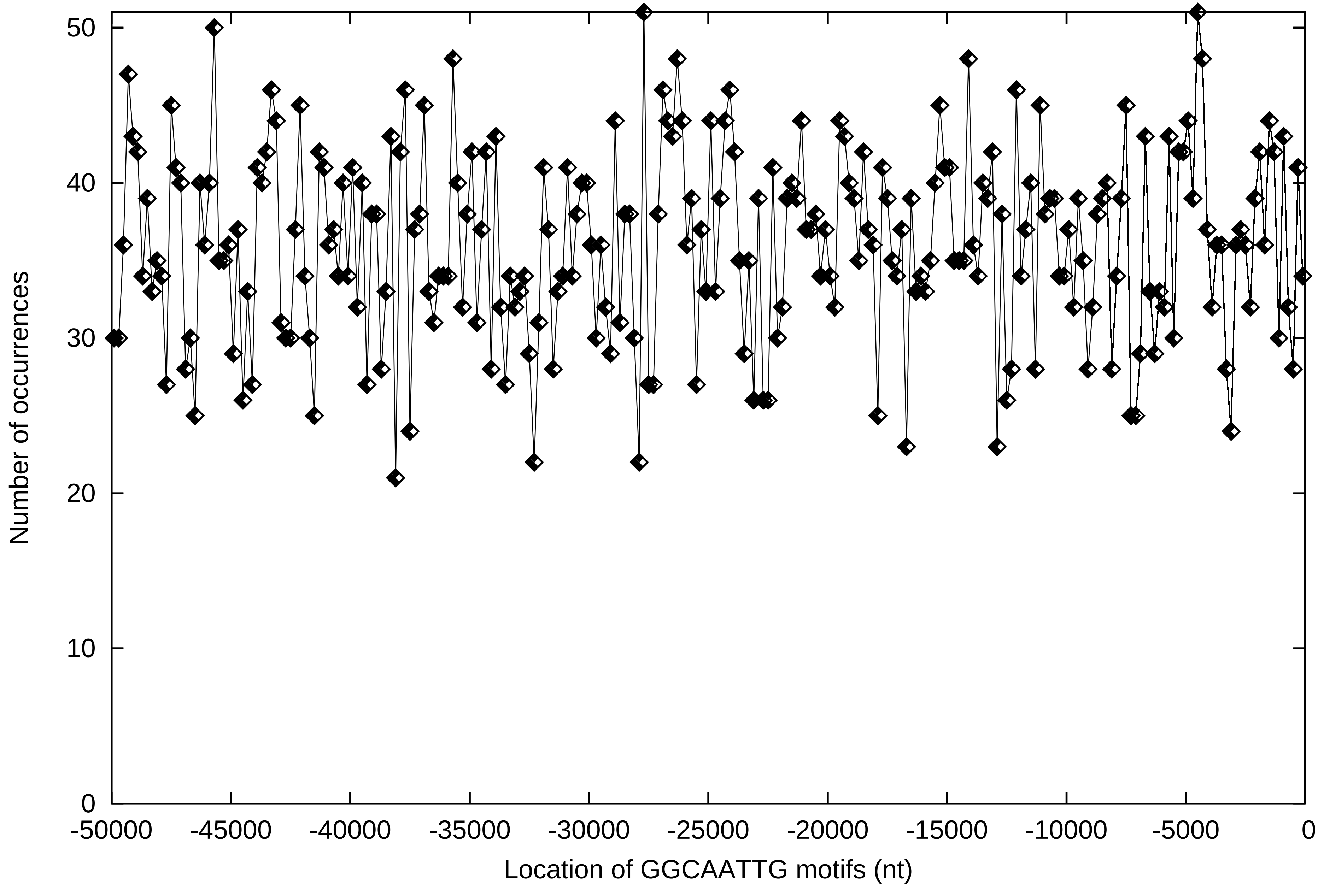

Number of occurrences
Location of GGCAATTG motifs (nt)
0
10
20
30
40
50
-50000
-45000
-40000
-35000
-30000
-25000
-20000
-15000
-10000
-5000
0

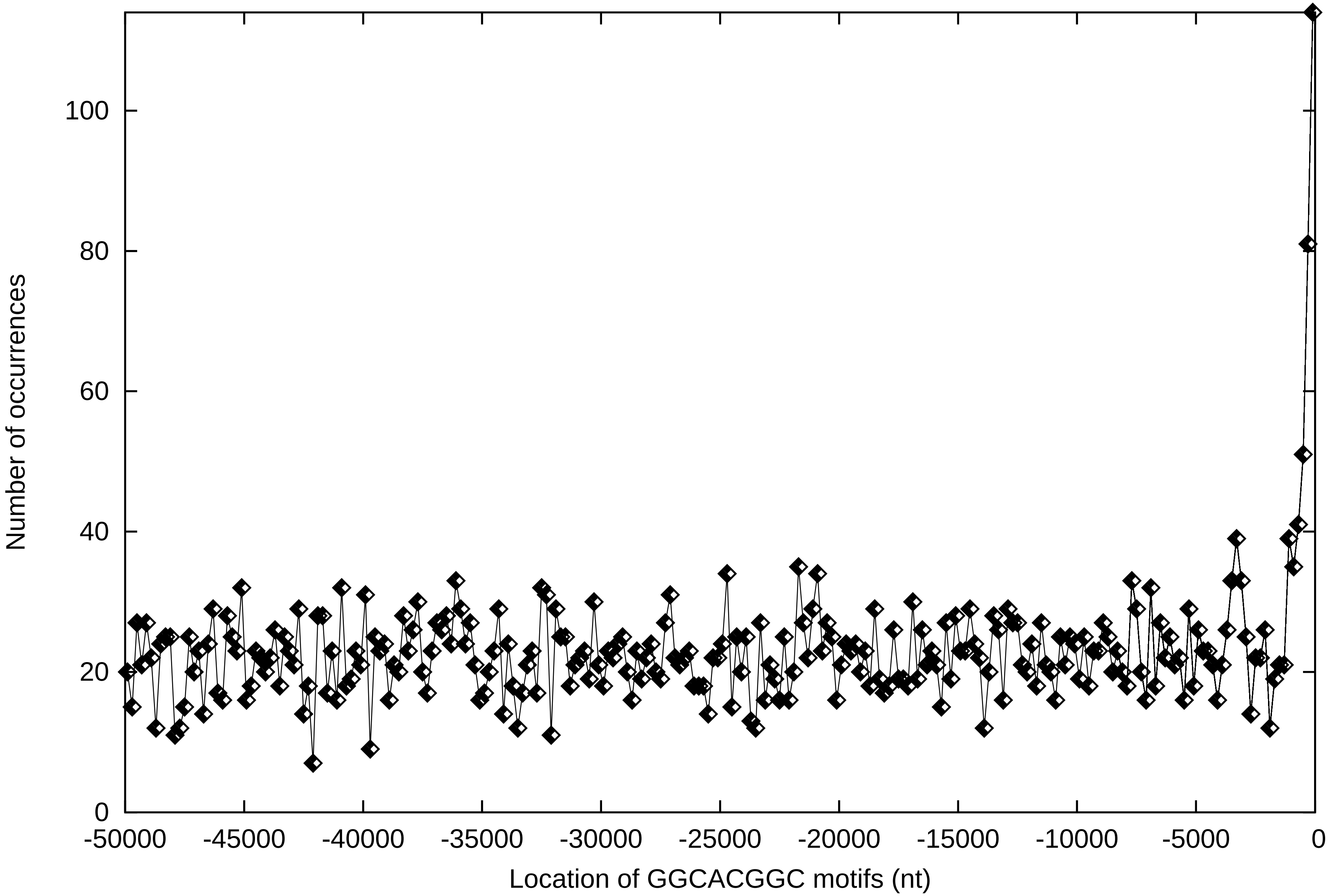

Number of occurrences
0
20
40
60
80
100
-50000
-45000
-40000
-35000
-30000
-25000
-20000
-15000
-10000
-5000
0
Location of GGCACGGC motifs (nt)

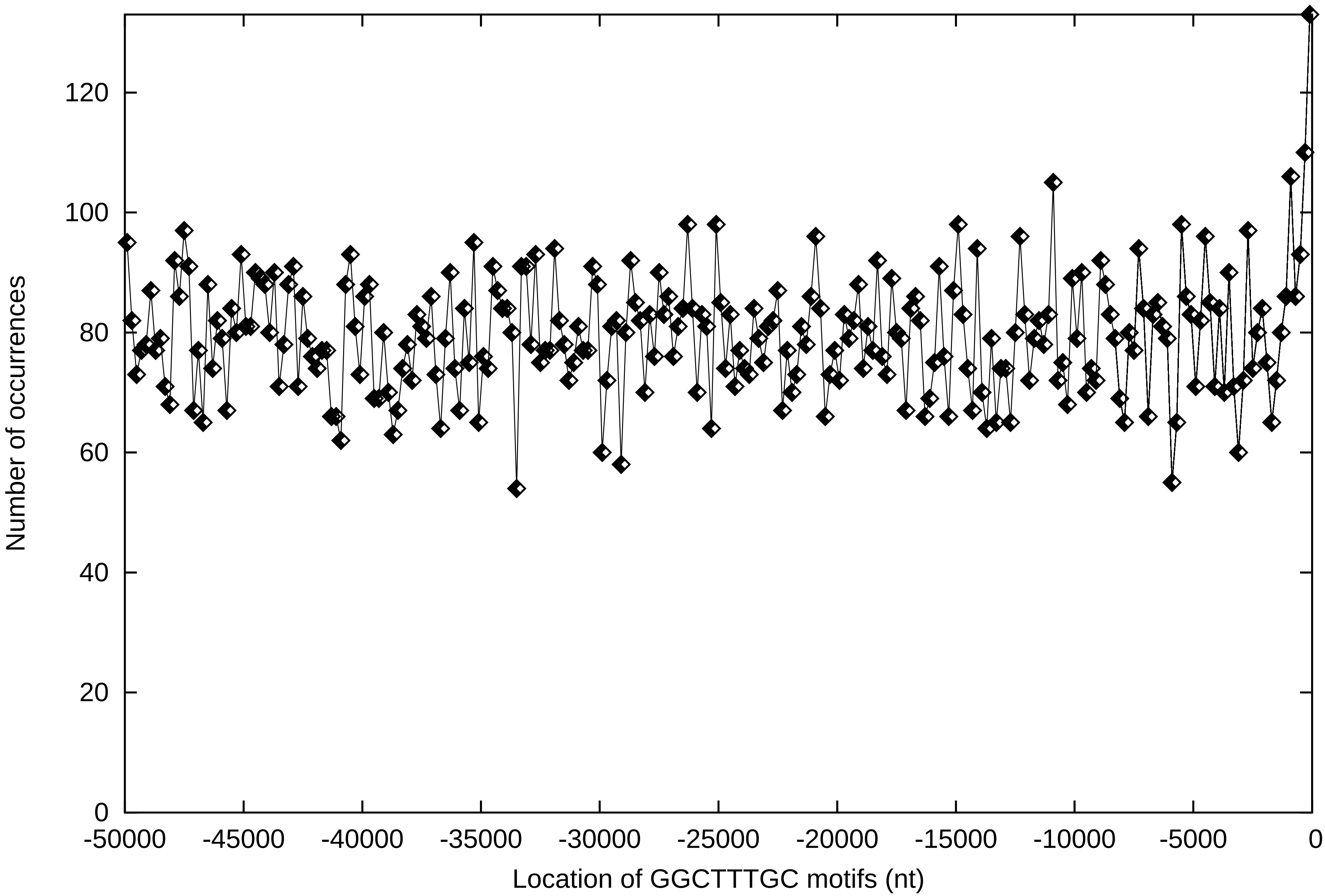

Number of occurrences
Location of GGCTTTGC motifs (nt)
0
20
40
60
80
100
120
-50000
-45000
-40000
-35000
-30000
-25000
-20000
-15000
-10000
-5000
0

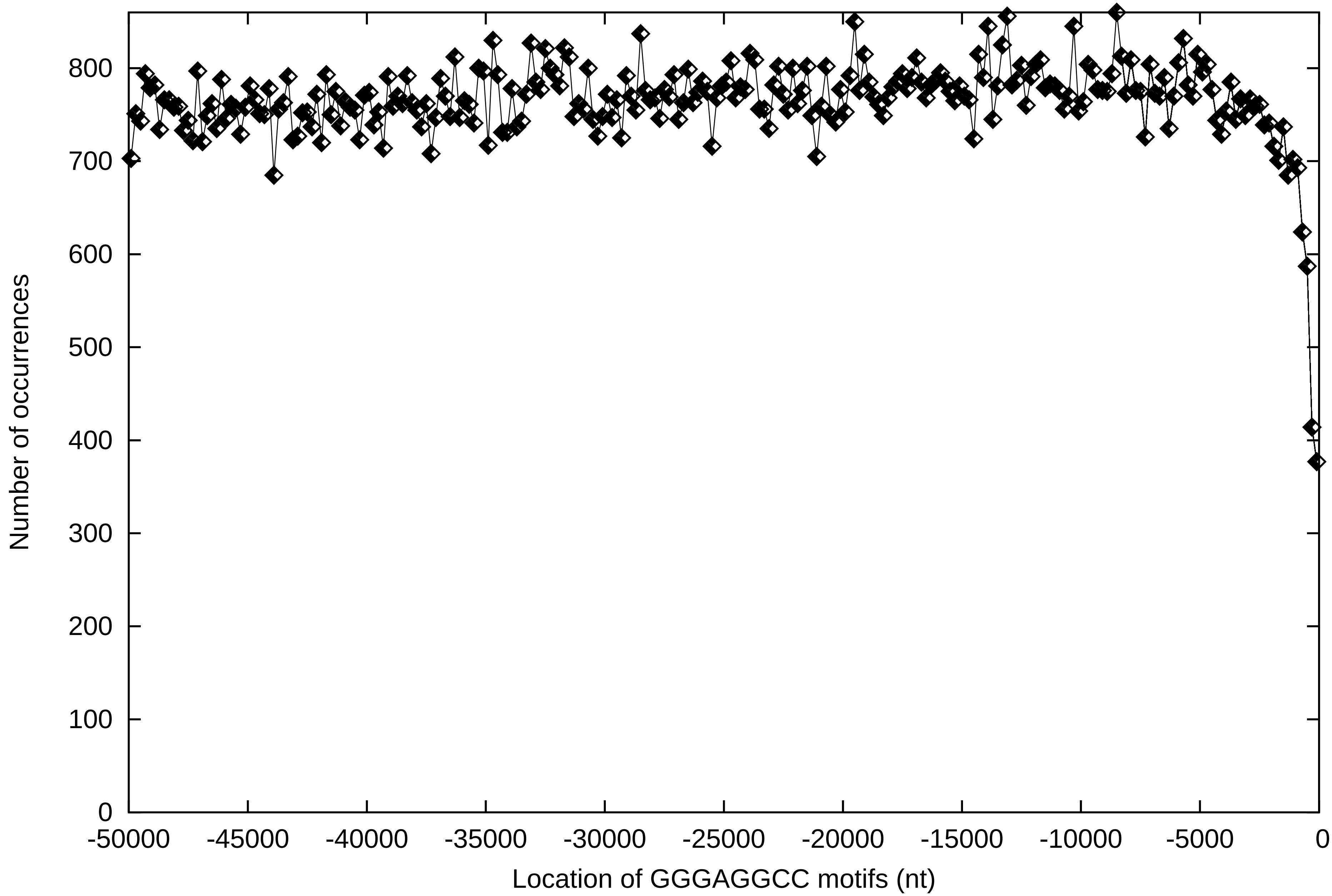

Number of occurrences
0
100
200
300
400
500
600
700
800
-50000
-45000
-40000
-35000
-30000
-25000
-20000
-15000
-10000
-5000
0
Location of GGGAGGCC motifs (nt)

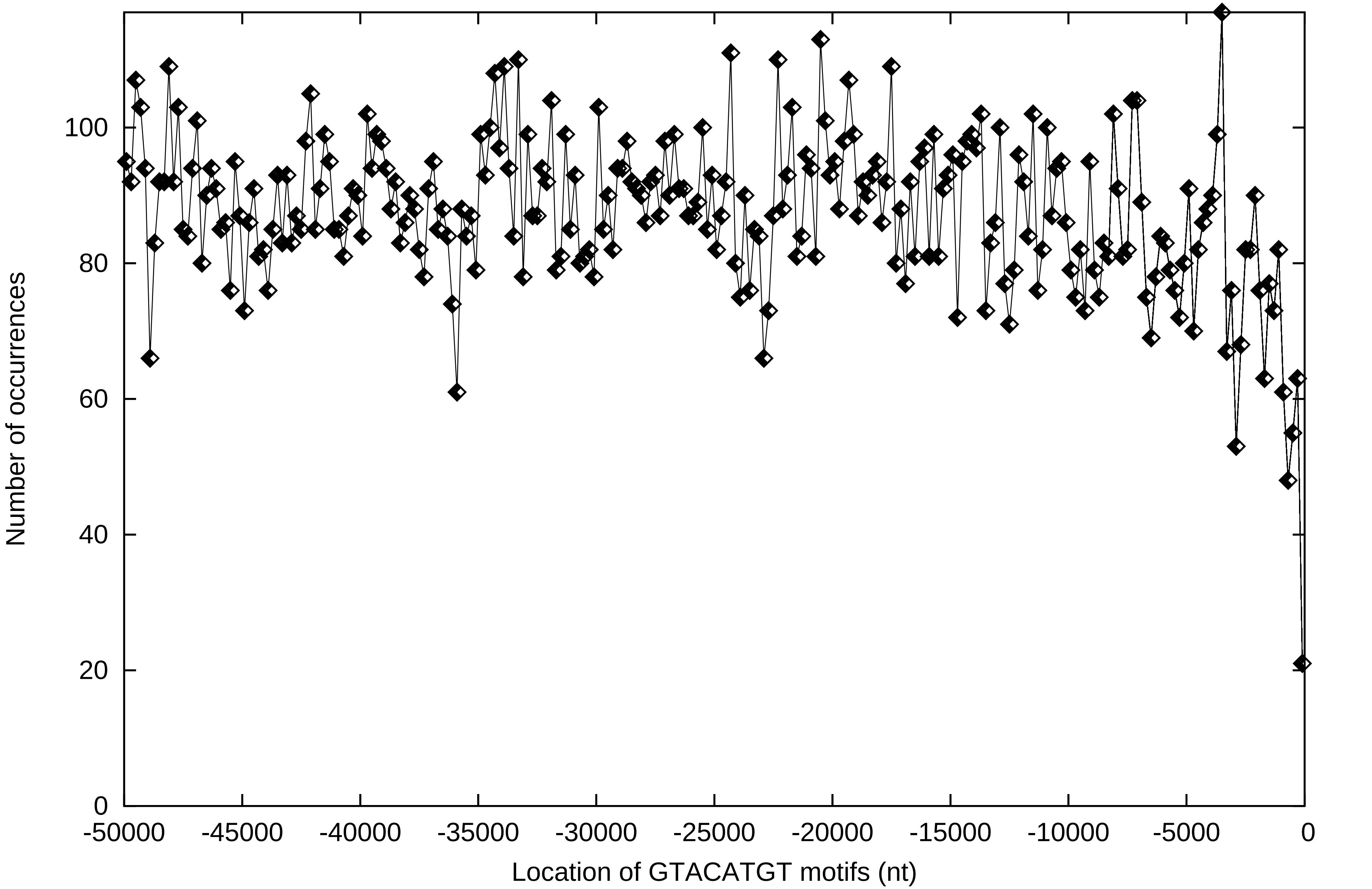

Number of occurrences
0
20
40
60
80
100
-50000
-45000
-40000
-35000
-30000
-25000
-20000
-15000
-10000
-5000
0
Location of GTACATGT motifs (nt)

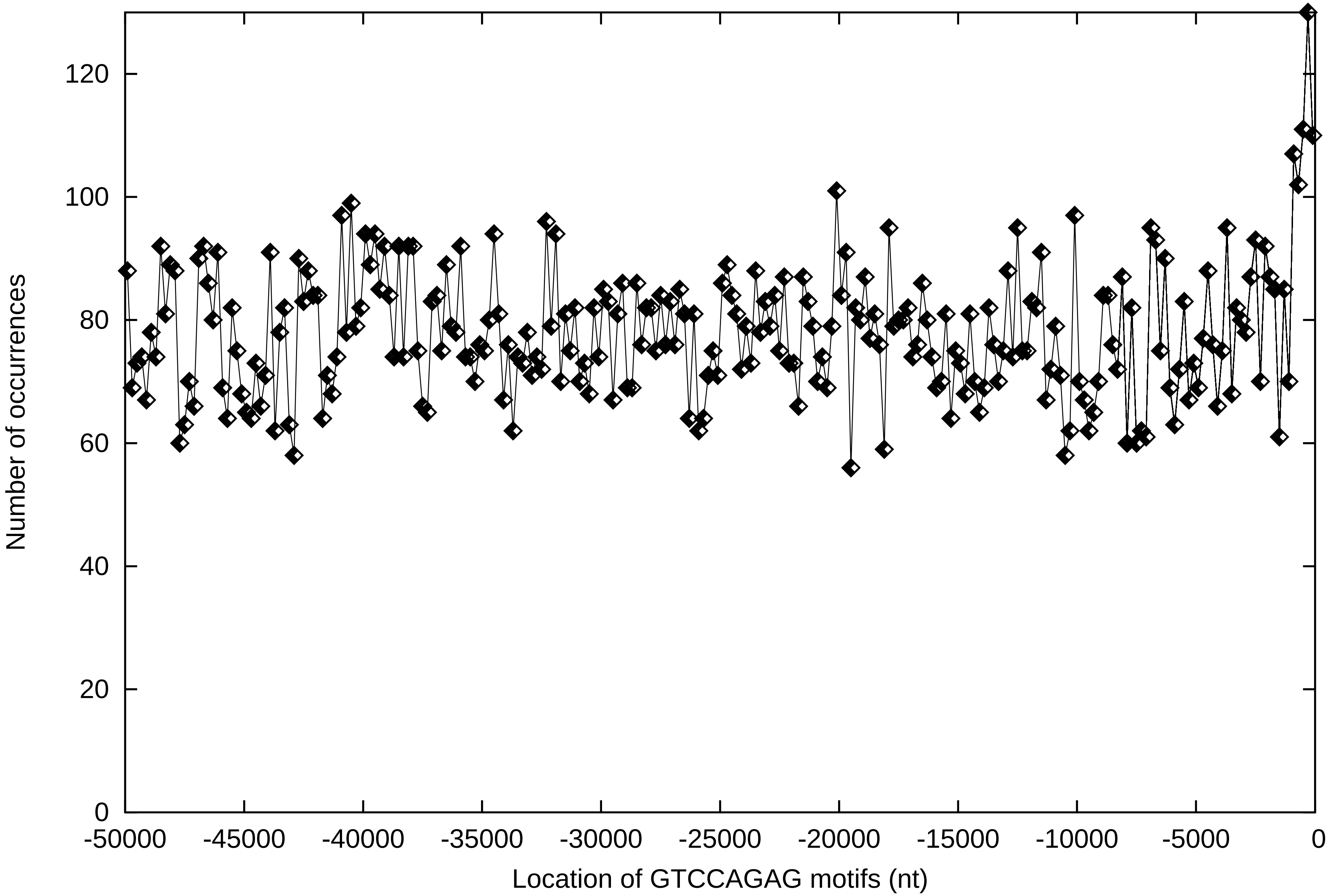

Number of occurrences
0
20
40
60
80
100
120
-50000
-45000
-40000
-35000
-30000
-25000
-20000
-15000
-10000
-5000
0
Location of GTCCAGAG motifs (nt)

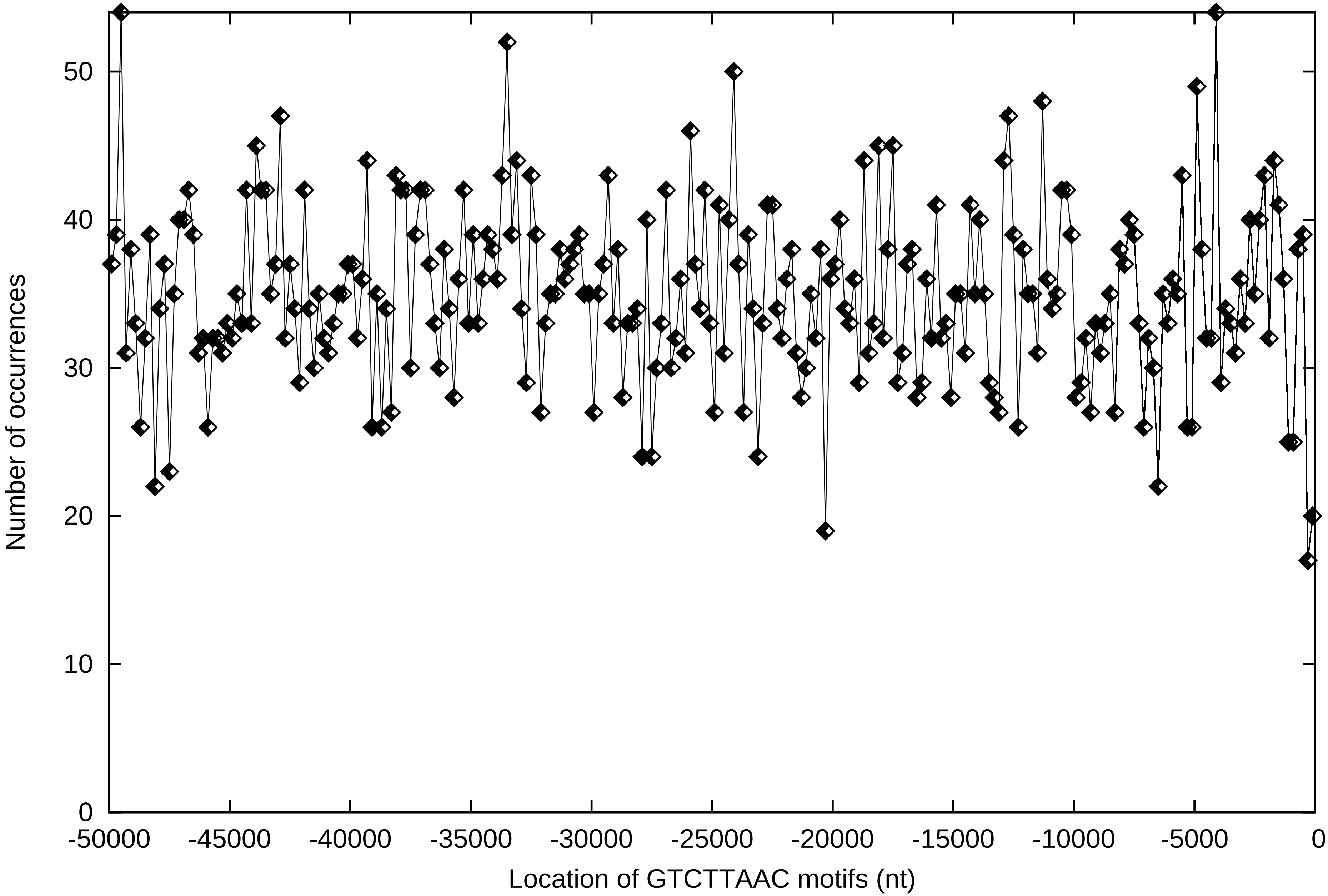
Number of occurrences
Location of GTCTTAAC motifs (nt)
0
10
20
30
40
50
-50000
-45000
-40000
-35000
-30000
-25000
-20000
-15000
-10000
-5000
0

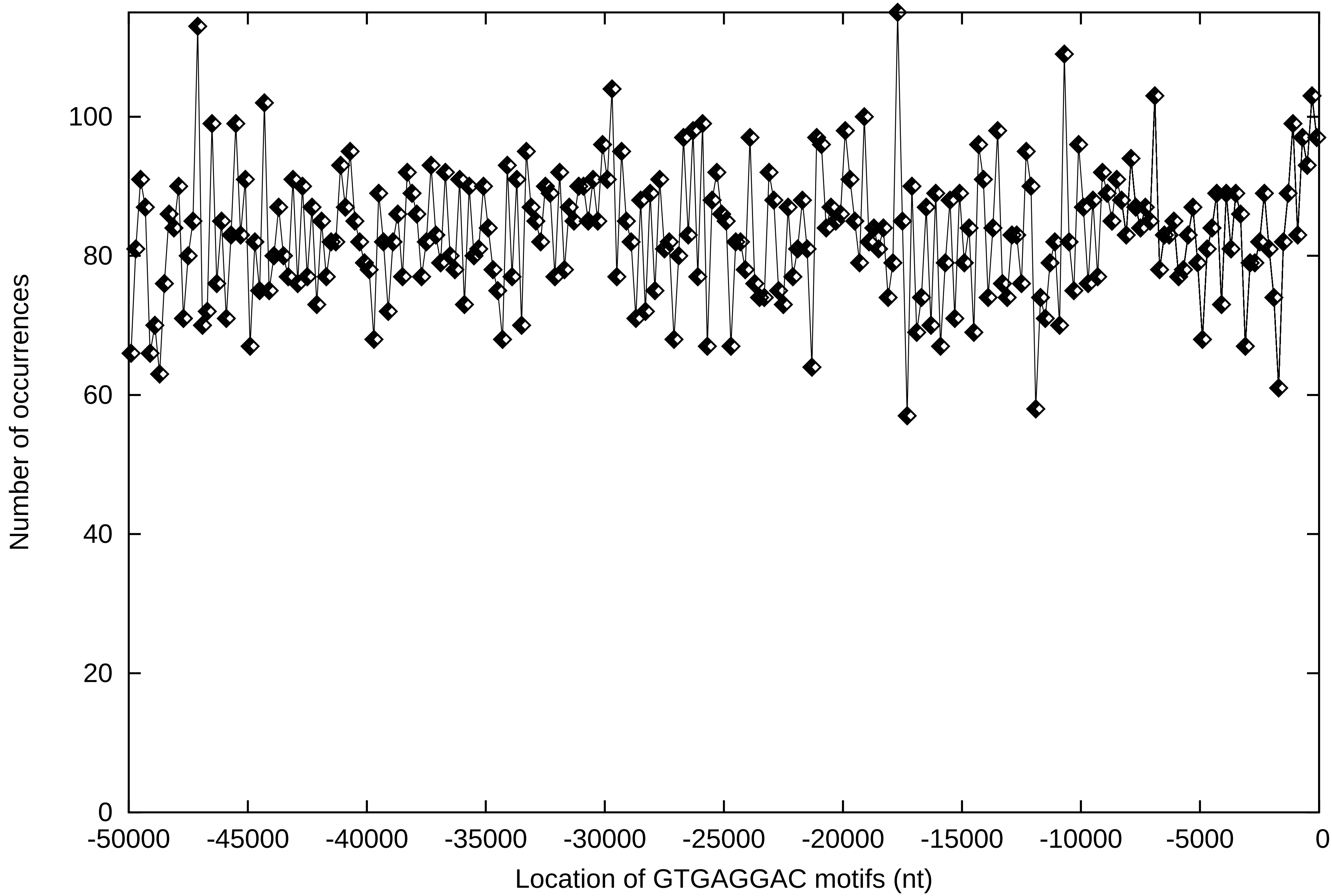

Number of occurrences
0
20
40
60
80
100
-50000
-45000
-40000
-35000
-30000
-25000
-20000
-15000
-10000
-5000
0
Location of GTGAGGAC motifs (nt)

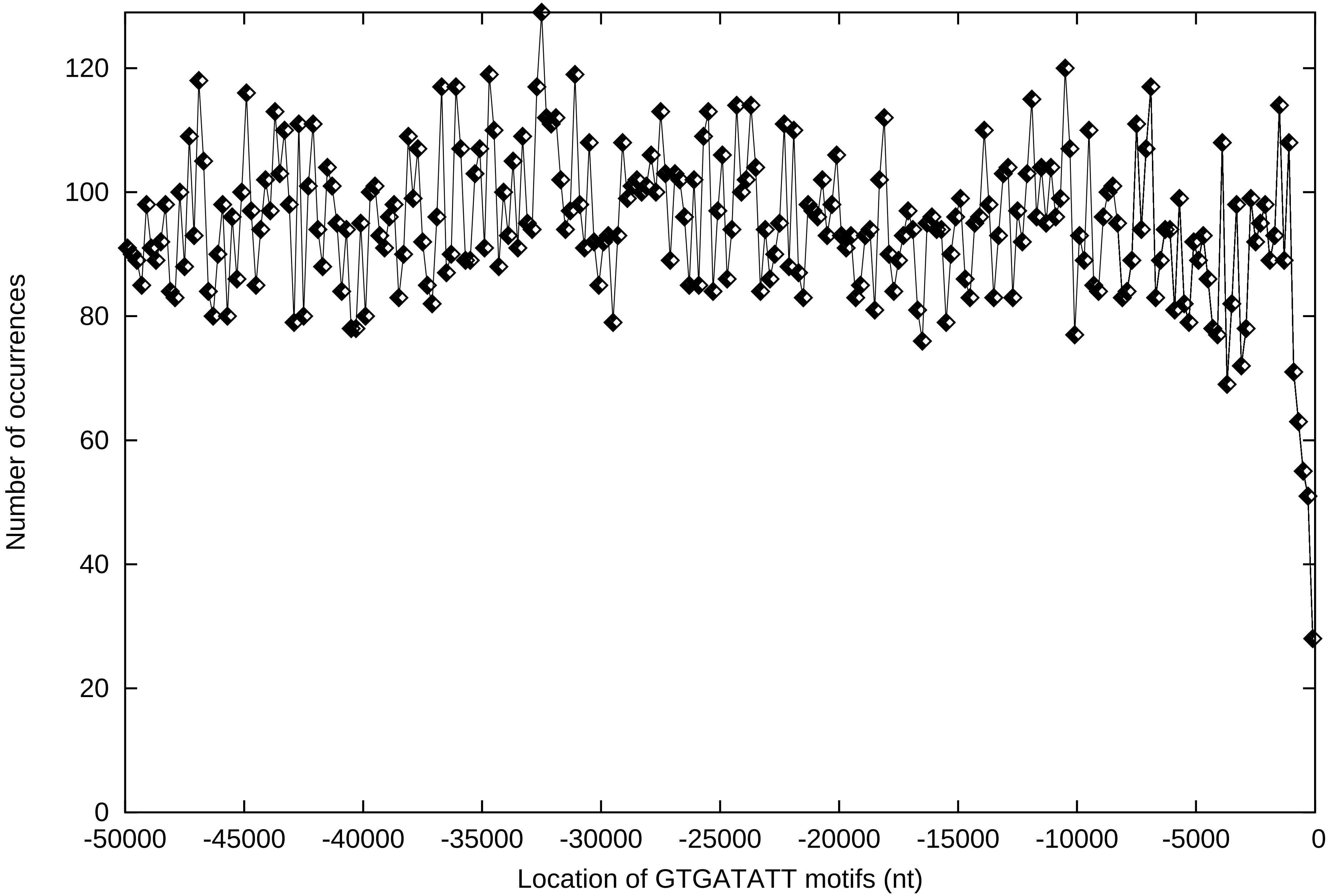

Number of occurrences
Location of GTGATATT motifs (nt)
0
20
40
60
80
100
120
-50000
-45000
-40000
-35000
-30000
-25000
-20000
-15000
-10000
-5000
0

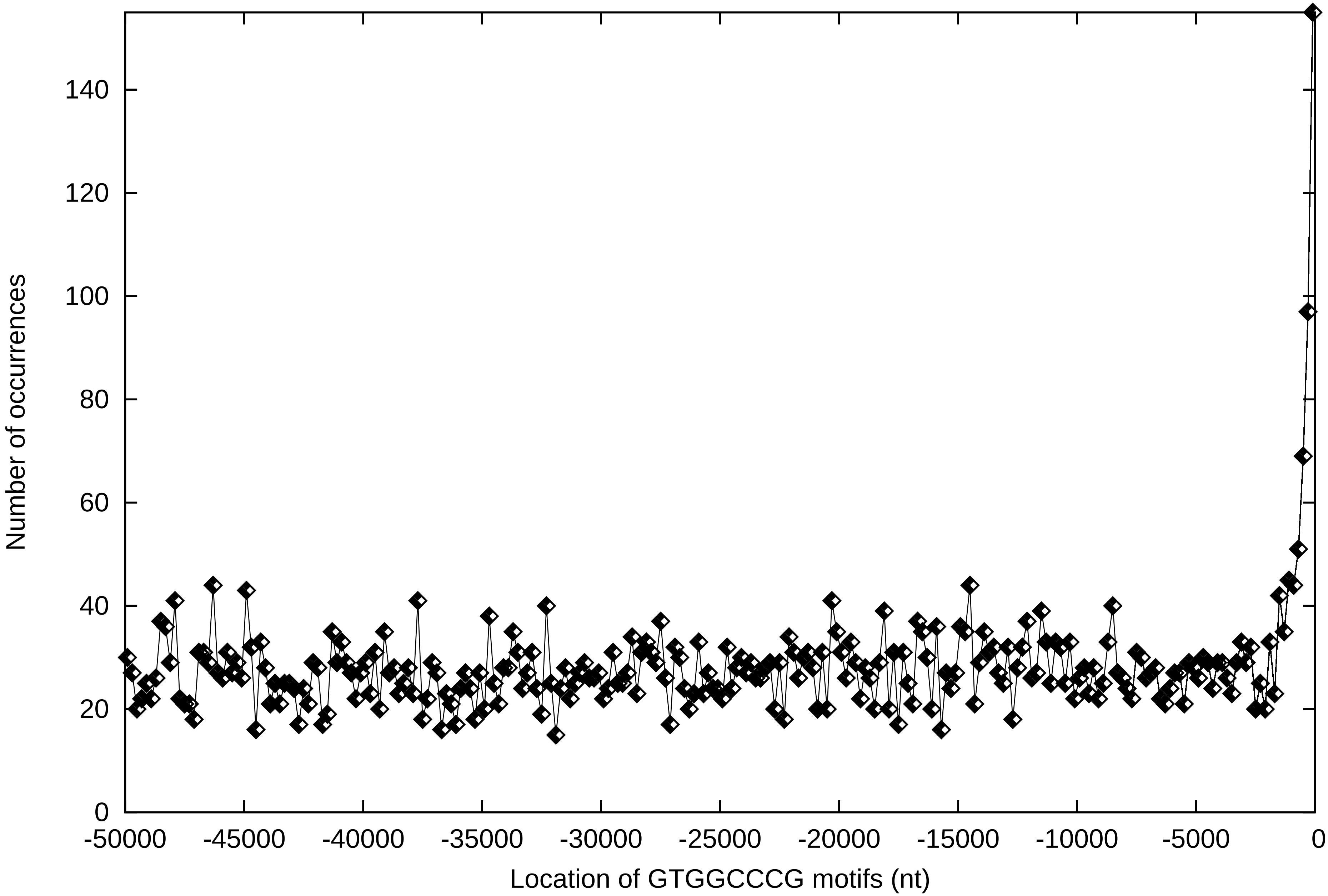

Number of occurrences
0
20
40
60
80
100
120
140
-50000
-45000
-40000
-35000
-30000
-25000
-20000
-15000
-10000
-5000
0
Location of GTGGCCCG motifs (nt)

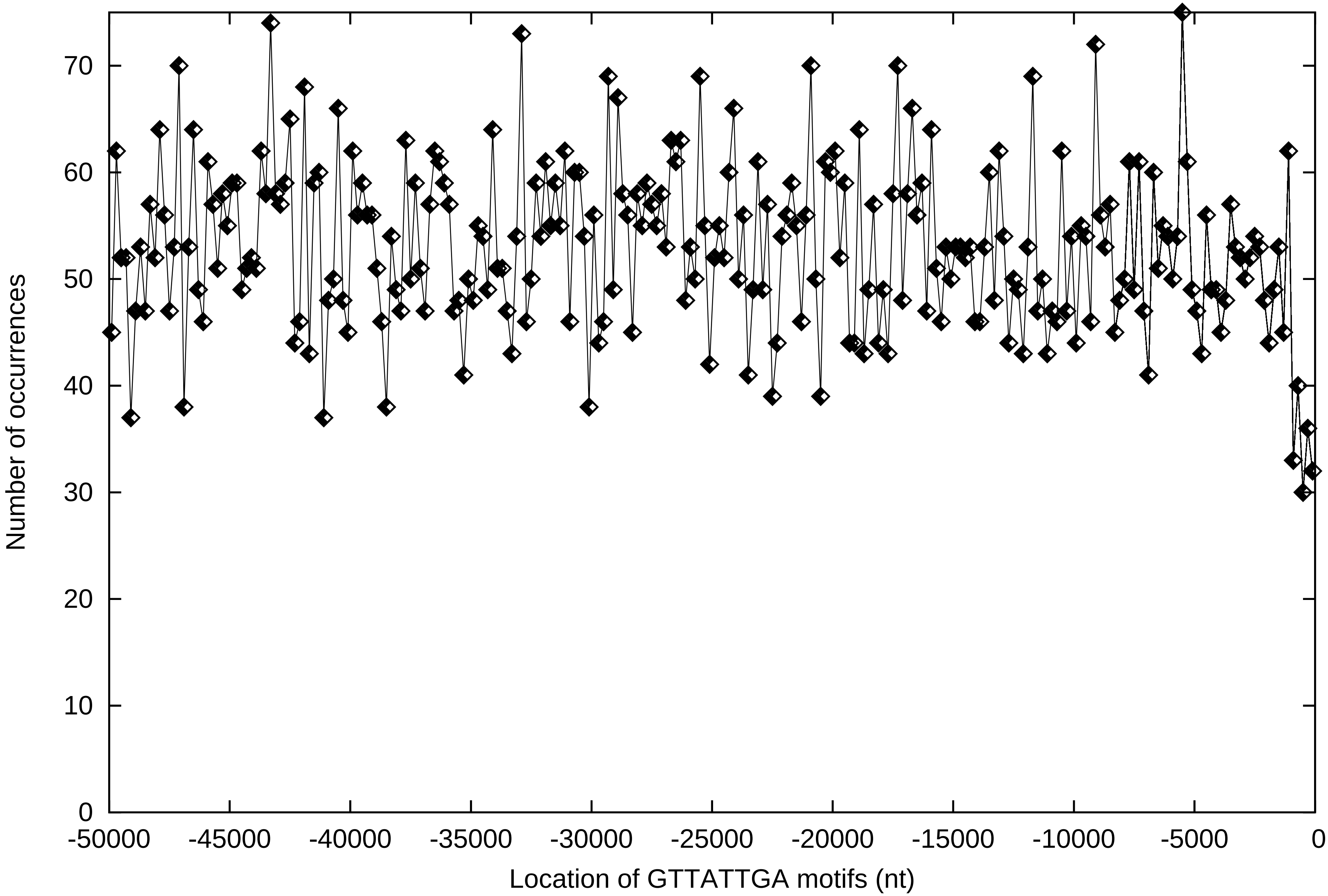

Number of occurrences
Location of GTTATTGA motifs (nt)
0
10
20
30
40
50
60
70
-50000
-45000
-40000
-35000
-30000
-25000
-20000
-15000
-10000
-5000
0

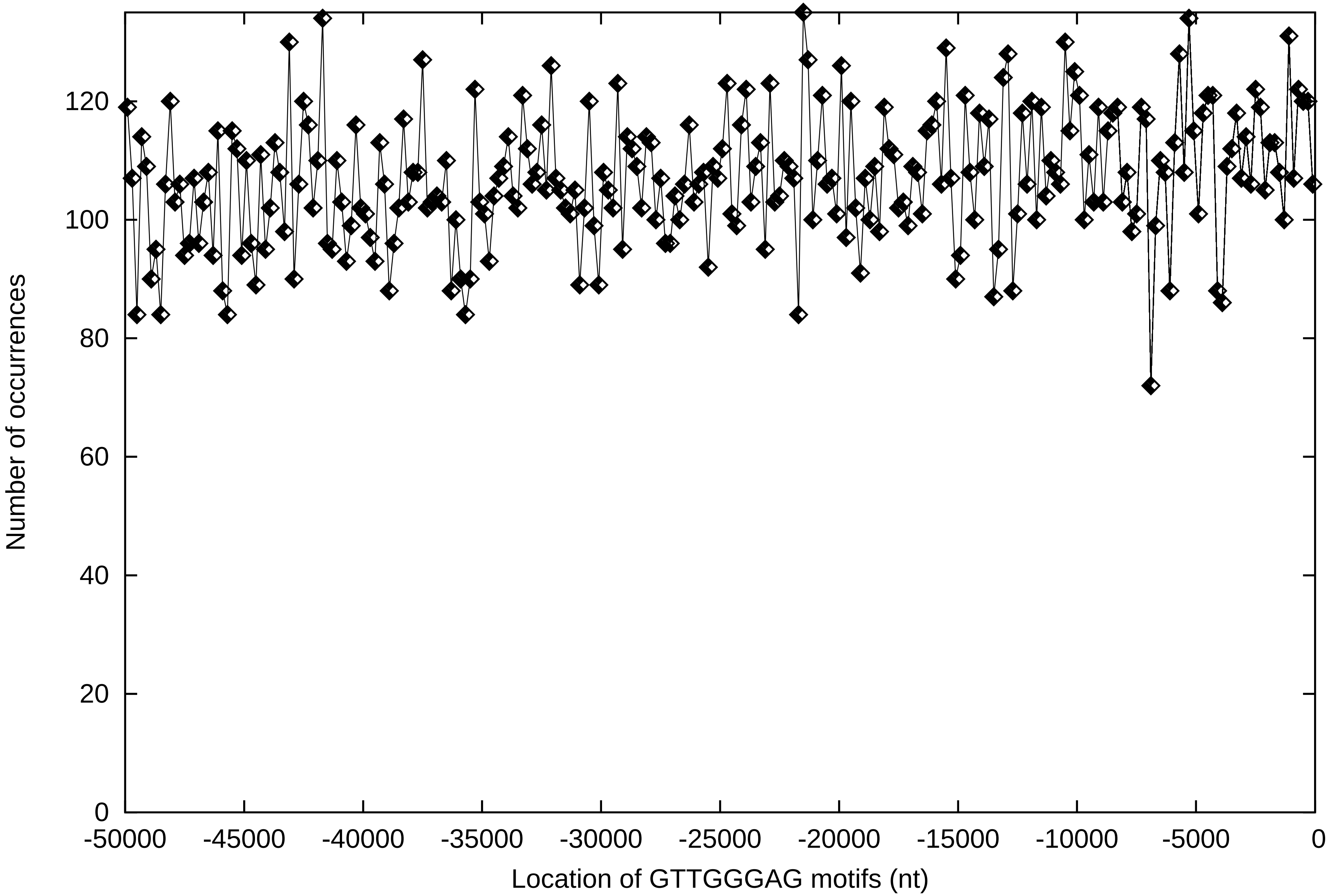

Number of occurrences
0
20
40
60
80
100
120
-50000
-45000
-40000
-35000
-30000
-25000
-20000
-15000
-10000
-5000
0
Location of GTTGGGAG motifs (nt)

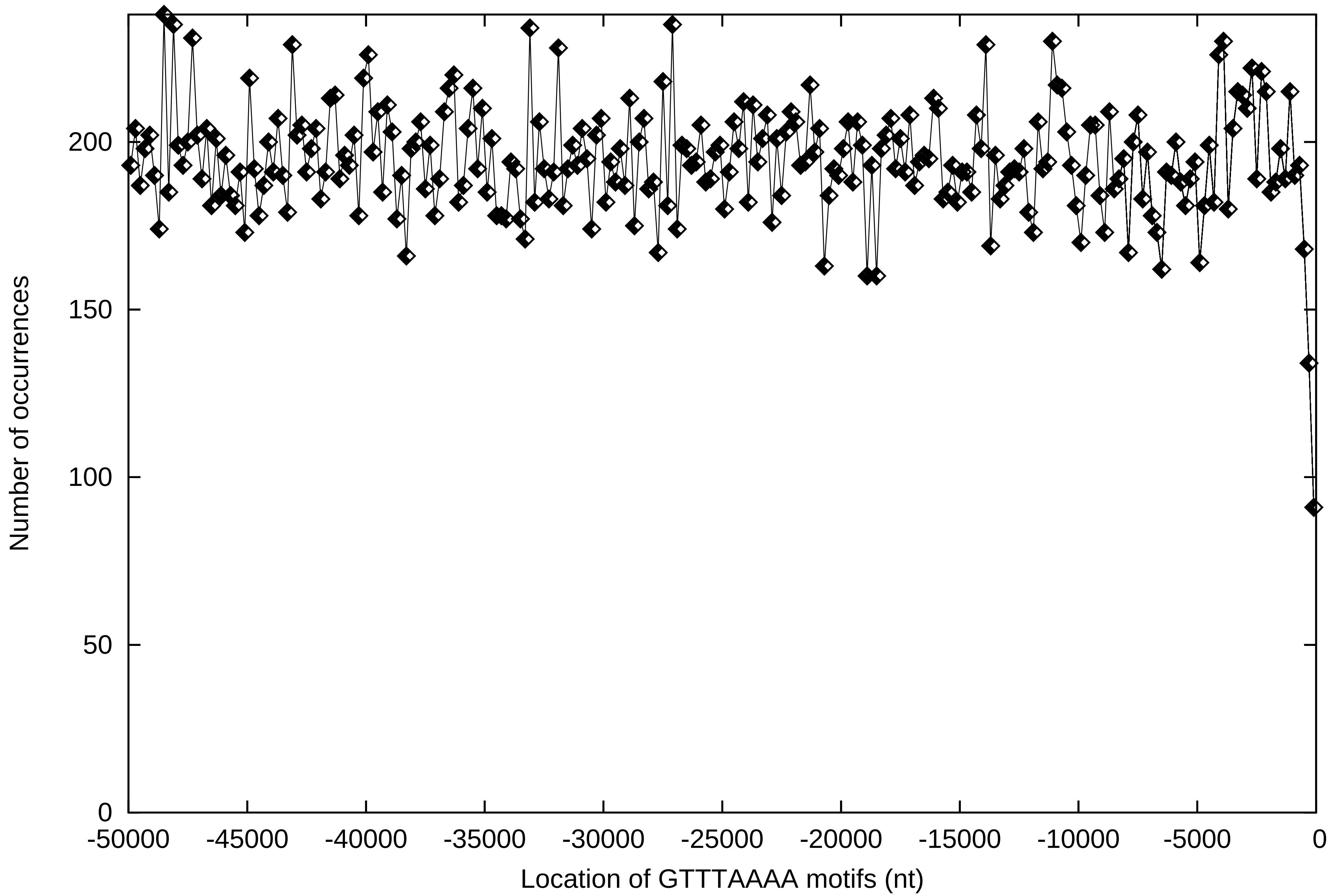
Number of occurrences
Location of GTTTAAAA motifs (nt)
0
50
100
150
200
-50000
-45000
-40000
-35000
-30000
-25000
-20000
-15000
-10000
-5000
0

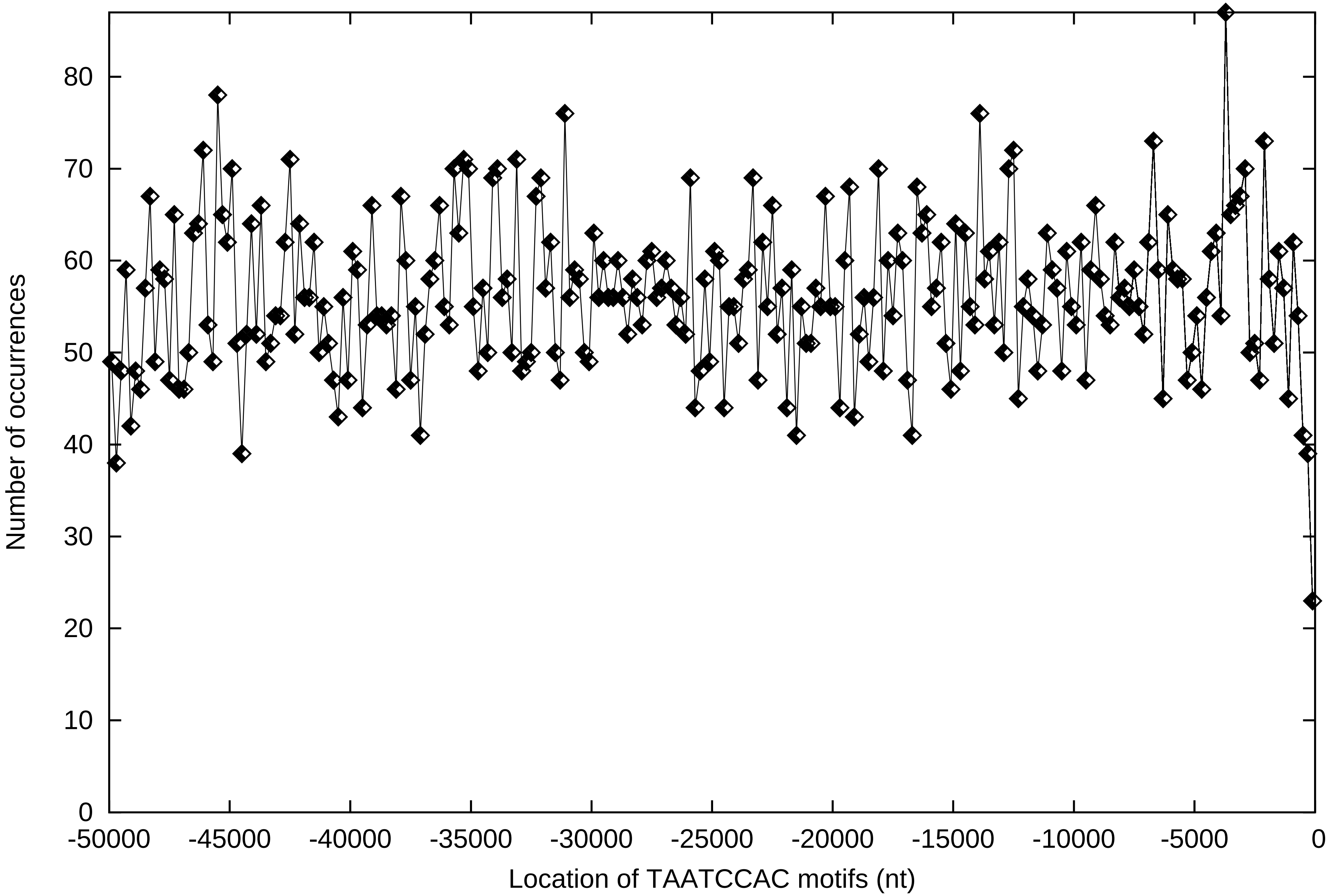

Number of occurrences
0
10
20
30
40
50
60
70
80
-50000
-45000
-40000
-35000
-30000
-25000
-20000
-15000
-10000
-5000
0
Location of TAATCCAC motifs (nt)

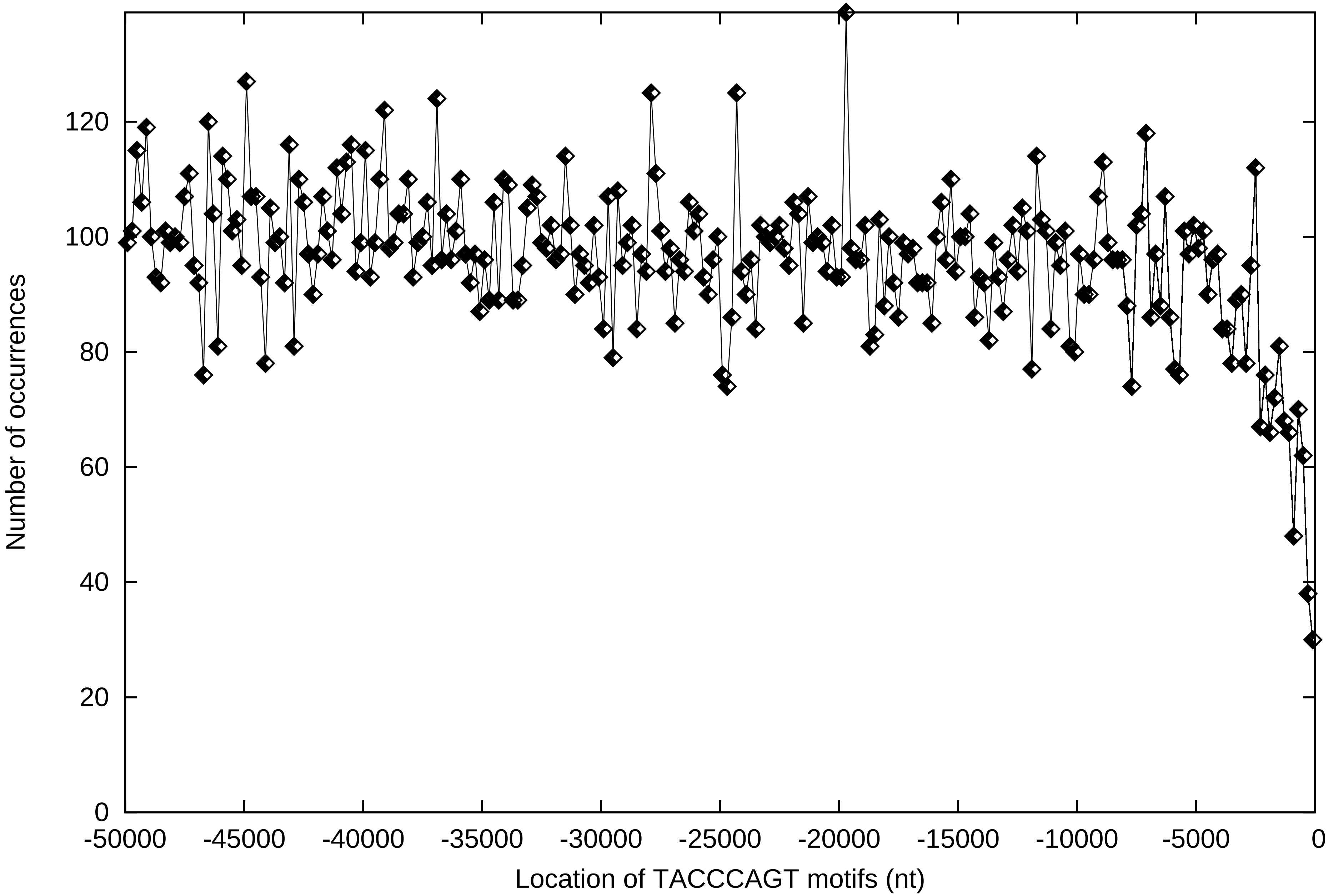

Number of occurrences
Location of TACCCAGT motifs (nt)
0
20
40
60
80
100
120
-50000
-45000
-40000
-35000
-30000
-25000
-20000
-15000
-10000
-5000
0

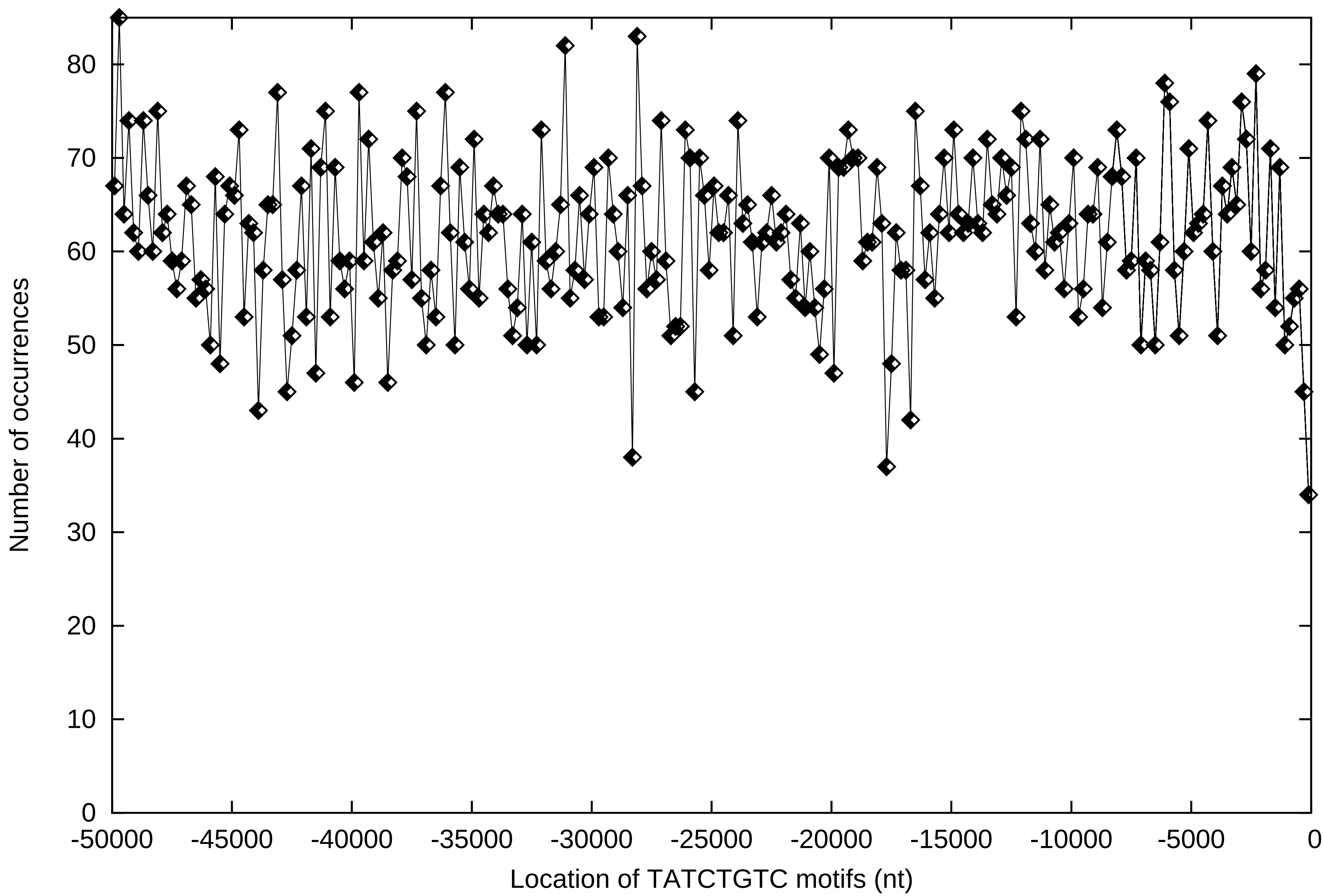
Number of occurrences
Location of TATCTGTC motifs (nt)
0
10
20
30
40
50
60
70
80
-50000
-45000
-40000
-35000
-30000
-25000
-20000
-15000
-10000
-5000
0

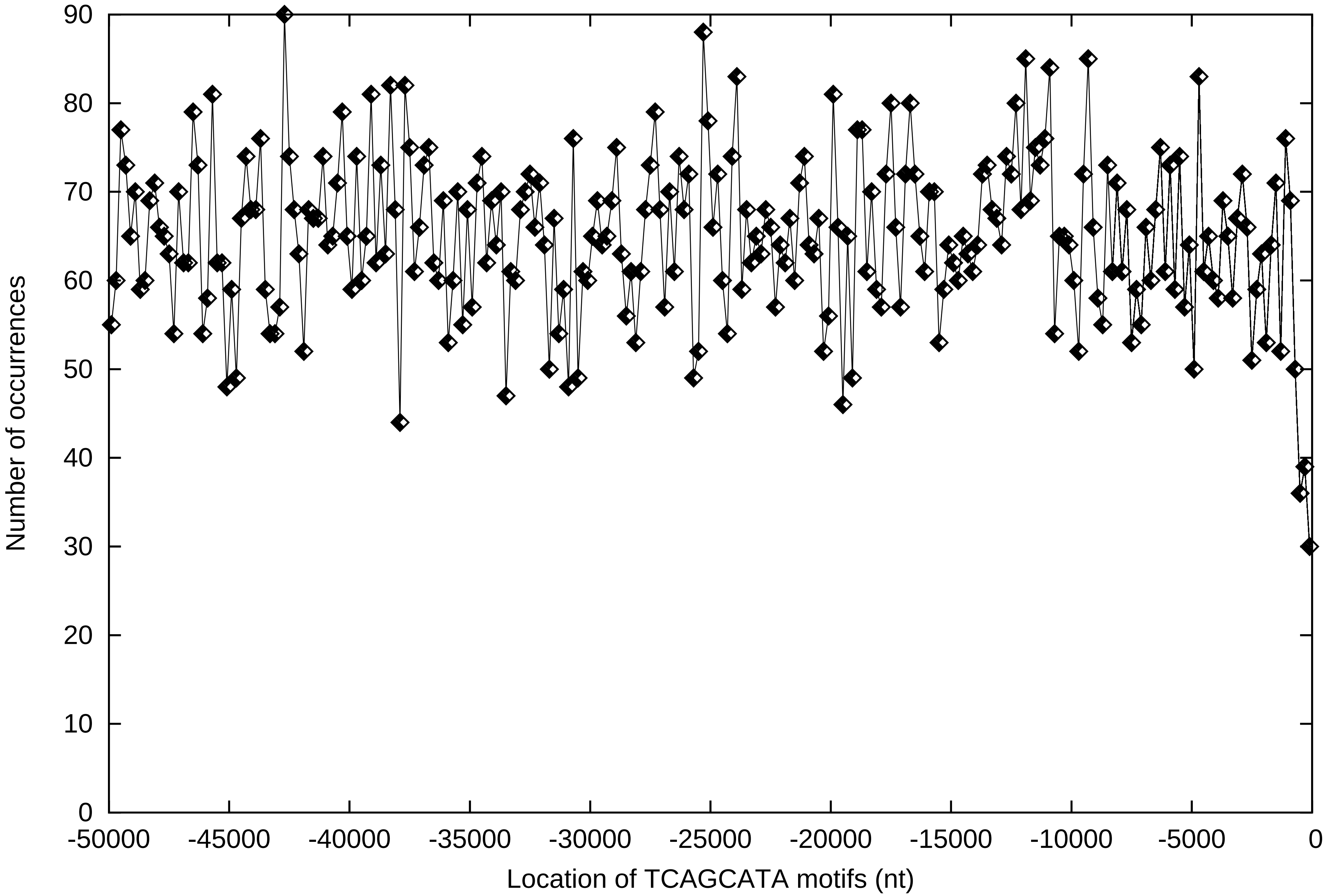

Number of occurrences
90
80
70
60
50
40
30
20
10
0
-50000
-45000
-40000
-35000
-30000
-25000
-20000
-15000
-10000
-5000
0
Location of TCAGCATA motifs (nt)

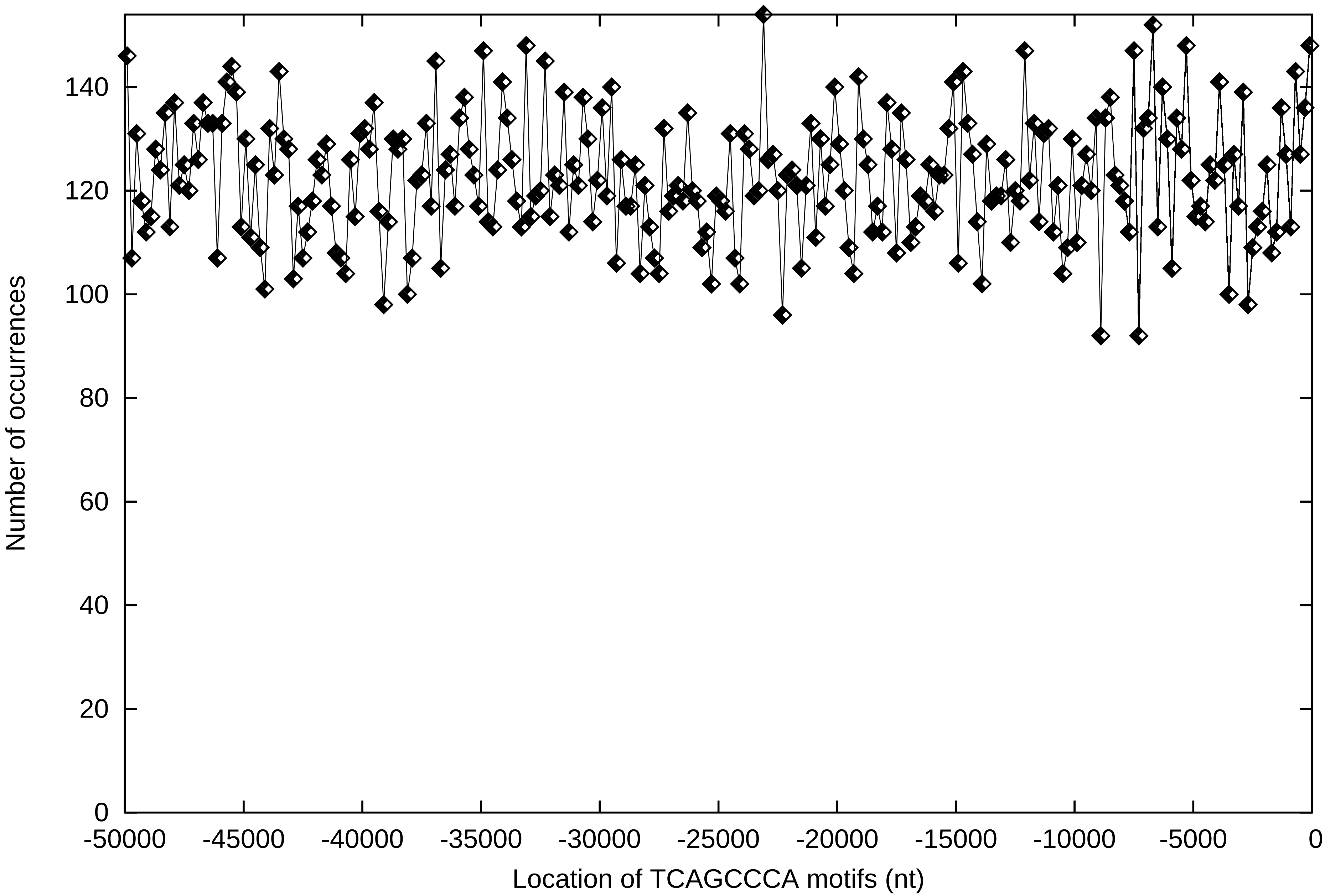
Number of occurrences
Location of TCAGCCCA motifs (nt)
0
20
40
60
80
100
120
140
-50000
-45000
-40000
-35000
-30000
-25000
-20000
-15000
-10000
-5000
0

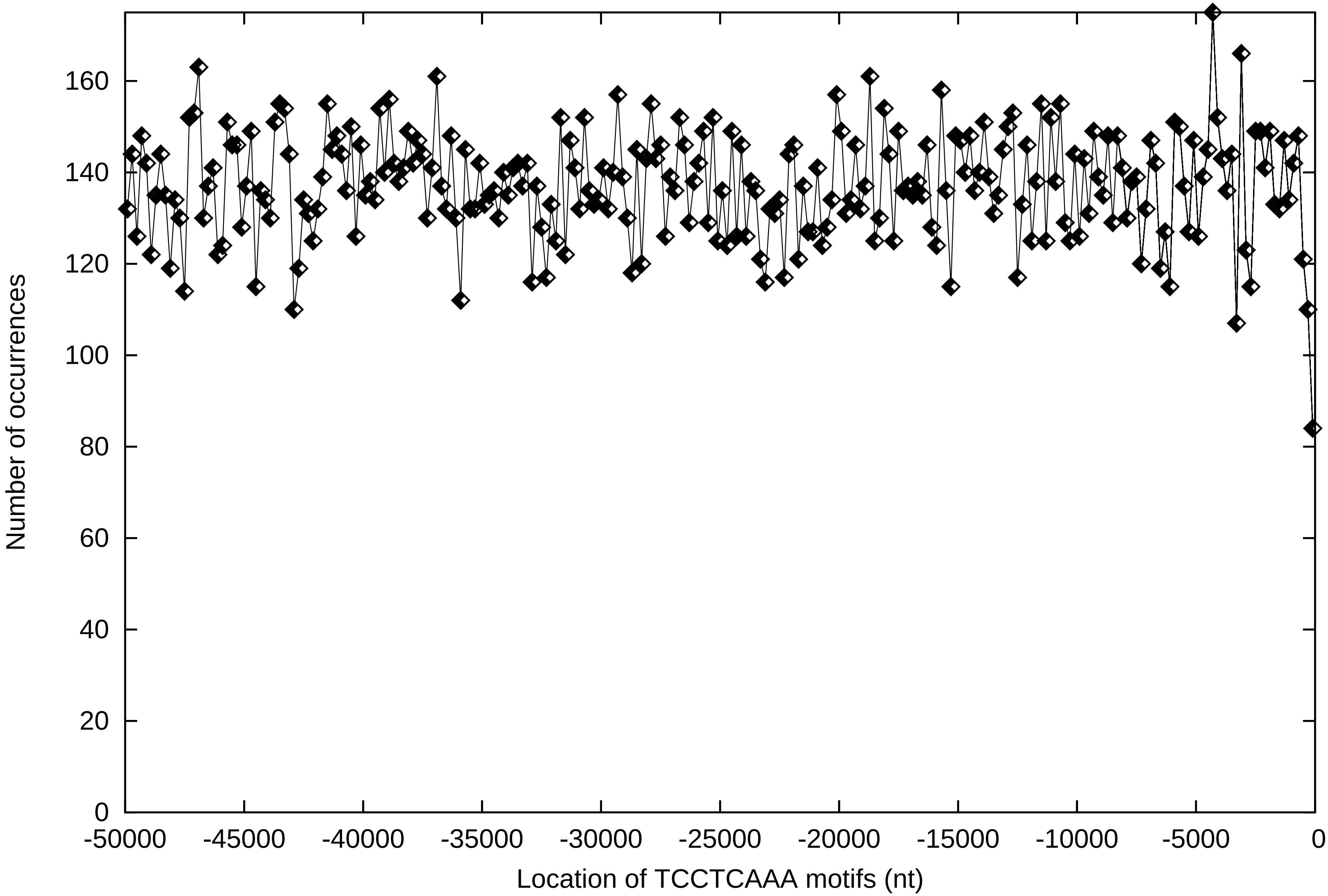

Number of occurrences
0
20
40
60
80
100
120
140
160
-50000
-45000
-40000
-35000
-30000
-25000
-20000
-15000
-10000
-5000
0
Location of TCCTCAAA motifs (nt)

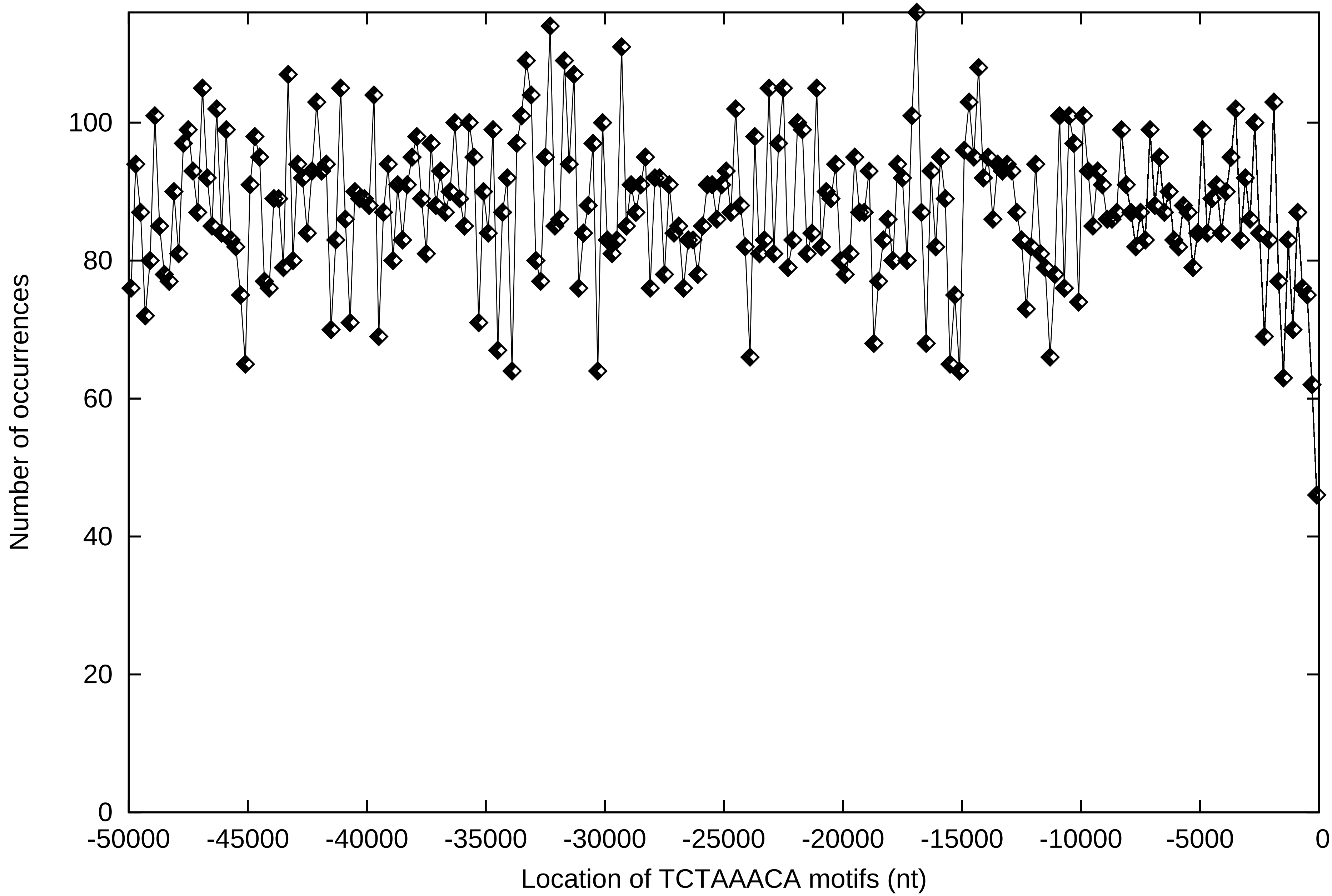

Number of occurrences
0
20
40
60
80
100
-50000
-45000
-40000
-35000
-30000
-25000
-20000
-15000
-10000
-5000
0
Location of TCTAAACA motifs (nt)

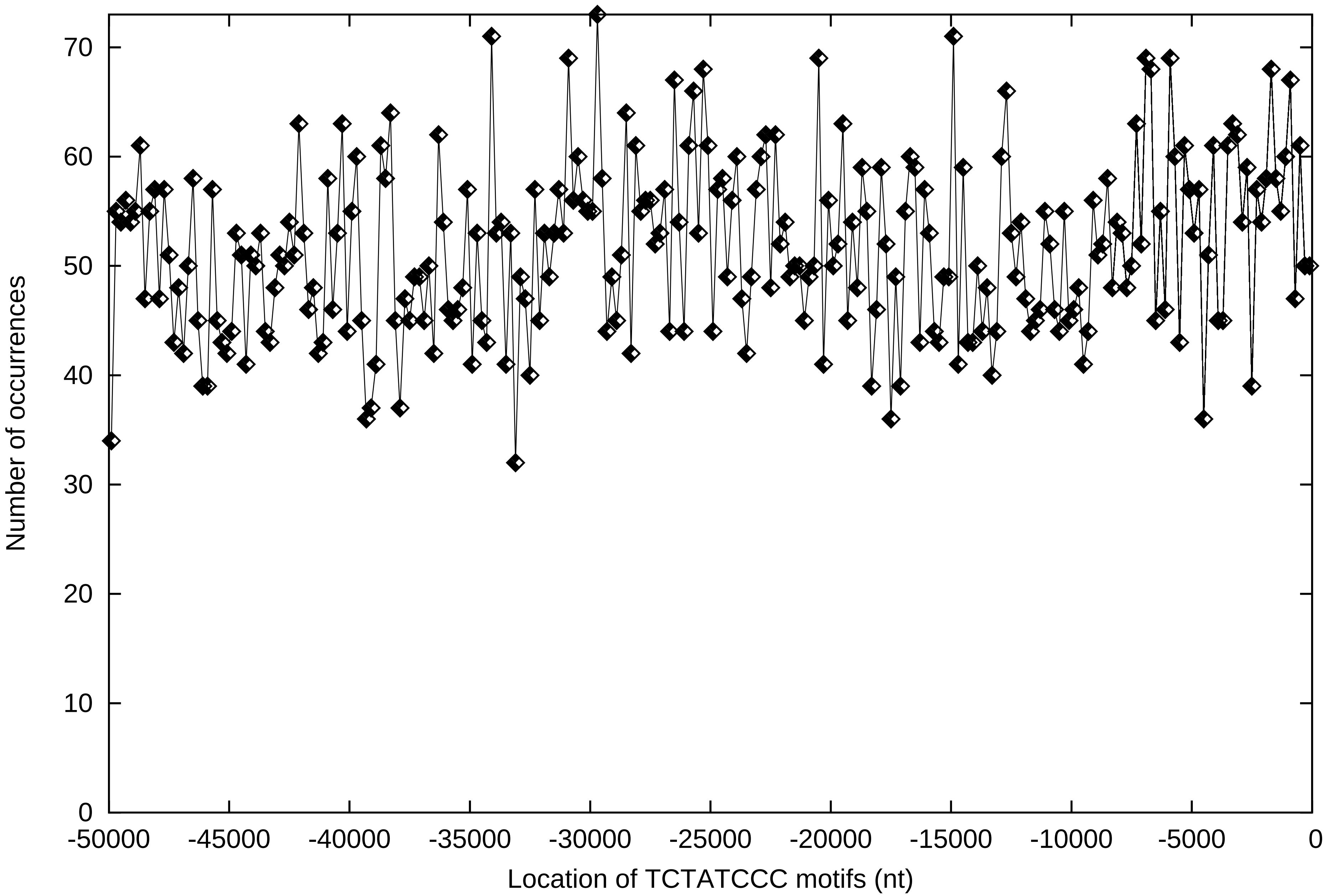

Number of occurrences
0
10
20
30
40
50
60
70
-50000
-45000
-40000
-35000
-30000
-25000
-20000
-15000
-10000
-5000
0
Location of TCTATCCC motifs (nt)

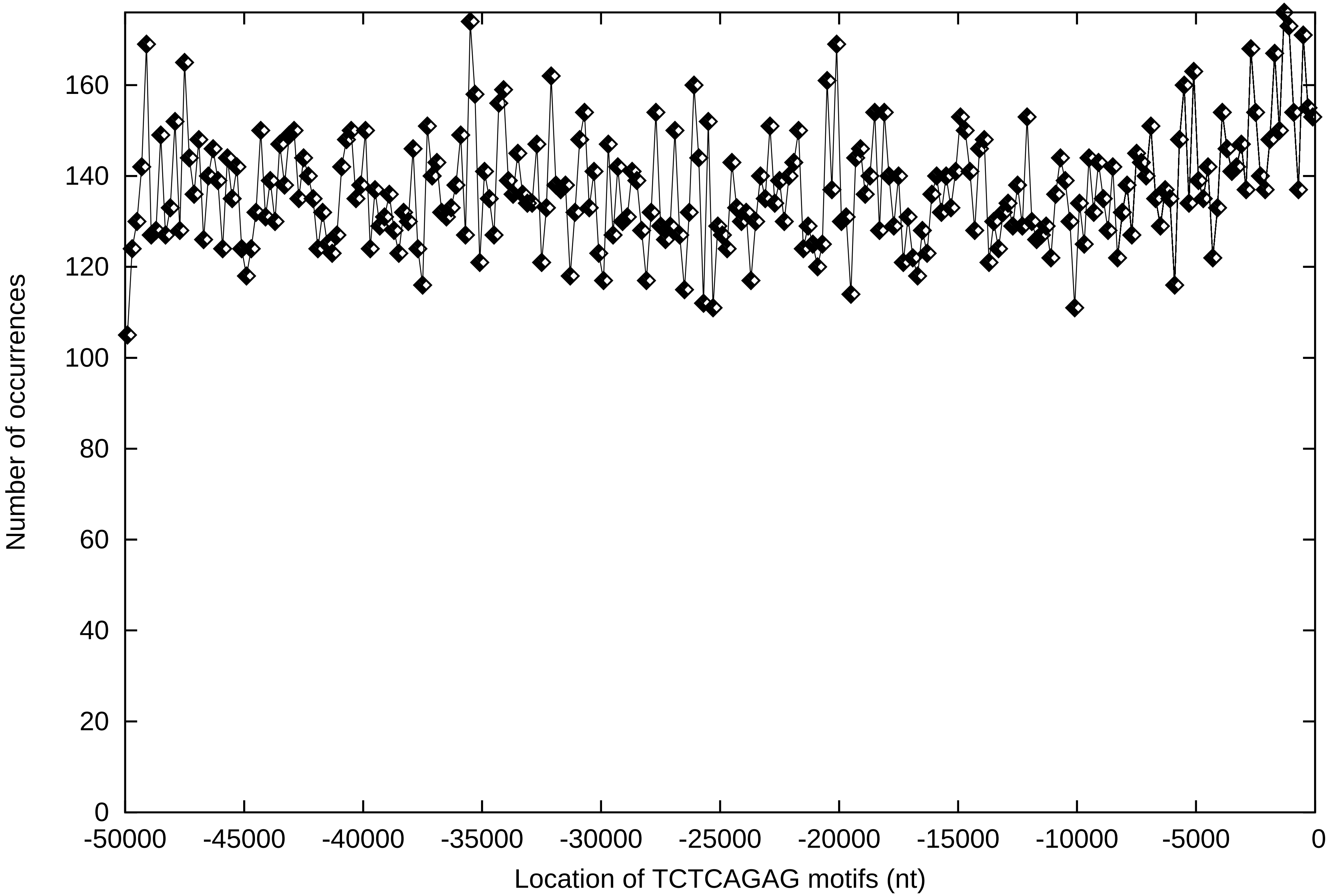
Number of occurrences
Location of TCTCAGAG motifs (nt)
0
20
40
60
80
100
120
140
160
-50000
-45000
-40000
-35000
-30000
-25000
-20000
-15000
-10000
-5000
0

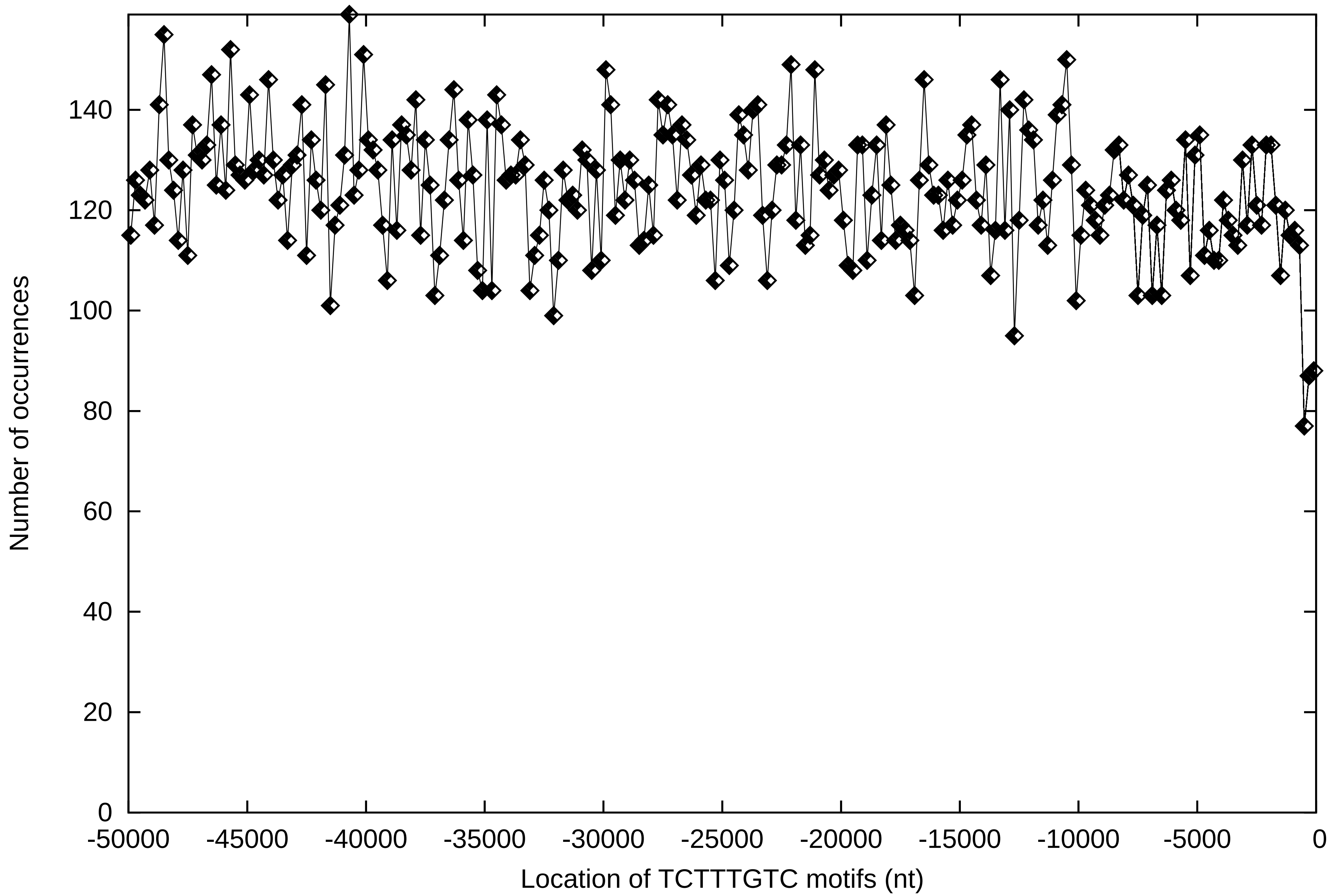

Number of occurrences
0
20
40
60
80
100
120
140
-50000
-45000
-40000
-35000
-30000
-25000
-20000
-15000
-10000
-5000
0
Location of TCTTTGTC motifs (nt)

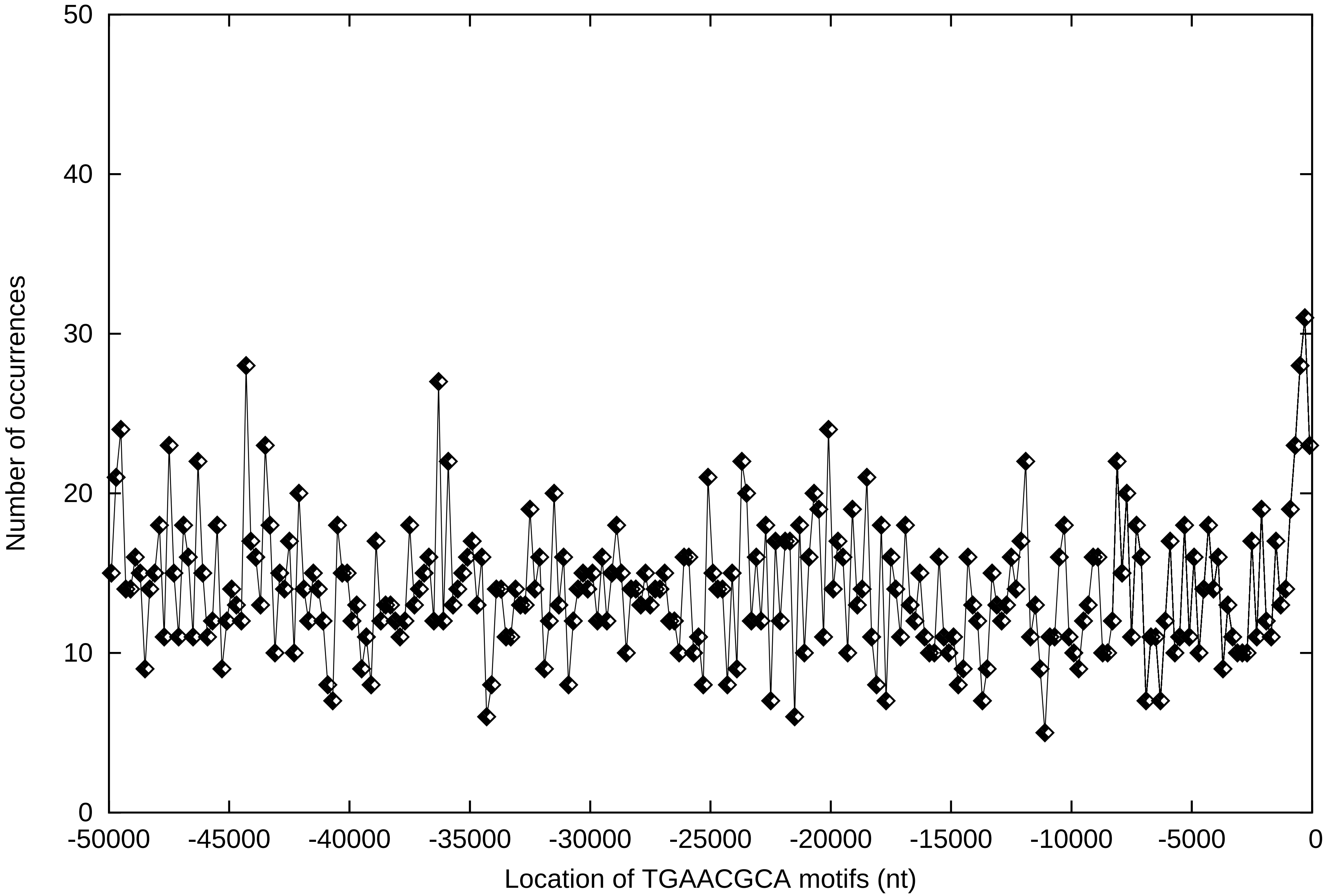

50
40
30
20
10
0
-50000
-45000
-40000
-35000
-30000
-25000
-20000
-15000
-10000
-5000
0
Number of occurrences
Location of TGAACGCA motifs (nt)

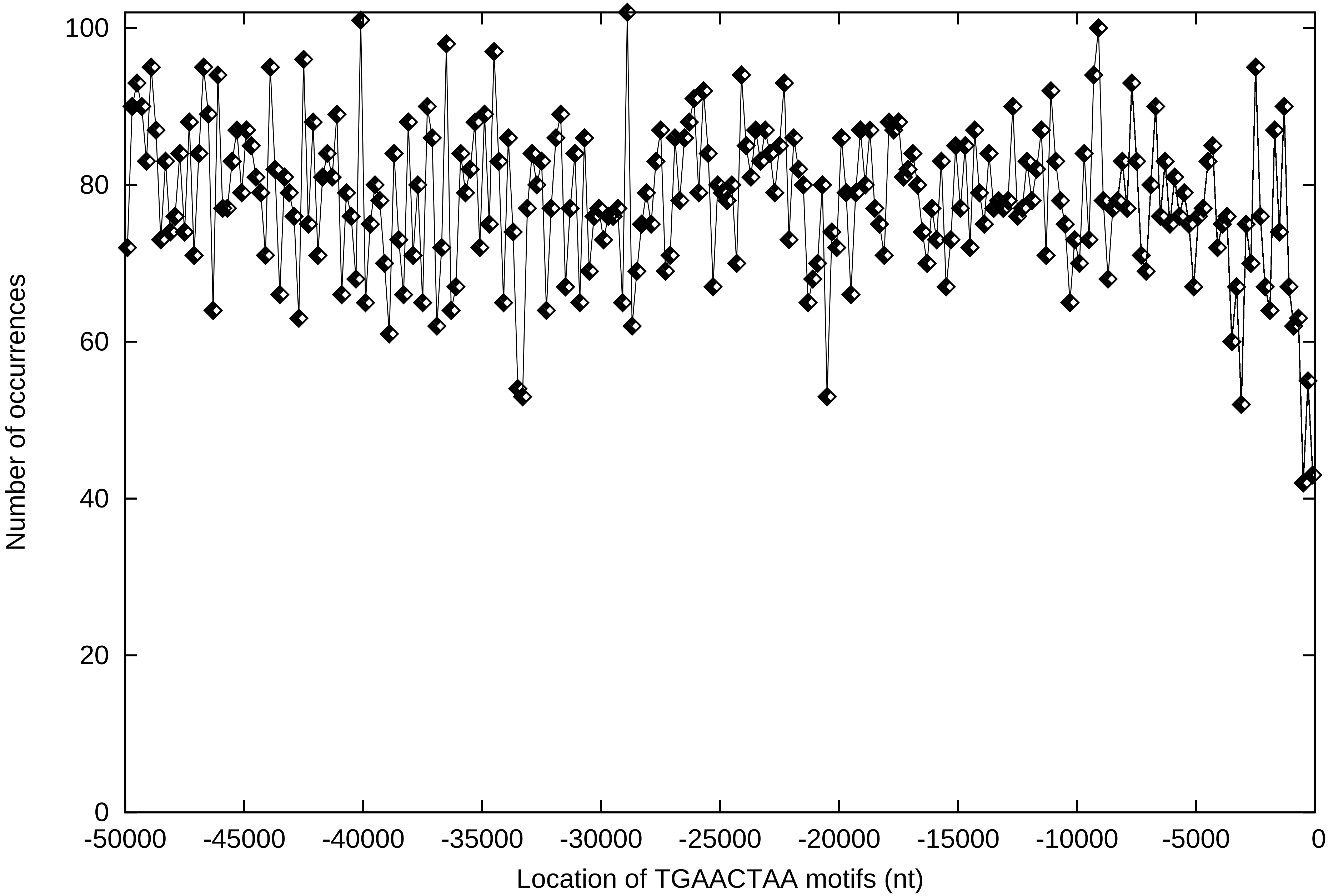

100
80
60
40
20
0
-50000
-45000
-40000
-35000
-30000
-25000
-20000
-15000
-10000
-5000
0
Number of occurrences
Location of TGAACTAA motifs (nt)

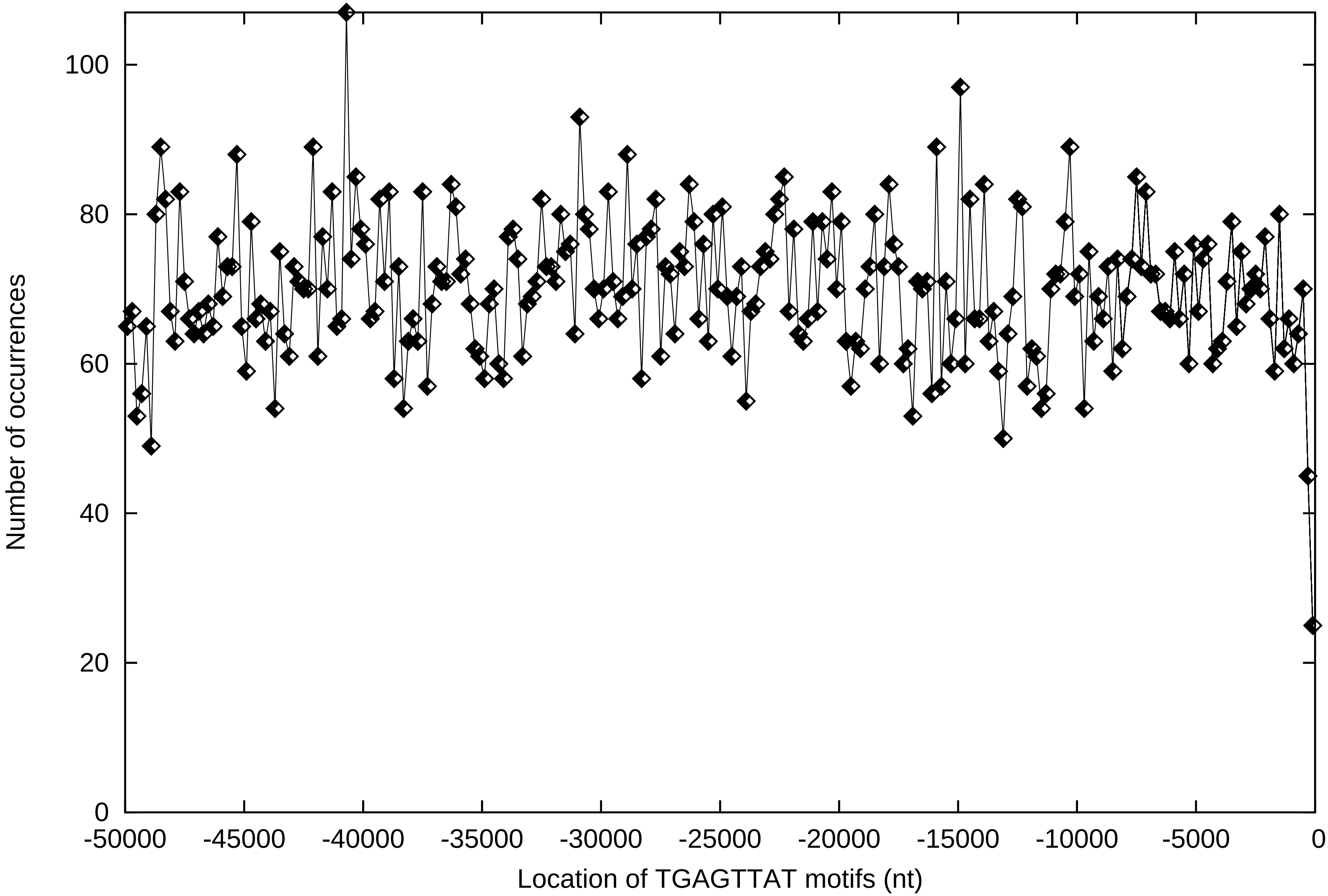

Number of occurrences
100
80
60
40
20
0
-50000
-45000
-40000
-35000
-30000
-25000
-20000
-15000
-10000
-5000
0
Location of TGAGTTAT motifs (nt)

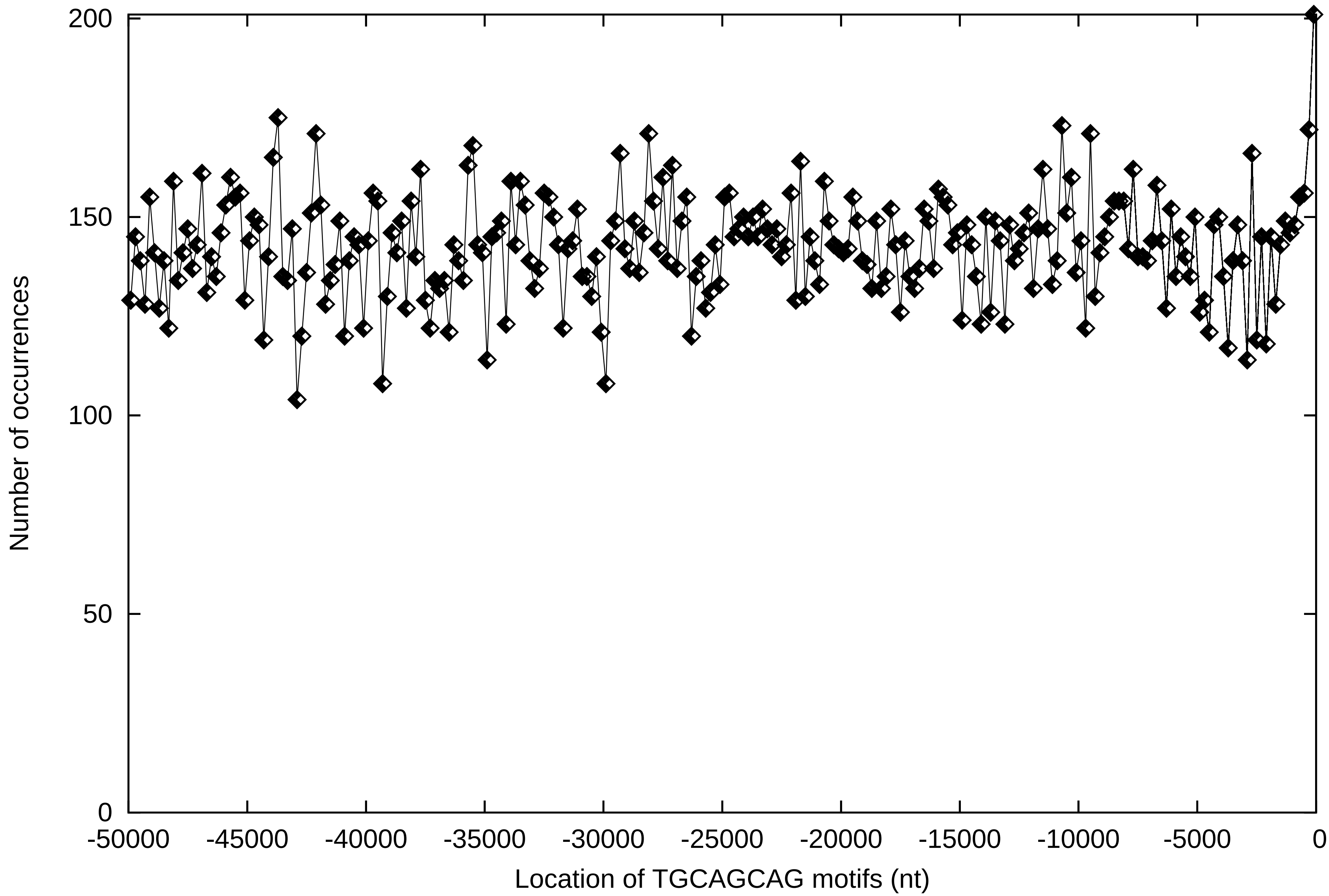

200
150
100
50
0
-50000
-45000
-40000
-35000
-30000
-25000
-20000
-15000
-10000
-5000
0
Number of occurrences
Location of TGCAGCAG motifs (nt)

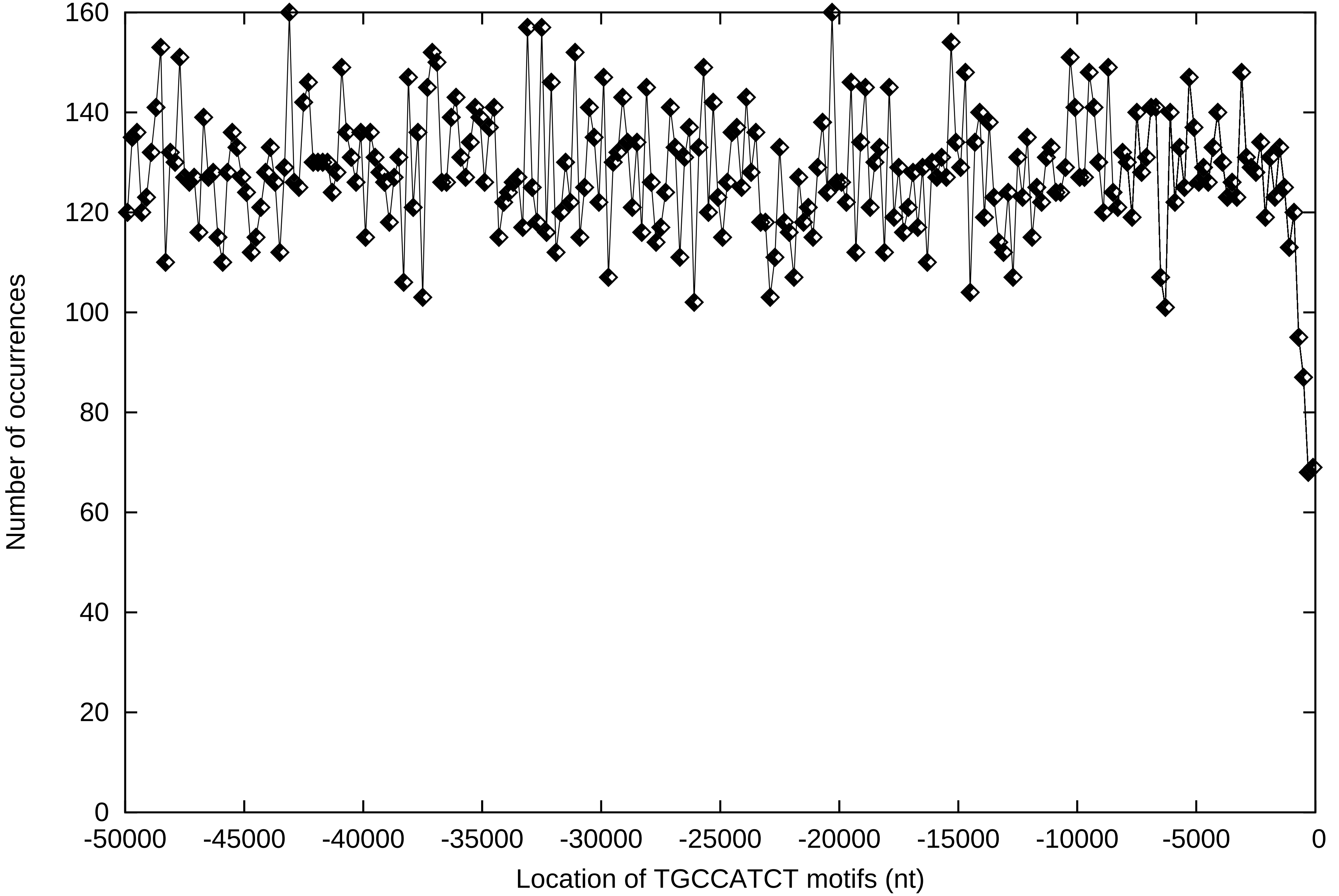

160
140
120
100
80
60
40
20
0
Number of occurrences
-50000
-45000
-40000
-35000
-30000
-25000
-20000
-15000
-10000
-5000
0
Location of TGCCATCT motifs (nt)

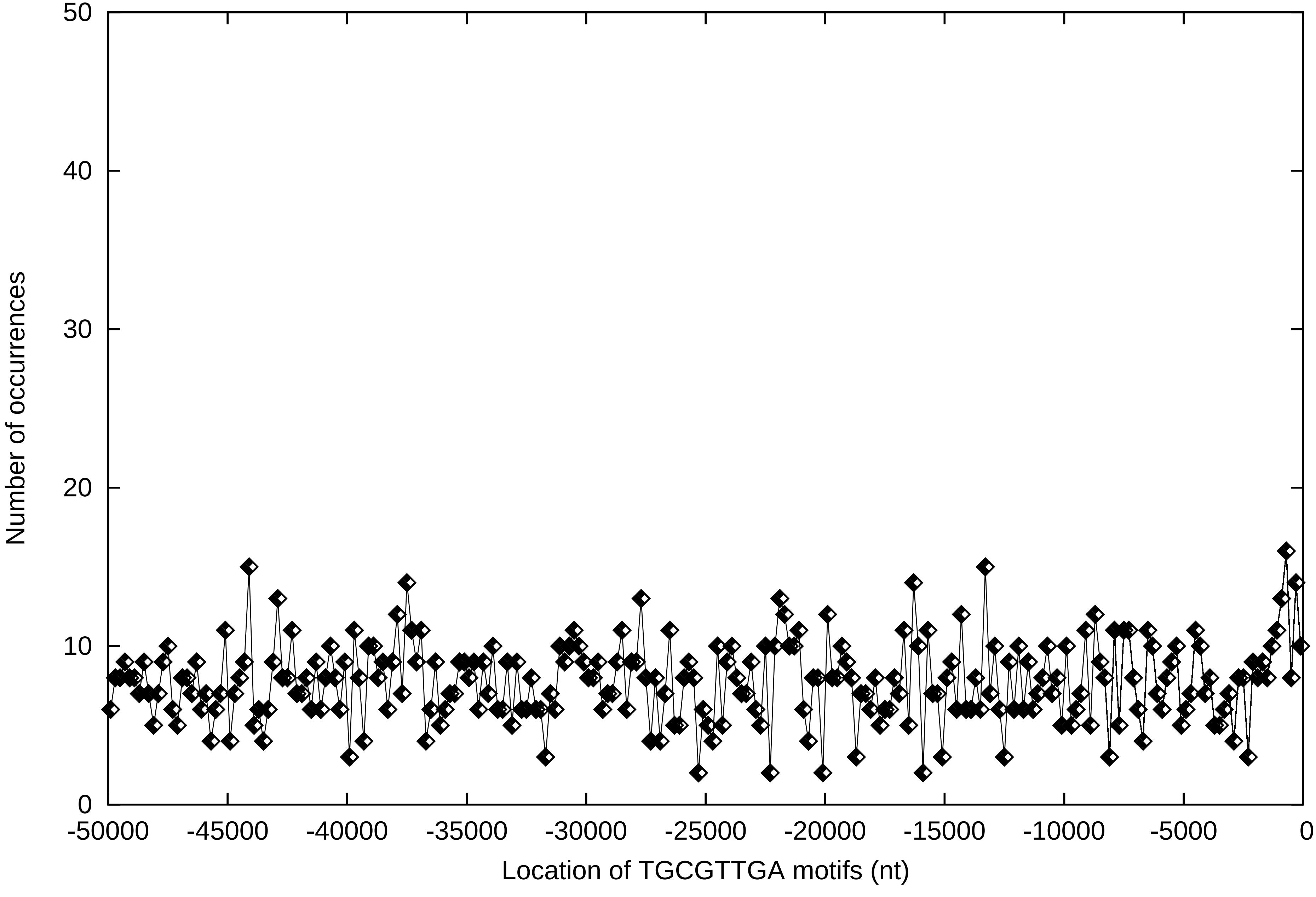

Number of occurrences
50
40
30
20
10
0
-50000
-45000
-40000
-35000
-30000
-25000
-20000
-15000
-10000
-5000
0
Location of TGCGTTGA motifs (nt)

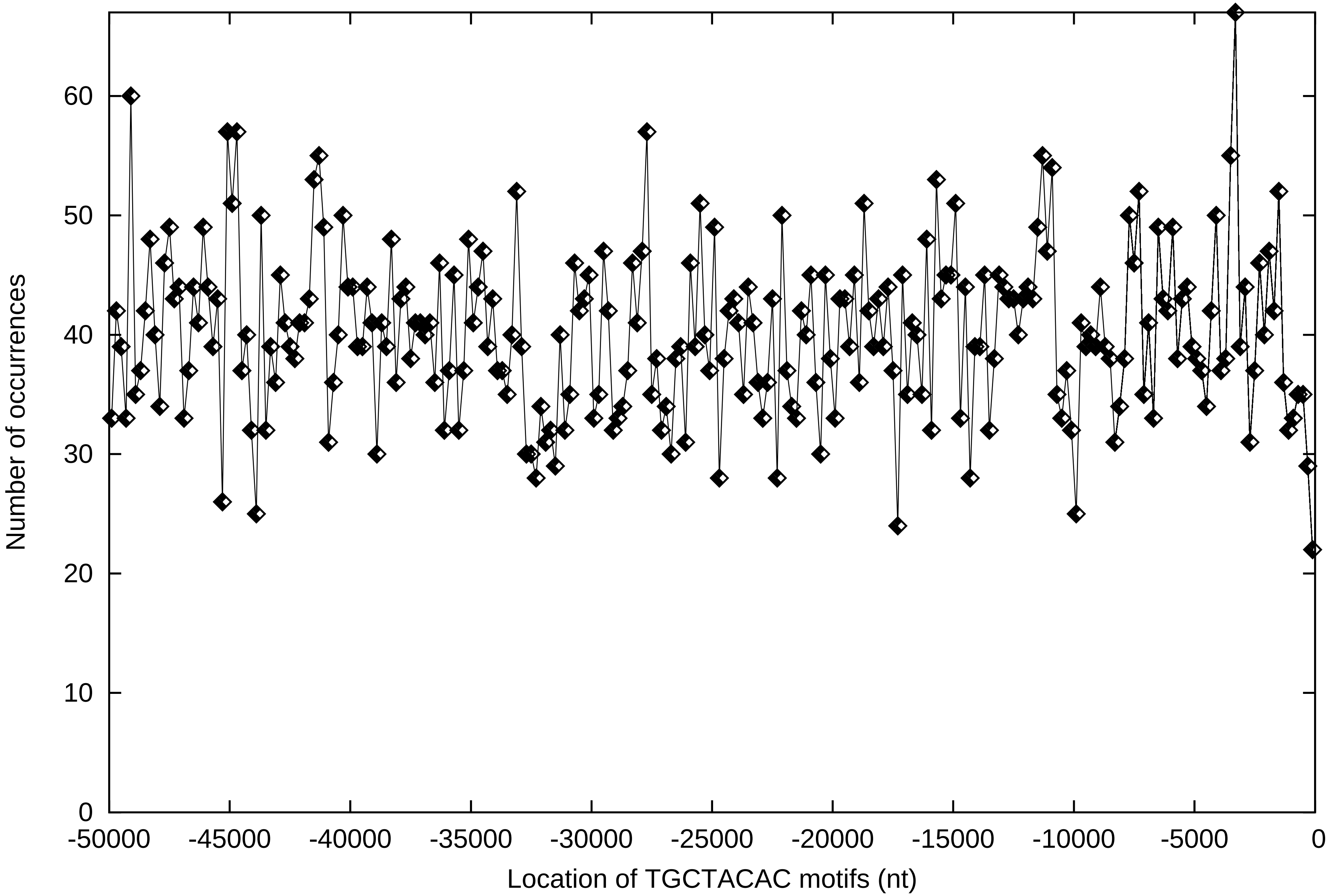

Number of occurrences
Location of TGCTACAC motifs (nt)
0
10
20
30
40
50
60
-50000
-45000
-40000
-35000
-30000
-25000
-20000
-15000
-10000
-5000
0

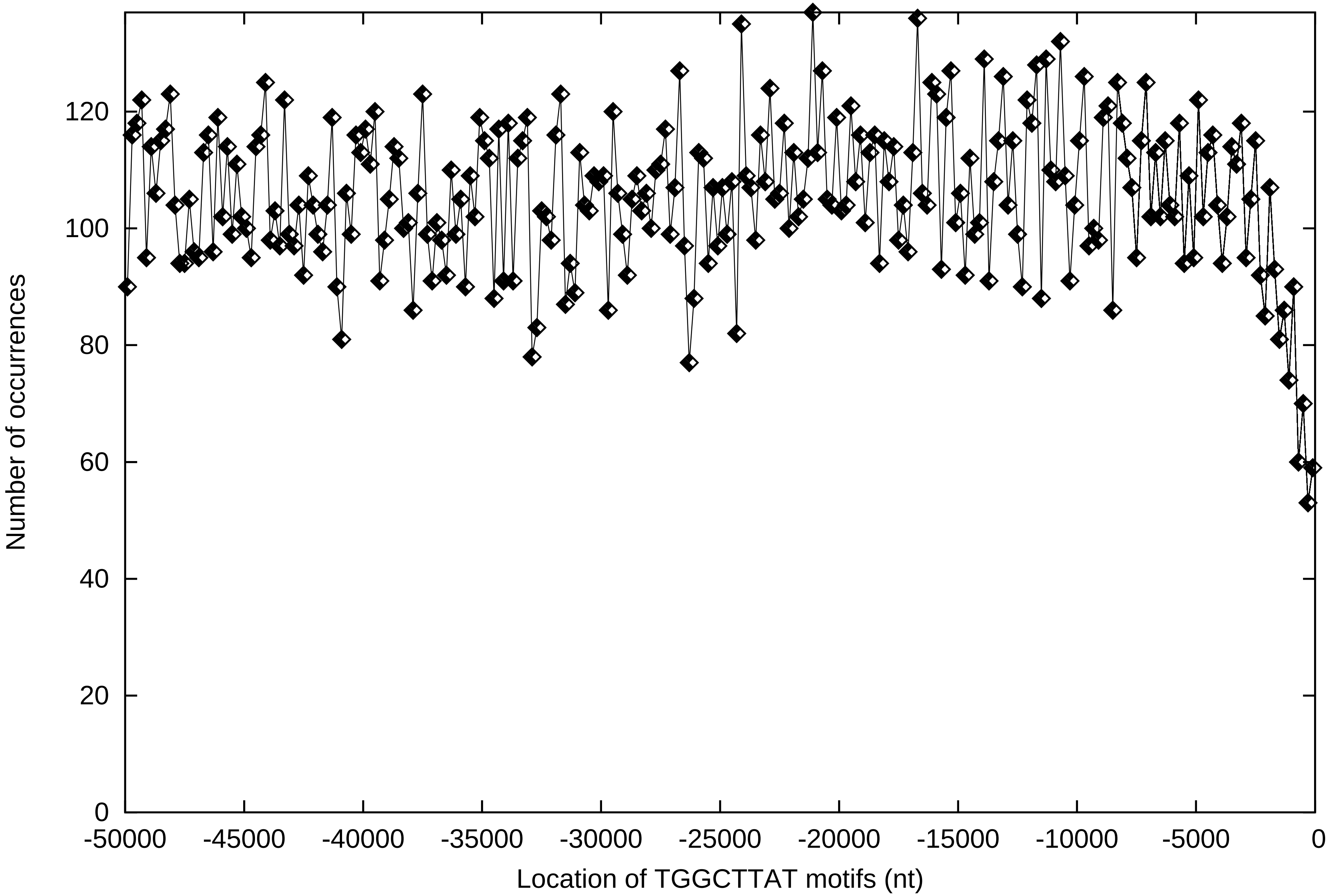

Number of occurrences
Location of TGGCTTAT motifs (nt)
0
20
40
60
80
100
120
-50000
-45000
-40000
-35000
-30000
-25000
-20000
-15000
-10000
-5000
0

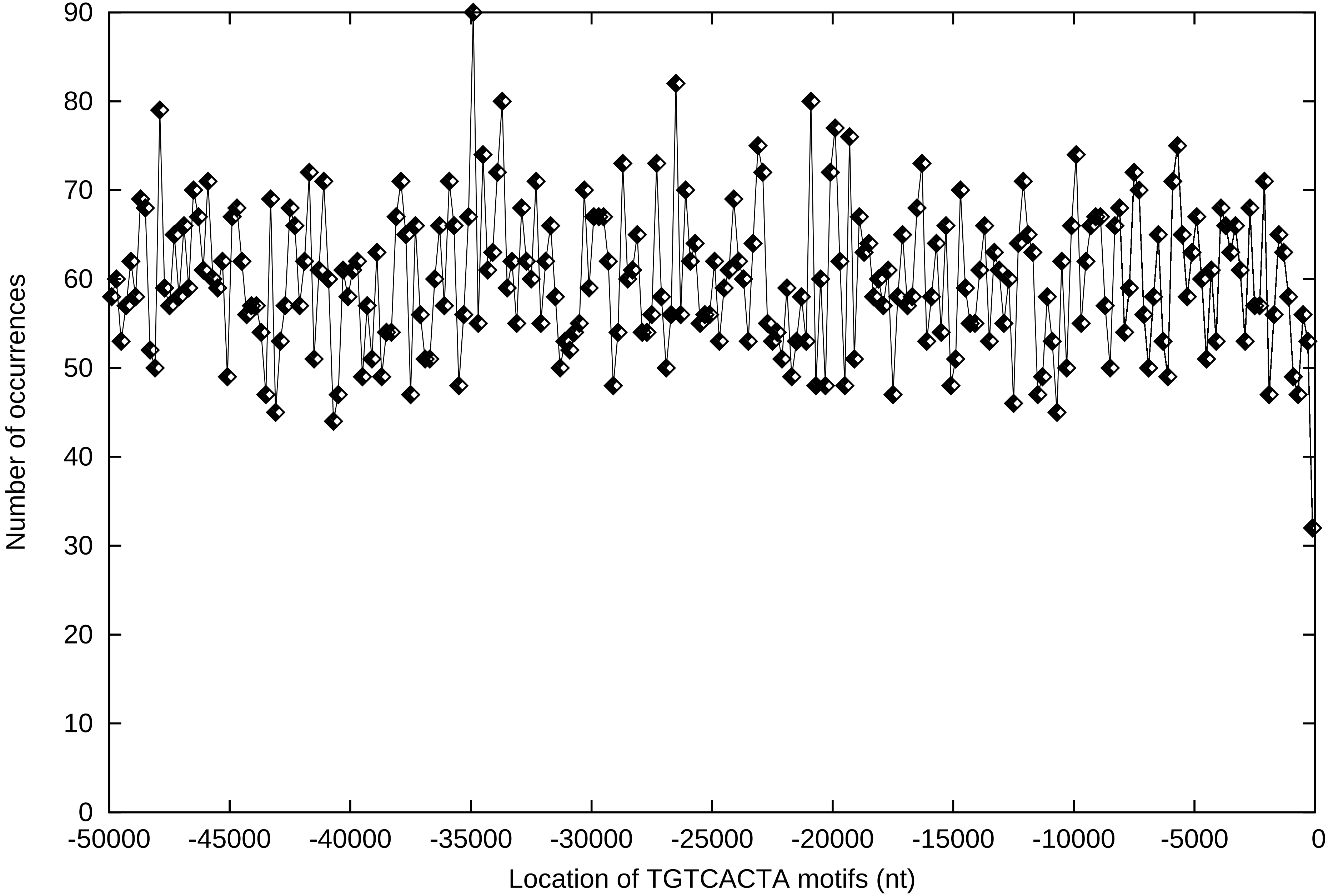

Number of occurrences
90
80
70
60
50
40
30
20
10
0
-50000
-45000
-40000
-35000
-30000
-25000
-20000
-15000
-10000
-5000
0
Location of TGTCACTA motifs (nt)

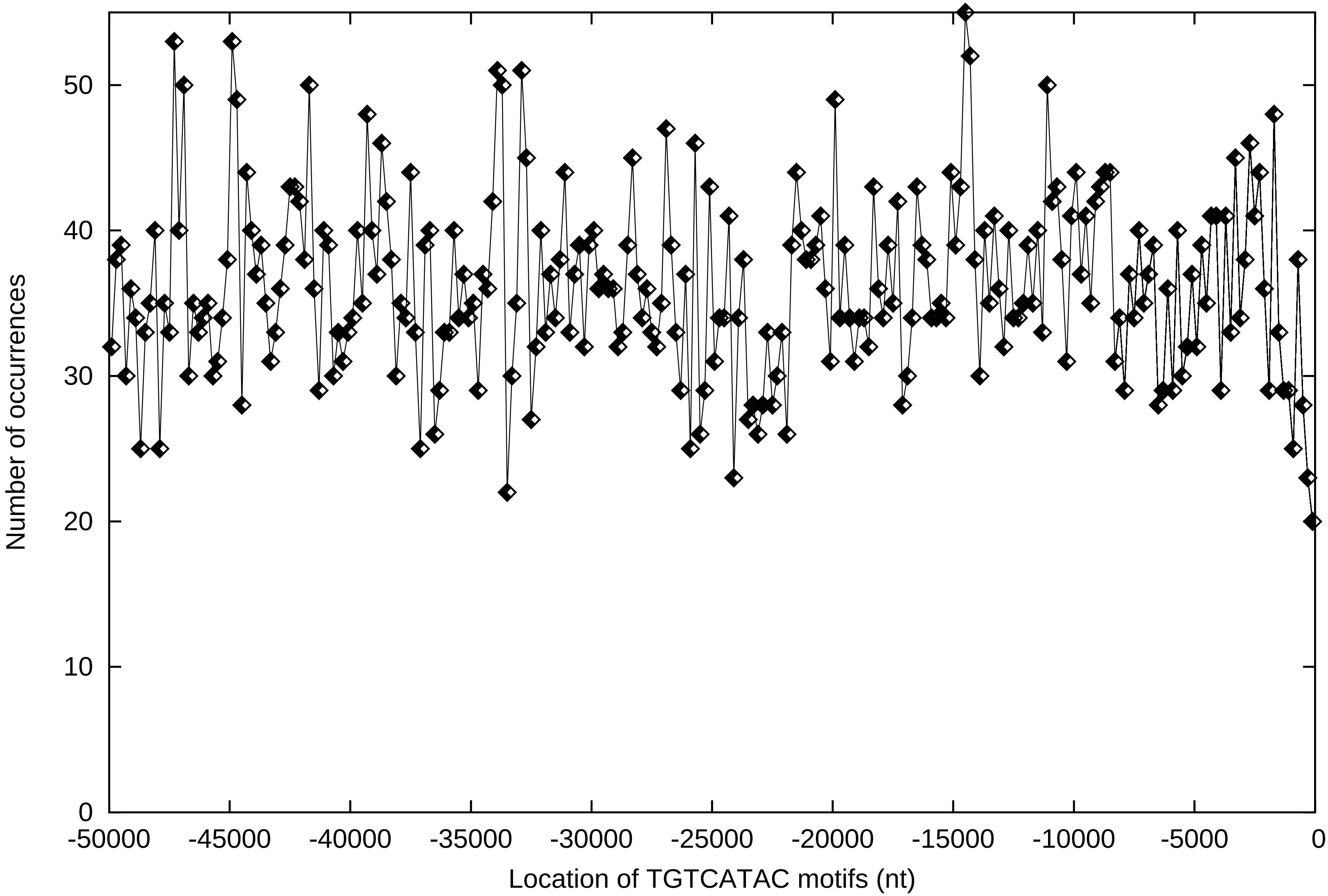

Number of occurrences
0
10
20
30
40
50
-50000
-45000
-40000
-35000
-30000
-25000
-20000
-15000
-10000
-5000
0
Location of TGTCATAC motifs (nt)

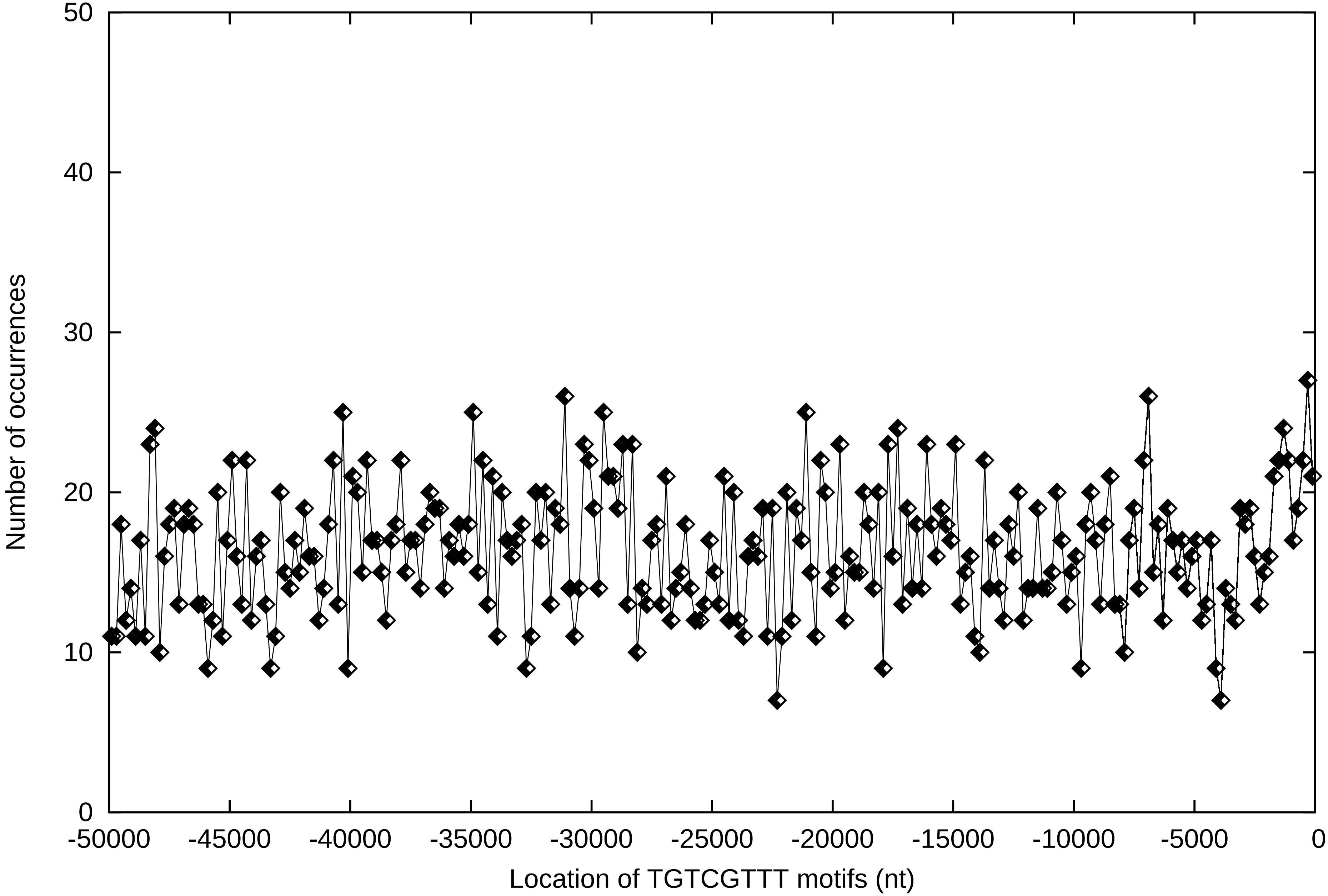

Number of occurrences
Location of TGTCGTTT motifs (nt)
0
10
20
30
40
50
-50000
-45000
-40000
-35000
-30000
-25000
-20000
-15000
-10000
-5000
0

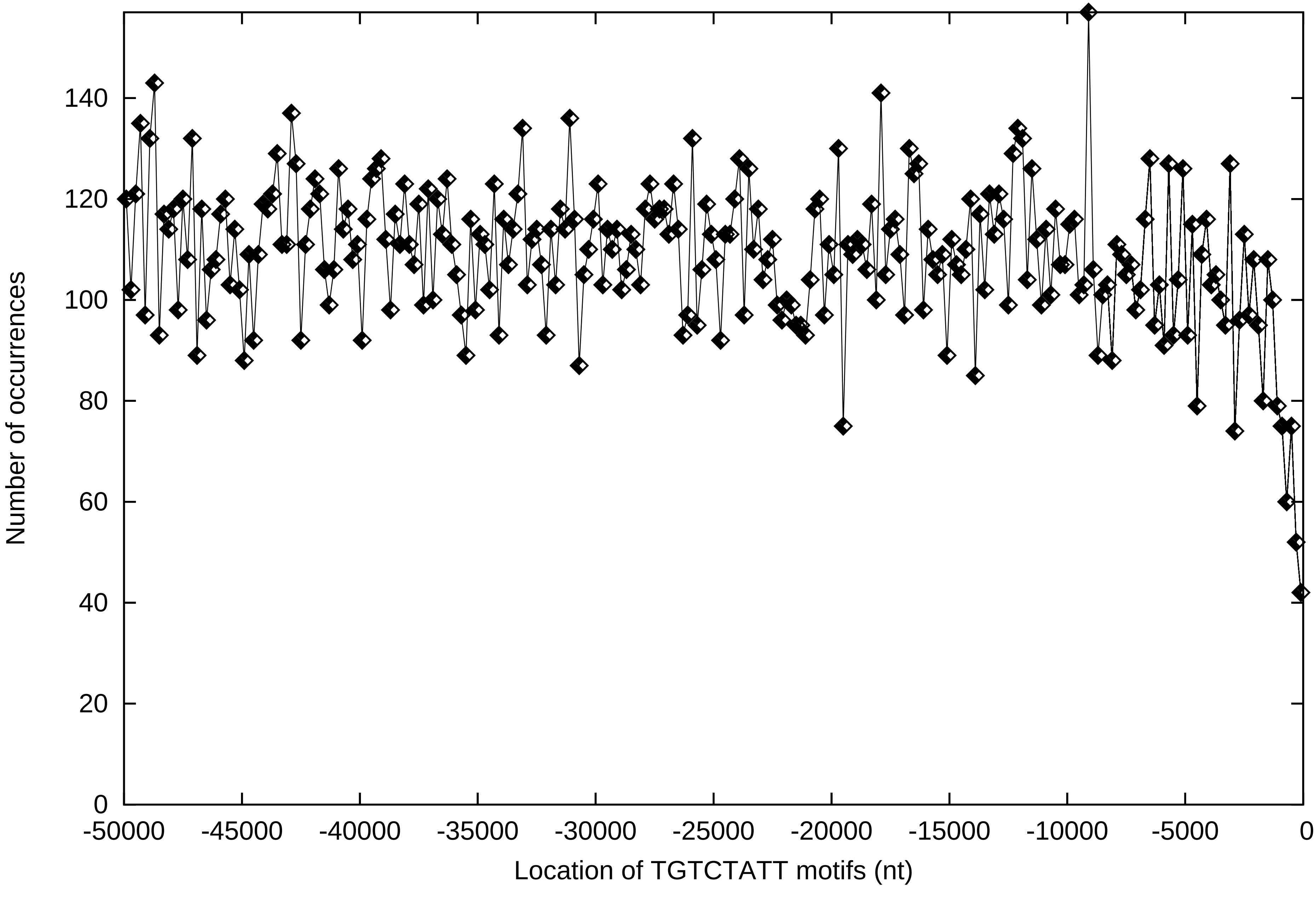

Number of occurrences
0
20
40
60
80
100
120
140
-50000
-45000
-40000
-35000
-30000
-25000
-20000
-15000
-10000
-5000
0
Location of TGTCTATT motifs (nt)

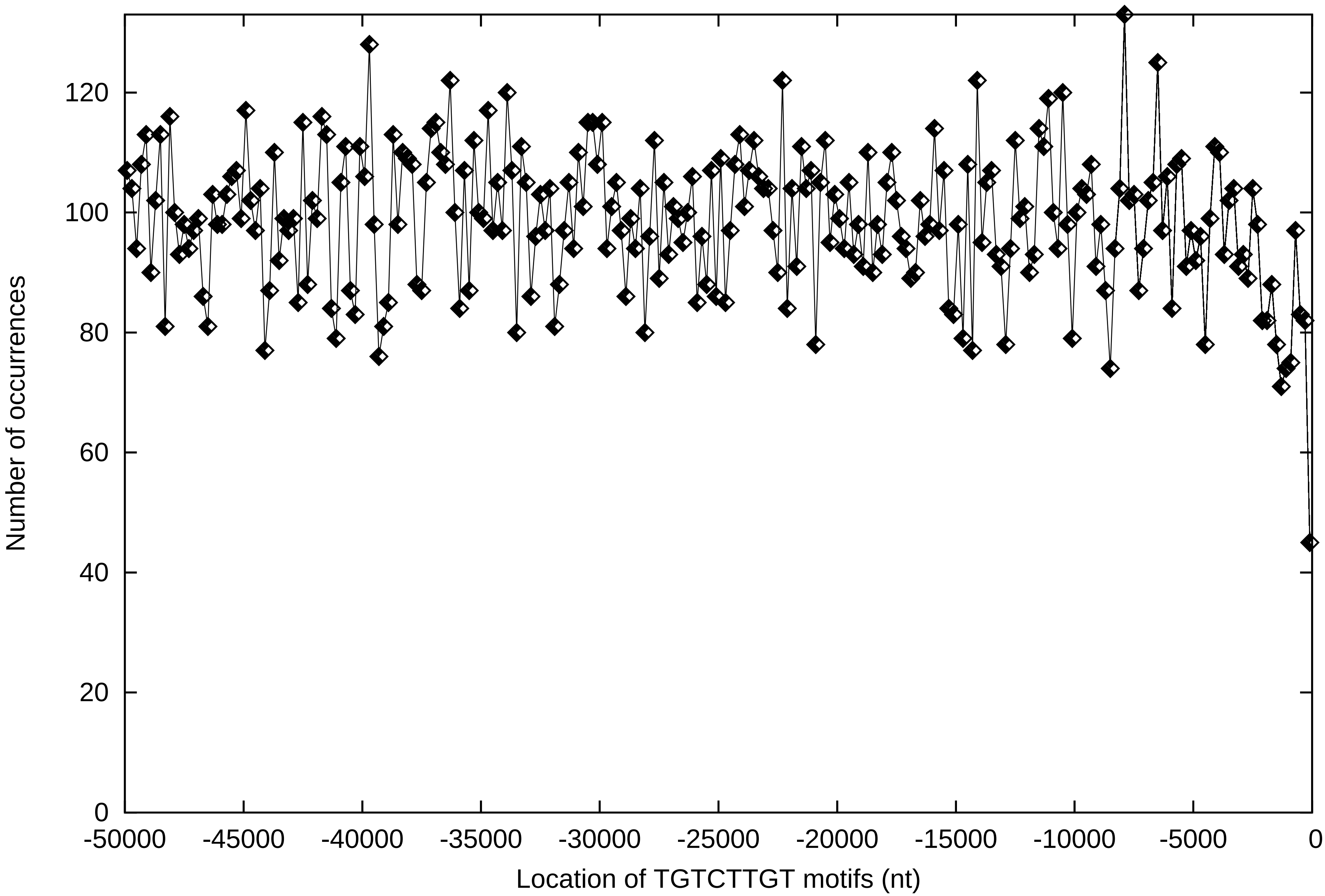

Number of occurrences
0
20
40
60
80
100
120
-50000
-45000
-40000
-35000
-30000
-25000
-20000
-15000
-10000
-5000
0
Location of TGTCTTGT motifs (nt)

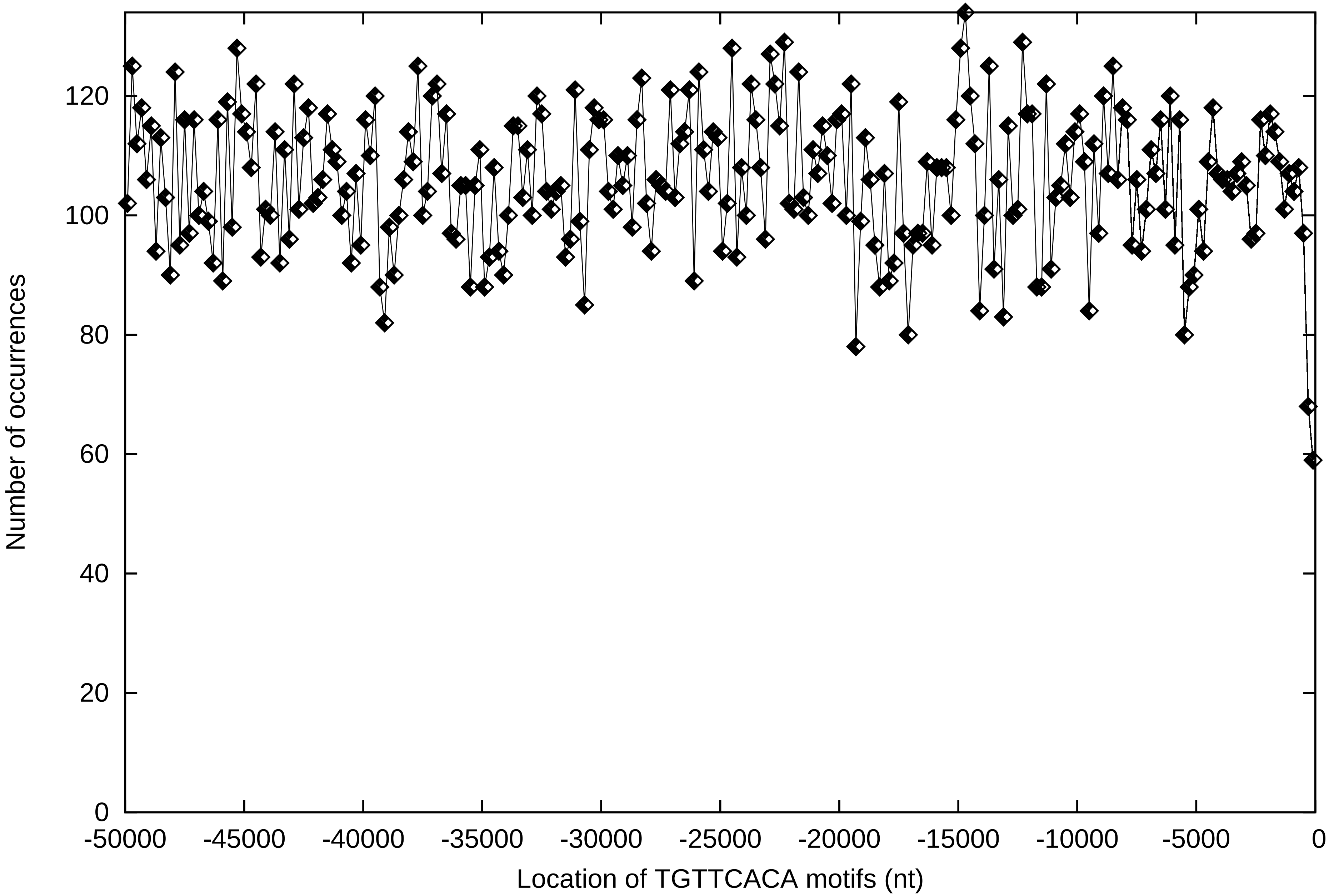

Number of occurrences
0
20
40
60
80
100
120
-50000
-45000
-40000
-35000
-30000
-25000
-20000
-15000
-10000
-5000
0
Location of TGTTCACA motifs (nt)

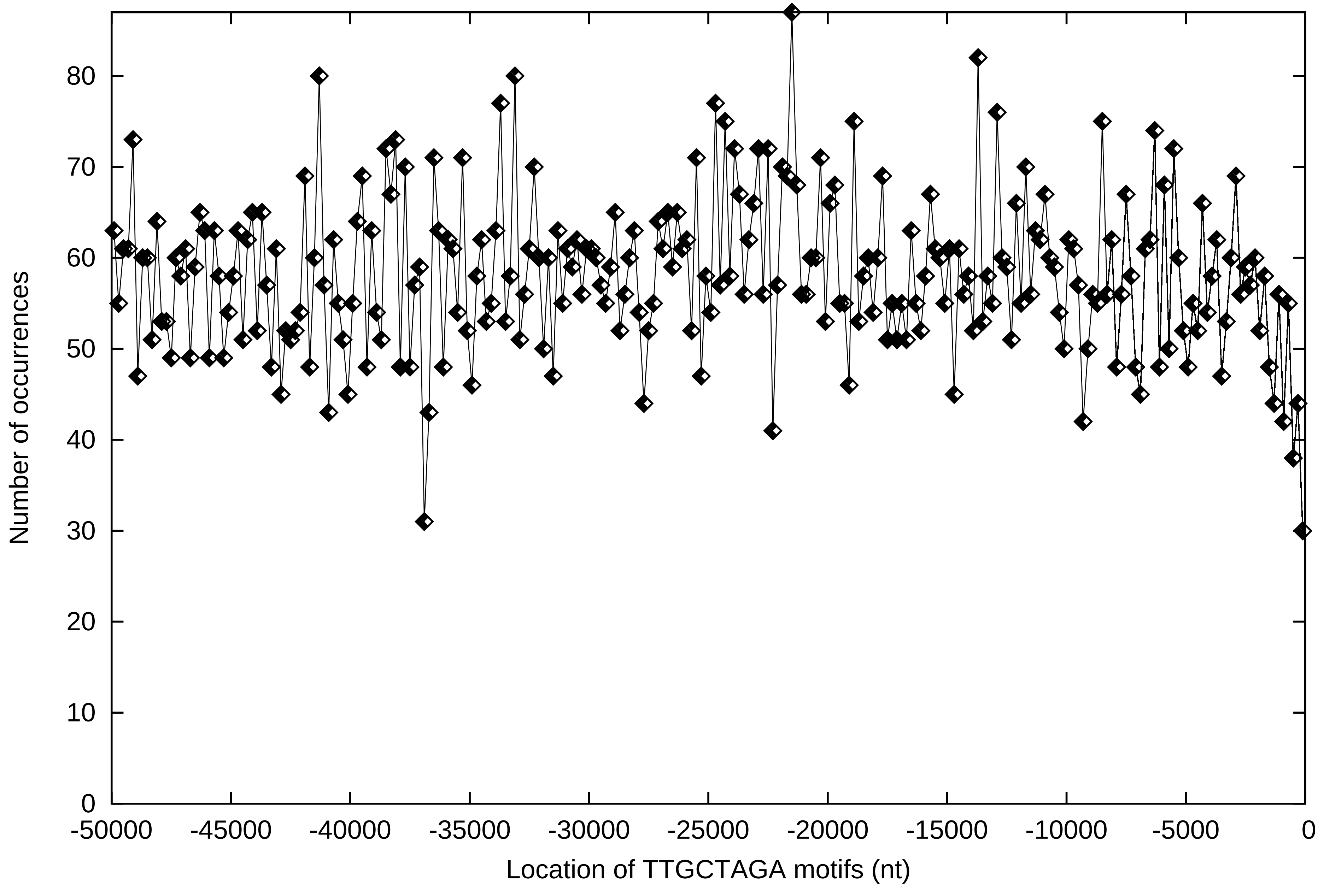
Number of occurrences
0
10
20
30
40
50
60
70
80
-50000
-45000
-40000
-35000
-30000
-25000
-20000
-15000
-10000
-5000
0
Location of TTGCTAGA motifs (nt)

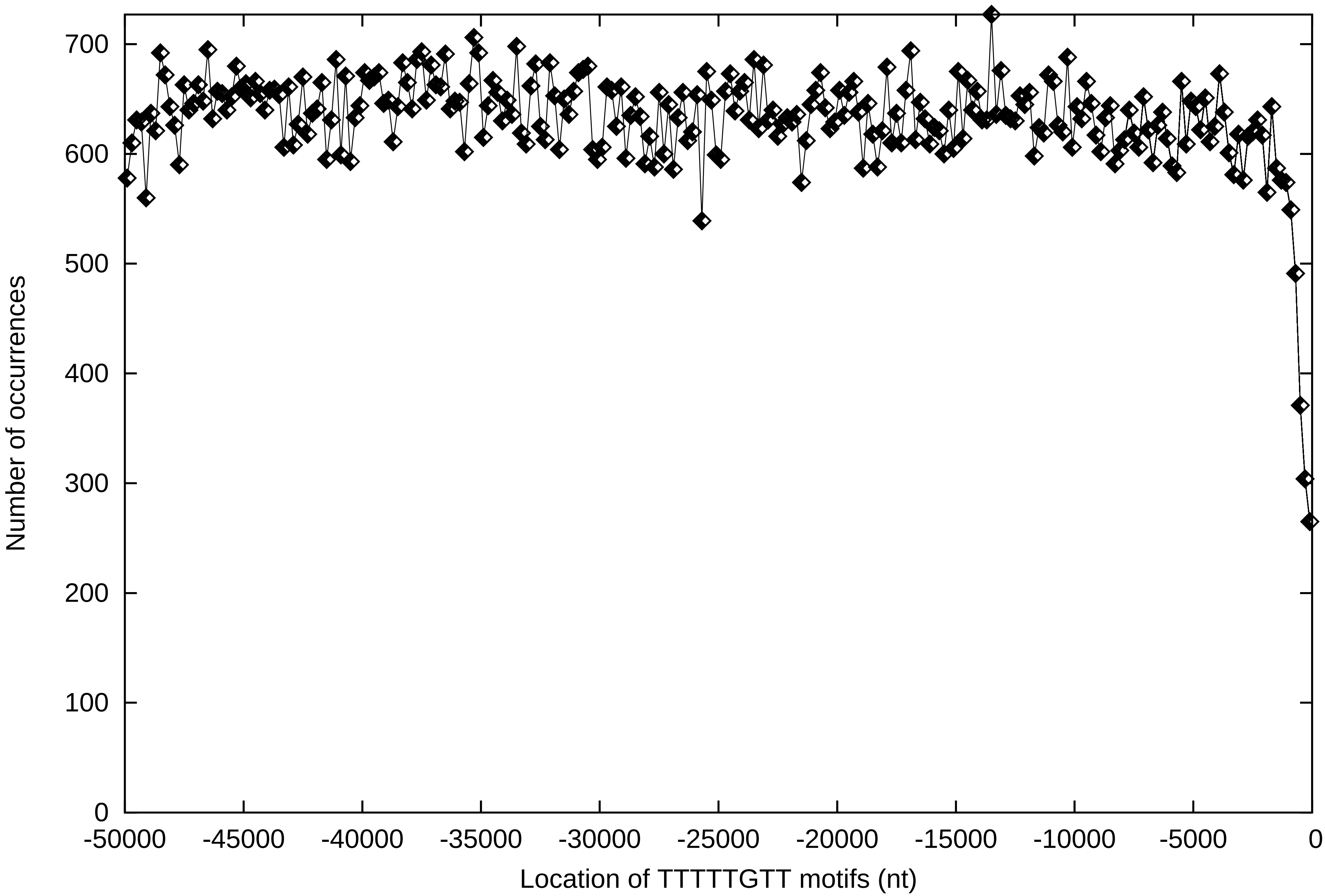

Number of occurrences
Location of TTTTTGTT motifs (nt)
700
600
500
400
300
200
100
0
-50000
-45000
-40000
-35000
-30000
-25000
-20000
-15000
-10000
-5000
0
