# Supplementary material for: Regulatory Circuit of Human MicroRNA Biogenesis
Source: PLoS Comput Biol. 2007 Apr 20;3(4):e67. doi: 10.1371/journal.pcbi.0030067 (PMC1853126; doi:10.1371/journal.pcbi.0030067)
Supplement: Supplementary file 9 [file pcbi.0030067.sg009.pdf]

Distance distribution of randomly selected 6 mers (pages 2-101). Each graph is a histogram that represents the number of occurrences of each motif (every 200 nt stretch) in the first 50 kb upstream region of protein-coding genes. In stark contrast to the top-predicted motifs, the control motifs do not have a strong preference to occur in the neighbor hood of the transcription start sites of protein-coding genes.

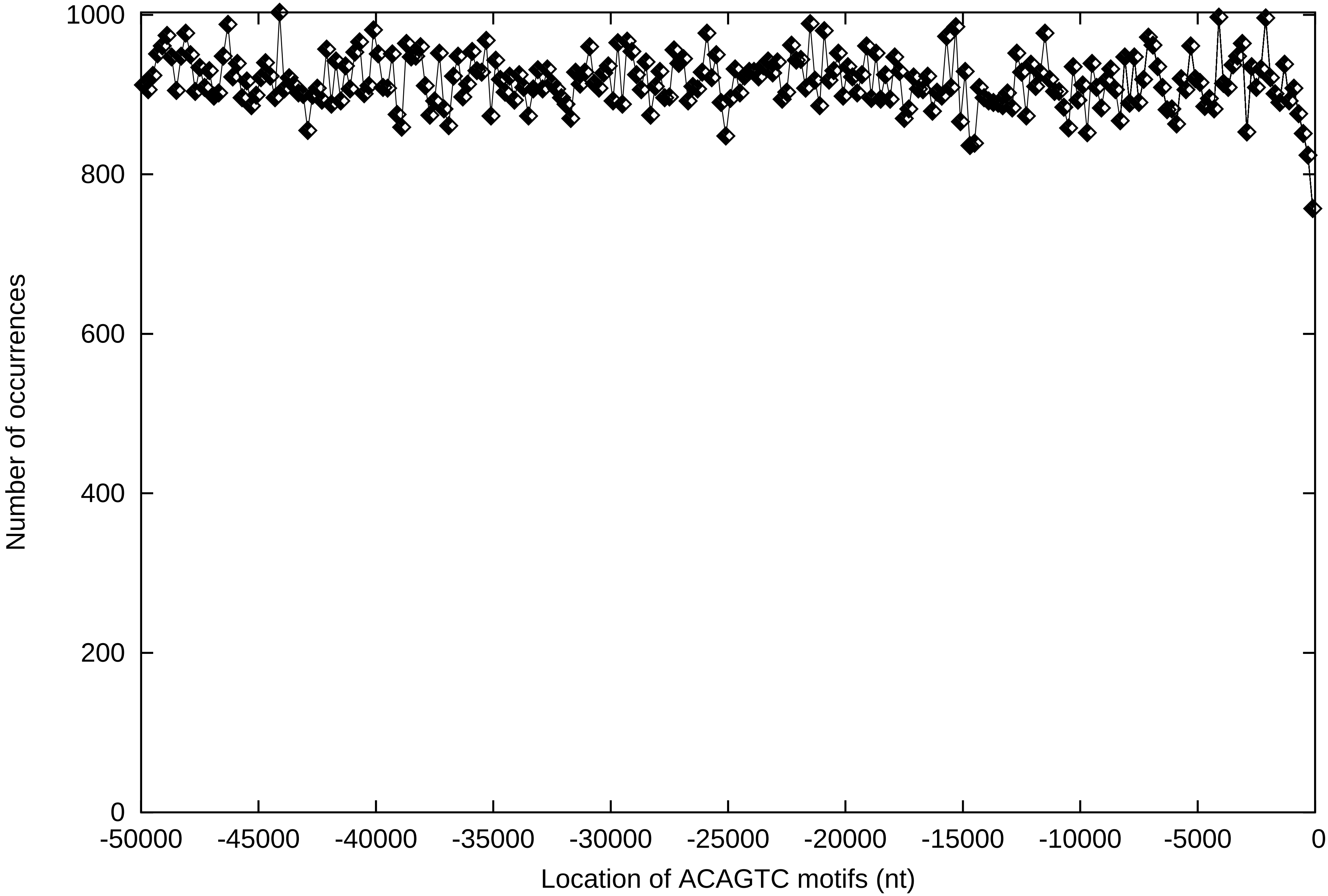

1000
800
600
400
200
0
-50000
-45000
-40000
-35000
-30000
-25000
-20000
-15000
-10000
-5000
0
Number of occurrences
Location of ACAGTC motifs (nt)

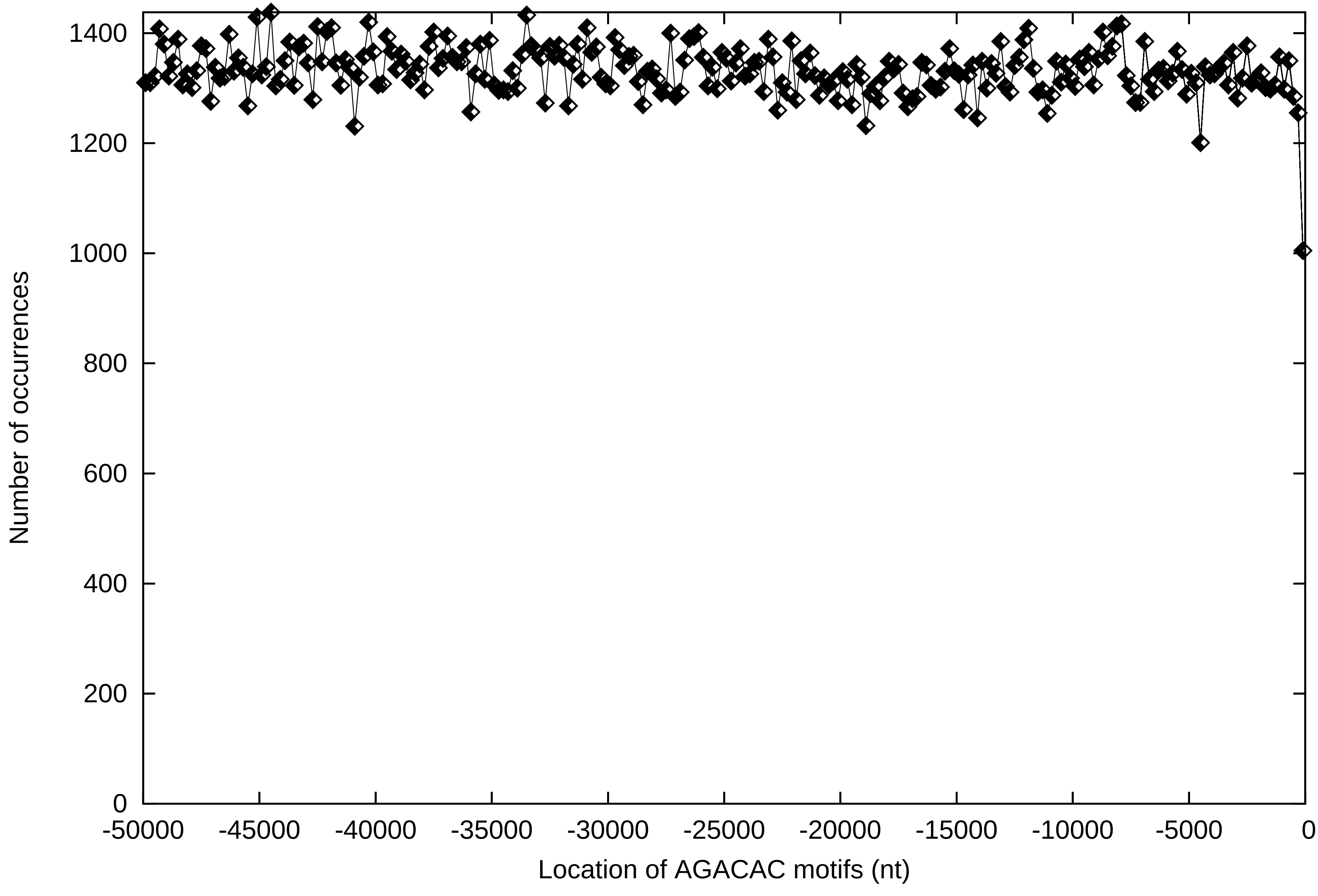
Number of occurrences
Location of AGACAC motifs (nt)
0
200
400
600
800
1000
1200
1400
-50000
-45000
-40000
-35000
-30000
-25000
-20000
-15000
-10000
-5000
0

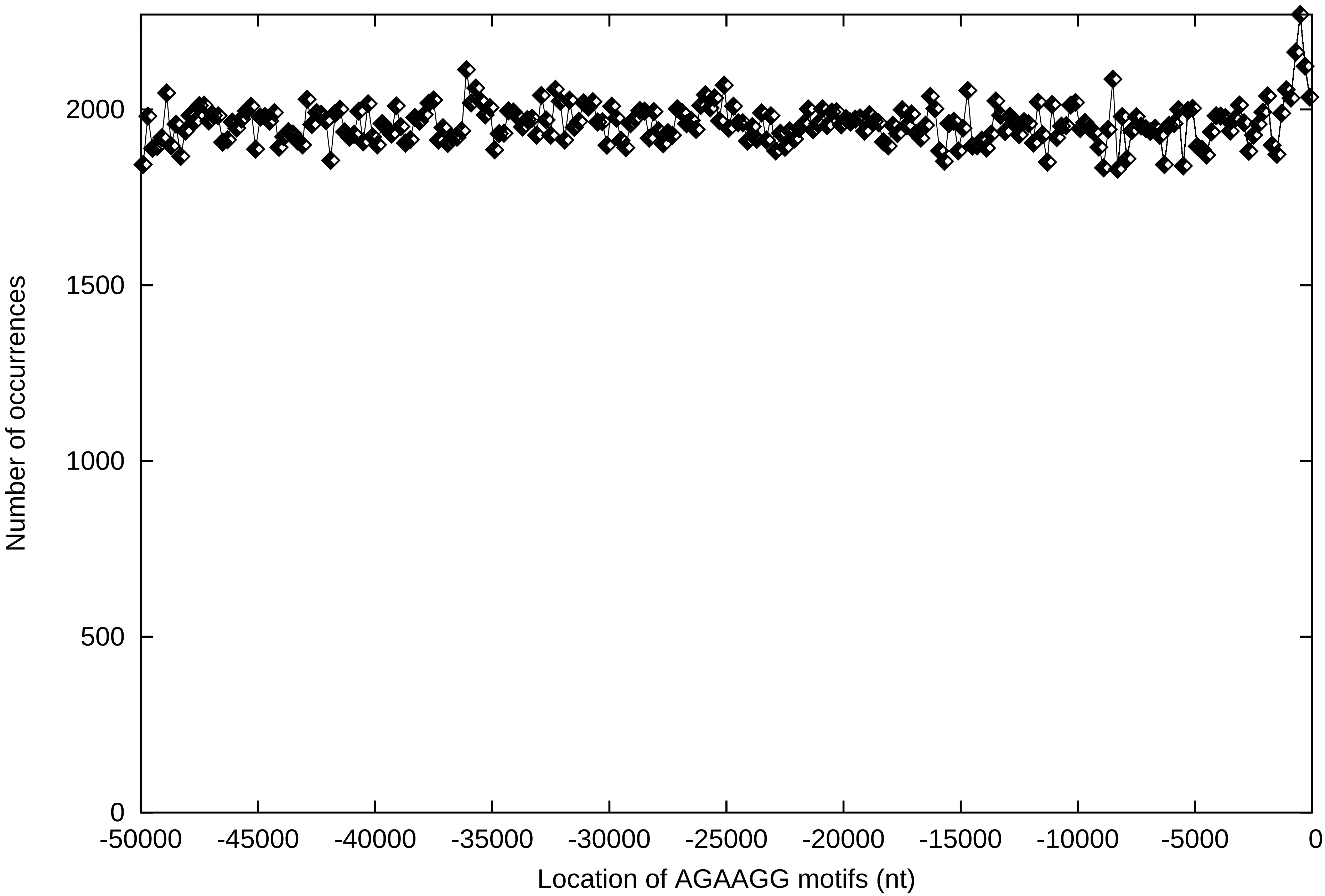

Number of occurrences
0
500
1000
1500
2000
-50000
-45000
-40000
-35000
-30000
-25000
-20000
-15000
-10000
-5000
0
Location of AGAAGG motifs (nt)

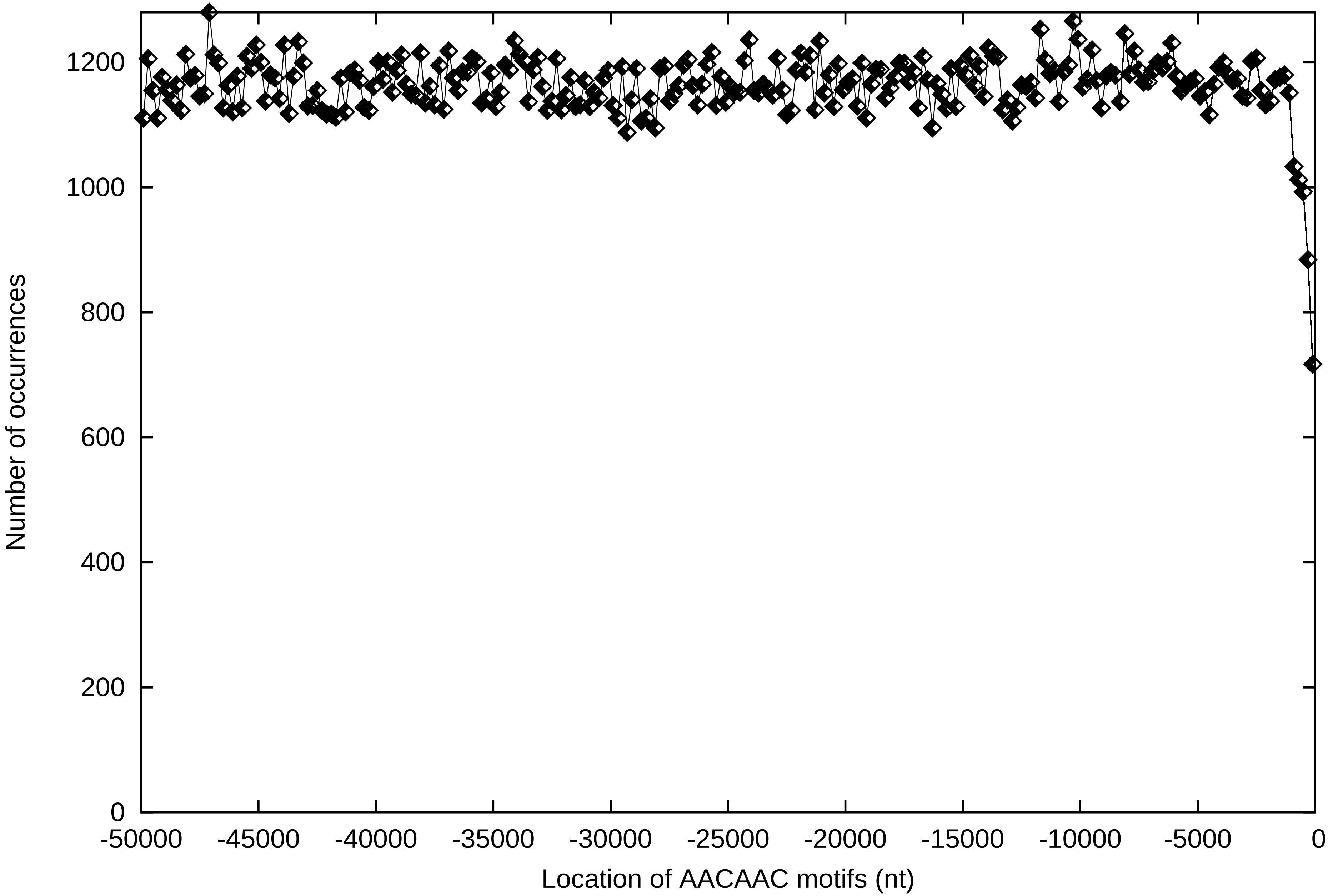

Number of occurrences
1200
1000
800
600
400
200
0
-50000
-45000
-40000
-35000
-30000
-25000
-20000
-15000
-10000
-5000
0
Location of AACAAC motifs (nt)

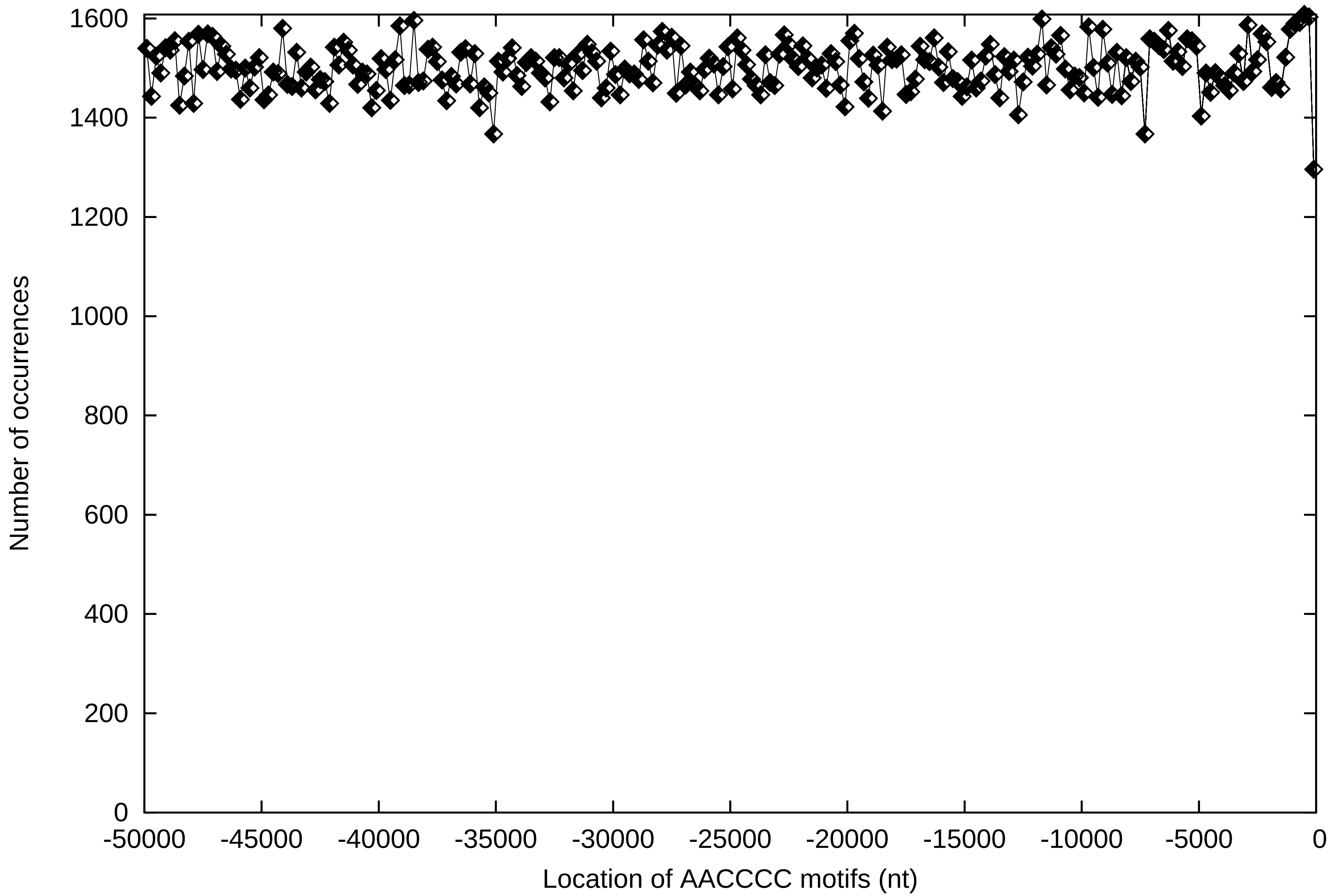
1600
1400
1200
1000
800
600
400
200
0
-50000
-45000
-40000
-35000
-30000
-25000
-20000
-15000
-10000
-5000
0
Number of occurrences
Location of AACCCC motifs (nt)

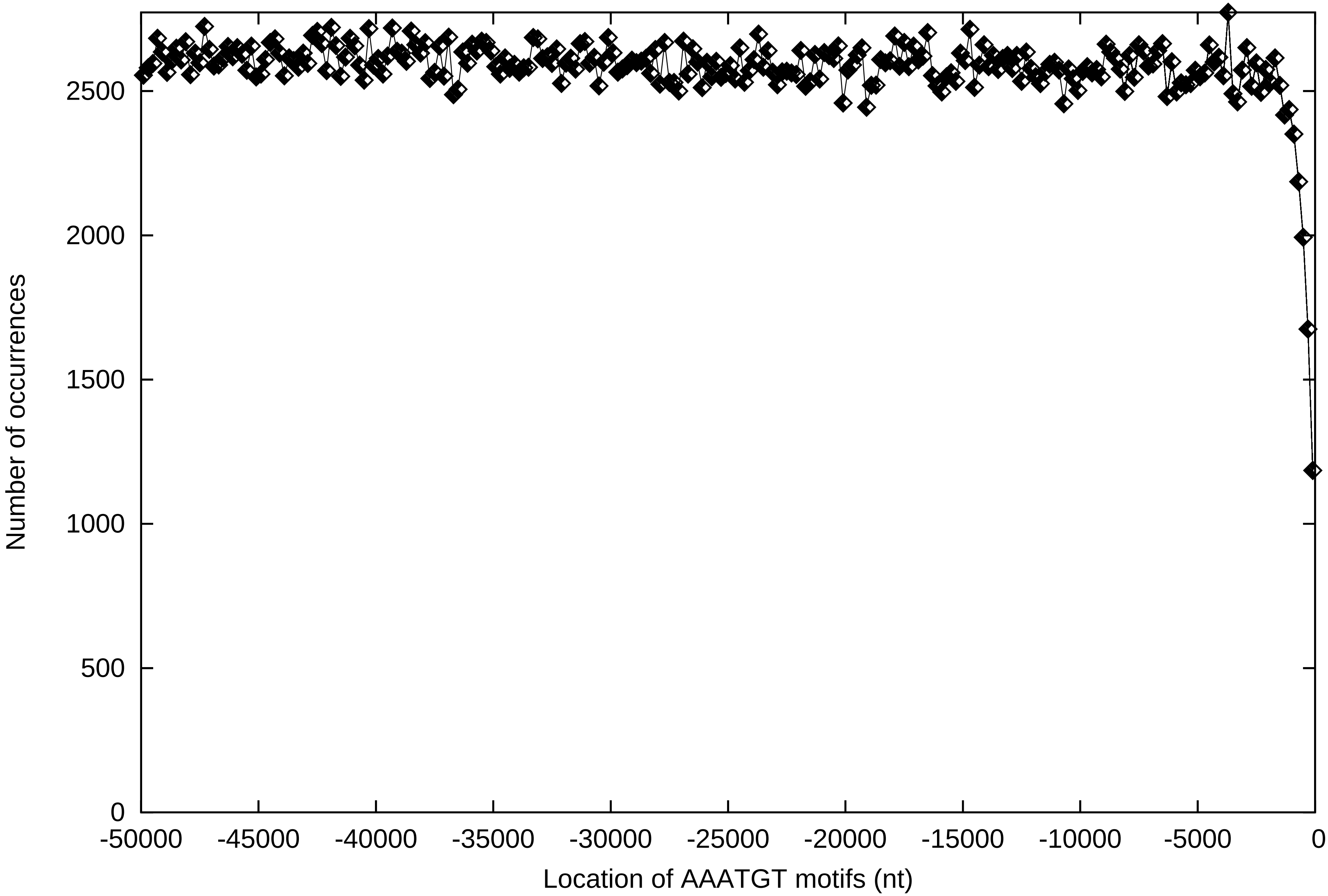
Number of occurrences
0
500
1000
1500
2000
2500
-50000
-45000
-40000
-35000
-30000
-25000
-20000
-15000
-10000
-5000
0
Location of AAATGT motifs (nt)

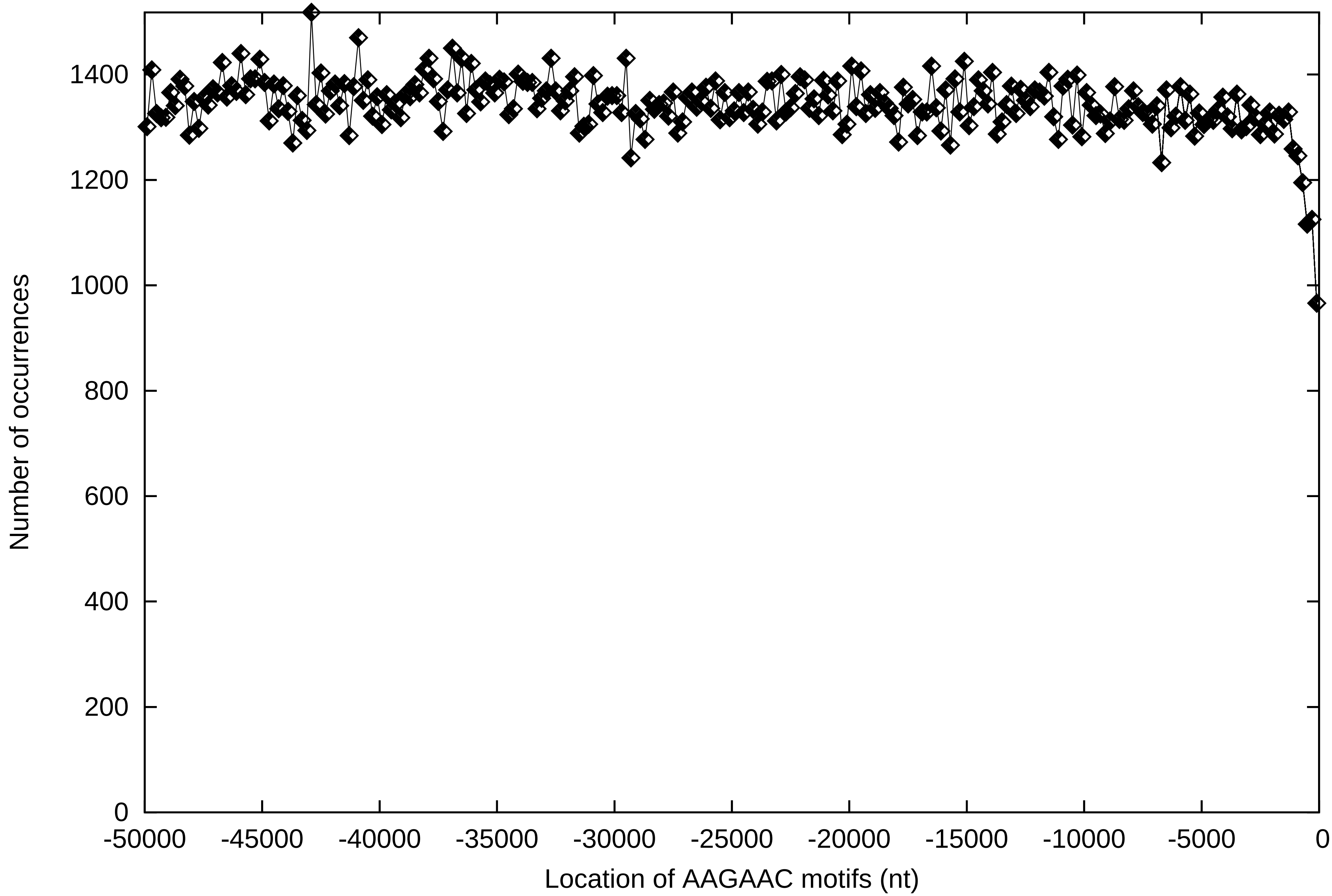

Number of occurrences
1400
1200
1000
800
600
400
200
0
-50000
-45000
-40000
-35000
-30000
-25000
-20000
-15000
-10000
-5000
0
Location of AAGAAC motifs (nt)

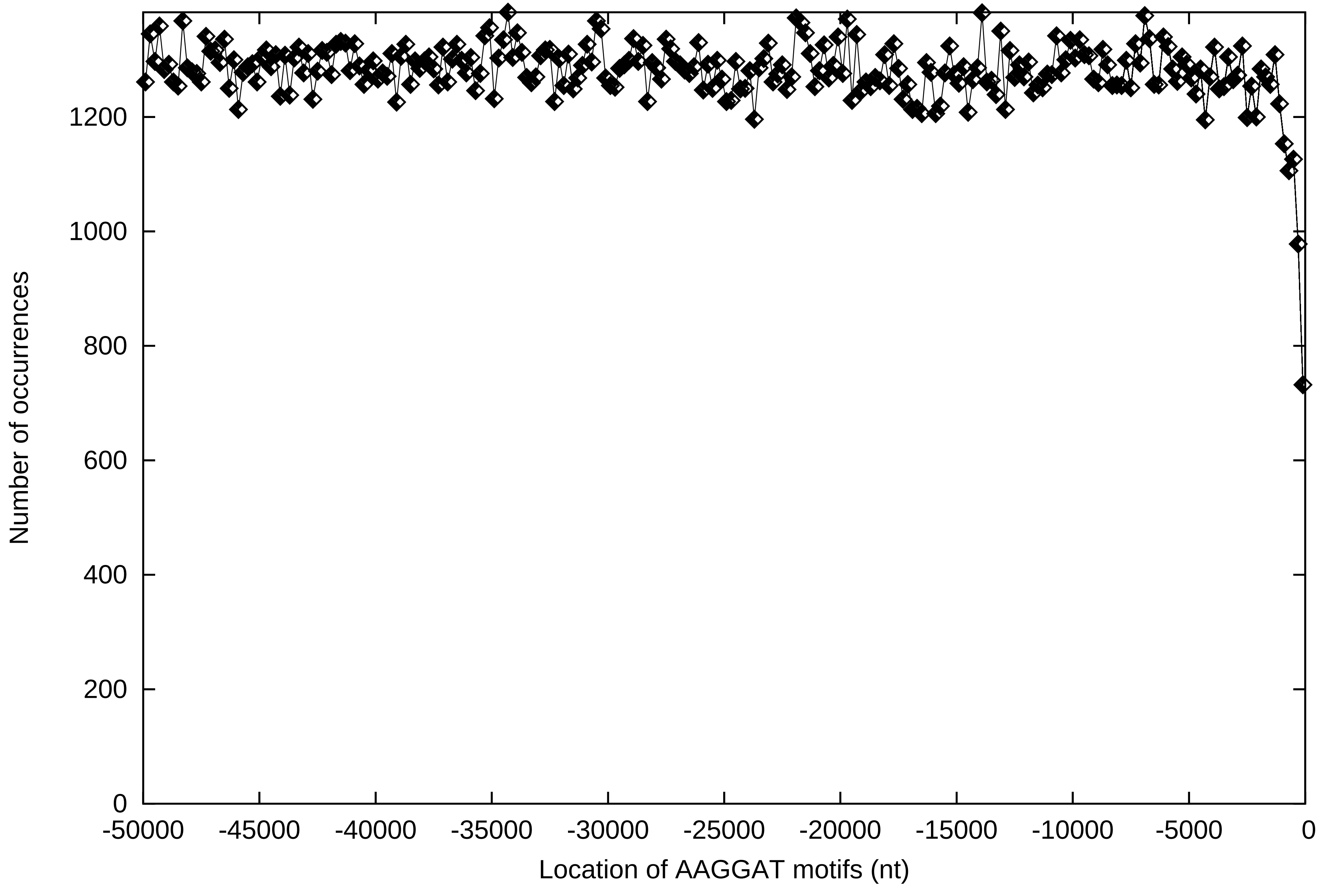

Number of occurrences
1200
1000
800
600
400
200
0
-50000
-45000
-40000
-35000
-30000
-25000
-20000
-15000
-10000
-5000
0
Location of AAGGAT motifs (nt)

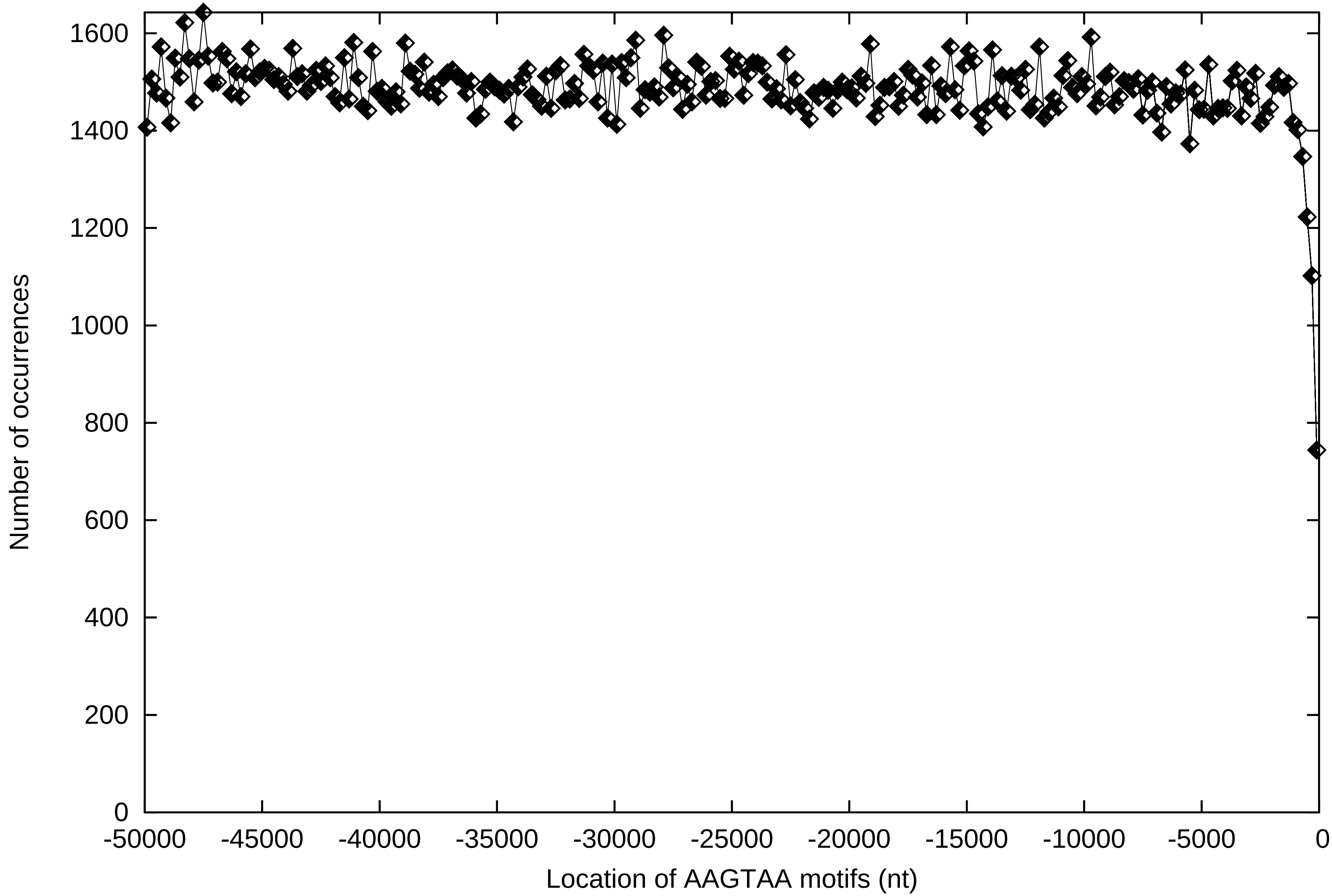

Number of occurrences
Location of AAGTAA motifs (nt)
0
200
400
600
800
1000
1200
1400
1600
-50000
-45000
-40000
-35000
-30000
-25000
-20000
-15000
-10000
-5000
0

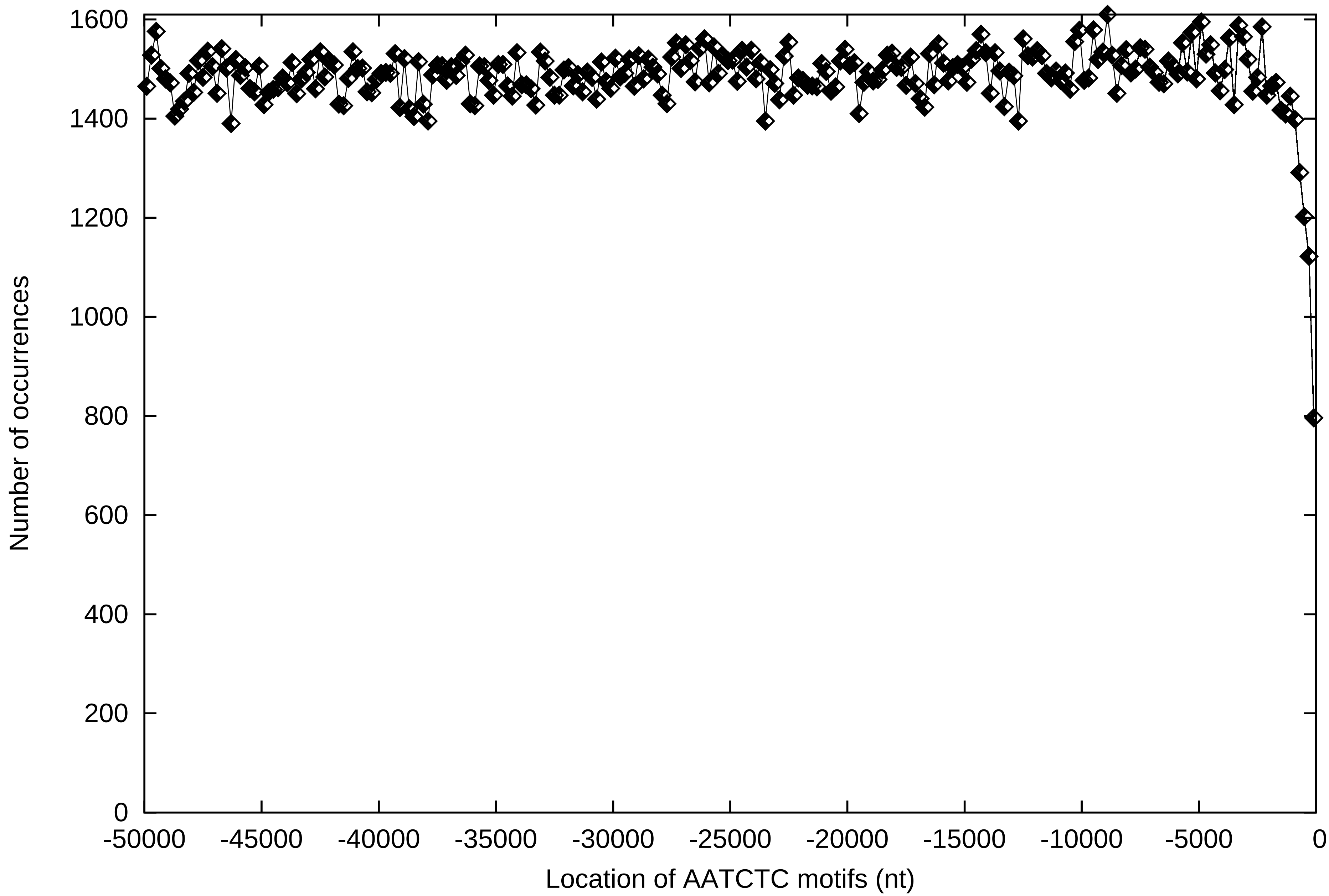

1600
1400
1200
1000
800
600
400
200
0
-50000
-45000
-40000
-35000
-30000
-25000
-20000
-15000
-10000
-5000
0
Number of occurrences
Location of AATCTC motifs (nt)

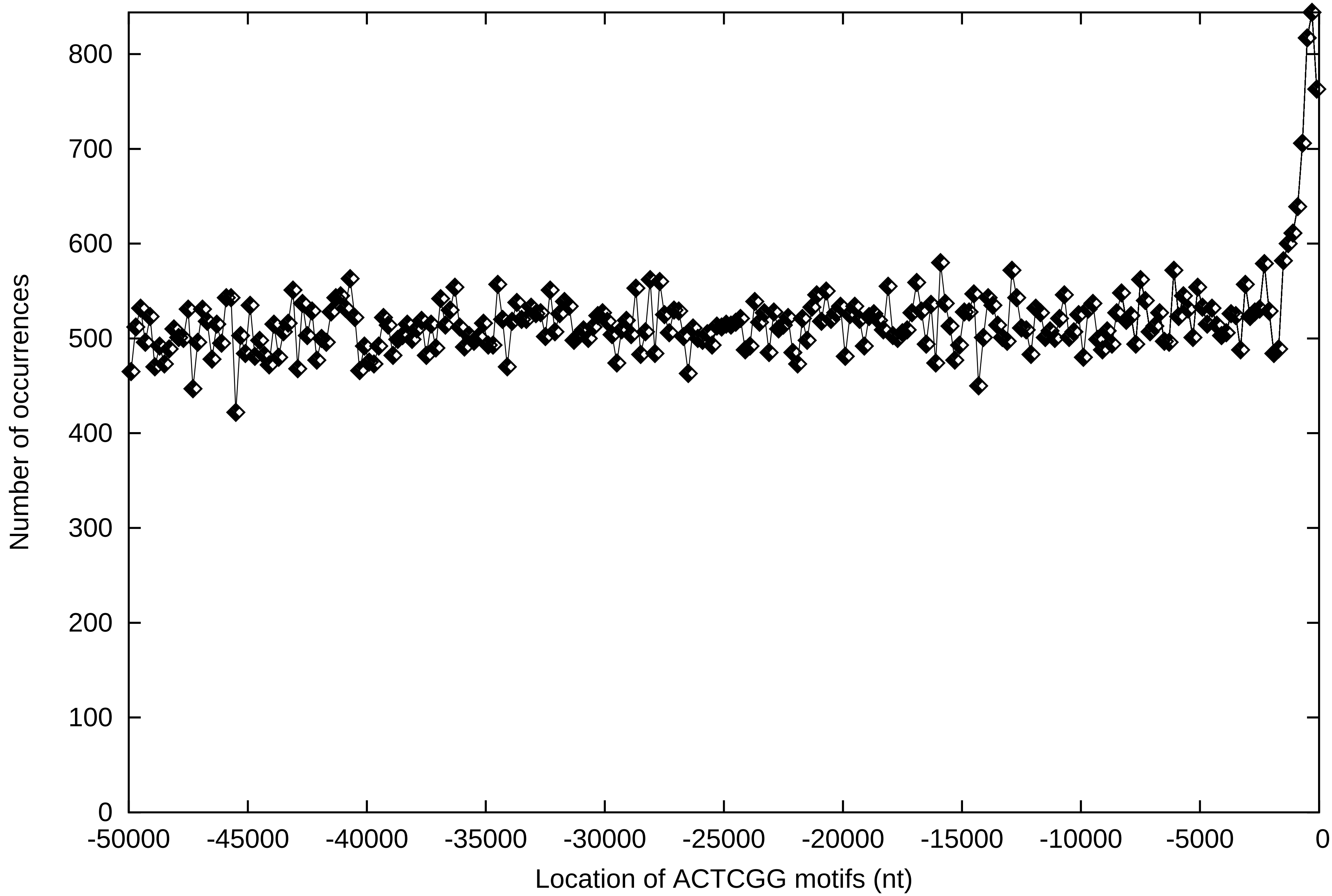

Number of occurrences
0
100
200
300
400
500
600
700
800
-50000
-45000
-40000
-35000
-30000
-25000
-20000
-15000
-10000
-5000
0
Location of ACTCGG motifs (nt)

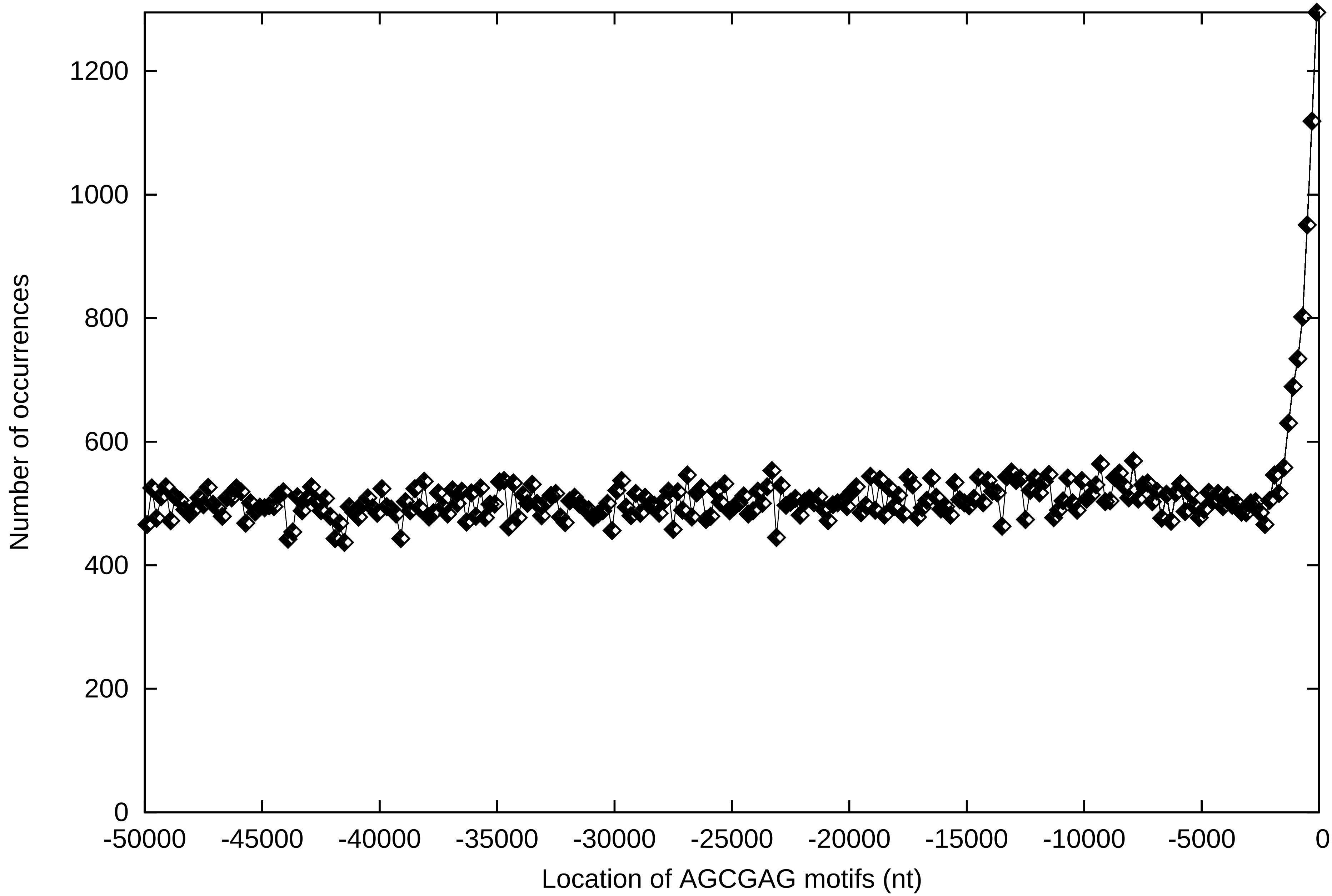

Number of occurrences
0
200
400
600
800
1000
1200
-50000
-45000
-40000
-35000
-30000
-25000
-20000
-15000
-10000
-5000
0
Location of AGCGAG motifs (nt)

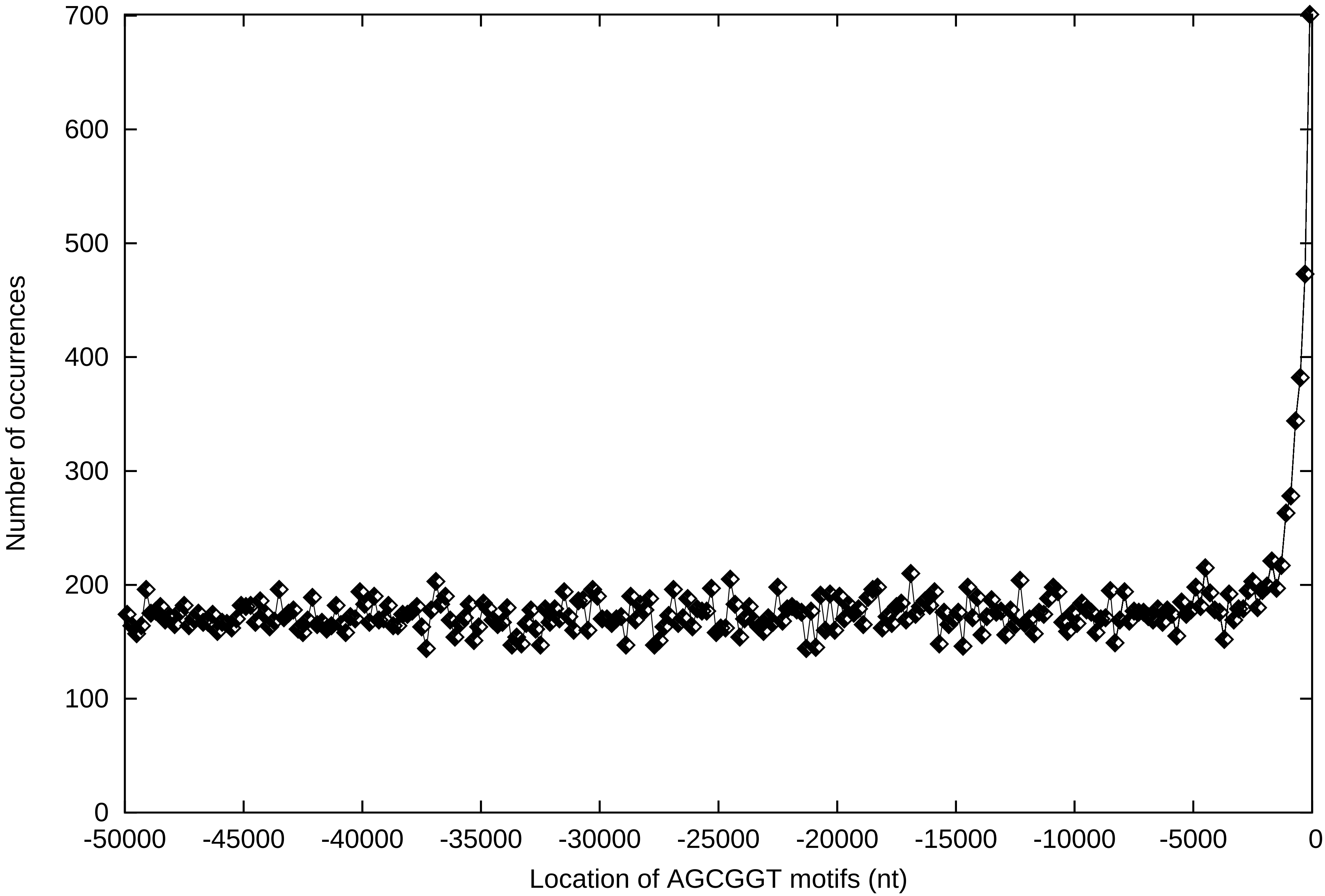

700
600
500
400
300
200
100
0
-50000
-45000
-40000
-35000
-30000
-25000
-20000
-15000
-10000
-5000
0
Number of occurrences
Location of AGCGGT motifs (nt)

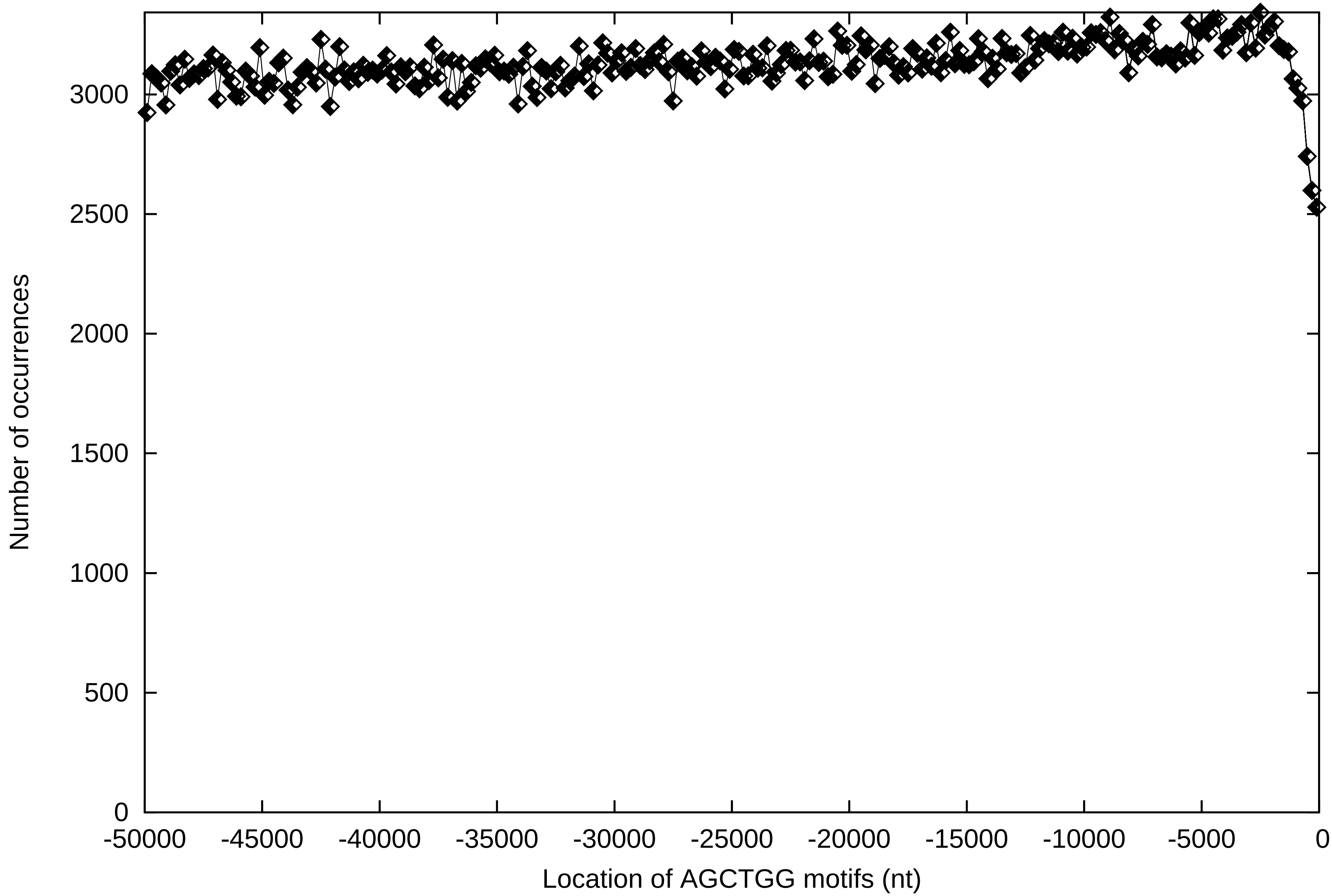

Number of occurrences
0
500
1000
1500
2000
2500
3000
-50000
-45000
-40000
-35000
-30000
-25000
-20000
-15000
-10000
-5000
0
Location of AGCTGG motifs (nt)

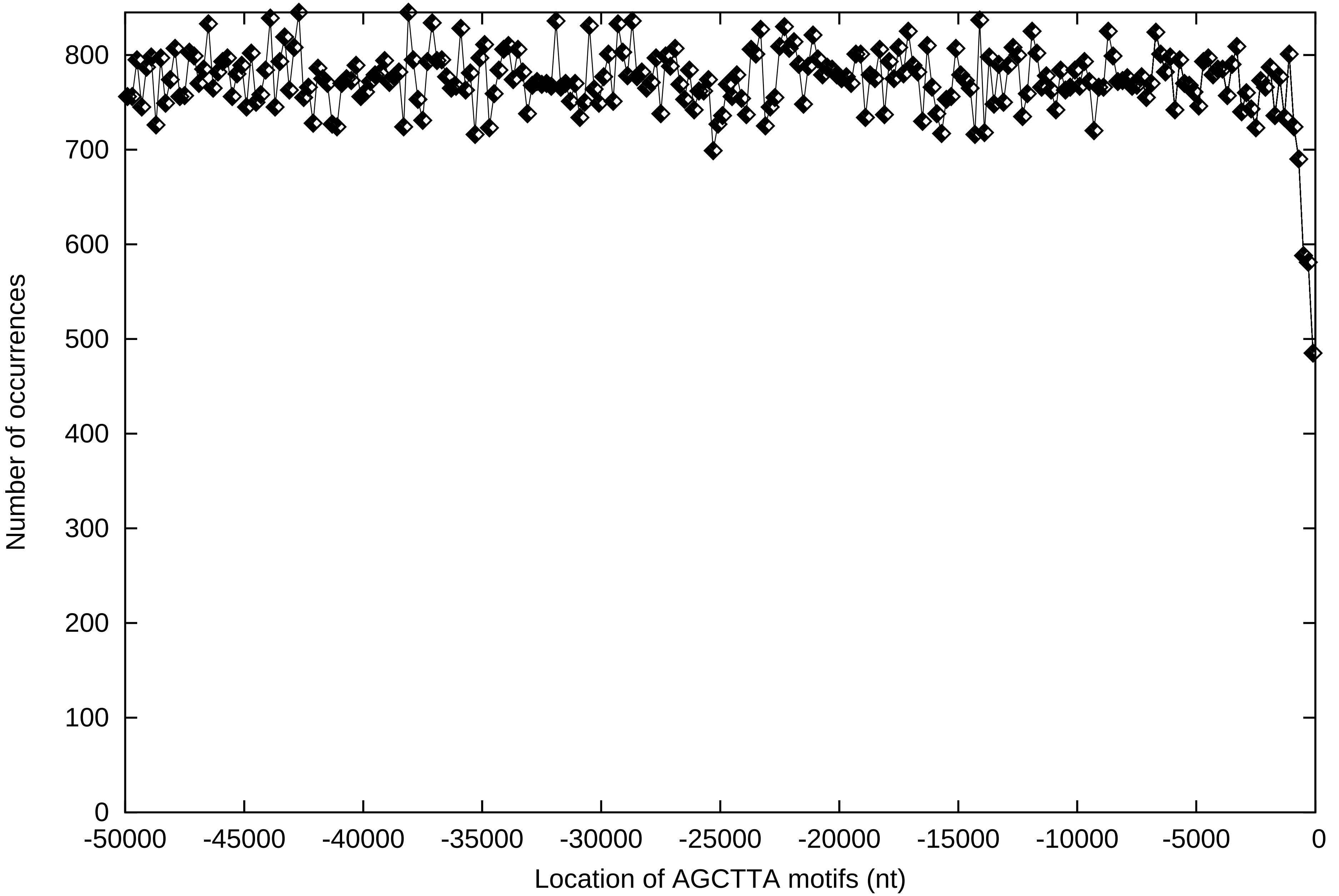

Number of occurrences
0
100
200
300
400
500
600
700
800
-50000
-45000
-40000
-35000
-30000
-25000
-20000
-15000
-10000
-5000
0
Location of AGCTTA motifs (nt)

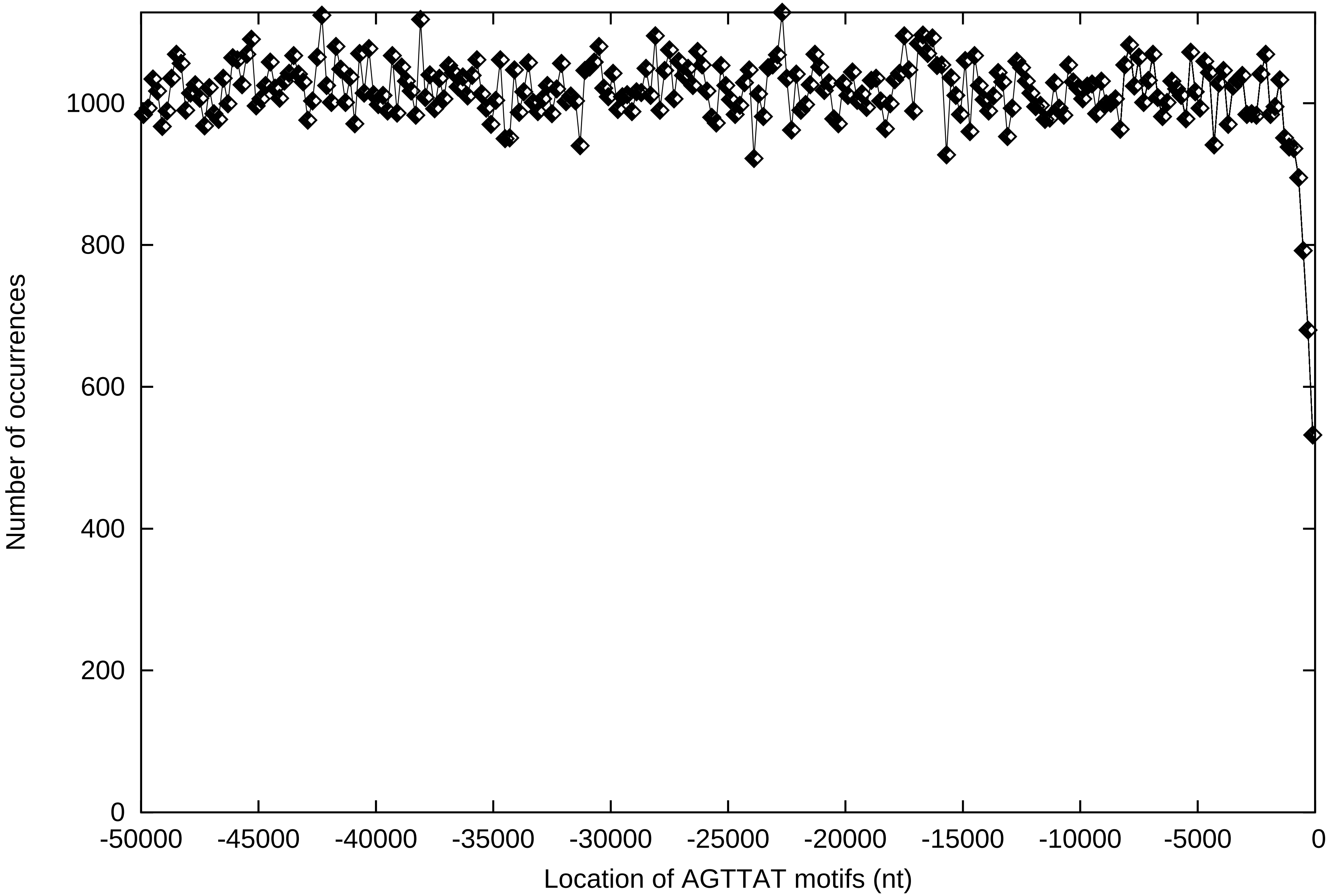

Number of occurrences
1000
800
600
400
200
0
-50000
-45000
-40000
-35000
-30000
-25000
-20000
-15000
-10000
-5000
0
Location of AGTTAT motifs (nt)

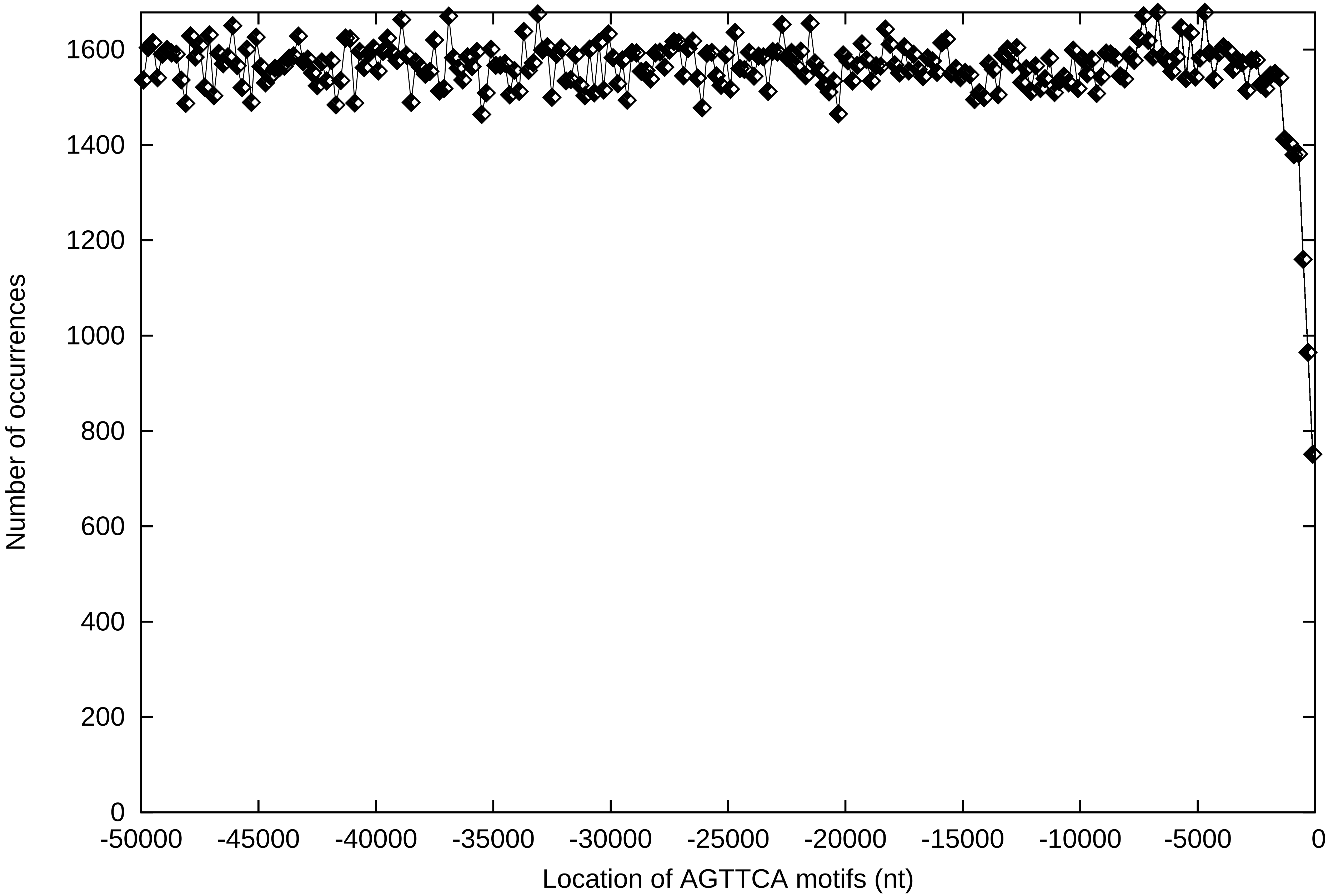

Number of occurrences
Location of AGTTCA motifs (nt)
0
200
400
600
800
1000
1200
1400
1600
-50000
-45000
-40000
-35000
-30000
-25000
-20000
-15000
-10000
-5000
0

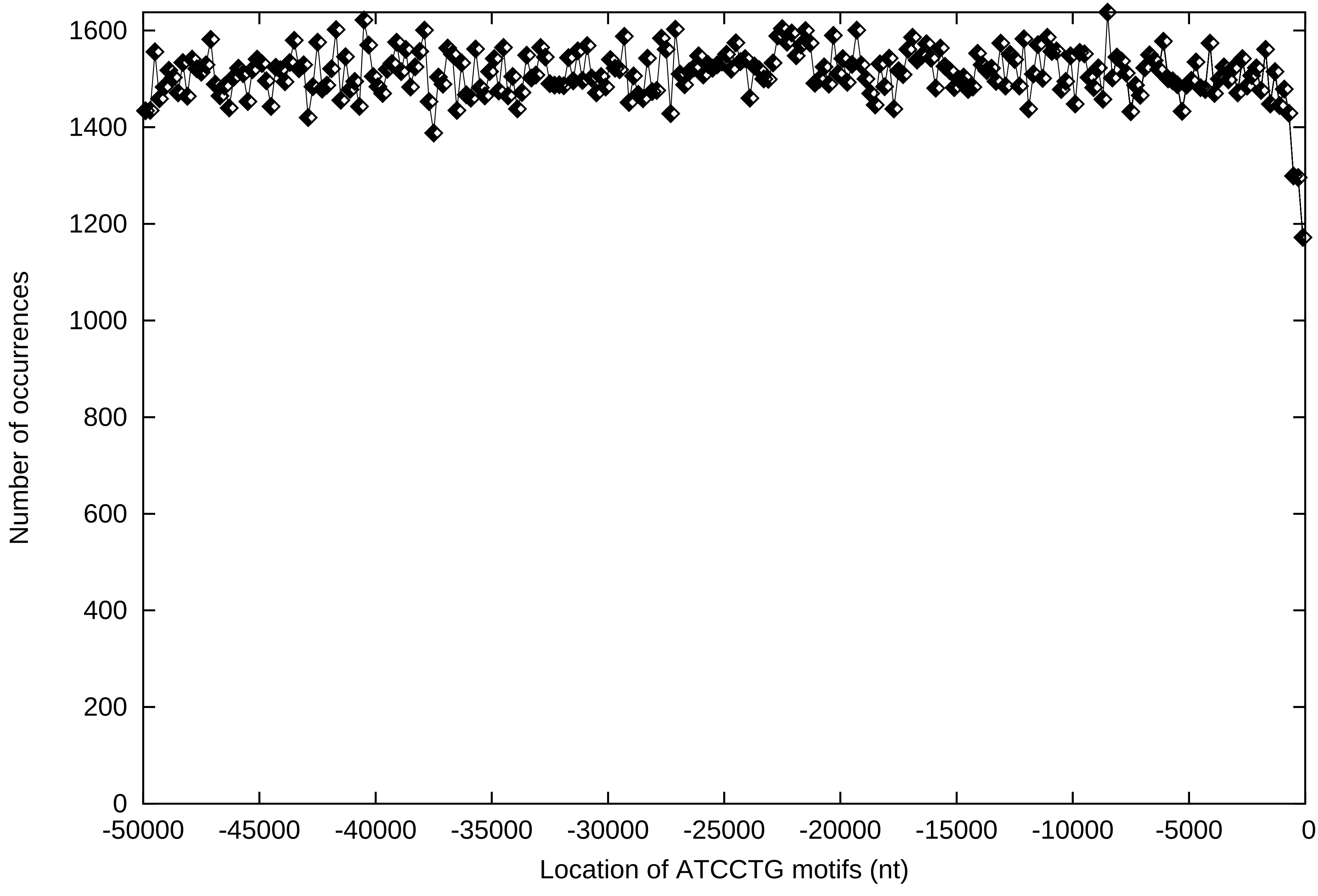

Number of occurrences
1600
1400
1200
1000
800
600
400
200
0
-50000
-45000
-40000
-35000
-30000
-25000
-20000
-15000
-10000
-5000
0
Location of ATCCTG motifs (nt)

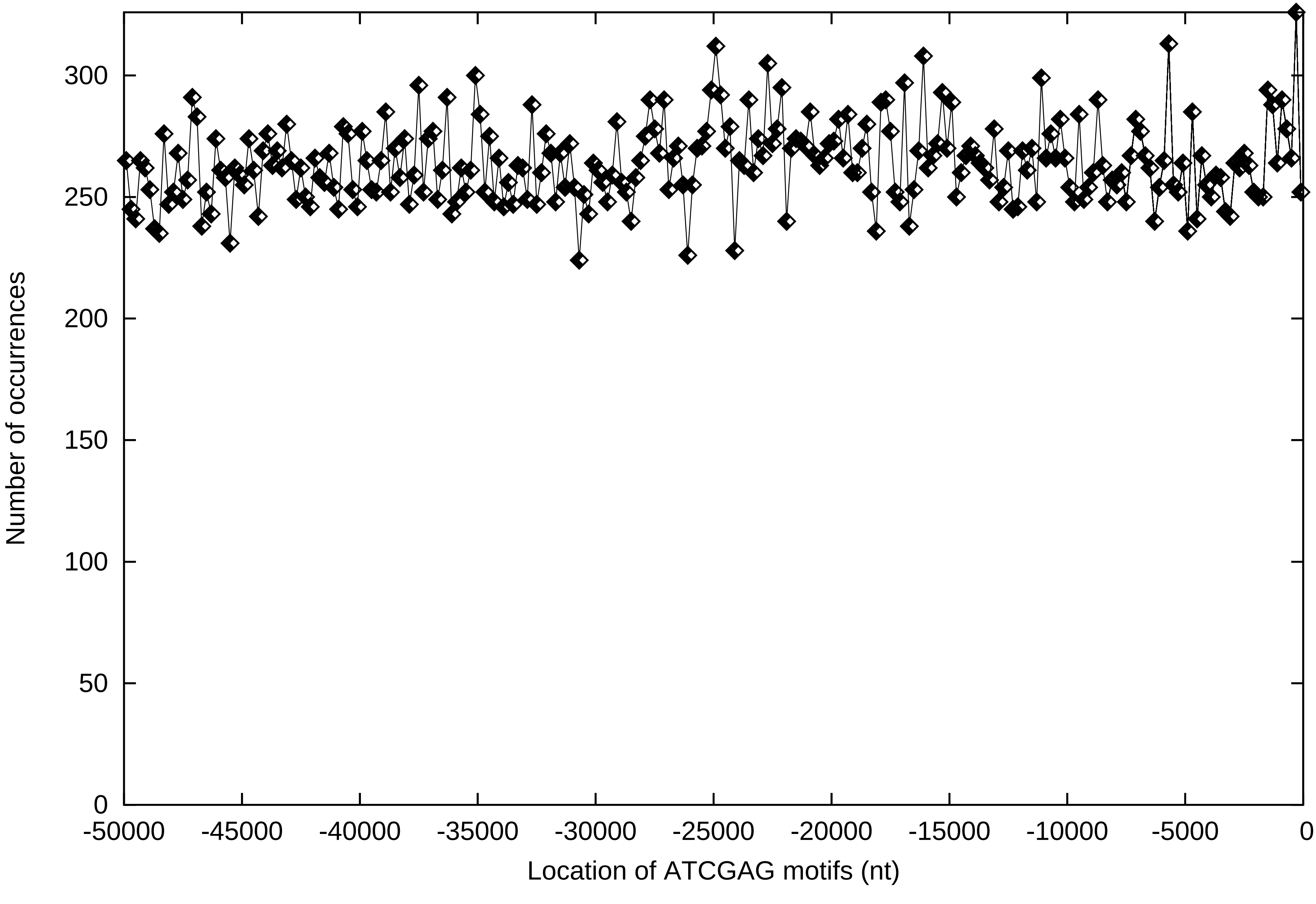

Number of occurrences
0
50
100
150
200
250
300
-50000
-45000
-40000
-35000
-30000
-25000
-20000
-15000
-10000
-5000
0
Location of ATCGAG motifs (nt)

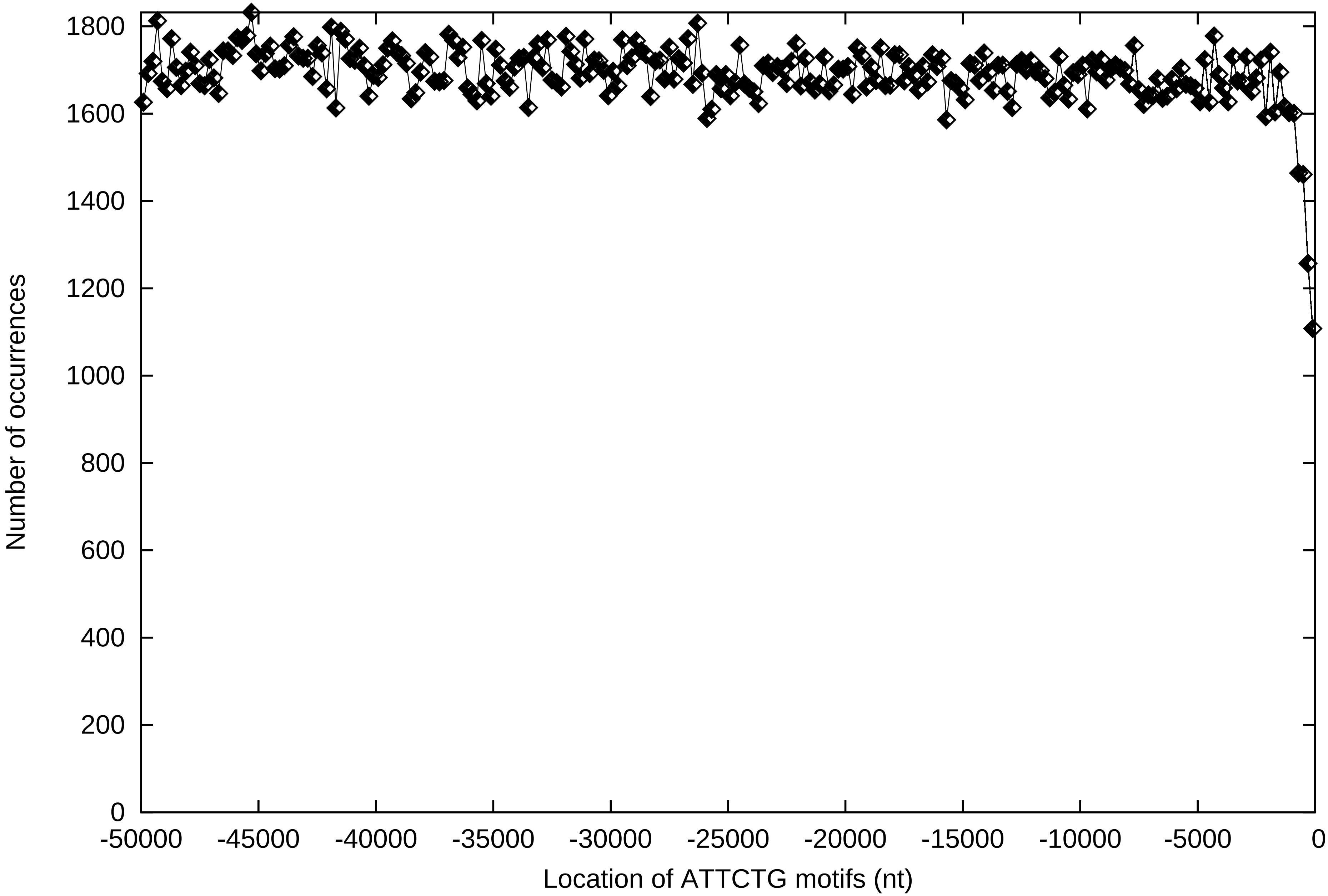
Number of occurrences
Location of ATTCTG motifs (nt)
0
200
400
600
800
1000
1200
1400
1600
1800
-50000
-45000
-40000
-35000
-30000
-25000
-20000
-15000
-10000
-5000
0

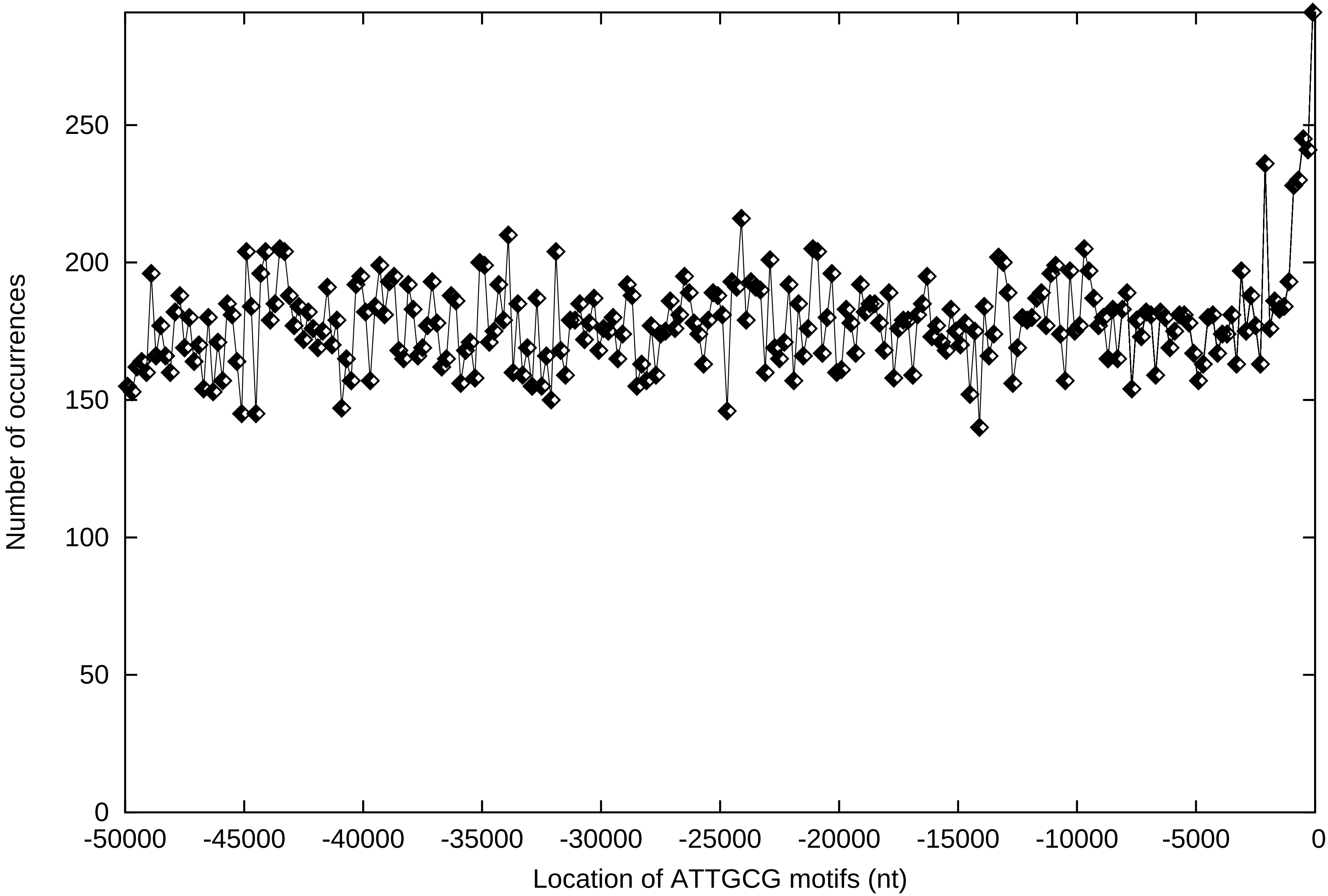

Number of occurrences
0
50
100
150
200
250
-50000
-45000
-40000
-35000
-30000
-25000
-20000
-15000
-10000
-5000
0
Location of ATTGCG motifs (nt)

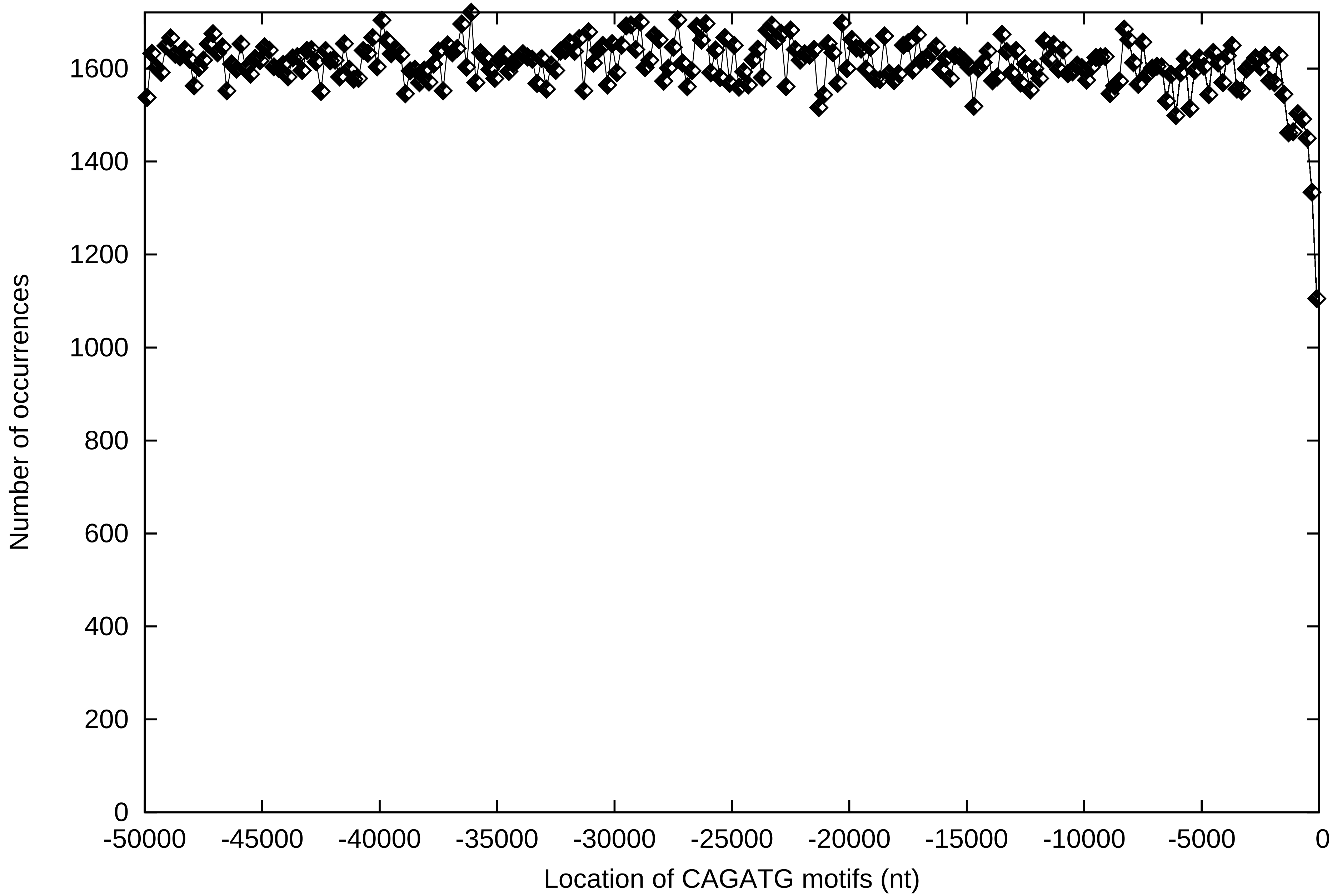

Number of occurrences
0
200
400
600
800
1000
1200
1400
1600
-50000
-45000
-40000
-35000
-30000
-25000
-20000
-15000
-10000
-5000
0
Location of CAGATG motifs (nt)

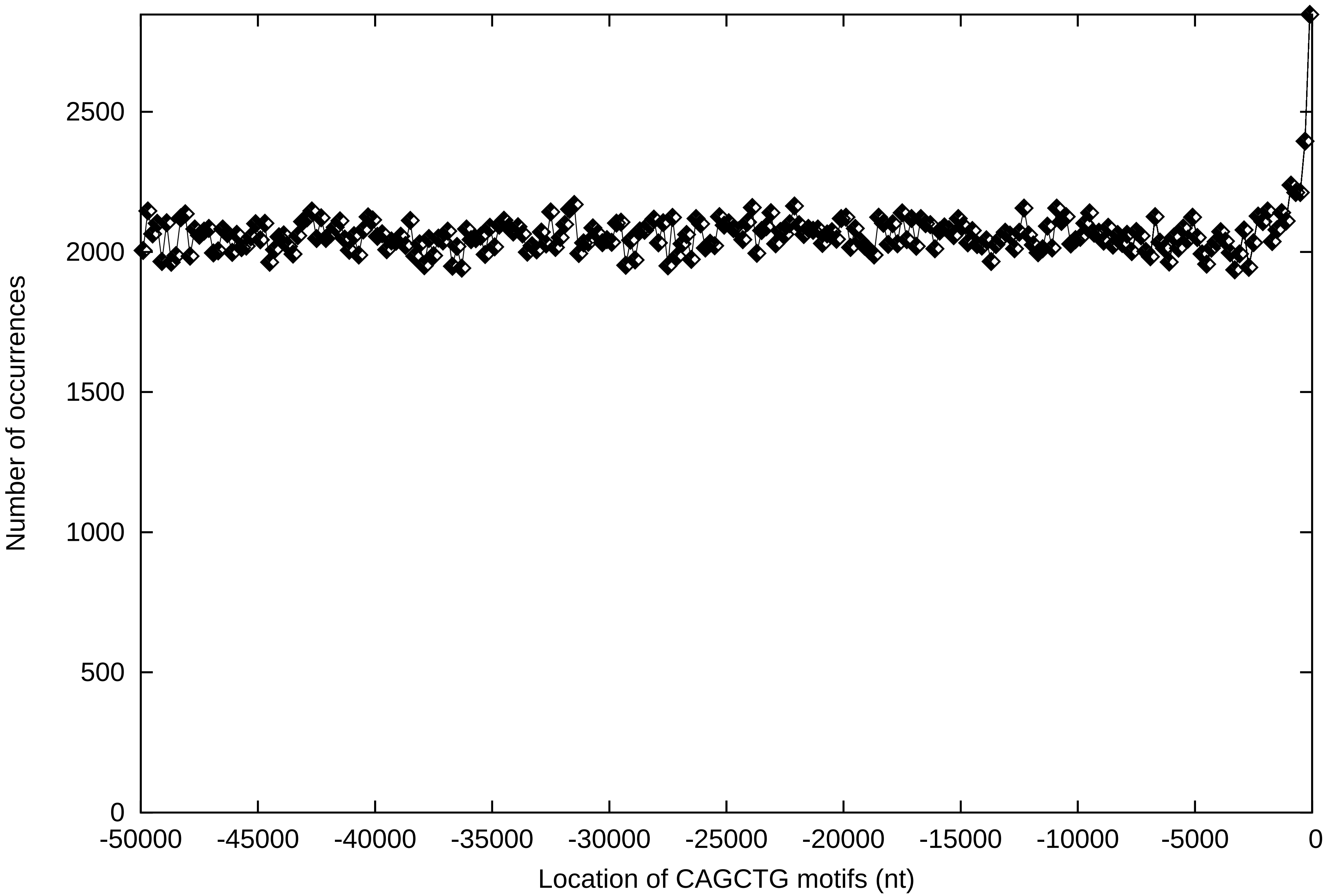

Number of occurrences
0
500
1000
1500
2000
2500
-50000
-45000
-40000
-35000
-30000
-25000
-20000
-15000
-10000
-5000
0
Location of CAGCTG motifs (nt)

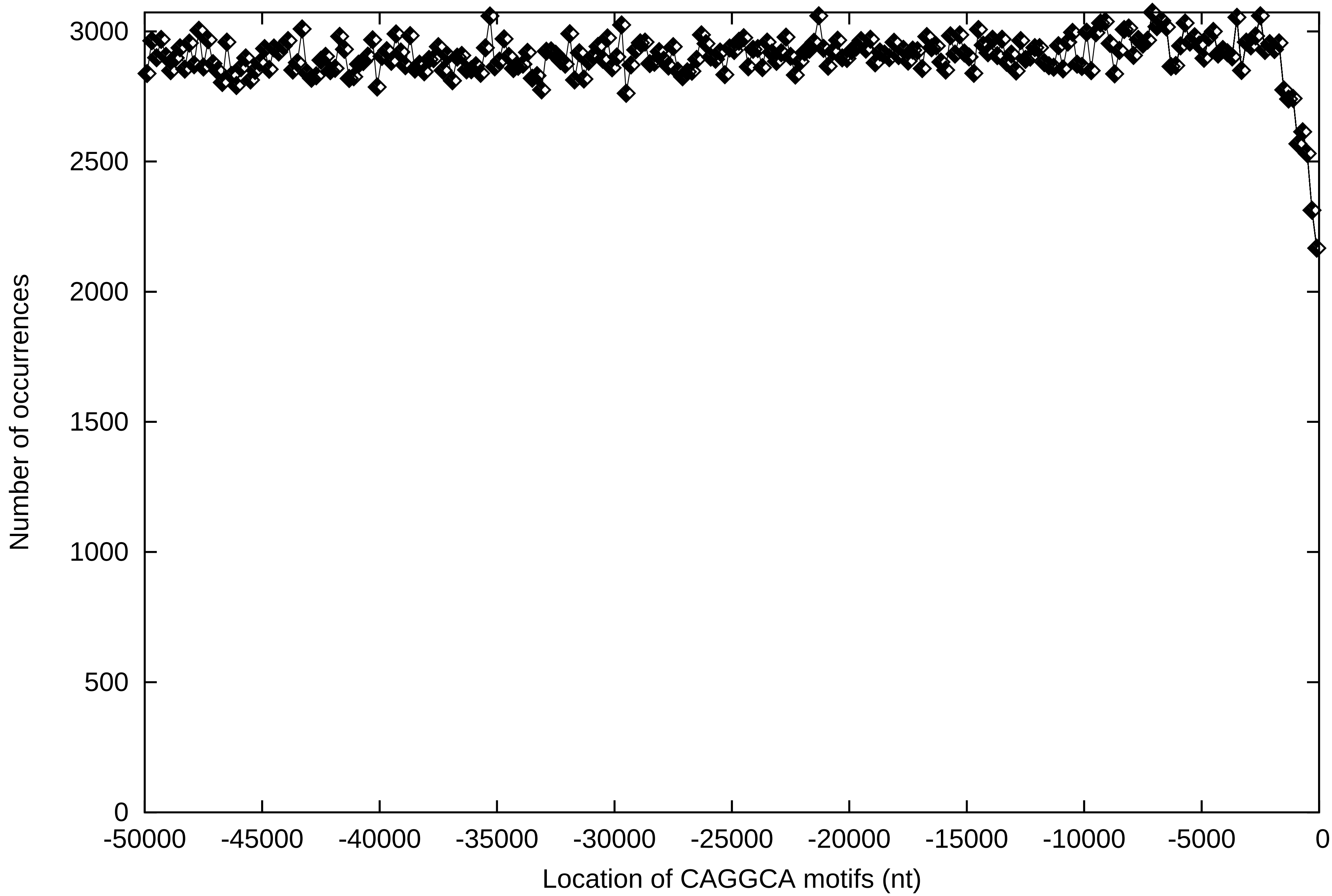

Number of occurrences
3000
2500
2000
1500
1000
500
0
-50000
-45000
-40000
-35000
-30000
-25000
-20000
-15000
-10000
-5000
0
Location of CAGGCA motifs (nt)

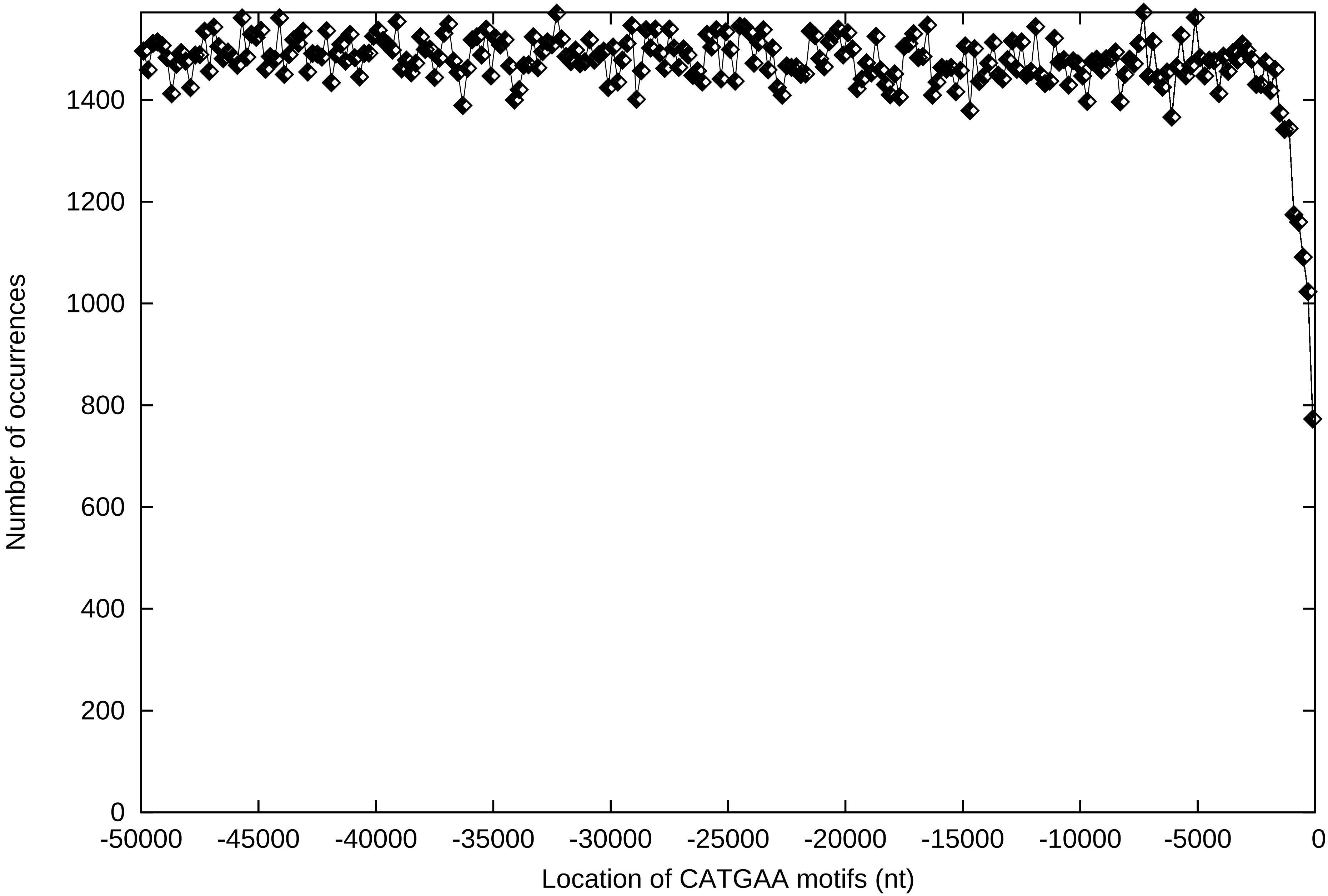

Number of occurrences
0
200
400
600
800
1000
1200
1400
-50000
-45000
-40000
-35000
-30000
-25000
-20000
-15000
-10000
-5000
0
Location of CATGAA motifs (nt)

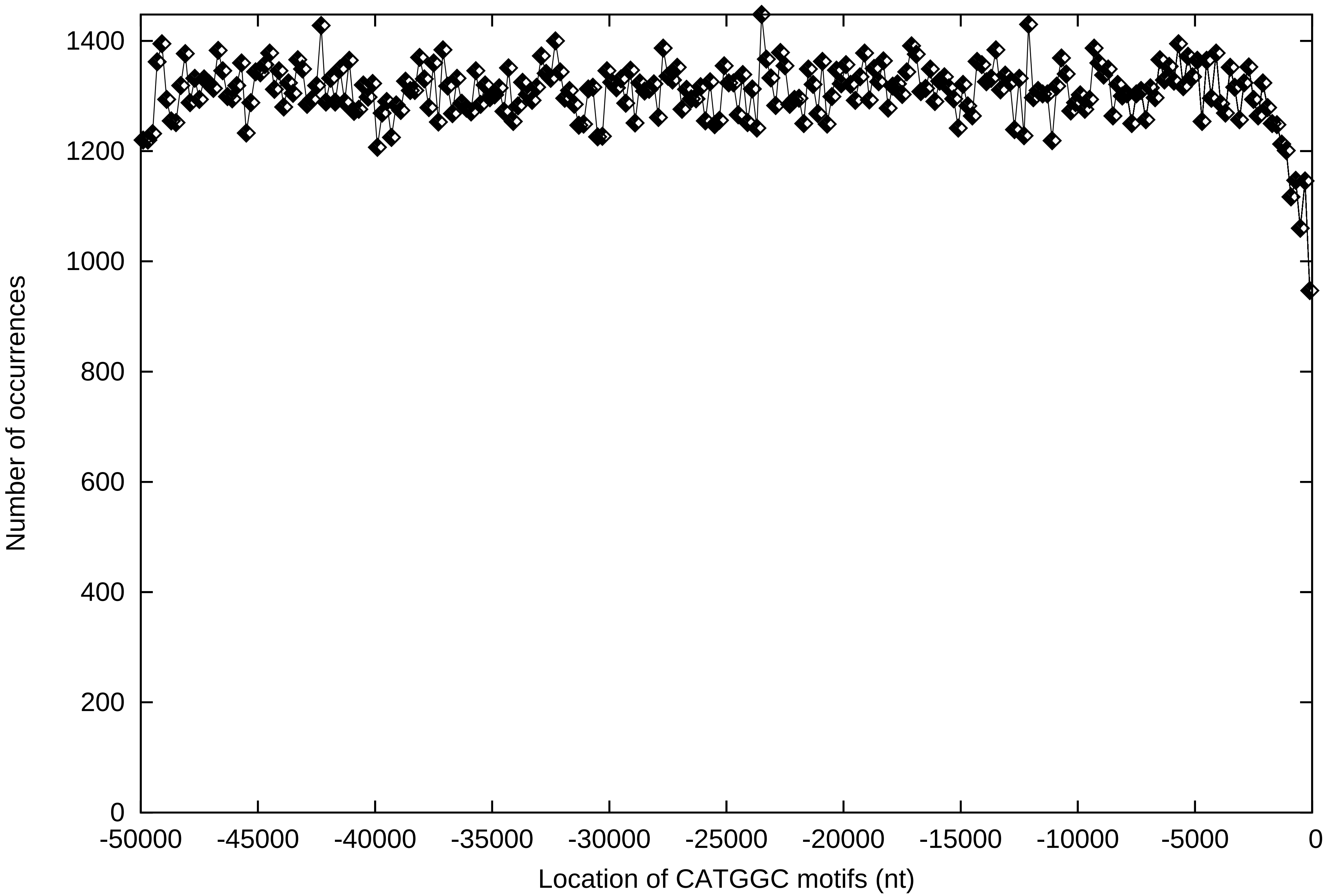
Number of occurrences
1400
1200
1000
800
600
400
200
0
-50000
-45000
-40000
-35000
-30000
-25000
-20000
-15000
-10000
-5000
0
Location of CATGGC motifs (nt)

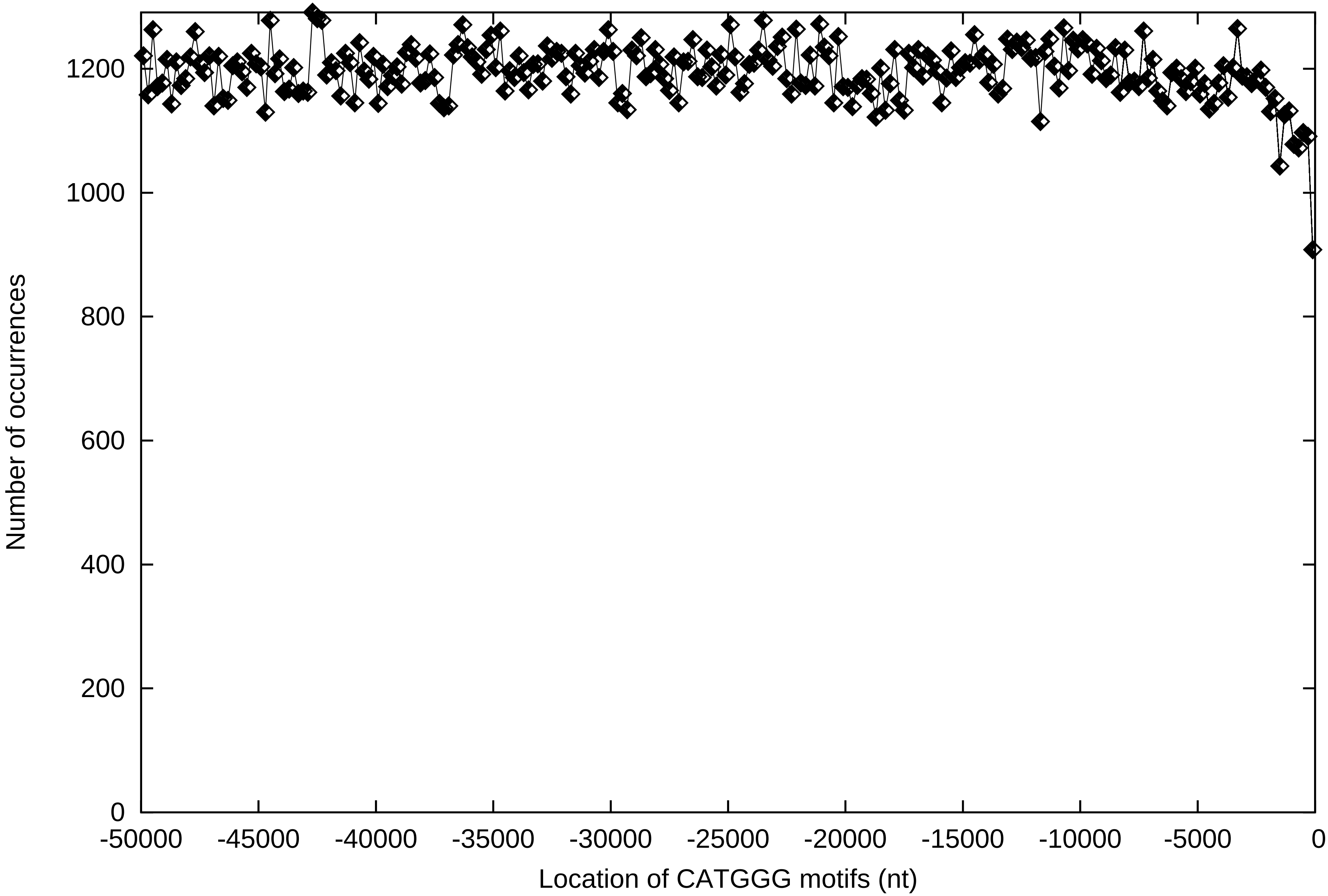

Number of occurrences
1200
1000
800
600
400
200
0
-50000
-45000
-40000
-35000
-30000
-25000
-20000
-15000
-10000
-5000
0
Location of CATGGG motifs (nt)

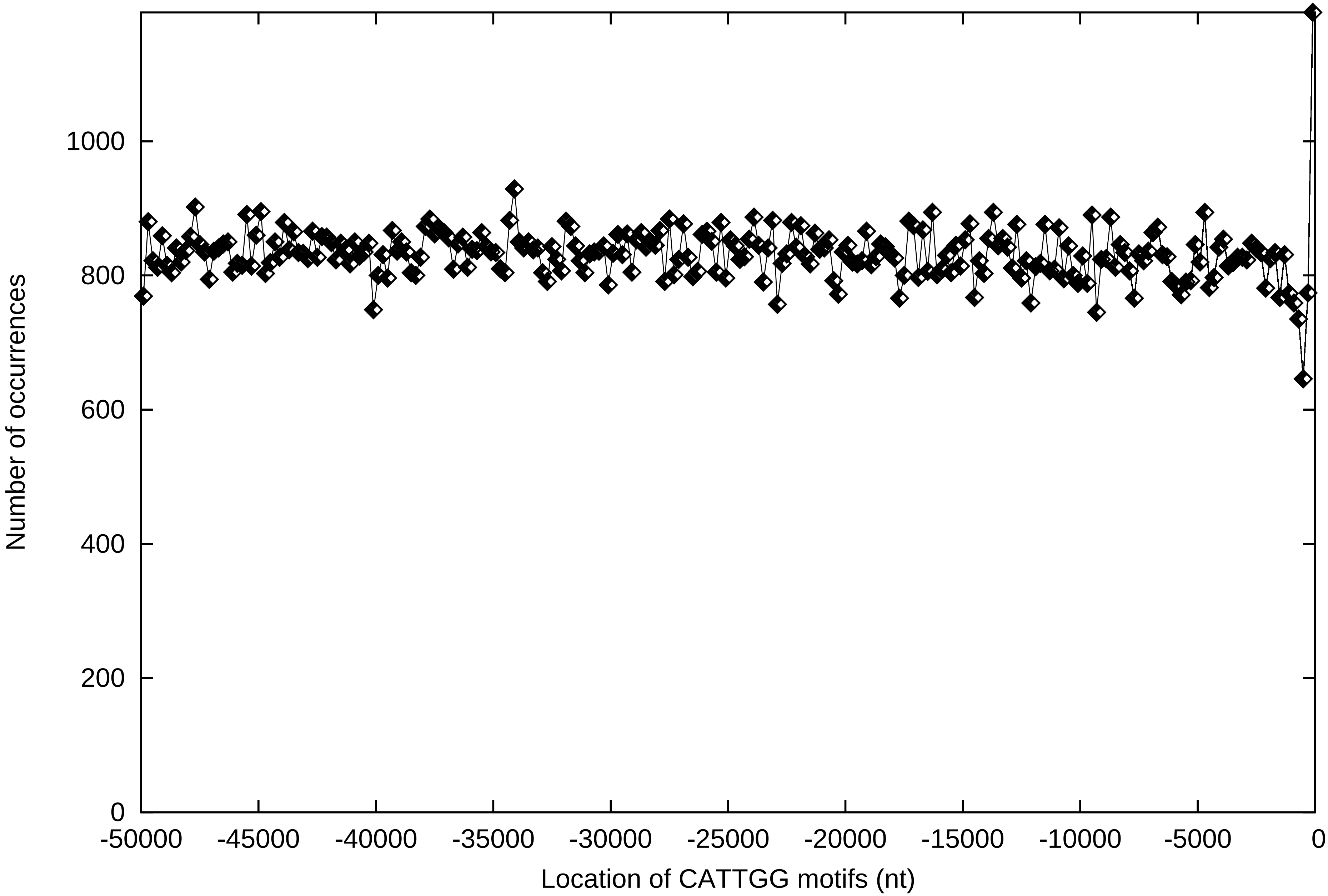

Number of occurrences
0
200
400
600
800
1000
-50000
-45000
-40000
-35000
-30000
-25000
-20000
-15000
-10000
-5000
0
Location of CATTGG motifs (nt)

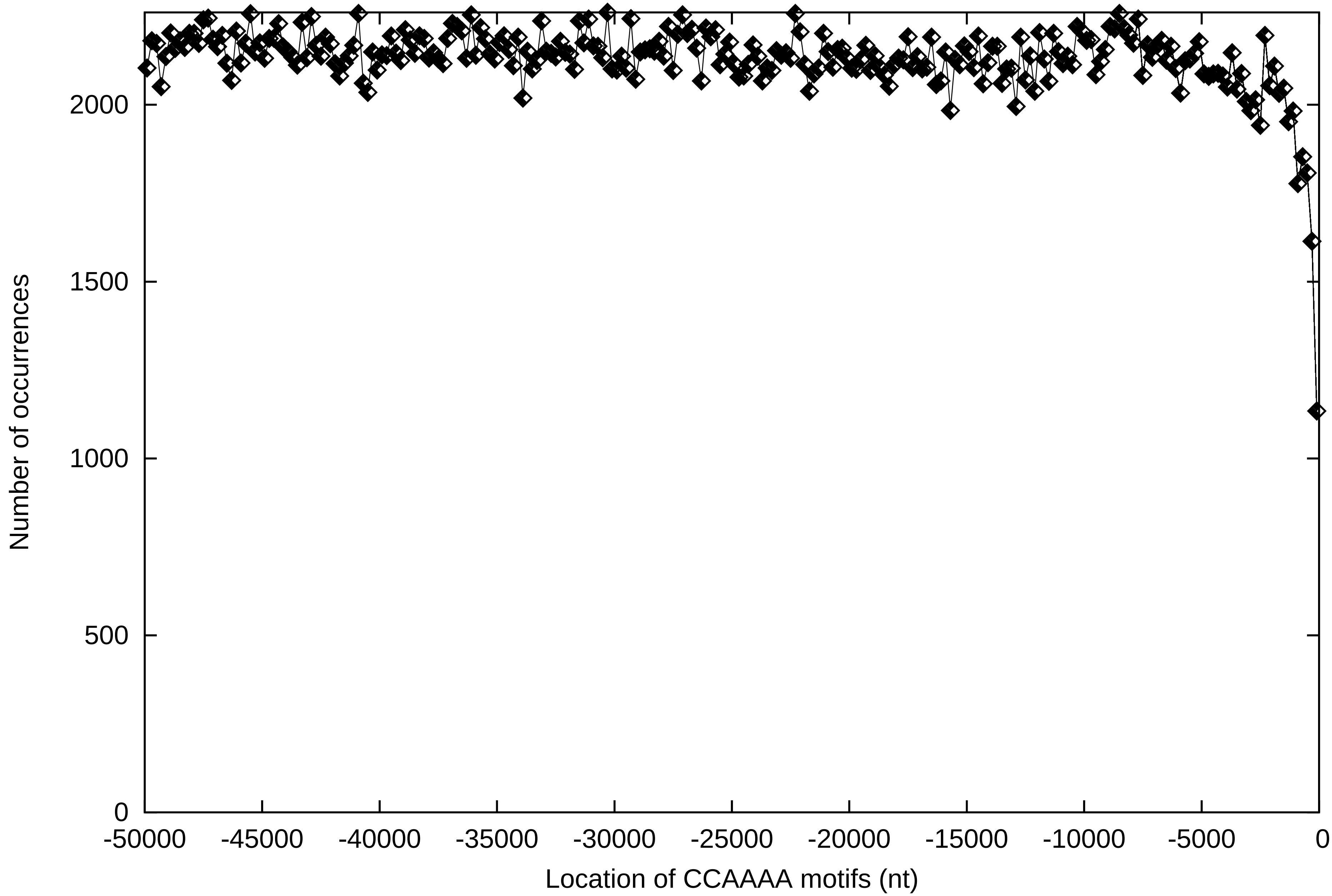

Number of occurrences
0
500
1000
1500
2000
-50000
-45000
-40000
-35000
-30000
-25000
-20000
-15000
-10000
-5000
0
Location of CCAAAA motifs (nt)

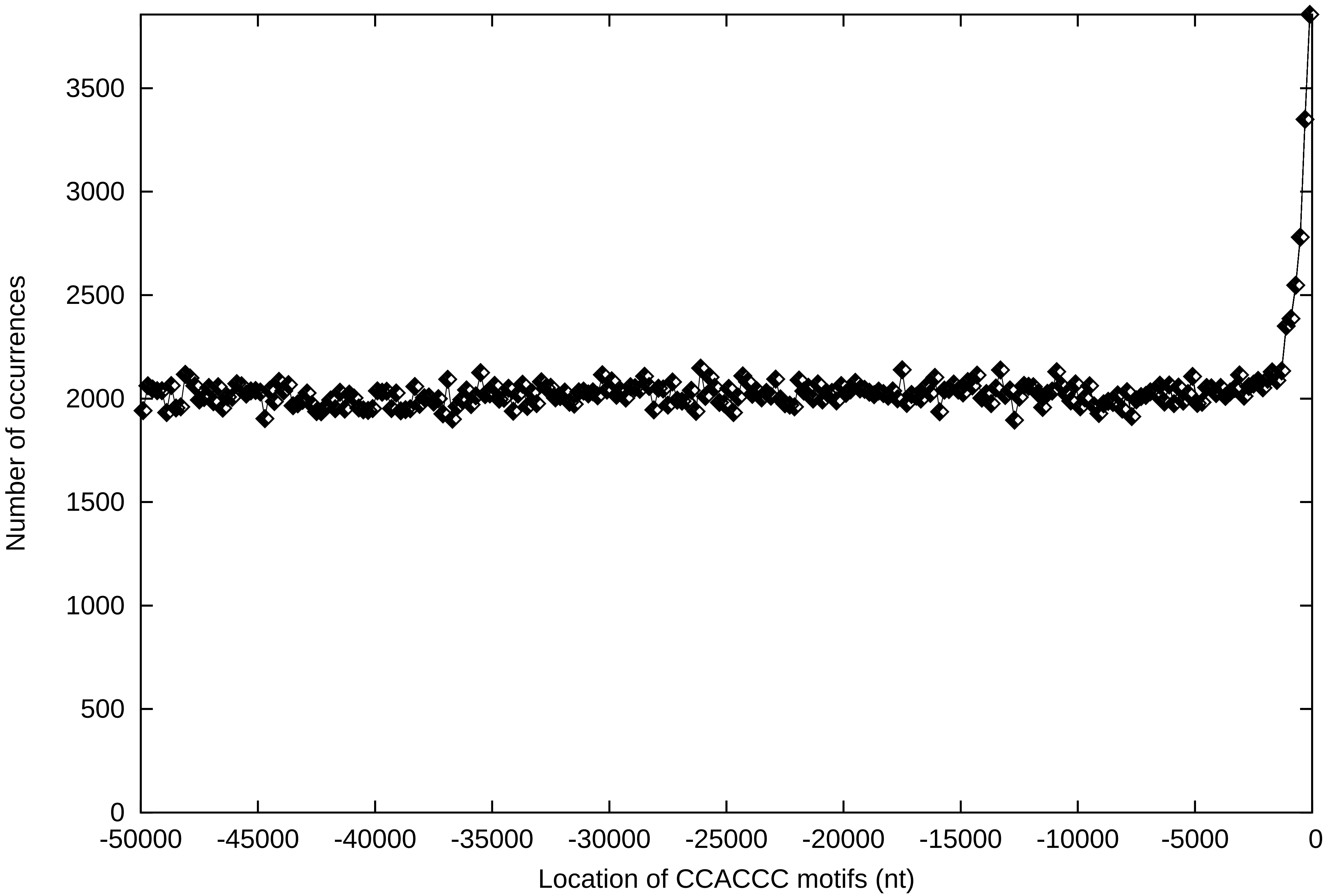

Number of occurrences
0
500
1000
1500
2000
2500
3000
3500
-50000
-45000
-40000
-35000
-30000
-25000
-20000
-15000
-10000
-5000
0
Location of CCACCC motifs (nt)

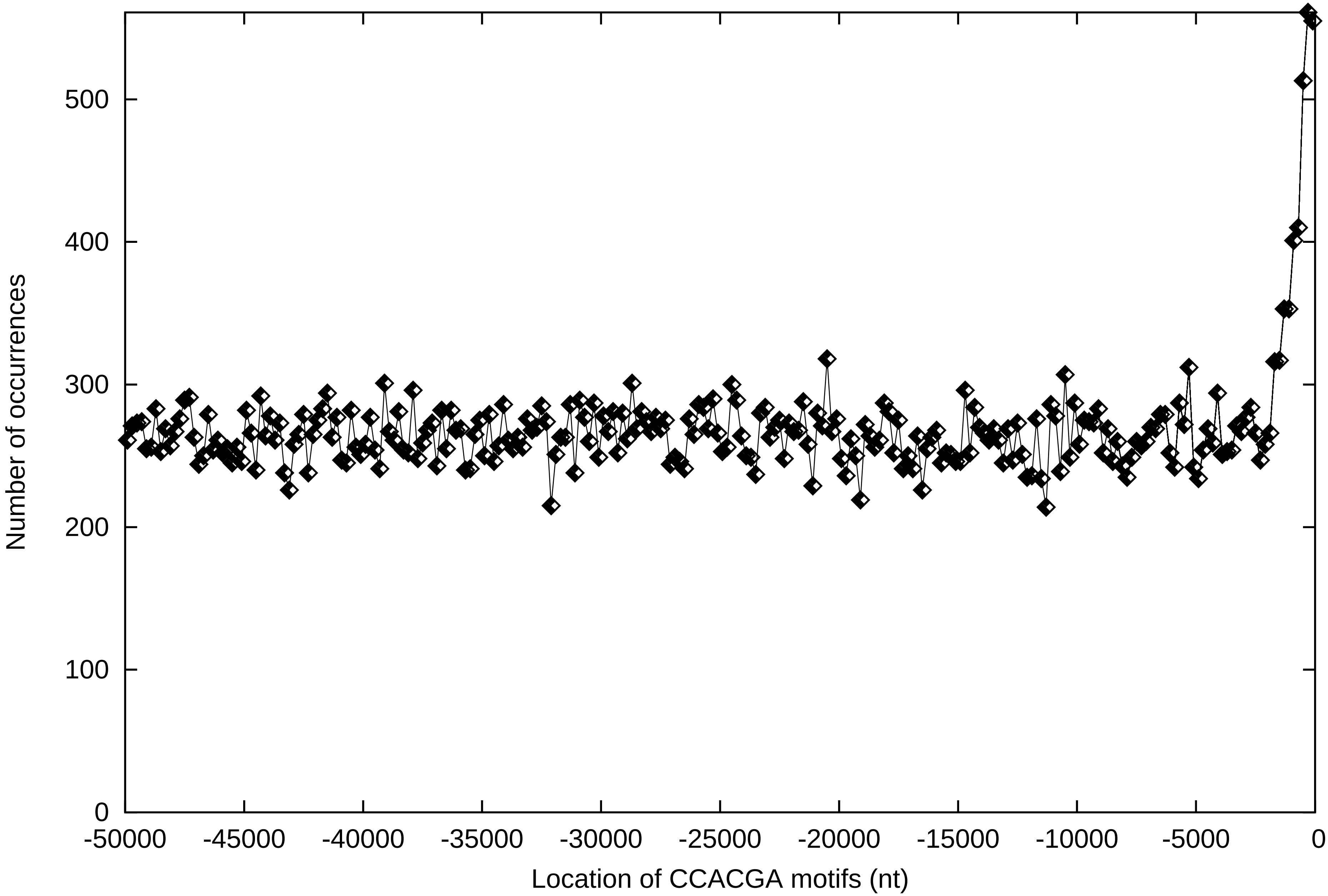

Number of occurrences
Location of CCACGA motifs (nt)
0
100
200
300
400
500
-50000
-45000
-40000
-35000
-30000
-25000
-20000
-15000
-10000
-5000
0

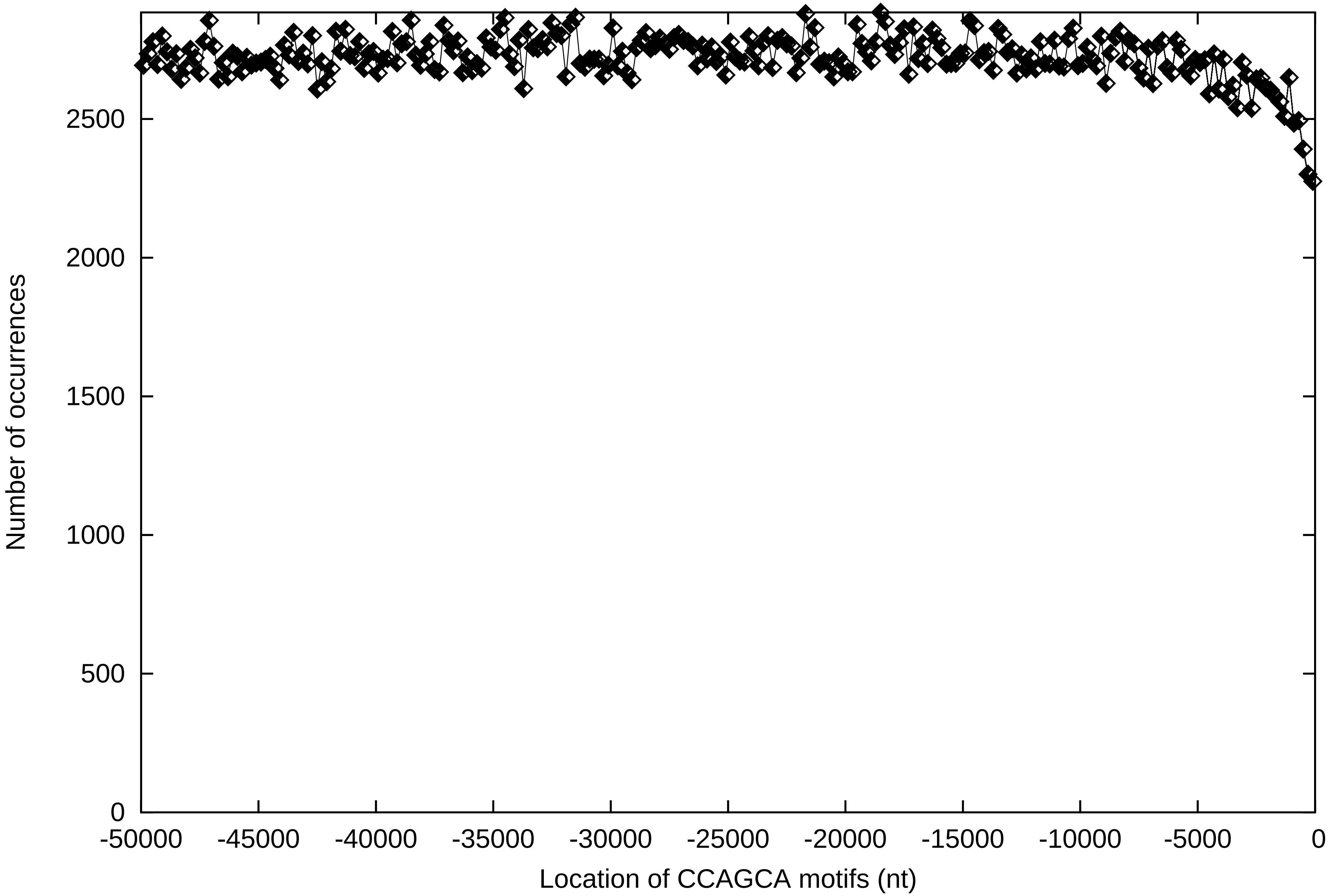

Number of occurrences
0
500
1000
1500
2000
2500
-50000
-45000
-40000
-35000
-30000
-25000
-20000
-15000
-10000
-5000
0
Location of CCAGCA motifs (nt)

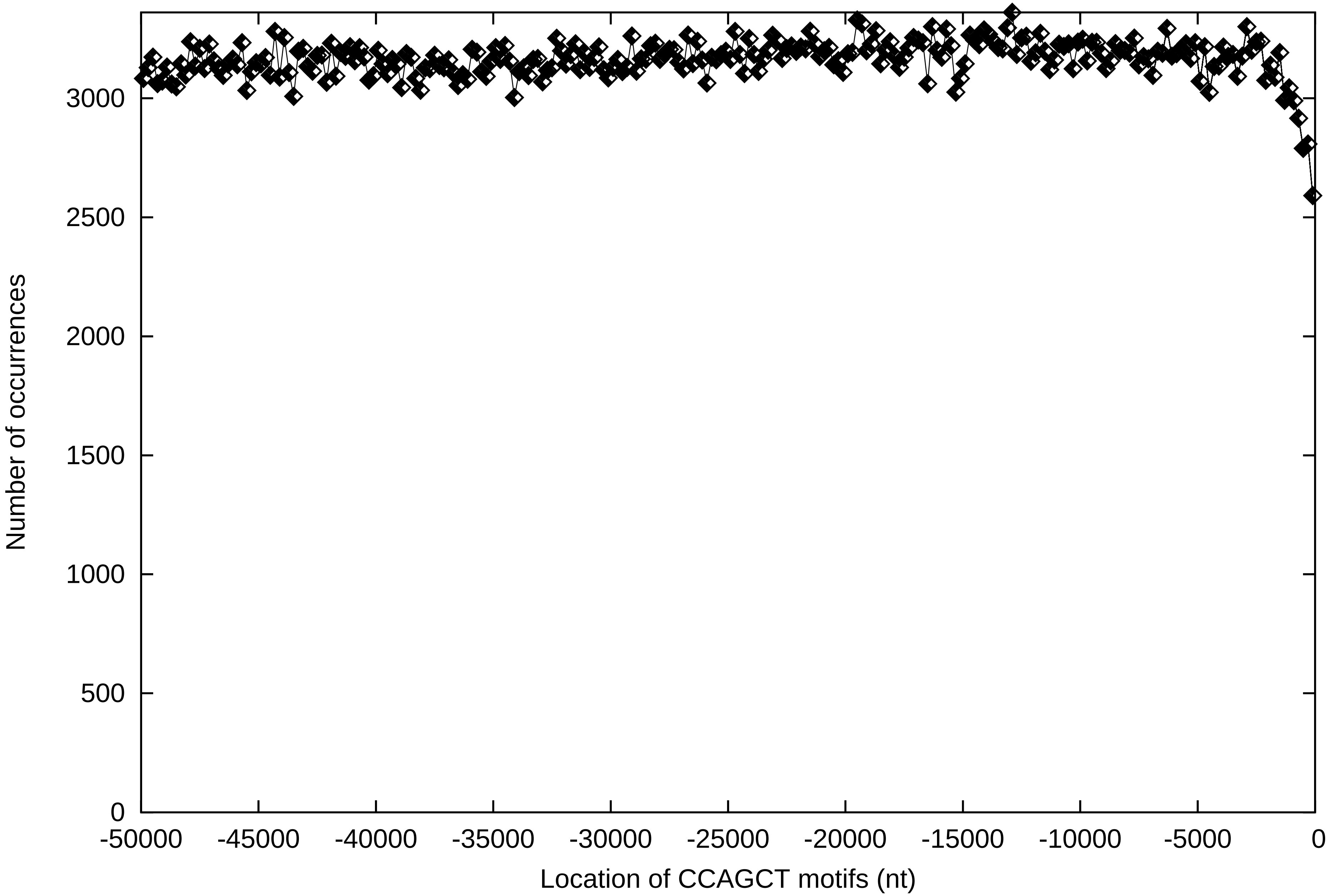

Number of occurrences
0
500
1000
1500
2000
2500
3000
-50000
-45000
-40000
-35000
-30000
-25000
-20000
-15000
-10000
-5000
0
Location of CCAGCT motifs (nt)

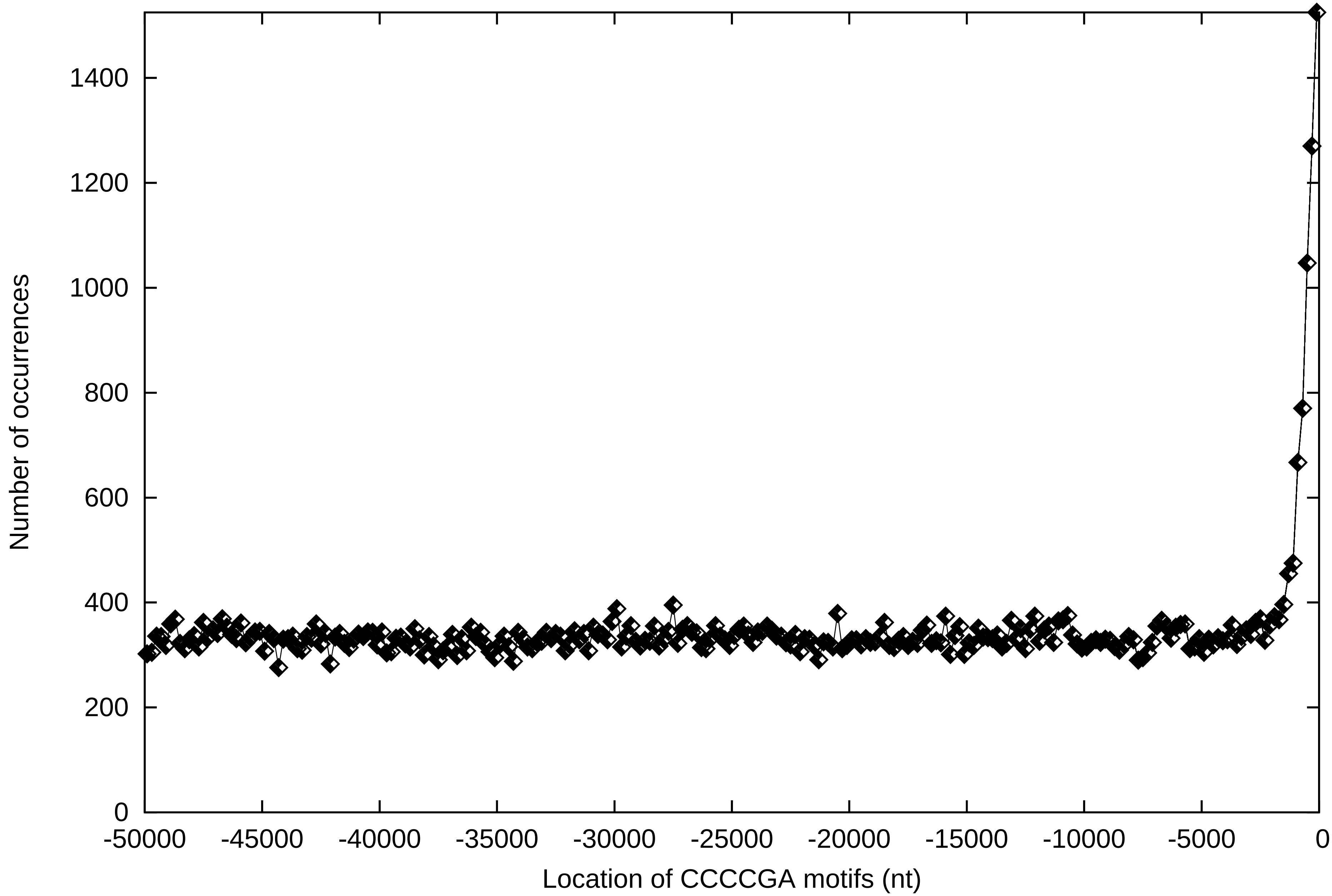
Number of occurrences
0
200
400
600
800
1000
1200
1400
-50000
-45000
-40000
-35000
-30000
-25000
-20000
-15000
-10000
-5000
0
Location of CCCCGA motifs (nt)

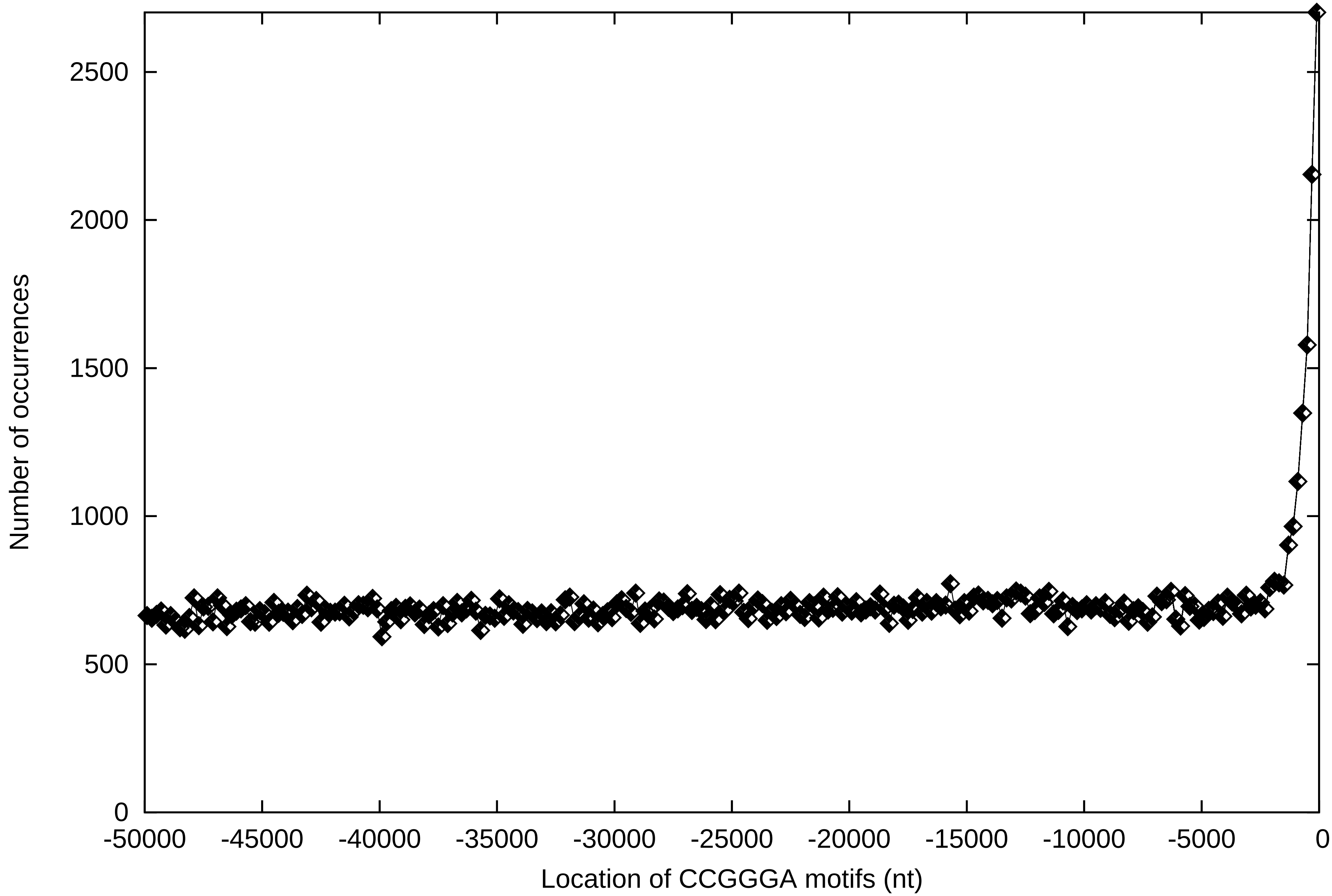

Number of occurrences
2500
2000
1500
1000
500
0
-50000
-45000
-40000
-35000
-30000
-25000
-20000
-15000
-10000
-5000
0
Location of CCGGGA motifs (nt)

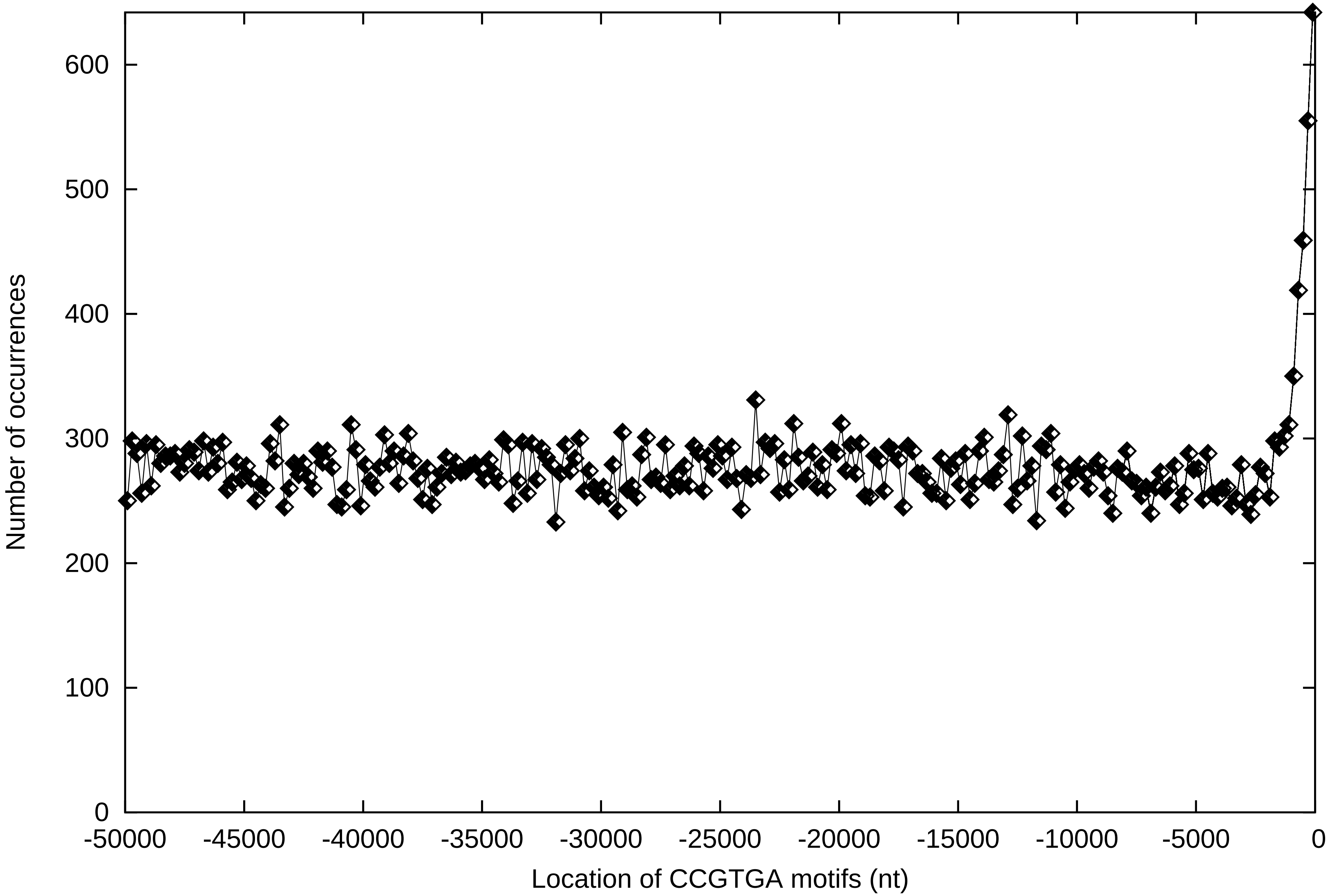
Number of occurrences
0
100
200
300
400
500
600
-50000
-45000
-40000
-35000
-30000
-25000
-20000
-15000
-10000
-5000
0
Location of CCGTGA motifs (nt)

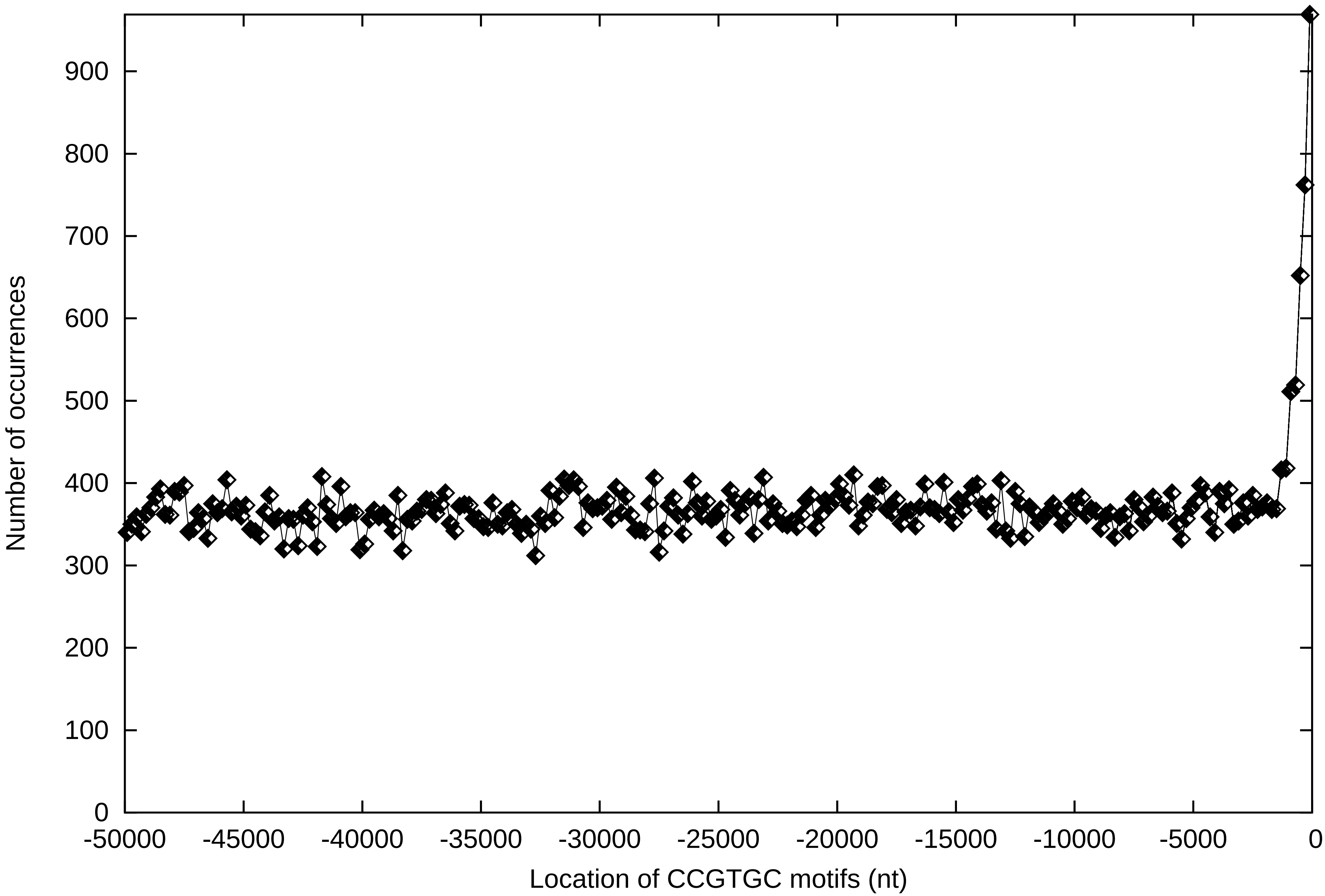
Number of occurrences
0
100
200
300
400
500
600
700
800
900
-50000
-45000
-40000
-35000
-30000
-25000
-20000
-15000
-10000
-5000
0
Location of CCGTGC motifs (nt)

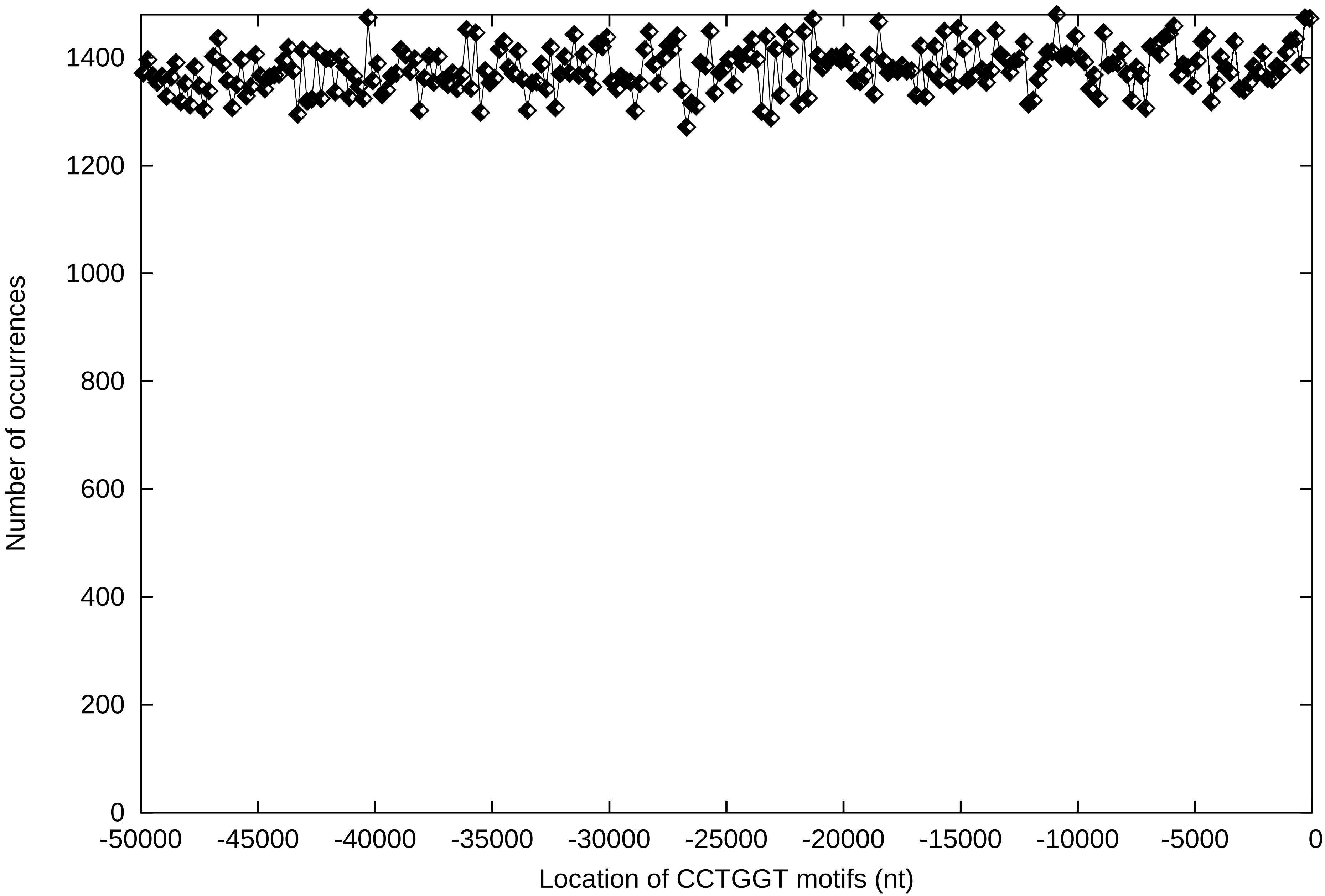

Number of occurrences
0
200
400
600
800
1000
1200
1400
-50000
-45000
-40000
-35000
-30000
-25000
-20000
-15000
-10000
-5000
0
Location of CCTGGT motifs (nt)

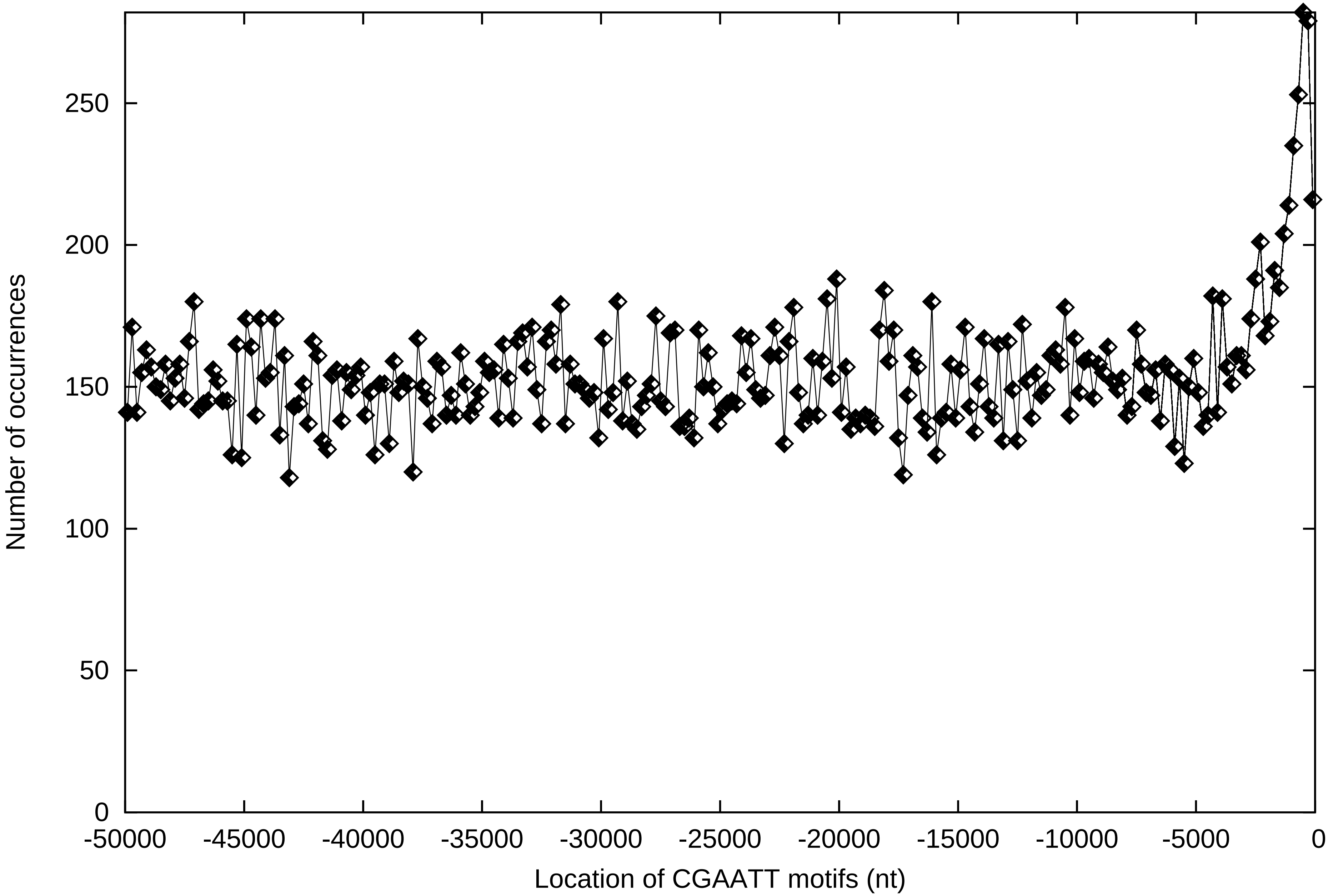

Number of occurrences
0
50
100
150
200
250
-50000
-45000
-40000
-35000
-30000
-25000
-20000
-15000
-10000
-5000
0
Location of CGAATT motifs (nt)

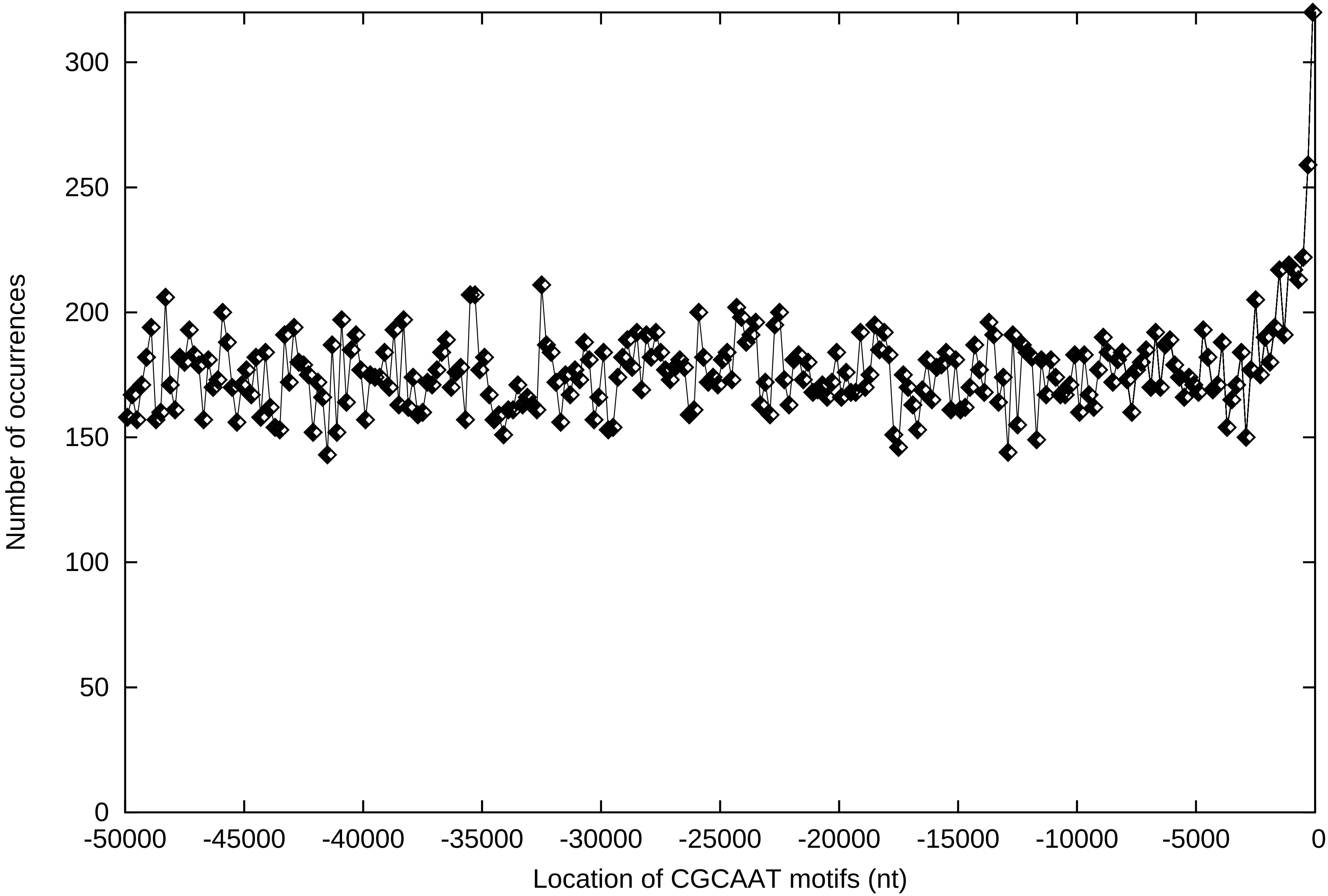

Number of occurrences
300
250
200
150
100
50
0
-50000
-45000
-40000
-35000
-30000
-25000
-20000
-15000
-10000
-5000
0
Location of CGCAAT motifs (nt)

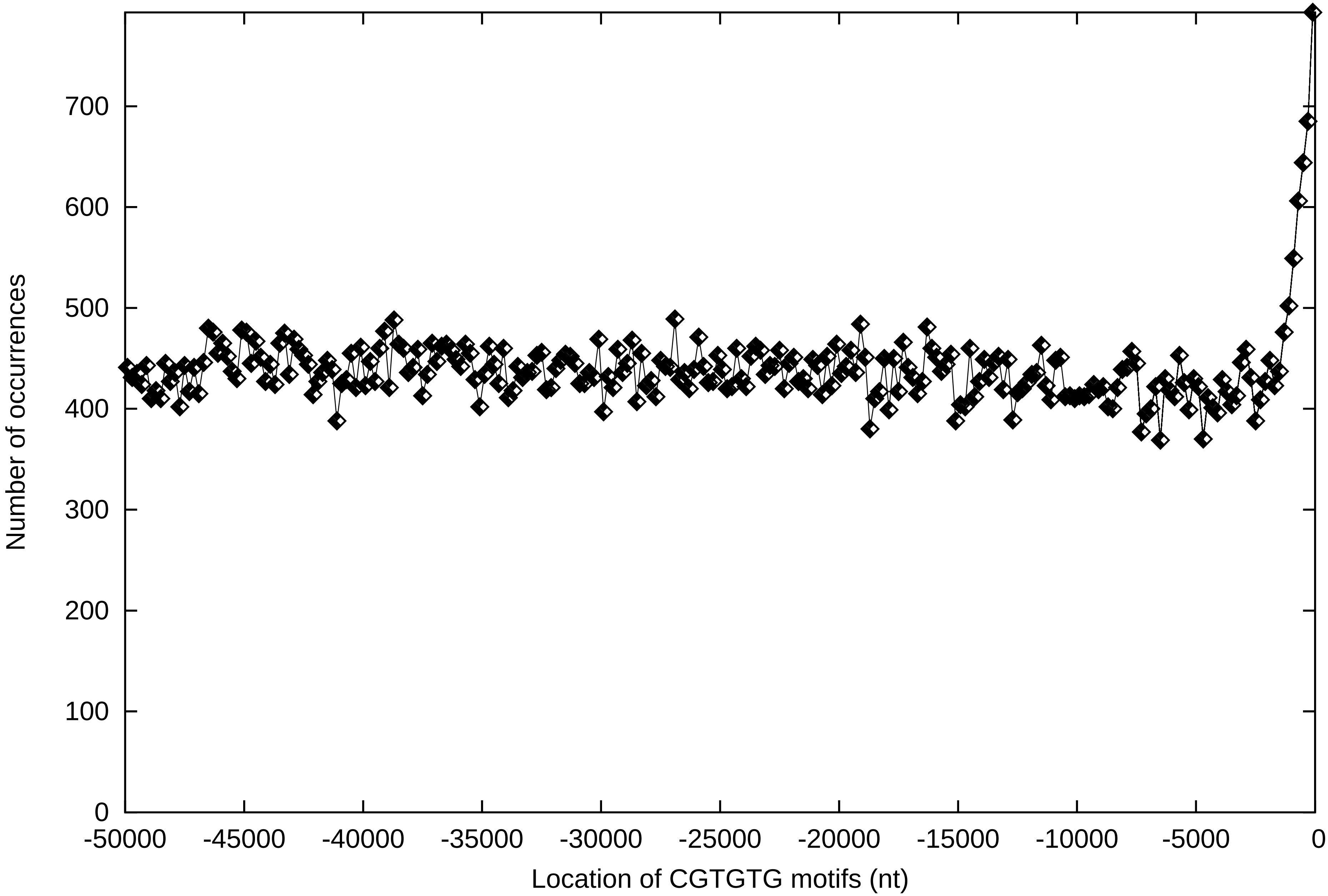
Number of occurrences
0
100
200
300
400
500
600
700
-50000
-45000
-40000
-35000
-30000
-25000
-20000
-15000
-10000
-5000
0
Location of CGTGTG motifs (nt)

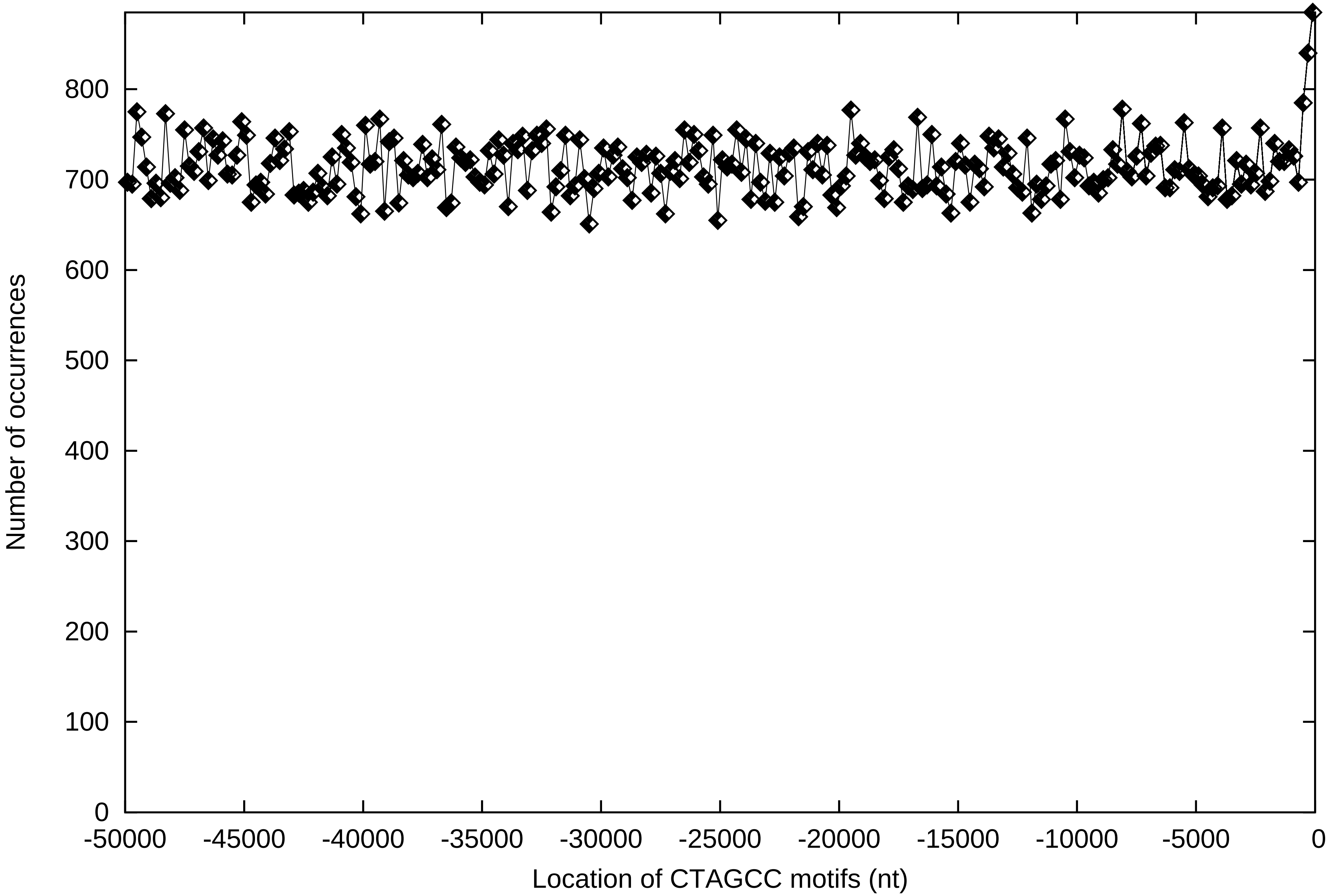
Number of occurrences
0
100
200
300
400
500
600
700
800
-50000
-45000
-40000
-35000
-30000
-25000
-20000
-15000
-10000
-5000
0
Location of CTAGCC motifs (nt)

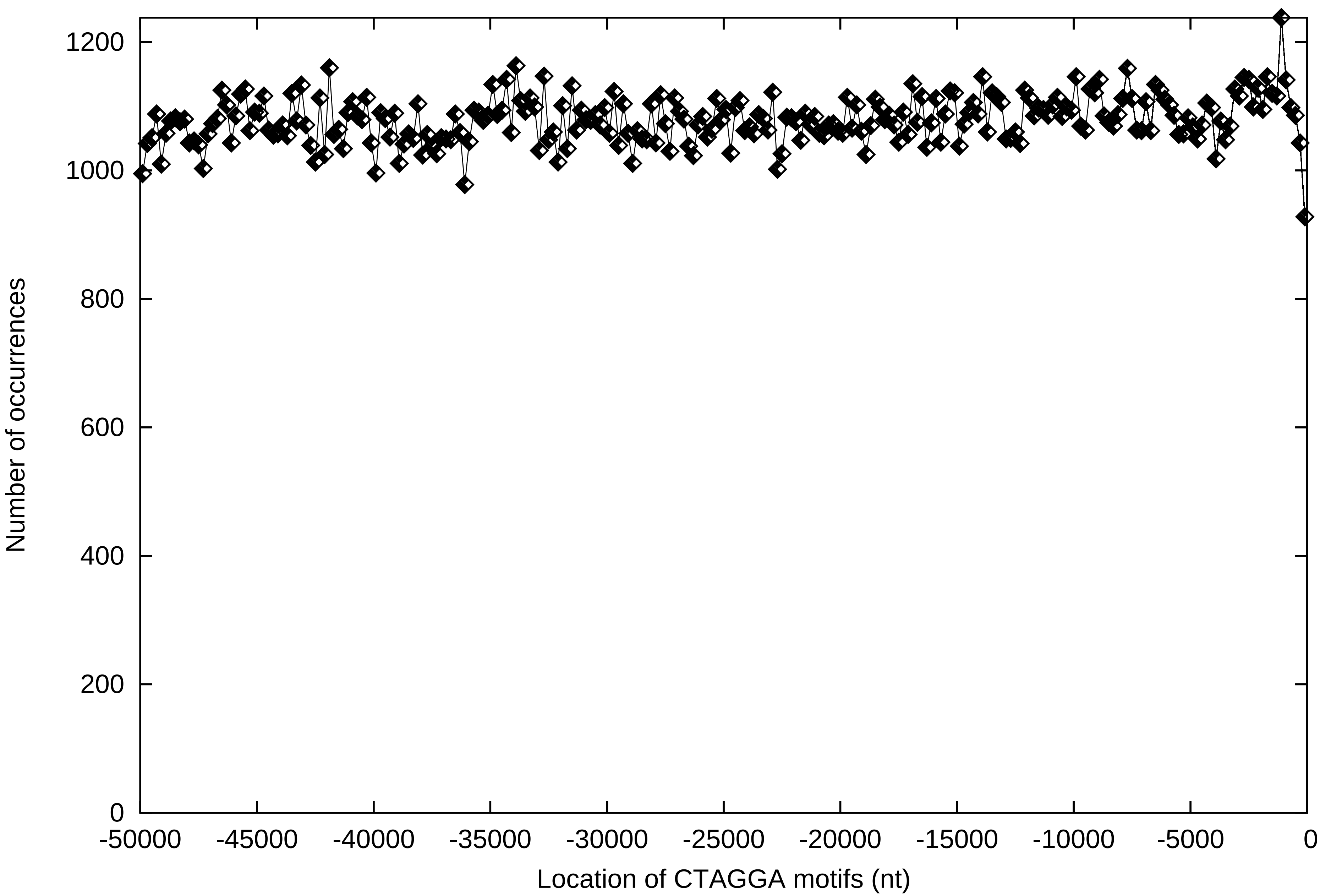

Number of occurrences
Location of CTAGGA motifs (nt)
0
200
400
600
800
1000
1200
-50000
-45000
-40000
-35000
-30000
-25000
-20000
-15000
-10000
-5000
0

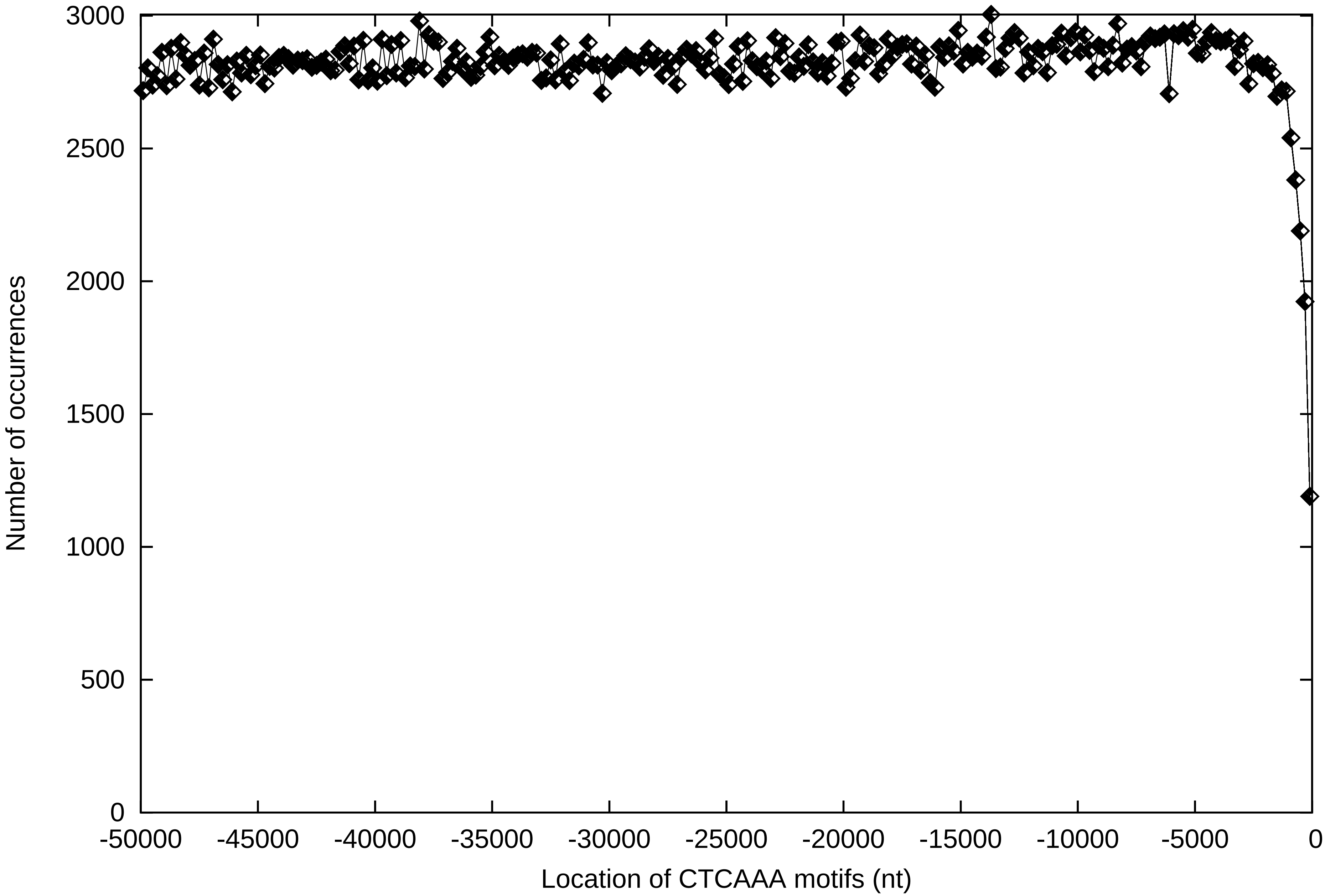

Number of occurrences
3000
2500
2000
1500
1000
500
0
-50000
-45000
-40000
-35000
-30000
-25000
-20000
-15000
-10000
-5000
0
Location of CTCAAA motifs (nt)

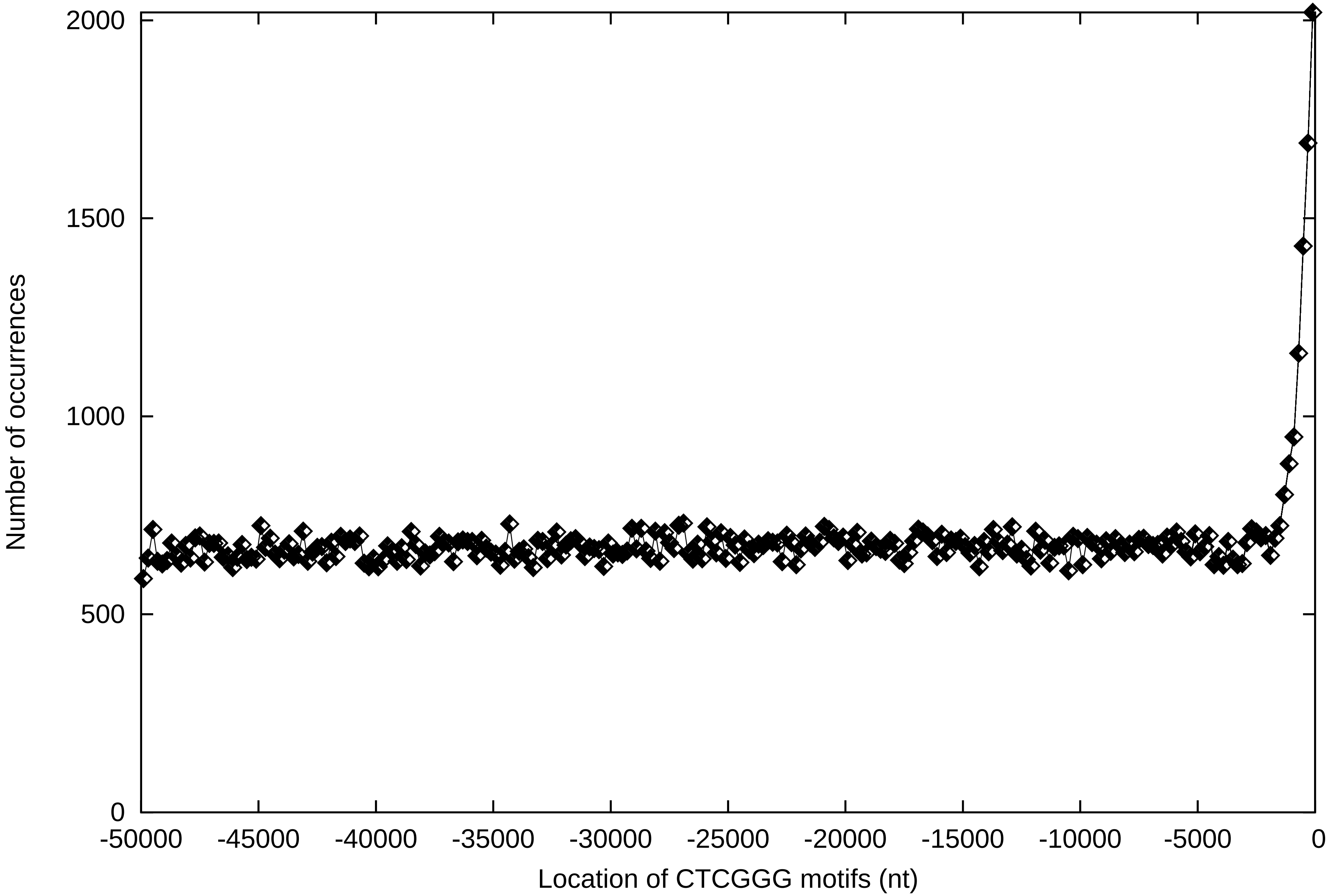

2000
1500
1000
500
0
Number of occurrences
-50000
-45000
-40000
-35000
-30000
-25000
-20000
-15000
-10000
-5000
0
Location of CTCGGG motifs (nt)

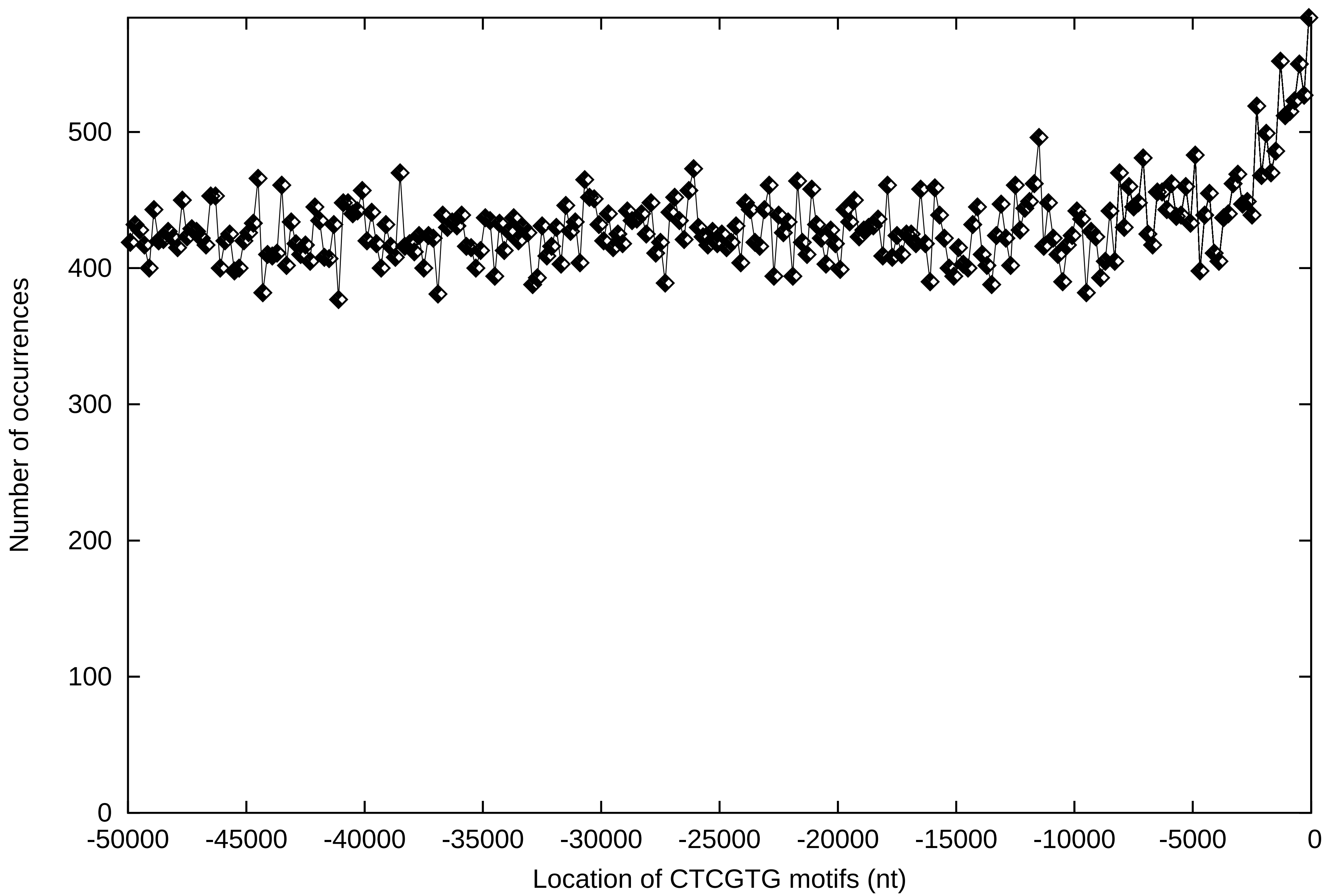

Number of occurrences
0
100
200
300
400
500
-50000
-45000
-40000
-35000
-30000
-25000
-20000
-15000
-10000
-5000
0
Location of CTCGTG motifs (nt)

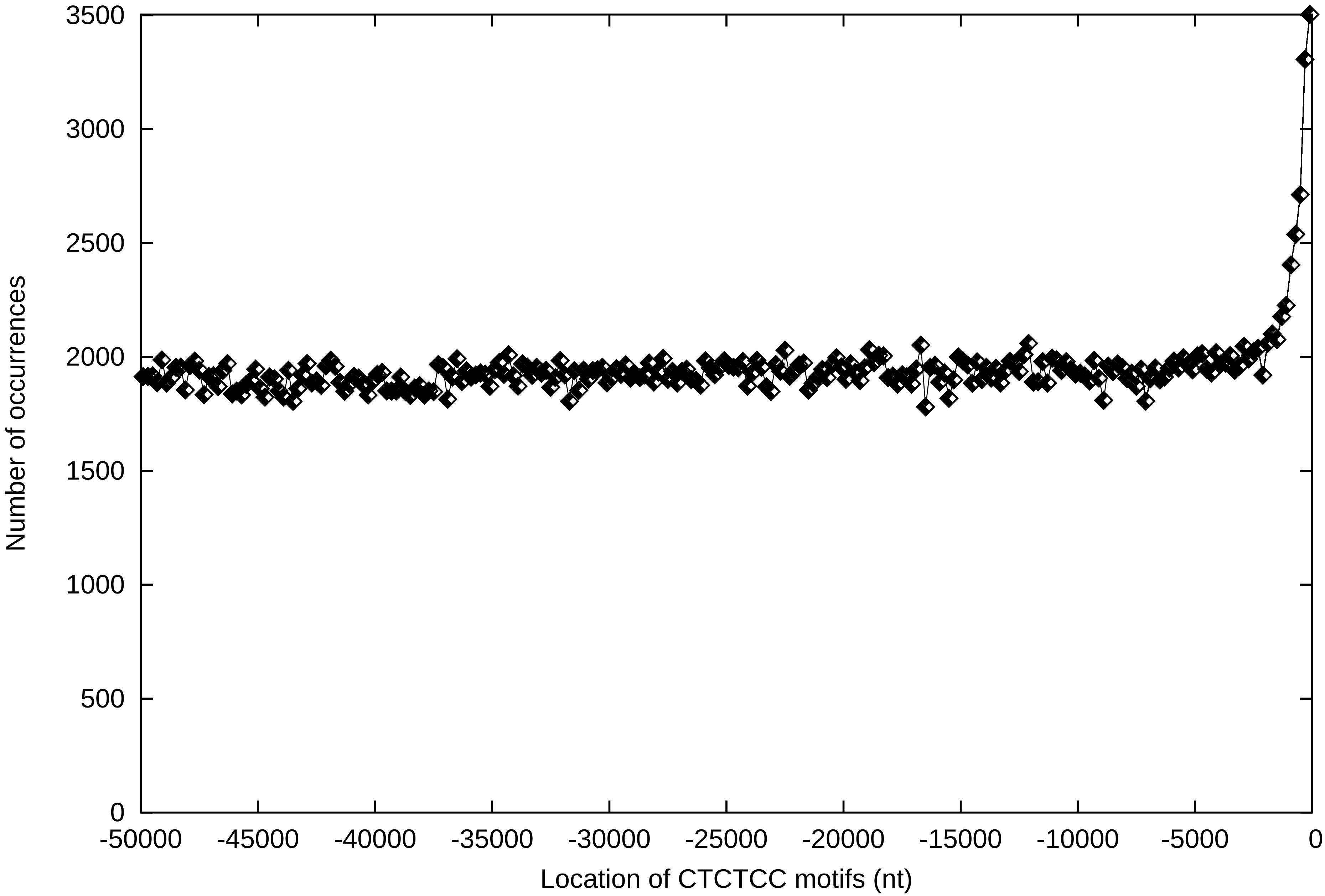

3500
3000
2500
2000
1500
1000
500
0
-50000
-45000
-40000
-35000
-30000
-25000
-20000
-15000
-10000
-5000
0
Number of occurrences
Location of CTCTCC motifs (nt)

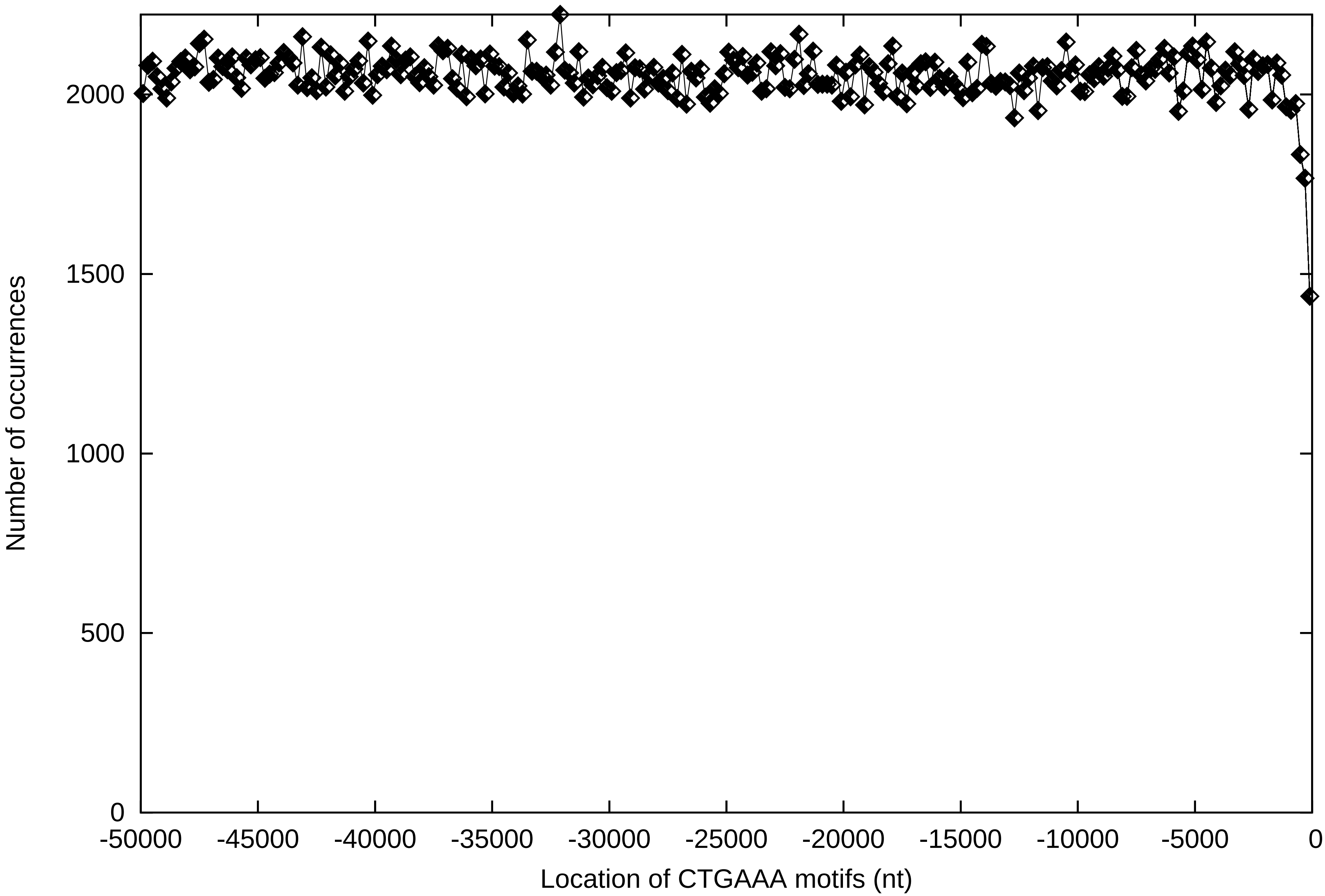

Number of occurrences
2000
1500
1000
500
0
-50000
-45000
-40000
-35000
-30000
-25000
-20000
-15000
-10000
-5000
0
Location of CTGAAA motifs (nt)

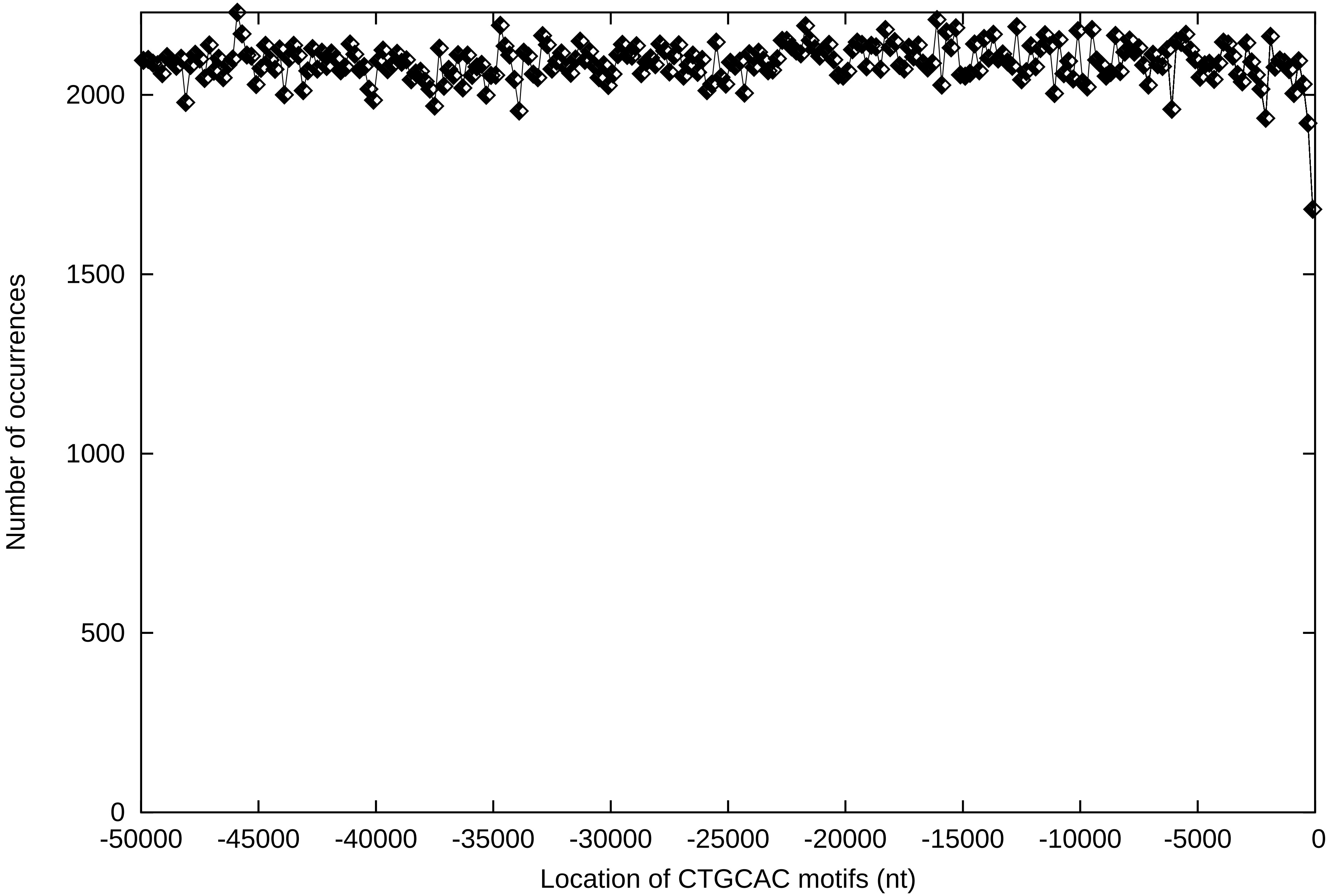

Number of occurrences
0
500
1000
1500
2000
-50000
-45000
-40000
-35000
-30000
-25000
-20000
-15000
-10000
-5000
0
Location of CTGCAC motifs (nt)

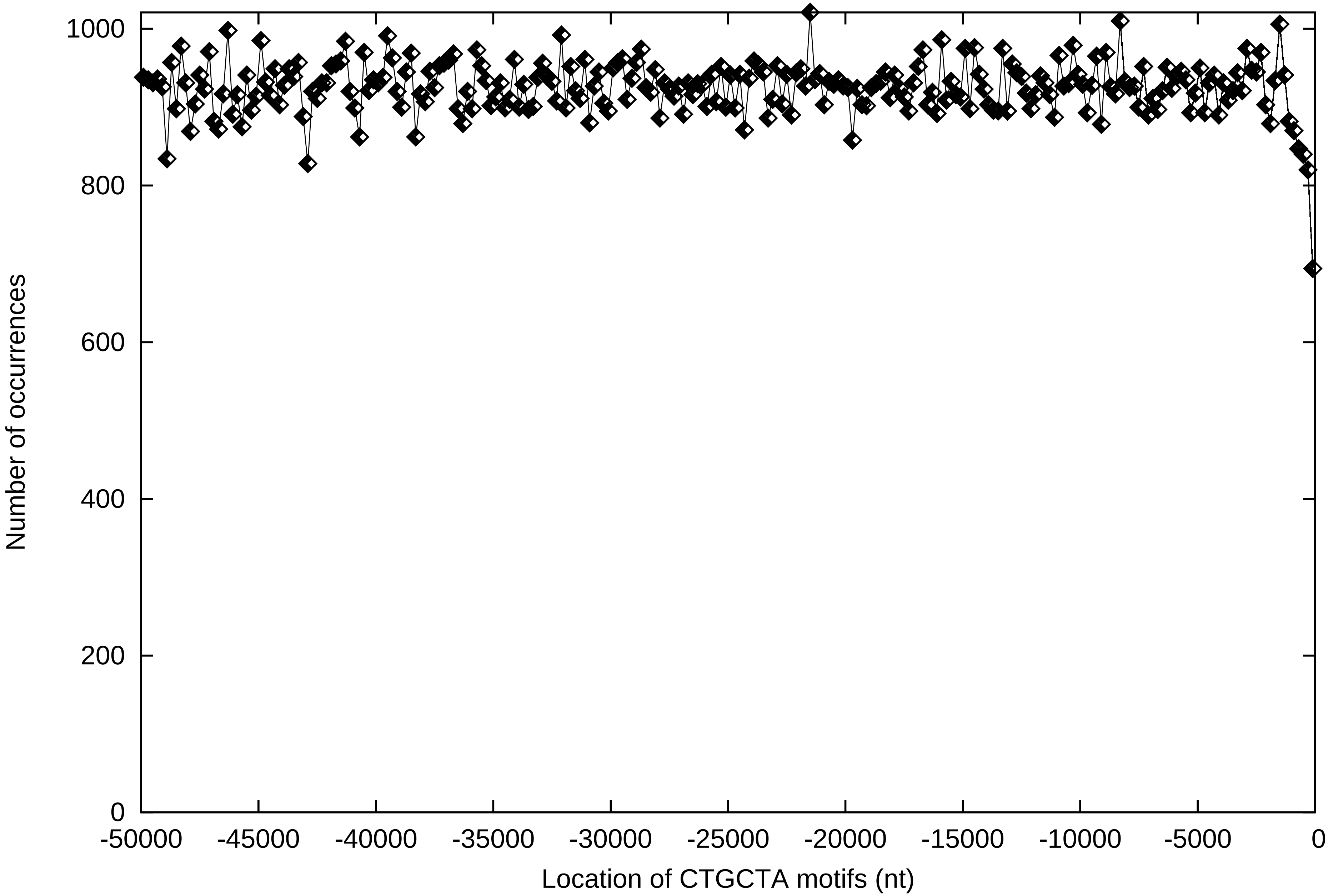

Number of occurrences
0
200
400
600
800
1000
-50000
-45000
-40000
-35000
-30000
-25000
-20000
-15000
-10000
-5000
0
Location of CTGCTA motifs (nt)

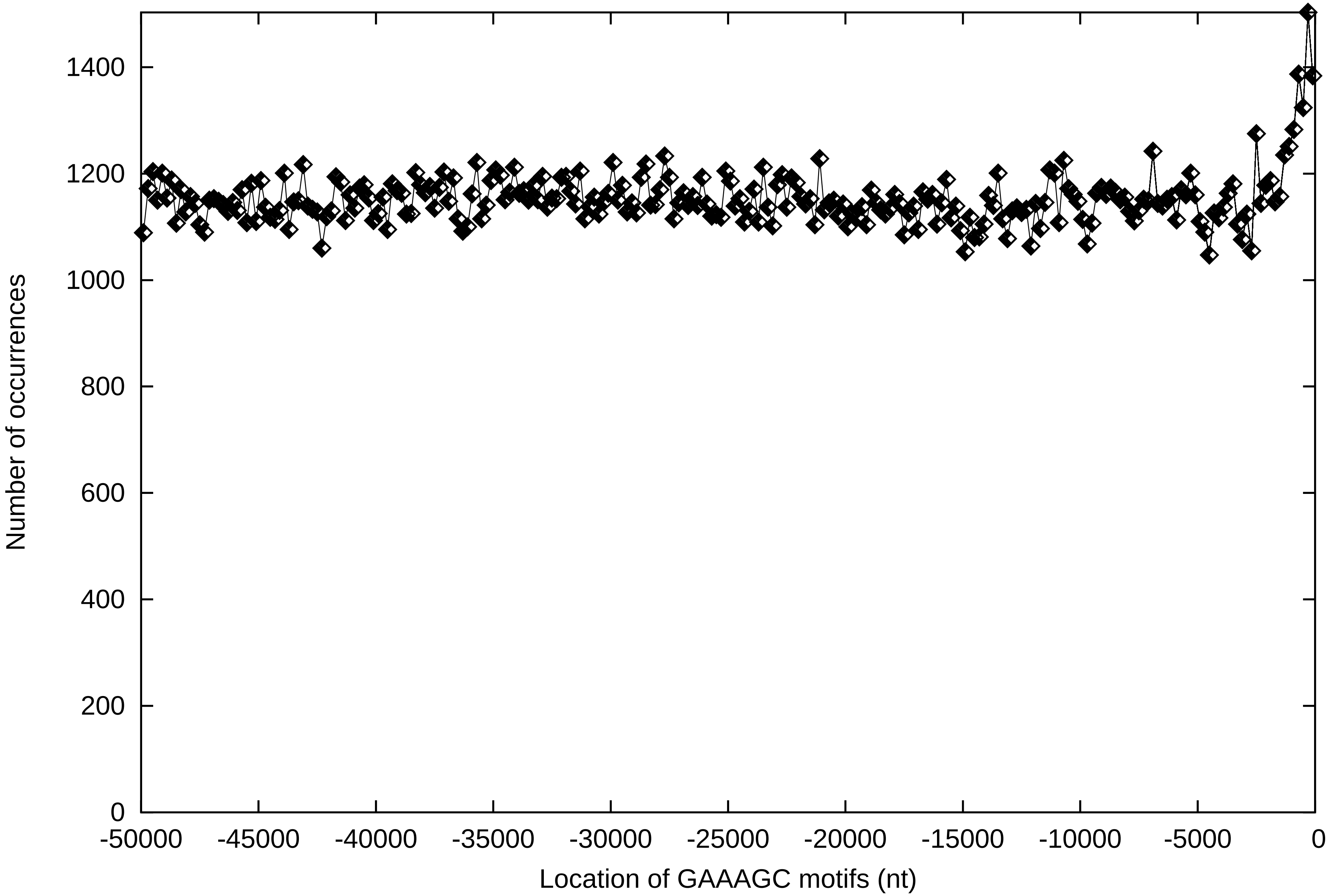

Number of occurrences
0
200
400
600
800
1000
1200
1400
-50000
-45000
-40000
-35000
-30000
-25000
-20000
-15000
-10000
-5000
0
Location of GAAAGC motifs (nt)

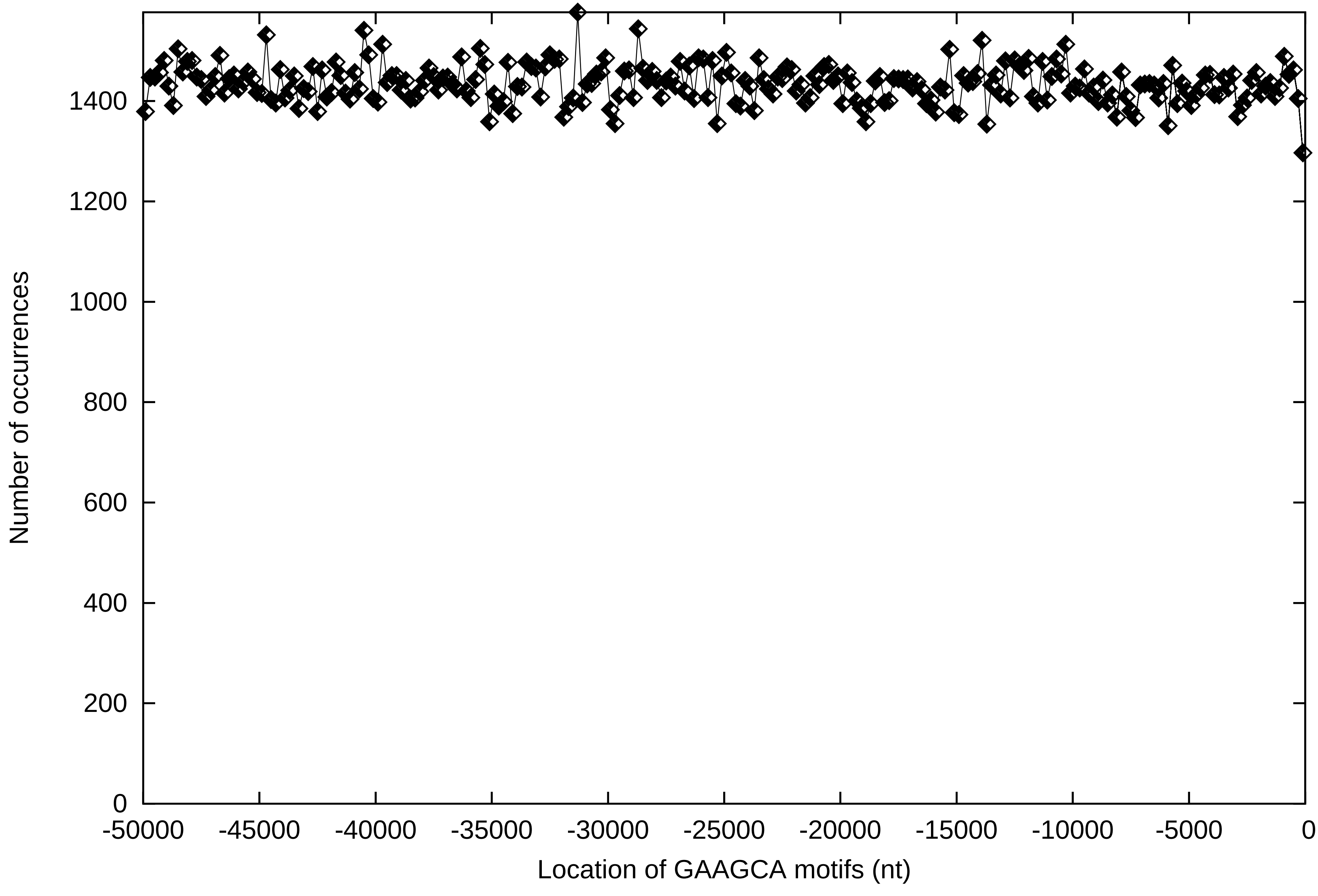

Number of occurrences
1400
1200
1000
800
600
400
200
0
-50000
-45000
-40000
-35000
-30000
-25000
-20000
-15000
-10000
-5000
0
Location of GAAGCA motifs (nt)

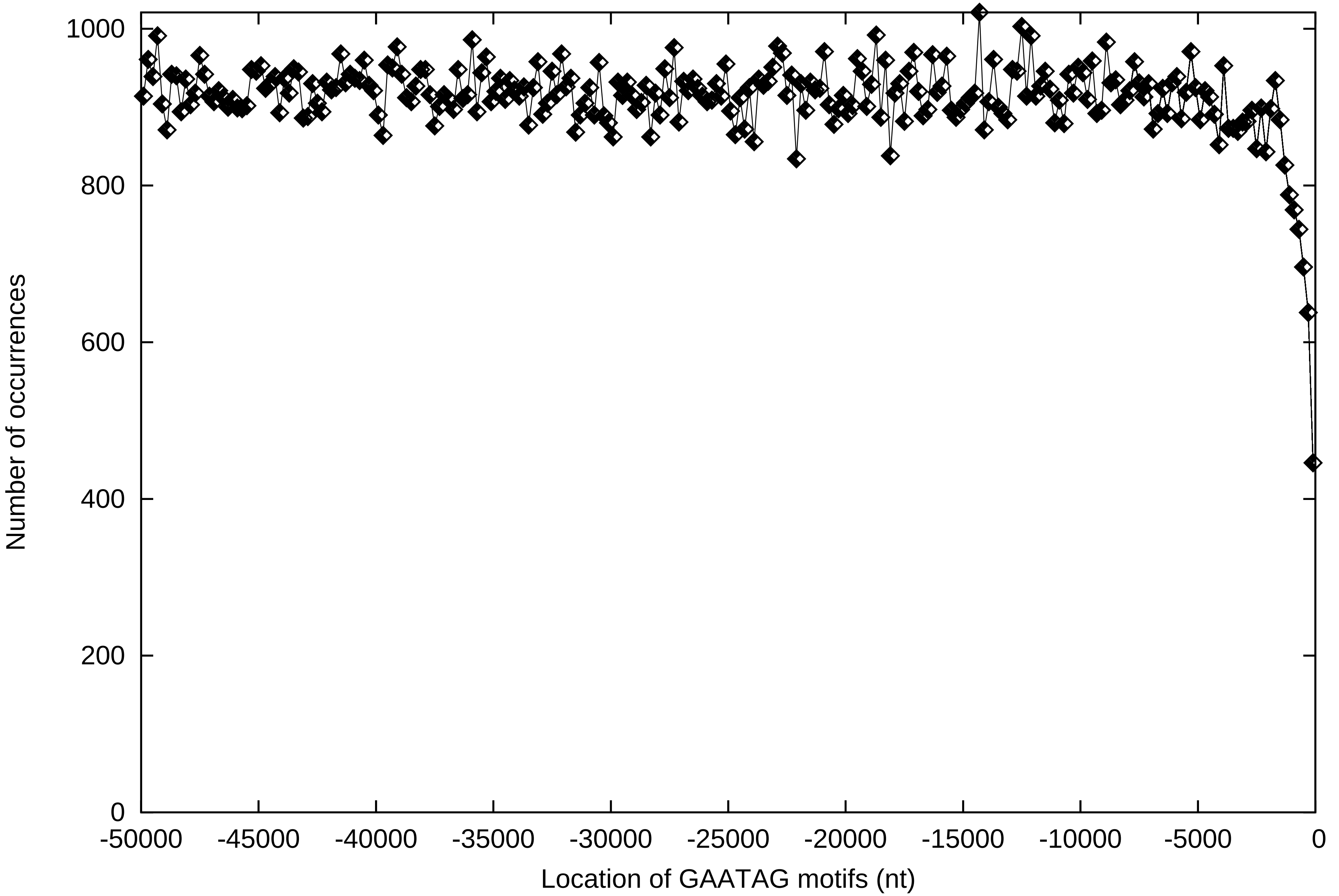

1000
800
600
400
200
0
-50000
-45000
-40000
-35000
-30000
-25000
-20000
-15000
-10000
-5000
0
Number of occurrences
Location of GAATAG motifs (nt)

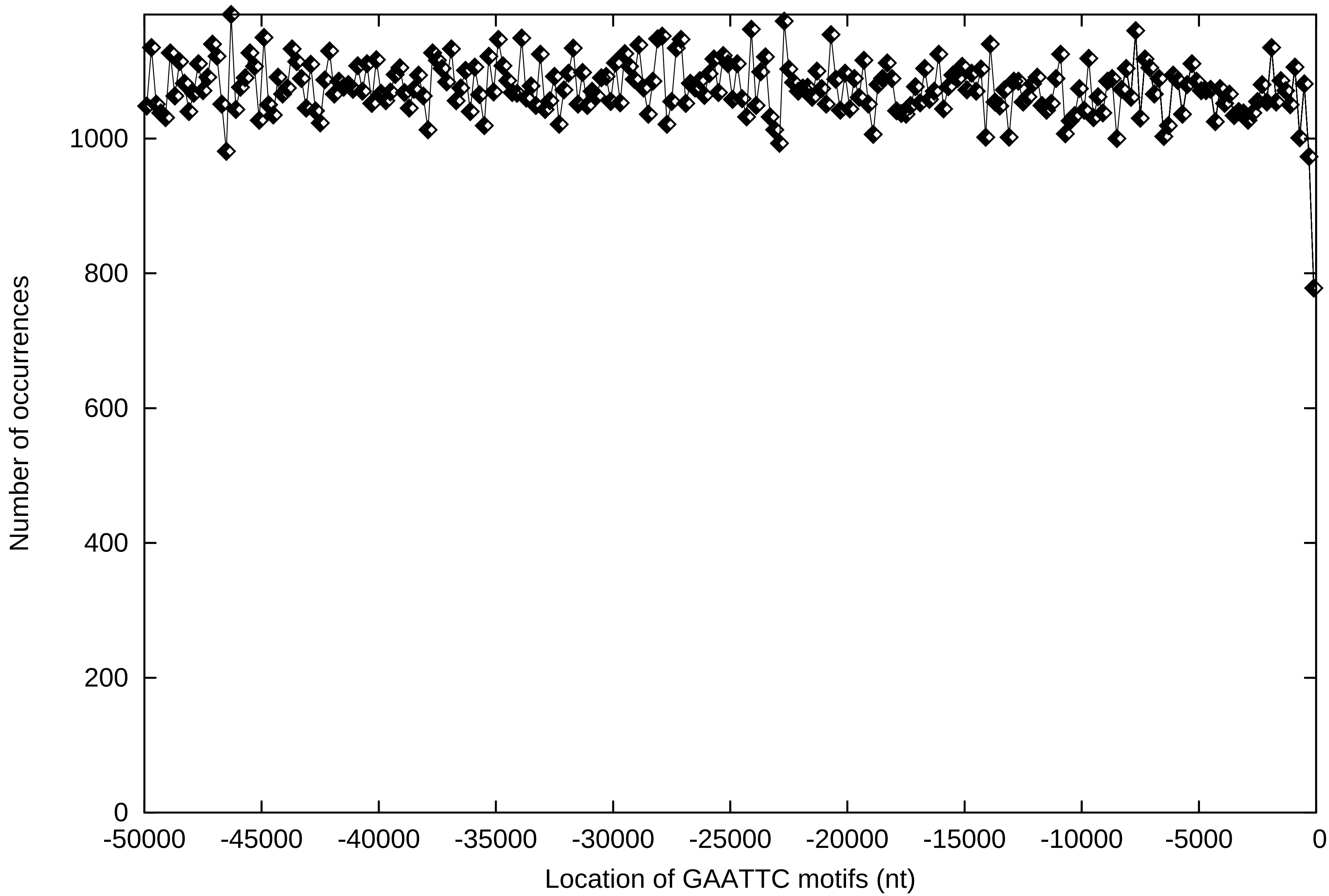
Number of occurrences
0
200
400
600
800
1000
-50000
-45000
-40000
-35000
-30000
-25000
-20000
-15000
-10000
-5000
0
Location of GAATTC motifs (nt)

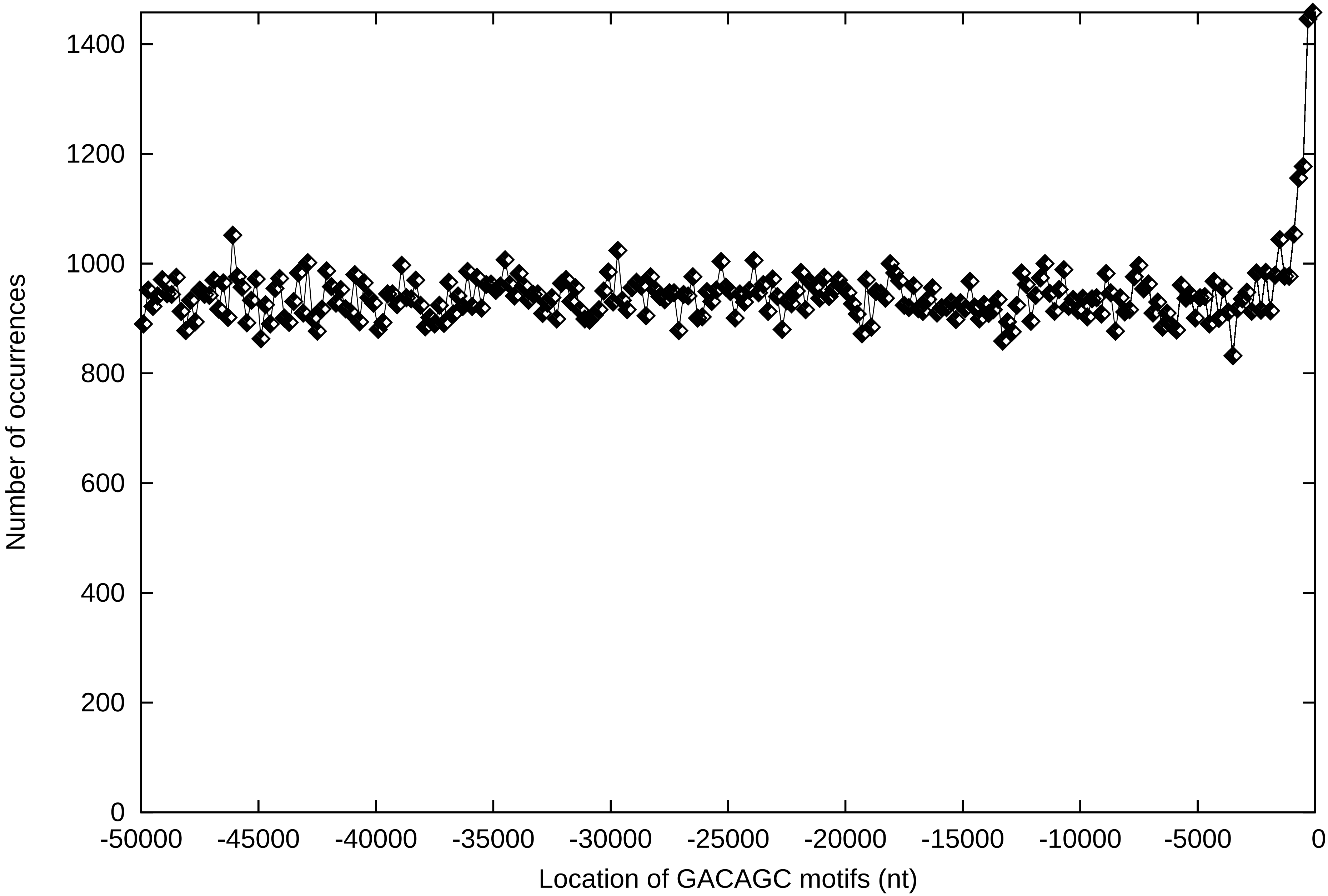

Number of occurrences
0
200
400
600
800
1000
1200
1400
-50000
-45000
-40000
-35000
-30000
-25000
-20000
-15000
-10000
-5000
0
Location of GACAGC motifs (nt)

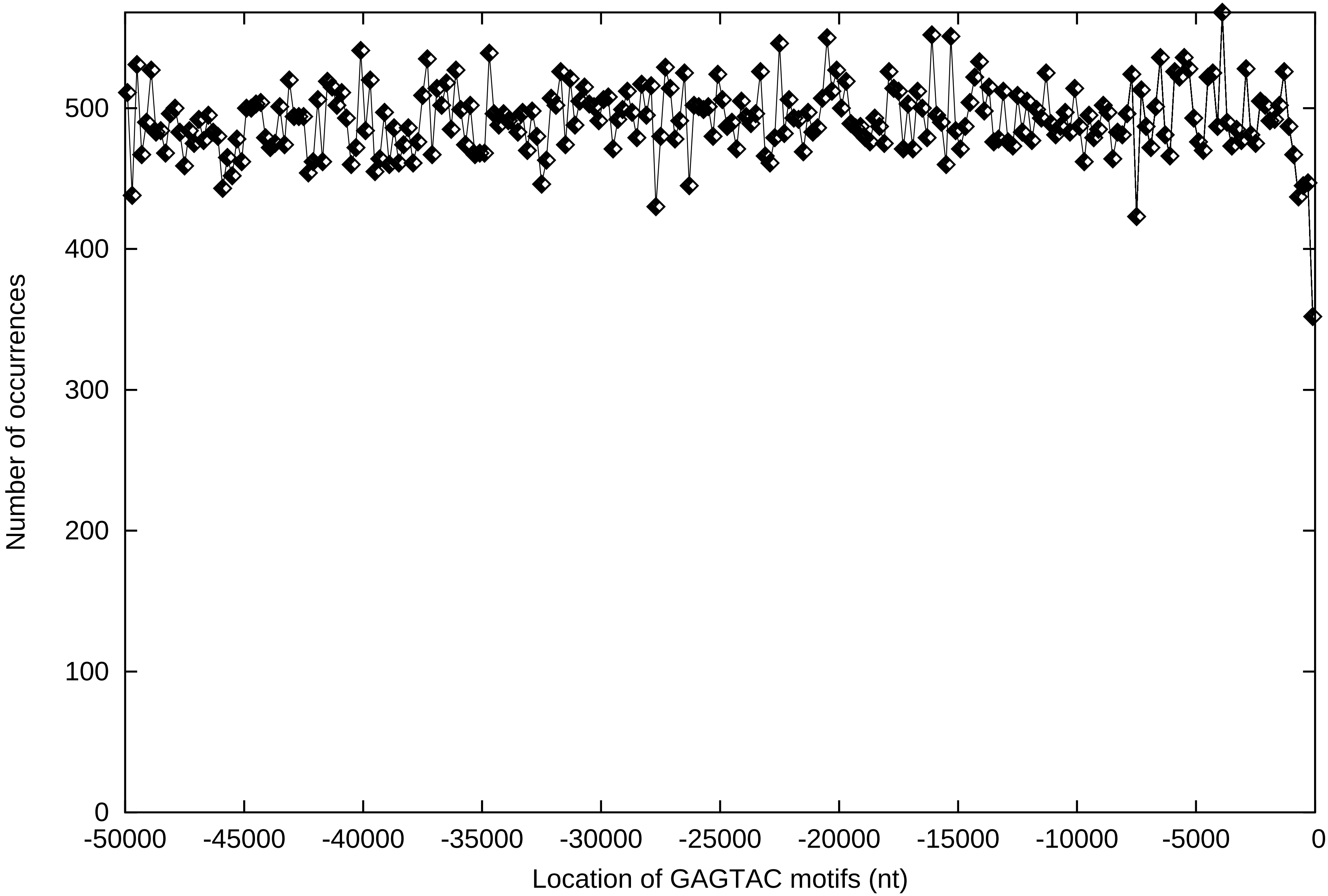

Number of occurrences
Location of GAGTAC motifs (nt)
0
100
200
300
400
500
-50000
-45000
-40000
-35000
-30000
-25000
-20000
-15000
-10000
-5000
0

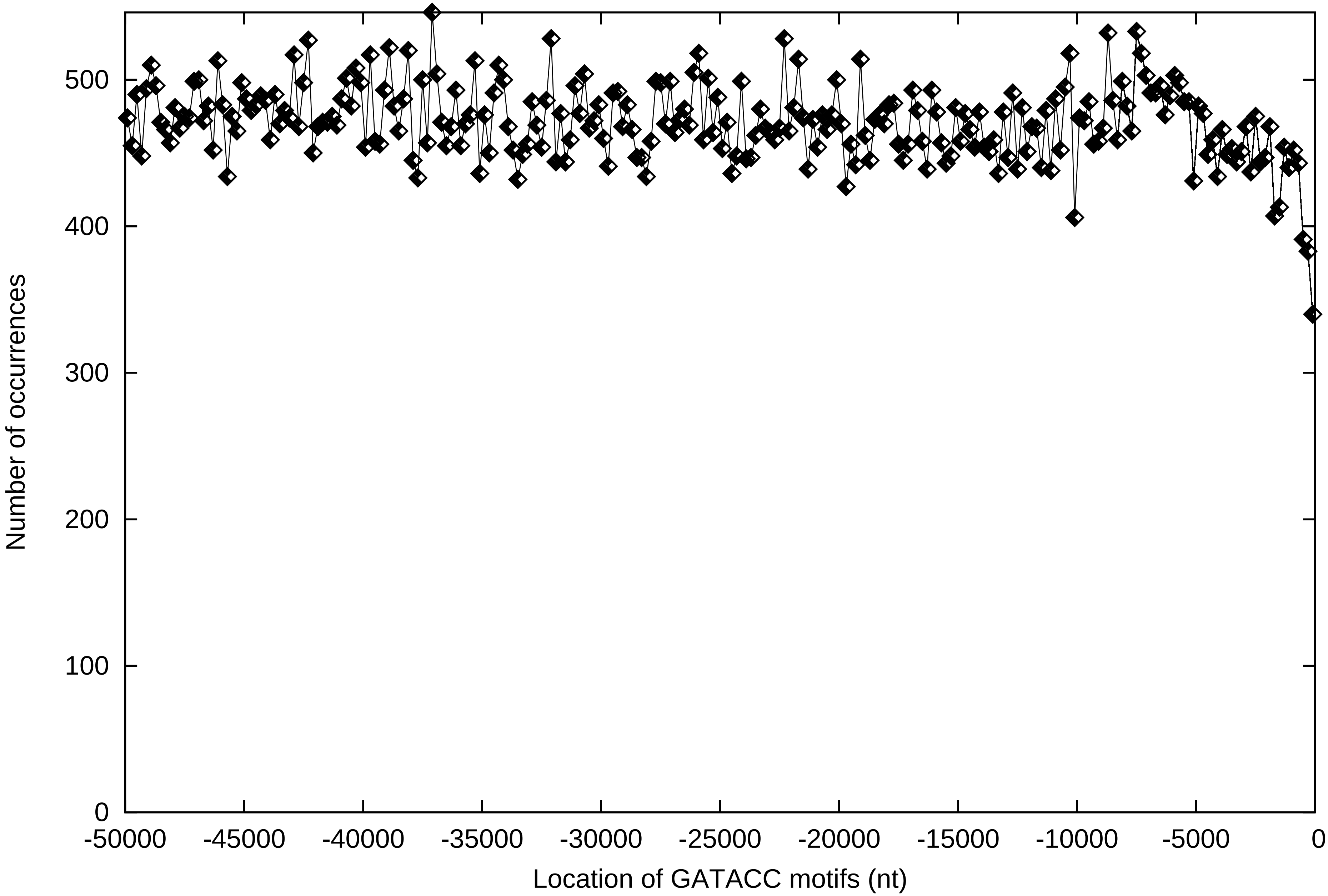

Number of occurrences
0
100
200
300
400
500
-50000
-45000
-40000
-35000
-30000
-25000
-20000
-15000
-10000
-5000
0
Location of GATACC motifs (nt)

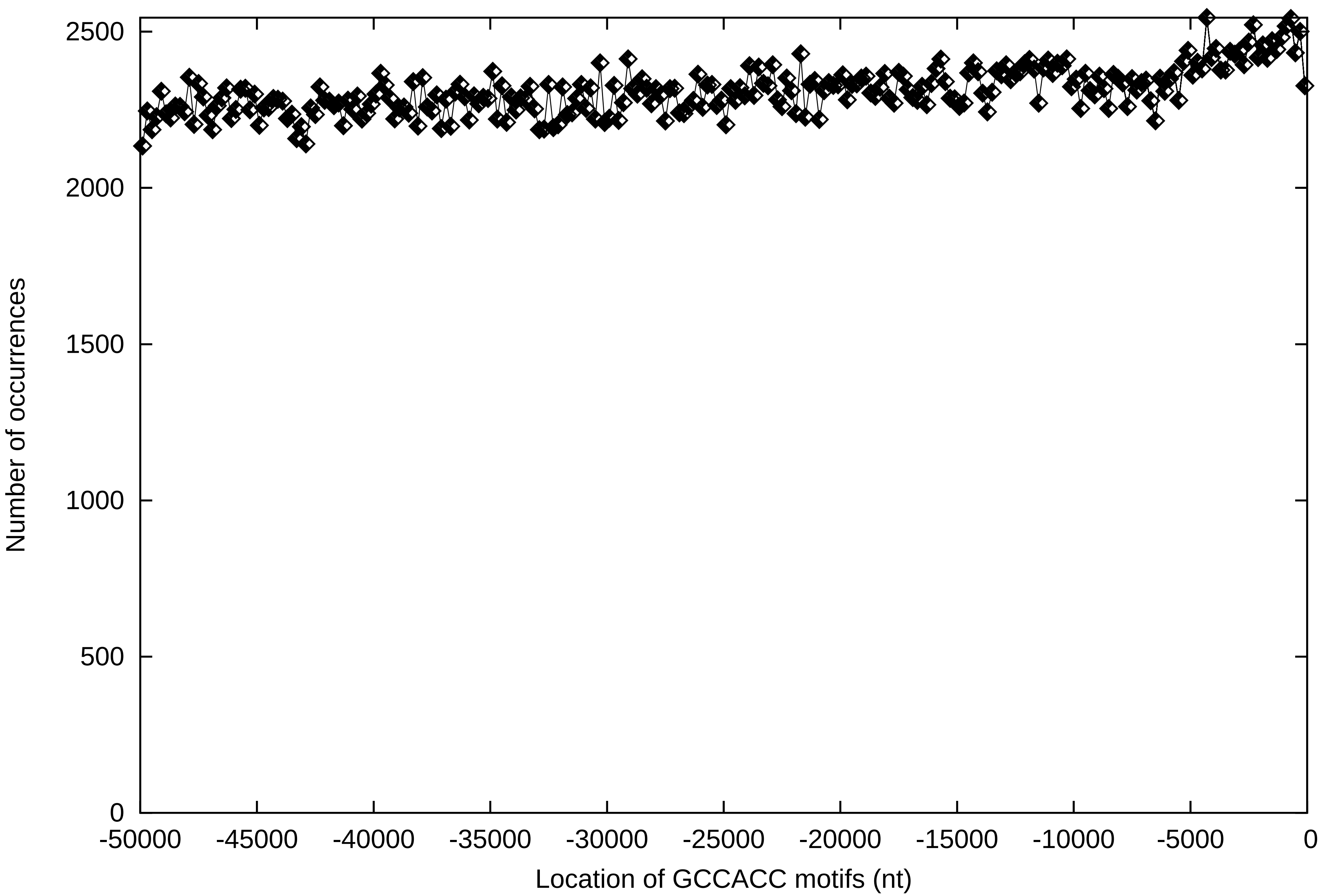

Number of occurrences
Location of GCCACC motifs (nt)
2500
2000
1500
1000
500
0
-50000
-45000
-40000
-35000
-30000
-25000
-20000
-15000
-10000
-5000
0

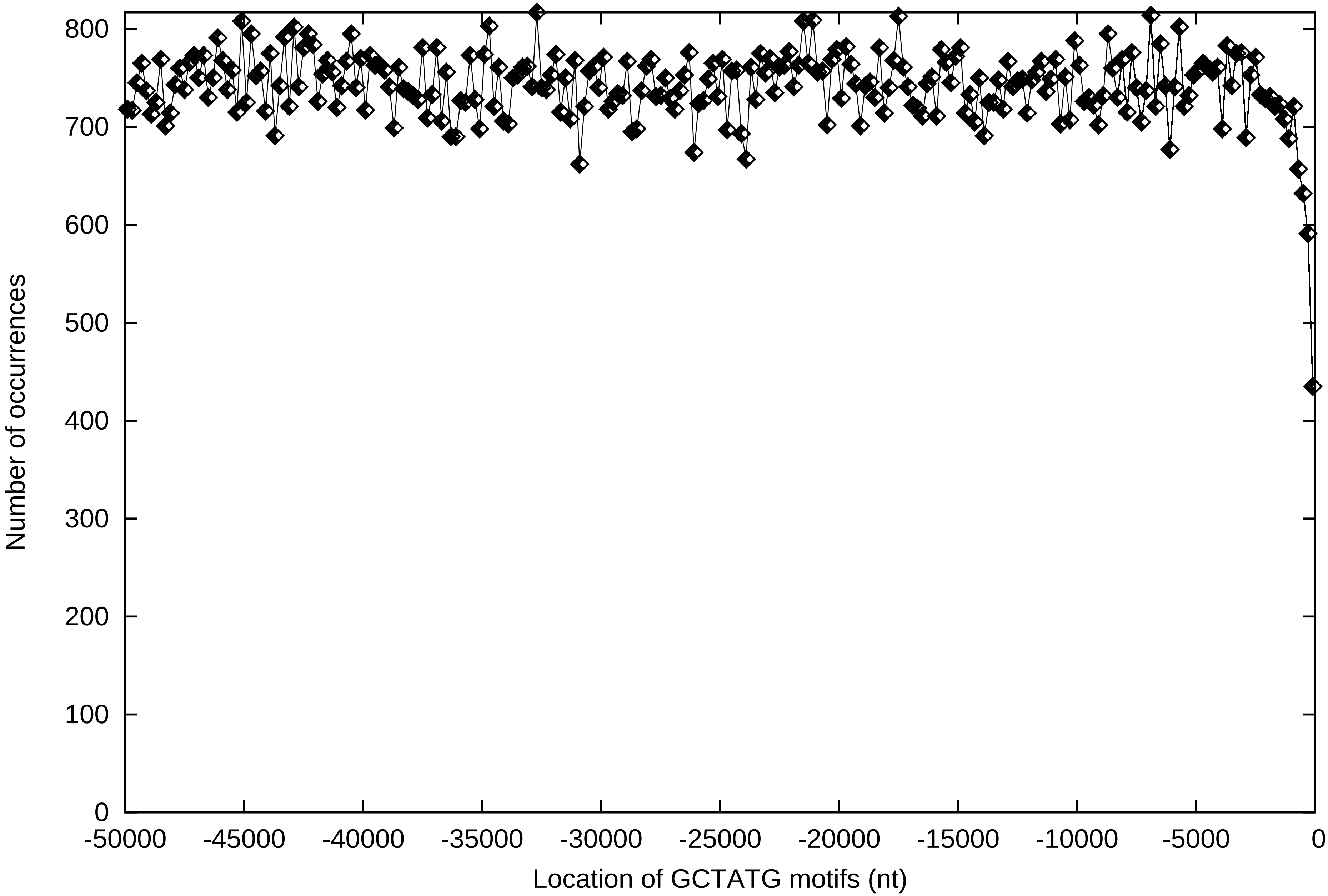

800
700
600
500
400
300
200
100
0
-50000
-45000
-40000
-35000
-30000
-25000
-20000
-15000
-10000
-5000
0
Number of occurrences
Location of GCTATG motifs (nt)

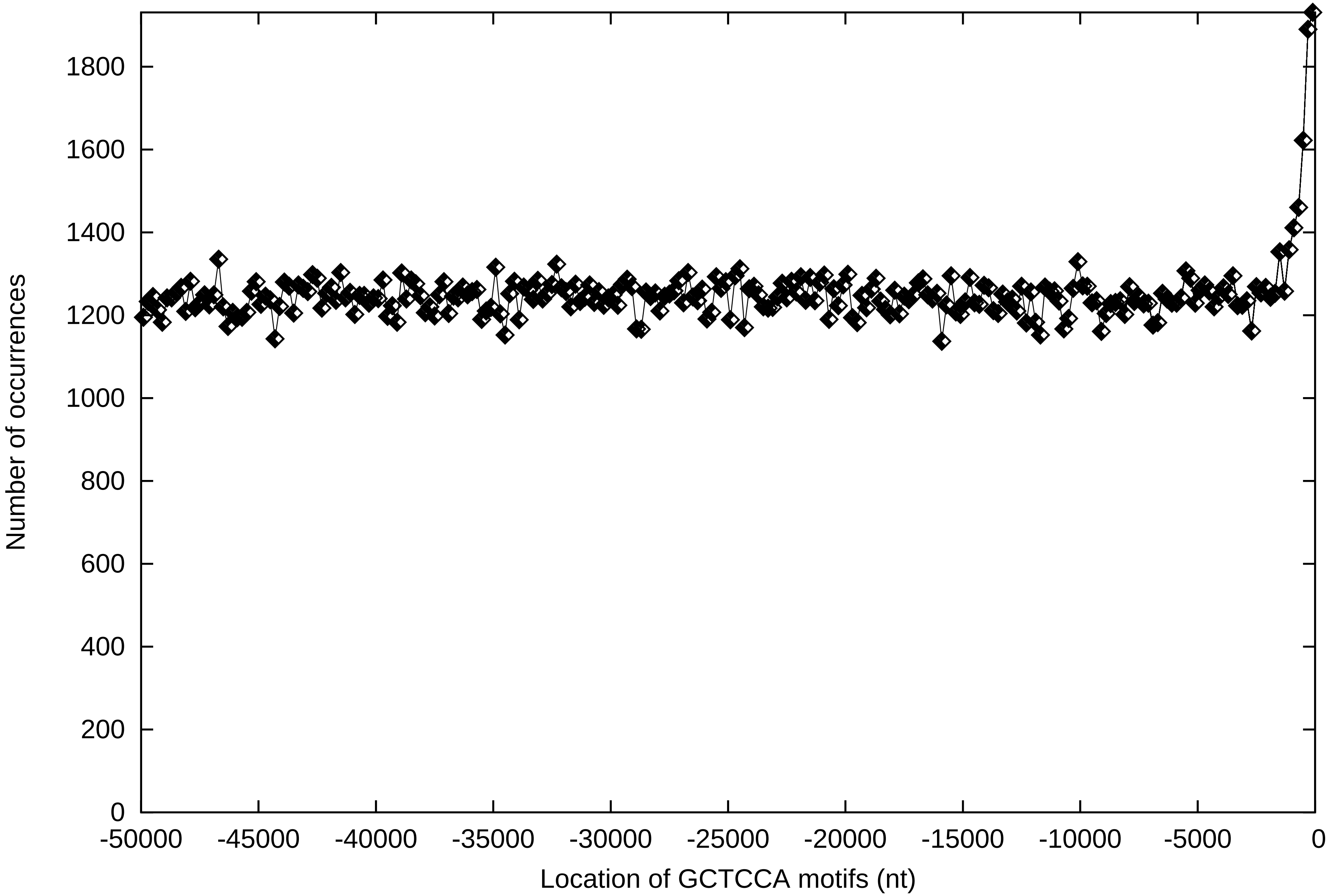

Number of occurrences
1800
1600
1400
1200
1000
800
600
400
200
0
-50000
-45000
-40000
-35000
-30000
-25000
-20000
-15000
-10000
-5000
0
Location of GCTCCA motifs (nt)

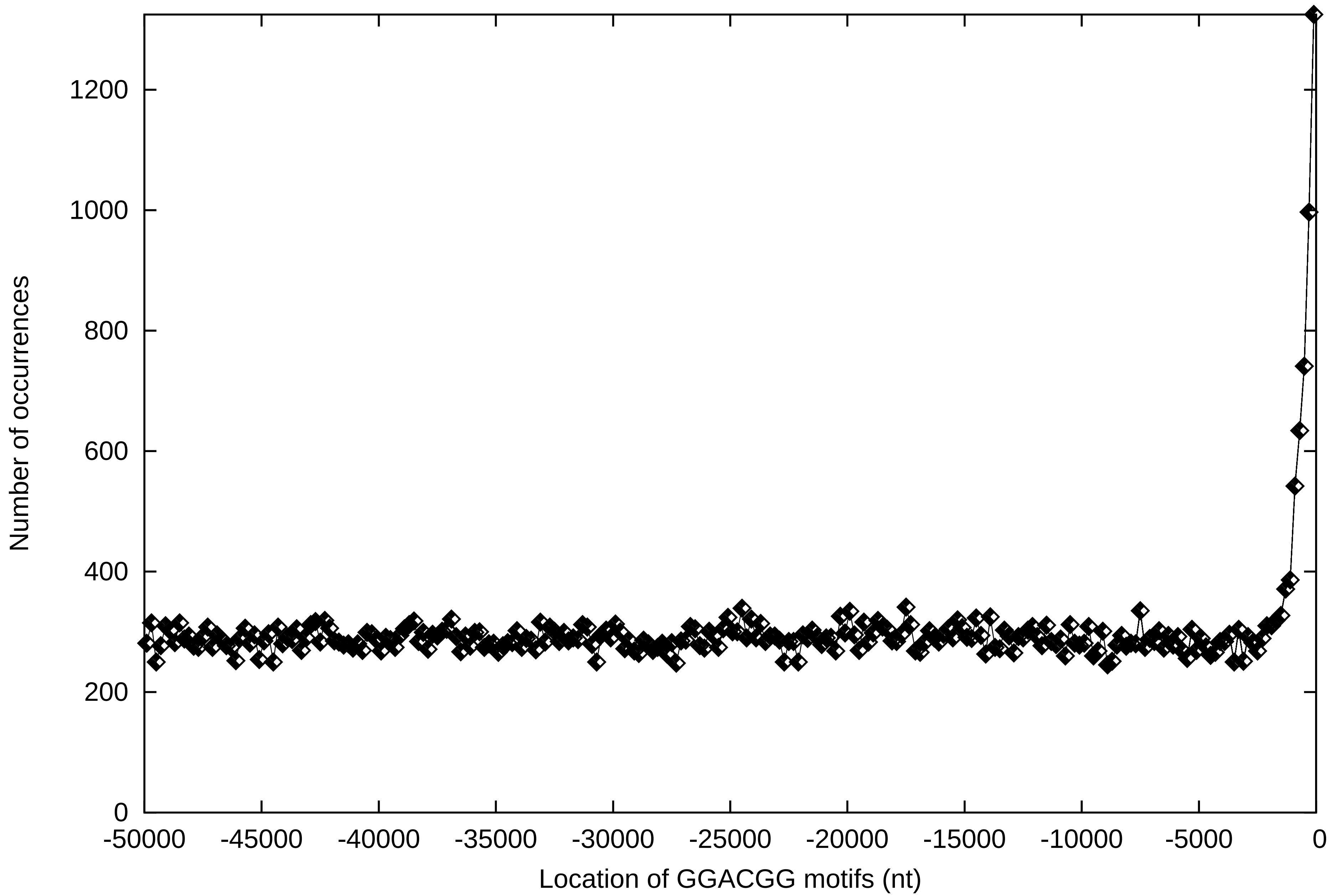
Number of occurrences
0
200
400
600
800
1000
1200
-50000
-45000
-40000
-35000
-30000
-25000
-20000
-15000
-10000
-5000
0
Location of GGACGG motifs (nt)

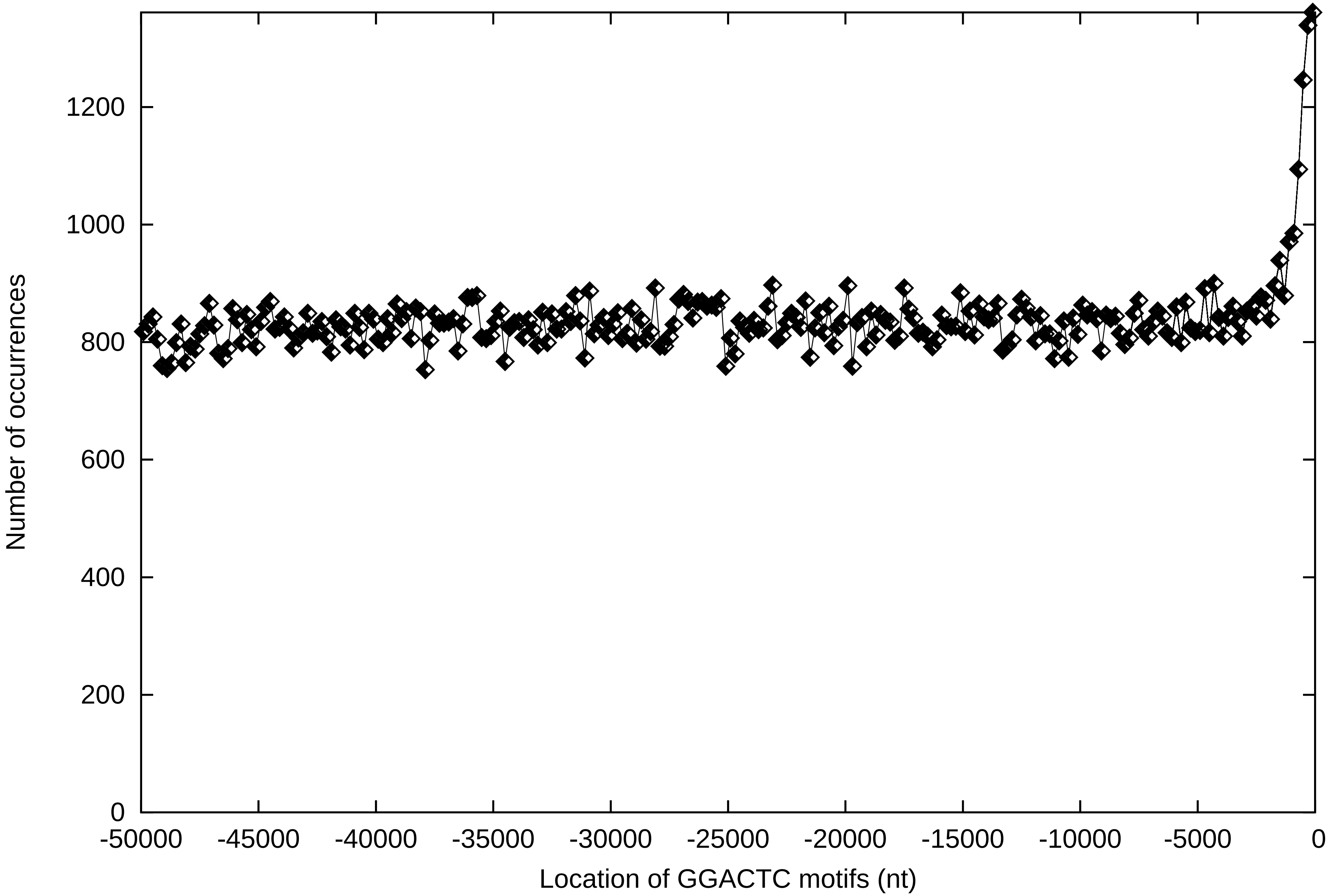

Number of occurrences
0
200
400
600
800
1000
1200
-50000
-45000
-40000
-35000
-30000
-25000
-20000
-15000
-10000
-5000
0
Location of GGACTC motifs (nt)

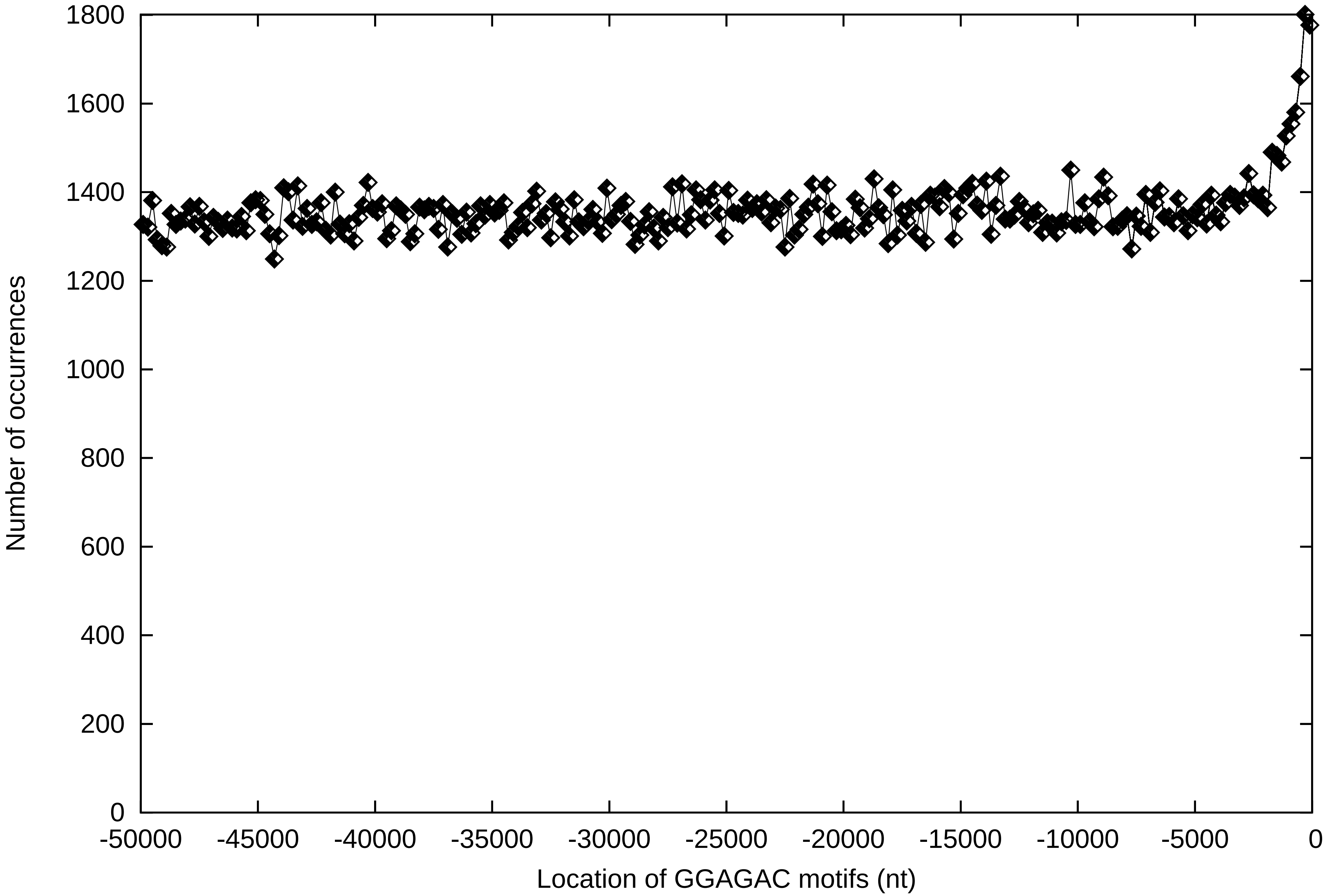
Number of occurrences
Location of GGAGAC motifs (nt)
1800
1600
1400
1200
1000
800
600
400
200
0
-50000
-45000
-40000
-35000
-30000
-25000
-20000
-15000
-10000
-5000
0

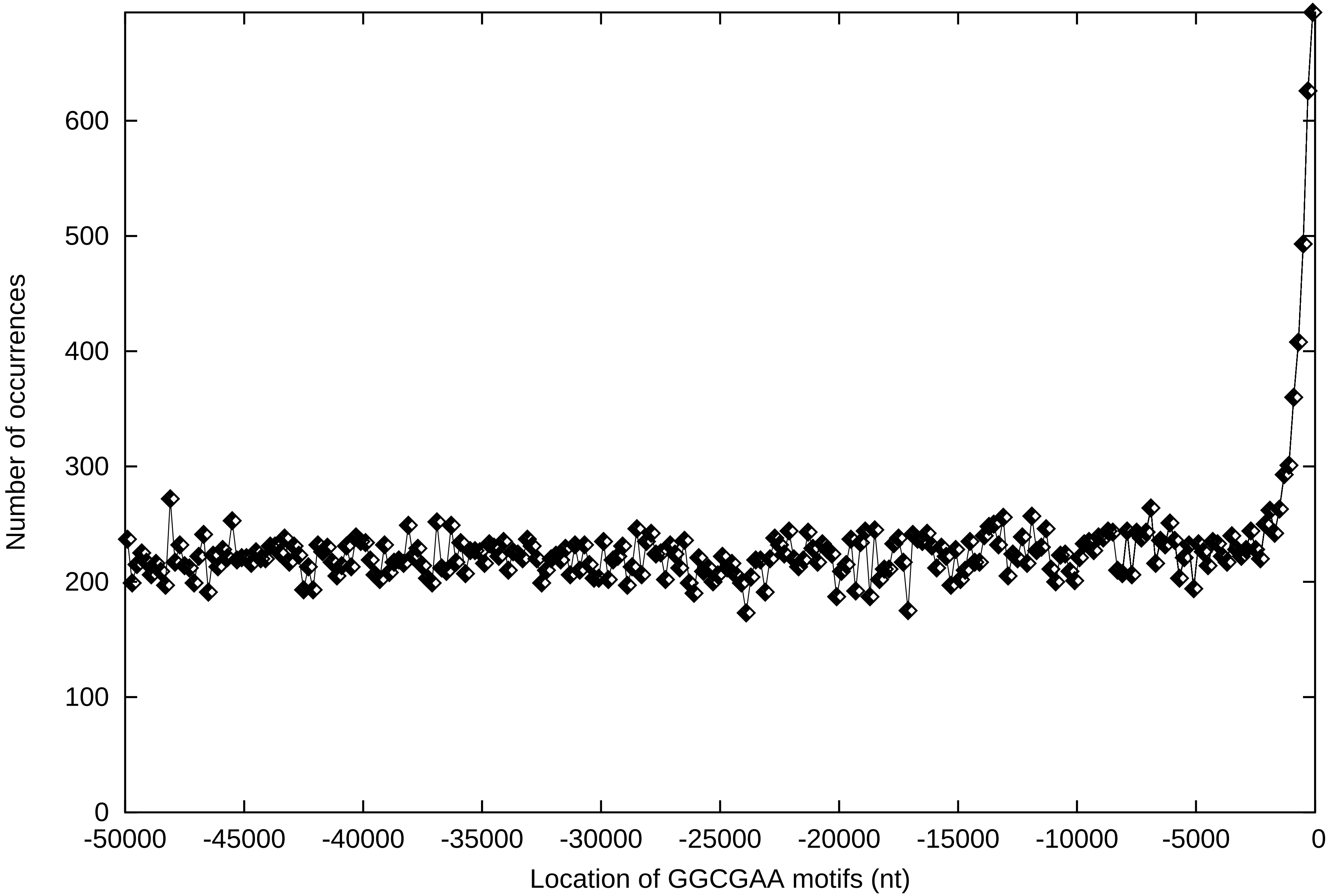

Number of occurrences
0
100
200
300
400
500
600
-50000
-45000
-40000
-35000
-30000
-25000
-20000
-15000
-10000
-5000
0
Location of GGCGAA motifs (nt)

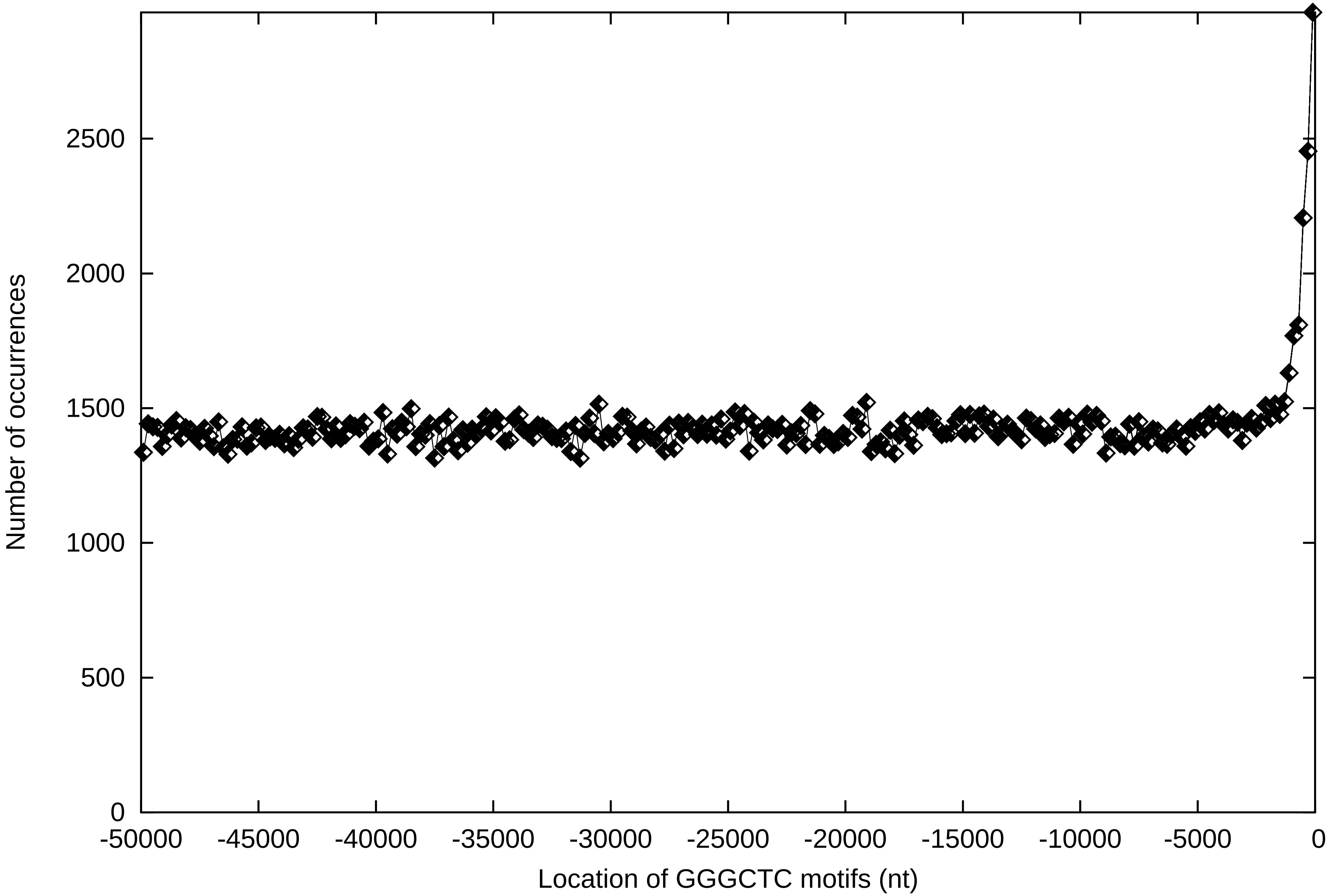

Number of occurrences
0
500
1000
1500
2000
2500
-50000
-45000
-40000
-35000
-30000
-25000
-20000
-15000
-10000
-5000
0
Location of GGGCTC motifs (nt)

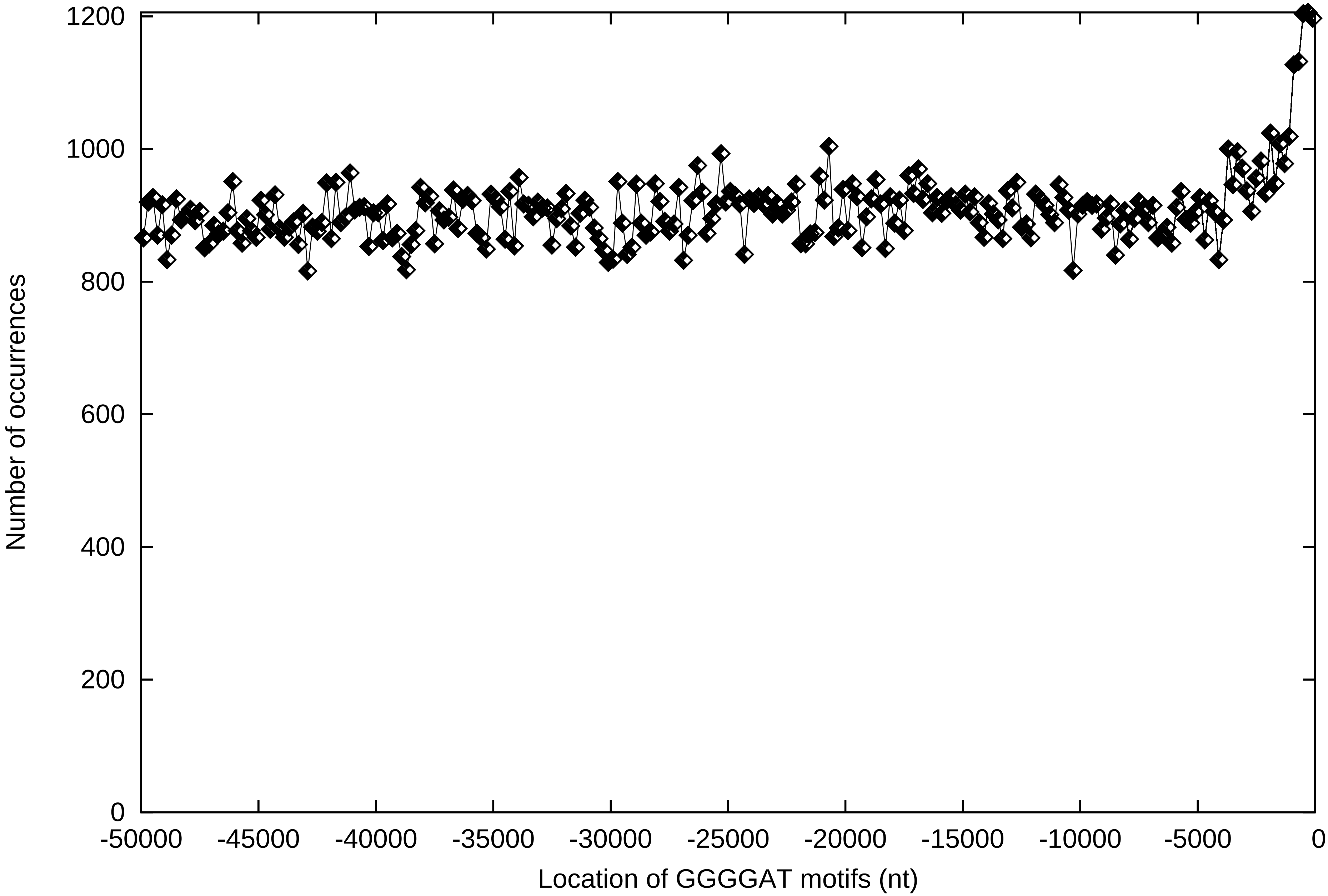

Number of occurrences
Location of GGGGAT motifs (nt)
0
200
400
600
800
1000
1200
-50000
-45000
-40000
-35000
-30000
-25000
-20000
-15000
-10000
-5000
0

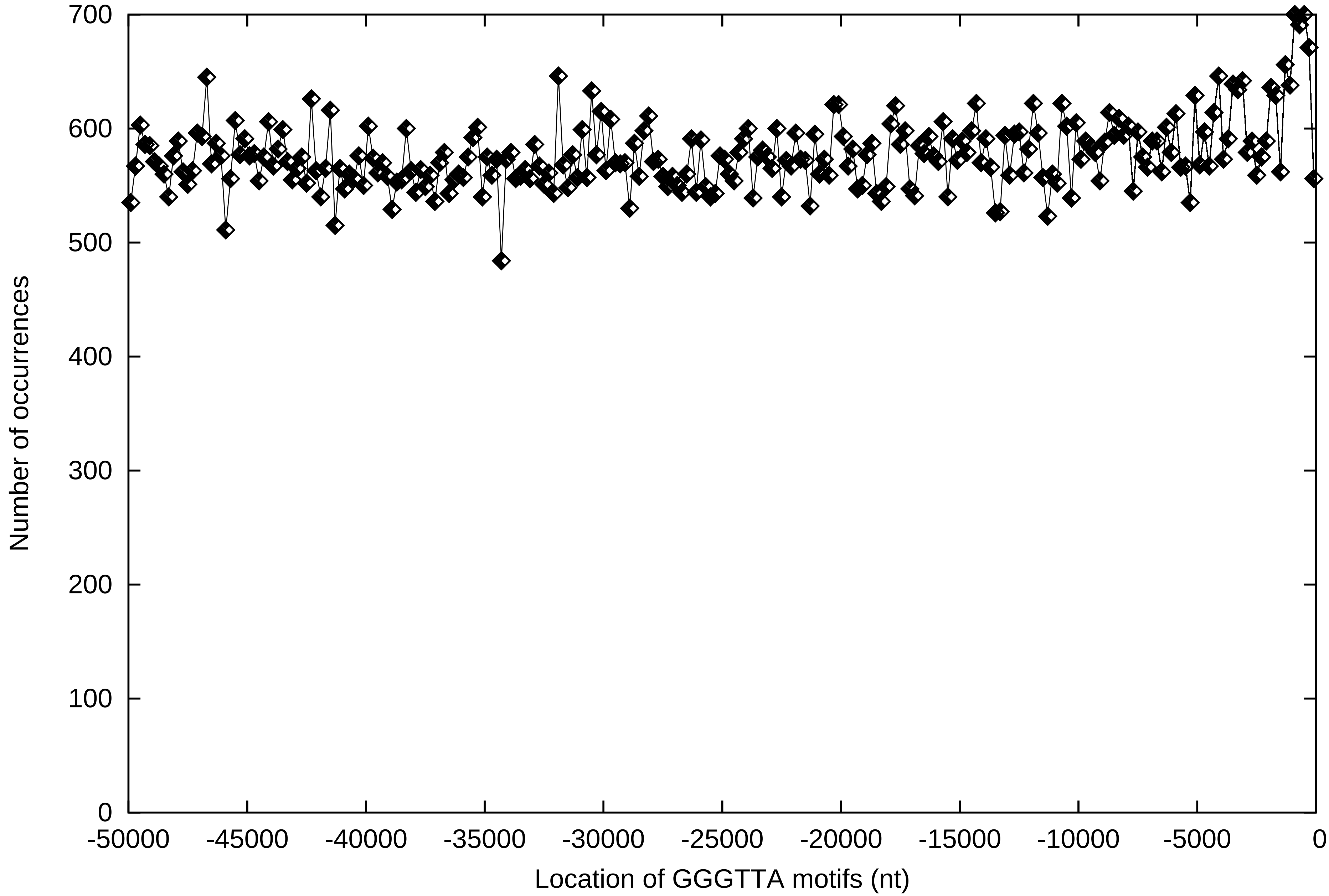

700
600
500
400
300
200
100
0
Number of occurrences
-50000
-45000
-40000
-35000
-30000
-25000
-20000
-15000
-10000
-5000
0
Location of GGGTTA motifs (nt)

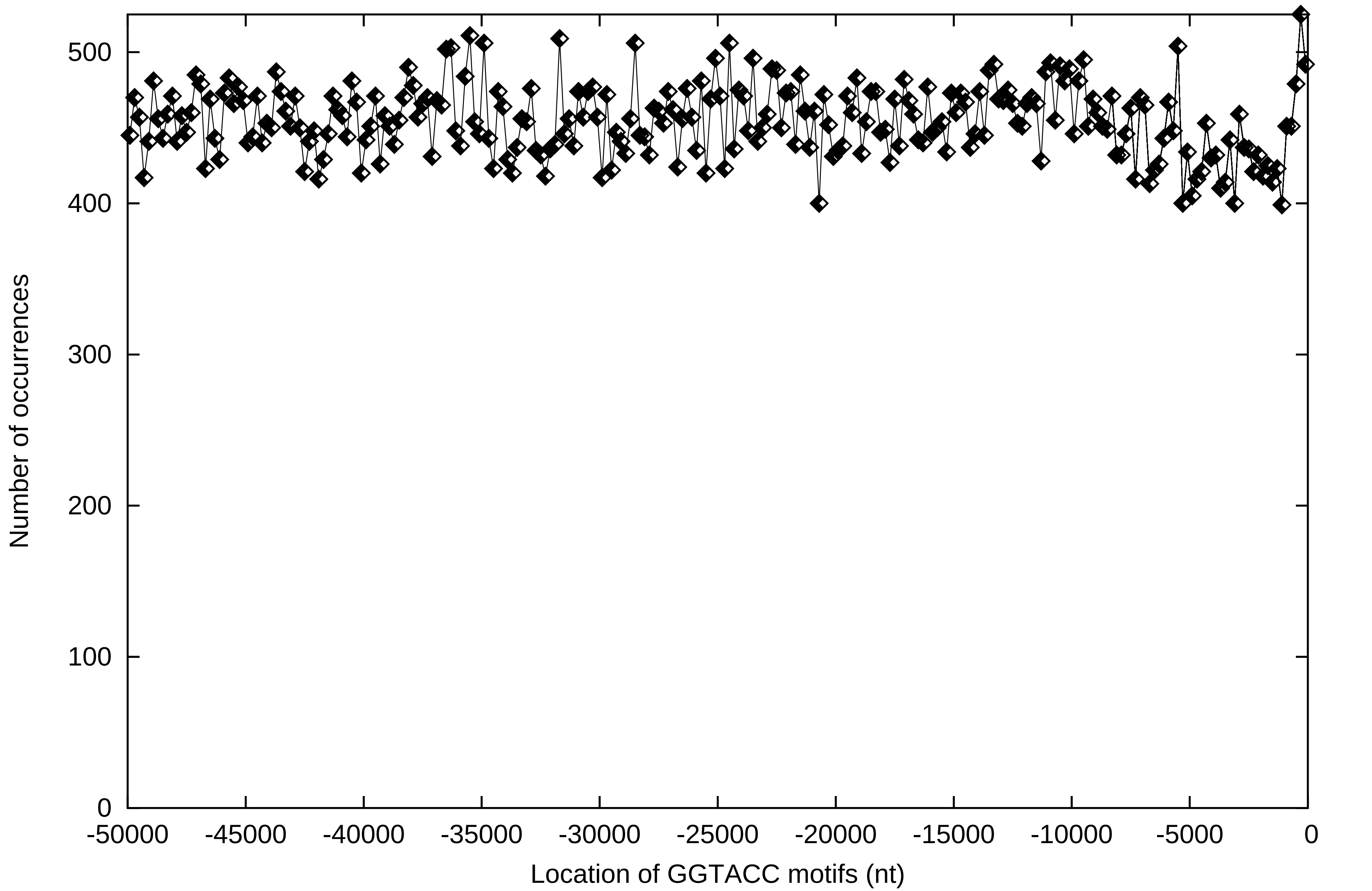

Number of occurrences
Location of GGTACC motifs (nt)
0
100
200
300
400
500
-50000
-45000
-40000
-35000
-30000
-25000
-20000
-15000
-10000
-5000
0

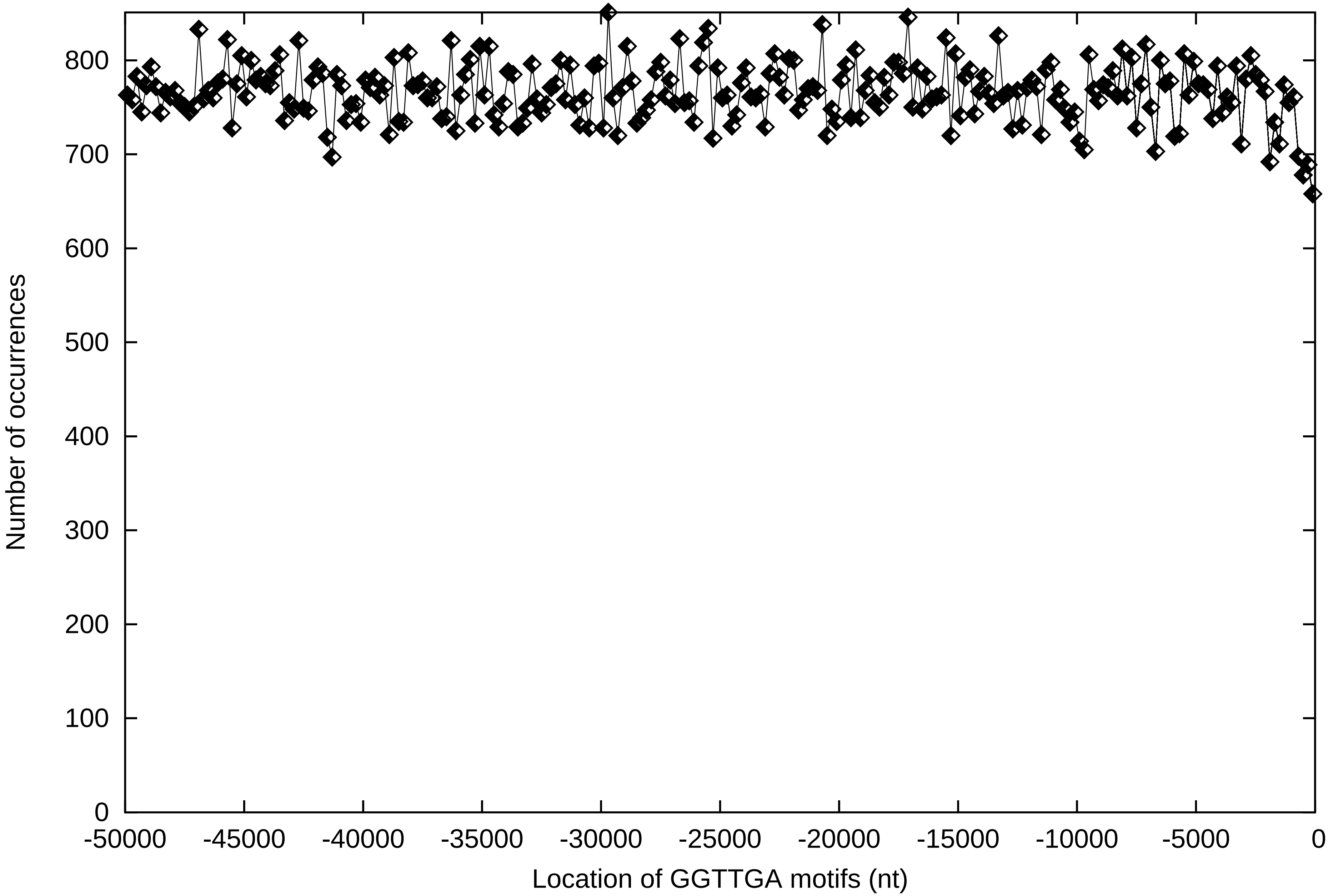

Number of occurrences
0
100
200
300
400
500
600
700
800
-50000
-45000
-40000
-35000
-30000
-25000
-20000
-15000
-10000
-5000
0
Location of GGTTGA motifs (nt)

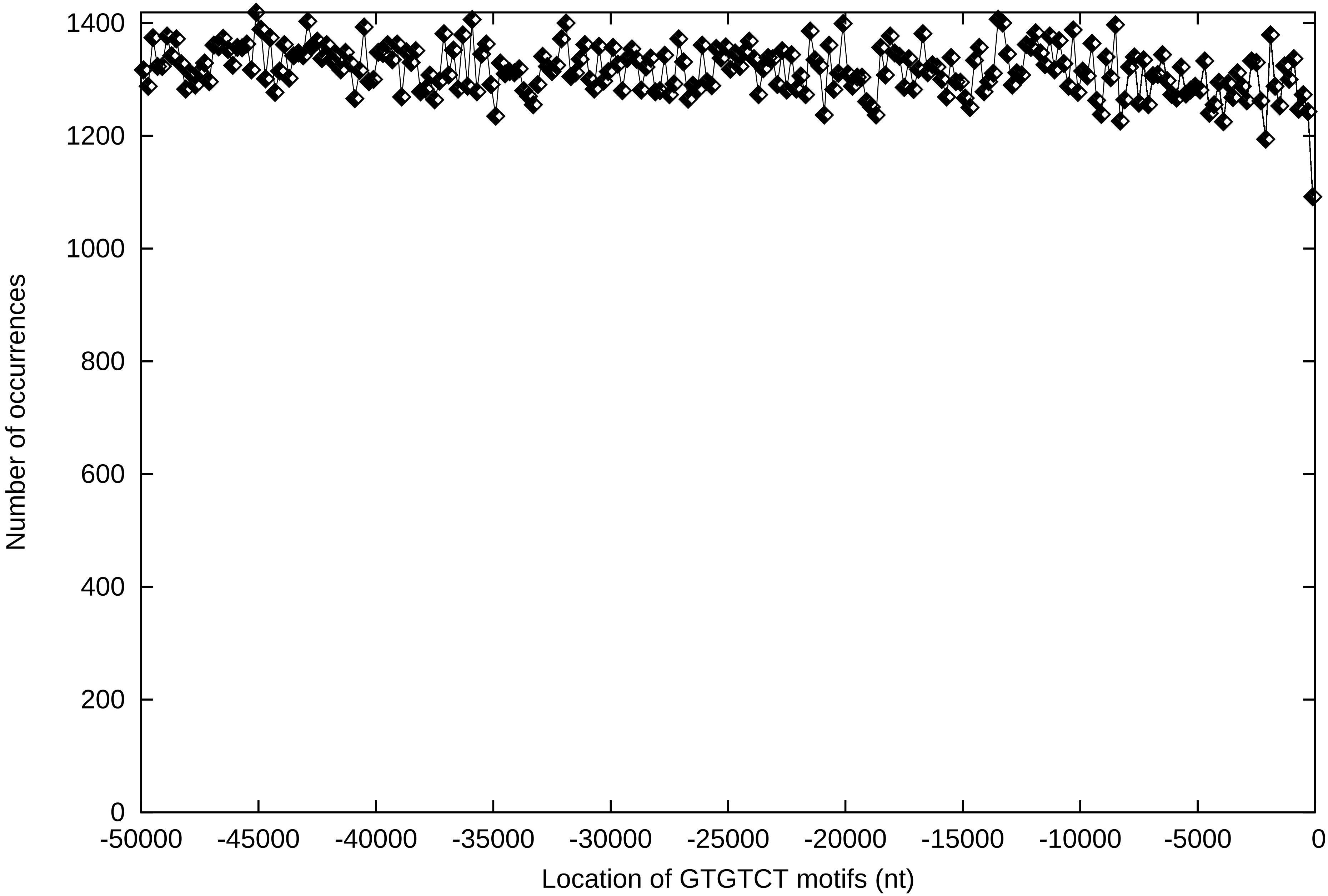

1400
1200
1000
800
600
400
200
0
Number of occurrences
-50000
-45000
-40000
-35000
-30000
-25000
-20000
-15000
-10000
-5000
0
Location of GTGTCT motifs (nt)

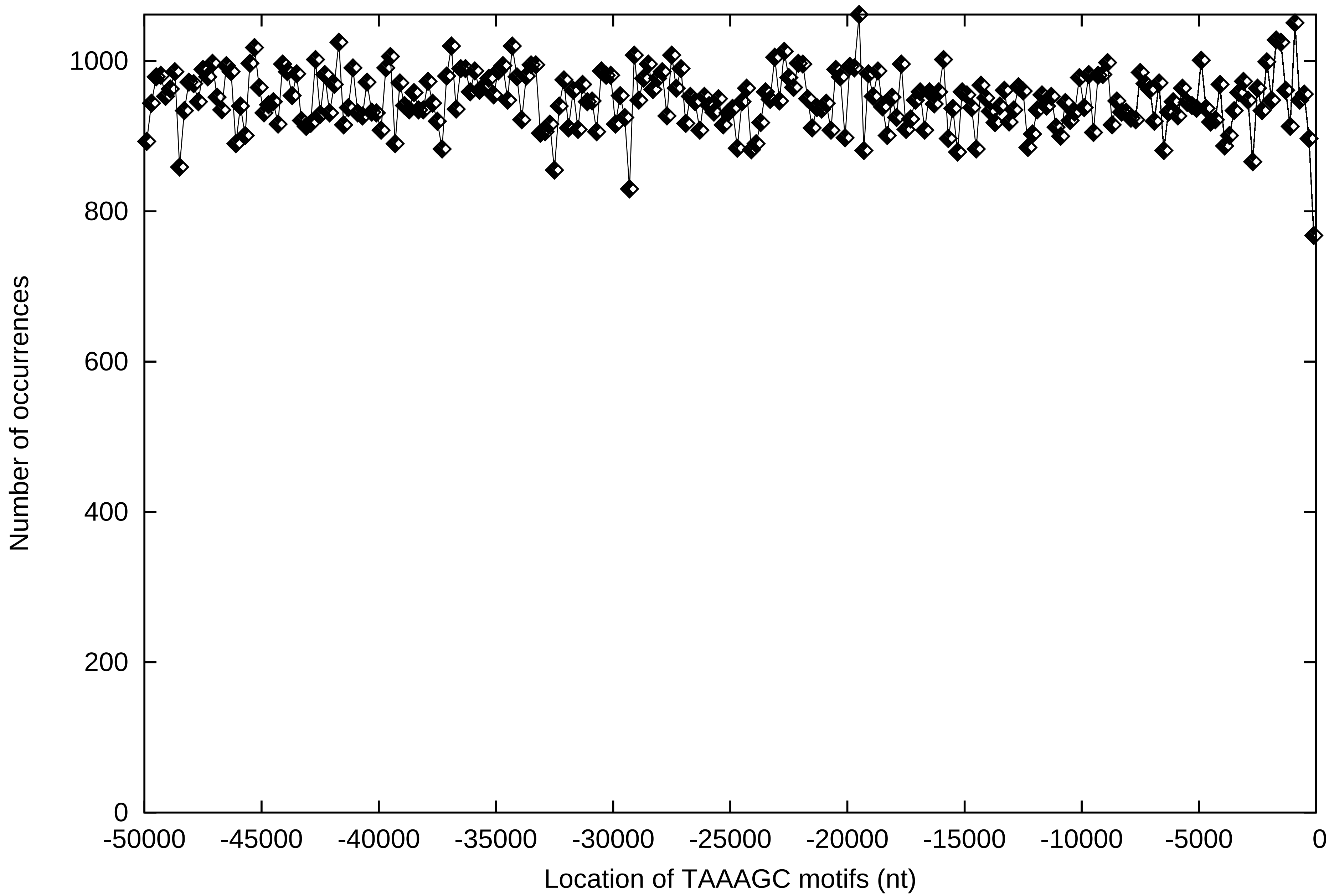

Number of occurrences
0
200
400
600
800
1000
-50000
-45000
-40000
-35000
-30000
-25000
-20000
-15000
-10000
-5000
0
Location of TAAAGC motifs (nt)

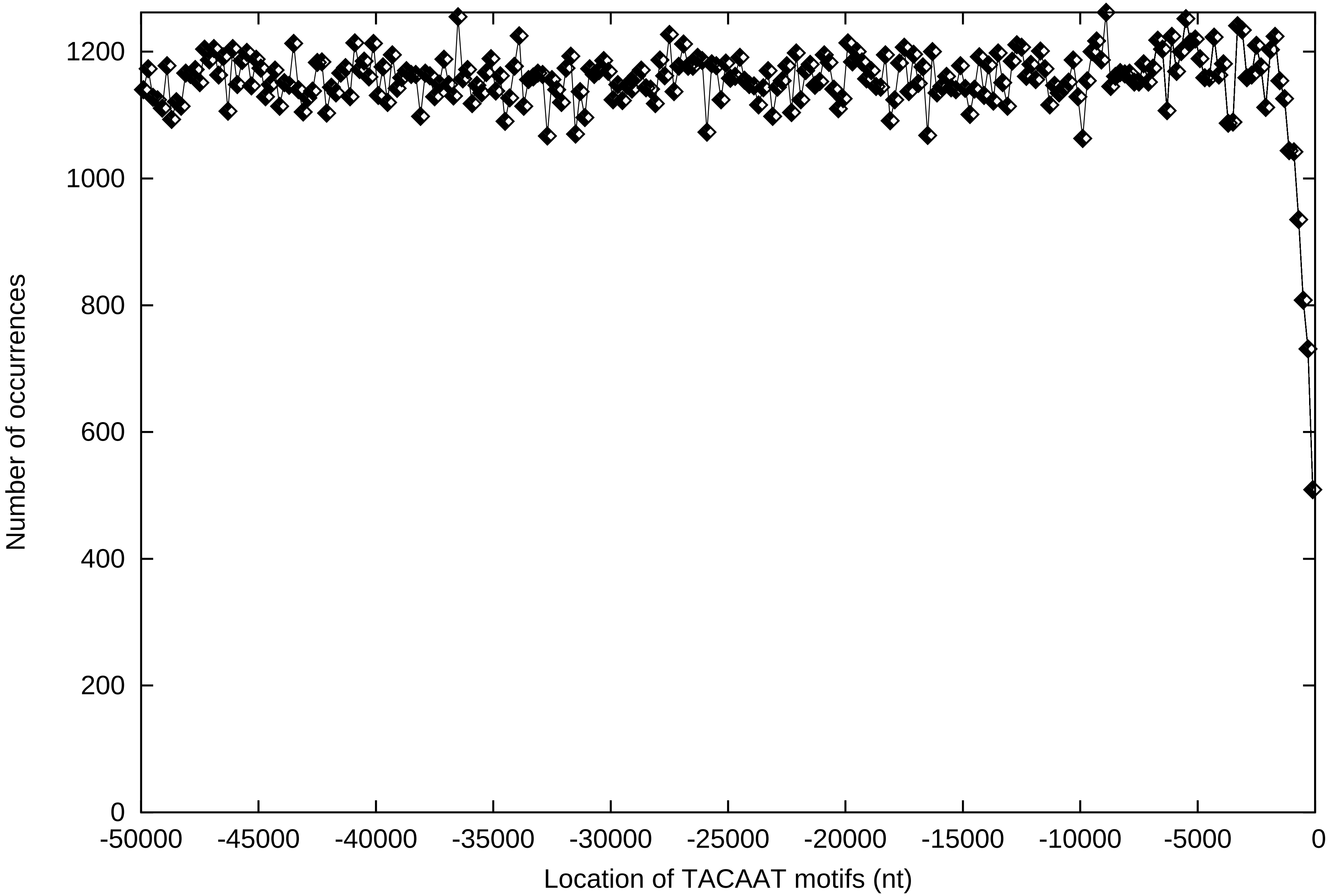
Number of occurrences
0
200
400
600
800
1000
1200
-50000
-45000
-40000
-35000
-30000
-25000
-20000
-15000
-10000
-5000
0
Location of TACAAT motifs (nt)

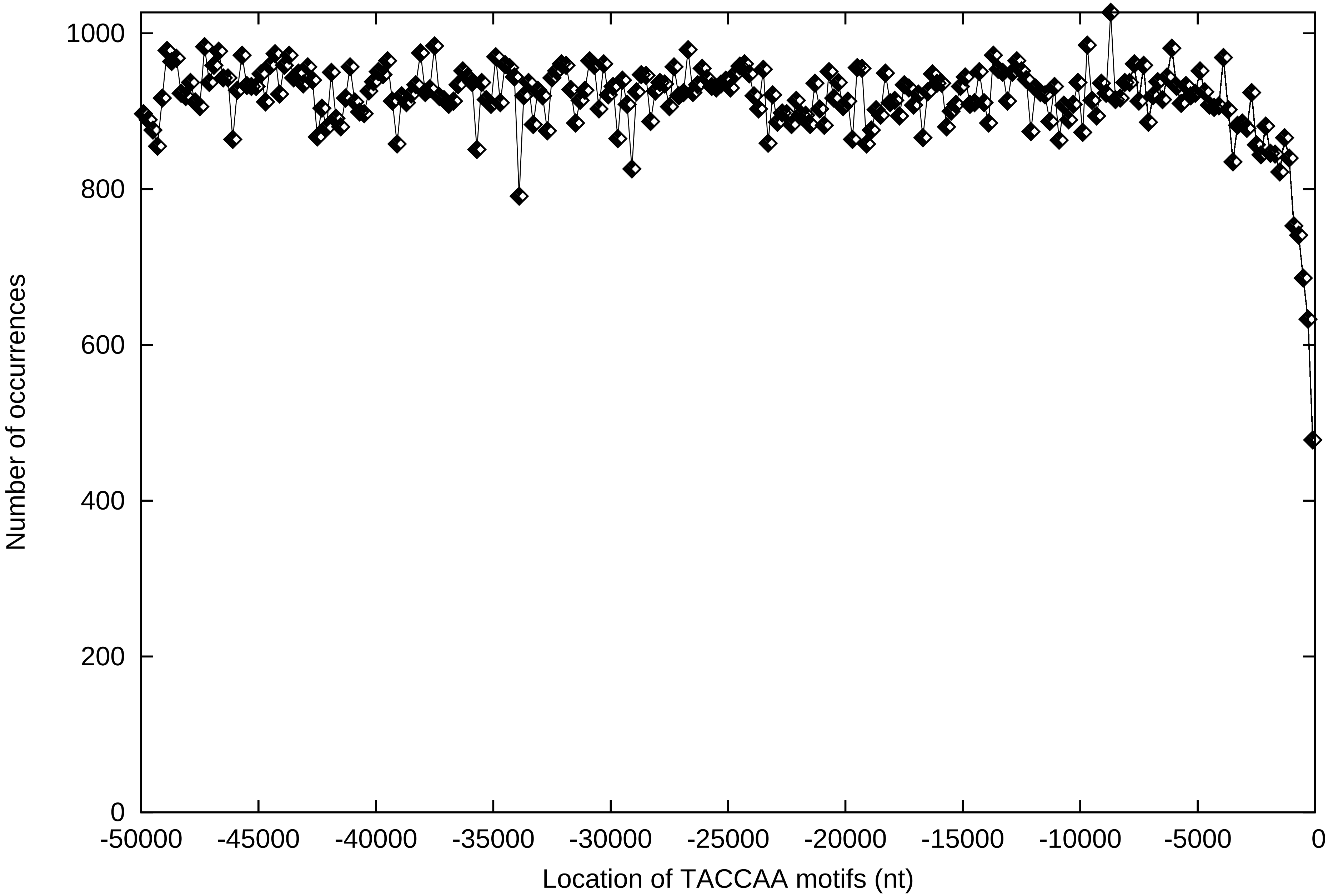

Number of occurrences
0
200
400
600
800
1000
-50000
-45000
-40000
-35000
-30000
-25000
-20000
-15000
-10000
-5000
0
Location of TACCAA motifs (nt)

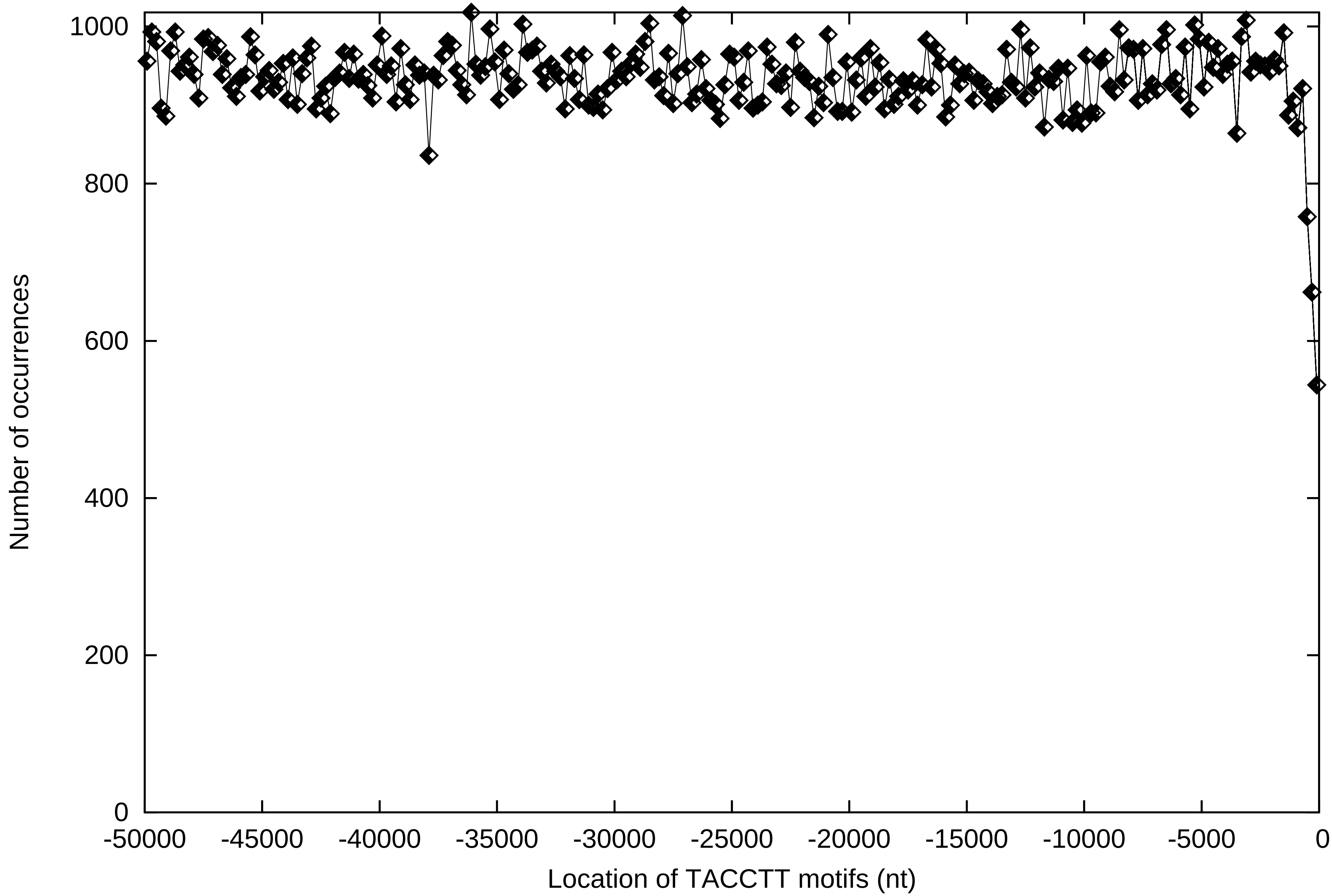

Number of occurrences
1000
800
600
400
200
0
-50000
-45000
-40000
-35000
-30000
-25000
-20000
-15000
-10000
-5000
0
Location of TACCTT motifs (nt)

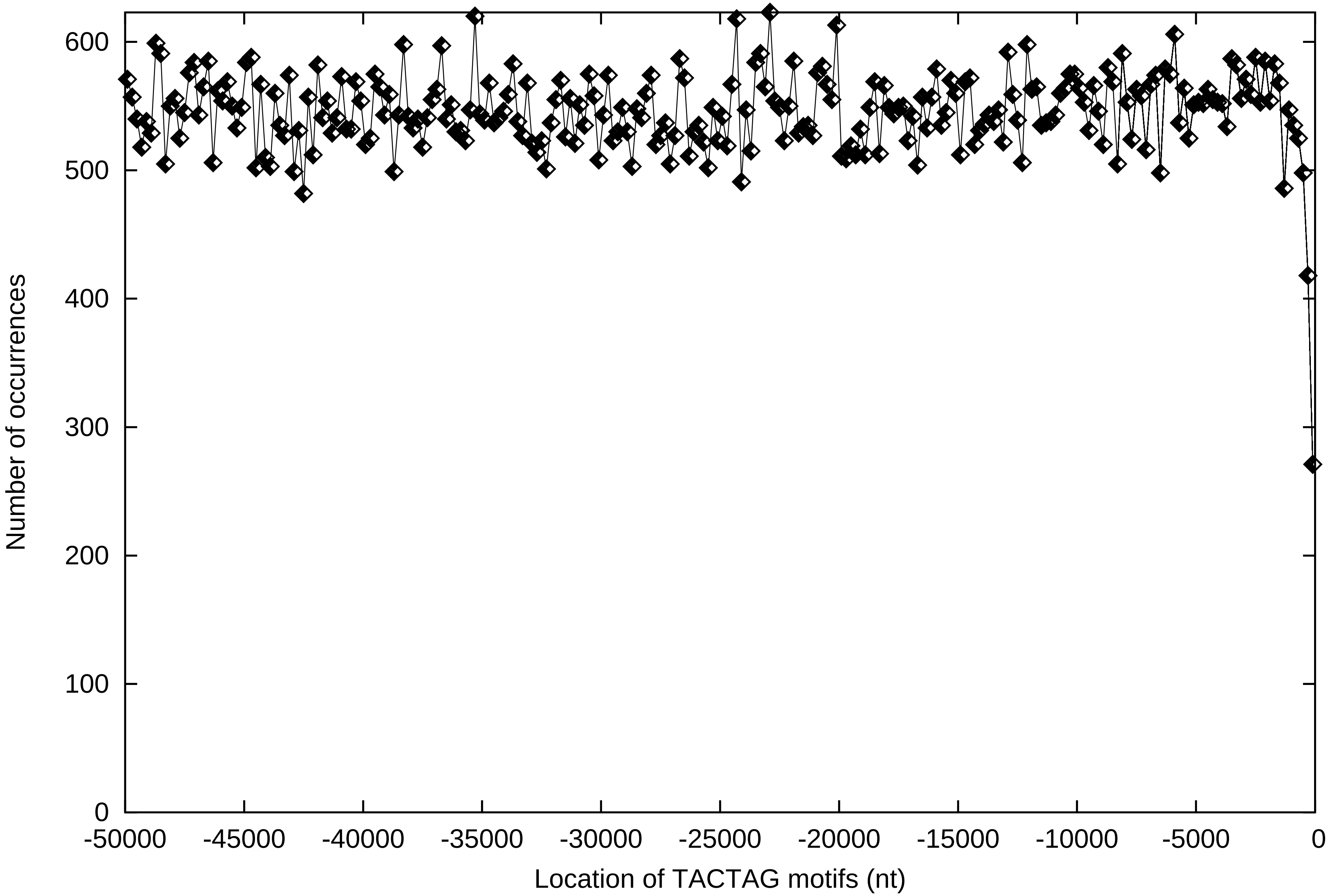

600
500
400
300
200
100
0
-50000
-45000
-40000
-35000
-30000
-25000
-20000
-15000
-10000
-5000
0
Number of occurrences
Location of TACTAG motifs (nt)

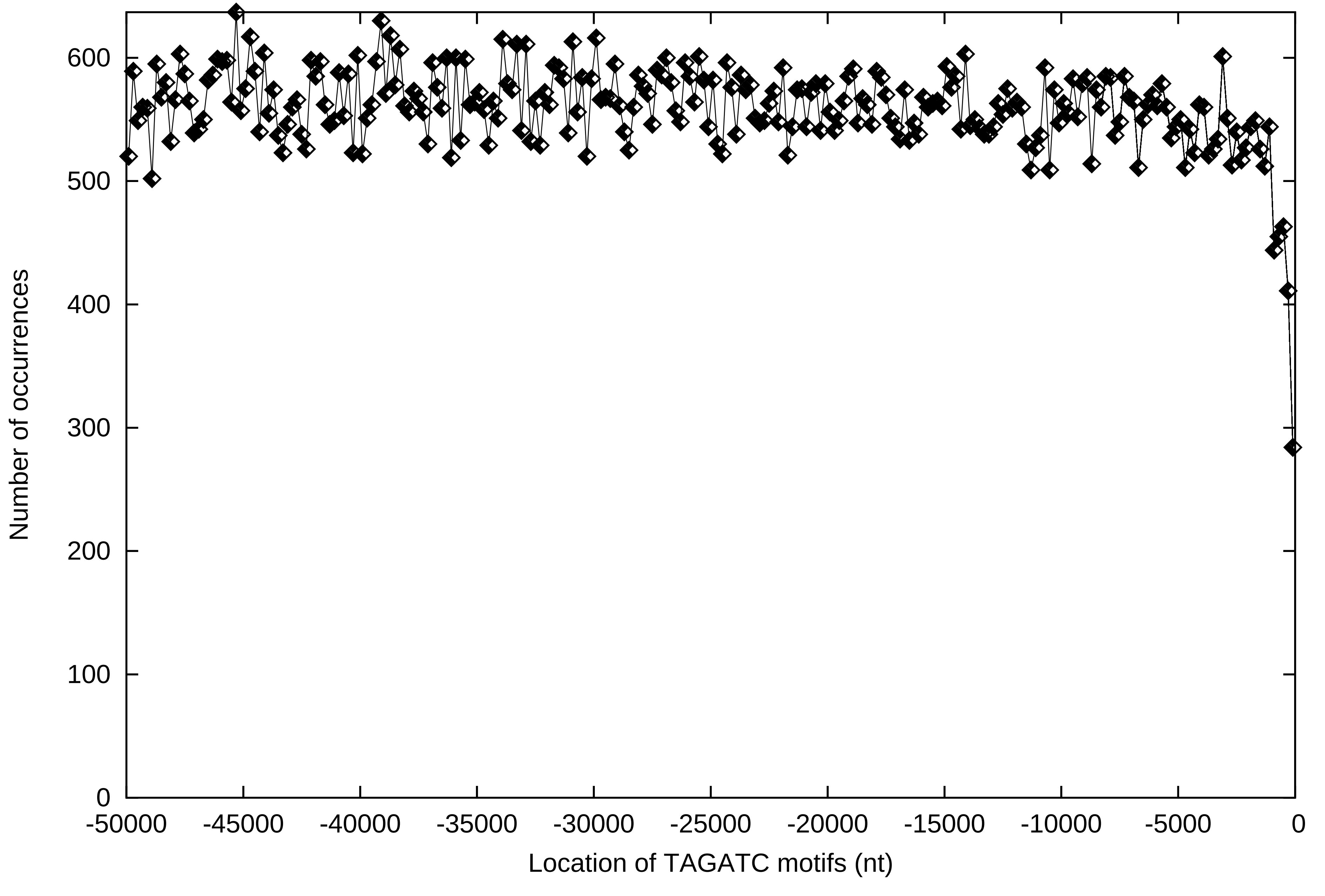
Number of occurrences
0
100
200
300
400
500
600
-50000
-45000
-40000
-35000
-30000
-25000
-20000
-15000
-10000
-5000
0
Location of TAGATC motifs (nt)

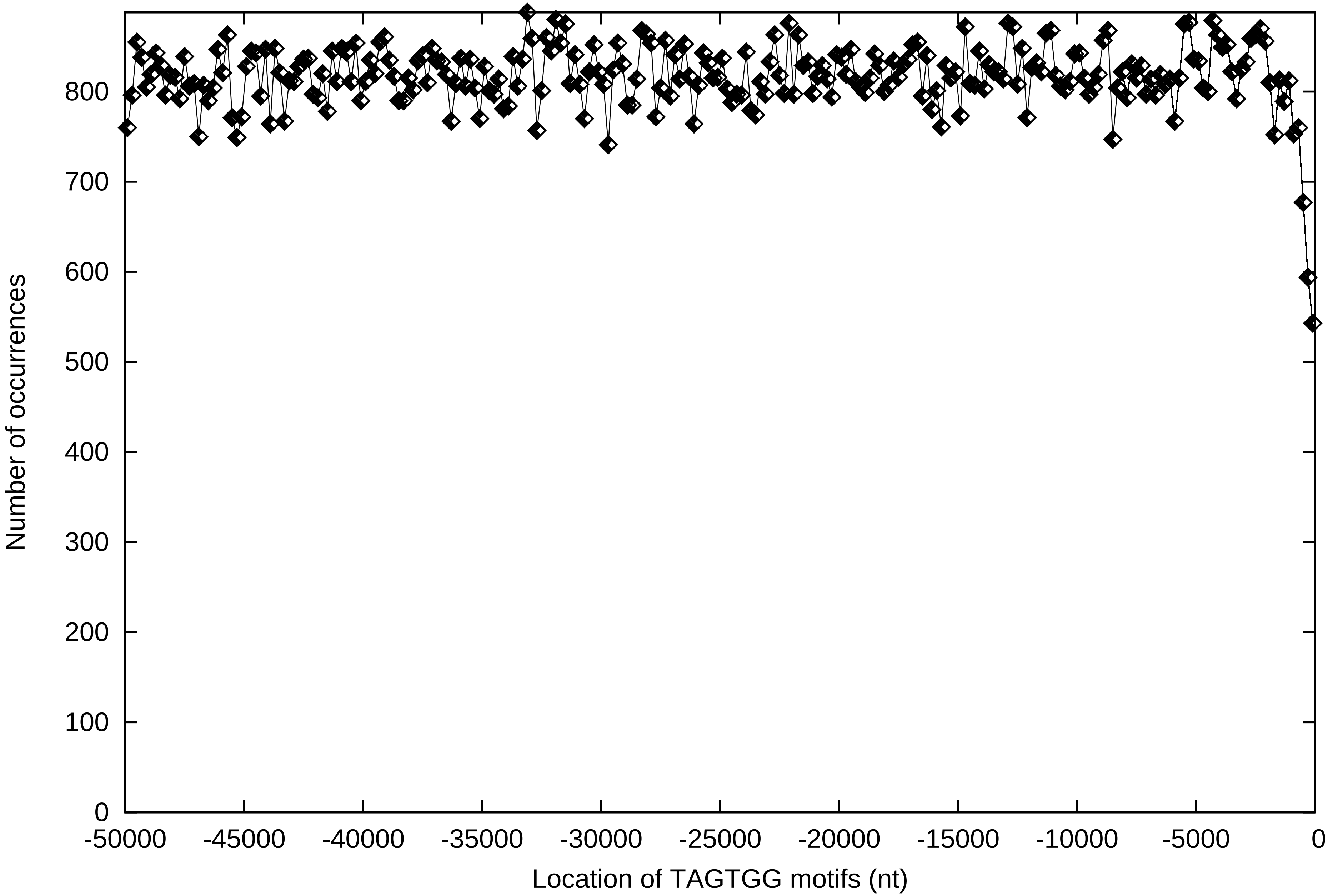

Number of occurrences
0
100
200
300
400
500
600
700
800
-50000
-45000
-40000
-35000
-30000
-25000
-20000
-15000
-10000
-5000
0
Location of TAGTGG motifs (nt)

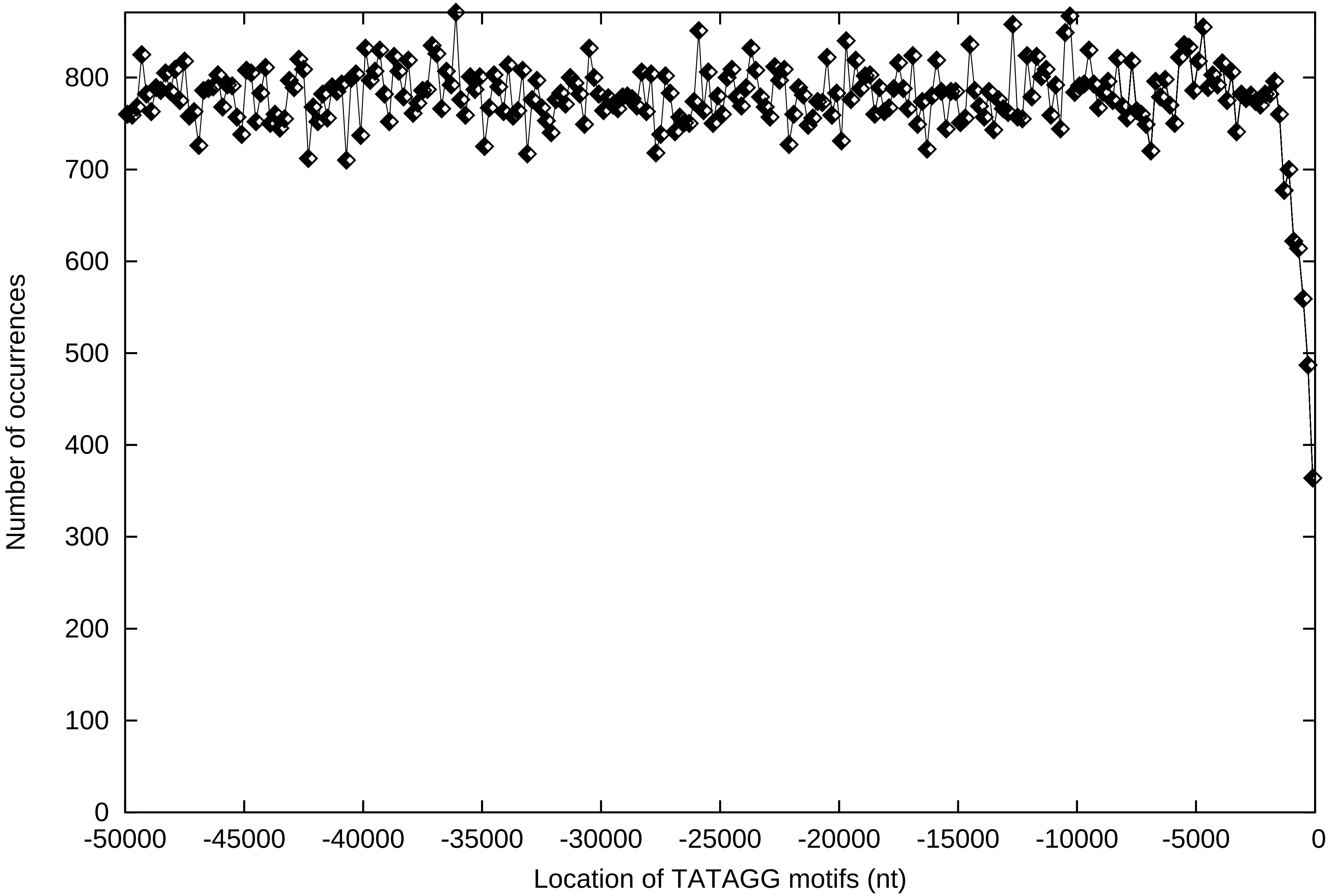

Number of occurrences
Location of TATAGG motifs (nt)
0
100
200
300
400
500
600
700
800
-50000
-45000
-40000
-35000
-30000
-25000
-20000
-15000
-10000
-5000
0

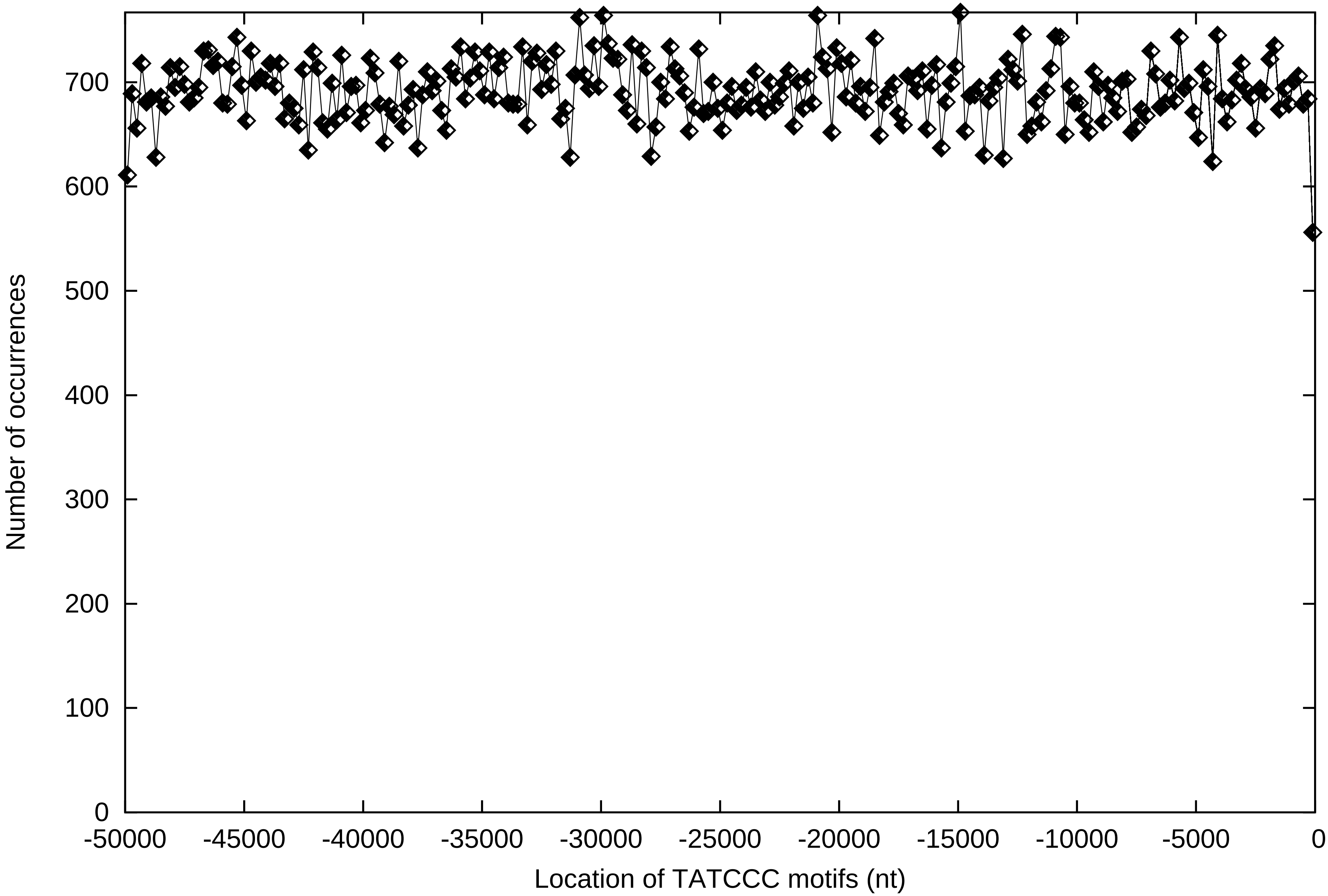
Number of occurrences
0
100
200
300
400
500
600
700
-50000
-45000
-40000
-35000
-30000
-25000
-20000
-15000
-10000
-5000
0
Location of TATCCC motifs (nt)

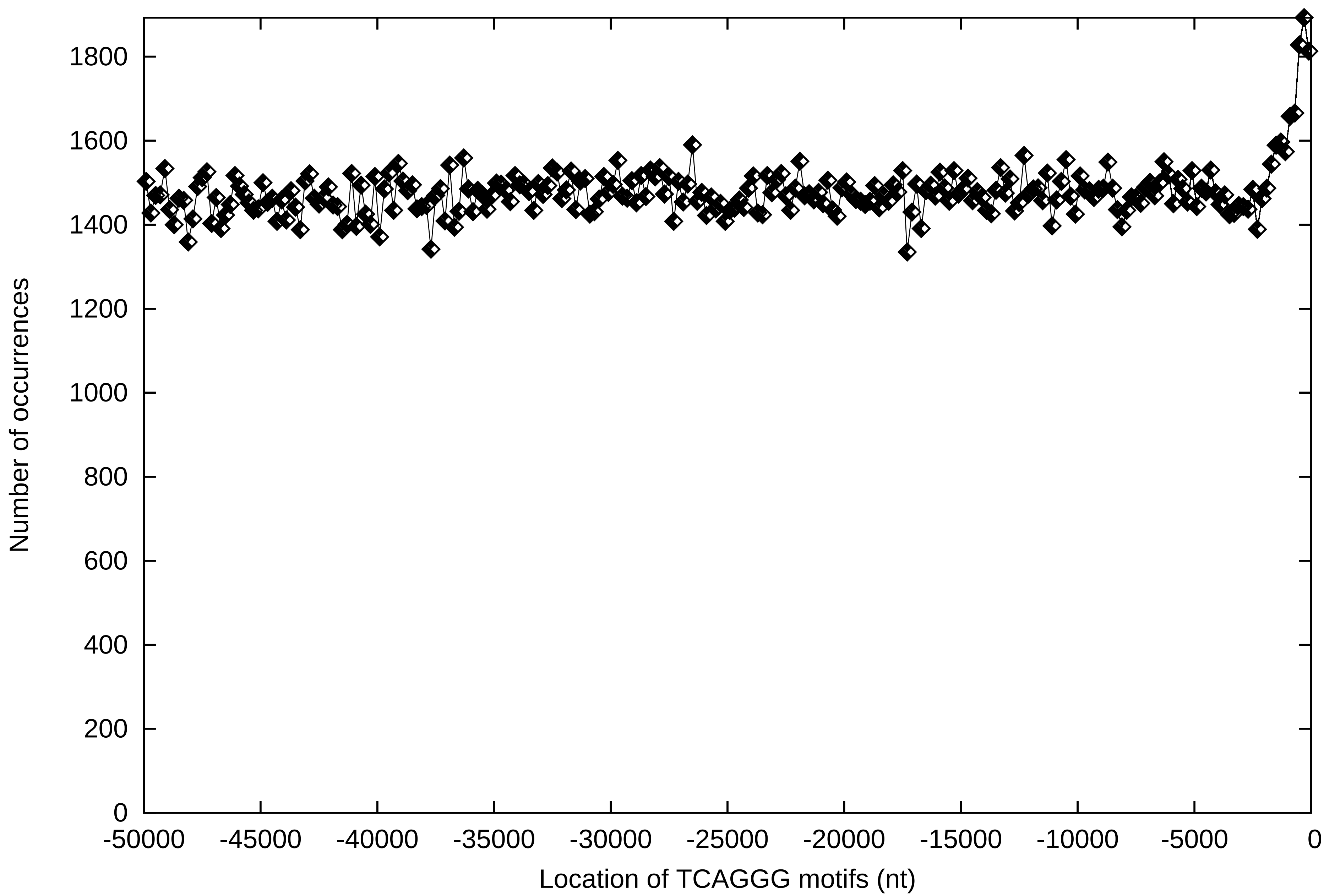
Number of occurrences
Location of TCAGGG motifs (nt)
0
200
400
600
800
1000
1200
1400
1600
1800
-50000
-45000
-40000
-35000
-30000
-25000
-20000
-15000
-10000
-5000
0

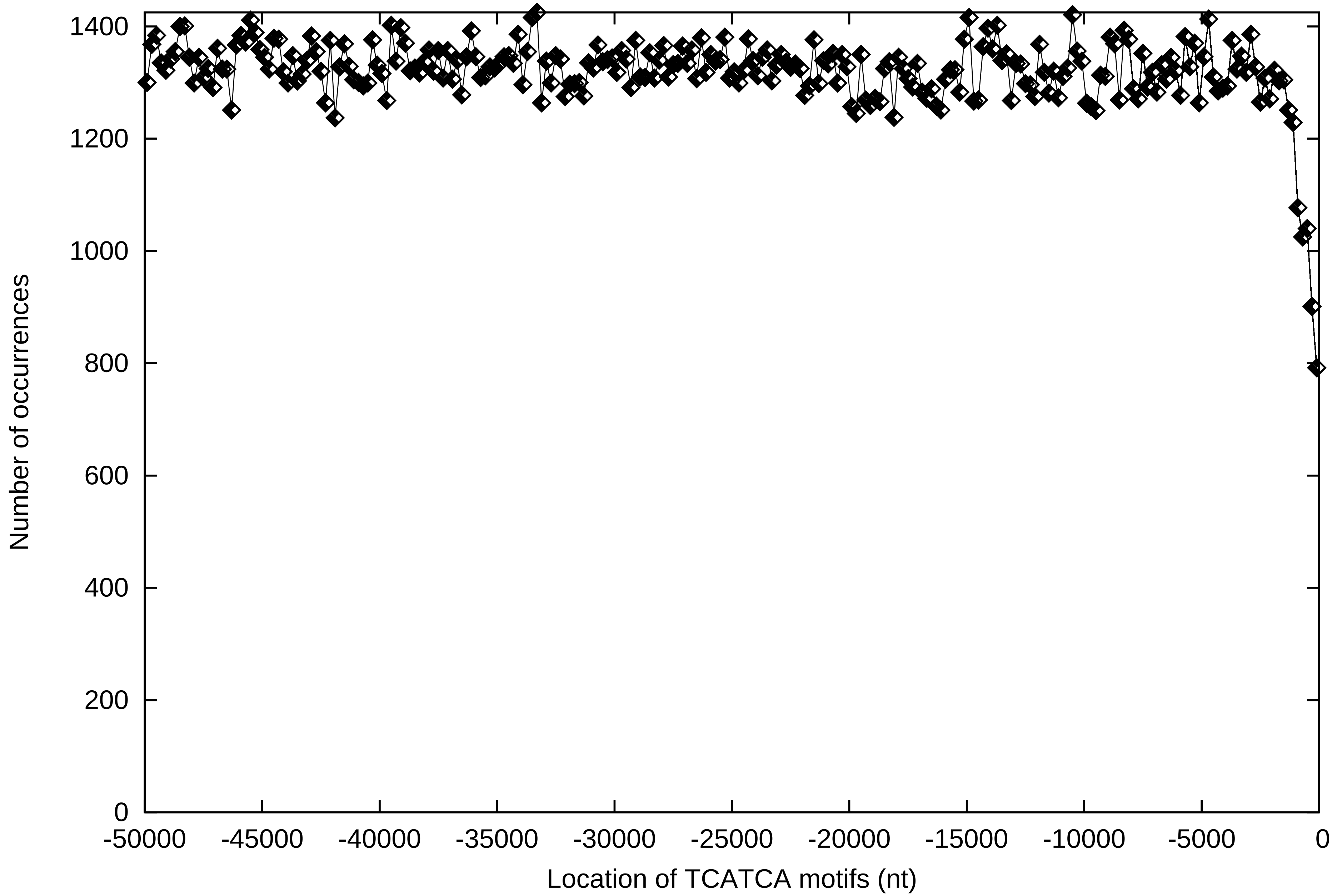

Number of occurrences
Location of TCATCA motifs (nt)
0
200
400
600
800
1000
1200
1400
-50000
-45000
-40000
-35000
-30000
-25000
-20000
-15000
-10000
-5000
0

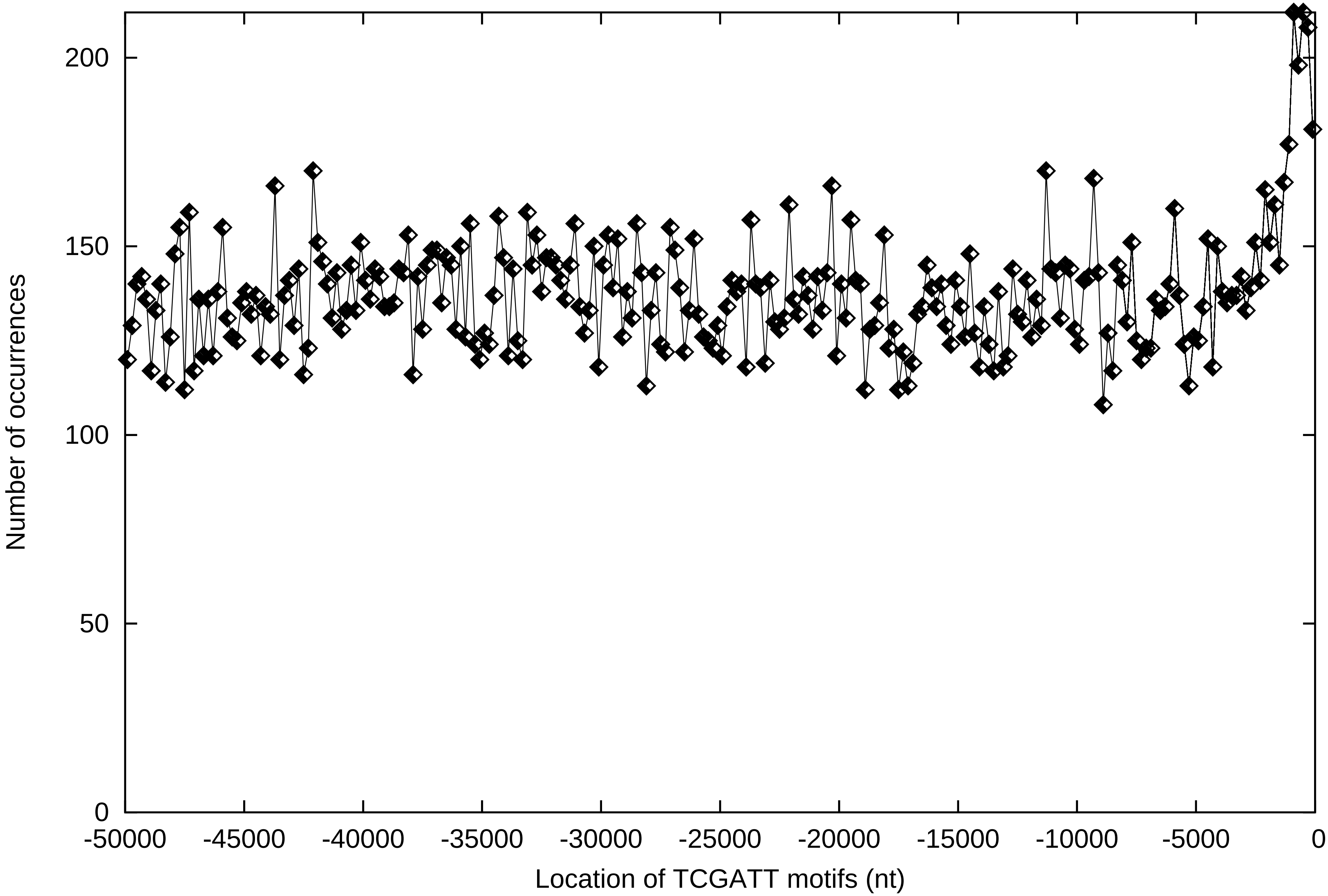
Number of occurrences
Location of TCGATT motifs (nt)
0
50
100
150
200
-50000
-45000
-40000
-35000
-30000
-25000
-20000
-15000
-10000
-5000
0

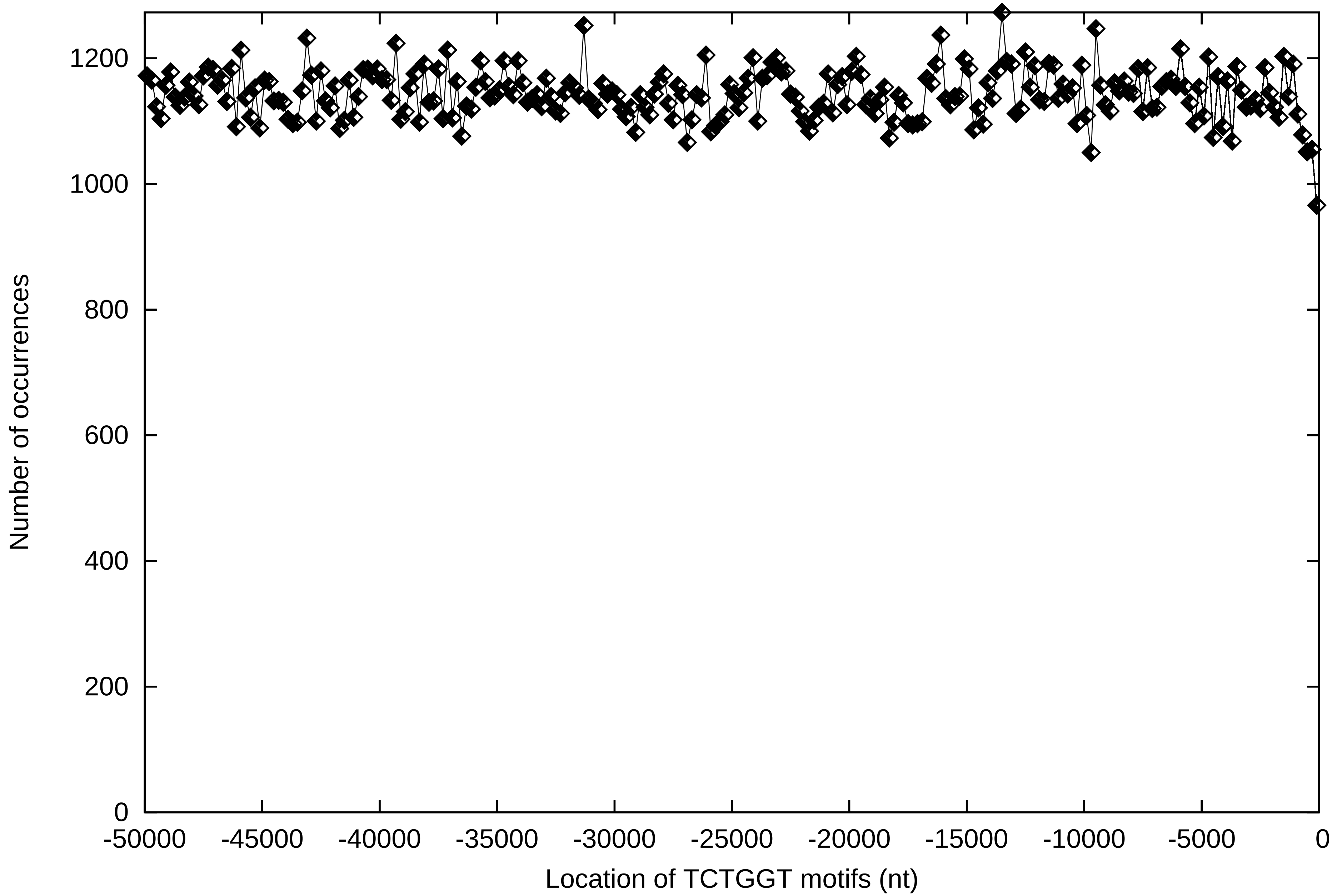

Number of occurrences
0
200
400
600
800
1000
1200
-50000
-45000
-40000
-35000
-30000
-25000
-20000
-15000
-10000
-5000
0
Location of TCTGGT motifs (nt)

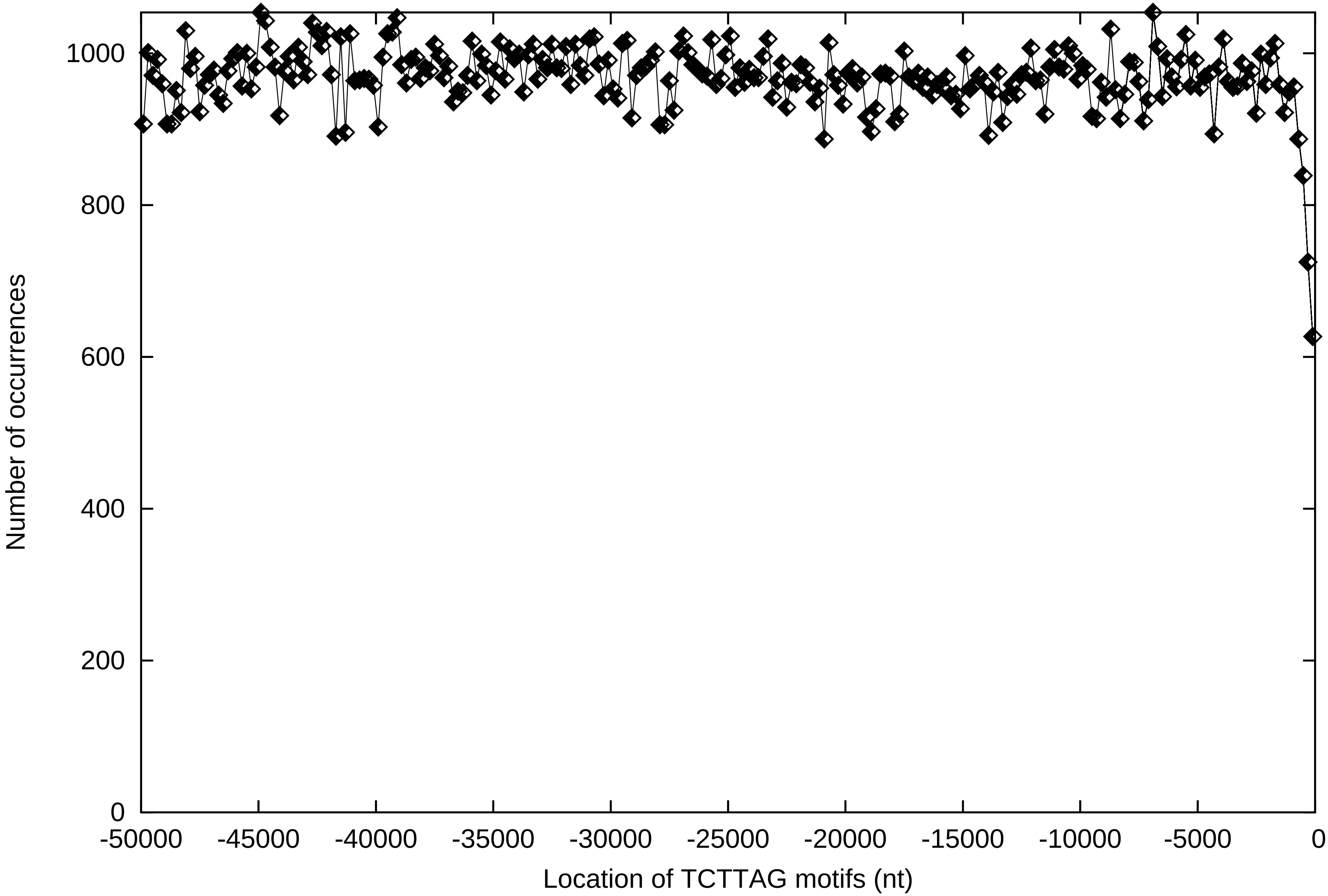

Number of occurrences
0
200
400
600
800
1000
-50000
-45000
-40000
-35000
-30000
-25000
-20000
-15000
-10000
-5000
0
Location of TCTTAG motifs (nt)

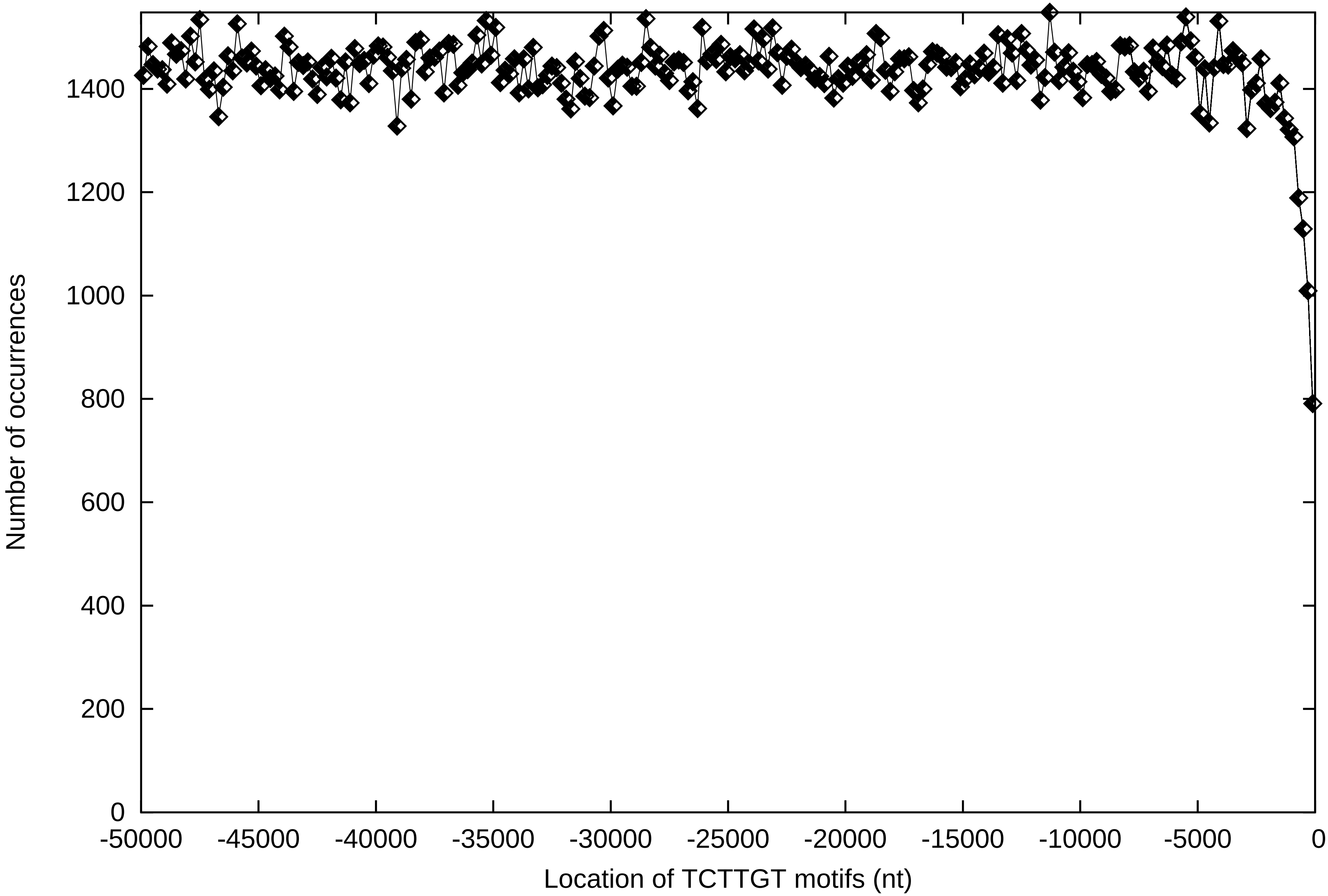

Number of occurrences
0
200
400
600
800
1000
1200
1400
-50000
-45000
-40000
-35000
-30000
-25000
-20000
-15000
-10000
-5000
0
Location of TCTTGT motifs (nt)

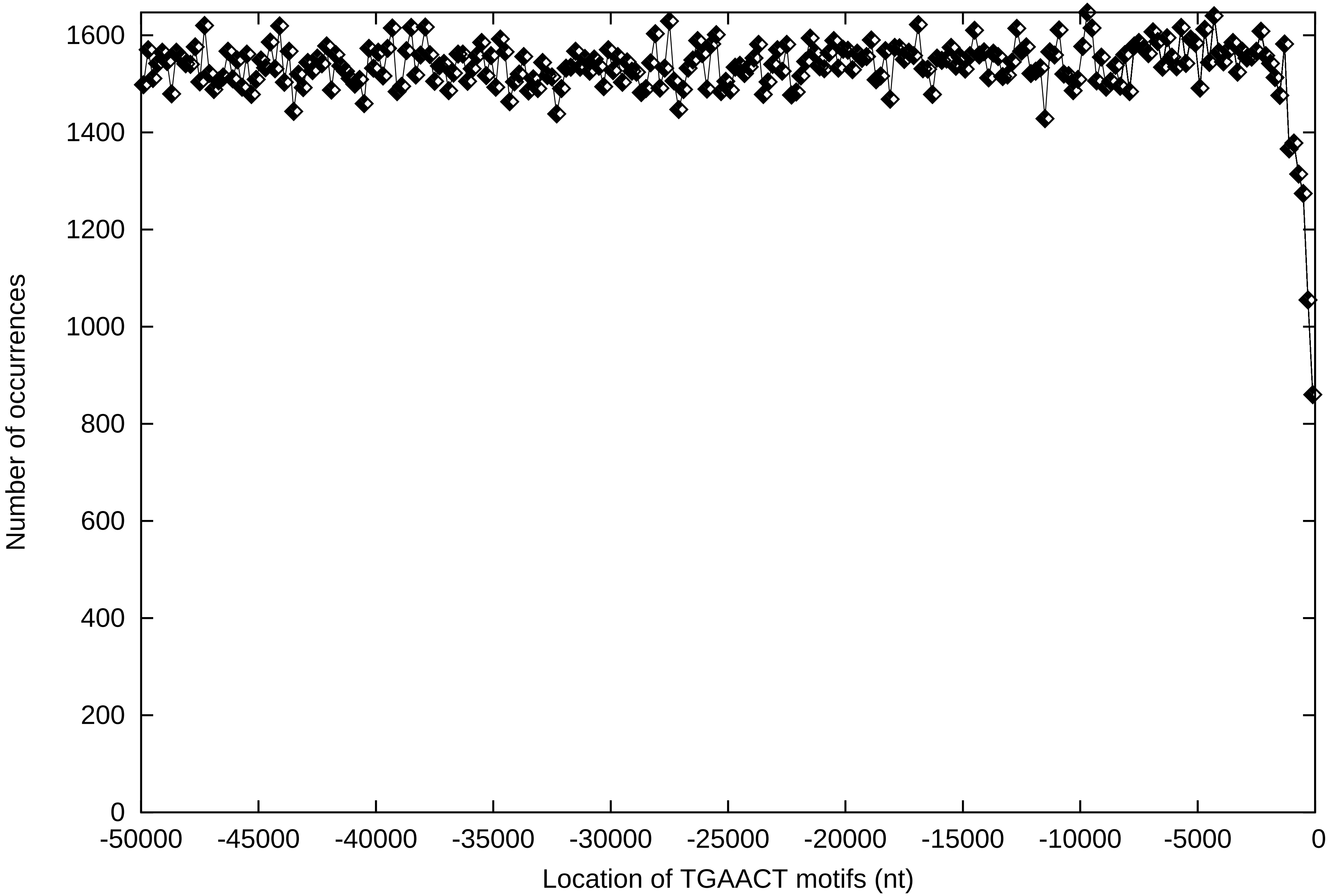

Number of occurrences
0
200
400
600
800
1000
1200
1400
1600
-50000
-45000
-40000
-35000
-30000
-25000
-20000
-15000
-10000
-5000
0
Location of TGAACT motifs (nt)

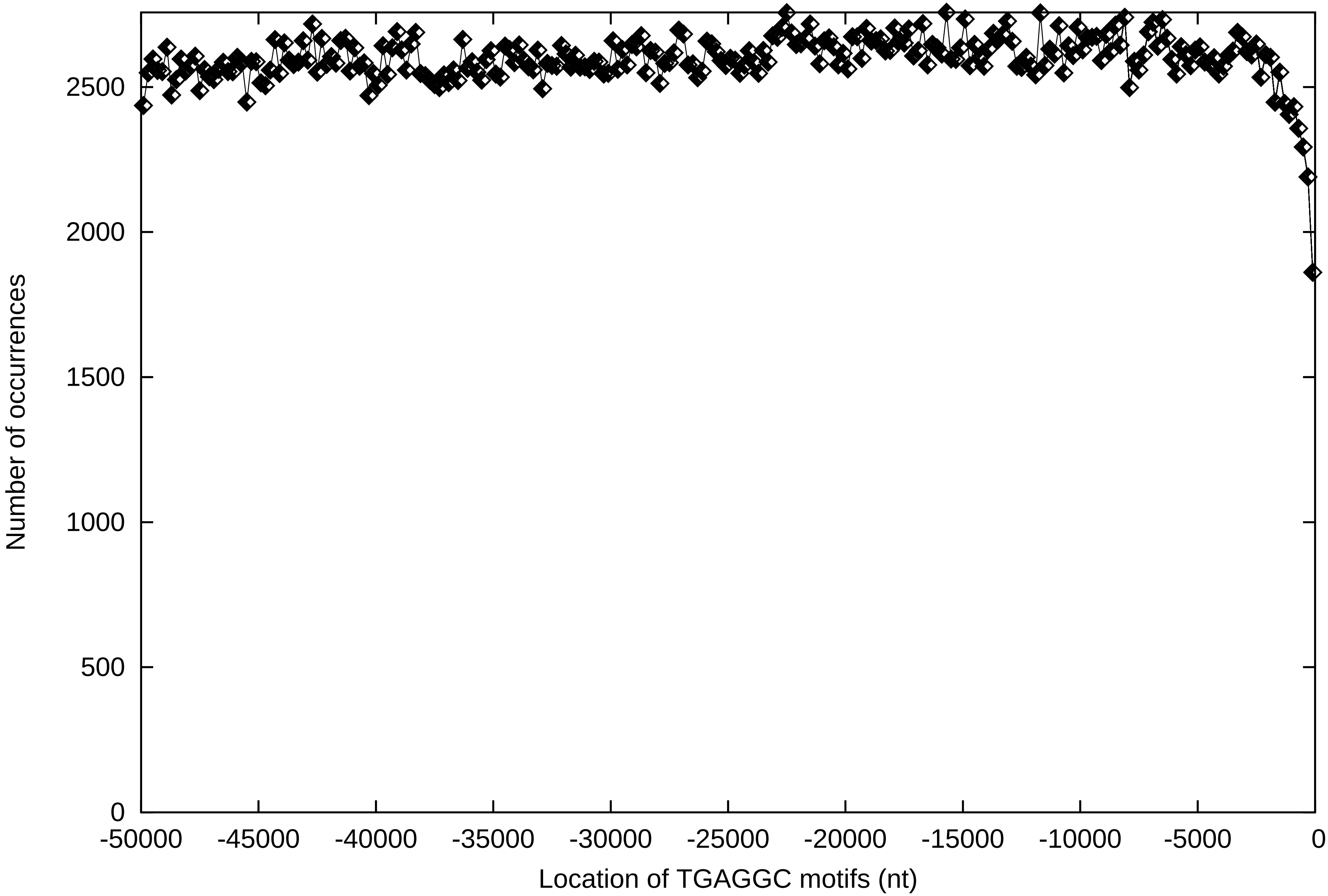
Number of occurrences
Location of TGAGGC motifs (nt)
0
500
1000
1500
2000
2500
-50000
-45000
-40000
-35000
-30000
-25000
-20000
-15000
-10000
-5000
0

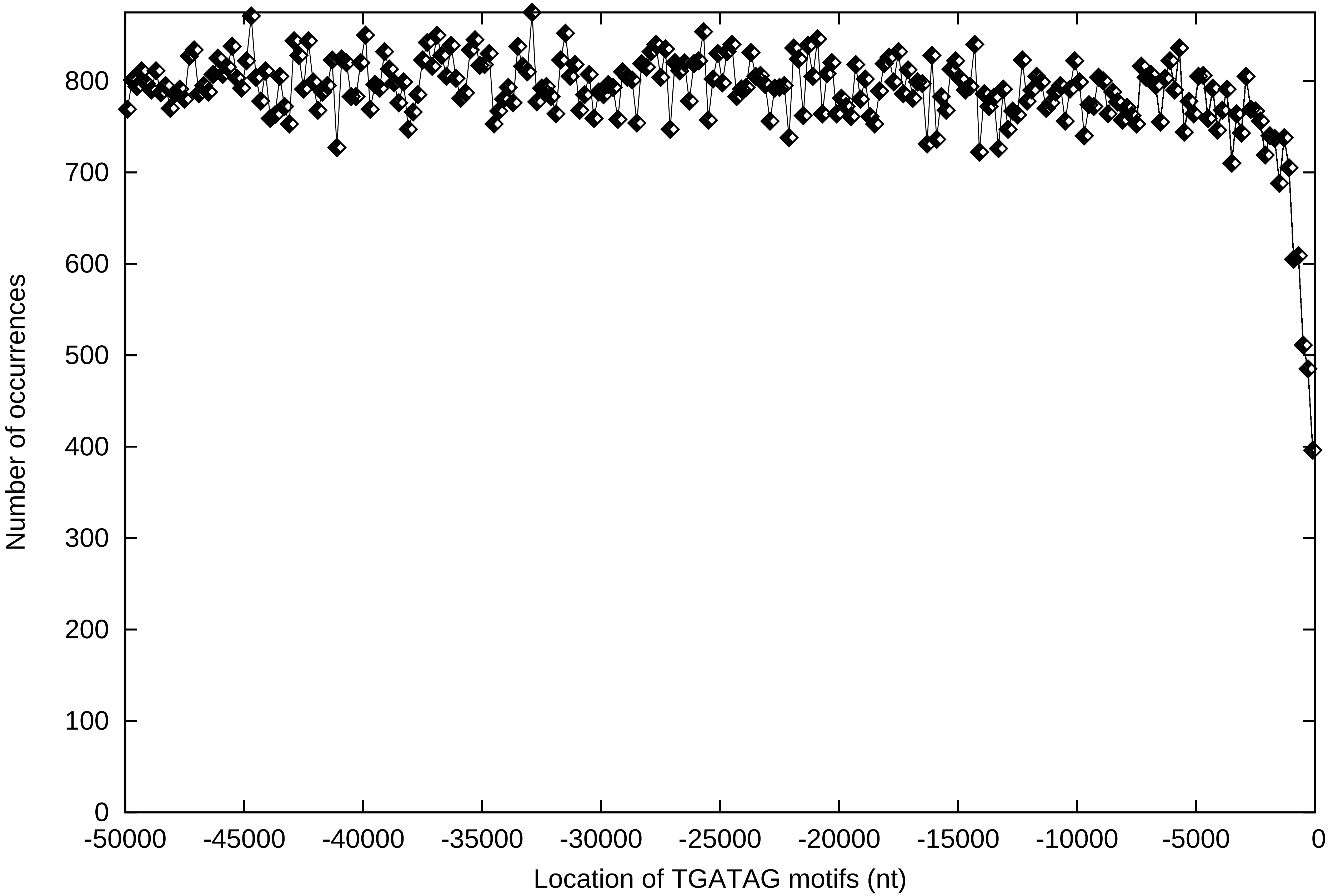

Number of occurrences
Location of TGATAG motifs (nt)
0
100
200
300
400
500
600
700
800
-50000
-45000
-40000
-35000
-30000
-25000
-20000
-15000
-10000
-5000
0

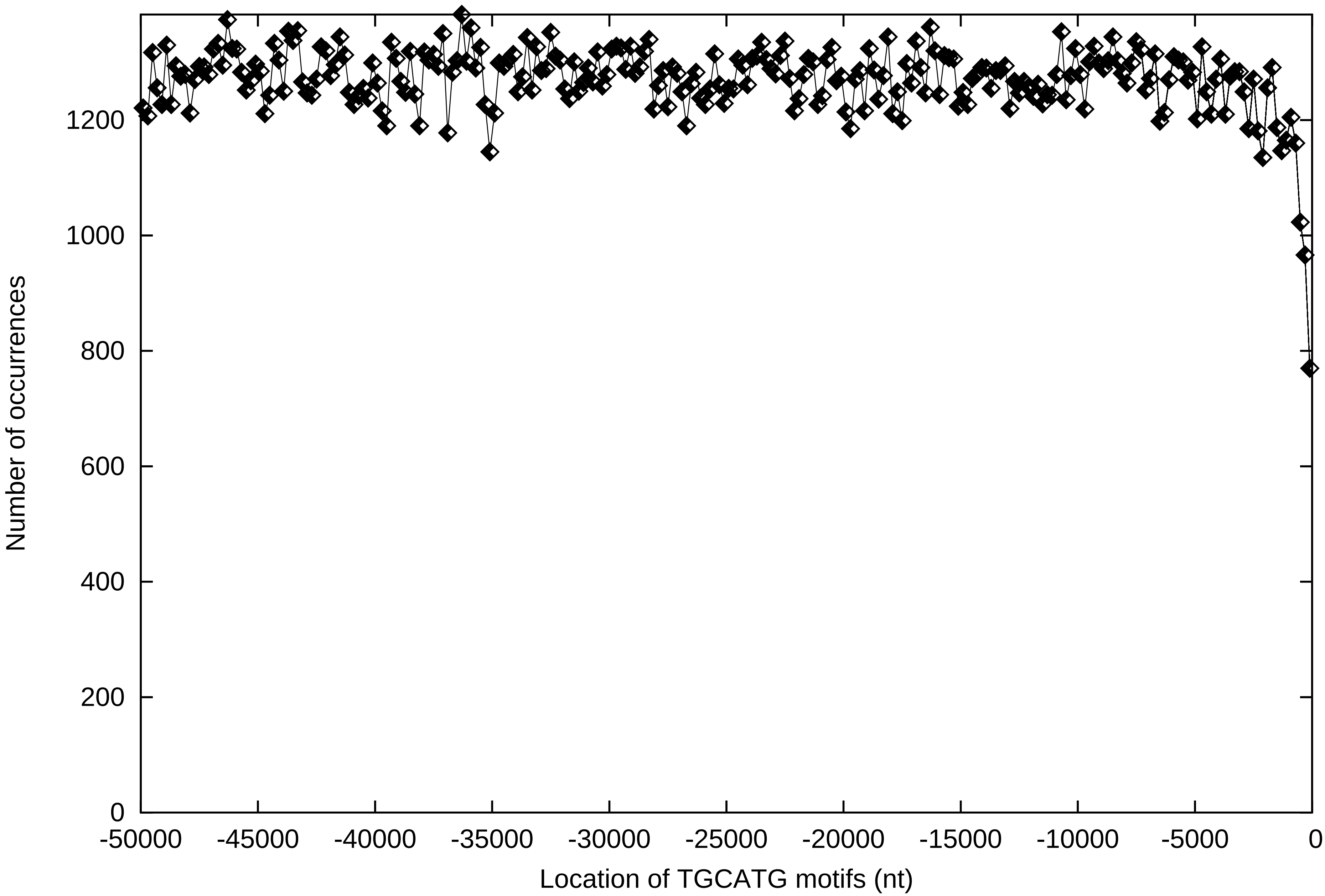

Number of occurrences
Location of TGCATG motifs (nt)
0
200
400
600
800
1000
1200
-50000
-45000
-40000
-35000
-30000
-25000
-20000
-15000
-10000
-5000
0

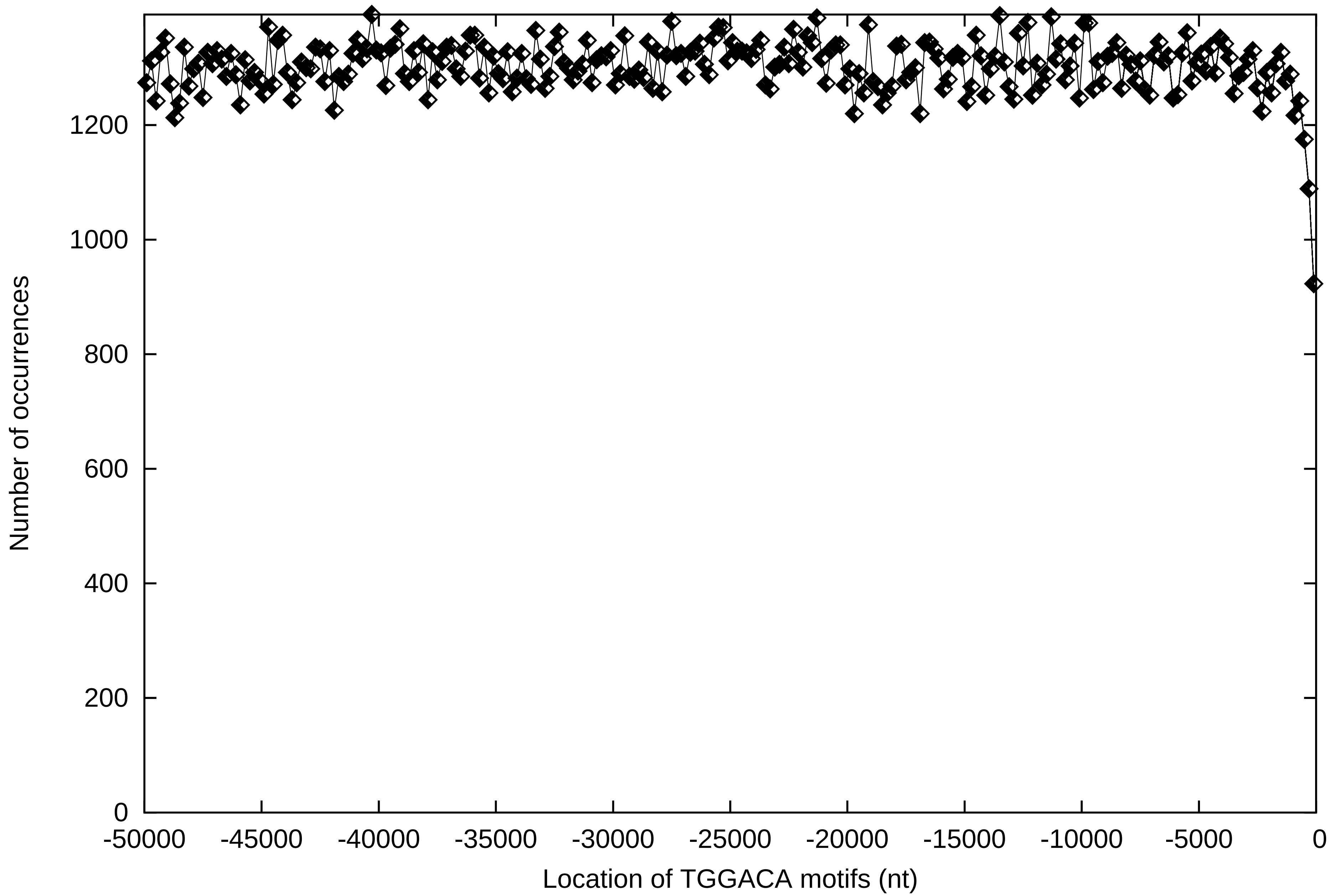
Number of occurrences
Location of TGGACA motifs (nt)
0
200
400
600
800
1000
1200
-50000
-45000
-40000
-35000
-30000
-25000
-20000
-15000
-10000
-5000
0

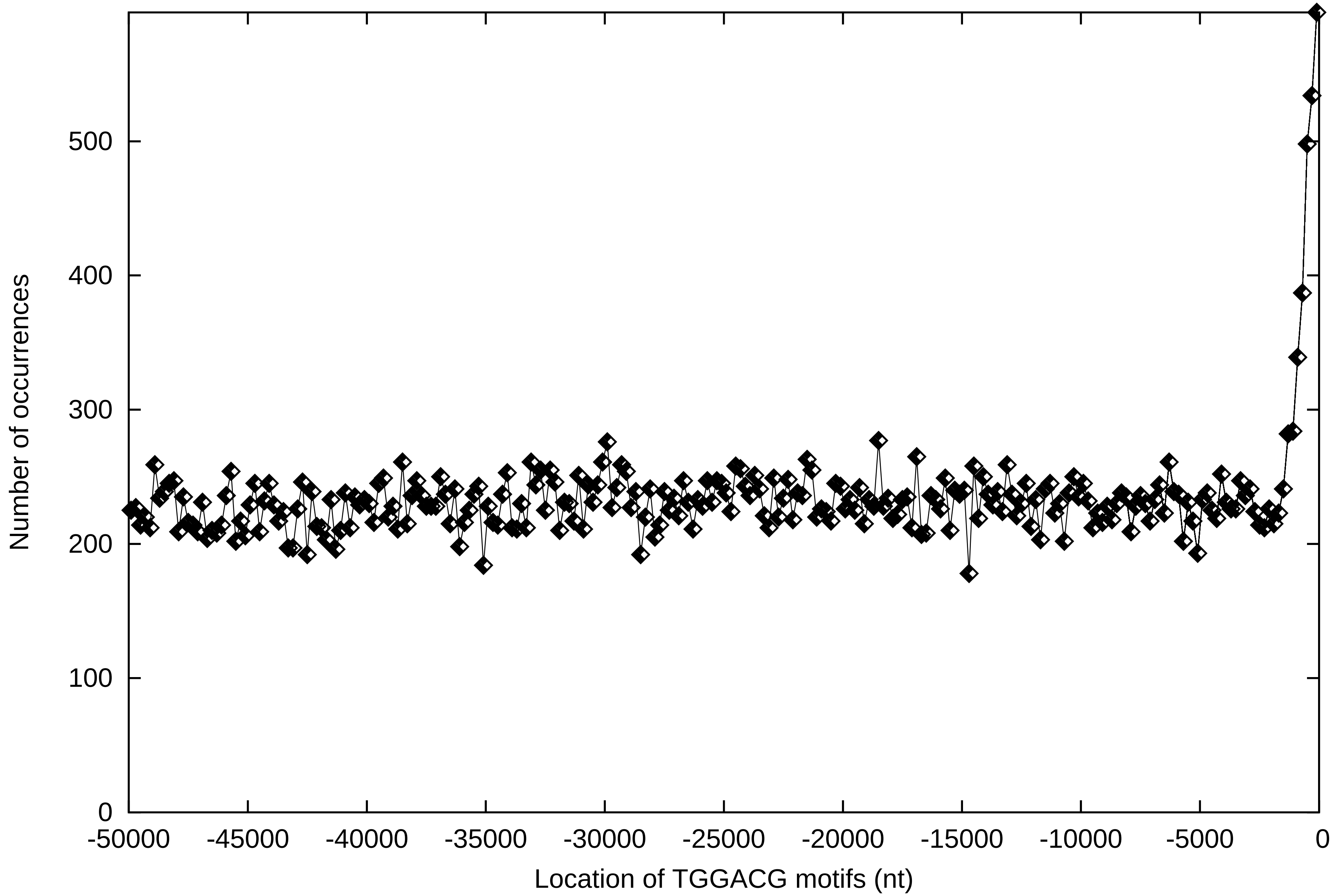

Number of occurrences
0
100
200
300
400
500
-50000
-45000
-40000
-35000
-30000
-25000
-20000
-15000
-10000
-5000
0
Location of TGGACG motifs (nt)

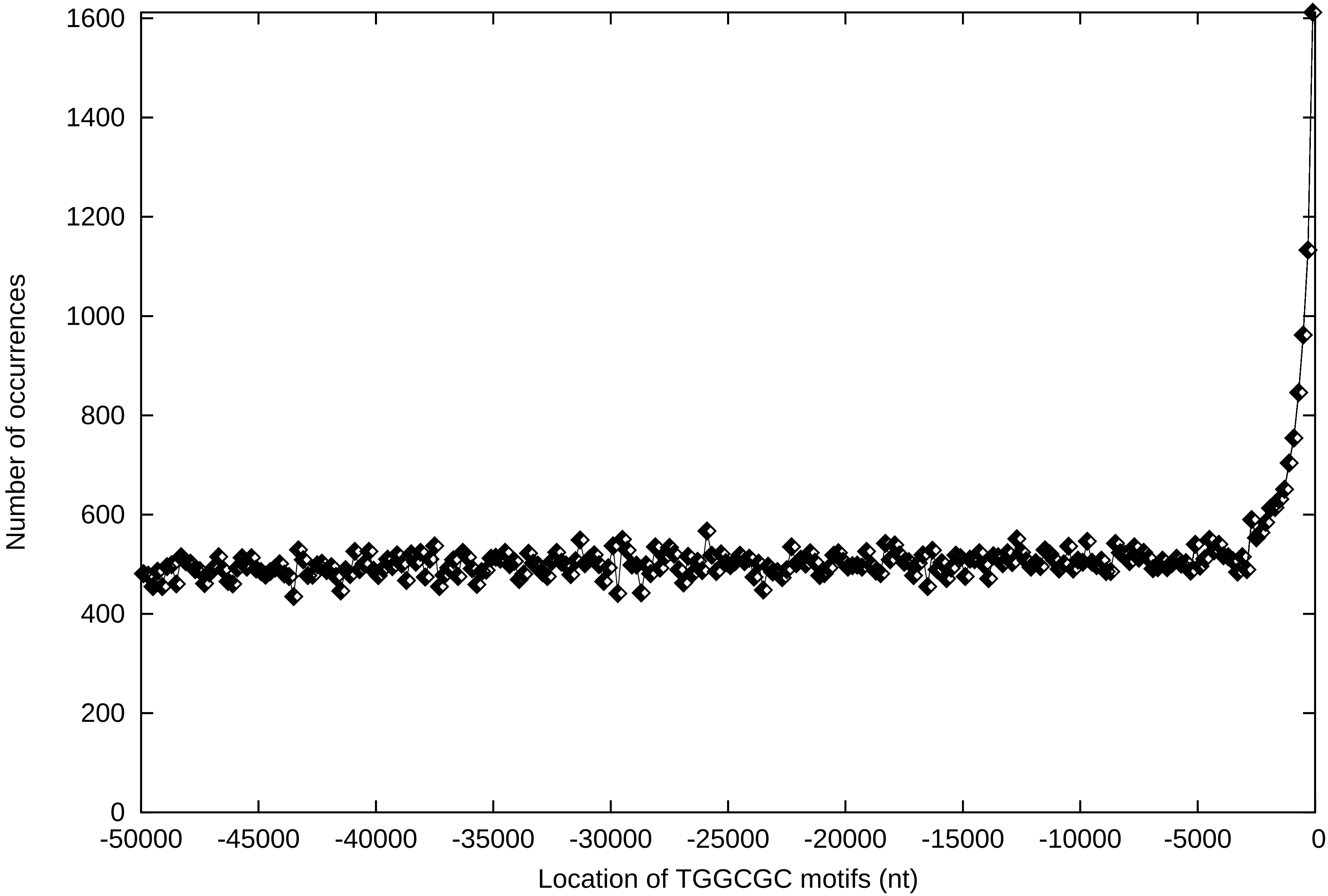

1600
1400
1200
1000
800
600
400
200
0
Number of occurrences
-50000
-45000
-40000
-35000
-30000
-25000
-20000
-15000
-10000
-5000
0
Location of TGGCGC motifs (nt)

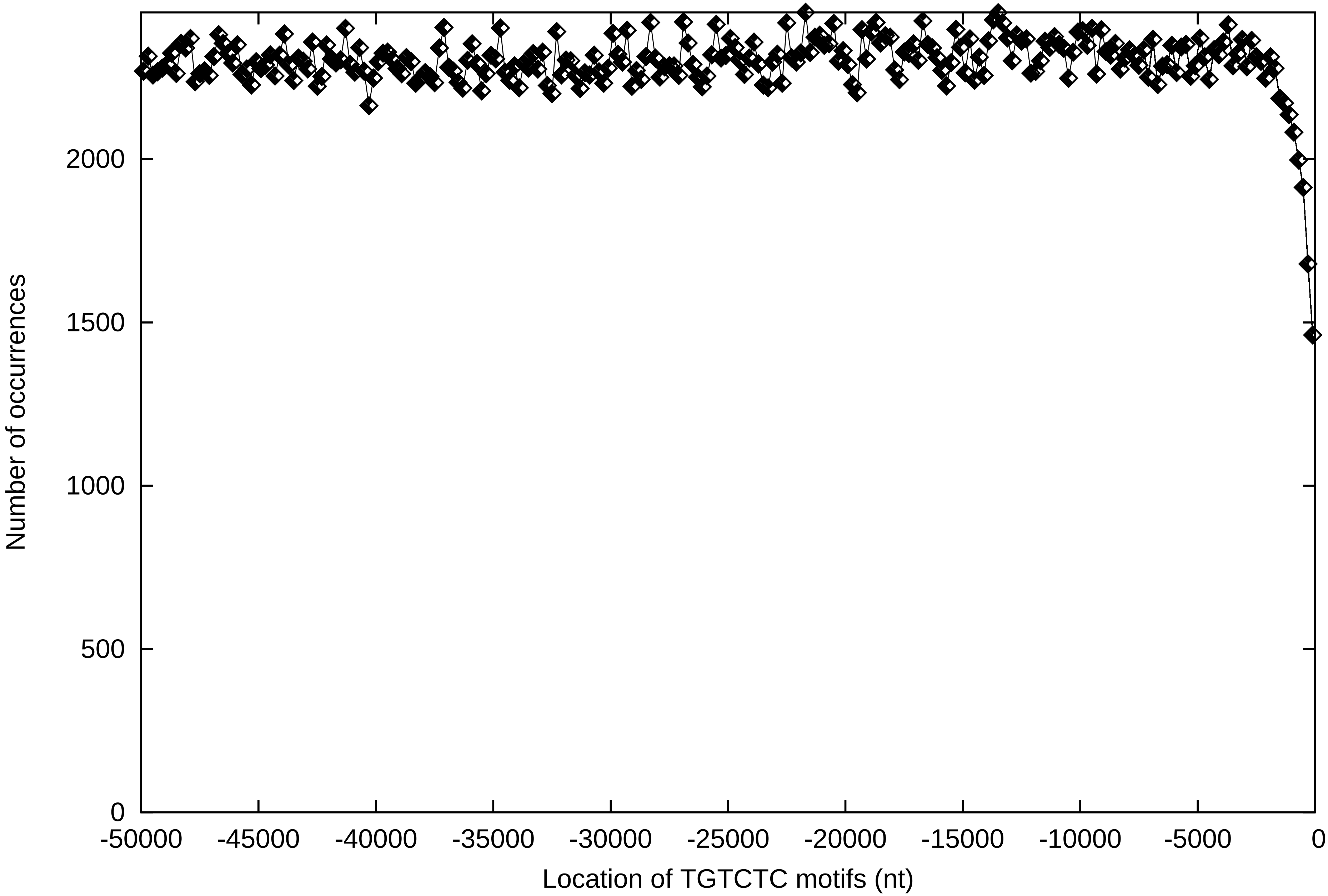
Number of occurrences
0
500
1000
1500
2000
-50000
-45000
-40000
-35000
-30000
-25000
-20000
-15000
-10000
-5000
0
Location of TGTCTC motifs (nt)

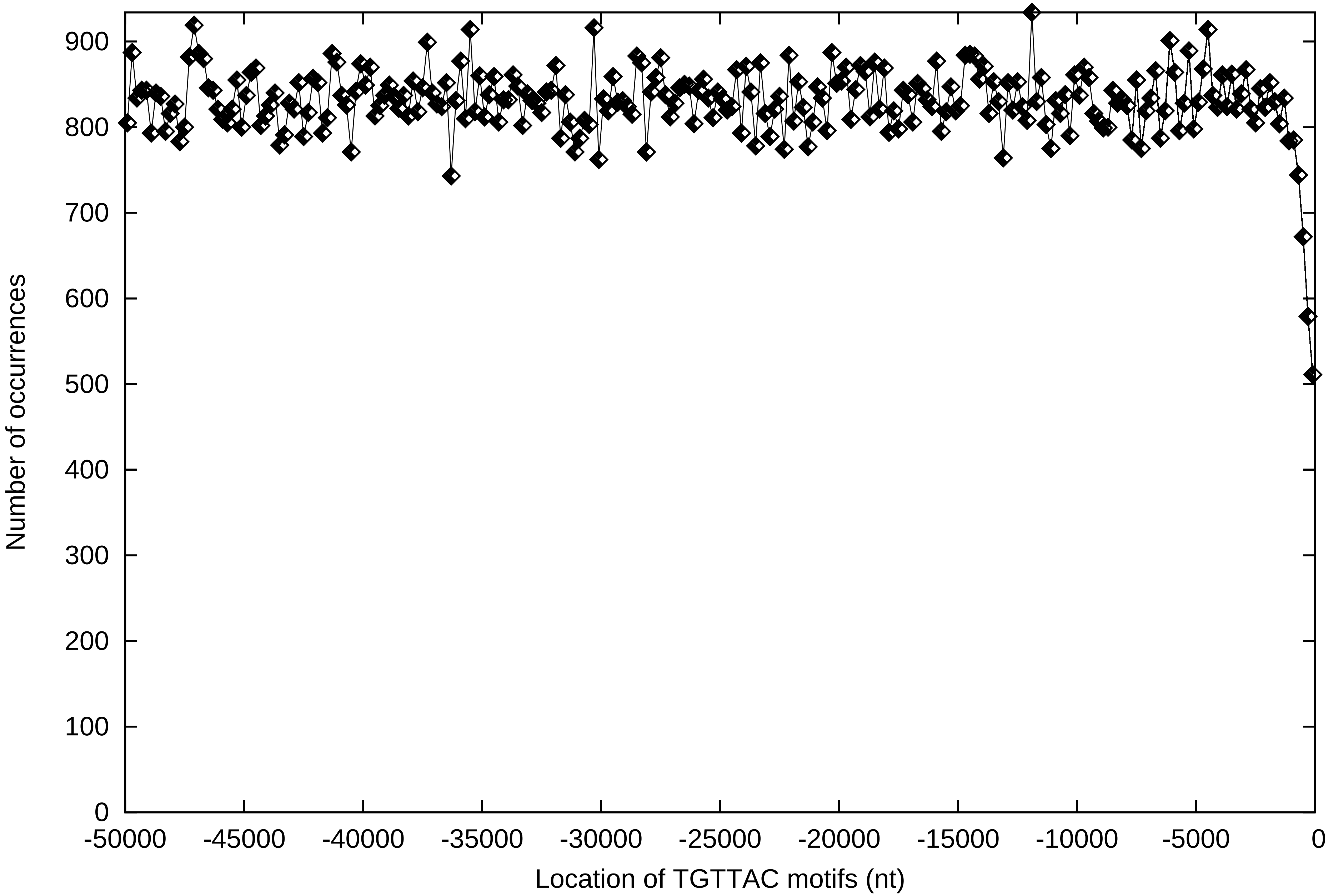

Number of occurrences
Location of TGTTAC motifs (nt)
0
100
200
300
400
500
600
700
800
900
-50000
-45000
-40000
-35000
-30000
-25000
-20000
-15000
-10000
-5000
0

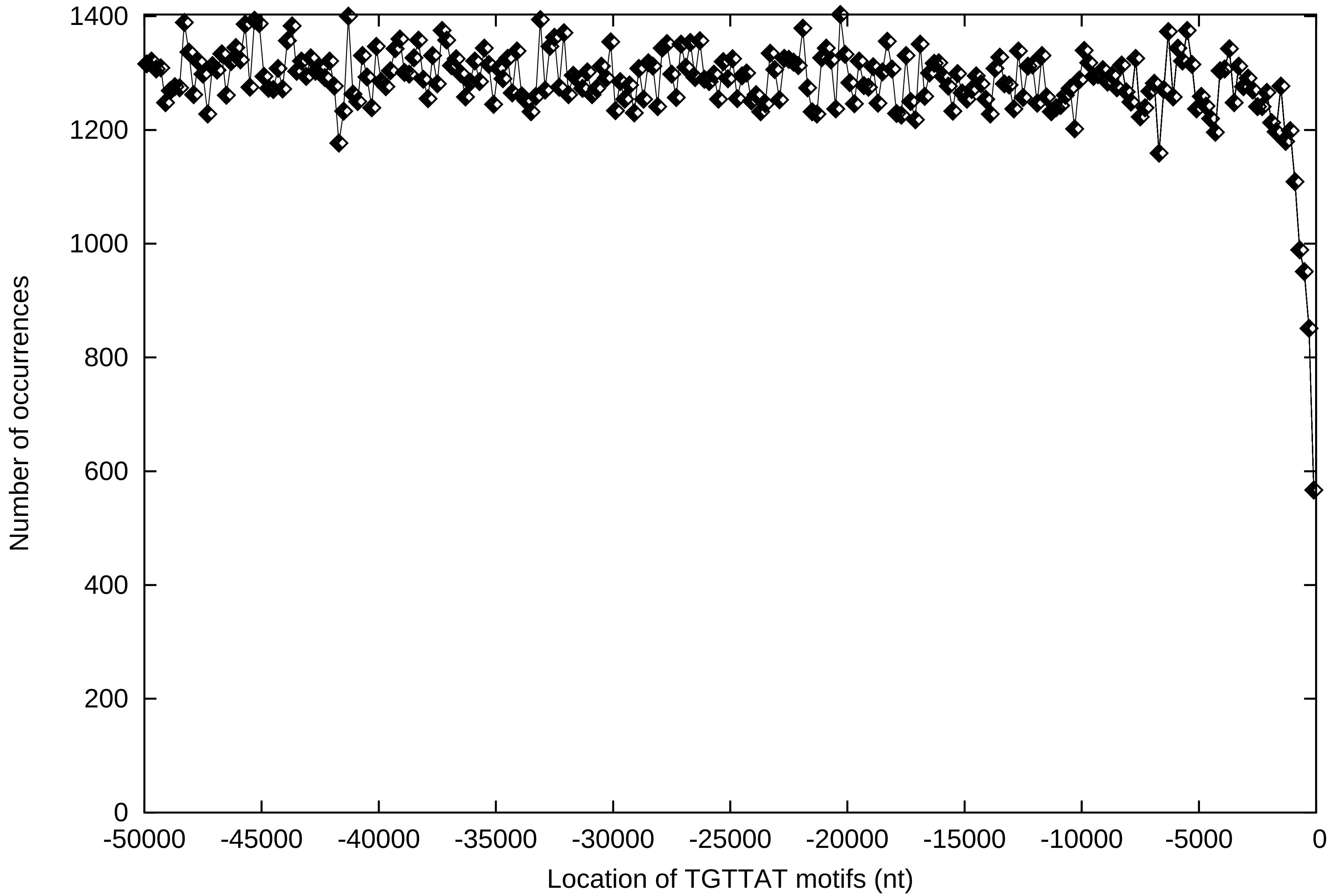

Number of occurrences
1400
1200
1000
800
600
400
200
0
-50000
-45000
-40000
-35000
-30000
-25000
-20000
-15000
-10000
-5000
0
Location of TGTTAT motifs (nt)

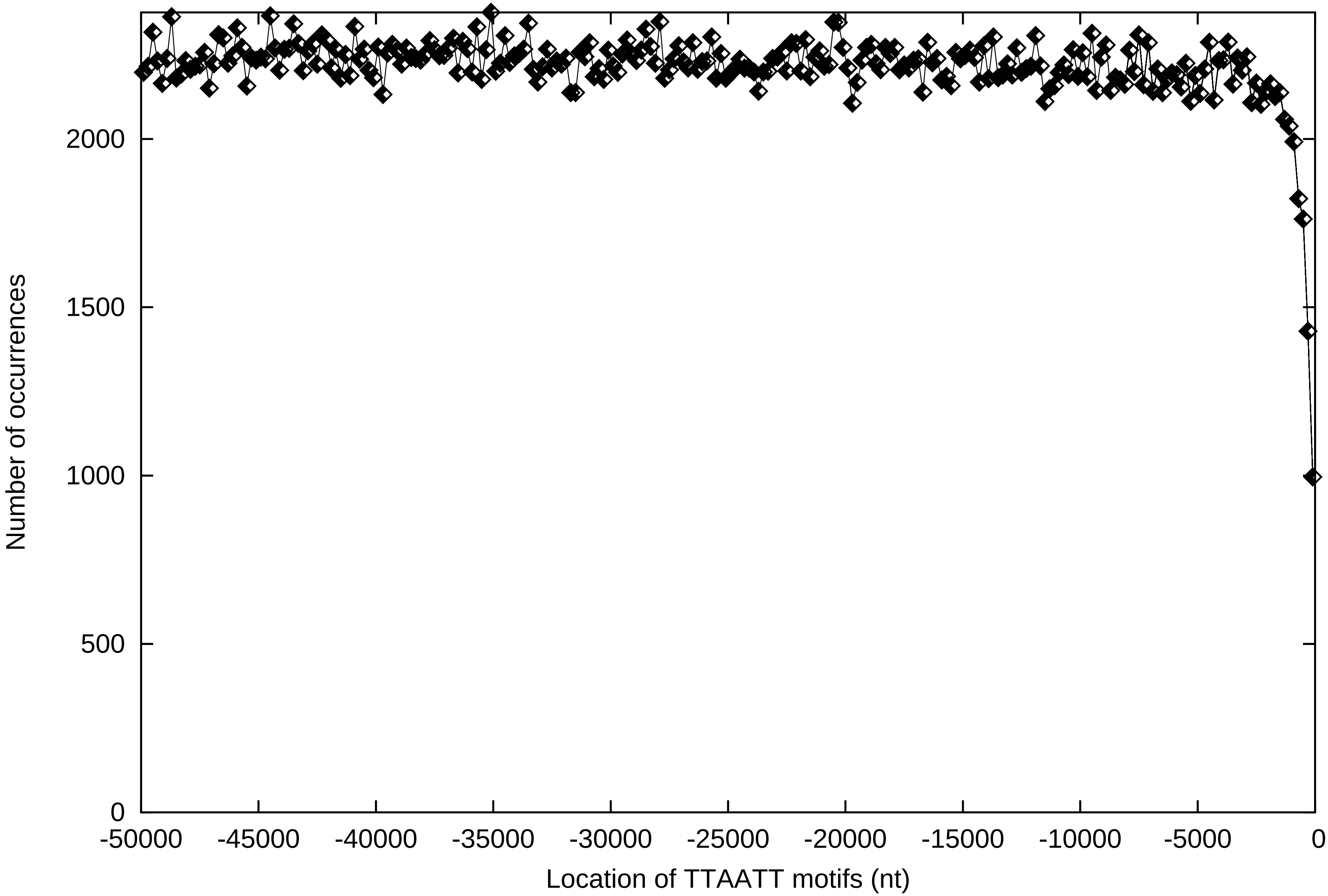

Number of occurrences
0
500
1000
1500
2000
-50000
-45000
-40000
-35000
-30000
-25000
-20000
-15000
-10000
-5000
0
Location of TTAATT motifs (nt)

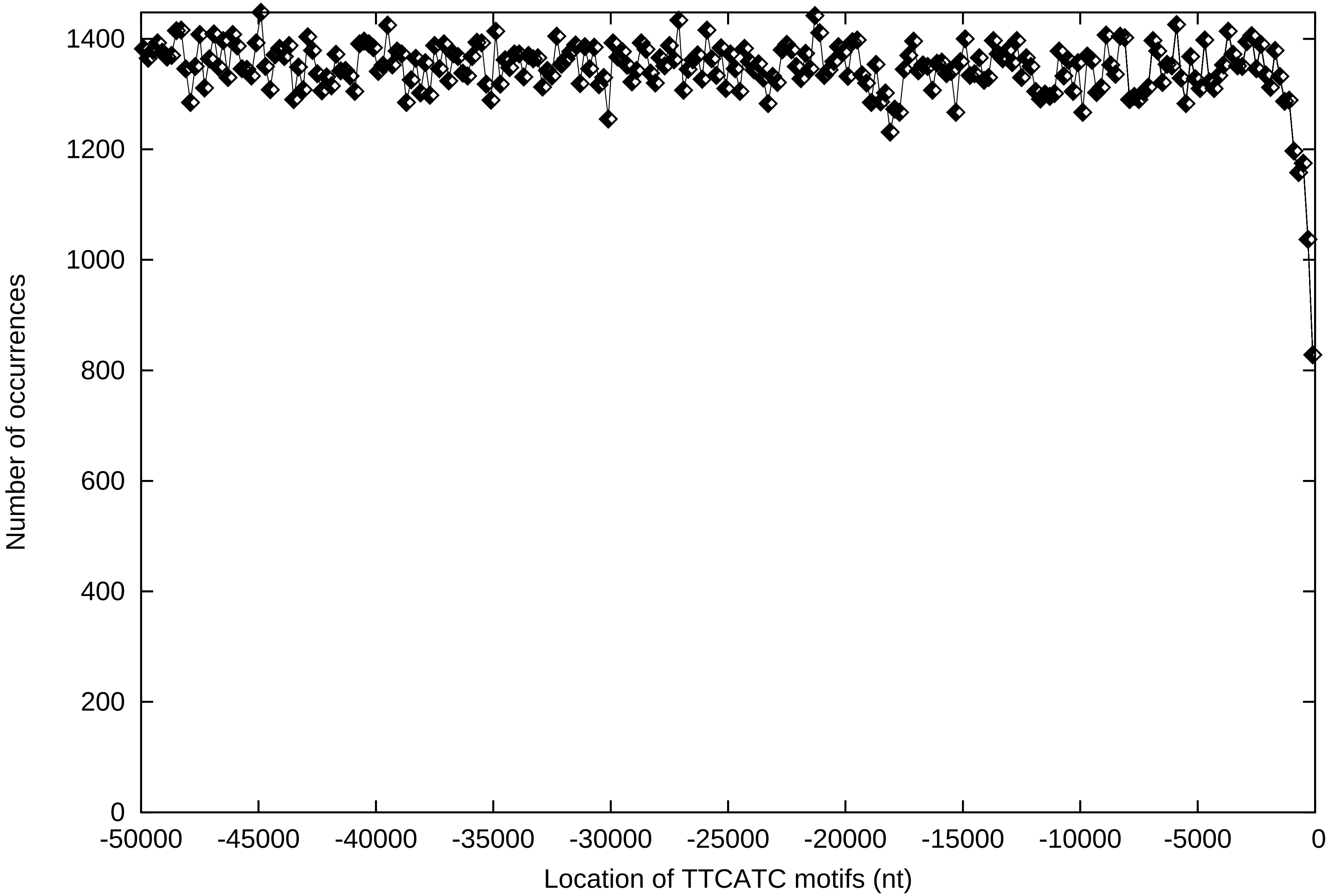

Number of occurrences
0
200
400
600
800
1000
1200
1400
-50000
-45000
-40000
-35000
-30000
-25000
-20000
-15000
-10000
-5000
0
Location of TTCATC motifs (nt)

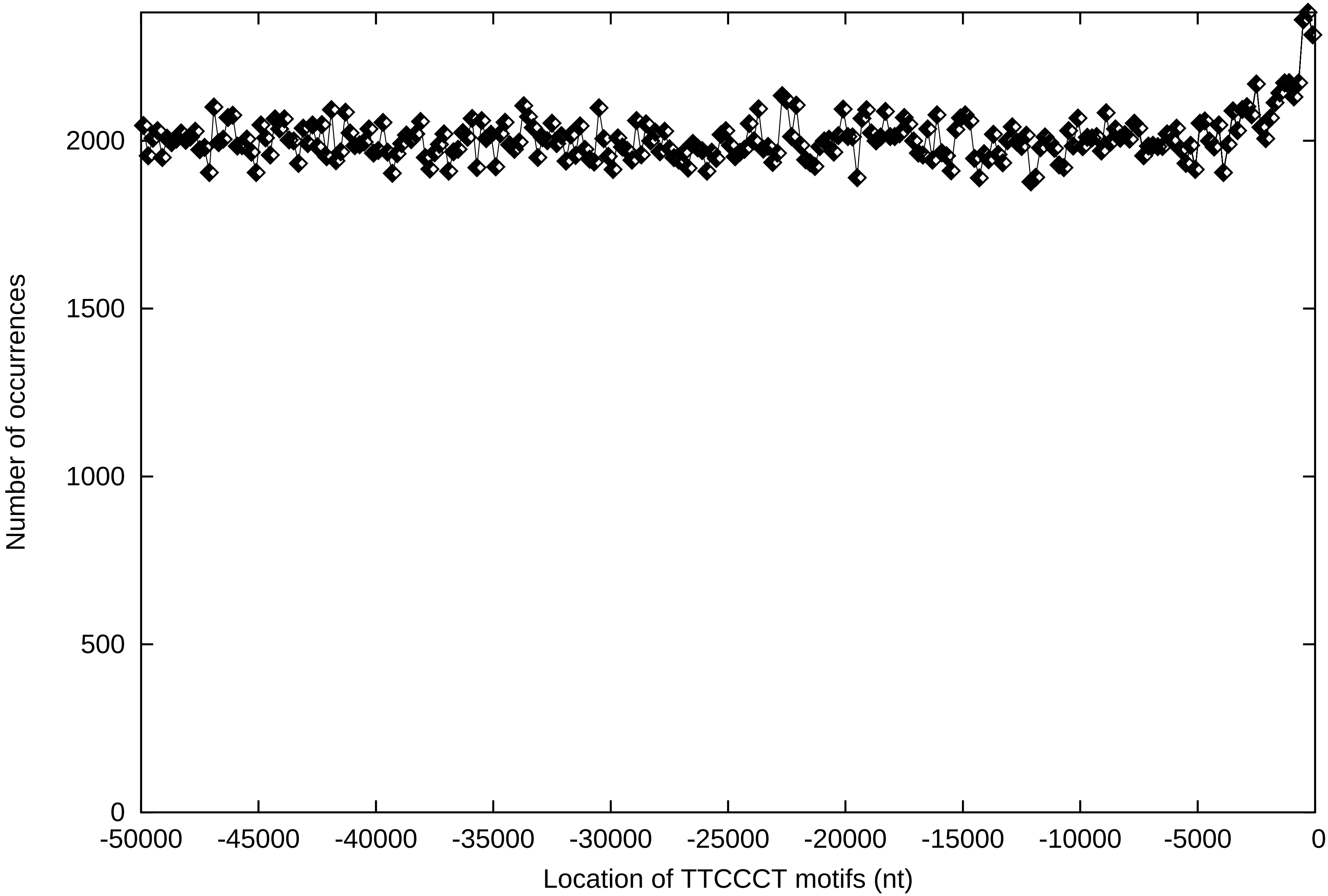

Number of occurrences
0
500
1000
1500
2000
-50000
-45000
-40000
-35000
-30000
-25000
-20000
-15000
-10000
-5000
0
Location of TTCCCT motifs (nt)

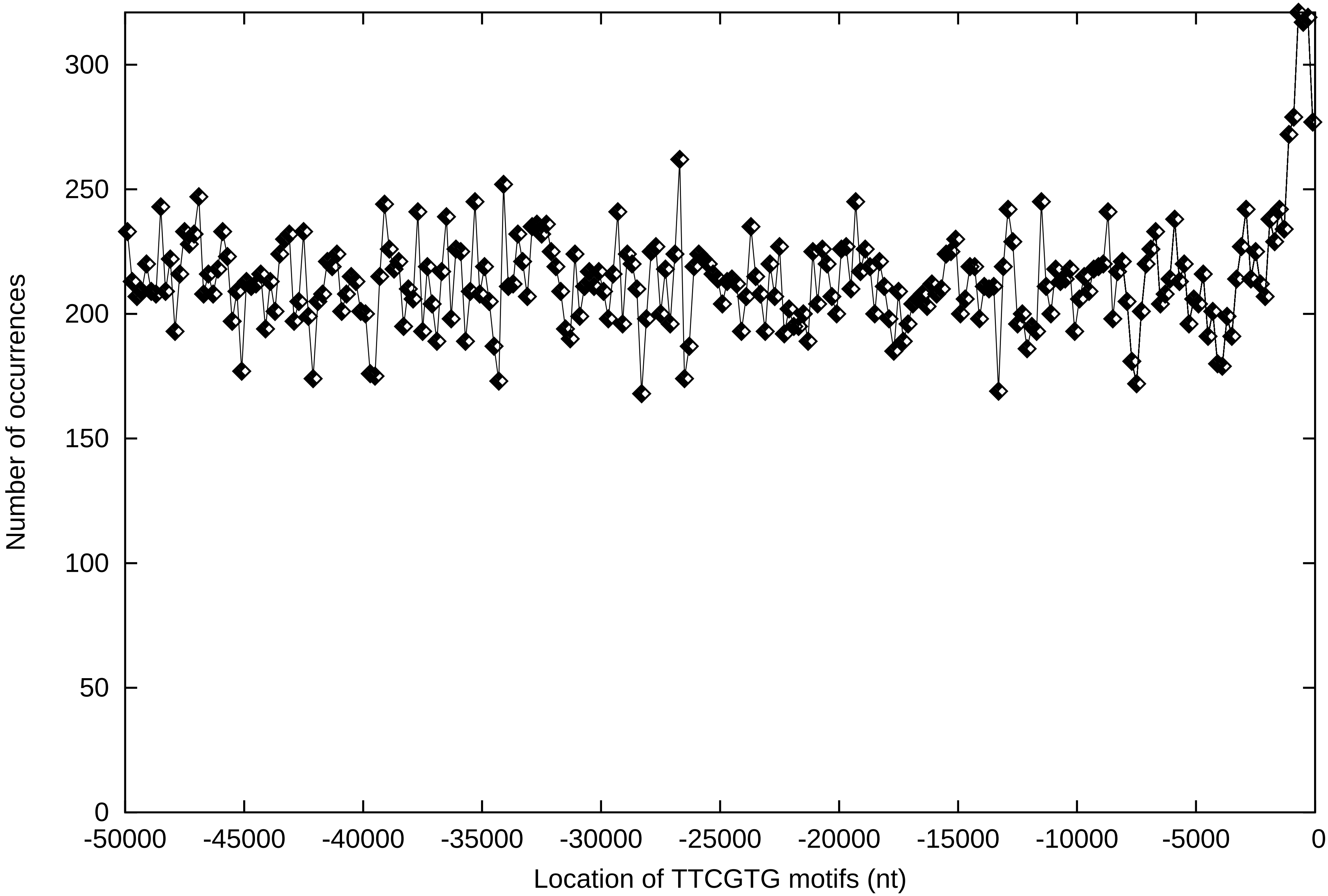
Number of occurrences
Location of TTCGTG motifs (nt)
0
50
100
150
200
250
300
-50000
-45000
-40000
-35000
-30000
-25000
-20000
-15000
-10000
-5000
0

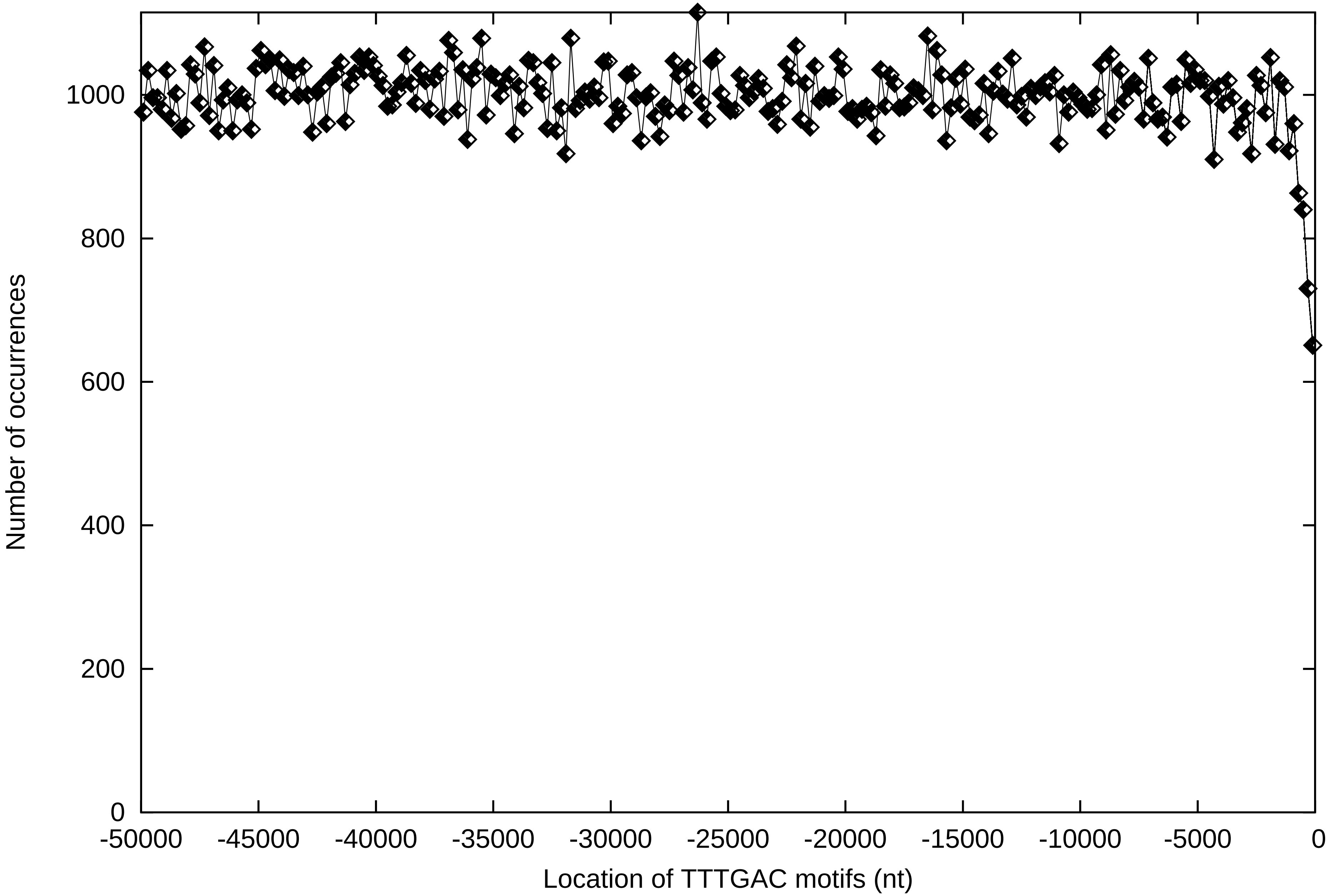

Number of occurrences
0
200
400
600
800
1000
-50000
-45000
-40000
-35000
-30000
-25000
-20000
-15000
-10000
-5000
0
Location of TTTGAC motifs (nt)
