# Supplementary material for: Regulatory Circuit of Human MicroRNA Biogenesis
Source: PLoS Comput Biol. 2007 Apr 20;3(4):e67. doi: 10.1371/journal.pcbi.0030067 (PMC1853126; doi:10.1371/journal.pcbi.0030067)
Supplement: Supplementary file 10 [file pcbi.0030067.sg010.pdf]

Distance distribution of randomly selected 5 mers (pages 2-15). Each graph is a histogram that represents the number of occurrences of each motif (every 200 nt stretch) in the first 50 kb upstream region of protein-coding genes. In stark contrast to the top-predicted motifs, the control motifs do not have a strong preference to occur in the neighbor hood of the transcription start sites of protein-coding genes.

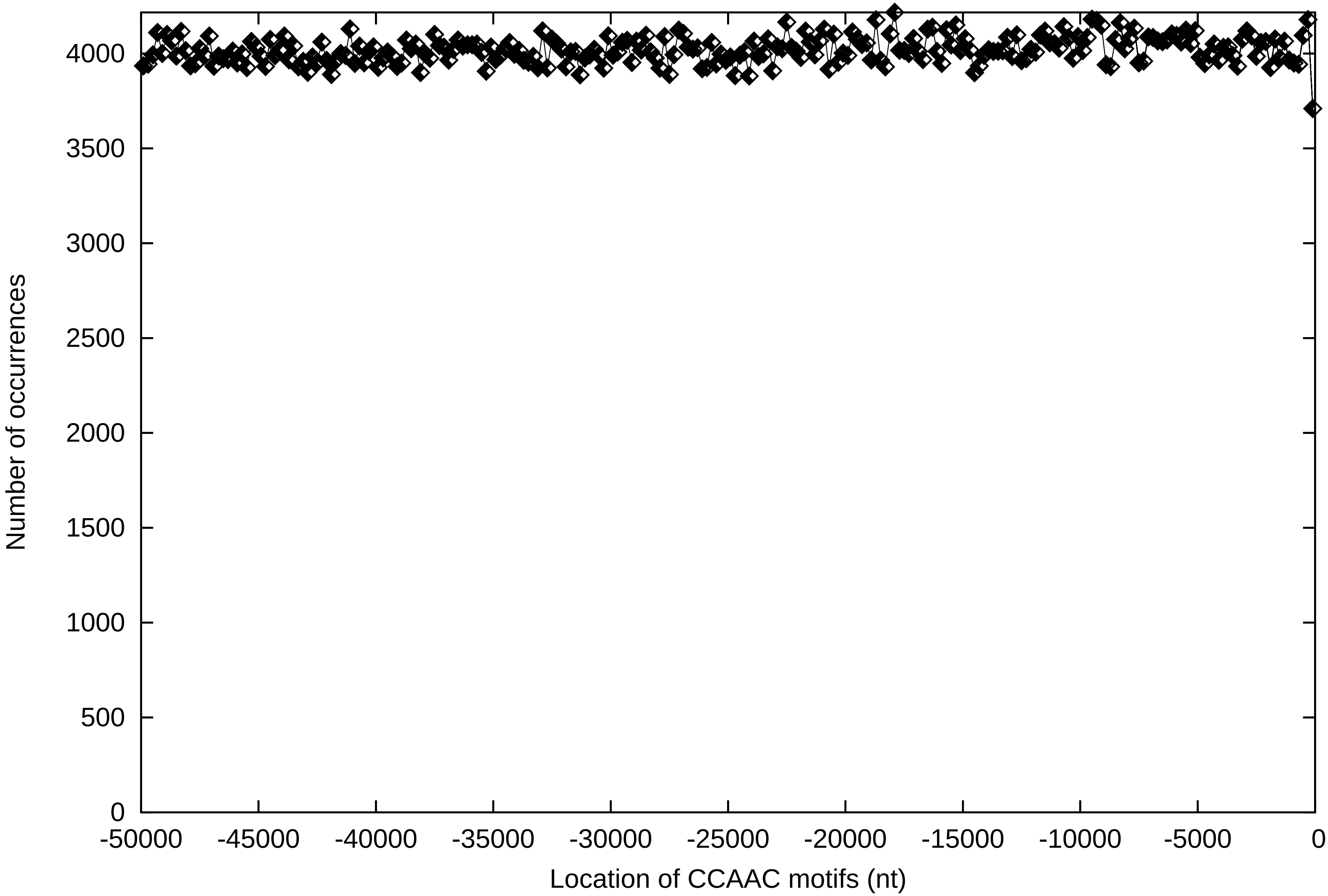

Number of occurrences
0
500
1000
1500
2000
2500
3000
3500
4000
-50000
-45000
-40000
-35000
-30000
-25000
-20000
-15000
-10000
-5000
0
Location of CCAAC motifs (nt)

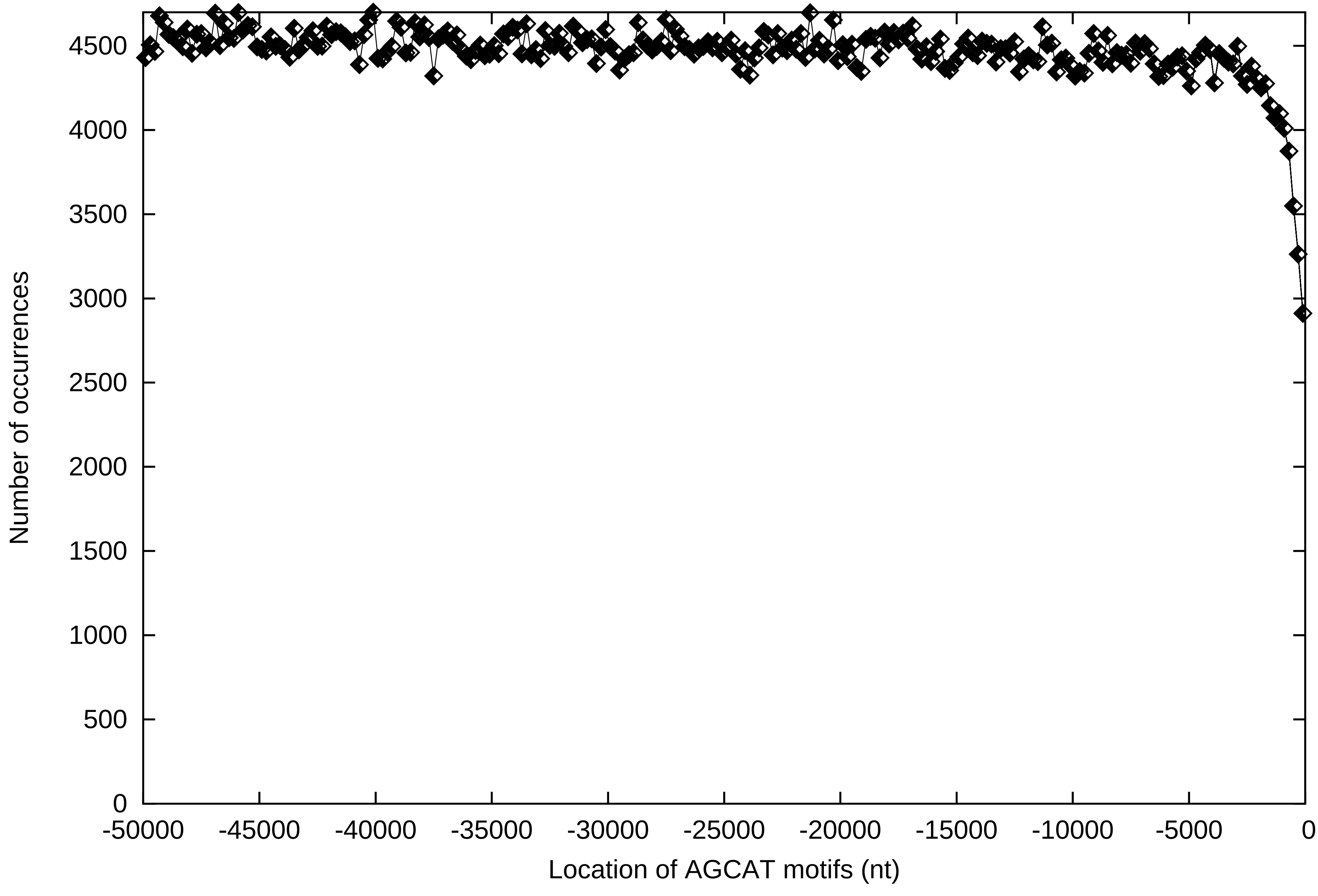

Number of occurrences
0
500
1000
1500
2000
2500
3000
3500
4000
4500
-50000
-45000
-40000
-35000
-30000
-25000
-20000
-15000
-10000
-5000
0
Location of AGCAT motifs (nt)

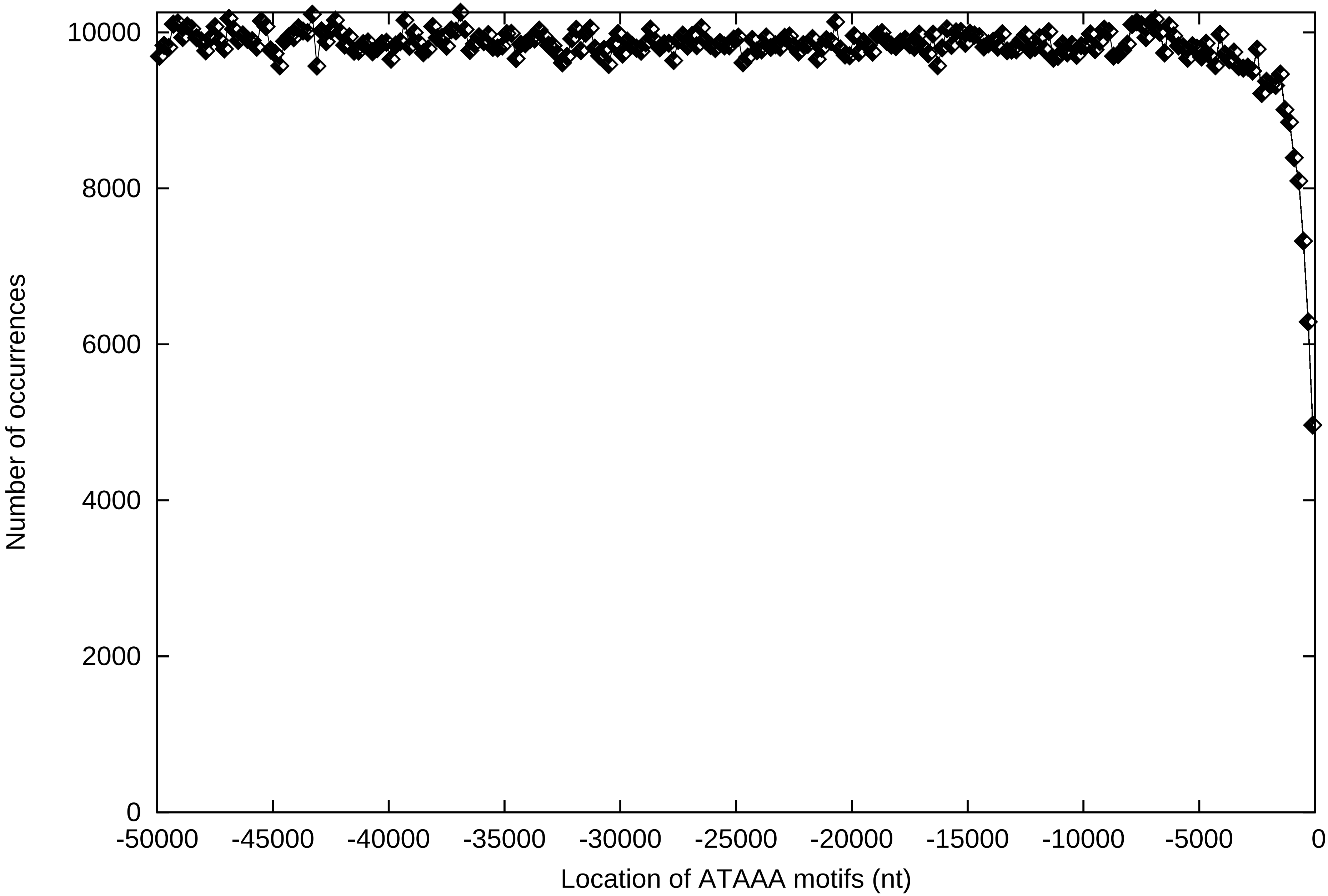

Number of occurrences
10000
8000
6000
4000
2000
0
-50000
-45000
-40000
-35000
-30000
-25000
-20000
-15000
-10000
-5000
0
Location of ATAAA motifs (nt)

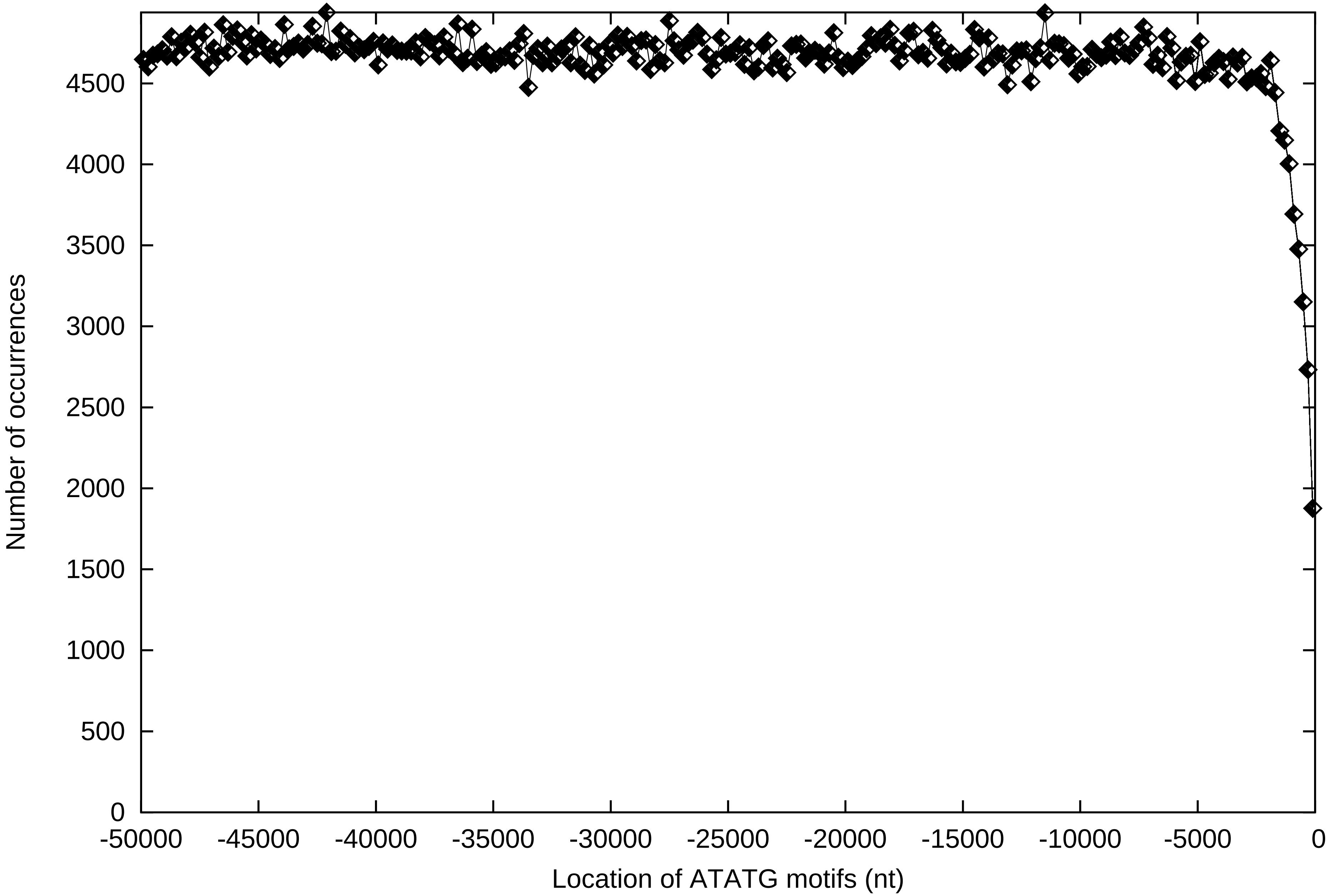

Number of occurrences
0
500
1000
1500
2000
2500
3000
3500
4000
4500
-50000
-45000
-40000
-35000
-30000
-25000
-20000
-15000
-10000
-5000
0
Location of ATATG motifs (nt)

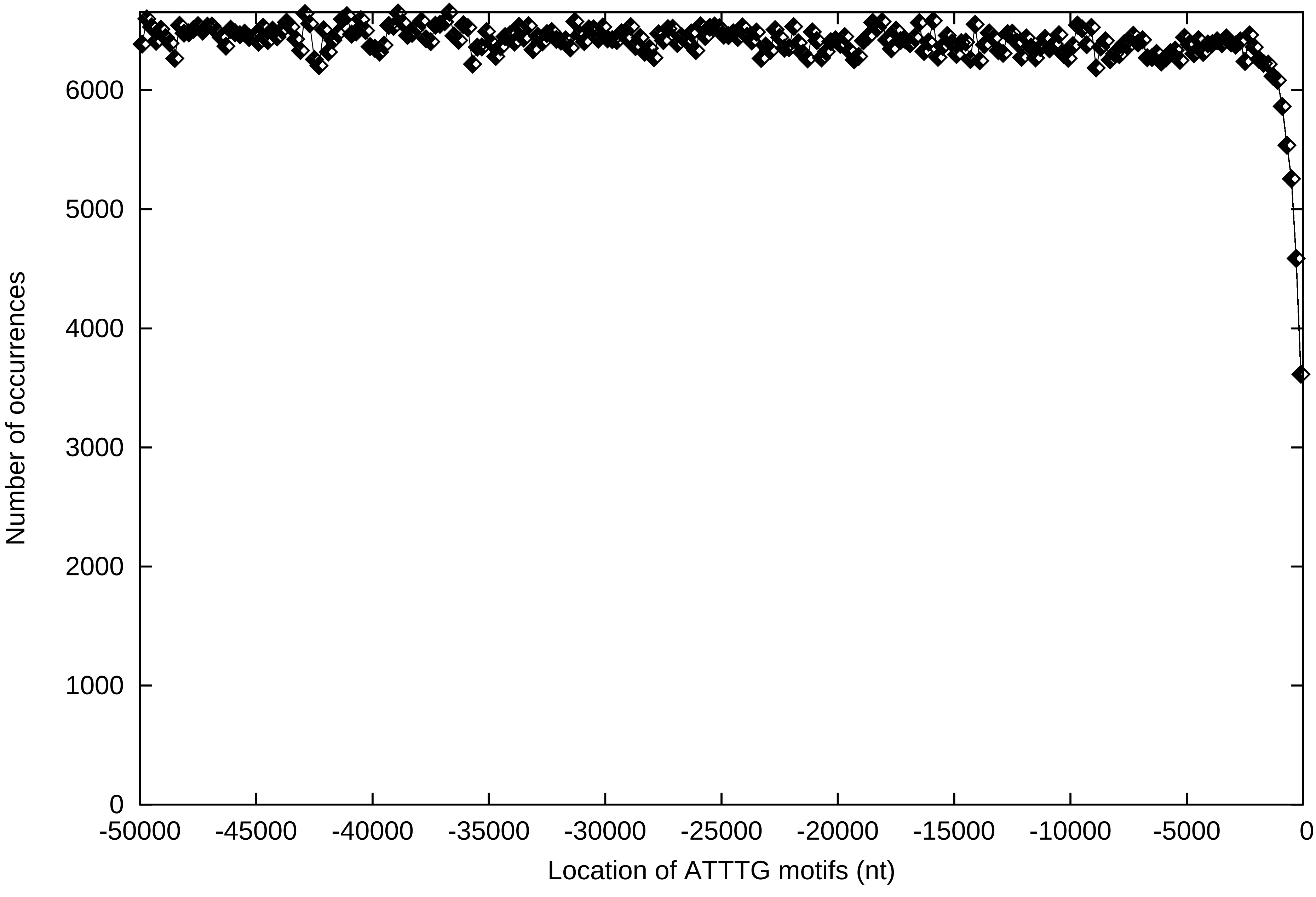

Number of occurrences
0
1000
2000
3000
4000
5000
6000
-50000
-45000
-40000
-35000
-30000
-25000
-20000
-15000
-10000
-5000
0
Location of ATTTG motifs (nt)

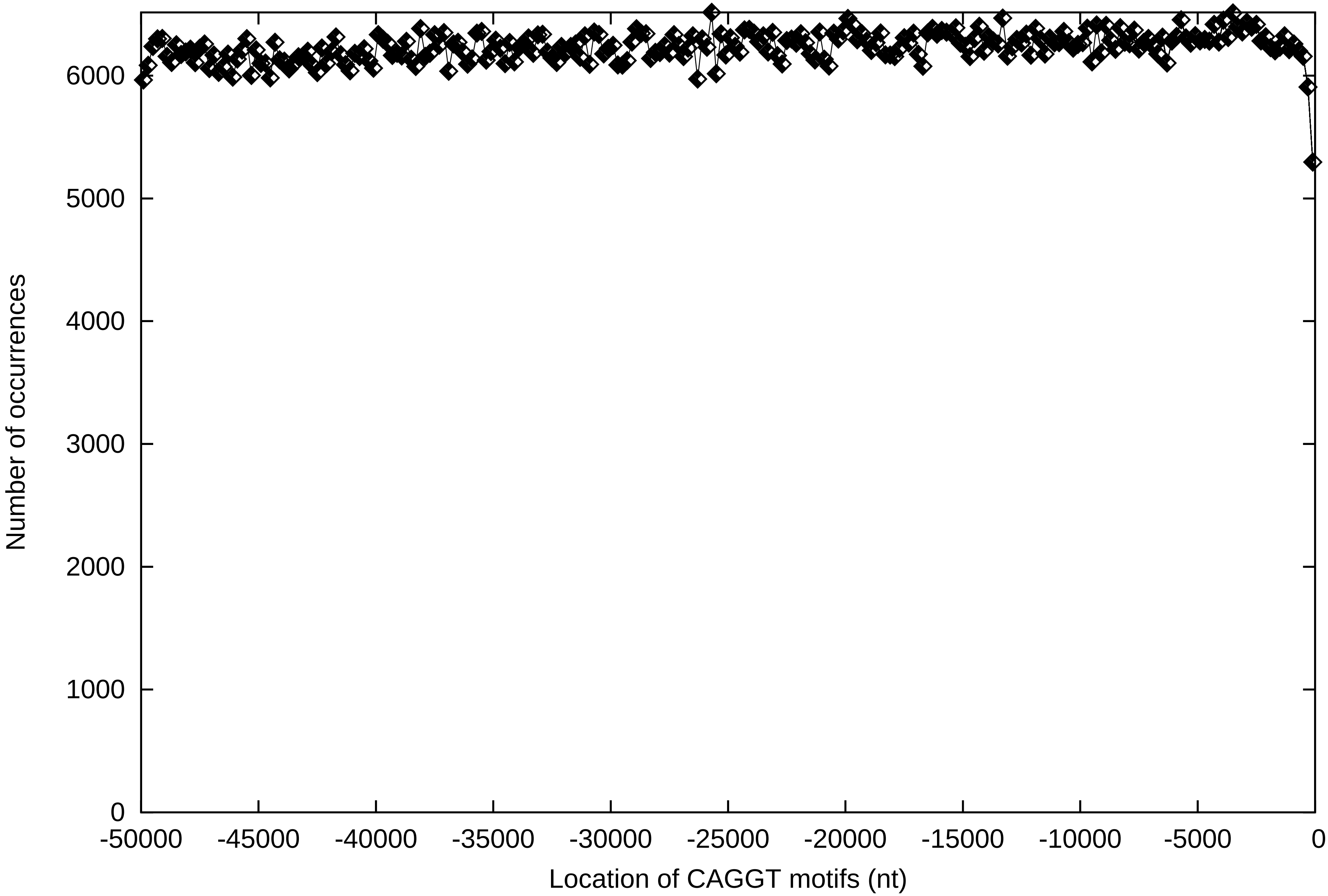

Number of occurrences
6000
5000
4000
3000
2000
1000
0
-50000
-45000
-40000
-35000
-30000
-25000
-20000
-15000
-10000
-5000
0
Location of CAGGT motifs (nt)

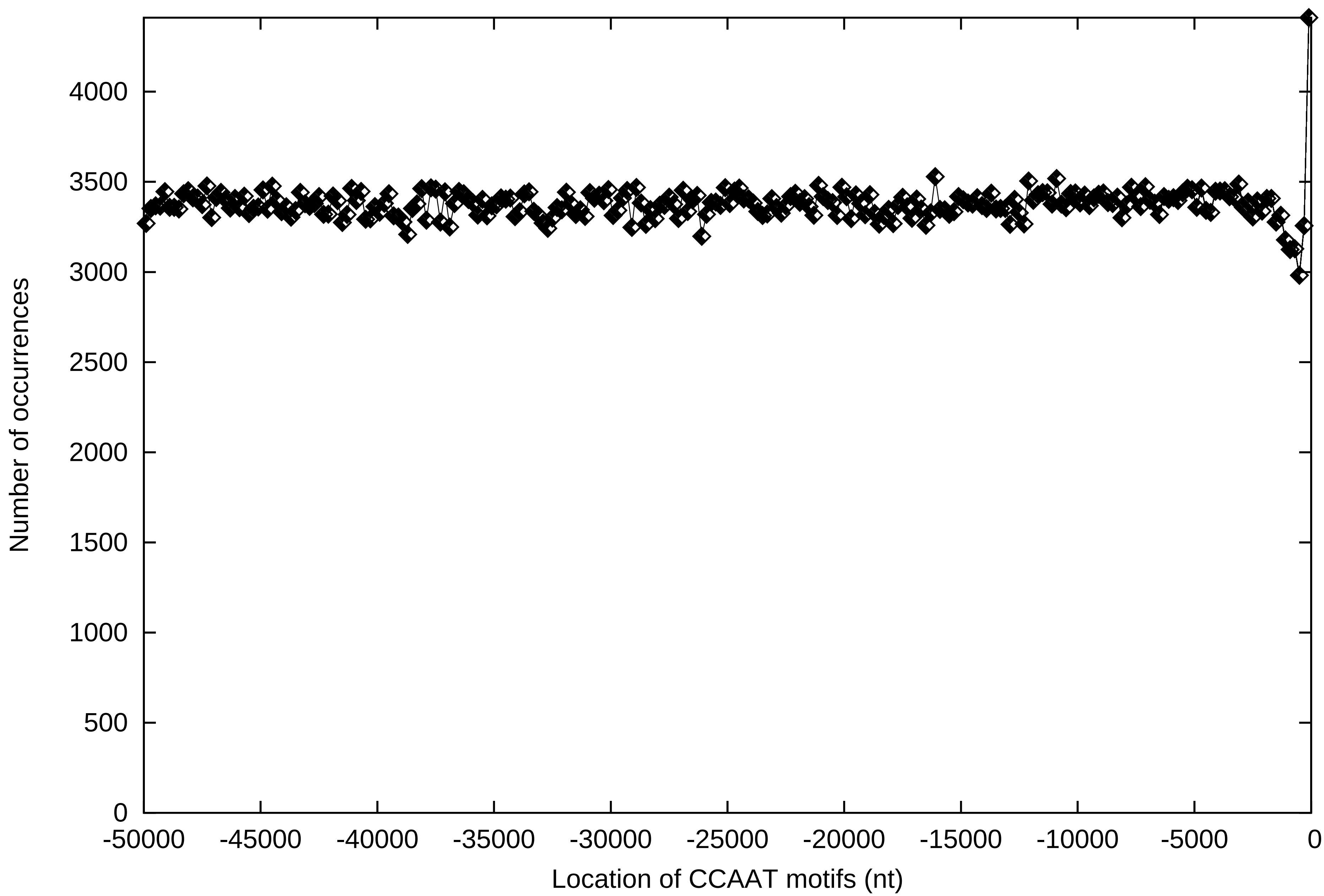
Number of occurrences
0
500
1000
1500
2000
2500
3000
3500
4000
-50000
-45000
-40000
-35000
-30000
-25000
-20000
-15000
-10000
-5000
0
Location of CCAAT motifs (nt)

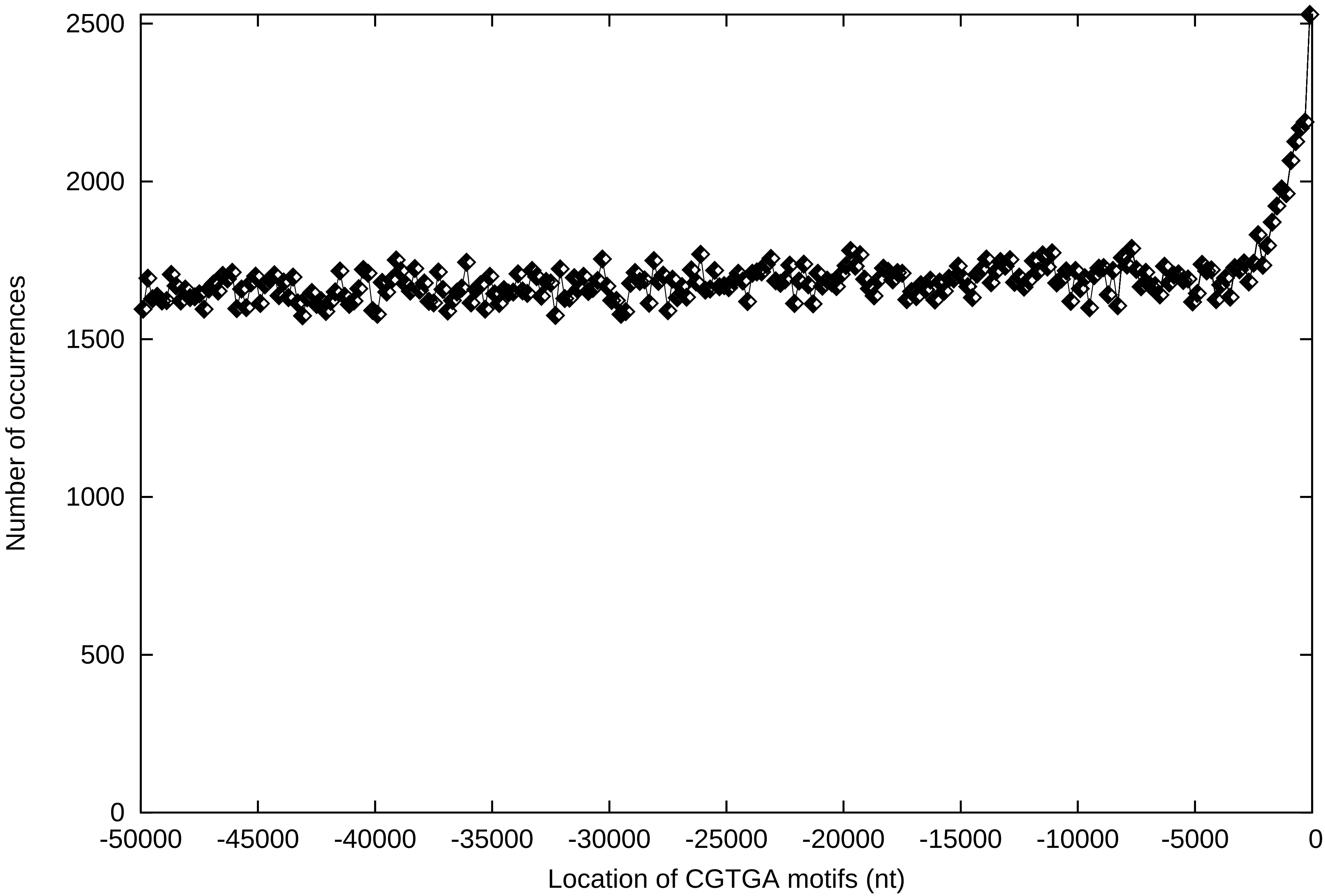

2500
2000
1500
1000
500
0
-50000
-45000
-40000
-35000
-30000
-25000
-20000
-15000
-10000
-5000
0
Number of occurrences
Location of CGTGA motifs (nt)

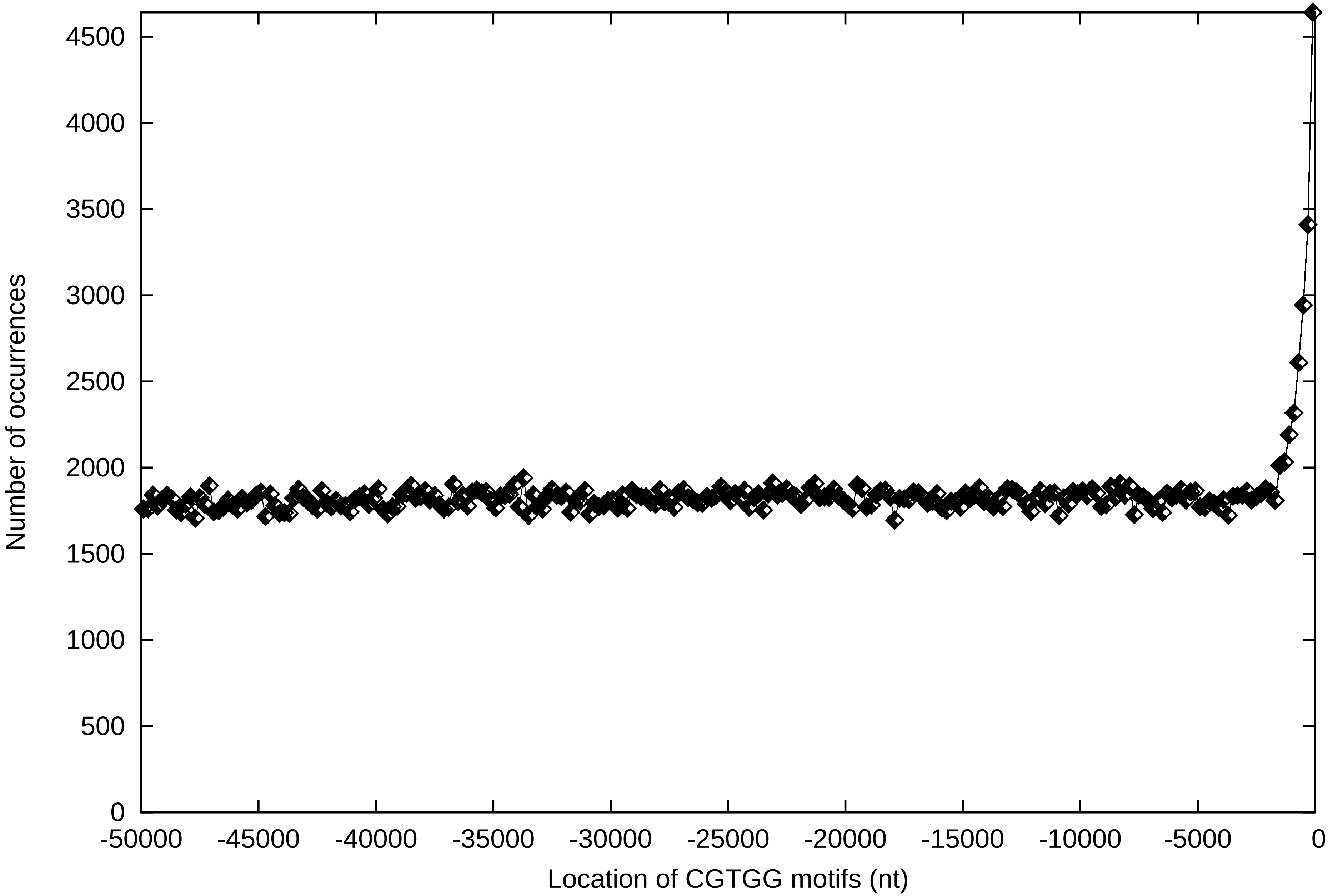

Number of occurrences
0
500
1000
1500
2000
2500
3000
3500
4000
4500
-50000
-45000
-40000
-35000
-30000
-25000
-20000
-15000
-10000
-5000
0
Location of CGTGG motifs (nt)

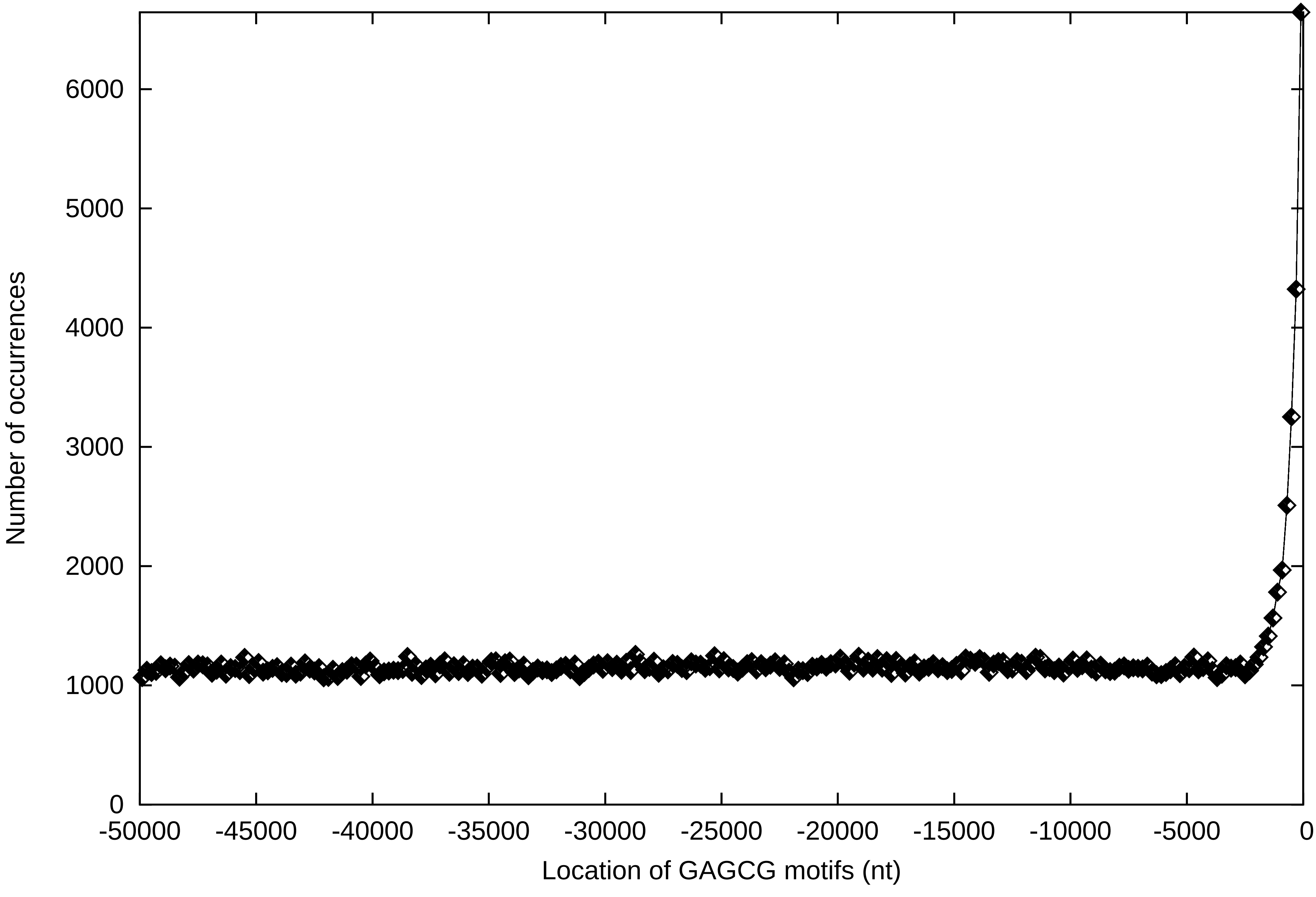

Number of occurrences
0
1000
2000
3000
4000
5000
6000
-50000
-45000
-40000
-35000
-30000
-25000
-20000
-15000
-10000
-5000
0
Location of GAGCG motifs (nt)

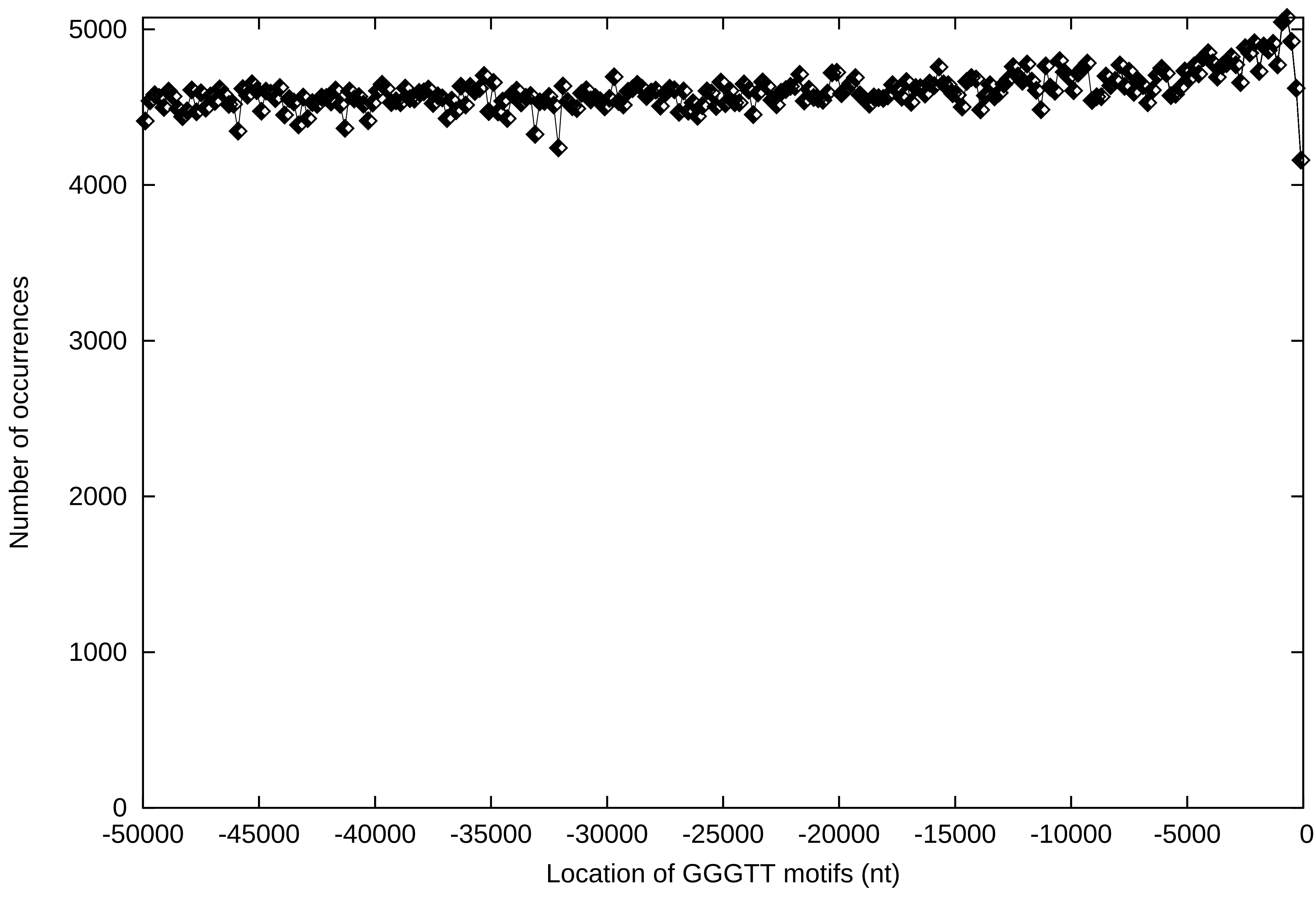

5000
4000
3000
2000
1000
0
Number of occurrences
-50000
-45000
-40000
-35000
-30000
-25000
-20000
-15000
-10000
-5000
0
Location of GGGTT motifs (nt)

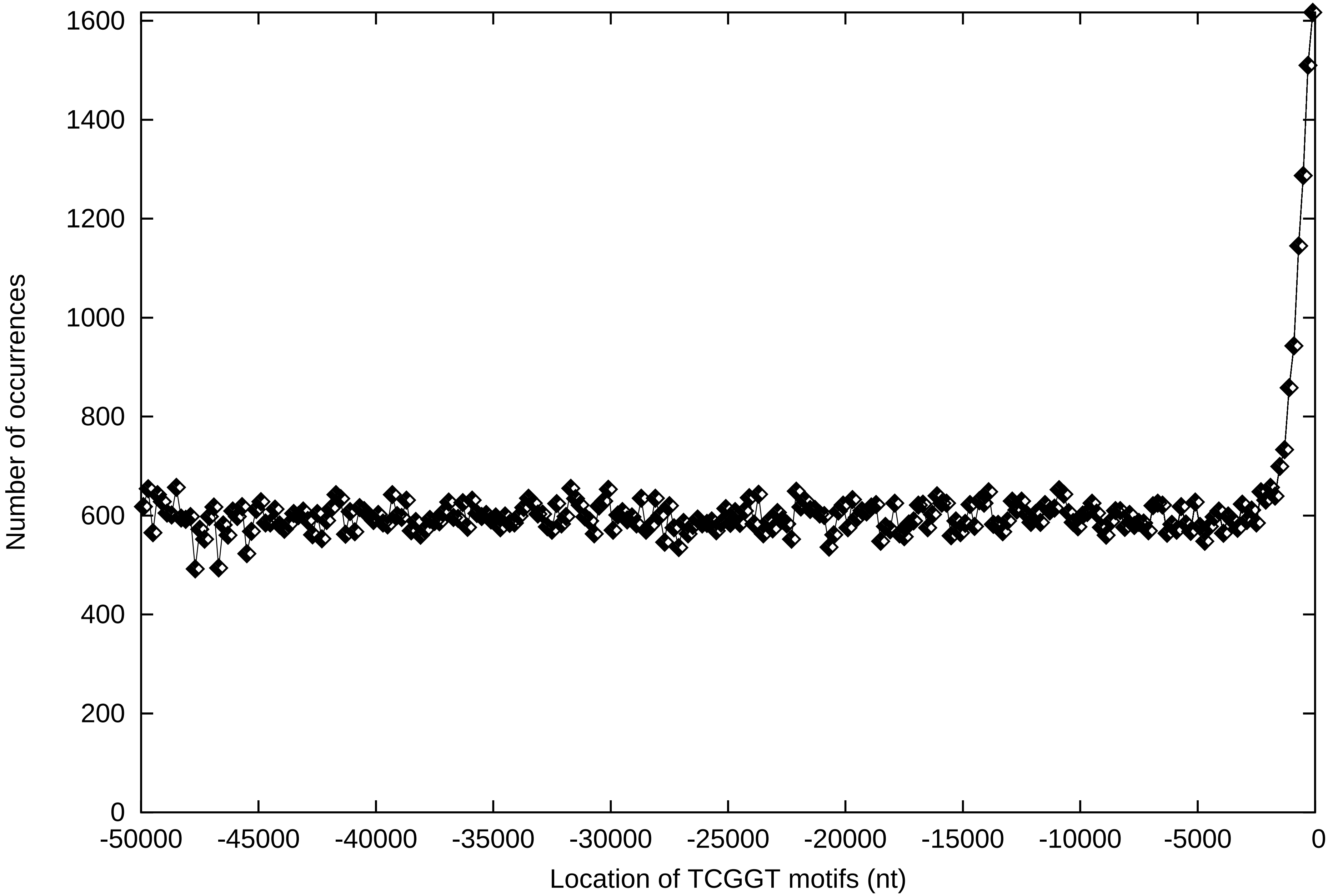

1600
1400
1200
1000
800
600
400
200
0
-50000
-45000
-40000
-35000
-30000
-25000
-20000
-15000
-10000
-5000
0
Number of occurrences
Location of TCGGT motifs (nt)

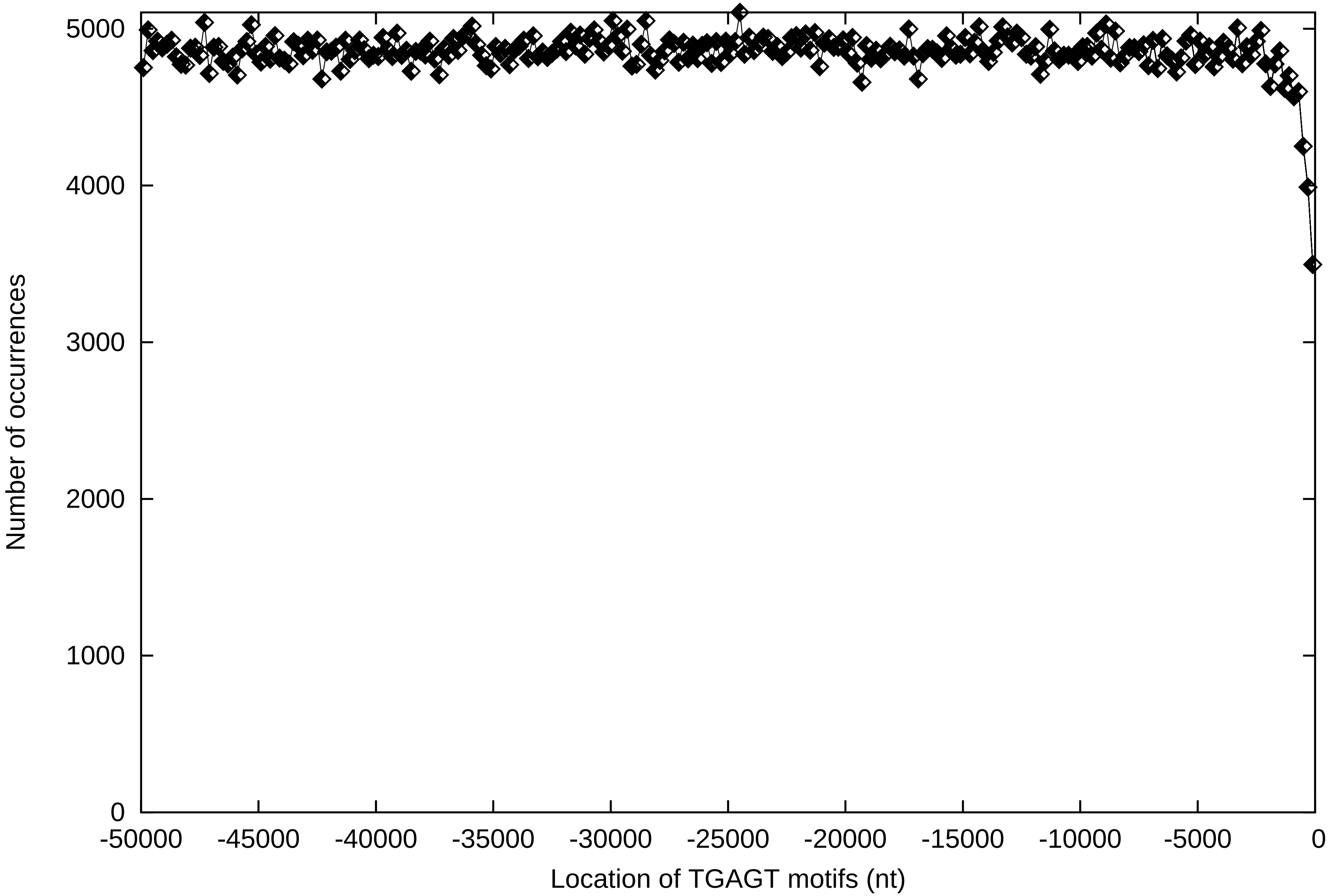

Number of occurrences
Location of TGAGT motifs (nt)
0
1000
2000
3000
4000
5000
-50000
-45000
-40000
-35000
-30000
-25000
-20000
-15000
-10000
-5000
0

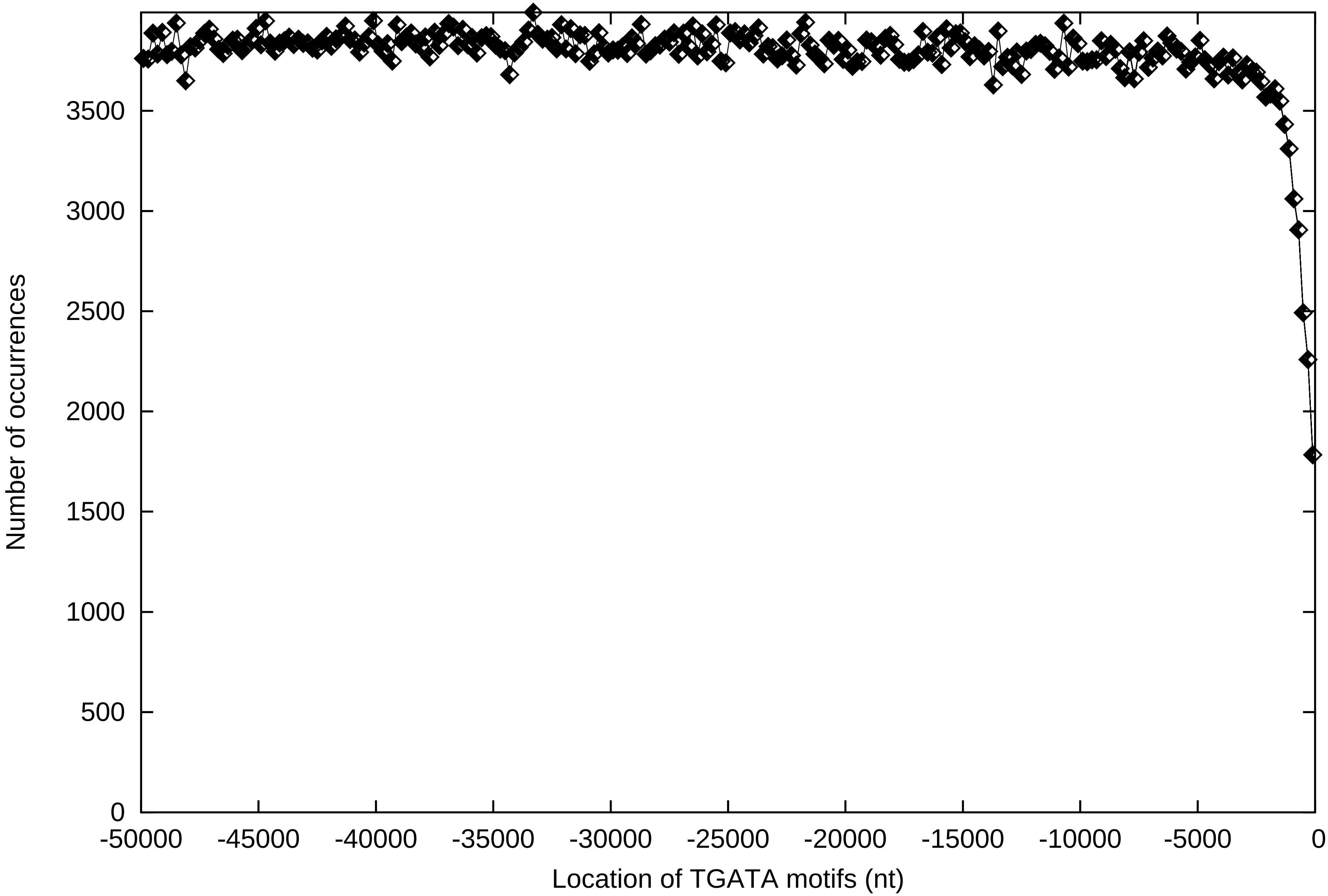

Number of occurrences
0
500
1000
1500
2000
2500
3000
3500
-50000
-45000
-40000
-35000
-30000
-25000
-20000
-15000
-10000
-5000
0
Location of TGATA motifs (nt)
