# Supplementary material for: Regulatory Circuit of Human MicroRNA Biogenesis
Source: PLoS Comput Biol. 2007 Apr 20;3(4):e67. doi: 10.1371/journal.pcbi.0030067 (PMC1853126; doi:10.1371/journal.pcbi.0030067)
Supplement: Supplementary file 11 [file pcbi.0030067.st001.pdf]

Single nucleotide frequencies in the various datasets containing 10kb long sequences (total of 214 sequences)

Nucleotide	upstream miRNA sequences	Upstream protein coding sequences (UPS)		Repeat-masked control sequences (RGS)		Unmasked control sequences (GS)	
		Average	Standard deviation	Average	Standard deviation	Average	Standard deviation
A	0.26	0.27	0.03	0.30	0.03	0.29	0.03
T	0.28	0.27	0.03	0.30	0.03	0.29	0.03
C	0.23	0.22	0.02	0.20	0.02	0.20	0.02
G	0.23	0.22	0.02	0.20	0.02	0.20	0.02
