# Supplementary material for: Regulatory Circuit of Human MicroRNA Biogenesis
Source: PLoS Comput Biol. 2007 Apr 20;3(4):e67. doi: 10.1371/journal.pcbi.0030067 (PMC1853126; doi:10.1371/journal.pcbi.0030067)
Supplement: Supplementary file 12 [file pcbi.0030067.st002.pdf]

Ref. miRNA	Cluster members	Chr	N	Positions	Expression pattern of mature miRNA
124a-3		20	8	7010 8768 8904 8977 9048 9095 9293 9498	Brain specific miRNA; Highly expressed during brain development[1,2]
425	191	3	7	754 782 8107 8607 8632 8936 9194	Neurons[3]
124a-1		8	6	6254 6601 6624 6911 7142 9426	Brain specific miRNA; Highly expressed during brain development[2]
607		10	6	5851 5882 6150 6171 6208 6659	Colorectal cancer cells (CC)[4]
550-1		7	6	4797 4976 5014 5540 5613 5852	CC[4]
132	212	17	6	3202 3883 4127 8816 9922 9999	Highly expressed in brain[5]
96	182, 183	7	6	1580 2833 4611 4765 4939 6201	Significantly expressed in dorsal root ganglia[6] (neuronal cells) and embryonic stem cells (ES)[7]
9-2		5	6	1152 1560 2303 2511 2788 2794	Highly expressed during brain development[3,8]
559		2	6	1443 1635 1825 2075 2417 2752	CC[4]
20a	19b-1,92-1, 17,19a,18a	13	5	6312 6328 7279 7474 7942	ES[9], Highly expressed during development of skin epidermis[10].
210		11	5	1460 2336 9447 9618 9858	Predominantly expressed in Thymus[9], Abundant in pancreatic-islets (PI)[11]
483		11	5	257 3108 3718 4188 4764	CC[4]
486		8	5	574 580 586 614 620	Fetal development of liver[12]
363	18b,106a, 20b,19b-2, 92-2	X	4	6572 7288 7445 7754	Prenatal embryonic development[8]
345		14	4	8465 8627 8789 8858	Fetal development of liver[12]
378		5	4	7349 8385 9218 9267	PI[13]
129-2		11	4	4064 8490 8496 9884	Expressed in cerebellum[1]
181d	181c	19	4	180 902 954 1060	Down-regulated in glioblastoma[14]
589		7	4	4275 4944 4955 4989	CC[4]
101-1		1	4	600 952 1001 1834	Predominantly expressed in brain[1]
135b		1	4	1334 1598 1666 2304	Expressed in cortical neurons[3]
375		2	3	8395 8613 8904	PI specific miRNA[11]
9-1		1	3	8845 8942 9646	Highly expressed during brain development[3,8]
92b		1	3	9528 9535 9812	Abundant in brain[4]
25	106b,93	7	3	2280 2775 2917	Widely expressed in ES[9]

Table S2: miRNA genes that contain atleast 3 occurrences of the motif, CGCGCG within a contiguous stretch of 2 kb in their upstream regions (<10 kb). The locations of the motif (Positions) are with respect to the start of the first 10 kb upstream region of the reference miRNA (Ref. miRNA). The miRNAs for which the upstream regions (Cluster members) overlap with that of the reference miRNA and satisfy the criteria for the occurrences of motifs are noted for clarity. The number of occurrences (N) of the motif upstream of the reference miRNA is also indicated.

Reference List

1. Lagos-Quintana, M., Rauhut, R., Yalcin, A., Meyer, J., Lendeckel, W. & Tuschl, T. Identification of tissue-specific microRNAs from mouse. *Curr. Biol.* **12**, 735-739 (2002).

2. Krichevsky, A. M., King, K. S., Donahue, C. P., Khrapko, K. & Kosik, K. S. A microRNA array reveals extensive regulation of microRNAs during brain development. *RNA.* **9**, 1274-1281 (2003).

3. Kim, J., Krichevsky, A., Grad, Y., Hayes, G. D., Kosik, K. S., Church, G. M. & Ruvkun, G. Identification of many microRNAs that copurify with polyribosomes in mammalian neurons. *Proc. Natl. Acad. Sci. U. S. A* **101**, 360-365 (2004).

4. Cummins, J. M., He, Y., Leary, R. J., Pagliarini, R., Diaz, L. A., Jr., Sjoblom, T., Barad, O., Bentwich, Z., Szafranska, A. E., Labourier, E., Raymond, C. K., Roberts, B. S., Juhl, H., Kinzler, K. W., Vogelstein, B. & Velculescu, V. E. The colorectal microRNAome. *Proc. Natl. Acad. Sci. U. S. A* (2006).

5. Sempere, L. F., Freemantle, S., Pitha-Rowe, I., Moss, E., Dmitrovsky, E. & Ambros, V. Expression profiling of mammalian microRNAs uncovers a subset of brain-expressed microRNAs with possible roles in murine and human neuronal differentiation. *Genome Biol.* **5**, R13 (2004).

6. Kloosterman, W. P., Wienholds, E., de, B. E., Kauppinen, S. & Plasterk, R. H. In situ detection of miRNAs in animal embryos using LNA-modified oligonucleotide probes. *Nat. Methods* **3**, 27-29 (2006).

7. Houbaviy, H. B., Murray, M. F. & Sharp, P. A. Embryonic stem cell-specific MicroRNAs. *Dev. Cell* **5**, 351-358 (2003).

8. Mineno, J., Okamoto, S., Ando, T., Sato, M., Chono, H., Izu, H., Takayama, M., Asada, K., Mirochnitchenko, O., Inouye, M. & Kato, I. The expression profile of microRNAs in mouse embryos. *Nucleic Acids Res.* **34**, 1765-1771 (2006).

9. Thomson, J. M., Parker, J., Perou, C. M. & Hammond, S. M. A custom microarray platform for analysis of microRNA gene expression. *Nat. Methods* **1**, 47-53 (2004).

10. Yi, R., O'carroll, D., Pasolli, H. A., Zhang, Z., Dietrich, F. S., Tarakhovsky, A. & Fuchs, E. Morphogenesis in skin is governed by discrete sets of differentially expressed microRNAs. *Nat. Genet.* (2006).

11. Poy, M. N., Eliasson, L., Krutzfeldt, J., Kuwajima, S., Ma, X., Macdonald, P. E., Pfeffer, S., Tuschl, T., Rajewsky, N., Rorsman, P. & Stoffel, M. A pancreatic islet-specific microRNA regulates insulin secretion. *Nature* **432**, 226-230 (2004).

12. Fu, H., Tie, Y., Xu, C., Zhang, Z., Zhu, J., Shi, Y., Jiang, H., Sun, Z. & Zheng, X. Identification of human fetal liver miRNAs by a novel method. *FEBS Lett.* **579**, 3849-3854 (2005).

13. Fernandez-Zapico, M. E., Bramati, P. S., Zakaria, S., Kaczynski, J. A. & Urrutia, R. Fundamentals of transcription factors and their impact on pancreatic development and cancer. *Pancreatology.* **3**, 276-283 (2003).

14. Ciafre, S. A., Galardi, S., Mangiola, A., Ferracin, M., Liu, C. G., Sabatino, G., Negrini, M., Maira, G., Croce, C. M. & Farace, M. G. Extensive modulation of a set of microRNAs in primary glioblastoma. *Biochem. Biophys. Res. Commun.* **334**, 1351-1358 (2005).
