# Supplementary material for: Regulatory Circuit of Human MicroRNA Biogenesis
Source: PLoS Comput Biol. 2007 Apr 20;3(4):e67. doi: 10.1371/journal.pcbi.0030067 (PMC1853126; doi:10.1371/journal.pcbi.0030067)
Supplement: Supplementary file 13 [file pcbi.0030067.st003.pdf]

Table S3: Hexamer motifs and TFs that are predicted to regulate specific miRNAs

MicroRNA	Predicted hexamer sequence motifs upstream of miRNA	Candidate transcription factors
hsa-let-7i	CGCCGC GCGGCG CCGCGT CGCGGC CGCGTG CCGGCG CCGCCG CGCGAG CGGCGA GCGCGG GGGCCG CGGCGC GCGCCG GGGGCG GCGGGG AGCGCG CGCGGG GCCGCC CGGCGG CGCCGG GCCGGC GCGCGC	Sp1 GLI1 EFI CTF-2 YY1 CTF-3 CTF c-Ets-2 CTF-1 GLI3 NF-Y AP-2alphaA GKLF Ikaros p53 ZNFN1A1 Copeb HNF-3beta COUP-TF1 CP2 GATA-2 GATA-1 MTF-1 CTCF AP-2 AP-2gamma HES-1 E2F-1 AP-2delta HDBP2 AP-2beta c-Myb HDBP1 AP-2alpha E12 M-Twist M-Twist:E12 TWIST TWIST:E12 MITF Smad3 AhR:Arnt Smad4 c-Jun:c-Fos AP-4 c-Jun Sp3 AP-1 Egr-1 GCF MAZ ENKTF-1 Egr-2 MZF-1 E2F-1:DP-1 EBF
hsa-mir-106a	GCGCGG TGCGCG CGCGCG CCCGCG AGCGCG CGCGGG GCGCGT CGCGCT GCGCGC	c-Jun:c-Fos Sp1 AP-4 GLI1 c-Myb c-Jun GLI3 MTF-1 GKLF Sp3 AP-1 p53 Egr-1 HNF-3beta HES-1 HDBP2 GCF HDBP1 E12 M-Twist M-Twist:E12 TWIST AhR:Arnt TWIST:E12 MITF E2F-1:DP-1 MAZ EBF AP-2 AP-2gamma GATA-2 GATA-1 AP-2delta CP2 AP-2beta AP-2alpha AP-2alphaA ENKTF-1 MZF-1 NF-Y Pmx2a HOXA5
hsa-mir-124a-1	GGCCGC GGGGCG GCGGGG GCGGCG CGGCGG CCGGCG CGGCCG CGGCGA	COUP-TF1 CTCF Sp1 Egr-1 GATA-2 GATA-1 Ikaros CP2 HOXA5 Pmx2a ZNFN1A1 MTF-1 Sp3 AP-1 c-Jun:c-Fos Egr-2 c-Jun AP-2alphaA Copeb MZF-1 c-Myb GKLF p53 NF-Y HNF-3beta MITF Smad3 Smad4 AhR:Arnt
hsa-mir-124a-3	CGCCGC CGCGTG CCGCCG CGGCCG GCCGCG GCGCGG GGCCGC CGCGGA GGGCCG CGGCGC GGGGCG GCGGGG CCCGCG CGCGGG CGGCGG GCGGCG CGCGGC CCGGCG CGGCGA CGCGCT CCGCGG GCGCCG TGCGCG CGCGCG AGCGCG GCCGCC CGCCGG GCGCGT GCCGGC GCGCGC TTCGCG	Sp1 GLI1 EFI CTF-2 YY1 CTF-3 CTF c-Ets-2 CTF-1 GLI3 NF-Y AP-2alphaA COUP-TF1 HES-1 E12 M-Twist M-Twist:E12 TWIST TWIST:E12 CTCF HDBP2 HDBP1 CP2 Pmx2a HOXA5 GATA-2 GATA-1 c-Jun:c-Fos AP-4 c-Myb c-Jun MTF-1 GKLF Sp3 AP-1 p53 Egr-1 HNF-3beta GCF Ikaros ZNFN1A1 Copeb Egr-2 MZF-1 MAZ EBF AP-2 AP-2gamma MITF AP-2delta AP-2beta AP-2alpha E2F-1:DP-1 Smad3 Smad4 E2F-1 AhR:Arnt ENKTF-1
hsa-mir-129-2	TCGCGG GCGGCG CGCGGC CGCGCT GCCGCG GCGCGG CGCGGA GGGCCG CGGCGC CCGCGG GGGGCG GCGGGG CGCGCG CGGCGG GCCGGC GCGCGC	CTCF E2F-1 GLI1 GLI3 GKLF Ikaros p53 ZNFN1A1 Copeb NF-Y HNF-3beta MTF-1 AP-2 AP-2gamma HES-1 AP-2delta HDBP2 AP-2beta c-Myb HDBP1 AP-2alpha AP-2alphaA Pmx2a HOXA5 E2F-1:DP-1 COUP-TF1 Sp1 c-Jun:c-Fos AP-4 c-Jun Sp3 AP-1 Egr-1 GCF EBF MZF-1 Egr-2 MITF Smad3 Smad4 AhR:Arnt MAZ ENKTF-1
hsa-mir-130b	GGCCGC GGGCCG CCGCGG GGGGCG GCGCCG GCGGGG CCGCGT CGCGGC CCCGCG GCCGCC CGCCGG CGCGTC CGGCCG GCCGGC GCCGCG	COUP-TF1 CTCF Sp1 Egr-1 GATA-2 GATA-1 Ikaros CP2 HOXA5 Pmx2a ZNFN1A1 Sp3 GCF Copeb MTF-1 EBF AP-2 p53 AP-2gamma MZF-1 AP-2delta AP-2beta AP-2alpha NF-Y AP-2alphaA AP-1 c-Jun:c-Fos Egr-2 c-Jun MAZ EFI CTF-2 ENKTF-1 CTF-3 CTF CTF-1 E2F-1:DP-1 c-Myb HNF-3beta HES-1 E2F-1 HDBP2 HDBP1 MITF YY1 c-Ets-2 Smad3 AhR:Arnt Smad4
hsa-mir-132	CGCCGC GCGGCG CGCGGC CCGGCG CCGCCG CGGCCG GCCGCG GCGCGG GGCCGC CCGCGG GGGCCG CGGCGC GCGCCG GGGGCG GCGGGG CGCGCG CCCGCG AGCGCG GCCGCC TCCGCG CGCCGG GCGCGT GCCGGC GCGCGC	Sp1 GLI1 EFI CTF-2 YY1 CTF-3 CTF c-Ets-2 CTF-1 GLI3 NF-Y AP-2alphaA GKLF Ikaros p53 ZNFN1A1 Copeb HNF-3beta MTF-1 CTCF AP-2 AP-2gamma HES-1 E2F-1 AP-2delta HDBP2 AP-2beta c-Myb HDBP1 AP-2alpha MITF Smad3 AhR:Arnt Smad4 CP2 Pmx2a HOXA5 GATA-2 GATA-1 COUP-TF1 c-Jun:c-Fos AP-4 c-Jun Sp3 AP-1 Egr-1 GCF EBF MZF-1 MAZ ENKTF-1 Egr-2 E2F-1:DP-1 E12 M-Twist M-Twist:E12 TWIST TWIST:E12
hsa-mir-148a	GCGCGG GGCCGC GGGGCG GCGGGG CGCGCG AGCGCG CGCGGG GCCGCC CCGCCG CGCCGG CGGCCG GCCGGC	c-Jun:c-Fos Sp1 AP-4 GLI1 c-Myb c-Jun GLI3 MTF-1 GKLF Sp3 AP-1 p53 Egr-1 HNF-3beta HES-1 HDBP2 GCF HDBP1 COUP-TF1 CTCF GATA-2 GATA-1 Ikaros CP2 HOXA5 Pmx2a ZNFN1A1 Egr-2 AP-2alphaA Copeb MZF-1 MITF E2F-1:DP-1 ENKTF-1 EBF NF-Y EFI CTF-2 YY1 CTF-3 CTF c-Ets-2 CTF-1 MAZ Smad3 AhR:Arnt Smad4
hsa-mir-152	GCGCGG GGCCGC GGGCCG CGGCGC CCGCGG GGGGCG GCGCCG GCGGGG GCGGCG CGCGCG CGCGGG CGGCGG CCGGCG GCCGCG GCGCGC	c-Jun:c-Fos Sp1 AP-4 GLI1 c-Myb c-Jun GLI3 MTF-1 GKLF Sp3 AP-1 p53 Egr-1 HNF-3beta HES-1 HDBP2 GCF HDBP1 COUP-TF1 CTCF GATA-2 GATA-1 Ikaros CP2 HOXA5 Pmx2a ZNFN1A1 Copeb NF-Y EBF AP-2 AP-2gamma MZF-1 AP-2delta AP-2beta AP-2alpha AP-2alphaA Egr-2 MAZ EFI CTF-2 ENKTF-1 CTF-3 CTF CTF-1 E2F-1:DP-1 MITF Smad3 Smad4 AhR:Arnt
hsa-mir-153-1	GGCCGC GCGGGG CGCGGC CCCGCG AGCGCG CGCGGG TCCGCG GCCGGC	COUP-TF1 CTCF Sp1 Egr-1 GATA-2 GATA-1 Ikaros CP2 HOXA5 Pmx2a ZNFN1A1 MTF-1 Sp3 AP-1 c-Jun:c-Fos Copeb MZF-1 c-Myb c-Jun AP-2 p53 AP-2gamma HNF-3beta HES-1 E2F-1 AP-2delta HDBP2 AP-2beta HDBP1 AP-2alpha AP-2alphaA MAZ EBF MITF E2F-1:DP 1 AP-4 GLI1 ENKTF-1 GLI3 GCF NF-Y CTF-3 CTF CTF-1 EFI CTF-2 Smad3 AhR:Arnt Smad4
hsa-mir-17	CGCCGC GCGGCG CCGCGT CGCGGC CGCGTG CCGCCG CGGCCG GCCGCG GCGCGG GGCCGC CGCGGA CCGCGG GGGCCG CGGCGC GCGCCG GGGGCG GCGGGG CCCGCG GCCGCC CGGCGG CGCCGG CGCGTC GCGCGC	Sp1 GLI1 EFI CTF-2 YY1 CTF-3 CTF c-Ets-2 CTF-1 GLI3 NF-Y AP-2alphaA GKLF Ikaros p53 ZNFN1A1 Copeb HNF-3beta COUP-TF1 CP2 GATA-2 GATA-1 MTF-1 CTCF AP-2 AP-2gamma HES-1 E2F-1 AP-2delta HDBP2 AP-2beta c-Myb HDBP1 AP-2alpha E12 M-Twist M-Twist:E12 TWIST TWIST:E12 Pmx2a HOXA5 c-Jun:c-Fos AP-4 c-Jun Sp3 AP-1 Egr-1 GCF EBF MZF-1 MAZ ENKTF-1 Egr-2 E2F-1:DP-1 MITF Smad3 Smad4
hsa-mir-18a	CGCCGC GCGGCG CCGCGT CGCGGC CCGCCG GCCGCG GGCCGC CGCGGA GGGCCG CGGCGC CCGCGG GCGCCG GGGGCG GCGGGG GCCGCC CGGCGG CGCCGG CGCGTC GCGCGC	Sp1 GLI1 EFI CTF-2 YY1 CTF-3 CTF c-Ets-2 CTF-1 GLI3 NF-Y AP-2alphaA GKLF Ikaros p53 ZNFN1A1 Copeb HNF-3beta COUP-TF1 CP2 GATA-2 GATA-1 MTF-1 CTCF AP-2 AP-2gamma HES-1 E2F-1 AP-2delta HDBP2 AP-2beta c-Myb HDBP1 AP-2alpha Pmx2a HOXA5 Egr-1 Sp3 GCF EBF MZF-1 MAZ ENKTF-1 Egr-2 E2F-1:DP-1 AP-1 c-Jun:c-Fos c-Jun MITF Smad3 Smad4
hsa-mir-18b	GCGCGG CGCGGG GCGCGT CGCGCG AGCGCG	c-Jun:c-Fos Sp1 AP-4 GLI1 c-Myb c-Jun GLI3 MTF-1 GKLF Sp3 AP-1 p53 Egr-1 HNF-3beta HES-1 HDBP2 GCF HDBP1 EBF MZF-1 NF-Y E12 M-Twist M-Twist:E12 TWIST TWIST:E12 MITF E2F-1:DP-1 ENKTF-1

hsa-mir-191	CGCGGC CCGGCG CGCGAG CGCGCT CGGCGA GCCGCG GCGCGG CGCGGA CCGCGG GGGCCG CGGCGC GGGGCG GCGGGG CGCGCG CCCGCG AGCGCG CGCGGG GTCGCG CGCCGG GCGCGT CGCGTC GCCGGC GCGCGC	MTF-1 CTCF AP-2 p53 AP-2gamma HNF-3beta HES-1 E2F-1 AP-2delta HDBP2 AP-2beta c-Myb HDBP1 AP-2alpha AP-2alphaA MITF Sp1 Copeb Smad3 AhR:Arnt NF-Y Smad4 Pmx2a HOXA5 E2F-1:DP-1 COUP-TF1 c-Jun:c-Fos AP-4 GLI1 c-Jun GLI3 GKLF Sp3 AP-1 Egr-1 GCF EBF MZF-1 Ikaros ZNFN1A1 Egr-2 MAZ GATA-2 GATA-1 CP2 ENKTF-1 c-Ets-2 E12 M-Twist M-Twist:E12 TWIST TWIST:E12
hsa-mir-193a	CGCCGC GCGGCG CGCGGC CCGGCG CCGCCG CGGCGA CGGCCG GCCGCG GCGCGG GGCCGC CCGCGG GGGCCG GCGCCG GGGGCG GCGGGG CGCCGA CCCGCG CGCGGG GCCGCC CGGCGG CGCCGG GCCGGC GCGCGC	Sp1 GLI1 EFI CTF-2 YY1 CTF-3 CTF c-Ets-2 CTF-1 GLI3 NF-Y AP-2alphaA GKLF Ikaros p53 ZNFN1A1 Copeb HNF-3beta MTF-1 CTCF AP-2 AP-2gamma HES-1 E2F-1 AP-2delta HDBP2 AP-2beta c-Myb HDBP1 AP-2alpha MITF Smad3 AhR:Arnt Smad4 CP2 Pmx2a HOXA5 GATA-2 GATA-1 COUP-TF1 c-Jun:c-Fos AP-4 c-Jun Sp3 AP-1 Egr-1 GCF EBF MZF-1 MAZ ENKTF-1 Egr-2 E2F-1:DP-1
hsa-mir-193b	CGCCGC GCGGCG CGCGGC CCGCCG CGCGCT GCCGCG GCGCGG GGCCGC GGGCCG CGGCGC GGGGCG GCGGGG CGCGCG AGCGCG GCCGCC CGGCGG GCCGGC GCGCGC	Sp1 GLI1 EFI CTF-2 YY1 CTF-3 CTF c-Ets-2 CTF-1 GLI3 NF-Y AP-2alphaA GKLF Ikaros p53 ZNFN1A1 Copeb HNF-3beta MTF-1 CTCF AP-2 AP-2gamma HES-1 E2F-1 AP-2delta HDBP2 AP-2beta c-Myb HDBP1 AP-2alpha Pmx2a HOXA5 E2F-1:DP-1 COUP-TF1 c-Jun:c-Fos AP-4 c-Jun Sp3 AP-1 Egr-1 GCF GATA-2 GATA-1 CP2 Egr-2 MZF-1 MITF ENKTF-1 Smad3 Smad4 AhR:Arnt MAZ
hsa-mir-196a-1	CGCGGG GCGCGG GGCCGC GCCGCC GGGCCG CGGCGG CGCCGC CCGCCG CGGCCG GCCGCG	MTF-1 EBF Sp3 AP-1 c-Jun:c-Fos Sp1 p53 Egr-1 AP-4 GLI1 MZF-1 GCF c-Jun GLI3 NF-Y c-Myb GKLF HNF-3beta HES-1 HDBP2 HDBP1 COUP-TF1 CTCF GATA-2 GATA-1 Ikaros CP2 HOXA5 Pmx2a ZNFN1A1 EFI CTF-2 YY1 CTF-3 CTF c-Ets-2 CTF-1 AP-2alphaA Copeb MITF Smad3 Smad4
hsa-mir-196b	GCCGCC GGGGCG CGGCGG GCGGCG GCGCGC	CTCF Sp1 Egr-1 EFI CTF-2 GATA-2 GATA-1 YY1 CTF-3 CTF CP2 c-Ets-2 CTF-1 AP-2alphaA MTF-1 Sp3 AP-1 c-Jun:c-Fos Egr-2 Ikaros ZNFN1A1 c-Jun p53 Copeb MITF Smad3 Smad4 NF-Y GKLF HNF-3beta MAZ c-Myb E2F-1:DP-1 ENKTF-1
hsa-mir-19a	CGCGGA GGGCCG CGGCGC CGCCGC CCGCGT GCCGCC CCGCCG CGCCGG CGCGTC GCCGCG	GLI1 GLI3 COUP-TF1 Sp3 Ikaros GCF Egr-1 ZNFN1A1 Copeb GKLF p53 HDBP2 c-Myb HDBP1 NF-Y Sp1 EFI CTF-2 YY1 CTF-3 CTF c-Ets-2 CTF-1 AP-2alphaA CP2 GATA-2 GATA-1 CTCF MAZ Egr-2 E2F-1:DP-1 Pmx2a HOXA5
hsa-mir-203	CGCCGC GCGGCG CGCGGC CCGGCG CCGCCG GCGCGG GGGCCG CGGCGC GGGGCG GCGCCG GCGGGG CGCGCG AGCGCG CCCGCG CGCGGG GCCGCC CGGCGG CGCCGG	Sp1 GLI1 EFI CTF-2 YY1 CTF-3 CTF c-Ets-2 CTF-1 GLI3 NF-Y AP-2alphaA GKLF Ikaros p53 ZNFN1A1 Copeb HNF-3beta MTF-1 CTCF AP-2 AP-2gamma HES-1 E2F-1 AP-2delta HDBP2 AP-2beta c-Myb HDBP1 AP-2alpha MITF Smad3 AhR:Arnt Smad4 c-Jun:c-Fos AP-4 c-Jun Sp3 AP-1 Egr-1 GCF COUP-TF1 Egr-2 MAZ ENKTF-1 MZF-1 E2F-1:DP-1 EBF GATA-2 GATA-1 CP2
hsa-mir-210	CGCCGC GCGGCG CGCGGC CCGGCG CCGCCG CGCGAG CGGCCG GCCGCG GCGCGG GGCCGC CGCGGA CCGCGG GGGCCG GCGCCG GGGGCG GCGGGG CGCGCG CCCGCG CGCGGG GCCGCC CGGCGG CGCCGG GCCGGC	Sp1 GLI1 EFI CTF-2 YY1 CTF-3 CTF c-Ets-2 CTF-1 GLI3 NF-Y AP-2alphaA GKLF Ikaros p53 ZNFN1A1 Copeb HNF-3beta MTF-1 CTCF AP-2 AP-2gamma HES-1 E2F-1 AP-2delta HDBP2 AP-2beta c-Myb HDBP1 AP-2alpha MITF Smad3 AhR:Arnt Smad4 CP2 Pmx2a HOXA5 GATA-2 GATA-1 COUP-TF1 c-Jun:c-Fos AP-4 c-Jun Sp3 AP-1 Egr-1 GCF EBF MZF-1 MAZ ENKTF-1 Egr-2 E2F-1:DP-1
hsa-mir-212	CGCCGC GCGGCG CGCGGC CCGCCG GCCGCG GCGCGG GGCCGC GGGCCG CGGCGC CCGCGG GCGCCG GGGGCG GCGGGG CCCGCG GCCGCC TCCGCG CGCCGG GCGCGT GCCGGC	Sp1 GLI1 EFI CTF-2 YY1 CTF-3 CTF c-Ets-2 CTF-1 GLI3 NF-Y AP-2alphaA GKLF Ikaros p53 ZNFN1A1 Copeb HNF-3beta MTF-1 CTCF AP-2 AP-2gamma HES-1 E2F-1 AP-2delta HDBP2 AP-2beta c-Myb HDBP1 AP-2alpha COUP-TF1 Pmx2a HOXA5 c-Jun:c-Fos AP-4 c-Jun Sp3 AP-1 Egr-1 GCF GATA-2 GATA-1 CP2 EBF MZF-1 MAZ ENKTF-1 Egr-2 E2F-1:DP-1 MITF E12 M-Twist M-Twist:E12 TWIST TWIST:E12 Smad3 AhR:Arnt Smad4
hsa-mir-219-2	GGCCGC GGGCCG CGGCGC GGGGCG CGCCGC GCGGGG GCGGCG CCGCGT CGCGTG CGCGGG GCCGCC TCCGCG CCGCCG CGCCGG CGGCCG GCCGCG	COUP-TF1 CTCF Sp1 Egr-1 GATA-2 GATA-1 Ikaros CP2 HOXA5 Pmx2a ZNFN1A1 Sp3 GCF Copeb GKLF p53 HDBP2 c-Myb HDBP1 NF-Y MTF-1 AP-1 c-Jun:c-Fos Egr-2 c-Jun AP-2alphaA GLI1 EFI CTF-2 YY1 CTF-3 CTF c-Ets-2 CTF-1 GLI3 MZF-1 HNF-3beta HES-1 E12 M-Twist M-Twist:E12 TWIST TWIST:E12 EBF AP-4 MAZ E2F-1:DP-1
hsa-mir-22	GGGCCG GGGGCG GCGGGG GCGGCG CGGCGG CCGGCG CGGCGA GCCGGC	COUP-TF1 Sp3 Ikaros GCF Egr-1 ZNFN1A1 Copeb MTF-1 AP-1 c-Jun:c-Fos Egr-2 c-Jun AP-2alphaA MZF-1 c-Myb GKLF p53 NF-Y HNF-3beta Sp1 MITF Smad3 Smad4 AhR:Arnt
hsa-mir-23b	CCGCGG CGCGGC GCCGGC AGCGCG	MTF-1 EBF AP-2 p53 Sp1 AP-2gamma MZF-1 AP-2delta AP-2beta AP-2alpha NF-Y AP-2alphaA CTCF HNF-3beta HES-1 E2F-1 HDBP2 c-Myb HDBP1 Sp3 Copeb Smad3 AhR:Arnt Smad4 AP-1 c-Jun:c-Fos AP-4 GLI1 ENKTF-1 c-Jun GLI3
hsa-mir-27b	CCGCGG CGCGGC GCCGGC AGCGCG	MTF-1 EBF AP-2 p53 Sp1 AP-2gamma MZF-1 AP-2delta AP-2beta AP-2alpha NF-Y AP-2alphaA CTCF HNF-3beta HES-1 E2F-1 HDBP2 c-Myb HDBP1 Sp3 Copeb Smad3 AhR:Arnt Smad4 AP-1 c-Jun:c-Fos AP-4 GLI1 ENKTF-1 c-Jun GLI3
hsa-mir-320	GCGCGG GGCCGC GGGGCG GCGCCG CGCCGC GCCGCC CCGGCG CGCCGG CGGCCG GCCGGC	c-Jun:c-Fos Sp1 AP-4 GLI1 c-Myb c-Jun GLI3 MTF-1 GKLF Sp3 AP-1 p53 Egr-1 HNF-3beta HES-1 HDBP2 GCF HDBP1 COUP-TF1 CTCF GATA-2 GATA-1 Ikaros CP2 HOXA5 Pmx2a ZNFN1A1 Egr-2 AP-2alphaA MAZ EFI CTF-2 ENKTF-1 MZF-1 CTF-3 CTF CTF-1 NF-Y E2F-1:DP-1 YY1 c-Ets-2 MITF Copeb Smad3 AhR:Arnt Smad4
hsa-mir-345	TCGCGG CGCCGC GCGGCG CGCGGC CCGGCG CCGCCG CGCGAG CGGCCG CGGCGA CGCGCT GCCGCG CGCGGA GCGCGG GGCCGC CGGCGC GGGCCG CCGCGG GGGGCG GCGCCG GCGGGG TGCGCG CGCGCG CCCGCG AGCGCG CGCGGG GCCGCC CGGCGG CGCCGG GCGCGC	CTCF E2F-1 GLI1 GLI3 Sp1 EFI CTF-2 YY1 CTF-3 CTF c-Ets-2 CTF-1 NF-Y AP-2alphaA GKLF Ikaros p53 ZNFN1A1 Copeb HNF-3beta MTF-1 AP-2 AP-2gamma HES-1 AP-2delta HDBP2 AP-2beta c-Myb HDBP1 AP-2alpha MITF Smad3 AhR:Arnt Smad4 CP2 Pmx2a HOXA5 GATA-2 GATA-1 E2F-1:DP-1 COUP-TF1 c-Jun:c-Fos AP-4 c-Jun Sp3 AP-1 Egr-1 GCF EBF MZF-1 Egr-2 MAZ ENKTF-1 E12 M-Twist M-Twist:E12 TWIST TWIST:E12

hsa-mir-34b	GCGCGG GGCCGC GGGCCG CCGCGG GGGGCG GCGGGG CCCGCG CGCGGG CGGCGG	c-Jun:c-Fos Sp1 AP-4 GLI1 c-Myb c-Jun GLI3 MTF-1 GKLF Sp3 AP-1 p53 Egr-1 HNF-3beta HES-1 HDBP2 GCF HDBP1 COUP-TF1 CTCF GATA-2 GATA-1 Ikaros CP2 HOXA5 Pmx2a ZNFN1A1 Copeb EBF AP-2 AP-2gamma MZF-1 AP-2delta AP-2beta AP-2alpha NF-Y AP-2alphaA Egr-2 MAZ MITF E2F-1:DP-1 Smad3 Smad4
hsa-mir-34c	CGCCGC GCGGCG CGCGGC CGGCCG GCGCGG GGCCGC GGGCCG CCGCGG GGGGCG GCGGGG CCCGCG CGCGGG GCCGCC CGGCGG GCCGGC TCGCGG CGCCGC CGCGGC CCGCCG CGCGAG	Sp1 GLI1 EFI CTF-2 YY1 CTF-3 CTF c-Ets-2 CTF-1 GLI3 NF-Y AP-2alphaA GKLF Ikaros p53 ZNFN1A1 Copeb HNF-3beta MTF-1 CTCF AP-2 AP-2gamma HES-1 E2F-1 AP-2delta HDBP2 AP-2beta c-Myb HDBP1 AP-2alpha CP2 Pmx2a HOXA5 GATA-2 GATA-1 c-Jun:c-Fos AP-4 c-Jun Sp3 AP-1 Egr-1 GCF COUP-TF1 EBF MZF-1 Egr-2 MAZ MITF E2F-1:DP-1 Smad3 Smad4 AhR:Arnt
hsa-mir-375	CGGCCG GCCGCG GCGCGG GGCCGC CGCGGA CCGCGG GGGGCG GCGCCG GCGGGG CGCGCG CGCGGG GCCGCC CGCCGG GCGCGC TCGCGG CGCCGC CGCGGC CGCGTG CGCGCT	CTCF E2F-1 GLI1 GLI3 Sp1 EFI CTF-2 YY1 CTF-3 CTF c-Ets-2 CTF-1 NF-Y AP-2alphaA MTF-1 AP-2 p53 AP-2gamma HNF-3beta HES-1 AP-2delta HDBP2 AP-2beta c-Myb HDBP1 AP-2alpha CP2 Pmx2a HOXA5 GATA-2 GATA-1 COUP-TF1 c-Jun:c-Fos AP-4 c-Jun GKLF Sp3 AP-1 Egr-1 GCF Ikaros ZNFN1A1 EBF MZF-1 Egr-2 MAZ ENKTF-1 E2F-1:DP-1 Copeb MITF
hsa-mir-378	GCCGCG GCGCGG GGCCGC CCGCGG GGGGCG GCGCCG GCGGGG CGCCGA TGCGCG CGCGCG CCCGCG CGCGGG GCCGCC CGCCGG GCGCGT GCCGGC GCGCGC CGCGGC CCGGCG CGCGAG CGCGCT GCCGCG	CTCF E2F-1 GLI1 GLI3 Sp1 EFI CTF-2 YY1 CTF-3 CTF c-Ets-2 CTF-1 NF-Y AP-2alphaA MTF-1 AP-2 p53 AP-2gamma HNF-3beta HES-1 AP-2delta HDBP2 AP-2beta c-Myb HDBP1 AP-2alpha COUP-TF1 E12 M-Twist M-Twist:E12 TWIST TWIST:E12 Pmx2a HOXA5 E2F-1:DP-1 c-Jun:c-Fos AP-4 c-Jun GKLF Sp3 AP-1 Egr-1 GCF GATA-2 GATA-1 Ikaros CP2 ZNFN1A1 EBF MZF-1 Egr-2 MAZ ENKTF-1 Copeb AhR:Arnt MITF Smad3 Smad4
hsa-mir-425	GCGCGG CGCGGA CCGCGG GGGCCG CGGCGC GGGGCG GCGGGG CGCGCG CCCGCG AGCGCG CGCGGG GTCGCG CGCGTC GCCGGC GCGCGC	MTF-1 CTCF AP-2 p53 AP-2gamma HNF-3beta HES-1 E2F-1 AP-2delta HDBP2 AP-2beta c-Myb HDBP1 AP-2alpha AP-2alphaA MITF Sp1 Copeb Smad3 AhR:Arnt NF-Y Smad4 Pmx2a HOXA5 E2F-1:DP-1 COUP-TF1 c-Jun:c-Fos AP-4 GLI1 c-Jun GLI3 GKLF Sp3 AP-1 Egr-1 GCF EBF MZF-1 Ikaros ZNFN1A1 Egr-2 MAZ GATA-2 GATA-1 CP2 ENKTF-1
hsa-mir-429	GGGCCG GCCGCC GGGGCG CCGCCG GCGGGG GCCGGC	COUP-TF1 Sp3 Ikaros GCF Egr-1 ZNFN1A1 Copeb CTCF Sp1 EFI CTF-2 GATA-2 GATA-1 YY1 CTF-3 CTF CP2 c-Ets-2 CTF-1 AP-2alphaA MTF-1 AP-1 c-Jun:c-Fos Egr-2 c-Jun HDBP2 HDBP1 MZF-1 c-Myb Smad3 AhR:Arnt NF-Y Smad4
hsa-mir-484	GCCGCC GGGGCG CGCCGC CCGCCG GCGGGG CGCGTC	CTCF Sp1 Egr-1 EFI CTF-2 GATA-2 GATA-1 YY1 CTF-3 CTF CP2 c-Ets-2 CTF-1 AP-2alphaA MTF-1 Sp3 AP-1 c-Jun:c-Fos Egr-2 Ikaros ZNFN1A1 c-Jun GLI1 GLI3 NF-Y HDBP2 HDBP1 Copeb MZF-1 c-Myb
hsa-mir-565	GGCCGC GGGCCG GCGCCG CGCCGC GCGGCG CGCCGA CGCGGC GCCGCC CCGCCG GCCGGC GCCGCG	COUP-TF1 CTCF Sp1 Egr-1 GATA-2 GATA-1 Ikaros CP2 HOXA5 Pmx2a ZNFN1A1 Sp3 GCF Copeb MAZ EFI CTF-2 ENKTF-1 Egr-2 MZF-1 CTF-3 CTF CTF-1 NF-Y E2F-1:DP-1 GLI1 YY1 c-Ets-2 GLI3 AP-2alphaA GKLF p53 HNF-3beta MTF-1 AP-2 AP-2gamma HES-1 E2F-1 AP-2delta HDBP2 AP-2beta c-Myb HDBP1 AP-2alpha Smad3 AhR:Arnt Smad4
hsa-mir-574	GCGCGG GGCCGC CGGCGC GGGGCG GCGGGG GCGGCG CGCGGC CCCGCG AGCGCG CGCGGG GCCGCC CGGCCG CGCGCT	c-Jun:c-Fos Sp1 AP-4 GLI1 c-Myb c-Jun GLI3 MTF-1 GKLF Sp3 AP-1 p53 Egr-1 HNF-3beta HES-1 HDBP2 GCF HDBP1 COUP-TF1 CTCF GATA-2 GATA-1 Ikaros CP2 HOXA5 Pmx2a ZNFN1A1 NF-Y Egr-2 AP-2alphaA Copeb MZF-1 AP-2 AP-2gamma E2F-1 AP-2delta AP-2beta AP-2alpha MAZ EBF MITF E2F-1:DP-1 ENKTF-1 EFI CTF-2 YY1 CTF-3 CTF c-Ets-2 CTF-1
hsa-mir-594	GCGCGG CGGCGC GCGCGT AGCGCG GCGCGC	c-Jun:c-Fos Sp1 AP-4 GLI1 c-Myb c-Jun GLI3 MTF-1 GKLF Sp3 AP-1 p53 Egr-1 HNF-3beta HES-1 HDBP2 GCF HDBP1 NF-Y E12 M-Twist M-Twist:E12 TWIST TWIST:E12 ENKTF-1 MITF MAZ E2F-1:DP-1 AP-2alphaA
hsa-mir-611	CGGCGG GCGGCG CGCGGC CGCGCT CCCGCG	Sp1 p53 Copeb MITF Ikaros Smad3 ZNFN1A1 Smad4 NF-Y GKLF HNF-3beta MTF-1 CTCF AP-2 AP-2gamma HES-1 E2F-1 AP-2delta HDBP2 AP-2beta c-Myb HDBP1 AP-2alpha AP-2alphaA Pmx2a HOXA5 E2F-1:DP-1 MAZ EBF GATA-2 GATA-1 CP2
hsa-mir-615	GGCCGC CGGCGC CCGCGG GCGGGG CGCGGC CCGGCG CCGCCG CGCCGG GCCGGC	COUP-TF1 CTCF Sp1 Egr-1 GATA-2 GATA-1 Ikaros CP2 HOXA5 Pmx2a ZNFN1A1 GKLF p53 HDBP2 c-Myb HDBP1 NF-Y MTF-1 EBF AP-2 AP-2gamma MZF-1 AP-2delta AP-2beta AP-2alpha AP-2alphaA Sp3 AP-1 c-Jun:c-Fos Copeb c-Jun HNF-3beta HES-1 E2F-1 MITF Smad3 AhR:Arnt Smad4 c-Ets-2 YY1 MAZ Egr-2 E2F-1:DP-1
hsa-mir-616	CCGCGG GGGGCG GCGGGG TGCGCG CGCGTG CGCGGG TCCGCG GCGCGT	MTF-1 EBF AP-2 p53 Sp1 AP-2gamma MZF-1 AP-2delta AP-2beta AP-2alpha NF-Y AP-2alphaA Sp3 AP-1 c-Jun:c-Fos Egr-1 Egr-2 Ikaros ZNFN1A1 c-Jun Copeb c-Myb E12 M-Twist M-Twist:E12 TWIST AhR:Arnt TWIST:E12 COUP-TF1 HES-1 AP-4 GLI1 GCF GLI3 CTF-3 CTF CTF-1 EFI CTF-2
hsa-mir-618	GCGGCG CGCGGC CCGGCG CCGCCG CGGCGA GCGCGG CCGCGG GCGCCG CGCCGA GCGGGG CGCGGG GCCGCC CGCCGG CGCGTC TCGCGG GCGGCG CGCGGC CCGGCG CCGCCG CGCGAG CGGCCG CGGCGA CGCGCT GCCGCG	GKLF Ikaros p53 ZNFN1A1 Copeb NF-Y HNF-3beta MTF-1 CTCF AP-2 AP-2gamma HES-1 E2F-1 AP-2delta HDBP2 AP-2beta c-Myb HDBP1 AP-2alpha AP-2alphaA MITF Sp1 Smad3 AhR:Arnt Smad4 c-Ets-2 YY1 c-Jun:c-Fos AP-4 GLI1 c-Jun GLI3 Sp3 AP-1 Egr-1 GCF EBF MZF-1 MAZ EFI CTF-2 ENKTF-1 Egr-2 CTF-3 CTF CTF-1 E2F-1:DP-1 GATA-2 GATA-1 CP2
hsa-mir-636	GCGCGG GGCCGC CGGCGC CCGCGG GGGCCG GGGGCG GCGGGG CGCGCG CCCGCG AGCGCG CGCGGG GCCGCC TCCGCG CGCCGG GCGCGT CGCGTC GCCGGC GCGCGC TTCGCG	CTCF E2F-1 GLI1 GLI3 GKLF Ikaros p53 ZNFN1A1 Copeb NF-Y HNF-3beta MTF-1 AP-2 AP-2gamma HES-1 AP-2delta HDBP2 AP-2beta c-Myb HDBP1 AP-2alpha AP-2alphaA MITF Sp1 Smad3 AhR:Arnt Smad4 c-Ets-2 YY1 CP2 Pmx2a HOXA5 GATA-2 GATA-1 E2F-1:DP-1 COUP-TF1 c-Jun:c-Fos AP-4 c-Jun Sp3 AP-1 Egr-1 GCF EBF MZF-1 Egr-2 MAZ ENKTF-1 EFI CTF-2 CTF-3 CTF CTF-1 E12 M-Twist M-Twist:E12 TWIST TWIST:E12
hsa-mir-638	GGGCCG CGGCGC CCGCGG GCGGGG GCGGCG CGCGGG CGCCGG GCCGGC	COUP-TF1 Sp3 Ikaros GCF Egr-1 ZNFN1A1 Copeb GKLF p53 HDBP2 c-Myb HDBP1 NF-Y MTF-1 EBF AP-2 Sp1 AP-2gamma MZF-1 AP-2delta AP-2beta AP-2alpha AP-2alphaA AP-1 c-Jun:c-Fos c-Jun HNF-3beta AP-4 GLI1 GLI3 CTCF MAZ Egr-2 c-Ets-2 E2F-1:DP-1 Smad3 AhR:Arnt Smad4
hsa-mir-663	TCGCGG CGCGGC CGCGTG CCGGCG CCGCCG CGCGAG CGGCGA GCCGCG GGCCGC CCGCGG CGGCGC GCGCCG GGGGCG GCGGGG TGCGCG CGCGGG GCCGCC CGCCGG GCCGGC GCGCGC	CTCF E2F-1 GLI1 GLI3 MTF-1 AP-2 p53 AP-2gamma HNF-3beta HES-1 AP-2delta HDBP2 AP-2beta c-Myb HDBP1 AP-2alpha AP-2alphaA COUP-TF1 E12 M-Twist M-Twist:E12 TWIST TWIST:E12 MITF Sp1 Copeb Smad3 AhR:Arnt NF-Y Smad4 c-Ets-2 YY1 Pmx2a HOXA5 Egr-1 GATA-2 GATA-1 Ikaros CP2 ZNFN1A1 EBF MZF-1 GKLF Sp3 MAZ EFI CTF-2 ENKTF-1 Egr-2 CTF-3 CTF CTF-1 E2F-1:DP-1 AP-1 c-Jun:c-Fos c-Jun AP-4 GCF

hsa-mir-9-1	GCGGCG CGCGGC CCGGCG CGGCGA CGGCCG GGGCCG CCGCGG GGGGCG GCGGGG CGCGCG CCCGCG CGGCGG CGCCGG GCGCGT CGCGTC GCCGGC	GKLF Ikaros p53 ZNFN1A1 Copeb NF-Y HNF-3beta MTF-1 CTCF AP-2 AP-2gamma HES-1 E2F-1 AP-2delta HDBP2 AP-2beta c-Myb HDBP1 AP-2alpha AP-2alphaA MITF Sp1 Smad3 AhR:Arnt Smad4 CP2 Pmx2a HOXA5 GATA-2 GATA-1 COUP-TF1 Sp3 GCF Egr-1 EBF MZF-1 AP-1 c-Jun:c-Fos Egr-2 c-Jun E2F-1:DP-1 MAZ c-Ets-2 E12 M-Twist M-Twist:E12 TWIST TWIST:E12
hsa-mir-92b	GCGCGG GGCCGC GGGCCG GCGCCG CGCCGC GCGGGG GCGGCG CGCGGC CGCGCG CCCGCG AGCGCG CGGCGA GCCGCG GCGCGC	c-Jun:c-Fos Sp1 AP-4 GLI1 c-Myb c-Jun GLI3 MTF-1 GKLF Sp3 AP-1 p53 Egr-1 HNF-3beta HES-1 HDBP2 GCF HDBP1 COUP-TF1 CTCF GATA-2 GATA-1 Ikaros CP2 HOXA5 Pmx2a ZNFN1A1 Copeb MAZ EFI CTF-2 ENKTF-1 Egr-2 MZF-1 CTF-3 CTF CTF-1 NF-Y E2F-1:DP-1 YY1 c-Ets-2 AP-2alphaA AP-2 AP-2gamma E2F-1 AP-2delta AP-2beta AP-2alpha MITF EBF
hsa-mir-9-3	GCGCGG GGGGCG GCGCCG GCGGGG CCGCGT CCCGCG CGCGGG CGCGTG TCCGCG CGCCGG GCCGGC	c-Jun:c-Fos Sp1 AP-4 GLI1 c-Myb c-Jun GLI3 MTF-1 GKLF Sp3 AP-1 p53 Egr-1 HNF-3beta HES-1 HDBP2 GCF HDBP1 Egr-2 Ikaros ZNFN1A1 AP-2alphaA MAZ EFI CTF-2 ENKTF-1 MZF-1 CTF-3 CTF CTF-1 NF-Y E2F-1:DP-1 Copeb COUP-TF1 CP2 GATA-2 GATA-1 EBF AP-2 AP-2gamma MITF AP-2delta AP-2beta AP-2alpha E12 M-Twist M-Twist:E12 TWIST TWIST:E12 CTCF c-Ets-2 Smad3 AhR:Arnt Smad4
