# Supplementary material for: Regulatory Circuit of Human MicroRNA Biogenesis
Source: PLoS Comput Biol. 2007 Apr 20;3(4):e67. doi: 10.1371/journal.pcbi.0030067 (PMC1853126; doi:10.1371/journal.pcbi.0030067)
Supplement: Supplementary file 14 [file pcbi.0030067.st004.pdf]

TFs are sorted based on the number of miRNAs predicted to be regulated by them

TF	Number of miRNAs regulated
c-Myb	48
NF-Y	48
Sp1	48
MTF-1	47
Sp3	47
AP-2alphaA	47
p53	46
AP-1	45
Egr-1	45
c-Jun:c-Fos	45
c-Jun	45
Copeb	45
HDBP2	44
HDBP1	44
HNF-3beta	44
MZF-1	44
ZNFN1A1	43
CTCF	43
Ikaros	43
GKLF	41
GCF	41
HES-1	41
CP2	40
GLI3	40
GATA-2	40
GLI1	40
GATA-1	40
Egr-2	40
E2F-1:DP-1	40
COUP-TF1	39
MAZ	39
MITF	39
EBF	38
Smad3	38

Smad4	38
AP-2	36
AP-2gamma	36
AP-2beta	36
AP-2delta	36
AP-4	36
AP-2alpha	36
HOXA5	34
AhR:Arnt	34
ENKTF-1	34
Pmx2a	34
c-Ets-2	34
EFI	33
CTF-3	33
CTF-2	33
CTF	33
CTF-1	33
YY1	30
E2F-1	30
M-Twist	17
M-Twist:E12	17
E12	17
TWIST	17
TWIST:E12	17
